# Guidelines for the use of flow cytometry and cell sorting in immunological studies (third edition)

**DOI:** 10.1002/eji.202170126

**Published:** 2021-12-07

**Authors:** Andrea Cossarizza, Hyun-Dong Chang, Andreas Radbruch, Sergio Abrignani, Richard Addo, Mübeccel Akdis, Immanuel Andrä, Francesco Andreata, Francesco Annunziato, Eduardo Arranz, Petra Bacher, Sudipto Bari, Vincenzo Barnaba, Joana Barros-Martins, Dirk Baumjohann, Cristian G. Beccaria, David Bernardo, Dominic A. Boardman, Jessica Borger, Chotima Böttcher, Leonie Brockmann, Marie Burns, Dirk H. Busch, Garth Cameron, Ilenia Cammarata, Antonino Cassotta, Yinshui Chang, Fernando Gabriel Chirdo, Eleni Christakou, Luka Čičin-Šain, Laura Cook, Alexandra J. Corbett, Rebecca Cornelis, Lorenzo Cosmi, Martin S. Davey, Sara De Biasi, Gabriele De Simone, Genny del Zotto, Michael Delacher, Francesca Di Rosa, James Di Santo, Andreas Diefenbach, Jun Dong, Thomas Dörner, Regine J. Dress, Charles-Antoine Dutertre, Sidonia B. G. Eckle, Pascale Eede, Maximilien Evrard, Christine S. Falk, Markus Feuerer, Simon Fillatreau, Aida Fiz-Lopez, Marie Follo, Gemma A. Foulds, Julia Fröbel, Nicola Gagliani, Giovanni Galletti, Anastasia Gangaev, Natalio Garbi, José Antonio Garrote, Jens Geginat, Nicholas A. Gherardin, Lara Gibellini, Florent Ginhoux, Dale I. Godfrey, Paola Gruarin, Claudia Haftmann, Leo Hansmann, Christopher M. Harpur, Adrian C. Hayday, Guido Heine, Daniela Carolina Hernández, Martin Herrmann, Oliver Hoelsken, Qing Huang, Samuel Huber, Johanna E. Huber, Jochen Huehn, Michael Hundemer, William Y. K. Hwang, Matteo Iannacone, Sabine M. Ivison, Hans-Martin Jäck, Peter K. Jani, Baerbel Keller, Nina Kessler, Steven Ketelaars, Laura Knop, Jasmin Knopf, Hui-Fern Koay, Katja Kobow, Katharina Kriegsmann, H. Kristyanto, Andreas Krueger, Jenny F. Kuehne, Heike Kunze-Schumacher, Pia Kvistborg, Immanuel Kwok, Daniela Latorre, Daniel Lenz, Megan K. Levings, Andreia C. Lino, Francesco Liotta, Heather M. Long, Enrico Lugli, Katherine N. MacDonald, Laura Maggi, Mala K. Maini, Florian Mair, Calin Manta, Rudolf Armin Manz, Mir-Farzin Mashreghi, Alessio Mazzoni, James McCluskey, Henrik E. Mei, Fritz Melchers, Susanne Melzer, Dirk Mielenz, Leticia Monin, Lorenzo Moretta, Gabriele Multhoff, Luis Enrique Muñoz, Miguel Muñoz-Ruiz, Franziska Muscate, Ambra Natalini, Katrin Neumann, Lai Guan Ng, Antonia Niedobitek, Jana Niemz, Larissa Nogueira Almeida, Samuele Notarbartolo, Lennard Ostendorf, Laura J. Pallett, Amit A. Patel, Gulce Itir Percin, Giovanna Peruzzi, Marcello Pinti, A. Graham Pockley, Katharina Pracht, Immo Prinz, Irma Pujol-Autonell, Nadia Pulvirenti, Linda Quatrini, Kylie M. Quinn, Helena Radbruch, Hefin Rhys, Maria B. Rodrigo, Chiara Romagnani, Carina Saggau, Shimon Sakaguchi, Federica Sallusto, Lieke Sanderink, Inga Sandrock, Christine Schauer, Alexander Scheffold, Hans U. Scherer, Matthias Schiemann, Frank A. Schildberg, Kilian Schober, Janina Schoen, Wolfgang Schuh, Thomas Schüler, Axel R. Schulz, Sebastian Schulz, Julia Schulze, Sonia Simonetti, Jeeshan Singh, Katarzyna M. Sitnik, Regina Stark, Sarah Starossom, Christina Stehle, Franziska Szelinski, Leonard Tan, Attila Tarnok, Julia Tornack, Timothy I. M. Tree, Jasper J. P. van Beek, Willem van de Veen, Klaas van Gisbergen, Chiara Vasco, Nikita A. Verheyden, Anouk von Borstel, Kirsten A. Ward-Hartstonge, Klaus Warnatz, Claudia Waskow, Annika Wiedemann, Anneke Wilharm, James Wing, Oliver Wirz, Jens Wittner, Jennie H. M. Yang, Juhao Yang

**Affiliations:** 1 Department of Medical and Surgical Sciences for Children & Adults, University of Modena and Reggio Emilia, Modena, Italy; 2 German Rheumatism Research Center Berlin (DRFZ), Berlin, Germany; 3 Istituto Nazionale di Genetica Molecolare Romeo ed Enrica Invernizzi (INGM), Milan, Italy; 4 Department of Clinical Sciences and Community Health, Università degli Studi di Milano, Milan, Italy; 5 Swiss Institute of Allergy and Asthma Research (SIAF), University of Zurich, Davos, Switzerland; 6 Institut für Medizinische Mikrobiologie, Immunologie und Hygiene, Technische Universität München, Munich, Germany; 7 Division of Immunology, Transplantation and Infectious Diseases, IRCSS San Raffaele Scientific Institute, Milan, Italy; 8 Department of Experimental and Clinical Medicine, University of Florence, Florence, Italy; 9 Mucosal Immunology Lab, Unidad de Excelencia Instituto de Biomedicina y Genética Molecular de Valladolid (IBGM, Universidad de Valladolid-CSIC), Valladolid, Spain; 10 Institute of Immunology, Christian-Albrechts Universität zu Kiel & Universitätsklinik Schleswig-Holstein, Kiel, Germany; 11 Institute of Clinical Molecular Biology Christian-Albrechts Universität zu Kiel, Kiel, Germany; 12 Division of Medical Sciences, National Cancer Centre Singapore, Singapore; 13 Cancer & Stem Cell Biology, Duke-NUS Medical School, Singapore, Singapore; 14 Dipartimento di Medicina Interna e Specialità Mediche, Sapienza Università di Roma, Rome, Italy; 15 Center for Life Nano & Neuro Science@Sapienza, Istituto Italiano di Tecnologia (IIT), Rome, Italy; 16 Istituto Pasteur - Fondazione Cenci Bolognetti, Rome, Italy; 17 Institute of Immunology, Hannover Medical School, Hannover, Germany; 18 Medical Clinic III for Oncology, Hematology, Immuno-Oncology and Rheumatology, University Hospital Bonn, University of Bonn, Bonn, Germany; 19 Laboratory of Molecular Genetics, Servicio de Análisis Clínicos, Hospital Universitario Río Hortega, Gerencia Regional de Salud de Castilla y León (SACYL), Valladolid, Spain; 20 Centro de Investigaciones Biomédicas en Red de Enfermedades Hepáticas y Digestivas (CIBERehd), Madrid, Spain; 21 Department of Surgery, The University of British Columbia, Vancouver, Canada; 22 BC Children’s Hospital Research Institute, Vancouver, Canada; 23 Department of Immunology and Pathology, Monash University, Melbourne, Victoria, Australia; 24 Charité – Universitätsmedizin Berlin, corporate member of Freie Universität Berlin, Humboldt-Universität zu Berlin, Berlin, Germany; 25 Department of Microbiology & Immunology, Columbia University, New York City, USA; 26 German Center for Infection Research (DZIF), Munich, Germany; 27 Institute for Biotechnology, Technische Universität, Berlin, Germany; 28 Department of Microbiology and Immunology, Peter Doherty Institute for Infection and Immunity, University of Melbourne, Melbourne, Victoria, Australia; 29 Australian Research Council Centre of Excellence in Advanced Molecular Imaging, University of Melbourne, Parkville, Victoria, Australia; 30 Institute for Research in Biomedicine, Università della Svizzera italiana, Bellinzona, Switzerland; 31 Instituto de Estudios Inmunológicos y Fisiopatológicos - IIFP (UNLP-CONICET), Facultad de Ciencias Exactas, Universidad Nacional de La Plata, La Plata, Argentina; 32 Peter Gorer Department of Immunobiology, School of Immunology and Microbial Sciences, King’s College London, UK; 33 National Institute for Health Research (NIHR) Biomedical Research Center (BRC), Guy’s and St Thomas’ NHS Foundation Trust and King’s College London, London, UK; 34 Department of Viral Immunology, Helmholtz Centre for Infection Research, Braunschweig, Germany; 35 Department of Medicine, The University of British Columbia, Vancouver, Canada; 36 Infection and Immunity Program, Department of Biochemistry and Molecular Biology, Biomedicine Discovery Institute, Monash University, Clayton, Victoria, Australia; 37 Laboratory of Translational Immunology, IRCCS Humanitas Research Hospital, Rozzano, Milan, Italy; 38 IRCCS, Istituto Giannina Gaslini, Genova, Italy; 39 Institute for Immunology, University Medical Center Mainz, Mainz, Germany; 40 Research Centre for Immunotherapy, University Medical Center Mainz, Mainz, Germany; 41 Institute of Molecular Biology and Pathology, National Research Council of Italy (CNR), Rome, Italy; 42 Immunosurveillance Laboratory, The Francis Crick Institute, London, UK; 43 Innate Immunity Unit, Department of Immunology, Institut Pasteur, Paris, France; 44 Inserm U1223, Paris, France; 45 Laboratory of Innate Immunity, Department of Microbiology, Infectious Diseases and Immunology, Charité – Universitätsmedizin Berlin, Campus Benjamin Franklin, Berlin, Germany; 46 Mucosal and Developmental Immunology, German Rheumatism Research Center Berlin (DRFZ), Berlin, Germany; 47 Cell Biology, German Rheumatism Research Center Berlin (DRFZ), An Institute of the Leibniz Association, Berlin, Germany; 48 Department of Medicine/Rheumatology and Clinical Immunology, Charité Universitätsmedizin Berlin, Berlin, Germany; 49 Institute of Systems Immunology, Hamburg Center for Translational Immunology (HCTI), University Medical Center Hamburg-Eppendorf, Hamburg, Germany; 50 Institut National de la Sante Et de la Recherce Medicale (INSERM) U1015, Equipe Labellisee-Ligue Nationale contre le Cancer, Villejuif, France; 51 Singapore Immunology Network (SIgN), Agency for Science, Technology and Research, Singapore, Singapore; 52 Institute of Transplant Immunology, Hannover Medical School, Hannover, Germany; 53 Regensburg Center for Interventional Immunology (RCI), Regensburg, Germany; 54 Chair for Immunology, University Regensburg, Regensburg, Germany; 55 Institut Necker Enfants Malades, INSERM U1151-CNRS, UMR8253, Paris, France; 56 Université de Paris, Paris Descartes, Faculté de Médecine, Paris, France; 57 AP-HP, Hôpital Necker Enfants Malades, Paris, France; 58 Department of Medicine I, Lighthouse Core Facility, Medical Center – University of Freiburg, Faculty of Medicine, University of Freiburg, Freiburg, Germany; 59 John van Geest Cancer Research Centre, School of Science and Technology, Nottingham Trent University, Nottingham, UK; 60 Centre for Health, Ageing and Understanding Disease (CHAUD), School of Science and Technology, Nottingham Trent University, Nottingham, UK; 61 Immunology of Aging, Leibniz Institute on Aging – Fritz Lipmann Institute, Jena, Germany; 62 Department of Medicine, Visceral and Thoracic Surgery, University Medical Center Hamburg-Eppendorf, Hamburg, Germany; 63 Department of Medicine, University Medical Center Hamburg-Eppendorf, Hamburg, Germany; 64 Hamburg Center for Translational Immunology (HCTI), University Medical Center Hamburg-Eppendorf, Germany; 65 Division of Molecular Oncology and Immunology, the Netherlands Cancer Institute, Amsterdam, The Netherlands; 66 Institute of Molecular Medicine and Experimental Immunology, Faculty of Medicine, University of Bonn, Germany; 67 Shanghai Institute of Immunology, Department of Immunology and Microbiology, Shanghai Jiao Tong University School of Medicine, Shanghai, China; 68 Translational Immunology Institute, SingHealth Duke-NUS Academic Medical Centre, Singapore, Singapore; 69 Institute of Experimental Immunology, University of Zurich, Zurich, Switzerland; 70 Department of Hematology, Oncology, and Tumor Immunology, Charité - Universitätsmedizin Berlin (CVK), Berlin, Germany; 71 Berlin Institute of Health (BIH), Berlin, Germany; 72 German Cancer Consortium (DKTK), partner site Berlin, Germany; 73 Centre for Innate Immunity and Infectious Diseases, Hudson Institute of Medical Research, Clayton, Victoria, Australia; 74 Department of Molecular and Translational Sciences, Monash University, Clayton, Victoria, Australia; 75 Peter Gorer Department of Immunobiology, King’s College London, London, UK; 76 Division of Allergy, Department of Dermatology and Allergy, University Hospital Schleswig-Holstein, Kiel, Germany; 77 Innate Immunity, German Rheumatism Research Center Berlin (DRFZ), Berlin, Germany; 78 Charité – Universitätsmedizin Berlin, corporate member of Freie Universität Berlin and Humboldt-Universität zu Berlin, Department of Gastroenterology, Infectious Diseases, Rheumatology, Berlin, Germany; 79 Friedrich-Alexander-University Erlangen-Nürnberg (FAU), Department of Medicine 3 – Rheumatology and Immunology and Universitätsklinikum Erlangen, Erlangen, Germany; 80 Deutsches Zentrum für Immuntherapie, Friedrich-Alexander-University Erlangen-Nürnberg and Universitätsklinikum Erlangen, Erlangen, Germany; 81 Institute for Immunology, Biomedical Center, Faculty of Medicine, LMU Munich, Planegg-Martinsried, Germany; 82 Experimental Immunology, Helmholtz Centre for Infection Research, Braunschweig, Germany; 83 Department of Hematology, Oncology and Rheumatology, University Heidelberg, Heidelberg, Germany; 84 Department of Hematology, Singapore General Hospital, Singapore, Singapore; 85 Executive Offices, National Cancer Centre Singapore, Singapore; 86 Vita-Salute San Raffaele University, Milan, Italy; 87 Experimental Imaging Center, IRCCS San Raffaele Scientific Institute, Milan, Italy; 88 Division of Molecular Immunology, Nikolaus-Fiebiger-Center, Department of Internal Medicine III, University of Erlangen-Nürnberg, Erlangen, Germany; 89 Department of Rheumatology and Clinical Immunology, Medical Center – University of Freiburg, Faculty of Medicine, University of Freiburg, Freiburg, Germany; 90 Center for Chronic Immunodeficiency, Medical Center – University of Freiburg, Faculty of Medicine, University of Freiburg, Freiburg, Germany; 91 Institute of Molecular and Clinical Immunology, Otto-von-Guericke University, Magdeburg, Germany; 92 Department of Neuropathology, Universitätsklinikum Erlangen, Germany; 93 Department of Rheumatology, Leiden University Medical Center, Leiden, The Netherlands; 94 Institute for Molecular Medicine, Goethe University Frankfurt, Frankfurt am Main, Germany; 95 Institute of Microbiology, ETH Zurich, Zurich, Switzerland; 96 School of Biomedical Engineering, The University of British Columbia, Vancouver, Canada; 97 Institute of Immunology and Immunotherapy, University of Birmingham, Birmingham, UK; 98 Michael Smith Laboratories, The University of British Columbia, Vancouver, Canada; 99 Division of Infection & Immunity, Institute of Immunity & Transplantation, University College London, London, UK; 100 Vaccine and Infectious Disease Division, Fred Hutchinson Cancer Research Center, Seattle, WA, USA; 101 Institute for Systemic Inflammation Research, University of Luebeck, Luebeck, Germany; 102 Clinical Trial Center Leipzig, Leipzig University, Härtelstr.16, −18, Leipzig, 04107, Germany; 103 Department of Immunology, IRCCS Bambino Gesù Children’s Hospital, Rome, Italy; 104 Radiation Immuno-Oncology Group, Center for Translational Cancer Research (TranslaTUM), Technical University of Munich (TUM), Klinikum rechts der Isar, Munich, Germany; 105 Department of Radiation Oncology, Technical University of Munich (TUM), Klinikum rechts der Isar, Munich, Germany; 106 Institute of Experimental Immunology and Hepatology, University Medical Center Hamburg-Eppendorf, Hamburg, Germany; 107 Department of Microbiology & Immunology, Immunology Programme, Life Science Institute, Yong Loo Lin School of Medicine, National University of Singapore, Singapore, Singapore; 108 School of Biological Sciences, Nanyang Technological University, Singapore, Singapore; 109 Department of Life Sciences, University of Modena and Reggio Emilia, Modena, Italy; 110 School of Biomedical and Health Sciences, RMIT University, Bundorra, Victoria, Australia; 111 Department of Biochemistry and Molecular Biology, Monash University, Clayton, Victoria, Australia; 112 Flow Cytometry Science Technology Platform, The Francis Crick Institute, London, UK; 113 Immunology Frontier Research Center, Osaka University, Japan; 114 Clinic for Orthopedics and Trauma Surgery, University Hospital Bonn, Bonn, Germany; 115 Mikrobiologisches Institut – Klinische Mikrobiologie, Immunologie und Hygiene, Universitätsklinikum Erlangen, Friedrich-Alexander-Universität (FAU) Erlangen-Nürnberg, Germany; 116 Charité Universitätsmedizin Berlin – BIH Center for Regenerative Therapies, Berlin, Germany; 117 Sanquin Research – Adaptive Immunity, Amsterdam, The Netherlands; 118 Institute for Medical Informatics, Statistics and Epidemiology (IMISE), University of Leipzig, Leipzig, Germany; 119 Department of Precision Instrument, Tsinghua University, Beijing, China; 120 Department of Preclinical Development and Validation, Fraunhofer Institute for Cell Therapy and Immunology IZI, Leipzig, Germany; 121 Institute of Biochemistry and Biophysics, Faculty of Biological Sciences, Friedrich-Schiller-University Jena, Jena, Germany; 122 Department of Medicine III, Technical University Dresden, Dresden, Germany; 123 Department of Pathology, Stanford University School of Medicine, Stanford, CA, USA

## Abstract

The third edition of Flow Cytometry Guidelines provides the key aspects to consider when performing flow cytometry experiments and includes comprehensive sections describing phenotypes and functional assays of all major human and murine immune cell subsets. Notably, the Guidelines contain helpful tables highlighting phenotypes and key differences between human and murine cells. Another useful feature of this edition is the flow cytometry analysis of clinical samples with examples of flow cytometry applications in the context of autoimmune diseases, cancers as well as acute and chronic infectious diseases. Furthermore, there are sections detailing tips, tricks and pitfalls to avoid. All sections are written and peer-reviewed by leading flow cytometry experts and immunologists, making this edition an essential and state-of-the-art handbook for basic and clinical researchers.

## Guidelines in the Time of COVID-19

The moments of great difficulties are those in which one can test if a marriage is truly stable, and, if overcome, greatly strengthen the union of a couple. The period that the marriage of immunology and cytometry has had to withstand, and continues to do so, is the one that makes your wrists tremble. But now, more than ever, has this scientific union shown its vital importance and strength. Indeed, the third edition of the Flow Cytometry Guidelines comes to light at a time when the international scientific community continues the battle that has started almost two years ago against the most dramatic threat that humanity has received since the last century, the pandemic caused by SARS-CoV-2, the virus responsible for COVID-19. We have all experienced first-hand the devastating effects of the pandemic, which for many months affected not only our work and activities, but also all aspects of our lives. From the very first moment, immunologists, infectivologists and virologists, along with thousands of other scientists, have with their joint efforts taken the field to understand some of the strategies the virus uses to evade an effective immune response and to activate a number of pathogenetic mechanisms that cause severe damage to the organism.

During these challenging times it has been more than impressive to witness a swift move from the first description of phenotypical modifications of peripheral blood lymphocytes, suggesting a strong inflammatory state that was indicative of the derangement of innate immunity, to an increasingly sophisticated identification of the specific T- and B-cell responses. Needless to say, counting, analysing or describing the role that flow cytometry techniques have had during these two years is almost immeasurable.

The previous versions of the Guidelines have been extremely well received by the community, being the most read articles in the *European Journal of Immunology*. The Guidelines have also been widely referred to, indicating that they are driving best flow cytometry practice within the immunological community. The third edition of the Guidelines was, as mentioned, prepared during a very difficult period, with ever greater enthusiasm and strength, with the aim of providing a document useful to our community of basic and clinical immunologists. Here, we have reorganized the content, by focusing on the phenotypes and functional assays, which were updated and expanded compared to the previous versions. To accommodate this, sections on advanced flow cytometry techniques, featured in the second version, are not part of this edition. Similarly, introductory chapter of the third edition is significantly reduced compared to the previous two editions – the content of the “classical” chapters *Principles of flow cytometry* and *Cell sorting* covered in the second version was not included into this version. This time round, condensed *Introduction* consists of key aspects to consider when performing flow cytometry experiments such as reproducibility, essential controls, data display and panel design in high-dimensional flow cytometry. Following *Introduction*, we have ordered the content by cell type and, within each cell type, highlighted human section followed by murine. To help researchers use this handbook to in addition study the function of a given cell type, *Functional Assays* are featured after the cell type in question and all sections contain novel pitfalls to avoid as well as top tricks. Another particular highlight of this edition is the *Clinical relevance statement* that will be a useful guideline for immediate application of the shown phenotypical or functional analysis in the context of e.g. COVID-19 or other disease types. This section also features examples from the clinic and discusses possible applications of flow cytometry in the context of a variety of autoimmune diseases, cancers as well as acute and chronic infections. I believe that this section will be of great help for clinical immunologists who already are or are about to initiate research focused on therapeutic targeting and will allow them to monitor a given cell subset in health and disease. Notwithstanding this special feature will certainly appeal to clinicians, we have given special consideration to what will be of particular interest to our loyal community of basic immunologists. In parallel to observing these amazing efforts in creating human immunology content of the Guidelines, it was nothing less impressive to witness how the authors of murine sections have worked tirelessly (and in many cases together with authors of human cell counterparts) to highlight key differences between human and murine cells featured in the *Key differences human vs murine* section. I believe that this section will allow the readers to not only have a better insight into phenotype differences between mouse and human immune cells, but will also be of help to those researchers who are keen to bridge the gap between basic and clinical immunology, allowing them to study immune system in both human and murine settings.

Finally, I owe great thanks to everyone who has helped us better this edition. We are indebted to all those who collaborated in the writing and revision of the text, first of all the authors. Then, to our fantastic referees who have helped us to critically review the content ensuring the published version is of highest quality and that it covers the most recent developments in the field. l would also like to thank the entire Editorial team, who played a major role in ensuring that Andreas Radbruch’s, Hyun-Dong Chang’s and my vision goes on. For this edition, Nadja Bakocevic (Deputy Managing Editor), Vanessa Boura (Associate Editor), Cate Livingstone (Managing Editor), and Laura Soto-Vazquez (Associate Editor) of the European Journal of Immunology, worked tirelessly ensuring that this revised version is a significant improvement. As these were tremendous efforts of our editors that have been working together with our fellow basic and clinical immunologists, it is only natural that such a job is never done, and I therefore invite you to send us your valuable feedback. I truly hope that you will find the presented content is helpful for day-to-day laboratory use as much as we do!

Andrea Cossarizza

## I Introduction

### 1 Essential controls and reproducibility in flow cytometry

#### 1.1 Overview

The construction of complex multicolor panels requires a detailed understanding of the instrumentation that is bigureeing used, including its limitations, and close attention being paid to fluorophore choice, Ag density, and Ab titration. Herein, we highlight the essential controls that should form part of any flow cytometry experiment, and issues relating to reproducibility that should be addressed when establishing good working practices within a research environment.

#### 1.2 Introduction

The rapid expansion of flow cytometry instrumentation and automation has driven an explosion in the use of this technology across a range of disciplines and indications. Although the development of “turnkey” instrumentation platforms that do not require the level of technical understanding to operate was required for the earlier platforms has greatly enabled access to this technology, it remains essential that the perceived ease of use does not compromise the appreciation for including the necessary essential controls to ensure robust data and for routinely applying and following necessary procedures that ensure reproducibility. The latter relies both on the practices of the laboratory performing the work and on the correct and comprehensive reporting of the data. However, controls are just one of several important experimental considerations in flow cytometry. Other examples include careful sample preparation, optimization of staining protocols, appropriate handling and storage of reagents and fluorophore products, as well as data acquisition such as doublet discrimination and robust and reproducible approaches for gating on relevant populations. With regards to the latter, several software-based approaches that remove the subjectivity of gating have been and are being developed.

Numerous resources and educational webinars covering all these subjects are available and so the aim of this article is only to highlight the key issues and considerations that should be made and direct the reader to additional, more comprehensive information that can be found elsewhere.

#### 1.3 Essential controls

Relevant controls are essential to “Good Flow Cytometry Practice,” without which it would be impossible to robustly generate and analyze data or interpret experimental findings. In a broad context, every experiment should contain three fundamental categories of controls, namely setup (instrument) controls, specificity (gating) controls, and biological controls [1, 2].Specifically:

##### 1.3.1 Setup/instrument controls/quality assurance.

Setup/Instrument Controls/Quality Assurance enable the instrument to be correctly set up on installation and at subsequent times thereafter. An essential component of any flow cytometry facility is the regular maintenance and quality control (QC) of the instrumentation. The fundamental purpose of such processes is not only to make sure that the instrument is working ok, but also to ensure longitudinal stability. Broadly speaking, these processes can be separated into three key steps, as defined by Perfetto et al. [3]:

- **Optimization** examines a wide array of critical instrument parameters: the efficiency and performance of optical filters, dichroic mirror reflection and transmission, the timing of lasers (laser delay), laser power, the length of time set to collect instrument signals (e.g., window extension), amplifier linearity, electronic noise, and synchronization of area and peak height signals (e.g., area scaling factor, ASF) [3].
- **Calibration** involves measuring/monitoring the dynamic range and photon efficiency of each photomultiplier tube (PMT) (e.g., detector). This can be achieved using stable hard-dyed fluorescent beads (either manufacturer supplied, or third party such as Rainbow beads). The use of four types of beads has been recommended: Quantum^™^ Simply Cellular^®^ beads (QSCBs), Cyto-Cal beads (or Duke beads), single-peak (1×) Rainbow beads, and unstained compensation beads (COMP beads) [3].
- **Standardization** tracks reproducibility and reliability on a daily basis in order to identify both existing and potential issues. Records of the daily monitoring should be kept electronically in order to better track trends and variances from established acceptable performance ranges and optical backgrounds.

These quality assurance processes ensure the correct and (crucially) reproducible performance of the instrument and provide confidence that any differences/changes in the sample that are detected are due to true biological events not to instrumentation issues. More comprehensive details on a framework that can be used to optimize, calibrate, and standardize flow cytometry for daily use and the periodic requirement for these, as well as advice on troubleshooting any issues have been considered in detail elsewhere [3, 4].

##### 1.3.2 Specificity/gating controls.

Specificity/Gating Controls allow specific staining to be distinguished from non-specific staining. Such controls are also used to establish the gates and regions that are used to identify and interrogate cell populations of interest. These controls are crucial for determining positive from negative populations, as well as the intensity of expression of given markers should this be part of the experimental design. Specifically,

- **Single-stained controls** are used to set the correct compensation value for the fluorochrome panel that is being used in the case of conventional flow cytometry. For spectral flow cytometry, the single-stained sample can be used to determine the contribution of each fluorophore in a mixed sample, using spectral deconvolution (unmixing) algorithms [5]. It is essential that the fluorescent “brightness” of the controls is as bright or brighter than the experimental sample, the background fluorescence for the different populations is equivalent, and the fluorochromes used for the control and sample values and the instrument settings used to analyze these populations are the same. It is also essential that the autofluorescence of the populations is the same and that sufficient events are collected. It is not possible to interpret data from multiparameter flow cytometry without including these essential controls [6].
- **Unstained/Autofluorescence controls** enable control for background autofluorescence and can either be unstained cells, or in some instances cells that are known not to express any of the Ags against which Abs are present in the panels being used. It should be appreciated that autofluorescence can change with “biology” and so the appropriate controls should be included for all untreated and treated populations.
- **Viability stains** are essential when analyzing viable samples, as non-viable (dead) cells are more likely to non-specifically bind Abs and be more highly autofluorescent. The inclusion of viability stains, of which there are very many, therefore enables the more definitive identification of true positive and true negative populations.
- **Gating controls** are key to correctly identifying the populations of interest. Although these have, for many years, been based on the use of isotype-matched control immunoglobulins, this approach has largely been superseded by the *“Fluorescence Minus One (FMO)”* approach [7]. The FMO control identifies spectral overlap of fluorochromes into the channel of interest and, as the terminology suggests, involves staining the cells of interest with all fluorochrome-conjugated Abs except one. More recently, the need for such controls, apart from when establishing a new panel in order to identify potential problem areas, has been questioned given advances in instrumentation and software [8-10]. The nature and relative merits of different gating controls, strategies, and software solutions have been discussed in great detail elsewhere [1, 2, 6-12].
- **Non-specific binding** negatively impacts the sensitivity of flow cytometry measurements and must therefore be minimized. In many instances, an isoclonic control, which involves staining cells with the fluorochrome-conjugated Ab in the presence of an excess of the identical unlabelled Ab, can reveal whether the conjugate is mediating non-specific binding to the sample. Although some continue to use an isotype control Ab to determine/exclude non-specific binding, the isotype control is not useful in this context [2]. Non-specific binding can be reduced using a number of different blocking agents (“Fc blockers”) or by using recombinant Abs that have been engineered not to express the Fc portion, for experiments that are analyzing cells that express Fc receptors.

##### 1.3.3 Biological comparison/experimental controls.

Biological Comparison/Experimental Controls are important as they provide biologically relevant comparisons relating to the difference between unstimulated/treated and stimulated/treated and/or insight into differences between “normal’ and “diseased.” These controls are therefore essential for determining if a treatment or condition induces a true detectable change in the observed biology of the cell population(s) of interest. Depending on the experimental question and design, these samples can also be used for setting relevant gates and regions for conditions. For example, unstimulated samples or healthy donor samples. In some cases, these can function as gating controls for identifying the biological consequences of different settings, treatments, etc. This category of control might also include a reference control that is either a defined cell type and/or stored samples that can be used to determine the longitudinal performance of the assay and/or instrument. If this control “fails,” then appropriate remedial action should be taken before undertaking additional experiments. These controls might also include fully characterized and validated cell lines that are known to be negative or positive for the relevant Ag(s), cells in which expression of the relevant Ag(s) has been knocked out or transfected to express/overexpress the relevant Ag, cells treated with commercial stabilization products, and unstimulated and stimulated populations that could also be generated in bulk and preserved using a range of cell stabilization solutions.

Although tempting to think otherwise, it is essential to appreciate that a well-performed and controlled flow cytometry experiment requires all the above considerations and controls to ensure the correct and robust generation and interpretation of the data that are generated.

#### 1.4 Experimental reproducibility

The reproducibility of published research (or rather the lack of it) has become an important and critical issue to the scientific, publishing, and research and development communities, as well as the funders of the work that is being performed. It is essential that research is undertaken in a way that delivers robust and reproducible findings. A landmark study conducted by Glenn Begley and his team in 2012 concluded that most cancer research studies were not reproducible, by alarmingly reporting that 47 of 53 published findings could not be replicated [13]. This observation has been followed by a number of additional reports, the summary of which is, in essence, that 77-90% of biomedical experiments are not reproducible [14-17]. The financial and other implications for biomedical research are clearly staggering considering the financial resources that are allocated to scientific research, and the impact that this lack of reproducibility has on the ability to translate findings into meaningful treatments and impact. Although many factors are likely to be involved (see below), research reagents, particularly Abs in the case of flow cytometry used in a wide variety of applications, have been at the center of the discussion.

Although clearly a complex issue, multiple factors contributing to a lack of reproducibility in research have been proposed [18]. These include selective reporting, pressure to publish, low statistical power or poor analysis, insufficient number of replications, insufficient oversight of the work being performed, lack of information on methodology, poor experimental design, lack of availability of original data, fraud, insufficient peer review, problems with efforts to reproduce data and technical expertise required to achieve this, variability of standard reagents, plus simple “bad luck.” Suggestions to improve reproducibility have included a better understanding of statistics, better mentoring/supervision and teaching, more robust experimental design and validation, provision of incentives to improve practice and formal reproduction of data, more external validation, more robust standards for evaluation and reporting by journals, and more oversight and routine checking of laboratory notebooks [18]. Inter-laboratory variations are also an issue, with Maecker et al. having reported that individual laboratories typically experience a ~20% CV in the data analysis, whereas data from a central laboratory only shows a variance of ~4% [19]. Again, these differences are likely to be driven, at least in part, by a lack, or suboptimal QC systems, an issue that is especially relevant for research-focused laboratories and environments.

With specific reference to flow cytometry, the potential for improving interlaboratory reproducibility of flow cytometry measurements and studies in general by consensual use of methodological approaches has been discussed in detail elsewhere [20]. Key questions that should be asked include (not in any order):

- Are my flow cytometry experiments reproducible – could/has another laboratory reproduce(d) my data and findings?
- Have I had problems reproducing experimental data reported in the literature, or data that I have seen presented at meetings?
- How can I ensure that the data I am reporting are “correct” and reproducible?
- Does my choice of Abs and controls, their titrations, and how I combine them as well as my instrumentation and instrument settings align with the needs of the experiment and best practice(s)?
- When was my cytometer last calibrated?
- Have PMT voltage and other relevant instrumentation Quality Control and optimization beads been used?
- Is my compensation strategy robust and appropriate/correct for the experiments being performed?
- Am I using the correct controls for gating on my population of interest?
- Am I using the correct statistical approaches?
- Am I maintaining adequate experimental records – could somebody repeat my experiment exactly based on my experimental records?
- What Quality Control procedures am I using for my instrument, reagents, and experimental assay?
- Do I have (and importantly, am I following) the Standard Operating Procedures (SOPs) for everything that have been established and approved in my laboratory…?

Complementing the extensive *“Guidelines for the use of flow cytometry and cell sorting in immunological studies*” published by the *European Journal of Immunology* in 2017 [21] and 2019 [22], the third edition of which is being presented herein, is A Special Issue of Cytometry Part A entitled “*Enhancement of Reproducibility and Rigor*” [23] and its associated Editorial entitled “*Drawing the Bow for Reproducibility*” [24] have considered the current landscape regarding the enhancement of reproducibility and rigor in scientific research, specifically in studies where cytometry is utilized.

#### 1.5 Standard operating procedures/quality management systems

Although the above issues go some way to ensuring robust data, all work needs to be undertaken in an environment that facilities and ensures reproducibility and thereby enables the translation of results into meaningful and impactful applications, for which appropriate QC and quality management systems (QMS) must be in place.

Systems, environments, and working practices to mitigate against issues of reproducibility and improve the rigor, reproducibility, and value/impact of basic and translational biomedical research are “routine” in clinical and commercial settings; however, such Quality Management Systems are less common in preclinical laboratory settings, despite these being particularly challenged with issues of experimental reproducibility. The introduction of Quality Control and Laboratory Quality Management Systems aligned to ISO 9001, Good Laboratory Practice (GLP), Good Clinical Laboratory Practice (GCLP), and Good Manufacturing Practice (GMP) into an idiosyncratic academic laboratory setting is challenging due to the procedures and documentation that are required, a high turnover of staff and prejudice, to name but a few [25]. Arguably, a more achievable approach for this setting would be to align work to Guidelines such as the WHO “Quality Practices in Basic Biomedical Research” [26] and the “Quality in Research Guideline for Working in Non-Regulated Research” published by the British Research Quality Association RQA [27].

The Enhancing Quality in Preclinical Data (EQIPD; originally called European Quality in Preclinical Data) consortium has developed a novel preclinical research quality system that can be applied in both public and private sectors and is free for anyone to use [28]. At the core of the EQIPD Quality System is a set of 18 key requirements that can be addressed flexibly, according to user-specific needs, and following a user-defined trajectory. The EQIPD Quality System proposes guidance on quality-related measures, criteria for adequate processes (i.e., performance standards) and exemplifies how such measures can be developed and implemented [28]. A practical approach for introducing a QMS into an academic setting has also been described by Hewera and colleagues [29], as has a pragmatic and risk-based quality system and associated assessment process to ensure reproducibility and data quality of experimental results while making the best use of the resources [30].

#### 1.6 Importance of correct reporting of data

Also integral to the delivery of reproducible science is the correct reporting of data, the importance of which cannot be underestimated as it is via this route that others typically base their own experiments and attempt to reproduce the data that has already been reported upon. The reproducibility of flow cytometry data is therefore critically dependent on correct reporting of these data and the provision of the key essential information on experimental design and other parameters that have been used to generate these data. It is for this reason that the flow cytometry community has defined the minimal amount of data that should be provided to understand and be able to reproduce the data via the MIFlow-Cyt [31] and MiSET RFC Standards [32], more information on which is provided elsewhere in these Guidelines.

### 2 MIFlowCyt compliance and data display of flow cytometry data in immunological research

#### 2.1 Overview

Basic research is often criticized to be non-reproducible. To ensure reproducibility of cytometry data it is the aim of experimental scientific journals to express standards which data should be minimally provided to understand the paper and in the best case to reproduce these data. This section describes the MIFlowCyt standard for reproducible flow cytometry, gives examples for good current data visualization, and highlights the necessity of providing example data for the readers in order to improve reproducibility of immunological science.

#### 2.2 Introduction - providing minimum information to warrant reproducibility of experiments

Lack of reproducibility is of great concern in biomedical research and rough estimates say that up to 50% of the results published are not reproducible, meaning billions or trillions of US dollars of funding money lost [33]. To reduce this problem, the MIBBI (Minimum Information for Biological and Biomedical Investigations) project was launched in 2008 [34]. Its goal is to provide comprehensive checklists for different types of experiments so that all essential information for repeating the experiment is provided. As of today, 39 standards are listed in the MIBBI project (https://fairsharing.org/collection/MIBBI) and a few of them are concerning immunological experiments. Table 1 and [31,32,35-40] provide examples of immunology-related Minimal Information standards.

These guidelines recommend which information needs to be provided in a publication, which controls should be used and presented, and how open access availability of original measurements should be provided; all in the effort to make research experiments and studies reproducible (see section I.1 Essential Controls and Reproducibility in Flow Cytometry). Many of these standards are under construction but for some finalized guidelines exist. Of particular relevance for flow cytometry are MIFlowCyt-EV [36] and MISet RFC [32], an update of MIFlowCyt for best practices in plant cytometry [41] is in work. MISet RFC is set very broad and covers under its umbrella a whole study from the design of an experiment to the interpretation of the result and embraces MIFlowCyt and other standards. Several of the guidelines or proposals have been adopted by journals, although not all enforce their application.

#### 2.3 MIFlowCyt compliance

Relevant for flow cytometry is MIFlowCyt (Minimum Information about a Flow Cytometry Experiment) [31]. This standard was defined by an international group of cytometry experts from bioinformatics, computational statistics, software development, and instrument manufacturers, from clinical and basic research. With the provided information, cross-experimental comparisons are possible. Several scientific journals, as first *Cytometry Part A,* have adopted these regulations, and also journals from the Nature Publishing Group, *PLOS One,* and others have accepted these standards. MIFlowCyt-compliant manuscripts should have a checklist table containing information on reagents, instrumentation, and experimental setup, including information on controls, gating strategies, among others (for details, see [31], Table 2). Importantly, it is required that original primary list-mode data are made publicly available in an open access database such as the FlowRepository. This allows to analyze published data by alternative methods and better understand the published material by the readers. In the following manuscripts, you can find examples for MIFlowCyt checklists with different MIFlowCyt score values and original FCS data in the FlowRepository for Flow [42, 43], Mass cytometry [44], and Full Spectrum flow cytometry [45]. Since October 2018 MIFlowCyt compliance and reposition of original data are mandatory for *Cytometry Part A* publications [46].

Good examples for comprehensive MIFlowCyt checklists are in the Optimized Multicolor Immunofluorescence Panels (*OMIP*) publications. This format was developed for *Cytometry Part A* [47, 48] in order to improve the quality of polychromatic flow cytometry experiments and for their reproducibility. The central issue of an OMIP is to demonstrate that the developed multiplexed panel has been optimized by testing different reagents and reagent combinations. Until now, nearly 80 OMIPs have been published including also OMIPs for Mass cytometry [44] and Full Spectrum flow cytometry [45] with the aims of (1) reducing the time to develop similar panels and (2) providing a starting point for the development of new panels, or (3) for optimizing existing ones (recent overview in [49]). OMIPs present unique reagent combinations, document the developing progress, explain the final choice, and should be useful to a wide range of readers. OMIPs are by nature MIFlowCyt compliant (see as examples: [44, 45, 50, 51].

**Figure 1.**
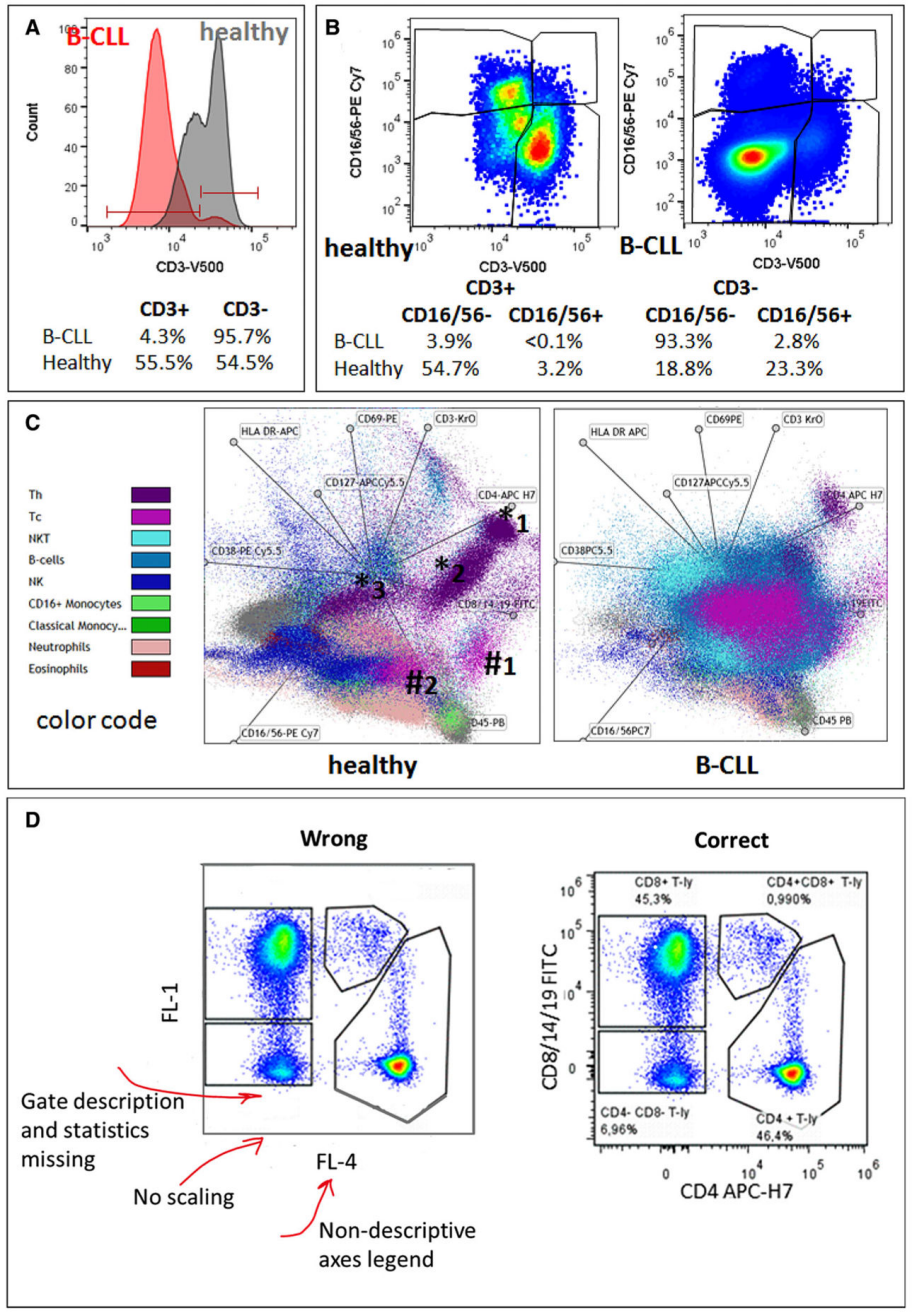
Uni-, bi- and multi-parameter and incorrect presentation of flow data. Comparison of two gender and age matched patients: a healthy one (67 years) and a patient with B-CLL (64 years). (A) 1D-histogram presentation of CD3 expression on lymphocytes (red: B-CLL, grey: healthy), (B) 2D-dot-plot presentation of CD3 expression on x-axis vs. CD16/56 expression on y-axis, (C) multivariate presentation of expression of 13 different Abs on 10 colors (OMIP-023 [50]) for nine different leukocyte subsets in a radar-plot. Abbreviations used: B-CLL (B cell chronic lymphocytic leukemia), Th (CD4^+^ T-helper cell), Tc (CD8^+^ cytotoxic T cell), NK (natural killer cell). (D) Incorrect and correct data presentation example (Data analysis: (A and B) FlowJo, V10.2; (C) Kaluza, Beckman-Coulter, V 1.1, (D) FCS Express V.6, De Novo Software).

Which data and information should be minimally displayed to fully understand research papers? First of all, the full gating strategy should be displayed so that the data analysis strategy used is obvious to the reader. This display should also include the position of positive and negative controls and essential statistical information, such as the percentage of cells in the region or gate or event count. Axis legends should include the marker (e.g., Ag) and the dye used, and show the scaling (log/lin) (Figure 1). Simple experiments with one or two colors can be presented in 1D histograms; this allows easy comparison of the expression level of the marker of interest for different samples (positive, negative controls, and samples) in overlay histograms. Within these histograms, positive and negative populations can be easily distinguished from one another. For better comparison, the histograms should be normalized, i.e., the maximum values set to 100%.

More common is a display using 2D pseudocolor density plots (Figure 1). Plotting the expression of two markers against each other allows a more precise distinction of double negative, single positive, and double positive, as well as weakly or strongly labeled subsets. The 2D-plot presentation also helps to identify errors of automated compensation for manual correction, as needed. Multicolor experiments are normally analyzed by a sequential gating strategy. A full gating strategy is performed in a step-by-step procedure (examples can be found in [50, 52]). To analyze discrete populations such as T-cell subsets within blood samples in a first step CD45 negative red blood cells (CD45 expression vs. scatter) are excluded. Furthermore, only lymphocytes are gated based on their scattering (FSClow, SSClow). By exclusion of CD3 negative B cells (CD16/56^−^) and NK cells (CD16/56^+^) only CD3 positive cells will be analyzed in the next step. By the expression of CD16/56 NKT-cells (CD3 vs. CD16/56) can be excluded from T cells. In a final step, CD4^+^ T helper cells and CD8^+^ cytotoxic T cells (CD4 vs. CD8) can be analyzed. This process is strongly driven by a priori expectation and knowledge of the scientist analyzing the data. That means the scientists will expect, e.g., to analyze within the T-cells at least four subsets: CD4^+^ CD8^−^ T-helper cells, CD8^+^ CD4^−^ cytotoxic T-cells, CD4^+^ CD8^+^ immature T-cells, and CD4^−^ CD8^−^ T-cells. But, within these subsets, additional T-cell subsets might be neglected, which will be taken into count by an automated approach. Keep in mind, by using small (conservative) gates instead of overlapping gates, disease-specific cells might be excluded already in the first step of the analysis, or novel subsets might not be recognized. Analyzing data by the conventional step-by-step method in sequential 2D-plots has several drawbacks, e.g., loss of information by the loss of rare cell subsets by pre-gating, and some marker combinations that might help to further subdivide a subset might not be analyzed.

#### 2.4 Minimal requirements for data display

The complexity of cytometric data requires careful consideration of how to display results in scientific presentations and publications in order to make them understandable “at a glance.” To easily reproduce published cytometric experiments, the used methods and results need to be described and presented comprehensively. Already back in 2004, a group of cytometry experts including the late Zbigniew Darzynkiewicz [53] recommended how cytometry data should be presented for a broad community [54] and which mistakes should be avoided. A unified way of data presentation enables to speak a common language and to convey the message more easily. Due to technological advances such as substantially higher level of complexity of flow cytometry experiments (many more colors), logicle display method of logarithmic scaling [55], or data reduction displays as well as new flavors of flow cytometry (30+ color flow cytometry, mass flow cytometry, imaging flow cytometry, and full-spectrum flow cytometry), these guidelines still apply. It is disappointing to see that even after over 19 years, flow cytometry data are still presented in an incomplete fashion even in highly prestigious journals, making their interpretation cumbersome or impossible.

Table 3 summarizes the key aspects of data visualization of flow cytometry data; an example is provided in Figure 1D. In brief, axes scaling and ticks should always be displayed for 1D and 2D histograms to allow estimation of brightness differences. Unfortunately, it is still not uncommon to show flow cytometry data without ticks and axes and we could present several very recent examples. This way data presentation is meaningless and can only show that two populations have different brightness or expression level but not by how large this difference is.

**Figure 2.**
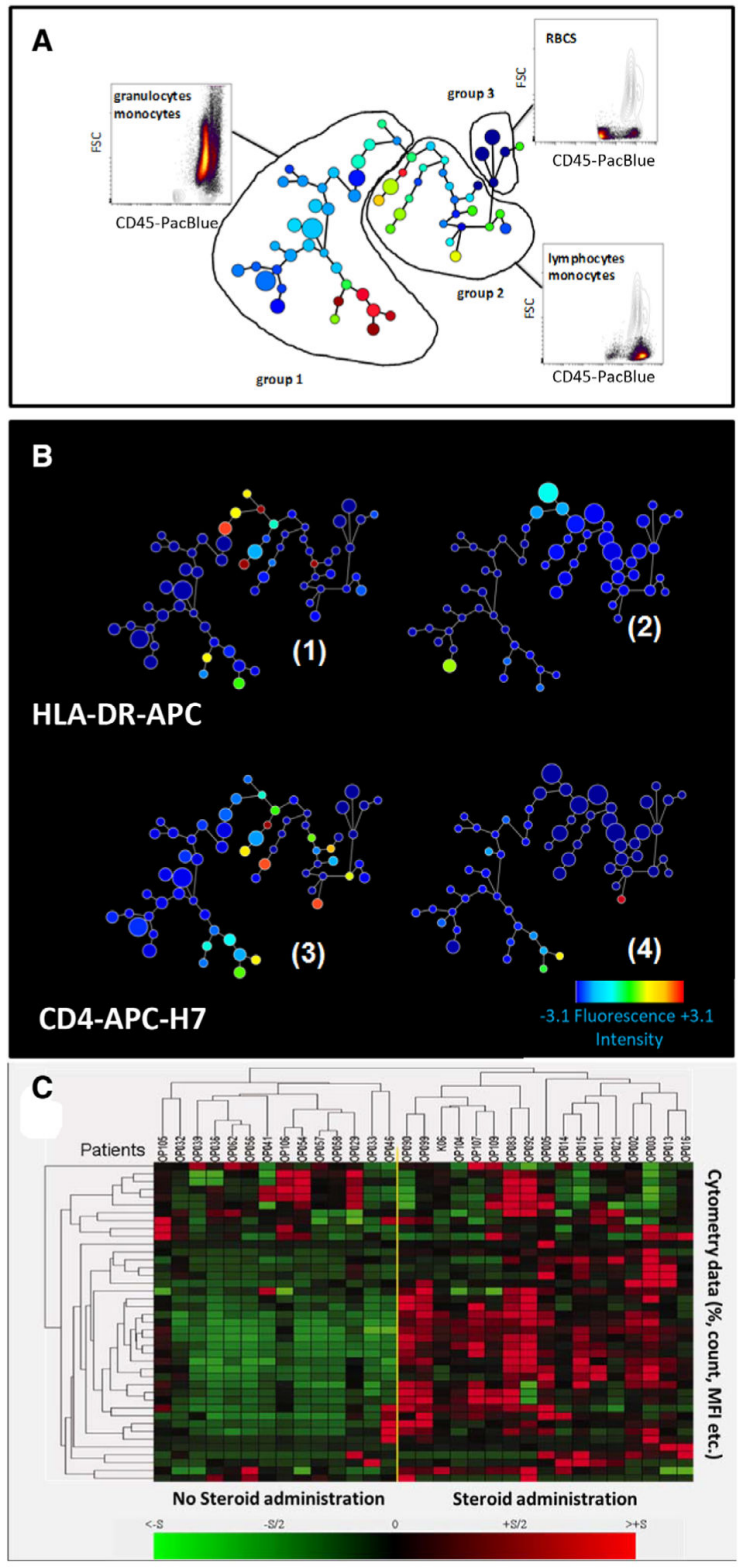
Semi-automated clustering and analysis of flow cytometric data by SPADE [32] **and hierarchical clustering**. (A) SPADE tree display and CD3 expression on blood cells from two male patients. Dot-plot analysis reveals groups of clusters (circles) belonging to the same cell type. (B) Color codes correlate with expression level from low (blue) to high (red) and size of the nodes correlate with cell frequencies (see also scale bar). Data of A and B are from a healthy (B1 and 3; 67 years) and a B-CLL patient (B.2 and 4; 64 years). (C) Hierarchical clustering of flow-cytometry data to visualize and distinguish immune response of pediatric patients (columns) who underwent elective cardiovascular surgery with (left of the yellow line) or without synthetic steroid administration (right) before surgery. PBL was immunophenotyped at day 1 after surgery. Flow cytometry parameters (MFI (mean fluorescence intensity) and cell counts) are displayed horizontally. Red indicates relative upregulation and green relative down-regulation of the respective parameter (see also scale bar). (Data and legend from [57]; reproduction with permission.) (SPADE analysis by Cytoscape, V 3.4.0, Nolan Lab; hierarchical clustering by free software Genes@Work).

**Figure 3.**
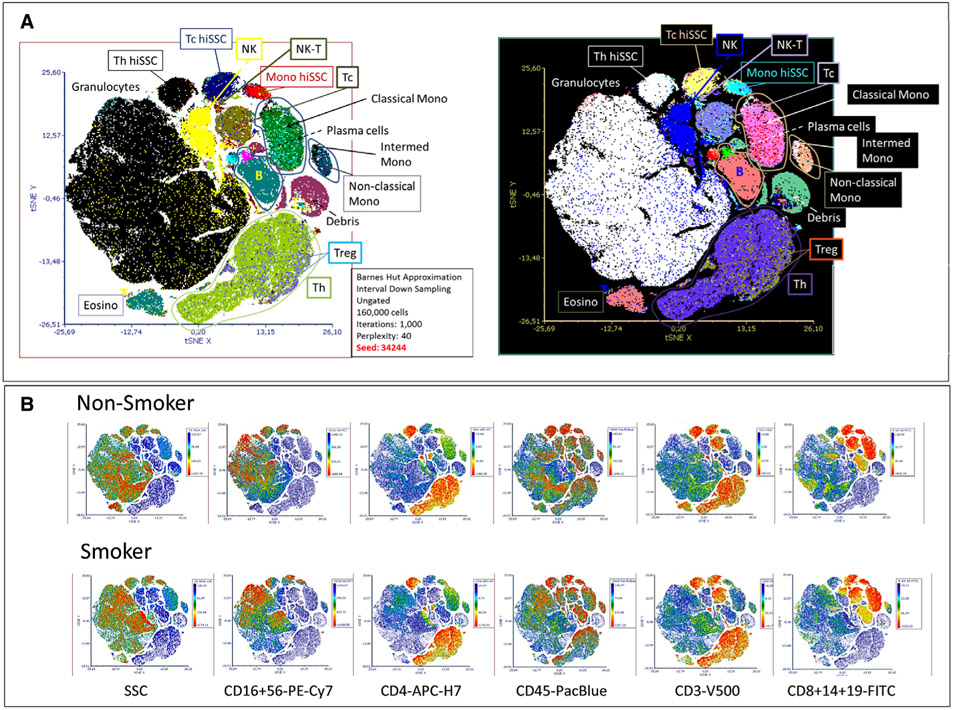
Semi-automated analysis of flow cytometry data by tSNE. (A) Sixteen-part differential of 10 individuals (5 smokers, 5 non-smokers) by OMIP-23 (10 colors, 13 Abs; [50]) showing the location of regular T-helper (Th) and cytotoxic T cells (Tc) with high side scatter (Th hiSSC, Tc hiSSC), T-regulatory cells (Treg), natural killer (NK) and NK-T cells on the tSNE map. Bottom center box contains information for calculating the tSNE plot. The image on the right shows the same figure in a inverted way with less red-green compound and better distinguishable for individuals with Deuteranomaly. (B) Heat map display of expression level of 5 activation markers in non-smokers and smokers and distribution of cell count (All). Scale bars right of each tSNE plot show color coding of fluorescence intensity or cell count levels. (Data of individuals from the LIFE study [58]; data analysis by FCS Express V.6, De Novo Software. Exemplary data and gating examples can be found in the supplementary materials and FlowRepository link of reference [50]).

1. Axis legends should include the parameter measured, the name of the dye used, and/or the emission frequency range analyzed and should show the scaling (log or lin). If the intrinsic fluorescence was analyzed then the axes legend should only display the emission spectrum range analyzed (e.g., 550-575 nm). For the selection of tag names, it is recommended to use the tag dictionary that standardizes the nomenclature of the numerous commercially available tags, may they be fluorochromes or metal isotopes [56].
2. Gating strategies to identify specific cell population need to be displayed (also a MIFLowCyt requirement). These strategies should be made in a way that they are easy to follow. Ideally, cell numbers or percentage values in the gates should be shown.
3. Color coding of different populations is useful but try to avoid red-green combinations for better distinction (see next paragraph on selecting the correct colors).
4. Display of appropriate controls: FMO, positive/negative cells, stimulated/unstimulated, blocking of binding, secondary Ab only, as appropriate.
5. For full spectrum flow cytometry, the display of emission spectra can be useful [55].
6. If clustering algorithms are used (see next paragraph) for data reduction show Fluorescence intensity distribution for each dye/wavelength range with scaling and the color-coded relevant clusters with explanation which cell type it is (and frequencies) (Figures 2 and 3 and [57, 58]).

Many of the data from points 4, 5, and 6 can be seen in the Supporting Information.

#### 2.5 Correct use of colors

In many cases, it is advantageous to use different colors to transport additional information. However, when selecting color combinations, it is often neglected that a high percentage of the human population is weak or is even blind in distinguishing certain colors or unable to see colors at all. Around 10% of males and 0.4% of females have some kind of color blindness. Most common is Deuteranomaly (6%/0.4%) and mainly green red discrimination is affected or not possible at all (https://en.wikipedia.org/wiki/Color_blindness#cite_note-62). The best way to convey information and allow barrier-free seeing is by using a grey-scale for coding instead of colors [59]. However, that might not look very attractive. For selecting the right colors that allow for most and a list of freely available software, the recent perspective from Crameri et al. [60] is recommended. (See also Figure 3A with example for two different color displays. The right version is easier readable with Deuteranomaly.)

#### 2.6 Data reduction and data analysis tools

With the constant increase of the complexity of cytometric measurements and data (in the past year several standardized OMIP protocols with 28 colors [61] and even 40 colors [55] with Full Spectrum cytometry became available. Therefore, there is also a need for algorithms to analyze and visualize these complex data.

Manual analysis of highly complex multi-dimensional data obtained by flow cytometry requires special software skills, gating knowledge, and time, and can be quite laborious. Still, manual gating is considered by most cytometrists to be the "gold standard," although semi-automated algorithms exist. Some basic rules for data visualization allow presenting these data in an intuitively understandable format. Here, we will show only examples for some of the popular tools. For a recent overview of the most popular data analysis tools, see [9].

To avoid biases by manual analysis of highly complex flow data software tools are used that work partly operator independent. This stresses also the importance of the reproducibility in complex, (semi)-automated data analysis [62]. O‘Neill and Brinkman [63] have suggested that certain data besides compensation, gating details, and mathematical algorithms should be shared for reproducible flow cytometry bioinformatics. These data include the source code, and free availability of the software (for details see: https://onlinelibrary.wiley.com/doi/10.1002/cyto.a.22804). One major aim is to make flow cytometry data easily accessible to the users by open-access databases for flow data (e.g., *FlowRepository)*, as well as the code sources. A series of data sets have already been provided by the *FlowCAP* (Critical Assessment of Population Identification Methods) project, comparing different mathematical models and automated methods for analysis. The cytometry community has already made great steps toward reproducible research by standardizing instrumentation, measurement, and data analysis, but still looks forward to optimize the reproducibility in different cytometry fields.

One example for a user-friendly visualization of multidimensional data at one glance is the radar plot (e.g., provided as a visualization tool in the Kaluza ^®^ software by Beckman-Coulter), which plots pregated subpopulations in a multiparameter way (Figure 2C); this allows analysis of the heterogeneity of the pregated populations and to identify new subpopulations (for further details see [22]).

Besides manual analysis and its visualization, several methods exist to perform software-assisted, unsupervised, or supervised analysis [64]. For example, using several open-source R packages and R source codes often requires manual pre-gating, so that they finally work just as a semi-automated computational method. For identification of cell populations, e.g., *FLAME* (suitable for rare cell detection based on clustering techniques), *flowKoh* (self-organizing map networks are produced), or *NMFcurvHDR* (density-based clustering algorithm) are available [65]. Histograms (*2DhistSVM, DREAM-A, fiveby-five*), multidimensional cluster maps (*flowBin*), spanning trees (*SPADE*), and tSNE (stochastic neighbor embedding) maps are suitable visualization tools for sample classification [64, 65, 66]. To find and identify new cellular subsets of the immune system in the context of inflammation or other diseases analysis in an unsupervised manner, such as by SPADE (spanning-tree progression analysis of density-normalized data) [67] can be a better approach.

SPADE is a density normalization, agglomerative clustering, and minimum-spanning tree algorithm that reduces multidimensional single-cell data down to a number of user-defined clusters of abundant but also of rare populations in a color-coded tree plot. In near vicinity, nodes with cells of similar phenotype are arranged. Therefore, related nodes can be summarized in immunological populations determined by their expression pattern. SPADE trees are in general interpreted as a map of phenotypic relationships between different cell populations and not as a developmental hierarchical map. But finally SPADE tree maps help to (1) reduce multi-parameter cytometry data in a simple graphical format with cell types of different surface expression, (2) overcome the bias of subjective, manual gating, (3) resolve unexpected, new cell populations, and (4) identify disease-specific changes (Figure 2A and B). Other ways for comprehensive analysis and display of complex data by unsupervised approaches can be found in ref. [68] and include Heatmap Clustering (Figure 2C; for details see captions and ref. [57]), viSNE/tSNE (Figure 3), and Phenograph, and FlowSOM [69]). Figure 3 shows an example of tSNE display of immunophenotyping data (10-colors, 13 Abs) from 10 individuals (five smokers, five nonsmokers). The position of the various leukocyte types in the tSNE map can be color-coded based on their Ag expression from 2D dot-plots (Figure 3A) and sufficient information should be provided to reproduce the calculations. Then (Figure 3B), for example, Ag expression levels for the different patient groups can be visualized (for more detail see captions).

Data reduction and display aids also improved visualization of between-group differences. A useful is hierarchical clustering of cytometry data that indicates color differences [57] (Figure 2) or color intensity differences [70] highly discriminative parameters. These can then be further visualized using SPADE or tSNE display. There are several new tools such as Phenograph, FlowSOM, and others for patient or experiment group discrimination that are explained in detail elsewhere.

Finally, irrespective which dimensionality approaches are used it is essential that all preprocessing information is provided (pregating procedures, data normalization) either with the graphs or as Supporting Information. Also, authors should provide information of the calculation of the SPADE, tSNE and other graphs (e.g., *n* iterations, perplexity, *n* nodes; Figures 2 and 3). Also, software tools used have to be named and in case of own development also made available for the readership.

### 3 Panel design in high-dimensional flow cytometry

#### 3.1 Overview

The characterization of the complex nature of immunological processes in health and disease requires multi-dimensionality as well as high resolution to detect all targets of interest. While the availability of novel technologies such as mass cytometry by time of flight (CyTOF) and single-cell RNA sequencing (sc-RNAseq) have greatly increased the number of features (protein and/or transcript) that can be measured at the single-cell level, fluorescence-based flow cytometry remains a primary tool for immunophenotyping due to its low cost, high dynamic range, and high throughput. Furthermore, the most recent generation of instruments with five or even more spatially different laser lines allows the detection of 40 parameters, with up to 50 on the horizon (based on personal communication).

Although the general principles of experimental design have not changed (for review, see [71, 72], reliable fluorescent panels of more than 10 parameters require not only a more thorough and systematic planning to ensure optimal resolution of all markers even at low Ag expression, but they also critically depend on validation and controls as a means to avoid misinterpretation of data. Within this section, we describe a step-by-step approach for panel design based on the concept of the spillover spreading matrix (SSM), pointing out important considerations for fluorochrome-Ag combinations and address some of the most common misconceptions and caveats. In addition, we outline key steps in visual quality control of the obtained data to ensure a meaningful subsequent multidimensional data analysis.

#### 3.2 Introduction

Most commonly, fluorescent flow cytometers dedicate one detector to the measurement of one fluorophore and use a compensation-based approach to correct for spectral overlap between the different fluorophores used. Improvements in electronics and the usage of multiple spatially separated laser lines have resulted in the latest generation of instruments that can measure up to 28 fluorescent parameters (such as the BioRad ZE5 or the BD FACSymphony) [61]. In turn, spectral cytometry instruments have been developed that detect every single fluorochrome across all available detectors, thus measuring a complex composite spectrum for every cell, with individual signals being separated by spectral unmixing algorithms (originally developed at Purdue University and now commercialized by Sony Biotechnology as well as Cytek Biosciences) [5, 73]. Currently, these instruments have reportedly been used for the measurement of up to 40 parameters [45]. The availability of new dyes, will advance the field and push these limits toward 50, and possibly even beyond. While this section focuses on conventional, compensation-based flow cytometry, most of the principles discussed are applicable to spectral cytometry as well.

Systematic panel design for a high-dimensional experiment requires multiple considerations. Inevitably, the used fluorochromes will show some degree of spectral overlap into more than one detector. The detector intended to capture the major emission peak of the respective fluorochrome is usually called the primary detector, and the secondary detector(s) is (are) the one(s) collecting the spillover. The mathematical process used to correct for spectral overlap is termed compensation (See Chapter II, Section 1- Compensation in [22] and [74]), and reports a percent value describing the relative fluorescence detected in the secondary detector compared to the primary detector. This signal portion is subtracted from the total signal detected in the secondary detector. A common misconception is that the magnitude of the compensation value is used as a representation for the amount of spectral overlap between fluorophores, while in fact the compensation value is highly dependent on detector voltages [75].

**Figure 4.**
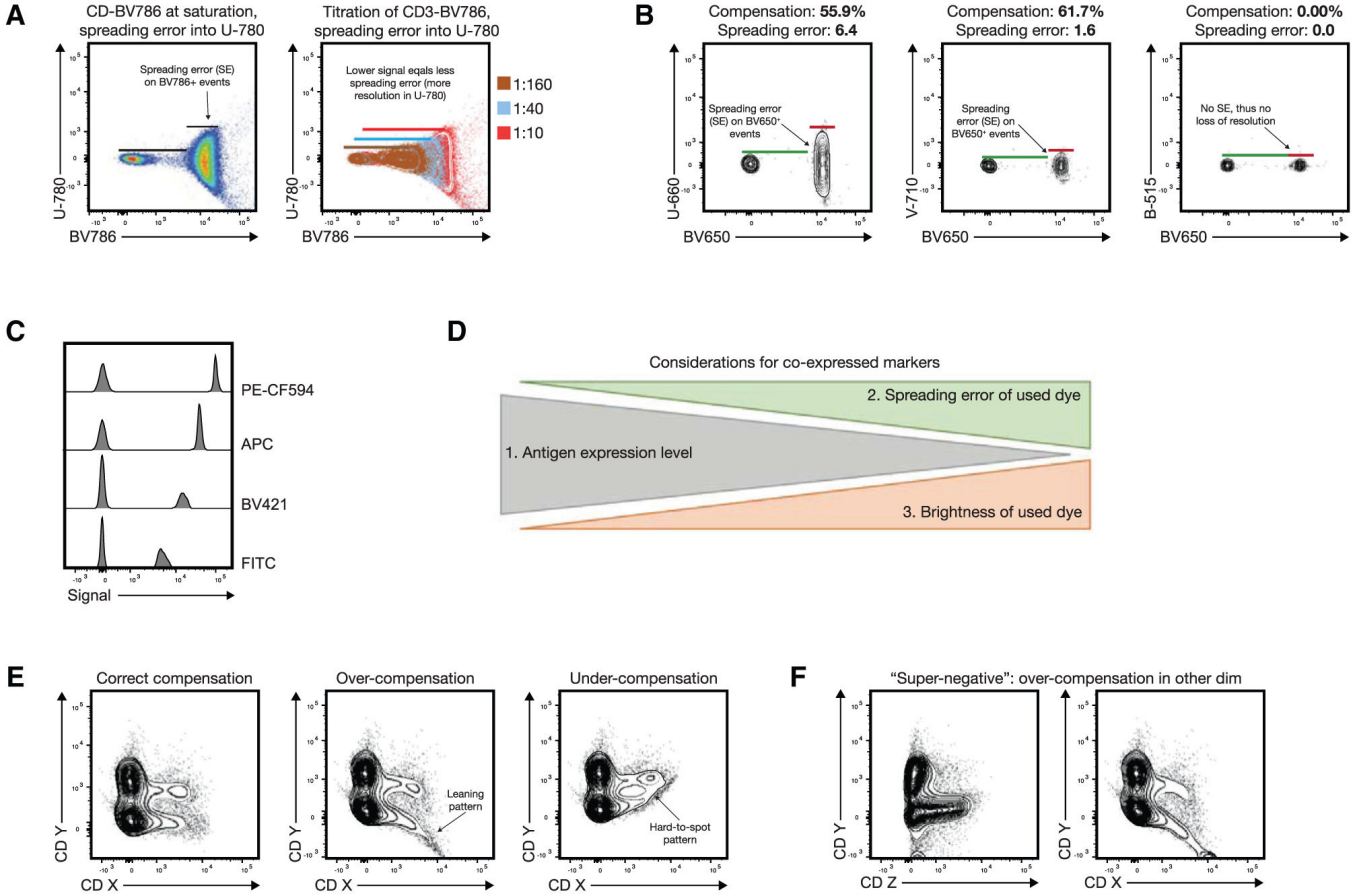
Spreading error and fluorochrome brightness in panel design and common compensation artifacts in quality control. (A) A typical example of spreading error is illustrated: BV786 shows significant spectral overlap in the U-780 detector (excitation by UV laser), which manifests as visible spread of the positive population. The relative loss of resolution on this population compared to the negatives is indicated by black bars on the left plot. Right plot shows how spreading error is proportional to signal intensity, and decreases with lower titers of the respective Ab. (B) The absolute compensation value and spreading error are not directly related, as exemplified for BV650+ events in different detectors (spreading error and compensation values for each combination are displayed above the plot). (C) Examples of staining intensities for different fluorochromes: FITC (dim), BV421 and APC (medium), and PE-CF594 (bright). Note that fluorochrome brightness can be instrument-specific. (D) Overview on the critical considerations for fluorochrome assignment for co-expressed markers. Highly expressed targets should preferably be paired with dim fluorochromes generating little spreading error. Dimly expressed (or unknown) targets should be paired with bright fluorochromes and utilize detectors that receive little spreading error. Numbers 1–3 indicate the relevance of the considerations. (E) and (F) show erroneous patterns that usually indicate incorrectly compensated data: (E) example of a correctly compensated plot, and respective over- and undercompensation of marker CD X into detector for CD Y. (F) Example of an incorrectly compensated population CD X (right plot) appearing as “super-negative” population if displayed against an unrelated detector measuring CD Z (left plot). The erroneous pattern is only visible if displayed against the detector measuring CD Y.

The most useful metric in this context is the so-called spreading error (SE), which was first described by the Roederer laboratory at NIH [76]. In short, the spreading error quantifies the spreading that the fluorochrome-positive population (in the primary detector) shows in any secondary detector. This increased spread (as measured by SD of the positive population) is sometimes erroneously attributed to compensation. In fact, compensation does not generate the spreading error, but rather makes it visible at the low end of the bi-exponential or logarithmic scale (Figure 4A, left panel). Spreading error is a consequence of the imprecise measurement of fluorescent signals at the detector (typically a photomultiplier tube (PMT)), which show some variance due to the Poisson error in photon counting.

In short, there are three key aspects of spreading error that need to be considered for panel design: First, spreading error is proportional to signal intensity, i.e., the brighter a signal in the primary detector, the more pronounced the spreading error in the secondary detector will be (Figure 4A, right panel). Second, spreading error reduces the resolution in the secondary detector, i.e., the detector that is collecting spillover (Figure 4B). Third, spreading error is additive, i.e., if a detector collects spreading error from multiple different fluorophores, the overall loss of sensitivity will be more pronounced (Table 4).

Besides considering spreading error, which will be discussed in more detail in the next section, other relevant aspects of panel design include the relative expression level of target Ags per cell, co-expression of target markers, and the relative brightness of the used fluorochromes. Importantly, the consideration of spreading error is overall more relevant than fluorochrome brightness if dealing with co-expressed markers (Figure 4C and D). Furthermore, for any high-dimensional fluorescence experiment the quality of single-stained controls is of utmost importance, thus these have to follow the four basic rules as described in detail in Chapter II Sections 1.3 (Measuring SOVs/compensation controls) and 1.4 (Compensation controls) and in [7]. Finally, the chemical properties of the used dyes can impact complex panels, as an unexpected dye-dye interactions or dye-cell/dye-buffer interactions can change the fluorescence spectrum of a given dye (also see “Top tricks” of this chapter). This aspect needs to be addressed by using appropriate controls, which will be discussed together with spreading error in the next section.

#### 3.3 Principles of the technique being described

The SSM is a fundamental tool for successful panel design. It is specific for each instrument and provides comprehensive information on the relative contribution of any fluorochrome to spreading error in secondary detectors, and the relative loss of resolution in all the available detectors. As such, the SSM provides a way to tackle spreading error in a systematic manner. It is important to note that the extent of spreading error cannot be predicted from the corresponding value in the compensation matrix, which is exemplified in the plots displayed in Figure 4B. Furthermore, an SSM can also be calculated for spectral flow cytometers, and be used in a similar way for panel design as described below.

The SSM can be calculated from single-cell stained controls in a common data analysis package, FlowJo (version 10.4 and higher), or manually using the formulas provided by Nguyen et al. [76]. The information on spreading error obtained from the SSM can be translated into panel design in two ways: First, the SSM highlights individual fluorophore-detector pairs with high spreading error, which in turn should be used for mutually exclusive markers (e.g., expressed on different cellular lineages such as CD3 for T cells and CD19 for B cells) as in this case spreading error will not interfere with detection of either signal. Second, the SSM can be used to assess the additive loss of resolution in a secondary detector by calculating the column sums, and to assess the additive contribution of spreading error from a single fluorochrome across all detectors by calculating the row sums. An example of an SSM and how to interpret it is shown in Table 4 and described in “Experimental workflow.”

In many applications, researchers aim to target as many markers of interest on the same cell type as possible. Hence, for these applications, the Ag expression levels play an important role and need to be assessed beforehand—either experimentally or by utilizing published work. Useful resources in this context are optimized multicolor immunophenotyping panels (OMIPs) (See Chapter VIII, Section 3 Analysis presentation and publication (MIFlowCyt) in [22]), which usually show raw data of every Ab in their Supporting Information [77]. Using the information of Ag density, low-expression Ags should be detected in channels receiving little spreading error and fluorochromes generating large spreading error can be used for their detection, as this will decrease the relative spreading error (which is proportional to signal intensity). In turn, highly expressed Ags should be paired with fluorochromes generating little spreading error. Alternatively, one can assign highly expressed targets to detectors that receive a lot of spreading error, as a bright signal will typically still remain above the spreading error. A step-by-step approach for this process is outlined in the section “Experimental workflow” below.

In order to draw accurate conclusion and to avoid interpreting artifacts that result from spreading error, validation of Ab combinations and using the right controls is mandatory. In most cases, and especially for markers with unknown Ag expression levels FMO controls are required as they can help to identify gating boundaries, especially in detectors with spreading error [72, 78] (see Chapter III 1.2 Fluorescence spreading into the channel of interest: Fluorescence minus one controls before you start controls in [22]). However, it is important to note that FMO controls cannot account for unspecific binding of the Ab that it controlled for, which can cause a shift of the entire negative population in the fully stained sample that is absent in the FMO control. In this case, either a biological control is required, or one can use another cell type in the same experimental sample as a gating control. Isotype controls can serve the function to identify staining issues, especially when secondary Abs are used. Unstained controls have historically often been used to give information about the background autofluorescence of the measured cells, but these controls are of little use in most complex polychromatic experiments.

Finally, high-dimensional cytometry data can only partially be analyzed by traditional manual gating, but rather benefits from using computational data analysis approaches. Prior to this, appropriate quality control and preprocessing of the data is mandatory, as specified below. For details on computational analysis techniques, we refer the reader to several recent reviews [64, 79, 80] and Chapter VII in [22].

#### 3.4 Applications

Multidimensional flow cytometry with up to 40 parameters enables a deeper phenotyping and characterization of the immune system, which is required as cellular subsets require more and more markers for accurate definition [81]. Besides basic research, clinical research can especially benefit from this analysis as a high amount of information can be extracted from limited, and thus precious, sample sources such as human tissue biopsies. Especially for longitudinal high-content immunomonitoring of big patient cohorts, multidimensional flow cytometry serves a fast and highly sensitive tool to correlate responses and observe changes of treatment as the basis to predict outcome of the myriads of immunotherapeutic approaches to treat diseases. The computational approaches allow for interrogating large data sets generated in these types of studies and enable the unbiased analysis of the data, possibly leading to the detection of rare cell types and can be of predictive value for treatment outcome.

#### 3.5 Experimental workflow

Here, we describe the key steps that should be taken for a systematic panel design approach.

1. Define the experimental hypothesis and the relevant cellular populations (e.g., CD8^+^ T cells)
2. Make a list of lineage markers that are necessary for consistent identification of the populations of interest (e.g., CD3/CD8 and CD45 for CD8^+^ T cells).
3. List all target markers of interest and categorize expected expression patterns and (if known) Ag density into low, medium, and high.
4. Generate an SSM on your instrument by running single-stained controls with all desired fluorochromes and calculating the SSM in FlowJo or another suitable analysis program.
5. Look for the three highest values in the SSM and assign the corresponding fluorochromes to mutually exclusive Ag targets, i.e., targets not expressed on the same cell (in our example SSM in Table 4 the most problematic pair would be BUV563 spread into the YG-586 PE detector).
6. Calculate the row sums in the SSM. The fluorophores with the lowest row sum overall contribute the least spreading error to your experiment—these should be assigned to your lineage markers, e.g., CD3 and CD8 for a CD8 T cell-centric analysis (in our example SSM in Table 4 this would be BV421 and BUV395).
7. Calculate the column sums in the SSM. The detectors with the lowest column sums receive the least amount of spreading error—these detectors are suitable for dim or unknown target markers (in our example SSM in Table 4 good examples would be the B-515 and V-510 detectors). Utilize bright fluorochromes for these Ags, if possible. The detectors with the highest column sums receive more spreading error—for these detectors perform preliminary experiments to assign target markers that deliver a bright enough signal to be above the spread (in our example SSM in Table 4 this would be YG-586 and YG-610 detectors). However, one has to keep in mind that there might be a single contribution that drives the total spreading error in a detector, and if not used on the target cell, this can improve the total spreading error received (e.g., in our example SSM in Table 4 the contribution of BUV661 and BUV563 to the YG-586 detector).
8. Run a test experiment including all relevant FMO controls. Perform data analysis and quality control as outlined in the next section.

#### 3.6 Data analysis

For general concepts of computational analysis of high-dimensional single-cell data, we refer the reader to Chapter VII “Data handling, evaluation, storage and repositories” of the guidelines in ref. [22]. Within this section, we focus primarily on quality control aspects prior to data analysis.

Most technical artifacts occur when samples are acquired over multiple days (i.e., batch effect), however, sometimes they also happen within one experiment due to the lack of appropriate controls or inconsistencies in instrument handling. In the authors’ experience, a common cause of artifacts in fluorescent cytometry is incorrect compensation, which in turn is mostly due to poorly prepared single-stained controls. To pinpoint such mistakes, visual inspection of N×N views of the final data should be performed, with N being the number of fluorescent parameters acquired, i.e., every marker against every marker. Within these plots, one should screen the data for typical erroneous patterns such as “leaning” triangular populations and “super-negative” events. Examples patterns are given in Figure 4E and F.

Sometimes fluorescent signals vary across different experimental days or even within one experiment even though the same staining panel was applied. Correct data transformation can help to diminish this effect [82]. Different transformation approaches such as the biexponential, arcsinh, and hyperlog display can be used, and the optimal transformation depends on the specific data and cannot always be computationally predicted [83]. It is important to note that appropriate transformation settings are also key for successful computational analysis of the data.

Dead cells, doublets, or staining artifacts, e.g., by Ab aggregates, can appear as false-positive data points or outliers in the analysis, potentially leading to wrong interpretation of the data. Thus, it is important to exclude these prior to unsupervised computational analysis by appropriate pregating or “data cleaning.” Depending on the immunological question asked a pregating on the population of interest can be part of the preprocessing and may speed up the computational procedure of the analysis (e.g., per-gating and exporting of live singlet CD45^+^ CD3^+^ cells). Even though conventional manual gating may not be suitable to capture all the correlations between the up to 28 fluorescent parameters, it still serves as an important quality check before, during, and after the computational data analysis.

#### 3.7 Advantages

Thorough panel design, not only for multiparametric flow cytometry panels, will award the researcher with robust and reproducible flow cytometry data with a satisfying resolution also of dimly expressed markers. Even though the optimization of a panel may appear time-consuming and requires various controls to assure reliable interpretation, it will save time in the downstream analysis and interpretation of the generated data. Usage and correct interpretation of an SSM will improve the process tremendously. It may not be obvious at first, but cost will be reduced, as the unnecessary repetition of experiments due to non-interpretable data will be minimized.

#### 3.8 Pitfalls

Pitfalls in high-dimensional fluorescent cytometry often arise from inappropriate planning of experiments and lack of controls. This can be avoided by systematic panel design and the inclusion of FMO controls as described above. Also, an inherent disadvantage is the necessity to obtain single-cell suspensions, which disrupts the natural architecture and interaction of cells in situ. Several emerging techniques allow high-dimensional cytometric measurements directly within tissues, as has been shown by histocytometry [84] or the recent commercial release of an imaging CyTOF system (Hyperion, Fluidigm) [85], or spatial profiling of transcripts (commercialized for example by 10× genomics or Akoya Biosciences [86].

#### 3.9 Top tricks

1. It is important that the detector voltages of the used flow cytometer have been optimized using an appropriate technique. The most widely accepted approach for this is a voltage titration [3], which will determine the minimally acceptable voltage yielding optimal resolution for each detector. Voltages should not be adjusted solely for the purpose of lowering compensation values [87].
2. To deal with spreading error beyond the above-mentioned approaches, one can utilize the fact that spreading error is directly proportional to the signal intensity. If assigning a fluorochrome to a lineage marker showing high and bimodal expression (e.g., CD8), one can utilize lower Ab titers (below saturating concentration) to lower the positive signal and in turn the spreading error generated. However, it is important to note that this approach requires consistent staining conditions in terms of cell numbers, staining temperature, and staining duration.
3. Many recent fluorochromes are based on organic polymers, which can under certain conditions show interaction due to their chemical properties. To alleviate this issue some manufacturers have released commercial buffers that are designed to minimize these unspecific interactions, and thus it is strongly encouraged to use these buffers whenever more than one polymer-based dye (e.g., all Brilliant Violet and Brilliant Ultra Violet dyes, SuperBright dyes, etc.) is included in a staining.
4. When performing experiments with staining and acquisition over several days, it is recommended to follow best practices for consistent setup of the used instrument [3] and to include a reference sample (which should be a replicate sample of the same donor, e.g., several aliquots from a healthy human Leukopak) on every experimental day in order to detect irregularities in staining, compensation or transformation. These control samples can then be overlayed in histograms of all markers to visually control for the aforementioned errors. Should artifacts occur in the control samples, it might be useful to either exclude specific parameters or samples from the computational analysis.
5. If performing intracellular or intranuclear staining it is important to note that certain Ab-fluorochrome conjugates are affected by the fixation and permeabilization reagents. Thus, it is important to test the panel accordingly, and also treat single-stained control samples the same way as the experimental samples (fixation, incubation).

## II. Rare cells-General rules

### 1 Overview

The identification of rare cell populations is relevant for the advancement of medical diagnostics and therapeutics, and indeed the detection and characterization of Sars-CoV-2 Ag-specific T cell response is now of pivotal importance for a better understanding of the formation of long-term immunological memory. In this section, the main issues of rare event detection, including steps of the pre-analytical phase, the amount of blood to use, the use of pre-enriched populations, the number of markers to use, the number of cells to acquire and the importance of using optimized methodologies will be described.

### 2 Introduction

In several clinical settings, counting rare cell provides valuable information on the status and stage of the patient’s disease. Rare circulating tumor cells in peripheral blood, tumor stem cells, circulating endothelial cells, hematopoietic progenitor cells and their subpopulations, and fetal cells in maternal circulation are main examples of detectable, rare cells. Interesting applications also include the detection of metastatic breast cancer cells [88] or neuroblastoma cells infiltrating the bone marrow [89], monitoring of minimal residual disease [90, 91], detection of stem cells and rare HIV-infected cells in peripheral blood [92], T-cells specific for an uncountable number of Ags, invariant NK T cells, innate lymphoid cells [93], and analysis of mutation frequencies in genetic toxicology [94]. Moreover, polyfunctional assays, such as the Ag-induced production of different cytokines by T lymphocytes, are often performed, raising the problem of finding cells able to produce more than one cytokine or exert more than one function also within T-cell populations.

### 3 Optimization

Studying rare cells requires optimization of methodologies in all phases, including the collection of biological samples, well-defined controls, and adequate use of software and hardware [95]. The term “rare” generally refers to events with a frequency of 0.01% or less, although the record claimed in the literature has long stood at one cell in 10 million for tumor cells spiked in the peripheral blood [96, 97]. For this, the acquisition of a large number of events (see section V1.2.3 Number of acquired events in [21]) and a high signal-to-noise ratio (see section V1.2.5 Thresholds, gating, and DUMP channel in [21]) are the most relevant aspects.

### 4 The quantity of the biological material

On the basis of the estimated frequency of the rare cells under investigation, it is crucial to calculate how much biological material is required. For example, if the endpoint of the experiment is to enumerate rare cell populations present in the cerebrospinal fluid, considering that only a few milliliters can be obtained from a patient, it is logical that all the cerebrospinal fluid obtained by a spinal puncture has to be all used. If blood is the biological matrix of interest, the rare cell population of interest and the pathology of the patient should be considered in depth. Should the endpoint of the study be the evaluation of cytokine production after in vitro stimulation by cells such as invariant natural killer T (iNKT) lymphocytes in patients with HIV infection, some pre-analytical considerations should be taken into the account. For example, iNKT cells are quite rare among peripheral blood mononuclear cells (0.01-1%), and in order to define this population several markers must be used, including those that identify CD3, CD4, CD8, invariant TCR, as well as those for cell viability. Different cytokines such as TNF, IFN-γ, IL-4, and IL-17 could be of interest. So, nine markers are required. HIV+ patients who do not take, or do not respond to antiretroviral therapy can be severely immunocompromised, and have a low number of peripheral CD4^+^ T lymphocytes. Thus, the amount of blood required to detect a reasonable number of rare cells (according to Poisson statistics) can be up to 50 mL of blood, since both resting or stimulated cells have to be analyzed [98].

### 5 Enrichment and choice of markers

**Figure 5.**
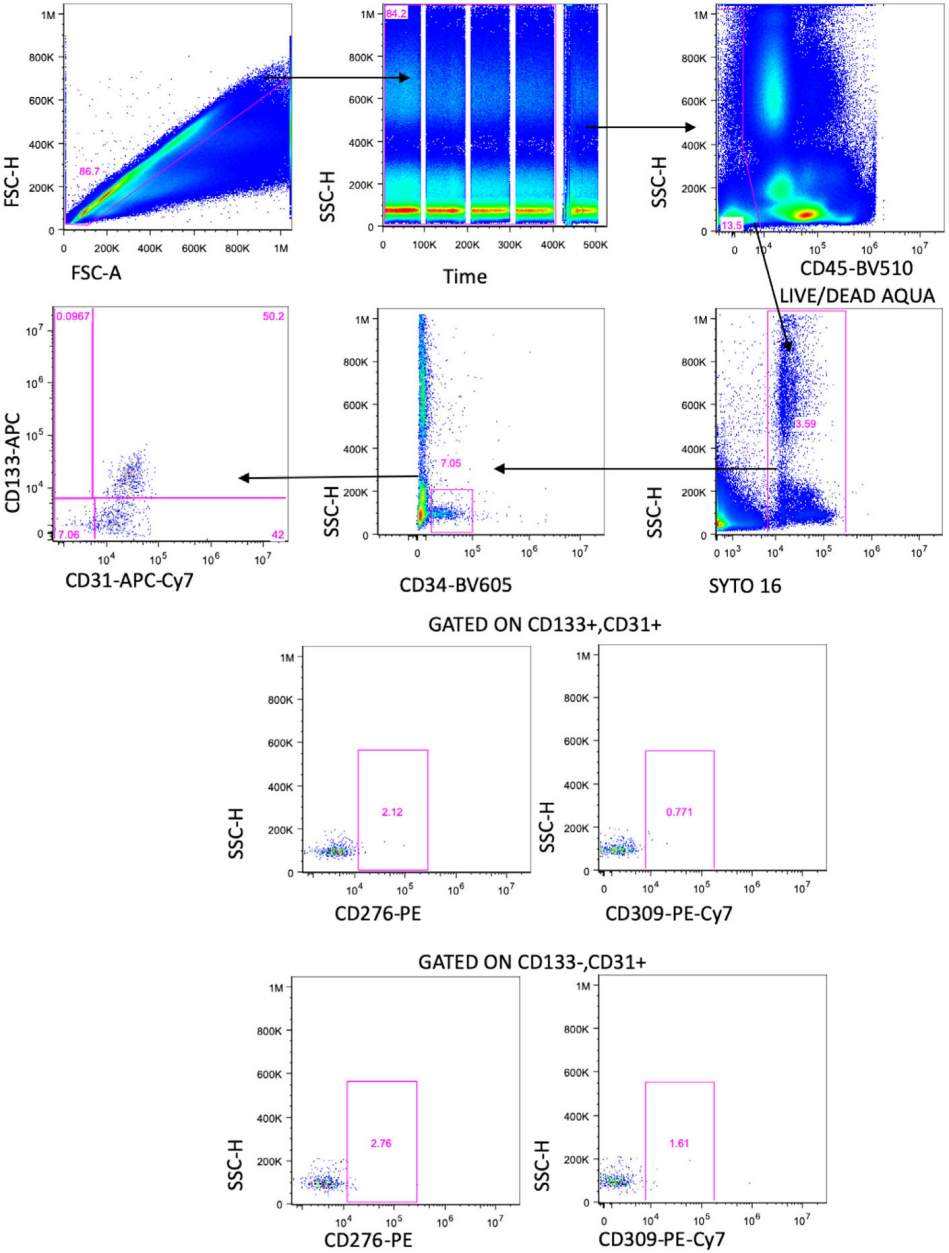
Detection of human circulating endothelial cells and their precursors. Gating stategy used to identify circulating endothelial cells (CEC) and their precursors (EPC) among peripheral blood leucocytes. Debris and aggregates were eliminated using FSC-Area vs FSC-Hight, while possible clogs were removed using the parameter Time vs SSC. Then, a DUMP channel was used to remove from the analysis CD45^+^ cells and dead cells. In the remaining population, nucleated cells were identified by positivity for Syto16. Stem cells were identified according to CD34 positivity, and among this population, EPC (CD133^+^,CD31^+^) and CEC (CD133^−^,CD31^+^) were identified. The expression of CD276, also named B7-H3, and CD309, also named VEGFR-2 or KDR, was evaluated in each subpopulation. In this example, more than ten million events were initially acquired.

On the basis of the experimental endpoint(s) (e.g., phenotyping, functional assays), the rare population may be enriched or not, and the number of markers that are needed to unambiguously identify a rare cell population needs to be defined. For example, the accurate quantification of circulating endothelial cells (CECs) and their progenitors (EPCs), shown in Figure 5, is a matter of debate. Several studies have been published, but no consensus has thus far been reached on either the markers that should be used to identify these cells, or on the necessity of a pre-analytical enrichment (by density gradient, buffy coat, and/or magnetic enrichment). The enrichment, however, can have negative effects if rare cells are lost, or positive if unwanted cells are removed [99-103]. Unfortunately, quite often, the lack of well-standardized methods influences the decision on the number of markers that are necessary for the identification of the population of interest. Depending on the technical characteristics of the flow cytometers, which have a varying number of fluorescence channels and speed of acquisition, the most important marker allowing the identification and characterization of such populations should be decided first. For example, in the case of iNKT cells, the Vα24Jα18 invariant TCR allows the unique identification of these cells. Having done that, the marker panel has to be built following a general rule that the brightest fluorochrome has to be used for the weakest marker expressed by our cells. Finally, attention should be paid to compensation, and acquisition should be considered to ‘Fluorescence Minus One’ (FMO) controls (covered in more details in Section II.1: Compensation and Section III.1: Controls: Determining positivity by eliminating false positives in [21]).

### 6 Number of acquired events

Concerning the number of events to acquire, it is recommended to use Poisson statistics, which defines the probability that a given number of events will occur in a fixed interval of time/space, assuming these events would occur with a known average rate and independently of the time elapsed from the previous event [104]. Therefore, Poisson statistics are applied to count randomly distributed cells in a certain volume. Let us consider a general case of enumerating a total of N events, of which R meet a certain criterion (i.e., they are positive, P). In this case, a proportion of *P* events is defined as *P* = R/N. The probability of any single event to be positive is obviously 0 ≤ *P* ≤ 1, and this is related to the random manner in which cells are selected for analysis. As with all statistical distributions, the variance, Var, is a fundamental parameter, and is defined as: Var(R)=NP(1-P). The standard deviation (SD) is defined as the square root of the variance, and the coefficient of variation (CV) is defined as the SD expressed as a percentage of the population: CV=(SD^*^100)/R [105]. These equations can be used to examine some practical situations. Let’s consider a phenotype analysis of human PBMCs stained with a mAb for detection of B cells (e.g., anti-CD19 mAb). In healthy individuals, 10% of the cells can be positive, so that: *P* = 0.1 and *P*(1 − *P*) = 0.09. Good experimental practice suggests to keep CV below 5%; thus, acquiring even 5,000 events could be sufficient, because the CV is 4.24. Using a number of cells such as 10,000 the CV becomes 3.16. However, should positive events be less frequent, a higher number of events must be acquired. Table 5 reports an example for events whose frequency is 0.01%, as often occurs studying Ag-specific T cells.

This is clearly the ideal methodology. However, real life is different from theory, and very often the final number of events cannot be high enough to satisfy this golden rule. For example, we can consider the case in which 1 million peripheral T cells are stimulated with an Ag that activates less than 0.01% of them, namely 100 cells in one million. Nowadays, by polychromatic flow cytometry, T cell activation can be analyzed by evaluating the polyfunctionality of these cells, and protocols have been developed that can identify in a relatively easy manner 4 or even 5 functions per cell. Thus, among responding cells, up to 32 populations can exist, likely with a different frequency, and each subpopulation contains a few cells, that are completely absent in the control, unstimulated sample. Can we consider such cells positive, even if their number is much lower than that indicated by a strict statistical approach? A pivotal paper by Mario Roederer provides very useful and clear suggestions [106]. Indeed, if alternative explanations for the presence of such positive events can be excluded (i.e., if there is no noise due to dead cells or fragments, and if cell activation is really due to the Ag used in vitro and not to a pre-activation in vivo of T cells, as in the case of studies on vaccination), the events can be considered positive, irrespective of their number. Thus, there is no reason to fix a threshold for the number of events below which any frequency must be considered ‘‘negative’’ [106]. In this case, "positivity’’ can be determined after comparison of the measurement against a set of control samples, among which the adequate negative controls, using standard statistical tools to compare the frequencies. For example, assuming that from the technical point of view the experiment is well performed, if T cells from "n" unvaccinated controls show no activation after the stimulation with the adequate peptides, while T cells from "n" vaccinated individuals do, even extremely low frequencies can be taken as positive. The same logics can be applied in a number of other cases, assuming that the relative controls are well chosen.

### 7 Sample concentration and flow rate

Because it is crucial to acquire a high(er) numbers of events for detection of rare cell population, sample concentration and flow rate are critical parameters, which can typically shorten acquisition time. However, care must be taken that increasing the flow rate results in an increase of coincidence, and thus higher CV, if flow cytometers use hydrodynamic focusing (which is the system currently used in most commercially available flow cytometers).

### 8 Thresholds, gating, and DUMP channel

A threshold should be fixed in order to distinguish the signal (using fluorescence or scatter) required to define the population of interest from the noise/background (see section V.2 - Organisms, cells, organelles, chromosomes, and extracellular vesicles in [21]). Hence, maximizing the signal-to-noise ratio of the cells of interest is mandatory. Gates should be drawn to exclude from the analysis dead cells and all the unwanted cell populations, identified by viability marker, doublets/aggregates/debris. Thus, a “DUMP” channel containing Abs that identify cells of no interest is highly recommended. Moreover, using a dot plot with the parameter “time” vs. that of interest allows the investigator to remove the event bursts caused by clogs or other transient problems during the acquisition. The instrument should be kept clean, and it is essential to wash the instrument between the acquisition of different samples in order to minimize sample contamination, which could cause the detection of false-positive events.

### 9 Data analysis

Finally, data analysis requires adequate software and powerful hardware (more than 8GB RAM or higher), because acquired data file tend to be huge, depending on how many events and parameters have been acquired (e.g., a sample with 10 colors and two scatters in 10 million events would be a good test for your computer). To minimize the file size, parameters that are not really needed can be unselected, and a fluorescence/scatter threshold trigger can be used. Data analysis will be covered in greater detail in Chapter VI, Section 1: Data analysis.

The possibility to create panels with more than 20 markers has increased the ability to generate high-throughput single-cell data. Single-cell data are often compiled under “R” from multiple experiments that may have differences in capturing times, personnel handling the samples, reagent lots, equipments, and even technology platforms. These differences lead to large variations or batch effects, and can create serious problems during data integration. As such, effective batch-effect removal is essential in particular when analyzing rare events. Nowdays, many different methods are freely available to correct bath-effect. For example, freely available tools such as BatchAdjust, CytoNorm, CytofRuv, and iMUBAC have proven their efficacy [107-109]. Thus, for the batch-effect correction, based on data normalization, it is highly recommended to run the same sample in different days [108].

### 10 Clinical relevance statement

The identification and characterization of rare events can be relevant in several clinical settings. Counting circulating tumor cells can help oncologists in defining the degree and severity of a given tumor, and eventually its capacity to form metastasis, as well as the effect of a treatment. The characterization of Ag-specific cells can provide indications not only to the response to a vaccine, but also to the formation of long-term memory after a given infection or after vaccination. In the era of COVID-19 pandemic, the importance of understanding the immediate immune response to the virus and the formation of immunological memory is self-evident.

#### 10.1 Rare events: focus on SARS-CoV-2 T cell responses

**Figure 6.**
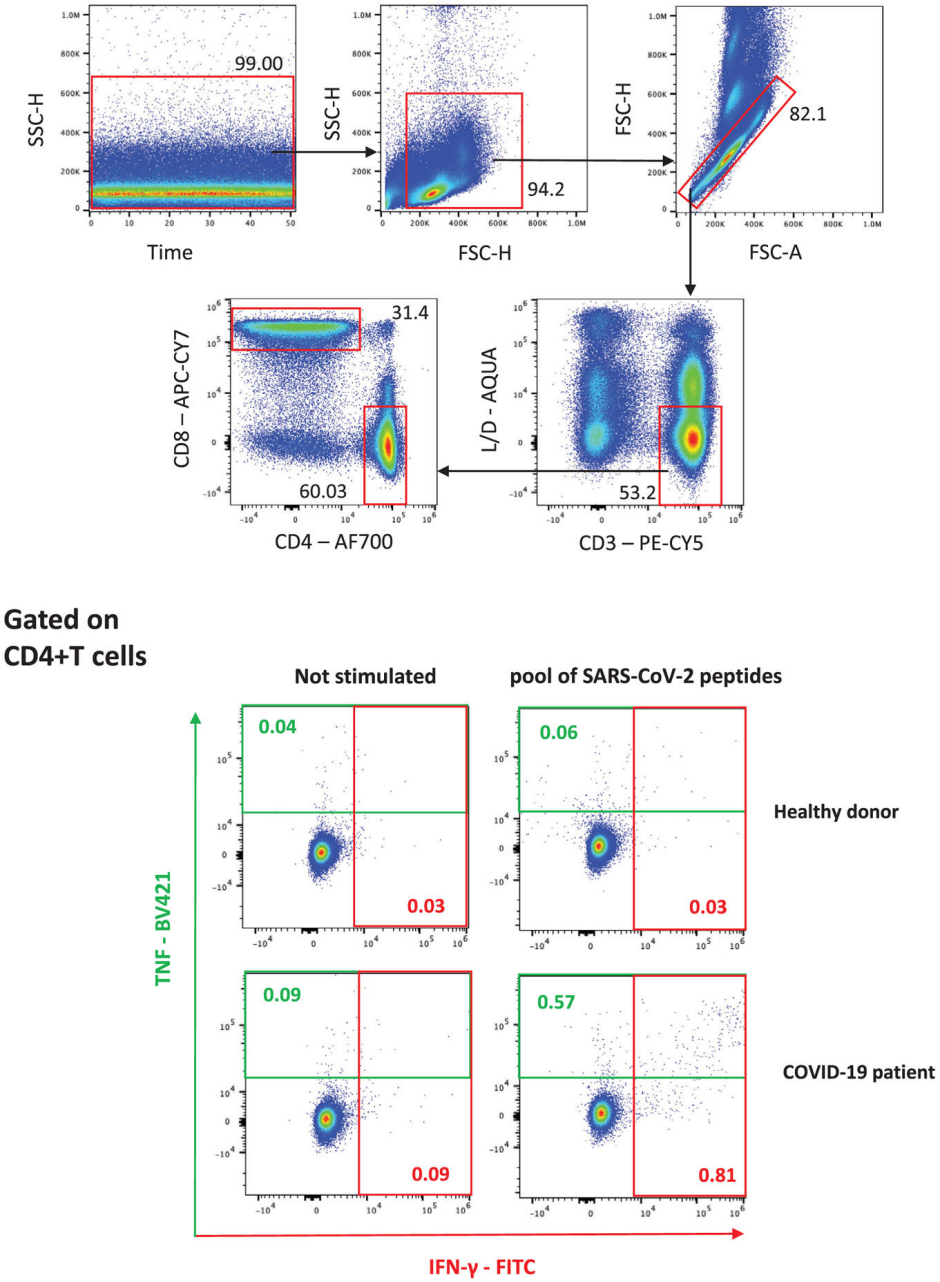
Detection of SARS-CoV-2 T cell-specific response. Gating strategy used to identify SARS-CoV-2 T cells among CD4^+^ and CD8^+^ T cells. Perturbancies during acquisition have been removed and PBMC have been identified according to physical parameters, doublets have been removed as well as dead cells. In this population CD3^+^ T cells have been selected and in this, CD4^+^ and CD8^+^ T cells have been recognized. Representative dot plots showing the percentages of human CD4^+^ T cells producing TNF and IFN-γ after 16 hours of *in vitro* stimulation with 1μg/ml SARS-CoV-2 Prot S PepTivator (Miltenyi). Unstimulated and stimulated conditions are shown. Upper panel: healthy donor; lower panel: COVID-19 patient.

The recent SARS-CoV-2 pandemic has raised growing interest in understanding how adaptive immune responses act to control the infection, provide protection from possible reinfections and sustain protection after vaccination. As data start to accumulate on the detection and characterization of SARS-CoV-2 T cell responses in humans, it has been reported that lymphocytes from 20% to 50% of unexposed donors display significant reactivity to SARS-CoV-2 Ag peptide pools (Figure 6). This cross-reactivity largely originates from previous exposure to circulating common cold coronaviruses [110-116]. The cross-reactivity could be a possible pitfall to consider, which could be tackled by using peptide pools from different viral proteins or protein precursors.

Information about Sars-CoV2-specific T cells frequency, phenotype, and functionality is necessary to evaluate the specific immune status, to understand the mechanisms of protective immunity, and to predict long-lasting immune protection. *In vitro* stimulation by peptides is the most used test to estimate the cells responsible for cytokine storm and long-lasting cellular memory [117]. A very high number of data have been published using both commercially available peptides and custom-made libraries of peptides. Using HLA class I and II predicted peptide “megapools,” circulating SARS-CoV-2-specific CD8^+^ and CD4^+^ T cells were identified in COVID-19 acute, mild and convalescent patients [110, 118-125]. A vast majority of responding CD4^+^ and CD8^+^ T cells displayed an activated/cycling (CD38^+^ HLA-DR^+^ Ki67^+^ PD-1^+^) phenotype [113] and predominantly produced effector and Th1 cytokines, although Th2 and Th17 cytokines were also detected [119, 126], suggesting a link between individual patient predisposition with respect to age and comorbidity [127, 128]. Moreover, thanks to the use of high-parameter flow cytometry data analyzed by unsupervised methods, it has been shown that SARS-CoV-2-specific T cells are diverse from other virus-specific T cells, they express CD127 and they possess lymphoid-homing Tfh cells. In particular, SARS-CoV-2-specific CD8^+^ T cells are predominantly less-differentiated terminally differentiated cell with different homing features between SARS-CoV-2-specific CD4^+^ and CD8^+^ T cells [129]. Then, 18 CD8^+^ T cell recognized SARS-CoV-2 epitopes, including an epitope with immunodominant features derived from ORF1ab and restricted by HLA-A*01:01 were idenfied, indicating the possibility to develop a vaccine based upon CD8^+^ T cells response against Ags different from the spike protein [123].

Given the paucity and rarity of SARS-CoV2-specific T cells, very often results were confirmed by using an activation-induced marker (AIM) assay to measure upregulation of CD69 and 4-1BB (CD137) [130]. Moreover, IFN-γ-producing cells responding to overlapping peptides spanning the immunogenic domains of the SARS- CoV-2 spike (S), membrane (M), and nucleocapsid proteins (N) usually are enumerated by ELISpot assays.

## III T cell phenotypes

### 1 Human conventional αβ CD4 T cells

#### 1.1 Overview

Human naïve and memory CD4^+^ T cells display remarkable heterogeneity, that can be dissected at high resolution by the use of flow cytometry. Specifically, flow cytometry can be adopted for the analysis as well as for the isolation of T lymphocyte subpopulations. Different T cell subsets can be identified based on the selective or combined expression of surface molecules and transcription factors, as well as on the production of specific cytokines. Proteins expressed on the cell membrane, such as chemokine receptors, guide T cell homing capacity; transcription factors, stained intranuclearly, indicate differentiation stages and polarization; and cytokine production, detected intracellularly before their secretion, underlies T cell function. Multiple features in different cellular compartments can be simultaneously interrogated by flow cytometry, depending on the scientific question to be addressed and the resolution required.

#### 1.2 Introduction

CD4^+^ T cells play a pivotal role in adaptive immunity to protect our body from pathogens’ attack. They can exert their protective function either directly, by acquiring effector and cytotoxic phenotypes, or indirectly, by the release of different types of cytokines that optimize durable CD8^+^ T cell responses and provide help to B cells for Ab production [131]. CD4^+^ T cells recognize the Ag in the form of short peptides assembled onto MHC class II molecules exposed on the cell surface of APCs. After being activated in secondary lymphoid organs by dendritic cells, Ag-specific naïve T cells start to proliferate and differentiate into effector cells that are able to migrate to peripheral tissues where they perform protective functions, and T follicular helper (Tfh) cells that migrate to B cell follicles to provide help for B cell responses. Once the pathogen has been cleared most of the effector T cells die by apoptosis, but some of them persist as long-lived memory T cells that can provide enhanced responses upon re-exposure to the cognate Ag [132, 133]. Influenced by the signals received by dendritic cells upon Ag recognition, naïve CD4^+^ T cells can differentiate into a multitude of phenotypes that can be distinguished based on differentiation stages, function, and homing capacity. The number of identified T cell subsets has increased in the last decades in parallel with the availability of improved profiling technologies for sensitive detection of cell heterogeneity. Moreover, the different subpopulations are not divided by sharp boundaries as T cells can exhibit intermediate phenotypes and can show phenotypic plasticity and functional reprogramming depending on the environmental cues. In this section, we will review the best-established cellular markers that can be exploited to shed light on this complexity.

##### 1.2.1 CD4^+^ T cell differentiation.

**Figure 7.**
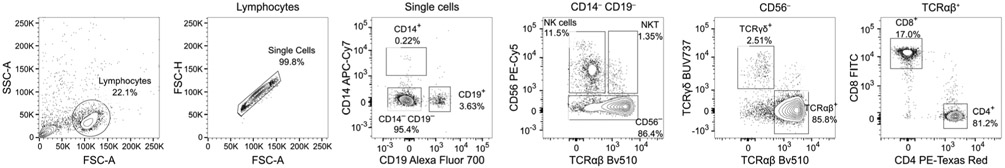
Gating strategy to study human CD4^+^ and CD8^+^ T cells in the peripheral blood. Lymphocytes are identified based on the FSC and SSC. Single cells are discriminated from doublets by plotting the pulse area and height against each other for the FSC. CD19^+^ cells (B cells), CD14^+^ cells (monocytes) and CD56^+^ cells (NK and NKT-like cells) are excluded as cell populations other than T lymphocytes. Within the CD14^−^CD19^−^CD56^−^ cells, αβTCR^+^ and γδTCR^+^ T cell subsets can be identified. αβTCR^+^ T lymphocytes include predominantly CD4^+^ and CD8^+^ T cells.

**Figure 8.**
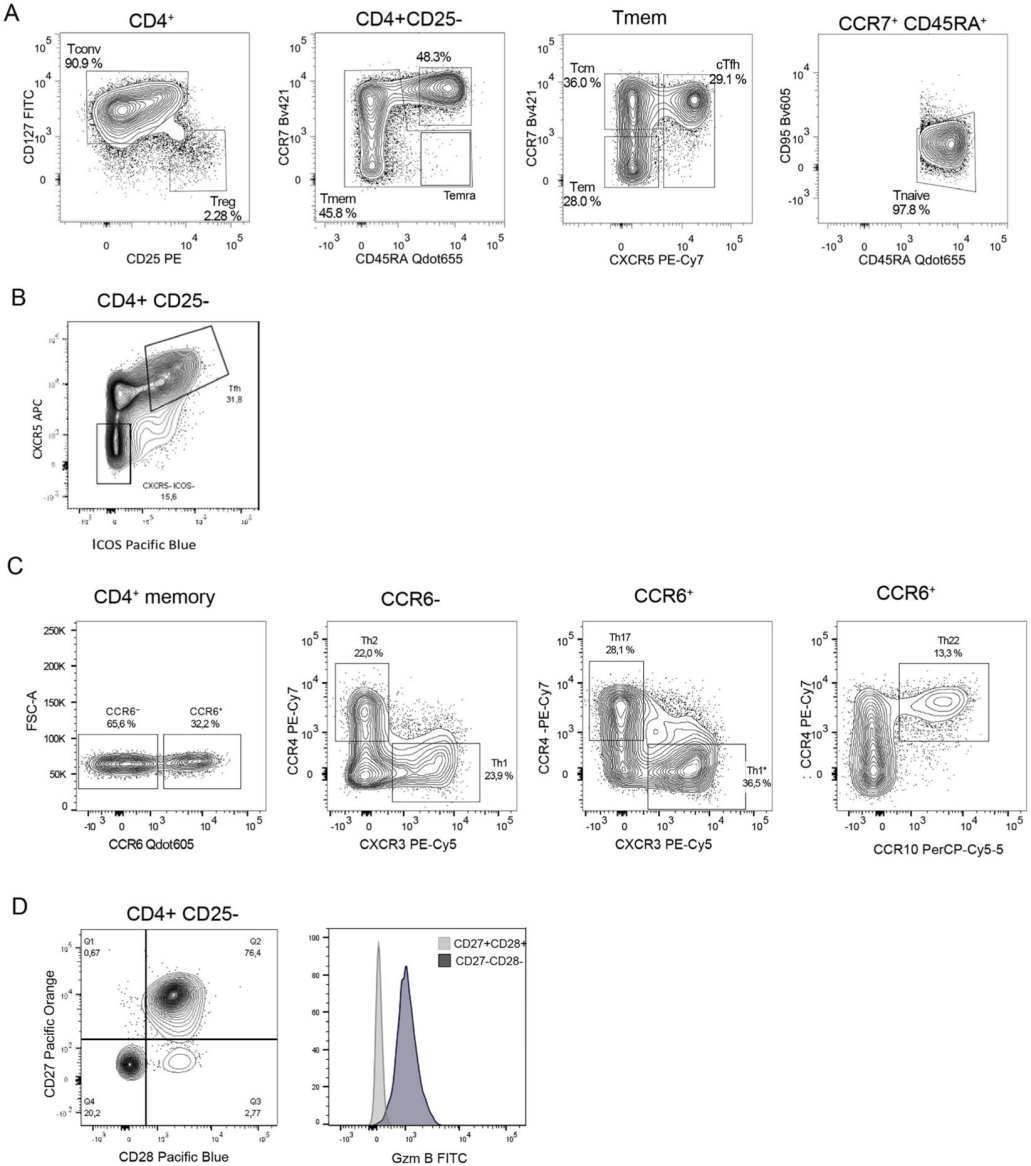
Gating strategy to identify the differentiation stages and memory subsets of human CD4^+^ T cells in the peripheral blood. (A) Conventional CD4^+^ T cells and Treg cells are identified based on the differential expression of the surface markers CD127 and CD25. Conventional CD4^+^ T cell population (Tconv CD127^+^CD25^low^) can be divided in naïve and memory T cell subsets (Tscm, Tcm, Tem and cTfh) based on the surface markers CCR7, CD45RA, CXCR5 and CD95. (B) Co-expression of the chemokine receptor CXCR5 and the activation marker ICOS among CD4^+^ T cells in human tonsils identifies Tfh cells. (C) At least 5 different memory T helper subsets can be detected in the CD4^+^ T cell memory population based on their differential expression of the chemokine receptors CCR6, CXCR3, CCR4 and CCR10. (D) CD4^+^ CTL expressing GzmB can be identified among CD25^−^ Tconv based on the absence of CD27 and CD28 expression.

Lymphoid progenitors migrate from the bone marrow to the thymus where T lymphocyte maturation occurs through a stepwise process characterized by the productive rearrangement of the T cell receptor (TCR) and the timely expression of the CD4 and CD8 co-receptors (See section III.3 Human conventional αβ CD8 T cells). Mature T lymphocytes express the CD3 protein complex together with a heterodimeric TCR that can be composed either by the alpha/beta (αβ)- or the gamma/delta (γδ)- chains. Most of the αβ-TCR conventional T cells express either CD4 or CD8 (Figure 7), although a small proportion of double-positive or double-negative T cells can be found in the circulation. In the thymus, potentially autoreactive T lymphocytes bearing a TCR that recognizes self-peptides presented by thymic epithelial cells with a high affinity are deleted through a mechanism known as negative selection. However, T cells bearing a TCR with an intermediate affinity to self-peptides can escape the negative selection and preferentially develop into a distinct CD4^+^ T cell population with immunosuppressive capacity, the regulatory T cell (Treg) subset (Section III.11 Human FOXP3^+^ regulatory T cells). Treg cells are required for peripheral tolerance and can be distinguished from conventional CD4^+^ T cells based on the constitutive high expression of the IL-2 receptor alpha chain (CD25) and the low expression of the IL-7 receptor alpha chain (CD127) (Figure 8A) [134].

Conventional naïve T (Tn) cells survey the body, especially in secondary lymphoid tissues, looking for their cognate Ag. Upon Ag encounter, they start to proliferate and differentiate rapidly into effector T cells. Following pathogen clearance, most of the effector cells die, while a minority of them develop into long-lived memory cells [132, 133]. Depending on their migratory capabilities and immediate effector functions, memory T cells can be divided into central memory (Tcm) cells that re-circulate in secondary lymphoid organs and are endowed with high proliferative capacity, and effector memory (Tem) cells that have immediate access to peripheral tissues and provide rapid and effective secondary responses through effector cytokine production [135, 136]. Naïve and memory lymphocytes can be distinguished based on the mutually exclusive expression of the CD45 isoforms CD45RA and CD45RO: naïve T cells express CD45RA and lack CD45RO; vice versa, memory cells preferentially express CD45RO and miss CD45RA expression. In addition, the lymphoid homing markers CCR7 and L-selectin (CD62L) allow the separation of naïve and Tcm from Tem [135, 137], although CD62L expression has been reported to be profoundly affected by cryopreservation [138] thereby limiting its use to freshly isolated cells. Thus, the combined staining of CD45RA and CCR7 can represent an effective experimental tool to distinguish naïve (CD45RA^+^CCR7^+^), Tcm (CD45RA^−^ CCR7^+^), and Tem (CD45RA^−^CCR7^−^) cells. A fourth, rare subset lacking CCR7 while re-acquiring CD45RA expression (CD45RA^+^CCR7^−^, Temra) can be identified through this strategy, and is populated by highly differentiated effector memory cells that are specific for persistent viruses [139]. Furthermore, a small population of memory T cells endowed with elevated proliferative and self-renewal capacities has been identified within the CD45RA^+^CCR7^+^ naïve T-cell gate. These cells have been termed stem cell-like memory (Tscm) cells [140] and can be distinguished from truly naïve T cells based on the expression of the FAS receptor (CD95) (Figure 8A) [81].

The heterogeneous phenotypes of T helper (Th) cells are intimately linked with their multiple effector functions that ensure efficient pathogen clearance and host protection. A useful approach to further classify memory T cell subsets relies on the expression pattern of additional chemokine receptors, which guide their extravasation from blood vessels and homing to the tissues where they exert their effector function [141]. For instance, Tfh cells express CXCR5 which directs their migration to B cell follicles in lymph nodes, where they can interact with Ag-specific B lymphocytes and support their activation and maturation to produce high-affinity Abs [142]. In addition to CXCR5, activated Tfh express high levels of PD-1 and ICOS, two markers defining the phenotype of Tfh effector cells in secondary lymphoid organs (e.g., tonsils) (Figure 8B) and the efferent lymph [143]. The presumed circulating counterpart of Tfh, termed circulating Tfh (cTfh), can be detected in human peripheral blood and identified within the CD4^+^ memory gate by surface expression of CXCR5 (Figure 8A).

Th1 cells are critical for cell-mediated immunity against intracellular pathogens and can be identified by the expression of CXCR3, a chemokine receptor that guides their homing to inflamed tissues. CCR5, another chemokine receptor with similar homing properties, has been also used to identify these cells [144, 145], but it is expressed only on the more differentiated Th1 effector memory population [146]. Th2 lymphocytes are responsible for the adaptive immune response against large extracellular pathogens, such as helminths, as well as for the detoxification of venoms and noxious xenobiotics. Excessive Th2-mediated immune responses are involved in allergy. Th2 lymphocytes are enriched in the expression of the chemokine receptors CCR3, CCR8, and in particular CCR4, while the prostaglandin D2 receptor CRTh2 is restricted to a subset of terminally committed Th2 effector cells [147-149]. Of note, CCR8 is also expressed on tissue- and tumor-infiltrating Treg cells [150, 151]. Th17 and Th22 subsets are pivotal to tackle extracellular pathogens, such as extracellular bacteria and fungi, and they both lack CXCR3 and express CCR6 and CCR4 [152-154]. However, Th17 can be distinguished by the expression of the killer-cell lectin-like receptor CD161 (KLRB1) [155,156], while Th22 expresses the skin-homing receptor CCR10 [154].

Finally, a subset of lymphocytes named Th1* or Th1/17 is critical for the defense against intracellular bacteria, such as *Mycobacteria* [157], has intermediate features between Th1 and Th17 cells and co-expresses CXCR3, CCR6, and CD161 [158-160]. Thus, the expression of a single chemokine receptor is not sufficient for the identification of distinct Th subsets, which rather requires the evaluation of the combined or mutual exclusive expression of multiple markers. Indeed, also cTfh cells can be further subdivided according to CXCR3 and CCR6 expression into subsets of CXCR3^+^ Tfh1, CCR6^+^ Tfh17, and CXCR3^−^CCR6^−^ Tfh2 cells with different capacities to induce IgG, IgA, and IgE from naïve B cells [161]. Here, we illustrate a gating strategy to distinguish Th1, Th2, Th17, Th1*, and Th22 populations based on the expression of CXCR3, CCR4, CCR6, and CCR10 (Figure 8C). It is important to keep in mind that Th subsets identified with this strategy, as far as the expression of effector cytokine and lineage-defining transcription factors are concerned, represent enriched rather than pure populations. However, it has the advantage that the T helper cells can be tracked directly *ex vivo* without the need of being activated in vitro (see below). Rather, in vitro activation can induce a relevant modification of chemokine expression patterns, in particular at later time points.

Notably, the CD4^+^ compartment contains also a minor population of cytotoxic T cells (Tctl), that can be identified by the loss of the costimulatory receptors CD28 and CD27 (Figure 8D). CD4^+^ Tctl express high levels of cytotoxic molecules s Granzyme B and, given the lack of costimulatory molecules, are considered to be terminally differentiated effector cells.

##### 1.2.2 CD4^+^ T cell cytokine production capacities.

**Figure 9.**
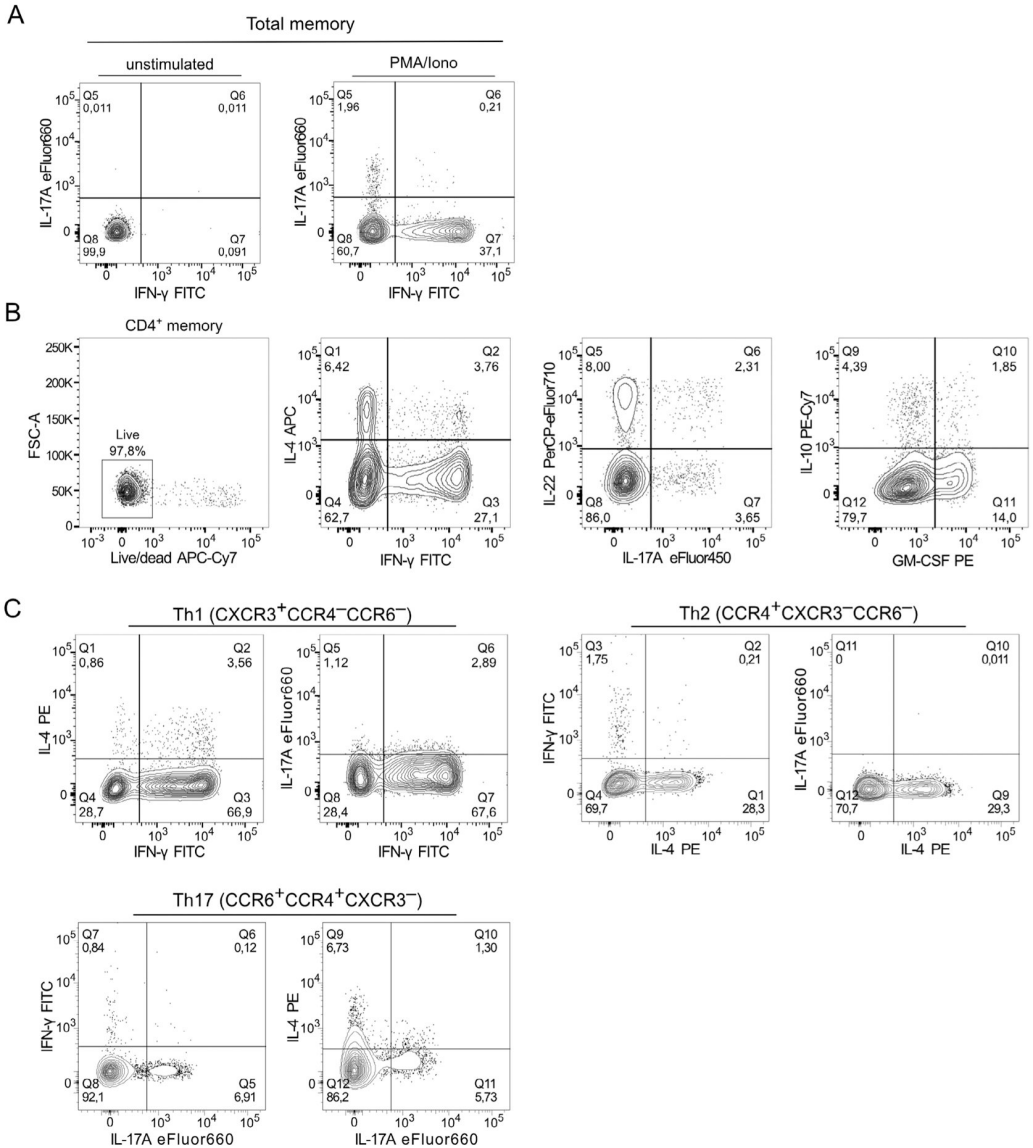
Human T-cell subsets as identified by intracellular cytokine staining. (A) Expression of IFN-γ and IL-17A with or without 5 h of PMA/Ionomycin stimulation in the presence of BFA (for the last 2.5 h). (B) Expression of IFN-γ and/or IL-4, IL-17A and/or IL-22, and of IL-10 and/or GM-CSF by total CD4^+^ memory T cells. (C) T cell populations are enriched by flow cytometry according to the gating strategy indicated in Figure 2C and then stimulated in vitro for 5 h with PMA and Iono in the presence of BFA (for the last 2.5 h). Shown is the expression of IFN-γ, IL-4, and IL-17A by Th1, Th2 and Th17 cell subsets sorted *ex vivo* from the blood.

In addition to chemokine receptor expression and homing capacity, Th subsets are defined based on their ability to produce specific cytokines (Figure 9). Since cytokines are not preformed or stored in granules, their levels are typically low to null in resting cells that are isolated *ex-vivo* from the blood. For this reason, an *in vitro* activation step of CD4^+^ T cell populations enriched from the blood is needed to allow the analysis of their cytokine expression (Figure 9A). Stimulation with phorbol 12-myristate 13-acetate (PMA) and ionomycin (Iono) represents a convenient way to interrogate T cells for their cytokine production capacities regardless of Ag specificity. Intracellular accumulation of cytokines within the ER is achieved by adding an inhibitor of protein transport to stimulated cells. The two most frequently used inhibitors are Monensin (MN) and Brefeldin A (BFA). The choice of protein transport inhibitors should be considered carefully, as they can have differential effects on surface and intracellular protein expression after stimulation. For example, BFA will help to maximize the capture of TNF-α, IFN-γ, and IL-17A, but blocks the surface expression of some molecules, such as the T-cell activation marker CD69 [162]. After polyclonal stimulation of T cells, cytokines may be produced with different kinetics. For most cytokines, a stimulation and accumulation period of 4–6 h is optimal (Figure 9B). However, in some circumstances, such as IL-10 produced by memory/helper T cells, the production kinetics are relatively slow and at least 24 h stimulation and different stimulation strategies may be required for optimal detection [163, 164]. Notably, IL-10 is however rapidly secreted by effector and regulatory T cells following standard PMA and Iono stimulation [164-166] (see section III.13 Human Tr1-cells). As both MN and BFA are moderately toxic, exposure of stimulated cells should be limited. Consequently, for general purposes, MN and BFA can be added in the last 2-4 h, while for the longer stimulations they may be added during the last 4–6 h to optimize the accumulation of slow-kinetics cytokines.

**Figure 10.**
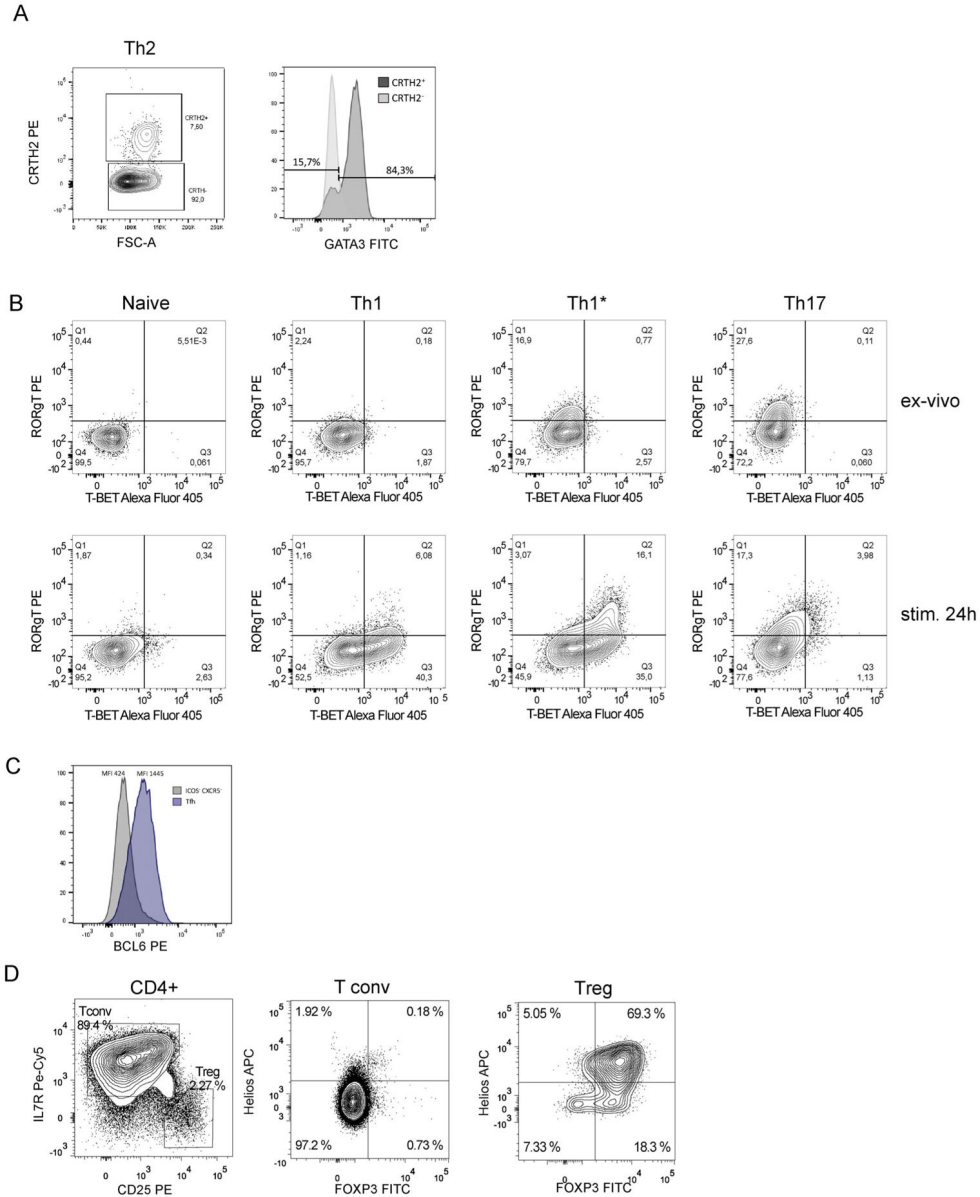
**Human** T-cell subsets analyzed by intranuclear staining for transcription factor expression. (A) *Ex vivo* GATA3 expression in CCR4^+^CCR6^−^ Th2 memory cells according to CRTh2 expression (B) Naïve, Th1, Th1* and Th17 cells were sorted from PBMC as indicated in Figure 2C and stained for their expression of T-BET and RORC2/RORγT either *ex-vivo* or after 24 h α-CD3/α-CD28 stimulation (stim.). (C) Tonsillar CXCR5^+^ICOS^+^ Tfh cells and non-Tfh cells gated as in Figure 2B were analyzed for BCL6 expression. (D) *Ex vivo* expression of the transcription factors Foxp3 and Helios in CD25^hi^CD127^lo^ Treg cells from peripheral blood.

The pattern of effector cytokine production is characteristic for different T-cell lineages. Thus, Th1 lymphocytes are characterized by the production of IFN-γ, Th2 by IL-4, as well as by IL-5 and IL-13 and Th17 by IL-17A, as well as by 17F, IL-22, and IL-26 (Figure 10C). Although they have an intermediate phenotype between Th1 and Th17, Th1* cells produce mostly IFN-γ and secrete only limited amounts of IL-17A.

##### 1.2.3 Transcriptional regulation of CD4^+^ T cells.

The polarization toward different Th phenotypes, which results in the acquisition of defined homing capacities and effector functions, is regulated by the expression of lineage-defining transcription factors (TF). These lineage-defining TFs can be analyzed by intranuclear staining to identify polarised T cell populations (Fig. 10). Cytokines produced by Ag-presenting dendritic cells drive the polarization of cognate naïve T cells, by inducing complex interactions between opposing TFs and their targets often, but not always, resulting in mechanisms of counter-regulation that stabilize the phenotype. Thus, for instance, T-bet which drives the differentiation of Th1 cells has been shown to antagonize the Th2-specific TF, GATA3 [167, 168]. GATA3, among human blood CD4^+^ helper cells, is highly expressed in CRTH2^+^ Th2 cells (Figure 10A). On the contrary, T-bet is expressed at high levels in CD4^+^ CTL [169], but only at a lower level in Th1 memory cells. T-bet is however selectively up-regulated in Th1 cells following TCR stimulation (Figure 10B). Interestingly, T-bet and RORC2/RORγT, the TF controlling the polarization of Th17 lymphocytes [157], are co-expressed in Th1* cells (Figure 10B). In addition to the mentioned TFs, the aryl hydrocarbon receptor (AHR) has been demonstrated to regulate Th22 polarization [170], while the differentiation of Tfh cells is orchestrated by BCL6 (B-cell lymphoma 6) [171, 172]. Notably, BCL6 is highly expressed in tonsillar Tfh cells (Figure 10C), but is low in cTfh. Finally, the TFs FoxP3 (Forkhead Box 3) and Helios are highly expressed in Tregs from peripheral blood ex vivo (Figure 10D). However, Tconv can transiently upregulate FoxP3 expression upon activation. In mice, the expression of Helios is used to distinguish natural and peripheral induced Treg cells, that developed in the thymus or periphery, respectively, but this model is controversial in humans [173].

Since TFs bind the DNA, they need to be detected in the nucleus. To allow Abs to reach their epitopes, T cells need to be fixed and permeabilized, as needed for intracellular cytokine staining. However, the permeabilization for intra-nuclear staining is stronger and may induce cell shrinkage and loss of surface marker staining intensity, and protocols should therefore be validated and optimized.

#### 1.3 Step-by-step sample preparation

##### 1.3.1 Isolation of PBMCs.

1. Isolate PBMCs from heparinized blood or buffy coat by using Ficoll according to manufacturer’s instructions.
2. Collect the PBMCs into one new 50 mL tube.
3. Add washing medium up to 50 mL, mix well by pipetting up and down three times, and centrifuge for 15 min at 400 × *g* at RT. You should see the cells as a white pellet at the bottom of the tube.
4. Aspirate supernatant, gently resuspend pellet in 40 mL washing medium, and centrifuge for 10 min at 300 × *g* at RT.
5. Aspirate supernatant, gently resuspend pellet in 40 mL washing medium, and centrifuge for 10 min at 200 × *g* at RT.
6. Count and wash cells in appropriate buffer for further analysis.

##### 1.3.2 Surface staining.

1. Transfer up to 1 × 10^6^ PBMC into a 5 mL FACS tube.
2. Add PBS up to 4 mL and centrifuge for 5 min at 400 × *g* at RT.
3. Meanwhile, prepare live/dead exclusion dye staining mix according to the manufacturer’s instruction.
4. Aspirate supernatant and resuspend cells by gently tapping the tube.
5. Add 50 μL live/dead staining and incubate for 30 min at RT, protected from light.
6. Add surface FACS buffer up to 4 mL and centrifuge for 5 min at 400 × *g* at RT.
7. Meanwhile, prepare surface staining mix in a total volume of 50 μL FACS buffer for each tube (prepare 1× extra).
8. Aspirate supernatant and resuspend cells by gently tapping the tube.
9. Add 50 μL surface staining mix and incubate for 30 min at RT, protected from light.
10. Add surface FACS buffer up to 4 mL and centrifuge for 5 min at 400 × *g* at RT.
11. Resuspend cells by tapping the tube.
12. Add 200 μL FACS buffer, and analyze or sort cells by flow cytometry, or continue with the intracellular staining protocol.

##### 1.3.3 Intracellular staining of cytokines.

1. Transfer up to 2 × 10^5^ PBMC to a 96-well round-bottom plate.
2. Wash cells 2× with wash buffer for 3 min 400 × *g* at RT.
3. Resuspend cells in each well in 200 μL culture medium supplemented with PMA and ionomycin (see concentrations in Media and Buffer section).
4. Incubate cells for 2.5 h in a 37°C 5% CO_2_ incubator.
5. Add 20 μL of 10× concentrated BFA (see the concentration in Media and Buffer section) into each well and incubate for another 2.5 h at 37°C. The total stimulation period is therefore 24 5h.
6. Spin down for 5 min at 400 × *g* at RT and wash 2× with PBS 1×.
7. Meanwhile, prepare live/dead exclusion dye staining mix according to manufacturer’s instruction.
8. Add 50 μL live/dead staining and incubate for 30 min at RT, protected from light.
9. Add 150 μL and wash 2× with FACS buffer by centrifugation for 5 min at 400 × *g* at 4°C.
10. Discard the supernatant and add 100 μL of Cytofix/Cytoperm reagent to each well and resuspend by pipetting three times up and down.
11. Incubate for 20 min on ice.
12. Add 100 μL and wash 2× with perm/wash buffer (1:10 dilution with H_2_O) by centrifugation for 5 min at 600 × *g* at 4°C.
13. Discard the supernatant and add 50 μL intracellular staining mix prepared in 1× perm/wash and resuspend by pipetting three times up and down.
14. Incubate for 30 min on ice, protected from light.
15. Add 150 μL 1× perm/wash to each well and centrifuge for 5 min at 600 × *g* at 4°C.
16. Wash 2x with FACS buffer and spin down by centrifugation for 5 min at 600 × *g* at 4°C.
17. Aspirate supernatant and resuspend cells in 100 μL FACS buffer and analyze by flow cytometry cell sorting in the desired format.

##### 1.3.4 Intranuclear staining of transcription factors.

1. Transfer up to 2 × 10^5^ PBMC to a 96-well round-bottom plate.
2. Wash cells 2× with wash buffer for 3 min 400 × *g* at RT.
3. Stimulate the cells if required and according to your needs.
4. Spin down for 5 min at 400 × *g* at RT and wash 2× with PBS 1×.
5. Meanwhile, prepare live/dead exclusion dye staining mix according to manufacturer’s instruction.
6. Add 50 μL live/dead staining and incubate for 30 min at RT, protected from light.
7. Add 150 μL and wash 2× with FACS buffer by centrifugation for 5 min at 400 × *g* at RT.
8. Prepare fresh fixation/permeabilization reagent (eBioscience) by mixing one part of fix/perm concentrate and three parts of fix/perm dilution reagent.
9. Discard the supernatant and add 100 μL of fixation reagent to each well and resuspend by pipetting three times up and down.
10. Incubate for 30 min RT.
11. Add 100 μL and wash 2× with permeabilization buffer (1:10 dilution with H_2_O) by centrifugation for 5 min at 600 × *g* at RT.
12. Discard the supernatant and add 50 μL intracellular staining mix prepared in 1× perm/wash and resuspend by pipetting three times up and down.
13. Incubate for 30 min RT, protected from light.
14. Add 150 μL 1× perm/wash to each well and centrifuge for 5 min at 600 × *g* at RT.
15. Wash 2× with FACS buffer and spin down by centrifugation for 5 min at 600 × *g* at RT.16. Aspirate supernatant and resuspend cells in 100 μL FACS buffer and analyze by flow cytometry in the desired format.

#### 1.4 Materials

##### 1.4.1 Monoclonal Abs.

###### 1.4.1.1 Surface staining.

####### Beckman Coulter:

CD8-PE/Cy5 (clone B9.11), CD19-PE/Cy5 (clone J3-119), CD56-PE/Cy5 (clone N901), CD8-FITC (clone B9.11).

####### BD Biosciences:

CD25-PE (clone M-A251), CD25-PE/Cy5 (M-A251), CD127-FITC (clone HIL-7R-M21), CD127-PE/Cy5 (clone A019D5), CD127-PE/Cy5 (clone M-A251), TCRγδ-BUV737 (clone 11F2), CD183-PE/Cy5 (CXCR3) (clone 1C6/CXCR3), CD194-PE/Cy7 (CCR4) (clone 1G1), CD196-PE (CCR6) (clone 11A9), CCR6-APC (clone 11Ag), CCR10-PerCP/Cy5.5 (clone 1B5), C27-BV510 (clone L128), CCR4-BV421 (clone 1G1 RUO),

####### Biolegend:

PE/Cy7-Streptavidin, CCR7-BV421 (clone G043H7), CD95-BV605 (clone DX2), CD19-AF700 (clone HIB19), CD14-APC/Cy7 (clone HCD14), CD183-AF647 (CXCR3) (clone G025H7), CD196-BV605 (CCR6) (clone G034E3), CD4-APC/Fire 750 (clone RPA-T4), CD127-BV510 (clone AO19D5), CD25-PE/Cy7 (clone M-A251), CD8-biotin (clone 28.2)

####### Bio-Techne:

anti-hu-CXCR5 (clone 51505).

####### Invitrogen:

CD127-PE (clone eBioRDR5), CD4-FITC (clone OKT4), ICOS-PB (clone ISA-3).

####### Miltenyi:

TCRαβ-VioGreen (REA652), eFluor450-Streptavidin

####### R&D Systems:

CXCR5-APC (clone#51505).

####### Southern Biotech:

biotinylated secondary goat anti-mouse IgG2b.

####### ThermoFisher Scientific:

CD4-PE/Texas Red (clone S3.5), CD45RA-Qdot 655 (clone MEM-56). 26

###### 1.4.1.2 Intracellular/intranuclear staining.

####### BD Biosciences:

IFN-γ-FITC (clone B27), IL-4-PE (clone 8D4-8) or IL-4-APC (clone MP4-25D2),

RORγT-PE (clone Q21-559), GATA3-AF488 (clone L50-823), BCL6-PE (clone K112-91),

Granzyme B-FITC (clone GB11).

####### Biolegend:

IL-10-PE/Cy7 (clone JES3-9D7), T-bet-BV421 (clone 4B10).

####### eBioscience:

IL-17A-eFluor660 or eFluor450 (clone eBio64DEC17), IL-22-PerCP/eFluor710 (clone 22URTI).

####### Miltenyi:

CRTH2-PE (clone REA598).

####### R&D Systems:

GM-CSF-PE (clone 6804).

####### ThermoFisher Scientific:

Foxp3-FITC (clone PCH101), Helios-AF647 (clone 22F6).

##### 1.4.2 Media and buffers.

- Ficoll-Hypaque Plus (GE Healthcare, endotoxin tested, cat. no. 17-1440-03)
- FCS (fetal calf serum) batch-tested for low endotoxin
- PBS 1× (Gibco DPBS, no calcium, no magnesium; cat. no. 14190144)
- Culture medium:
  - RPMI 1640
  - 2 mM glutamine
  - 1% non-essential amino acids
  - 1 mM sodium pyruvate
  - 50 μM β-mercaptoethanol
  - 1% penicillin/streptomycin
  - 5% human serum (HS) or 10% FCS
- Washing medium:
  - RPMI-1640 w/ Hepes (25mM)
  - 1% FCS or 0.5% HS
- Flow cytometry buffer (FACS buffer):
  - Phosphate buffered saline (PBS 1×)
  - 2.5% FCS or 1% HS
  - 0.01% (w/v) sodium azide (to be added in the case of long-term storage)
  - 2mM EDTA pH 8.0 (to prevent clots)
- Stimulation mix:
  - Culture medium
  - 1 μg/mL Ionomycin (Sigma-Aldrich, cat. no. I0634):
  - 2 × 10^−7^ M PMA (Sigma-Aldrich, cat. no. P8139)
  - 10 μg/mL BFA (Sigma-Aldrich, cat. no. B7651)
- Cytofix/Cytoperm 1× solution (BD Biosciences; cat. no. 554722)
- 1× Perm/Wash (always prepare freshly before use):
  - 10% 10× perm/wash (BD Biosciences; cat. no. 554723)
  - 90% ddH_2_O
- Fixation buffer (Foxp3 kit eBioscience; cat.no. 00-5523-00) for intranuclear staining of transcription factors:
  - 75% Fixation/Permeabilization Diluent (cat. 00-5223)
  - 25% Fixation/Permeabilization Concentrate (cat. 00-5123)
- Permeabilization Buffer (Foxp3 kit eBioscience; cat.no. 00-5523-00) for intranuclear staining of transcription factors:
  - 90% Fixation/Permeabilization Diluent (cat. 00-5223)
  - 10% Permeabilization Buffer (10×) (cat. 00-8333) 19

Long-pass dichroic filters were used to filter light so that only wavelengths lower than filter A and higher than filter B are detected. DPSS – Diode Pumped Solid State, APC- allophycocyanin, Cy- cyanin, QD- quantum dot, PE-R-phycoerythrin, Axalexa, FITC- fluorescein isothiocyanate, pacific blue, Percp, peridinin chlorophyll protein; Aqua Blue- LIVE/DEAD Fixable Aqua Dead Cell Stain.

#### 1.5 Data analysis

The following descriptions and figures specifically relate to the identification of T cells from human blood samples. However, these general principles can also be applied to the detection of T cells from other tissues. Standard procedures for the isolation of PBMCs can be used for the study of human T cells, including density gradient centrifugation with Ficoll-Paque at a density of 1.077 g/mL. For analysis of flow cytometry data (recorded with the optical setup described in Table 6), FACS Diva or FlowJo softwares (BD) can be used. A typical gating strategy for detecting human blood T cells is depicted in Figure 7. First, lymphocytes and single cells should be gated, after which the compensations should be verified and adjusted. Lymphocytes are identified based on the FSC and SSC, whereas single cells can be discriminated from doublets by plotting either the pulse area and height of the FSC against each other, or the SSC height versus SSC width parameters. Single-fluorochrome staining and fluorescence-minus-one controls should be used to calibrate the compensation. General gating includes the exclusion of dead cells using a live/dead fixable dye and the selection of CD14^−^CD19^−^CD56^−^ cells to identify the αβ-TCR^+^ and γδ-TCR^+^ T cell populations. αβ-TCR^+^ T lymphocytes are then separated into CD4^+^ and CD8^+^ T cells. Conventional CD4^+^ T cells and Treg cells can be distinguished based on the differential expression of the surface markers CD127 and CD25 and/or by the intracellular expression of the transcription factors Foxp3 and Helios, as shown in Figures 8A and 10D. To identify the differentiation stages of conventional CD4^+^ T cells (CD127^+^CD25^−/low^), the combined staining of the surface markers CCR7, CD45RA, CXCR5, and CD95 is instrumental to distinguish between naïve and memory T cell subsets (Tscm, Tcm, Tem, Temra, and cTfh) (Figure 8A). The different T helper subsets can be recognized in the CD4^+^ T cell memory population based on the differential expression of the chemokine receptors CCR6, CXCR3, CCR4, and CCR10 (Figure 8C) and/or by intracellular cytokine staining of IFN-γ, IL-4, IL-17A, and IL-22 (Figure 10). T helper subsets can be also characterized based on the intranuclear staining for the transcription factors T-BET, RORC2/RORγT, GATA-3 and BCL6 (Figure 10). CD4^+^ Ctl expressing GzmB can be identified among conventional T cells based on the absence of CD27 and CD28 expression, as shown in Figure 8D. The common markers of human CD4^+^ T cells are described above and listed in Table 7.

#### 1.6 Pitfalls

Multiparametric flow cytometry is a powerful technique but some pitfalls should be considered:

- Quality and storage of samples may interfere with flow cytometry markers:
  Fresh samples generally allow a better performance than cryopreserved ones. Especially when using samples like liquor from the central nervous system (CNS), fresh samples should be used within 2–3 h to avoid cell death. For blood, it is important to consider that the anticoagulant used to store the samples may interfere with some protein expression.
- Staining
  When many fluorochromes are used in a staining panel, particular attention should be paid regarding mix performances:
  - Conjugated Abs that work properly alone or in a small mix, may show a different performance when used in combination with many others and signal may be lost or covered;
  - Some conjugated Abs may work well in particular buffers and do not work with some others;
  - Some markers are not readily detectable on ex vivo samples and an in vitro stimulation or pre-treatment may be needed;
  - On the contrary, stimulation (e.g., with PMA/Iono) may interfere with the expression of some other markers by downregulating them (e.g., CD127, CXCR3, CCR5)
- Acquisition of data
  - Flow cytometry data should be acquired with carefully optimized cytometer settings in order to avoid overcompensation and loss of signal sensitivity. Always be sure to acquire with the best setting of parameters for the specific sample, checking for voltage and compensation.
  - Single-staining and fluorescence-minus-one (FMO) controls are necessary when building flow cytometry panels to help determining the compensation matrix and properly setting the gates.

#### 1.7 Top tricks

When isolating PBMCs (5.3.1. Isolation of PBMCs):

- accurately washing cells at step 3 is critical to remove residual Ficoll that may be toxic for the isolated cells.
- before the last wash (step 5) cells can be filtered on a 40 μm cell strainer to remove eventual dead cell aggregates.

When performing the surface staining (5.3.2. Surface staining):

- always use appropriately titrated Abs, which is usually not the concentration suggested by the supplier. The ins and outs of titrating Abs can be found in the publication of Lamoreaux et al. [174].
- the detection of some surface molecules, such as chemokine receptors, may need optimization also of staining temperatures [141].
- Final staining results may depend on fluorochromes conjugated to the Abs and their combinations. Always try your panels to optimize staining results.

When performing intranuclear staining of transcription factors (5.3.4. Intranuclear staining of transcription factors):

- Some transcription factors (e.g., T-bet) are not readily detectable upon ex vivo isolation and may need T cell stimulation, for instance with α-CD3/α-CD28 Abs, to be properly discerned.

Regarding the culture medium (5.4.2. Media and Buffers):

- Usually, a culture medium with human serum performs better for long-term cultures, but may be dispensable for short-term stimulation.

#### 1.8 Clinical relevance statement

The analysis of chemokine receptor expression in blood-circulating CD4^+^ T cells can provide important information on potential dysregulations of Th-mediated immune responses in pathological conditions. Here, we provide an example of Th subsets distribution analysis in the blood from a healthy control and a septic patient with *Klebsiella pneumoniae* bloodstream infection.

**Figure 11.**
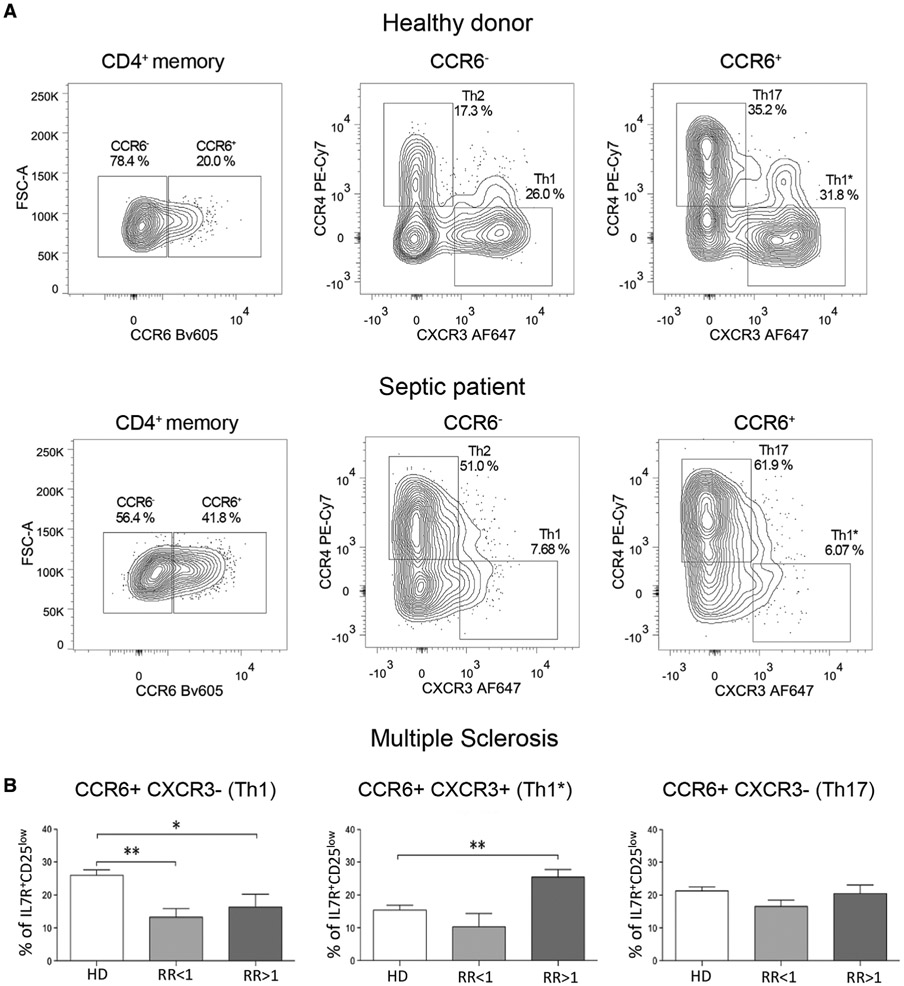
Clinical relevance: analysis of chemokine receptors expression in CD4^+^ T cells from patients. (A) T helper subsets distribution analysis based on chemokine receptors expression in the blood of a healthy donor (upper panels) and a patient (lower panels) suffering from *K. pneumoniae* bloodstream infection. (B) Reported frequencies of Th1, Th1* and Th17 cells in healthy donors (HD) and RR-MS patients (RR) with mild (MS score <1) or more severe (MS score >1) disease.

Septic patients showed a drastic reduction of circulating Th1 and Th1* subsets (Figure 11A) and selectively lacked *K. pneumoniae*-reactive memory Th cells as previously reported in ref. [175], thus suggesting that their impairment may correlate with the septic infection. Moreover, with a very similar monitoring strategy, we showed previously that CD4^+^ CXCR3^+^CCR6^+^ were selectively expanded in the circulation of severe relapsing-remitting multiple sclerosis (RR-MS) patients (Figure 11B) [176]. Shortly after an attack these Th1/17 or Th1* cells were enriched in the CSF and reacted strongly with myelin-derived self-Ags, suggesting a key pathogenic role in MS [176].

#### 1.9 Summary of the phenotype

This is detailed in Table 7.

### 2 Murine conventional αβ CD4 T cells

#### 2.1 Overview

CD4 T cells are central effector cells that crosstalk with many other components of the adaptive and innate immune system. Primed by signals they receive during development in the thymus and in the periphery, MHCII-restricted CD4 T cells specialize functionally into a range of distinct subsets that cover both regulatory and non-regulatory roles. Regulatory T cells (Treg) can suppress immune responses and balance between immune activation and tolerance (see also Chapter III, Section 12 Murine Foxp3^+^ regulatory T cells). Conventional non-regulatory CD4 T cells contribute to the efficiency of many vaccines and are vital for the protection against many infections with bacteria, parasites, and fungi, but they can also mediate autoimmune diseases. This section focuses on conventional non-regulatory CD4 T cells and gives an overview of surface markers used to identify the diverse CD4 T cells subsets. Furthermore, different populations of CD4 T cells can be identified by the detection of transcription factors and assays to analyze effector functions.

#### 2.2 Introduction

##### 2.2.1 *Conventional* αβ *CD4 T cells: Identification and surface markers.*

**Figure 12.**
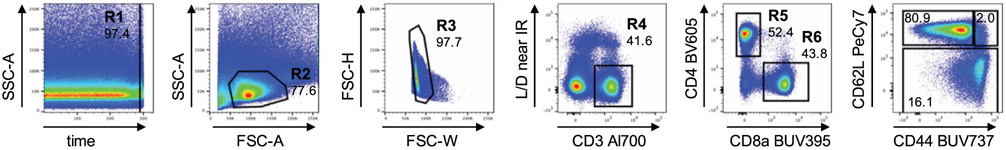
Murine CD4 and CD8 T cells. Sample gating tree for the identification of murine CD4 and CD8 T cell subsets from the spleen. Conventional CD4 and CD8 T cells can be identified by gating on time, lymphocytes according to FSC and SSC (R1, R2), exclusion of doublets (R3) and dead cells (R4) and gating on CD3ε^+^ or TCRβ^+^ cells (R4) and CD4^+^CD8α^−^ cells (R5) or CD4-CD8α^+^ cells (R6). Naïve, effector and memory T cell populations can then be defined within CD4 T cells using CD44 and CD62L expression to identify CD44loCD62Lhi naïve cells, CD44hiCD62Lhi central memory cells, and CD44^hi^CD62L^lo^ effector memory and effector cells.

Conventional TCRαβCD4^+^ T cells can be identified by gating on time, scatter parameters, and exclusion of duplicates and dead cells to identify viable lymphocytes and gating on CD3ε^+^ or TCRβ^+^ cells and CD4^+^CD8α^−^ cells (Figure 12). The use of CD3ε or TCR-β as selection markers is critical for the analysis of CD4 T cells to avoid the inclusion of myeloid cell subsets that express low levels of CD4. Additional markers may be required in specific tissues to differentiate conventional CD4 T cells from other non-conventional T cell subsets, such as from CD4^+^ NKT cells in the liver (see also section III.6 Murine tissue resident T cells). These markers can be used in a “dump” gate to exclude myeloid lineages and non-conventional T cells.

CD4 T cell differentiation states are often defined by the expression of surface markers that correlate with the location of cells within the body. Mature CD4 naïve T (Tn) cells are CD44loCD62Lhi and enriched in the lymphoid tissues (Figure 12 and Table 8). The L-selectin CD62L, mediates attachment to endothelia and access to lymph nodes. Upon activation, CD4 T cells acquire CD44, which binds hyaluronan to promote access to peripheral tissues, and lose CD62L to become CD4 effector T (Teff) cells. Antigen-activated Teff populations expand and mediate pathogen clearance, then contract after pathogen resolution and form CD4 memory T (Tmem) populations. Two main circulating memory populations are central memory T (Tcm) cells, which are CD44^hi^CD62L^hi^ and reside predominantly in the lymphoid tissue, and effector memory T (Tem) cells, which are CD44^hi^CD62L^lo^ and circulate through blood and peripheral tissues (Figure 12 and Table 8). Additionally, non-circulating tissue resident T cells (Trm) are primarily present in barrier tissues (see also section III.6 Murine tissue resident T cells).

**Figure 13.**
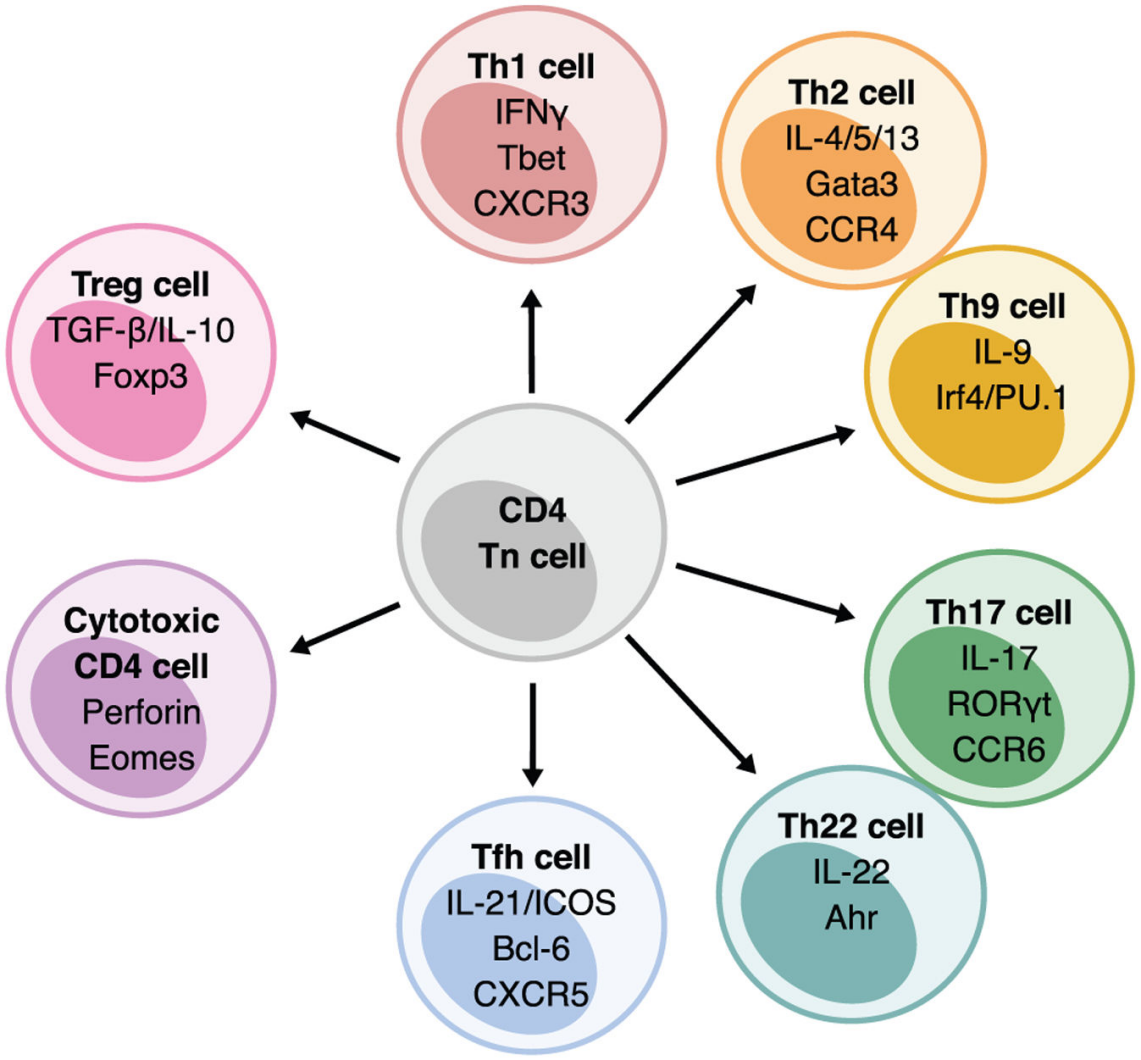
Schematic of murine CD4 T cell differentiation. An array of CD4 helper T cell subsets differentiate from CD4 Tn cells, including Th1, Th2, Th9, Th17, Th22, Tfh, Treg, and cytotoxic CD4 T cells. Molecules under each CD4 helper T cell subsets heading indicate the key effector cytokine/molecules, transcription factor/s, and chemokine receptors.

Pathogen-specific CD4 Teff (and Tmem) cells can be classified according to their phenotype and function into multiple T “helper” (Th) subsets; Th1, Th2, Th9, Th17, Th22, cytolytic CD4 T cells, and T follicular helper (Tfh) cells (Figure 13 and Table 8) (see also section III.1 Human conventional αβ CD4 T cells). These subsets are each equipped with a unique set of transcription factors, chemokine receptors, and effector molecules. However, the current view is that CD4 Th cell subsets are not separate lineages but have plasticity and form a continuum of mixed functional capacities [177, 178]. Alongside these conventional CD4 Th cell subsets, natural and induced Treg cells have a predominant immunomodulatory phenotype with the ability to suppress autoreactive immune responses and promote resolution of active immune responses, by a variety of mechanisms (for more details see Chapter III, Section 12 Murine Foxp3^+^ regulatory T cells).

CD4 Th cell subsets participate in a range of diverse and overlapping adaptive immune responses [179, 180]. Th1 cells are vital in the defense against intracellular infections, such as *Mycobacterium tuberculosis* and protozoa. Th2 cells protect against parasitic infections, including helminths, but also mediate much of the pathology associated with allergic reactions and correlate with asthma severity. Th17 cells, originally described in mice as being pathogenic in murine models of autoimmune disease [181, 182], have more recently been shown to protect against certain pathogens, including fungal infections [183]. Th9 and Th22 cells are relatively newly described subsets, which share some functional and developmental features with Th2 and Th17 cells, respectively. Tfh cells cross-talk with B cells to stimulate the production of high-affinity Abs in germinal center reactions. Intriguingly, in certain infections such as influenza, unique populations of CD4 T cells can exhibit cytolytic capacity [184].

**Figure 14.**
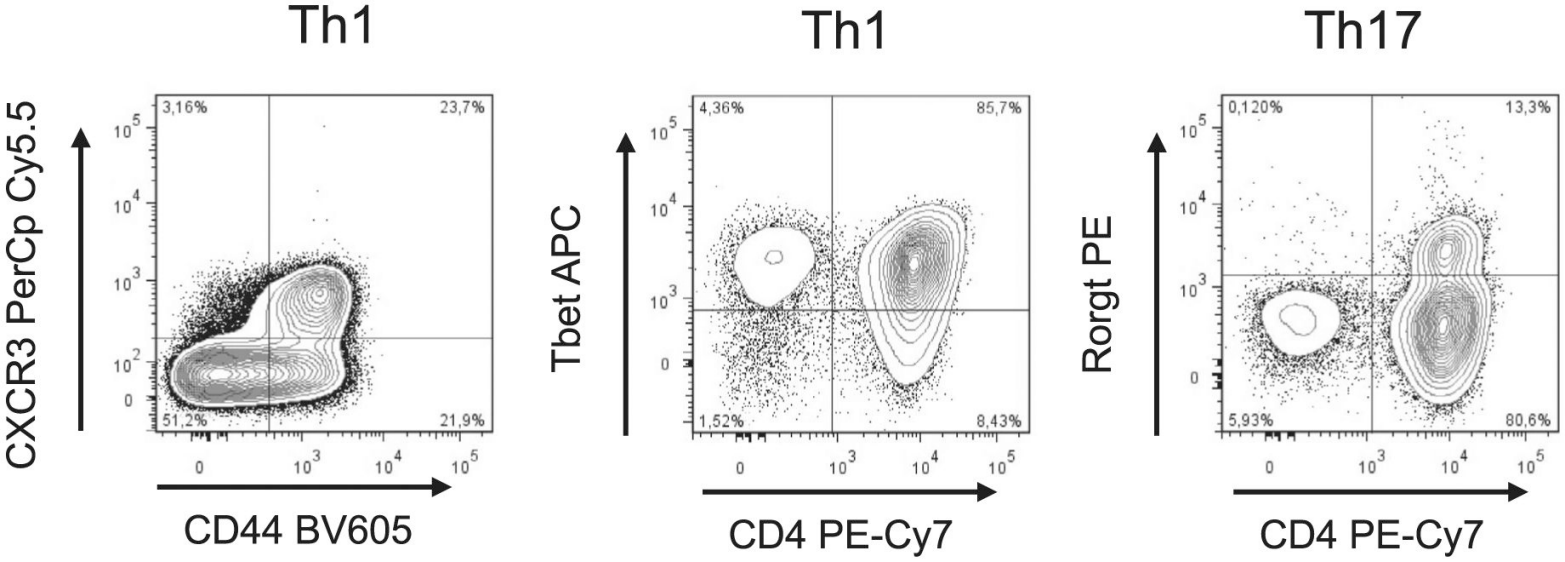
Chemokine receptors and transcription factors for identification of murine Th1 and Th17 CD4 cells. Subsets of CD4 T cells can be identified based on the expression of chemokine receptors. CD4 T cells were gated on lymphocytes according to scatter parameters, live cells and CD3^+^/CD4^+^ (see Figure 12) on murine splenocytes *ex vivo* or after expansion under polarizing conditions for the detection of the indicated chemokine receptors or transcription factors.

**Figure 15.**
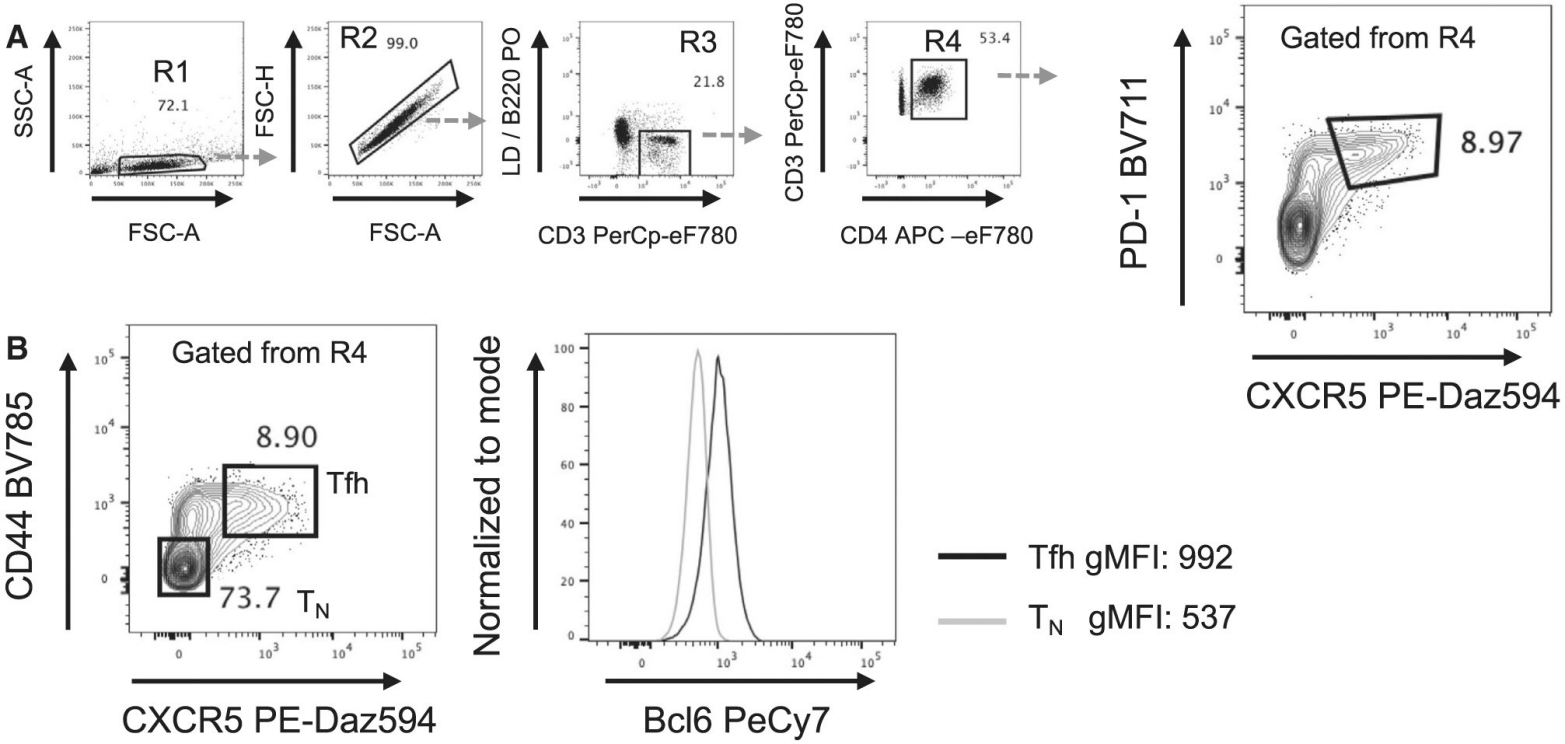
Assessment of Tfh cells. From murine splenocytes A) Tfh cells were identified by gating on lymphocytes (R1), single cells (R2), live, non-B, CD3^+^ cells (R3) and CD4^+^ cells (R4) before identifying Tfh cells through co-expression of high levels of CXCR5 and PD1. B) Higher expression of the transcription factor Bcl6 is detected in Tfh (CD44high/CXCR5high) compared to naïve (TN CD44low/CXCR5low) CD4 T cells.

In CD4 Th cells, the expression of chemokine receptors is associated with skewing toward specific effector functions and migratory behavior. Rapid upregulation of CXCR3 facilitates the migration of Th1 cells to inflamed tissue sites along gradients of chemokines, such as CXCL9, CXCL10, and CXCL11 (Figures 13 and 14 and Table 8) [185]. The specific interaction of CCR4 on Th2 cells with CCL17 and CCL22 is critical for the movement of Tmem into the skin [186]. Th17 preferentially utilize CCR6, also expressed by Treg cells, for migration to mucosal tissues that are enriched for CCL20 [153]. Tfh cells express the chemokine receptor CXCR5, which is vital in the migration of Tfh cells from the T cell zone into B cell follicles within the spleen [187] (Figure 13/15 and Table 8) and high levels of PD-1 (Figure 15 and Table 8) to facilitate B cell interactions.

##### 2.2.2 *Conventional* αβ *CD4 T cells: transcription factors, effector functions, and Ag-specificity.*

The differentiation of specific CD4 Th cell lineages is induced by specific cytokine stimulation and is guided by master transcription factors (Figure 13/14 and Table 8), which control the expression of downstream effector molecules. Priming of Th1 cells by IL-12 [188] and IFN-γ [189] results in expression of their master transcription factor T-bet [190], Th2 cell priming by IL-4 [191, 192] leads to expression of GATA-3 [193] and priming by IL-23, IL-6, and TGF-β drives RORγt expression in Th17 cells [194]. Th22 cells are regulated by expression of the transcription factor AHR [195, 196], while Th9 cells do not appear to be regulated by an individual transcription factor but rather a combination of factors, including IRF4 and PU.1 [195, 196]. Tfh cells are controlled by the transcription factor Bcl6 [197] (Figure 15) and the development of cytotoxic CD4 T cells can be mediated by the transcription of Eomes.

Transcription factors are mainly located intranuclearly and, to assess these by flow cytometry, staining buffers are used that efficiently permeabilize the nucleus and enable intranuclear access of Abs. When no reliable Abs are available, reporter mice are a valuable tool for the flow cytometric analysis of transcription factor expression [168]. Additionally, the use of reporter constructs can also enable functional assays based on transcription factor expression that are not possible with fixed and permeabilized cells.

Alongside surface markers and transcription factors, the helper phenotype and functional capacity of CD4 T cells can be examined by defining their production of cytokines and other effector molecules (Table 8) and their Ag specificity can be evaluated. This section will only highlight commonly used assays to detect effector function and Ag-specificity of murine CD4 T cells. Please refer to the linked specialized sections of the guidelines that discuss these methods in detail.

Most CD4 T cells are quiescent in steady state. Consequently, analysis of CD4 Th cell functional capacity by flow cytometry usually involves in vitro restimulation and subsequent effector protein profiling by intracellular staining (see Chapter V Section 14. Intracellular parameters and Chapter V Section 17.5 Functional read-outs). Similar to human CD4 T cells, hallmark cytokines for CD4 Th cell subsets can be detected (Figure 13). Although yet to be fully characterized, cytotoxic CD4 T cells have been identified through the expression of cytolytic molecules such as perforin [184]. CD4 T cells also upregulate the expression of the co-stimulatory molecule CD40L after activation [198], which is crucial for their T helper functions [199]; [200].

To trigger and detect their effector functions, CD4 T cells can be stimulated polyclonally (See V 14. Intracellular parameters in [22]) or stimulated in an Ag-specific manner with their cognate peptide presented by MHCII (Chapter V Section 17.5 Functional read-outs in [21]). After activation, T cells start to produce and/or secrete effector molecules including costimulatory surface molecules, cytokines, and chemokines.

The detection of effector functions by flow cytometry can be used to gain information about the properties of specific T cell subsets, but it is also utilized to enumerate Ag-specific CD4 T cells. To evaluate the total magnitude of an Ag-specific response using functional measures, an effector response should be selected that will be present in the majority of Ag-specific CD4 T cells following restimulation. A particularly useful marker for assessing total response magnitude for CD4 T cells is CD40L, as this marker is rapidly expressed on CD4 T cells after activation [201]. Unfortunately, it can be difficult to stain for CD40L, as it is transiently expressed on the cell surface and then rapidly secreted or internalized and degraded after interaction with its ligand, CD40. To circumvent this issue, cells can be either stained intracellularly for CD40L in the presence of BrefA or stimulated *in vitro* in the presence of both fluorescently labeled Ab against CD40L and blocking CD40 Ab [201]. Staining for CD40L in this way thereby permits evaluation of the majority of the Ag-specific CD4 T cell response.

For the identification of live CD4 T cells producing a certain cytokine, a cytokine secretion assay can be used (see section IV.8 Live cytokine-producing cell sorting with cytokine secretion assay^™^).

Ag-specific CD4 T cells can be detected indirectly using *in vitro* restimulation with defined Ags, but Ag-specific CD4 T cells can also be directly identified, analyzed, and tracked in mice using a number of methods. First, CD4 T cells specific for a given Ag can be detected by MHCII tetramers/multimers (see section IV.3 Antigen-specific T cell cytometry: MHC multimers). Of note, tetramer staining can exhibit non-specific binding and high background. To improve the identification of low-frequency tetramer^+^ T cells, staining with the same MHCII tetramer labeled with two different fluorophores can be used (see section IV.3 Antigen-specific T cell cytometry: MHC multimers). Another strategy to track Ag-specific responses *in vivo* is to transfer congenically labeled or fluorescently labeled TCR transgenic T cells. Different transgenic mouse strains expressing TCRs specific for a number of Ags and derived from CD4 T cells have been developed, including ovalbumin (OT-II), LCMV glycoprotein (SMARTA), and malarial Ag (pBT-II). Allelic variants of the cell surface molecules CD90 (Thy-1) and CD45 (Ly-5) can be distinguished with selective Abs and allow tracking of adoptively transferred T cells in congenically distinct recipients. T cells can also be genetically modified to stably express fluorescent proteins, such as Green Fluorescent Protein (GFP), yellow (Y)FP, and red (R)FP, to track transferred cells or act as reporters for deletion or expression of genes in genetically modified mice. Fluorescent dyes, such as carboxyfluorescein succinimidyl ester (CFSE) and CellTrace^™^ Violet (CTV) (see Chapter IV, Section 5: Adoptive T cell transfers as a readout for Ag-specific immune responses in mice), can be used to label cells, which are then transferred into hosts to track migration or cell division *in vivo,* although the fluorescent signal from these dyes is lost with protein turnover and so they can only be detected for a finite period. Functional indicators of Ag-specificity can also be used. Activated T cells that are actively dividing, can be identified *in vivo* or *in vitro* by uptake of Bromodeoxyuridine (BrdU) (See Chapter V Section 6.3 DNA synthesis—Nucleotide incorporation in ref. [22]) or by intranuclear staining with the proliferation-associated marker Ki-67.

#### 2.3 Step-by-step sample preparation protocol for staining of Tfh CD4 T cells

##### 2.3.1 Cell isolation.

- Dissect draining spleen, lymph nodes (LNs), and non-draining LNs in HBSS (or PBS).
- Generate single-cell suspension by gently disrupting the tissue and filtering the cell suspension through 70 μm cell strainer or equivalent.
- Recommended: Red blood cell lysis can be performed using ACK lysing buffer or equivalent on spleen samples.
- Resuspend cells in FACS buffer and count.

##### 2.3.2 Staining.

- Transfer 1–2 × 10^6^ cells in 100 μL of FACS buffer into 96-well round-bottom plate
- Centrifuge at 400 × *g*, 5 min, remove supernatant
- Resuspend cells in 100 μl of FcγR block solution.
- Incubate for 10 min at 4°C.
- Centrifuge at 400 × *g*, 5 min, remove supernatant
- Resuspend cells in 100 μl of CXCR5 Stain (anti-CXCR5 PE-Daz594 in cRPMI).
- Incubate for 25 min at 4°C.
- Wash cells: add 100 μl FACS buffer, centrifuge at 400 × *g* 5 min at 4°C, remove supernatant
- Resuspend cells in 50 μl of Viability Stain (LIVE/DEAD^™^ Fixable Aqua viability dye at 1:1000 in PBS).
- Incubate for 15 min at 4°C.
- Wash cells once with 100 μl of FACS Buffer.
- Resuspend cells in 80ul of Surface Stain (anti-B220, -CD3, -CD4, -CD44, and -PD1 in FACS buffer).
- Incubate for 15 min at 4°C.
- Wash cells once with 200 μl of FACS Buffer.
- Resuspend cells in 100 μL Fixation/Permeabilization buffer from the eBioscience Foxp3/Transcription Factor Staining Buffer set.
- Incubate overnight at 4°C.
- Add 100 μl 1× FoxP3 perm buffer, centrifuge at 400 × *g* for 5 min at 4°C
- Resuspend cells in 100 μL of Intranuclear Stain (anti Bcl6-PECy7 in 1× FoxP3 permeabilization buffer from the eBioscience Foxp3/Transcription Factor Staining Buffer set).
- Incubate for 45 min at room temperature.
- Wash cells twice with 100 and 200 μl of 1× FoxP3 permeabilization buffer, respectively.
- Resuspend cells in 200 μl of FACS buffer and analyze on a flow cytometer.

#### 2.4 Materials

- Single cell suspension containing T cells
- FACS buffer: PBS with 0.2% BSA
- cRPMI: RPMI with 10% FCS
- Foxp3/Transcription Factor Staining Buffer Set (eBioscience, cat# 00-5523-00)

Antibodies:

- FcγR block: anti-mouse CD16/32 (clone 2.4G2)
- Anti-mouse B220-Pacific orange (PO; in house conjugated, clone RA3.6B2)
- Anti-mouse CD4 APC-eF780 (eBioscience, cat# 47-0042, clone RM4-5, 1:400)
- Anti-mouse CD3 PerCP-eF710 (eBioscience, cat# 46-0033-82, clone ebio 500A2, 1:300)
- Anti-mouse CXCR5-PE-Daz594 (Biolegend, cat# 145521, clone L138D7, 1:100)
- Anti-mouse CD44-BV785 (Biolegend, cat# 103041, clone IM7, 1:100)
- Anti-mouse PD1-BV711 (Biolegend, cat# 109110, clone RMP1-30, 1:100)
- Anti-Bcl6-PECy7 (BD Biosciences, cat# 563582, clone K112-91, 1:20)
- LIVE/DEAD^™^ Fixable Aqua viability dye (Invitrogen, L34957, 1:1000)

#### 2.5 Data analysis

After gating on lymphocytes in FSC versus SSC, single cells, and live B220 negative cells, CD3^+^ and CD4^+^ T cells are selected (see Figure 15). Cells are then displayed on CXCR5 versus PD-1 and Tfh cells are identified as CXCR5^++^PD-1^++^ cells.

#### 2.6 Pitfalls

- B cells also exhibit high expression of CXCR5 and upregulate Bcl6 to enter B-cell follicles. It is therefore critically important to ensure gating for Tfh excludes the inclusion of B cells, such as through negative depletion prior to flow cytometry or identification and gating out B cells by positive staining for CD19 and/or B220 during acquisition.

#### 2.7 Top tricks

- Currently, the Abs available for staining CXCR5 and Bcl6 for flow cytometry are relatively challenging to work with. To ensure optimal staining:
  - Include a viability dye, as dead cells often autofluoresce and stain with markers nonspecifically.
  - Apply a stringent singlet gate, as doublets of CXCR5^+^ B cells bound to PD-1^+^CXCR5^−^ T cells will appear to be PD-1^+^CXCR5^+^ Tfh cells.
  - Use a control tissue that contains very few Tfh, such as LN from an unimmunized mouse, to set your Tfh gates.

#### 2.8 Summary of the phenotype

This is detailed in Table 8.

#### 2.9 Key information human versus murine

Markers used to identify human and murine cells can differ. Always check that the marker is appropriate for the species you are analyzing. For example, while CD44 and CD62L (or CCR7) are used to identify CD4 Tn versus Tcm versus Tem cells in mice, human analyses use CD45RA in combination with CCR7, CD62L, or CD27.

### 3 Human conventional αβ CD8 T cells

#### 3.1 Overview

The expression of specific cell surface or intracellular proteins identifies the differentiation state of CD8^+^ T cells, which may hold clinical relevance in several settings. Recent discoveries have added new entries in the classification of the CD8^+^ T cell compartment, even in fractions previously considered to be homogeneous such as naïve and stem cell-like memory T cells. Flow cytometry is a powerful tool that allows to address CD8^+^ T cell heterogeneity in a high-dimensional and high-throughput manner. Depending on the specific questions raised, flow cytometry panels can be adapted to obtain the desired resolution of CD8^+^ T cell heterogeneity.

#### 3.2 Introduction

Conventional αβ CD8^+^ T cells form a crucial part of our defense system against viruses and malignant cells. In contrast to innate immune cells, which respond to a broad range of pathogens in a non-specific fashion, conventional CD8^+^ T cells harbor specificity for defined epitopes through the expression of an αβ TCR. CD8^+^ T cell responses are typically primed by professional APCs in the secondary lymphoid organs (SLOs). Upon recognition of their cognate Ag(s), naïve CD8^+^ T cells are activated, proliferate, and differentiate into short-lived effector cells (SLEC) or memory-precursor effector cells (MPEC), which can migrate to peripheral and inflamed tissues where they eliminate their target cells. Once the infection is resolved, the vast majority of effector T cells undergoes apoptosis, but a small fraction of memory precursors survives and develops into a population of long-lived memory cells [202]. Ag-specific memory T cells are more abundant than their naïve counterpart and furthermore display a poised epigenetic state that, among other factors, allow faster and stronger recall responses [202], thereby conferring enhanced protection against secondary infections. Yet, the memory CD8^+^ T cell pool is highly heterogeneous, being comprised of a multitude of subsets, each with specific features of homing, proliferation, self-renewal, multipotency, effector function, and metabolism [81, 203, 204]. Detection or targeting of specific CD8^+^ T cell subsets is key to successful T cell-based immunotherapies [205]. For instance, several studies have shown that a subset of early differentiated T cells with stem-like characteristics, i.e., with the capability to persist and exert effector functions in the long term, is responsible for superior tumor regression upon adoptive transfer [81, 203]. High-dimensional flow cytometry allows to dissect memory T cell heterogeneity in a high-throughput manner. Here, we review the cellular markers that best define human conventional αβ CD8^+^ T cell heterogeneity.

Naïve CD8^+^ T cells (Tn CD8) develop in the thymus and following positive and negative selection are released in the circulation. The naïve CD8^+^ T cell pool has traditionally been viewed as phenotypically homogeneous, identified through expression of the CD45RA isoform of the CD45 gene, and lymph node homing receptors CCR7 and/or CD62L (L-selectin). In contrast to naïve cells, memory T cells preferentially express the CD45RO isoform. A memory T cell population with high multipotency potential, labeled stem cell memory T cells (T_SCM_), was found to be included in this original Tn CD8 gating strategy [140, 206]. T_SCM_ could be distinguished from Tn CD8 by the expression of surface receptor CD95, which can be used as a general marker of memory CD8^+^ T cells. Therefore, to correctly identify Tn CD8, a flow cytometry panel should include at least CD95 combined with CCR7 or CD62L. Since cryopreservation leads to a significant loss of CD62L expression, CD95 and CCR7 is the preferred combination for frozen samples. In addition, we have recently shown that CCR7^+^CD95^−^CD45RA^+^ Tn CD8 cells still exhibit signs of heterogeneity at the population level, as CXCR3 expression identifies Tn CD8 cells with increased potential to differentiate into effector cells [207]. The identification of naïve precursors with functional attributes may be useful to derive more potent effectors for future immunotherapeutic approaches (e.g., CAR-T cell therapy; CAR = chimeric Ag receptor). As cryopreservation heavily affects CXCR3 expression, staining of fresh samples is recommended for the correct identification of the two Tn CD8 subsets.

The exact model of how memory CD8^+^ T cells differentiate from precursors is still debated. Nevertheless, it is accepted that a fraction of the memory CD8^+^ T cell pool represents an early differentiated stage that has the capacity to self-renew and generate more differentiated progeny. Adoptive transfer studies have demonstrated that early differentiated memory T cells, such as those with stem-like properties, exhibit high persistence *in vivo* [140].

These stem-like memory T cells initially separated CD95^+^CD45RA^+^CD45RO^−^CCR7^+^CD62L^+^ T_SCM_ from the more differentiated, yet long-lived CD95^+^CD45RA^−^CD45RO^+^CCR7^+^CD62L^+^ central memory T cells (**Tcm**). However, a recent report from our lab has shown that contamination from a transcriptionally, epigenetically and clonally distinct subpopulation of progenitor exhausted T cells (**T**_**PEX**_; see below) expressing CCR7 and CD95, yet discordant for expression of the inhibitory receptors PD-1 and TIGIT, is at the basis of the functional and phenotypic difference previously ascribed to T_SCM_ and Tcm [208]. These data raise the question whether maintaining the distinction between the latter two remains meaningful. Both T_SCM/CM_ and T_PEX_ express many markers associated with naïve T cells, including co-stimulatory molecules CD27 and CD28. In addition, they express IL-7 receptor alpha chain (CD127) and IL-2 receptor beta chain (CD122), which together with the common gamma chain (CD132) allow long-term survival and self-renewal in response to the homeostatic cytokines IL-7 and IL-15. T cell factor 1 (TCF-1) is a key transcription factor responsible for stem-like memory CD8^+^ T cell maintenance. Instead, T-bet, a transcription factor promoting effector differentiation, is generally low in T_SCM/CM_, while it is increased in T_PEX_ *ex vivo.* These transcription factors undergo dynamic changes that are subset-specific upon activation *in vitro.*

More differentiated effector memory T cells (**Tem**) generally express high levels of T-bet and of other transcription factors driving terminal differentiation, such as ZEB2 and BLIMP1, and lack lymphoid organ-homing molecules CCR7 and CD62L. Tem often express chemokine receptors such as CXCR3 and CCR5, which enable their migration into tissues where they can unleash their rapid cytotoxic potential. As such, Tem highly express cytotoxic effector molecules including granzyme B, perforin and granulysin. Though Tem typically lack expression of CD27 and CD28, a subset of peripheral blood CD8^+^ T cells has been shown in some reports to be phenotypically intermediate between T_SCM/CM_ and Tem (sometimes referred to as transitional memory, or T_TM_ cells; reviewed in [81]). These cells, which are particularly abundant among HIV-specific CD8^+^ T cells [209-212], have downregulated CCR7 and CD62L, but still express CD27 and/or CD28.

As humans age, the relative abundance of effector memory CD8^+^ T cells over naïve and stem-like memory CD8^+^ T cells increases, an effect that is significantly enhanced in individuals chronically infected with cytomegalovirus (CMV). Many CMV-specific CD8^+^ T cells exhibit a terminal effector T cell (**T**_**TE**_) phenotype with re-expression of CD45RA (sometimes referred to as **TEMRA**). The functional significance for this switch in CD45 isotype with terminal differentiation is still unknown. Terminal differentiation at the functional level might therefore be more specifically assessed through detection of CD57 or high levels of KLRG1. Furthermore, T_TE_ preferentially express high levels of the fractalkine receptor CX_3_CR1 [213, 214], which facilitates their migration into inflamed tissues.

A population of noncirculating, tissue resident memory T cells (**Trm**) offers protection in mucosal and epithelial layers against invading pathogens, and can be identified through expression of tissue retention molecules CD103 and/or CD69. T_RM_ are reviewed in detail in the section Human tissue resident memory T cells).

It has recently become apparent that T cell exhaustion is a separate branch of T cell differentiation. Exhaustion is an epigenetically-hardwired cell state characterized by reduced effector functions [215]. It is hypothesized that this differentiation pathway results from the immune system trying to balance protection and immunopathology in settings of persistent antigenic stimulation, such as in chronic viral infections and cancer [216]. In cancer patients, terminally exhausted cells (**T**_**EX**_) can be distinguished from conventional effector CD8^+^ T cells by increased expression of inhibitory receptors including PD-1, TIGIT, TIM-3 and LAG-3. T_EX_ have little capacity to proliferate or self-renew and are continuously generated from precursor/progenitor cells that express TCF-1, as described in chronic infection models and cancer [217-220]. These **T**_**PEX**_ are not just present in tumors [217], but also within SLOs and peripheral blood of healthy individuals, thereby suggesting that this trajectory of differentiation is not limited to the cancer setting, but that it is a physiological mechanism of differentiation in response to chronic Ags. T_PEX_ share traits with stem-like memory T cells including expression of CCR7, CD27 and TCF-1, but at the same time they display traits of exhaustion including PD-1 and TIGIT expression and diminished effector functions compared to T_SCM/CM_ [208]. Hence, the inclusion of these phenotypic markers in a flow cytometry panel enables their separation from T_SCM/CM_. Since PD-1 is not only a marker of exhaustion but also of recent activation, more specific identification of T_PEX_ can be achieved by combining staining for PD-1 with staining for intracellular granzyme K.

#### 3.3 Step-by-step sample preparation

##### 3.3.1 Freezing PBMC.

1. Dilute the heparinized blood or buffy coat 1:2 with 0.9% NaCl saline (Baxter).
2. Isolate PBMC by using Ficoll or lymphoprep according to manufacturer’s protocol (centrifuge for 30 min at 400 × *g*, without brake, at RT).
3. Collect the PBMC ring within the interphase in 50 mL tubes.
4. Add washing medium up to 50 mL and centrifuge for 10 min at 500 × *g* at room temperature.
5. To remove platelets, aspirate the supernatant, resuspend the pellet in 50 mL washing medium, and centrifuge for 10 min at 160 × *g* at room temperature. Repeat this step.
6. Resuspend in 10 mL washing medium and put on ice.
7. Count the cells and centrifuge for 10 min at 500 × *g* at 4 °C.
8. Make sure your cells, cryovials, and freezing medium are cold before freezing.
9. Resuspend in cold freezing medium to a concentration of up to 100 × 10^6^ cells/mL and aliquot 1 mL/vial.
10. Freeze the cryovials by using a Mr. Frosty (Nalgene), CoolCell (Corning), or a freezing apparatus at −80°C for a period of 24 h.
11. Store the vials until further use in liquid nitrogen.

##### 3.3.2 Thawing PBMC.

1. Thaw the vials by gently shaking in a 37°C water bath, until little ice remains.
2. Add 1 mL thawing medium (pre-warmed at 37°C) dropwise into the cryovial containing the PBMC.
3. Gently resuspend the cells and transfer them into a 50 mL tube with 18 mL thawing medium (pre-warmed at 37°C).
4. Centrifuge the cell suspension at 500 × *g* for 10 min at room temperature.
5. Aspirate supernatant, resuspend the pellet in 50 mL washing medium and centrifuge at 500 × *g* for 5 min at room temperature.
6. Gently resuspend the cells with washing medium and count the cells.

##### 3.3.3 Surface staining.

1. Transfer up to 10^7^ PBMC to 5 mL round-bottom polystyrene tubes.
2. Wash the samples with 2 mL 1× PBS.
3. Centrifuge at 500 × *g* for 5 min at room temperature and aspirate the supernatant.
4. Prepare live/dead exclusion dye in a total volume of 100 μL 1× PBS for each tube and add the mix to the samples.
5. Incubate for 15 min at room temperature, protected from light.
6. Meanwhile prepare the surface staining mix in a total volume of 100 μL FACS buffer for each well (prepare for 1 tube extra). If the Ab panel includes chemokine receptors prepare a dedicated mix with these Abs. Spin down every mix prior to use, in order to remove aggregated Abs.
7. Wash the samples with 2 mL FACS Buffer.
8. Centrifuge at 500 × *g* for 5 min at room temperature and aspirate the supernatant.
9. Add 100 μL chemokine receptor mix to each tube and incubate for 20 min at 37°C, protected from light.
10. Wash the samples with 2 mL FACS buffer.
11. Centrifuge at 500 × *g* for 5 min at room temperature and aspirate the supernatant.
12. Add 100 μL surface staining mix to each tube and incubate for 20 min at room temperature, protected from light.
13. Wash the samples with 2 mL FACS Buffer.
14. Centrifuge at 500 × *g* for 5 min at room temperature and aspirate the supernatant.
15. Fix the cells by adding 150 μL of fixing solution and incubate for 10 min under the chemical hood.
16. Wash the cells with 2 mL FACS buffer.
17. Centrifuge at 650 × *g* for 5 min at room temperature and aspirate the supernatant.
18. Resuspend the samples in 200 μL FACS buffer.
19. Keep the samples at 4°C until the acquisition.

Note: Always use appropriately titrated Abs, which is usually “not” the concentration suggested by the supplier. The ins and outs of titrating Abs can be found in the ref. [221].

#### 3.4 Materials

##### 3.4.1 Monoclonal Abs.

1. Surface staining:

###### BD Biosciences:

CD3 BUV496 (UCHT1), CD8 BUV805 (SK1), CD4 BUV615 (SK3), CCR7 PE-CF594 (150503), CD95 BUV563 (DX2), CD45RA BUV563 (HI100), and PD-1 BV480 (EH12.1).

###### eBioscience:

CD127 PE-Cy5 (eBioRDR5), TIGIT PerCP-eF710 (MBSA43).

###### BioLegend:

CD161 BV605 (HP-3G10), CXCR3 PE (G025H7), CD28 BV786 (CD28.2).

2. Live/dead exclusion dye: Zombie Aqua^™^ Fixable Viability Kit (423102, BioLegend).

##### 3.4.2 Flow cytometer.

All experiments were performed on a BD FACSymphony A5 flow cytometer with a 355 nm, 405 nm, 488 nm, 561 nm, and 640 nm configuration (BD Bioscience). Filters: 780/60(488) for BB790; 710/50(488) for PerCP-eF710; 670/25(488) for BB660; 610/20(488) for BB630; 530/30(488) for FITC or AF488; 780/60(640) for APC-H7; 730/45(640) for APC-R700; 670/30(640) for APC; 800/30(405) for BV786; 710/20(405) for BV711; 675/20(405) for BV650; 605/20(405) for BV605; 575/25(405) for BV570; 515/20(405) for BV510 or Zombie Aqua^™^ Fixable Viability Kit; 470/15(405) for BV480; 450/50(405) for BV421; 820/60(355) for BUV805; 740/35(355) for BUV737; 670/25(355) for BUV661; 615/24(355) for BUV615; 580/20(355) for BUV563; 515/30(355) for BUV496; 379/28(355) for BUV395; 780/60(561) for PE-CY7; 710/50(561) for PE-CY5.5; 670/30(561) for PE-CY5; 610/20(561) for PE-CF594; and 586/15(561) for PE.

##### 3.4.3 Media and buffers.

###### Thawing medium:

RPMI-1640, 10% FBS, 1% l-glutamine, 1% Penicillin/Streptomycin

###### Freezing medium:

FBS, 10% DMSO

###### Washing medium:

1× PBS without calcium and magnesium Culture medium: RPMI-1640, 10% FBS, 1% l-glutamine, 1% Penicillin/Streptomycin

###### Fixing solution:

1× PBS without calcium and magnesium, 1% formalin

###### FACS Buffer:

1× PBS without calcium and magnesium, 2% FBS

#### 3.5 Data analysis

**Figure 16.**
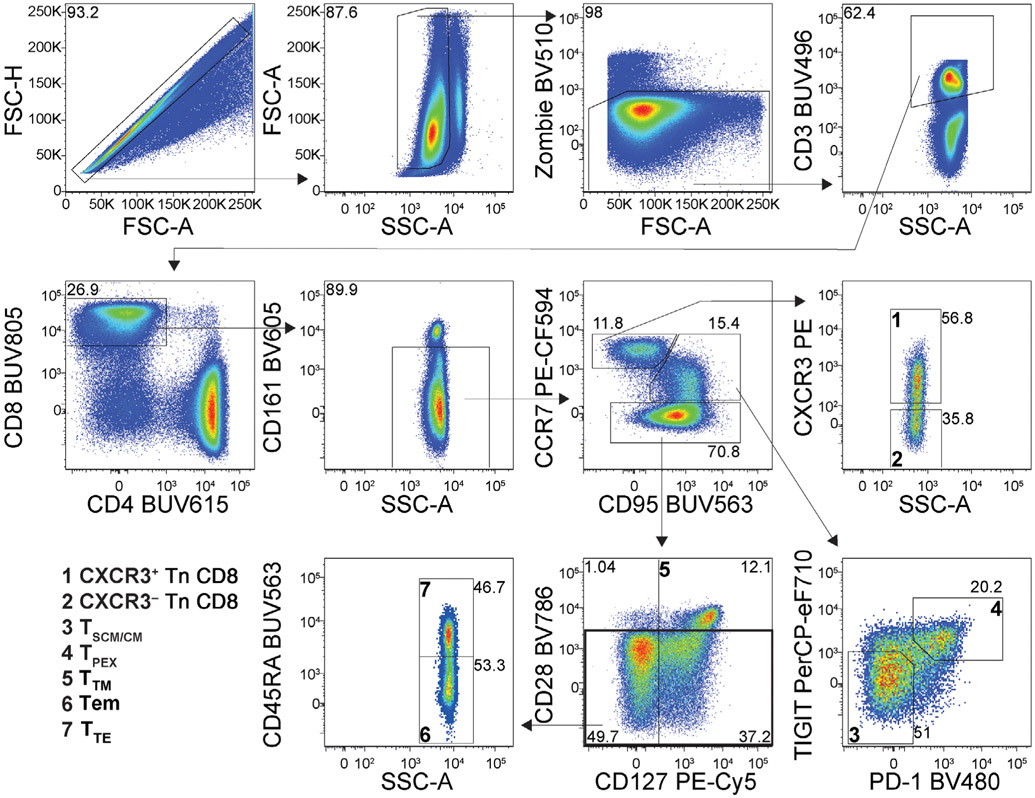
Gating strategy of human CD8^+^ T cell subsets in the peripheral blood. After doublet exclusion, lymphocytes are selected on the basis of physical parameters. Gating on live CD3^+^ T cells is followed by discrimination of CD8^+^ and CD4^+^ T lymphocytes. Within CD8^+^CD4^−^ T cells, the CD161^high^ MAIT population is excluded. CCR7^+^CD95^−^ cells Tn CD8 cells can be subdivided on the basis of CXCR3 expression. CCR7^+^CD95^+^ early memory T cells can be classified as TIGIT^+^PD-1^+^ (T_PEX_) or TIGIT^−^PD-1^−^ T_SCM/CM_. The CCR7^−^CD95^+^ compartment includes a population of CD28^+^CD127^+^ T_TM_, while the CD28^−^ cells can be divided into CD45RA^−^ Tem and CD45RA^+^ T_TE_.

The flow cytometric gating strategy of for the isolation of CXCR3^+^ Tn CD8, CXCR3^−^ Tn CD8, T_SCM/CM_, T_PEX_, T_TM_, Tem, and T_TE_ is shown in Figure 16 and phenotypes are summarized in Table 9. Data were acquired with a BD FACSymphony A5 and analyzed with FlowJo 9 as indicated in the figure legend.

#### 3.6 Pitfalls and top tricks

- Some markers (e.g., CD62L, CXCR3) are susceptible to freeze–thaw cycles. Thus, starting from fresh material when investigating these targets is mandatory.
- If evaluating intracellular molecules, we suggest to use the Fixation/Permeabilization Solution Kit (554714, BD Biosciences) according to the manufacturer’s instructions.
- If evaluating intranuclear molecules, we suggest to use the Transcription Factor Buffer Set (562574, BD Biosciences) or the FoxP3 Transcription Factor Staining Buffer Set (00-5523-00, eBioscience) according to the manufacturers’ instructions.
- Even though staining mixes have been properly spun down, some aggregated Abs can still be present. Be sure to exclude them with a proper gating strategy.
- When comparing patient cohorts, be sure to control for patient gender, age and CMV status, as this can strongly influence the skewing of phenotypes in the CD8^+^ T cell compartment.

#### 3.7 Clinical relevance statement

The gating strategy shown in this section is applicable for analysis of conventional αβ CD8^+^ T cells in any kind of physiological or clinical setting [207, 208, 222]. Note that in pathological settings, which are generally characterized by heightened inflammation, phenotypic changes may occur, so the final classification of conventional αβ CD8^+^ T cells should always be accompanied by defined functional assays.

#### 3.8 Summary of the phenotype

This is shown in Table 9.

### 4 Murine conventional αβ CD8 T cells

#### 4.1 Overview

MHCI-restricted CD8 T cells are one of the central effector cell populations of the adaptive immune system and contribute to protection against viruses, intracellular bacteria, and malignant cells. In this section, we provide examples for how to identify conventional CD8 T cells and use surface markers to determine functionally distinct populations of CD8 T cells in steady state and during an immune response. Furthermore, we give an overview of methods that can be used to analyse transcription factors, track Ag-specific CD8 T cell responses and measure CD8 T cell effector function.

#### 4.2 Introduction

##### 4.2.1 *Conventional* αβ *CD8 T cells: Identification and surface markers.*

Conventional TCRαβ CD8^+^ T cells can be identified by gating according to time, forward and side scatter, exclusion of doublets and dead cells, gating on CD3ε^+^ or TCR-β^+^ cells and finally gating on CD4^−^CD8α^+^ cells (Figure 12; see section III.4 Murine conventional CD8 αβ T cells). Gating on CD3ε^+^ or TCR-β^+^ T cells is useful to exclude myeloid cells or NK cells that express CD8α. Of note, this gating strategy can lead to the inclusion of unconventional T cells, such as intraepithelial lymphocytes (IELs), γδ T cells, NK T cells, and MAIT cells (see section III.8 Mouse intestinal intraepithelial T cells, section III.16 Unconventional murine T cells: γδ T cells, section III.18 Unconventional murine T cells: NKT cells and section III.20 Unconventional murine T cells: MAIT cells), as some of these cells express a CD8αα homodimer. These unconventional T cell populations can together comprise up to 50% of the CD8 T cell populations in some peripheral tissues, such as the small intestine. To avoid this misclassification, CD8β Abs should be included in gating strategies to exclude unconventional T cells that do not express this marker. The use of CD8β Abs can, however, reduce binding of MHCI tetramers and thereby limit the identification of Ag-specific CD8 T cells [223] (see section IV.3 Antigen-specific T cell cytometry: MHC multimers). These factors should therefore be taken into consideration when identifying Ag-specific populations in tissues that are rich in unconventional T cells populations.

**Figure 17.**
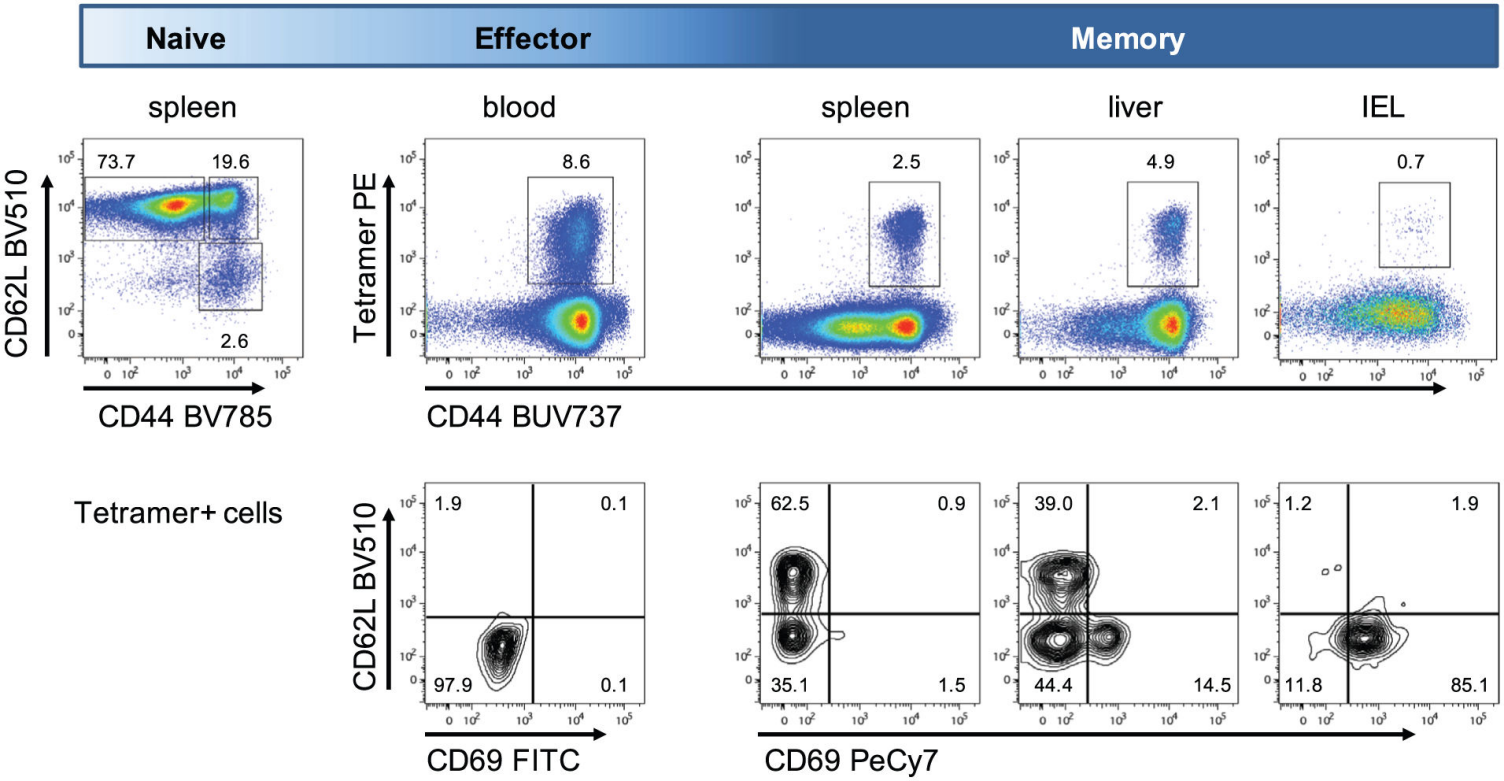
Discriminating murine CD8 T cell subsets. The expression of CD44, CD62L, and CD69 can be used to identify CD8 T cell populations in the different phases of the immune response. CD8 T cells from the spleen displayed in the top row were gated as shown in Figure 12. Naïve mice mainly contain naïve CD8 T cells. Pathogen-specific T cells can be identified using tetramer staining, here GP33-specific CD8 T cells after LCMV infection. During the effector phase (d8 post infection), the majority of LCMV-specific CD8 T cells upregulate CD44 and downregulate CD62L. In the memory phase (day 30+ post infection), T cells retain high expression of CD44 and can be divided in Tcm (CD62L^+^/ CD69^−^), Tem (CD62L^−^/ CD69^−^), and Trm (CD62L^−^/ CD69^+^).

**Figure 18.**
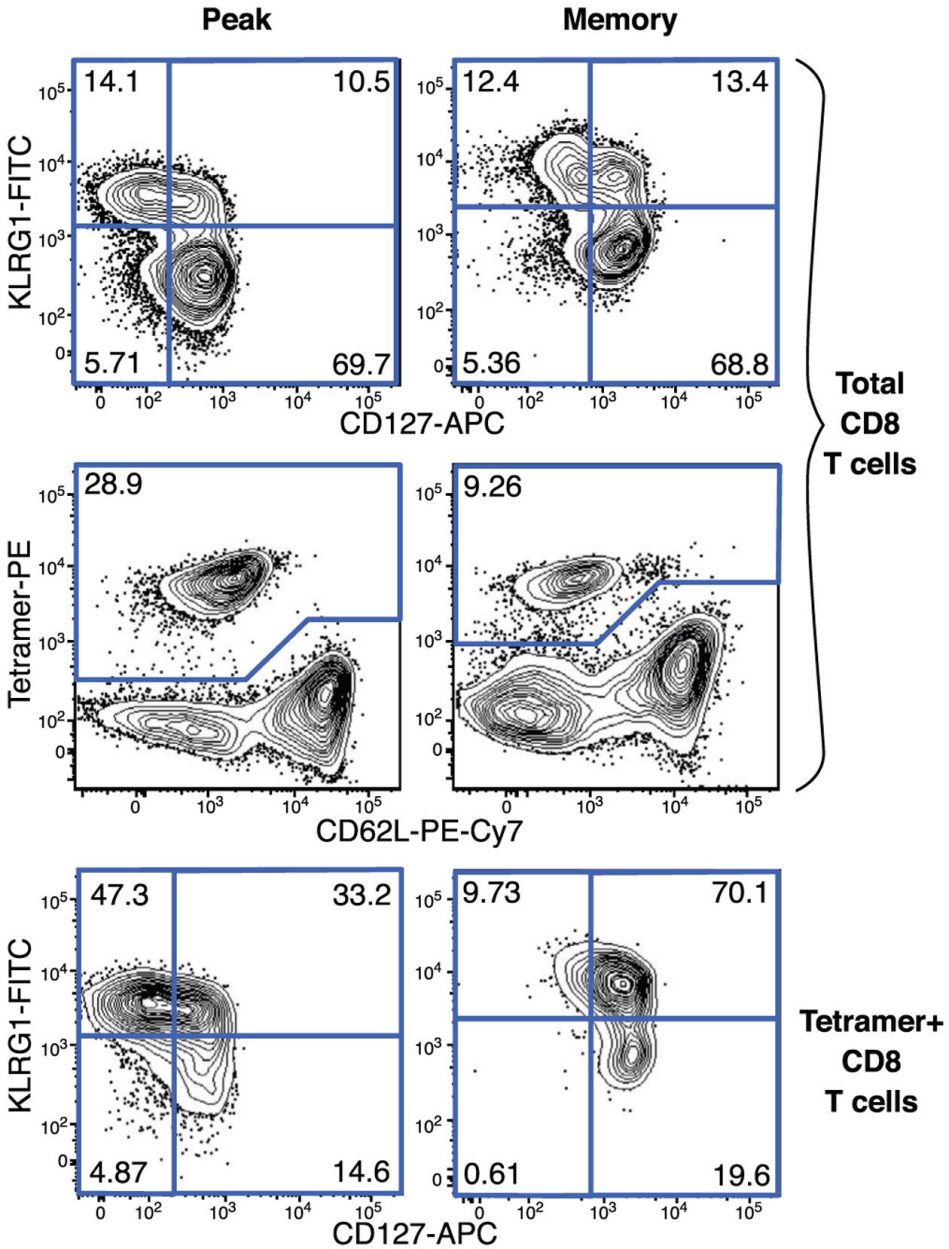
Delineating murine SLEC and MPEC populations. The expression of KLRG1 and CD127 can be used to differentiate SLECs (KLRG1^+^CD127^−^) from MPECs (KLRG1^−^CD127^+^). Plots are gated on CD8α^+^ T cells as in Figure 12 (total CD8 T cells, top two rows) and additionally on tetramer+ cells as in Figure 17 (bottom row). Cells are derived from peripheral blood at the peak (day 27, left) or memory timepoint (day 230, right) post-vaccination with recombinant adenoviral vector (serotype 5) expressing SIV-Gag as a target Ag.

The differentiation state of CD8 T cells is defined by CD44 and CD62L expression (Figure 12, 17, and Table 10). CD8 Tn cells are CD44^lo^CD62L^hi^. After infection or immunization, Ag-activated CD8 T cells upregulate expression of CD44 and lose CD62L during differentiation into CD8 Teff cells (CD44^hi^CD62L^lo^; Figure 18 and Table 10). The expression of additional surface markers during activation and expansion can be indicative of cellular fate in developing CD8 Teff cells. Two such markers are CD127, which is the IL-7 receptor α chain that promotes T cell survival in the periphery, and KLRG1, which is upregulated with strong or sustained Ag encounter and is regarded as a marker of terminal differentiation (Figure 18 and Table 10). Ag-specific CD8 T cells derived from the effector phase of a response can express various combinations of CD127 and KLRG1, which define either more short-lived effector cells (SLECs; CD127^−^KLRG1^+^), which are lost during the contraction phase of the immune response, or memory precursor effector cells (MPECs; CD127^+^KLRG1^−^), which are more likely to persist and contribute to memory populations [224, 225]. These differentiation stages of effector T cells however retain a certain flexibility and additional molecules such as CX3CR1, CXCR3, and transcription factors like TCF1 and TOX can be used to distinguish between populations of effector T cells with terminal effector differentiation and memory potential in acute and chronic infection as recently reviewed [226].

After resolution of infection, the CD8 Teff cell population contracts and memory populations begin to form. Similar to CD4 T cells, CD8 Tmem cells are often defined as Tcm cells (CD44^hi^CD62L^hi^, although this definition also includes virtual memory (Tvm) cells (see section III.10 Immune senescence (aging) in murine T cells and Table 19) and Tem cells (CD44^hi^CD62L^lo^), as well as tissue resident memory cells (Trm; CD44^hi^CD62L^lo^CD69^hi^; see section III.6 Murine tissue resident memory T cells.; Figure 17 and Table 10). Additionally, the differential expression of the fractalkine receptor CX3CR1 can been used to identify CX3CR1^int^ peripheral memory T cells (Tpm), which have direct access to peripheral tissues for surveillance [213].

##### 4.2.2 CD8 T cells: Transcription factors, effector functions, and Ag specificity.

The differentiation of CD8 T cells from Tn into Teff, Tcm, Tem, and Trm cells is co-ordinated by a network of transcription factors. Tn cells exhibit high expression of Bach2, which maintains naivety and multipotency [227]. After activation, some transcription factors favor Teff cell differentiation, such as Tbet, Id2, Blimp1, while others favor Tcm or Tem cell differentiation, such as Eomes, Bcl6, and Id3 (Table 10). Eomes in particular has been correlated with Tcm cell development [228] but it is also crucial in Tvm cell development [229]. Additionally, Blimp and Hobit (homolog of Blimp1 in T cells), mediate Trm generation [230]. To assess transcription factors by flow cytometry, intranuclear staining is used (See ChVI Section 1.2.3 CD4 T cells: transcription factors in ref. [21]).

During activation, CD8 Tn cells start to express surface molecules and produce and secrete proteins that are necessary for their effector function. Directed killing of infected or malignant cells is the main effector function of activated CD8 Teff cells. This cytotoxicity is typically mediated by secretion of cytokines, such as IFN-γ and TNF-α, the release of cytotoxic granules containing granzymes and perforin, and/or surface expression of FasL, which can induce apoptosis of Fas expressing cells (Table 10). Similar to CD4 T cells, different subsets of CD8 cytotoxic T (Tc) cells have been described, including Tc1, Tc2, Tc9, and Tc17 cells [231], and a subset of CD8 T cells can mediate help via CD40L [232]. Here, we provide an overview about the different methods that can be used for the assessment of effector functions and Ag-specificity of murine CD8 T cells. For more details, please consult the specific sections on T cell functions.

To assess cytokine production quantitatively and qualitatively, intracellular cytokine staining is commonly used. Like CD4 T cells, cytokine production in CD8 T cells is generally analyzed after *in vitro* restimulation, either polyclonally using PMA/Ionomycin or anti-CD3/28, or in an Ag-specific manner using protein (i.e., purified protein, pathogen lysate, or live pathogen) or peptide (see Chapter V Section 14 Intracellular parameters and Section 17.5 Functional Read-outs in ref. [21]). CD8 T cells recognize epitopes presented on MHCI, which mainly presents intracellular peptides. Accordingly, short peptides are very efficiently loaded onto MHCI (and II) and restimulation with peptides that contain known epitopes is therefore an effective way to induce and assess CD8 T cell responses (see section IV.1 Antigen-specific T cell cytometry: Functional read-outs and IV.2 Measuring Ag-specific CD8 T cell responses). Additionally, cells directly infected with bacteria/virus or cell lines expressing MHCI-peptide conjugates, such as SAMBOK (MEC.B7.SigOVA) [233] or RMA-S, can be used to stimulate CD8 T cells, as these cells exhibit efficient presentation of peptide on MHCI.

During stimulation, cells will start to express cytokines and other effector molecules. To drive the accumulation of these molecules within the cell and increase the detection of secreted effector molecules, protein transport inhibitors like brefeldin A (BrefA) or monensin can be included during T cell activation (see section IV.1 Antigen-specific T cell cytometry: Functional read-outs). These protein transport inhibitors are toxic; thus, it is optimal to limit the time of cell exposure. Typically, 4-6 h are used to accumulate cytokines like IFN-γ, IL-2, and TNF-α. In addition, BrefA or monensin can be administered to mice during an active immune response, with mice euthanized shortly after administration and immediate analysis of cytokine production directly *ex vivo* [234]. The advantage of this approach is that it allows measurement of cytokine production with *in situ* Ag presentation, which is more relevant to understanding immune priming in the lymph node and site of infection.

Cytotoxic potential can be assessed directly *ex vivo* by intracellular staining for cytotoxic proteins such as granzyme B and perforin. CD8 Teff and some Tmem cells contain vesicles of preformed cytotoxic granules, including granzymes and perforin, that are detected via intracellular staining directly *ex vivo* without the need for stimulation. Cytotoxic capacity can be directly assessed using *in vitro* or *in vivo* killing assays using target cells (see section IV.6 Cytotoxicity). Finally, degranulation capacity can also be assessed by detection of lysosomal markers, such as CD107a and -b, which become transiently accessible at the cell surface before being recycled (see section IV.6 Cytotoxicity).

To identify, analyze, and track Ag-specific CD8 T cells in mice, a number of methods previously described in the section on murine CD4 T cells can be used (see section III.2.2.2 Conventional αβ CD4 T cells: transcription factors, effector functions, and Ag-specificity). Briefly, Ag-specific CD8 T cells can be identified directly *ex vivo* using MHCI tetramers/multimers (see section IV.3 Antigen-specific T cell cytometry: MHC multimers). CD8 Teff cells can be restimulated with cognate Ag and proliferation or cytokine production can be used to indirectly identify Ag-specific CD8 T cells (see section VI.5 Adoptive T cell transfers as a read-out for Ag-specific immune responses in mice). Ag-specific CD8 T cell responses can also be tracked using transfer of congenically marked or fluorescently labeled TCR transgenic CD8 T cells from mouse strains such as OT-I, p14, and gBT-I, and subsequent challenge with their cognate Ag (see section IV.5 Adoptive T Cell Transfers as a readout for Ag-specific immune responses). Additionally, during an ongoing immune response, activation markers such as CD11a and CD49d [235], as well as markers of proliferation (BrdU or Ki67) (see section IV.5 Adoptive T cell transfers as a read-out for Ag-specific immune responses in mice) can be used to directly identify Ag-experienced CD8 T cells immediately *ex vivo.*

#### 4.3 Step-by-step sample preparation

##### 4.3.1 Cell isolation.

- Draw blood (e.g., using Microvette), sacrifice animal, and isolate tissues.
- Optional for tissue:
  - Generate single cell suspension by gently disrupting the tissue and filtering the cell suspension through 70μm cell strainer or equivalent.
  - Tissue digestion can be necessary (see section III.6 Murine tissue resident memory T cells and section III.12 Murine Foxp3^+^ regulatory T cells).
  - Gradient centrifugation can be necessary to purify immune cell fraction (see section III.6 Murine tissue resident memory T cells and section III.12 Murine Foxp3^+^ regulatory T cells).
- Optional for blood and some highly vascularised tissues:
  - Red blood cell lysis can be performed using ACK lysing buffer or equivalent.
- Resuspend cells in FACS buffer and count.

##### 4.3.2 Staining.

- Transfer 1–5 × 10^6^ cells per sample to a 96 well V-bottom plate
- Pellet cells at 500 × *g* for 5 min at 4°C and remove supernatant.
- Resuspend cells in 50 μl stain Ab mix (in FACS Buffer).
- Incubate at 4°C for 15-30 min.
- Wash with 150 μl of FACS Buffer, centrifuge for 5 min at 500 × *g* at 4°C and remove supernatant.
- Resuspend in FACS buffer for analysis on a flow cytometer.

#### 4.4 Materials

- Murine blood and tissue (stored in PBS)
- Microvette^®^ (Kent Scientific corporation (EDTA or Heparin)) or similar blood collection tubes
- ACK lysing buffer (155 mM NH_4_Cl, 10 mM KHCO_3_, 0.1 mM EDTA, pH 7,4)
- FACS buffer: PBS with 2% FCS
- Surface stain mix (in PBS with 2% FCS):
  - Anti-murine CD8α BUV395 (BD, cat # 563786, 53-6.7, 1:200)
  - Tetramer (D^b^ GP33 PE, R. Arens, LUMC)
  - Anti-murine CD44 BUV737 (BD, cat # 612799, IM7, 1:400)
  - Anti-murine CD62L BV510 (Biolegend, cat # 104441, MEL-14, 1:400)
  - Anti-murine CD69 PeCy7 (eBioscience, cat # 25-0691-82, H1.2F3, 1:200)
  - LIVE/DEAD^™^ Fixable Near-IR Dead Cell Stain Kit (ThermoFisher, cat # L10119, 1:1000)

#### 4.5 Data analysis

After gating on lymphocytes in FSC versus SSC, single cells and live CD3^+^ cells, CD4^−^ CD8^+^ T cells are selected (see Figure 12). In naïve mice, naïve (CD44^lo^CD62L^hi^) as well as memory-phenotype (CD44^hi^) populations can be identified. In infected mice expanded populations of tetramer^+^ virus-specific CD8 T cells can be identified that express high levels on CD44 (Figure 15). Within the tetramer^+^ CD44^+^ population, CD62L^+^ Tcm, CD69^+^ Trm, and double negative Tem cells are identified. Additional markers (Figure 18) and functional assays can be combined to further define important properties of CD8 T cells.

#### 4.6 Pitfalls

In response to cytokine signals, naïve T cells can acquire CD44 expression and differentiate into Tvm cells (see section III.10 Immune senescence (aging) in murine T cells). While Tvm cells are Ag-naïve, they are functionally distinct from Tn and Tcm cells. CD49d can be used to discriminate Tvm and Tcm in conditions where no Ag-specific T cells are identified, especially during aging when Tvm cells become more dominant (see section III.10 Immune senescence (aging) in murine T cells).

#### 4.7 Top tricks

- Some molecules, such as CD62L, can be lost from the surface due to proteolytic shedding, which is accelerated after activation and tissue processing [236, 237] – working fast and cold is recommended
- Some tetramers might benefit from performing the staining at room temperature or prolonging the incubation time for more efficient binding.
- Centrifugation of the Ab and/or tetramer mix before addition to the cells (e.g., 1-min full speed) can pellet aggregates and improves staining by removing these highly fluorescent particles.

#### 4.8 Summary of the phenotype

This is detailed in Table 10.

#### 4.9 Key information human versus murine

- Markers used to identify human and murine cells can differ. Always check that the marker is appropriate for the species you are analyzing. For example, while CD44 and CD62L (or CCR7) are used to identify CD8 Tn versus Tcm versus Tem cells in mice, human analyses use CD45RA in combination with CCR7 or CD27 and have an additional population known as Tem cell that re-express CD45RA (Temra cells).

### 5 Human tissue resident memory T cells

#### 5.1 Overview

To date, the vast majority of human T cell knowledge has been inferred from studies of peripheral blood. Tissue resident memory CD4^+^ and CD8^+^ T cells (Trm) represent recently identified populations that persist in various tissues without circulating. Animal studies show that they are important for rapid and enhanced local and systemic immune responses. Methods of isolation and identification CD4^+^ and CD8^+^ Trm from human tissues by flow cytometry have been developed and verified by multiple research groups and are described below.

#### 5.2 Introduction

In 1963, McGregor and Gowans made a revolutionary discovery that secondary immune reactions can be independent on circulating lymphocytes, in contrast to primary immune reactions [238]. Until the last decade or so, significant populations of non-circulating CD4^+^ and CD8^+^ Trm have been identified for a variety of lymphoid and non-lymphoid tissues. They have been defined as independent populations by tissue retention in parabiosis experiments [239, 240], by distinguishing transcriptional profiles [230, 241-245], by exclusive Ag receptor repertoires and specificities [243, 244, 246], and by exclusive persistence in transplanted tissues [247, 248]. These Trm are specially programmed to persist in their host tissues [249, 250]. While it is generally accepted that Trm provide enhanced effector function at the site of infection, recent research suggests that upon antigenic challenges Trm can also be mobilized to re-join the circulation and contribute to secondary systemic immune reactions [251, 252]. Both CD8^+^ and CD4^+^ Trm have been identified in the human skin, lung, intestines, salivary glands, bone marrow, brain, spleen, tonsils, and lymph nodes [241, 244, 246, 253-259]. However, our understanding of the human Trm is still in its infancy.

The most widely used marker of Trm is CD69. CD69 inhibits the function of S1PR1, which is required for S1P-mediated egress into the blood [260-262]. Thus, (CD69^+^) Trm are not attracted to the high SIP gradient in the blood and lymph, resulting in their tissue retention [263]. It should be noted that steady state Trm may be heterogeneous with regards to CD69 expression. Through parabiosis experiments, it has been shown that both CD69^+^ and CD69^−^ memory T cells of non-lymphoid tissues, like kidney and liver, are tissue resident [239]. However, markers categorizing Trm within CD69^−^ T cells in human tissues are yet to be confirmed. Furthermore, expression of CD69 is induced upon activation of T lymphocytes, which should be kept in mind especially when performing functional assays. Trm do not express other putative activation markers, such as CD25, CD137, CD38, or HLA-DR [244, 253, 258, 264]. Trm typically lack the expression of lymph node homing molecule CCR7 and tissue egress molecule S1PR1. Human Trm have a memory phenotype of being CD45RA^−^CD45RO^+^ and CCR7^−^CD27^+/−^CD28^+/−^ in CD4^+^ and CD8^+^ T cells. A subset of Trm are also confined to the CCR7^−^CD45RA^+^ T_EMRA_ CD8^+^ T cells from tonsils, spleen, and bone marrow [244, 265].

An additional identifier of Trm at mucosal sites is CD103 (α subunit of αEβ7 integrin), which helps Trm dock to E-cadherin expressing epithelial cells [266]. Trm that co-express CD69 and CD103 are mainly found in the skin, lung, salivary glands, brain, and intestines [241, 256-258]. Trm from other tissues, such as the liver and bone marrow, express CD69 but not CD103 [244]. Trm also express chemokine receptors CXCR6, CXCR3, CCR5, and integrin CD49a (α subunit of α1β1 integrin) in various tissues [241]. CD69^+^ Trm lack the expression of CX3CR1 [241], which is mainly expressed by effector T cells.

Trm rapidly produce cytokines upon activation and are polyfunctional [241, 244, 257, 267], which can be quantified by in vitro stimulation in the presence of brefeldin A and staining for cytokines intracellularly. In the skin, CD49a identifies CD8^+^ Trm poised for cytotoxic function [268]. This rapid effector function needs to be tightly controlled in order to protect the tissues where Trm reside. Inhibitory receptors PD-1, CTLA-4, and 2B4 are expressed by Trm [241, 256, 257], which may control the effector function of these Trm.

In recent years, Trm have also taken the spotlight in cancer immunotherapy. The use of tumor-infiltrating lymphocytes (TILs) and Ab immunotherapy was one of the big breakthroughs of cancer immunotherapy. Further research revealed that the presence of CD103^+^CD8^+^ TILs is a positive prognostic marker in several cancers [269-272]. A subset of TILs in tumors of NSCLC shares the phenotype of Trm in adjacent lung tissue (expression of CD103, CD69, CXCR6, CD49a) [272, 273]. A proportion of PD-1^++^ TILs with a Trm phenotype also express CXCL13, the ligand of CXCR5 [273-275]. Furthermore, tumors consist of tumor-specific TILs as well as tumor nonspecific T cells so-called bystander T cells. While tumor-specific TILs express high levels of CD39, the bystander T cells lack CD39 expression [276].

Further characterization of human Trm may be performed on a global level by transcriptomes and epigenomes of individual cells, and their Ag receptor repertoires. Cell isolation and data validation can be performed by multicolor flow cytometry and functional assays, respectively.

#### 5.3 Step-by-step sample preparation

The following protocol was designed for isolating mononuclear cells from various tissues, including intestine, lungs, tumor tissue, skin, and bone marrow, as described previously [244, 253, 267, 277, 278].

Step I. Store or ship issue samples at 2–8°C in a tissue preservation solution for less than 48 h

- Intestine, lung, tumor tissue, and skin samples:
  1a. Rinse tissue with cold PBS and remove fat, mucosa, or hair
  2a. Cut tissue with sterile scissors into small (2-4 mm) pieces and incubated for 1–2 h (intestines, lung and tumor tissue) or 6–12 h (skin) at 37°C in digestion medium (50 U/mL DNase I; 0.8 mg/mL collagenase IV) while gently shaking/rolling
  3a. (Optional) Dissociate the digested tissue fragments using a gentleMACS^®^ Tissue Dissociator (Miltenyi) using an appropriate program. This procedure can be repeated to improve the outcome.
  4a. Pass the digested tissue through a sterile sieve to separate the dispersed cells and tissue fragments from the larger pieces using the upside of a 25 mL sterile syringe to achieve a single cell suspension. Further disaggregation can be achieved by incubating these pieces in fresh digestion medium for shorter than the initial digestion period (e.g., 0.5–2 h, depending on the sample tissue) and repeat the separation step.
  5a. After centrifugation, incubate the samples with 50 μg/mL DNase I for 15 min in a 37°C water bath
  6a. To isolate mononuclear cells from the cell suspension, use standard Percoll density gradient technique (40% and 70% solutions)
  7a. Collect the interphase through a 70 or 100 μM cell strainer
- Bone marrow biopsy sample:
  1b. Wash sample with pre-warmed (37°C) PBS, cut bone tissue, if any, into small pieces with a bone shear (Solingen; HSM 023-12)
  2b. Load cell suspension over a 70 or 100 μM cell strainer
  3b. Isolate mononuclear cells by density gradient sedimentation using Ficoll-Hypaque

Step II. Isolated mononuclear cells can be used directly for experimentation or cryopreserved in liquid nitrogen until further analysis

Step III. For cryopreservation, resuspend cells (5–20 × 10^6^) in 500 μL of cold 100% FCS^†^, and then add drop-wise, with continual swirling the cryovial, 500 μL of 20% DMSO, using the standard procedure as described previously (See Chapter VI Section 1.11.7.1 Human CD4 and CD8 T cells/Step-by-step sample preparation/Freezing PBMC in ref. [22]).

##### 5.3.1 Flow cytometry staining of human Trm.

1. Prepare surface staining mix in flow cytometry buffer
2. For staining use 1 × 10^5^−2 × 10^6^ cells
3. Add cells to a 96-well V-bottom plate and centrifuge (350 × *g*, 5 min, 4°C)
4. Remove the supernatant
5. Resuspend cells in 50 μL staining mix per well and incubate for 15–20 min on ice
6. Wash cells by adding 150 μL flow cytometry buffer and centrifuge (350 × *g*, 5 min, 4°C)
7. Remove supernatant
8. For only surface staining, resuspend in 80–120 μL flow cytometry buffer (depending on the amount of cells) and measure
9. Cells can also be fixed with a mild fixative (2% PFA)
10. For intracellular stainings, fix cells by resuspending the cell pellet in 50 μL of fixation solution
11. Incubate for 10 min at the room temperature
12. In the meantime, prepare intracellular staining mix in 1× permeabilization buffer
13. Wash cells by adding 150 μL flow cytometry buffer and centrifuge (550 × *g*, 5 min, 4°C)
14. Resuspend cells in 50 μL intracellular staining mix per well Incubate for 30 min at room temperature
15. Wash by adding 150 μL 1× permeabilization buffer and centrifuge (550 × *g*, 5 min, 4°C)
16. Remove supernatant and resuspend cells in 80–120 μL of flow cytometry buffer (depending on the amount of cells) and measure

#### 5.4 Materials

- Tissue preservation solutions: MACS Tissue Storage Solution (Miltenyi Biotec) or CUSTODIOL HTK solution (Köhler Chemie, Germany).
- Medium: RPMI with 20 mM Hepes, 10% FCS or 5% human AB serum, 1% pen/strep, 1% l-glutamine
- Digestion mix: medium, 50 U/mL DNase type I (Sigma–Aldrich), 0.8 mg/mL collagenase IV (Worthington)
- DNase (Sigma–Aldrich) (dilute in medium)
- Percoll, Ficoll-Hypaque (both GE Healthcare)
- Cell strainer: 70 or 100 μM (Corning; BD Falcon cell strainer or MACS SmartStrainer)
- Flow cytometry buffer: 0.5% FCS or 0.5% BSA PBS
- PFA: paraformaldehyde
- Freeze media: equal volumes of 100% FCS and 20% DMSO (in 100% FCS)
- Fixation solution from Foxp3 Transcription Factor Staining kit (Thermofisher)
- 1× Permeabilization buffer from Foxp3 Transcription Factor Staining kit (Thermofisher)
- Flow cytometer: LSR II, LSRFortessa or FACSymphony (BD)
- Table 11 is a list of Abs that can be used to identify and characterize human Trm. The list is extendable. In addition, Abs recognizing the same epitope that have been applied by other research groups are also listed.
- Live/dead fixable dead cell dyes (Thermofisher)/Zombie fixable viability kits (Biolegend) should be used in the surface staining mix to distinguish live cells

#### 5.5 Data analysis

**Figure 19.**
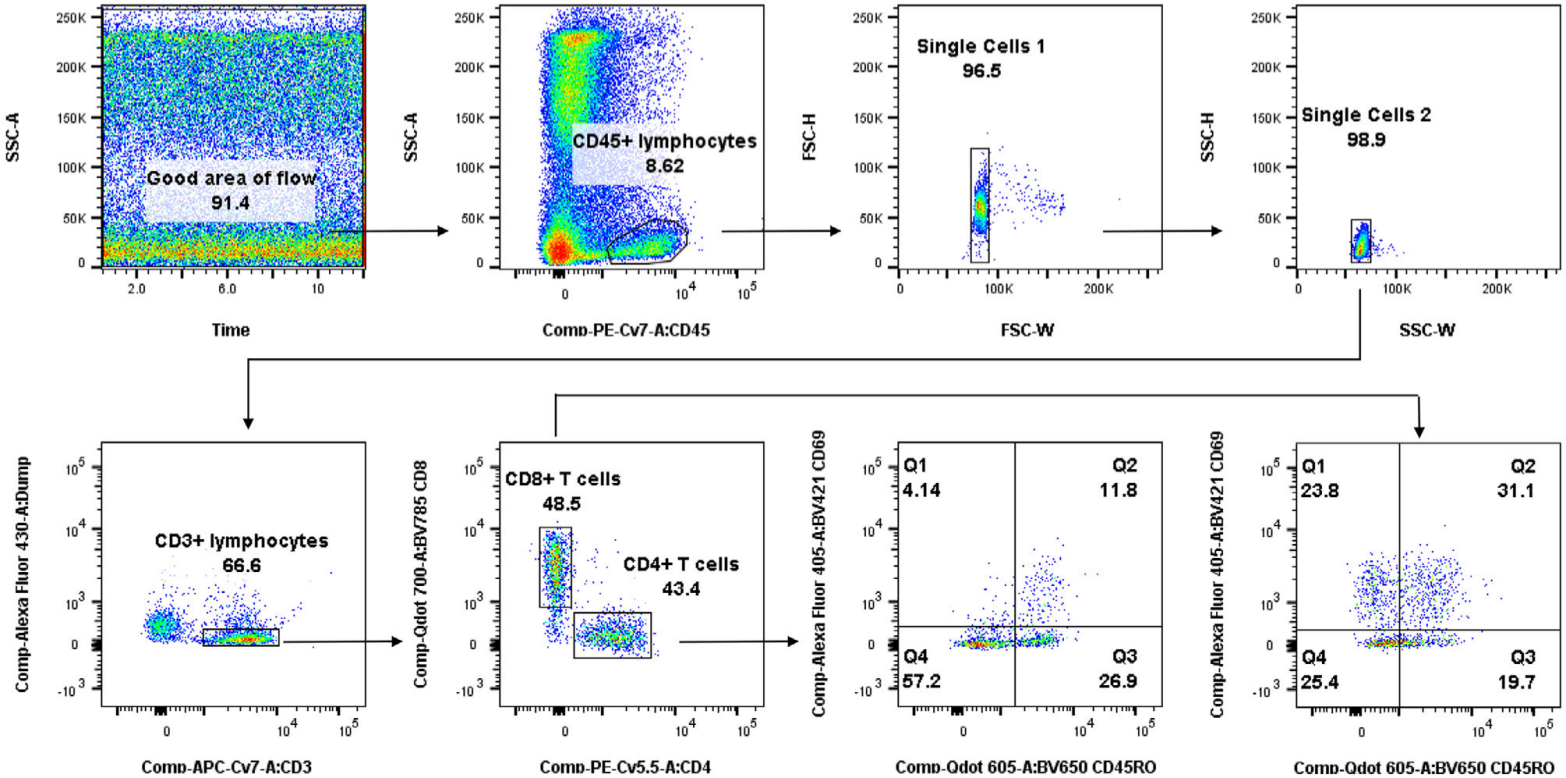
Gating strategy for analyzing CD4^+^ and CD8^+^ human Trm from bone marrow. Similar gating strategies also apply for Trm from other tissues. The Time gate is used in relation to a scatter parameter like SSC-A to identify and remove potential bubbles, clogs, or air. SSC and FSC are used to gate on lymphocytes expressing CD45, followed by gating out doublets using SSC and FSC (both width vs height). Live/dead marker is used to exclude dead cells and CD3 to gate on T cells. CD4 and CD8 are used to gate on CD4^+^ and CD8^+^ T cells. CD69^+^ Trm can be further gated in relation to CD45RO.

To analyze flow cytometry data, FACS Diva (BD) or FlowJo (Tree Star) software should be used. It is important to apply a robust gating strategy. In general, the time, that is, the area(s) of good flow, (CD45^+^) lymphocytes, and single cells should be gated, after which the compensations should be checked. Single stainings should be used as compensation controls. Once the compensations have been adjusted, viable CD3^+^ T cells are gated by excluding the dump (also called exclusion) channel of dead cells using a live/dead fixable dye and lineage negative cells that are stained with the same or similar fluorochrome. CD3^+^ T cells are further separated into CD4^+^ and CD8^+^ T cells. The common marker of Trm is CD69 (as shown in Figure 19), but many more markers have been identified, as described above and listed in the summary phenotype tables (see Tables 12 and 13 in the Summary Phenotype Tables section).

#### 5.6 Pitfalls

Isolation of T cells from human tissues often requires enzymatic digestion with collagenases, which may affect the expression of certain proteins, particularly chemokine receptors, such as CCR6 [279], due to their large structure and multiple transmembrane domains. This should be kept in consideration when staining tissue cells that have undergone enzymatic digestion. The preferred collagenase used by multiple research groups is collagenase IV [253, 256, 257, 267]. Furthermore, cryopreservation can also affect expression of proteins, such as the l-selectin/CD62L. While various markers of Trm are shared between tissues, some markers and ratios of certain populations can vary per tissue compartment. The amount of unconventional T cells, such as TCR-γδ, MAIT cells, and NK T cells [280], should also be determined, which also varies per tissue. If there are significant amounts of these unconventional T cells, a dump channel should be applied, in addition to CD14^+^ (monocytes) and CD19^+^ (B-lineage) cells.

#### 5.7 Top tricks

1. Careful panel design is important, especially with more than 10 color flow
  - The major problem is the scatter caused by compensation of some fluorochromes, not per se the amount needed to compensate
2. Some examples of compensation issues
  - To use BUV805, BV737, and BV785 in the same panel, BD FACSymphony is required
  - BUV661 spills into APC, so BUV661 signal should not be too bright and that of APC should be relatively bright
  - BUV737 spills into BUV805, so using markers that are clearly distinct from each other such as CD4 and CD8 is recommended
  - When used in a panel with BV605 and BV650 on the violet laser, PMT voltage balancing will be required between these neighboring channels to minimize spillover values
  - Properly titrating Abs and not using Abs at too high concentrations can significantly help
3. Some tissues suffer from autofluorescence
  - It can help to keep a Brilliant violet channel empty or sometimes the auto fluorescence can be gated out when two fluorochromes without double positive signal is expected are plotted against each other
4. Isotype controls or FMOs (fluorescence minus one) should be used if there is only a shift for a population instead of a clear staining/bimodal populations
5. To avoid aggregates caused by some fluorochromes, the Ab mixes can be centrifuged at 1400 rpm for 10 min. Afterward, the aggregates should be pelleted and the Ab mix should not be pipetted from the bottom of the tube or resuspended again.
6. A dump channel may be necessary for non-T cells such as CD19^+^ and CD14^+^ cells, and unconventional T cells such as TCR-γδ, MAIT cells, and NK T cells, which can be for example put in the same channel as the live/dead
7. To isolate or analyze subpopulations of Trm cells, a pre-enrichment of CD3^+^ T cells, or depletion of CD15^+^ granulocytes or unconventional T cells may help

#### 5.8 Clinical relevance statement

The gating strategy shown in this section (Figure 19) is applicable for analysis of about 1 million mononuclear cells isolated from the bone marrow from patients undergoing hip replacement surgery. The key conclusion from this analysis is that about 30% and 60% of CD45RO^+^ memory CD4^+^ and CD8^+^ T cells of the human bone marrow express the resident marker CD69 [244].

Additionally, CD69-expressing cells can also be found in CD45RO^−^ cells, especially in CD8^+^ T cells, which presumably consist of a subset of effector memory T cells re-expresses CD45RA, also termed T_EMRA_ [244, 265]. To characterize these cells, CD45RA and CCR7 Abs should be used in addition to or instead of CD45RO Ab in a staining panel.

To better understand human Trm cells, it is helpful to directly compare them with paired blood T cells at the same sample collection time [241, 244, 253, 267].

#### 5.9 Summary phenotype tables

This is shown in Table 12 and 13.

### 6 Murine tissue resident memory T cells

#### 6.1 Overview

tissue resident memory T cells (Trm) cells are non-circulating T cells that form the first line of defence against reinfection in tissues [281-283]. They are enriched in barrier tissues, such as the lung, skin, and intestine, but they are also present in internal organs, such as brain, liver, bone marrow, white adipose tissue, and lymphoid organs. The tissue residency phenotype was initially described for CD8 T cells, but analogous tissue residency programs for CD4 T cells and other lymphocyte populations are currently being defined [284]. This section focuses on the flow cytometric analysis of conventional Trm cells in peripheral tissues. More detailed information for the analysis of intra epithelial lymphocytes (see section III.10 Murine IEL), MAIT cells (see section III.20 Murine MAIT), as well as regulatory T cells in different tissues (see section III.12 Murine FoxP3^+^ regulatory T cells) can be found in the respective chapters.

#### 6.2 Introduction - Methods to identify and analyze tissue resident T cells

Most Trm cells are located within a dense network of parenchymal cells and extracellular matrix. This cellular network needs to be dissociated to analyze the lymphocytes in a single-cell suspension by flow cytometry. It is important to note that the isolation procedure of murine T cells from tissues can have a significant impact on cell viability and detected phenotype. Enzymes like collagenase and dispase, which are used to enzymatically dissociate tissue, can cleave off lineage-defining markers such as CD4 and CD8 [285]. Additionally, tissue dissociation not only causes sheer stress and destruction, but signals released during the dissociation of tissue can influence the phenotype, function and viability of CD4 and CD8 T cells. For example, activation of the danger receptor P2RX7 by extracellular NAD or ATP, released by dying cells during processing, can lead to shedding of surface molecules such as CD62L and CD27, the masking of CD8β and the induction of cell death in susceptible cells like Treg, NKT, and Trm cells [286-288]. This is especially important when T cells are used in functional assays. As a result, the method of tissue dissociation should be carefully chosen, and viability of cell preparations used in flow cytometry should always be assessed.

**Figure 20.**
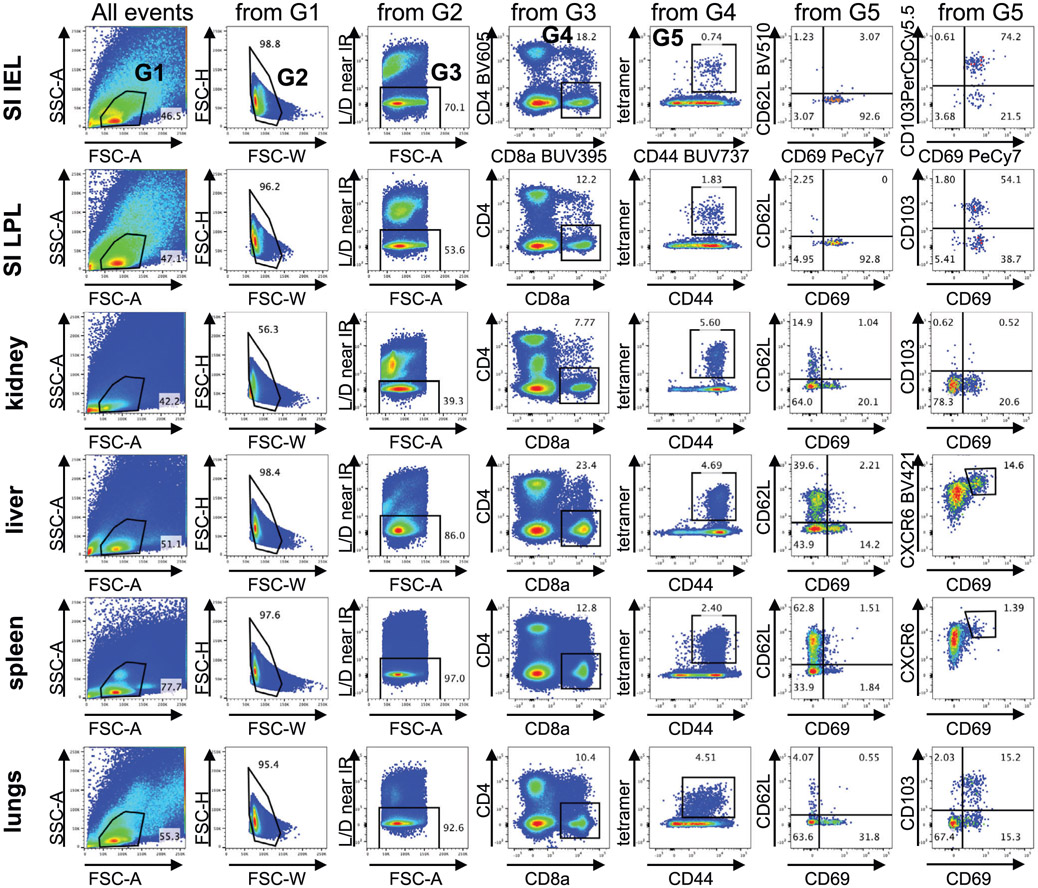
Identifying murine Trm cells from small intestine, liver, spleen, kidney, and lungs using surface markers. Sample gating tree for the identification of pathogen-specific CD8 Trm cells from the intraepithelial lymphocyte fraction (SI IEL) and lamina propria fraction (SI LPL) of the small intestine, liver, spleen, and kidney of LCMV-infected mice as well as lungs of Influenza-infected mouse >d30 post infection. LCMV-specific memory CD8 T cells can be identified by gating on lymphocytes according to FSC and SSC (G1), exclusion of doublets (G2) and dead cells (G3) and gating CD4-CD8α+ cells (G4), GP33-tetramer (LCMV) or NP366-tetramer (Influenza) positive CD44 high cells (G5). Trm can be identified by gating on CD69+/ CD62L− cells in contrast to CD69− circulating T cells. Additionally, CXCR6 is highly expressed on many Trm populations and CD103 is expressed on a subpopulation of epithelial Trm.

Once a single cell suspension is generated, conventional T cells are identified via gating on lymphocytes in scatter, exclusion of doublets, gating on live cells and CD3^+^ cells. TCR-β can be used instead of CD3, with the advantage of excluding γδ T cells from the analysis. Consecutively, CD4 and CD8 T cells can be identified (Figure 20, see section Murine CD4 and Murine CD8 T Cells). For the analysis of T cells specific for a certain epitope additional strategies, such as MHC class I or II tetramers can be used. By using this additional molecule to gate on the population of interest, CD3 or TCR-β could be skipped in interest of including additional phenotypic markers in the panel. To identify Trm cells within the T cell population in the tissue by flow cytometry different strategies can be used. Characteristic surface markers or transcription factors are a suitable tool to detect tissue resident populations.

**Figure 21.**
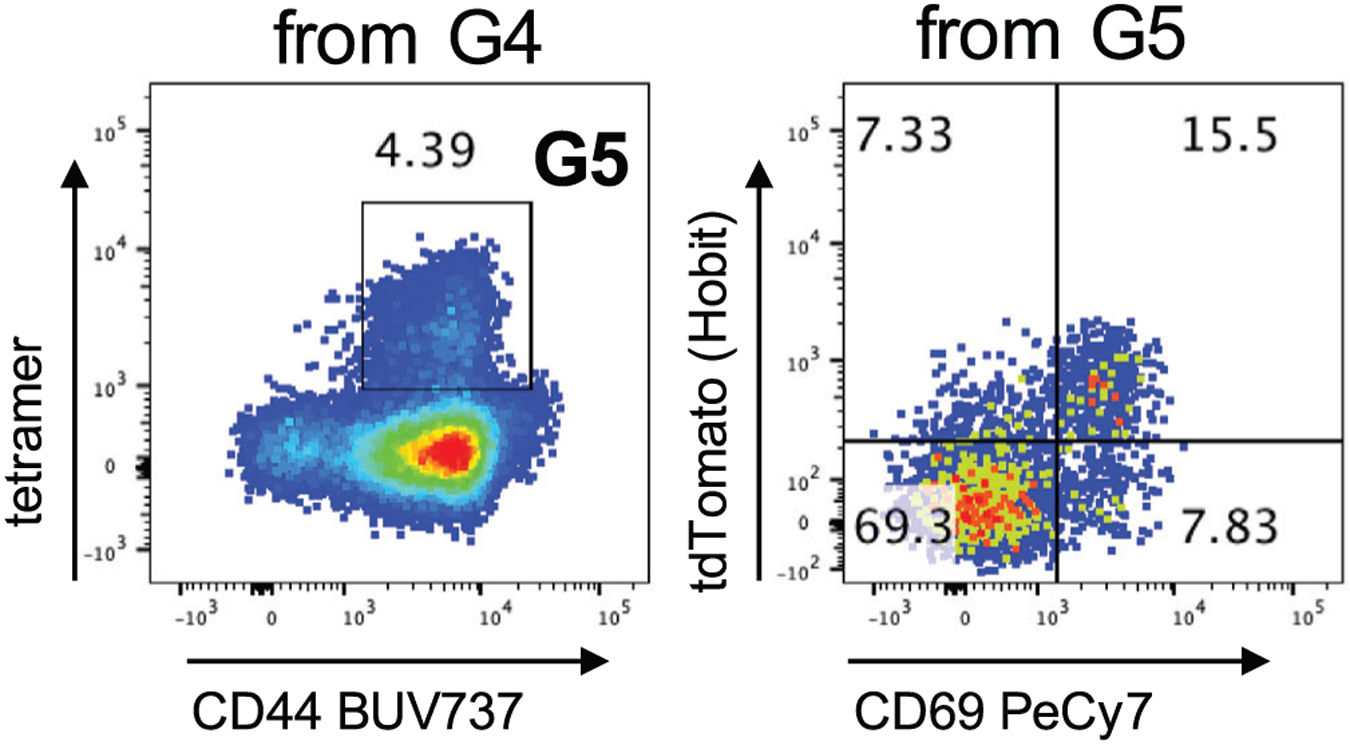
Identification of murine Trm cells in liver using Hobit reporter expression. LCMV-specific CD8 T cells were identified using tetramers as described above (G5) in liver preparations of LCMV-infected Hobit reporter mice (>d30 post infection). Coexpression of tdTomato, reporting the Trm master transcription factor Hobit, and CD69, was used to identify Trm cells.

The expression profile of T cells is determined by their location and signals of the local microenvironment determine. Accordingly, the physiologically quite diverse parenchyma of tissues can drive a tissue-specific phenotype of Trm [281]. CD69 is constitutively expressed by many Trm cells, it is functionally important for their residency and it is therefore a commonly used marker for Trm identification (Figure 20). Trm are usually identified as CD69^+^/CD62L^−^ to discriminate from CD69^−^/CD62L^+^ central memory cells and CD69^−^/CD62L^−^ effector memory cells (see section III.4 Murine conventional αβ CD8 T cells). While the majority of Trm cells throughout different tissues constitutively express CD69, parabiosis, and in vivo labeling experiments have highlighted that there are CD69^−^ cells that reside long-term in tissues [239]. Additionally, the chemokine receptor CXCR6 (Figure 20), Ig CD101, P2RX7, CXCR3, and CD49a, the α-chain of the α1β1 integrin (VLA-1), are also highly expressed on Trm cells in many tissues. CD8 Trm cells in epithelial and neuronal tissues can also express CD103 (Figure 20), which is the α-chain of the αEβ7 integrin [281]. CD103 is also expressed by a subset of CD8 Tn cells, which makes the use of CD44 or CD62L essential to discriminate between CD8 Trm and Tn cells. In addition to surface molecules, the expression of the transcription factor Hobit can be used to identify tissue resident T cells. Hobit is a master regulator of tissue residency [230] and selectively expressed by tissue resident immune cells within the CD8 T cell lineage. As currently no Abs are available to detect murine Hobit accurately by flow cytometry, Hobit reporter mice are the tool of choice to identify and manipulate Trm [251, 289] (Figure 21). See table 10 in Chapter 3 section III.4 - Murine Conventional αβ CD8 T cells with a summary of the phenotypes.

Additionally, *in vivo* labeling with Abs is an established protocol for Trm identification by providing information about the location of T cells. During in vivo labeling, fluorescently labeled Abs directed against CD4, CD8, or pan-T cell markers, such as CD90 and CD45, are injected intravenously shortly before tissue harvest [290]. This brief exposure labels leukocytes in circulation, but not extravasated leukocytes in the peripheral tissues, and therefore, can be used to distinguish between the labeled cells in the vasculature and unlabeled T cells located within the tissue. This approach can be particularly important when working with highly vascularized tissue such as the lung to enrich for Trm cells [290]. For other tissues, where Trm are located in very close contact with vasculature or in the sinusoids, such as the bone marrow or liver, in vivo labeling is less applicable to identify Trm [259, 290].

**Figure 22.**
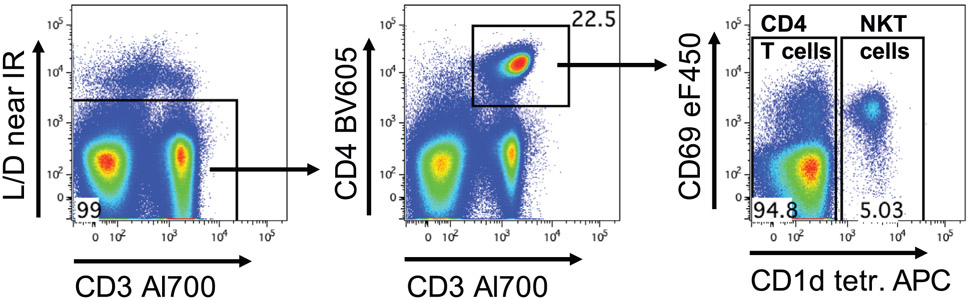
Identification of unconventional and conventional murine T cells. Unconventional and conventional murine T cells can have overlapping phenotypes. Splenocytes were gated on scatter parameters (see Figure 20), live cells, and CD3^+^/CD4^+^ T cells. Staining with CD1d PBS-57 tetramers (obtained through the NIH Tetramer Core Facility) was used to identify NKT cells that mainly express CD69.

In many peripheral tissues, unconventional innate-like T cells are increased and can even dominate (see section III.8 Murine Intestinal Intraepithelial Cells), which is important to consider when identifying conventional Trm cell populations. These unconventional T cells can have overlapping expression patterns with conventional TCR-αβ CD4 and CD8 T cells for basic surface markers. For example, in liver and spleen, the CD3^+^ CD4^+^ gate is comprised of both conventional TCR-αβ CD4 T cells and a population of NKT cells with a tissue resident phenotype (Figure 22). Similarly, in the small intestine, the CD3^+^ CD8α^+^ gate for intraepithelial lymphocytes (IELs) can include conventional TCR-αβ CD8αβ T cells and CD8αα IELs and γδ T cells. Therefore, careful selection of the surface markers is key for the correct identification of target cells, particularly across tissues.

#### 6.3 Step-by-step sample preparation for the analysis of Trm from the small intestine, liver, kidney, or lungs

##### 6.3.1 Isolation of intraepithelial lymphocytes from the small intestine.

- Remove small intestine and store in cold HBSS with 2% FCS^†^ (wash medium)
- Cut intestine into three pieces and store in HBSS with wash medium
- Clean each piece of intestine on a wet tissue soaked with wash medium from fat, remove peyers patches, cut longitudinally and remove faeces, keep intestine wet during the whole time
- Wash two times with 5 ml wash medium (e.g., 6-well plate)
- Cut into pieces of 1 cm and transfer to 10 ml wash buffer, vortex 10 s, pour over cell strainer, and collect tissue pieces, repeat 2×
- Transfer tissue pieces to 20 ml EDTA medium (HBSS, 10% FCS^†^, 5mM EDTA, 1mM DTT)
- Incubate 30 min at 37°C in water bath, vortexing every 10 min
- Filter over cell strainer and collect the flow-through containing intraepithelial lymphocytes
- Remaining tissue pieces can be used for the isolation of lamina propria lymphocytes

##### 6.3.2 Isolation of lymphocytes from the liver and spleen.

- Dissect organ out and store in cold FACS buffer (PBS with 0.5% FCS^†^)
- Remove gall bladder from liver and other connective tissue attached
- Mash tissue over cell strainer with FACS buffer using the plunger of a 3 ml syringe

##### 6.3.3 Isolation of immune cells from lungs, kidney and small intestine lamina propria.

- Dissect kidney and lungs out and store in cold wash medium or PBS; prep small intestine as described above to remove intraepithelial lymphocytes
- Cut tissues into pieces using scissors or scalpel in digestion buffer (3-5 ml, 1× digestion medium for kidney and lungs and 0.5× digestion medium for small intestine lamina propria; 1× digestion medium: 750 U/ml Collagenase Type I (Invitrogen), 0.31 mg/ml DNase I (Roche, from bovine pancreas, grade II) in RPMI,
- incubate 30 min at 37°C in water bath, vortex every 10 min
- mash over cell strainer with wash medium using the plunger of a 3 ml syringe

##### 6.3.4 Enrichment of lymphocytes by gradient centrifugation (all organs except spleen).

- Pellet cells (500 × *g*, 5 min, 4°C)
- Resuspend cells in 5 ml 40% isotonic percoll and overlay to 3 ml 60% isotonic percoll in a 15 ml falcon
- Centrifuge for 20 min at 800 × *g*, acceleration 5, deceleration 0 (no break)
- Remove top layer of fat and tissue cells
- Collect lymphocytes from the interphase
- Wash with FACS buffer

##### 6.3.5 Staining of lymphocytes.

- Transfer up to 4 × 10^6^ cells to FACS tubes or 96-well V-bottom plate
- Pellet cells (500 × *g*, 5 min, 4°C)
- Resuspend in 50 μl staining mix, incubate 30 min at 4°C
- Wash cells with FACS buffer and analyze by FACS

#### 6.4 Materials

- Wash Medium: HBSS/2% FCS^†^
- EDTA Medium: HBSS 10% FCS^†^ mix, 5 mM EDTA, 1 mM DTT
- FACS buffer: PBS/0.5% FCS^†^
- PBS
- 10× digestion mix: 7500 U/ml Collagenase Type I (Worthington, LS004196), 3.1 mg/ml DNase I (Roche, cat # 10104159001); freeze aliquots and dilute with RPMI supplemented 10% FCS^†^ to required concentration
- Percoll (GE, 17-0891-01, density 1,13g/ml, prepare isotonic 90% Percoll by mixing with 10× or 20× PBS, dilute with PBS to 60% and 40%)
- Surface stain mix (in PBS with 0,5% FCS):
  - Anti-murine CD8α BUV395 (BD, cat # 563786, 53-6.7, 1:200)
  - MHC class I tetramer (D^b^ GP33 PE, R. Arens, LUMC; D^b^ NP366 APC, Sanquin)
  - Anti-murine CD69 PeCy7 (eBioscience, cat # 25-0691-82, H1.2F3, 1:200)
  - Anti-murine CD62L BV510 (Biolegend, cat # 104441, MEL-14, 1:400)
  - Anti-murine CD103 PerCpCy5.5 (BD, cat # 563637, M290, 1:200)
  - LIVE/DEAD^™^ Fixable Near-IR Dead Cell Stain Kit (Thermo Fischer, cat # L10119, 1:1000)

#### 6.5 Pitfalls and Top tricks

- Know the cells you study: correct selection of markers for identification and exclusion of other cell populations is key to avoid misclassification.
- Sample processing can affect detection of your markers, cell viability, and the outcomes of functional assays.

#### 6.6 Summary of the phenotype

See Chapter 8 section VIII (Murine Conventional αβ CD8 T cells) for key information on human versus murine cells and the summary phenotype Table 10 noting the key differences between human and murine cells.

### 7 Human intestinal intraepithelial T cells

#### 7.1 Overview

Intestinal intraepithelial lymphocytes (iIELs) are a phenotypically heterogeneous population of T cells located amid epithelial cells of the GI tract, where they fulfill a critical role in preserving a healthy intestinal mucosa by protecting against pathogens and maintaining epithelial integrity.

According to their developmental pathways and recognition properties, there are two main subsets of iIELs: adaptively induced CD8αβ^+^ or CD4^+^ TCRαβ^+^ T cells, and naturally occurring TCRγδ^+^ T cells. The infiltration of iIELs is a histological hallmark of celiac disease, which is a chronic inflammatory disorder of the small intestine caused by gluten consumption that occurs in genetically susceptible individuals. Flow cytometry is a powerful technique for the study of immune cells and mechanisms in the intestinal mucosa, and one of its successful clinical applications has been the analysis of iIELs as a rapid and precise tool for the diagnosis and follow-up of celiac disease.

Here, we describe a protocol for the isolation and staining of iIELs from human duodenal biopsies, after routine endoscopy procedures. Besides the clinical use of iIELs phenotyping in the diagnosis of celiac disease, future applications include lymphocyte extracellular and intracellular staining, and cell sorting for ex vivo functional studies.

#### 7.2 Introduction

##### 7.2.1 Intraepithelial lymphocytes: Subsets and function.

Intestinal intraepithelial lymphocytes (iIELs) represent one of the largest populations of T cells in the body that reside among the epithelial cells of the GI tract, with an average ratio of 10-20 per 100 villus enterocytes in the human small intestine [291-293]. Its location at the interface between the lumen and the lamina propria allows this phenotypically heterogeneous cell population to fulfill a critical role in preserving a healthy intestinal mucosa by eliminating damaged or infected epithelial cells and by controlling the local inflammation, with differences in the distribution between the small and large intestine [292, 294]. iIELs-driven mechanisms are involved in host defense in a permanently threatened tissue by intestinal pathogens [295].

Intestinal IELs have been classified into two main subsets, according to their developmental pathways and recognition properties: adaptively induced iIELs, and naturally occurring IELs. It is worth mentioning that most of our knowledge of these cells comes from their murine counterparts. Induced iIELs develop from conventional CD8αβ^+^ or CD4^+^ TCRαβ^+^ T cells, and acquire the phenotype of effector T cells after the recognition of foreign Ags in the periphery. Natural IELs, which in humans mainly include TCRγδ^+^ T cells, are tissue resident cells that acquire the effector phenotype after the recognition of self ligands. There is a small remaining group of lymphocytes lacking in TCR, among them, innate lymphoid cells [293, 294, 296]. In the healthy human small intestine, CD8αβ^+^ TCRαβ^+^ T cells represent 80–90% of iIELs; <20% are TCRαβ^+^ CD4^+^; 5-15% TCRγδ^+^; while the murine counterpart TCRαβ^+^ CD8αα^+^ iIELs are absent [297, 298].

Both subsets of iIELs (induced and natural) express the integrin CD103, which interacts with E-cadherin on epithelial cells [299-301], and the activation marker CD69 [302, 303]. Moreover, most iIELs show a profile of cytotoxic effector cells characterized by intracellular granules containing granzyme B [297]. In healthy individuals, CD8αβ^+^ TCRαβ^+^ IELs express the inhibitory NK receptors CD94/NKG2A, with a low expression of activating receptor NKG2D [304, 305].

Most of the information available on human iIEL has been obtained from studies on the pathogenesis of celiac disease. Celiac disease is a chronic inflammatory disorder of the small intestine caused by gluten consumption that occurs in genetically susceptible individuals expressing the HLA-DQ2 or DQ8 haplotypes. Adaptive and innate immunity are necessary for the development of the enteropathy. The adaptive immune response depends on lamina propria CD4^+^ T cells that, upon gluten recognition in HLA-DQ2/DQ8 molecules, produce IFN-γ. This cytokine has multiple effects on the mucosal damage, and together with other signals (i.e., IL-12 and IL-15), potentiates the cytotoxic activity of iIELs and the destruction of epithelial cells. Under stress or inflammatory conditions, such as in celiac disease, iIELs upregulate the expression of activating NKG2D and CD94/NKG2C receptors, (which recognize MICA/B and HLA-E, respectively) while losing the expression of the inhibitory receptors CD94/NKG2A [302, 306-308].

iIELs as resident cells in the intestinal epithelium depend on the expression of gut-specific homing molecules, particularly β7 integrin and the chemokine receptor, CCR9. E-cadherin and CCL25, ligands for β7 integrin and CCR9, respectively, are expressed by epithelial cells [293]. In addition, CXCL10, a chemokine produced in great quantities in the inflamed intestine, also mediates the recruitment of CXCR3^+^ iIELs [309].

#### 7.3 Step-by-step sample preparation

For this study, small intestinal biopsies obtained by upper GI endoscopy are required. Biopsies from bulb or distal duodenum showed similar performance for this analysis [310]. At least 1000 live iIELs are needed for a reliable analysis. Results can be obtained from one piece of biopsy, however the inter- and intra-individual variability in the samples, led us to recommend the use of a minimum of two pieces of 6 mg each of mucosal tissue for this protocol.

Samples must be kept at 4°C in RPMI until processing. When samples cannot be processed within 24 h, the cryopreservation protocol is recommended.

iIEL isolation from human duodenal biopsies:

1. Transfer the samples into a 15 mL tube (tube 1) containing 5 mL of the IEL isolation medium.
2. Incubate the tube at 37°C and 250 rpm for 30 min. Horizontally in a falcon tube support fixed to the shaker plate if it is possible.
3. Shake the tube in the vortex for 10 s.
4. Transfer the solution to a new 50 mL tube (tube 2) with a 70 or 100 μm cell strainer containing 5 ml of cold RPMI.
5. Repeat the incubation for the residual tissue in the shaker using the same tube (tube 1).
6. Add the solution to the tube (tube 2) with a 70 or 100 μm cell strainer.
7. Centrifuge the tube (tube 2) at 400 × *g* for 5 min at 4°C.
8. Aspirate the supernatant before shaking the tube in the vortex for 10 s.
9. Proceed to staining.

Cryopreservation of duodenal samples:

1. Transfer the biopsies into a 2 ml tube (tube 0) containing 1 mL of frozen medium.

The tube (tube 0) is immediately stored at −80°C in a freezing container (CoolCell^™^ LX Freezing Container - Corning^™^ ref 432001) for at least 8 h. Then, it can be stored at −80°C for 6 months maximum. If longer storage is needed, vials may be transferred to liquid nitrogen.

The isolation of iIEL from cryopreserved samples requires some additional considerations:

- Defrost the samples immersing them at the water bath at 37°C for 1–2 min while moving.
- Place the samples in a Petri plate and wash with HBSS. Then, continue with the protocol as if they were fresh samples.

Lymphocyte extracellular staining:

1. Add 1 mL of FACS-Buffer and transfer to a 5 mL cytometry tube (tube 3).
2. Add 0.5 μL of Viability dye and incubate for 30 s.
3. Centrifuge the tube at 400 × *g* for 5 min at 4°C.
4. Aspirate the supernatant before shaking the tube in the vortex for 10 s.
5. Add 100 μL of FACS-Buffer and 2 μL of Fc block.
6. Incubate for 10 min, room temperature in the dark.
7. Centrifuge the tube at 400 × *g* for 5 min at 4°C.
8. Aspirate the supernatant before shaking the tube in the vortex for 10 s.
9. Add the extracellular Ab mix with FACS Buffer up to a total volume of 100 μL.
10. Incubate 20 min at 4°C.
11. Centrifuge the tube at 400 × *g* for 5 min at 4°C.
12. Aspirate the supernatant before shaking the tube in the vortex for 10 s.
13. Add 250 μL of Fixing Medium.
14. Incubate for 10 min at 4°C.
15. Centrifuge the tube at 400 × *g* for 5 min at 4°C.
16. Aspirate the supernatant before shaking the tube in the vortex for 10 s.
17. Add 500 μL of FACS-Buffer and preserve at 4°C until cytometry acquisition.

In some cases, an intracellular staining is also required. For example, for the staining of cytoplasmic CD3 to evaluate the presence of aberrant or abnormal lymphocytes that may be helpful for the diagnosis of Refractory celiac disease. For this, after step 12 of the extracellular staining proceed with an intracellular staining:

1. Fix the extracellular staining with 100 μL of Reagent A.
2. Incubate for 15 min at room temperature in the dark.
3. Centrifuge the tube at 400 × *g* for 5 min at 4°C.
4. Aspirate the supernatant before shaking the tube in the vortex for 10 s.
5. Add the intracellular Ab or Ab mix with Reagent B up to a total volume of 100 μL.
6. Incubate for 15 min at 4°C.
7. Centrifuge the tube at 400 × *g* for 5 min at 4°C.
8. Aspirate the supernatant before shaking the tube in the vortex for 10 s.
9. Continue as in steps 13 to 17 of the extracellular staining.
10. Cytometry acquisition: tubes are acquired at high speed during 5 min on a flow cytometer.

#### 7.4 Materials

##### Reagents

- IEL Isolation Medium: HBSS 1 mM DTT 0.5 mM EDTA.
- Hanks’ Balanced Salt Solution (HBSS) (Gibco, cat # 24020117)
- Dithiothreitol (DTT) (Sigma-Aldrich, cat # 43816)
- EDTA (Invitrogen, cat # 11568896)
- RPMI 1640 (Gibco, cat # 11875093)
- Fetal Calf Serum (FCS) (Gibco, cat # 10500064)
- Dimethyl Sulfoxide (DMSO) (MP Biomedicals, cat # 190186)
- Frozen Medium: FBS with 10% DMSO.
- DPBS/Modified (Cytiva, cat # SH30028.02)
- FACS Buffer: DPBS containing 1 mM EDTA and 0.02% sodium azide
- Sodium azide (Sigma-Aldrich, cat # S2002-25G)
- Fixing Medium: PBS 2% Buffered Formalin
- Buffered Formalin (Protocol, cat # 032-059)
- PBS (Lonza, cat # 17-516F)

##### Consumables

- Strainers 70/100 μm (Fisherbrand, cat #22363548, cat # 22363549)

##### Antibodies

Fc block (BD Pharmingen, cat # 564220)

Simple extracellular Ab mix for IEL phenotyping:

**Table:** 

Antigen	Fluorochrome	Company	Clone	Catalog number
CD45	PE-Cy7	BD Biosciences	HI30	557748
TCRγδ	FITC	BD Biosciences	B1	559878
CD3	APC	BioLegend	HIT3a	300312
CD7	BV421	BD Biosciences	M-T701	562635
CD4	BV510	BD Biosciences	SK3	562970
CD8α	Alexa Fluor700	BD Biosciences	RPA-T8	561453
Viability dye (Live/Dead)	–	Invitrogen	–	L10119

Other extracellular Abs can be added, for example:

**Table:** 

Antigen	Fluorochrome	Company	Clone	Catalog number
NKG2D	PE/Dazzle 594	BioLegend	1D11	320828

For intracellular staining, many Abs can be used, for example:

**Table:** 

Antigen	Fluorochrome	Company	Clone	Catalog number
CD3	PE-Cy5	BD Biosciences	UCHT1	555334

#### 7.5 Data analysis

**Figure 23.**
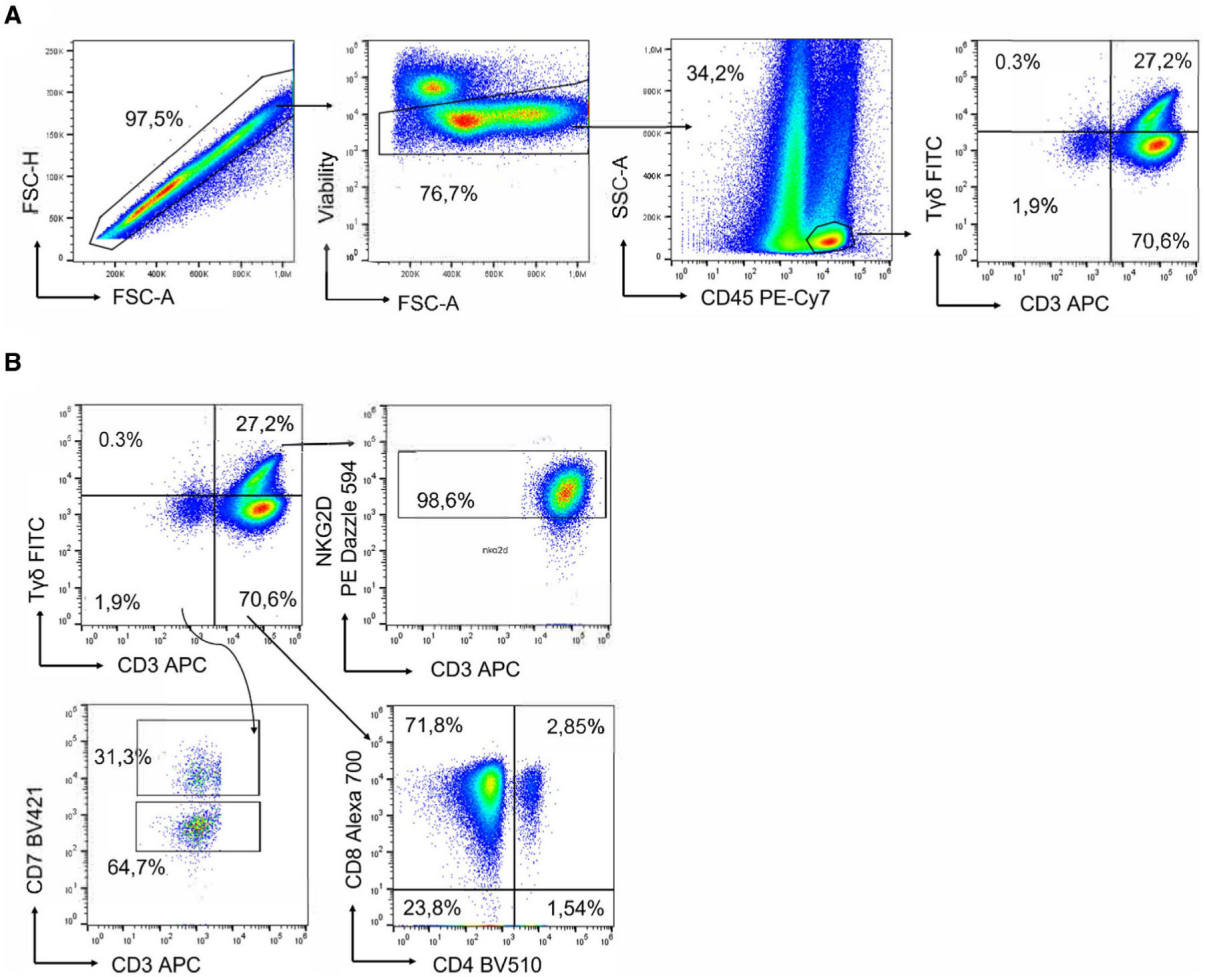
Identification and further analysis of intraepithelial lymphocytes in the human duodenum. A.- Total human intraepithelial lymphocytes (ilELs) from the duodenum were identified within singlet (97,5%) viable cells (76,7%) as CD45^+^ (34,2%), and further divided into classical T-cells (70,6%), TCRγδ T cells (27,2%) or NK-like cells (1,9%) based on the expression of CD3 and TCRγδ. This gating strategy is representative of a patient with active celiac disease, as noted by the high proportion of TCRγδ T cells coupled with the low proportion of NK-like cells. (**B)** The human iIEL subpopulations were further analyzed: NKG2D expression (98,6%) was measured for TCRγδ T cells, NK-like cells were divided into CD7^+^ (31,3%) and CD7^−^ cells (64,7%), and the proportion of CD8^+^ cells (74,65%), CD4^+^ cells (4,39%), double positive cells (CD4^+^CD8^+^) (2,85%) and double negative cells (CD4^−^CD8^−^) (23,8%) was assessed for T-cells.

The gating strategy to identify the different IEL populations start with the selection of SSC^low^CD45^+^ events (Figure 23). The singlet viable cells are divided based on the expression of CD3 and TCRγδ cells into classical T cells (CD3^+^ TCRγ8^−^), TCRγδ cells (CD3^+^TCRγδ^+^), or NK-like cells (CD3^−^TCRγδ^−^). Two groups of NK-like cells can be studied based on CD7 expression. For all subsets, the proportion of CD8^+^ cells, CD4^+^ cells, double positive cells (CD4^+^CD8^+^), and double negative cells (CD4^−^CD8^−^) can also be considered.

#### 7.6 Pitfalls

- Do not exceed incubation times during the viability and other staining procedures (in particular when reagent B is used for intracellular staining).

#### 7.7 Top tricks

- On the contrary to the evaluation of murine IELs, it is not required pre-warming the IELs isolation medium, since we do not observe any change in the final IEL counting using media at 4°C.
- During the second incubation with the IELs isolation medium, cells obtained in the first incubation can be stored at room temperature.

#### 7.8 Clinical relevance statement

The expansion of iIELs is a hallmark feature of the small intestine of celiac disease patients. The assessment of these cells was introduced years ago as a complementary tool in the diagnosis of the disease by immunohistochemistry of sections of duodenal biopsies [311, 312]. The rapid expansion of flow cytometry as a robust technique has made it the most used laboratory tool for the quantification and characterization of iIELs. In the healthy duodenum, more than 70% of iIELs are CD3^+^ T lymphocytes composed by 80% TCRα/β CD8^+^ T cells and 10–12% TCRγ/δ T cells, and a small proportion (<10%) of CD4^+^ cells; whereas the remaining 10–20% of IELs form a heterogeneous group of TCR^−^ CD3^−^ cells [299, 300, 313, 314].

The diagnosis of celiac disease is based on analysis by flow cytometry where an overall increase in the number of CD3^+^ iIELs, both TCRαβ and TCRγ/δ T cells, is observed. A significant increase in TCRγ/δ T lymphocytes and a marked reduction in CD3^−^ lymphocytes are the hallmark finding in celiac disease diagnosis [299]. Remarkably, TCR-γ/δ T cells remain at a higher number in spite of adherence to a GFD [315]. The diagnostic efficacy of the assessment of iIELs by flow cytometry has been confirmed for the differentiation of active celiac disease patients, Celiac disease in remission and subclinical or potential celiac disease in both pediatric and adult patients [315-318], and in the diagnosis and follow-up of the different forms of Refractory celiac disease [310, 319].

Flow cytometry allows the rapid and precise characterization of cell subpopulations from the small intestine (immunophenotyping), for clinical purposes: differential diagnosis between celiac disease and other disorders with villous atrophy such as post-enteritis syndrome, food allergy to soy/ milk/ cereals, giardia and other enteric parasitosis, immunodeficiencies, lymphomas, Crohn‘s Disease, etc.

Further investigations on cell function may include *ex vivo* studies by using extra- and intracellular staining and cell sorting, which are useful for the analysis of lymphocyte activation, cytokine profiles, programmed cell death, and detection of Ag-specific T cells by MHC-peptide tetramers.

#### 7.9 Summary of the phenotype

This is detailed in Table 14.

### 8 Mouse intestinal intraepithelial T cells

#### 8.1 Overview

In this section, we describe protocols to isolate and analyze murine intestinal intra-epithelial lymphocytes (iIELs) and lamina propria lymphocytes (LPLs) by flow cytometry. In particular, the protocol iIEL isolation and most of the subsequent flow cytometric analysis applies similarly to αβ and γδ iIELs, which are very similar cell types.

#### 8.2 Introduction

The intestinal epithelium constitutes one of the greatest surface barriers in mammals and is in continuous contact with the (gut luminal) environment. Composed by a mucosa, the intestine wall is made up of primary two layers, namely a one cell layer epithelium and the underlying lamina propria [320, 321]. In addition, the mouse small intestine contains five to seven Peyer’s patches in anti-mesenteric position, which are excised and removed during the protocol below, and numerous smaller aggregates of immune cells called cryptopatches and isolated lymphoid follicles, which will be largely included within the fraction of lamina propria lymphocytes. Continuously exposed to environmental cues and highly susceptible for pathogen assault, the intestine bears sophisticated and complex immune cell networks specific to each of the compartments [294]. In order to study the immune cells resident in both compartments of the murine intestine, a refined isolation of intra-epithelial lymphocytes (IELs) and lamina propria lymphocytes (LPLs) is advised according to the following protocols.

#### 8.3 Step-by-step sample preparation of lymphocytes from the mouse small intestine

##### 8.3.1 Isolation of IELs.

1. Pre-heat the IEL isolation medium at 37°C at the water bath
  - IEL isolation medium: 1 mM DTT + 10 μM KN-62 (stock at 50 mM) [322] + complete T cell medium (30 mL/sample)
  - complete T cell medium – RPMI 1640 + 10% FBS +1% Penny-strep + 1% NEAMM +0.1% β- mercapto-ethanol +1%HEPES 1M +1% Sodium Pyruvate
2. Harvest SI into ice cold PBS
3. Flush the intestine with ice cold PBS with a syringe and a gavage needle until it is clean.
4. Carefully remove fat and the Peyer’s patches.
5. Open longitudinally and clean it again in a Petri dish in ice cold PBS.
6. Cut the tissue into 1–2 cm pieces and transfer it to a 50 mL falcon tube (n1) in ice with PBS.
7. Vortex the tubes to further clean the intestine.
8. Transfer the tissue to a new clean 50 mL falcon (n2) containing 10 mL of the pre-warmed IEL isolation medium.
9. Shake the tube in the vortex for 10 s (optional).
10. Incubate the 50 mL falcon tubes at 37°C and 220 rpm for 15 min. (inside plastic beakers - 4/5 tubes- or in a falcon tube support fixed to the shaker plate).
11. After incubation vortex each tube for 10 s.
12. Transfer the solution to a new 50 mL tube (n3) with a 70 or 100 μm cell strainer and containing ≈15–20mL of ice cold complete T cell medium.
13. Repeat points 5–9 for two more times (using the same tube - n2).
14. Wash the intestine one last time with 10 mL of cold complete T cell medium and a quick vortex. Transfer the wash to the respective tubes (n3). For the LPL isolation, keep the intestines in a falcon tube on ice and proceed with the LPL protocol.
15. Centrifuge the tubes (n3) at 1250 rpm for 10 min at 4°C.
16. Aspirate the supernatant.
17. Resuspend the pellet in 4 mL of the 40% Percoll in complete T cell medium (5 ml per sample) and transfer to a 15 mL tube.
18. Wash the 50 mL tube with 1 mL of the 40% Percoll solution and transfer to the same 15 mL tube
19. Underlay the 80% Percoll in complete T cell medium (3 ml per sample) and centrifuge the tubes at 2000 rpm for 20 min at RT (without acceleration or break - 1 up and 1 down)
20. Remove the waste on top and recover the pinkish/white ring in-between the two phases. Place it in another falcon containing 3-5 mL of the complete T cell medium or MACS Buffer and top it up to 5mL
21. Centrifuge the samples for 10 min at 1250 rpm 4°C
22. Proceed to staining

##### 8.3.2 Isolation of LPLs.

1. Pre-warm the Digestion Medium in the water bath at 37°C
  - Digestion medium: DNAse I 125 μg/mL (stock 10 mg/mL) + Collagenase D 250 μg/mL (stock 50 mg/mL) + Complete T cell Medium (30 mL/sample)
2. After line 12. on IEL isolation protocol transfer the intestines into a petri dish and cut the tissue into smaller pieces with curved scissors (≈0.1cm).
3. Transfer the intestine to a 50 mL tube (n4) containing the warmed 10 mL digestion medium (at 37°C).
4. Just like for the IEL isolation protocol: Incubate the 50 mL falcon tubes at 37°C and 220 rpm for 15 min. (Inside plastic beakers - 4/5 tubes or in a falcon tube support fixed to the shaker plate – avoid that the tissue settles in the bottom of the tubes, as this will compromise digestion, by fixing the tubes at an angle).
5. After incubation, with a plastic transfer-pipet, pipet up and down the solution containing the intestine (5-10 times in order to help disrupt the tissue).
6. With the same transfer-pipet transfer the solution and filter it through a 70-μm cell strainer placed on new 50-mL tube (n5) containing ice-cold complete T cell medium + 100 μL of 4 mM EDTA. (We recommend the use of the MACS^®^ Smart-Strainers for these steps as they withhold higher volumes when filtering).
7. Collect the tissue in the cell strainer and repeat the procedure in points 2-5, two more times (using the same tube n4).
8. After filtering the last time, with a syringe lid (green) smash the pieces of tissue left behind in the strainer adding some more 4°C complete T cell medium.
9. Centrifuge the tubes (n5) at 1250 rpm for 10 min at 4°C.
10. Aspirate the supernatant.
11. Resuspend the pellet in 4 mL of the 40% Percoll solution in complete T cell medium (5 ml per sample) and transfer to a 15 mL tube
12. Wash the 50 mL tube with 1 mL of the 40% Percoll solution and transfer to the same 15 mL tube
13. Underlay the 80% Percoll solution in complete T cell medium (3 ml per sample) and centrifuge the tubes at 2400 rpm for 30 min at RT (1 up and 1 down).
14. Remove the waste on top and recover the pinkish/white ring in-between the two phases. Place it in another falcon containing 3–5 mL of the complete T cell medium or MACS Buffer and top it up to 5 mL.
15. Centrifuge the samples for 10 min at 1250 rpm, 4°C
16. Proceed to staining

#### 8.4 Materials

##### 8.4.1 Reagents.

- Dithiothreitol (DTT) (Sigma-Aldrich, cat # 43816)
- KN-62 – Selleckchem, cat. number: S7422
- RPMI 1640 (Gibco, cat # 11875093)
- FBS (Sigma, cat # F7524)
- Penny-strep (Gibco, cat #: 1514-122)
- MEM Non-Essential Amino Acids solution (MEM NEAA) 100X (Gibco, cat # 11140050)
- βmercapto-ethanol (Sigma, cat # M3148)
- HEPES (Sigma, cat # H0887)
- Sodium Pyruvate (Gibco, cat # 11360-039)
- Percoll (GE Healthcare, cat # 17-0891-01)
- PBS 1× (Gibco, cat # 1419-09)
- DNAse (Roche, cat # 11284932001)
- Collagenase D (Roche, cat # 1108886601)
- EDTA (Roth, cat # 8043.4)
- MACS Buffer – PBS 1×, 3% FBS, 5 mM EDTA

##### 8.4.2 Consumables.

- MACS^®^ SmartStrainers (70/100 μm) (Miltenyi Biotec, cat #130-098-462/130-098-463)

##### 8.4.3 Antibodies.

**Table:** 

Antigen	Company	Clone	Catalog number
CD45.2	Biolegend/Miltenyi	104/104-2	109836/130-103-787
CD4	Biolegend	GK1.5	100453
CD8α	Biolegend	53-6.7	100742
CD8ß	Biolegend	YTS156.7.7	126615
TCRß	Miltenyi	REA318	130-104-811
TCRδ	Biolegend	GL3	118120
Vδ4	BD Bioscience	GL2	745116
Vδ6.3	eBioscience	C504.17C	555321
Vγ1	Biolegend	2.11	141108
Vγ4	Biolegend	UC3-10A6	137706
Vγ7	Biolegend	F2.67	161702
Viability dye (Zombie)	Biolegend	–	423102

#### 8.5 Data analyses of mouse iIELs and LPLs

**Figure 24.**
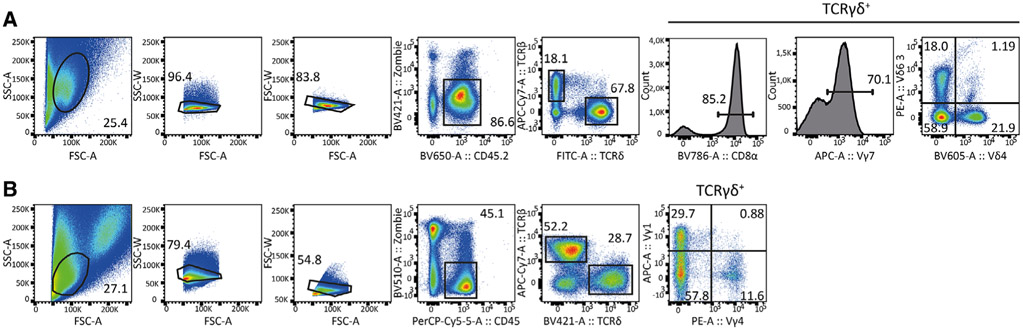
Representative gating strategy for TCRγδ^+^ population analysis of (A) murine small intestine intraepithelial lymphocytes (IEL) and (B) lamina propria (LPL). After isolation, lymphocytes were stained with Zombie (Live/Dead -Biolegend), CD45 (104 -Biolegend), TCRβ (REA318 - Miltenyi), TCRγ/δ (GL3 -Biolegend), CD8α (53-6.7 -Biolegend), Vγ7 (F2.67 - provided by P. Pereira: Institut Pasteur, Paris, France), Vδ6.3 (C504.17C -eBioscience), Vγ4 (GL2 -Biolegend) for the IEL cell suspension A and Vγ1 (2.11 -Biolegend) and Vγ4 (UC3-10A6 -Biolegend) for the LPL cell suspension.

**Figure 25.**
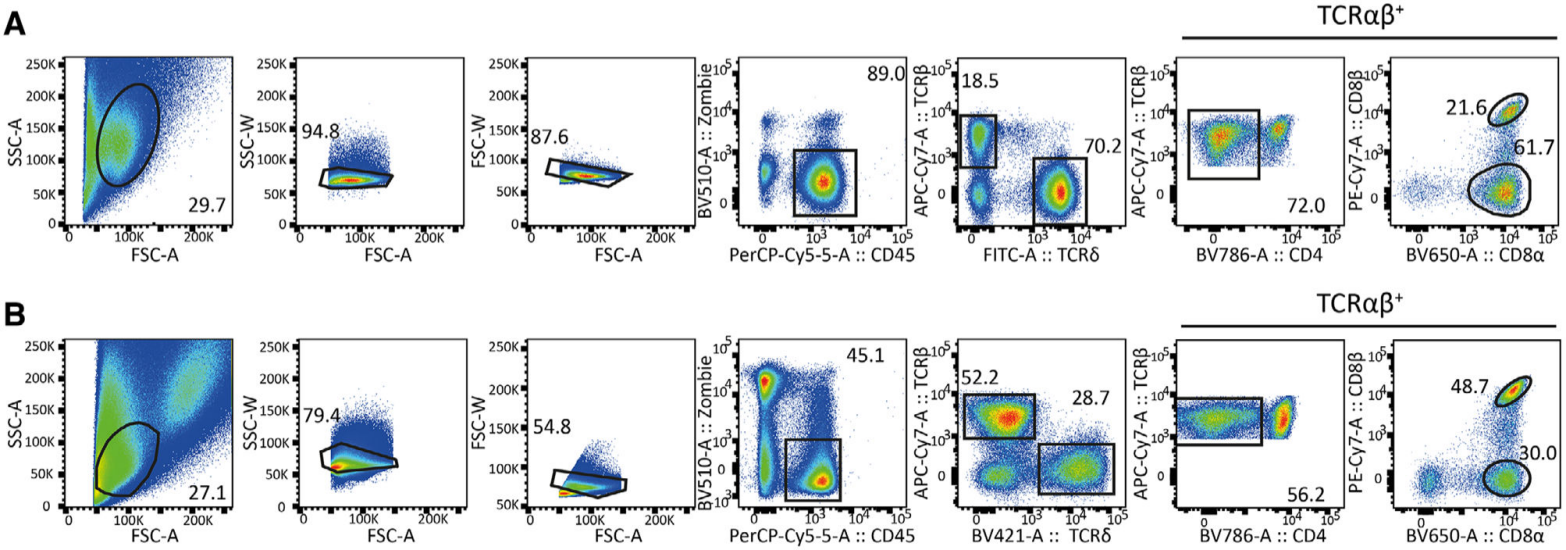
Representative gating strategy and analysis of **A** TCRαβ^+^ murine small intestine intraepithelial lymphocytes (IEL) and **B** lamina propria lymphocytes (LPL). After isolation, lymphocytes were stained with Zombie (Live/Dead - Biolegend), CD45 (104- Biolegend), TCRβ (REA318 - Miltenyi), TCRγ/δ (GL3 - Biolegend), CD8α (53-6.7- Biolegend), CD8β (YTS156.7.7 - Biolegend) and CD4 (GK1.5 - Biolegend).

The intestinal mucosa harbors lymphocytes, which are responsible not only for its protection but also to maintain integrity. Scattered along the intestinal epithelia, IELs are a heterogeneous population of T cells. Distinguished by their development and origin, IELs can be divided in two populations: the “natural” and the “induced” IELs. Derived from conventional αß T cells expressing CD4 (TCRαβ^+^CD4^+^) and classical CD8αβ molecules (TCRαβ^+^CD8αβ^+^), “induced” IELs relocate in the intestine mucosal tissue after cognate Ag engagement in the periphery and accumulate over time [294]. On the other hand, “natural” IELs differentiate in the thymus and are characterized by their TCRs’ ability to recognize self-Ags. Composed of both γδ T cells and αβ T cells, the large majority of “natural” IELs express the homodimer CD8αα but neither CD4 or CD8αβ [323] (Figure 24A and 25A). Moreover, acquisition of surface markers during development such as CD103 (αE integrin), α_4_β_7_ and CCR9 ensure homing and tissue residency of IELs [324] (see Table 15). In addition, “natural” CD8αα^+^ IELs display a chronically activated phenotype that can be translated by the expression of some activation markers such as CD69, CD122, and CD44 [325] (see Table 15).

Interestingly, γδ T cells homing in the small intestine display a (see Table 15) biased expression of TCRγ chains according to their localization. While γδ IELs are mostly Vγ7^+^, γδ T cells that home in the γδ LPLs express a broader variety of TCRγ chains (Figure 24) [326, 327].

Whereas the small intestine epithelium is enriched in “unconventional” CD8αα^+^ γδ T cells, approximately 60% of all lymphocytes (Figure 25A), the lamina propria is mostly composed of conventional αß T cells expressing CD8αß and CD4 (Figure 25B) [322]. Thereby, frequencies of CD4^+^ αß T cells within the iIEL preparation, and CD8αα^+^ αß T cells within the LPL preparation serve as reliable indicators of the level of unavoidable cross contamination during the isolation process.

#### 8.6 Pitfalls

- Be cautious to not exceed the incubation times point 8 (IEL) and point 3 (LPL) as this would decrease viability and yield of the protocols.

#### 8.7 Top tricks

- Adding the KN-62 reagent is helpful to support the viability of the cells once isolated, but does not increase the yield.
- Do not omit the DTT or try to replace it with beta-mercaptoethanol.
- Some IEL isolation protocols suggest to use HBSS medium, which we have not found helpful as compared to RPMI-based complete T cell medium.
- Follow the advice to pre-warm the isolation and digestion media and to use chilled PBS for cleaning the intestine.

#### 8.8 Summary of the phenotype

This is detailed in Table 15.

#### 8.9 Key differences human versus murine

This is detailed in Table 16.

### 9 Immune senescence (aging) of human T cells

#### 9.1 Overview

The use of flow cytometry allows determining the main quantitative and qualitative changes that occur in the T cell compartment of the immune system in humans. Aging T cells change mainly in the relative distribution of T cell subsets, and in the progressive tendency of T cells to undergo functional exhaustion. The most relevant age-related changes can be captured by addressing surface markers that are informative of differentiation stages, chemokine receptors to address homing capacities and co-stimulatory molecules – either with inhibitory or activating functions – which are related to activation and functional exhaustion.

#### 9.2 Introduction

Aging is associated with quantitative and qualitative changes of CD4^+^ or CD8^+^ αβ T cell subsets. The reduction in the frequency of naïve T cells, and the increase in the percentage of terminally differentiated effector T cells are hallmarks of immune aging [328]. The functional decline of the thymus observed with age ultimately results in a significant decrease in the output of naïve T cells [329], and leads to reduced number of T cells [330]. The reduction of naïve T cells is highlighted by the constant reduction over time of T cells that contain T cell receptor rearrangement excision circles i.e., circular DNA molecules produced during somatic recombination of TCR alpha chain, present exclusively in recent thymus emigrants [331]. Lymphopenia leads to an increase in the homeostatic proliferation of naive T cells, which is usually not sufficient to keep their number constant [332, 333]. Reduced numbers of naïve T cells are associated with a less diverse TCR repertoire of the total T cell compartment [334], even though the decline in the diversity of the repertoire is modest, as compared to the reduction of the thymic output [335]. The decline of naive T cell counts is paralleled by an increase in terminally differentiated effector T cells, which are also characterized by reduced clonality in old people. This phenomenon is mainly due to the continuous stimulation of T cells by chronic viral infections, in particular by cytomegalovirus (CMV) and, at a lesser extent, Epstein Barr virus (EBV) and human immunodeficiency virus 1 (HIV-1) infection [336]. Terminally differentiated T cells display a reduced proliferative capacity, evidenced by telomeres shortening [337]; they do not express co-stimulatory molecules such as CD27 and CD28, but do express markers such as KLRG-1 (a co-receptor bearing ITIM motif) and CD57 [337, 338], whose expression is largely overlapping. Although the ligand of CD57 is not known, compelling evidence indicates that the proliferative capacity of T cells expressing CD57 is severely impaired. Finally, exhaustion of T cells, i.e., a state of poor effector function observed in senescent T cells, is associated with the surface expression of immune checkpoints, such as PD-1 [339]. It must be noted that senescence does not equate exhaustion: replicative senescence seems to be irreversible whereas exhaustion is reversible, for instance by using anti-PD-1 Abs. Senescent T cells are prevalent among Tem cells, and adopt a pro-inflammatory profile, similarly to the senescence-associated secreting phenotype (SASP) that was observed in fibroblasts [340]. Exhausted T cells are usually CM/EM T cells that have been chronically stimulated [341]. Thus, the combination of differentiation and senescence markers allows to determine the change of T cell subsets with aging, and their “age,” in terms of senescence and/or exhaustion. As terminally differentiated T cells are characterized by enhanced cytolytic activity and a marked pro-inflammatory profile, the accumulation of these cells contributes to the chronic, low-grade inflammatory status, named inflammaging [342], which is characteristic of the old person.

#### 9.3 Step-by-step sample preparation

Aging of T-cells compartment can be monitored by analyzing CD4^+^ and CD8^+^ T cells by using 18 parameter flow cytometry, which allows the simultaneous analysis of parameters that vary at great extent with aging – T cell differentiation status, activation, and exhaustion. A classical approach based upon sequential gating to identify markers of activation, differentiation, senescence, exhaustion, regulatory CD4^+^ T cells, and memory stem T cells (T_SCM_) can be used. The protocol described below uses the Beckman Coulter DuraClone IM T cell panel (Beckman Coulter, Miami, FL, USA; Catalog n. B53328); supplemented with five additional mAbs to increase the number of parameters detected at the same time. Duraclone tubes are precoated with dried-down Ab cocktails. The use of pre-set cocktail reduces pipetting time and increases reproducibility by minimizing the by day-to-day variation in the preparation of mAb mix.

1. Thaw PBMC (if starting from frozen samples) washing twice with RPMI 1640 supplemented with 10% fetal bovine serum and 1% each of l-glutamine, sodium pyruvate, nonessential amino acids, antibiotics, 0.1 M HEPES, 55 μM β-mercaptoethanol, and 0.02 mg/ml DNAse;
2. Count cells and transfer 1 million of PBMC in a round bottom tube;
3. Wash the cells with 1 mL of phosphate-buffered saline (PBS) for 5 min at 930 × *g* at room temperature;
4. Discard the supernatant and resuspend the cells in 100 μL of PBS and stain with 0.3 μL/tube of the viability dye promokine-840 for 20 min at room temperature in the dark;
5. Wash the cells with 1 mL of PBS for 5 min at 930 × *g* at room temperature;
6. Discard the supernatant and resuspend the cells in 100 uL of brilliant stain buffer (BD Biosciences, Catalog No.563794) added with another five fluorescent mAbs (CD38, HLA-DR, CD95, CD127, CD25);
7. Transfer the cells within the Duraclone IM T cell subset tube incubating for 20 min at room temperature in the dark;
8. Wash the cells with 2 mL of FACS buffer (PBS + 2% FBS) for 5 min at 930 × *g* at room temperature;
9. Discard the supernatant and resuspend the cells in 500 μL of FACS buffer;
10. Acquire the samples in a flow cytometer.

Acquire a minimum of 500,000 cells per sample.

#### 9.4 Materials

##### 9.4.1 Media and buffers.

PBS

FACS buffer: PBS + 2% FBS.

##### 9.4.2 Surface staining.

Table 17 details the Ab information.

##### 9.4.3 Flow cytometer.

The experiment reported below has been performed on a CytoFLEX LX flow cytometer (Beckman Coulter)

#### 9.5 Data analysis

**Figure 26.**
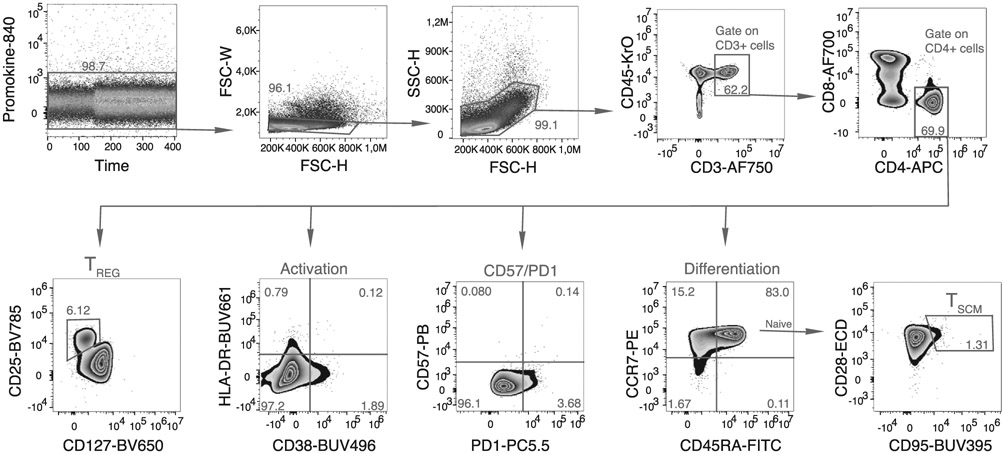
Gating strategy used to analyze markers related to differentiation, activation status, senescence, and exhaustion within human CD4^+^ T cells from a 45 y.o. healthy donor. Gating strategy is identical to that used in [126). Gate on Promokine-840 negative cells is used to exclude dead cells. Doublets are excluded based on FSC-H and FSC-W. T cells are identified based on physical parameters, and as CD45^+^CD3^+^ cells. Among CD3^+^CD4^+^ cells, naïve T cells are identified as CCR7^+^CD45RA^+^CD28^+^CD27^+^ cells; T_SCM_ are CCR7^+^CD45RA^+^CD28^+^CD27^+^CD95^+^; central memory (CM) are CCR7^+^CD45RA^−^CD28^+^CD27^+/−^; effector memory (EM) are CCR7^−^CD45RA^−^CD28^+/−^CD27^+/−^; terminal effector memory (Tem) are CCR7^−^CD45RA^+^CD28^−^CD27^+/−^. Activated cells are CD38^+^HLA^−^DR^+^; Treg are CD127^−^CD25^+^; exhausted/senescent are PD1^+^CD57^+^.

Please see Figure 26, which uses the gating strategy of ref. [126], for the analysis of markers related to differentiation, activation status, senescence, and exhaustion within human CD4^+^ T cells.

#### 9.6 Pitfalls/Top tricks

Before performing any analysis on T cell phenotype, check for positivity to CMV [343], which is a heavy confounding factor in aging of the immune system [344].

#### 9.7 Clinical relevance statement

The gating strategy shown in this section is applicable for analysis of 500,000 cells in healthy donors of different age (20-100 years old). Such analysis should evidence a lower percentage of Tn and T_SCM_ and higher percentage of Tem cells in old persons if compared to young donors, and an increase of senescent/exhausted cells in elderly donors [345].

#### 9.8 Summary of the phenotype

This is shown in Table 18.

### 10 Immune senescence (aging) in murine T cells

#### 10.1 Overview

Aging leads to loss of immune functionality with a well-documented impact on adaptive immunity, and in particular the T cell lineage [346]. Such changes have shown similarities in humans and mice. Therefore, this section will focus on the phenotyping of T cells in models of aging in mice.

#### 10.2 Introduction

**Figure 27.**
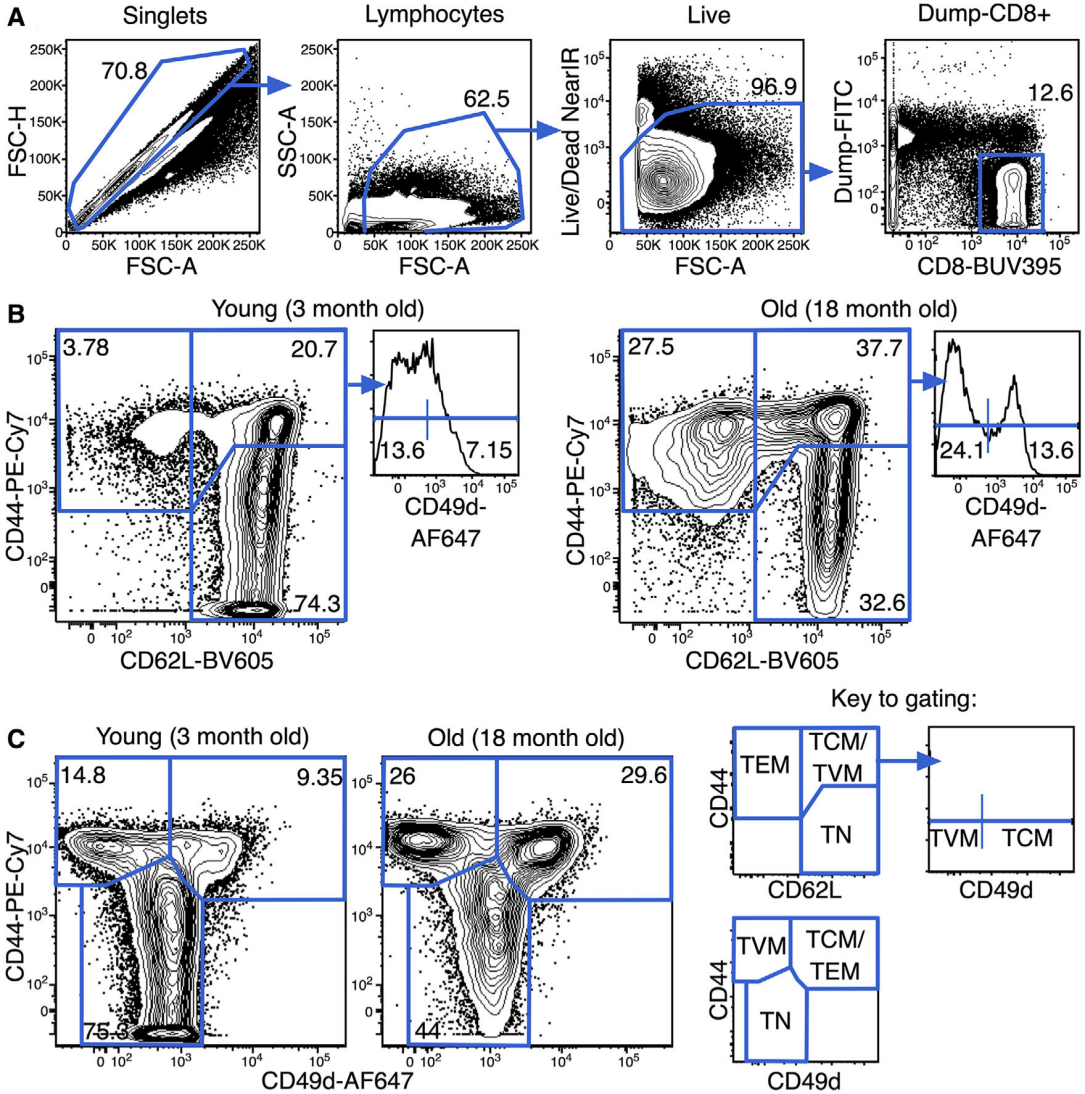
Gating strategy used to define Tn, TVM, Tcm, and Tem CD8 T cell subsets in naive mice, using splenocytes from naive SPF 3 month old and 18 month old C57BL/6J mice. (A) Gating strategy, where cells are gated on singlets, lymphocytes, live, dump-, CD8^+^ T cells and then (B) CD44 vs CD62L then CD49d or (C) CD44 vs CD49d to define the populations indicated in the key. Frequencies indicate the frequency of indicated subsets within the CD8 T cell population.

To study immune aging in mouse models, we need to consider the overlaps and the differences between the aging process in mice and humans. Such differences may reflect intrinsic differences between the two species (e.g., in lifespan, body mass, telomere length or telomerase activity [347], or the fact that humans experience a range of environmental exposures in the real world, whereas mice are typically studied in controlled and sterile environments of SPF facilities. Therefore, long-term exposure of mice to ubiquitous environmental microorganisms, such as chronically persistent viral infections, most prominently CMV infection (see section III.9 Immune senescence (aging) of human T cells), may reflect natural aging processes better than their maintenance in sterile environments [348, 349]. When working with mice, 18 months of age or older is considered truly aged [350], as many mouse strains survive longer than 600 days in standard SPF housing [351]. Similar to differences between young and old people, 3 month old young mice have high frequencies of naïve T cells in blood and lymphoid tissue, but the relative frequency (Figure 27) and absolute counts of naïve T cells decline substantially with age as the thymus involutes. In contrast, the frequency (Figure 27) and counts of memory T cell subsets, particularly more differentiated populations, increase with age as the collective history of antigenic encounters makes a mark on the aging host (see section III.9 Immune senescence (aging) of human T cells).

Phenotyping of naïve and memory T cell subsets by flow cytometry relies on a combination of markers that are acquired or lost during T cell differentiation, from naïve and memory to terminally differentiated T cells [352]. Some markers that are used to identify naïve and memory T cell subsets in humans such as CD45RA [352] (see section III.9 Immune senescence (aging) of human T cells) are not suitable for phenotyping murine T cell subsets, mainly because they do not allow reasonable separation in discrete positive and negative fractions. As a result, markers such as CD44, CD62L, and CCR7 are used in mice to identify naïve (Tn), central memory (Tcm), and effector memory (Tem)/effector (Teff) subsets, as well as KLRG1 and CD127, which are used to identify memory precursor effector cells (MPEC) and the short-lived effector cells (SLEC) populations, as described previously (see section III.4 Murine Conventional αβ CD8 T cells and section III.6 Murine tissue resident memory T cells).

**Figure 28.**
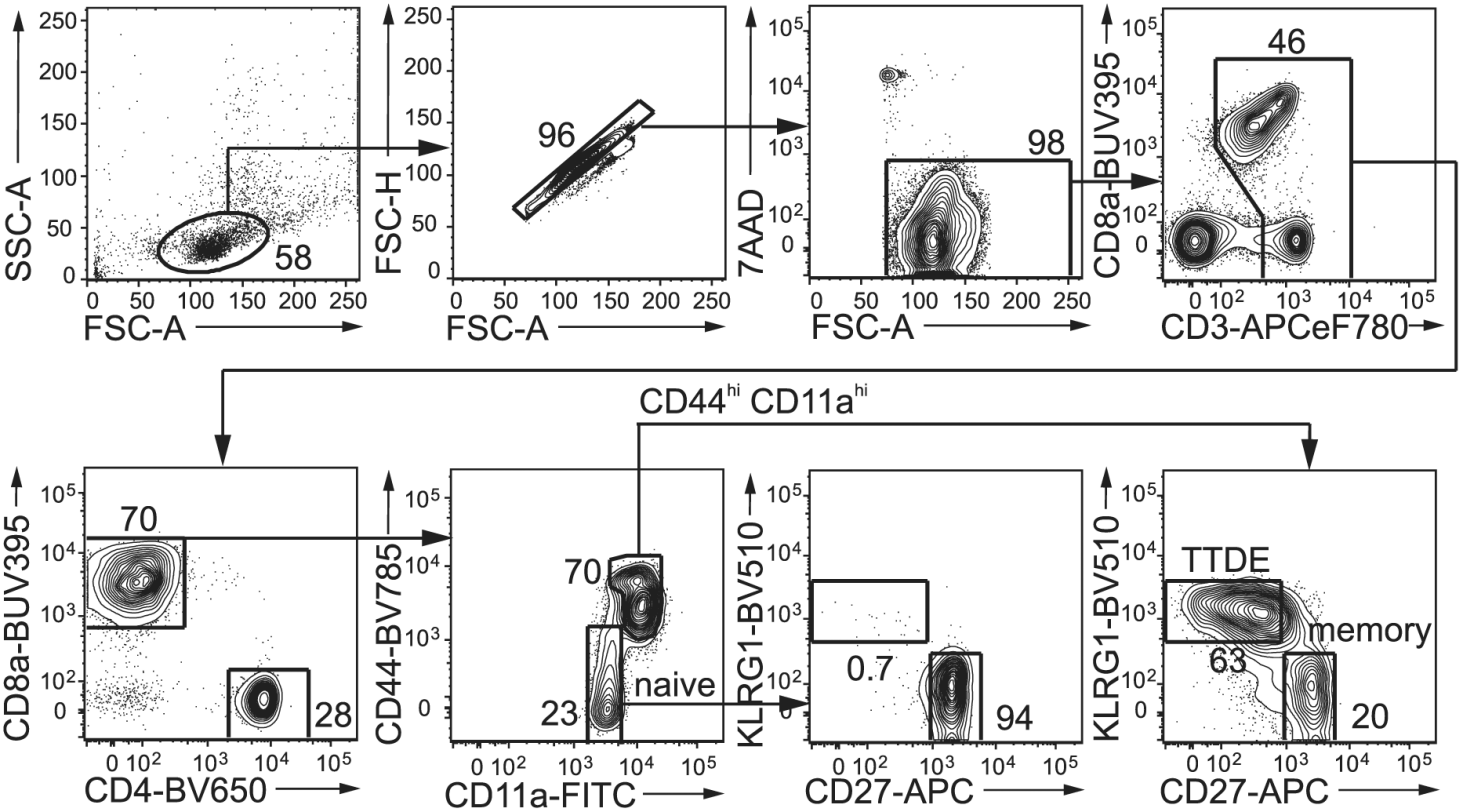
Gating strategy used to define naïve, memory, and TTDE CD8 T cell subsets in aged chronically infected mice (applies also to Figure 29 and 30). Flow cytometry analysis of the peripheral blood of 8 month old C57BL/6J mouse experimentally infected for 6 months with 10^6^ PFU of a chronically persistent β-herpesvirus, murine cytomegalovirus (MCMV).

In addition to these classical T cell subsets, we can assess senescence markers in T cells. Some surface markers used in humans (see section III.9 Immune senescence (aging) of human T cells) such as CD57, the lack of CD28 and the re-emergence of CD45RA expression, do not translate into mice. Telomere length is also commonly assessed in humans as an indicator of cellular age and replicative senescence, sometimes by flow cytometric methods, but this approach is limited in mice as telomeres are relatively long, meaning that telomere erosion may not be a major driver of immune aging [347]. However, senescent T cells in mice do exhibit increased expression of NK cell related markers, such as KLRG1, and the loss of CD27, allowing us to robustly separate memory subsets and more terminally differentiated populations in mice (Figure 28). Senescent T cells in mice and humans both exhibit an increase in phosphorylated γH2Ax subunits in the cytosol as an indicator of increased ATM kinase activity, increased DNA damage, and a DNA-damage senescence phenotype [353, 354].

Accordingly, for analysis of aging phenotypes in mice, one should profile the differentiation status of the overall T cell population and assess senescence markers in these subsets, but the exact method of T cell phenotyping may differ depending on the experimental context and infection history of the mice.

#### 10.3 Step-by-step sample preparation

##### 10.3.1 Sample collection and RBC lysis.

1. Collect a defined volume of blood (up to 75 μl using a heparinized hematocrit capillary and dispense it into an Eppendorf tube containing 300 μl of HBSS-EDTA buffer.
2. Remove 75 μl for absolute blood cell counting and process as indicated in section 10.3.2.
  Proceed with the remaining blood in HBSS as indicated below.
3. Centrifuge for 5 min at 700 × *g* at 4°C.
4. Aspirate supernatant and resuspend pellet in 600 μl of distilled water. Immediately thereafter (max 5-10s), add 200 μl of 4× PBS and briefly mix by pulse vortexing.
5. Centrifuge for 5 min at 700 × *g* at 4°C.
6. Aspirate most of the supernatant (leave approximately 100 μl), resuspend cells in the remaining volume, and transfer into a 96-well plate.
7. Centrifuge for 3 min at 700 × *g* at 4°C.
8. Flick off the supernatant and resuspend pellet in 150 μl of distilled water using a multichannel pipette. Immediately thereafter (max 5–10 s), add 50 μl of 4× PBS with a multichannel pipette, and mix thoroughly by pipetting. Discard tips between rows to avoid carryover cell contaminations.
9. Centrifuge for 3 min at 700 × *g* at 4°C
10. Flick off supernatant and proceed with Ab staining as described in previous chapters (see section III.4 Murine Conventional αβ CD8 T cells).

##### 10.3.2 Absolute cell counts.

Lymphocyte counts per volume of blood can be obtained using automated hematology analyzers according to manufacturer’s guidelines. For measurements using VETSCAN HM5 (Abaxis), a minimum of 75 μl of HBSS-EDTA diluted blood is transferred to an Eppendorf tube and acquired. Absolute values are calculated in relation to the volume of blood and HBSS-EDTA:

Cr=Co(VT∕VB)

where $C_{r}$ is the real count of blood cells, $C_{o}$ is the count observed in the analyzer, $V_{B}$ is the volume of the blood in the acquired sample, and $V_{T}$ is the total volume of the blood with HBSS-EDTA at the time of acquisition. Alternatively, absolute number of cells in a stained sample can be determined using flow cytometry counting beads (e.g., Precision Count Beads, BioLegend) according to manufacturer’s protocol.

#### 10.4 Materials

##### 10.4.1 Media and buffers.

HBSS-EDTA: HBSS 5mM EDTA; Staining buffer: phosphate-buffered saline (PBS) 2% (v/v) FBS;

##### 10.4.2 Antibodies for uninfected mice.

Dump-FITC (anti-B220 (clone RA3-6B2), anti-CD11c (clone HL3), anti-CD11b (clone M1/70), anti-F4/80 (clone BM8), anti-NK1.1 (clone PK136) all 1:400 dilution), anti-CD49d-Alexa Fluor 647 (clone R1-2; 1:200), anti-CD44-PE-Cy7 (clone IM7; 1:800), anti-CD62L-Brilliant Violet 605 (clone MEL-14; 1:800) (all from BioLegend), anti-CD8a-Brilliant Ultraviolet 395 (clone 53-6.7; 1:400) (BD Biosciences); Cell Viability Stain: LIVE/DEAD Fixable Near-IR Dead Cell Stain (Molecular Probes; 1:800)

###### 10.4.2.1 Antibodies for infected mice.

Anti-CD11a-FITC (clone M17/4), anti-CD122-PE (clone TM-β1), anti-CD27- APC (clone LG.3A10), anti-CD3e-APC-eFluor 780 (clone 17A2), anti-CD62L-Pacific Blue (clone MEL-14), anti-KLRG1-Brilliant Violet 510 (clone 2F1/KLRG1), anti-CD4-Brilliant Violet 650 (clone GK1.5), anti-CD44-Brilliant Violet 785 (clone IM7) (all from BioLegend), anti-CD8a-Brilliant Ultraviolet 395 (clone 53-6.7) (BD Biosciences); Cell Viability Stain: 7-AAD (BioLegend)

####### Flow cytometer:

Experiments were performed on an LSR Fortessa (BD Bioscience) equipped with laser excitation lines of 360, 405, 488, 561, and 640 nm and the following filter configuration: 386/23(365) for BUV395; 450/50(405) for Pacific Blue; 525/50(405) for BV510, 655/40(405) for BV650; 785/60(405) for BV785; 525/50(488) for FITC; 685/35(488) for 7-AAD; 585/15(561) for PE; 780/60(561) for PE-Cy7; 670/14(640) for APC; 710/40(640) for Alexa700; 780/60(640) for APC-eF780.

#### 10.5 Data analysis

##### 10.5.1 Identification of T cell subsets in aged, uninfected mice.

Naïve aged mice that are held in an SPF facility will have had limited antigenic exposure. After using the classical markers, CD44 and CD62L, for defining naïve and memory subsets in peripheral blood and lymphoid tissue, naïve mice exhibit a clear shift with age in T cell subset frequencies, with a decrease in the naïve subset and an increase in memory subsets. Of note, this shift in frequency with age is driven by a marked decrease in naïve T cell numbers, particularly CD8 T cells, while memory cell numbers increase more modestly, consistent with their limited antigenic exposure.

Tn cells are CD44^−^CD62L^+^ and Tem (and Teff) cells are CD44^hi^CD62L^−^ cells but, for CD8 T cells, the CD44^hi^CD62L^+^ population contains both Tcm and virtual memory (TVM) cells (Figure 27 and Table 19). Cells must be stained with CD49d to differentiate between Tcm and TVM cells, with CD49d^hl^ denoting Ag-experienced Tcm cells and CD49d^lo^ denoting Ag-inexperienced but cytokine-exposed TVM cells. This distinction becomes important in ageing research as the proportion of CD8 T cells that are TVM cells increases markedly with age (Table 19) and these cells have been misclassified in the past as Tcm cells [355]. In addition, TVM cells express high levels of CD122 and NK cell markers, both of which increase with age and would otherwise be misattributed to Tcm cells [354, 356]. Of note, recent work has shown that age-related accumulation of TVM cells is independent of their environmental microbial exposure [357], so their accumulation appears to be an age-dependent effect.

An additional feature of ageing in mice is that the expression level of CD44 on Tn cells increases, not to become CD44^hi^, but Tn cells become predominantly CD44^int^ (Figure 27). This may indicate that the average post-thymic age of aged Tn cells is increased or that aged Tn cells are exposed to the inflamed aged environment, which is driving modest activation and increased CD44 expression.

##### 10.5.2 Identification of T cell subsets in aged chronically infected mice.

**Figure 29.**
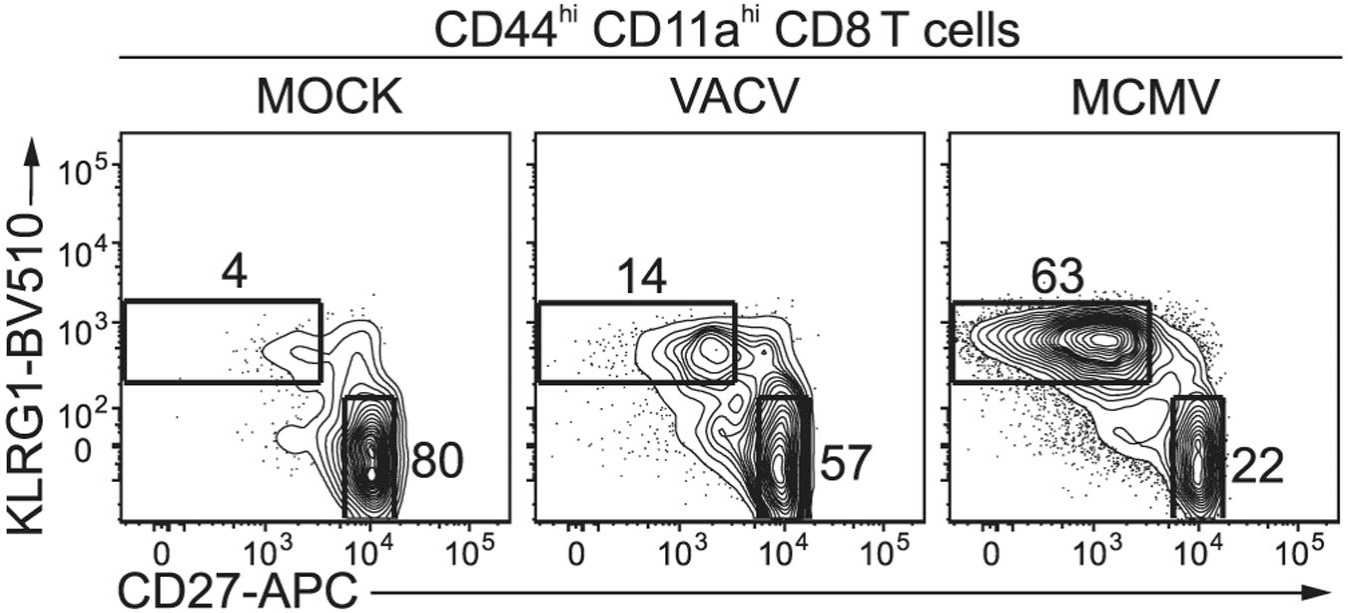
Flow cytometry analysis of KLRG1 and CD27 expression on total CD44^hi^CD11a^hi^ CD8 T cells (pre-gated according to the gating strategy shown in Figure 28) in the peripheral blood of 15 month old (BALB/c×DBA/2) F1 mice experimentally infected for 9 months with 10^6^ PFU of a non-persistent virus, Western Reserve vaccinia virus (VACV) or 10^5^ PFU of a chronically persistent β-herpesvirus, murine cytomegalovirus (MCMV) compared to uninfected littermate mice (MOCK).

**Figure 30.**
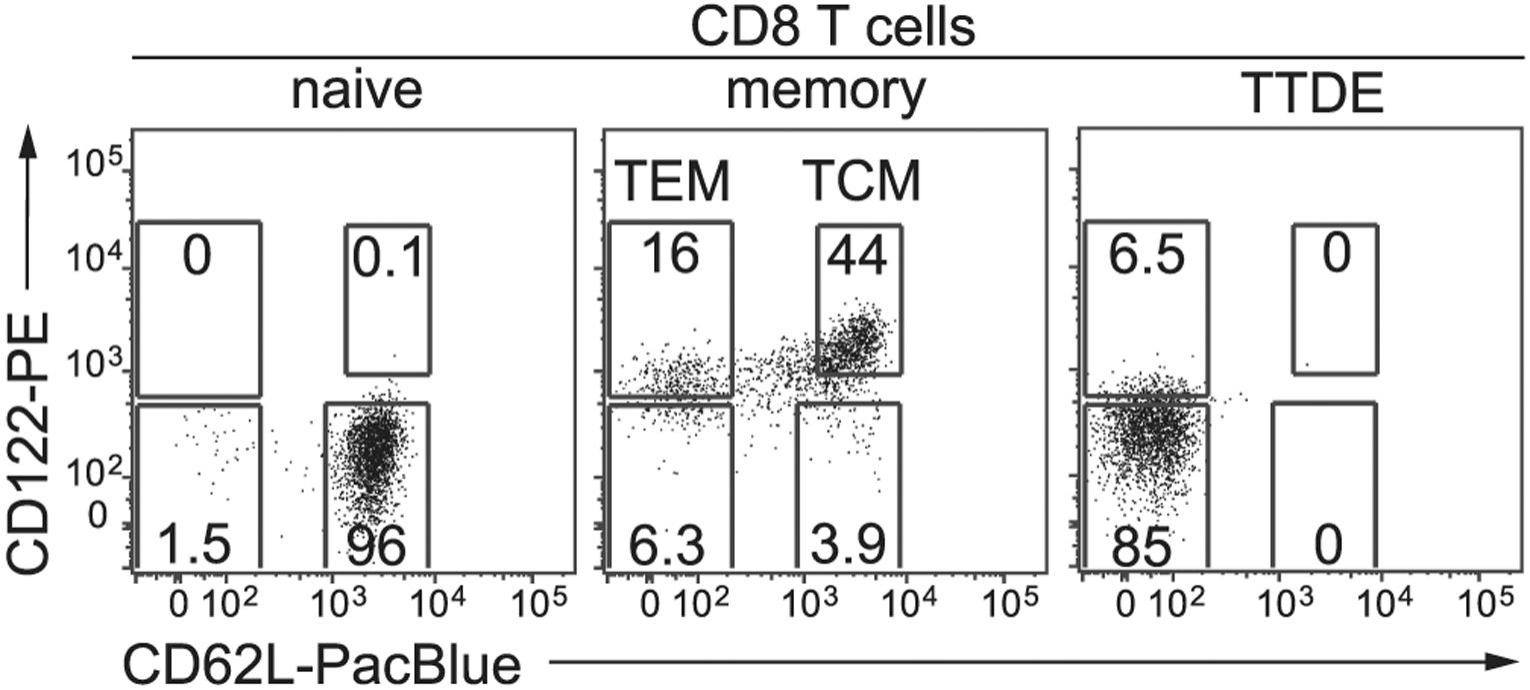
Flow cytometry analysis of CD122 and CD62L expression in naïve, memory, and TTDE CD8 T cell subsets (pre-gated according to the gating strategy shown in Figure 28) in the peripheral blood of 8 month old C57BL/6J mouse experimentally infected for 6 months with 10^6^ PFU of MCMV.

Upon infection, particularly infection with persistent pathogens such as the chronically persistent β-herpesvirus, murine cytomegalovirus (MCMV), T cell populations progress more rapidly toward an aged phenotype, with more terminally differentiated subsets and increased expression of senescence markers (Figure 29 and reference [348]). Therefore, a shorthand for the progression of immune aging phenotypes is given by the frequency and absolute counts of KLRG1^+^CD27^−^ terminally differentiated effector T cells (TTDE). A common strategy to define naïve cells is to combine CD44 and CD62L staining, where CD44^−^ CD62L^+^ cells are considered naïve. Some commonly used mouse strains (e.g., BALB/c) show a poor separation of naïve from memory cells based on the CD44 marker so an improved separation of naïve CD8 T cells may be achieved by combining CD44 and CD11a labeling, where CD44^−^CD11a^lo^ correspond to naïve cells, although neither of these markers alone robustly separates naïve from primed cells (Figure 28). In addition, CD122, which is expressed on TVM and Tcm cells, but not on Tn cells, can be used in combination with CD62L to more efficiently separate naïve cells from other subsets (Figure 30).

It is important to emphasize that phenotyping for immune aging will necessarily require concurrent measurements of absolute lymphocyte counts per ml of blood. Namely, lowered percentages, but not absolute counts of naïve cells may also be observed due to expansions of TTDE population in persistent herpesviral infections, such as cytomegalovirus infection [348], but this does not impair immune protection against infections [358]. In conclusion, a combination of six markers (CD11a, CD44, CD27, KLRG1, CD62L, and CD122) allows the distinction between Tn, Tcm/TVM, Tem, and TTDE T cell populations in mice experimentally infected for 6 months with 10^6^ PFU MCMV (Table 20), with a robust identification of age-related losses of naïve cell populations and increases in terminally differentiated CD8 T cells, matching functional changes in aging humans.

#### 10.6 Pitfalls and top tricks

When working with aged mouse models, consider that mice will be housed in SPF conditions, which is quite different to humans, where pathogen exposure accumulates over the lifespan.

Aged mice can accumulate age-related abnormalities, such as tumors, or they can overgroom, which can lead to skin abrasions and infections. This can lead to immune activation in individual aged mice, so many researchers exclude mice with overt abnormalities from analyses.

TVM cells are selectively retained with increasing age and are often misidentified as Tcm cells. Including CD49d in staining panels enables identification of Tcm cells as distinct from TVM cells.

Aged leukocytes can be more sensitive to physical manipulation, especially RBC lysis, so make sure to time this reaction carefully.

#### 10.7 Summary of the phenotypes

These are detailed in Tables 19 and 20.

#### 10.8 Key differences human versus murine

- CD45RA expression in mice cannot clearly discriminate positive vs negative cells, as in humans;
- CD57, CD28 expression are useful for identifying T cell subsets in humans, but not in mice;
- CD44 and CD62L are commonly used in mice instead of CD45RA and CCR7.
- CD62L is usually used in mice, but CCR7 can be used as well.
- CD11a is not used in human T cell phenotyping.

This is summarized in Table 21.

### 11 Human FOXP3^+^ regulatory T cells

#### 11.1 Overview

Regulatory T cells (Tregs) are necessary to protect against autoimmune disease and maintain immune homeostasis. Human Tregs are usually defined by high co-expression of the FOXP3 transcription factor and CD25, as well as low expression of CD127. Other aspects of their phenotype can vary widely depending on their state of activation and location throughout the body. In order to identify human Tregs on the basis of FOXP3 expression, flow cytometric staining protocols need to ensure effective permeabilization of both cellular and nuclear membranes. Another consideration is how to differentiate between Tregs and activated conventional T cells (Tconvs) that transiently express FOXP3 and CD25. In this section, we will discuss protocols and key considerations for staining human Tregs in whole blood and peripheral blood mononuclear cells (PBMCs) as well as protocols for digesting and analyzing Tregs in a variety of human tissues including intestine, thymus, skin, and adipose.

#### 11.2 Introduction

##### 11.2.1 Human Treg frequencies and distribution.

Tregs are present throughout the human body and their abundance in circulation and tissues is age dependent [264, 359]. For example, in early life (i.e., under 2 years), Tregs (defined as CD25^hi^CD127^lo^FOXP3^+^ cells) make up 30-40% of CD4^+^ T cells in the lung and gut but these proportions decline to 1-10% in adults [360]. In peripheral blood, Tregs decrease from ~20% of total CD4^+^ T cells in infants (i.e., under 2 years) to ~5% in healthy adults [360]. However, once adult proportions of Tregs are reached, their frequencies in blood do not appear to change with age (from 20 to 75 years; Tregs defined as CD25^hi^CD127^lo^ cells in this study) and they maintain suppressive capacity [361, 362].

##### 11.2.2 Human Treg subsets.

As in mice, it is generally accepted that human Tregs can be thymically derived or induced from Tconv cells in the periphery under specific conditions [363]. In mice, high expression of Helios and low expression of Neuropilin-1 (Nrp-1) has been proposed to discriminate between thymus Treg and peripherally-induced Tregs [364, 365] (see section III.12 Murine Foxp3^+^ regulatory T cells). In humans, however, the validity of these markers is less clear because not all naive/thymus-derived Tregs express Helios [173] and it has been reported that this protein can also be expressed by activated T cells [366]. On the other hand, human Tregs that express high levels of Helios have a potent suppressive phenotype and are more stable [367]. We have recently shown that expression of Helios is a better predictor of Treg stability than FOXP3 [368] so measuring expression of Helios should be routinely included in all human Treg staining protocols. Neuropilin-1 (Nrp-1) has been proposed to mark stable mouse Tregs, but expression of this protein is almost undetectable in human peripheral Tregs [369].

Of particular interest is that Tregs subsets can be readily identified in healthy adults with phenotypes similar to the well-described CD4^+^ T helper (Th) cell subsets. Specifically, Th1, Th2, Th17, and Th17.1-like Tregs can be detected in peripheral blood and identified on the basis of expression of Th-cell-associated chemokine receptors and/or transcription factors [370]. In contrast to Th cell subsets, however, in healthy individuals, Treg subsets typically do not make high amounts of lineage-associated cytokines (e.g., IFN-γ, IL-2, IL-4, IL-13) [371], likely because of the transcriptional repressor function of FOXP3. An exception is IL-17: Th17 Tregs co-express FOXP3 and IL-17 yet remain functionally suppressive [372, 373]. Although the relevance of Th-like Tregs in human disease and homeostasis is an area of intense investigation, it currently appears that they are tailored to regulate immune responses driven by their corresponding Th cell subset. Mechanistically, this could occur by differential homing receptor expression, thus ensuring that Th- like Tregs co-localize with their Th cell subset counterparts [374].

Recently, a subset of human Tregs with putative tissue repair capabilities was described. Based on previous work in mice, [375-377] human FOXP3^+^BATF^+^CCR8^+^ Tregs were characterized using single-cell chromatin accessibility assay and single-cell RNA- and TCR-sequencing. This subset of Tregs is present at a low frequency in the blood but dominates the Treg population in peripheral tissues, including the skin and fat [378]. In contrast to Th1-, Th2-, or Th17-like Tregs, FOXP3^+^BATF^+^CCR8^+^ Tregs are characterized by a Tfh-like program, exemplified by the expression of BCL-6, ICOS and PD-1 [378].

##### 11.2.3 Measuring human Tregs by flow cytometry.

Identifying human Tregs using flow cytometry is complicated by the facts that FOXP3 is an intranuclear marker with a relatively low intensity of expression, and there is currently no known single marker that is unique to human Tregs. Moreover, even within Tregs the intensity of FOXP3 expression can change, with naive or resting populations of Tregs expressing lower levels of FOXP3 than activated Tregs [379, 380]. Hence, accurate separation between Tconv, resting Tregs, and activated Tregs can only be done if there is a relatively high dynamic range of FOXP3 staining and often requires addition of other makers such as CD45RA. Currently the only way to confidently quantify human Tregs is to use a panel of different markers and then carry out parallel functional [381], gene expression [382], and/or epigenetic analyses [383, 384].

In terms of surface phenotype, the best accepted combination of markers is high expression of the IL-2 receptor α chain (CD25) and low expression of the IL-7R α chain (CD127) [134, 385]. Importantly, this CD25^hi^CD127^lo^ Treg definition can be used to isolate relatively pure populations of viable peripheral Tregs in both the memory (CD45RO^+^) and naïve (CD45RA^+^) compartments. As with FOXP3, CD25 and CD127 are both expressed as a continuum so in order to be confident in gating strategies, it is essential that there is good separation between low, medium, and high expressing populations.

An outstanding question is should human Tregs be defined as CD4^+^ cells that are CD25^hi^CD127^lo^, CD25^hi^FOXP3^+^, FOXP3^+^, or CD25^hi^CD127^lo^FOXP3^+^. Currently the literature reports "Tregs" as cells defined by any one of these different variations. It is therefore critical to consider how studies define "Tregs" and whether or not the gating strategies used are sufficiently stringent. On the basis of current knowledge, the most rigorous way to define Tregs is as CD25^hi^CD127^lo^FOXP3^+^ cells. However, use of FOXP3 is not possible when viable cells are being sorted and sometimes it may not be feasible to include all three markers due to instrument limitations. If all three markers are not possible then at a minimum two should be used, either CD25^hi^FOXP3^+^ or CD25^hi^CD127^lo^. Measuring only CD25 or only FOXP3 does not provide sufficient resolution on a dot plot to set an accurate gate. Since Tconvs can also upregulate CD25 and FOXP3, and downregulate CD127 [380, 386], it is critical to set gates for all of these markers on resting populations of T cells, typically using blood cells from healthy individuals. This is particularly helpful when analyzing Tregs and Tconvs in tissues where there can be a significant proportion of activated Tconvs and it thus becomes important to differentiate between these cells on the basis of the intensity of CD25 and FOXP3 expression. Addition of other human Treg-associated markers can help increase confidence in Treg identification in these settings.

Here, we detail optimized protocols for detecting human Tregs in whole blood and PBMCs as well as a variety of human tissues including thymus, intestine, skin, and adipose. These protocols differ from those provided in Mouse FoxP3^+^ Tregs (see Section III.12 Murine Foxp3^+^ regulatory T cells) both in terms of the tissue processing approach used and the subsequent method by which Tregs are identified. These differences are due, in part, to the fact that human Tregs cannot be identified by expression of a fluorescent FoxP3 reporter molecule. We also provide some tips in Table 37 (see below) for staining additional human Treg markers that are not specifically included in the protocols outlined and highlight some key differences between mouse and peripheral human Tregs. The reader is also referred to our previously published methods for staining human Tregs within omental adipose tissue [387], thymus tissue [388], and using a mass cytometry platform [389, 390], as well as a review describing a comprehensive comparison of mouse and human Treg markers [391]. The methods described here are focused on studying Tregs in healthy subjects. The markers of choice may vary in different disease contexts. For example, tumor-infiltrating Tregs often have a distinctive phenotype associated with T cell exhaustion and the addition of markers such as PD-1 may be beneficial in a flow cytometry panel [392].

The following points are key for analyzing human Treg by flow cytometry:

- In order to accurately define Tregs, at least two markers must be used - either CD25 and CD127 or CD25 and FOXP3; use of all three is optimal. If FOXP3 cannot be used then CD25 must be used in combination with CD127.
- Inclusion of Helios is useful to discriminate between Tregs and activated Tconvs and to identify stable Tregs (defined as FOXP3^+^Helios^+^ cells)
- To set an accurate FOXP3 gate, a biologically negative population (e.g., CD4^+^ Tconv or CD8^+^ T cells) is required.
- The selected anti-CD25 and anti-FOXP3 mAbs must be conjugated to a bright fluorochrome so that there is good separation between mid and high expressing cells (e.g., BB515, PE- Cy7, or PE).
- Separation of cells expressing mid and high levels of CD25 and FOXP3 can be enhanced by the use of two monoclonal Ab clones recognizing independent binding epitopes, conjugated to the same fluorochrome.

#### 11A.3 Step-by-step sample preparation: Staining Tregs from unmanipulated whole blood protocol 1A

In a clinical context, it is desirable to quantify Tregs in unmanipulated whole blood. In contrast to the more widely used approach of phenotyping cryopreserved PBMCs, this method allows quantification of absolute Treg numbers and avoids variability introduced by cryopreservation [393]. Several studies have compared different anti-human FOXP3 Ab clones, and although there is some debate, there is a general consensus that the 236A/E7 and 259D clones are optimal [394-398]. The following protocols and associated Ab panels should be used as a guide; substitution of Ab clones/conjugations requires titration and testing in combination with the selected buffer system. Below we present two protocols using reagents from different manufacturers to quantify Tregs in whole blood.

##### 11A.3.1 Staining CD25^hi^CD127^lo^FOXP3^+^ Tregs from whole blood using pre-formatted DuraClone tubes from Beckman Coulter.

**Figure 31.**
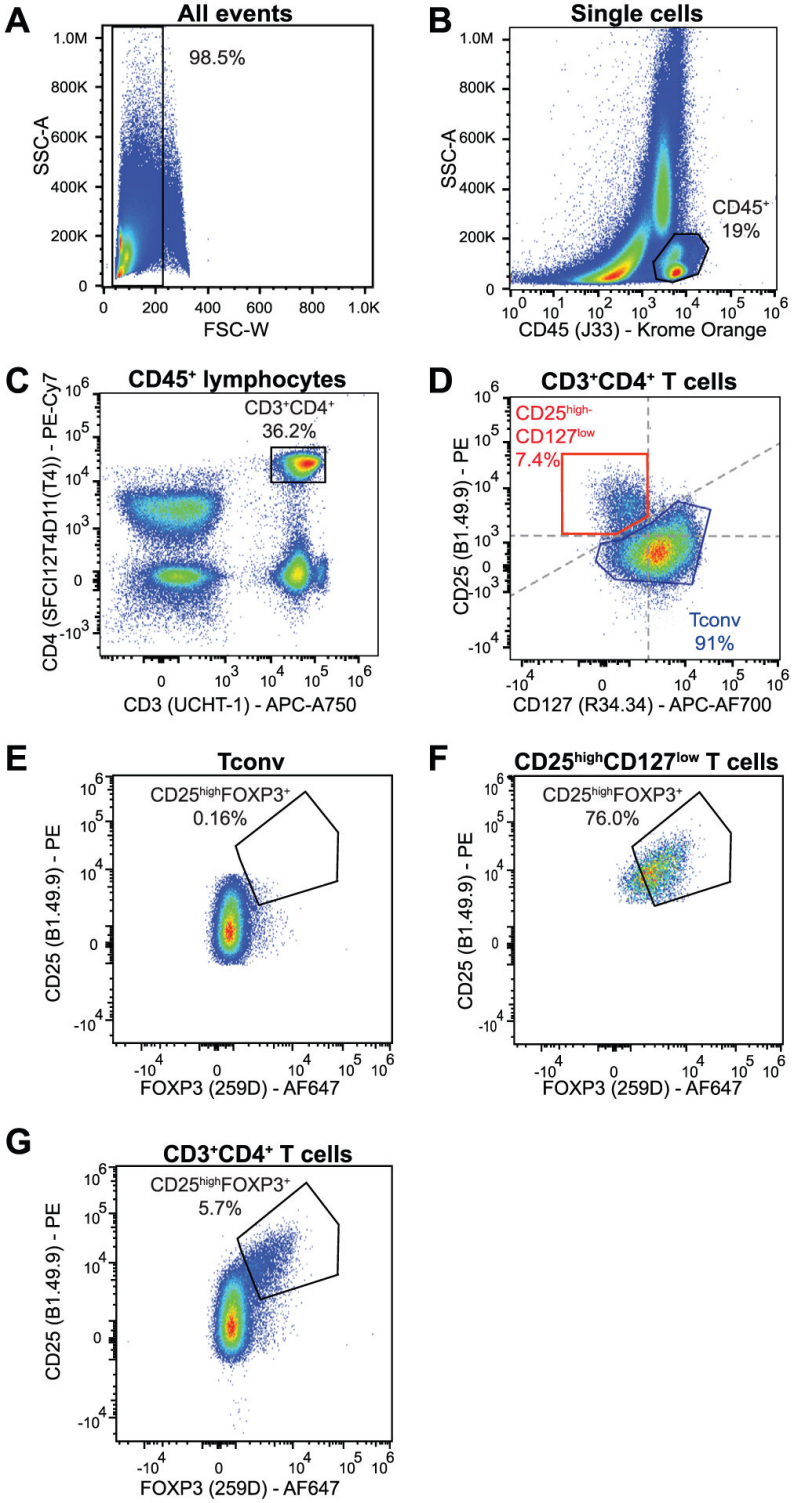
Gating strategy to quantify human CD25^hi^CD127^lo^FOXP3^+^ Tregs using whole blood and DuraClone tubes. (A-C) From total events, single cells were selected and CD45^+^ lymphocytes were gated based on SSC properties and CD45 expression. (D) From CD3^+^CD4^+^ T cells the CD25^hi^CD127^lo^ gate was identified. If the CD25 resolution is adequate then typically there is a clear separation of this population on a diagonal axis indicated by the grey dashed line. (E and F) show the expression of FOXP3^+^ within the indicated CD25^hi^CD127^lo^ or Tconv cells gates. (G) Identification of CD25^hi^FOXP3^+^ Tregs from total CD3^+^CD4^+^ T cells (panel C). Data were collected on a BD Fortessa X20 cytometer (Table 34).

Beckman Coulter DuraClone tubes (Figure 31) are precoated with dried down Ab cocktails, thus reducing pipetting time, and increasing reproducibility because there is no variation introduced by day-to-day mixing of wet Ab cocktails. The use of these reagents is an ideal way to standardize the flow cytometry of longitudinally samples collected in multi-site clinical trials [393]. Many Beckman-Coulter Abs are designed for clinical use so they have low lot-to-lot variation and are thus ideal for use as drop-in Abs with DuraClone tubes (providing fluorochrome brightness, clone affinity, etc. is acceptable). For optimal results with these tubes cytometers must be calibrated with standardized beads to maintain target voltages over time.

###### 11A.3.1.1 Surface and intracellular staining.

1. Add 100 μL of whole blood to the DuraClone Treg tube (Beckman Coulter, #B53346, see Table 22) and vortex immediately. Add any extracellular drop in Abs at this step (e.g., we drop in 5 μL of CD127 APC-AF700, Beckman Coulter, #A71116).
2. Incubate for 15 min at room temperature (RT) in the dark.
3. Wash with 3 mL of PBS.
4. Remove the supernatant with a 1 mL pipette followed by a 200 μL pipette.
5. Adjust volume to exactly 100 μL with FBS.
6. Add 10 μL of PerFix-nc reagent buffer 1 (Fixing buffer – Beckman Coulter, #B31164).
7. Incubate for 15 min in the dark.
8. Add 800 μL of PerFix-nc Buffer 2 (Permeabilization buffer – Beckman Coulter, #B31165).
9. Incubate for 15 min at RT in the dark.
10. Centrifuge for 3 min 500 × g and remove only the top 400 μL of buffer with a 1 mL pipette.
11. Transfer contents from original to Treg Tube 2 and vortex at high speed for 2 × 4 s.
12. Incubate at RT for 60 min in the dark.
13. Wash with 3 mL of PBS, vortex, and incubate at RT for 5 min.
14. Centrifuge at 500 × g for 5 min at RT.
15. Decant in one smooth motion and gently blot tube.
16. Vortex for 8 s.
17. Resuspend the cell pellet in 3 mL of 1× perFix nc buffer 3 (Beckman Coulter, #B31166) and vortex.
18. Centrifuge at 500 × g for 5 min at RT.
19. Decant supernatant in one smooth motion and gently blot tube.
20. Vortex the cell pellet for 8 s.
21. Add 350 μL of 1× Perfix-nc Buffer 3 (final volume should be 400 μL as residual volume will be around 50 μL) for data acquisition.

#### 11A.4 Materials

See section 11.4 below.

#### 11A.5 Data analysis

From total events, doublets were excluded and CD45^+^ lymphocytes were gated based on SSC properties and CD45 expression (Figure 31A and B). CD3^+^CD4^+^ T cells were then gated, followed by CD25^hi^CD127^lo^ cells (Figure 31C and D). If the CD25 resolution is adequate then typically there is a clear separation of this population on a diagonal axis indicated by the grey dashed line (Figure 31D). The remainder of the cells in the CD3^+^CD4^+^ gate were classified as Tconvs (Figure 31D). The FOXP3 gate is made on the basis of FOXP3 and CD25 expression in Tconvs (gated as CD25^−^ cell); i.e., the gate should be set such that there are very few FOXP3^+^ cells in the Tconv population (Figure 31E). This FOXP3 gate is then applied to the CD25^hi^CD127^−^ cells (Figure 31F). If CD127 is not used, then Tregs can be identified as CD25^hi^FOXP3^+^ cells (Figure 31G), but this population is harder to clearly discriminate.

#### 11A.6 Pitfalls

- Ensure that the volume is exactly 100 μL before the fix-perm buffers are added for optimal transcription factor staining. The Beckman SOP suggests using 50 μL of whole blood but the reagents also work well with 100 μL of blood. Use of more blood allows collection of more cells and thus better quantification of rare populations.

#### 11A.7 Top tricks

- In step 1, other markers of interest can be dropped in as wet Abs (e.g., CD127- APC-AF700).
- Dropping in anti-CD25 M-A251 PE improves CD25 resolution.

#### 11B.3 Step-by-step sample preparation: Staining Tregs from unmanipulated whole blood protocol 1B

##### 11B.3.1 Staining CD25^hi^CD127^lo^FOXP3^+^ Tregs using Ab cocktails.

**Figure 32.**
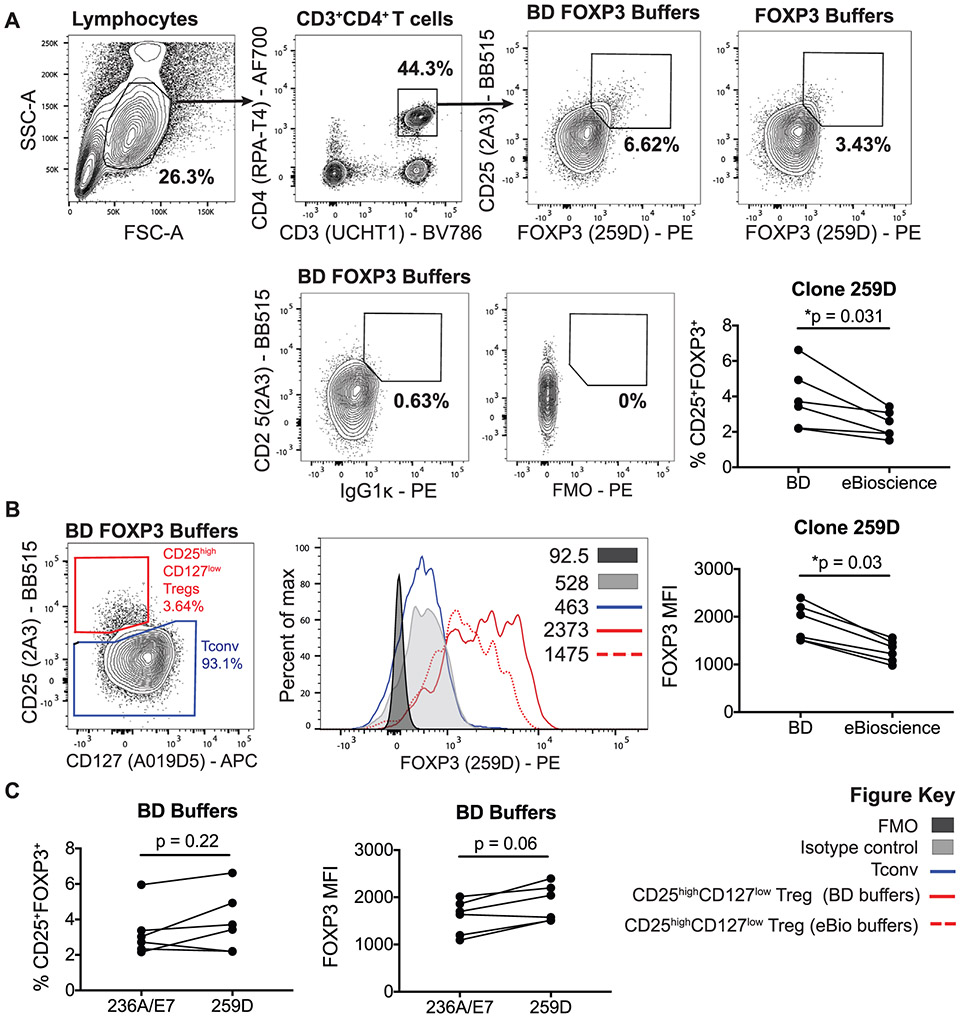
Phenotyping human CD25^hi^CD127^lo^FOXP3^+^ Tregs in whole blood. Representative staining of healthy adult peripheral whole blood with the Ab panel listed in Table 23. (A) Gating strategy and representative data for CD25^hi^FOXP3^+^ staining following fixation and permeabilization with either BD or eBioscience FOXP3 buffer kits. Gates were set on the basis of an isotype control (for comparison the lack of utility of an FMO control for setting the FOXP3 gate is shown). (B) Representative data for CD25^hi^CD127^lo^ staining and FOXP3 MFI with the indicated gated populations of CD25^hi^CD127^lo^ or Tconv cells. Right graph shows the FOXP3 MFI if samples are processed with BD or eBioscience buffers. (C) CD25^hi^FOXP3^+^ frequencies and FOXP3 MFI in CD25^hi^CD127^lo^ cells if staining is performed with the 236A/E7 or 259D anti-FOXP3 mAbs. All graphs show data from 6 healthy adults. Wilcoxon signed-rank tests were performed on paired samples. Data were collected on a BD Fortessa X20 cytometer (see Table 34).

This protocol was optimized by carrying out comparisons between the BD FOXP3 Buffer Kit (#560098) and the Thermo Fisher eBioscience FOXP3 Fix/Perm Buffer Set (#00-5523-00), as well as the anti-FOXP3 259D and 236A/E7 mAb clones (Figure 32), as per Table 23.

1. Aliquot whole blood (up to 500 μL) into 5 mL round bottom tubes.
2. Add extracellular Abs and stain for 15 min at RT.
3. Lyse red blood cells by adding 2 mL of BD FACSLyse solution (10× concentrate diluted to 1× stock in distilled water). Vortex to mix and incubate for 10–15 min at RT.
4. Add 2 mL staining buffer to wash and centrifuge at 500 × *g* for 5 min.
5. Transfer cell pellet (usually up to 10^6^ cells per condition) into a V-bottom 96-well plate.
6. Add 200 μL of Buffer A solution from BD Human FOXP3 buffer kit (10× buffer A concentrate diluted to 1× stock in distilled water) and incubate for 10 min at RT.
7. Centrifuge plate at 1000 × *g* for 3 min, flick off supernatant, and blot to dry. It is important that the cell pellet is as dry as possible.
8. Add 100 μL of Buffer C solution from the BD Human FOXP3 buffer kit (50× buffer B concentrate diluted to 1× stock in Buffer A) for at least 45 min in the dark at RT.
9. Add 100 μL of PBS and spin at 1000 × *g* for 3 min, flick off supernatant.
10. Repeat step 9 as residual Buffer C can interfere with Ab staining.
11. Add intracellular Abs to cells and incubate for 30 min in the dark at RT.
12. Wash cells in 100 μL of staining buffer, spin at 1000 × *g* for 3 min, flick off supernatant.
13. Resuspend in at least 150 μL of staining buffer for data acquisition.

#### 11B.4 Materials

See section 11.4 below.

#### 11B.5 Data analysis

Optimal staining of FOXP3 requires efficient fixation and permeabilization. Here, two commercially available FOXP3 staining buffer kits were compared. Cells in whole blood were stained as described above using either BD or eBioscience FOXP3 staining buffers. FOXP3 and CD25 staining in total CD3^+^CD4^+^ T cells was analyzed and the percent of cells which were CD25^hi^FOXP3^+^ was determined using a gate set on cells stained with an isotype control (Figure 32A). The data show that in this case there is better resolution of FOXP3 when cells are prepared with BD buffers compared to eBioscience buffers (Figure 32B). No difference in FOXP3 staining was observed between the 236A/E7 or 259D anti-human FOXP3 Ab clones (Figure 32C).

#### 11B.6 Pitfalls

- Insufficient removal of residual buffer A will result in less effective permeabilization in buffer B. If staining in a 96-well V bottom plate is not possible, then use a tube with a conical (not round) bottom tube to aid in removing all residual buffer after centrifugation steps
- Buffer B deteriorates with exposure to light so always make fresh (i.e., on the same day) working stocks of FOXP3 buffers.

#### 11B.7 Top tricks

- Human FOXP3 buffer set (BD, #560098) is superior to FOXP3 Fix/Perm Buffer Set (Thermo Fisher eBioscience, #00-5523-00) for FOXP3 staining when whole blood is used.
- An alternate to step 2 is to add all extracellular and intracellular Abs together in step 10 (provided extracellular Ab epitopes are not affected by FOXP3 buffers; this must be determined empirically).

#### 11C.3 Step-by-step sample preparation: Staining Tregs from unmanipulated whole blood protocol 1C

##### 11C.3.1 Determination of CD25^hi^CD127^lo^ Treg absolute counts using BD Trucount tubes, supplemented with drop in Abs.

**Figure 33.**
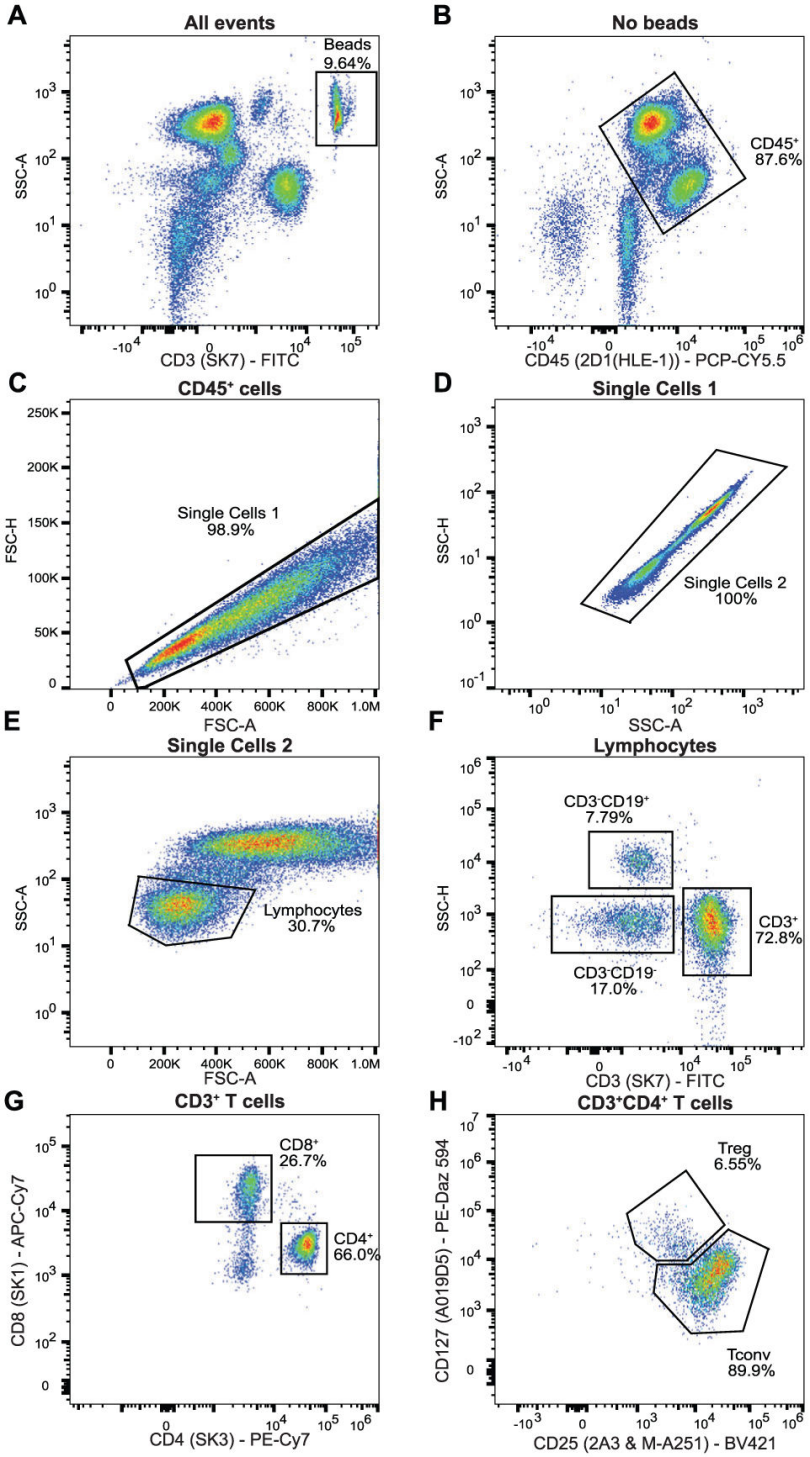
Quantification of human CD25^hh^CD127^lo^ Tregs using whole blood. (A) Count beads were gated based on SSC properties and CD3 expression. (B-E) After the exclusion of the beads, CD45^+^ whole blood cells were selected, doublet cells were excluded, and total lymphocytes were gated based on SSC and FSC properties. (F-H) From CD3^+^ T cells, CD4^+^CD8^−^ T cells were selected. Within the latter gate, CD25^+^CD127^lo^ Tregs and T conventional cells were identified. The Trucount tubes contain a number of beads that is used to calculate the absolute counts of the Tregs per μL based on the equation: (Number of positive Treg events/Number of bead events) *(Number of beads per tube/Test blood volume). Data were collected on a BD Fortessa X20 cytometer (see Table 34).

As cells can be lost during wash and centrifugation steps involved in routine flow cytometry protocols, the use of a lyse-no-wash procedure (LNW) is optimal to accurately determine the absolute count of leucocyte populations (Figure 33) [399]. However, as LNW procedures preclude fixing and permeabilization, in this protocol, Tregs can only be identified using CD25 and CD127. The protocol is based on combining of BD Trucount tubes and a six color TBNK Ab cocktail (commonly used for leucocyte enumeration in clinical immunology laboratories) with drop in Abs to identify Tregs as listed in Table 24. This procedure can be used to enumerate both Tregs and other leucocyte populations (CD3, CD4, CD8, B cells, and NK cells).

1. Add the Abs listed in Table 24 to a Trucount tube (Cat number 340334).
2. Invert tube containing whole blood several times to ensure homogeneity.
3. Aliquot exactly 100 μL whole blood into the bottom of a Trucount tube and vortex. Adopting a sound pipetting technique (e.g., a low immersion depth of the pipette tip) and possibly reverse pipetting in this step is advisable to ensure accuracy.
4. Incubate for 15 min at RT in the dark.
5. Prepare 1× lysing solution by adding 900 μL distilled water to 100 μL BD 10× Lysing solution (Cat number 349202).
6. Once the 15-min incubation is complete, add 900 μL of diluted 1× lysing Buffer and vortex immediately*.
7. Incubate at RT for 15 min in the dark, vortex* halfway through incubation.
8. Vortex again* and leave on ice until acquisition.
9. Vortex thoroughly immediately before acquisition.

#### 11C.4 Materials

See section 11.4 below.

#### 11C.5 Data analysis

Count beads were gated based on SSC properties and CD3 expression (Figure 33A). After the exclusion of the beads, CD45^+^ whole blood cells were selected, doublet cells were excluded, and total lymphocytes were gated based on SSC and FSC properties (Figure 33B-E). From CD3^+^ T cells, CD4^+^CD8^−^ T cells were selected. Within the latter gate, CD25^hi^CD127^lo^ Tregs and Tconv cells were identified. The Trucount tubes contain a number of beads that is used to calculate the absolute counts of the Tregs per μL based on the equation: (Number of positive Treg events/Number of bead events) * (Number of beads per tube/Test blood volume).

#### IIC.6 Top tricks

- Efficient lysis of red blood cells is vital for optimal assay performance. Ensure tubes are vortexed vigorously at each step indicated*.
- Enumeration of Tregs is stable for up to 44 hours post staining.

#### 11D.3 Step-by-step sample preparation: Staining Th-like Treg subsets in PBMCs

**Figure 34.**
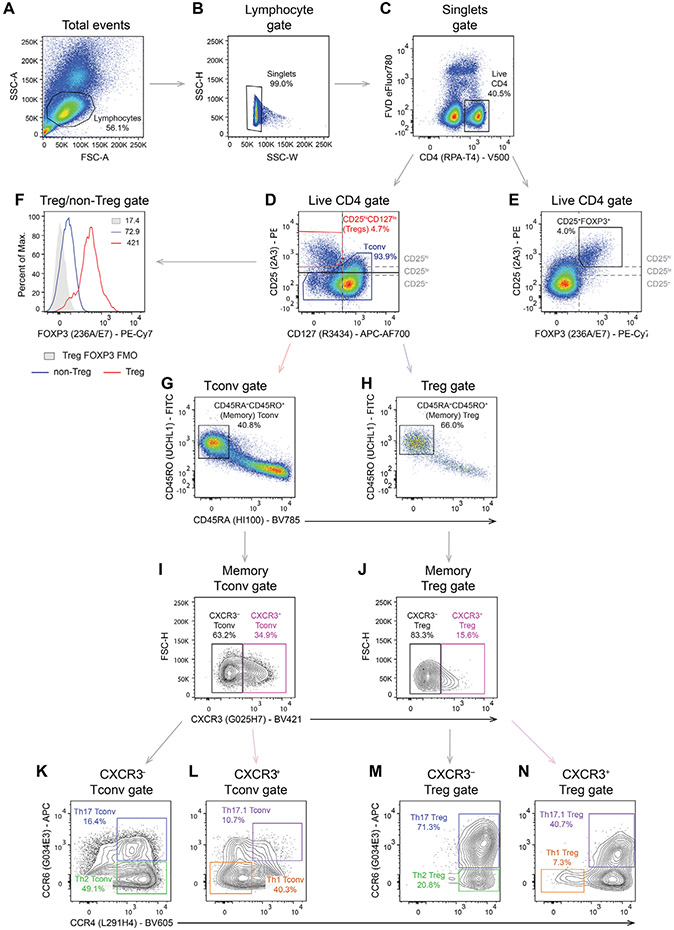
Identification of human Treg subsets in PBMCs. (A-C) Lymphocytes were gated according to their size and granularity, doublets excluded, and live CD4^+^ T cells gated. (D) Regulatory T cells (Treg) were identified as CD4^+^CD25^hi^CD127^lo^ (red gate) and the remaining cells were identified as “non-Treg” cells (blue gate). (E & F) If the cells are fixed and permeabilized, FOXP3 staining can be performed. In (E), Dashed lines show how CD25 negative, low and high expression are defined. In (F), FOXP3 expression in the CD4^+^CD25^hi^CD127^lo^ Tregs (red line) and non-Tregs (blue line) is shown, relative to a Treg FOXP3 fluorescence minus one (FMO) control (solid grey). Mean fluorescence intensity (MFI) values are provided. (G-H) Memory Tregs and non-Tregs were selected as CD45RA^−^CD45RO^+^. (I-N) Treg and non-Treg Th subsets were defined according to their expression of CXCR3, CCR4, and CCR6 as follows: Th17 (CXCR3^−^ CCR4^+^CCR6^+^), Th1 (CXCR3^+^CCR4^−^CCR6^−^), Th17.1 (CXCR3^+^CCR4^+^CCR6^+^), and Th2 (CXCR3^−^CCR4^+^CCR6^−^). Data were collected on a BD Fortessa X20 cytometer (see Table 34).

The following protocol details how to stain Th-like Treg subsets in PBMCs (isolated as previously described [393]). The gating strategy is in Figure 34 and the panel is detailed in Table 25. This gating strategy is based on the current literature [370, 400-403]. Additional markers such as CD161 [156], CRTH2 [149], and CCR10 [154] can also be added to further subcategorize the Th-like Treg subsets.

1. Transfer up to 10^5^ cells per condition into a V-bottom 96-well plate and wash in PBS.
2. Resuspend cells in PBS and add extracellular Abs and fixable viability dye (FVD).
3. Incubate for 30 min in the dark at 4°C.
4. Top up with staining buffer, spin at 350 × *g* for 5 min at RT, flick off supernatant, and blot dry.
5. Wash cells with Fix/Permeabilization solution (1:4 dilution of eBiosciencefix/perm concentrate (#00-5123) in fix/perm diluent (#00-5223)).
6. Add 100 μL Fix/Permeabilization solution and incubate either for 1 h at RT, or for optimal results, at 4°C overnight (NOTE: this is contrary to the manufacturer’s protocol).
7. Wash cells with 1× eBioscience permeabilization buffer (#00-8333; diluted with distilled water) and centrifuge at 1000 × *g* for 7 min at RT.
8. Flick off supernatant and blot dry. It is critical that the cell pellet is dry so that the subsequent step is optimal, see below.
9. Resuspend cells in 1× permeabilization buffer and add intracellular Abs.
10. Incubate for 40 minutes in the dark at 4°C.
11. Wash cells in 100 μL of permeabilisation buffer.
12. Wash cells in 100 μL of staining buffer.
13. Resuspend in at least 150 μL of staining buffer for data acquisition.

Note – this protocol can be modified to only carry out extracellular staining so that viable Th- like Tregs can be isolated. In this case it is highly recommended to first perform a CD25 pre- enrichment step, for example using Miltenyi Biotech’s human CD25 microbeads II (#130-092- 983). Note that this product blocks the epitope for the anti-CD25 2A3 mAb, so alternate mAb clones such as M-A251 or 4E3 are required for staining. See section IIE for further details.

#### 11D.4 Materials

See section 11.4 below.

#### 11D.5 Data analysis

Total lymphocytes were gated according to their forward and side scatter properties (Figure 34A), doublet events were excluded (Figure 34B) and live CD4^+^ T cells were gated (Figure 34C). Tregs can be identified from the total live CD4^+^ T cells according to their expression of CD25, CD127 and/or FOXP3 (Figure 34D and E). As previously mentioned, at least two of these markers should be used to defined human Tregs and where possible, the use of all three markers is ideal. In this analysis, Tregs were defined as CD4^+^CD25^hi^CD127^lo^ (Figure 34D) or CD4^+^CD25^hi^FOXP3^+^ (Figure 34E). As shown in Figure 34F, the majority of CD4^+^CD25^hi^CD127^lo^ cells are FOXP3^+^ but FOXP3^−^ cells still exist in this population, emphasizing the importance of using a combination of different markers to identify human Tregs. CD4 helper T cell and Treg subpopulations can be delineated from CD45RA^−^CD45RO^+^ memory cells (Figure 34G-H) by analyzing the expression of various homing receptors. Here, Th cell subsets were defined according to their expression of CXCR3, CCR4 and CCR6. Memory cells were separated according to their expression of CXCR3 (Figure 34I-J), after which CCR4 and CCR6 expression was used to subgate Th cell subsets (Figure 34K-N). Th cell subsets were defined as follows (Table 26): Th17 (CXCR3^−^CCR4^+^CCR6^+^), Th17.1 (CXCR3^+^CCR4^+^CCR6^+^), Th1 (CXCR3^+^CCR4^−^CCR6^−^), and Th2 (CXCR3^−^CCR4^+^CCR6^−^).

#### 11D.6 Pitfalls

- If the cell pellet is not effectively dried before the fixation or permeabilization steps, then fixation and intracellular staining are not optimal. Use a V-bottom plate to stain and blot plate dry after flicking off supernatant for best results. (Steps 5 and 8)

#### 11D.7 Top tricks

- Ensure Fixable Viability Dye is stained in PBS as the presence of FCS will inhibit staining.
- If the panel contains more than one fluorescent polymer dye conjugated Ab, then staining should be performed in Brilliant Stain Buffer Plus (BD, #566385) or Super Bright Complete Staining Buffer (ThermoFisher Scientific SB-4401-75).
- Make up FOXP3 buffers on the same day for each experiment.
- Wash with fix/perm before step 5 and wash with perm buffer before step 8.

#### 11E.3 Step-by-step sample preparation: Isolating Tregs from peripheral blood by cell sorting for cell culture

**Figure 35.**
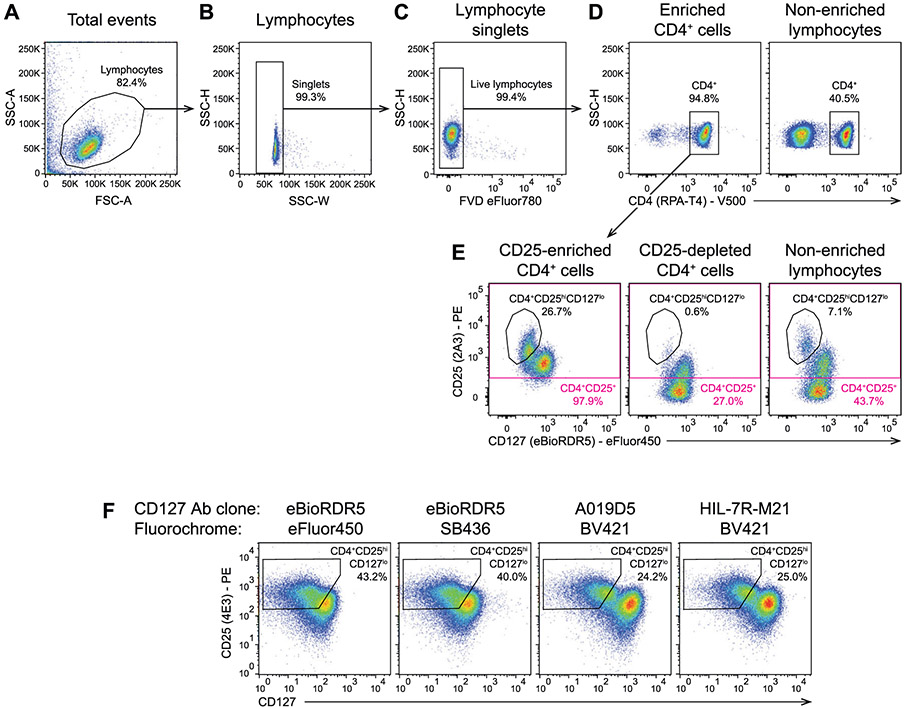
Gating strategy to sort CD4^+^CD25^hi^CD127^lo^ Tregs from human peripheral blood. Lymphocytes were gated according to their size and granularity (A), doublets excluded (B) and live cells were gated (C). (D) CD4^+^ T cells from an enriched population of cells were gated during the cell sort. CD4 staining of total lymphocytes is shown for reference. (E) CD25^hi^CD127^lo^ Tregs were gated from the CD4^+^ gate. In this representative plot, cells were enriched using the STEMCELL Technologies CD25-enrichment kit and subsequently stained with the CD25 (clone 2A3) PE Ab. CD25 and CD127 staining in CD25-depleted cells and non-enriched total lymphocytes are shown for reference. Data for panels A-E were collected on an LSR Fortessa X20. (F) CD25-enriched cells (Miltenyi kit) were stained with CD25 (clone 4E3) PE and one of four different CD127 Abs. Data were collected on a Beckman Coulter Moflo Astrios cell sorter (see Table 35).

As Tregs represent a small proportion of total circulating CD4^+^ T cells in healthy adults, it is advantageous to enrich these cells prior to cell sorting. Pre-enrichment of CD4^+^CD25^+^ cells reduces the time required to sort Tregs, which enhances viability post-sort and limits the quantity of Abs required to stain the cells. The following protocols describe how to enrich CD4^+^ T cells from peripheral blood by negative selection and subsequently enrich CD25^+^ cells by positive selection (Figure 35). We provide two CD25-enrichment protocols that use kits available from different manufacturers (Miltenyi and STEMCELL Technologies). For sorting CD4^+^CD25^hi^CD127^lo^ Tregs, we provide a comparison of four different anti-CD127 Abs and demonstrate how using certain Abs can help to accurately gate CD4^+^CD25^hi^CD127^lo^ Tregs (see Table 27; Figure 35F).

##### 11E.3.1 Enriching CD4^+^ T cells from peripheral blood (Figure 35A-D).

1. Add RosetteSep^™^ Human CD4^+^ T Cell Enrichment Cocktail (STEMCELL Technologies, # 15022) to peripheral whole blood at a concentration of 50 μL RosetteSep^™^ per mL blood*.
2. Mix and incubate for 20 min at room temperature.
3. Add an equal volume of PBS and mix.
4. Layer diluted blood onto lymphoprep and centrifuge at 600 × *g* for 20 minutes (no brakes).
5. Harvest enriched CD4^+^ T cells from the buffy coat layer and transfer to a fresh tube.
6. Wash cells with PBS, centrifuge (450 × g) and discard supernatant.
7. If red blood cells (RBCs) are visible, resuspend the cell pellet in Ammonium Chloride Solution (STEMCELL Technologies) and incubate for 5 min at room temperature. If RBCs cannot be seen in the cell pellet, skip this step and proceed to step 8.
8. Wash cells with PBS, centrifuge (120 × *g*) and discard supernatant.
9. Resuspend cells in PBS, count, centrifuge (450 × *g*) and discard supernatant.
10. Proceed to enrich CD25^+^ cells (section 11E.3.2 or 11E.3.3).

*Although RosetteSep^™^ has been optimised for use with whole blood, this kit can be used with alternative blood sources (e.g., buffy coats and leukapheresis products). The quantity of RosetteSep^™^ required for other sources should be optimised by the user.

Note – this protocol describes how to enrich CD4^+^ T cells by negative selection using RosetteSep^™^. Kits from alternative manufacturers may be used to isolate CD4^+^ T cells but positive selection kits that use magnetic beads should be avoided to prevent interference with the subsequent CD25^+^ cell enrichment.

##### 11E.3.2 Enriching CD25^+^ cells from CD4^+^ T cells (Miltenyi protocol).

1. Resuspend enriched CD4^+^ T cells in cold (4°C) MACS buffer (PBS with 1% Fetal Bovine Serum and 2 mM EDTA) at a concentration of 10^7^ cells per 90 μL MACS buffer.
2. Add 10 μL CD25 Microbeads II (Miltenyi, # 130-092-983) per 90 μL cells, mix and incubate at 4°C for 15 min.
3. Top up with cold MACS buffer, centrifuge (450 × *g*) and discard supernatant.
4. During the centrifuge in step 3, place an LS column* (Miltenyi, # 130-042-401) onto a MACS Separator magnet (e.g., Miltenyi, # 130-042-501) and add 3 mL MACS buffer to rinse the column. Do not let the column dry out.
5. Resuspend cells in 3 mL cold MACS buffer and when the LS column from step 4 has drained, add the cells and collect the flow-through. This flow-through contains the CD25^−^ fraction of cells.
6. Once the cells have finished draining, add another 3 mL cold MACS buffer to the column and collect the flow-through in the tube containing CD25^−^ cells**.
7. Once the MACS buffer has finished draining, remove the column from the magnet, place in a fresh tube, add 5 mL cold MACS buffer and use the plunger to flush out the CD25^+^ cells from the column.
8. Remove the plunger from the column, add another 5 mL MACS buffer to the column and use the plunger to flush out any remaining CD25^+^ cells from the column.
9. Centrifuge cells (450 × g), resuspend in PBS, count, and proceed to stain cells (section 11E.3.4).

*LS columns are recommended if the number of enriched CD4^+^ T cells used is >2×10^8^ and <2×10^10^. Manufacturers protocols should be consulted if the number of cells obtained falls outside this range.

**In this protocol, the MACS column is washed fewer times than is recommended by Miltenyi. In our experience, this has a minor effect on purity of the CD25-enriched cell product that is inconsequential if the cells are subsequently isolated by cell sorting.

Note – CD25 microbeads II block the 2A3 and BC96 epitopes of CD25. If using this Miltenyi CD25-enrichment protocol, cells must be stained with an alternate Ab clone, such as M-A251 or 4E3.

##### 11E.3.3 Enriching CD25^+^ cells from CD4^+^ T cells (STEMCELL Technologies protocol) (Figure 35E).

1. Resuspend enriched CD4^+^ T cells (section 11E.3.1) in cold (4°C) EasySep^™^ Buffer (PBS with 2% Fetal Bovine Serum and 2 mM EDTA) at a concentration of 10^8^ cells/mL and transfer to a 5 mL polystyrene round-bottom tube.
2. Add 50 μL Pan-CD25 Positive Selection and Depletion Cocktail (STEMCELL Technologies, # 17861) per mL cells, mix and incubate for 10 min at room temperature.
3. Vortex RapidSpheres^™^ (STEMCELL Technologies, # 17861) for 30 s, add 100 μL RapidSpheres^™^ per mL cells and incubate for 3 min at room temperature
4. Top up tube to 2.5 mL with EasySep^™^ Buffer and place in an EasySep^™^ magnet (STEMCELL Technologies, # 18000) for 3 min.
5. Pick up magnet and gently pour off supernatant. This supernatant contains the CD25^−^ fraction of cells.
6. Remove the tube from the magnet, resuspend the cells in 2.5 mL EasySep^™^ Buffer, place the tube back in the magnet and incubate for 3 min.
7. Repeat steps 5 and 6 three more times (four pours in total)*
8. Centrifuge cells (450 × *g*), resuspend in PBS, count and proceed to stain cells (section 11E.3.4).

*This is the number of washes recommended by STEMCELL Technologies. Unlike the Miltenyi CD25-enrichment approach, we suggest that the total number of recommended washes be performed as each of these washes has a dramatic effect on the CD25-enrichment efficiency. In our experience, the number of resulting cells will be almost halved by performing the recommended four washes instead of just two.

Note – the Pan-CD25 Positive Selection and Depletion Cocktail blocks the 4E3 and M-A251 epitopes of CD25. If using this CD25-enrichment protocol, cells must be stained with an alternate Ab clone, such as 2A3 or BC96.

##### 11E.3.4 Staining enriched CD4^+^CD25^+^ T cells for cell sorting.

1. Wash cells (from sections 11E.3.2 and 11E.3.3) with PBS and resuspend in PBS at a concentration of 200x10^6^ cells/mL.
2. Add Abs and fixable viability dye (FVD). Ensure that the CD25 Ab clone used is compatible with the CD25-enrichment protocol used (See “Notes” above)
3. Incubate for 30 min in the dark at 4°C
4. Top up with staining buffer, spin at 350 × *g* for 5 min at RT, remove supernatant and resuspend in staining buffer at 20 × 10^6^ cells/mL
5. Pass cells through a 70 μm cell strainer and store in the dark on ice until ready to sort.

#### 11E.4 Materials

See section 11.4 below.

#### 11E.5 Data analysis

Total lymphocytes were gated according to their forward and side scatter properties (Figure 35A), doublet events were excluded (Figure 35B) and FVD^−^ live cells were gated (Figure 35C), after which CD4^+^ T cells were gated (Figure 35D). Total lymphocytes that were not enriched for CD4 expression are shown for reference (Figure 35D). From the live CD4^+^ T cells, Tregs were gated according to their high expression of CD25 and low expression of CD127 (Figure 35E). CD25-depleted CD4^+^ T cells and CD4^+^ T cells that were not CD25-enriched are shown for reference (Figure 35E).

Figure 35F shows data that were collected on a Beckman Coulter Moflo Astrios cell sorter (Table 34; see below). These cells were stained with FVD eF780, CD4 V500 (RPA-T4), CD25 PE (4E3) and one of four different anti-CD127 Abs (Table 27). For this machine, using an eBioRDR5 clone anti-CD127 Ab conjugated to eFluor450 did not provide a good resolution for gating CD4^+^CD25^hi^CD127^lo^ Tregs, despite a reasonable resolution being achieved with this Ab when data were acquired on an LSR Fortessa X20 (Figure 35E). This issue was not resolved by changing the fluorochrome to Super Bright (SB)436. A better resolution was observed when the cells were stained with alternative Ab clones conjugated to BV421. We recommend that Abs used to stain CD25 and CD127 are both conjugated to bright fluorochromes and that different Ab clones and fluorochrome conjugates be tested by users for specific cell sorters.

#### 11E.6 Pitfalls

- Poor staining for CD25 will be observed if the CD25-enrichment approach is not compatible with the CD25 Ab clone chosen. See “Notes” under each CD25-enrichment protocol for more details.
- A conservative gating strategy should be used to gate CD4^+^CD25^hi^CD127^lD^ Tregs. Setting a gate that is too generous will increase the chances of sorting activated Tconvs. One can further reduce the chances of sorting activated Tconvs by also selecting naïve cells (CD45RA^+^CD45RO^−^) during the sort, should memory Tregs not be required.

#### 11E.7 Top tricks

- If you use RosetteSep^™^ to enrich CD4^+^ T cells from blood sources that are not whole blood, ensure that there are sufficient RBCs to allow the kit to function properly. A minimum of 100 RBCs per nucleated cell is required to efficiently deplete contaminating cells.
- Contaminating RBCs should be lysed before staining as these will appear as “debris” during the cell sort and complicate the gating process.
- For brightest staining ensure Fixable Viability Dye is stained in protein-free PBS as the presence of FBS can reduce staining intensity of dead population.
- If the panel contains more than one fluorescent polymer dye conjugated Ab, then staining should be performed in Brilliant Stain Buffer Plus (BD, #566385) or Super Bright Complete Staining Buffer (ThermoFisher Scientific SB-4401-75).
- Culture media for sorted cells should be supplemented with antibiotics.

#### 11F.3 Step-by-step sample preparation: Isolating CD25^+^FOXP3^+^ Tregs from human thymus

Paediatric thymus is an alternative source of Tregs with advantages including the number of Tregs that can be isolated per gram of tissue, the relative ease of isolating Tregs by magnetic selection (due to the absence of CD25-expressing activated CD4^+^ conventional T cells), and the relative homogeneity of Tregs that can be isolated compared to peripheral blood [388, 404]. For these reasons, the thymus is an attractive source of Tregs for clinical applications or for process development settings where it is desirable to test a large number of conditions in parallel using cells from the same donor. Below we detail a protocol to isolate and stain Tregs from human paediatric thymus tissue. A counterpart protocol for isolating and analyzing Tregs from mouse thymus tissue is provided in section 12A.

##### 11F.3.1 Isolation and analysis of Tregs isolated from human paediatric thymus (Figure 36).

1. Pediatric thymuses removed during cardiac surgery are collected in UW solution (Bridge to Life) and placed on ice. Thymocytes or thymus-derived Tregs are isolated within 24 h of collection, following our published protocol [404], as detailed below.
2. Thymus tissue is cut into pieces of ~3 g, which are dissociated manually using a McIlwain tissue chopper (Campden Instruments Ltd., Loughborough, England) or by using the gentleMACS^™^ Dissociator (Miltenyi Biotec, Bergisch Gladbach, Germany).
3. For tissue dissociation using the gentleMACS^™^, the ~3 g piece of tissue is transferred to a gentleMACS^™^ C tube with 5–10 mL of ImmunoCult-XF T Cell Expansion Medium (STEMCELL Technologies). The tissue is dissociated by running the m_spleen_01.0.1 C program twice.
4. After dissociation, the cell suspension is passed through a 100 μm cell strainer. Cells are washed once with ImmunoCult-XF T Cell Expansion Medium, passing the cell suspension through a 70 μm cell strainer.
5. To isolate Tregs, cells are washed once more with EasySep^™^ Buffer (PBS with 2% Fetal Bovine Serum and 2 mM EDTA), passing the cell suspension through a 70 μm cell strainer, then resuspended at 200 × 10^6^ cells/mL in EasySep^™^ Buffer for magnetic cell selection. Thymus-derived Tregs are isolated by CD25-positive selection using Releasable Rapidspheres, followed by CD8 depletion using custom isolation kits (STEMCELL Technologies).
6. Average expected yields are ~1×10^9^ thymocytes/g tissue and ~5-10×10^6^ CD25^+^CD8^−^ cells isolated from 2.4×10^9^ thymocytes.
7. Staining of thymocytes and isolated Tregs is performed using the PBMC staining protocol (see section 11D) and the panel outlined in Table 28.

#### 11F.4 Materials

See section 11.4 below.

#### 11F.5 Data analysis

**Figure 36.**
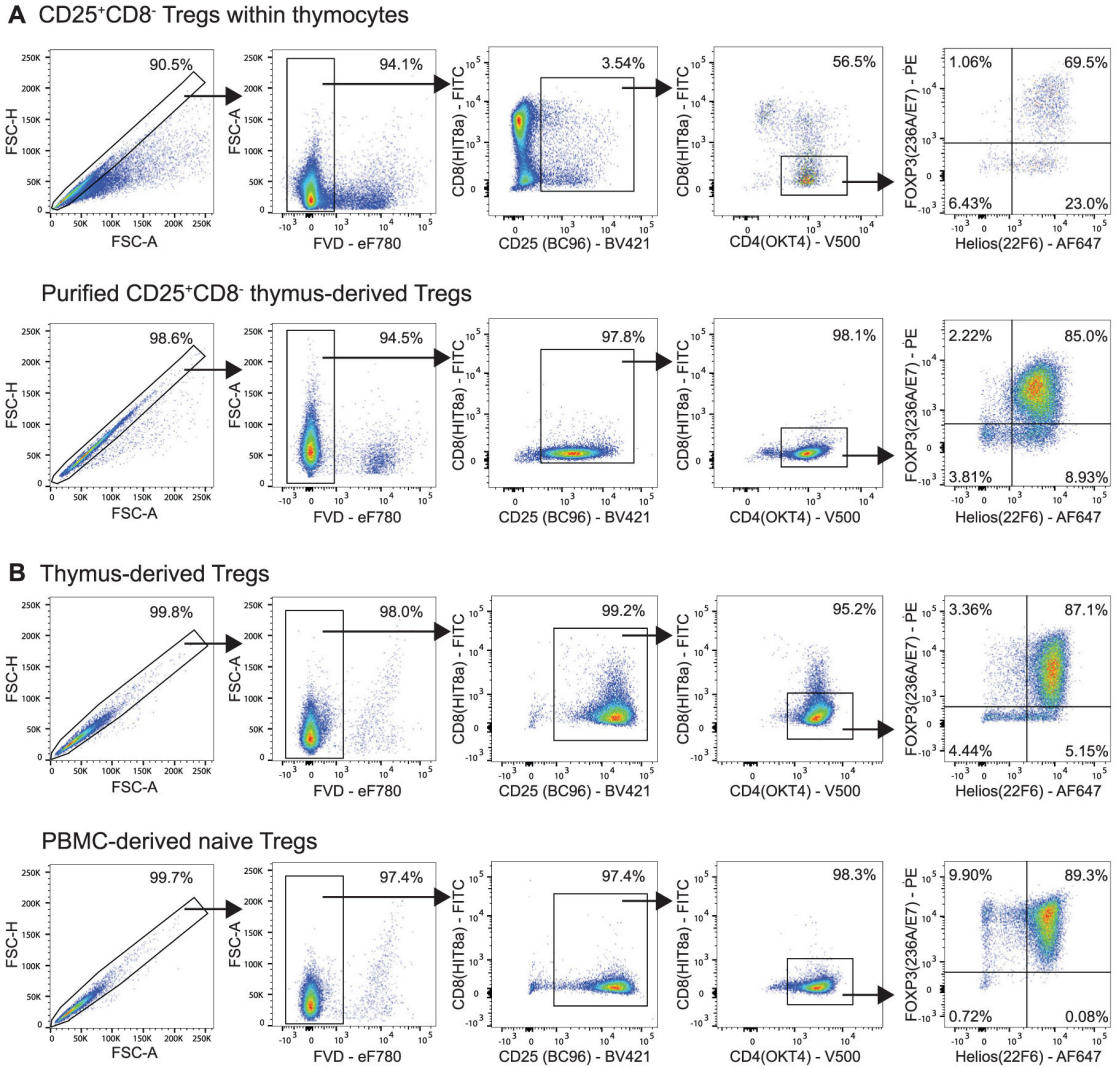
Gating strategy to identify CD25^+^FOXP3^+^ Tregs in human thymus. (A) Representative Treg staining from total thymocytes and purified CD25^+^CD8^−^ thymus-derived Tregs *ex vivo*, and (B) from thymus-derived Tregs and peripheral blood-derived naïve Tregs after 7 days of *in vitro* expansion. From total events, doublets were excluded based on FSC-H and FSC-A, then live cells were selected based on negative expression of FVD. CD25^+^ cells were gated from live cells, then CD4^+^CD8^−^ T cells were gated from the CD25^+^ gate. From the CD4^+^CD8^−^ T cell gate, the expression of FOXP3 and Helios are shown. Data were collected on a BD Fortessa X20 cytometer (see Table 34).

From total events, doublets were excluded based on FSC-H and FSC-A and live cells were selected based on negative expression of FVD. From live cells, CD25^+^ cells were identified, then Tregs were gated as CD4^+^CD8^−^. From the Treg gate, the expression of FOXP3 and Helios are shown (Figure 36A).

Given that CD25 expression precedes FOXP3 expression during Treg development in the thymus [405], ~20% of CD25^+^CD4^+^CD8^−^ cells in the thymus do not yet express FOXP3 upon isolation. These cells upregulate FOXP3 expression after TCR activation, such that the frequency of FOXP3^+^Helios^+^ cells isolated using this method is similar after 7 days of in vitro expansion to that of CD25^hi^CD127^−^CD45RA^+^ naïve Tregs expanded using the same method (Figure 36B).

#### 11F.6 Pitfalls

- Considerable cell clumping may be observed while washing thymocytes after tissue dissociation. Cells should be passed through a 100 μm or 70 μm filter after each centrifugation step to remove clumps.
- Expression of surface markers, such as CD25, can be affected by the use of digestive enzymes, so the use of enzymes should be avoided or all Ab clones (including isolation reagents) used should be tested for their sensitivity to digestion.
- Thymus tissue may be stored overnight at 4°C in UW solution before processing. Storing tissue samples at 4°C for longer than 24 h results in reduced viability of isolated thymocytes and Tregs.
- Magnetic selection reagents will block some CD25 or CD8 Ab clones. Use alternate CD25 clones, such as 2A3 or BC96, and alternate CD8 clones, such as RTF-8, OKT8, or HIT8a, when analyzing cells isolated by magnetic selection using these kits from STEMCELL Technologies.

#### 11F.7 Top tricks

- EasySep^™^ Buffer used for Treg isolation can be prepared using 0.5% human serum albumin to avoid using fetal bovine serum.
- At least 5 × 10^5^ thymocytes should be stained when using the PBMC staining protocol (see section 11D). If fewer cells are stained, many cells will be lost during centrifugation and supernatant removal steps.
- If staining Tregs isolated by magnetic selection with more than one fluorescent polymer dye conjugated Ab, then staining should be performed in Brilliant Stain Buffer Plus (BD, #566385) or Super Bright Complete Staining Buffer (ThermoFisher Scientific SB- 4401-75).

#### 11G.3 Step-by-step sample preparation: Staining CD25^hi^FOXP3^+^ Tregs from human intestinal biopsies

There is increasing interest in the role of tissue resident Tregs [406]. As discussed above, it is significantly more difficult to confidently identify Tregs in tissues versus blood because of the high proportion of activated Tconv cells. In addition, tissues must often be treated with collagenase which can lead to removal of Treg-associated cell surface proteins. Below we detail a protocol to stain Tregs in human intestinal biopsies and illustrate how the resulting data compare to that obtained with PBMCs. A counterpart protocol for isolating and analyzing Tregs from mouse colon tissue is provided in section 12G.

##### 11G.3.1 Isolation and analysis of lymphocytes from intestinal biopsies (Figure 37).

1. Intestinal biopsies (usually 4-6 punches ~ 4 mm in diameter) are collected in complete media at RT and lamina propria mononuclear cells (LPMCs) are isolated within 2-4 h of collection following the protocol described in [407].
2. Briefly, biopsy specimens are transferred to a 10 mL solution of Collagenase VIII and DNAse and incubated for 1 h at 37°C, shaking vigorously every 20 min. After incubation, cells are passed through a 100 μm cell strainer, washed and LPMCs are isolated by centrifugation over a Percoll gradient (resuspended in 40% Percoll, underlayed by an equal volume of 80% Percoll).
3. After washing, cells are resuspended in complete medium at a concentration of 2 × 10^6^ cells/mL and incubated overnight at 37°C before staining. Expected average yields are 2 × 10^6^ LPMCs per sample (if 4-6 biopsies are pooled together).
4. Staining of LPMCs is then performed using the PBMC staining protocol (see section 11D) using the panel outlined in Table 29.

#### 11G.4 Materials

See section 11.4 below.

#### 11G.5 Data analysis

From total events, doublets were excluded based on FSC-H and FSC-A and live CD4^+^ T cells were selected based on negative expression of FVD and positive expression of CD3 and CD4. From CD4^+^ T cells, Tregs were gated as CD25^hi^FOXP3^+^ cells. From the Treg gate, the expression of CD161 and Helios are shown. Dashed lines show how CD25 negative, low and high expression are defined. This strategy was used to gate Tregs based on CD25^hi^ and CD127^lo^ expression from PBMCs and LPMCs.

#### 11G.6 Pitfalls

- Many surface markers are affected by collagenase digestion so it is imperative to systematically test all Ab clones before finalizing the staining panel (Summary table of validated clones is shown in Table 30.)

#### 11G.7 Top tricks

- Ensure that the collagenase incubation is done in a 50 mL, rather than a 14 mL, falcon tube even if only using 10 mL of solution. The added room allows more vigorous shaking, which is key to getting a good yield.

#### 11H.3 Step-by-step sample preparation: Staining CD25^hi^FOXP3^+^ Tregs from human skin and fat tissue

This protocol describes how to isolate and analyze Tregs from human skin and fat. Both human skin and fat require collagenase-based digestion protocols, and tissue preparations need to be handled carefully to ensure cell viability and recovery. In general, the use of sharpened scissors or scalpels is recommended to avoid mechanical strain on the tissue. In addition, filtering and mashing steps should be performed without too much mechanical pressure to avoid damage to cells and release of cytosolic components or DNA into the digestion medium. Counterpart protocols for isolating and mouse Tregs in murine skin and fat tissues are provided in sections 12D and 12E, respectively.

##### 11H.3.1 Isolation and analysis of lymphocytes from human skin tissue (Figure 38C and D).

1. Human skin and underlying subcutaneous fat tissue are mechanically separated, followed by tissue-individual digestion and preparation techniques.
2. Skin is cleared from all subcutaneous fat tissue and weighed. We recommend using <1 g of skin per 10 mL of digestion buffer.
3. Skin is transferred into digestion buffer (Table 31) placed in a GentleMACS^™^ C tube. Using sharp scissors, skin is cut into small pieces.
4. Digestion is performed for 90 min in the GentleMACS^™^ using the program “37_C_Multi_H”.
5. When digesting manually, the sample is digested for 90 min on a rotating shaker in the incubator (37°C) or in a shaking water bath pre-heated to 37°C.
6. Optional: cut skin again after 45 min of digestion.
7. Place sample on a metal strainer located in a petri dish and use a syringe plunger to dissociate remaining tissue pieces.
8. Filter sample via a 100 μm filter unit.
9. Centrifuge for 5 min with 800 × *g* at RT.
10. Filter sample via a 70 μm filter unit.
11. Centrifuge for 5 min with 800 × *g* at RT.
12. If required: perform ACK lysis (Gibco #A1049201) to remove red blood cells.
13. Remove dead cells using a commercial dead cell removal kit to enrich target cells for sorting or analysis.
14. Stain cells according to the panel outlined in Table 32 (see below).

##### 11H.3.2 Isolation and analysis of lymphocytes from human fat tissue (Figure 38E).

1. Human skin and underlying subcutaneous fat tissue are mechanically separated, followed by tissue-individual digestion and preparation techniques.
2. Fat tissue is weighed. We recommend to use <5 g of fat per 10 mL of digestion buffer.
3. Fat is transferred into digestion buffer (Table 32) in a 50 mL conical tube. Using sharp scissors, skin is cut into small pieces.
4. Digestion is performed for 60-90 min on a rotating shaker in the incubator (37°C) or in a shaking water bath pre-heated to 37°C.
5. Optional: cut fat tissue again after 45 min of digestion
6. Filter sample via a 100 μm filter unit.
7. Centrifuge for 5 min with 500 × *g* at RT.
8. Filter sample via a 40 μm filter unit.
9. Centrifuge for 5 min with 500 × *g* at RT.
10. Perform ACK lysis (Gibco #A1049201) to remove red blood cell contamination.
11. Optional: if red blood cells remain after the first lysis, perform another ACK lysis.
12. Stain cells according to the panel outlined in Table 33.

#### 11H.4 Materials

See section 11.4 below.

#### 11H.5 Data analysis

The surface receptor CCR8 is vulnerable to fixation and permeabilization protocols, which are the prerequisite for intracellular protein measurements. Therefore, to perform intracellular staining of CCR8-Ab-stained cells in high quality and verify the expression of, e.g., FOXP3 and BATF, we performed a sorting-fixation-staining approach to determine the expression levels of FOXP3 and BATF in FOXP3^+^BATF^+^CCR8^+^ Treg cells isolated from blood (Figure 38A-B). To do so, we sorted blood-derived CD3^+^CD4^+^CD8^−^CD25^−^CD127^+^CD45RA^+^ naive Tconv, blood-derived CD3^+^CD4^+^CD8^−^CD25^+^CD127^−^CD45RA^+^ naive Treg, blood-derived CD3^+^CD4^+^CD8^−^CD25^+^CD127^−^CD45RA^−^CCR8^−^ and CCR8^+^ memory-type Treg. After sorting, cells were fixed, stained intracellularly with either FOXP3 (206D, Biolegend) and BATF (D7C5, Cell Signalling) or FOXP3 without primary BATF Ab (using FOXP3 buffer kit, eBioscience), followed by secondary intracellular staining and re-acquisition of fixed cells. All parameters were again recorded. From total events, fixed lymphocytes were gated.

Singlets were isolated based on FSC-H versus FSC-A, and live CD45^+^ hematopoietic cells were identified. Following staining of the T-cell receptor, CD4^+^ T cells were gated and Treg cells were identified by CD25^hi^ and CD127^lo^ expression. From Treg cells, naive and memory-like Treg cells were sub-gated using CD45RA, and CD45RA-negative memory Treg cells contained both CCR8^+^ and CCR8^−^ Treg cells. For those, expression of FOXP3 and BATF is shown (Figure 38B). In tissues, the gating is slightly modified (Figure 38C): first, cells of hematopoietic origin are pre-selected based on CD45 expression, followed by the same gating strategy as shown before. Then, Tregs are identified in human skin and fat tissue. To optimize CCR8 staining intensity, results for skin and fat tissue are based on unfixed cells isolated from fresh tissues.

#### 11H.6 Pitfalls

- Many surface markers are affected by collagenase digestion so it is imperative to systematically test all Ab clones before finalizing the staining panel.
- Filter clogging during filtration: Filter samples sequentially from 100 μm via 70 μm to 40 μm.
- GentleMACS^™^ mechanical failure: Cut the skin into very small pieces using sharp scissors. When using thick skin patches or large (>1 g) amounts of skin, re-cut skin tissues during incubation on the GentleMACS^™^ machine. Use more skin digestion buffer or distribute skin among more C tubes if required.
- It is recommended to use CD45 conjugated to BUV737 or another bright colour to clearly identify cells of hematopoietic origin in tissues.
- Poor sort purity or contamination with skin keratinocytes after sorting: Use a double-sort approach (yield followed by 4-way-purity, see ‘Top tricks’ below) to avoid contaminating your sample with unwanted cells. Use negative (“dump”) markers such as CD206 (expressed on macrophages) or Podoplanin (expressed on fibroblasts) to exclude unwanted cells.
- Machine clogging or no events: Wash machine and filter sample again and re-acquire. Keep the sample cool at 4°C and continuous rotation (300 rpm).
- Sorter clogging: Filter sample during acquisition if acquisition time >10 min.

#### 11H.7 Top tricks

- Ensure that the fat collagenase incubation is done in a 50 mL, rather than a 15 mL, falcon tube even if only using 10 mL of solution. The added room allows more vigorous shaking, which is key to getting a good yield.
- We were unable to perform pre-enrichment using magnetic beads for skin-based or fat-based samples. Still, because of the very low frequency of FOXP3^+^ Tregs as well as the diverse cell mixture in skin samples, enrichment would be beneficial to decrease staining and measurement time.
- Sorting total skin Tregs can lead to poor recovery of cells (low “sort efficiency”) and, based on the parameters of the sorting instrument, also to contamination with skin keratinocytes (aggregates with immune cells). Therefore, we propose a two-step sorting protocol: first, a pre-enrichment sort (sort strategy: "yield") where target cells are sorted into FACS buffer. Second, the sample is re-acquired and sorted again with high purity (sort strategy: "purity" or "4-way-purity"). Using this strategy, skin samples can be sorted at high speed with minimal loss of target cells.
- For flow cytometric analysis, samples should be filtered immediately before acquisition. If acquisition time is >10 min, the sample should be filtered again to avoid clogging of the instrument. Samples should be cooled at 4°C to avoid clogging.
- Fixing samples will generally increase the sample flow rate through cytometers due to reduced cell size. Be careful when setting your FSC/SSC voltages to include your target cells. Include a positive staining control (e.g., PBMCs) to validate the panel and Ab staining before acquiring skin or fat cells.

#### 11.4 Materials

##### 11.4.1 Subjects and samples.

**Figure 37.**
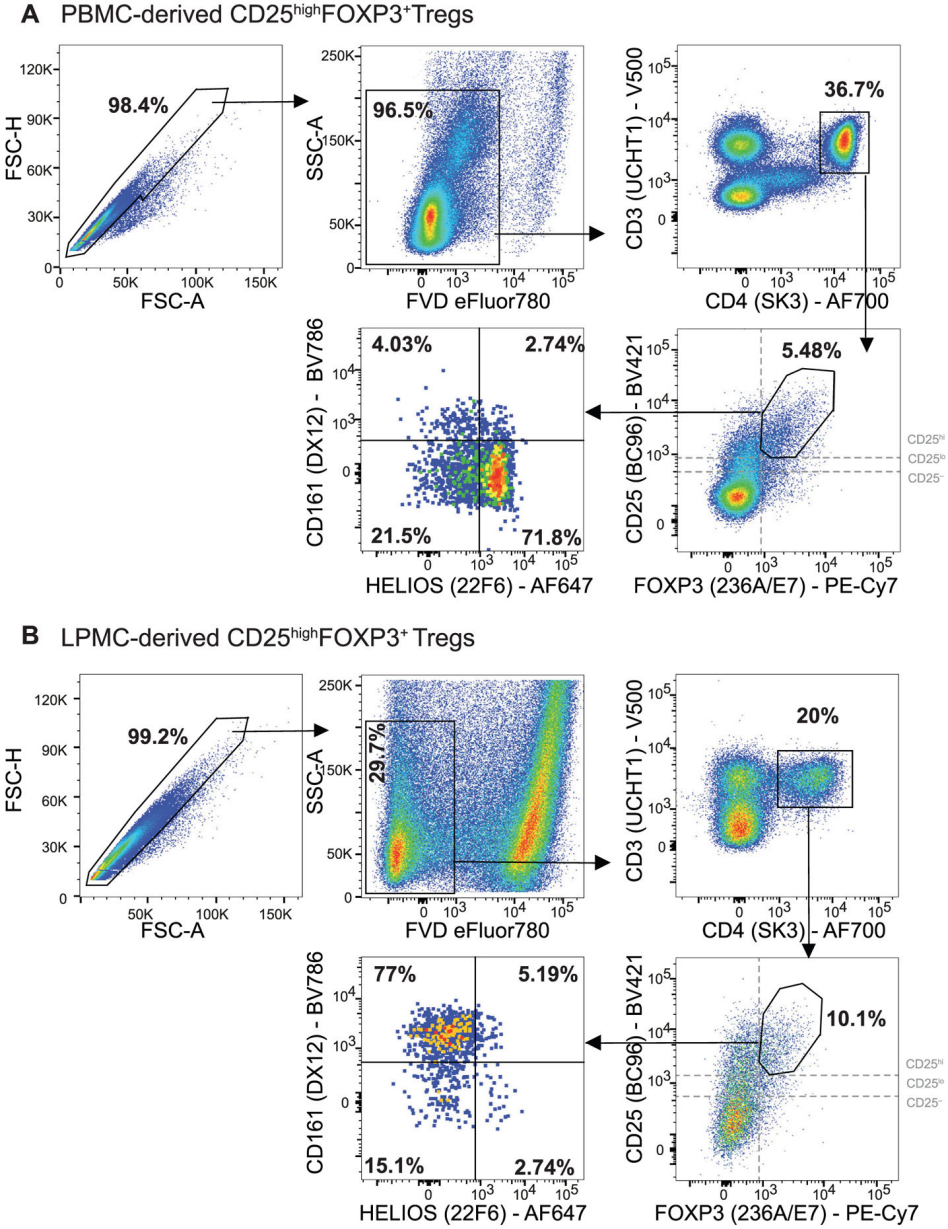
Gating strategy to identify CD25^hi^FOXP3^+^ Tregs in human intestinal biopsies. (A) Representative Tregs staining from PBMCs and (B) LPMCs. From total events, doublets were excluded based on FSC-H and FSC-A. Live cells were selected based on negative expression of FVD and CD4^+^ T cells were gated based on CD3 and CD4 expression. From CD4^+^ T cells, Tregs were gated as CD25^hi^FOXP3^+^ cells. From the Treg gate, the expression of CD161 and Helios are shown. Dashed lines show how CD25 negative, and high expression are defined. Data were collected on a BD Fortessa X20 cytometer (see Table 34).

**Figure 38.**
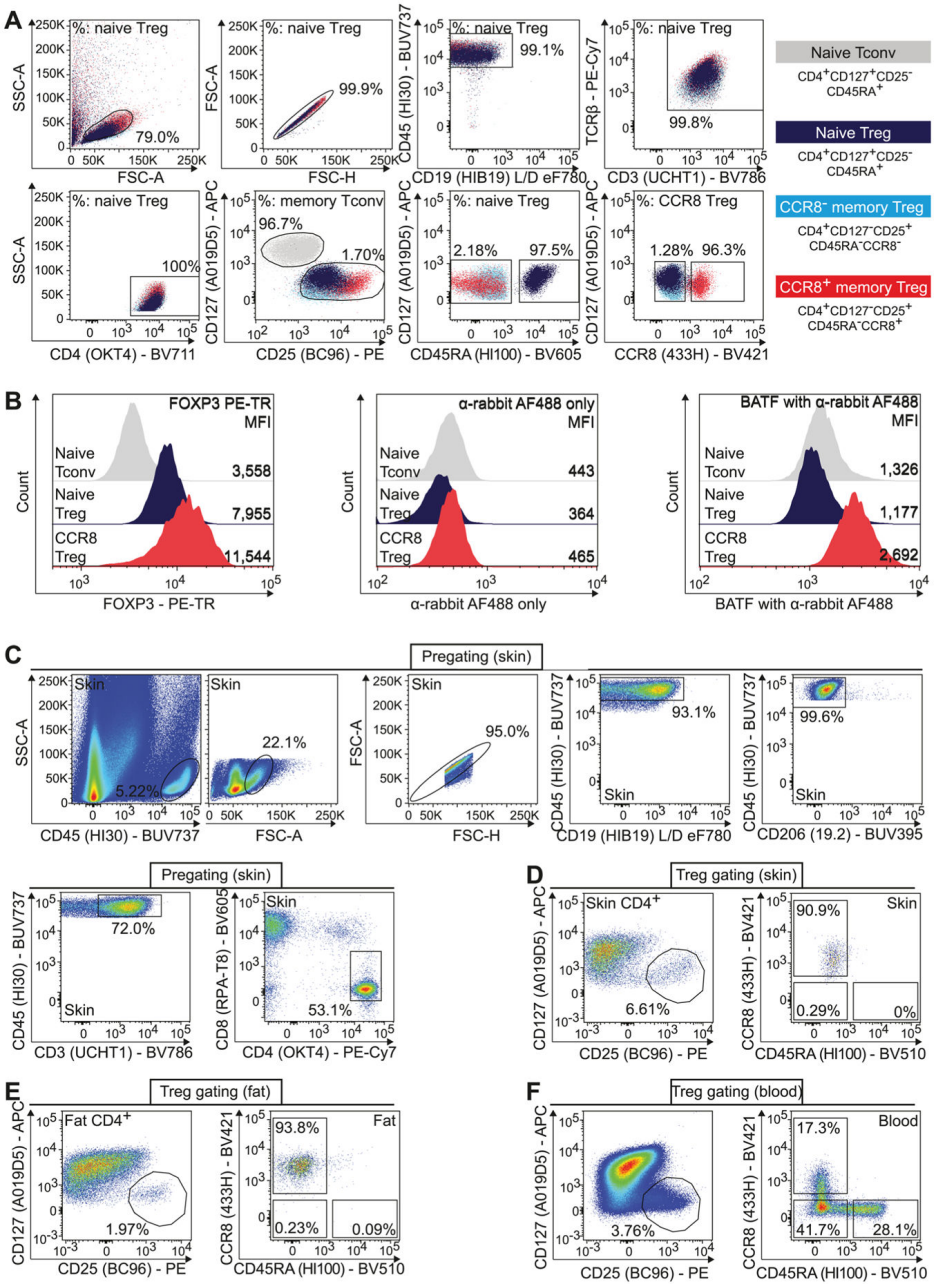
Gating strategy to identify CD25^hi^FOXP3^+^ Tregs in human skin and fat tissue. (A) Human blood-derived CD3^+^CD4^+^CD8^−^CD25^−^CD127^+^CD45RA^+^ naive Tconv, blood-derived CD3^+^CD4^+^CD8^+^CD25^+^CD127^−^CD45RA^+^ naive Treg, blood-derived CD3^+^CD4^+^CD8^−^CD25^+^CD127^−^CD45RA^−^CCR8^−^ and CCR8^+^ memory Treg were sorted, fixed, and stained intracellularly, followed by re-acquisition of fixed cells. Contribution of cell subtypes in the respective gates based on color code. (B) Expression of FOXP3 (left) and BATF (right), with BATF control staining (middle, no primary Ab). (C) Example gating strategy for human tissue Treg cells using skin tissue. (D) Identification of CD25^+^CD127^−^CD45RA^−^CCR8^+^ Treg cells in human skin tissue. (E) Identification of CD25^+^CD127^−^ CD45RA^−^CCR8^+^ Treg cells in human fat tissue. (F) Identification of CD25^+^CD127^−^CD45RA^−^CCR8^+^ Treg cells in human blood. Data were collected on a BD Symphony cytometer (see Table 36).

Human peripheral blood (Figure 31-35), thymus (Figure 36), and colon biopsies (Figure 37) were obtained following protocols approved by Clinical Research Ethics Boards of the University of British Columbia (H18- 02553 and H15-01034). Collection of human skin, fat and blood samples (Figure 38) was performed following protocols approved by the local ethical committee (Regensburg University, reference number 19-1453-101) and signed informed consent.

##### 11.4.2 Buffers and medias.

- Staining buffer: 1× Dulbecco’s Phosphate-Buffered Saline (PBS; ThermoFisher Scientific, #14190-144) supplemented with 2% Fetal Bovine Serum (VWR, #97068-085).
- MACS^™^ isolation buffer: 1× PBS supplemented with 0.5% bovine serum albumin (Sigma- Aldrich #A4503) and 2 mM EDTA (Roth #X986.2).
- EasySep^™^ Buffer: PBS with 2% Fetal Bovine Serum and 2 mM EDTA.
- Dissociation and washing media: ImmunoCult-XF T Cell Expansion Medium (STEMCELL Technologies, #10981) supplemented with 1% Penicillin/Streptomycin (ThermoFisher Scientific, #15140122).
- Complete media: RPMI 1640 (ThermoFisher Scientific, #11879020) supplemented with 10% FBS, 1% Penicillin/Streptomycin (ThermoFisher Scientific, #15140122) and 1% GlutaMAX (ThermoFisher Scientific, #35050061).

##### 11.4.3 Cell isolation/enrichment reagents.

- Lymphoprep (STEMCELL Technologies, #07861).
- Percoll (GE Healthcare, #17-0891-02).
- Ammonium Chloride Solution (STEMCELL Technologies, #07850).
- RosetteSep^™^ Human CD4^+^ T Cell Enrichment Cocktail (STEMCELL Technologies, # 15022).
- Custom EasySep^™^ Human Positive Selection Cocktail (STEMCELL Technologies, #18309- 0008).
- EasySep^™^ Releasable Rapidspheres (STEMCELL Technologies, #50201).
- EasySep^™^ Release Buffer (STEMCELL Technologies, #20145).
- Custom EasySep^™^ Human CD8 Negative Selection Cocktail (STEMCELL Technologies, #18309-0009).
- EasySep^™^ Dextran Rapidspheres (STEMCELL Technologies, #50100).
- CD25 Microbeads II (Miltenyi, # 130-092-983).
- Pan-CD25 Positive Selection and Depletion Cocktail (STEMCELL Technologies, # 17861).
- GentleMACS^™^ C tube (Miltenyi #130-096-334).
- MACS separation columns, size L (Miltenyi #130-042-401).
- Dead cell removal kit (Miltenyi 130-090-101).
- Collagenase VIII (Sigma-Aldrich, #C2139).
- DNAse (STEMCELL Technologies, #7900).
- UW Solution (Bridge to Life, Northbrook, IL)

##### 11.4.4 Common cell staining reagents.

- FOXP3 Fix/Perm Buffer Set (Thermo Fisher eBioscience, #00-5523-00).
- Human FOXP3 buffer set (BD, #560098).
- BD FACS^™^ Lysing Solution (BD, #349202).
- Brilliant Stain Buffer Plus (BD, #566385).
- Super Bright Complete Staining Buffer (ThermoFisher Scientific SB-4401-75).

##### 11.4.5 Cytometer and analysis.

BD Fortessa X20, configuration as per Table 34. Beckman Coulter Moflo Astrios cell sorter, configuration as per Table 35. BD Symphony, configuration as per Table 36. Analysis performed using Flowjo^™^ Version X (Treestar Inc, v10.5.3 or v10.7.1).

#### 11.5 Data analysis

See Section 11H.5.

#### 11.6 Pitfalls

See Section 11H.6.

#### 11.7 Top tricks

See Section 11H.7.

#### 11.8 Clinical relevance statement

The gating strategies shown in Figures 31-35 are applicable for the analysis and isolation of human Tregs in peripheral blood. We also provide protocols for processing and analyzing Tregs in a variety of tissues including pediatric thymus tissue (Figure 36), intestinal biopsies (Figure 37), and skin and fat tissues (Figure 38). The data included show the analysis of Tregs in healthy individuals but these gating strategies can also be used to analyze Tregs in patients with a variety of diseases [371, 387, 408]. The key conclusion from these analyses is that multiple markers are required to identify human Tregs. In human peripheral blood and a variety of peripheral tissues, Tregs can be identified by using a combination of high CD25, high FOXP3 expression, and low CD127 expression. Helios expression can also be included in such analyses to more confidently identify stable Tregs.

#### 11.9 Summary of the phenotype

This is shown in Table 37.

### 12 Murine Foxp3^+^ regulatory T cells

#### 12.1 Overview

Treg cells are a subset of CD4^+^ T cells that have fundamental functions in the maintenance of immune homeostasis and peripheral tolerance, among others. Treg cells can be found in almost any tissue including primary lymphoid organs (thymus, bone marrow), secondary lymphoid organs (spleen, lymph nodes, Peyer’s patches) as well as various non-lymphoid organs (e.g., intestine, skin, lung, liver, fat). Importantly, the Treg cell population is extremely heterogeneous and consists of numerous, highly specialized subsets that vary tremendously between the different organs and tissues. In this section, guidelines for widely accepted flow cytometry-based phenotyping strategies for murine Treg cells in both lymphoid organs and selected non-lymphoid tissues will be provided, and particular emphasis will be laid on markers for the identification of Treg cell subsets.

#### 12.2 Introduction

In the murine system, the lineage-defining transcription factor Foxp3 is widely used for the identification of Treg cells. In addition, CD25 is majorly expressed by Treg cells, but this marker is also stimulation-dependently upregulated by Tconv, as described for the human system [432,433]. Foxp3 proves to be more than a marker as stable Foxp3 expression is crucial for maintenance of Treg cell-specific transcription signatures and function. Loss of function mutations of *Foxp3* lead to the manifestation of the *scurfy* phenotype in mice, corresponding to the IPEX-syndrome in humans [434].

Depending on their origin, Treg cells are referred to as thymus-derived Treg cells (tTreg cells) or peripherally induced Treg cells (pTreg cells), which develop under tolerogenic conditions preferentially at mucosal sites [435,436]. As for the human system, markers to distinguish pTreg and tTreg cell subpopulations, especially under inflammatory conditions, are currently missing. Yet, Helios, Nrp-1 or ROR-γt are widely accepted for the discrimination of tTreg and pTreg cells under homeostatic conditions [364-366, 437-440]. Co-expression of Foxp3 and a second Th-lineage-defining transcription factor, such as T-bet and Gata-3, enables Treg cells to specifically suppress the respective Th-mediated immune response [441]. Treg cell subset composition in lymphoid and non-lymphoid organs depends on a variety of aspects, such as age, sex, genetic background, and several microbiota-related factors.

##### 12.2.1 Treg cells in murine lymphoid organs.

###### 12.2.1.1 Treg cells in the murine thymus.

**Figure 39.**
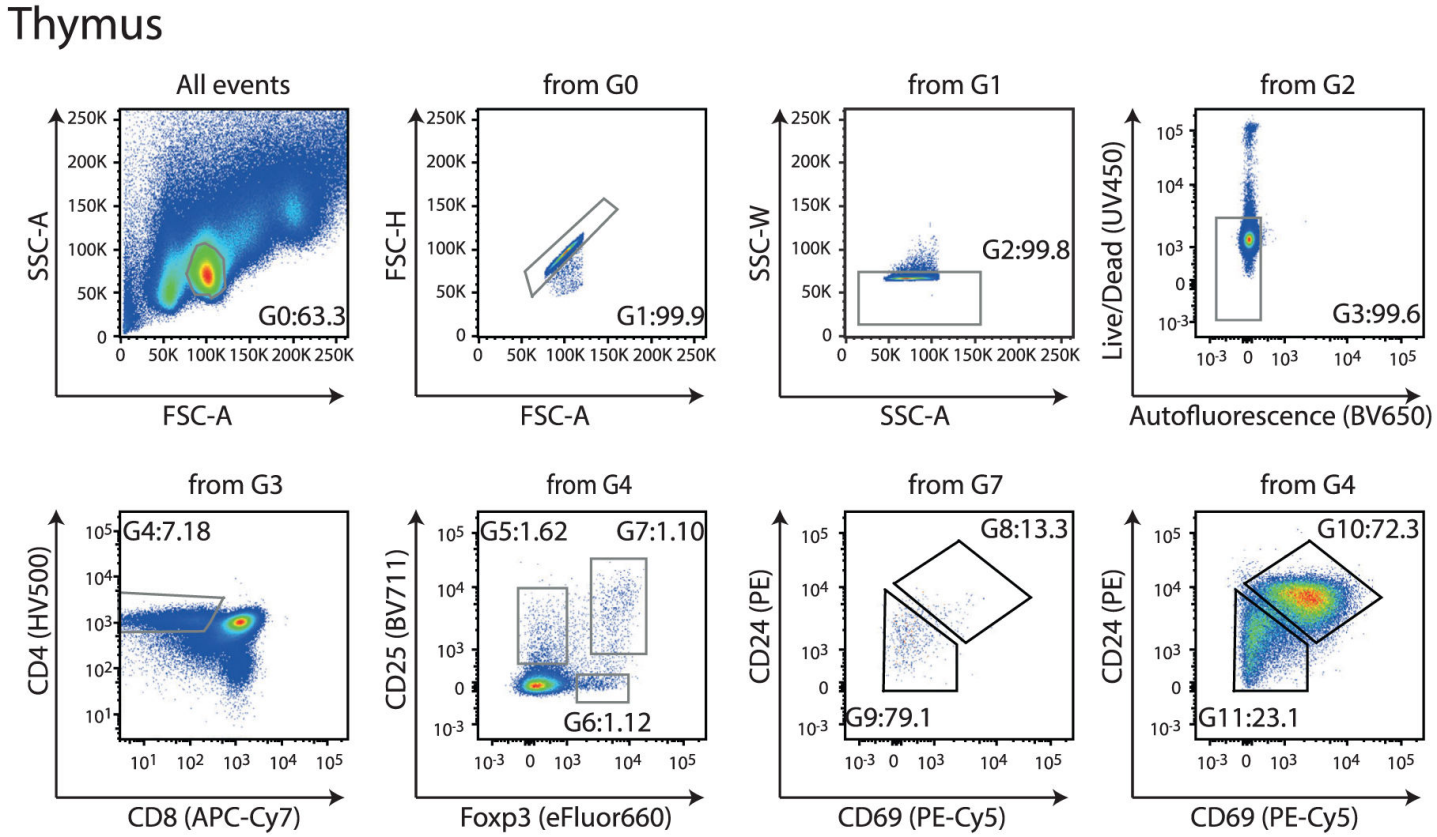
Phenotyping of Treg cells from the murine thymus. Gating strategy to identify Treg cells in the thymus. From all events, lymphocytes can be distinguished by their FSC/SSC properties (gate G0). After lymphocyte gating, doublets are excluded twice (gates G1 and G2), followed by exclusion of dead or autofluorescent cells (gate G3). From G3, CD4SP thymocytes (gate G4) are gated, from which two Treg cell precursors (G5 and G6) and thymic Treg cells (G7) can be identified. Thymic Treg cells (G7) and CD4SP thymocytes (G4) can be subdivided into two subsets of CD24^high^CD69^+^ immature (G8 and G10) and CD24^dim/low^CD69^−^ (G9 and G11) mature cells. Numbers indicate frequencies of cells within respective gates. Figures are based on thymocyte isolations from Foxp3^EGFPCreERT2^ROSA26^YFP^ mice.

After CD4 lineage commitment, some CD4 single-positive (SP) thymocytes, upon TCR stimulation, can develop into CD25^+^Foxp3^+^ tTreg cells through two distinct developmental programs involving CD25^+^Foxp3^−^ and CD25^−^Foxp3^+^ Treg cell precursors (Figure 39) [435]. Recent data suggest that the two distinct developmental programs are both required for the generation of a comprehensive Treg cell repertoire [442]. CD25^+^Foxp3^+^ tTreg cells can be further subdivided into subsets with different maturity based on CD69 and also CD24 expression (Figure 39), which is known to correlate inversely to the maturity of CD4SP and CD8SP thymocytes [443]. Gating for Treg cells in the human thymus is provided in section 11F.3.1 in Isolation and analysis of Tregs isolated from human paediatric thymus (Figure 36).

###### 12.2.1.2 Treg cells in murine spleen and lymph nodes.

**Figure 40.**
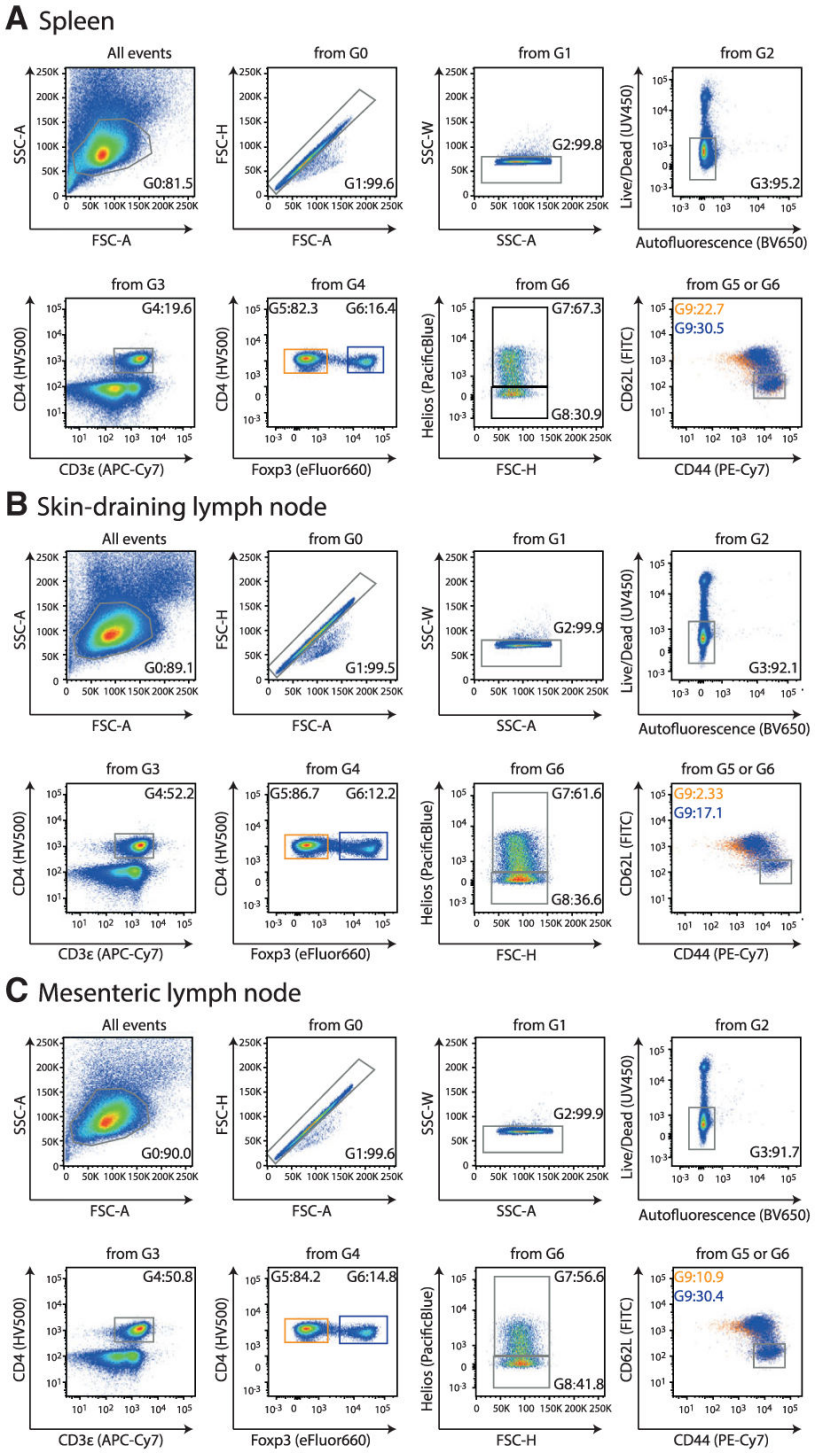
Phenotyping of Treg cells from murine spleen and lymph nodes. (A) Gating strategy to identify Treg cells in the spleen. From all events, lymphocytes can be distinguished by their FSC/SSC properties (gate G0). Based on G0, doublets are excluded twice (gates G1 and G2) followed by exclusion of dead or autofluorescent cells (gate G3). From G3, CD4^+^CD3ε^+^ T cells (gate G4) are gated, from which Foxp3^+^ Treg cells (gate G6) and Foxp3^−^ Tconv cells (gate G5) can be further identified. From G6, Helios^+^ tTreg (gate G7) and Helios^−^ pTreg cells (gate G8) are gated. Finally, a staining for CD62L and CD44 on Treg cells (gate G6, blue) and Tconv cells (gate G5, orange) are shown together, with CD62L^−^CD44^+^ effector/memory cells being gated (gate G9). (B and C) Gating strategy to identify Treg cells in skin-draining lymph nodes (B) and mesenteric lymph nodes (C). Gates as described in panel A. Numbers indicate frequencies of cells within respective gates. Figures are based on spleen and lymph node isolations from wild-type mice.

The frequency of murine Foxp3^+^ Treg cells among CD4^+^ T cells usually ranges from 10% to 20% in secondary lymphoid organs such as spleen, skin-draining lymph nodes, and mesenteric lymph nodes (Figure 40). The Treg cell population in any secondary lymphoid organ is a mixture of tTreg and pTreg cells, and Helios staining is most frequently used to discriminate tTreg (Foxp3^+^Helios^+^) and pTreg (Foxp3^+^Helios^−^) cells (Figure 40). On a functional basis, murine Treg cells in secondary lymphoid organs can be subdivided into CD62L^+^CD44^−^ naive-like and CD62L^−^CD44^+^ effector/memory-like Treg cells. In comparison to Foxp3^−^ Tconv cells, Treg cells in secondary lymphoid organs display a higher frequency of cells with a CD62L^−^CD44^+^ effector/memory phenotype (Figure 40).

##### 12.2.2 Treg cells in murine non-lymphoid tissues.

Apart from their fundamental immune regulatory function, Treg cells perform highly specialized functions in non-lymphoid tissues [406]. They have been shown to support tissue homeostasis and regeneration, ranging from regulating metabolic parameters in the adipose tissue to potentiating tissue repair [444-446], e.g., in skeletal muscles [447], lung tissue [448], or brain [449]. In addition, Treg cells in non-lymphoid tissues can manipulate tissue precursor cells to maintain tissue homeostasis. For example, Treg cells can promote oligodendrocyte progenitor cell differentiation and, thereby, myelin regeneration in the central nervous system [450]. In the skin, Treg cells promote hair follicle regeneration by augmenting hair follicle stem cell proliferation and differentiation[451]. Several publications identified the epidermal growth factor receptor ligand amphiregulin as a key factor of tissue Treg cells to maintain homeostasis or induce tissue regeneration in a diverse set of tissues, including lung, muscle, and brain [447, 448]. All data show that these non-canonical Treg cell functions to directly or indirectly promote organ homeostasis and tissue repair warrant a new definition of Treg cells: Treg cells are not only regulatory as their historic name implies, but subpopulations of Treg cells residing in non-lymphoid tissues are tissue-supporting and have the ability to promote tissue regeneration. Recently, Treg cells residing in non-lymphoid tissues were studied on an epigenetic and transcriptional level, and a subset of Treg cells expressing the marker KLRG1 and the IL-33 receptor ST2 was identified [376]. This subset of tissue resident Treg cells expressing ST2 was termed tisTregST2. This population can be found in every organ and tissue analyzed so far, vigorously increases in number upon IL-33 treatment *in vivo*, and is dependent on the transcription factor Batf. TisTregST2 cells are strongly Th2-like biased (amongst others, high expression of Gata-3) compared to other Treg cell populations or Tconv cells found in the same tissue, and express the epidermal growth factor receptor ligand amphiregulin in high amounts [376]. Tissue Treg cell precursor development is initiated in lymphatic organs and, thereby, follows a multistep model [375, 377, 452, 453]. Very recently, the human counterpart of murine tissue-repair Treg cells was identified and described as FOXP3^+^BATF^+^CCR8^+^ Treg cells, also present in low numbers in peripheral blood and in high number in human tissues such as skin, fat, or liver [378]. More details on the isolation and characterization of this cell type in human blood, skin, and fat tissue is provided in sections 11H.3.1 and 11H.3.2 (Figure 38) in Human FOXP3^+^ regulatory T cells. In the sections that follow, we describe the isolation and characterization of these tisTregST2 cells from different murine organs, including liver, skin, adipose tissue, lung, and colon

#### 12A.3 Step-by-step sample preparation of Treg cells from the thymus

##### Protocol: Isolation and analysis

- Sacrifice 6-10 weeks old animals.
- Expose thorax.
- Collect both thymic lobes using forceps.
- Place thymus on a 100 μm strainer.
- Use a syringe plunger to dissociate thymus in the presence of FACS buffer.
- Centrifuge cell suspension for 5 min with 300 × *g* at 4°C.
- Aspirate supernatant and resuspend cellular pellet with FACS buffer.
- Filter cell suspension with a 30 μm strainer and count cell number.

##### Surface and intracellular staining

- Transfer 2 × 10^6^ cells to a 5 ml FACS tube.
- Centrifuge cell suspension for 5 min with 300 × *g* at 4°C.
- Aspirate supernatant and resuspend cellular pellet with 100 μl Live/Dead fixable buffer (1:1000 diluted), keep cell suspension in the dark at 4°C for 30 min.
- Add 500 μl FACS buffer and centrifuge cell suspension for 5 min with 300 × *g* at 4°C.
- Aspirate supernatant and resuspend cellular pellet with 100 μl FACS buffer with diluted surface Abs, anti-mouse CD16/CD32 and rat IgG, keep cell suspension in the dark at 4°C for 30 min.
- Add 500 μl FACS buffer and centrifuge cell suspension for 5 min with 300 × *g* at 4°C.
- Aspirate supernatant and resuspend cellular pellet with 100 μl Fixation/Permeabilization working solution, keep cell suspension in the dark at 4°C for 30 min.
- Add 500 μl 1× Permeabilization buffer and centrifuge cell suspension for 5 min with 300 × *g* at 4°C.
- Aspirate supernatant and repeat the above step.
- Aspirate supernatant and resuspend cellular pellet with 100 μl 1× Permeabilization buffer with intracellular Abs, anti-mouse CD16/CD32 and rat IgG, keep cell suspension in the dark at 4°C for 30 min.
- Add 500 μl 1× Permeabilization Buffer and centrifuge cell suspension for 5 min with 300 × *g* at 4°C.
- Aspirate supernatant and repeat the above step.
- Resuspend cellular pellet with 200 μl 1× Permeabilization Buffer, and cell suspension can be used for immediate analysis.

#### 12A.4 Materials

##### FACS buffer

1× PBS (Gibco, #10010-056)

0.2 % Albumin from bovine serum (Sigma-Aldrich, #SI A3912-100G)

##### Erythrocyte lysis buffer

0.01 M KHCO_3_, 0.155 M NH_4_Cl, 0.1 mM EDTA

LIVE/DEAD^™^ Fixable Blue Dead Cell Stain Kit, for UV excitation (Thermo Fisher, #L23105)

Foxp3 Fix/Perm Buffer Set (Thermo Fisher eBiosciences, #00-5523-00)

100 μm + 30 μm cell strainer (Greiner Bio, #542070 and Partec #04-0042-2316)

###### Antibodies

**Table:** 

Dilution	Antibody	Manufacturer
1:1000	CD4 HV500 (RM4-5)	BD
1:1000	CD8α APC-Cy7 (53-6.7)	BioLegend
1:200	CD25 BV711 (PC61.5)	BioLegend
1:1000	CD44 PE-Cy7 (IM7)	BioLegend
1:1000	CD62L FITC (MEL-14)	eBioscience
1:1000	CD69 PE-Cy5 (H1.2F3)	BioLegend
1:400	Foxp3 eFluor660 (FJK-16S)	eBioscience
1:100	Helios PacificBlue (22F6)	BioLegend
1:400	Rat IgG (11.5 mg/ml)	JacksonImmuno Research
1:400	Anti-mouse CD16/CD32 (1mg/ml)	Bioxcell

###### Mice

Foxp3^EGFPCreERT2^ROSA26^YFP^ mice (C57BL/6 background) and wild type (WT) mice (BALB/c background) were bred under SPF conditions in isolated, ventilated cages (Helmholtz Centre for Infection Research, Braunschweig, Germany).

###### Cytometer

BD LSR Fortessa^™^ 5-laser cytometer (UV Violet, Blue, Yellow-Green, Red)

#### 12A.5 Data analysis

FlowJo Version 10.5.3 (Windows 10). The data are shown in Figure 39.

#### 12A.6 Pitfalls: Isolation and analysis of Treg cells from thymus

- Strict CD4SP gating is critical, otherwise, contamination from CD4^−^CD8^−^ double-negative (DN) cells might substantially increase the frequency of CD25^+^Foxp3^−^ Treg cell precursors. Yet, the CD25 expression level within DN thymocytes is much higher than within CD25^+^Foxp3^−^ Treg precursors. On the other hand, Treg cells have lower CD4 expression compared to their CD4^+^Foxp3^−^ Tconv cell counterpart [454,455]. Thus, too strict gating can negatively influence the frequency of Treg cells among CD4SP cells (Figure 39). Additional staining for and gating on CD3ε^+^ or TCRβ^+^ cells might be beneficial for an accurate CD4SP gating.
- Mediastinal lymph nodes are located in proximity to the thymus and can swell under inflammatory conditions. When removing thymi from mice with local inflammation, particular caution has to be paid to avoid “contamination” of the thymus material with mediastinal lymph nodes.

#### 12A.7 Top tricks: Isolation and analysis of Treg cells from thymus

- A substantial portion of Treg cells found within the thymus are Treg cells recirculating from the periphery [456]. These recirculating cells can be identified as CCR6^+^CCR7^−^ cells [457], or more easily when using RAG^gfp^ reporter mice. Only recently developed tTreg cells are RAG^GFP^ positive, while recirculating Treg cells are RAG^GFP^ negative.
- Not only αβ^+^ T cells but also γδ^+^ T cells and NKT cells develop within the thymus. An extra dump panel for NK1.1^+^ and TCRγ/δ^+^ cells results in higher specificity.
- Thymi will shrink upon aging. Six to 10 weeks mice are most commonly used to study thymocytes. Younger or older mice may result in lower numbers of Treg cells for analysis or sorting.
- Sacrificing mice with cervical dislocation can result in bleeding into the thoracic cavity. Washing the blood-stained thymus with PBS containing 30 μM EDTA removes the ‘contaminating’ blood.

#### 12A.8 Summary of the phenotypes

This is shown in Table 38.

#### 12B.3 Step-by-step sample preparation of Treg cells from spleen and lymph nodes

##### Protocol: *Isolation and analysis*

- Sacrifice animals.
- Expose abdominal cavity.
- Remove spleen, skin-draining lymph nodes (axillary, brachial, and inguinal lymph nodes), and mesenteric lymph nodes with forceps.
- Place spleen, skin-draining lymph nodes, and mesenteric lymph nodes on a 100 μm strainer separately.
- Use a syringe plunger to dissociate spleen and lymph nodes in the presence of FACS buffer.
- Centrifuge cell suspension for 5 min with 300 × *g* at 4°C.
- Step for spleen only: Aspirate supernatant and resuspend splenocytes pellet with 1 ml 37°C prewarmed erythrocyte lysis buffer and incubate for 3 min at room temperature (RT). Add 9 ml FACS buffer and centrifuge cell suspension for 5 min with 300 × *g* at 4°C.
- Aspirate supernatant and resuspend cellular pellet with FACS buffer.
- Filter cell suspension with a 30 μm strainer and count cell numbers.

##### Surface and intracellular staining

- Transfer 2×10^6^ cells to a 5 ml FACS tube.
- Centrifuge cell suspension for 5 min with 300 × *g* at 4°C.
- Aspirate supernatant and resuspend cellular pellet with 100 μl Live/Dead fixable buffer (1:1000 diluted), keep cell suspension in the dark at 4°C for 30 min.
- Add 500 μl FACS buffer and centrifuge cell suspension for 5 min with 300 × *g* at 4°C.
- Aspirate supernatant and resuspend cellular pellet with 100 μl FACS buffer with diluted surface Abs, anti-mouse CD16/CD32 and rat IgG, keep cell suspension in the dark at 4°C for 30 min.
- Add 500 μl FACS buffer and centrifuge cell suspension for 5 min with 300 × *g* at 4°C.
- Aspirate supernatant and resuspend cellular pellet with 100 μl Fixation/Permeabilization working solution, keep cell suspension in the dark at 4°C for 30 min.
- Add 500 μl 1× Permeabilization buffer and centrifuge cell suspension for 5 min with 300 × *g* at 4°C.
- Aspirate supernatant and repeat the above step.
- Aspirate supernatant and resuspend cellular pellet with 100 μl 1× Permeabilization buffer with intracellular Abs, anti-mouse CD16/CD32 and rat IgG, keep cell suspension in the dark at 4°C for 30 min.
- Add 500 μl 1× Permeabilization Buffer and centrifuge cell suspension for 5 min with 300 × *g* at 4°C.
- Aspirate supernatant and repeat the above step.
- Resuspend cellular pellet with 200 μl 1× Permeabilization Buffer, and cell suspension can be used for immediate analysis.

#### 12B.4 Materials

##### FACS buffer

1× PBS (Gibco, #10010-056)

0.2 % Albumin from bovine serum (Sigma-Aldrich, #SI A3912-100G)

##### Erythrocyte lysis buffer

0.01 M KHCO_3_, 0.155 M NH_4_Cl, 0.1 mM EDTA

LIVE/DEAD^™^ Fixable Blue Dead Cell Stain Kit, for UV excitation (Thermo Fisher, #L23105)

Foxp3 Fix/Perm Buffer Set (Thermo Fisher eBiosciences, #00-5523-00)

100 μm + 30 μm cell strainer (Greiner Bio, #542070 and Partec #04-0042-2316)

###### Antibodies

**Table:** 

Dilution	Antibody	Manufacturer
1:100	CD3ε APC-Cy7 (145-2C11)	BioLegend
1:1000	CD4 HV500 (RM4-5)	BD
1:200	CD25 BV711 (PC61.5)	BioLegend
1:1000	CD44 PE-Cy7 (IM7)	BioLegend
1:1000	CD62L FITC (MEL-14)	eBioscience
1:1000	CD69 PE-Cy5 (H1.2F3)	BioLegend
1:400	Foxp3 eFluor660 (FJK-16S)	eBioscience
1:100	Helios PacificBlue (22F6)	BioLegend
1:400	Rat IgG (11.5 mg/ml)	JacksonImmuno Research
1:400	Anti-mouse CD16/CD32 (1mg/ml)	Bioxcell

###### Mice

Foxp3^EGFPCreERT2^ROSA26^YFP^ mice (C57BL/6 background) and wild-type (WT) mice (BALB/c background) were bred under SPF conditions in isolated, ventilated cages (Helmholtz Centre for Infection Research, Braunschweig, Germany).

###### Cytometer

BD LSR Fortessa^™^ 5-laser cytometer (UV Violet, Blue, Yellow-Green, Red)

#### 12B.5 Data analysis

FlowJo Version 10.5.3 (Windows 10). The data are shown in Figure 40.

#### 12B.6 Pitfalls: Isolation and analysis of Treg cells from spleen and lymph nodes

- Properly collecting lymph nodes requires practice. Taking out fat instead of lymph nodes is one common mistake. Thus, for practicing it is easier to use young mice with lower body fat content.
- Erythrocyte lysis of spleen samples before staining is mandatory to avoid high background staining or clogging of the cytometer.

#### 12B.7 Top tricks: Isolation and analysis of Treg cells from spleen and lymph nodes

- Inspect collected lymph nodes in FACS buffer before dissociating them. Lymph nodes should sink, while fat pads float.
- Besides Helios, Nrp-1 is a commonly used marker to distinguish tTreg and pTreg cells. As Helios^−^Nrp-1^−^ Treg cells in mesenteric lymph nodes are mainly RORγt^+^, this marker was also claimed as a good tool to identify pTreg cells within the intestinal system.
- For the sorting of intact Foxp3^+^ Treg cells for the collection of RNA, various Foxp3 reporter mouse lines can be used to avoid the fixation and permeabilization during the intracellular staining.
- Stimulation of CD4^+^ T cells before staining will result in down-regulation of CD3ε and CD4, which could complicate the gating (Figure 40). Alternatively, CD90 instead of CD3ε can be used.

#### 12B.8 Summary of the phenotype

This is shown in Table 39.

#### 12C.3 Step-by-step sample preparation: Isolation and analysis of Treg cells from liver

**Figure 41.**
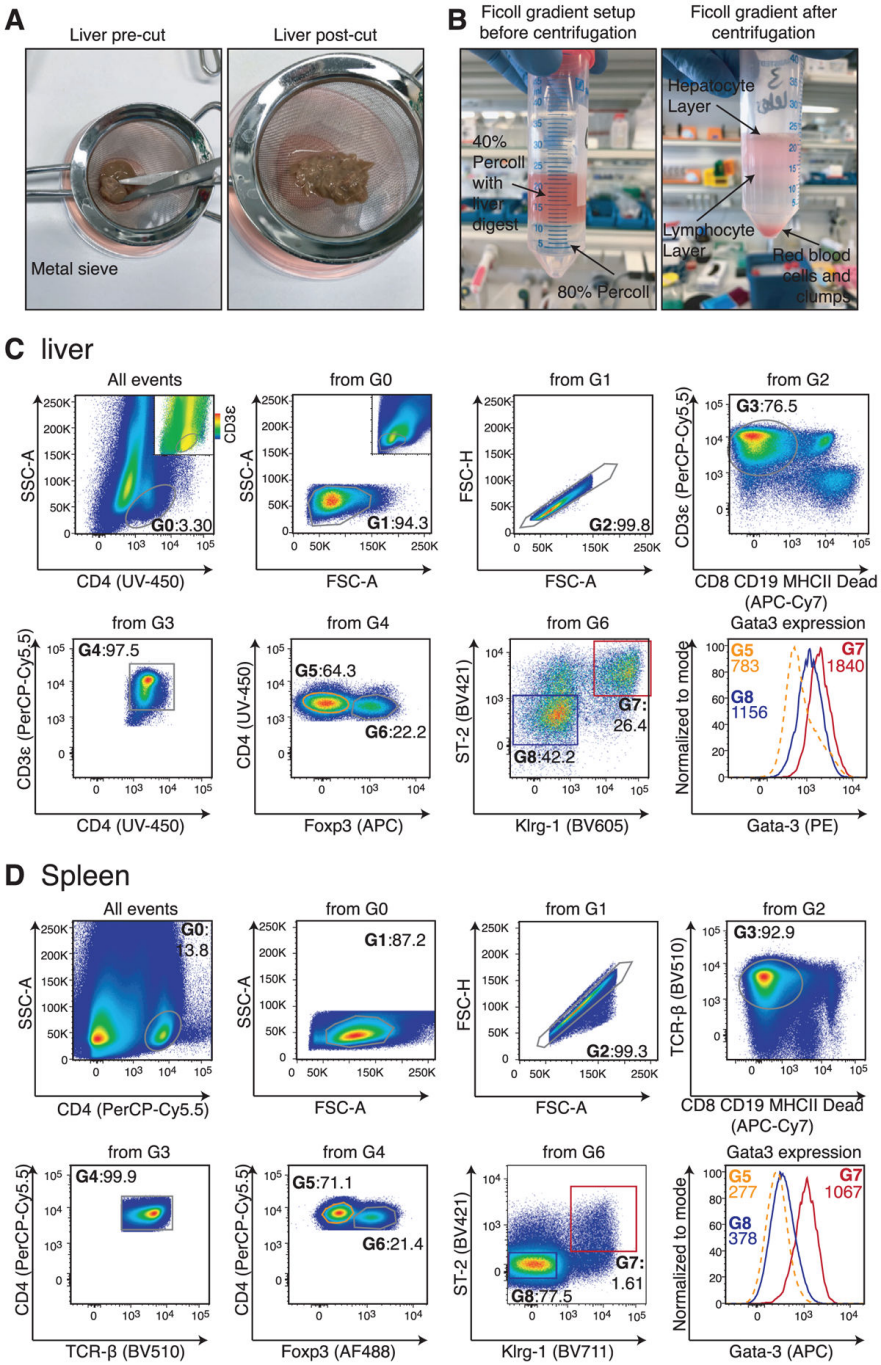
Isolation and analysis of Treg cells from murine liver and spleen. (A) Image of liver tissue pre-cut (left) and after cutting (right) in a metal sieve. After cutting, a syringe plunger can be used to disseminate the tissue. (B) Image of the preparation of a liver suspension in the Percoll gradient (left). The bottom phase consists of 80 % Percoll-PBS, the top phase of 40% Percoll-PBS and the digested liver cells. On the right, a representative image of a sample after centrifugation is shown. Three layers can be discriminated: a top layer consisting mainly of hepatocytes, the middle layer with target cells, and a bottom layer with unwanted cells. (C) Gating strategy to identify tisTregST2 cells in liver. From all events, a CD4-gate to identify T cells can be drawn (gate G0). In the plot, the smaller color-coded plots indicate expression of CD3ε in the same SSC-A versus CD4 plot. Presence of CD3ε^+^ cells in the G0 gate can be appreciated. Based on G0, lymphocytes can be identified by their FSC/SSC properties (gate G1). Next, doublets are excluded (gate G2) as well as unwanted, dead or autofluorescent cells (gate G3). From G3, CD4^+^CD3ε^+^ T cells (gate G4) are gated, from which Treg cells (gate G6) and Tconv cells (gate G5) can be identified. Finally, Klrg1^+^ST2^+^ tisTregST2 (gate G7) are gated from Treg cells (gate G6). A staining of Gata-3, shown in the histogram, exemplifies the expression of this marker in liver Tconv cells (gate G5, orange, dotted line), liver Klrg1^+^ST2^+^ tisTregST2 cells (gate G7, red), and liver Klrg1^−^ST2^−^ Treg cells (gate G8, blue), numbers indicate geometric mean fluorescence intensity of Gata-3. In (D), the same gating strategy as described for liver is applied to a spleen sample. In both tissues, CD4^+^Foxp3^+^Klrg1^+^ST2^+^Gata-3^high^ tisTregST2 cells can be identified with the proposed gating strategy. CD3ε or TCRβ Abs can be used. Numbers indicate frequencies of cells within respective gates. Figures are based on liver digestions and spleen isolations from Foxp3^DTR, GFP^ animals.

- Sacrifice animals.
- Expose thorax as well as abdominal cavity.
- Open inferior vena cava and inject PBS-filled syringe into left ventricle of heart and flush with >10 ml PBS to clear body circulation; liver should change from red color to pale.
- Remove whole organ including right, left, caudate, and quadrate lobes.
- Place pieces on metal strainers, add 5 ml liver digestion buffer, and cut liver lobes into small pieces as shown in Figure 41A. A syringe plunger is used to mash liver, and the metal strainer and petri dish can be flushed with additional 5 ml of liver digestion buffer to collect all remaining cells and fragments.

Collagenase mix for digestion of murine liver tissue

**Table:** 

Name	Concentration	Company	Catalog
Collagenase	1 mg/ml	Sigma-Aldrich	C5138
Type IV DNAse I	20 μg/ml	Roche	11284932001
Bovine	5 mg/ml	Sigma-Aldrich	A4503
serum			
albumin			
DMEM	Fill up to 10 ml	Gibco	41965

- Digest sample for 25–40 min on a rotating shaker in an incubator (37°C) or in horizontal-shaking water bath pre-heated to 37°C.
- Add 50 mM EDTA-PBS to a final concentration of 2 mmol/l and incubate for 2 min.
- Centrifuge for 5 min with 300 × *g* at RT.
- Remove supernatant and resuspend cellular pellet in 10 ml of 40% Percoll-PBS solution; use a 5 ml pipette to dissociate pellet completely.
- Use pipetting aids to slowly and carefully place 10 ml of 80% Percoll-PBS beneath cell suspension to establish a two-phase system as shown in Figure 41B. It is helpful to turn off the electric force in the pipet aid to slowly release the 80% Percoll-PBS.
- Centrifuge for 20 min with 2000 × *g* at 4°C, acceleration off, deceleration off. If successful, hepatocytes will float on top of gradient and can be removed via aspiration. The middle phase contains immune cells and should be collected in a separate tube, while the pellet contains red blood cells and other cell types and can be discarded (Figure 41B).
- Dilute middle phase with PBS to a volume of 50 ml.
- Centrifuge for 5 min with 300 × *g* at 4°C. Cellular pellet contains lymphocyte fraction and, following red blood cell lysis, can be used for immediate analysis or sorting.

#### 12C.4 Materials: Isolation and analysis of Treg cells from liver

FACS staining buffer

Base medium 1× PBS
2% Fetal bovine serum

MACS isolation buffer

Base medium 1× PBS
0.5% w/v bovine serum albumin (Sigma-Aldrich #A4503)
1 mM EDTA (Roth #X986.2)

40% Percoll solution

Base medium DI water
40% Percoll (GE Healthcare #17-0891-01)
1% PBS (from 10× PBS Gibco #14200-067)

80% Percoll solution

Base medium DI water
80% Percoll (GE Healthcare #17–0891-01)
1% PBS (from 10× PBS Gibco #14200-067)

##### Antibodies

1:100 CD8 APC-Cy7 (53-6.7)
1:100 CD19 APC-Cy7 (6D5)
1:200 MHCII APC-Cy7 (M5/114.15.2)
1:500 Fixable Viability dye eF780 (Thermo Fisher eBioscience #65-0865-14)
1:100 CD4 PerCP-Cy5.5 or BUV-395 (RM4-5)
1:100 TCRβ BV 510 (H57-597)
1:100 CD3ε PerCP-Cy5.5 (145-2C11)
1:100 CD25 APC (PC61)
1:100 IL-33R/ST2 BV 421 (DIH9)
1:100 Klrg1 BV 711 (2F1)
1:100 Foxp3 AF488 (FJK-16S)
1:100 Gata-3 PE (16E10A23)

Foxp3 Fix/Perm Buffer Set (Thermo Fisher eBiosciences #00-5523-00)

##### Mice

Foxp3^GFP,DTR^ mice (C57BL/6 background) were bred in the animal facility of the Regensburg University Hospital.

##### Cytometer

BD LSRII^™^ 3-laser cytometer (blue-red-violet)

#### 12C.5 Data analysis

BD Flowjo^™^ Version X (10.5.3 Mac OS). The data are shown in Figure 41.

#### 12C.6 Pitfalls: Isolation and analysis of Treg cells from liver

- Incomplete perfusion of the animal will result in red blood cell contamination. Fast experimental protocols and fast animal handling are required. Do not forget to open the vena cava prior to flushing the circulation with PBS.
- Poor recovery after mashing step with large livers: Add more digestion buffer to completely wash filter mesh. Do not use medium or PBS to wash filter mash since collagenase levels will be diluted.
- Gradient setup fails and poor lymphocyte recovery after gradient centrifugation: Slowly add 80% Percoll to solution and use a pipetting aid without acceleration/deceleration to avoid mixing 40% and 80% solutions. Handle tubes carefully to avoid mixing both phases. Carefully balance the centrifuge to avoid imbalance or rotor damage.
- Low CD4^+^ T cell content (<0.5%) in final preparation: Avoid collecting cellular pellet after gradient centrifugation since it contains unwanted cells. Completely remove top layer containing hepatocytes.

#### 12C.7 Top tricks: Isolation and analysis of Treg cells from liver

- If you analyze animals <12 days of age, the liver can be measured without the need of gradient centrifugation.
- Even after complete perfusion, a red blood cell contamination can occur. Perform red blood cell lysis to deplete red blood cells.
- If you are unsure about the phases after gradient centrifugation (top: hepatocytes; middle phase: lymphocytes and other cells; pellet: other cells), harvest each phase and perform a T-cell staining to calculate your yield.
- Stain for CD45 to discriminate bone marrow (BM)-derived cells such as T or B cells from other cell types.

#### 12C.8 Summary of the phenotype

This is shown in Table 40.

#### 12D.3 Step-by-step sample preparation: Isolation of Treg cells from skin with or without GentleMACS^®^

- Sacrifice animals.
- Animals older than 10 days require hair removal via a small animal electric shaver.
- Treat shaved skin with commercially available hair removal creme and incubate for 3 min at RT.
- Wash off hair removal creme with tap water and try to remove any remaining patches of hair.
- Separate hair-free skin from dorsal surface tissue and place in 10 ml skin digestion buffer.

Collagenase mix for digestion of murine skin tissue

**Table:** 

Name	Concentration	Company	Catalog
Collagenase	4 mg/ml	Sigma-Aldrich	C5138
Type IV			
DNAse I	10 μg/ml	Roche	11284932001
Fetal bovine	2%	NA	NA
serum			
DMEM	Fill up to 10 ml	Gibco	41965

**Figure 42.**
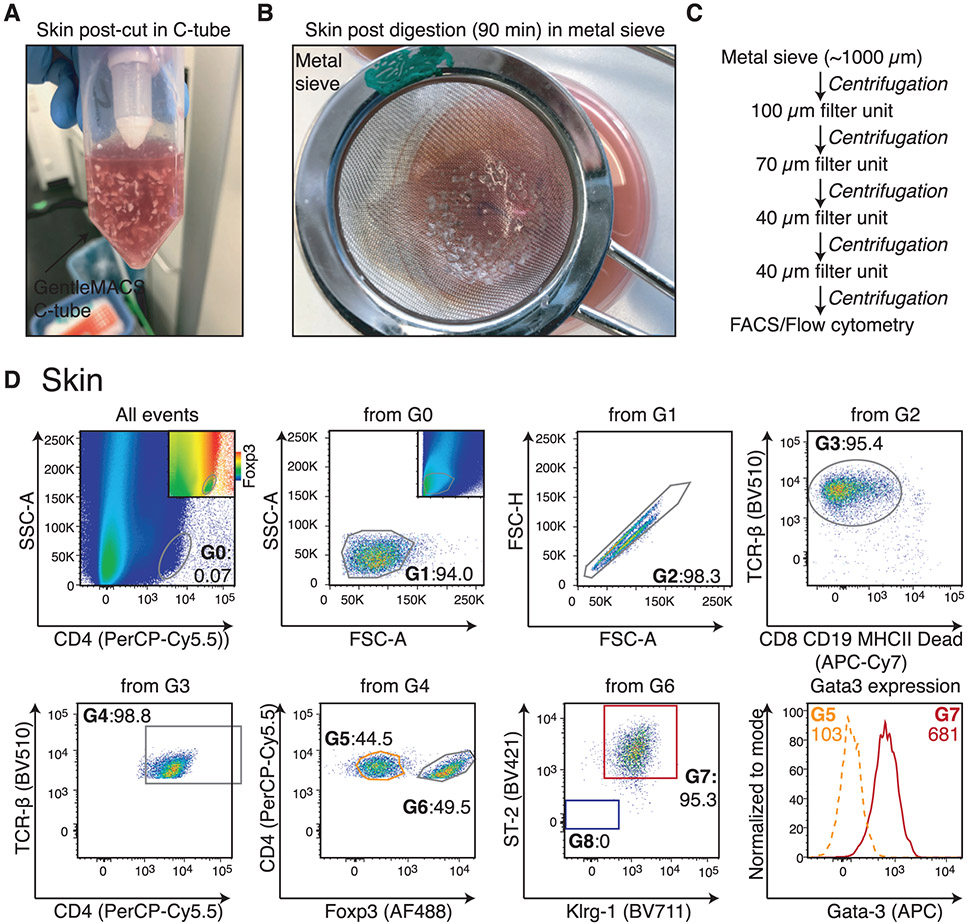
Isolation and analysis of Treg cells from murine skin. (A) Representative image of skin tissue in digestion buffer after cutting with scissors. Cutting can be performed directly in the GentleMACS^®^ C tube. (B) Image of the skin tissue after digestion. The sample is poured onto a metal mesh and can be dissociated manually using a syringe plunger. (C) Sequential filtration workflow for skin samples. (D) Gating strategy to identify tisTregST2 cells in skin tissue. From all events, a CD4-gate to identify T cells can be drawn (gate G0). In the plot, the smaller color-coded plots indicate expression of Foxp3 in the same SSC-A vs CD4 plot. Presence of Foxp3^+^ cells in the G0 gate can be appreciated. Based on G0, lymphocytes can be identified by their FSC/SSC properties (gate G1). Smaller plot shows FCS/SSC of all events without CD4 pre-gating. Next, doublets are excluded (gate G2) as well as unwanted, dead, or autofluorescent cells (gate G3). From G3, CD4^+^TCRβ^+^ T cells (gate G4) are gated, from which Treg cells (gate G6) and Tconv cells (gate G5) can be identified. Finally, Klrg1^+^ST2^+^ tisTregST2 (gate G7) are gated from Treg cells (gate G6). A staining of Gata-3, where numbers indicate geometric mean fluorescence intensity, exemplifies the expression of this marker in skin Tconv cells (gate G5, orange, dotted line) and skin Klrg1^+^ST2^+^ tisTregST2 cells (gate G7, red). Numbers indicate frequencies of cells within respective gates. Figures are based on skin digestions from Foxp3^DTR, GFP^ animals.

- Cut into small pieces either in a 50 mL tube or directly in the GentleMACS C tube as shown in Figure 42A.
- When using the GentleMACS^®^, the sample can be incubated using the program “37_C_Multi_H” for 90 min.
- When digesting manually, the sample is digested for 60 min on a rotating shaker in the incubator (37°C) or in a shaking water bath preheated to 37°C.
- Place sample on a metal strainer located in a Petri dish and use a syringe plunger to dissociate remaining tissue pieces (see Figure 42B).
- Filter sample via a 100 μm filter unit (Figure 42C).
- Centrifuge for 5 min with 300 × *g* at RT.
- Filter sample via a 70 μm filter unit.
- Centrifuge for 5 min with 300 × *g* at RT.
- Filter sample via a 40 μm filter unit.
- Centrifuge for 5 min with 300 × *g* at RT.
- Stain sample for analysis or cell sorting.

#### 12D.4 Materials: Isolation and analysis of Treg cells from skin

FACS staining buffer

Base medium 1× PBS
2% Fetal bovine serum

MACS isolation buffer

Base medium 1× PBS
0.5% w/v bovine serum albumin (Sigma-Aldrich #A4503)
1 mM EDTA (Roth #X986.2)

*GentleMACS C tube* (Miltenyi Biotec #130-096-334)

##### Antibodies

1:100 CD8 APC-Cy7 (53-6.7)
1:100 CD19 APC-Cy7 (6D5)
1:200 MHCII APC-Cy7 (M5/114.15.2)
1:500 Fixable Viability dye eF780 (Thermo Fisher eBioscience #65-0865-14)
1:100 CD4-PerCP-Cy5.5 or BUV-395 (RM4-5)
1:100 TCRβ-BV 510 (H57-597)
1:100 CD25 APC (PC61)
1:100 IL-33R/ST2 BV 421 (DIH9)
1:100 Klrg1 BV 711 (2F1)
1:100 Foxp3 AF488 (FJK-16S)
1:100 Gata-3 PE (16E10A23)

Foxp3 Fix/Perm Buffer Set (Thermo Fisher eBiosciences #00-5523-00)

##### Mice

Foxp3^GFP,DTR^ mice (C57BL/6 background) were bred in the animal facility of the Regensburg University Hospital.

##### Cytometer

BD LSRII^™^ 3-laser cytometer (blue-red-violet).

#### 12D.5 Data analysis

BD Flowjo^™^ Version X (10.5.3 Mac OS). The data are shown in Figure 42.

#### 12D.6 Pitfalls: Isolation and analysis of Treg cells from skin

- Filter clogged during filtration: Remove hair completely, either by shaving or hair removal cream. Repeat hair removal if patches of hair remain.
- Filter clogged during filtration: Filter samples sequentially as listed in Figure 42C.
- In case of abnormally high lymphocyte content/low Treg cell percentage (<15 %): Possible lymph node contamination. Avoid collecting inguinal lymph nodes during the separation of skin tissue from dorsal surface tissue. A typically sized patch of skin from the back of a >100 days old animal contains not more than 5000–10,000 Foxp3^+^ Treg cells when applying the proposed method, and Foxp3^+^ Treg cell frequency of CD4^+^ T cells is usually above 40%.
- GentleMACS^®^ mechanical failure or tube squeaking sounds: Cut the skin into very small pieces using sharp scissors. When using thick skin patches or large (>2 g) amounts of skin, re-cut skin tissues during incubation on the GentleMACS^®^ machine. Use more skin digestion buffer or distribute skin among more C tubes if required.
- Being unable to find lymphocytes: It is sometimes tricky to identify lymphocytes during flow cytometric analysis of the skin. Use the gating strategy provided in Figure 42D to identify T cells. If in doubt, use additional T-cell markers (other than CD4) to clearly identify the T-cell population, such as TCR-β, CD45, or CD90. Be aware that CD4 staining is weak if you use the protocol described above. It is helpful to use an autofluorescence-free channel with high staining index (such as PE or APC) for CD4 staining.
- Poor sort purity or contamination with skin keratinocytes after sorting: Use a double-sort approach (yield and 4-way-purity) to avoid contaminating the sample with skin-resident cells. See top tricks for more details.
- Machine clogging or no events: Wash machine and filter sample again and re-acquire. Keep the sample cool at 4°C and continuous rotation (300 rpm).

#### 12D.7 Top tricks: Isolation and analysis of Treg cells from the skin

- We were unable to perform pre-enrichment using magnetic beads for murine skin-based samples. Still, because of the very low frequency of Foxp3^+^ Treg cells as well as the high viscosity of the resulting cell mixture in murine skin samples, enrichment would be beneficial to decrease staining and measurement time.
- Sorting bulk skin Treg cells can lead to poor recovery of cells (low “sort efficiency”) and, based on the parameters of the sorting instrument, also to contamination with skin keratinocytes (aggregates with immune cells). Therefore, we propose a two-step sorting protocol: first, a pre-enrich sort (sort strategy: “yield”) where target cells are sorted into FACS buffer. Second, the sample is re-acquired and sorted again with high purity (sort strategy: “purity” or “4-way-purity”). Using this strategy, skin samples can be sorted at high speed without losing many target cells.
- For flow cytometric analysis, samples should be filtered again immediately before acquisition. If the acquisition takes more than 5 min, the sample should be filtered again to avoid clogging of the instrument. Samples should be cooled at 4°C to avoid clogging.
- Fixing samples will generally increase the sample flow through cytometers. Be careful when setting your FCS/SSC voltages to include your target cells. Include a positive staining control (e.g., splenocytes) to validate the panel and Ab staining before acquiring skin cells.

#### 12D.8 Top tricks: Summary phenotype

This is shown in Table 41.

#### 12E.3 Step-by-step sample preparation: Isolation of Treg cells from fat

- Sacrifice animals.
- Excise abdominal/epididymal fat pads (male mice) and move into 10 ml fat digestion buffer in a 50 ml tube. Avoid collecting the gonads.

Collagenase mix for digestion of murine skin tissue

**Table:** 

Name	Concentration	Company	Catalog
Collagenase	1 mg/ml	Sigma-Aldrich	C6885
Type II			
DNAse I	20 μg/ml	Roche	11284932001
Bovine serum	20 mg/ml	Sigma-Aldrich	A4503
albumin			
DMEM	Fill up to 10 ml	Gibco	41965

**Figure 43.**
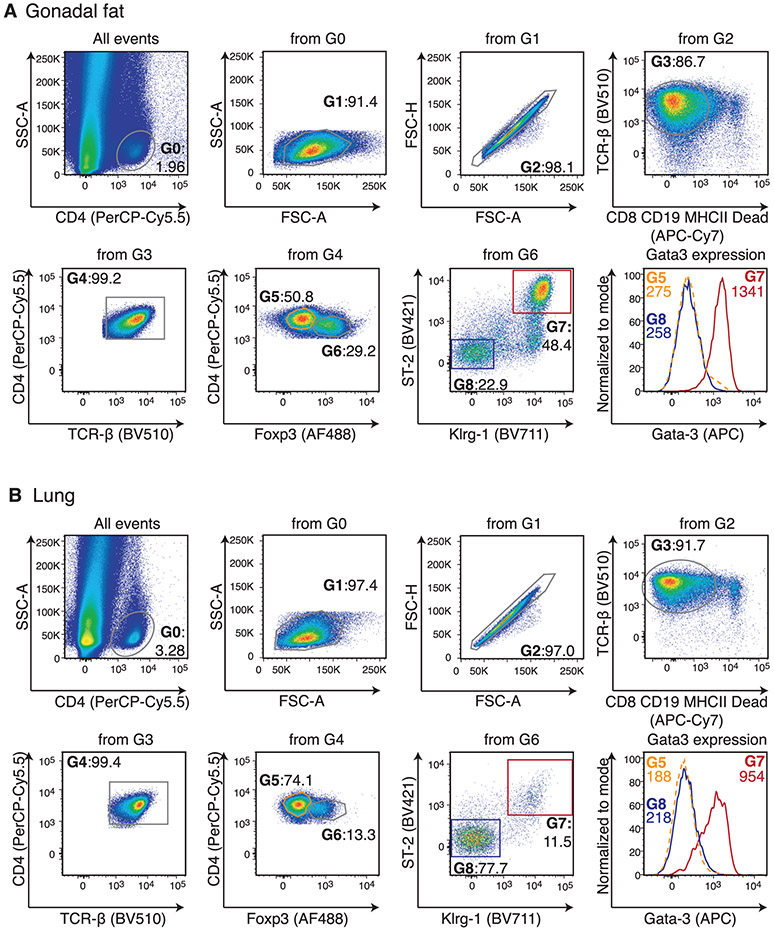
Isolation and analysis of T cells from the murine fat and lung tissue. Gating strategy to identify Treg cells in fat (A) and lung (B) tissue. From all events, a CD4-gate to identify T cells can be drawn (gate G0). Based on G0, lymphocytes can be identified by their FSC/SSC properties (gate G1). Next, doublets are excluded (gate G2) as well as unwanted, dead, or autofluorescent cells (gate G3). From G3, CD4^+^TCRβ^+^ T cells (gate G4) are gated, from which Treg cells (gate G6) and Tconv cells (gate G5) can be identified. Finally, Klrg1^+^ST2^+^ tisTregST2 (gate G7) are gated from Treg cells (gate G6). A staining of Gata-3, where numbers indicate geometric mean fluorescence intensity, exemplifies the expression of this marker in Tconv cells (gate G5, orange, dotted line), Klrg1^+^ST2^+^ tisTregST2 cells (gate G7, red), and Klrg1^−^ST2^−^ Treg cells (gate G8, blue). Numbers indicate frequencies of cells within respective gates. Figures are based on lung and fat digestions from Foxp3^DTR, GFP^ animals.

- Cut fat pads into small pieces with scissors and digest for 40–45 min on a rotating shaker in the incubator (37°C) or in a shaking water bath preheated to 37°C.
- Add EDTA-PBS to a final concentration of 2 mmol/l and incubate for 2 min.
- Centrifuge for 5 min with 300 × *g* at RT.
- Remove supernatant containing fat cells and lipids and perform erythrocyte lysis as described in spleen section.
- Stain sample for flow cytometry or cell sorting (Figure 43A).

#### 12E.4 Materials: Isolation and analysis of Treg cells from fat

FACS staining buffer:

Base medium 1× PBS
2% Fetal bovine serum

MACS isolation buffer:

Base medium 1× PBS
0.5 % w/v bovine serum albumin (Sigma-Aldrich #A4503)
1 mM EDTA (Roth #X986.2)

EDTA-PBS 50 mM

Base medium 1× PBS
50 mM EDTA (Roth #X986.2)

##### Antibodies

1:100 CD8 APC-Cy7 (53-6.7)
1:100 CD19 APC-Cy7 (6D5)
1:200 MHCII APC-Cy7 (M5/114.15.2)
1:500 Fixable Viability dye eF780 (Thermo Fisher eBioscience #65-0865-14)
1:100 CD4-PerCP-Cy5.5 or BUV-395 (RM4-5)
1:100 TCRβ-BV 510 (H57-597)
1:100 CD25 APC (PC61)
1:100 IL-33R/ST2 BV 421 (DIH9)
1:100 Klrg1 BV 711 (2F1)
1:100 Foxp3 AF488 (FJK-16S)
1:100 Gata-3 PE (16E10A23)

Foxp3 Fix/Perm Buffer Set (Thermo Fisher eBiosciences #00-5523-00)

##### Mice

Foxp3^GFP,DTR^ mice (C57BL/6 background) were bred in the animal facility of the Regensburg University Hospital.

##### Cytometer

BD LSRII^™^ 3-laser cytometer (blue-red-violet)

#### 12E.5 Data analysis

BD Flowjo^™^ Version X (10.5.3 Mac OS). The data are shown in Figure 43.

#### 12E.6 Pitfalls: Isolation and analysis of Treg cells from fat

- Little abdominal/epididymal fat depots in the abdominal cavity: Animals might be too young (<10-12 weeks), sick, or fasting. Gonadal fat depots increase with age, and so does the lymphocyte recovery. Gender also influences fat, with male mice having larger depots.
- Abnormally low Treg cell frequency: Animals might be too young. Frequency and total number change with age and/or disease. In general, older animals have more Treg cells in their abdominal/epididymal fat depots.
- Filter clogged and abnormal big pellet after digestion: Be careful not to include gonads in your digestion. When using old animals with large gonadal fat depots, use 20 ml of fat digestion buffer per animal.

#### 12E.7 Top tricks: Isolation and analysis of Treg cells from fat

- Older animals harbor bigger fat depot, and, in general, a higher frequency and total number of Treg cells can be expected. Use retired breeding animals for fat isolation.
- Treg cells from gonadal fat express Gata-3, while Tconv cells express T-bet. This can serve as a quality control to detect contaminations.

#### 12E.8 Summary of the phenotype

This is shown in Table 42.

#### 12F.3 Step-by-step sample preparation: Isolation and analysis of Treg cells from lung

- Sacrifice animals.
- Expose thorax as well as the abdominal cavity.
- Open inferior vena cava and inject PBS-filled syringe into the right ventricle of the heart and flush with >10 ml PBS to clear the lung circulation; the lung should change from reddish to colorless.
- Excise lungs and move into 10 ml lung digestion buffer using a 50 ml tube.

Collagenase mix for digestion of murine lung tissue

**Table:** 

Name	Concentration	Company	Catalog
Collagenase	1 mg/ml	Sigma-Aldrich	C5138
Type IV			
DNAse I	20 μg/ml	Roche	11284932001
Bovine serum	5 mg/ml	Sigma-Aldrich	A4503
albumin			
DMEM	Fill up to 10 ml	Gibco	41965

- Cut lungs into small pieces with scissors and digest for 30–45 min on a rotating shaker in the incubator (37°C) or in a shaking water bath preheated to 37°C.
- Filter lungs via a 100 μm filter unit into a new 50 ml tube. Add PBS or DMEM to wash the filter and use a syringe plunger to dissociate all tissue pieces.
- Centrifuge for 5 min with 300 × *g* at RT.
- The cellular pellet contains lymphocyte fraction and can be resuspended buffer in 500 μl MACS buffer following filtration.
- Add 20 μl Fc-blocking reagent (e.g., Miltenyi Biotec #130-092-575) and incubate for 5 min at 4°C.
- Add 5 μl anti-CD25 Ab (e.g., Biolegend clone PC61) or anti-CD4 Ab (e.g., Biolegend clone RM4-5) and incubate for 10 min at 4°C.
- Add 500 μl MACS buffer (when using 1.5 ml tube) or 10 ml MACS buffer (when using 15 ml tube).
- Centrifuge for 4 min with 800 × *g* at 4°C.
- Add 50 μl of magnetic-labeled beads in 500 μl MACS buffer and incubate for 10 min at 4°C.
- Add 500 μl MACS buffer (when using 1.5 ml tube) or 10 ml MACS buffer (when using 1 ml tube).
- Centrifuge for 4 min with 800 × *g* at 4°C.
- Filter sample and load onto primed magnetic column.
- Collect eluted cells and stain for sorting or analysis (Figure 43B).

#### 12F.4 Materials: Isolation and analysis of Treg cells from lung tissue

FACS staining buffer:

Base medium 1× PBS
2% Fetal bovine serum

MACS isolation buffer:

Base medium 1× PBS
0.5 % w/v bovine serum albumin (Sigma-Aldrich #A4503)
1 mM EDTA (Roth #X986.2)

MACS separation columns, size L (Miltenyi Biotec #130-042-401)

Anti-biotin ultrapure magnetic beads (Miltenyi Biotec #130-105-637)

Anti-PE ultrapure magnetic beads (Miltenyi Biotec #130-105-639)

Anti-APC magnetic beads (Miltenyi Biotec #130-090-855)

##### Antibodies

1:100 CD8 APC-Cy7 (53-6.7)
1:100 CD19 APC-Cy7 (6D5)
1:200 MHCII APC-Cy7 (M5/114.15.2)
1:500 Fixable Viability dye eF780 (Thermo Fisher eBioscience #65-0865-14)
1:100 CD4-PerCP-Cy5.5 or BUV-395 (RM4-5)
1:100 TCRβ-BV 510 (H57-597)
1:100 CD25 APC (PC61)
1:100 IL-33R/ST2 BV 421 (DIH9)
1:100 Klrg1 BV 711 (2F1)
1:100 Foxp3 AF488 (FJK-16S)
1:100 Gata-3 PE (16E10A23)

Foxp3 Fix/Perm Buffer Set (Thermo Fisher eBiosciences #00-5523-00)

###### Mice

Foxp3^GFP,DTR^ mice (C57BL/6 background) were bred in the animal facility of the Regensburg University Hospital.

###### Cytometer

BD LSRII^™^ 3-laser cytometer (blue-red-violet)

#### 12F.5 Data analysis

BD Flowjo^™^ Version X (10.5.3 Mac OS). The data are shown in Figure 43.

#### 12F.6 Pitfalls: Isolation and analysis of Treg cells from lungs

- Incomplete perfusion of the animal will result in red blood cell contamination. Fast experimental protocols and fast animal handling are required. Do not forget to open the vena cava prior to flushing the circulation with PBS.
- Blood in the thoracic cavity: Do not use cervical dislocation to avoid bleeding into the thoracic cavity. Rupture of the thoracic vessels will make the perfusion more difficult.
- High CD25 or CD4-negative fraction following column-based enrichment: Use Fc-blocking reagents and perform the procedure at 4°C to avoid unspecific binding to beads and columns.

#### 12F.7 Top tricks: Isolation and analysis of Treg cells from lungs

- Be aware of the thymus. The thymus is located in the apex of the heart and in relatively close proximity to the lung tissue; avoid rupturing the thymus to avoid thymocyte contamination. If in doubt, use CD4 and CD8 staining in separate channels to identify CD4^+^CD8^+^ thymocytes. There are almost no CD4^+^CD8^+^ cells in lung tissue, but they are the majority of cells in the thymus.
- Be aware of the mediastinal lymph nodes. Lymph node contamination can be identified by a strong decrease in the proportion of lung tisTregST2 cells (lymph node: <1 %; lung: >10 %) and a general increase in total T and B cell numbers.
- To save reagents, pre-purification of T cells can be skipped; this will increase acquisition times and affect sort efficiency, but has no effect on cell frequency or percentages.

#### 12F.8 Summary of the phenotype

This is shown in Table 43.

#### 12G.3 Step-by-step sample preparation: Isolation and analysis of Treg cells from colon with lamina propria dissociation kit and GentleMACS^®^

**Figure 44.**
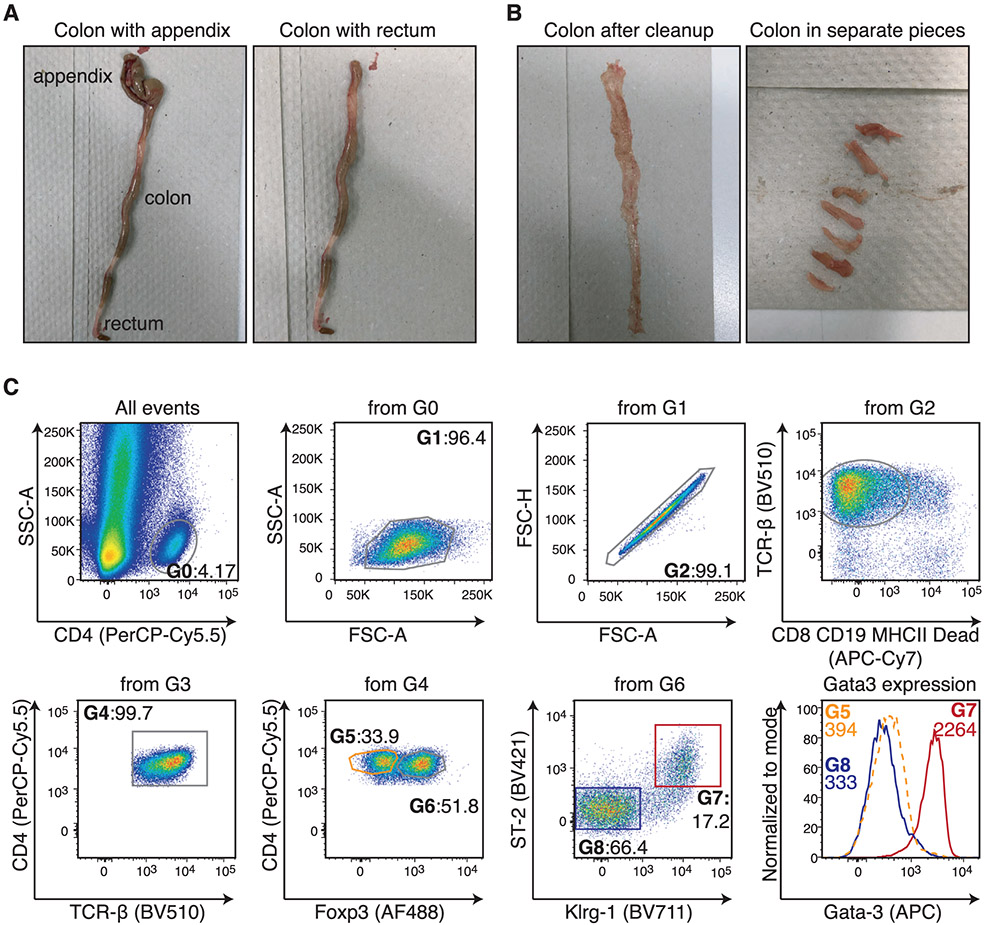
Isolation and analysis of Treg cells from the murine colon tissue. (A) Image of colon tissue after excision. The appendix is still attached (left image) and should be removed (right image). (B) Image of the colon tissue after cleanup (left). Feces have been removed and the colon has been cut longitudinally. The colon is then cut into 1 cm pieces (right) and can be washed. (C) Gating strategy to identify Treg cells in colon tissue. From all events, a CD4-gate to identify T cells can be drawn (gate G0). Based on G0, lymphocytes can be identified by their FSC/SSC properties (gate G1). Next, doublets are excluded (gate G2) as well as unwanted, dead, or autofluorescent cells (gate G3). From G3, CD4^+^TCRβ^+^ T cells (gate G4) are gated, from which Treg cells (gate G6) and Tconv cells (gate G5) can be identified. Finally, Klrg1^+^ST2^+^ tisTregST2 (gate G7) are gated from Treg cells (gate G6). A staining of Gata-3, where numbers indicate geometric mean fluorescence intensity, exemplifies the expression of this marker in Tconv cells (gate G5, orange, dotted line), Klrg1^+^ST2^+^ tisTregST2 cells (gate G7, red), and Klrg1^−^ST2^−^ Treg cells (gate G8, blue). Numbers indicate frequencies of cells within respective gates. Figures are based on colon digestions from Foxp3^DTR, GFP^ animals.

- Sacrifice animals.
- Expose abdominal cavity and excise colon from appendix to rectum; it is usually filled with feces (Figure 44A).
- Remove feces and open colon longitudinally (Figure 44B).
- Cut colon into 1 cm pieces (Figure 44B) and wash two times with pre-digestion and one time with HBSS w/o Ca/Mg with HEPES buffer as described in the methods section of the Miltenyi Biotec lamina propria dissociation kit (Miltenyi Biotec #130-097-410).
- Digest samples in a GentleMACS C tube with respective digestion solution (contents provided in kit) for 25 min with program “37C_m_LDPK_1”.
- Filter sample on a 100 μm filtration unit and mash using a syringe plumber.
- Use more PBS to flush the filter and the C tube.
- Centrifuge for 5 min with 300 × *g* at RT.
- Filter and transfer cells to 1.5 ml tube and in 500 μl MACS buffer.
- Add 20 μl Fc-blocking reagent (e.g., Miltenyi Biotec #130-092-575) and incubate for 5 min at 4°C
- Add 5 μl anti-CD4 Ab (e.g., Biolegend clone RM4-5) and incubate for 10 min at 4°C.
- Add 500 μl MACS buffer (when using 1.5 ml tube) or 10 ml MACS buffer (when using 15 ml tube)
- Centrifuge for 4 min with 800 × *g* at 4°C.
- Add 50 μl of magnetic-labeled beads in 500 μl MACS buffer and incubate for 10 min at 4°C.
- Add 500 μl MACS buffer (when using 1.5 ml tube) or 10 ml MACS buffer (when using 15 ml tube).
- Centrifuge for 4 min with 800 × *g* at 4°C.
- Filter sample and load onto primed magnetic column.
- Collect eluted cells and stain for sorting or analysis

#### 12G.4 Materials: Isolation and analysis of Treg cells from colon tissue

FACS staining buffer:

Base medium 1× PBS
2% Fetal bovine serum

MACS isolation buffer:

Base medium 1× PBS
0.5% w/v bovine serum albumin (Sigma-Aldrich #A4503)
1 mM EDTA (Roth #X986.2)

GentleMACS C tube (Miltenyi Biotec #130-096-334)

Lamina Propria Dissociation Kit (Miltenyi Biotec #130-097-410)

MACS separation columns, size L (Miltenyi Biotec #130-042-401)

Anti-biotin ultrapure magnetic beads (Miltenyi Biotec #130-105-637)

Anti-PE ultrapure magnetic beads (Miltenyi Biotec #130-105-639)

Anti-APC magnetic beads (Miltenyi Biotec #130-090-855)

##### Antibodies

1:100 CD8 APC-Cy7 (53-6.7)
1:100 CD19 APC-Cy7 (6D5)
1:200 MHCII APC-Cy7 (M5/114.15.2)
1:500 Fixable Viability dye eF780 (Thermo Fisher eBioscience #65-0865-14)
1:100 CD4-PerCP-Cy5.5 or BUV-395 (RM4-5)
1:100 TCRβ-BV 510 (H57-597)
1:20 CD25 APC (REA568 for colon samples)
1:100 IL-33R/ST2 BV 421 (DIH9)
1:100 Klrg1 BV 711 (2F1)
1:100 Foxp3 AF488 (FJK-16S)
1:100 Gata-3 PE (16E10A23)

Foxp3 Fix/Perm Buffer Set (Thermo Fisher eBiosciences #00-5523-00)

##### Mice

Foxp3^GFP,DTR^ mice (C57BL/6 background) were bred in the animal facility of the Regensburg University Hospital.

##### Cytometer

BD LSRII^™^ 3-laser cytometer (blue-red-violet)

#### 12G.5 Data analysis

BD Flowjo^™^ Version X (10.5.3 Mac OS). The data are shown in Figure 44.

#### 12G.6 Pitfalls: Isolation and analysis of Treg cells from colon

- Few T cells in colon of young animals: T cell seeding starts from day 10 to 15 after birth. Younger animals have no detectable Foxp3^+^ Treg cell population in the colon.
- Column is clogged: Use a large column (LS) for positive selection of T cells from colon.
- Poor CD25 staining: Use a tested clone for this protocol (e.g., Miltenyi Biotec clone REA568) or stain for Foxp3 intracellularly to identify Treg cells.

#### 12G.7 Top tricks: Isolation and analysis of Treg cells from colon

- Feces can be removed from the intact colon by carefully squeezing the colon with forceps.
- After each 20-min-digestion step in the incubator, the sample is vortexed. Filters can be re-used until they are fully clogged.
- To save reagents, pre-purification of T cells can be skipped; this will increase acquisition times and affect sort efficiency, but has no effect on cell frequency or percentages.

#### 12G.8 Summary of the phenotype

This is shown in Table 44.

As a reference, the tissue staining panels (Figures 41-44) were applied also on a spleen sample (Figure 3D) from the same animal. The population in gate G7 (tisTregST2: CD8^−^CD19^−^MHCII^−^CD4^+^TCRβ^+^CD25^+^Foxp3^+^Klrg1^+^ST2^+^Gata3^+^) comprises less than 2% of all Treg cells found in lymphatic organs. Non-lymphoid tissues showed a clear enrichment for the tisTregST2 population, with organ-to-organ variation (liver: 26.4%, skin: 95.3%, abdominal/epididymal fat: 48.4%, lung: 11.5%, colon: 17.2% of all Foxp3^+^ Treg cells isolated from the individual tissues) (Figures 41-44). The frequency and number of tisTregST2 cells are dependent on the age of the analyzed animals. For example, the frequency of tisTregST2 cells in abdominal/epididymal fat tissue can vary depending on the age of the animals, with older animals showing higher frequencies. In 20–30-weeks old male mice, the frequency of tisTregST2 cells among all Treg cells isolated from the abdominal/epididymal fat depot can be up to 95%. The efficacy of lymphocyte preparations can vary between different methodologies and even between individual preparations.

#### 12G.9 Key differences human versus murine

- Digestion protocols should be optimized for each species; murine digestion protocols might not work in human tissues, and vice versa.
- Both murine and human Treg cells express Foxp3; in the human system, FOXP3 expression can be transiently up-regulated by activation. Addition of Helios can help identify Tregs, see section 11F.3 Step-by-step sample preparation: Isolating CD25^+^FOXP3^+^ Tregs from human thymus and (Figure 36).
- Key phenotypic differences are detailed in Table 45 and the associated references [375, 376, 378, 451, 458, 459]

### 13 Human IL-10 producing regulatory T cells (Tr1 cells)

#### 13.1 Overview

Regulatory T-cells are a minor fraction of the CD4^+^ T-cell compartment and contain excessive immune reactions. Besides the well-defined FOXP3^+^ Tregs also other T-cell populations have been reported to possess regulatory functions. Several different types of FOXP3− regulatory T-cells have been described in humans, but the best-characterized ones are those that produce high amounts of the anti-inflammatory cytokine IL-10. These cells are referred to as type 1 regulatory T-cells (Tr1), and have been studied initially exclusively in *in vitro* cultures. However, recent progress allows now to identify cells with Tr1-like characteristics also directly ex vivo by flow cytometry in human tissues. We will explain here some special features to be considered to induce T-cell IL-10 production and to measure IL-10 by flow cytometry, and discuss strategies to track populations of Tr1-like cells by surface markers, transcription factors, and cytotoxic molecules in human tissues.

#### 13.2 Introduction

Regulatory T-cells that express the lineage-defining transcription factor FOXP3 and the high-affinity receptor for IL-2, CD25, represent approximately 5-10% of CD4^+^ T-cells in human blood, and are required to suppress multi-organ autoimmune diseases (see Section III.11 Human FOXP3^+^ regulatory T cells). Besides FOXP3^+^Tregs, several other T-cell populations were reported to exert regulatory functions, but most of these populations are poorly defined and can thus not be easily monitored by flow cytometry. Similar to CD25^+^Tregs, IL-10 producing regulatory T-cells (“type 1 regulatory T-cells, Tr1”) were identified more than 20 years ago [409, 455]. However, due to the lack of specific surface markers and transcription factors they remained for a long time an enigmatic population, and in humans, they were mainly identified after *in vitro* culture according to IL-10 production [460]. However, a caveat of this approach is that IL-10 is not exclusively produced by regulatory T-cells, and that it can be acquired or lost in culture [461-463]. It is therefore critical to track Tr1-like cells directly *ex vivo,* with no or minimal *in vitro* manipulation. Recent progress in the field have this goal made now feasible also in the human system, and revealed that Tr1-like cells play key roles in several human diseases, including autoimmune diseases [166, 464], IBDs [465, 466], allergy [467], graft-versus-host disease [409], and cancer [468, 469]. Clinical trials to treat graft-versus-host-disease and IBDs with *in vitro* generated Tr1-cells have been performed [470, 471]. We discuss here the different strategies that were reported to identify Tr1-cells by flow cytometry.

##### 13.2.1 Flow cytometric detection of T-cell IL-10 production.

**Figure 45.**
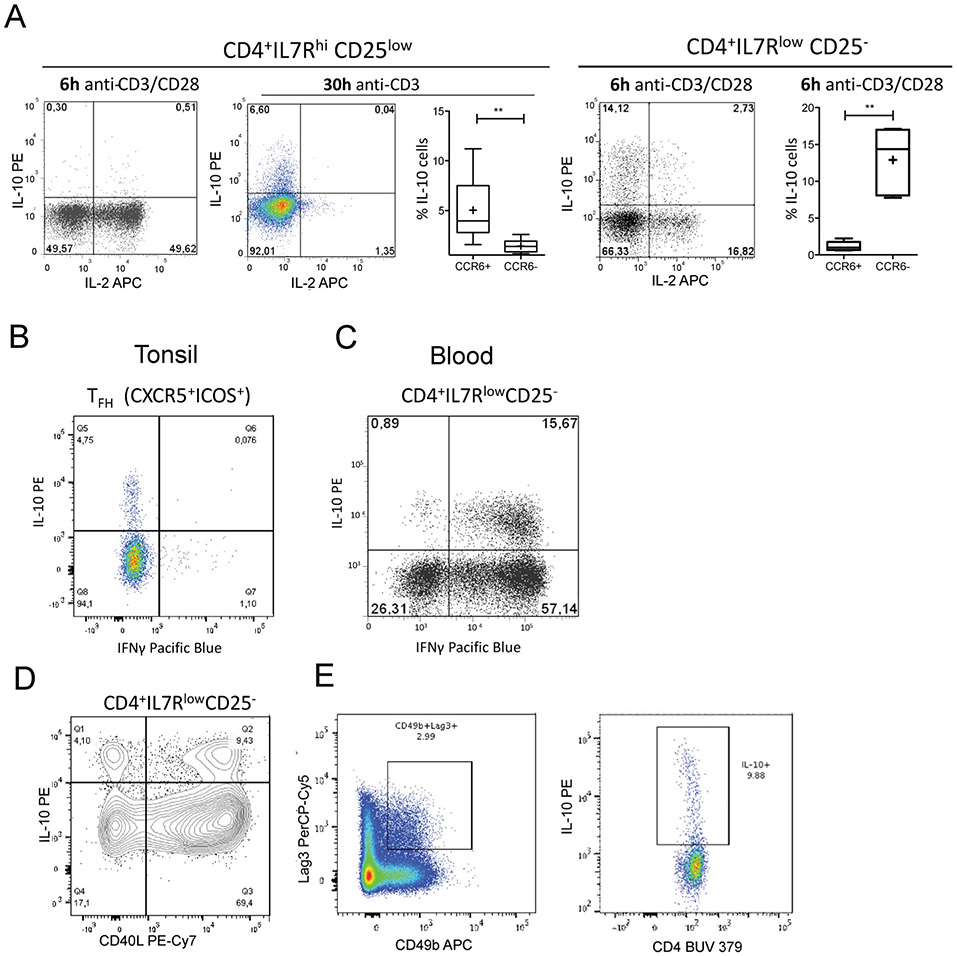
Flow cytometric analysis of IL-10 production by human CD4^+^ T-cell subsets. (A) Conventional human CD4^+^ T-cells (Complete gating strategy see Chapter human CD4^+^T-cells) were isolated according to IL-7R and CCR6 expression and stimulated for 4 or 30 hours with anti-CD3 Abs in the absence or presence of anti-CD28 Abs or 100 U/ml IL-2. The production of IL-2 and IL-10 is shown. The frequencies of IL-10^+^ cells are reported (Statistics: paired student’s t-test, n=5). (B and C) Representative intracellular IL-10 and IFN-γ stainings of FACS-purified CD4^+^CXCR5^+^ICOS^+^ tonsillar TFH-cells (B) or of human blood CD4^+^IL-7R^low^CD25^−^T-cells (C) stimulated with PMA and Ionomycin for 4 h. (D) Same as in C, but IL- 10 was analyzed with a secretion assay and combined with CD40L surface staining. (E) IL-10 secretion of human CD4^+^T-cells following overnight stimulation of total PBMC with SEB gated on CD4^+^LAG3^+^CD49b^+^Tr1- cells. 2010 Häringer et al. Originally published in *J. Exp. Med.* https://doi.org/10.1084/jem.20091021 [163] (Fig. 45A) and https://doi.org/10.1084/jem.20082238 [165] (Fig. 45C).

IL-10 is the characteristic cytokine of Tr1-cells, and is often, but not always [472], associated with regulatory functions. Suppression assays are therefore mandatory to confirm that populations of IL-10 producing T-cells contain indeed Tr1-like cells (see section Tregs/functional assays). IL-10 is a difficult cytokine to be measured with standard intracellular staining protocols (see section IV.10 Treg suppression assays). Indeed, IL-10 production in human CD4^+^ T-cells has a complex regulation, and may require peculiar stimulation conditions. Naïve helper T-cells are devoid of IL-10 producing capacities, and start to produce IL-10 following TCR stimulation in the presence of permissive cytokines only after several days [462, 473]. Conversely, Ag-experienced CD45RO^+^ memory T-cells possess significant IL-10 producing capacities *ex vivo.* The frequencies of IL-10^+^ cells among CD4^+^T-cells after brief polyclonal standard stimulation, like 4–6 h with PMA and Ionomycin or anti-CD3 and anti-CD28 Abs, is however low (approximately 1%) [165]. Consequently, IL-10 is often measured by ELISA, which does not provide though any information on the frequencies or the characteristics of IL-10 producing T-cells. The large majority of Ag-experienced CD4^+^ T-cells in human peripheral blood are resting memory T-cells. IL-10 production in the latter has delayed kinetics when compared to other cytokines [163], and is consequently hardly detectable in response to brief polyclonal standard stimulation (Figure 45A). However, IL-10 production by memory T-cells can be quite efficiently induced by a more sustained TCR stimulation. Thus, stimulation of purified CD4^+^ memory T-cells with anti-CD3 Abs for 30 h [163], or super-Ag stimulation overnight induces more robust frequencies of IL-10^+^ T-cells (approximately 5%). Notably, some memory T-cells can produce IL-10 in the absence of CD28 co-stimulation. This anti-CD3-induced IL-10 production requires however IL-2, and is largely confined to CCR6^+^ T-cells [163] (Figure 45A). *In vitro* or *in vivo* activated T-cells in contrast produce IL-10 after brief polyclonal standard stimulation. Thus, tonsillar TFH-cells, i.e., activated B helper effector cells (see section Human CD4 T cells), produce IL-10 upon brief PMA and Ionomycin stimulation (Figure 45B). CD4^+^ effector T- cells in peripheral blood of healthy donors are rare, but are present among CD25^−^ IL-7R^−^ cells and produce rapidly high levels of IL-10 together with IFN-ϒ [165] (Figure 45C). Notably, also FOXP3^+^ Tregs in human peripheral blood are activated cells, and produce rapidly some IL-10 (<5%) [165]. Intracellular staining inevitably kills the analyzed cells, but viable IL-10 producing T-cells can be purified with a cytokine secretion assay [474]. The latter allows to isolate T-cells according to the secretion of up to two cytokines, and can thus be exploited to isolate for example IL-10 and IFN-ϒ co-producing T-cells [461]. Moreover, it can be combined with surface markers. An efficient approach to isolate IL-10 producing T-cells that can suppress B-cell responses is the combination of IL-10 secretion and the lack of the helper molecule CD40L [166] (Figure 45D). CD40L is upregulated by virtually all helper T-cells upon activation [198], but FOXP3^+^ Tregs and terminally differentiated IL-7R^−^ Tr1/effector cells have lost this capacity [166, 475]. IL-10 secreting cells that suppress CD4^+^ T- cell proliferation can also be purified according to Tr1-associated surface markers, like CD49b and LAG3 [415], after stimulation of total CD4^+^T-cells with super-Ag overnight [466] (Figure 45E). In conclusion, IL-10 production can be quantified by flow cytometry, but it is critical to use appropriate stimulation conditions for the population of interest.

##### 13.2.2 Phenotypic markers to enrich for IL-10 producing Tr1-like cells in human tissues and diseases.

**Figure 46.**
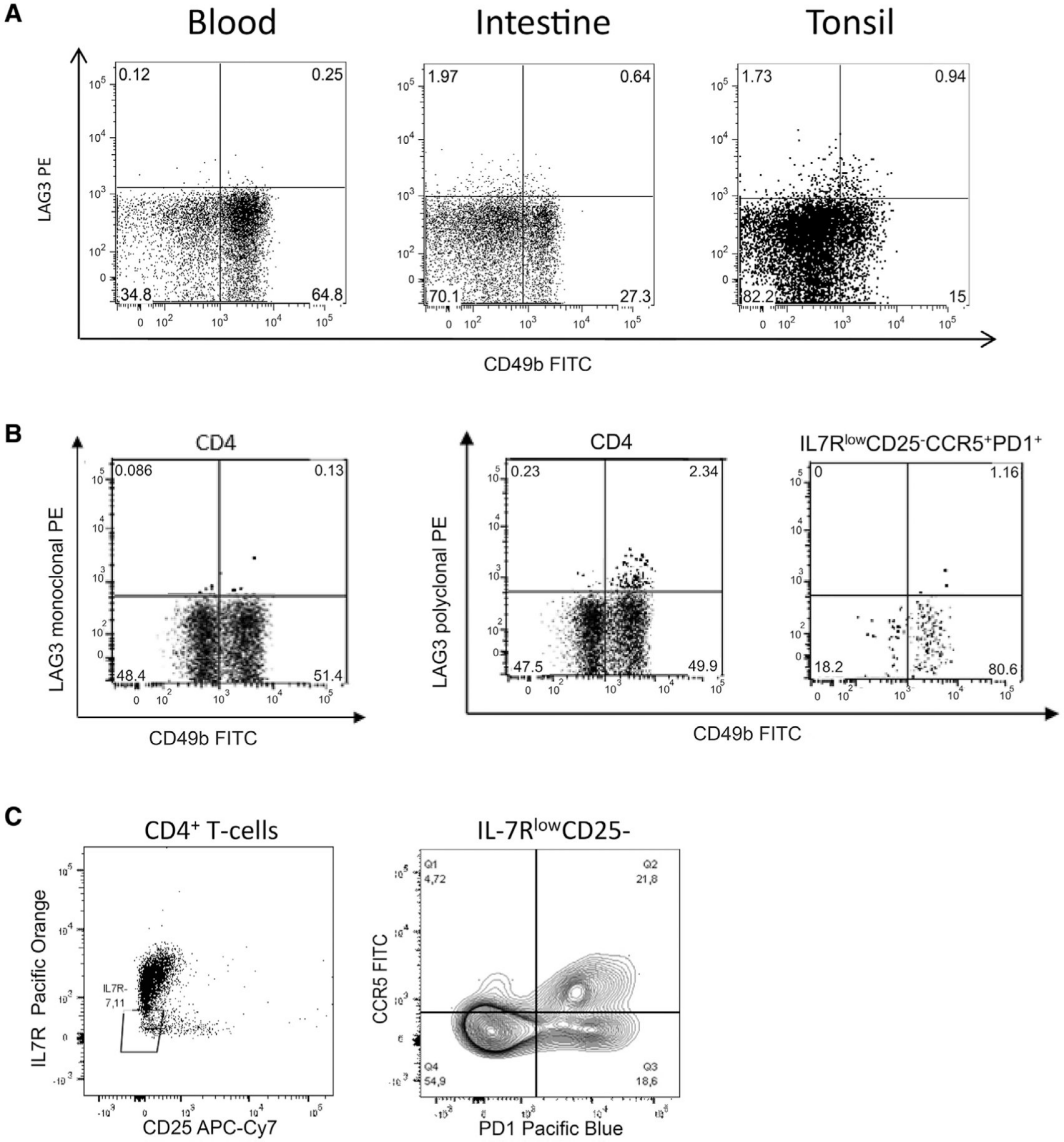
Expression of phenotypic markers associated with human Tr1-cells. (A) LAG3 and CD49b surface stainings of human peripheral blood of a healthy donor, in an inflamed tonsil and in the intestinal lamina propria *ex vivo*. (B) *Ex vivo* LAG3 and CD49b surface staining with a polyclonal or a monoclonal anti-LAG3 Ab on total CD4^+^ T-cells, or on gated IL-7R^−^CCR5^+^ Tr1-like cells in human peripheral blood of a healthy donor. C CCR5 and PD1 co-expression among gated CD4^+^IL-7R^low^CD25^−^T-cells allows to enrich for Tr1-like cells in different tissues. Shown is peripheral blood of a representative healthy donor. 2010 Rivino et al.

**Figure 47.**
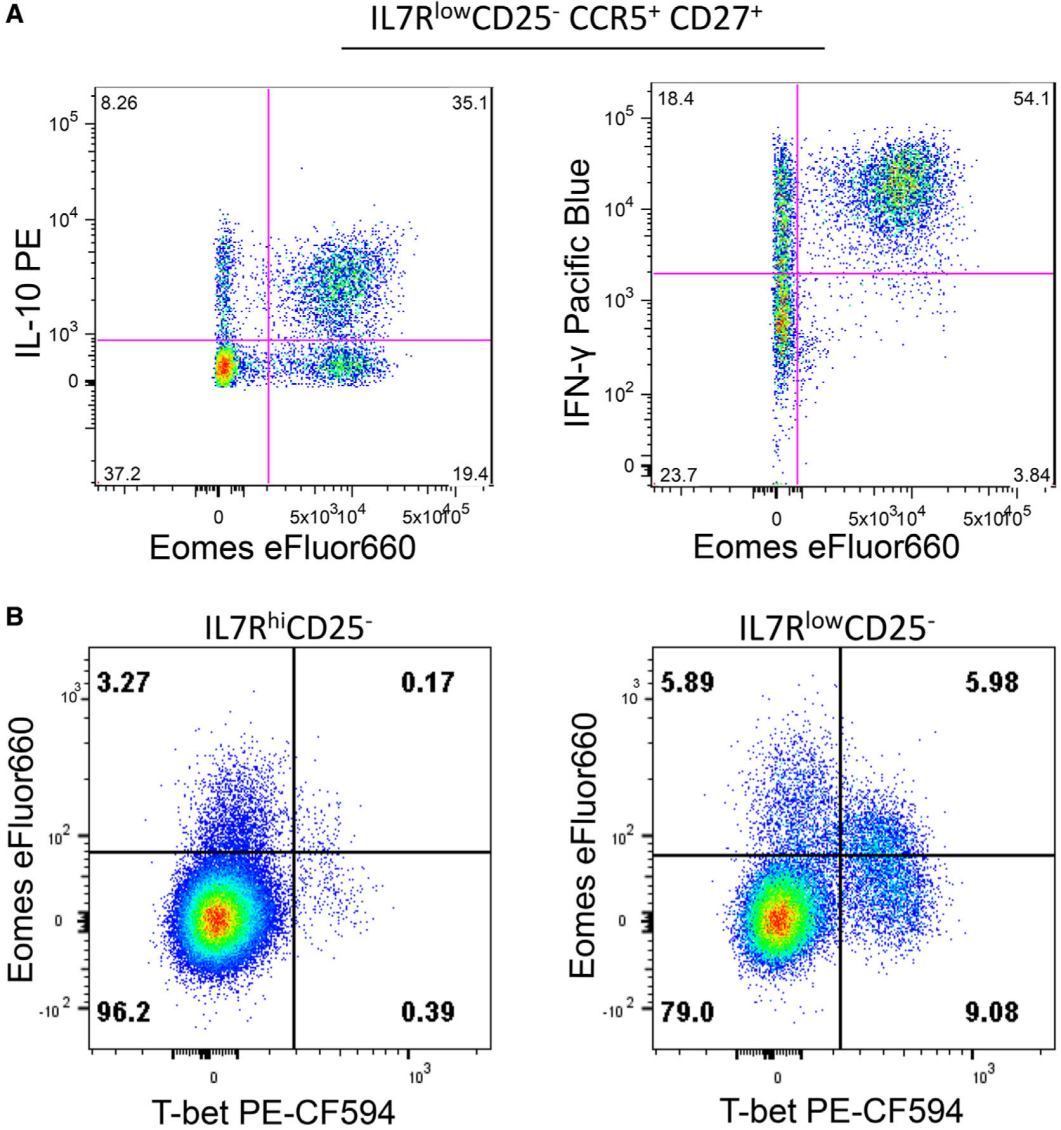
Human Tr1-like cells tracked by intracellular staining for transcription factors. A. Intracellular Eomes versus IL-10 or IFN-γ expression in FACS-purified human blood CD4^+^IL-7R^low^CD25^−^ CCR5^+^CD27^+^Tr1-like cells after PMA and Ionomycin stimulation for 4 h. (B) *Ex vivo* Eomes versus T-betexpression in PBMC gated on conventional CD4^+^T-cells according to IL-7R expression. Among IL-7R^low^CD25^−^ CD4^+^ T-cells, Tr1-like cells can be identified as Eomes^hi^T-bet^lo^, while CTL are T-bet^hi^Eomes^lo^.

Phenotypic markers that are associated with IL-10 production allow to enrich for IL-10 producing T-cells with regulatory functions without activating the TCR. The most used surface marker to track and enrich Tr1-cells is LAG3, an activation-induced co-inhibitory receptor, alone or in combination with the integrin CD49b [415, 476]. CD49b and/or LAG3 were used to track and purify suppressive Tr1-cells from peripheral blood [415], tonsils [477], and the intestinal *lamina propria* [466]. CD49b^+^ LAG3^+^ T-cells are rare in peripheral blood of healthy donors, but increase in inflamed tissues (Figure 46A). Caveats of LAG3 surface staining are the different frequencies of LAG3^+^ cells that are obtained with the original polyclonal [415] and the more recent monoclonal Abs (Figure 46B), and the fact that LAG3 may be cleaved from the surface by proteolysis [478] (see also Tips and Tricks). An alternative strategy that identifies IFN-ϒ and IL-10 co-producing Tr1-like cells is to gate “conventional” CD25^−^ CD4^+^ T-cells that have down-regulated IL-7R expression and that co-express the Th1- associated chemokine receptor CCR5 and the co-inhibitory receptor PD1 [166, 465] (Figure 46C) (see also Tips and Tricks). Tr1-like cells with this phenotype can produce very high levels of IL-10 following brief polyclonal stimulation *ex vivo* (Figure 47A), although the frequencies of IL-10^+^ cells vary strongly in individual donors [166]. Notably, cytotoxic CD4^+^T-cells (CTL) have a very similar phenotype, but can be discriminated since they lack CD28 and CD27 expression [169] (see also section human CD4^+^ T-cells). IL-7R^−^ CCR5^+^ Tr1-like are rare in peripheral blood of healthy donors and in tonsils (<1%) [166], but are increased in the circulation of SLE patients (>1%) [166] and in the intestine (approx. 2%) [465], and can become quite abundant (>5%) in tumors [468].

The publication of two different strategies to identify human Tr1-cells raises the question if the reported surface markers are largely redundant, since most of these markers reflect chronic and/or recent activation. Indeed, LAG3, CD49b, CCR5, and PD1 are co-expressed on Tr1-cells in a mouse colitis model [465] (see section Murine Tr1-cells), and in a relevant fraction of human Tr1-cells identified according to IL-10 secretion following overnight stimulation with super-Ags [466]. In contrast, in unstimulated peripheral blood of healthy donors the overlap between CD49b^+^LAG3^+^ and IL-7R^−^ CCR5^+^ PD1^+^ Tr1-cells is surprisingly minimal [465] (Figure 46B). In other words, in this experimental standard condition, CD49b^+^LAG3^+^ and IL-7R^−^ CCR5^+^ PD1^+^ Tr1-cells represent unfortunately two largely distinct populations. Notably, the frequencies of IL-7R^−^CCR5^+^ T-cells that co-express LAG3 on the cell surface are often increased in inflamed human tissues. Nevertheless, given the heterogeneity of Tr1-cells, the co-expression of Tr1-associated surface markers with IL-10 should first be experimentally determined in order to identify the best strategy for the tracking or purification of Tr1-cells, in particular in tissues or patients where Tr1-cells have not been previously analyzed.

##### 13.2.3 Transcription factor expression in Tr1 cells.

The identification of the lineage-defining transcription factor FOXP3, which is selectively expressed in CD25^+^ Tregs and required for their function, was a milestone in the field of regulatory T-cells [479]. A Tr1-specific transcription factor with the same characteristics was unfortunately not identified, possibly because Tr1-cells are more heterogeneous. Several transcription factors have been shown to regulate T-cell IL-10 production and could thus in principal be exploited to track Tr1-cells. However, these transcription factors are normally not unique for Tr1-cells, and are often broadly expressed among human CD4^+^ T-cells. Thus, c-Maf regulates not only IL-10 production by Tr1-cells, but is also critical for IL-4 production and the generation of TFH-cells [480-482]. Similarly, AHR controls not only regulatory T-cell differentiation, but also IL-22 production in Th17/22-cells [170, 483]. Blimp-1 is an effector T-cell-associated transcription factor that is required for IL-10 production by Tr1-cells [484-486], but it is unfortunately difficult to detect by intracellular staining in human CD4^+^ T-cells. In murine GvHD, the T-box transcription factor Eomesodermin (Eomes) was shown to control generation and IL-10 production of Tr1-cells in concert with Blimp-1 [487]. Eomes is also highly expressed in human IL-7R^−^Tr1-like cells, acts as a lineage-defining transcription factor [169, 487-490] and can be easily analyzed by flow cytometry (Figure 47A). Notably, also CD4^+^CTL express Eomes, but in contrast to Eomes^+^Tr1- like cells they express also high levels of T-bet [169]. Thus, the combination of IL-7R, T-bet, and Eomes is a simple and powerful strategy to track IFN-ϒ producing Tr1-like cells (as IL-7R^lo^Eomes^hi^ T-bet^lo^, Figure 47B) in peripheral blood and in other human tissues [169]. Notably, Lag3^+^ Tr1-cells express only low levels of Eomes *ex vivo* [169], but they were reported to express the transcription factor Egr-2 in mice and in humans [476]. However, a strategy to track human Lag3^+^ Tr1-cells according to transcription factor expression by flow cytometry has to our best knowledge not yet been reported.

##### 13.2.4 Cytotoxic molecules expressed by Tr1-cells.

**Figure 48.**
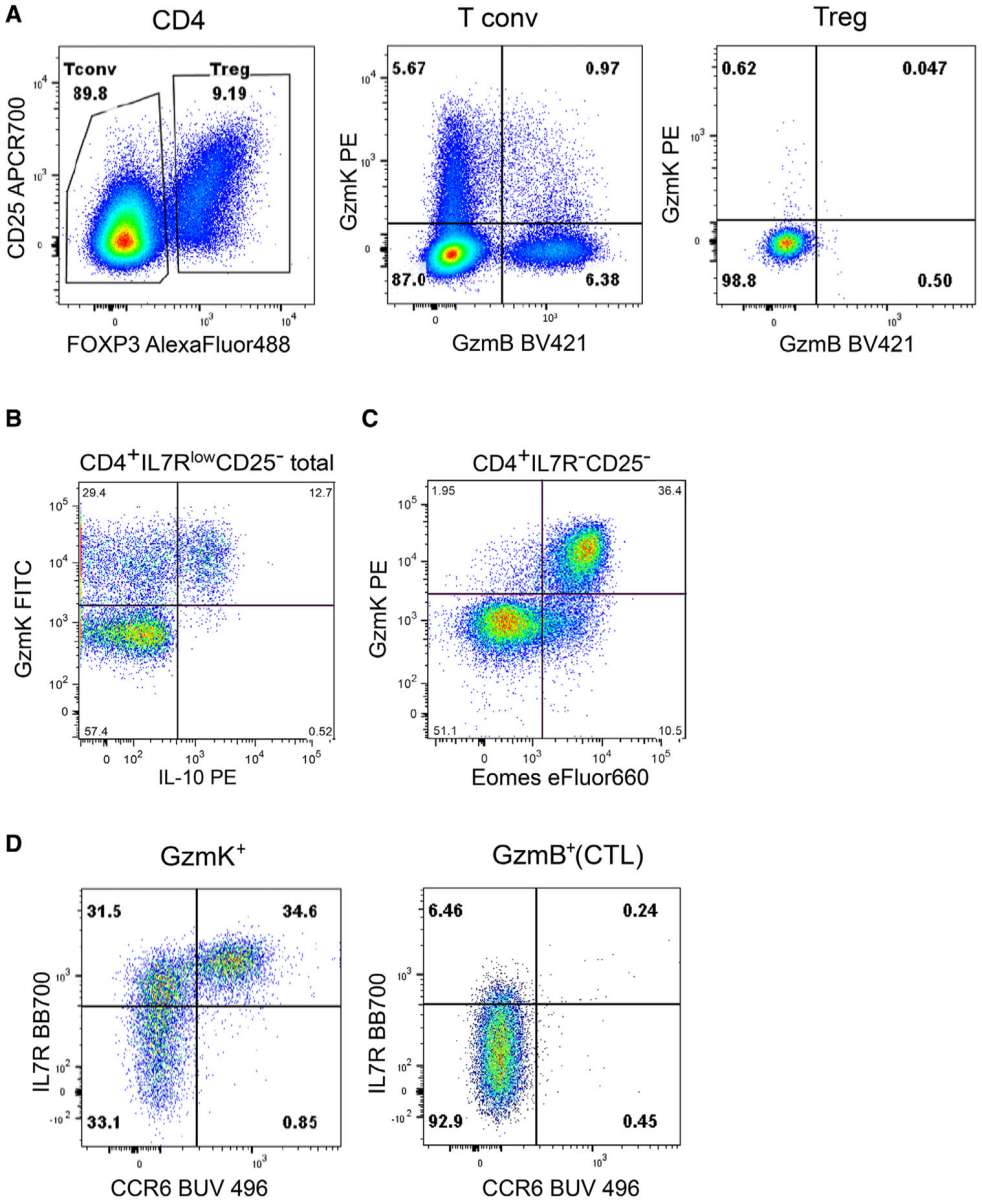
Human Tr1-like cells tracked by intracellular staining for cytotoxic molecules. (A) *Ex vivo* GzmK and GzmB expression in gated conventional CD4^+^T-cells and FOXP3^+^Tregs. (B) GzmK and IL- 10 co-expression in FACS-purified CD4^+^IL-7R^low^CD25^−^T-cells after 4 hours of stimulation with PMA and Ionomycin. (C) Co-expression of GzmK and Eomes in FACS-purified CD4^+^IL-7R^low^CD25^−^T-cells *ex vivo* D: *Ex vivo* IL-7R and CCR6 expression patterns in among conventional CD4^+^ T-cells gated as GzmK^+^ or GzmB^+^ (see A).

Tr1-cells have been consistently reported to possess cytotoxic functions. In particular, they can kill myeloid APC via a perforin-dependent pathway [491], and were reported to express the cytotoxic molecule GzmB, but not GzmA [492]. A caveat of these studies is, however, that the analyzed Tr1-cells were generated and activated *in vitro*, since GzmB is rapidly induced upon *in vitro* stimulation in human CD4^+^ T-cells [492]. In addition, the expression patterns of GzmB might be different in humans and mice. GzmB can be easily stained intracellularly in conventional, FOXP3^−^ CD4^+^ T-cells from human peripheral blood in the complete absence of *in vitro* stimulation (Figure 48A). These *in vivo* occurring GzmB^+^ CD4^+^ T-cells correspond to terminally differentiated CD4^+^CTL that lack CD27 and CD28 expression and express high levels of Granulysin [468], perforin and GzmA [493] (see also section III.1 Human conventional CD4^+^ T cells). Notably, *ex vivo* isolated CD4^+^ GzmB^+^ CTL are largely devoid of IL-10 producing and suppressive capabilities [169]. This is surprising, since IL-7R^−^ Tr1-cells express Eomes, which controls cytotoxic lymphocyte functions [487, 494] and is involved in the regulation of GzmB expression. Indeed, Eomes^+^ Tr1-like cells do possess cytotoxic functions, but they lack GzmB *ex vivo* and exhibit instead high levels of GzmK (Figure 48A, B) and also of GzmA [169]. The selective expression of GzmK in Tr1-cells and of GzmB in CD4^+^ CTL is conserved in different human tissues and tumors and can thus be exploited to monitor these 2 cytotoxic T-cell subsets *ex vivo* [169]. GzmB and perforin were also reported to be expressed in FOXP3^+^ Tregs in mice, in particular in tumor models [495], but circulating human FOXP3^+^ Tregs express only very low levels of GzmB ([496], Figure 48A). Nevertheless, it is recommended to exclude FOXP3^+^Tregs before analyzing “conventional” CD4^+^ T-cells for the expression of GzmK and GzmB (Figure 48A). GzmK expression among CD4^+^T-cells is largely restricted to Eomes^+^ cells (Figure 48C), and largely absent from CD4^+^CTL. Thus, if the number of parameters that can be analyses simultaneously by flow cytometry is limiting, GzmK is actually the most reliable single marker to track Tr1-like cells among CD4^+^ IL-7R^low^T-cells. Notably, among total GzmK^+^ CD4^+^ T-cells only a subset corresponds to Eomes^+^ Tr1-like cells, which can however be easily identified by the low levels of IL-7R surface expression (Figure 48D). GzmK^+^ Eomes^+^ Tr1-like cells lack CCR6 (Figures 45A and 48D) and CD161 expression, while a relevant fraction of IL-7R^+^ GzmK^+^ cells co-expresses CCR6 (Figure 48D) and CD161. This heterogeneity reconciles an apparently conflicting report on the expression of Eomes in human “unconventional” (CCR6^+^ CD161^+^) Th1-cells [497], which produce GM-CSF, but not IL-10 and possess thus pro-inflammatory properties. In conclusion, intracellular staining for GzmK, alone or in combination with Eomes, among CD4^+^ CD25^−^ IL-7R^low^ effector T-cells allows to monitor a population of Tr1- like cells *ex vivo,* without the absolute need to induce IL-10 by *in vitro* stimulation. This is particularly helpful in tissues and diseases where the number of viable T-cells is limiting, or where Tr1-cells down-regulate IL-10 production, like the inflamed intestine of IBD patients [465].

#### 13.3 Step-by-step sample preparation

##### 13.3.1 Isolating PBMC.

1. Isolate PBMC from heparinized blood or buffy coat by using Ficoll-Paque according to manufacturer’s protocol.
2. Collect the PBMC ring in 50 mL tubes.
3. Add PBS up to 50 mL and centrifuge for 8 min at 515 × *g* at room temperature (RT).
4. Decant supernatant, resuspend pellet up to 50 mL of phosphate-buffered saline (PBS) and centrifuge for 10 min at 200 × *g* at RT.
5. Count cells and adjust concentration to 2.5–5 × 10^6^ cells/mL. Note: continue skip the point 2 for the (ex-vivo) surface phenotype.

##### 13.3.2 Stimulating PBMC for the detection of cytokines.

1. Transfer up to 5 × 10^5^ PBMC to a 96-well U bottom plate (3788, Corning) in 200 μL of culture medium (RPMI containing 10% FCS, 1× nonessential aminoacids, 1× Na pyruvate and glutamax)
2. Then add Ionomycin (500 ng/mL) and PMA (50 ng/mL) to the correct wells.
3. Incubate for 4 h in a cell incubator at 37°C, 5% CO2.
4. After 90 min, add Brefeldin A (BFA; 10 μg/mL) in order to block the secretion pathway and retain cytokines intracellularly.
5. At the end of incubation, centrifuge plate for 5 min at 450 × *g* at RT
6. Decant supernatant, re-suspend cells in 200 μL *PBS* and continue with point 3.2.

##### 13.3.3 Surface staining.

1. Transfer up to 1 × 10^6^ PBMC to a 96-well V bottom plate (Greiner BioOne).
2. Centrifuge the plate for 5 min at 450 × *g* at RT.
3. Meanwhile prepare surface staining mix (containing a pretitrated appropriate amount of Ab and including the *live/dead exclusion dye*) in a total volume of 35 μL *Brilliant Staining Buffer (BSB, BD)* for each well (prepare 1× extra every 5).
4. Decant supernatant and add 35 μL surface staining Ab-cocktail for each well gently resuspending cells by pipetting 3 times.
5. Incubate for 20 min at RT, protected from light.
6. Add 200 μL *PBS* and centrifuge at 450 × *g* at RT for 5 min.
7. Decant supernatant and add 150 μL *PBS* gently resuspending cells by pipetting to analyze by flow cytometry or continue with the intracellular staining protocol.

##### 13.3.4 Intracellular stainings of cytoplasmatic molecules and transcription factors.

1. After the wash 3.6, decant the supernatant and add 100 μL 1 × *Fixation/Permeabilization* buffer.
2. Gently resuspend the cells by pipetting up and down 5 times.
3. Incubate for 20 min at RT, protected from light.
4. Add 100 μL of 1× *Permeabilization/Wash Buffer* directly into the well, without washing the cells.
5. Centrifuge for 5 min at 580 × *g* at RT
6. Decant supernatant and resuspend cells by pipetting 3 times in 35 μL of the intracellular staining mix prepared in 1× *Permeabilization/Wash Buffer.*
7. Incubate 50 min at 4°C, protected from light.
8. Add 150 μL 1 × *Permeabilization/Wash Buffer* to each well and centrifuge for 5 min at 580 × *g* at 4°C.
9. Aspirate supernatant and resuspend cells in 150 μL *PBS* and analyze by flow cytometry.

##### 13.3.5 Sorting and secretion assay for IL7R-10+ Tr1 cells.

In order to isolate Eomes+Tr1-like cells (IL-7R^−^IL10^+^) cells a double round of sorting is recommended (see section 13.2.1).

###### 13.3.5.1 Day one sorting.

1. Isolate CD4^+^ T cell using human CD4^+^ T Cell Isolation Kit (Miltenyi, cat number 130-096-533) according to manufacturer’s instructions.
2. Re-suspend pellet up to 50 mL of PBS and centrifuge for 10 min at 200 × *g* at RT.
3. Collect and count cells; stain for surface markers: CD4, CD127, CD25 for 20 min at 37°C.
4. Wash cells with 10 mL of PBS and centrifuge for 10 min at 200 × *g* at RT.
5. Proceed to the first round of FACS sorting (70 μM nozzle) to isolate CD4^+^ CD127^low^ CD25^−^ cells (CD4^+^ CD127^+^ CD25^−^ and CD127loCD25+Tregs may be sorted as control cells).
6. Transfer up to 3 × 10^5^ sorted cells to a 96-well U bottom plate (3788, Corning) in 200 μL of culture (RPMI added with 5% Human serum) and let them rest ON at +37°C 5% CO_2_.

###### 13.3.5.2 Day two secretion assay and sorting.

1. The day after stimulating cells with PMA/Ionomycin as in paragraph 2, without blocking the secretion (skip point 2.4).
2. At the end of incubation collect stimulated cells in a 15 mL tube and proceed with secretion assay for IL- 10 (Miltenyi, human IL-10 Secretion Assay-Detection Kit, cat. Number 130-090-434) following the manufacturer’s instruction. Be careful and add opportune quantity of anti-CD40-L Ab after the incubation with IL-10 detection primary Abs.
3. Wash cells and proceed to a second round of FACS sorting to isolate IL10^+^ CD40L^−^ Tr1 cells

Alternatively, Tr1 can be isolated from total CD4+T-cells according to surface markers, such as Lag3 and CD49b, and IL10 secretion. CD4^+^ T-cells are first purified using CD4-coated beads (Miltenyi).

1. CD4^+^ Tcells are re-stimulated overnight in full media (1×10^6^ cell/mL) supplemented with staphylococcal enterotoxin B (SEB) 1μg/mL at 37°C, 5%CO2.
2. Perform human Miltenyi secretion assay as stated from point 5.7 to 5.8 counterstaining with TR1 associated surface markers Lag3 and CD49b.
3. Wash cells and proceed to FACS sorting to isolate Lag3^+^CD49b IL10^+^ Tr1 cells

#### 13.4 Materials

##### 13.4.1 Live/dead exclusion dye.

Fixable Viability Stain 780 (FVS780, BD).

##### 13.4.2 Antibodies.

These are detailed in Tables 46 and 47

##### 13.4.3 Flow cytometer.

Experiments were performed either on a FACS canto or on a BD FACSymphony flow cytometer with five lasers (488 nm, 561 nm, 640 nm, and 405 nm and 355 nm), 29 colors (6-5-3-8-7) configuration (BD Bioscience).

Filters(laser):

- 780/60(488) for BB790-p; 750/30 for BB755; 710/50(488) for BB700 or PerCP-eFluor710; 670/30 for BB660; 610/20 for BB630; 530/30(488) for FITC or Alexa Fluor 488.
- 780/60(561) for PE-Cy7; 710/50(561) for PE-Cy5.5; 670/30(561) for PE-Cy5; 610/20(561) for PE- CF594; 586/15(561) for PE.
- 680/60(640) for FVS780 or APC-Cy7 or APC-Fire750 ; 730/45(640) for APC-R700; 670/30(640) for APC or eFluor 660 or Alexa Fluor 647.
- 780/60(405) for BV786; 750/30(405) for BV750; 710/50(405) for BV711; 677/20(405) for BV650;
- 605/40(405) for BV605; 586/15(405) for BV570; 525/50(405) for BV510 or Pacific Orange; 450/50(405) for BV421 or eFluor450 or Pacific Blue.
- 810/40(355) for BUV805; 735/30(355) for BUV737; 670/25 for BUV661; 605/20 for BUV615; 580/30 for BUV563; 515/30 for BUV496; 379/28 for BUV395.

##### 13.4.4 Reagents.

- Ficoll-Hypaque Plus (GE Healthcare, endotoxin tested, cat. no. 17-1440-03)
- FCS (fetal calf serum) batch-tested for low endotoxin
- PBS 1× (Gibco DPBS, no calcium, no magnesium; cat. no. 14190144)
- Culture medium: RPMI 1640
  2 mM glutamine
  1% non-essential amino acids 1 mM sodium pyruvate
  50 μM β-mercaptoethanol
  1% penicillin/streptomycin
  5% human serum (HS) or 10% FCS
- Washing medium:
  RPMI-1640 w/ Hepes (25 mM) 1% FCS or 0.5% HS
- Flow cytometry buffer (FACS buffer):
  Phosphate buffered saline (PBS 1×) 2.5% FCS or 1% HS

0.01% (w/v) sodium azide (to be added in the case of long-term storage) 2mM EDTA pH 8.0 (to prevent clots)

- Stimulation mix:
  Culture medium
  1 μg/mL ionomycin (Sigma-Aldrich, cat. no. I0634):
  2 × 10^−7^ M PMA (Sigma-Aldrich, cat. no. P8139)
  10 μg/mL BFA (Sigma-Aldrich, cat. no. B7651)
- Cytofix/Cytoperm 1× solution (BD Biosciences; cat. no. 554722)
- 1× Perm/Wash (always prepare freshly before use): 10% 10× perm/wash (BD Biosciences; cat. no. 554723) 90% ddH2O
- 1× Fixation Buffer working solution (eBioscience Foxp3 / Transcription Factor Staining Buffer Set; cat. 00-5523-00) for intra-nuclear staining of transcription factors:

75% Fixation/Permeabilization Diluent (component cat. 00-5223)

25% 4x Fixation/Permeabilization Concentrate (component cat. 00-5123)

- 1× Permeabilization Buffer working solution (eBioscience Foxp3/Transcription Factor Staining Buffer Set; cat. 00-5523-00) for intra-nuclear staining of transcription factors:
  10% 10× Permeabilization Buffer (component cat. 00-8333) 90% ddH_2_O
- 1× Fixation Buffer working solution (BD Pharmingen Transcription Factor Buffer Set, cat. 562574) for intra-nuclear staining of transcription factors:

75% TF Fix/Perm Diluent Buffer (component cat. 51-9008101)

25% 4× TF Fix/Perm Buffer (component cat. 51-9008100)

- 1× Permeabilization Buffer working solution (BD Pharmingen Transcription Factor Buffer Set, cat. 562574) for intra-nuclear staining of transcription factors:
  20% 5x TF Perm/Wash Buffer (component cat. 51-9008102)
  80% ddH2O

#### 13.5 Data analysis

This is detailed in Figures 45-48.

#### 13.6 Top tricks

##### 13.6.1 Top tricks Isolating PBMC (see section 13.2.1).

- When possible, use Sodium Citrate or heparin as anticoagulant. EDTA may interfere with cytokine production.
- See also section II.1 Human conventional CD4^+^ T cells for additional tricks

##### 13.6.2 Top tricks Surface staining.

- Some surface receptors, like IL-7R or LAG3, may be lost or acquired during the stimulation period. If ex vivo expression is critical, cells may be sorted first according to phenotypic markers, and then stimulated.
- Ex vivo purification: Most IL-7R^−^CCR5^+^ Tr1-like cells express PD1 *ex vivo* (Figure 46 C), and therefore PD1 only moderately improves their enrichment. Moreover, anti-PD1 Abs used for surface staining are often neutralizing Abs, and may thus interfere with suppressive capabilities [468]. In contrast, CD27 allows the separation of Tr1-cells from CTL and should always be included. In addition, exclusion of CCR6^+^T-cells may be helpful to eliminate contaminating pro-inflammatory T-cells.
- LAG3 surface staining works very well with *in vitro* activated T-cells, but is challenging ex vivo. Fresh blood may give better results than buffy-coated blood. The use of a monoclonal Ab is recommended.
- IL-7R staining is critical for the analysis Eomes^+^Tr1-like cells, so the anti-IL-7R Ab should be tested and titered. Since IL-7R expression is not bimodal, co-staining with CD25 or FOXP3 is helpful to set the gate for IL-7R^low^ T-cells. Notably, the cells that have completely lost IL-7R expression produce the highest levels of IL-10.
- See also section II.1 Human conventional CD4^+^ T cells for additional tricks

##### 13.6.3 Top tricks: Intracellular stainings of cytoplasmatic molecules and transcription factors.

- After step 4.3 cells can be washed and stored in PBS at + 4°C. To continue the protocol is important to resuspend cells in 200 ul of Permeabilization/Wash Buffer for 15 min.
- The use of the eBioscience Set is generally preferable. The BD Pharmingen Set allows a better visualization of transcription factors. Howewer, the BD Fixation Buffer contains methanol and it is harmful for some fluorochromes (e.g., PE and APC and relative tandems, BB700) that have been used for the surface staining.
- To see the cytokines only the use of classic protocol of fixation with PFA 2% and permeabilization with Saponin 0.05% is sufficient.

##### 13.6.4 Top tricks: Sorting and secretion assay for IL7R-10^+^ Tr1 cells (section IV.8 Live cytokine-producing cell sorting with cytokine secretion assay^™^).

- Secretion assay: work fast and use media at the indicated temperature. Up to two different cytokines can be assessed simultaneously.
- If you do not have rotation device for tubes be sure to gently mix the tubes each 5 min for all the times of incubation.
- Before sorting pass the cells through a filter (50 micron filter siringe type; 340601, cat number 340601) to avoid clumps.
- Very high frequencies of cytokine-secreting cells (>50%) may lead to false-positive events.

### 14 Murine Tr1 cells

#### 14.1 Overview

CD4^+^ type 1 regulatory T (Tr1) cells represent a suppressive cell population that is induced in the periphery after Ag recognition. In the following sections, we describe strategies to identify murine Tr1 cells under steady-state conditions in the small intestine and during acute infection in the liver.

#### 14.2 Introduction

Tr1 cells are suppressive cells that can be identified by the expression of the cytokine IL-10, absence of the transcription factor FOXP3 and the co-expression of several co-inhibitory receptors, e.g., LAG-3 and PD1, the chemokine receptor CCR5 and the integrin CD49b [460]. Key features of Tr1 cells are summarized in Tables 51 and 52.

IL-10 expression alone is not sufficient to identify Tr1 cells, since IL-10 positive cells with low expression of co-inhibitory receptors are not suppressive and in some settings even pro-inflammatory [466]. Whether co-inhibitory receptors serve not only as markers for Tr1 cells, but also contribute to their suppressive function *in vivo* still needs to be investigated. Of note, the suppressive capacity of Tr1 cells is the key functional aspect for their identity. Therefore, cellular characterization of Tr1 cells should be backed up by functional testing of the identified cells e.g., by *in vitro* and/or *in vivo* suppression assays.

#### 14.3 Step-by-step sample preparation

Tr1 cells can be isolated from various secondary lymphoid and non-lymphoid tissues. Considering that the development of Tr1 cells relies on antigenic stimulation, during steady-state conditions Tr1 cells are mainly restricted to barrier sites such as the small intestine, but are only rarely found systemically, e.g. in spleen or blood. The frequency of Tr1 cells under steady-state conditions depends – in our experience – on the microbiota and might differ across animal facilities and institutes. Here, we show examples for Tr1 identification using IL-10 Foxp3 double reporter mice in the small intestine under steady-state conditions, and in a mouse model of malaria, a disease well known to be associated with Tr1 cells both in mouse and human [498]. Given the central role of the function of Tr1 cells for their identification, we also provide a protocol for an *in vitro* suppression assay.

Depending on the available tools, IL-10 can either be detected using IL-10 reporter mice (e.g., by the expression of IL-10 eGFP using Tiger mice [499]), intracellular cytokine staining [500], or an IL-10 secretion assay [466]. The intracellular staining has the disadvantage to not allow for functional validation since the cells need to be fixed and permeabilized.

**Figure 49.**
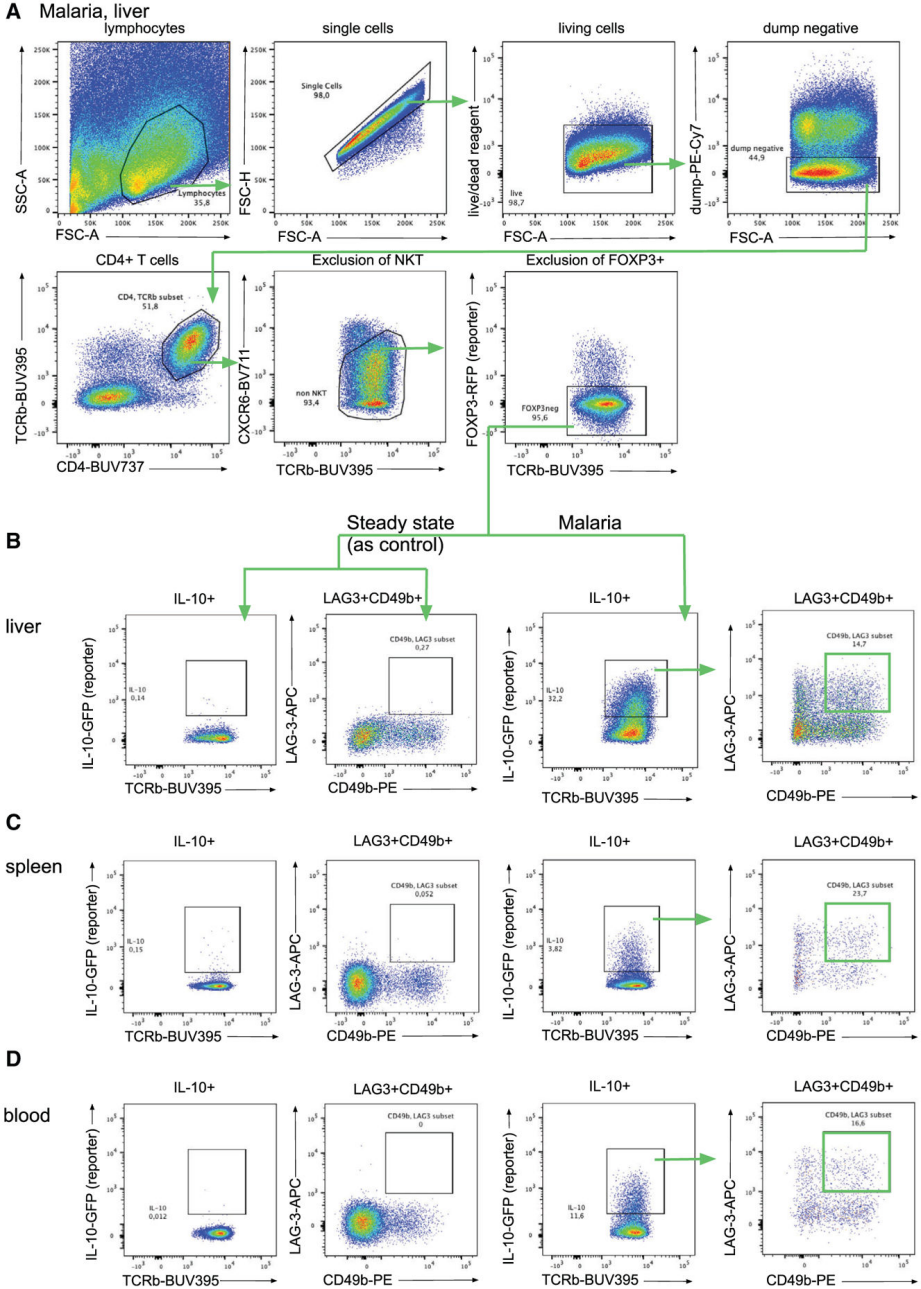
Gating strategy to identify murine Tr1 cells in *Plasmodium berghei* ANKA-infected mice. A representative gating strategy is shown (A). The dump channel allows exclusion of the majority of myeloid cells (CD11c, CD11b, MHCII), NK cells (NK1.1), gd T cells (gd TCR), CD8^+^ T cells (CD8), B cells (CD19) and – importantly - platelets (CD41), which might stick to CD4^+^ T cells. Especially in the liver, additional gating for exclusion of NKT cells is recommended. NKT cells are TCRb dim and bright for CXCR6. Stringent gating for CD4^+^ T cells is crucial, since LAG-3 and CD49b not only identify Tr1 cells within the CD4^+^ T cell subset, but also enrich for IL-10-expressing cells within other cell types. Tr1 cells co-expressing LAG-3, CD49b, and IL-10 can be gated according to the strategy shown or alternatively, cells can be first gated for LAG-3^+^ CD49b^+^ followed by gating for IL-10^+^ cells. Staining for LAG3/CD49b and IL-10 GFP reporter is shown for cells from liver (B), spleen (C) and blood (D), both during steady state (left panel) and malaria (right panel).

Therefore, when IL-10 cytokine reporter mice are not available, we propose to isolate murine Tr1 cells at least based on their co-expression of LAG-3 and CD49b (adding IL-10 by secretion assay when possible). Then we suggest to perform an *in vitro* suppression assay with the isolated cells. In this case, the gating strategy can be applied identically as shown in Figure 49, only omitting the gate for IL-10 and using CD25 exclusion instead of FOXP3.

Once the suppressive activity is confirmed, one might want to combine the surface markers with the IL-10 intracellular staining for a routine quantification.

##### A) Mouse Tr1 identification in a malaria model

Reporter mice for IL-10 (eGFP) and FOXP3 (RFP) were infected by intraperitoneal injection of 1 × 10^5^ *Plasmodium berghei* ANKA parasites, one of the causative agents of malaria in mice. Mice were sacrificed at day 6 post-infection and cells were isolated from the liver, spleen, and blood. Additionally, naïve non-infected mice were analyzed following the same steps as described below and serve as controls.

1. Sacrifice the mice with carbon dioxide/dioxygen (CO_2_/O_2_) followed by CO_2_. Of note, euthanasia must be performed in accordance with ethical approval and may be done using alternative methods than CO_2_. Be aware that cervical dislocation might impact the efficacy of the subsequent blood draw and liver perfusion.
2. Draw 200 μl blood from the heart using a 27G needle and a 1 ml syringe. Immediately dilute in 5 ml PBS/EDTA to avoid coagulation.
3. Expose heart, liver, and vena cava.
4. Perfuse the liver via the heart with 20 ml ice-cold PBS/ethylenediaminetetraacetic acid (EDTA). Use a 20G needle and insert it in the left ventricle pointing to the right atrium. After the first milliliter cut the vena cava, allowing the PBS/EDTA to drain after passing the liver. Perfuse slowly to avoid cell stress.
5. Remove the gall bladder
6. Transfer the liver tissue in flow cytometry buffer
7. Dissect the spleen and transfer in flow cytometry buffer

###### Isolation of cells from the liver

8. Mash tissue through 100 μm cell strainer in the presence of flow cytometry buffer using a plunger of a syringe
9 Wash with 20 ml flow cytometry buffer, spin down (7 min, 4°C, 350 × *g*), discard the supernatant
10. Resuspend the pellet in 5 ml 40% Percoll
11. Centrifuge (10 min, room temperature, 400 × *g*, acceleration 9 brake 1)
12. Remove the supernatant (lymphocytes will be at the bottom of the tube)
13. Resuspend the pellet in 500 μl ACK lysis buffer, incubate 3 min at room temperature
14. Wash with 14 ml flow cytometry buffer, spin down (7 min, 4°C, 350 × *g*), discard supernatant
15. Transfer cells in 96 well plate (round bottom)

###### Isolation of cells from the spleen

16. Mash tissue through 40 μm cell strainer in the presence of flow cytometry buffer using a plunger of a syringe
17. Wash with 10 ml flow cytometry buffer, spin down (7 min, 4°C, 350 × *g*), discard supernatant
18. Resuspend the pellet in 500 μl ACK lysis buffer, incubate 3 min at room temperature
19. Wash with 10 ml flow cytometry buffer, spin down (7 min, 4°C, 350 × *g*), discard supernatant
20. Transfer cells in a 96-well plate (round bottom)

###### Isolation of cells from the blood

21. Spin down (7 min, 4°C, 350 × *g*), discard supernatant (pipet or use a vacuum pump since the pellet is very loose due to red blood cells)
22. Resuspend the pellet in 1000 μl ACK lysis buffer, incubate 7 min at room temperature
23. Repeat step 21 and 22 to a total of two times red blood cell lysis
24. Transfer cells in a 96-well plate (round bottom)

Continue with cells from all organs:

25. Wash with 200 μl PBS, spin down (4 min, 4°C, 350 × *g*), discard supernatant
26. Repeat washing step
27. Add 50 μl Ab cocktail as indicated in Table 1 in PBS (do not add FBS at this step, because its presence inhibits the live/dead staining), resuspend cells
28. Incubate for 30 min at 37°C
29. Wash with 200 μl flow cytometry buffer, spin down (4 min, 4°C, 350 × *g*), discard supernatant
30. Repeat wash
31. Filter cells, resuspend in 300 μl flow cytometry buffer
32. Run at appropriate flow cytometer on low speed. Do not exceed 10,000 events/s.

##### B) Mouse Tr1 identification in the small intestine under steady-state conditions

Development of Tr1 cells relies on antigenic stimulation and mainly occurs in the intestine [460]. We have already extensively characterized Tr1 cells upon treatment with anti-CD3 mAb, a treatment able to induce a transient inflammation in the small intestine [466, 415, 501, 502]. Therefore, in this protocol, we decided to focus on the identification of intestinal Tr1 cells under steady-state conditions. Of note, in our experience, the frequency and number of Tr1 cells in the small intestine depends on the hygiene status of the mouse facility and might differ across institutes.

1. Sacrifice the mice with CO_2_/O_2_ followed by CO_2_ and cervical dislocation. Of note, euthanasia must be performed in accordance with ethical approval and may be done using alternative methods than CO_2_.
2. Dissect the small intestine, remove fat and Peyer’s patches, open longitudinally and remove the content by shaking in 20 ml PBS/EDTA in a 50 ml falcon. Repeat the washing step.
3. Cut tissue in 0.5 cm pieces, transfer in 15 ml falcon, add 10 ml DTT buffer.
4. and shake at 37°C for 20 min, in the end of the incubation shake 10 times by hand.
5. Filter through a metal sieve, wash sieve with PBS, the flow through contains intraepithelial lymphocytes.
6. Spin intraepithelial lymphocytes (350 × g, 4°C, 7 min) and resuspend the pellet in 5 ml flow cytometry buffer. Store on ice.
7. Cut remaining tissue very small and digest for 30 min in 6 ml digestion media containing Collagenase and DNase at 37°C while shaking.
8. Spin the cell suspension of step 6 (350 × g, 4°C, 7 min), discard supernatant and place 100 μm cell strainer on the tube.
9. Mash the digested tissue through the cell strainer thereby combining lamina propria lymphocytes with the intraepithelial fraction. If of interest, the lamina propria and intraepithelial fraction can be stained separately.
10. Wash with flow cytometry buffer, spin (350 × g, 4°C, 7 min) and discard supernatant.
11. Prepare 40% and 67% Percoll buffer.
12. Resuspend cell pellet in 3 ml 40% Percoll and carefully lay on 3 ml 67% Percoll in a 15 ml tube.
13. Spin at 400 × g for 20 min at room temperature, acceleration, and brake on 1.
14. After centrifugation, some of the epithelial cells will accumulate on top of the 40% layer, while dead cells and red blood cells can be found at the bottom of the tube. Lymphocytes, including Tr1 cells, will be in the interphase; remove 1 ml of the interphase.
15. Wash the cells of the interphase with 14 ml flow cytometry buffer to remove Percoll residues.
16. Transfer cells in 96 well plate (round bottom).
17. Spin (350 × g, 4°C, 7 min), discard supernatant.
18. Wash with 200 μl PBS, spin down (4 min, 4°C, 350 × g), discard supernatant.
19. Repeat washing step.
20. Add 50 μl surface staining Ab cocktail as indicated in Table 1 in PBS (do not add FBS at this step, because its presence inhibits the live/dead staining), resuspend cells.
21. Incubate for 30 min at 37°C.
22. Wash with 200 μl flow cytometry buffer, spin down (4 min, 4°C, 350 × g), discard supernatant.
23. Repeat wash.
24. Filter cells, resuspend in 300 μl flow cytometry buffer.
25. Run at appropriate flow cytometer on low speed. Do not exceed 10,000 events/s.

#### 14.4 Test of the suppressive function of murine Tr1 cells using an in vitro suppression assay

The suppressive function is the key feature of Tr1 cells. We recommend to test the function of the identified cells, especially if (1) Tr1 cells are identified in a disease model or tissue that has not been well described so far, (2) the markers used to identify Tr1 cells are limited, e.g., no IL-10 reporter is available.

Here, we provide an example of how to perform an *in vitro* suppression assay testing the function of Tr1 cells induced and identified as described in (A) during malaria.

Note: Keep the cells sterile during all steps.

##### I) Isolation of responder cells (CD4^+^ T cells) and APCs

1. Dissect the spleen of a naive wild-type mouse.
2. Mash tissue through 40 μm cell strainer in the presence of flow cytometry buffer using a plunger of a syringe.
3. Spin (350 × *g*, 4°C, 7 min), discard supernatant

###### Deplete Treg cells

4. Resuspend the pellet in 250 μl flow cytometry buffer, add aCD25-biotin 1:400
5. Incubate for 15 min in fridge
6. Wash with 5 ml flow cytometry buffer, spin (350 × *g*, 4°C, 7 min), discard supernatant
7. Resuspend the pellet in 250 μl flow cytometry buffer, add 10 μl Streptavidin-beads
8. Incubate for 30 min in fridge
9. Wash with 5 ml flow cytometry buffer, spin (350 × *g*, 4°C, 7 min), discard supernatant
10. Equilibrate LS MACS column on the MACS rack using 3 ml flow cytometry buffer
11. Resuspend cells in 1 ml flow cytometry buffer, filter though 30 μm filter
12. Add filtered cells to column, wash three times with 3 ml flow cytometry buffer
13. Collect the flow through

###### Split into CD4^+^ T cells and rest

14. Spin flow through from step 13 (350 × *g*, 4°C, 7 min), discard supernatant.
15. Resuspend pellet in 250 μl flow cytometry buffer, add 25 μl CD4 beads (L3T4, Miltenyi, 130-049-201).
16. Incubate for 10 min in the fridge.
17. Equilibrate LS MACS column on the MACS rack using 3 ml flow cytometry buffer.
18. Add cells to the column. Wash three times with 3 ml flow cytometry buffer.
19. Collect flow through (depleted from CD4^+^ T cells).
20. Remove column from the magnet, add 5 ml flow cytometry buffer, and flush CD4^+^ T cells in a fresh 15 ml falcon.

###### Prepare APCs

21. Spin down flow through from step 19 (350 × *g*, 4°C, 7 min), discard supernatant.
22. Resuspend the pellet in 200 μl flow cytometry buffer, add aCD3-biotin 1:200.
23. Incubate for 15 min in fridge.
24. Wash with 5 ml flow cytometry buffer, spin (350 × *g*, 4°C, 7 min), discard supernatant.
25. Resuspend the pellet in 250 μl flow cytometry buffer, add 10 μl Streptavidin-beads.
26. Incubate for 30 min in fridge.
27. Wash with 5 ml flow cytometry buffer, spin (350 × *g*, 4°C, 7 min), discard supernatant.
28. Equilibrate LS MACS column on the MACS rack using 3 ml flow cytometry buffer.
29. Resuspend cells in 1 ml flow cytometry buffer, filter though 30 μm filter.
30. Add filtered cells to column, wash three times with 3 ml flow cytometry buffer.
31. Collect the flow through (depleted from T cells).
32. Spin down (350 × *g*, 4°C, 7 min), discard supernatant.
33. Add 500 μl ACK lysis buffer, incubate 3 min at room temperature.
34. Stop lysis by adding 5 ml of Clicks full media, spin down (350 × *g*, 4°C, 7 min), discard supernatant.
35. Resuspend in 5 ml Clicks full media, transfer in non-adhesive cell culture flask.
36. Irradiate with 30 Gy.
37. Count cells.

###### Prepare and label responder cells

38. Prewarm 500 μl PBS and 1 μl violet dye (CellTracer; final conc. 4 μM, stock 2 mM) at 37°C.
39. Spin down cells from step 20 (350 × *g*, 4°C, 7 min), discard supernatant.
40. Wash with PBS, spin (350 × *g*, 4°C, 7 min), discard supernatant.
41. Resuspend the pellet with staining solution (step 36).
42. Incubate 6 min, 37°C.
43. Stop reaction by adding 3 ml FBS, spin (350 × *g*, 4°C, 7 min), discard supernatant.
44. Repeat wash.
45. Count cells.

##### II) Preparation of suppressor cells

1. Isolate splenocytes from *Plasmodium berghei* ANKA infected mice as described in (A).
2. Enrich for CD4^+^ T cells (Miltenyi CD4^+^ T cell isolation kit 130-104-454).
  - Resuspend cell pellet in 400 μl flow cytometry buffer, add 100 μl biotin-Ab cocktail per spleen.
  - Incubate for 5 min at 4°C.
  - Add 300 μl flow cytometry buffer and 200 μl anti-Biotin beads per spleen.
  - Incubate for 10 min at 4 °C.
  - Wash with 5 ml flow cytometry buffer.
  - Equilibrate LS column on MACS rack with 3 ml flow cytometry buffer.
  - Resuspend cells in 1 ml flow cytometry buffer and add to column.
  - Collect flow-through (containing CD4^+^ T cells).
3. Stain according to (A).
4. Sort Tr1 cells according to the gating strategy shown in Figure 49; Use the 70 μm nozzle.
5. Verify purity after sort.
6. Count cells.

##### III) Set up assay/plating

General notes:

- Make replicates.
- Plate cells in a 96-well round bottom plate.
- Required control: responder cells adjusted for the total T cell number present in the condition with Tr1 cells (negative control of suppression).
- Use at least 5.000 Tr1 cells per well. If enough cells are available, scale up to 20,000 Tr1 cells per well.
- Adjust the number of responder cells and APCs according to the available number of Tr1 cells.
- Use a ratio of 1,5:1 for responder cells to Tr1 cells. If enough Tr1 cells are available, other ratios e.g., 1:1, 1:2, 1:4 may be added.
- Use a ratio of 3:1 for APCs to T cells (Responder + Tr1 cells).

Example:

Experimental condition: 20,000 Tr1 cells, 30,000 Responder cells, and 150,000 APCs.

Negative control of suppression: 50,000 Responder cells and 150,000 APCs.

1. Pool APCs and Responder cells (CD4^+^ T cells), adjust the concentration to obtain the final number of cells needed per well in 100 μl Clicks full media.
2. Add 3 μg/ml aCD3 Ab (final concentration will be 1.5 μg/ml).
3. Plate 100 μl per well (containing APCs, Responder cells, and aCD3).
4. For experimental condition: add suppressor cells in 100 μl Clicks full media.
5. For negative control: add the same number of responder cells as suppressor cells used in the experimental condition in 100 μl Clicks full media.
6. Incubate for 5 days at 37°C and 5% CO_2_.

##### IV) Read-out

1. Spin plate (350 × g, 4°C, 5 min), discard supernatant.
2. Wash with 200 μl PBS.
3. Stain according to surface staining in (A).
4. Resuspend in 200 μl flow cytometry buffer.
5. Run at appropriate flow cytometer on low speed.

##### v) Analysis

1. Gate for T cells, distinguish responder cells and suppressor cells by CellTrace staining or congenic markers, if mice with different background have been used to isolate responder and suppressor cells.
2. Within the responder cells, analyze the overall number of cells after each division according to the CellTracer. Calculate the frequency of dividing cells for all wells and set the negative control as 100% of division (since this is the maximum proliferation to be expected in this assay).
3. Calculate the suppression in frequency compared to the negative control
  example: In negative control (ControlDivided), 74% of cells proliferated (equals maximum); in the experimental condition (Tr1Divided), 13% proliferated
  Suppression= 100-(%Tr1Divided*100/%ControlDivided) 100-(13*100/74) → 82% suppression
4. Of note, alternative methods such as tools provided by FlowJo may be used to analyze and quantify the suppression.

#### 14.5 Materials

The materials are listed in Tables 48-50.

#### 14.6 Data analysis

The analysis of flow cytometry data shown in this protocol was performed using the FlowJo software (version 10.6.2).

**Figure 50.**
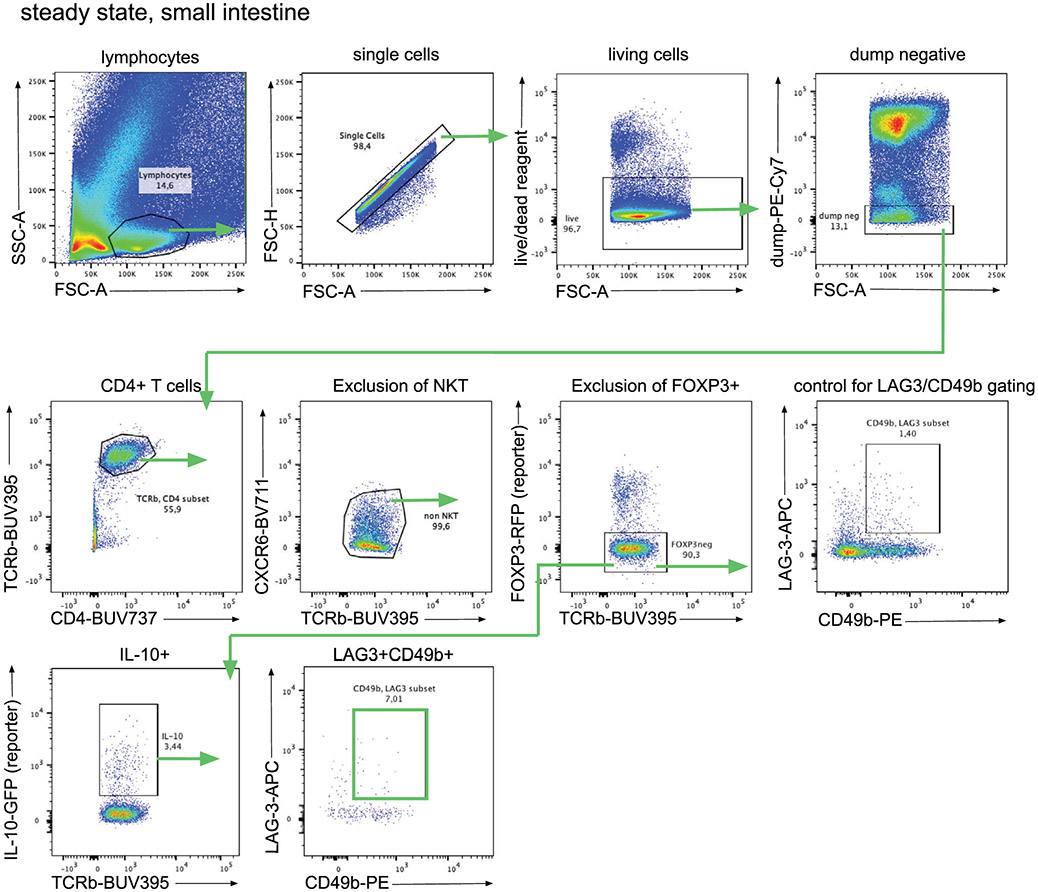
Gating strategy to identify murine Tr1 cells in the small intestine. A representative gating strategy is shown (A). The dump channel allows exclusion of the majority of myeloid cells (CD11c, CD11b, MHCII), NK cells (NK1.1), gd T cells (gd TCR), CD8^+^ T cells (CD8), B cells (CD19), and – importantly - platelets (CD41), which might stick to CD4^+^ T cells. Stringent gating for CD4^+^ T cells is crucial, since LAG-3 and CD49b not only identify Tr1 cells within the CD4^+^ T cell subset, but also enrich for IL-10-expressing cells within other cell types. Tr1 cells co-expressing LAG-3, CD49b, and IL-10 can be gated according to the strategy shown or alternatively, cells can be first gated for LAG-3^+^ CD49b^+^ followed by gating for IL-10^+^ cells.

Gating murine Tr1 cells start with gating for lymphocytes (FSC-A/SSC-A) and then for single cells (e.g., FSC-A/FSC-H, alternative gating such as SSC-A/SSC-H can be applied). Live/Dead dye positive cells need to be excluded. We recommend to include a dump channel to exclude myeloid cells, B cells, gamma delta T cells, and CD8^+^ T cells. Additionally, special attention should be paid to exclude CD4^+^ T cell - platelet doublets since platelets express CD49b and by sticking to CD4^+^ T cells could lead to false-positive results. Next, the combination of CD4 and TCRb is recommended for gating CD4^+^ T cells. Alternatively, CD3 instead of TCRb may be used. Dependent on the tissue analyzed, NKT cells should be excluded. This is in particular important in the liver (Figure 49), while other organs such as the small intestine contain few NKT cells (Figure 50). To identify NKT cells, tetramer staining or CXCR6 bright/TCRb^dim^ (or CD3^dim^, or CD4^dim^) gating can be used. Furthermore, FOXP3^+^ Tregs may be excluded by either FOXP3 reporter (e.g., RFP FOXP3 reporter mice) or CD25 expression if FOXP3 reporter mice are not available. Finally, murine Tr1 cells within the CD4^+^ T cell population can be identified by LAG-3 and CD49b surface expression in combination with IL-10 expression.

**Figure 51.**
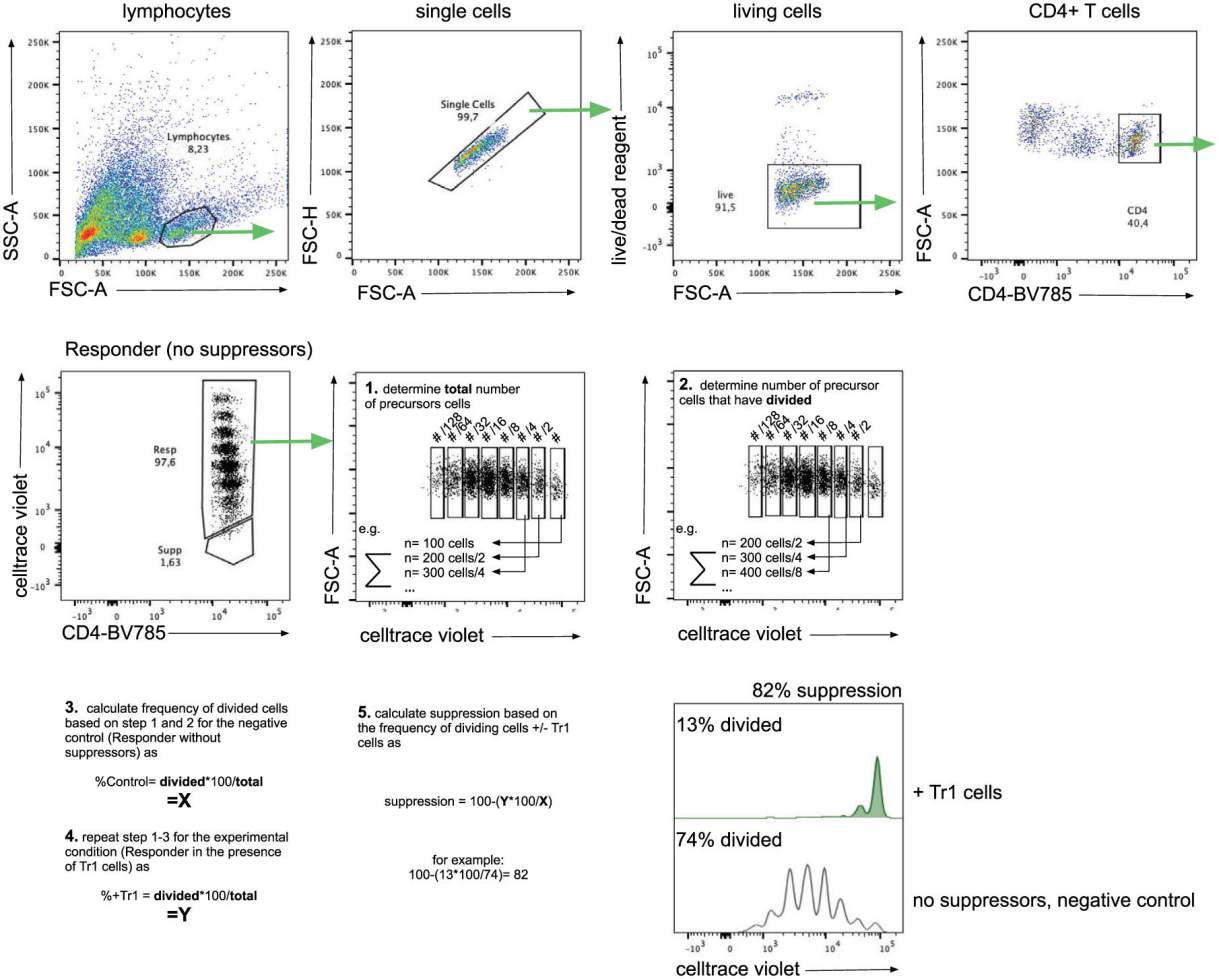
Analysis of an *in vitro* suppression assay testing the function of murine Tr1 cells. First, responding CD4^+^ T cells are identified by gating for physical parameters (lymphocyte and single cell gate), living cells, CD4^+^ T cells, which are positive for the CellTrace. Within the Responder population, the number of cell divisions can be tracked according to the fluorescence intensity of the CellTrace, since the fluorescence intensity is reduced by half after every division. In order to calculate the number of cells that underwent division out of the population of precursor cells, the number of cells (#) is adjusted to the number of divisions of each cell. For example, the number of cells that have divided once is divided by two (#/2), since two cells originate from one precursor cell. Based on the frequency of total precursor cells and divided precursor cells, the frequency of division can be calculated. To calculate the suppressive capacity of the experimental group (=Tr1 cells), the frequency of division in the negative control is set to 100%.

For the suppression assay, in short, after gating for responder CD4^+^ T cells, the number of dividing cells within the total population is determined. Cell division is tracked by dilution of a proliferation dye/cell trace. The frequency of cell division is determined for both the negative control and the experimental condition in the presence of putative Tr1 cells. The proliferation of the negative control (without suppressors) can be considered as the maximum of proliferation expected in this assay. Based on the maximum proliferation, the frequency of suppression in the experimental condition can be calculated as indicated in Figure 51.

After function validation, staining of the markers and intracellular IL-10 may be used for more routine quantification of Tr1 cells if cytokine reporter mice are not available.

#### 14.7 Pitfalls

1. Make sure to use the correct clone for CD49b (clone HMα2).
2. CD49b is highly expressed on platelets. Platelets tend to stick to other cells such as CD4^+^ T cells [503]. To avoid CD49b false-positive cells, include a platelet marker (e.g., CD41) in the dump channel when analyzing samples containing platelets. Strict single cell gating is not sufficient to remove CD4 and platelet doublets.
3. The CD4^+^ T cell population, in particular in the liver, contains NKT cells. For exclusion of NKT cells, CXCR6^bright^ TCRb^dim^ cells should be removed from the analysis.
4. IL-10 expression alone is not sufficient to identify Tr1 cells. Always combine with staining for co-inhibitory receptors.

#### 14.8 Top tricks

1. Stain at 37°C for 30 min in order to improve LAG-3 staining.
2. IL-10, LAG-3, and CD49b staining must be combined with gating for CD4^+^ T cells (excluding IL-10 producing CD8^+^ T cells) and FOXP3-negative cells (excluding IL-10-producing Foxp3^+^ Tregs). B cells have to be excluded by gating for T cells (TCRb, CD4). Additionally, CD19 may be used in the dump channel.
3. When IL-10 reporter mice are not available LAG-3 and CD49b co-expression can be used as a biomarker to identify Tr1 cells [415, 460].
4. Confirm suppressive capacity of the cells identified in specific tissue/disease model by *in vitro/in vivo* suppression assay, especially if the number of markers used is limited, e.g., no IL-10 reporter available and/or Tr1 cells have not been characterized in this model or tissue yet.

#### 14.9 Summary of the phenotypes

This is detailed in Table 51.

#### 14.10 Key information human versus murine

This is detailed in Table 52

### 15 Unconventional human cells: Gamma delta (γδ) T cells

#### 15.1 Overview

Recently, there has been a surge of interest in gamma delta (γδ) T cells, as this cell type is increasingly being recognized to play important roles in multiple disease settings, such as in cancer, autoimmune disorders, and microbial infections. A relatively easy and quick method to analyze these cells is by flow cytometry. New markers and Ab combinations have been deployed recently that identify the major γδ T cell subsets. However, phenotyping of these cells using flow cytometry remains challenging and thus here we provide an optimized protocol and monoclonal Ab (mAb) combinations to detect human γδ T cell subsets.

#### 15.2 Introduction

**Figure 52.**
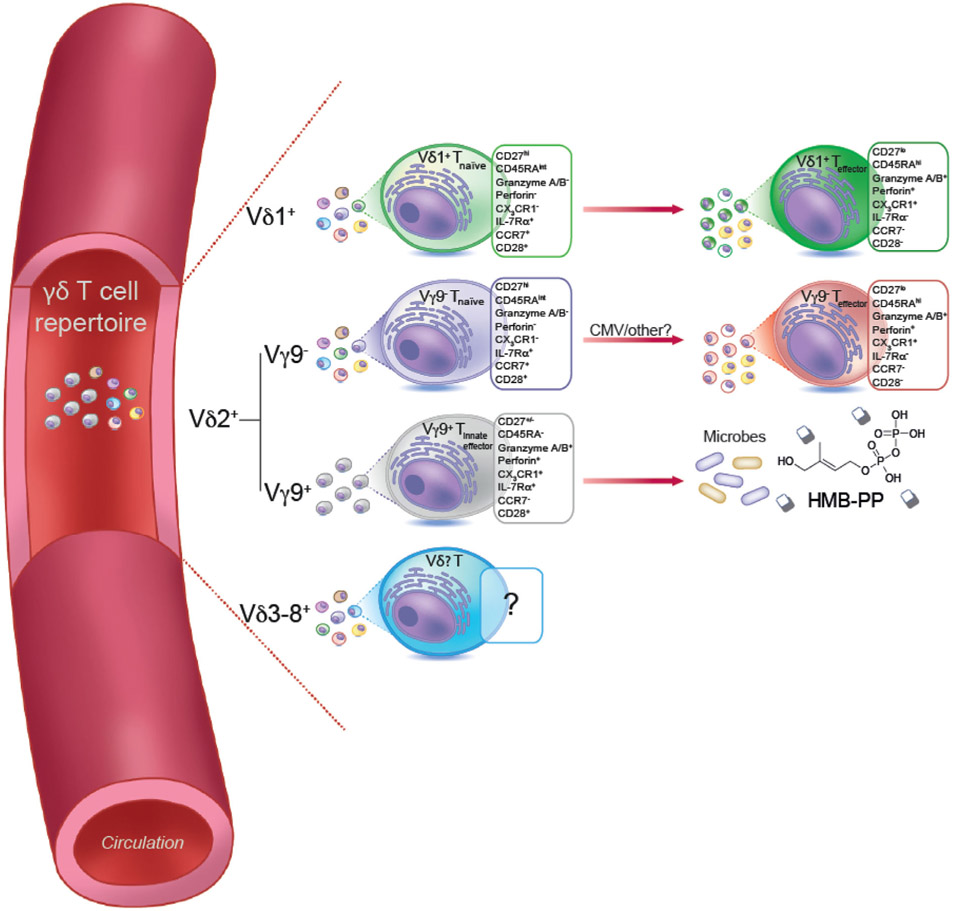
Human γδ T cells found in the peripheral blood. Each population is divided based on their Vδ chain usage, primarily due to the availability of TCR Vδ1 and TCR Vδ2 mAbs. Each subset is displayed alongside a set of cell surface markers that accurately define them in the steady state. Vγ9^−^/Vδ2^+^ and Vδ2^−^ T cells seem to undergo postnatal selection in the periphery from a naïve γδ T cell pool. Vγ9^+^/Vδ2^+^ T cells are established in the perinatal period and are rapidly matured after birth, resulting in a uniform responsiveness to pAgs. Vδ1^−^Vδ2^−^ T cells express a Vδ3-8 TCR chain pairing and are rare in the peripheral blood but enriched in the tissues such as the liver. The markers that define them and if they form further subsets are unclear.

Gamma delta (γδ) T cells represent an unconventional T cell subset that expresses a T cell receptor (TCR) that is constructed of a γ and δ chain. These immune cells play important roles in the responses against microbes [508] and tumors [509]. In contrast to conventional αβ T cells, γδ T cells have been described to respond to an array of non-peptide Ags [510] independent of MHC proteins [511]. Instead, these cells seem to respond to non-peptidic metabolite Ags as well as other diverse ligands [510, 512]. γδ T cells are typically divided into Vδ2^+^ and Vδ2^−^ subsets in humans [513]. Most γδ T cells in the peripheral blood are Vγ9^+^/Vδ2^+^ [514] and respond to phosphoAgs (pAgs) such as prenyl pyrophosphate metabolites that are commonly produced by microbes and host-derived isopentyl pyrophosphate that is upregulated in some tumor cells [515, 516]. Conversely, Vδ2^−^ T cells are positioned across multiple tissues and are a minor subset in the peripheral blood [517, 518]. A current overview of human γδ T cell subsets is provided in Figure 52.

##### 15.2.1 Human Vγ9^+^/Vδ2^+^ T cells (innate-like).

Vγ9^+^/Vδ2^+^ T cells (also referred to as Vγ2^+^/Vδ2^+^ T cells in some publications) expand extra-thymically and microbial-derived pAgs potentially trigger polyclonal expansion of these cells in the periphery following birth [519, 520]. Enriched Vγ9^+^/Vδ2^+^ T cell numbers are present in fetal peripheral blood and these cells show restricted complementarity determining region 3 (CDR3) γ9 usage [521]. In addition, similar Vγ9 TCR sequences are detected in multiple donors (i.e., “public” sequences) and are shared in samples from cord and adult blood [515, 522]. Vγ9^+^/Vδ2^+^ T cells are generally enriched in circulating blood (5-10% of CD3^+^ T cells) and respond to pAgs such as isopentenyl pyrophosphate (IPP) (i.e., elevated in tumor cells) and (*E*)-4-hydroxy-3-methyl-but-2-enyl pyrophosphate (HMB-PP), which is produced by bacteria and parasites [515]. HMB-PP is recognized in the context of butyrophilin (BTN) family members 3A1 [523-526] and 2A1 [527, 528] expressed on APCs. Together, this evidence hints at the innate-like functions of these γδ T cells.

##### 15.2.2 Human Vδ2^−^ and Vγ9^−^/Vδ2^+^ T cells.

The exact functions of Vδ2^−^ γδ T cells are currently unclear, but they have been demonstrated to expand in response to tumor cells, bacteria, parasites, and viruses. The majority of Vδ2^−^ T cells express a Vδ1 TCR chain pairing, while a minority express other Vδ TCR chains, notably Vδ3, Vδ4, Vδ5, and Vδ8. Studies investigating Vδ1 and Vδ3 T cells have been aided by critical commercial Ab reagents, although anti-Vδ3 Abs are now only available upon request (from Beckman Coulter; clone P11.5B). The identification of Vδ4, Vδ5, and Vδ8 T cells has been restricted to sequencing-based approaches as commercial flow cytometry reagents are not available. Studies focussed on Vδ1 and Vδ3 T cells have now shed light on the receptor diversity and physiology of these subsets in the tissues and in infection, such as cytomegalovirus [515, 529]. Vδ1^+^ T cells display a CD27^lo/−^CD45RA^+^ effector phenotype when clonally expanded and heterogeneous γ chain usage [515, 529]. Moreover, uniformly in cord and, at mixed levels, in adult blood, Vδ1^+^ T cells exhibit a diverse and polyclonal population, expressing markers of a naïve T cell population – this is reviewed in [530]. This subset of γδ T cells is suggested to play a role in the adaptive immune response, as clonal expansions of these cells can be found in peripheral blood and liver tissue of adults, whereas clonal expansion is not evident in cord blood [515, 531]. These clones are likely generated in response to cellular stress or microbial infection, as observed in acute cytomegalovirus infection [531, 532]. Additionally, a sub-population of Vδ2^+^ T cells has been identified that generally do not express the Vγ9 chain pairing (termed Vγ9^−^/Vδ2^+^ T cells). This population of undergoes dramatic clonal expansion in cytomegalovirus infection and can be identified using flow cytometry markers similar to Vδ1^+^, T cells, i.e., CD27^lo/−^CD45RA^+^ CX_3_CR1^+^ cells (effector) and a naïve polyclonal compartment expressing CD27^hi^CD45RA^+/−^CX_3_CR1 [515]. Together, these recent findings surrounding Vδ2^−^ and Vγ9^−^/Vδ2^+^ T cells indicated that these cells follow an adaptive biology.

##### 15.2.3 Human γδ T cells in cancer immunotherapy.

γδ T cells are now considered a major target for the development of novel cellular immunotherapies. This is in part due to their ability to recognize tumor cells through the γδTCR or natural killer cell receptors (NKRs), such as NKG2D, NKp30, NKp44, and NKp46 that decorate their cell surface [533]. The endogenous host pAg, IPP, and the ligands for NKRs are often overexpressed in transformed cells. As such, Vγ9^+^/Vδ2^+^ T cells have been a principal candidate for immunotherapy as they exert strong lytic activity toward tumor cell lines [534], which can be mediated via the production pro-inflammatory cytokines and apoptosis-inducing molecules [535]. More recently, Vδ1^+^ T cells have rapidly become a major avenue for the creation of cellular immunotherapy and display potent cytolytic capacity toward a range of tumor cell lines [536]. The main avenue of delivery is via adoptive transfer of *in vitro* expanded γδ T cells into cancer patients and some clinical trials have shown that these cells may be effective at limiting tumor progression.

##### 15.2.4 Flow cytometry for human γδ T cells.

A uniform staining and gating strategy is needed to identify γδ T cells and their subsets. The need for an optimized protocol has recently been high-lighted, as it was shown that Vγ9^−^/Vδ2^+^ T cells could only be identified by flow cytometry when using the anti-Vδ2 mAb clone 123R3 (Miltenyi Biotec), while this population could not be identified when the anti-Vδ2 mAb clone B6 (BD Biosciences and BioLegend) was used [515]. Moreover, the combination of some commercial pan-γδTCR mAbs (Biolegend (B1), BD Biosciences (11F2) and Beckman Coulter (IMMU510)) can interact with Vγ9, Vδ1 and Vδ2 [22], causing disruption to γδTCR staining. Thus, here we describe the materials and a well-optimized protocol to identify subsets of human γδ T cells.

#### 15.3 Step-by-step sample preparation

##### 15.3.1 Peripheral Blood Mononuclear Cell Isolation.

Peripheral blood mononuclear cells (PBMCs) can be isolated from heparinized venous blood or a buffy coat. First, the buffy coat/peripheral blood has to be diluted 1× in phosphate-buffered saline (PBS), followed by transfer of the cell suspension on Lymphoprep^™^, which is density gradient centrifuged according to the manufacturers’ instructions. PBMCs are frozen in 90% foetal calf serum (FCS) and 10% dimethyl sulfoxide (DMSO). See Table 53 for details on buffers and reagents.

##### 15.3.2 Flow Cytometry – Surface Marker Staining.

After mononuclear cells have been obtained from either tissue or peripheral blood, the following protocol should be followed for the staining of surface markers on γδ T cell subsets:

1. Thaw the PBMCs, centrifuge for 2 min at 400 × *g* at 4°C, and add 500,000 PBMCs per well in a U-bottomed 96-well plate.
2. Wash PBMCs in 200 μl PBS per well for 2 min at 400 × *g* at 4°C. Discard the supernatant.
3. Add 50 μl Zombie Aqua^™^ diluted (1:500) in PBS (to stain dead cells; Table 53) per well and incubate for 10 min at room temperature (RT) in the dark.
4. Add 150 μl FACS buffer per well, consisting of sterile PBS + 20% FCS + 0.04% sodium azide, and centrifuge the plate for 2 min at 400 × *g* at 4°C. Discard the supernatant.
5. Block Fc receptors to prevent non-specific binding of mAbs by adding 50 μl TruStain FcX (diluted 1:100 in FACS buffer). Incubate for 10 min on ice in the dark.
6. Prepare a cocktail of the mAbs according to Table 53. Dilute the mAbs in FACS buffer. Stain the cells with 50 μl Ab cocktail per well on ice in the dark for 15 min.
7. Add 150 μl FACS buffer per well and centrifuge the plate for 2 min at 400 × *g* at 4°C. Discard the supernatant.

Resuspend cells in 200 μl FACS buffer per well, keep in the plate or transfer the cell suspension to polypropylene tubes, and keep on ice until acquisition at the BD LSRFortessa^™^ X-20 (BD Biosciences, San Jose, CA, USA).

##### 15.3.2.1 Flow Cytometry – Intracellular Marker Staining.

The protocol can be extended by staining for intracellular targets, such as Granzyme A and B and perforin. The following steps should be followed after step 6 of the surface marker staining protocol:

1. Add 50 μl IC Fixation buffer (eBioscience; Table 53) per well and incubate for 30 min at 4°C in the dark. Centrifuge the plate for 2 min at 400 × *g* at 4°C. Discard the supernatant.
2. Add 50μl Permeabilisation buffer (Perm buffer; eBioscience; Table 53) per well and centrifuge the plate for 2 min at 400 × *g* at 4°C. Discard the supernatant.
3. Add 50 μl Fc block, diluted 1:100 in Perm buffer to block specific binding of mAbs to Fc receptors. Incubate for 10 min at RT in the dark.
4. Prepare a cocktail of the mAbs (example for intracellular targets in Table 54). The mAbs should be diluted in Perm buffer. Per well, add 50 μl Ab cocktail and incubate for 30 min at RT in the dark. Centrifuge the plate for 2 min at 400× *g* at 4°C. Discard the supernatant.
5. Resuspend cells in 200 μl FACS buffer per well, transfer to FACS tubes, and keep on ice until acquisition at the BD LSRFortessa^™^ X-20.

#### 15.4 Materials

Materials are detailed in Tables 53 and 54.

#### 15.5 Data analysis

**Figure 53.**
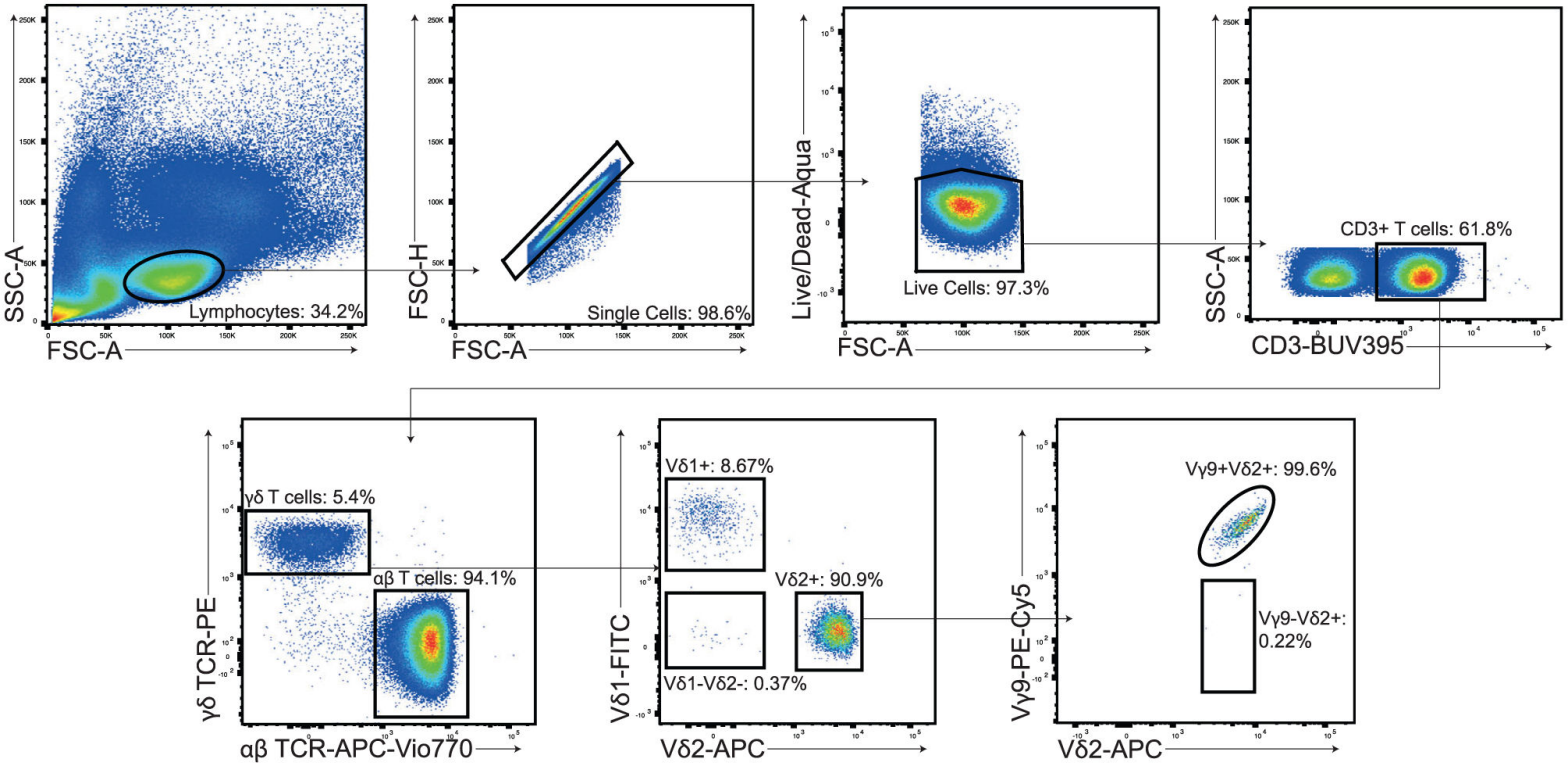
Gating strategy to define human cells γδ T cells in the peripheral blood. The gating strategy used to define human γδ T cells involves manual gating of Lymphocytes > Single Cells > Live cells > CD3^+^ cells > TCRγδ^+^. The use of γδTCR vs αβTCR mAbs provides the consistent ability to accurately discriminate γδ T cells in even the most challenging samples, i.e., where γδ T cell numbers are very low or viability is poor. γδ T cell subsets are then defined based on expression of TCRγδ^+^ > Vδ1^+^, Vδ2^+^, Vδ1/2^−^. Vδ2^+^ T cells can be further sub-divided in cells that are Vγ9^+^ or Vγ9^−^ (rare in peripheral blood).

We analyzed our data using the FlowJo software (version 10.7.2, Tree Star). In Figure 53, we show the gating strategy that was used. First, the lymphocytes are gated in the FSC-A/SSC-A plot. After exclusion of doublets in the FSC-A/FSC-H plot, we gated on live CD3^+^ T cells in the CD3/Live/dead (L/D) plot. In the αβTCR/γδTCR plot, γδTCR^+^ T cells and αβTCR^+^ T cells were gated. The γδTCR^+^ T cell population can be further divided into Vδ1^+^ and Vδ2^+^ T cells using the Vδ2/Vδ1 plot. Lastly, within the Vδ2^+^ T cells we gated on Vγ9^+^/Vδ2^+^ T cells. Within the αβTCR^+^ T cell population, we gated on CD8^+^ T cells in the CD8/SSC-A plot (plot not shown).

**Figure 54.**
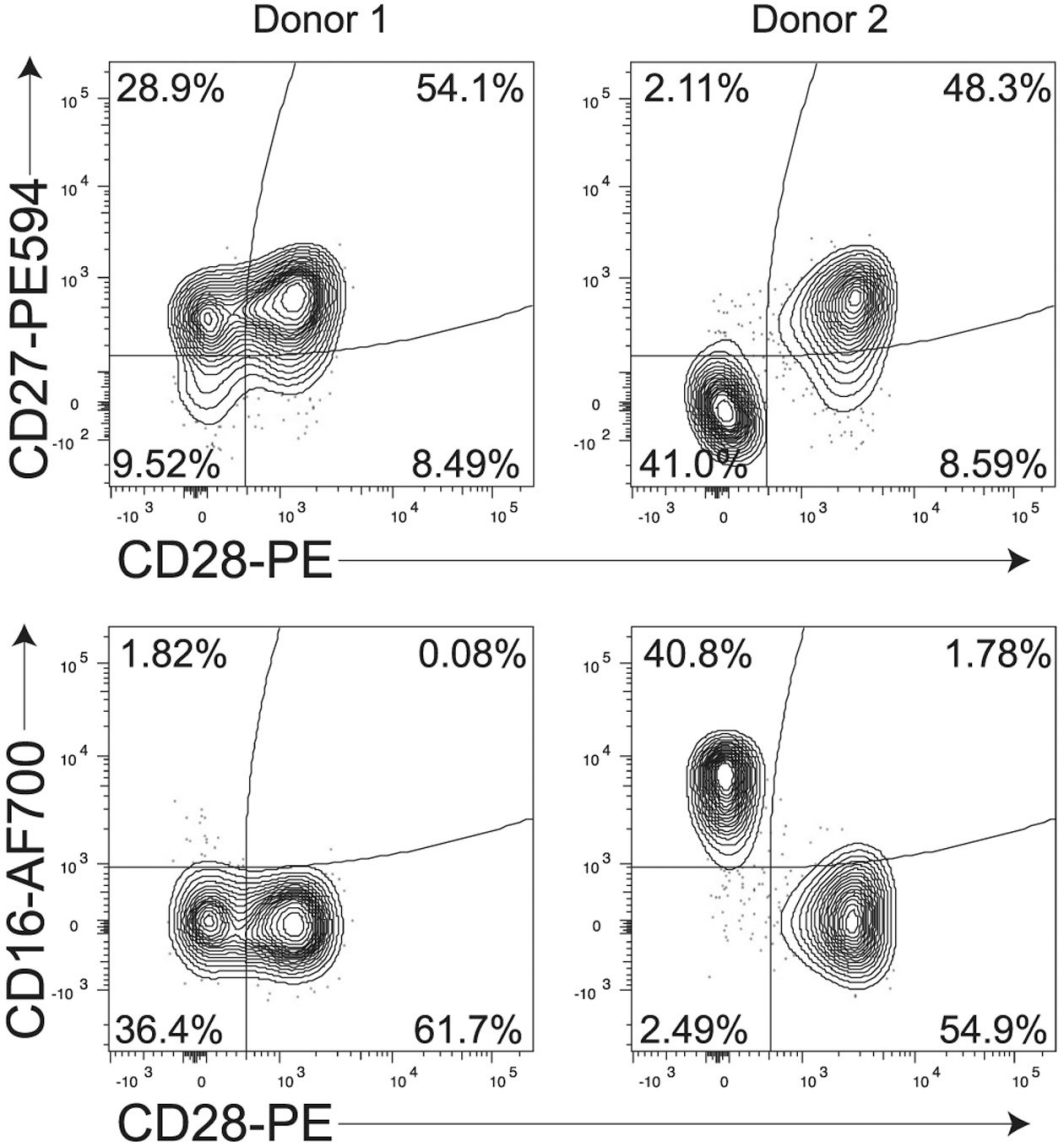
Functional sub-populations of human Vγ9^+^/Vδ2^+^ T cells. After using the gating strategy to define human γδ T cells described in Figure 53, human circulating Vγ9^+^/Vδ2^+^ T cells can be further split into effector subsets based on CD27, CD28, and CD16 expression. These populations are highly variable between individuals and it is unclear how these populations are derived.

Vγ9^+^/Vδ2^+^ T cells can be further delineated into functional subsets based on the expression of CD27, CD28 and the acquisition of CD16 (Figure 54). Definitions of these subsets are detailed in Ryan et al. [537]. These subsets may play a role in the potent antimicrobial activity of these cells in bacterial infections producing HMB-PP.

**Figure 55.**
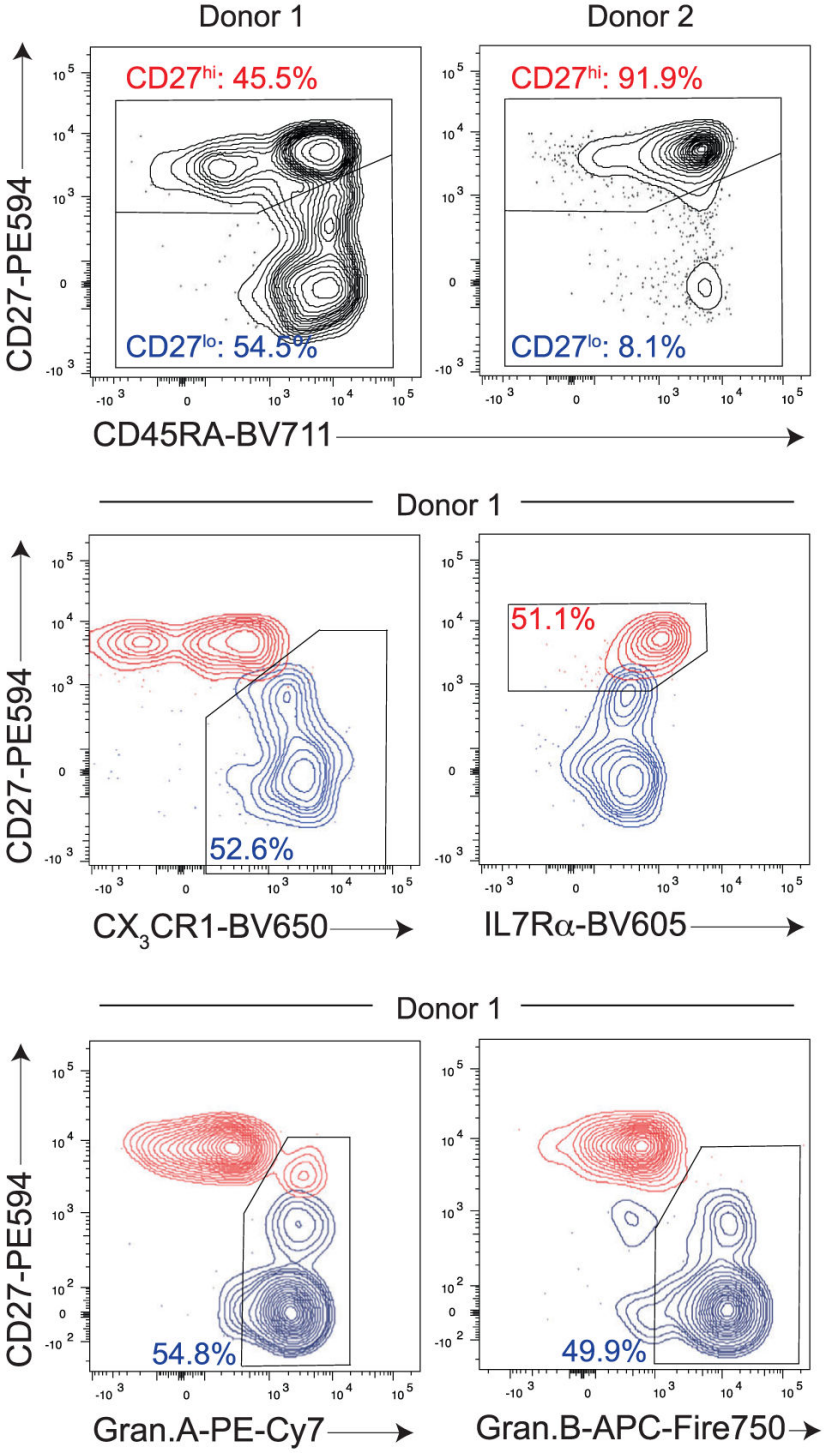
Identifying human naïve and effector sub-groups of adaptive Vγ9^−^/Vδ2^+^ and Vδ2^−^ γδ T cells in the circulation. After application of the gating strategy described in Figure 53, the distribution of clonally diverse naïve human γδ T cells can be identified by the expression of CD27 and CD45RA (CD27^hi^; marked in red) and clonally expanded effector γδ T cells (CD27^lo^; marked in blue), see Davey et al. [531]. These naïve and effector subsets display very distinct phenotypes and can be further defined by the expression of CX_3_CR1, Granzyme A/B or IL7Rα. The data shown here is an example of the expression of these markers in human Vδ1^+^ γδ T cells.

Vγ9^−^/Vδ2^+^ and Vδ2^−^ T cell subsets can be further divided into naïve (CD27^hi^) and effector (CD27^lo^) cells (Figure 55) [514]. CD8^+^ T cells were included as a control subset. Within each subset, CD27^hi^ γδ T cells are characterized by the absence of CX_3_CR1 and presence of IL7Rα expression (Figure 55). On the other hand, CD27^lo^ γδ T cells do express CX_3_CR1, whereas these cells do not express IL7Rα (Figure 55).

#### 15.6 Pitfalls

When analyzing γδ T cells by flow cytometry, several pitfalls should be kept in mind. First, with this 12-color staining panel, it is very important to set-up a good compensation. Second, the mAbs should be tested and titrated so that correct dilutions can be used. This not only saves valuable mAbs but also ensures that the dilution is matched to the expression level of the target and the fluorochrome intensity. Third, γδ T cells represent only a small proportion within T cells in the peripheral blood and in a scatter plot it might be harder to set gates. In this case, a contour or pseudocolor plot might be used to be better able to detect different populations. Lastly, flow cytometric analysis of *in vitro* stimulated human γδ T cells expanded with mitogenic anti-CD3 (OKT-3 or UCHT-1 clones), anti-Vδ1 (TS8.2), or anti-TCRγδ (B1) mAbs can be particularly challenging. The internalization of the γδTCR complex upon stimulation with these mAbs usually occurs within minutes, causing a decreased surface expression of the γδTCR. Additionally, these mitogenic Abs remain bound to their epitopes and can block the staining of γδTCR and CD3 molecules in later Ab staining protocols, hampering the discrimination of CD3^+^ γδTCR^+^ and CD3^+^ αβTCR^+^ cells. This problem can be circumvented by sub-culturing cells for 2-3 days in the absence of anti-CD3 and/or −γδTCR mAbs, thereby restoring the availability of the γδTCR complex. Of note TCR internalization does not occur when γδ T cells are cultured in the presence of pAgs (IPP or HMB-PP).

#### 15.7 Top tricks

Top tricks for the staining protocol are to work cold and quick when following this staining protocol. When analyzing intracellular targets, the FoxP3 intranuclear staining buffer set should be used as it gives the best staining results.

#### 15.8 Clinical relevance statement

The gating strategy in this section is applicable for analysis of γδ T cell subsets in acute cytomegalovirus infected patients, as can be seen in studies by Davey et al. [531]. The key conclusion from such analysis is that CD27^lo^ Vγ9^−^/Vδ2^+^ T cells are expanded in individuals infected with cytomegalovirus.

#### 15.9 Summary of the phenotypes

This is detailed in Table 55.

### 16 Unconventional murine T cells: γδ T cells

#### 16.1 Overview

In this section, we discuss the specific requirements to analyze γδ T cells by flow cytometry. This includes general recommendations, an overview of the specific tools available to study γδ T cells by flow cytometry, a detailed protocol to stain for a specific subset of γδ T cells, and a point-by-point protocol to isolate and analyze γδ T cells from mouse ear skin. Protocols to isolate γδ T cells (and αβ T cells) from the mouse intestine are given in a subsequent related section.

#### 16.2 Introduction

γδ T cells develop in the thymus together with αβ T cells but rear-range a different T cell receptor (TCR) consisting of a TCR-γ and TCR-δ chain (see section Human γδ T cells). These TCRγδ are not MHC restricted, thus their Ag recognition does not rely on CD4 or CD8 co-receptors, although the majority of mouse intestinal intraepithelial γδ T lymphocytes (γδ iIELs) express the CD8αα dimer. Mouse γδ T-cells comprise significantly different populations, and these γδ T-cell subsets are typically grouped by the variable γ-chain (Vγ) segments employed by their TCRγδ [327]. Alternatively, γδ T-cell subsets may also be grouped according to γδ T-cell tissue location or with respect to γδ T-cell function [517, 538].

#### 16.3 γδ T cells in peripheral lymph nodes

Flow cytometry of γδ T cells is technically not different to flow cytometry of αβ T cells. However, γδ T cells are up to 100-fold less frequent than αβ T cells in blood and secondary lymphoid organs and therefore their detection faces the usual challenges connected to identifying rare cell types, i.e., make sure to include all real γδ T cells but avoid false-positive events due to autofluorescence and unspecific staining. On the other hand, γδ T cells can make up the majority of lymphocytes in tissues such as the skin and the small intestinal epithelium.

##### 16.3.1 Step-by-step sample preparation.

A dedicate step-by-step sample preparation protocol to obtain single cell suspensions from peripheral lymphoid organs is described in the Murine Treg section. Briefly, peripheral lymph nodes (pLN) are collected and mashed through a cell strainer or similar and filtered through or a piece of gaze (pore size 70 - 100 μm) before adding FcBlock and staining with mAbs for analysis by flow cytomety (see Materials).

##### 16.3.2 Materials.

###### flow cytometry buffer:

PBS 3% FCS 4mM EDTA

###### Fc-Block:

Anti-FcR Ab, clone F2.4G2, homemade

###### Antibodies:

CD45-APC-eFluor780 (clone 104, eBiosciences, 1:100), γδTCR-FITC (clone GL-3, homemade, 1:100), αβ TCR-PerCPCy5.5 (clone H57-597, BD Biosciences, 1:100), and CD3-PECy7 (clone 145-2C11, eBiosciences, 1:200).

The data in this section were acquired using a BD LSRII Flow Cytometer equipped with blue, red, and violet lasers. Data were analyzed using FlowJo Version 10.

##### 16.3.3 Data analysis.

**Figure 56.**
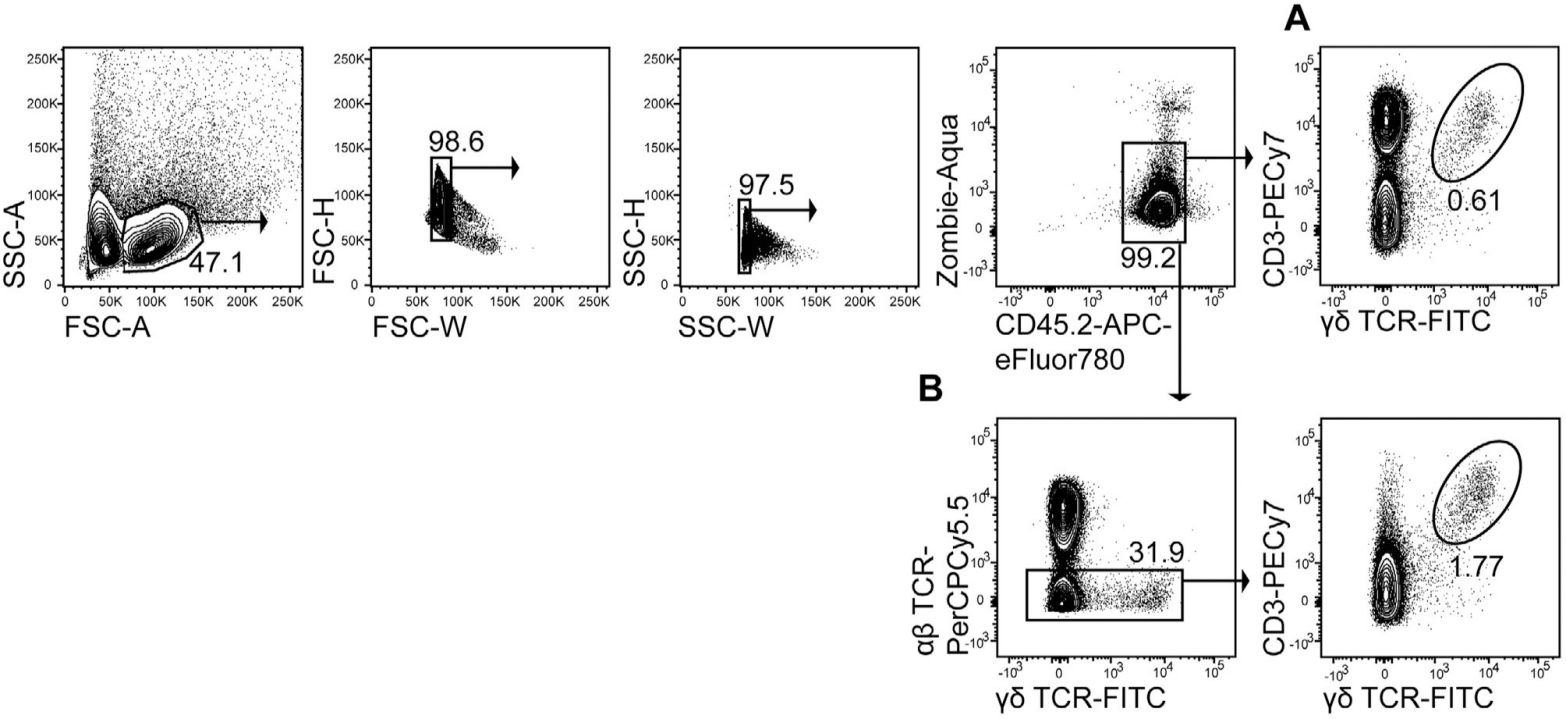
Murine pLNγδ T cells. Representative gating Lymphocytes were defined in FSC-A vs SSC-A plot. Subsequently doublets were excluded by FSC-H vs FSC-W and SSC-H vs SSC-W gating followed by exclusion of dead (Zombie-Aqua positive) and non-hematopoietic cells (CD45 negative) cells. (A) Representative contour plot for direct gating of γδ T cells defined as CD3^+^ γδ TCR^+^. (B) Representative contour plots for exclusion of αβ T cells before gating γδ T cells.

Practically, faithful detection of γδ T cells is warranted by gating on the lymphocyte area in the FSA versus SSA plot, gating out doublets, exclusion of dead and auto-fluorescent cells, followed by a positive gate according to the two parameters expression of γδ TCR, the best clone is GL3 [539] and CD3ε in the same plot (Figure 56A). If the experimental design and equipment allow further parameters, it is advisable to gate out αβ T cells (αβ TCR Ab, clone H-57) and eventually B cells (anti-CD19) prior to the γδ TCR versus CD3ε gate (Figure 56B). There are a number of mAb clones available for detection of γδ TCR using specific Vγ and Vδ segments. Below is a list of commercially available mAb clones (Table 56). However, be aware that several conflicting nomenclatures exist for mouse (and human) Vγ and Vδ segments, which can be misleading even for insiders to the field. Please check online resources such as the IMGT website (http://www.imgt.org) for further explanation. Here we use the so-called Heilig/Tonegawa nomenclature [540], while suppliers like BD Bioscience or BioLegend mainly utilize the Garman nomenclature [541].

One additional important, but not currently commercially available mAb clone is the clone 17D1 directed against Vγ5 from Bob Tigelaar (Yale University, New Haven, USA) [542]. Later, Christina Roark and colleagues found that 17D1 was cross-reactive to Vγ6 under certain conditions, see below [543].

##### 16.3.4 Pitfalls/top tricks.

**Figure 57.**
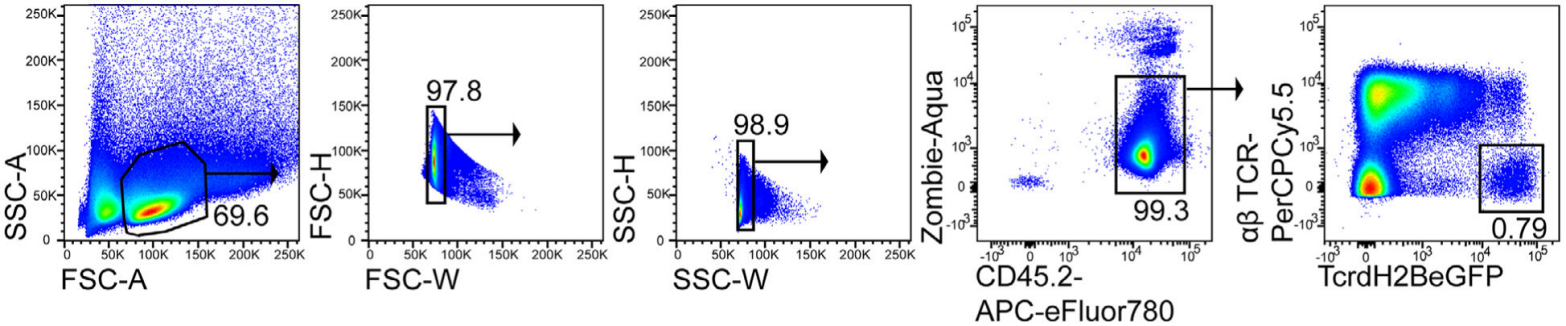
γδ T cells in Tcrd-H2BeGFP reporter mice. Representative gating strategy for the identification of genuine γδ T cells in pLN. Lymphocytes were defined in FSC-A vs SSC-A plot. Subsequently, doublets were excluded by FSC-H vs FSC-W and SSC-H vs SSC-W gating followed by exclusion of dead (Zombie-Aqua positive) and non-hematopoietic cells (CD45 negative) cells. Genuine γδ T cells were defined based on the H2BeGFP fluorescence in Tcrd-H2BeGFP mice and counterstaining with anti-TCRβ.

Furthermore, the *Tcrd*-H2BeGFP reporter mouse [544], JAX Stock No. 016941, can serve to detect γδ T cells independent of TCR expression on the cell surface (e.g., after TCR downregulation following strong TCR activation *in vitro*). Note that for visualizing γδ T cells in secondary lymphoid organs or other tissues containing a large excess of αβ T cells over γδ T cells in microscopy applications, the use of F1 heterozygotes from *Tcrd*-H2BEGFP mice and *Tcra*^−/−^ mice (B6.129S2-Tcratm1Mom/J, JAX Stock No. 002116) will genetically exclude highly fluorescent false-positive cells. In flow cytometry applications it is sufficient to counterstain for αβ TCR (Figure 57).

Notably, these *Tcrd*-H2BeGFP reporter mice were used to demonstrate that *in vivo* application of the mAb clones GL3 and UC7-13D5 does not lead to a depletion of γδ T cells, but rather renders them invisible for flow cytometric detection due to TCR downregulation [545]. However, a new genetic knock-in model for diphtheria toxin-mediated conditional γδ T cell depletion will circumvent these problems [546].

#### 16.4 Intracellular cytokine staining for IL-17-versus IFN-γ-producing γδ T cells

**Figure 58.**
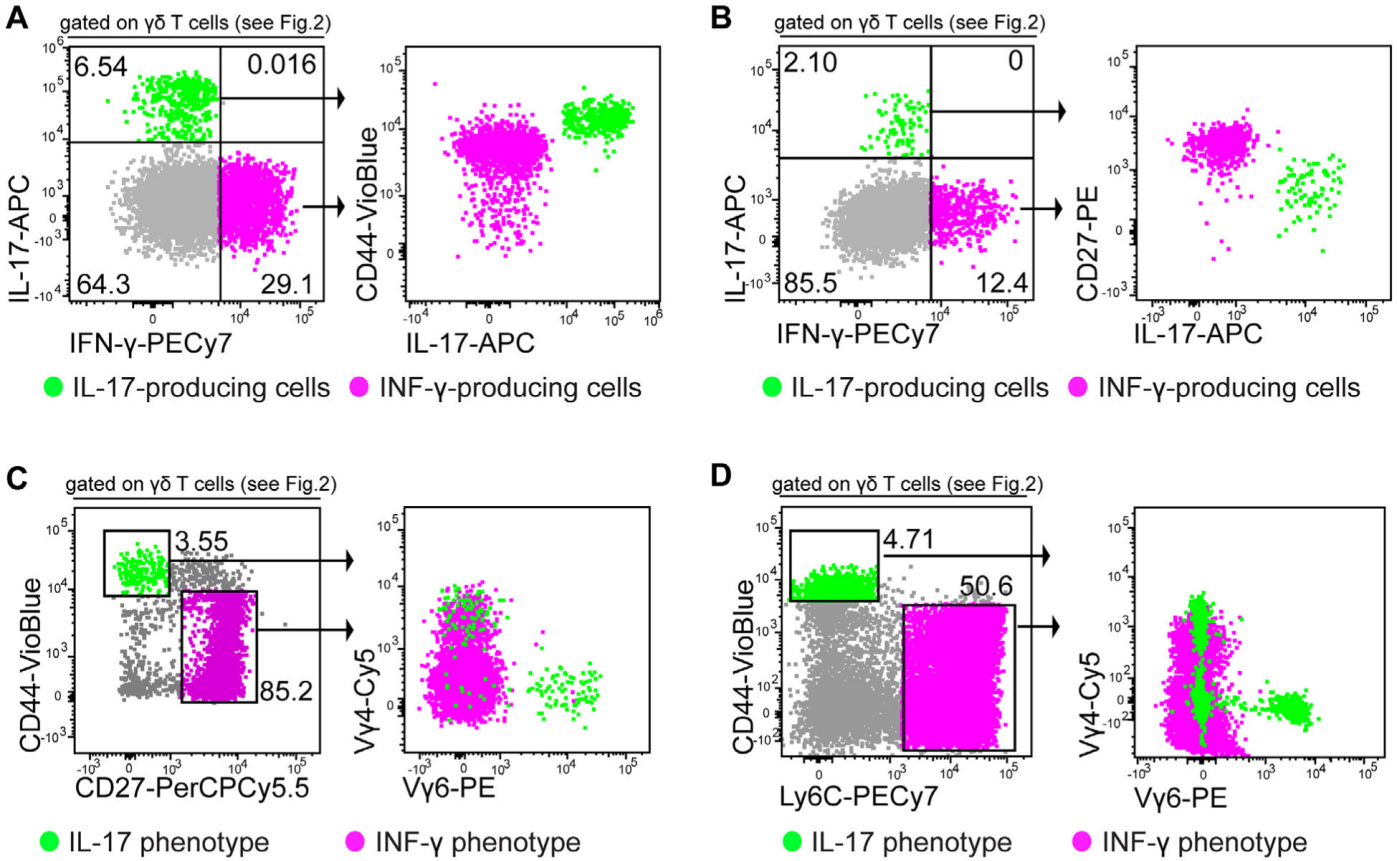
Murine IL-17- versus IFN-γ-producing γδ T cells. γδ T cells from pLN of Tcrd-H2BeGFP mice were gated as in Figure 57 above. (A-B) Intracellular cytokine staining in correlation to CD44 (A) and CD27 (B) surface marker expression. (C-D) Representative analyses of γδ T cells from pLN of Tcrd-H2BeGFP mice correlate CD27, CD44, and Ly6C surface staining to expression of Vγ4 and Vγ6.

Recent data supported the idea that discrete subsets of γδ T cells; namely IFN-γ- and IL-17-producing γδ T cells, develop and act as pre-activated effector cells. Notably, this becomes relevant since the two major effector subpopulations, IL-17 (Tγδ17 cells) and IFN-γ (Tγδ1 cells) γδ T cells, exert opposing roles in tumor microenvironments. Whereas IL-17 producing γδ T cells act mainly pro-tumorigenic, IFN- γ secreting γδ T cells are anti-tumoral [547]. In particular, the surface markers CD27, CD44, and Ly6C can be used to indirectly discriminate between those subsets without the need of intracellular cytokine staining. Surface staining against CD27, CD44, and Ly6C serves to discriminate activated γδ T cells with an IL-17-producing effector phenotype (CD44^high+^CD27^−^Ly6C^−^) or with an IFN-γ-producing effector phenotype (CD44^low/int^CD27^+^ Ly6C^+^) (Figure 58). In addition to this subdivision into either IL-17 or IFN-γ producing γδ T cells, CD44 and Ly6C can be used to discriminate naïve-like and memory-like cells similar to adaptive αβ T cells [548]. Further, the effector function is also associated with distinct γ-chain usage. Whereas Vγ4^+^ γδ T cells can either express IL-17 or IFN-γ, Vγ6^+^ γδ T cells are biased towards IL-17 secretion (Figure 58).

##### 16.4.1 Step-by-step sample preparation.

For Intracellular cytokine staining, isolated cells are first stimulated ex vivo with PMA and ionomycin together with brefeldin A for 3 h at 37°C. Next, stimulated cells are stained against extracellular surface molecules as described above. After fixation and permeabilization using the BD Cytofix/Cytoperm kit according to the manufacturer’s protocol, cells are stained for intracellular cytokines for 45 min on ice, washed once with flow cytometry buffer, and analyzed.

##### 16.4.2 Materials.

Phorbol-12-myristate-13-acetate (PMA, 50 ng/ml, Calbiochem)

Ionomycin (2 μg/ml, Invitrogen)

Brefeldin A (1 μg/ml, Sigma)

BD Cytofix/Cytoperm Kit (BD Biosciences)

###### Surface Abs:

CD27-PerCPC5.5 (LG.7F9, Biolegend, 1:100), CD44-VioBlue (clone IM7.8.1, Miltenyi Biotech, 1:50), Vγ4-Cy5 (clone 49.2-9, homemade, 1:50), Ly6C-PECy7 (AL21, BD Biosciences, 1:100), staining for Vγ6 see below.

###### Intracellular Abs:

IL-17A-APC (TC11-18H10.1, Biolegend, 1:100), IFN-γ-PECy7 (XMG1.1, Invitrogen, 1:100)

##### 16.4.3 Data analysis.

See section 16.3.3 Data analysis for peripheral lymph node γδ T cells.

##### 16.4.4 Pitfalls/Top tricks.

See section 16.3.4 Pitfalls/toptricks for peripheral lymph node γδ T cells.

#### 16.5 Isolation of lymphocytes from mouse ear skin

**Figure 59.**
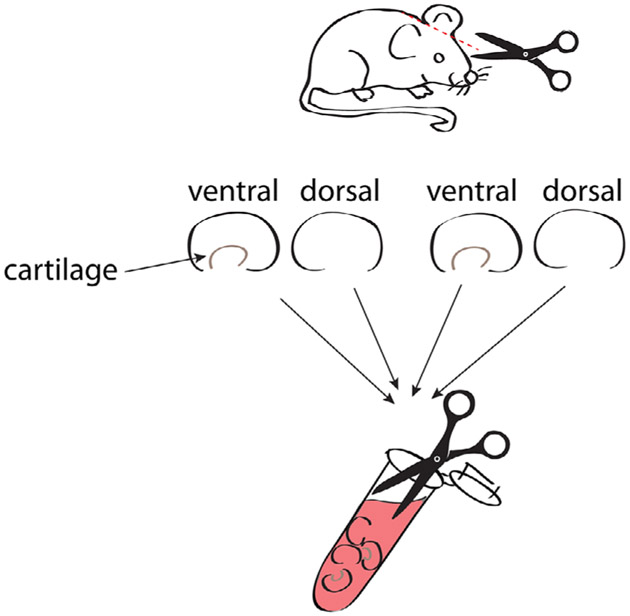
Dissection of mouse ear skin. Scheme depicting dissection of ear skin for subsequent isolation of lymphocytes.

The skin as well as several mucosal tissues such as the intestine harbor strong and thick walls on their surface, which are composed of different layers. Hence, the isolation of lymphocytes from these tissues warrants elaborated protocols. The skin consists of two primary layers, the keratinized epidermis on the surface and the beneath the dermis. In mice, lymphocytes of both, epidermal and dermal layers, can be preferably isolated from ear skin according to the following protocol (Figure 59).

##### 16.5.1 Step-by-step sample preparation.

1. Separate dorsal and ventral sites of the ears.
2. Remove the cartilage from the ventral sites.
3. Place the tissue (4 separated halves) in one 2 ml Eppendorf tube containing 1900 μl digestion medium and cut it into small pieces.
  - digest medium: RPMI (1810 μl)+ 2 mg/ml Col IV (40 μl of 100 mg/ml) + 187,5 μg/ml DNAseI (150 μl of 2.5 mg/ml)
4. incubate at 37°C, 1400 rpm, 75 min in an Eppendorf ThermoMixer
5. add EDTA, final concentration approx. 37,5 mM (+150 μl 0.5 M EDTA)
6. incubate for additional 15 min at 37°C, 1400 rpm (ThermoMixer)
7. dissociate the remaining tissue by sucking up and down the sample through an approx. 1-2 cm long 19G syringe needle
8. filter the sample through a Cellstainer (70 μm) and separate lymphocytes by density gradient centrifugation using Percollgradients (40% and 70% Percoll solutions)
9. isolated lymphocytes can be stained as described for pLN with the following Abs CD45.2, αβTCR, γδTCR, and CD3 and DAPI for live/dead discrimination.

##### 16.5.2 Materials.

RPMI 1640 Medium, Gibco

Collagenase IV, Worthington

DNAseI, Roche

DAPI, Sigma Aldrich (final concentration 0.1μg/ml)

Percoll, GE Healthcare

###### Antibodies:

γδTCR-APC (clone GL-3, Biolegend, 1:100), remaining Abs see above for peripheral lymph node γδ T cells.

##### 16.5.3 Data analysis.

**Figure 60.**
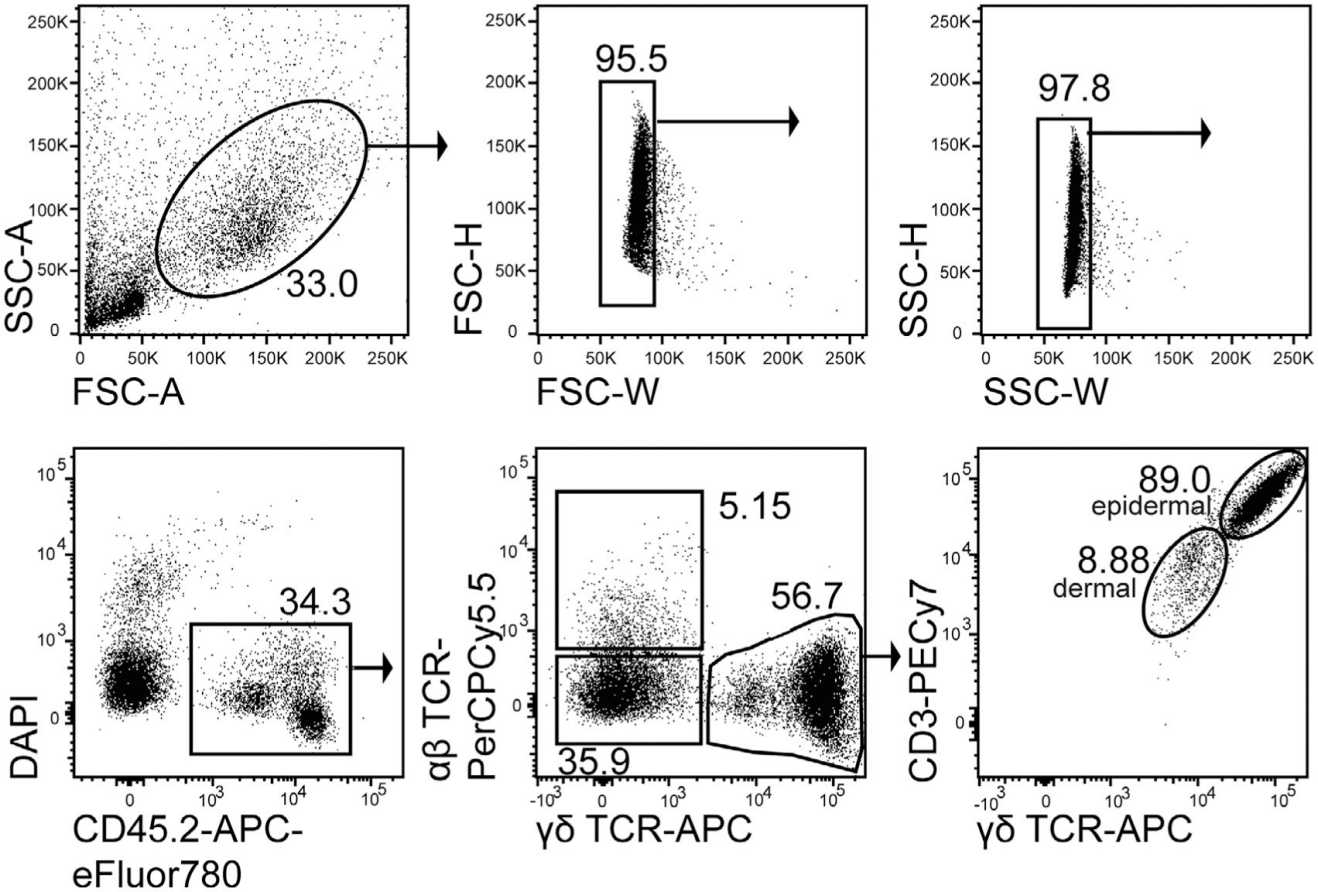
Murine ear skin γδ T cells. Representative gating strategy of murine ear skin cells stained with DAPI, CD45, αβ TCR, γδ TCR (GL3) and CD3 to detect dermal γδ T cells (CD3^+^ and γδ TCR^+^) and epidermal γδ T cells (DETC, CD3^hi^, and γδ TCR^hi^). Backgating was done by gating on lymphocytes in FSC-A vs SSC-A plot. Subsequently, doublets were excluded by FSC-H vs FSC-W and SSC-H vs SSC-W gating followed by exclusion of dead (DAPI positive) and non-hematopoietic cells (CD45 negative) cells. Total γδ T cells were defined as γδ TCR^+^ and αβ TCR^−^. Among total γδ T cells dermal and epidermal γδ T cells were discriminated based on the expression level of CD3 and γδ TCR.

The skin harbors a high amount of lymphocytes. Detection of γδ T cells is warranted by gating on the lymphocyte area in the FSA versus SSA plot, gating out doublets, exclusion of dead and CD45^−^ non-hematopoietic cells, followed by a γδ T cell gate according to the expression of γδTCR and lack of αβTCR expression. While αβ T cells are barely present in the mouse skin, the vast majority of lymphocytes are γδ T cells. γδ T cells localized in the epidermis (dendritic epidermal T cells (DETC)) can be easily distinguished from γδ T cells present in the dermis due to their high TCR expression levels as detected by γδTCR (GL3) and CD3 staining in (Figure 60).

Data were acquired using the same flow cytometer and analysis software as mentioned for pLN.

##### 16.5.4 Pitfalls/Top tricks.

See section 16.3.4 Pitfalls/toptricks for peripheral lymph node γδ T cells.

#### 16.6 The auxiliary Ab-assisted direct staining of Vγ6^+^ γδ T cells

Vγ6^+^ γδ T cells solely develop in embryonic thymus before birth, and later persist as long-lived self-renewing lymphocytes in the skin dermis and in many mucosal tissues such as the uterus or the tongue [549]. Vγ6^+^ γδ T cells recently sparked a lot of interest because they rapidly produce interleukin-17 and thus contribute to bacterial homeostasis and clearance, but also enhance autoimmunity and inflammatory diseases [550, 551]. The detection of Vγ6^+^ γδ T cells requires combined staining of γδ TCR together with the unconjugated rat 17D1 IgM Ab followed by a secondary staining with labeled anti-rat IgM. A validated staining protocol for the identification of Vγ6^+^ γδ T cells works as follows.

##### 16.6.1 Step-by-step sample preparation.

1. Prepare single-cell suspension
  Live dead discrimination was achieved either by staining with DAPI subsequent to surface marker staining or prior to blocking using Aqua Dead dye according to manufactures instructions.
2. Block cells with 5% Fc receptor block 5 min on ice.
3. Stain cells in Ab mix with extracellular surface markers (αβ TCR, CD3, Vγ4) and γδ TCR (GL3) diluted in flow cytometry buffer 15 min on ice
4. add unconjugated 17D1 (final dilution 1:25) and mix thoroughly (for example: add 4 μl of 17D1 to 100μl cell suspension) 30 min on ice
5. wash cells with flow cytometry buffer.
6. Stain cells with labeled secondary anti-IgM-PE Ab diluted in flow cytometry buffer 30 min on ice.
7. Wash cells with flow cytometry buffer, analyze cells by flow cytometry.

##### 16.6.2 Materials.

Zombie Aqua^™^ Fixable Viability Kit, Biolegend

αβ TCR-APCVio77 (clone REA318, Miltenyi, 1:50)

Vγ6 Ab, clone 17D1 provided by Bob Tigelaar

IgM-PE, clone RM-7B4, eBiosciences

Remaining Abs and reagents see above

##### 16.6.3 Data analysis.

See section 16.3.3 Data analysis for peripheral lymph node γδ T cells.

##### 16.6.4 Pitfalls/top tricks.

**Figure 61.**
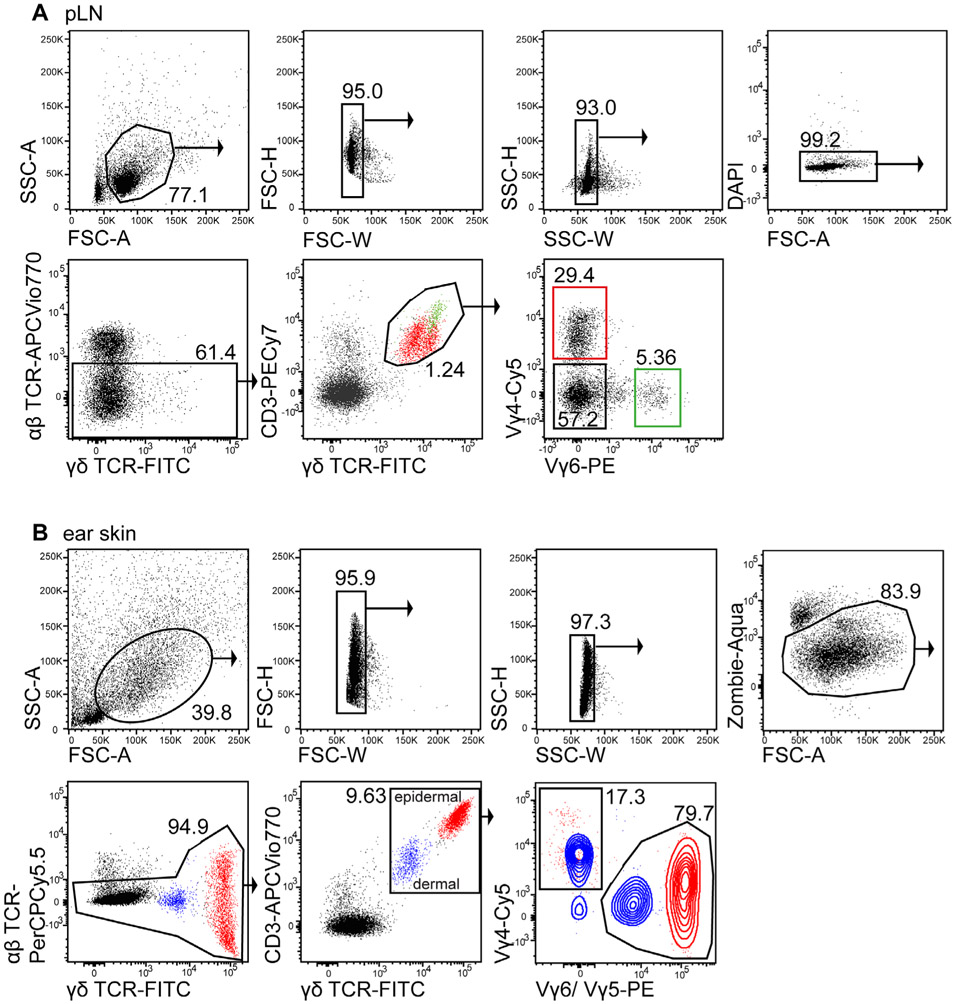
Murineγδ T cell subpopulations according to TCR expression. Representative gating strategies of murine Vγ4^+^ (red) and Vγ6^+^ (green) _γ_δ T cells in pLN (A) as well as epidermal murine Vγ5^+^ γδ T cells (DETCs, red) and dermal murine Vγ4^+^ and Vγ6^+^ γδ T cells (blue) in ear skin (B). Backgating was done by gating first on lymphocytes in FSC-A vs SSC-A plot. Subsequently doublets were excluded by FSC-H vs FSC-W and SSC-H vs SSC-W gating followed by exclusion of dead (DAPI positive or Zombie-Aqua+) cells. Among TCRß^−^ cells total γδ TCR ^+^CD3^+^ γδ T cells were gated and finally separated into different γδ subsets by staining with anti-Vγ4 as well as 17D1 followed by conjugated anti-IgM to detect Vγ6^+^/Vγ5^+^ γδ T cells.

Importantly, in skin, clone17D1 not only stains Vγ6^+^ γδ T cells in combination with GL3, but also recognizes the Vγ5 gene segment expressed in dendritic epidermal T cells (DETC). However, dermal Vγ6^+^ γδ T cells and epidermal Vγ5^+^ γδ T cells can be easily distinguished because of the very high TCR levels in Vγ5^+^ γδ T cells leading to bright γδ TCR and CD3 staining. The epidermis solely contains Vγ5^+^ γδ T cells, while the dermal compartment comprises high frequencies of Vγ4^+^ and Vγ6^+^ γδ T cells. (Figure 61).

It follows that an additional counterstaining of 17D1^+^ skin T cells with a specific anti-Vγ5 mAb clone 536, see Table 56, would further help to discriminate between dermal and TCR^high^ epidermal T cells (Figure 60B and *not shown*). In contrast, peripheral lymph nodes lack Vγ5^+^ γδ T cells. While Vγ6^+^ γδ T cells only represent a small population in peripheral lymph nodes, a large proportion of γδ T cells are Vγ4^+^ γδ T cells and Vγ6^−^Vγ4^−^ γδ T cells (mainly Vγ1^+^ T cells).

#### 16.7 Summary of the phenotype

This is detailed in Table 57.

#### 16.8 Key information human vs murine

This is detailed in Table 58.

### 17 Unconventional human T cells: NKT cells

#### 17.1 Overview

Natural Killer T cells (NKT cells) are a subset of lipid-reactive T cells restricted to the MHC I-like molecule CD1d. Like other ‘unconventional’ T cell subsets (such as MAIT and γδ T cells), NKT cells display a memory-like phenotype, rapidly releasing a broad array of cytokines following activation [511]. Indeed, the functional diversity displayed by NKT cells underpins their immunomodulatory role within various diseases, including infection, autoimmunity, and cancer [552-554]. The NKT cell family can be subdivided into Type I and Type II NKT cells based on TCR gene usage and/or CD1d-lipid Ag reactivity [555]. As methods to definitively identify human Type II NKT cells are still developing, this section will only provide guidelines for the identification of the more extensively described Type I, or invariant (iNKT) NKT cell subset using flow cytometry, with an emphasis upon the different reagents and techniques required to study these evolutionarily conserved cells.

#### 17.2 Introduction

NKT cells were initially described in mice as a lymphocyte population displaying restricted αβ TCR usage that co-express the NK cell-associated receptor NK1.1, which resulted in the moniker ‘Natural Killer T cells’ being adopted [556] (See section Murine NKT cells). However, expression of NK1.1 (*Klrb1c*) in mice, and CD161 (*KLRB1*) in humans have since been shown to be inconclusive markers of this T cell subset. As such, these cells are now more definitely categorized by their T cell receptor (TCR) usages, and recognition of particular CD1d-ligands. The canonical human NKT cell TCR is composed of an invariant α-chain comprising Vα24 and Jα18 (TRAV10 and TRAJ18) gene segments, paired with a Vβ11^+^ (TRBV25-1) β-chain. This represents a TCR heterodimer that is highly conserved throughout the human population, with orthologous sequence usages common to other mammalian species [557-560]. Due to this limited TCR diversity, this T cell subset is often referred to as ‘invariant’, or iNKT cells from primates to rodents [556]. One of the hallmarks of iNKT cell TCRs is their recognition of the prototypic CD1d-ligand α-galactosylceramide (α-GalCer) [561]. This interaction is used to demarcate Type I (α-GalCer-reactive) NKT cells from the more TCR-diverse (α-GalCer-non-reactive) Type II NKT cell subset, which can recognize CD1d in the context of other Ags. However, not all NKT cells capable of recognizing the CD1d-α-GalCer complex express the Vα24-Jα18/Vβ11 NKT cell TCR. For example, human CD1d-α-GalCer reactive αβ T cells that lack Vα24 and/or Vβ11 expression have been widely described [562-566] and γδ T cells that interact with CD1d-α-GalCer have also been reported [567]. Thus, an appreciation of the complexities that exist within the CD1d-restricted T cell pool is crucial for those wanting to investigate glycolpid-reactive T cells. Although it is common for the terms “NKT cells,” “iNKT cells,” and “Type I NKT cells” to be used synonymously, there are significant differences between these classifications, and differences in the approaches used to identify the cells. The best choice for individual researchers will depend on the specific question(s) they are aiming to address. Herein, we discuss the various ways that NKT cells can be identified using flow cytometry, and the potential advantages and disadvantages of these alternate methodologies.

##### 17.2.1 Identifying human NKT cells via their antigenic recognition.

The prototypic NKT cell Ag KRN7000 was developed by the Kirin brewing company in the 1990s [561, 568, 569]. This α-linked galactosylceramide compound was modeled on glycolipids isolated from extracts of marine sponge displaying anti-tumor activity in murine disease models. This reagent (KRN7000) has since been widely used to study the functional properties of both mouse and human NKT cells [570]. Despite the curious origins of this T cell Ag, structurally similar glycolipids have since been identified from numerous bacterial species [571], supporting the physiological relevance of NKT cell-specificity for such compounds. In addition to KRN7000, various α-GalCer analogs have also been synthesized, which elicit distinct functional responses [572-574] and display varied affinities for NKT cell TCRs [575, 576]. Whilst the potency of KRN7000 has made it the most widely used Ag for investigating NKT cell function, its saturated lipid-component renders it a relatively insoluble reagent. Thus, for the purpose of identifying NKT cells using CD1d-tetramers it can prove suboptimal, due to inefficient loading into CD1d in a cell-free, *in vitro* setting. Due to this, more soluble α-GalCer analogs (with unsaturated lipid fractions), such as PBS-44 [577] and PBS-57 [578] have become the preferred alternatives. For researchers wanting to investigate Type I or iNKT cells, PBS-57-loaded CD1d tetramers are currently available from the NIH tetramer core facility (http://tetramer.yerkes.emory.edu/reagents/cd1), conjugated to a number of fluorochromes. Alternatively, the manufacture of soluble CD1d for tetramer manufacture is a viable option, described elsewhere within the literature [579-581]. For those wishing to explore the differential antigenic-recognition characteristics within the larger CD1d-restricted T cell pool [555], the ‘in-house’ production of CD1d tetramers loaded with the Ag of choice will provide scope to interrogate NKT cell interactions with the diverse range of established and putative CD1d-ligands.

##### 17.2.2 Identifying human NKT cells via their TCR usage.

The highly conserved TCR usage displayed by Type I or iNKT cells is a factor that can be exploited to identify these cells via flow cytometry, and there are two approaches commonly used to achieve this. First, co-staining with anti-Vα24 and anti-Vβ11, which enriches T cells expressing the canonical iNKT cell TCR αβ heterodimer. However, there is no assurance that all cells isolated by this means will be α-GalCer-reactive, or CD1d-restricted. The second, and arguably more accurate approach is using the monoclonal Ab 6B11, which displays specificity toward the CDR3α loop of the Vα24-Jα18 iNKT cell TCR [582, 583]. This method, in conjuncture with anti-Vβ11 staining will further ensure isolation of cells that express the canonical iNKT cell TCR.

#### 17.3 Step-by-step sample preparation

**Figure 62.**
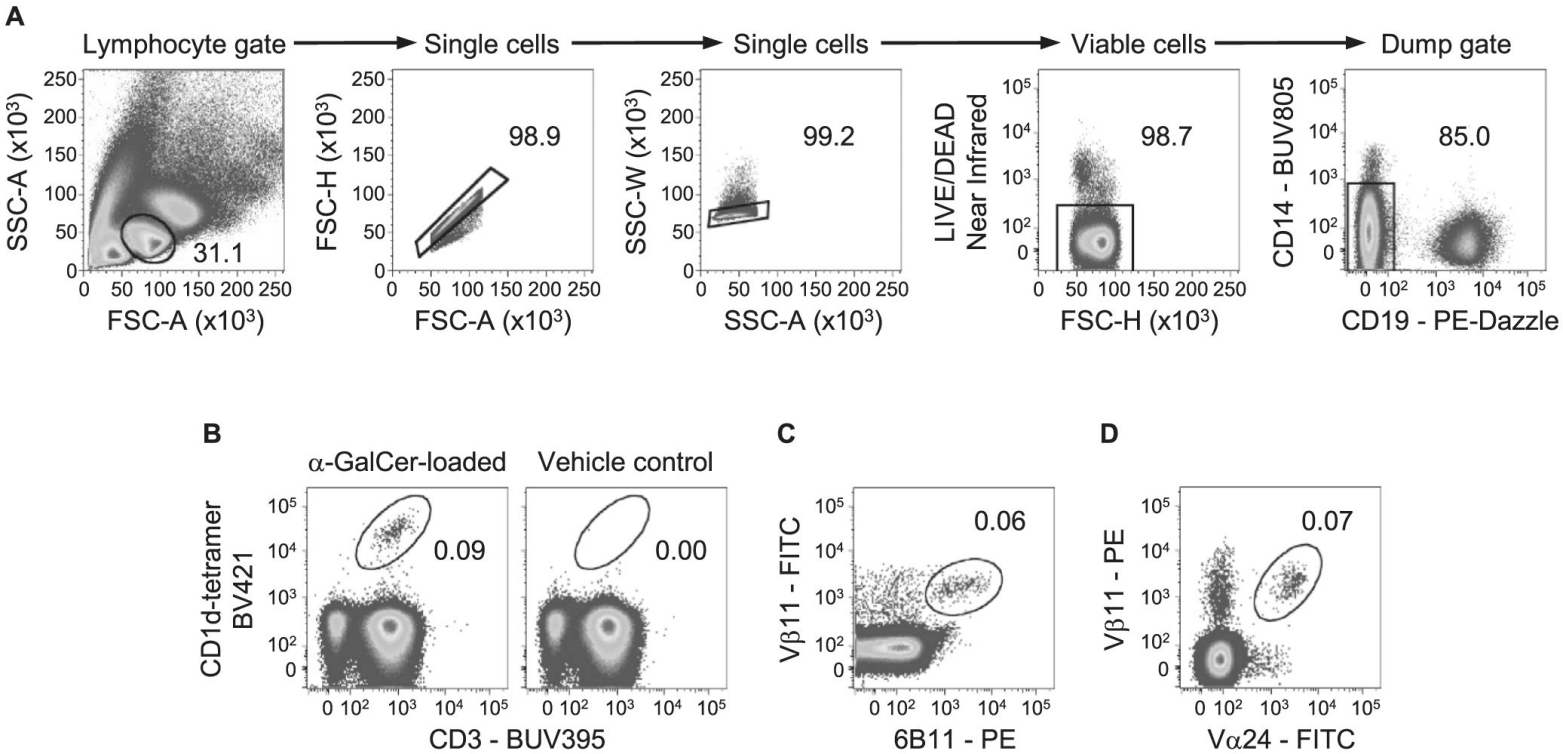
Gating on human blood NKT cells. (A) Lymphocytes are distinguished amongst PBMCs based on their relative FSC-A and SSC-A intensities. Single cells are then isolated by their relationship between FSC-H versus FSC-A, and SSC-W versus SSC-A. To remove any non-specific or TCR-independent CD1d-tetramer staining, dead cells are removed from analysis based on their uptake of LIVE/DEAD^™^ Fixable Near-IR viability dye. Monocytes and B cells are also excluded based on their CD14 and CD19 expression respectively. (B) The frequency of circulating Type I NKT cells, as determined by co-staining for CD3ε and α-GalCer (PBS-44)-loaded CD1d-tetramer (left) in relation to a vehicle control CD1d-tetramer (right). (C) The frequency of iNKT cells was assessed by co-staining with 6B11 and anti-Vβ11. (D) Co-staining with anti-Vα24 and anti-Vβ11, which non-exclusively enriches for iNKT cells.

The following methodologies specifically relate to the experiment depicted in Figure 62. However, adherence to these general principles will enable the specifics to be adjusted to suit other experimental requirements.

##### 17.3.1 Protocol: Generating α-GalCer-loaded CD1d-tetramer.

Human CD1d was generated in modified human embryonic kidney 293S cells (HEK293S cells) and enzymatically biotinylated as previously described [584, 585].

- Biotinylated monomeric CD1d was loaded with α-GalCer (0.5% [v/v] tyloxapol in TBS) by incubation overnight at 37C at a 6:1 molar ratio (α-GalCer:CD1d). Vehicle control included the tyloxapol in TBS solution only.
- CD1d tetramers were generated in house using 10 μg of biotinylated CD1d monomer, mixed with a total 2 μg of streptavidin BV421 added in eight sequential steps (in order to optimize CD1d tetramerization) at 10 min intervals at 4°C.

##### 17.3.2 Protocol: Isolation and analysis of NKT cells in cryopreserved PBMCs.

- Add thawed PBMCs (5 × 10^7^ cells) to a 50 ml canonical tube containing 40 ml of RF-10 (Gibco) media.
- Centrifuge cell suspension for 4 min with 400 × *g* at 4C.
- Aspirate supernatant and resuspend cellular pellet with 20 ml RF-10 (Gibco) containing 100 μg DNase I (Sigma-Aldrich) keep cell suspension at 4C for 30 min.
- Pass cell suspension through a 70μm cell strainer.

##### 17.3.3 Protocol: Surface staining.

- Centrifuge cell suspension for 4 min with 400 × *g* at 4C.
- Aspirate supernatant and resuspend cellular pellet with 500 μl of (1:10 diluted) human FcR-blocking reagent (Miltenyi Biotec) in FACS buffer and incubate at 4°C for 20 min.
- Aspirate supernatant and resuspend cellular pellet with 500 μl with of LIVE/DEAD^™^ Fixable Near-IR (ThermoFisher) diluted in PBS, keep cell suspension in the dark at 4°C for 30 min (The concentration of this reagent should be titrated to ensure optimal staining).
- Add 20 ml of FACS buffer and centrifuge cell suspension for 4 min with 400 × *g* at 4°C.
- Aspirate supernatant and resuspend cellular pellet with 200 μl FACS buffer with diluted anti-CD3ε, anti-CD14, and anti-CD19 (Table 59). Keep cell suspension in the dark at 4°C for 30 min.
- Add 20 ml of FACS buffer and centrifuge cell suspension for 4 min with 400 × *g* at 4°C.
- Aspirate supernatant and resuspend cellular pellet in 20 ml of FACS buffer, then split sample evenly into four 10 ml conical tubes.
- Centrifuge cell suspensions for 4 min with 400 × *g* at 4°C.
- Aspirate supernatant and resuspend cellular pellet with 100 μl FACS buffer containing CD1d tetramers (1 μg/ml), or a combination of anti-Vβ11 and anti-Vα24, or anti-Vβ11 and 6B11 (Table 59). Keep cell suspension in the dark at 4°C for 30 min.
- Add 5 ml of FACS buffer and centrifuge cell suspension for 4 min with 400 × *g* at 4°C. Aspirate supernatant and repeat this step.
- Aspirate supernatant and resuspend cellular pellet with 400 μl FACS buffer.
- Analyse samples on the flow cytometer.

#### 17.4 Materials: Isolation and analysis of NKT cells in human blood

RF-10 media

- Base Medium RPMI media 1640, no glutamine (Gibco)
- 10% heat-inactivated fetal calf serum (Gibco)
- 2 mM l-Glutamine (Gibco)
- 0.1 mM Non-Essential Amino Acids (Gibco)
- 15 mM HEPES (Gibco)
- 100 U/ml of Penicillin/Streptomycin (Gibco)
- 1 mM Sodium Pyruvate (Gibco)
- 50 mM 2-Mercaptoethanol (Sigma-Aldrich)

##### FACS buffer

- Base Medium 1× PBS (made in-house)
- 2% heat-inactivated fetal bovine serum (Gibco)

DNAse I (Sigma-Aldrich)

Human FcR-blocking reagent (Miltenyi Biotec)

LIVE/DEAD^™^ Fixable Near-IR Dead Cell Stain Kit, for 633 or 635 nm excitation (ThermoFisher)

Tyloxapol (Sigma-Aldrich)

Streptavidin BV421 (BioLegend)

- α-GalCer (PBS-44) was supplied by Prof. P. Savage, Brigham Young University
- α-GalCer (KRN7000) is commercially available from several suppliers (http://www.enzolifesciences.com/BML-SL232/krn7000/, https://avantilipids.com/product/867000)
- α-GalCer (PBS-57)-loaded CD1d tetramer is available from the NIH tetramer core facility (http://tetramer.yerkes.emory.edu/reagents/cd1)

##### 17.4.1 Flow cytometer.

BD LSR Fortessa^™^ 5-laser configuration (UV, Violet, Blue, Yellow-Green, Red) was used in the experiments depicted here. However, similar results can be achieved using other multi-parameter flow cytometers.

##### 17.4.2 Analysis.

FlowJo (TreeStar)

#### 17.5 Data analysis

The following descriptions and figures specifically relate to the identification of NKT cells from human blood samples. However, these general principles can also be applied to the detection of NKT cells from other tissues. Standard procedures for the isolation of peripheral blood mononuclear cells (PBMCs) can be used for the study of human NKT cells, including density gradient centrifugation with Ficoll-Paque at a density of 1.077 g/ml [586]. A typical gating strategy for detecting human blood NKT cells is depicted in Figure 62A. This firstly involves gating on lymphocytes based on a combination of their size and granularity, assessed by their forward scatter area (FSC-A) and side scatter area (SSC-A) intensities, respectively. The exclusion of doublets should also be included, as the failure to do so may lead to false-positive staining being incorporated during analysis. There are several techniques to exclude doublets, Figure 62A depicts an example of single cells being identified based on their relative forward-scatter area (FSC-A) and forward-scatter height (FSC-H). As these two parameters both provide readouts of cell-size, single cells exhibit a linear relationship between these two parameters enabling doublets to be excluded. Whilst a secondary doublet exclusion gate is not essential, their elimination can be further assured based on assessing relative side-scatter (SSC) intensities. For example, the relationship between SSC-A and SSC-H is used to exclude doublets within Figure 62A. Although it is not uncommon for researchers to rely solely on FSC and SSC to differentiate between live and dead cells, this technique is not 100% effective, as seen in Figure 62A. In this example, LIVE/DEAD fixable Near-IR cell viability dye has been used, which shows that some dead cells have been incorporated within the previous gates. Thus, the inclusion of a viability dye is highly recommended to exclude non-specific Ab, or CD1d-tetramer staining of dead cells, or cellular debris - a consideration that is particularly important when investigating rare populations, such as NKT cells. In order to further discount any TCR-independent CD1d-tetramer binding that may occur, B cell (CD19^+^) and monocyte (CD14^+^) markers are typically included within Ab cocktails to facilitate their exclusion (Figure 62A). From this point, Type I, or iNKT cells can be identified, enumerated, or sort-purified by the methods described below.

##### 17.5.1 α-GalCer-loaded CD1d-tetramer.

Type I NKT cells can be identified based on their double-positive staining for α-GalCerloaded CD1d-tetramer and anti-CD3ε. This is depicted within Figure 62B in relation to a CD1d-tetramer control, which has been exposed to the vehicle reagent (0.5% tyloxapol/TBS) used to solubilize α-GalCer (PBS-44). As this technique relies on CD1d-Ag recognition rather than TCR usage, it has the potential to isolate cells that do not express the canonical iNKT cell TCR [562-567]. As such, cells isolated by this manner are more accurately described as Type I NKT cells, rather than iNKT cells. However, iNKT cell TCR usage amongst α-GalCer-loaded CD1d-tetramer positive cells can be addressed by co-staining with anti-Vα24 [587].

##### 17.5.2 6B11 and anti-Vβ11 co-staining.

The 6B11 Ab clone recognizes the canonical iNKT cells TCR α-chain [582, 583]. Hence, the vast majority of 6B11-reactive T cells from individual donors will also co-stain with anti-Vβ11, as seen within Figure 62C. However, a point worth mentioning is that the proportion of 6B11^+^, Vβ11^+^ cells detected within any given sample may differ from that of α-GalCer-loaded CD1d-tetramer^+^ CD3^+^ cells (Figure 62B), as the recognition of this complex can be achieved by T cells with atypical sequences [562-567]. While in the majority of cases these differences may appear negligible [588], variations can occur based on the technique being employed (Figure 62B and C).

##### 17.5.3 Anti-Vα24 and anti-Vβ11 co-staining.

T cells expressing the canonical iNKT αβ TCR heterodimer can be enriched for by co-staining with Abs against both Vα24 and Vβ11, as depicted within Figure 62D. Whilst this technique may identify a population of cells that largely overlap with that of Type I (Figure 62B), or iNKT cells (Figure 62C), there is no assurance that cells isolated by this approach will contain the iNKT cell TCR, or even be CD1d-restricted. Hence, despite this method providing a useful means of enriching for, or approximating ‘NKT cell’ numbers, this technique is considered the less stringent of those exemplified here.

#### 17.6 Pitfalls: Isolation and analysis of NKT cells in human blood

- Adherence to the strategies described above should prevent autofluorescent cells from being falsely incorporated within NKT cell (α-GalCer-loaded CD1d-tetramer versus CD3ε) gates during the analysis of human PBMC samples. However, the presence of autofluorescent cells can be more pronounced with other tissue samples. Therefore, an appreciation of the potential for autofluorescence to adversely impact flow cytometric data [589], and the knowledge of techniques that can prevent such complications are crucial for all flow cytometry users, particularly those wishing to study rare populations such as NKT cells. When designing Ab cocktails it is advisable to leave a channel open for autofluorescence detection, such as 530/30-blue (FITC), 450/40-violet (BV421), or 525/50-violet (BV510). If spectral overlap has been correctly compensation for, positive signaling within this parameter can be used to exclude autofluorescence.

#### 17.7 Top tricks: Isolation and analysis of NKT cells in human blood

- Whilst not typically required when working with fresh PBMCs, it can be beneficial to treat cryopreserved PBMCs with DNase after thawing. This will digest ‘sticky’ DNA released by cells lysed during this process, preventing cellular-pellets from irreversibly clumping following centrifugation steps.
- The efficiency of glycolipid-loading into CD1d in an *in vitro*-setting is typically Ag-dependent. Therefore, various conditions such as molar loading ratio, loading temperature, and the pH condition all need to be considered when assessing NKT cell-recognition of CD1d-ligands via the use of lipid-loaded CD1d-tetramers.
- Despite CD1d-ligands such as α-GalCer typically being solubilized in Tween 20-based (0.5% [v/v] Tween 20, sucrose [56 mg/ml] and L-histidine [7.5 mg/ml] in PBS) vehicle reagent or DMSO for *in vitro* studies, the use of a tyloxapol-based (0.5% [v/v] tyloxapol in TBS) vehicle may enhance loading efficiencies of some glycolipid-Ags for CD1d-tetramer studies [590].
- Although the strength of the interaction between the iNKT cell TCR and α-GalCer-loaded CD1d-tetramer is sufficient to enable clear detection of iNKT cells with CD1d-tetramers generated with streptavidin conjugated to a number of different fluorochromes (http://tetramer.yerkes.emory.edu/reagents/cd1), the study of lower avidity interactions may require the use of streptavidin conjugated to fluorochromes displaying optimal signal-to-noise ratios, such as phycoerythrin (PE).
- Due to the rarity of NKT cells within human blood (typically ranging from 0.01 to 0.1% of lymphocytes) [587, 588], it can be useful to enrich these cells prior to cell sorting, or general Flow cytometry analysis using techniques such as anti-PE-magnetic microbead enrichment (https://www.miltenyibiotec.com/AU-en/products/macs-cell-separation/cell-separation-reagents/microbeads-and-isolation-kits/any-cell-type/anti-pe-microbeads.html). These procedures can be employed to enrich for α-GalCer-loaded CD1d-tetramer, Vα24, Vβ11 or 6B11 positive cells. The choice depending on the individual needs of the researcher, and the availability of reagents conjugated to a suitable fluorochrome.

#### 17.8 Clinical relevance statement

The highly conserved nature of human CD1d and the NKT cell TCR makes this molecular interaction an exploitable immune target with applicability across all individuals. The potential for the CD1d-mediated activation of NKT cells to treat or prevent disease has been demonstrated extensively in preclinical models of cancer, autoimmunity, and infectious disease [570, 591-593]. Informed by these promising preclinical results, several human clinical trials have been undertaken that targeted NKT cell-activation as a therapeutic device, the bulk of which being focussed on the effectiveness of stimulating NKT cells in a cancer setting [594]. However, clinical trials have also been carried out to assess the therapeutic scope of NKT cells during chronic viral infections [595-597]. While there have been some encouraging results from these clinical trials, this in an evolving field with new techniques being continually developed and updated in order to improve the efficacy of NKT cell-based therapies, such as CAR-NKT cell therapies [598, 599], and α-GalCer-peptide Ag conjugation [600]. These novel and improved techniques, together with further research of human NKT cell biology will be essential to fully exploit this population within the clinic.

#### 17.9 Summary of the phenotypes

This is detailed in Table 60, with references [601-605].

### 18 Unconventional murine T cells: NKT cells

#### 18.1 Overview

Murine NK T (NKT) cells were originally defined by their coexpression of surface markers characteristic for T cells (i.e., the TCR) and NK cells (e.g., NK1.1 in C57BL/6 mice) [606, 607]. This chapter focuses on the phenotypic characterization of so-called murine invariant (i)NKT cells, which express an invariant Vα14Jα18 TCRα chain and a limited set of TCRβ chains with a preference for Vβ8, Vβ7, and Vβ2 [608, 609]. iNKT cells recognize lipids, such as α-galactosyl ceramide (αGalCer), in the context of the non-classical MHC molecule CD1d [610]. As a consequence, iNKT cells can be unambiguously identified by surface staining using CD1d tetramers loaded with αGalCer or its derivatives, such as PBS-57 [579, 611]. Subphenotyping of developmental stages in the thymus and effector subsets based on surrogate surface markers and key transcription factors is described.

#### 18.2 Introduction

Development of iNKT cells diverges at the CD4^+^CD8^+^ double-positive stage of T-cell development. Selection of iNKT cells is mediated by cortical thymocytes rather than epithelial cells. Similar to other unconventional T cells, iNKT cells are selected by strong TCR signals in a process referred to as agonist selection [612]. iNKT cells, with the notable exception of some tissue resident subsets, express and are dependent on the prototypical transcription factor for innate-like T cells, PLZF (encoded by *Zbtb16*) [604, 613]. Intrathymic development of iNKT cells has originally been described to progress through four phenotypically distinct stages (stage 0 – stage 3), characterized by differential expression of the surface markers CD24, CD44, and NK1.1 (in C57BL/6 mice) as well as cell size [614-616]. More recent studies showed that stage 3 iNKT cells represent long-term resident cells in the thymus [617, 618]. The thymus of young adult C57BL/6 mice contains around 3 - 6*10^5^ iNKT cells, corresponding to an overall frequency of 0.3–0.5% of all thymocytes.

More recently, iNKT cells have been categorized into functional subsets based on the expression of type 1, type 2, or type 17 cytokines [619]. Like their conventional T-cell counterparts, NKT1 cells are characterized by expression of the transcription factor T-bet, NKT17 cells express RORγt, whereas NKT2 cells are most frequently characterized by absence of expression of both transcription factors while simultaneously expressing very high levels of PLZF. The prototypic type 2 transcription factor GATA-3 is variably expressed in all iNKT cells and cannot be employed for discrimination of NKT2 cells. As a consequence, in the thymus, PLZF^hi^ NKT cells contain both, precursors (NKTp) and NKT2 cells. These cells can be further distinguished by differential expression of CCR7 (NKTp) and PD-1 (NKT2) [617].

Notably, relative proportions of the three NKT subsets vary widely between mouse strains with BALB/c mice showing a strong bias towards NKT2 cells in the thymus, whereas thymi in C57BL/6 mice predominantly contain NKT1 cells [619]. To circumvent intracellular staining for transcription factors, discrimination of NKT-cell subsets can also be achieved by analysis of surface expression of CD4 and CD122 (thymus) or CXCR3 (periphery) or CD43HG and ICOS [620, 621]. Consistent with the phenotypic overlap and the late acquisition of NK1.1 in the “linear” differentiation model, developmental trajectories proposed based on single-cell RNAseq data suggested that at least some NKT2 cells are transitory and contain progenitors for both NKT1 and NKT17 subsets [622]. An integration of the initial “linear” model of NKT-cell differentiation from stage 0 to stage 3 and differentiation into defined effector subsets has recently been outlined by Benlagha and colleagues [623].

Outside the thymus, iNKT cells can be found in lymphoid and as tissue resident cells in non-lymphoid organs with distinct subset cmposition for each organ (for review [624, 625]. In mice (but not humans) up to 40% of all mononuclear cells in liver constitute iNKT cells [579, 606]. The vast majority of these cells are of the NKT1 type. Upon stimulation iNKT cells rapidly produce large amounts of cytokines essentially according to their transcription factor profile with the notable exception of IL-4, which can be produced by all subsets. Similar to other unconventional T cells, iNKT cells are considered innate-like, because they can be stimulated both by cognate ligand via the TCR and in a non-cognate manner through LPS or cytokines like IL-12 and IL-18 [626, 627]. Whereas stimulation through the TCR results in rapid release of multiple cytokines, non-cognate stimulation results mostly in the production of IFN-γ.

NKT cells serve a vast variety of functions shaped by their distinct tissue distribution (reviewed in [624, 628]). Thus, NKT cells may protect from infection in lung and liver, but may exacerbate inflammatory conditions and asthma. Although being comparatively rare in intestinal tissues, NKT cells contribute to tissue homeostasis and to shaping the intestinal microbiota. Other roles in tissue homeostasis comprise regulation of T-cell development and egress from the thymus through IL-4 as well as protective functions in type 1 diabetes and graft-versus-host disease [629].

#### 18.3 Step-by-step sample preparation

##### Cell isolation:

Single-cell suspensions of whole lymphoid organs (thymus, spleen, lymph nodes) are generated by crushing organs through a 70-μm filter. Red blood cells (RBCs) are lysed (spleen only) using Qiagen RBC Lysis Solution according to the manufacturer’s instructions. For lymphocyte isolation from the lung and liver, mice are euthanized and liver/lungs are immediately perfused with phosphate-buffered saline (PBS). Lymphocytes are then isolated using standard procedures for solid organs or using commercially available kits, for instance as described in [630].

##### Surface staining:

Following incubation with Fc block (anti-mouse CD16/32, clone 2.4G2) cells are stained using APC-conjugated CD1d-PBS-57 or CD1d-unloaded (background control) tetramers for 30 min at room temperature in FACS buffer [631]. Cells are washed once in FACS buffer followed by Ab staining for surface markers for 10 min at 4°C. In order to minimize background, it is pivotal to perform lineage exclusion by staining for the following markers: B220, CD19, CD11b, and CD11c. Dead cells are excluded using the Zombie Aqua Fixable Viability kit as per manufacturer’s instructions (Biolegend).

##### Magnetic-bead enrichment:

Following CD1d-PBS57-APC tetramer staining, iNKT cells may be enriched using anti-APC magnetic microbeads following the manufacturer’s instructions (Miltenyi Biotec).

##### Intracellular staining:

To analyze transcription factor expression, magnetic-bead-enriched CD1d-PBS-57 tetramer^+^ cells from lymphoid organs are stained for surface markers and viability as described above. Samples are then fixed and permeabilized using the Foxp3/Transcription Factor Staining Buffer Set (eBioscience) as per the manufacturer’s instructions, following which, cells are stained for intracellular transcription factors for 30 min or overnight.

#### 18.4 Materials

FACS buffer: PBS, 3% FCS

RBC lysis buffer (Qiagen)
Zombie Aqua Fixable Viability kit (Biolegend)
Anti-APC magnetic microbeads (Miltenyi Biotec)
Foxp3/Transcription Factor Staining Buffer Set (eBioscience)

Tetramers: mouse CD1d-PBS-57-APC (NIH tetramer core facility, Atlanta, USA)

Unloaded mouse CD1d-APC (NIH tetramer core facility, Atlanta, USA)

Antibodies: anti-CD16/32 (clone 2.4G2)
Anti-CD19 (clone 6D5)
Anti-B220 (clone RA3-6B2)
Anti-CD11b (clone M1/70)
Anti-CD11c (clone N418)
Anti-TCRβ (clone H57-597)
Anti-CD4 (clone GK1.5)
Anti-NK1.1 (clone PK136)
Anti-CD44 (clone IM7)
Anti-CD24 (clone M1/69)
Anti-PLZF (clone Mags.21F7)
Anti-T-bet (clone O4-46)
Anti-RORγt (clone Q31-378)
Anti-CXCR3 (CD183, clone CXCR3-173)
Anti-CD122 (clone TM-b1)

#### 18.5 Data analysis

**Figure 63.**
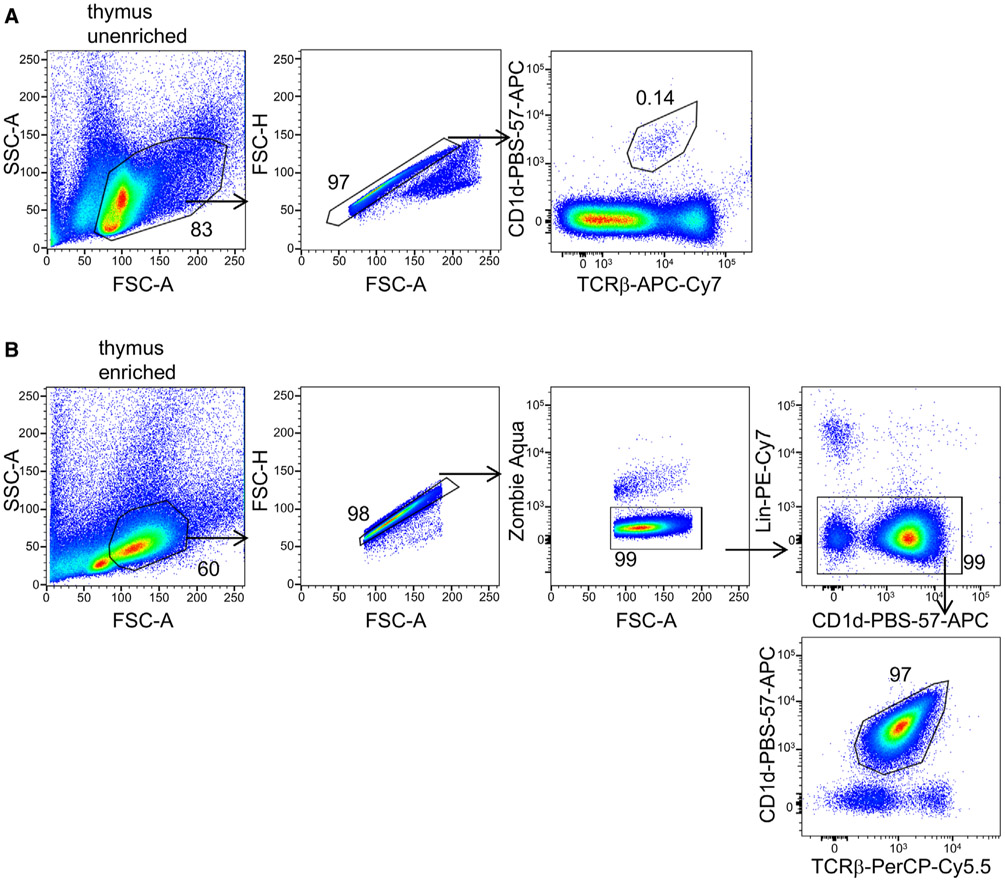
Basic gating strategy for murine thymic iNKT cells. (A) Basic gating strategy for non-enriched murine thymic iNKT cells. (B) Basic gating strategy for thymic iNKT cells following magnetic-bead enrichment. Sample was additionally stained with Zombie Aqua viability dye and Abs against lineage markers. Numbers adjacent to gates indicate frequency of parent population.

**Figure 64.**
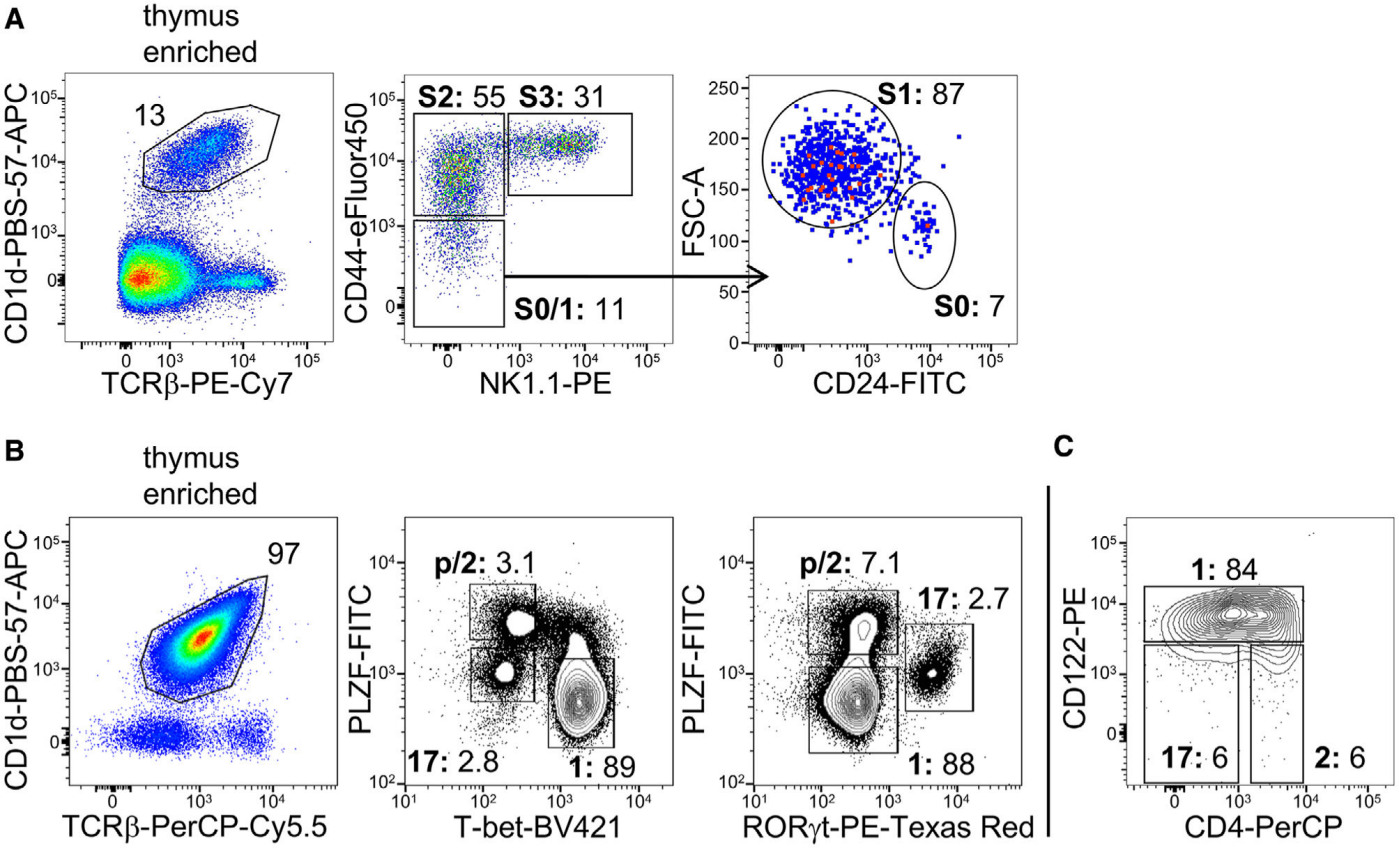
Murine thymic iNKT cell populations. (A) Magnetic-bead enriched iNKT cells from C57BL/6 mice were additionally stained with Abs against CD44, NK1.1, and CD24. The upstream gating strategy is shown in NKT Figure 63. (B) Magnetic-bead enriched iNKT cells from C57BL/6 mice were additionally stained intracellulary with Abs against PLZF, T-bet and RORγt. The upstream gating strategy is shown in NKT Figure 63. (C) Magnetic-bead enriched iNKT cells from C57BL/6 mice were additionally stained with Abs against CD122 and CD4. Numbers adjacent to gates indicate frequency of parent population. The upstream gating strategy is shown in NKT Figure 63. Boldface S0, S1, S0/1, S2, S3 adjacent to gates indicate developmental stages. Boldface p, 1, 2, and 17 adjacent to gates indicate NKTp, NKT1, NKT2, and NKT17 subsets, respectively.

**Figure 65.**
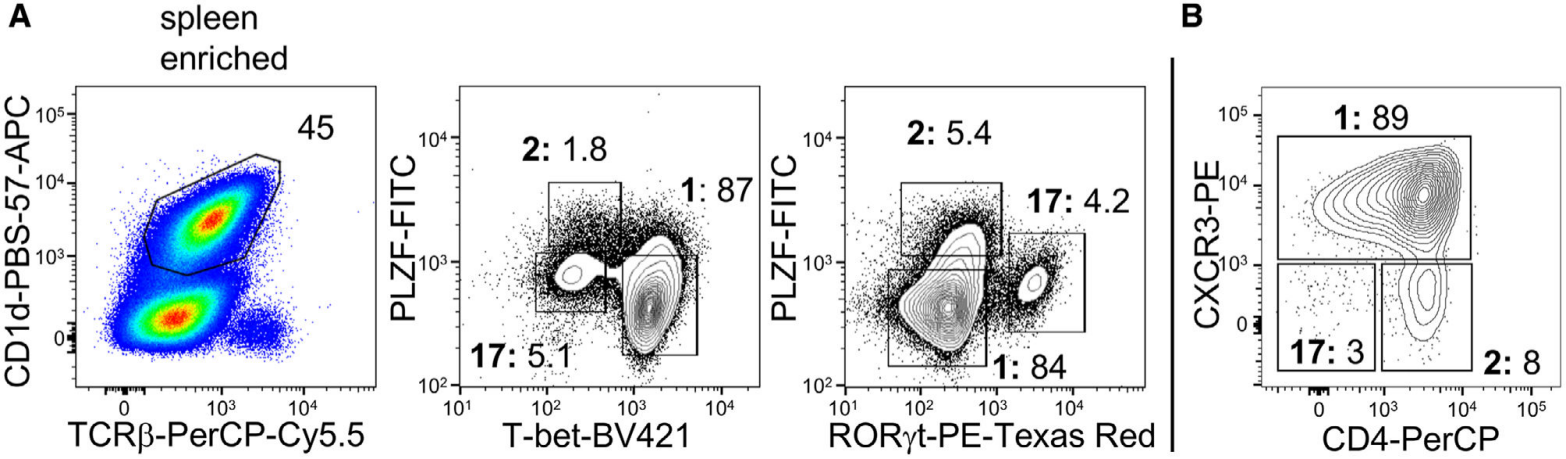
Murine peripheral iNKT cell populations. (A) Magnetic-bead enriched iNKT cells from C57BL/6 mice were additionally stained intracellulary with Abs against PLZF, T-bet, and RORγt. The upstream gating strategy is analogous to that shown NKT Figure 63. (B) Magnetic-bead enriched iNKT cells from C57BL/6 mice were additionally stained with Abs against CXCR3 and CD4. Numbers adjacent to gates indicate frequency of parent population. The upstream gating strategy is analogous to that shown NKT Figure 63. Boldface 1, 2, and 17 adjacent to gates indicate NKT1, NKT2, and NKT17 subsets, respectively.

Data analysis typically follows a sequence of electronically gating on lymphocytes (FSC/SSC), doublet discrimination (FSC-A/FSC-H), and life/dead discrimination followed by exclusion of cells of other lineages (Figure 63B). NKT cells are then identified as CD1d-PBS-57 tetramer/TCRβ double-positive cells (Figure 63). The resulting population forms the basis for further phenotypic analysis (Figures 64 and 65).

#### 18.6 Pitfalls

Simultaneous staining of cells with tetramer and anti-TCRβ is possible. However, due to distinct staining conditions, it may result in different staining intensities. Anti-CD24 Ab staining is sensitive to EDTA. Distribution of iNKT cell subsets varies between organs and also between mouse strains. For instance, in liver, iNKT1 cells constitute the predominant iNKT cell subset, whereas mesenteric lymph nodes predominantly contain iNKT2 cells [632]. Furthermore, BALB/c mice display a strong bias towards iNKT2 cells when compared to C57BL/6 mice [619].

#### 18.7 Top tricks

iNKT cells are a rare population of T cells. Therefore, for some downstream analyses, it is advisable to perform enrichment using magnetic beads (see section Rare Cell Analysis). We and others have found that differences in frequencies of iNKT cells in mouse strains with iNKT cell deficiency, such as miR-181a/b-1-deficient mice, compared to wild-type mice are essentially retained upon enrichment via tetramers [633]. The underlying reason remains elusive but may be attributed to lower affinity of tetramers when compared to Ab–Ag interaction. We and others have employed Rag-GFP reporter mice to delineate developmental progression of iNKT cells in the thymus. Such a mouse model may help to further resolve NKT cell precursors and mature NKT cell populations in the thymus [617, 634].

#### 18.8 Summary of the phenotype

This is detailed in Table 61

#### 18.9 Key information human versus murine

In humans, iNKT cells are scarce when compared to MAIT cells, whereas the opposite is the case in mice [635].

Murine iNKT cell subsets are clearly defined as iNKT1, 2, and 17 based on their cytokine expression profile and lineage defining transcription factors (note that iNKT1 and NKT17 cells also express type 2 cytokines) [619]. In humans, iNKT cell subsets are defined based on CD4 and CD8 co-receptor expression [580, 587].

Expression of IL-17 and type I cytokines is not mutually exclusive in human NKT cells and is more prevalent in chronic inflammatory disease [636]. Note that cross-species similarities in functionally distinct NKT cell subsets may be masked by a relative lack of studies in humans compared to mice.

### 19 Unconventional human T cells:

Mucosal-Associated Invariant T (MAIT) cells

#### 19.1 Overview

Mucosal-associated invariant T (MAIT) cells are a population of unconventional T cells with potent anti-microbial function. In humans, these cells are highly abundant and have been implicated in wide-ranging disease settings including infectious disease, autoimmunity, allergy, and cancer [511]. Accordingly, the high abundance and unique biology of MAIT cells have garnered the attention of researchers and clinicians alike, and there is great interest in studying the biology of these cells and understanding how they may contribute to disease clearance or pathology or be manipulated for novel immunotherapies. Key to studying MAIT cells is the efficient use of tools for isolating them from biological samples. This was a major challenge for the field for many years; however, the advent of MR1-Ag tetramers to detect and isolate MAIT cells has facilitated a rapid progression in our understanding of these cells. In this section, we provide recommended guidelines for flow-cytometry-based identification strategies for human peripheral blood MAIT cells, with particular emphasis on comparing tetramer and Ab-based identification techniques, and analysis of MAIT cell phenotypic diversity.

#### 19.2 Introduction

In contrast to conventional CD8^+^ and CD4^+^ αβ T cells that express diverse T cell receptors to recognize polymorphic major histocompatibility complex (MHC) class I and II molecules respectively [637], MAIT cells are defined by expression of a semiinvariant T cell receptor (TCR) that recognizes microbial-derived vitamin-B2 (riboflavin) derivatives, presented by the monomorphic MHC-related protein 1 (MR1) [638-640] (See also section Murine MAIT cells). This unique Ag-restriction drives a divergent thymic developmental pathway relative to conventional αβ T cells, resulting in a unique, transcriptional landscape characterized by expression of the innate transcription factor promyelocytic leukemia zinc finger (PLZF) [604, 641, 642] that drives an innate-like, antimicrobial functional capacity. In humans, mature MAIT cells comprise ~3% of total T cells within adult peripheral blood, although this can range from 0.1 to 10% depending on the individual [643, 644]. MAIT cells are also highly enriched in liver where they can compromise up to 40% of T cells [645, 646], and are abundant in certain mucosal sites, such as the gut [647, 648]. Moreover, upon activation, MAIT cells rapidly produce large quantities of proinflammatory cytokines and chemokines [646, 649] and lyse infected cells [650]. Accordingly, MAIT cells are emerging as key players in antimicrobial immunity [651, 652]. More recently, MAIT cells have been shown to respond to inflammatory queues independent of TCR-mediated signaling [653], providing a mechanism for MAIT cells to play a role during the many viral [654-656] and non-microbial diseases in which MAIT cells have been implicated, including autoimmune disease [657] and cancer [554]. In humans, there is evidence of distinct functionality in peripheral tissues [658]. MAIT cells are largely CD8^+^, expressing either CD8αα homodimers or CD8αβ heterodimers, or are CD4^−^CD8^−^ double negative (DN) [646, 648, 659]. Rare populations of CD4^+^ and CD4^+^CD8^+^ DP MAIT cells also exist [643, 660]. Whether these populations represent functionally distinct subsets remains unclear, although some reports suggest the CD8^+^ population may have enhanced cytotoxic capacity [661], while CD8αα^+^ cells only emerge post-thymic development of mature MAIT cells [641]. Likewise, CD4^+^ MAIT cells may have distinct tissue localization [662] and cytokine profiles [643]. Further studies on this axis are needed, but nonetheless, inclusion of CD4 and CD8 mAbs in flow cytometry experiments analyzing MAIT cells may prove informative. Indeed, several studies have noted modulation of these markers during progression of diverse diseases [663].

Central to MAIT cell biology is their expression of a “semi-invariant” αβTCR that binds MR1-Ag complexes. The MAIT TCR-α chain is composed of the TRAV1-2 gene segment, which is joined with TRAJ33, or less commonly TRAJ12 or TRAJ20. These TRAV1-2^+^ TCR α-chains display heavily biased pairing with TCR-β gene segments including TRBV6 family members and TRBV20-1 [664]. The development of a mAb against the TRAV1-2 TCR-α chain segment of the MAIT TCR provided the first means to isolate these cells from human samples [665]. This was then further refined to include surface-markers highly expressed by MAIT cells, such as the C-type-lectin CD161, the IL-18Rα CD218, and the ectopeptidase CD26. Co-staining of samples with anti-TRAV1-2 and either anti-CD161, CD218, or CD26 mAbs were the gold standard to identify MAIT cells for many years. MAIT cells were thus identified as TRAV1-2^+^ and either CD161^HI^[665], IL-18Rα^HI^[644] or CD26^HI^[666]. To date, 4 clones of anti-TRAV1-2 mAbs have been produced (3C10[665], D5[638], OF5A12[667], and REA179 (Miltenyi); however, the original clone, 3C10, produced by Lantz and colleagues [665] is by far the most widely used. A major drawback to the use of this surrogate identification technique however is that is has been unclear as to whether all MAIT cells express high levels of the surrogate markers, and likewise, whether all TRAV1-2^+^ cells that express high levels of the surrogate markers are MAIT cells, particularly in tissues. Indeed, clinical studies analyzing MAIT cells in HIV [668] and rheumatoid arthritis [669] have suggested that MAIT cells may downregulate CD161 during disease progression; however, there have also been contrasting findings where MAIT cells in HIV patients were not likely to be present in the TRAV1-2^+^ CD161^−^ population [670, 671]. Altogether, these raise concerns about the use of surrogate markers to identify MAIT cells in tissues and in disease settings.

The discovery that the MAIT TCR specifically recognizes the Ag (Ag) 5-(2-oxopropylideneamino)-6-D-ribitylaminouracil (5-OP-RU), derived from an intermediate in the microbial riboflavin biosynthesis pathway, facilitated the development of tetramerized soluble MR1 molecules, loaded with 5-OP-RU (MR1-5-OP-RU tetramers) [640, 648]. These fluorescently tagged tetramers bind all cells expressing TCRs that confer reactivity to MR1-5-OP-RU and provide a highly specific method for the detection and isolation of MAIT cells from human blood and other tissues. As a control, MR1 tetramers loaded with non-stimulatory Ag 6-FP (MR1-6-FP) [648] or synthetic analog acetyl (Ac)-6-FP[672] (MR1-Ac-6-FP) are used to validate the specificity of MR1-5-OP-RU tetramers, similar to a conventional isotype control.

A recent direct comparison of MR1 tetramers and surrogate mAb-based identification techniques revealed that while the surrogate markers were generally highly enriched for CD8^+^ and CD4^−^CD8^−^ DN MAIT cells, they were poor at identifying CD4^+^ MAIT cells, as many TRAV1-2^+^ CD4^+^ T cells expressing high levels of CD161, IL-18Rα, or CD26 were not labeled with MR1-5-OP-RU tetramer [643, 660, 673]. Similarly, the existence of many CD161^++^ TRAV1-2^+^ T cells in thymus and neonatal blood samples that do not stain with MR1 tetramer (and are not MAIT cells) [641, 643, 674] also limits the use of these surrogate marker combinations in developmental studies. Moreover, across a large cohort of healthy blood donors, there were outliers in which a substantial proportion of MAIT cells lacked expression of surrogate markers, and immature MAIT cells in thymus are defined by a lack of CD161 expression [641]. More recently, MR1 tetramers have been used to identify a population of non-classical MAIT cells that are phenotypically equivalent to TRAV1-2^+^ MAIT cells, but utilize a TRAV36^+^ TCR and thus do not stain with anti-TRAV1-2 mAbs[675]. Accordingly, where possible, MR1 tetramers should be used for the highly specific isolation of MAIT cells, particularly when studying CD4^+^ MAIT cells, or when analyzing samples from patient cohorts where the modulation of MAIT cell surface marker expression is unknown, or in developmental studies where thymus or neonatal blood is being assessed. MR1 tetramers can also identify a diverse population of MR1-reactive T cells, many of which are quite distinct from MAIT cells, as they use different and diverse TCRs ([675, 676], reviewed in [677]). Apart from distinct TCRs, some of these cells do not express canonical markers associated with MAIT cells such as the master transcription factor PLZF, and hence are not MAIT cells. Thus, combining MR1 tetramers with TRAV1-2 can help to determine which type of MR1-restricted or MR1-reactive T cells are being assessed. MR1 tetramers are available from the NIH tetramer core-facility upon request conjugated to a variety of fluorochromes or in the form of biotinylated monomers [678].

#### 19.3 Step-by-step sample preparation: Isolation using MR1-tetramers

1. If using biotinylated MR1 monomers, tetramerize with Streptavidin conjugated to fluorochrome of choice at a 4:1 to 8:1 ratio of monomers to streptavidin, adding Streptavidin sequentially (1/4 of the required volume) at a series of 10-minute incubations at 4°C. This sequential addition facilitates the formation of tetramers and prevent a final mixture of lower degree monomers/dimers/trimers [679]. A titration of monomer to streptavidin ratio assessed by optimal staining is recommended as monomer and streptavidin conjugate stock concentration can differ between batches or companies.
2. The simplest staining protocol when using fluorochrome-conjugated-MR1-tetramers (herewith referred to as MR1-tetramers) is to include them within an appropriate Ab cocktail, as the interaction between MR1-5-OP-RU tetramers and MAIT cells is of sufficient avidity to achieve clear staining during a standard 30 min incubation at room temperature or 4°C.

#### 19A.3 Materials: Isolation using MR1-tetramers

Ligand loaded MR1-monomers or tetramers, available from the NIH tetramer core facility [678].

FACS buffer

1× Phosphate-buffered saline (PBS)
2% Fetal calf serum (FCS)

Ficoll-Paque density 1.077 g/ml (Sigma, #GE17-1440-02)

Dako Biotin blocking system (Agilent Dako, #X059030)

Dasatinib (Sigma-Aldrich, #CDS023389)

Magnetic-activated cell sorting (MACS) buffer
1× Phosphate Buffered Saline (PBS)
0.5% FCS and 2 mM EDTA.

Anti-PE MACS^®^ MicroBeads for magnetic labeling of cells (Miltenyi Biotec, Order no: 130-048-801)

MACS^®^ LS Columns (Miltenyi Biotec, Order no: 130-042-401)

MACS^®^ Separator with LS column adaptor (Miltenyi Biotec, Order no: 130-091-051)

Flow Cytometer: example: BD LSR Fortessa equipped with yellow-green laser or similar.

Analysis: Flowjo Version 10 (macOS)

Antibodies, see Table 62.

#### 19A.4 Data analysis: Isolation using MR1-tetramers

**Figure 66.**
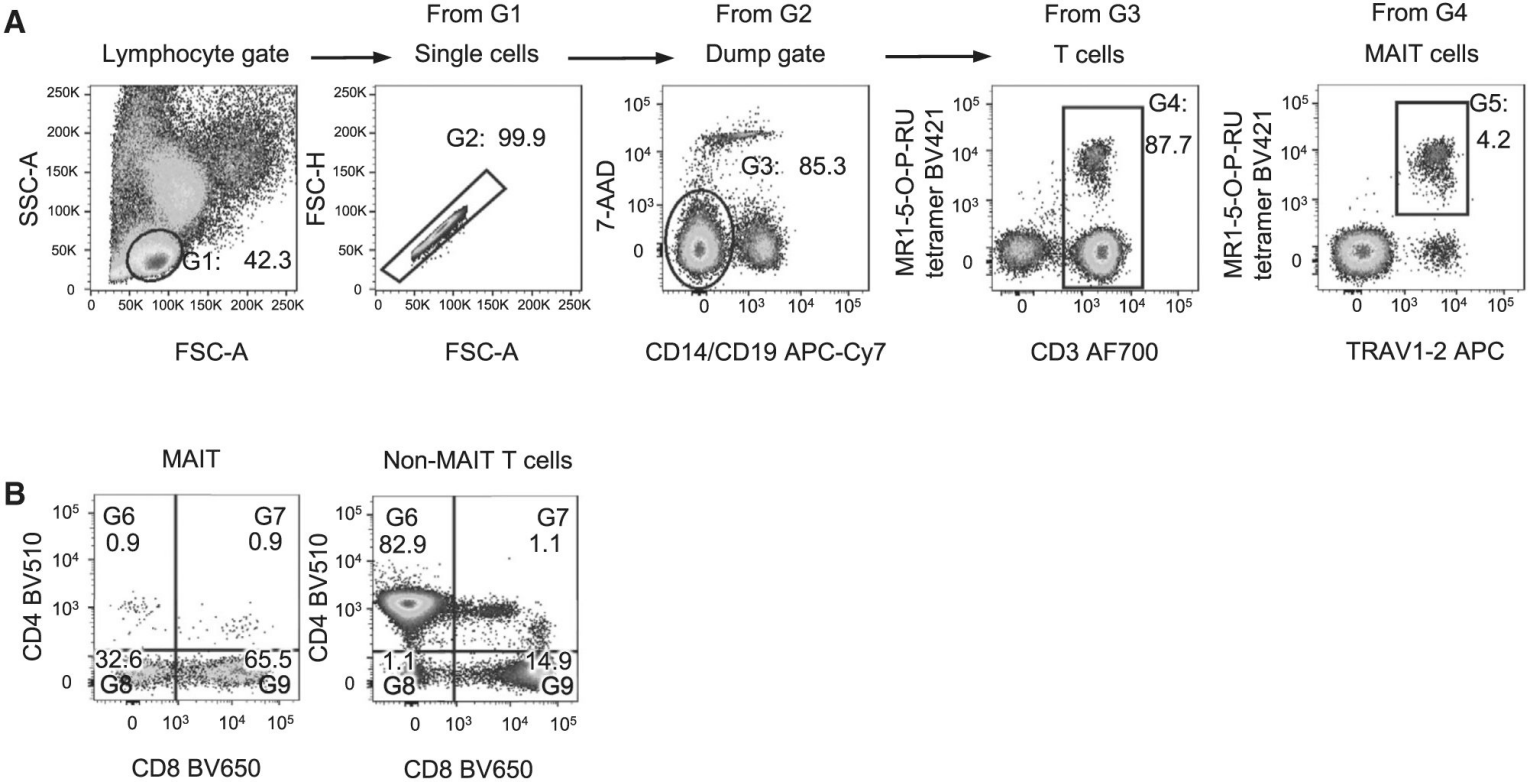
Flow cytometry detection of human peripheral blood MAIT cells. (A) Gating strategy. Lymphocytes are distinguished from PBMC preparations based on their FSC-A and SSC-A. Single cells are identified by their linear relationship between FSC-H versus FSC-A, enabling doublets to be excluded. To remove any non-specific or TCR-independent MR1-5-OP-RU tetramer staining, dead cells are excluded with the use of a viability dye (7-AAD), and monocytes and B cells are excluded based on the expression of CD14 and CD19 respectively. MAIT cell frequencies can be presented as a percentage of CD19^−^ lymphocytes, or as a percentage of T cells. (B) MAIT cells can be divided into subsets based on expression of CD4 and CD8 co-receptors and, relative to non-MAIT T cells, are typically enriched for CD8^+^ and CD4^−^CD8^−^ double negative (DN) subsets, with only minor populations of CD4^+^ or CD4^+^CD8^+^ double-positive (DP) cells.

**Figure 67.**
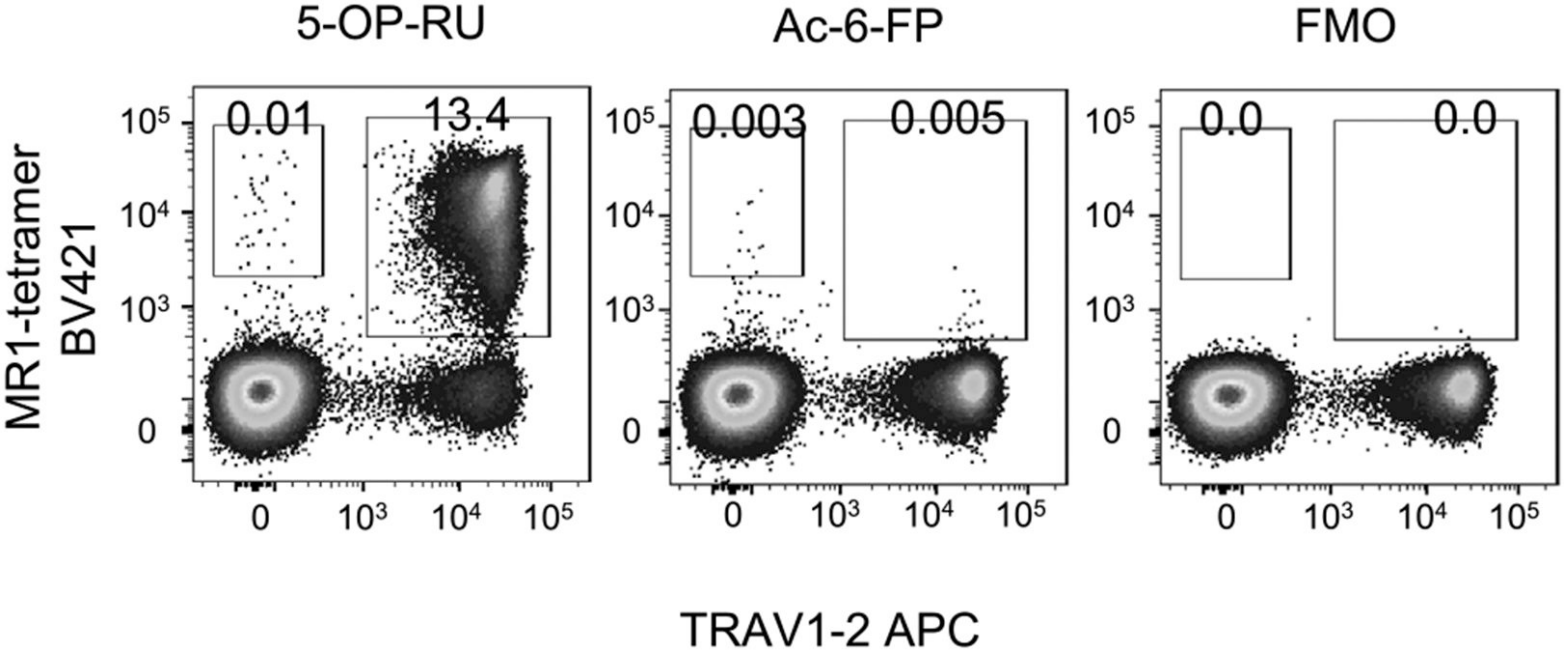
MR1-tetramer staining controls. Representative plots depict MR1-5-OP-RU tetramer staining among CD19^−^ lymphocytes from human PBMCs in comparison to a MR1-Ac-6-FP tetramer control and a fluorescence minus one (FMO) control. Refer to Figure 66A for gating strategy.

The following descriptions and figures specifically relate to the identification of MAIT cells from human peripheral blood mononuclear cells (PBMCs), however, these general principles can also be applied to the detection of MAIT cells in single-cell suspensions prepared from other human tissue samples. As these cells can be relatively rare, it is important to carefully apply gates to focus on viable lymphoid cells. A typical gating strategy for detecting human blood MAIT cells by flow cytometry is depicted in (Figure 66A). When identifying any T cell population using tetramer staining, a negative control tetramer is recommended, particularly when the target population is rare, of low-intensity staining, or there is possible non-TCR-specific background staining. Thus, MR1-5-OP-RU tetramers that bind MAIT cells are used in parallel with MR1-6-FP or MR1-Ac-6-FP that do not stain the majority of MAIT cells[648, 676]. As such, MR1-6-FP or MR1-Ac-6-FP tetramers provide a valuable negative control for the vast majority of donors, as shown in a representative example of MAIT cell staining of human blood (Figure 67).

The most commonly used surrogate identification method prior to the advent of MR1-tetramers was co-expression of the TRAV1-2 TCR-α chain and high levels of CD161 (CD161^++^ or CD161^HI^), often including a gate on CD8α^+^ T cells. By comparing these markers to MR1-5-OP-RU tetramer stained cells, it has been shown that these surrogate markers are generally quite effective for detecting human CD8^+^ MAIT cells in the absence of MR1-tetramer [643, 673, 680]; however, this efficiency can vary somewhat between individuals and is less stringent when studying CD8^−^ and CD4^+^ MAIT cells [643] (Figure 66B and Table 63).

#### 19A.5 Pitfalls: Isolation using MR1-tetramers

**Figure 68.**
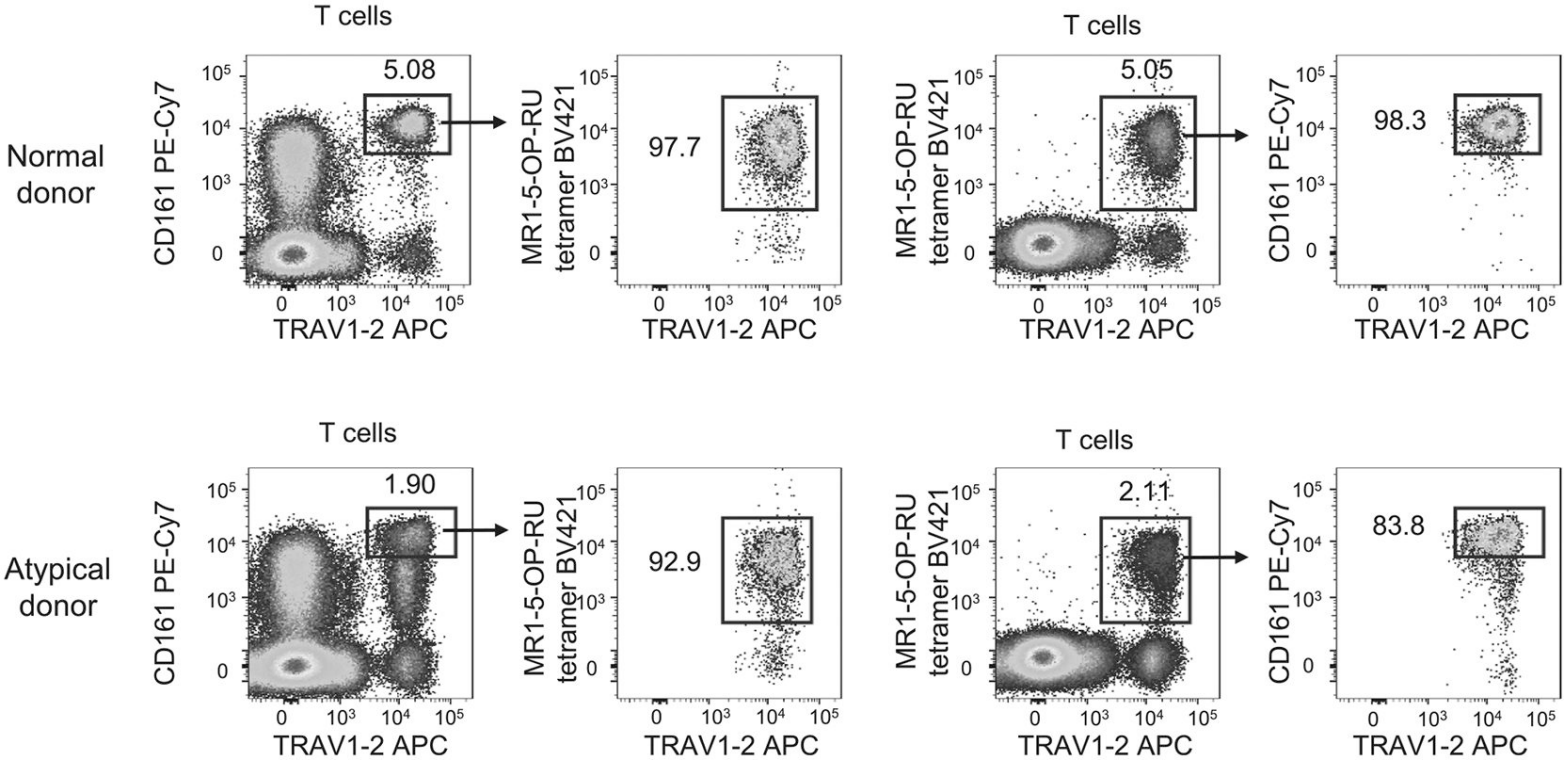
Identifying human peripheral blood MAIT cells using surrogate markers. Gating strategy utilized identical to Figure 66A. Plots depict the identification of human MAIT cells among CD19^−^, CD3^+^ lymphocytes via their expression of TRAV1-2 and CD161 and how this relates to MR1-5-OP-RU tetramer staining from a normal donor (top) and an abnormal donor (bottom).

- It should be noted that in most individuals, minor populations of TRAV1-2^+^ MAIT cells can be isolated that display reactivity to both 5-OP-RU and 6-FP. Further, populations of cells that lack the classical MAIT cell TRAV1-2^+^ TCR α-chain have been identified that preferentially bind MR1-6-FP in comparison to MR1-5-OP-RU [676]. Therefore, while these atypical MAIT cell subsets may be rare in comparison to classical MAIT cells, caution needs to be taken when using MR1-6-FP tetramers as a negative control, as positive staining cannot automatically be ascribed as non-specific or background.
- Surrogate markers are slightly less reliable for CD4^−^CD8^−^ MAIT cells, and they do not work well for CD4^+^ MAIT cells [643, 673, 680]. Therefore, when using surrogate markers to study these populations, it is important to consider that not all TRAV1-2^+^, CD161^++^ cells will necessarily be MAIT cells and not all MAIT cells will necessarily be TRAV1-2^+^, CD161^++^, and that CD4^+^ MAIT cells cannot be reliably detected using this approach. The inclusion of other cell-surface receptors typically expressed at high levels by MAIT cells such as the IL-18 receptor alpha chain CD218a (IL-18αR), the ectopeptidase CD26, and the chemokine receptor CD195 (CCR5) may be useful to improve the stringency of their identification[644, 646, 666, 680]. Of note, these markers are validated to be associated with MAIT cells in the blood, but not as extensively in tissues, where some of them can be expressed by conventional T cells both at a steady state or during disease.
- There are potential problems in using these surrogate markers to detect MAIT cells in settings other than healthy adult human blood. For example, there is growing evidence that the normal CD161^++^ phenotype of MAIT cells may be perturbed among donors (Figure 68), under certain disease settings [668, 669], and the proportion of CD161^++^ cells that are MAIT cells as defined by MR1 tetramer also alters with age [641, 659, 674, 681]. Furthermore, it is also worth considering that a reliance on TRAV1-2 expression to detect MAIT cells will fail to detect those with atypical TCR α-chain usages [641, 664, 682, 683].
- It should be noted particularly for functional studies that the use of either TRAV1-2 mAb or MR1-tetramer poses the possibility of positive selection or activation of MAIT cells. Furthermore, Ag (such as 5-OP-RU) originally on the tetramers may be recycled for presentation causing subsequent cell-mediated MAIT cell activation. In cases like this, it is worth considering a mAb cocktail that enriches for MAIT cells but does not include tetramers for labeling or isolating. Alternatively, cells isolated by tetramer can be rested 37°C overnight prior to performing downstream functional assays.

#### 19A.6 Top Tricks: Isolation using MR1-tetramers

- Like standard mAbs, MR1-tetramers should be titrated prior to use in formal experiments to ensure optimal signal-to-noise separation of staining. MR1-tetramers provided from the NIH facility are used within a staining concentration range of 1:500 to 1:1000[678] depending on the fluorochrome conjugated. MR1 tetramer staining conditions (time and temperature) should also be initially tested to ensure the best signal-to-noise results. MR1 tetramers work at 4°C, RT, and 37°C, with staining intensity proportional to temperature.
- In order to exclude any TCR-independent MR1-5-OP-RU tetramer binding and maximize the potential scope of MAIT cell phenotyping that can be achieved within a single mAb cocktail, the detection of B cells, monocytes, and dead cells can be restricted to one fluorescence parameter or ‘dump channel’ akin to a lineage marker dump. For example, a combination that can be used to achieve this is: anti-CD14 APC-Cy7, anti-CD19 APC-Cy7, and Live/Dead fixable Near-IR (ThermoFisher) (Figure 66A). Gating on CD3/TCR^+^ cells can also be helpful to exclude TCR-independent MR1 tetramer binding (Figure 66A).
- The protein-kinase inhibitor dasatinib can greatly enhance the detection of lower affinity TCR interactions that may otherwise go undetected via tetramer staining [684]. Whilst unnecessary for the identification of MR1-5-OP-RU tetramer-reactive, TRAV1-2^+^ MAIT cells, pretreating cells with dasatinib (working concentration 50nM) may prove advantageous for detecting other populations of MR1-reactive T cells with lower affinity for the MR1 ligands being assessed [676].
- If staining includes more than one tetramer (such as MR1-5-OP-RU tetramer on one color with MR1-6-FP tetramer on another color), it is highly recommended that tetramer incubations are sequentially applied, with an intervening avidin and biotin blocking step [685], such as with the Dako Biotin blocking system (see section (A) Materials: Isolation and staining of MAIT cells from single-cell suspensions using MR1-tetramers). This will prevent any potential excess streptavidin-conjugated fluorochrome from one tetramer binding available biotin sites that may be present on the other tetramer, which may falsely lead to double-positive tetramer staining.

#### 19B.3 Step-by-step sample preparation: MAIT cell enrichment

1. Resuspend cells in 10^7^ cells per ml of FACS buffer, stain with PE-conjugated MR1-tetramer OR PE-conjugated anti-TRAV1-2.
2. Wash cells twice with FACS buffer after staining and resuspend cells in 80 μl of MACS buffer/10^7^ total cells.
3. Mix in 20 μl of Anti-PE MACS^®^ MicroBeads/10^7^ total cells and incubate for 30 min at 4°C.
4. Wash cells twice with MACS buffer and resuspend up to 10^8^ cells in 5 ml MACS buffer.
5. Prepare LS column on LS separator by rinsing with 5ml MACS buffer, and discard flow-through.
6. Prepare a flow-through collection tube under the column.
7. Apply 5ml cell suspension onto the column reservoir.
8. After the column reservoir is empty, wash the column with 3 ml of MACS buffer as the unlabeled cells pass into the flow-through.
9. Wash the column with 3 ml of MACS buffer three times.
10. Remove LS column and place away from magnet separator, on top of a new 10 ml collection tube.
11. Elute magnetically labeled cells by adding 5ml MACS buffer in column reservoir, and firmly pushing the LS plunger into the LS column.
12. Centrifuge to pellet the cells and to use for flow cytometry without further staining with PE-conjugated mAbs or tetramers.

#### 19B.4 Materials: MAIT cell enrichment

See A.Materials: Isolation and staining of MAIT cells from single-cell suspensions using MR1-tetramers

#### 19B.5 Data analysis: MAIT cell enrichment

**Figure 69.**
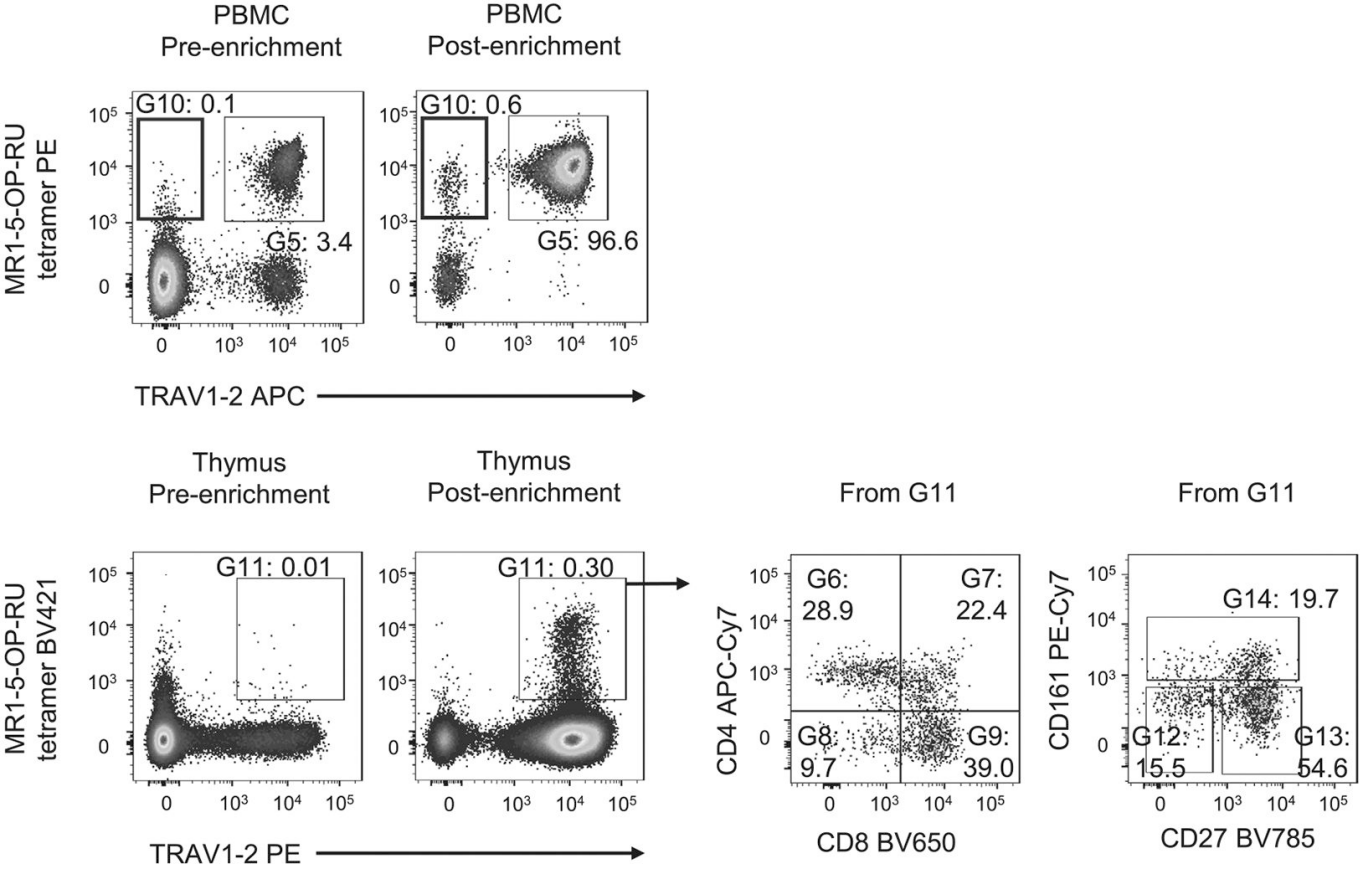
Human MAIT cell enrichment. Gating strategy utilized identical to Figure 66A. Top panel depict plots with the percentages of MAIT cells among CD19^−^, CD3^+^ lymphocytes from PBMCs either prior to (first panel) or following MR1-5-OP-RU tetramer enrichment (second panel). Bottom panel depict plots with the percentages of MAIT cells among among CD19^−^, CD3^+^ thymocytes either prior to (first panel) or following TRAV1-2 Ab enrichment (second panel). Further phenotypic analysis of MAIT cells reveal heterogeneous subpopulations based on CD4, CD8, CD27 and CD161 (third and fourth panel).

MAIT cell numbers vary widely among individuals, and the factors influencing that remain poorly understood. Therefore, whilst it is possible to analyze or sort-purify MAIT cells via flow cytometry directly from PBMC preparations, this is not always sufficient to get enough cells for downstream experiments. Thus, a useful approach is to first enrich for either MR1-5-OP-RU tetramer^+^ or TRAV1-2^+^ cells using magnetic-activated cell sorting (MACS^®^). Figure 69 depicts the enrichment of MAIT cells following PE-microbead enrichment of PBMCs that have been labeled with PE-conjugated MR1-5-OP-RU tetramer. This technique may also prove useful for investigating a minor population of MAIT cells, such as the TRAV1-2^−^ cells that can become evident following the enrichment of MR1-tetramer^+^ cells (Figure 69). Furthermore, MAIT cell numbers can be extremely rare in organs such as the thymus, but become clearly detectable following enrichment based on TRAV1-2 expression (Figure 69 and Table 63).

#### 19B.6 Pitfalls: MAIT cell enrichment

The choice of either TRAV1-2 or MR1-tetramer to enrich MAIT cells will depend on the particular aims of the experiment. While both approaches are highly effective, the enrichment of TRAV1-2^+^ cells will not enrich MAIT cells with atypical TCR usages [676]. This technique may also prove advantageous when aiming to isolate MAIT cells from tissues where they are consistently present at low frequencies, such as the thymus (Figure 69) [641].

#### 19B.7 Top tricks: MAIT cell enrichment

This protocol describes the use of PE-conjugated MR1-5-OP-RU tetramer or PE-conjugated anti-TRAV1-2 enrichment using anti-PE microbeads, but can be adapted to use with the other fluorochrome options available via MACS technology or from other manufacturers offering similar magnetic strategies. It is highly recommended that researchers familiarise themselves with detailed tools, resources, and manufacturers’ datasheets to determine the most suitable enrichment strategy.

#### 19.4 Clinical relevance statement

Apart from their ability to detect and clear microbial infection by riboflavin-synthesizing bacteria and yeast [677, 686], MAIT cells also play critical roles in non-bacterial disease settings [677, 687] including viral immunity [654], tumor immunity [688, 689], autoimmunity [657], vaccine efficacy [690], and wound healing [691]. Indeed, MAIT cell deficiencies have been associated with impaired or aberrant immunity in many of these settings [677, 687, 692, 693]. Strikingly, while MAIT cell frequencies are relatively high, reaching up to 10% of T cells in peripheral blood, they vary widely between individuals [511, 646, 659, 694], and decline with age [659, 681, 695], which may contribute to impaired immunity associated with aging. Thus, the nature of the public MAIT cell TCR recognizing the same target Ags presented by monomorphic human MR1 imbues this immune axis with great potential as a target for Ag and cell-based immunotherapy, potentially including chimeric Ag receptor (CAR) armed MAIT cells [554]. With the dramatic rise in MAIT cell research in various disease settings, it seems likely that ultimately MAIT cell-based therapies will provide new ways to treat infectious and non-infectious diseases.

#### 19.5 Summary of the phenotypes

This is detailed in Tables 63 and 64 (with ref. [696]).

### 20 Unconventional murine T cells: MAIT cells

#### 20.1 Overview

Murine mucosal-associated invariant T cells (MAIT) share many features with iNKT cells. They express a semi-invariant TCR comprised of an invariant Vα19Jα33 TCRα chain, preferentially paired with Vβ6 and Vβ8. MAIT cells recognize vitamin B metabolites, such as 5-(2-oxopropylideneamino)-6-D-ribityl-aminouracil (5-OP-RU), in the context of the non-classical MHC molecule MHC class I-related protein 1 (MR1) [647]. Despite their virtually simultaneous discovery with NKT cells, understanding of MAIT cell biology is substantially more limited because they are exceedingly rare in mice [697, 698]. This section describes the characterization of MAIT cell subsets based on MR1-tetramers 648, 694], surface markers, and key transcription factors. In addition, magnetic-bead-based enrichment of MAIT cells is described.

#### 20.2 Introduction

The study of MAIT cells in mice is of profound interest, mostly because MAIT cells constitute a very abundant population in various human tissues, comprising almost 10% of all blood T cells and 20-40% of all liver T cells (see section Human MAIT). In contrast, in C57BL/6 mice, thymus contains only around 5,000 MAIT cells, corresponding to 0.002% of all thymocytes. Comparably low frequencies are also found in peripheral lymphoid organs.

Intrathymic development of MAIT cells shares some similarities with that of NKT cells: MAIT cells are selected on cortical CD4^+^CD8^+^ double-positive thymocytes. They progress through phenotypically distinct precursor stages (stage 1 -3) characterized by differential expression of CD24 and CD44 [641]. Development of MAIT cells depends on the transcription factor PLZF and miRNA, in particular miR-181a/b-1, as well as signaling through SLAM-associated protein (SAP), ZAP-70, and chemokine receptors, most notably CXCR6 [633, 634, 641, 699, 700]. These similarities are further underscored by characterization of T-bet^+^RORγt^lo^ MAIT1 and T-bet^−^RORγt^hi^ MAIT17 cell transcriptomes, which within matching tissues are virtually identical to those of NKT1 and NKT17 cells, respectively [625]. Whereas in naive mice, MAIT1 and MAIT17 phenotypes appear to be mutually exclusive, it has been recently demonstrated that upon bacterial infection MAIT cells with a mixed T-bet^+^ RORγt^+^ phenotype can emerge [701]. MAIT cells also display a large degree of tissue residency in non-lymphoid organs [625]. In addition to these similarities between MAIT cells and iNKT cells, there are a number of critical differences. MAIT cell development is characterized by a later onset of PLZF expression at developmental stage 3 only, whereas at least some NKTp already express high levels of PLZF [617, 641]. Although a subset of MAIT cells corresponding to NKT2 cells has recently been described in BALB/c mice based on transcriptional signatures, these MAIT2 cells are virtually absent from C57BL/6 mice and are characterized by low-level expression of type 2 cytokines, such as IL-4 [702]. Furthermore, the ratio between MAIT1 and MAIT17 cells is geared towards the latter, whereas NKT1 cells are more abundant than NKT17 cells. It remains an open question whether MAIT cells undergo agonist selection in a similar manner as NKT cells.

Analysis of in vivo function of MAIT cells in immunity is compromised by their scarcity in mice. In addition, many Vα19Jα33 TCRα^+^ T cells in Vα19Jα33 TCR transgenic mice lack expression of PLZF, indicating that they do not represent true MAIT cells [648]. These obstacles may be overcome by employing B6-MAIT^CAST^ congenic mice that contain high frequencies of MAIT cells due to increased usage of Vα19 in TCR gene rearrangements [703]. This mouse model revealed that MAIT cells alleviated urinary tract infections. MR1-deficient mice are more susceptible to a broad range of bacterial infections (for review see [704]). Given that MAIT cells have also been implicated in clearance of viral infections suggests that Ag-independent stimulation via cytokines, such as IL-12 and IL-18, is also possible, in keeping with their innate-like nature and overall similarity to iNKT cells.

#### 20.3 Step-by-step sample preparation

##### Cell isolation:

Single-cell suspensions of whole lymphoid organs (thymus, spleen, lymph nodes) are generated by crushing organs through a 70μm filter. Red blood cells (RBCs) are lysed (spleen only) using Qiagen RBC Lysis Solution according to manufacturer’s instructions. For lymphocyte isolation from the lung and liver, mice are euthanized and liver/lungs are immediately perfused with phosphate-buffered saline (PBS). Lymphocytes are then isolated using standard procedures for solid organs or using commercially available kits for instance as described in [630]. It is advisable to pool cell suspensions from at least three animals to obtain sufficient cell numbers for analysis.

##### Surface staining:

Following incubation with Fc block (anti-mouse CD16/32, clone 2.4G2) cells are first stained using APC- or PE-conjugated MR1-5-OP-RU or MR1-6-FP (background control) tetramers for 40 min at room temperature in FACS buffer [640]. Cells are washed once in FACS buffer followed by Ab staining for surface markers for 10 min at 4°C. In order to minimize background, it is pivotal to perform lineage exclusion by staining for the following markers: B220, CD19, CD11b, CD11c. Dead cells are excluded using the Zombie Aqua Fixable Viability kit as per the manufacturer’s instructions (Biolegend).

##### Magnetic-bead enrichment:

Due to the scarcity of murine MAIT cells in typical laboratory strains it is strongly advised to bead-enrich MAIT cells prior to downstream analysis. Bead enrichment should be performed in between tetramer staining and staining for additional surface markers. Single-cell suspensions are stained with biotinylated anti-CD19 and anti-B220 Abs. B cells are then depleted using streptavidin microbeads as per the manufacturer’s instructions (Miltenyi Biotec). Following MR1-5-OP-RU-APC tetramer staining, MAIT cells are enriched using anti-APC magnetic microbeads following the manufacturer’s instructions (Miltenyi Biotec).

##### Intracellular staining:

To analyze transcription factor expression, magnetic-bead-enriched MR1-5-OP-RU tetramer^+^ cells from lymphoid organs are stained for surface markers and viability as described above. Samples are then fixed and permeabilized using the Foxp3/Transcription Factor Staining Buffer Set (eBioscience) as per the manufacturer’s instructions, followed by Ab staining for 30 min or overnight.

#### 20.4 Materials

FACS buffer: PBS, 3% FCS

RBC lysis buffer (Qiagen)
Zombie Aqua Fixable Viability kit (Biolegend)
Streptavidin microbeads (Miltenyi Biotec)
Anti-APC magnetic microbeads (Miltenyi Biotec)
Foxp3/Transcription Factor Staining Buffer Set (eBioscience)

Tetramers: mouse MR1-5-OP-RU-APC/-PE (NIH tetramer core facility, Atlanta, USA)

Mouse MR1-6-FP-APC (NIH tetramer core facility, Atlanta, USA)

Antibodies: anti-CD16/32 (clone 2.4G2)
Anti-CD19 (clone 6D5)
Anti-B220 (clone RA3-6B2)
Anti-CD11b (clone M1/70)
Anti-CD11c (clone N418)
Anti-TCRβ (clone H57-597)
Anti-NK1.1 (clone PK136)
Anti-CD44 (clone IM7)
Anti-CD24 (clone M1/69)
Anti-PLZF (clone Mags.21F7)
Anti-T-bet (clone O4-46)
Anti-RORγt (clone Q31-378 or B2D)

#### 20.5 Data analysis

**Figure 70.**
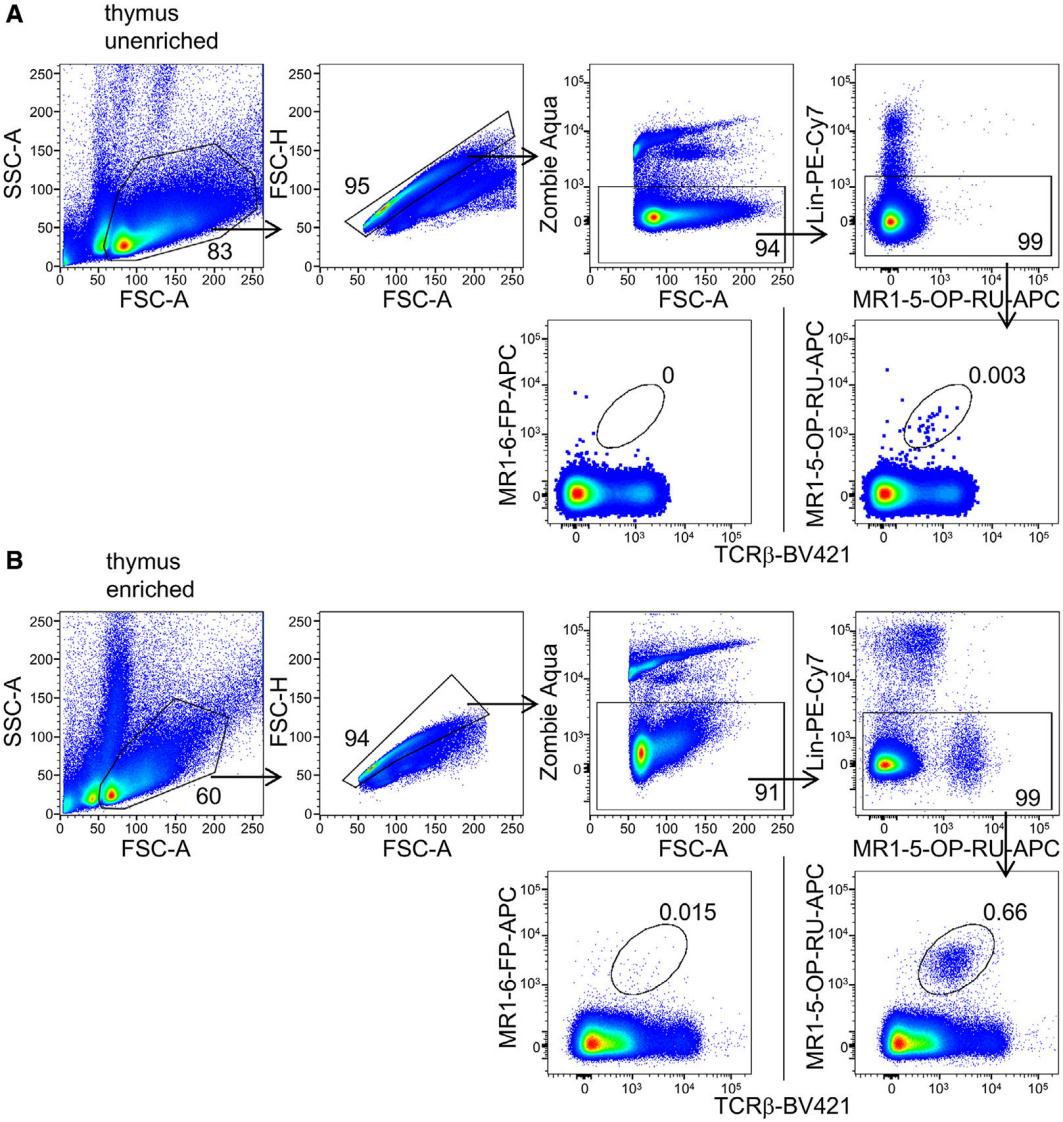
Basic gating strategy for murine thymic MAIT cells. (A) Basic gating strategy for non-enriched murine thymic MAIT cells. (B) Basic gating strategy for thymic MAIT cells following magnetic-bead enrichment. Numbers adjacent to gates indicate frequency of parent population. Stainings with control tetramer MR1-6-FP-APC are displayed as well.

**Figure 71.**
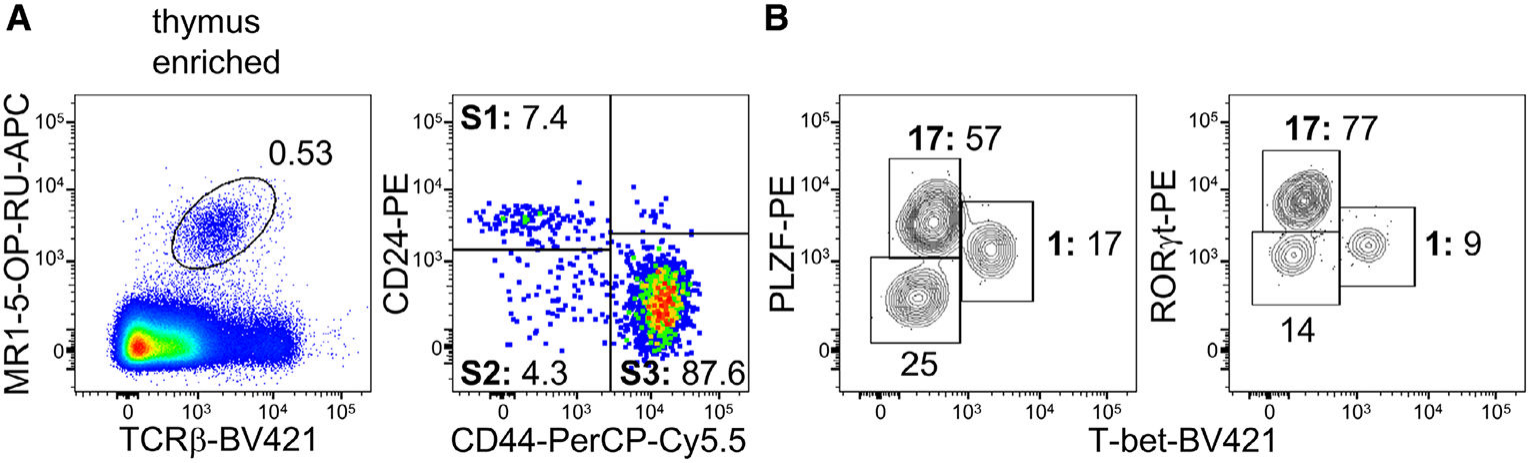
Murine thymic MAIT cell populations. (A) Magnetic-bead enriched MAIT cells from C57BL/6 mice were additionally stained with Abs against CD44 and CD24. Upstream gating was performed as shown in MAIT Figure 70B. (B) Magnetic-bead enriched iNKT cells from C57BL/6 mice were additionally stained intracellulary with Abs against PLZF, T-bet, and RORγt. Numbers adjacent to gates indicate frequency of parent population. Upstream gating was performed as shown in MAIT Figure 70B. Boldface S1, S2, S3 adjacent to gates indicate developmental stages. Boldface 1 and 17 adjacent to gates indicate MAIT1 and MAIT17 subsets, respectively.

Data analysis typically follows a sequence of electronically gating on lymphocytes (FSC/SSC), doublet discrimination (FSC-A/FSC-H), and live/dead discrimination followed by exclusion of cells of other lineages. MAIT cells are then identified as MR1-5-OP-RU tetramer/TCRβ double-positive cells (Figure 70). The resulting population forms the basis for further phenotypic analysis (Figure 71).

#### 20.6 Pitfalls

MAIT cells constitute an extremely rare cell population, rendering subset analysis prone to errors based on background staining (see section Rare Cell Analysis). This difficulty is exacerbated in the analysis of genetically modified mice with developmental defects in the MAIT cell lineage. To minimize background, it is pivotal to include lineage markers in a dump channel and/or enrich prior to downstream analysis. B cells in particular show a high degree of non-specific binding of the MR1 tetramer (both 5-OP-RU and 6-FP loaded). Simultaneous staining of cells with tetramer and anti-TCRβ is possible. However, due to distinct staining conditions, it may result in different staining intensities. Anti-CD24 Ab staining is sensitive to EDTA.

#### 20.7 Top tricks

In order to overcome problems associated with low frequencies of MAIT cells, it is generally recommended to enrich for MR1-5-OP-RU-tet^+^ cells for subset analysis whenever possible. Notably, it has been demonstrated that magnetic-bead-based enrichment via tetramers essentially retains differences between wild-type frequencies and reduced MAIT-cell frequencies observed in genetically modified mice [634, 641]. The underlying mechanism remains unclear, but may be related to the relative inefficiency of tetramer-based enrichment, which in turn may be due to lower affinity of tetramer when compared to Ab-mediated binding. Furthermore, it is absolutely essential to exclude non-T lineage cells, most notably B cells, during gating to limit background staining. It is also advisable to include non-binding MR1-6-FP tetramers as background controls. Finally, for exact quantitation of MAIT cells, dual tetramer staining using a combination of MR1-5-OP-RU-APC and PE-labeled tetramers may help to reduce background [634]. We and others have employed Rag-GFP reporter mice to delineate developmental progression of MAIT cells in the thymus. Such a mouse model may help to further resolve MAIT cell precursors and mature MAIT cell populations in the thymus [617, 634].

#### 20.8 Summary of the phenotype

This is shown in Table 65 (with ref. [680]).

#### 20.9 Key information human versus murine

- MAIT cell development follows similar trajectories in mouse and human, but mature MAIT cells appear to undergo further peripheral expansion in humans [641].
- MAIT cells are scarce in mice, but abundant in humans [635].
- Murine MAIT cell subsets (MAIT1, MAIT17) are distinct and clearly defined [699]. In human blood, MAIT cells are characterized by intermediate, but homogeneous expression of MAIT1 and MAIT17 lineage-defining transcription factors [643]. Skewing toward the production of select cytokines has been observed in various tissues [705].
- Murine MAIT cells are inefficient producers of type 2 cytokines [702]. However, chronically activated human MAIT cells express substantial amounts of IL-13 [696].

## IV T cell assays

### 1 Antigen-specific T cell cytometry: Functional read-outs

#### 1.1 Overview

Ag-specific T cells play a pivotal role in immune protection toward infection and are frequently used for cancer immunotherapy. Ag-specific T cells are also crucially involved in the pathophysiology of chronic inflammatory diseases, such as allergies, inflammatory bowel disease, or autoimmune diseases. The recent SARS-CoV-2 pandemic has dramatically raised the interest in monitoring Ag-specific immune responses with particular emphasis on CD4 and CD8 T cells in addition to standard measurements of serum Ab levels. It has also emphasized the need to validate and standardize assays to make research results comparable. Therefore, the direct visualization, quantification, and characterization of these cells have important diagnostic and therapeutic implications. Peptide-MHC (pMHC) molecules present antigenic peptide (epitopes) to T cells, which are recognized by specific binding of a suitable T cell receptor (TCR), which is expressed in multiple identical copies (usually > 1 × 10^5^ molecules) on the T-cell surface. CD8^+^ T cells recognize peptides presented by MHC class I, while CD4^+^ T cells recognize Ag via MHC class II molecules. Two main experimental approaches have been developed for the detection of Ag-specific T cells: direct labeling of Ag-specific TCRs with soluble peptide/MHC multimers, and function-based assays (such as intracellular cytokine staining, ELISPOT, cytokine capture technology, or the upregulation of so-called activation markers). Both approaches have successfully been combined with magnetic enrichment approaches to cope with the low frequencies of Ag-specific T cells. Their advantages and limitations are described below.

#### 1.2 Introduction

**Figure 72.**
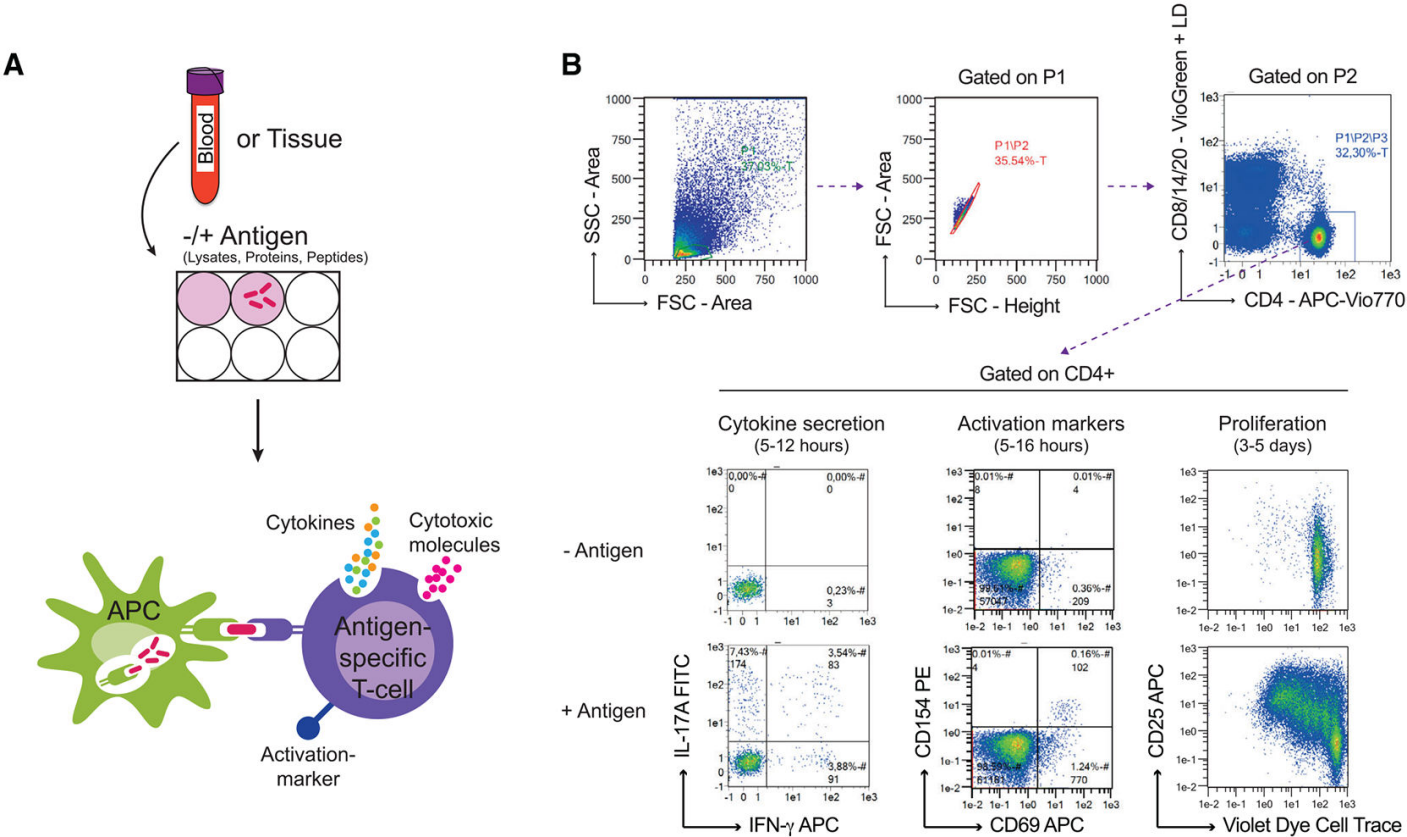
Principal of Ag-specific stimulation assays. (A) Peripheral blood mononuclear cells (PBMC) or single cell suspensions from tissues are incubated with the Ag of interest or without Ag as negative control to determine background levels of the assay. If whole proteins are used for stimulation, the Ag has to be taken up by the autologous Antigen-presenting cells of the cell source, processed and presented on MHC molecules. Peptides of a certain length can bind externally to MHC molecules. (B) The Ag-specific T-cells will start to secrete cytokines and/ or cytotoxic molecules (5-12 hours), express activation markers (5-16 hours) and at later time points start to proliferate (3-5 days). For the different functions of T-cells, such as cytokine release, cytotoxicity, expression of activation markers and proliferation single-cell flow-cytometric assays are available and for most technologies also selection markers on the cell surface are available allowing additional isolation of the specific cells.

As Ag-specific T-cells are rare, a major goal in Ag-specific cytometry is to analyze as much parameters as possible from each single Ag-specific T-cell. Recent advances in multi-color flow cytometry have increased the number of markers that can be analyzed but have also complicated the design and optimization of multi-color Ab panels, as well as the multi-dimensional analysis of such experiments. These important topics have been reviewed elsewhere [706-710] and are also discussed in section IV.8 Key Concepts for the Design and Testing of Multicolor Panels and Chapter VI Section 1–3 Evaluation/Data handling in ref. [22]. In this chapter, we will focus on the use of flow cytometric methods for the detection of Ag-specific T-cells following stimulation with an Ag. Direct labeling of specific TCRs can be achieved by peptide/MHC (pMHC)-multimers (see section Antigen-specific T-cell cytometry - MHC multimers). However, pMHC-multimers can only be generated for a limited number of pre-defined pMHC combinations, in particular for MHC class I peptides and CD8^+^ T-cell analysis. In contrast, MHC class II multimers for the identification of Ag-specific CD4^+^ T-cells are still less well established. In addition, tetramer use is limited for complex Ags or Ags not fully characterized, e.g., microbes, tumors, or autoantigens, and for the heterogeneous MHC background in humans. As an alternative, functional tests provide more flexibility, since they rely on T-cell stimulation by autologous APCs, which can process and present all types of Ags, peptides, proteins, or crude cellular extracts in the context of the physiological MHC background. Following *in vitro* Ag-stimulation, the Ag-induced T-cell response is analyzed as an indirect read-out indicating specific T cells, i.e., proliferation, activation-induced surface or secreted molecules or cytotoxicity [711] (Figure 72).

##### 1.2.1 Selection of the optimal read-out parameter: Minimal manipulation.

Functional assays require stimulation, which may affect T-cell frequency, function, and phenotype [711]. Cellular proliferation as a result and readout of stimulation requires usually several days (typically 3-5 days) of stimulation (see also Chapter VII. Section 7 – DNA synthesis, cell cycle and proliferation in ref. [22]) and introduces an unpredictable bias due to significant *in vitro* selection and “bystander” proliferation. Therefore, it is difficult to extrapolate from frequency, phenotype, and function of cells after proliferation to the original sample, and proliferation-based assays should be used with caution for quantitative or qualitative T-cell analyses. Therefore, short stimulation times should be preferred; for instance cytokines and rapid activation markers (e.g., CD154, CD137, CD69, CD134 (OX40)) typically require only 5–12 h of stimulation before their levels are measurable intracellularly, on the cell surface or in culture supernatants, ensuring minimal manipulation [711]. Furthermore, β2-integrin activation can be detected on activated T cells which occurs even within minutes [712].

For Ag-specific stimulation experiments, it should also be considered that the source of material (whole blood; peripheral blood mononuclear cells (PBMCs); different tissues sources), as well as the treatment of the cell source (fresh or frozen material; resting periods before stimulation; culture medium), might have a profound influence on T-cell marker expression and the detection of Ag-specific T cell responses [713-717]. However, in multi-center trials, cryopreservation of PBMCs is often unavoidable. Therefore, standardized procedures are needed to compare Ag-specific T-cell data from different laboratories [174, 718]. When analyzing and comparing Ag-specific T-cell responses from blood and tissue, also the presence of functional APCs with comparable processing and presenting capacity should be considered.

##### 1.2.2 Selection of the optimal read-out parameter - Integrate all T-cell subsets.

T-cells are heterogeneous and cover a wide range of different phenotypical and functional subsets. Information about the frequency, differentiation stage (e.g., naive, memory), phenotype, and functional properties of Ag-specific T-cells is essential to gain a comprehensive picture about the immune response against a certain Ag and the immune status of an individual. As CD4^+^ and CD8^+^ T-cells provide different functions, also different readouts apply for the detection of Ag-specific CD4^+^ and CD8^+^ T-cells (Table 66; with refs. [719, 723, and 729]).

In particular, CD4^+^ T-cells can acquire a highly diverse set of functional properties. Therefore, Ag-induced cytokine secretion is widely used as a functional read-out for CD4^+^ T-cells. Cytokines can be detected intracellularly in fixed cells, when cytokine secretion is inhibited by the addition of secretion inhibitors like Brefeldin A or Monensin to allow their accumulation [730]. Live cytokine secreting cells may also be identified using a cytokine capture matrix which retains the secreted cytokine on the surface of the secreting cells [731, 732] (see also chapter VII. Section 3 - Intracellular parameters, chapter *V.* Section 1-4 Data acquisition and cell sorting with secretion assay in [22]). Differences may apply with the usage of different secretion inhibitors [174], for example, Monensin has been shown to only insufficiently inhibit TNF-α secretion [733]. Due to the heterogeneity of CD4^+^ T-cells, ideally, the functional read-out should encompass all relevant T-cell types to obtain a complete picture of the immune status, i.e., all conventional T (Tconv) cells, i.e., naïve, all memory subsets as well as Foxp3^+^ regulatory T (Treg) cells, which typically comprise 5-10% of all CD4^+^ T-cells and are essential for tolerance. An alternative to individual cytokines, such as IFN-γ which are often only expressed by a minor fraction of all Ag-specific CD4^+^ T-cells [198, 724, 734], and thus may ignore a significant fraction of specific T cells, are so-called activation markers, that are upregulated on the T-cell surface upon specific T-cell receptor triggering. The combination of the activation markers CD154 (CD40L; which is expressed on all Tconv subsets) and CD137 (4-1BB; which is expressed on Treg) following short-term (6 h) stimulation allows in parallel detection of naive and memory Tconv and Tregs reacting against the same Ag [475, 725, 734, 735].

For CD8^+^ T-cells, cytokines such as TNF-α and IFN-γ are widely used, which are expressed by the majority of the Ag-activated CD8^+^ population. The activation marker CD137 is also expressed by CD8^+^ T-cells following stimulation for >12 h [110, 727, 728], but may also be induced due to bystander activation. Furthermore, for CD8^+^ T-cells detection of cytotoxic activity by staining for cytotoxic effector molecules (e.g., granzyme or perforin) can be used. In contrast to most other mediators, these molecules are found pre-formed in the cells and can be immediately released following Ag stimulation. An alternative approach for measuring cytotoxicity is the detection of CD107a, which is only present on the cell surface transiently following degranulation [736, 737] (see section VII.11 Cytotoxicity).

A common drawback of these techniques is that they all rely on up-regulation or de novo synthesis of the read-out markers, e.g., activation markers or cytokines, and therefore require at least several hours of stimulation. Recently an approach for rapid identification of activated CD8^+^ T cells has been established, based on immediate changes of surface integrins that occur within minutes following Ag stimulation [712]. The authors made use of the fact that resting Ag-experienced T cells express high levels of membrane-bound β2-integrins [738, 739]. TCR activation leads to clustering of the membrane-bound integrins within seconds following stimulation, which can be detected by intercellular adhesion molecule 1 (ICAM-1)-multimers, that specifically bind to activated β2-integrins [712]. An advantage of the assay is the short stimulation time of only several minutes that allows the detection of functional (producing cytokines and/or expressing CD107a) CD8^+^ T cells. However, comparison with peptide MHC multimers showed that only a fraction of the peptide MHC multimer positive T cells stained positive for ICAM-1. Further analyses revealed that activated β2-integrins mark T cells with immediate, strong effector function, but for example miss non-functional Ag-specific cells. In addition, the protocol requires stimulation of low cell numbers in relatively high volumes (7.6x10e5 PBMCs in 380μl test), which limits the detection limit and makes it difficult to scale-up the assay for the detection of low-frequent Ag-specific T cells.

##### 1.2.3 Selection of the optimal read-out parameter – Interassay variability.

Recently, for CD4 T-cells the activation-induced marker (AIM) assay has been increasingly used to identify and investigate Ag-specific T cells. This assay uses various combinations of activation markers, e.g., CD134 together with CD25, PD-L1, CD154, or CD137, and also different stimulation times ranging from 9 to 24 h have been reported [113, 116, 119, 130, 720-722, 740-742]. Unfortunately, to date, the different assays have not been systematically compared with each other. However, especially the highly variable results reported during the recent SARS-CoV-2 pandemic (see below) indicate significant differences between different methods, with significant impact on data interpretation. One study directly compared the use of CD69^+^ CD154^+^ *versus* AIM assays using CD134 plus CD25 or PD-L1, revealing up to 16 fold differences in specific T cell frequencies and also in the background levels [130]. Unfortunately, the specificity of the cells detected by these different assays has not been confirmed, e.g., via single-cell cloning and restimulation (see Section 6.2.6 Controls and statistical analyses in ref. [22]). For CD154 after 7 h of stimulation, we have confirmed the high specificity of the identified T-cells in various settings [726, 734, 735, 743]. However, it cannot be excluded that not all specific T-cells react. Thus, the sensitivity and specificity of the different assays may strongly vary and therefore this has to be determined for the specific experimental conditions applied.

##### 1.2.4 Combination with magnetic enrichment of rare cells.

**Figure 73.**
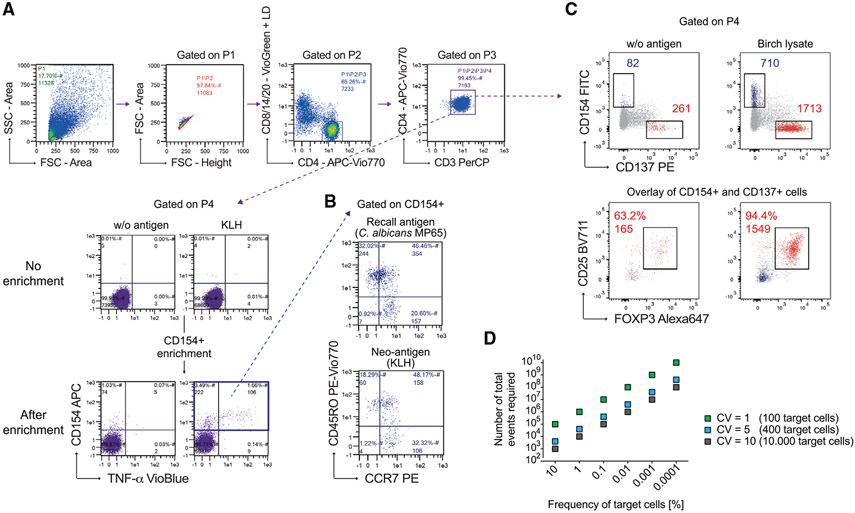
Enrichment of human Ag-specific T-cells increases sensitivity for the detection of rare cells. (A) CD154 and TNF-α expression was analyzed on human CD4^+^ T-cells without addition of an Ag and following stimulation with the neo-Ag keyhole limpet hemocyanin (KLH). Cells are gated on CD4^+^ T-cells and percentage and absolute numbers of CD154^+^ cells after acquiring 5x10e5 PBMCs (upper plots) or obtained from 1×10e8 PBMCs after enrichment of CD154^+^ cells (lower plots). (B) Phenotypic characterization of the enriched CD154^+^CD4^+^ T-cells to discriminated between CD45RO^+^ memory cells and CD45RO-CCR7^+^ naive T-cells, following stimulation with a peptide pool of *C. albicans* MP65 as recall Ag or KLH as neoantigen. (C) Parallel detection of Ag-specific Tconvs (CD154^+^) and Tregs (CD137+) following stimulation with birch pollen lysate and magnetic enrichment for CD154^+^ and CD137^+^ cells from 2x10e7 stimulated PBMC. Upper plots: cells are gated on CD4^+^ T-cells and absolute cell counts of CD154^+^ and CD137^+^ cells with and without stimulation are indicated. Lower plots: Overlayed flow-cytometric analysis of birch-specific CD154^+^ and CD137^+^ cells. Numbers indicate percentages among CD137+CD154-CD4^+^ T-cells and absolute numbers of CD137+CD25+FOXP3^+^ Treg. (D) To describe the precision of flow cytometry data, the coefficient of variance (CV) can be calculated from the variance and the standard deviation (SD). For rare cell analysis, the approximations SD = √r and CV [%] = 100/√r can be used, where r is the number of positive events (53). From CV [%] = 100/√r follows r = [100/CV]^2^. Using this approximation, the number of total required events is illustrated depending on the frequency of target cells for different CVs.

Ag-specific T-cells typically comprise <1% and often <0.1% of the total T-cell population [711]). Therefore, magnetic pre-selection of rare Ag-specific T-cells from large cell samples is frequently used to decrease background and improve optical resolution. Pre-selection increases the sensitivity for the detection of Ag-specific T-cells, i.e., frequencies down to 1 cell within 10^−5^-10^−6^ and thus even detection of specific T cells within the naïve repertoire is possible [731, 734, 744-747]. Enrichment allows the collection of sufficient target cells for subsequent multi-parameter analysis and resolution of small cell subsets. Magnetic enrichment may employ surface markers, e.g., tetramers, CD154, CD137, ICAM-1-multimers, or secreted cytokines [711, 712, 731, 734, 744-747] (Figure 73).

##### 1.2.5 Type of Ag.

As for the functional read-out, there are differences between the Ags used for stimulation of CD4^+^ and CD8^+^ T-cells. CD4^+^ T-cells recognize Ags that are presented via the exogenous pathway of Ag presentation on class II MHC molecules [749]. Accordingly, for CD4^+^ T-cells, peptides, proteins, and even cellular extracts can be used for stimulation. Presentation of peptides from whole proteins depends on the processing activity of the available APCs, which may vary between cell sources (blood, (lymphoid-) organs) and donors. Antigen preparations containing potential innate immune signals (pathogen-associated molecular patterns, PAMPs) may cause bystander activation and specificity of the Ag-reactive T-cells has to be confirmed for each Ag (see also section 6.2.5 Controls and statistical analyses in [22]).

In contrast, stimulation of CD8^+^ T-cells with whole proteins is difficult, since MHC class I epitopes are not easily generated from endocytosed proteins that depend on cross-presenting capacity of the APCs. Therefore, short synthetic peptides are preferable. The use of peptides as Ag stimulants is advantageous as peptides are instantly presented by all APCs expressing MHC molecules, including B cells or other non-classical APCs. Peptides can be used individually or in pools, such pools being able to cover complete protein amino acid sequences (protein spanning peptide pools). The use of peptides of 15 amino acids length and 11 overlaps has proven very successful for both CD4^+^ and CD8^+^ T-cells [750, 751]. The use of 15mers is in conflict with the concept that the binding groove of class I MHC molecules can only accommodate a peptide of 9 amino acids in length. Since approaches using 15mer peptides are successful, it is assumed that mechanisms exist that shorten these peptides in the extracellular space (clipping or trimming) [752, 753].

##### 1.2.6 Type of APCs.

Different types of APCs may be used for Ag-specific T cell stimulation. Ag-presenting cell quality and functional capacity regarding Ag uptake, protein processing, or costimulatory molecules, impacts on the T cell reactivation. Therefore the impact of the APCs used on background and activation level has to be determined for each experimental setting individually. Here, we only describe standard procedures using whole PBMC, which contain monocytes, B cells, and dendritic cells as APCs with the right HLA haplotypes. Typically, these APCs are suitable to take up and process all Ags described above. However, the individual subsets may contribute differentially. For example monocytes and dendritic cells preferentially take up and process proteins from the solution as compared to B cells, which efficiently process and present only proteins binding to their specific Ag receptor but can be externally loaded with short peptide Ags. Thus any strong alteration of the Ag-presenting cell composition or functionality in individual patient samples, e.g., by drug treatment, diseases, such as leukemias, or the genetic background, has to be considered.

##### 1.2.7 Controls and statistical analyses.

Standard controls for flow-cytometric multicolor analyses which apply here (single color, compensation, FMO-controls, exclusion of doublets and dead cells, as well as a dump channel), are described in Chapter IV. Section 3. Controls – determining positivity by eliminating false positives in [22]. However, special emphasis has to be given to elimination of background due to the low frequencies of Ag-specific T-cells, as noted above. A non-stimulated sample processed under identical conditions is absolutely required to determine background. Specificity should be verified for each pMHC-multimer and Ag, especially for preparations containing PAMPs, as well as for different cell sources (blood, tissue). Specificity can be determined, for example, by MHC blocking Abs, the use of fixed APCs (for processing dependent Ags), or expansion of cell lines, and ideally single-cell clones for confirmation of specificity by Ag re-stimulation [734].

Also, a positive control for the assay should be included, to determine functionality of the T-cells and APCs. Polyclonal stimulation can be achieved by e.g., agonistic Abs against CD3 and CD28 or by stimulation with the chemicals phorbol-12-myristate-13-acetate (PMA) and ionomycin. However, these controls only apply for the T-cells and are independent of the presence of functional APCs. Alternatively, super-Ags like *Staphylococcus* enterotoxin B (SEB) can be used, which crosslinks MHC molecules and specific Vβ regions of T-cell receptors. Thus, usage of SEB might be limited in samples with restricted Vβ repertoires. Since polyclonal stimuli are usually very strong, an Ag-specific control might represent a more physiological control, e.g., an Ag derived from an ubiquitous pathogen like *Candida albicans*, or a standard vaccine-like tetanus, to which typically all donors react [734]).

When frequencies of Ag-specific T-cell are calculated, background values have to be subtracted from that of the Ag sample. Regarding statistical significance of rare event analyses, considerations have to be applied to determine the minimal number of events that have to be acquired for statistically relevant analyses. To describe the precision of flow cytometry data, the coefficient of variance (CV) can be calculated from the variance and the standard deviation [748] (Figure 73D). For example, for a CV of 5% at least 400 Ag-specific T-cells have to be acquired. If the Ag-specific cells occur with a frequency of 0.1%, at least 400,000 total events should be acquired. If the frequency of specific cells is just 0.01%, at least 4,000,000 have to be acquired, and so on. This illustrates that for many Ags, magnetic pre-selection of the rare Ag-specific T-cells from large cell samples is necessary to increase the sensitivity of the assay and obtain sufficient target cells for statistically relevant analyses (see also Chapter VI. Section 3 - Statistics for flow cytometry in [22]).

For methods employing enrichment, the absolute count of target cells obtained from a certain input cell number has to be determined to calculate frequencies in the original sample. The frequency of positive cells after enrichment is not relevant for quantification. A minimal signal-to-noise ratio and minimal number of events per input cell number have to be determined for each test system independently (see also section Rare cells (general rules)).

##### 1.2.8 Interpretation of results.

Originally, specific T-cell analysis relied on the idea that Ag-specific memory-type T-cells can only be detected in Ag-experienced individuals. However, recent advances, in particular in the enrichment of rare cells, have allowed the detection of rare specific T-cells even within the naïve repertoire [734, 746, 747, 754-757] (Figure 73B). These analyses also showed that the memory compartment contains a significant fraction of specific T-cells against bona fide “neo-Ags,” i.e., Ags not previously encountered by the immune system. This may result from specific (structurally related epitopes) or from statistical cross-reactivity, i.e., recognition of a neo-epitope by TCRs from a polyclonal repertoire [734, 755, 757]. Thus, the presence of memory-type T-cells does not *per se* imply that this results from a genuine Ag-specific immune response. Therefore, additional biological parameters have to be considered to determine the actual immune status: overall ratio between specific memory to naive and Treg cells, the ratio of memory T-cells in the Ag-specific population *versus* the total T-cell population (is expected to be >1 in genuine memory responses), clonal composition of TCRs (deep sequencing), and affinity or functional avidity which can be estimated by restimulation of expanded Ag-specific clones or cell lines with decreasing Ag concentrations or via reversible MHC-multimers [758].

Taken together, Ag-specific cytometry allows combination with multiparametric single-cell analysis tools for full resolution of the Ag-specific immune response.

#### 1.3 Step-by-step sample preparation

##### 1.3.1 Stimulation of freshly isolated PBMCs for induction of CD154 and CD137 expression.

1. Isolate PBMCs from whole blood or buffy coats using Biocoll Separating Solution under sterile conditions. For the isolation of the PBMCs dilute the blood with two to five times the volume of sterile 1× PBS and slowly layer 35 mL of this blood-PBS mix over 15 mL of the Biocoll Separating Solution in a 50 mL canonical tube. Centrifuge for 20 min at 700 × g at room temperature without break. Carefully aspirate and discard the upper layer and collect the mononuclear cell layer in a new 50 mL canonical tube. Fill-up the tube with PBS and centrifuge for 15 min at 400 × g at 4 °C. Discard the supernatant, resuspend the cell pellet with 50 mL RPMI 1640, and centrifuge for 10 minutes at 200 × g. Discard the supernatant, resuspend the cell pellet in an appropriate amount of RPMI 1640, and store at 4°C until further treatment.
2. Seed cells at a density of 2 × 10^7^/mL per well onto 12-well plates for each non-stimulated control and Ag-stimulated sample. A negative control is necessary to determine the background levels of the assay.
3. Take sample (1 × 10^6^ cells) to determine CD4^+^ T cell proportion in the original fraction.
4. Cultivate cells in RPMI 1640 medium supplemented with 5% human AB serum at 37°C, 5% CO2 until further treatment.
5. Add human CD40 pure–functional grade to a final concentration of 1 μg/mL to each well.
6. Add human CD28 pure–functional grade to a final concentration of 1 μg/mL to each well.
7. Add Ag of interest to corresponding wells according to manufacturer’s instructions
8. Incubate cells for 5.5 h at 37°C, 5% CO2.
9. Add brefeldin A to a final concentration of 1 μg/mL to each well.
10. Incubate cells for further 1.5 h at 37°C, 5 % CO2.

##### 1.3.2 Magnetic labeling for enrichment of CD154^+^ and CD137^+^ cells.

1. Harvest cells into 1.5 mL Eppendorf Tubes by constantly scraping and resuspending.
2. Wash each well with 400 μL PEB buffer and add to corresponding Eppendorf tubes.
3. Centrifuge cells for 5 min at 400 × *g* at 4°C.
4. Carefully aspirate supernatant and resuspend cell pellet in 80 μL PEB buffer.
5. Pre-mix each 10 μL of CD154-Biotin and 10 μL of CD137-PE and add 20 μL of pre-mixed Abs to the cells - incubate for 15 min at 4°C (mix well).
6. Add 1 mL PEB buffer to wash the cells and centrifuge for 5 min at 400 × *g* at 4°C.
7. Carefully aspirate supernatant, pre-mix each 10 μL of Anti-Biotin MicroBeads and 10 μL of Anti-PE MicroBeads and add 20 μL of MicroBead mix to cell pellet (mix well).
8. Incubate cells for 15 min at 4°C.
9. Add 1 mL PEB buffer to wash the cells and centrifuge for 5 min at 400 × *g* at 4°C.
10. Carefully discard supernatant and resuspend cell pellet in 500 μL buffer.

##### 1.3.3 Magnetic enrichment and surface staining.

1. Place MS Column in the magnetic field of suitable MACS^®^ Separator and prepare MS column by rinsing with 500 μL of PEB buffer.
2. Apply cells onto the column. Let cell suspension completely flow through the column before proceeding with the next step.
3. Wash cells by rinsing the column once with 1 mL PEB buffer. Let buffer completely flow through the column before proceeding with the next step.
4. Wash cells by rinsing the column with 500 μL 1×PBS. Let buffer completely flow through before proceeding with the next step.
5. Add 60 μL of mix of required surface staining Abs to each column and incubate for 15 min in the dark at room temperature.
6. Wash cells by rinsing the column with 500 μL 1×PBS.
7. Remove the column from the separator and place it onto a 1.5 mL Eppendorf tube for collecting the cells.
8. Elute the cells from the column by adding 800 μL of PEB buffer and pushing the plunger into the column. When staining transcription factors elute the cells by adding 600 μL PEB buffer.
9. Add 200 μL of Inside Fix, mix well and incubate for 10 minutes in the dark at room temperature. When staining transcription factors use the Foxp3 Staining Buffer Set, add 400 μL of fixing solution and incubate for 20 min in the dark at 4°C.

##### 1.3.4 Intracellular staining.

1. Place a new MS Column in the magnetic field of suitable MACS^®^ Separator and prepare MS column by rinsing with 500 μL of PEB buffer.
2. Apply the fixed cell suspension onto column. Let cell suspension completely flow through the column before proceeding with the next step.
3. Wash cells by rinsing the column with 200 μL Inside Perm. Let buffer completely flow through the column before proceeding with the next step. When staining transcription factors use the Perm buffer included in the Foxp3 Staining Buffer Set.
4. Add 60 μL of mix of required intracellular staining Abs to each column and incubate for 15 min in the dark at room temperature. For transcription factor staining, incubate 30 min in the dark at room temperature.
5. Wash cells again by rinsing the column with 200 μL Inside Perm or Perm buffer included in the Foxp3 Staining Buffer Set.
6. Remove the column from the separator and place it onto a new 1.5 mL Eppendorf or FACS collection tube.
7. Elute the cells from the column by adding 1000 μL PEB buffer and pushing the plunger into the column.
8. Centrifuge cells for 5 min at 400 × g at 4°C and carefully remove 800 μL of the supernatant; resuspend cells pellet in the remaining 200 μL PEB buffer.
9. Cells are now ready for flow cytometric analysis.

##### 1.3.5 Statistical analyses.

1. Determine the absolute cell numbers of CD154^+^ and CD137^+^ cells obtained from your input cell number and subtract the background value of the non-stimulated control from the Ag samples to calculate the frequencies in the original sample.

#### 1.4 Materials

**Table:** 

Media, Buffer, Staining Reagents, and Flow Cytometer	Manufacturer Information
Biocoll Seperating Solution	Ficoll-Paque Plus Cytiva #17144003
1×PBS	DPBS Lonza #17-512F
RPMI 1640 medium	Gibco #52400-025
Human AB serum	Sigma #H4522-100ML
CD40 pure – functional grade, human	Miltenyi Biotec #130-094-133
CD28 pure – functional grade, human	Miltenyi Biotec #130-093-375
Antigens of interest	
Brefeldin A	Sigma Aldrich #B7651
CD154 MicroBead Kit, human	Miltenyi Biotec #130-092-658
CD137 MicroBead Kit, human	Miltenyi Biotec #130-093-476
MS columns	Miltenyi Biotec #130-042-201
MACS Separator for MS columns	Mitenyi Biotec #130-042-109
MACS MultiStand	Miltenyi Biotec #130-042-303
Bovine serum albumin (BSA) fraction	Serva #11930.04
EDTA	Promega Corporation #V4233
PEB buffer: 0,5 % BSA and 2 mM EDTA in PBS	
Antibodies for surface staining	
Tandem Signal Enhancer, human	Miltenyi Biotec #130-099-888
Inside Stain Kit	Miltenyi Biotec #130-090-477
Foxp3 Staining Buffer Set	Miltenyi Biotec #130-093-142
Antibodies for intracellular staining	
Viobility 405/520 Fixable Dye	Miltenyi Biotec #130-110-206
LSRFortessa^™^	BD Biosciences

#### 1.5 Pitfalls

1. Before isolation of the PBMCs, do not store blood at 4°C.
2. For all stainings on the column, bubble generation should be avoided since this can lead to clogging and therefore negatively influence the quality of the staining and the magnetic enrichment.
3. When using viability fixable dyes a washing step only with PBS is absolutely essential since these amino-reactive dyes would bind to the amine-groups of the proteins in the buffer.
4. Don’t change stimulation times since these times are optimized in terms of up-regulation of CD154 and CD137 and at the same time of blocking cytokine secretion.
5. To make sure that all cells are stained, do not change the final staining volume of 60 μL, since the void volume of the MS column is 60 μL.

#### 1.6 Top tricks

1. PBMCs should be isolated on the same day the blood samples are donated.
2. For practical reasons: Overnight resting (<16 h) at 37°C in the incubator of freshly isolated PBMCs before addition of Ag in our hands generated optimal results (signal/noise ratio) and allows easier integration of the ARTE assay into two working days (day 1: blood processing and cell seeding, day 2: stimulation and enrichment).
3. Since this assay has been established to analyze rare cells, it is very important to minimize background and therefore some critical points should be considered:
  - Work under sterile conditions
  - Do not use reagents that contain any kind of non-human proteins (e.g., BSA or FCS)
  - Pre-screen different lots of human AB serum and use the same for all experiments.
4. For extremely rare cells like neoantigen- or autoantigen-specific T cells increase the number of seeded cells to 5x10^7 in a 6-well plate. If you increase the number of cells, scale up volumes accordingly.
5. When harvesting the cells, check microscopically if the wells are empty to avoid cell loss, because activated T cells might stick to the bottom.
6. If tandem dyes are used, add Tandem Signal Enhancer to the staining mix.
7. Until flow cytometric acquisition samples should be stored in the dark and preferably at 4°C.
8. Determine a minimal signal-to-noise ratio and a minimal number of target cells per input cell number reasonable for your experimental setup. For us, a threshold of >50 target cells/10^5^ input CD4^+^ T cells and a minimal signal-to-noise ratio of 1.5-fold have been approved.

#### 1.7 Clinical Relevance Statement

Assays to detect and characterize Ag-specific T cells play an important role in immunological research and are broadly used to investigate for example the role of T cells in autoimmune diseases, infectious diseases, or vaccination reactions. Recently, this has been highlighted in the context of SARS-Cov-2 infection during the global COVID-19 pandemic. However, depending on the selected assay (Cytokine secretion (ELISpot), Proliferation assay, AIM assays, or ARTE (Ag-reactive T cell enrichment)), highly variable results have been published, with regard to SARS-CoV-2-specific T cells in response to infection and vaccination, as well as in unexposed donors (“pre-existing memory”). These discrepancies also led to opposing conclusions, for example regarding a protective role of pre-existing memory induced by related human coronavirus [114, 116, 119, 122, 740, 742, 743, 759] or regarding the quantity or quality of the T cell response underlying severe versus mild disease outcome [113, 120, 743]. This demonstrates once more the high assay variability regarding sensitivity and specificity to detect Ag-reactive T cells. As already discussed in sections 6.2.3 “Selection of the optimal read-out parameter – interassay variability” and 6.2.6 “Controls and statistical analyses”, this highlights that the proof of specificity for each Ag is absolutely required to ensure that the detected frequencies are not biased by the technological approach and for correct interpretation of the data.

### 2 Measuring Ag-specific CD8 T cell responses

#### 2.1 Overview

CD8 T cells play a central role in the adaptive immune response to control disease. Identification of Ag-specific CD8 T cells within the large pool of bulk CD8 T cells is essential in order to understand the disease-specific T cell immune response. Here, we describe a high-throughput technology that can be used to identify, monitor, and characterize the Ag-specific T cell response in human disease.

#### 2.2 Introduction

CD8 T cells express clone-specific T cell receptors (TCRs) that recognize their cognate peptide restricted by the major histocompatibility complex (MHC) class I of the target cell. Over two decades ago, Davis and colleagues developed a technique that mimics the interaction between the TCR and the peptide-MHC (pMHC) complex, which allowed them to monitor human immunodeficiency virus (HIV)-specific T cells in patients [760]. Their approach built the foundation for the development of an indispensable tool to monitor Ag-specific T cell immunity.

One major limitation of the classical approach is that the refolding process of individual pMHC complexes is time consuming and laborious. To overcome this limitation, we make use of the ultraviolet (UV) light-mediated peptide exchange method [761, 762]. With this technology, the MHC complex is refolded in the presence of a peptide containing a UV light-sensitive amino acid and a rescue peptide of choice. Exposure to UV light results in the degradation of the UV-sensitive peptide leading to the dissolution of the MHC complex, which is stabilized through the immediate binding of the rescue peptide of choice. The UV-mediated exchange can be performed in a multi-well format, allowing the generation of thousands of unique pMHC complexes in parallel. Alternative methods that allow the rapid generation of various pMHC multimers in parallel include the use of temperaturelabile peptides [763], periodate-cleavable peptides that may be cleaved by sodium dithionite [764], azobenzene-containing peptides [765], or the use of certain di-peptides peptides that bind specifically to the F pocket of MHC class I molecules, catalyzing rapid exchange with peptides in the environment [766].

The classical approach to identify Ag-specific T cells is based on flow cytometry and the use of pMHC complexes conjugated to fluorescent streptavidin dyes [760]. Advances in mass cytometry have provided additional avenues to identify Ag-specific T cells using heavy metal isotope-labeled pMHC complexes, however, this method destroys the cognate cells and therefore does not allow further downstream analysis [767]. The major drawback of both technologies is that each individual pMHC complex is coupled to a single fluorescent streptavidin dye or heavy metal isotope and therefore the number of epitopes that can be screened for T cell reactivity in parallel is limited by the number of available fluorochromes or heavy metal isotypes.

To overcome this limitation, several multiplexing strategies based on combinatorial encoding of pMHC multimers described in detail below have been developed, which greatly increase the number of T cell reactivities that can be detected in a single sample [768-771]. More recent high-throughput approaches make use of pMHC multimers coupled to peptide-specific DNA oligo barcodes [772-774]. Although the cell mixture can be sequenced as a whole, the sequencing costs can be reduced if Ag-specific T cells are isolated prior to sequencing using fluorescent pMHC multimers. This approach also allows for simultaneous quantification of surface expression markers on the protein level using DNA oligo barcoded Abs. Ultimately, proper use of DNA barcoded reagents allows simultaneous identification of the TCR sequence, transcriptome, surface protein expression and peptide-specificity of T cells.

In this chapter, we describe a multiplexing strategy based on flow cytometry and the generation of dual fluorochrome encoded pMHC multimers [770, 771]. Using this labeling approach, the number of unique codes can be calculated using factorial operations. Theoretically, the use of 14 distinct fluorochromes, for example, would yield 91 possible unique dual codes: (14 × 13)/(1 × 2) = 91. However, in reality, the combination of some fluorochromes results in spillover issues that would mask the real signal and we are therefore currently using 14 distinct fluorochromes to make up to 75 unique dual codes. Besides the major benefit of increasing the number of specificities that can be screened in parallel, the use of dual fluorochrome codes significantly reduces the background signal. This is because the vast majority of background signal is detected in either one or more than two detectors. The Boolean gating strategy used to include the signal that is only dual fluorochrome positive is central to improve the signal-to-noise ratio up to 10-fold, which greatly increases the sensitivity of the assay. Using this gating strategy, we are currently working with a cutoff of 0.005% of total CD8^+^ T cells and a minimum of 5 recorded events. Furthermore, with the continuous development of new fluorescent dyes and better flow cytometers additional Abs can be included in the analysis to assess surface and/or intracellular expression of phenotype or functional T cell markers. On the basis of the high sensitivity and robustness, this method is highly suitable for high-throughput analysis of Ag-specific T cell responses in patient material.

#### 2.3 Step-by-step sample preparation

##### 2.3.1 Generation of fluorescent pMHC multimers.

**Figure 74.**
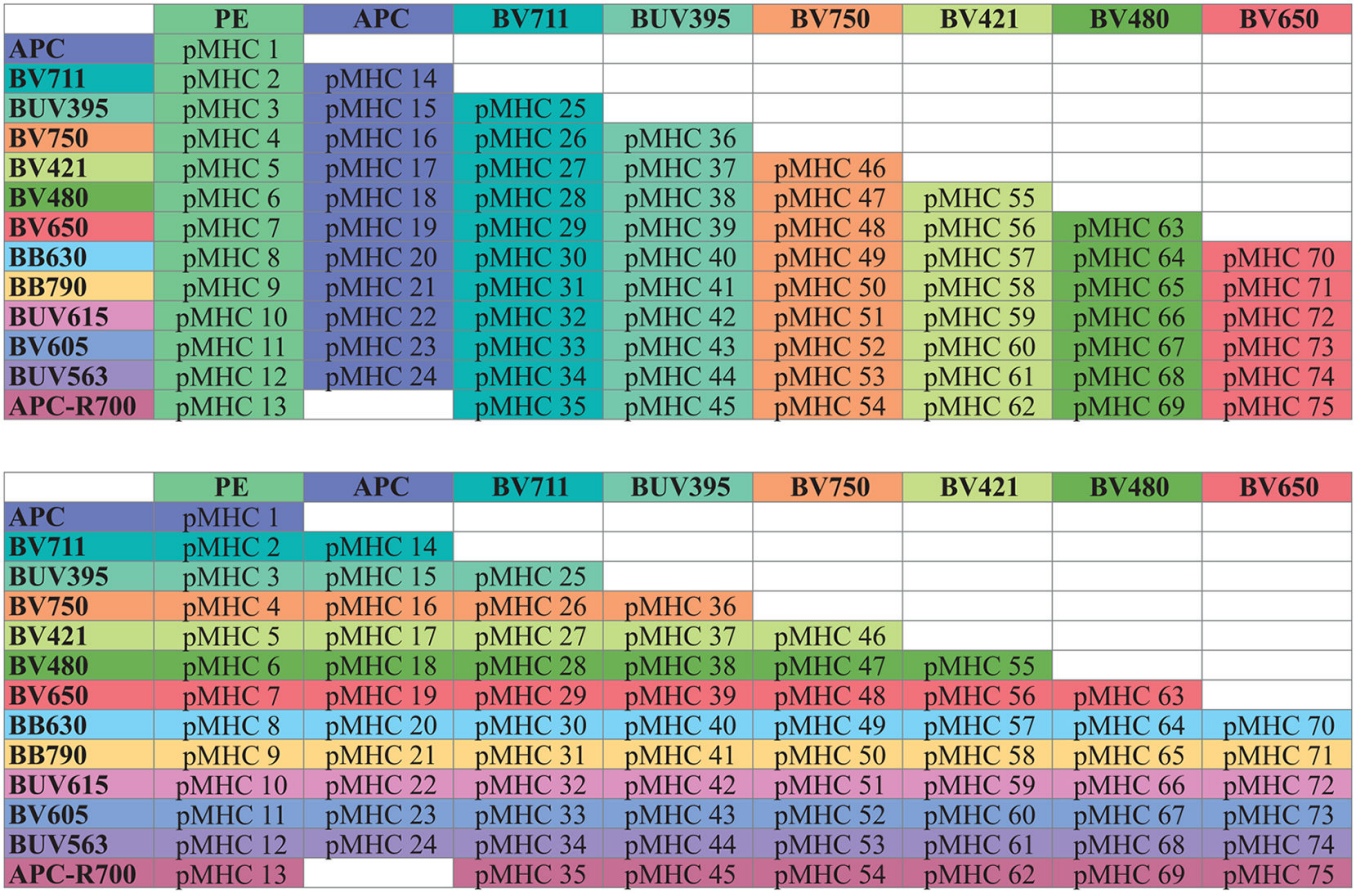
Schematic overview of the combinatorial staircase encoding 75 unique pMHC complexes with 75 unique dual fluorochrome combinations, allowing the detection of 75 different T cell responses in parallel.

UV-cleavable pMHC monomers [762,775] are loaded (1 h, 4°C) with an array of selected peptides in a multi-well format via the UV-induced ligand exchange method [761,770]. Protein aggregates are removed by centrifugation (10 min, 4°C, 4,210 × *g*). Each individual pMHC monomer is then conjugated (30 min, on ice) to two fluorescent streptavidin dyes [761,770]. Milk can be added if the actual binding of the peptide to the MHC has not yet been established to block and capture unspecific peptide binding residues and thereby reduce aggregate formation. A representative example of our current panel setup in which 14 fluorescent streptavidin dyes are used to encode 75 pMHC complexes is illustrated in Figure 74. Finally, the mixture is incubated with D-biotin (20 min, on ice) in the presence of sodium azide (NaN_3_) to block residual streptavidin binding sites to prevent binding of unconjugated pMHC complexes to other fluorochromes when combining all fluorescent pMHC complexes. The fluorescent pMHC multimers can either be used immediately or stored at 4°C for up to 24 h.

##### 2.3.2 Staining.

Peripheral blood mononuclear cell (PBMC) samples are thawed, washed, and incubated (30 min, 37°C) in T cell medium supplemented with deoxyribonuclease (DNase) to degrade DNA/RNA released from dead cells to prevent cell death. When working with oligo-barcoded pMHC complexes, make sure to remove the DNAse by sufficient washing. The pMHC multimers are pooled in the presence of Brilliant Staining Buffer Plus and aggregates are removed by centrifugation (4°C, 2 min, 10,000 × g). Antigen-specific T cells are then first stained with the non-aggregated pMHC multimer mixture (15 min, 37 °C) and subsequently with the surface marker Abs (20 min, on ice). Cells are washed twice before acquisition.

#### 2.4 Data analysis

**Figure 75.**
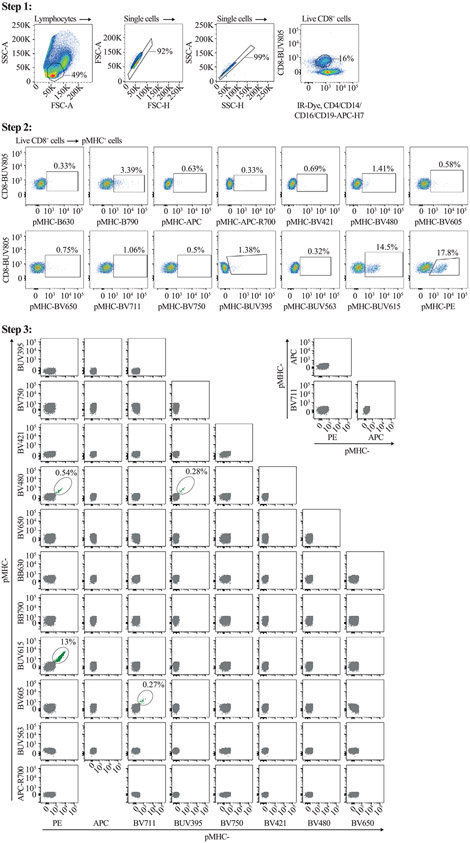
Representative gating strategy used to identify human SARS-CoV-2-specific CD8 T cell responses. Data used in this figure is from [776], which is licensed under CC BY 4.0. Step 1: Gating strategy used to identify single and live CD8^+^ cells. Step 2: Gating strategy used to identify pMHC^+^ CD8^+^ cells required for the Boolean gating. Step 3: Representative overview of all 75 pMHC dual color code combinations after Boolean gating. Antigen-specific CD8 T cells (double-positive pMHC^+^ CD8^+^ cells) are shown in green and bulk CD8 T cells (pMHC^−^ CD8^+^ cells) in grey. Percentage of Ag-specific CD8 T cells of total CD8 T cells is indicated if applicable.

The following gating strategy is applied to identify CD8^+^ T cells: (i) selection of single-cell lymphocytes [forward scatter (FSC)-W/H low, side scatter (SSC)-W/H low, FSC/SSC-A], (ii) selection of live (IRDye low-dim) CD8 positive and ‘dump’ (anti-CD4, anti-CD14, anti-CD16, anti-CD19) negative cells, and (iii) selection of pMHC positive (pMHC-APC, pMHC-APC-R700, pMHC-BB630, pMHC-BB790, pMHC-BUV-395, pMHC-BUV563, pMHC-BUV615, pMHC-BV241, pMHC-BV480, pMHC-BV605, pMHC-BV650, pMHC-BV711, pMHC-BV750, pMHC-PE) CD8 T cells, (iv) Boolean gating is used to identify Ag-specific T cells that are positive for only two and none of the other fluorescent pMHC multimers. An example of the full gating strategy is shown in Figure 75. Cut-off values for the definition of positive Ag-specific CD8 T cell responses were ≥ 5 events and ≥ 0.005% of total CD8 T cells.

#### 2.5 Materials

##### 2.5.1 Generation of fluorescent pMHC multimers.

- 254/366 nM UV lamp
- UV-cleavable pMHC monomers (100 μg/ml)
- Peptides of interest dissolved in dimethylsulfoxide (DMSO, Sigma, 276855)
- Fluorescent streptavidin dyes: APC-Streptavidin (Invitrogen, clone: S868, dilution: 1/16), APC-R700-Streptavidin (BD Biosciences, 565144, dilution: 1/10), BB630-Streptavidin (BD Biosciences, custom, dilution: 1/10), BB790-Streptavidin (BD Biosciences, custom, dilution: 1/10), BUV395-Streptavidin (BD Biosciences, 564176, dilution: 1/5), BUV563-Streptavidin (BD Biosciences, 567655, dilution: 1/6), BUV615-Streptavidin (BD Biosciences, 613013, dilution: 1/10), BV241-Streptavidin (BD Biosciences, 563259, dilution: 1/5), BV480-Streptavidin (BD Biosciences, 564876, dilution: 1/5), BV605-Streptavidin (BD Biosciences, 563260, dilution: 1/5), BV650-Streptavidin (BD Biosciences, 563855, dilution: 1/5), BV711-Streptavidin (BD Biosciences, 563262, dilution: 1/8), BV750-Streptavidin (BD Biosciences, custom, dilution: 1/10), PE-Streptavidin (Invitrogen, S866, dilution: 1/9)
- Milk (Sigma, LP0031, 1% w/v) in phosphate buffered saline (PBS)
- D-biotin (Sigma, B4501, 26.3 mM) and (NaN3, 0.02% w/v) in PBS

##### 2.5.2 Staining.

- T cell Medium: Roswell Park Memorial Institute 1640 (RPMI 1640, Life Technologies, 21875-034) supplemented with fetal calf serum (FCS, Sigma, F7524, 10% v/v), penicillin-streptomycin (Life Technologies, 15140-122, 1% v/v) and deoxyribonuclease (DNase, Merck-Millipore, 70746-4, 2500 U/mL)
- Non-aggregated fluorescent pMHC multimer mixture
- Surface marker Abs: CD8-BUV805 (BD Biosciences, clone: SK8, cat: 612889, dilution: 1/50), CD4-APCH-H7 (BD Biosciences, clone: SK3, cat: 641398, dilution: 1/100), CD14-APC-H7 (BD Bioscience, clone: MφP9, cat: 561080, dilution: 1/100), CD16-APC-H7 (BD Biosciences, clone: 3G8, cat: 560715, dilution: 1/100), CD19-APC-H7 (BD Biosciences, clone: SJ25C1, cat: 560177, dilution: 1/100), LIVE/DEAD Fixable IR Dead Cell Stain Kit (Invitrogen, cat: L10199, dilution: 1/200)
- Brilliant Staining Buffer Plus (BD, 563794)
- Appropriate compensation controls for each fluorochrome
- BD FACSymphony A5: blue laser (488 nm at 200 mW): BB630, 600LP, 610/20BP; BB790, 750LP, 780/60BP. Red laser (637 nm at 140 mW): APC, 670/30BP, APC-R700, 690LP, 630/45BP, IRDye and APC-H7, 750LP, 780/60BP. Violet laser (405 nm at 100 mW): BV421, 420LP, 431/28BP; BV480, 455LP, 470/20BP; BV605, 565LP, 605/40BP; BV650, 635LP, 661/11BP; BV711, 711/85, 685; BV750, 735LP, 750/30BP, BV786, 780/60BP, 750LP. UV laser (355 nm at 75 mW): BUV395, 379/28BP; BUV563, 550LP, 580/20BP; BUV615, 600LP, 615/20BP; BUV805, 770LP, 819/44BP. Yellow-green laser (561 nm at 150 mW): PE, 586/15BP; PE Dazzle-594, 600LP, 610/20BP

#### 2.6 Pitfalls

- Light exposure: Fluorochromes, in particular tandem dyes, that are exposed to prolonged light exposure may bleach or break down. It is therefore crucial to work fast and keep the pMHC complexes in the dark as much as possible.
- Temperature: pMHC complexes can be unstable at temperatures above 4°C. It is therefore highly recommended to work on ice to keep the reagents as cool as possible.
- Peptide solubility: As the solubility of the peptide influences the ligand exchange, it is possible to add ligands that have a poor solubility in water from stocks in DMSO. It has been shown that the ligand exchange reactions proceed normally in conditions up to 10% DMSO [761].
- Protein stickiness: Proteins stick to plastic and it is therefore essential to perform the protein-containing reactions using polypropylene material to avoid loss of protein through sticking to the plates/tubes.
- Protein aggregates: Indicated centrifugation steps of the pMHC complexes are essential to eliminate aggregates in our staining that can cause significant background issues.
- Staining artifacts: If more than two BD Horizon Brilliant fluorescent polymer dyes are used in one staining, fluorescent interactions of these reagents may cause staining artifacts. The use of the BD Brilliant Stain Buffer that has been designed to resolve these issues is therefore highly recommended.
- Cross-reactivity: T cell receptors can be cross-reactive across human leukocyte Ag (HLA) alleles that belong to the same HLA supertype [777] as well as across peptides with a significant level of sequence identity [778]. Due to the Boolean gating strategy cross-reactive T cells may be lost as they are no longer positive for only two but three or more fluorescent pMHC multimers. It is therefore recommended to screen for T cell reactivity for epitopes with a significant level of sequence identity or similar restriction elements in different tubes.
- Sub-optimal signal to noise ratio: Titrations of each individual Ab and each individual fluorescent pMHC multimer used are crucial to ensure optimal signal to noise ratio. Once the optimal dilution has been determined, it is crucial to test for spillover and compensation issues by a step-by-step approach each fluorochrome used.

#### 2.7 Top Tricks

- The addition of milk to the pMHC multimers after adding the fluorescent streptavidin dyes can help reduce the level of aggregates when using rescue peptides that may not be able to bind sufficiently to stabilize the MHC complex.
- To ensure that the identified Ag-specific T cells are indeed real, a confirmation is required in an independent experiment. For this purpose, it is recommended to make new reagents for the potential hits and stain the same sample with a different dual fluorochrome code. We have previously demonstrated that the reproducibility between these independent experiments is high (R2 = 0.9638) [779].
- When selecting what fluorochromes are better suited for the generation of pMHC multimers, the main determinant is the configuration of the flow cytometer that will be used. Next is a consideration of brightness. In case the goal of using the technology to detect viral responses, the brightness is of less concern compared to detecting T cell responses against self-Ags. Nevertheless, it is advisable to select bright fluorochromes, and when using less bright fluorochromes to only combine them in the dual-codes with the fluorochromes that give a bright signal to ensure the Ag-specific T cell population is separated from the background.
- After multimer formation, addition of D-biotin ensures any remaining free binding sites on the streptavidin-conjugated fluorochromes are blocked, thereby preventing the binding of unconjugated pMHC complexes to other fluorochromes when collecting the pMHC multimer collections prior to staining.
- T cells are stained with non-aggregated multimers for a maximum of 15 min at 37°C if the goal is to solely measure T cell responses, or for a maximum of 30 min at 4°C if the goal is to also sort out the cells for downstream transcriptional analyses. Staining at 37°C results in improved binding of the pMHC complexes due to pMHC-TCR dissociation being significantly delayed at this temperature [780].
- It is advised to pick and choose a certain fluorochrome, ideally with the same emission spectrum as the live/dead-marker, that is conjugated to Abs targeting cell surface markers unique to unwanted cells to act as a “dump-channel.” These unwanted cell surface markers include CD4, CD14, CD16, and CD19, specific for (among others) T helper cells, monocytes, NK cells, macrophages, and B cells.

#### 2.8 Clinical relevance statement

We have previously demonstrated the value of the technology to map tumor-specific T cells specific for shared Ags in large patient cohorts as well as neoantigen-specific T cells on a patient-specific basis [779,781-783]. In addition, we have recently used the assay described in this section to identify and characterize the SARS-CoV-2-specific CD8 T cell response in COVID-19 patients, as can be seen in Figure 75 [776].

### 3 Antigen-specific T cell cytometry: MHC multimers

#### 3.1 Overview

The reliable identification of Ag-specific T cells requires new staining methods apart from conventional surface staining protocols due to the high variability of TCR structure within the Ag binding site. These challenges were historically addressed by generating, with the help of molecular biology, multimerized peptide MHC structures, so-called multimers, which mimic the natural occurring TCR receptor on APCs. Hereafter we discuss recent improvements of these staining reagents especially concerning reversible TCR staining and a flexible broadly applicable variation of multimers, the so-called FLEXamers. Further on we provide insights to the challenges in assay design to reliable stain Ag-specific T cells and avoid the pitfalls of staining artifacts with the use of multimers.

#### 3.2 Introduction

Function-independent Ag-specific T cell identification can be applied directly to a sample *ex vivo* and does not rely on *in vitro* T cell activation, in contrast to many function-based assays. Compared to the broadly applied detection of Ags by mAbs, detection of TCR-ligand (=pMHC)-binding Ag-specific T cells has turned out to be challenging. This is mainly due to the relatively low binding affinity of TCR–pMHC monomer interactions, which does not allow using soluble (monomeric) pMHC for stable T cell staining. Altman and Davis addressed this problem by the development of so-called “MHC tetramers” [760]. The principle behind this approach is the multimerization of the natural TCR ligand, e.g., to tetrameric complexes, thereby increasing the binding avidity to surface-expressed TCRs. Dimerization of pMHC via immune globulin fusion proteins can be sufficient to detect Ag-specific T cells [784], but such pMHC dimers often fail to identify all Ag-reactive T cells present in a polyclonal population [785]. However, also pMHC tetramers might not label all epitope-reactive T cells, which could be due to very low-affinity TCRs [786] or TCR/ co-receptor downregulation or variable surface distribution [787].

**Figure 76.**
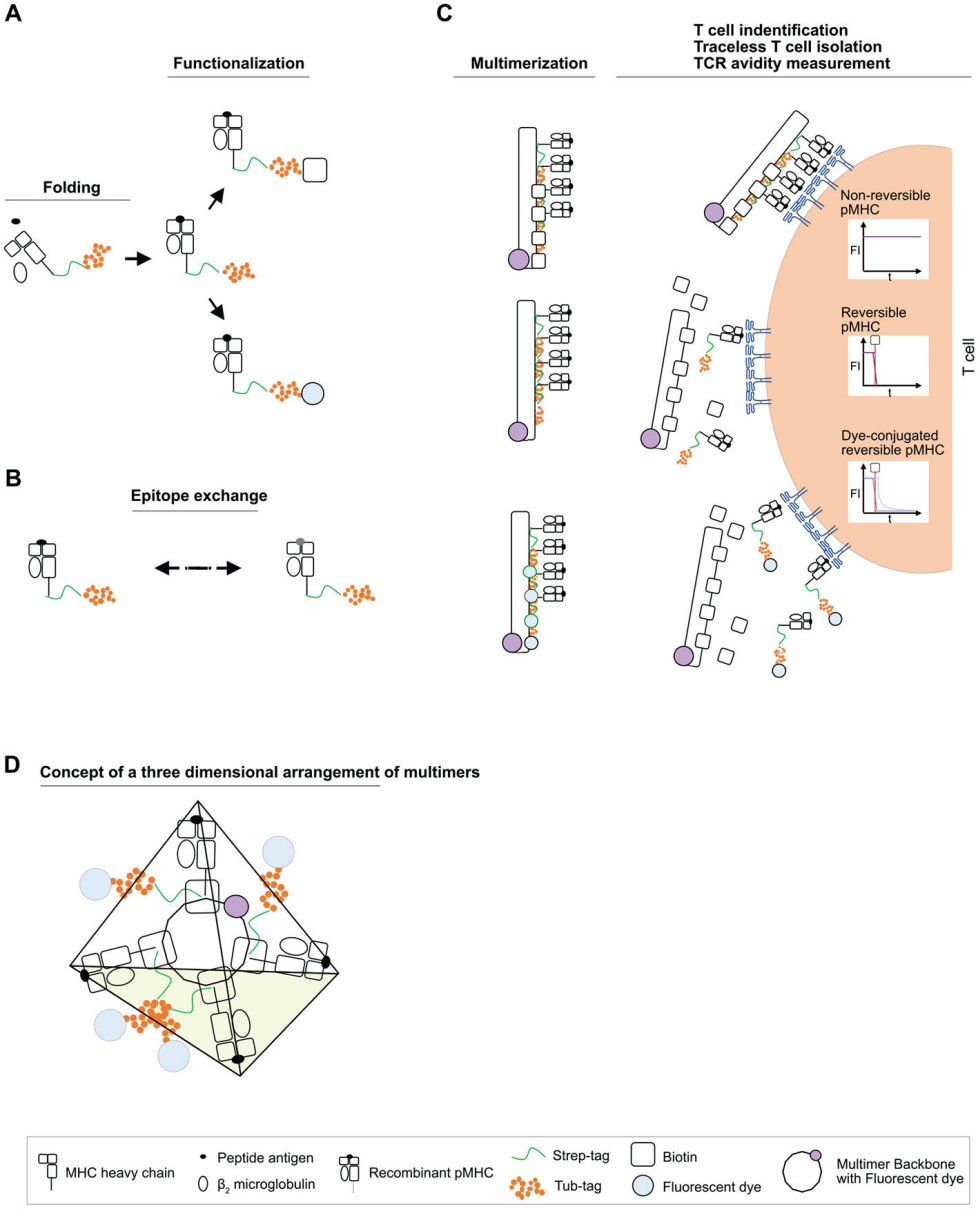
Production and usage of pMHC multimers (Originally published in The Journal of Immunology [790]). (A) pMHC monomer generation through folding and functionalization; (B) Epitope exchange technologies enable high-throughput generation of pMHC complexes for different Ag-specificities; (C) Different usage of nonreversible, reversible, and dye-conjugated reversible pMHC multimers; (D) The steric structure of multimers in a three dimensional space forms tetrahedron like structures limiting the binding layer to three pMHC molecules at once.

Reagents with different degrees of multimerization have been developed, as multimerization seemed to be relevant for stable and Ag-specific binding. Surprisingly, a direct comparison of MHC tetramers, pentamers, dextramers, octamers, and higher polymerization reagents has failed to show significantly improving binding properties with increasing degrees of multimerization [788]. It seems that an avidity gain with MHC trimers represents the crucial threshold to result in stable MHC multimer staining for most TCRs. This interpretation was based on the finding that also in conventional PE-conjugated MHC “tetramers,” three of the four MHC molecules simultaneously take part in binding to surface-expressed TCRs, although they stain polyclonal T cell populations effectively with high staining intensity (Fig. 76D) [789].

#### 3.3 Step-by-step sample preparation

MHC tetramers are based on multimerization with biotinylated ligands and avidin/streptavidin (Figure 76C, “non-reversible pMHC”). Conjugation with fluorochromes allows usage in flow cytometry-based applications and conjugation with paramagnetic particles promotes combination with magnetic purification technologies [791, 792]. However, binding of TCR ligands can lead to T cell stimulation/ activation and labeling-reagent internalization, as well as apoptosis and cell death [793-795]. Therefore, the reversible MHC Streptamer technology was developed, allowing removal of staining reagents from the cell surface after their application (Figure 76C, “reversible pMHC”) [796,797]. This is achieved by targeted disruption of multimer complexes, leaving only MHC monomers that rapidly dissociate from the cell surface. With directly fluorochrome-labeled MHC molecules, the dissociation can be precisely measured and serves as an important parameter for TCR avidity (Figure 76C, “dye-conjugated reversible pMHC”) [758,798]. Reversible staining has been further transferred to low-affinity Ab-derived Fab fragments (Fab Streptamer), extending the applicability of this labeling technology to virtually any surface Ag [799].

A large spectrum of MHC reagents is commercially available for the analysis of Ag-specific CD8^+^ T cells. Assembly of pMHC monomers requires folding of MHC heavy chain and β2 microglobulin in the presence of the antigenic peptide (Figure 76A). For downstream biotinylation or fluorophore-conjugation, pMHC monomers need to carry a functionalization site. An Avi-tag, for example, enables BirA-mediated biotinylation for pMHC multimerization on a streptavidin backbone (as is the case for classical tetramers) [760]. Furthermore, solvent-exposed cysteine residues have been used for fluorophore conjugation using maleimid chemistry [798, 800]. Reversibility of Streptamers (Figure 76C, “reversible pMHC”) is achieved through a Strep-tag, which allows stable multimerization on a streptactin (rather than streptavidin) backbone in a biotin-free manner [796]. Due to the higher binding affinity of d-biotin to the strep-tag, this multimeric complex can be disrupted through addition of d-biotin. As the affinity of monomeric pMHC complexes to the TCR is not high enough for stable binding, pMHC monomers consequently dissociate from the TCR (Figure 76C, “reversible pMHC” and “dye-conjugated reversible pMHC”).

In the past, different pMHC production strategies were necessary to generate the pMHC reagent (reversible/ nonreversible; probe-conjugated/unconjugated) desired for a specific application. In order to streamline and standardize the production process, the group of Prof. Dirk Busch at the TU Munich has developed the so-called “FLEXamer technology,” which allows flexible generation of pMHC reagents from a single precursor pMHC protein [790]. These FLEXamers possess a dual-tag consisting of a Strep-tag for reversibility and a Tub-tag for versatile functionalization with biotin, fluorophores, or other probes such as DNA oligos [772] (Figure 76A). Flow cytometric cell sorting of Ag-specific T cells stained with DNA-conjugated pMHC multimers (typically, these reagents are conjugated with a fluorophore, e.g., PE, in addition to the DNA barcode) enables extremely multiplexed analyses of different Ag specificities [772], and provides a very powerful analytical tool through further combinatorial detection of cellular transcriptome and TCR sequence [801].

In order to enable versatility also on the epitope level, a technology based on UV light-cleavable surrogate peptides has been developed (for more information also see Chapter V Section 17.2.2 UV light-mediated peptide exchange method in [22]) [762], but also dipeptides can be used for this purpose [766] (Figure 76B). Furthermore, multiplexed staining of samples with different fluorescence conjugated MHC multimers is possible and promotes simultaneous analysis or sorting for multiple epitope specificities (for more information also see Chapter V Section 17.5 Functional readouts) [768, 770]. Combinatorial MHC multimer staining can also be used not only to combine and distinguish large numbers of different MHC molecules within the same sample, but also to increase staining sensitivity for the detection of rare cell populations. Cell incubation with two MHC multimers, which are specific for the same Ag but are conjugated to different fluorophores, results in double-staining of Ag-specific T-cell populations. This approach significantly reduces background staining (for more information also see Chapter V Section 17.5 Functional read-outs in [22]), which is fundamentally important to identify rare cell populations.

**Figure 77.**
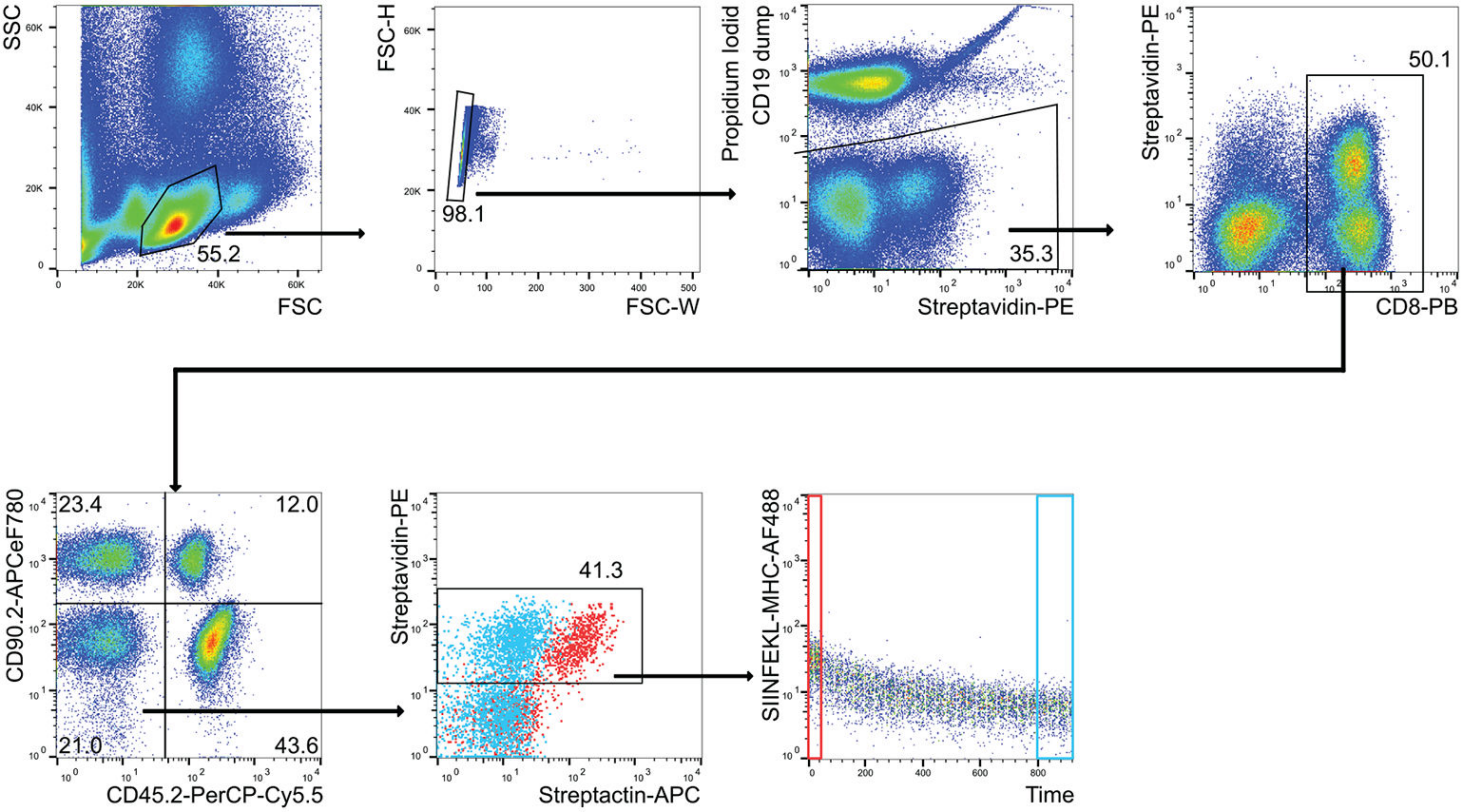
Versatile analysis of a murine H2-k^b^/SIINFEKL-specific T cell population. Double staining with nonreversible pMHC multimerized with streptavidin-PE (“Tetramer”) and reversible pMHC multimerized with streptactin-APC (“Streptamer”) before (red) and after (blue) addition of D-biotin; dissociation of Alexa488-conjugated monomeric SIINFEKL-pMHC molecules over time after addition of D-biotin (outside red box); pregating on lymphocytes, singlets, living CD19^−^, CD8^+^ CD45.2 and CD90.2-congenic coded T cells; gate of SIINFEKL-MHC-A488 additionally pregated on streptavidin-PE^+^ T cells.

The pMHC multimer stainings shown in Figure 77 summarize many of the above-introduced aspects. Figure 77 shows enhanced specificity through the use of two pMHC multimers, with the same pMHC but backbones with different fluorophores. The Ag-specific T cell population in Figure 77 was stained with a nonreversible pMHC multimerized with streptavidin-PE and a reversible (“Streptamer”) pMHC multimerized on streptactin-APC. After the addition of d-biotin only the biotinylated pMHC multimer staining prevails (Figure 77), demonstrating reversibility of Streptamer stainings. The breakup of Streptamer pMHC complexes is followed by dissociation of pMHC monomer from the TCR. Fluorophore conjugation of pMHC monomers thereby allows tracking of dissociation kinetics, and quantification of TCR-pMHC koff-rates (Figure 77). Continuous tracking of the dissociating pMHC monomers can still be linked to the Ag-specific population through gating on the population positive for the nonreversible pMHC. This emphasizes that not only the versatile nature of the different pMHC constructs themselves, but also their combinatorial usage, have made them become indispensable tools for in-depth T cell characterization.

Co-receptor (CD8 or CD4) interaction is often required for stable binding of MHC multimers. Therefore, parallel surface staining for CD8 or CD4 has to be controlled carefully to avoid artifacts by blocking (or sometimes even enhancement) of co-receptor binding. In order to control this problem, most staining protocols are based on an incubation period with MHC multimers alone before Ab reagents for co-receptors are added. An initial incubation with MHC multimer reagent alone for 25 min, followed by the addition of co-staining mAbs for further 20 min, has proven to be applicable to most MHC multimers in practice. In particular, when using PE-conjugated MHC multimers, background staining — especially coming from B cells and dead cells — can complicate the analysis. Therefore, implementation of a CD19 dump channel and live/ dead discrimination has become standard for most MHC multimer staining protocols. By using covalently linkable DNA staining probes (such as ethidium monoazide bromide (EMA)), it is also possible to combine live/ dead discrimination with cell fixation [802].

Optimal MHC multimer concentrations have to be determined for each batch by using positive and negative controls, as done for all other cellular labels used in flow cytometry. Besides reagent concentration, the duration of incubation time and staining temperature are crucial parameters for MHC multimer labeling. Since this technology relies on binding of the natural TCR ligand to the cell surface, at higher temperatures (above 10–15°C), signaling events and potential cell changes (e.g., up-or downregulation of cell surface markers, activation-induced cell death) can occur. Therefore, whenever possible, MHC class I multimer staining should be performed at low temperatures, i.e., 4°C. For reversible MHC multimer staining, cell labeling/sorting at low temperatures is essential, as reagent internalization would negatively interfere with its subsequent removal. In contrast, for most of the currently available MHC class II multimers, successful Ag-specific cell labeling is only possible at higher temperatures (usually at 37°C for 1 h), since signal accumulation by reagent internalization seems to be required in this case [803, 804].

In addition to conventional experimental controls (single color-, compensation-, and FMO-controls), biological controls for MHC multimer staining are recommended to determine the degree of background staining (e.g., by MHC mismatch controls). General considerations regarding minimal numbers of positive events that have to be acquired and optimal gating strategy (FSC/SSC, singlets, live/dead discrimination, co receptor/ multimer, etc.) are important to achieve meaningful and highly reproducible results. A detailed protocol for MHC multimer staining including some examples for staining artifacts is described in Cellular Diagnostics — Karger 2009 [805].

For more information, including instructions for the development of MHC class I reagents, please visit our website https://www.mikrobio.med.tum.de/de/seite/ag-busch.

#### 3.4 Materials

This is detailed in Table 67.

#### 3.5 Data analysis

For Data Analysis of Figure 77 FlowJo^™^ software version 10.4 (vendor: Becton Dickinson) was used. Hierarchical gating was used to further on analyze cells of interest starting with a separation of lymphocytes by discerning them from other cell types via morphological parameters FSC/SSC (both on Height). Subsequently lymphocyte singlets were gated by displaying FSC-W against FSC-H signals. A dotplot using Streptavidin-PE against CD19 PEDazzle594 identified both, the CD19 dump channel as well as dead cells via PI staining (diagonal in both channels). In addition, by gating very strictly only on dump negative, living and PE positive cells wrongly positive labeled Streptavidin^+^ and CD19^+^ cells were excluded. In the following plot only Pacific Blue CD8^+^ cells regardless of the Streptavidin-PE were gated. A quadrant gate for two congenic marker CD45.2 PerCP-Cy5.5 and CD90.2 APCeF780 identified four different CD8 populations from pooled mice. The population negative for the two congenic marker was finally analyzed by displaying the SIINFEKL double multimer staining with the reversible Streptactin-APC against an irreversible Streptavidin-PE. That gate was colored simulating an overlay of two populations visible in a subsequent gate visualizing the dissociation of monomeric SIINFEKL-MHC-AF488 against time after the addition of D-Biotin. The red population at early time points is still stained multimer double positive with Streptactin and Streptavidin, while over time the blue late population lost the reversible Streptactin-APC stain but retains the irreversible Streptavidin-PE signal.

#### 3.6 Pitfalls/Top Tricks

This is detailed in Table 68.

#### 4 Identification of SARS-CoV-2 specific human CD4^+^ T cells by 8-color flow cytometry

##### 4.1 Overview

The aim of this section is to describe a method to identify SARS-CoV-2 specific T cells in the circulation of COVID-19 patients or Sars-CoV-2 vaccinated individuals.

#### 4.2 Introduction

CD4^+^ T cells are crucial components of the immune system, given their role in orchestrating the activities of both innate and adaptive immune cells with the aim of eliminating the invading pathogen. Following Ag recognition in the lymph nodes, naïve CD4^+^ T cells proliferate and acquire effector functions (i.e., cytokine production), then egress into the circulation to reach peripheral tissues to fight the invading pathogen. Once the pathogen has been eliminated, a pool of memory CD4^+^ T cells survives and represents the subset of memory cells that will confer protection in case of secondary exposure to the same pathogen [806]. CD4^+^ T cells have a primary role in the context of SARS-CoV-2 infection [807]. The frequency of circulating CD4^+^ T cells correlates with the severity of the disease and with clinical conditions [113, 808-810] in the acute phase of the disease. Moreover, SARS-CoV-2 specific memory CD4^+^ T cells have also been detected in the circulation several months after recovery from the infection [741, 811]. Finally, CD4^+^ T cells specific for Spike protein of SARS-CoV-2 have been observed following mRNA vaccination [812]. Defining the presence of circulating virus-specific memory T cells, as well as Abs, is crucial for research but also for diagnostic and epidemiological purposes. This section describes how to adapt a well-established technology for the flow cytometric identification of Ag-specific T cells to the characterization of SARS-CoV-2 reactive CD4^+^ T cells following stimulation with Spike (S), Membrane (M), or Nucleoprotein (N) peptide pools.

#### 4.3 Step-by-step sample preparation

##### 4.3.1 Starting material.

This protocol has been tested on peripheral blood mononuclear cells (PBMCs), although in principle it may be applied also on whole blood. Fresh cells are preferred but cryopreserved cells can be also used.

##### 4.3.2 Cell stimulation.

Isolate human PBMCs from whole blood via density gradient centrifugation.

Resuspend cells in RPMI 1640 supplemented with 5% human AB serum. Final cell concentration must be 10×10^6^/ml.

Plate 1.5 × 10^6^ cell/well in 96-well plates, flat bottom.

Add the specific peptide pool (0.6 nmol peptide/ml).

Always include a positive control (SEB 1μg/ml) and a negative control (medium alone without stimulus). Incubate for 2 hours at 37°C with 5% CO2.

Add Brefeldin A (5μg/mL) and then incubate for additional 4 hours.

###### NB:

This protocol has been set up on subjects who recovered from SARS-CoV-2 infection no longer than 6 months. For longer periods from infection or vaccination, when a lower frequency of circulating specific CD4^+^ T cells is expected, it is possible to increase the total amount of stimulated cells as well as of recorded cells. This is also true when studying immunocompromised patients who may not develop vigorous immune responses.

##### 4.3.3 Cell staining.

At the end of incubation, collect cells from each well and transfer in tubes for staining with Fixable Viability Stain 780.

Incubate 15 min at room temperature in the dark.

Wash cells with PBS and then centrifuge at 300 × *g* for 7 min.

Aspirate the supernatant and fix cells with 2% formaldehyde for 15 min at room temperature.

Wash cells with PBS-BSA buffer and centrifuge at 300 × *g* for 7 min.

Aspirate the supernatant and stain for 15 min at room temperature with anti-TNFα FITC, anti-CD154 PE, anti-CD3 PerCP, anti-CD4 PE Cy7, anti-CD8 SB600, anti-IL-2 APC, anti-IFNγ PacificBlue in presence of 0.5% saponin.

Wash cells with PBS-BSA buffer with 0,5% saponin and centrifuge at 300 × *g* for 7 min.

Aspirate the supernatant, resuspend in PBS-BSA buffer, and measure by flow cytometry.

#### 4.4 Materials

Culture Medium: RPMI 1640 supplemented with 5% human AB serum. Do not use fetal cow serum or bovine serum to avoid unspecific stimulation.

PBS-BSA Buffer: phosphate-buffered saline (PBS), pH 7.2. PBS supplemented with 0.5% bovine serum albumin (BSA).

Peptide pools covering SARS-CoV-2 S, N and/or M protein (Miltenyi Biotech).

Staphylococcal enterotoxin B (SEB, Sigma-Aldrich) as positive control.

Brefeldin A (BFA), saponin, and formaldehyde (Sigma-Aldrich).

Fixable Viability Stain 780 from BDBiosciences or similar reagent able to identify and exclude dead cells (it possible to choose different fluorescence or different company according to your own needs).

Fluorochrome-conjugated mAbs for surface and intracellular markers: anti-TNFα FITC (BDBiosciences), anti-CD154 PE (BDBiosciences), anti-CD3 PerCP (BDBiosciences), anti-CD4 PE Cy7 (BDBiosciences), anti-CD8 SB600 (eBioscience), anti-IL2 APC (BDBiosciences), anti-IFNγ PacificBlue (Biolegend). Otherwise mABs conjugated with different fluorochromes or derived from different company could be used based on instrument setting and on your own needs.

Moreover, to improve the characterization of Ag-specific T cells is possible to insert in the staining panel additional surface markers (i.e., CD137, CD134, CD69) or intracellular cytokines (i.e., IL-10, IL-17, GMCSF).

#### 4.5 Data analysis

**Figure 78.**
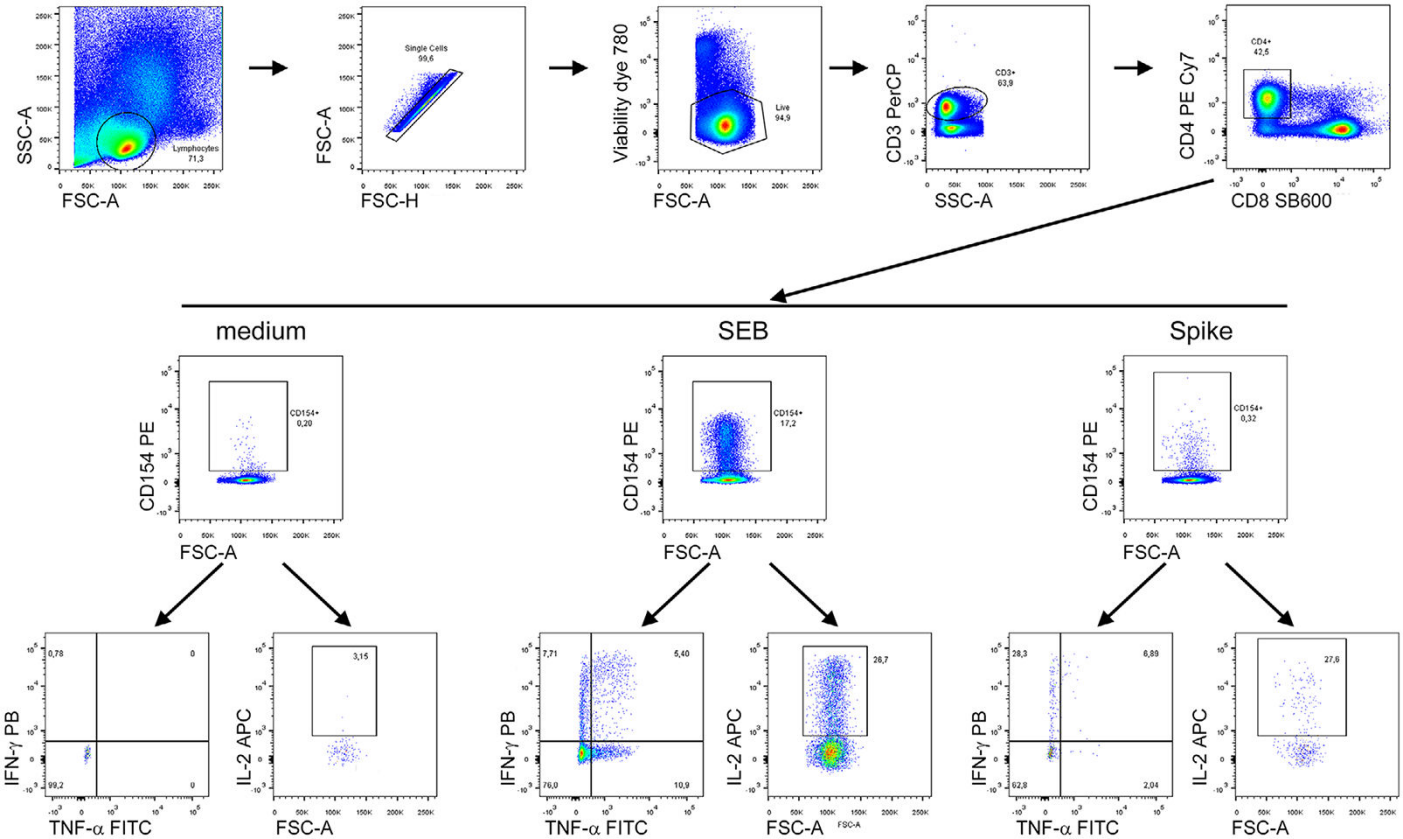
Gating strategy to identify Ag specific human CD4^+^ T cells. Human lymphocytes were gated based on physical parameters (FSC-SSC), then doublets were excluded using FSC-A and FSC-A parameters. Dead cells were excluded using viability stain 780. T cells were identified as CD3^+^ and among these CD4^+^ T cells were selected. Antigen-specific CD4^+^T cells were identified as cells expressing CD154; CD4^+^CD154^+^ T cells were then evaluated for IFN-γ, TNF-α, and IL-2 expression. Representative plots of PBMCs unstimulated (medium, negative control) stimulated with SEB (positive control) or Spike peptide pools, from a COVID-19 recovered subject evaluated at 7 days from SARS-CoV-2 vaccination, are shown.

Data analysis requires the exclusion of doublets and death cells using a live-dead marker. This is especially true if using cryopreserved cells instead of freshly isolated. As shown in Figure 78, lineage markers allow the identification of the population of interest for the identification of cytokine-producing cells.

#### 4.6 Pitfalls

Given that Ag-specific CD4 T cells are rare, it is recommended to acquire at least 100000 events in the CD4 gate. This may be tricky when working with samples from immunocompromised patients. If low numbers of specific CD4^+^ T cells are expected (immunocompromised patients, long time since infection/vaccination) the number of analyzed cells should increase up to 300000.

#### 4.7 Top tricks

This assay can be used to evaluate the response to peptide pools covering individual SARS-CoV-2 protein (N, M, S). Alternatively, peptide pools can be mixed together to identify the percentage of CD4^+^ T cells reactive to all these proteins.

#### 4.8 Clinical relevance statement

The assay shown in this section is applicable for understanding the magnitude of cellular immune response to SARS-CoV-2 in COVID-19 patients in the acute phase or in the memory phase. It can also be used to define the response to vaccination procedures (see Figure 78).

### 5 Adoptive T cell transfers as a read-out for Ag-specific immune responses in mice

#### 5.1 Overview

For over three decades now, adoptive transfer of TCR-transgenic (TCRtg) and BCR-transgenic (BCRtg) cells, followed by challenge with cognate Ag in various experimental settings such as immunization, infection, autoimmunity, and tumors, has proven to be an elegant tool to study Ag-specific immune responses *in vivo.* These experiments have generated a wealth of information on the activation requirements, kinetics, magnitude, and effector as well as memory responses of T and B cells and adoptive transfer experiments continue fueling research in these areas. Here, we describe critical parameters for performing adoptive transfer experiments with TCRtg cells and discuss advantages and disadvantages of this approach in regards to study design and data interpretation. It should be noted that the same procedures can also be used for adoptive transfer of BCR-transgenic cells (See section VI.4 Adoptive B cell transfers as a read-out for Ag-specific immune responses in mice).

#### 5.2 Introduction

Experimental immunization or infection of mice is frequently used to study immune responses *in vivo.* Using various activation marker combinations, polyclonal T and B cell responses can be easily analyzed by flow cytometry. Activated T cells can be identified in mice by staining for activation markers such as CD69 or CD44 (see section IV.1 Antigen-specific T cell cytometry: Functional read-outs). However, this generally does not provide information on the differentiation history or the Ag specificity of these cells. For the detection of Ag-specific CD8^+^ or CD4^+^ T cells in bulk cell populations, MHC multimers may be used in humans and mice (see section IV.3 Antigen-specific T cell cytometry: MHC multimers). While each multimer covers one antigenic specificity, thereby allowing quantification of Ag-specific cells, functional and fate-mapping assays are rather limited. Complementary to this approach, TCRtg T cells have been widely used for studying Ag-specific T cell responses in various *in vivo* settings. The advantage of using TCRtg cells is the known specificity of these cells and their suitability for adoptive transfer experiments. Various TCRtg mouse lines have been described in the literature. Prominent examples for CD8^+^ T cells include P14, which are specific for LCMV GP_33-41_ peptide [813], or OT-I, which are specific for OVA_257-264_ peptide [814]. Examples for CD4^+^ T cells include SMARTA, which are specific for LCMV GP_61-80_ peptide [815], and OT-II, which are specific for OVA_323-339_ peptide [816]. All these lines are on the C57BL/6 background. DO11.10 mice, which are on the BALB/c background, carry a TCRtg that also recognizes OVA_323-339_ peptide [817]. For this mouse strain, a clonotypic Ab has been generated that allows detecting DO11.10 TCRtg cells without the need of additional markers such as congenes or fluorescent reporter alleles. TCRtg mice can also be used for inducing autoimmunity. For example, adoptively transferred P14 TCRtg cells can kill genetically engineered LCMV GP-expressing beta cells in the pancreas, thus causing diabetes [818]. Another example are 2D2 mice, in which 95% of CD4^+^ T cells carry a TCR specific for MOG_35-55_ peptide [819]. These cells can be used to track autoantigen-specific T helper cell responses in the CNS after MOG/CFA/PTX-induced active EAE. 2D2 cells can also be activated and transferred into secondary hosts, where they are sufficient to induce full-blown disease (passive EAE). While TCRtg mice usually harbor only very few Treg cells, if any, polyclonal Foxp3 reporter mice such as Foxp3-GFP may be used instead for isolation of GFP^+^ polyclonal Treg and Tfr cells with unknown specificity for adoptive transfer experiments.

To limit the precursor frequencies of Ag-specific TCRtg cells in adoptive transfer experiments as much as possible to physiological levels, low numbers of purified naïve TCRtg cells should be transferred into wild-type recipients. For functional questions, these donor cells can be derived from control or knock-out backgrounds and are then being compared in separate or competitive adoptive transfers into wild-type mice. Alternatively, for examination of extrinsic factors important for T cell biology, TCRtg cells can be transferred into hosts that lack certain genes (i.e., knock-out mice). In order to distinguish the transferred cells from host lymphocytes, it is advisable to intercross the TCRtg lines to different congenic alleles. Since wild-type C57BL/6 mice are CD45.2, TCRtg cells that carry one or two alleles of the congene CD45.1 can be easily identified by flow cytometry or immunofluorescence microscopy by staining with fluorescencelabeled Abs against CD45.1 and CD45.2. Using combinations of CD45.1 and CD45.1/2, it is even possible to perform competitive co-transfers into CD45.2 wildtype C57BL/6 mice, e.g., comparing control and knockout TCRtg cells within the same host. For T cells, combinations of the congenic markers Thy1.2 (CD90.2, expressed by wildtype C57BL/6 mouse T cells) and Thy1.1 (CD90.1) have been regularly used as an alternative to the CD45.2/CD45.1 system. While CD45 is expressed by all hematopoietic cells, Thy1 is mainly expressed by T cells and innate lymphoid cells, but not by B cells. By intercrossing OT-I TCRtg mice that carry various homo- or heterozygous combinations of CD45 and Thy1 congenes, a matrix of up to eight TCR-identical congenic combinations has been previously used in adoptive single cell transfers into wildtype hosts to dissect CD8^+^ T cell fates *in vivo* [820]. Another possibility is to cross TCRtg mouse lines to fluorescent reporter alleles, e.g., containing GFP, which can also be used for intravital two-photon microscopy studies. For short-term assays or for the assessment of cell proliferation *in vivo* for up to 3 to 4 days, naïve TCRtg cells can be labeled with CFSE, CellTrace^™^ Violet (CTV), or similar fluorescent dyes prior to adoptive transfer (see Chapter V, Section 6 in [22]).

**Figure 79.**
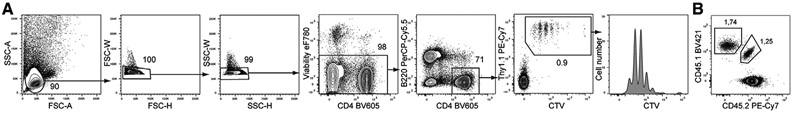
Use of congenic markers in adoptive transfer experiments in mice. (A) Gating strategy for the identification of CTV-labeled, Thy1.1^+^ OT-II cells by flow cytometry. Wildtype C57BL/6 mice were injected i.v. with 5x10^5^ naïve OT-II TCRtg CD4^+^ T cells. In this setting, such high numbers of naïve OT-II TCRtg CD4^+^ T cells (in contrast to classical adoptive transfer experiments with typically less than 1-5 × 10^4^ naïve OT-II cells per mouse) are required for recovering enough events for proper cell division analyses. One day later, recipient mice were immunized with 5 μg OVA and 2 μg LPS in the hock. Three and a half days later, draining popliteal lymph nodes were dissected, single-cell suspensions were prepared and the cell surface was stained with appropriate combinations of fluorescently labeled monoclonal Abs. Thereafter, samples were fixed and stained with the Foxp3 transcription factor staining set and samples were then acquired on a BD LSRFortessa. Single lymphocytes were first gated based on FSC/SSC characteristics. CD4^+^ T cells were further gated to exclude dead cells and B cells, and finally with the congenic marker Thy1.1 and CTV to differentiate transferred OT-II cells from endogenous (Thy1.1^−^) T cells of the recipient. The CTV profile of the identified OT-II cells is shown in the histogram. To reduce the overall size of the acquisition data file, 50,000 lymphocytes were acquired first and then only TCRtg Thy1.1^+^ CD4^+^ T cells were appended to the file. (B) Competitive co-transfer of CD45.1^+^ and CD45.1/2 double-positive CD4^+^ T cells into wild-type C57BL/6 recipients. Equal numbers of CD45.1^+^ and CD45.1/2 double-positive naïve OT-II TCRtg CD4^+^ T cells of two different genotypes (1×10^4^ cells each) were injected i.v. into wild-type C57BL/6 recipient mice. It is recommended to check for the correct ratio of transferred cells by flow cytometry, e.g., by analyzing a left-over aliquot of the injected cell suspension on a flow cytometer. One day after adoptive transfer, recipient mice were immunized i.p. with 100μg NP-OVA in alum. Seven days later, spleens were dissected, single-cell suspensions were prepared and stained with appropriate combinations of fluorescently labeled mAbs. The samples were acquired on a BD LSRFortessa and gated on live CD4^+^ T-cells. Staining for CD45.2 versus CD45.1 allows distinguishing the two transferred TCRtg cell populations from the CD45.2^+^CD45.1^−^ host T cells.

The following protocol provides a framework for adoptive transfer experiments with CD4^+^ and CD8^+^ TCRtg T cells. The same protocol can also be adopted for adoptive transfer experiments with BCRtg cells (see section VI.4 Adoptive B cell transfers as a readout for Ag-specific immune responses). The protocol can be easily modified and tailored to the specific question of interest. Examples of how this protocol can be used for the assessment of TCRtg cell proliferation *in vivo* or for adoptive co-transfer of two different TCRtg cell populations into the same host are shown in Figure 79.

#### 5.3 Step-by-step sample preparation

1. Prepare single-cell suspensions from pooled spleen and lymph nodes of TCRtg donor mice of interest (see Chapter III, Section 3 in ref. [22]).
2. Enrich naïve T cells with magnetic beads (preferentially by negative selection) (see Chapter IV, Section 1 and 2 in ref. [22]) and/or by cell sorting (see Chapter IV, Section 3 in ref. [22]). If the scope of the study is to analyze the fate of already differentiated cells (in vivo or in vitro generated), these cells may also be used for adoptive transfer experiments.
3. To track proliferation and expression kinetics of transferred cells, they can be optionally labeled with a cell proliferation dye (e.g., CFSE or CTV) prior to adoptive transfer (see Chapter V, Section 6 in ref. [22] and Figure 79).
4. Inject TCRtg cells into host mice (e.g., wild-type C57BL/6), usually per i.v. route. Keeping in mind to aim for the lowest feasible number of cells to be injected, adjust the required cell number to the characteristics of the specific TCRtg, to the immunization or infection model used, and to the intended readout (e.g., short term vs. long-term) as the number of endogenous and transferred cells can strongly influence the outcome of the experiment [821, 822]. While typical cell numbers will range from hundreds to hundreds of thousands, even as few as one or ten transferred cells may be sufficient for certain experimental settings [747, 823]
5. Before challenging the transferred cells in the new host with the cognate Ag, allow the transferred cells to equilibrate in the host for a few hours to days.
6. Immunize or infect the recipient mice with the cognate Ag. For protein and peptide immunizations, it is usually required to mix the Ag with an adjuvant to elicit a strong response.
7. Analyze the adoptively transferred cells by flow cytometry, taking advantage of congenic markers or fluorescent labels that allow distinguishing the transferred cells from the endogenous host cells (for examples, see Figure 79). To this end, prepare single-cell suspensions of secondary lymphoid tissues or other tissues of interest (see Chapter III, Section 3 in ref. [22]).) and stain the cells with appropriate combinations of fluorescence-labeled Abs for subsequent acquisition on a flow cytometer or cell sorter.

#### 5.4 Materials

For detailed materials see Chapter III, Section 3; Chapter IV; and Chapter V, Section 6 in [22]). In brief, the following reagents and tools can be used:

- TCR-tg donor mouse lines as well as appropriate recipient mouse lines that carry appropriate combinations of congenic markers
- Appropriate Ags/adjuvants or infectious agents for immunization or infection of recipient mice
- Glass slides with frosted ends for tissue disruption
- flow cytometry buffer: PBS, 2% fetal calf serum (FCS), 2mM EDTA, 0.05% sodium azide (NaN_3_); do not add sodium azide to the sorting buffer used for pre-enrichment before adoptive transfers
- To block Fc receptors, add Purified anti-mouse CD16/32 Anti-body (clone 93 or 2.4G2) to flow cytometry buffer
- EasySep^™^ Mouse Naïve CD4^+^ T Cell Isolation Kit (Stemcell Technologies, 19765) or EasySep^™^ Mouse Naïve CD8^+^ T Cell Isolation Kit (Stemcell Technologies, 19858) or similar for negative selection of naïve CD4^+^ or CD8^+^ T cells, respectively
- CellTrace^™^ Violet Cell Proliferation Kit (Life Technologies, C34557) or similar for assessment of TCRtg cell proliferation *in vivo*
- Antibodies against congenic markers, e.g., anti-CD45.1 (clone A20) and/or anti-CD45.2 (clone 104), which label all hematopoietic cells except mature erythrocytes and platelets. Alternatively, anti-mouse CD90.1/Thy1.1 (clone HIS51 or OX-7), and/or anti-mouse CD90.2/Thy1.2 (clone 53-2.1 or 30-H12) can be used for T cell experiments
- Foxp3 transcription factor staining set (eBioscience, 00-5523-00) or similar for transcription factor staining
- Viability dye (e.g., Fixable Viability Dye eFluor^™^ 780, eBioscience, 65-0865-14) for dead cell exclusion
- Flowcytometer for the acquisition of samples (e.g., BD LSR-Fortessa)

#### 5.5 Data analysis

Flowjo (BD) or alternative software can be used to compensate and analyze the flow cytometry data. Firstly, using acquired single-color stains, a compensation matrix is generated and applied to all samples. Next, FSC/SSC combinations are used to gate on lymphocytes and to exclude doublets (see gating strategy in Figure 79). As dead cells tend to bind Abs unspecifically and/or exhibit autofluorescence, it is common practice to exclude dead cells using a viability dye. In order to reach the cells of interest, gate on their specific marker (e.g., CD3, CD4, and/or CD8 in the case of T cells), while excluding other lineages (e.g., by gating out B cells, which are positive for CD19 and B220). Finally, congenic markers and combinations thereof (i.e., CD45.1, CD45.2, Thy1.1, Thy1.2) and/or fluorescent labels (e.g., CFSE, CTV, GFP) can be used to differentiate the adoptively transferred TCRtg (e.g., control vs. KO) and/or BCRtg cells from each other and from the host.

#### 5.6 Pitfalls

While adoptive transfer experiments with TCRtg and BCRtg cells represent an elegant and powerful approach to study T and B cell responses *in vivo,* several important points need to be considered for generating valid and reproducible results:

##### 5.6.1 Purity of adoptively transferred cells.

Most often, naïve TCRtg or BCRtg cells are being used for adoptive transfer experiments. To purify naïve T cells from recipients, it is advisable to enrich naïve CD4^+^ or CD8^+^ T cells with magnetic bead-coupled Abs, preferentially using negative enrichment that yields untouched cells for downstream applications (see Chapter IV in ref. [22]).). Alternatively, or in addition, naïve cells can be further purified using cell sorting. In the case of T cells, naïve CD4^+^ or CD8^+^ T cells can be sorted as CD44^int/low^CD62L^hi^ cells. CD25 can be included as well to exclude activated T cells and Treg cells among CD4^+^ cells. The TCR should not be stained directly (e.g., CD3ε), as this may crosslink the TCR and activate the cells. Untouched resting B cells can be efficiently enriched using CD43 magnetic beads.

##### 5.6.2 Precursor frequency.

It is highly advisable to transfer as few TCRtg or BCRtg cells as possible. Endogenous Ag-specific precursor frequencies are usually very low (in the range of tenth to hundreds of T cells per mouse) [821]. Since TCRtg or BCRtg mice harbor millions of cells specific for the same Ag, it is tempting to also transfer hundreds of thousands or millions of these cells. However, since all these cells would compete with each other in the new host for the specific Ag after infection or immunization, such high precursor frequencies are unphysiological and results of these experiments need to be interpreted with care [747]. In addition, while most transferred TCRtg cells will die and disappear during the transfer procedure, only a small percentage (often less than 10%) of transferred cells will be eventually “parked” in the host. Nevertheless, under certain conditions, it can be required to transfer higher cell numbers in order to recover enough cells for analyses, e.g., in the case of proliferation experiments using CFSE or CTV, in which it is often difficult to recover enough cells that are within the first cell division(s) [824].

##### 5.6.3 High-affinity TCRs and BCRs.

TCRtg and BCRtg cells often carry Ag receptors with very high affinities for the specific Ag, which may confound the conclusions derived from adoptive transfer experiments utilizing these cells. For example, the HEL-specific BCRs of MD4, SWHEL, and Hy10 BCRtg mouse lines bind HEL with extremely high affinity. To adapt for this problem, mutated HEL proteins and peptide sequences have been engineered that exhibit much lower binding affinities to these BCRs, thus providing a more physiological setting [825]. Alternatively, the HEL-related duck egg lysozyme, which exhibits lower binding affinity to these BCRs, has been used as well [826].

##### 5.6.4 Rejection of transferred cells.

Congenic markers or fluorescent proteins expressed by adoptively transferred cells can potentially facilitate rejection. While this may not be such a big issue for short-term experiments, long-term experiments require more careful planning and interpretation by taking this potential caveat into account. As an example, when transferred into CD45.2 hosts, heterozygous CD45.1/2 cells might be less prone to rejection than CD45.1 homozygous cells. The use of CD45.1/2 heterozygous hosts could provide an elegant solution to this problem, as CD45.1 and CD45.2 homozygous cells would be much less likely rejected in these mice. In addition, for critical issues, allelic marker combinations of CD45.1 and CD45.2 (or similar) may also be switched in complementary adoptive transfer experiments to test whether the same conclusions are reached. To further decrease the possibility of rejection, TCRtg and BCRtg mice should be bred on and/or regularly back-crossed to the same background strain of the host mice used in the adoptive transfer experiments. Another strategy for reducing the risk of GFP^+^ cells being rejected after adoptive transfer is the use of host mice that express GFP under an endogenous promotor that is not active in the same host cell type as the transferred cells, thus rendering these hosts tolerant towards GFP.

##### 5.6.5 Exclusion of dead and contaminating cells.

It is imperative to carefully exclude dead cells as well as “sticky” cells. To exclude dead cells, which often show autofluorescence and unspecific binding of fluorescently labeled Abs, a viability dye should be incorporated in the flow cytometry staining panel (see Chapter III, Section 4 in ref. [22].). Similarly, it is advantageous to block unspecific binding by preincubation with rat or mouse serum (according to the primary Abs used for flow cytometry) and Fc receptor blocking reagents. Finally, a dump channel may be incorporated to exclude cells that are “sticky” and/or may share marker expression with the cell type of interest. Typical target Ags that could be used in a dump channel are CD11c, CD19 (if T cells are the cells of interest) or CD3 (if B cells are the cells of interest), and other lineage-defining markers [827]. It is of importance to ensure that the Ag used in a dump channel is not expressed by the cells of interest though.

##### 5.6.6 TCRtg and BCRtg mice are often “leaky”.

This means that not all T and B cells are monoclonal and some polyclonal T cells have expanded that do not express the Ag-specific tg. The degree of T and B cells not carrying the TCRtg and BCRtg, respectively, varies considerably between the individual mouse lines. One possibility to generate true monoclonal TCRtg and BCRtg mice is to cross these mice onto Rag1- or Rag2-deficient backgrounds. This is particularly important if TCRtg cells will be transferred into lymphopenic hosts, e.g., Rag1, Rag2, TCRαβ, or CD3ε knockout mice, as naïve polyclonal T cells will undergo considerable homeostatic proliferation and may even cause disease, such as IBD after transfer of naïve polyclonal CD4^+^ T cells into Rag1 knockout mice.

#### 5.7 Top tricks

Pre-enrichment of target populations: Low numbers of transferred cells and/or poor expansion of these cells in the host may limit recovered cell numbers that can be analyzed by flow cytometry. To accommodate for this problem, pre-enrichment of the adoptively transferred cells may be used before acquisition on a flow cytometer/sorter. Similar to the techniques described in the “Purity of adoptively transferred cells” section above, congenic TCRtg and BCRtg cells can be pre-enriched from whole host spleen or lymph node tissues by labeling with magnetic beads coupled to monoclonal Abs against the respective congenic marker (e.g., CD45.1 or Thy1.1). Small bead sizes (e.g., Miltenyi MACS or Stemcell Technologies) should be preferred over big bead sizes (Thermo Dynabeads) for this positive selection approach. To avoid potential interference with subsequent staining steps with a similar FC Ab clone, fluorescence coupled primary Abs can be used, followed by anti-fluorochrome-directed magnetic beads (e.g., anti-CD45.1-FITC followed by anti-FITC magnetic beads). Interference may also be circumvented by implementing enrichment procedures that are based on negative selection.

### 6 Cytotoxicity

#### 6.1 Overview

Priming of naive pathogen- or tumor-reactive CD8^+^ T cells (Tn CD8) occurs in secondary lymphoid organs (SLOs), where they undergo clonal expansion and differentiate into effector CD8^+^ T (TE) lymphocytes. In the course of their functional maturation, CD8^+^ TE acquire the ability to leave SLOs, enter non-lymphoid organs (NLOs), produce inflammatory cytokines, and lyse target cells displaying cognate MHC class I-peptide complexes [828, 829]. Besides TE, immune activation also leads to the generation of long-lived memory T lymphocytes (TM). CD8^+^ TM can be found in SLOs and NLOs where they exert immediate effector functions upon secondary Ag contact [830, 831]. Peptide-specific target cell lysis is a cardinal feature of cytotoxic CD8^+^ TE/TM (CTLs) [831, 832] and its quantification is a valuable means to track CD8^+^ T cell responses. Here we review methods to quantify cytotoxic function *in vivo* and *ex vivo* and present exemplary data using an *in vivo* multiplex assay to simultaneously determine the cytotoxic activity and functional avidity of multiple murine virus-specific CTLs.

#### 6.2 Introduction

Traditionally, *in vitro* CTL assays relied on the detection of compounds released from dying target cells. For example, target cells loaded with radioactive sodium chromate lose their radioactive label as a result of CTL-mediated lysis. Hence, the amount of radioactivity in the supernatant of effector (CTL)/target cell co-cultures directly correlates with the lytic activity of the respective CTL population [833]. To achieve suitable effector-to-target cell (E:T) ratios of at least 50:1, high numbers of CTLs are required for this type of assay. This usually requires Ag-dependent CTL expansion *in vitro*, a process that may alter the composition and/or function of the starting CTL population.

In order to replace radioactive CTL assays, several flow cytometry-based techniques were established in the past years. Their major aim is to visualize the biochemical processes involved in CTL-mediated target cell lysis. CTLs induce target cell apoptosis via the Fas/Fas ligand pathway [834] or the release of cytotoxic granules containing perforin and granzymes [835]. Either pathway results in the activation of caspase-dependent target cell apoptosis. To visualize this process, cell-permeable fluorogenic caspase substrates were developed [836]. They consist of two fluorophores, which are linked by a caspase-sensitive peptide. Only upon caspase-dependent cleavage, these substrates become activated and can be detected by flow cytometry. Alternatively, target cell apoptosis can be visualized with the help of fluorochrome-labeled inhibitors of caspase (FLICA), which bind specifically to active caspases [837, 838]. Hence, in both cases, fluorescence intensities correlate with CTL-dependent target cell destruction. However, similar to the chromium release assay, relatively high E:T ratios are required for these experimental approaches.

A more sensitive assay relies on the co-incubation of CTLs with a mixture of target cells consisting of at least two different populations. For this so-called fluorometric assessment of T lymphocyte Ag-specific lysis (FATAL) assay [839], the first target cell population is loaded with the MHC I-restricted peptide of interest and stained with one dye (e.g., PKH-26). The second population is loaded with an irrelevant peptide, stained with a different dye (e.g., CFSE), and serves as negative control [839]. Different concentrations of the same dye can be used to stain both target cell populations, which are discriminated based on their differential fluorescence intensities. Alternatively, amine-reactive dyes such as Cell Tracer Violet (CTV) can be used, which are less prone to dye transfer between cells observed with lipophilic dyes. The extent of CTL activity is determined by the relative numeric decrease of labeled target cells loaded with the desired peptide over non-specific target cells after a period of time, usually 5h. Significant advantages of this assay are its high sensitivity and favorable signal-to-noise ratio due to negligible amounts of spontaneous tracer release, a common side effect of the chromium release assay. Due to these advantages, the FATAL assay is often well-suited to directly measure CTL function *ex vivo* without prior expansion and at comparably low E:T ratios.

Target cells may be immune (e.g., splenocytes) or somatic cells (e.g., epithelial cells or fibroblasts) to more closely resemble the physiological CTL targets. CTLs can be purified from any organ of interest, either lymphoid or non-lymphoid. Depending on the research question, purification of total CD8^+^ T cells or Ag-specific CD8^+^ T cells may be required. In the former case, the frequency of Ag-specific CTLs can be determined in parallel by MHC/peptide multimer staining to determine CTL frequencies and adjust E:T ratios for different tissue samples accordingly.

However, if the frequency of Ag-specific CD8^+^ T cells is very low, it may be necessary to enrich them prior to the cytotoxicity assay. In this case, it is not advisable to sort Ag-specific CD8^+^ T cells by means of TCR labeling (e.g., by MHC/peptide multimers) since this may alter their lytic function. If available, the use of congenically marked TCR-transgenic (TCR^tg^) CD8^+^ T cells might be useful to circumvent this problem. This allows their marker-based, TCR-independent enrichment prior to the *ex vivo* CTL assay. Hence, direct *ex vivo* CTL assays have several advantages: (i) they are very sensitive, (ii) CTLs may be isolated from any organ, (iii) the type of target cell may be adapted to the nature of the experiment, (iv) E:T ratios can be adjusted to compare different samples. However, it is important to note that the tissue microenvironment affects CTL activity [840]. Hence, the lytic potential of tissue resident CTLs may differ from those purified for *ex vivo* CTL assays.

To circumvent this problem CTL activity can be measured *in vivo* [832, 841, 842]. Again, at least two target cell populations are required. One is labeled with the peptide of interest and, e.g., a high concentration of a suitable dye such as CFSE (CFSE^hi^ population). The control population is loaded with an irrelevant peptide and a tenfold lower concentration of CFSE (CFSE^lo^ population). Equal numbers of CFSE^hi^ and CFSE^lo^ cells are co-injected into effector mice. After 3–18 h, SLOs can be isolated to analyze single-cell suspensions by flow cytometry. Similar to the direct *ex vivo* assay described above, the relative loss of CFSE^hi^ target cells over CFSE^lo^ cells indicate the extent of CTL-mediated lysis. This method provides the most sensitive and physiological assessment of CTL activity [22].

**Figure 80.**
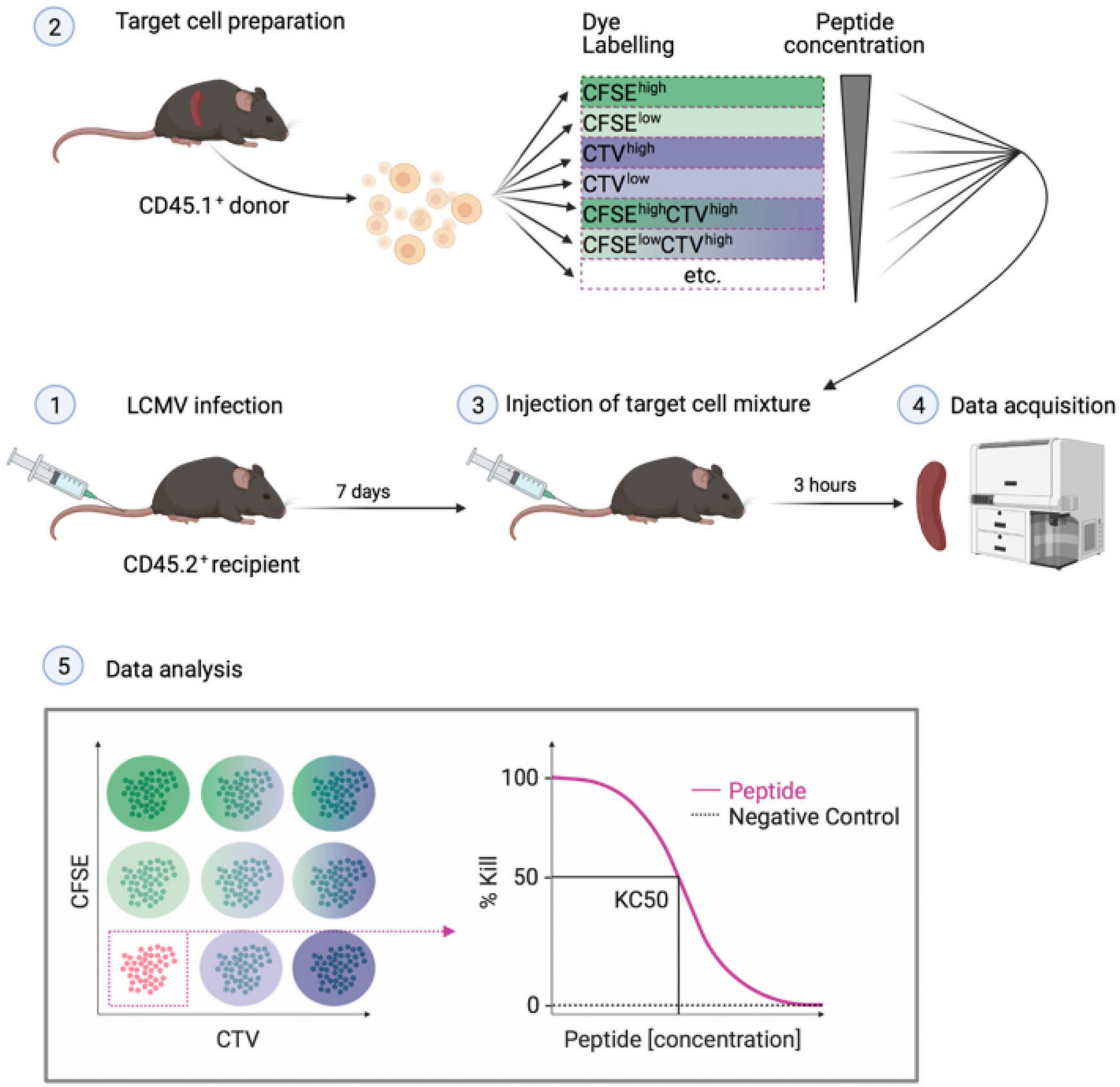
Experimental procedure for a 2-color “*in vivo* Multiplexed Antigen-Specific Cytotoxicity Assay” (iMASCA) in the mouse with nine target cell populations. Seven days after LCMV infection of CD45.2^+^ mice (1), target cells are isolated from the spleen of CD45.1^+^ donor mice and labeled with high or low concentrations of CFSE or Cell Tracker Violet (CTV) or combinations of both (2). Subsequently, nine differentially labeled target cell populations are loaded with distinct concentrations of the respective peptide, washed and mixed prior to injection into LCMV-infected recipient mice (3). Three hours later, recipient splenocytes are analyzed by flow cytometry (4). Relative target cell frequencies are determined, and peptide-specific lysis is calculated for each target cell population (5).

*In vivo* CTL assays can also be used to determine (i) the lytic potential of multiple CTL populations with different specificities at the same time, and/or (ii) the cytotoxic efficiency of a given CTL population by using target cells loaded with titrated amounts of specific peptides. This requires the simultaneous use of more than two target cell populations. This can be achieved by a combination of multiple dyes, which are used at different concentrations to stain target cell populations [843]. A scheme summarizing a simplified 2-color “*in vivo* Multiplexed Antigen-Specific Cytotoxicity Assay” (iMASCA) with nine target cell populations is shown as an example of the power of this assay in Figure 80.

**Figure 81.**
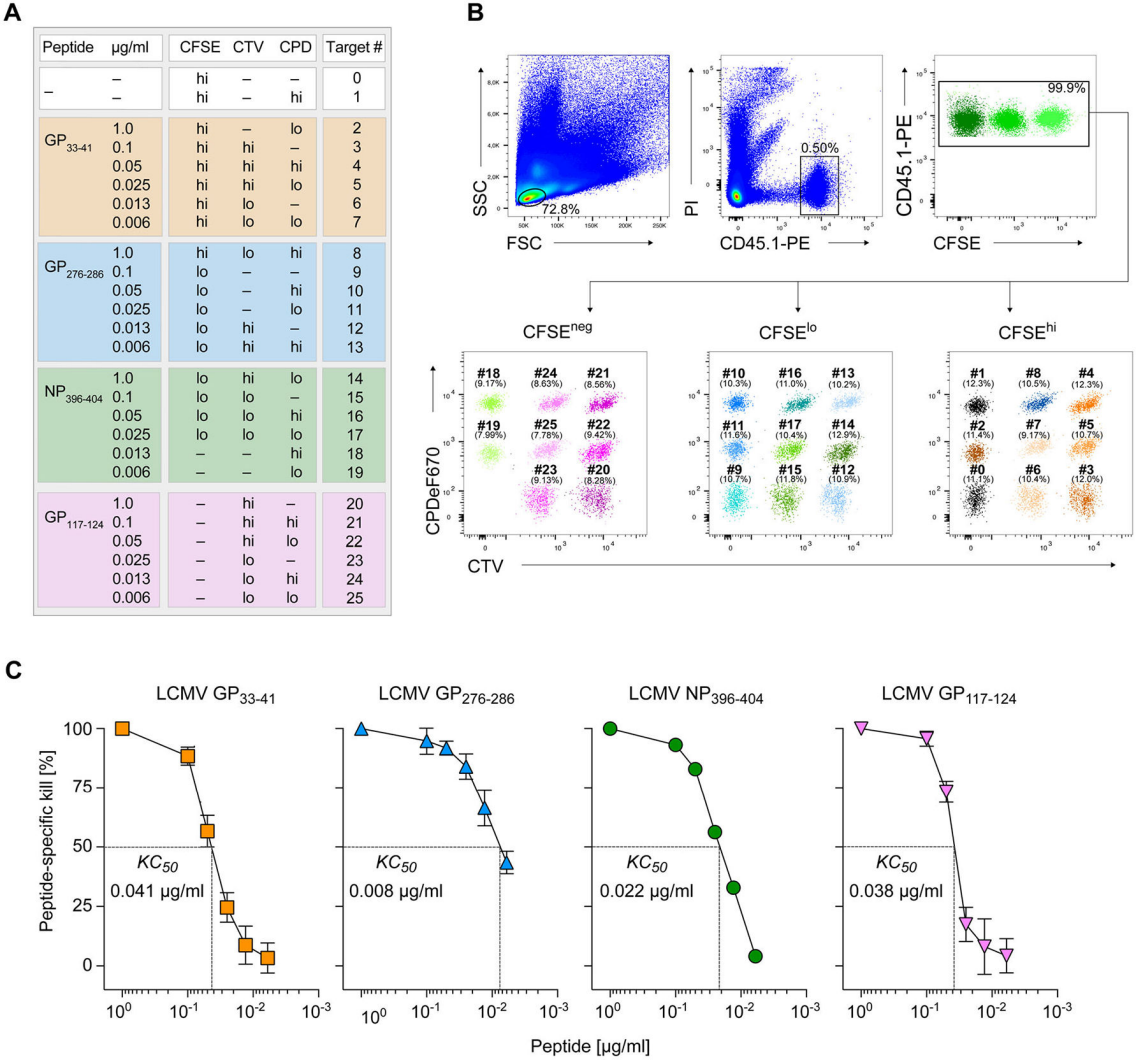
Three-color iMASCA in the mouse with 26 target cell populations (For experimental details see protocol). Responder C57BL6/J mice were infected with LCMV and 7 days later cytotoxic CD8^+^ T cell activity was quantified in uiuo against six different concentrations of 4 different epitopes each to calculate the respective KC_50_. (A) Table summarizing the 26 targets used for cytotoxicity assay. (B) Flow cytometric gating strategy and deconvolution to identify the different CTL targets recovered from spleen of responder mice 3h after adoptive transfer. Numbers on the dot plot indicate target populations as listed in (A). (C) Quantification of *in vivo* CTL activity for the indicated peptide complexed to the respective MHC-I molecule. KC_50_ was calculated by using regression analysis in the linear range. Data represents mean ± SD (n = 10 mice). CFSE, Carboxyfluorescein succinimidyl ester; CTV, CellTrace violet; CPD, Cell proliferation dye eFluor 670.

We have applied 3-color iMASCA to quantify the killing efficiency of CTL populations against 4 different LCMV-derived epitopes by employing 26 cell targets loaded with decreasing amounts of specific peptides. For this, CD45.1^+^ target cells were subdivided into 26 samples, labeled with different concentrations and combinations of three cell dyes, and loaded with LCMV-derived peptides at the indicated concentrations (Figure 81A). Three hours after adoptive i.v. transfer, target cell frequencies were determined in spleens of CD45.2^+^ recipient mice infected with LCMV 7 days before (Figure 81B). Based on these results, the peptide concentration to achieve 50% of the maximum kill activity (KC_50_) was calculated (Figure 81C). Despite previous identification of D^b^/GP33- and D^b^/NP396-specificities as immunodominant in the anti-LCMV acute response based on IFN-γ^+^ CTL frequencies [235, 844], we found a much higher cytotoxic efficiency (KC_50_) directed against D^b^/GP276 complexes followed by D^b^/NP396, D^b^/GP117 and D^b^/GP33 suggesting that immunodominance in terms of Ag-specific CD8^+^ T cell frequency may not reflect immunodominance in cytotoxic activity. The following protocol was used to generate the data depicted in Figure 81.

#### 6.3 Step-by-step sample preparation for in vivo Multiplexed Ag-Specific Cytotoxicity Assay (iMASCA)

##### Immunization of responder mice

1. Infect CD45.2^+^ C57BL/6J mice i.v. with 2x10^4^ p.f.u. LCMV WE in 100 μL PBS.

##### *In vivo* multiplexed cytotoxicity assay (7 days after infection) Dilution of cell dyes (8:00 am)

2. Label as many 5mL tubes as targets to be used (“Dye 0”, “Dye 1”, … “Dye 25”).
3. Add at least 2.2mL of PBS or PBS containing diluted dye(s) at a 2x final working concentration as indicated in Figure 81A (Column “Cell dyes”).
2xfinal[High]=2μM2xfinal[Low]=0.2μM
4. Leave in the dark at room temperature until step 14.

##### Preparation of target cell suspension (9:00 am)

5. Make a single-cell suspension of pooled spleens (if necessary, skin-draining lymph nodes and mesenteric lymph nodes can be included) in ice-cold PBS isolated from naive CD45.1^+^ donor mice (B6.SJL-*Ptprc^a^ Pepc^b^*/BoyJ) using standard procedures.

One donor mouse will provide enough targets for four recipient mice.

Optional: red blood cells may be lysed at this stage.

6. Wash twice in ice-cold PBS and adjust to 2 × 10^7^ live cells/mL in ice-cold PBS.
7. Keep on ice until step 11.

##### Peptide pulsing of target cells (11:00 am)

8. Label as many 1.5mL tubes as targets to be used (*“Peptide 0”,* … *“Peptide 25”).*
9. Label as many 15mL tubes as targets to be used (*“Target 0”,* … *“Target 25”).*
10. Prepare at least 1.2mL of 2x peptide concentration in “Peptide” tubes from step 8.

For this experiment, the peptides and their final concentrations are indicated in Figure 81A.

11. Add 1mL cell suspension (step 6) to “Target” tubes from step 9.
12. Add 1mL of the corresponding peptide dilution (“Peptide” tubes) to “Target” tubes.

Each “Target” tube contains now 2mL PBS with 2 × 10^7^ target cells and 1× [final peptide].

13. Incubate 15 min at room temperature (approx. 22°C) for peptide loading of MHC-I molecules. Alternatively, peptide loading can be performed for 1 h at 37°C for peptides binding with very low affinity.

##### Labeling of targets with cell dyes and adoptive transfer into responder mice (12:30-3:00 pm)

14. To the “Target” tubes, add 2mL of the corresponding “Dye” tube from step 3 and mix immediately.

Each “Target” tube contains now 4mL PBS, 2×10^7^ target cells, 1× [final dye], and 0.5× [final peptide].

15. Incubate exactly 15min at room temperature in the dark.
16. Add 10mL ice-cold PBS containing 10% FCS.
17. Centrifuge at 470 × *g*, 10 min, 4°C, and decant supernatant avoiding cross-contamination.
18. Repeat step 17 three times washing with 14 mL ice-cold PBS-10% FCS.
19. Resuspend cells in each tube with 1mL ice-cold PBS-2% FCS.
20. Transfer all cells to a 50 mL tube on ice.
21. Filter through a 70 μm cell strainer to remove cell clumps.
22. Count viable cells and adjust at 2.6 × 10^8^ viable cells/mL in ice-cold PBS.
23. Inject 100 μL cell suspension i.v. into LCMV-infected responder mice.

Each responder mouse receives 2.6x10^7^ total targets (1×10^6^ cells of each target populations)

##### Recovery of target cells from responder mice and calculation of CTL activity (6:00 pm)

24. Humanely kill responder mice 3 h after adoptive transfer of targets.
25. Make a single-cell suspension of splenocytes (lymph nodes and blood are optional) and stain 20% of the total spleen with anti-CD45.1-PE (clone A20) using standard flow cytometry protocols.

In this protocol, each target cell population will represent only about 0.05% of all leukocytes in the responder spleen.

26. Perform flow cytometry and acquire 1–2 × 10^3^ of live Target #0 or #1 populations (control targets without peptide).

##### Quantification of Ag-specific kill activity

27. Calculate the percentage of peptide-specific kill for each Target cell population using the following equation:
$$\%\;Specific\;kill=100-\left(100\;x\;\frac{R_{i}}{R_{n}}\right)$$

Where, for example, to calculate %specific kill of Target population #10 in Figure 81:

$$R_{i}=\frac{Number\;of\;Target\;\#10\;cells\;in\;immune\;mice}{\left[\begin{array}{c} \;Average\;number\;of\;unloaded\;Target\;cells\\ \;(\#0,\;\#1)\;in\;immune\;mice \end{array}\right]}$$

$$R_{n}=\frac{Number\;of\;Target\;\#10\;cells\;in\;naive\;mice}{\left[\begin{array}{c} \;Average\;number\;of\;unloaded\;Target\;cells\\ \;(\#0,\;\#1)\;in\;naive\;mice \end{array}\right]}$$

Optional: Staining Ag-specific CTLs with MHC-I/peptide multimers allows to normalize the % of CTL activity to Effector-to-Target ratios across the different target cell populations or mice.

#### 6.4 Materials

**Table:** 

Product	Company
DPBS	Gibco
Heat inactivated FCS	Gibco
Peptides	Xaia peptides
Cell Proliferation Dye eFluor670	Thermofisher
CFSE	Thermofischer
CellTrace Violet	Thermofischer
Propidium iodide	Sigma Aldrich
BD FACSCantoII	BD Biosciences

#### 6.5 Data analysis

Samples are processed for standard flow cytometric analysis. Specially, for *in vivo* cytotoxicity assays, it is necessary to acquire a relatively large number of events since the proportion of target cells amongst the total acquired population is very low, typically below 1–2%. The gating strategy for quantifying Ag-specific cytotoxic activity is illustrated in Figures 80 and 81. It is recommended that an “empty channel” is used to gate out autofluorescent cells. The degree of cytotoxic activity is determined by the relative decrease in the number of target cells displaying the specific epitope over those displaying an irrelevant epitope at the end of the assay time. To calculate the percentage of specific killing the formula described in Step 27 above is used.

#### 6.6 Top tricks

##### 6.6.1 Labeling with cell dyes.

1. This assay relies on a clear **separation of multiple target populations** by flow cytometry. Each lot of cell dyes should be tested, aliquoted, and thawed only once for reproducible results.
2. **Keep extracellular proteins to a minimum** not to quench labeling with cell dyes.

##### 6.6.2 Peptide pulsing of target cells.

1. MHC-I peptide loading is **most efficient at room temperature** [845].
2. Keep cells at **4°C once peptides are loaded** at all times to avoid decay from MHC-I molecules.
3. The **peptide concentration range** of choice depends on several parameters, including peptide affinity for MHC-I receptor (K_on_ and K_off_ rates), TCR affinity, and killing efficiency, and therefore it should be optimized before first use. 1 μM peptide (approx. 1 μg/ml for 9mer peptides) usually results in saturated kill activity. However, once peptide concentration is not saturating, cytotoxicity may quickly decrease with diminishing concentrations (see Figure 81C). Therefore, it is recommended to employ **small step dilutions** (e.g., 1:2) to calculate the KC_50_ dose.
4. Maximum care should be taken to avoid peptide **cross-contamination** between samples.
5. Working fast will minimize variability in fluorescence intensity between different tubes.

##### 6.6.3 Choice of target cells, route of administration, and period of time for killing.

1. The choice of target cells depends on the experimental question. One pre-requisite is that target cells enter organs where CTLs are present. Therefore, spleen and/or lymph node cells are suitable targets to quantify CTL activity in secondary lymphoid organs (SLOs). Naive lymphocytes, however, do not efficiently enter non-SLOs and thus cannot be used to monitor CTL activity there.
2. Intravenous injection of 10^6^ cells per target population is sufficient to monitor cytotoxicity in SLOs. This cell number may be increased to reduce the acquisition time (Protocol Step 26), but care should be taken not to reach saturating numbers (in our experience, up to 15 × 10^6^ cells loaded with the same peptide can be utilized as targets loaded with a given peptide during the acute or memory phases of antiviral responses).
3. Splenocytes can also be used to quantify **CTL activity in lung** airways by intratracheal administration and recovery of target cells by bronchoalveolar lavage [22].
4. To avoid vascular obliteration, reduce injection speed with increasing target cell number and/or size. If cell clumps are visible, filter cell suspension again through a 70μm cell strainer before injection.
5. The optimal **period of time for killing** depends on several factors, most importantly (i) experimental question, (ii) frequency and killing capacity of specific CTLs, and (iii) peptide decay from target cells (K_off_ rate). Excessive peptide decay or saturated CTL activity can be avoided by limiting the period of killing time. Three hours or less are normally sufficient for immunodominant epitopes during an acute anti-viral immune response (Figure 81C, data not shown for influenza, and [832]), whereas longer times (e.g., 5-12h) may be required in cases of suboptimal CTL responses.

##### 6.6.4 Modifications.

1. Besides the modifications described above, the **number of target populations can be scaled up by** adding an additional concentration of cell dyes, more cell dyes or an additional set of targets (e.g., target cells expressing tdTomato or another reporter not interfering with the cell dyes’ emission).
2. The assay can be easily modified to quantify **cytotoxicity by CD4^+^ T cells** [846, 847] by employing MHC-II ligands and gating for MHC-II^+^ cells (e.g., B cells) during quantification of CTL activity, as well as to quantify NK cell-mediated cytotoxicity against, e.g., TAP1^−/−^ splenocytes [848, 849].

#### 6.7 Pitfalls

Although this assay is very powerful to accurately describe killing activity *in vivo*, it has some limitations:

1. **Location where killing takes place** may be ambiguous due to the ability of target cells to recirculate between SLOs and blood. Shortening the period of killing time will reduce the likelihood of target recirculation, and proper controls may be required to demonstrate killing in a specific SLO.
2. Killing of lymphocytes is used as a read out for CTL activity but may not represent the **actual killing capacity of the CTL population** during infection, autoimmunity, or tumor growth because (i) naive lymphocytes may not be the physiological targets in those diseases and/or (ii) SLOs may not be the location where effector CTLs perform their cytotoxic function during that given disease.

### 7 Measurement of signal transduction pathways in human T cells

#### 7.1 Overview

In this section, we describe how to investigate, in human CD4^+^T cells, the phosphorylation status of S6 ribosomal protein (pS6Ribo) as an indicator of PI3K-Akt-mTOR signaling pathway activation following TCR stimulation [850]. However, this protocol can be applied to other signaling pathways in T cells, for example, cytokine stimulation or costimulatory molecules triggering [851].

#### 7.2 Introduction

T cell activation requires TCR engagement by peptide-MHC complex together with additional costimuli such as CD28 triggering by CD80/86 molecules expressed on Antigen-presenting cells, as well as cytokine stimulation. Surface receptor stimulation is followed by intracellular events that rely mainly on the phosphorylation or dephosphorilation of molecules involved in the signaling cascade. This is important to amplify and transmit the information originated by receptor stimulation. Signaling cascades are usually connected downstream of different surface receptors, thus leading to an intracellular integration of distinct signaling events. The final outcome is the activation or inhibition of specific transcription factors, and then the expression of a specific gene signature. The investigation of the phosphorylation status of intracellular mediators is a useful tool to understand step-by-step how the extracellular information is propagated inside the cell. By this way, it is also possible to understand if any alteration is present in a given signaling pathway. (See also CHV Sect IV.7 Section IV.7 – Measurement of signal transduction pathways by flow cytometry).

#### 7.3 Step-by-step sample preparation

##### 7.3.1 Mononuclear cells’recovery.

1. Collect whole blood in a tube coated with an anticoagulant.
2. Gently stratify 9 ml blood onto 6 ml Ficoll in a 15 ml tube.
3. Centrifuge at room temperature, 1500g without break for 20 minutes.
4. Collect the ring between the phases, containing mononuclear cells, and transfer in a new 15 ml tube. Fill up the tube with PBS 7.2 and centrifuge 300 × *g* for 7 minutes.
5. Discard the supernatant and resuspend cells in 15 ml PBS 7.2. Repeat the centrifugation step.
6. Resuspend cells in complete medium (RPMI+10% FBS) and count. At least 200000 cells for each experimental condition are needed.

##### 7.3.2 Cell stimulation.

1. Stain cells with mouse anti-human CD3 Ab (clone HIT3a, IgG2a, 5μ;/ml) and mouse anti-human CD28 Ab (clone CD28.2, IgG1, 5μg/ml) in 50 μl of complete medium in a 1.5 ml Eppendorf tube. Incubate at 4°C for 5 min.
2. Cap primary Abs by adding 50 μl complete medium containing anti-mouse IgG1 and anti-mouse IgG2a. Final concentration of anti-mouse IgG1 and anti-mouse IgG-2a is 5μg/ml. Incubate at 37°C for the kinetics experiment. We recommend the following kinetics: 0’ (no stimulation), 10’, 20’, 30’.
3. At each time point of the kinetics experiment, fill up the appropriate tube with cold PBS 7.2 and centrifuge 300 × *g* for 7 minutes at 4°C.
4. Discard the supernatant and resuspend cells in 250 μl of PBS 7.2. Add an equal amount (250 μl) of pre-warmed (37°C) BD Cytofix and incubate for 10’ at 37°C.
5. Fill up the tube with 1 ml wash buffer (PBS 7.2 +BSA 0.5%) and centrifuge 300 × *g* for 7 minutes.
6. Resuspend cells in 500 μl wash buffer.
7. Centrifuge the tubes at 300 × *g* for 7 min.
8. Discard the supernatant and resuspend cells in 500 μl pre-cooled (-20°C) BD Perm Buffer III. Incubate 30’ on ice.
9. Fill up the tubes with wash buffer and centrifuge 300 × *g* for 7 min.
10. Discard the supernatant and stain cells with anti-human CD3-PB, anti-human CD4-PECy7, anti-human CD8-APCCy7, anti-human pS6Ribo (Ser235/236)-Alexa Fluor 488 for 20’ at room temperature.
11. Fill up the tubes with wash buffer and centrifuge 300 × *g* for 7 min to remove unconjugated Abs.
12. Discard the supernatant and resuspend in 500 μl wash buffer for flow cytometry analysis

#### 7.4 Materials

- Complete medium: RPMI+10% heat inactivated FBS
- Wash buffer: PBS 7.2+0.5%BSA
- PBS 7.2
- Fixation buffer: BD Cytofix
- Permeabilization buffer: BD Perm Buffer III
- mouse anti-human CD3 Ab (BD, clone HIT3a, IgG2a)
- mouse anti-human CD28 Ab (clone CD28.2 IgG1, 5μg/ml)
- Goat anti-mouse IgG1
- Goat anti-mouse IgG2a
- anti-human CD3-PB (BD, clone UCHT1)
- anti-human CD4-PECy7 (BD, clone SK3)
- anti-human CD8-APCCy7 (BD, clone SK1)
- anti-human pS6Ribo (Ser235/236)-Alexa Fluor 488 (Cell Sig-naling, clone 2F9)

#### 7.5 Data analysis

**Figure 82.**
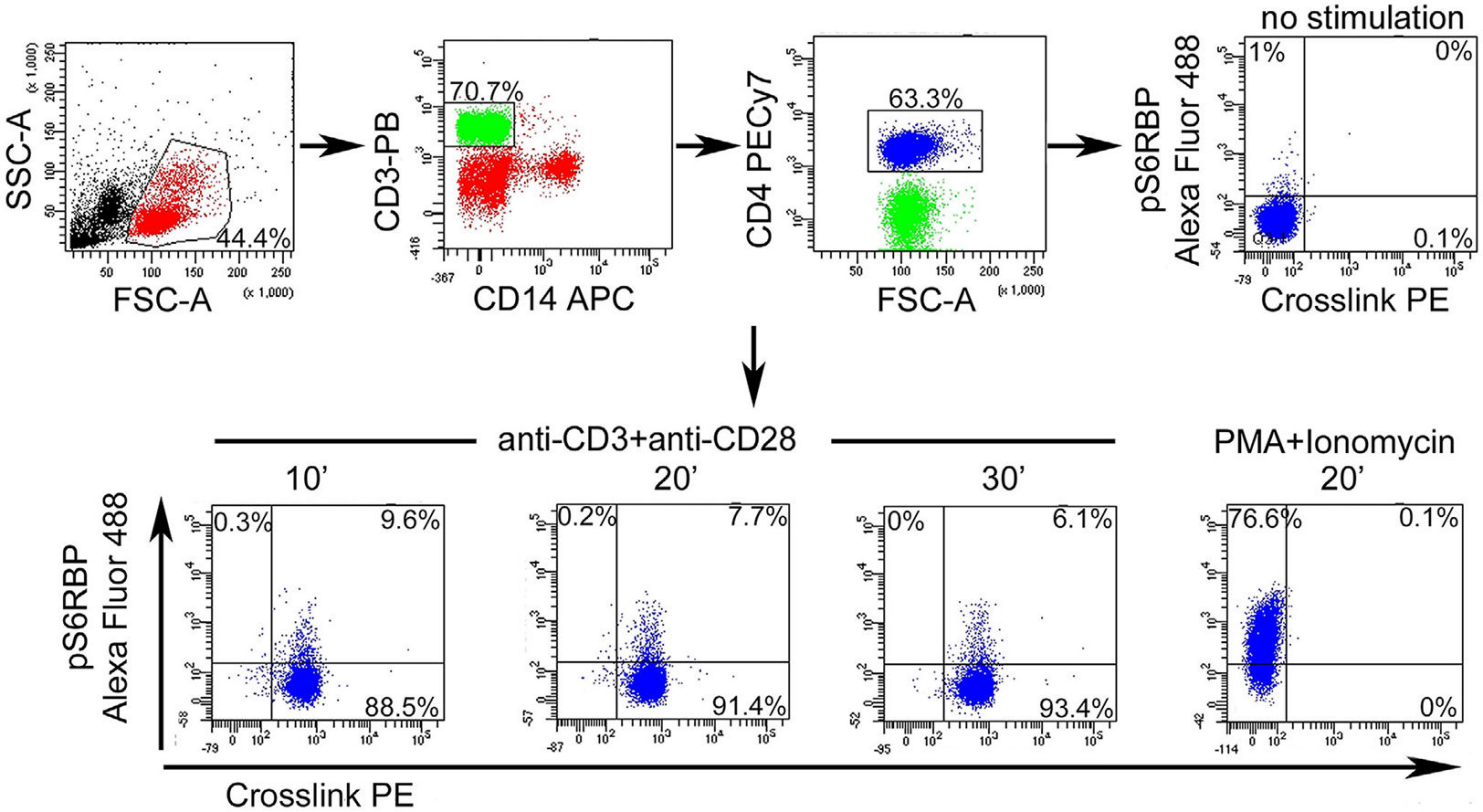
pS6ribo evaluation on PBMNC following TCR stimulation. Lymphocytes were gated based on physical parameters, then T cells were identified as CD3^+^CD14−. T helper cells were gated as CD4^+^. pS6ribo was evaluated either on unstimulated cells or upon anti-CD3 and anti-CD28 stimulation for 10, 20 or 30 minutes. As a positive control of the procedure PBMNC were stimulated with PMA and Ionomycin for 20 minutes. In this experiment anti-CD3 and anti-CD28 capping was performed by the addition of PE-conjugated anti-isotype mAbs (anti-mouse IgG1 and anti-mouse IgG2a). Thus, cells with an efficient crosslink can be detected as PE-positive.

Data analysis can be performed via the identification of the percentage of cells that display the phosphorylated protein of interest (as in Figure 82). However, sometimes two clear cell subsets (phosphorylated versus unphosphorylated) cannot be identified. In those cases, it is very useful to evaluate the MFI of the protein of interest.

#### 7.6 Pitfalls

In case of TCR signaling study, staining of surface CD3 requires the usage of an Ab clone distinct from that used for cell stimulation. The two Abs should not compete for the same epitope.

#### 7.7 Top Tricks

PI3K-Akt-mTOR activity lasts only several minutes following stimulation. Alterations of the signaling pathway can be observed either as a delayed/shorter kinetic of phosphorylation or as a reduced/increased magnitude of phosphorylation. For these reasons, we recommend to perform kinetics experiments rather than a single time point observation. The same can be applied either to STAT protein phosphorylation or other signaling cascades.

#### 7.8 Clinical relevance statement

The assay shown in this section is applicable for the analysis of T cells in immunodeficient patients, with suspected alterations of signaling transduction pathways. This may include both signaling downstream TCR or cytokine receptors [852]. Functional assays are indeed fundamental to confirm suspected pathogenic mutations identified by whole genome or exome sequencing.

### 8 Live cytokine-producing cell sorting with cytokine secretion assay^™^

#### 8.1 Overview

The aim of this section is to illustrate how to recover live human T cells depending on their capacity to produce specific cytokines. This technique can be applied to both identification and sorting of Ag-specific cells, as well as polyclonal T cells with a common cytokine production profile. Although the data presented in this protocol refer to CD4 T cells, the assay can also be applied on CD8 T cells.

#### 8.2 Introduction

Following Ag recognition, T cells acquire effector properties that guarantee pathogen clearance. Cytokine secretion is one of the most effective properties of activated T cells as it orchestrates a functional immune response involving both cells of adaptive and innate immunity. Different pathogens evoke different cytokine responses; thus T cells can be functionally distinguished based on their cytokine profile. Indeed, there are at least three major types of cell-dependent immunity, classically defined as type 1, type 2, and type 3 responses (see section Human CD4 T cells). Type 1 immunity defends from intracellular bacteria and viruses, involves Th1 and CTL T (Tc)1 cells, and is orchestrated by the transcription factor Tbet with the production of IFN-γ. Type 2 immunity fights extracellular parasites and is mediated by Th2 and Tc2 cells, which express the transcription factor GATA3 and produce IL-4, IL-5, and IL-13. Finally, ROR-_γt_^+^IL-17^+^ Th17 and Tc17 cells mediate type 3 immunity, which protects from extracellular bacteria and fungi [853]. Despite these distinctions, it has been described more recently that distinct effector programs can coexist within the same cell. Indeed, cells simultaneously producing IFN-γ and IL-17 (Th1/17), IL-4 and IL-17 (Th2/17) and IFN-*γ* and IL-4 (Th1/2) have been identified [152, 854-856]. Moreover, it has been demonstrated that a single pathogen can evoke functionally heterogeneous T cell responses [857]. In this complex scenario, the cytokine secretion assay^™^ (Miltenyi Biotec) is a versatile tool that allows the identification and recovery of live Ag-specific T cells based on their cytokine production profile. First, cells are shortly stimulated with Ag or with polyclonal stimuli. Then, cells are labeled with the Catch Reagent specific for the cytokine of interest. Catch Reagent is made up of two Abs linked for their Fc regions. One Ab is specific for the pan-leukocyte marker CD45 and allows binding to the leukocyte surface. The other Ab is specific for the cytokine of interest. Cells are then incubated again at 37°C to favor cytokine production. If a cell secretes the specific cytokine, it will bind to the catch reagent on the cell surface. The addition of a secondary fluorochrome-linked Ab, recognizing a distinct epitope of the cytokine from that of the catch reagent allows the detection of cytokine-producing cells. The cytokine secretion assay^™^ can be applied either on whole blood, PBMNC or even directly on T cells when using polyclonal stimulation. Staining with lineage-specific Abs allows the identification of a specific cell subset that is producing the cytokine, when working on whole blood or PBMNC [497] or on cells from biological fluids [858]. Live cytokine-producing cells can then be recovered either by immunomagnetic or flow cytometric sorting.

#### 8.3 Step-by-step sample preparation

##### A. Starting material.

The protocol can be applied either on whole blood, PBMNC, or isolated T cells. Whole blood must be collected with anticoagulant sodium heparin. Since calcium is critical for lymphocyte activation, chelating anticoagulants cannot be used for blood collection. When working with PBMNC, fresh cells are preferred but cryopreserved cells can be also used.

##### B. Cell stimulation.

1. Wash cells at 300 × *g* for 7 minutes.
2. Resuspend cells in RPMI 1640 supplemented with 5% human serum. Final cell concentration must be 10×10^6^/ml.
3. Add the specific peptide/protein at the desired concentration. Always include a positive control (SEB, PMA/Ionomycin) and a negative control (no stimulus). The optimal positive control must be chosen based on the cytokine of interest. Incubation period ranges from 3 h in case of polyclonal stimuli to 6–16 h for proteins. Incubation must be performed at 37°C with 5% CO_2_.
4. Following incubation, collect cells in a 15 ml polypropylene tube.

##### C. Cytokine Secretion Assay

1. Prepare 100 ml cold buffer; 100 μl cold medium; 10 ml warm medium. Volumes are adjusted for up to 10×10^6^ cells. Scale up for larger numbers. Do not reduce volumes if working with less than 10×10^6^ cells.
2. Wash cells from step B4 with 10 ml cold buffer and spin down at 300 × *g* for 7 minutes.
3. Resuspend up to 10× 10^6^ cells with 80 μl cold medium, then add 20 μl of catch reagent. Mix and incubate 5 min on ice.
4. Add warm (37°C) medium and dilute cells depending on the expected amount of cytokine-producing cells. Proper dilution is critical to prevent unspecific binding of secreted cytokines to close cells. If less than 5% cytokine producing cells are expected, add 10 ml of warm medium to achieve a final concentration of 10^6^ cells/ml. If more than 5% cytokine producing cells are expected, add 100 ml of warm medium to a final concentration of ≤10^5^ cells/ml. Further dilution is required for expected frequencies of cytokine producing cells >20%.
5. Incubate cells 45 min at 37°C 5%CO_2_ to allow cytokine secretion and binding to catch reagent. During this incubation period rotate tubes every 5 min or use MACSMix^™^ rotator to avoid cell to settle, thus leading to cytokine unspecific binding.
6. Following incubation put the tubes on ice. Spin down cells in a pre-cooled centrifuge at 300 × *g* for 7 minutes.
7. Wash cells with cold buffer to block cytokine secretion and repeat the centrifugation step.
8. Resuspend cells up to 10×10^6^ cells with 80 μl cold medium, then add 20 μl of cytokine detection fluorochrome-conjugated Ab. Additional Abs can be added at this step to allow simultaneous detection of other markers. Mix and incubate 10 minutes on ice.
9. Wash cells with cold buffer and centrifuge at 300 × *g* for 7 min.
10. Cells are now ready for flow cytometry analysis or sorting. Always add Propidium Iodide to exclude dead cells from the analysis.

#### 8.4 Materials

- **Buffer:** Phosphate-buffered saline (PBS), pH 7.2, supplemented with 0.5% bovine serum albumin (BSA) and 2mM EDTA
- **Medium:** RPMI 1640 supplemented with 5% human serum. Do not use foetal cow serum or bovine serum to avoid unspecific stimulation.
- Peptide/Protein of interest
- **Phorbol 12-myristate 13-acetate (PMA) and Ionomycin; Staphylococcal Enterotoxin B (SEB)** for polyclonal stimulation
- **Propidium Iodide** for flow cytometric exclusion of dead cells
- Cytokine secretion assay kit (Miltenyi Biotech)

#### 8.5 Data analysis

**Figure 83.**
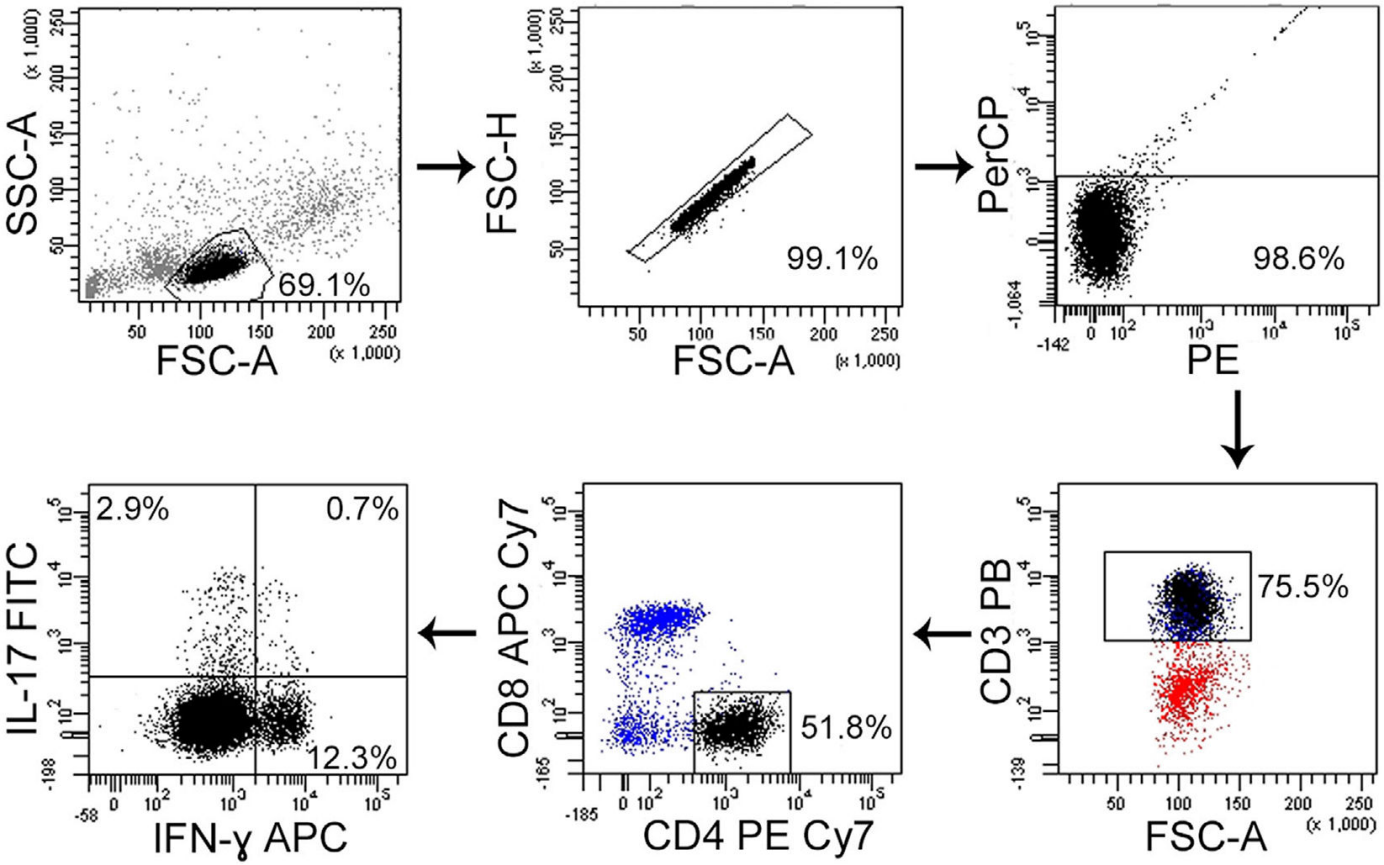
Cytokine secretion assay performed on PBMNC for the detection of IFN-γ and IL-17 producing human T helper cells. Cells were stimulated with PMA/Ionomycin. Lymphocytes were gated based on physical parameters, then doublets removed using FSC Height and Area (FSC-H and FSC-A, respectively). Dead cells were excluded as PerCP-positive and PE-positive following Propidium Iodide addition. T helper cells were then identified as CD3 positive, CD4 positive, CD8 negative. IFN-γ, and IL-17 expression were subsequently analyzed.

Data analysis requires the exclusion of doublets and death cells using a live-dead marker. This is especially true if using cryopreserved cells instead of freshly isolated. As in Figure 83, lineage markers allow the identification of the population of interest for the identification of cytokine-producing cells.

#### 8.6 Pitfalls

Dilution steps and continuous rotation during the incubation period are critical to avoid cytokine binding to non-producing cells.

#### 8.7 Top tricks

Secretion of two distinct cytokines can be evaluated simultaneously via combining cell staining with two distinct catch reagents and detection Abs. The only requirement is that detection Abs must be conjugated to distinct fluorochromes. Dilution factor during the incubation period must be calculated based on the expected higher percentage of cytokine producing cells.

#### 8.8 Clinical relevance statement

The assay shown in this section is applicable for the isolation of viable Ag-specific T cells, which may represent a novel strategy for cell-based therapies in chronic infections and cancer. It has already been shown that this assay can be used for the identification of pathogen-reactive T cells [731, 859]. However, the kit is currently approved for research use only. In any case, it is a powerful tool for clinical research, allowing in-depth characterization of Ag-specific T cells.

### 9 Quantification of soluble cytokines with cytometric bead array

#### 9.1 Overview

Cytokines are the main soluble proteins secreted by various cells of the immune system that play different roles in regulation of immune responses, since they influence migration, activation, and proliferation of various cell types, including tissue resident cells. These mediators show commonly pleiotropic features, exhibit redundant and overlapping properties and mediate the production or regulate the function of other cytokines. The final effect on a specific cell type depends on the balance among multiple cytokines that again depends on their activity or concentration. Thus, the evaluation of an extended number of cytokines in a biological fluid rather than a single cytokine may represent the best strategy to better investigate various physiologic and/or pathologic settings. In this context, the multiplex beads-based array described in this section is a valuable tool for the analysis of several cytokines. Indeed, it allows to measure by flow cytometry several analytes at the same time in a small sample volume.

#### 9.2 Introduction

Different methods have been developed to define the cytokine concentration in biological fluids, mainly based on competitive or sandwich principles. In these systems, Ags or Abs are labeled with an enzyme or with a fluorescent, luminescent or radioactive molecule. Historically, the classical method that belongs to this family is the enzyme-linked immunosorbent assay (ELISA), in which the targeted cytokine is sandwiched by two Abs specific for a different epitope of the same cytokine. In this method, the first Ab is linked to a plastic plate support and is defined as the “capture” Ab; the second Ab instead is conjugated with the detection molecule and is defined as the “detector” Ab. ELISA is characterized by high specificity and sensitivity, however, it allows to measure a single analyte and the procedure requires the use of a relevant volume of samples for each measurement.

The introduction of multiplex beads-based immunoassays significantly changed the approach for quantification of cytokines and other soluble factors in biological fluids or culture supernatants. The principle of this method is the use of a specific Ab coated on microbeads that serve as a ‘’solid’’ support as in the version of the ELISA technique. Microbeads can be detected by flow cytometry instruments, based on their fluorescence. During the incubation with the sample, the analyte of interest binds to the Ab-microbead complex. The addition of a fluorochrome-conjugated secondary Ab allows the detection of the analyte-microbead complex. Quantification is performed via referring to a standard curve, prepared with known scalar doses of protein concentration.

The combination of beads with different size and/or beads with different florescence intensity, represents the flexibility and the power of this method allowing to evaluate simultaneously up to 100 analytes in the same sample. Several kits for multiplex beads-based assay are available from different commercial vendors, each with specific properties, i.e., sample volume (generally ranging between 50 and 15 assay duration (on average only a few hours, depending on the length of incubation and washing steps), the possibility to customize the combination of primary beads, and sensitivity of the test, which also depends on the range of the standard curve [850, 860-863].

Here we provide the detailed protocol of Cytometric Beads Array^™^ (CBA) from BDBioscience as an example. Specific protocols from other vendors must be followed according to the manufacturer’s instructions. For example cytometric beads array can also be purchased from Biolegend (LEGEND Plex) or Miltenyi (MACSPlex assays).

#### 9.3 Step-by-step sample preparation

The BD CBA kit can detect: human, mouse, and rat soluble proteins, immunoglobulins, cell signaling factors. BD CBA solutions are available in two formats to meet diverse needs. BD CBA Kits are preconfigured with routine panels, while BD CBA Flex Sets provide an open and configurable method of detection, so that researchers can design their own multiplex kit. Beads are coated with an Ab specific to the protein of interest; each bead in the array has a unique red fluorescence intensity so that different beads can be mixed and run simultaneously in a single tube. These beads are incubated with a small sample volume and then further incubated in the presence of a capture Ab tagged with the fluorochrome phycoeryithrin (PE). At the same time a curve of standard samples between 10-2500 pg/ml, is performed to enable protein quantification.

##### 9.3.1 Standard preparation.

1a. Prepare the highest concentration of the standard curve for all the analytes by pooling all the lyophilized standard spheres in a single 15ml polypropylene tube. Add the appropriate amount of assay diluent following manufacturer’s instructions.

1b Mix well and wait 15 min RT (room temperature);

1c. Perform 1:2 serial dilutions in flow cytometric tubes adding the appropriate volume of assay diluent. Usually 10 standard points are recommended including the 0 (zero) tube that contains only assay diluent.

##### 9.3.2 Beads and sample preparation.

2a. Calculate the number of total tubes of the experiment (including both standards and samples). For each tube, you need 1 μl of beads for each analyte. Take the sufficient volume of beads for all the tubes. Mix all the beads specific for all the analytes in a single tube.

2b. Add 500μl of Wash Buffer from the kit.

2c. Centrifuge at 200 × *g* for 5 min.

2d. Aspirate the supernatant and resuspend in the appropriate volume of Capture Beads Diluent to reach a final volume of 50 μl per tube of the experiment.

Use the appropriate Capture Beads Diluent depending on the type of sample (serum, plasma, or culture supernatants)

2e. *Optional*. Depending on the type of experiment and expected protein concentration, perform the appropriate dilution of the samples using the assay diluent;

2f. Dispense 50 μl of standard or sample (or its appropriate dilution) in a tube;

2g. Add 50 μl of bead mix in each tube of standard or sample;

2h Incubate one hour at RT;

2i. Prepare the total mix of PE reagent containing the secondary Ab specific for each analyte included in the experiment, based on the number of total tubes to acquire (including both standards and samples), as reported in point 2a;

2l. Add 50 μl of PE reagent in each tube of standard or sample;

2m. Incubate 2 h at RT;

2n. Wash each tube with 1 ml of Wash Buffer, centrifuge at 200 × *g* for 5 min.

2o. Remove supernatants, then resuspend the beads in 300μl wash buffer and vortex before flow cytometry acquisition.

##### 9.3.3 Instrument setup.

**Figure 84.**
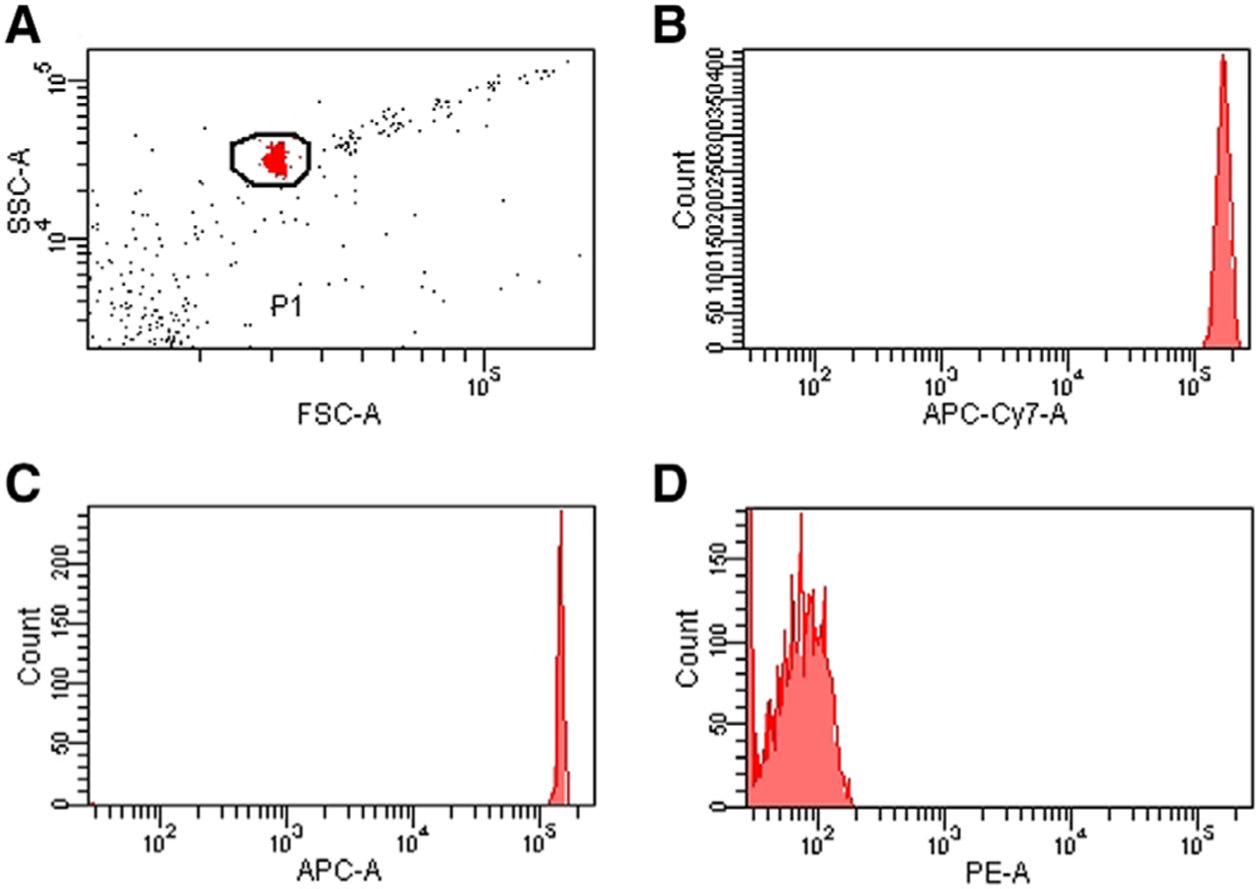
Flow Cytometer setup for multiplex-beads based array. A. FSC-SSC plot for the identification of beads based on their physical parameters. Histogram plots of APC-Cy7 (B) and ACP (C) channels showing that PMT voltages are optimally set to the highest visible MFI. By this way, it is possible to properly distinguish the different types of beads used. Panel (D) represents histogram plot of PE channel (the fluorochrome bound to the secondary Ab) measured on unstained beads.

**Figure 85.**
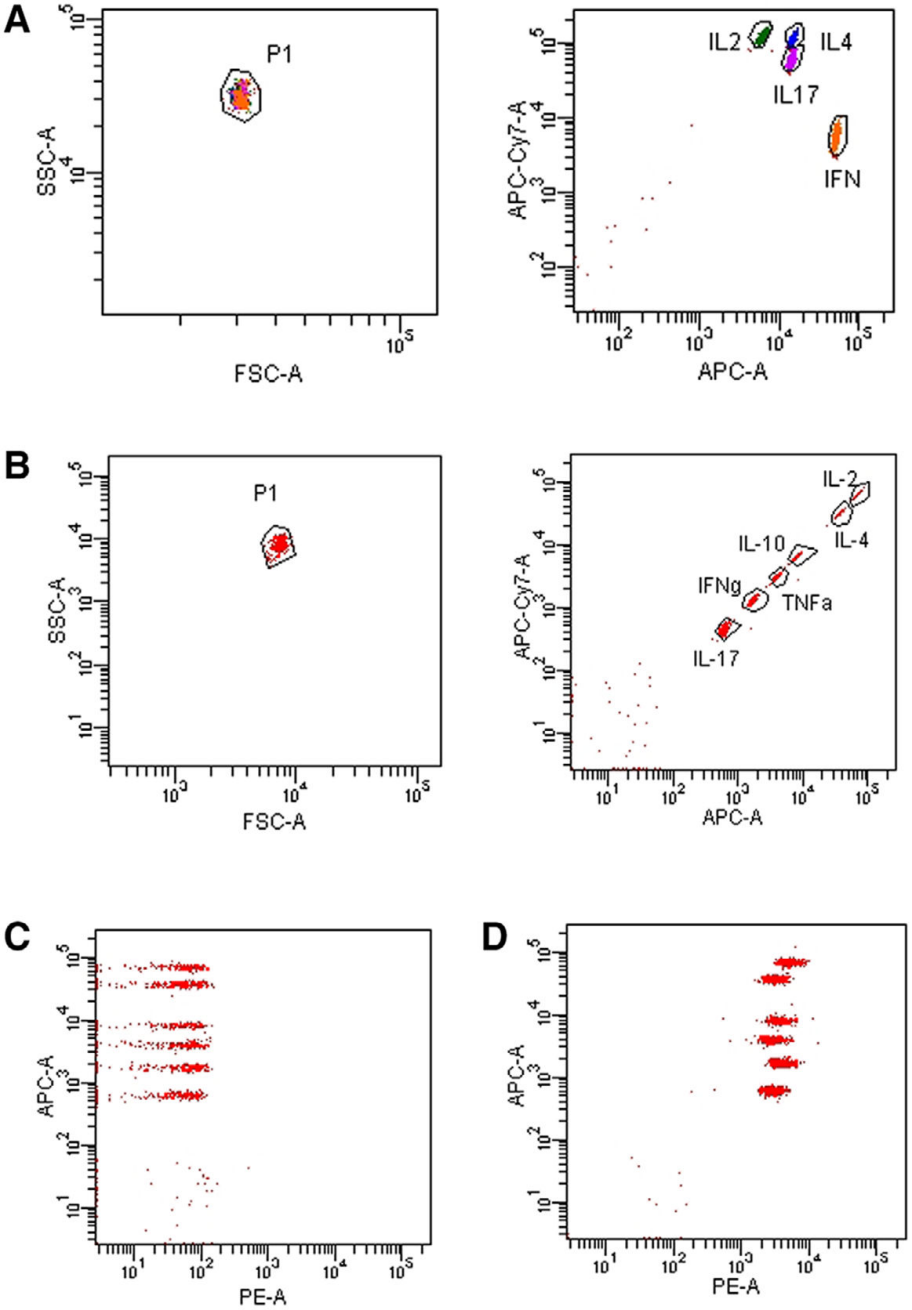
Quantification of human soluble cytokines with cytometric bead array (CBA). (A) Representative flow cytometry analysis of an experimental setting for evaluation of four different cytokines from culture supernatants of polyclonally stimulated human CD4^+^ T cells. The FSC/SSC plot allows identification of the total beads population; the APC-APC-Cy7 plot allows the identification of each bead corresponding to a specific analyte. Single beads are clustered based on the conjugation with different quantities of two different fluorochromes. (B) Representative flow cytometric plots of an experiment for evaluation of six different cytokines from culture supernatants of polyclonally stimulated human CD4^+^ T cells. The FSC/SSC plot allows identification of the total bead population; the APC-APCCy7 plot allows identification of each bead corresponding to a specific analyte. Single beads are clustered based on their fluorescence intensity; in this case each bead population is conjugated with the same quantity of the two different fluorochromes used for its identification. (C and D) Representative flow cytometric plots of a standard curve from an experiment for the evaluation of six different cytokines, as reported in panel B: the “zero” tube in panel C (0 pg/ml) and the “top” tube in panel D (2500 pg/ml). Beads clusters are identified in APC (or APC-Cy7) channel and the different quantities of each analyte are defined by PE MFI.

It is necessary to setup the instrument to correctly define the optimal voltage for different channels. First of all, it necessary to set the FSC and SSC parameters to identify the bead population as singlets and exclude doublets (Figure 84A). Subsequently, use compensation beads provided by the kit to set up the APC and APC-Cy7 voltages to the reach the highest MFI (see Figure 84B and 84C). This is of importance to allow proper identification of the different beads, since they have different APC and APC-Cy7 emissions (Figure 85A and 85B). Use the provided unstained beads to set up the minimum voltage of PE channel (Fig. 84D).

##### 9.3.4 Sample acquisition.

4a. Apply the final instrument setup obtained in the previous step to all samples within the same experiment;

4b. Acquire the standard tubes from 0 to the highest concentration (this sequence is important as the data analysis with the FCAP software will be easier, see below in section “data analysis”);

4c. Acquire each sample tube. In both standard and sample tubes it is important to record at least 300 events for each of the beads included in the mix.

##### 9.3.5 Materials not included in the kit.

- classical tubes for flow cytometry
- 15 ml polypropylene tubes for preparation of standards solutions
- vortex, centrifuge, pipets, and tips

#### 9.4 Data analysis

Data analysis is performed with FCAP Array software (available for Windows or MAC). Data generated for each acquired sample (including the standards) are exported from the acquisition software as .fcs files and then imported in the FCAP Array software. The first step is to select within the software menu the fluorescence of the beads (APC-APC Cy7 in this example) and the secondary Ab (PE in the example) used in the experiment. Subsequently, import the fcs file of the first tube of the standard curve. Using this first .fcs file, assign in the software the name of the specific target analyte to each bead cluster. Next, import the rest of the .fcs files (both standards and samples) and apply to all of them the beads-recognition setting as set for the first file imported. Then, indicate which .fcs files are standards and which are samples. For each sample it is possible to include the dilution factor (step 2e). Using the MFI of the fluorochrome on the detector Ab (PE in this example) the software calculates the standard curve from .fcs files of the standard tubes. A specific standard curve is generated for each analyte. Unknown protein concentrations in sample .fcs files are then calculated by the software comparing the MFI of each bead cluster to the corresponding standard curve.

#### 9.5 Pitfalls

- Fluidic alteration during the acquisition of different samples can induce a wrong bead clusterization impairing the analysis by the FCAP software;
- Different dilution factors may be required to study different cytokines in the same sample. In case of preconfigured commercial kits, it is necessary to repeat the experiment on diluted and undiluted samples. Instead, in case of customized experiments, it is possible to have separate kits so that cytokines with the same dilution factor are analyzed in the same experiment and separated from cytokines that require a distinct dilution.

#### 9.6 Top tricks

- Appropriate resuspension of standards, samples, and their dilutions are important to define the correct final concentration of the cytokines;
- Usually serum and plasma need higher dilution than culture supernatants but this depends on the type of analyzed cytokines as well as the culture conditions of in vitro supernatants;
- Gently mix standards and tubes prior to acquisition, by pipetting. Do not use vortex. Use vortex only to resuspend beads before beads preparation (step 2a and 2e).
- Samples can be stored −30°C before evaluation; in this case, it is important to completely defrost samples and mix well before their dilution and/or usage.

#### 9.7 Clinical relevance statement

This assay can be applied to define the concentration of different cytokines and/or chemokines from serum/plasma of patients affected by a wide range of diseases (i.e., allergic diseases, acute or chronic inflammation, autoimmune diseases, tumors, metabolic diseases, infections). This can be useful to perform a characterization of the disease or to evaluate the response to a specific treatment [864, 865]. Even if cytometric beads commercial kits are currently available for research use only, they are powerful tools for clinical research. Moreover, this assay can be applied to evaluate the concentration of different kinds of cytokines and/or chemokines from culture supernatants from a wide range of cells and different in vitro experimental conditions (Ag-specific response, activation stimuli, inhibition stimuli, drug treatment, co-culture experiments) [866-868].

### 10 Treg suppression assays

#### 10.1 Overview

Regulatory T (Treg) cells are critical for the maintenance of immune homeostasis. However, since many of their markers are shared by activated T-cells, accurately defining Treg cells can be difficult by phenotype alone. One defining feature of Treg cells is that they are capable of suppressing the proliferation and activation of other cells both *in vitro* and *in vivo.* As a result, measurement of their *in vitro* suppressive capacity is an important part of defining and characterizing a putative Treg cell population. This chapter details several methods for the assessment of the suppressive function of polyclonal or Ag-specific regulatory T-cells in humans or mice.

#### 10.2 Introduction

The ability to measure the capacity of Treg cells to prevent the proliferation of conventional CD4 and CD8 T-cells is an important factor in understanding their function. Tregs have been described to use a range of suppressive mechanisms with CTLA-4 dependent depletion of the co-stimulatory molecules CD80 and CD86 from the surface of APCs known to have a critical role [869]. Several methods for the assessment of cellular proliferation by incorporation of radioactive isotopes or cells counting have been used to measure cellular proliferation and suppressive function. However, these assays have difficulty in determining which cells are proliferating and cannot give detailed information on the number of divisions undertaken by individual cells. More recently cytometry-based assays relying on staining a responder population with an aminereactive fluorescent dyes such as CFSE and cell trace violet (CTV) that are diluted in a predictable manner during cell division has proven an effective method to measure cell proliferation. Utilizing this system, it is possible to add Treg cells to culture and observe the effects of varied ratios of Tregs on the proliferation of the responder population [381]. In addition to assays utilizing polyclonal stimuli such as anti-CD3, the measurement of the suppression of human Ag-specific T cells *in vitro* provides information closer to the physiology. However, suppression assays using Ag-specific T cells is made difficult by the low frequency of T cells specific to a single Ag in the T cell repertoire *in vivo.* In addition, highly functional CD8^+^ T effector cells, in contrast to their naïve counterparts, can resist Treg cell suppression *in vitro,* and can display multiple molecular strategies (including cell cytotoxicity targeting Tregs) to counteract excessive Treg cell suppression [870, 871]. In doing so, they can preserve their effector functions, which can produce protective or detrimental effects depending on the context (e.g., infection recovery vs. autoimmunity). As a result, measurement of their *in vitro* killing capacity is important to discriminate the highly functional CD8^+^ T effector cells that are not susceptible to Treg cell suppression, from those dysfunctional that have lost the capacity to resist Treg cells, because they become exhausted in tumor or chronic infection settings. Suppression assay of Ag-specific T cells is in general more used for clinical samples from patients, but it may be useful in murine models too, when immune responses against selective tumor or pathogen epitopes need more in depth analyses. Here we describe protocols allowing the measurement of human and murine Treg suppressive function in both a polyclonal manner and using a low number of Ag-specific CD8^+^ T cells, by selectively gating the latter with multimers of MHC class I molecules complexed with relevant antigenic peptides.

#### 10.3 Step-by-step sample preparation: Human polyclonal suppression assay

**Figure 86.**
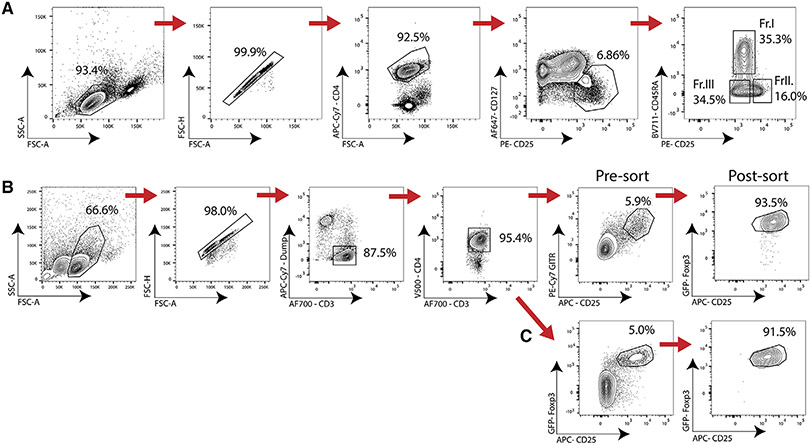
Sorting strategies for human and murine Treg. (A) Gating strategy of CD4 pre-magnetically enriched human PBMC CD4^+^CD25^+^CD127^lo^FOXP3^+^ cells and further sub gating into fractions I (Naïve Tregs), II (effector Tregs) and III (Non-Tregs/Tfr). In this example,CD4 APC-Cy7 was used to avoid clash with CXCR5 BV421 but we would recommend CD4 V500 and IR live/dead when this is not the case. (B) Gating example of murine CD3^+^CD4^+^B220^−^CD25^+^Foxp3-GFP^+^ Treg cells from lymph nodes. (C) Alternative gating strategy of murine CD3^+^CD4^+^B220^−^CD25^+^Foxp3-GFP Treg cells from lymph nodes if Foxp3 reporter is available.

1. Initially PBMCs are isolated from fresh blood via Ficol-Paque centrifugation in Leucosep tubes. CD4 T-cells are enriched by negative selection of CD4 cells with magnetic beads (Miltenyi). Cells are stained with Abs for CD4, CD45RA, CD127, and CD25 for 30 minutes at 4°C.
2. Bulk Treg cells can be sorted as CD3^+^CD4^+^CD127^lo^CD25^+^ (Figure 86A). If finer fractionation of Treg cells is required, CD127^lo^CD25^+^ cells can then be further separated into fraction I Naïve Tregs, fraction II effector Tregs and fraction III non-suppressive cells (Figure 86A) [379]. It should be noted that while fraction III as a whole is mostly made up of Foxp3 expressing non-Treg cells it may contain 20-30% CXCR5^+^ effector Tfr which are functionally suppressive Treg cells [872].
3. Naïve responder Tconv cells are sorted as CD25^−^CD45RA^+^CD4^+^ CD3^+^ and then stained with 1μM CFSE. A total of 1×10^4^ Tconv cells are co-cultured with various ratios of Tregs cells (0:1, 1:1, 1:2, 1:4, 1:8 Treg:Tconv) and 1×10^5^ γ-irradiated Ag-presenting cell (18.5Gy irradiated CD4 depleted PBMCs obtained by magnetic separation in step 1) and stimulated with 1 μg/mL soluble anti-CD3 (Clone: OKT3) for 4-5 days in 96-well round-bottom plates in RPMI medium containing 10% AB serum, 55 μM2-ME, L-glutamine, 25 mM HEPES and Penicillin/streptomycin in a final volume of 200μl. In all cases the number of Tconv and APCs is fixed while the number of Tregs is changed to obtain the intended ratios.
4. After a culture period of 4-5 days cells were then stained with CD4, CD25 and IR Live/Dead dye and data collected on a BD LSR Fortessa.

##### Materials: Human polyclonal suppression assay

The materials for the human polyclonal suppression assay are listed in Table 69.

#### 10.4 Step-by-step sample preparation: Murine polyclonal suppression assay

1. Single cell suspensions of lymph nodes or spleen of a wildtype or Foxp3- reporter mouse are subjected to negative selection of CD4 T-cells by magnetic beads (CD4^+^ T Cell Isolation Kit, Miltenyi Biotec).
2. Cells are then stained for 30 minutes at 4°C with Abs for CD3, CD4, CD11c, CD11b, CD25, B220 and Fixable Near-IR Dead Cell Stain and sorted on a BD Aria-II. Tregs are sorted as CD3^+^CD4^+^dump^−^GITR^+^CD25^+^ and confirmed to have a post sort purity of 90%+ (Figure 86B). Conventional T (Tconv) cells are sorted as CD3^+^CD4^+^dump^−^Foxp3^−^GITR^−^. Dump channel is CD11c, CD11b, B220 and dead cell stain. If a Foxp3 reporter mouse is available CD25 and Foxp3 can be used although if the reporter is GFP cell trace violet (CTV) rather than CFSE may be the optimal proliferation dye (Figure 86C).
3. CD4 Tconv are then stained with 1γM CFSE for 10 minutes in serum free RPMI media at RT. Excess CFSE is then quenched by addition of RPMI media+10%FCS before washing three times.
4. A total of 1×10^4^ Tconv cells per well are cultured with or without Treg cells at varied ratios (0:1, 1:1, 1:2, 1:4, 1:8 Treg:Tconv) for 3 days in the presence of 1×10^5^ γ-irradiated CD4 depleted APCs (18.5Gy irradiated CD4 depleted splenocytes obtained by magnetic separation in step 1) and 1γg/ml soluble anti-CD3 (Clone: 145-2C11) in 96 well U-bottomed plates, in RPMI media containing 10% FCS, 2-ME, L-glutamine and Penicillin/streptomycin with a final volume of 200μl. In all cases the number of Tconv is fixed while the number of Tregs is changed to obtain the intended ratios.
5. At the end of the three-day culture period, cells are then stained with anti-CD4, anti-CD25 and IR Live/Dead dye and data collected on a BD LSR Fortessa.

##### Materials: Murine polyclonal suppression assay

The materials for the murine polyclonal suppression assay are listed in Table 70.

#### **10.5** Step-by-step sample preparation: Human suppression assay of Ag-specific T cells

PBMCs are isolated from fresh heparinized blood by density gradient centrifugation with Lympholyte (Cedarlane, Burlington, Canada).

**Figure 87.**
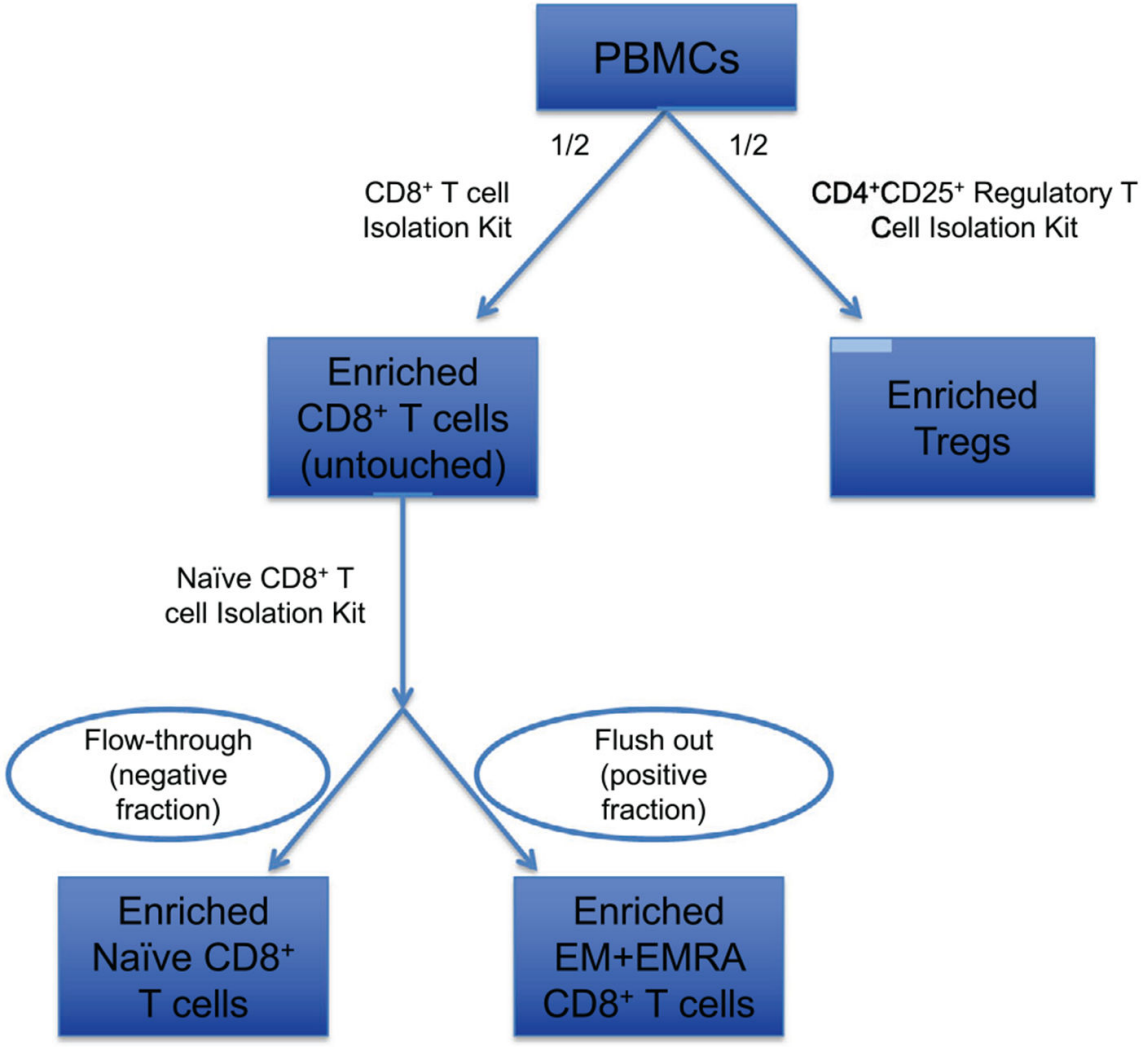
Flow chart illustrating steps of cell subsets isolation from human PBMCs. Flow chart illustrating steps of cell subsets isolation. A portion of PBMCs is used for enrichment of CD8^+^ T cells, another portion is used for enrichment of Tregs. The enriched CD8^+^ T cells fraction (untouched) is used for isolation of Naïve (negative fraction) and EM+EMRA (positive fraction) CD8^+^ T cells, with Naïve CD8^+^ T cell isolation kit.

**Figure 88.**
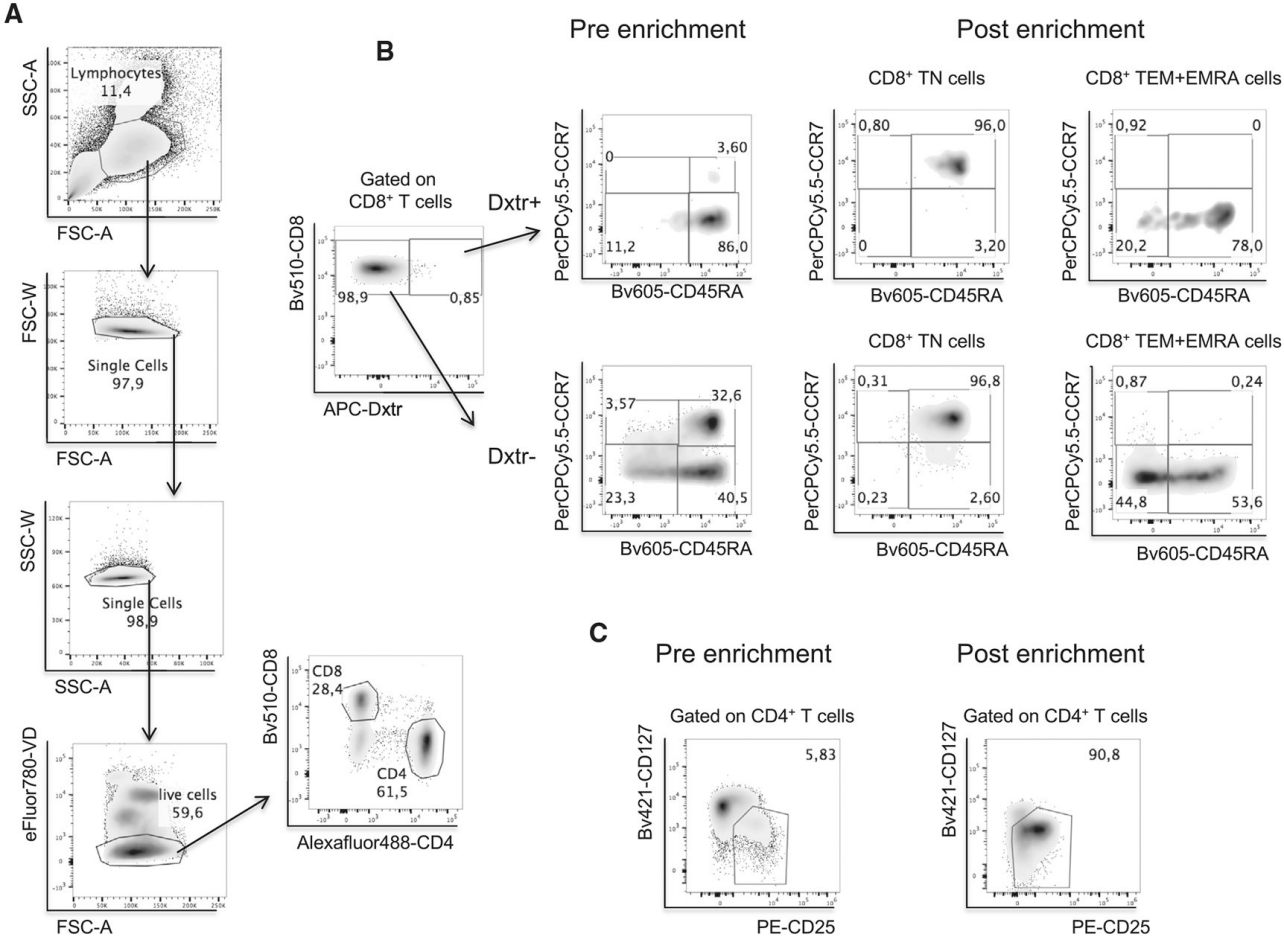
Gating strategy of cell subsets from human PBMCs. (A) Representative flow cytometry analysis of the gating strategy applied for the identification of CD8^+^ and CD4^+^ T cells from human PBMCs. Briefly, lymphocytes were first gated by the physical parameter Forward and Side scatter area (FSC-A and SSC-A) and doublets and debris were eliminated by plotting the width against the area of FSC and SSC parameters (FSC-W and SSC-W). Dead cells were excluded using Viability Dye (VD), and gating into live cells we identified CD8^+^ and CD4^+^ T cells. B) Representative FC analysis of pre- and post-enrichment of naïve (N) or effector memory plus effector memory RA^+^ (EM+EMRA) CD8^+^ T cells, gated on (dextramer^+^)-CD8^+^ T cells (upper) or (dextramer^−^)-CD8^+^ T cells (lower). (C) Representative FC analysis of pre- and post-enrichment of Treg cells with magnetic beads.

**Figure 89.**
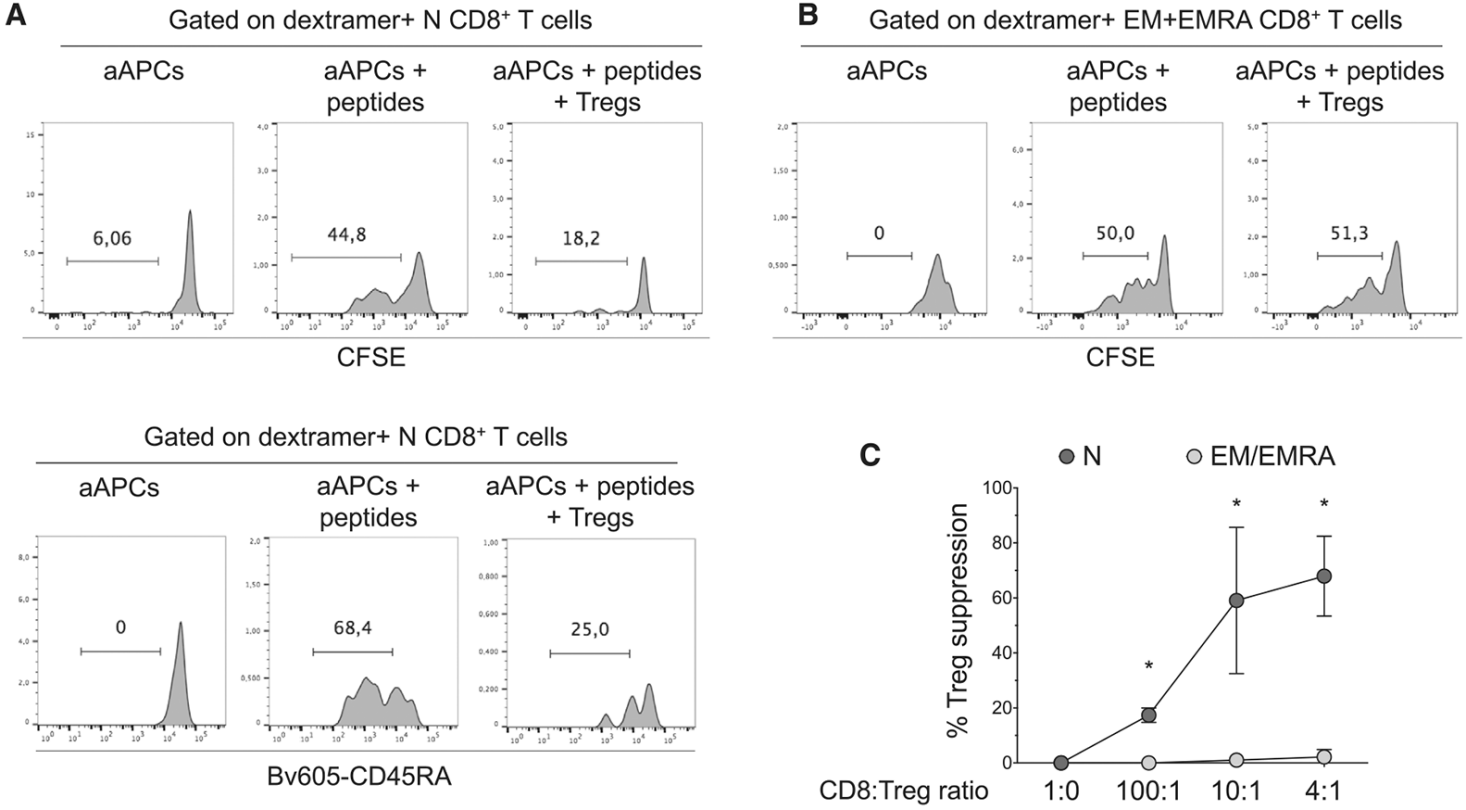
Human suppression assay of Ag-specific T cells. Representative histograms of purified CFSE-stained CD8^+^ T (N) cells (A) or effector memory plus effector memory RA^+^ (EM+EMRA) CD8^+^ T cells (B), from human PBMCs, stimulated with autologous APCs (aAPCs) pulsed (or not) with 20 μg/ml of peptides (aAPCs + peptides) and co-cultured (or not) with Treg cells at a CD8:Treg ratio of 10:1 for 7 days. Histograms indicate the percentage of cell proliferation (as detected by CFSE dilution) and differentiation (as detected by CD45RA downregulation) in (dextramer^+^)-CD8^+^ T cells. C) Mean values of four independent suppression assays at different CD8:Treg ratios. %Treg suppression = (MFI CFSE-stained dextramer^+^ CD8^+^ T cells with Treg cells – MFI CFSE-stained dextramer^+^ CD8^+^ T cells without Treg cells) / (MFI CFSE-stained dextramer^+^ CD8^+^ T cells unstimulated − MFI CFSE-stained dextramer^+^ CD8^+^ T cells without Treg cells) x 100. *p < 0.05 one-way ANOVA with Tukey’s multiple comparison test.

1. CD8^+^ T cells are pre-enriched from PBMCs with the corresponding CD8^+^ T Cell Isolation Kit (Miltenyi Biotec, Bergisch Gladbach, Germany) and then highly purified CD8^+^ T naïve (Tn CD8; CCR7^+^CD45RA^+^) cells are enriched from CD8^+^ T cells by magnetic bead-separation with the Naïve CD8^+^ T Cell Isolation Kit (Miltenyi Biotec). The combination of highly purified CD8^+^ T effector memory (EM; CCR7^−^CD45RA^−^) and effector memory RA^+^ (EMRA; CCR7^−^CD45RA^+^) cell population is obtained by using the positive fraction after enrichment of Tn cells. Treg cells are isolated from PBMCs with the CD4^+^CD25^+^ Regulatory T Cell Isolation Kit (Miltenyi Biotec) (Figure 87). Each purified cell subset is used in the various experiments only when the purity of the corresponding cells is >96% and 90% for T cell populations and Treg cells, respectively (Figure 88 A, B and C).
2. Highly purified autologous CD8^+^ T cell subpopulations (isolated as described above) are labeled with 10 μM of CFSE (Thermo Fisher Scientific, Massachusetts, USA) for 15 min at 37°C in RPMI complete medium containing 10% fetal bovine serum (FBS) (up to 10×10^6^ cells/ml). To quench the reaction, an isovolume of cold FBS is added and cells are washed twice.
3. Then, they (500,000-1 × 10^6^/well) are cocultured with both autologous γ-irradiated (70Gy) PBMCs as APCs (at a 1:1 ratio), which had previously been pulsed or not with 20 μg/mL of Ag or peptide(s) (self-peptides sequences detailed in Table 71) plus 1 μg/mL of anti-CD28 mAb, and highly purified Treg cells, which had previously been stained with 5 μM of CellTrace Violet (Cell Proliferation Kit, Thermo Fisher Scientific) at different CD8^+^ T cell:Treg cell ratios (100:1, 10:1, 4:1, and 1:0), in RPMI medium containing 5% human serum AB, 2mmol/L l-glutamine, penicillin/streptomycin, non-essential amino acids and sodium pyruvate, in 48-well plate (0,5-1mL/well). The number of CD8^+^ T cells is changed while the number of Tregs is fixed. Cells are cultured for 7 days, and half of the medium is replaced with fresh medium containing 20 IU/mL of IL-2 at day 4.
4. Cells are stained with Fixable Viability Dye eFluor780 for exclusion of dead cells in PBS 30 minutes at room temperature. After washing, cells are incubated with the pool of Ag-presenting-labeled-multimers of MHC class I molecules complexed with the relevant peptides, in PBS containing 2% FBS at room temperature for 10 minutes. Surface staining are performed incubating cells with labeled mAbs to CD8, CD4, CCR7, CD45RA and with a cocktail of labeled mAbs to CD14, CD16, CD56, CD19, (dump channel was included for the exclusion of monocytes, NK cells, and B cells, respectively) for 20 min at 4°C. After washing, cells are fixed and permeabilized using the FOXP3/Transcription Factor Staining Buffer Set (eBioscience, Massachusetts, USA) at 4°C for 30 minutes, washed, and then stained with mAbs to FOXP3 for 30 minutes at room temperature (Ab details reported in Table 72) (Figure 89A A, and B). All the incubations are performed in the dark. In the representative experiments shown in the Figure 89, as multimers of MHC class I molecules, we used APC-labeled-HLA-A*0201 dextramers complexed with self-peptides (MYH9^478-486^, MYH9^741-749^, VIME^78-87^, VIME^225-233^, ACTB_266-274_) (Immudex, Copenhagen, Denmark) to detect autoreactive CD8^+^ T cells in various forms of autoimmune diseases [870]. The percentage of Tregmediated suppression is calculated using the following formula: %Treg suppression = (MFI CFSE-stained dextramer^+^ CD8^+^ T cells with Treg cells – MFI CFSE-stained dextramer^+^ CD8^+^ T cells without Treg cells) / (MFI CFSE-stained dextramer^+^ CD8^+^ T cells unstimulated − MFI CFSE-stained dextramer^+^ CD8^+^ T cells without Treg cells) x 100 (Figure 89C).

#### 10.6 Step-by-step sample preparation: Human killing assay of Treg cells by Ag-specific CD8^+^ T effector cells

**Figure 90.**
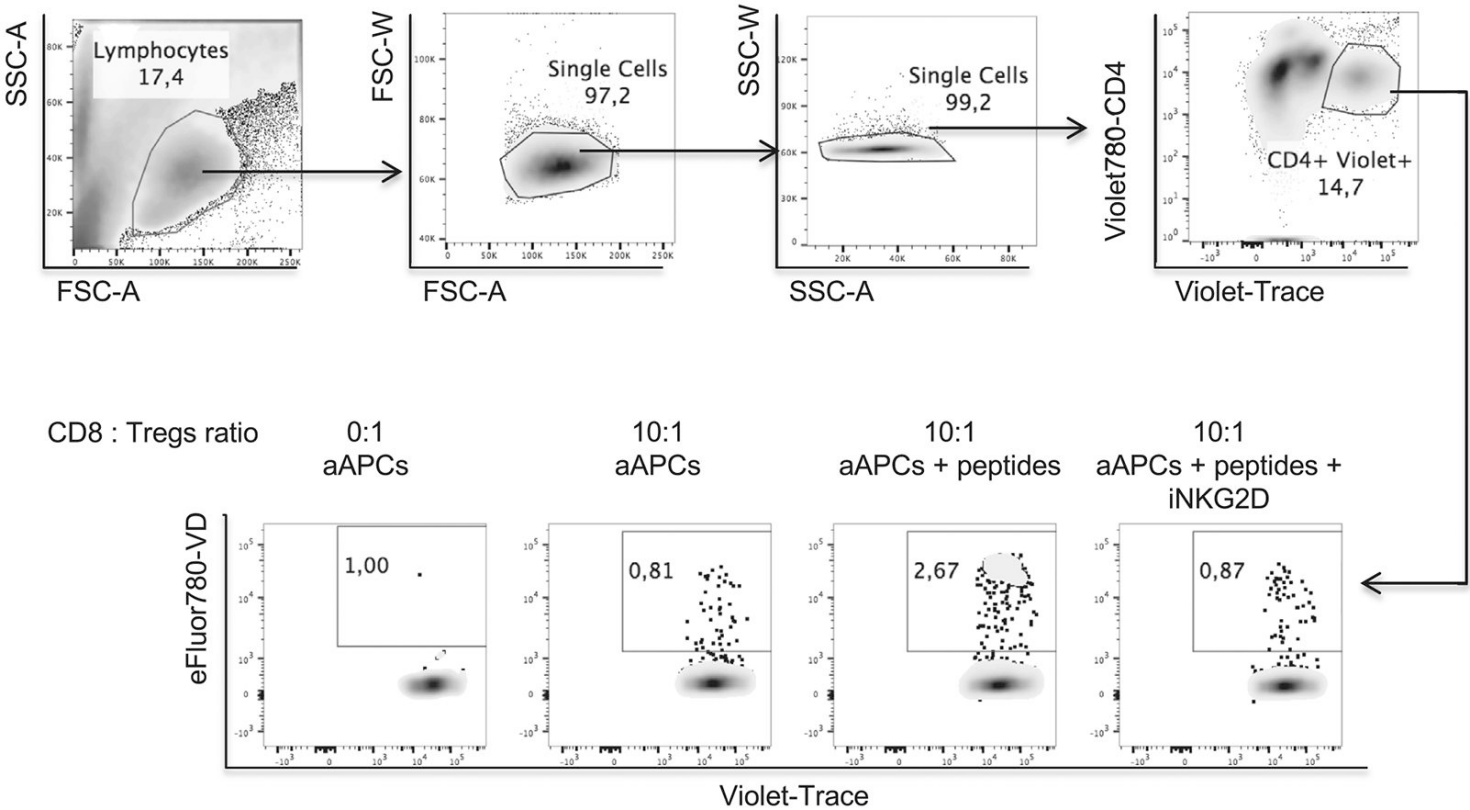
Human killing assay of Treg cells by Ag-specific CD8^+^ T effector cells. Representative FC analysis of dead Tregs (isolated from human PBMCs), as detected by the percentage of VD+ cells in Tregs, alone (0:1) or co-cultured with purified CD8^+^ Tem + EMRA cells (10:1) and aAPCs stimulated or not with peptides in the presence or absence of iNKG2D. aAPCs: autologous APCs.

1. Highly purified CD8^+^ T, Tem+EMRA (effectors), or TN cells are stained with 10 μM of CFSE and co-cultured with autologous γ-irradiated (70Gy)-PBMCs (1:1 ratio), which had previously been pulsed (or not) with 20 μg/mL of Ag or peptide(s) plus 1 μg/mL of anti-CD28, and highly purified autologous or allogeneic target cells (purified T cells, Treg cells, or others), which had previously been stained with 5 μM of CellTrace Violet (CellTrace Cell Proliferation Kit).
2. CD8^+^ T cells and target cells are co-cultured (or not) at a ratio of 10:1 for 7 days in complete RPMI medium containing 5% human serum AB, as previously described; at day 3, half of the medium is replaced with fresh medium plus 20 IU/mL of IL-2.
3. To investigate the granzyme B (GZMB)-mediated killing effect of CD8^+^ Tem+EMRA on targets, the assays are performed in the presence of GZMB inhibitor (Santa Cruz Biotechnology, Dallas, USA) or NKG2D neutralizing Ab (R&D Systems, Minneapolis, USA). Specifically, target cells are treated (or not) with 20 μM of GZMB inhibitor for 1 hour at 37°C, and CD8^+^ Tem+EMRA cells are treated with 1 μg/1 × 10^6^ of NKG2D neutralizing Ab for 15 min at room temperature. Cells are stained with Fixable Viability Dye eFluor780, APC-labeled-HLA-A*0201 multimers complexed with the relevant peptides (previously described), labeled mAbs to CD8, CD4, CCR7, CD45RA and with a cocktail of labeled mAbs to CD14, CD16, CD56, CD19 (dump channel was included for the exclusion of monocytes, NK cells, and B cells, respectively) for 20 min at 4°C. After washing, cells are fixed and permeabilized for the subsequent intra-nuclear staining with mAb to FOXP3, as previously described (Ab details reported in Table 72) (Figure 90).

#### 10.7 Materials: Human suppression assay of Ag-specific T cells and Human killing assay of Treg cells by Ag-specific CD8^+^ T effector cells

This is detailed in Tables 71 and 72.

#### 10.8 Data Analysis

There are several possible approaches to analyzing proliferation data. A common approach is to place a gate based on the non-divided peak measuring the % of cells that have divided at least once. This method has the benefit of simplicity and is commonly used. However, this method is also insensitive as it fails to take into account the number of divisions undertaken by the dividing cells. For example, if two populations have 75% that have divided at least once but the first has most cells in the second peak and the second has most cells in the fourth peak, then this method will report the same result despite their being a clearly observable difference in proliferation.

**Figure 91.**
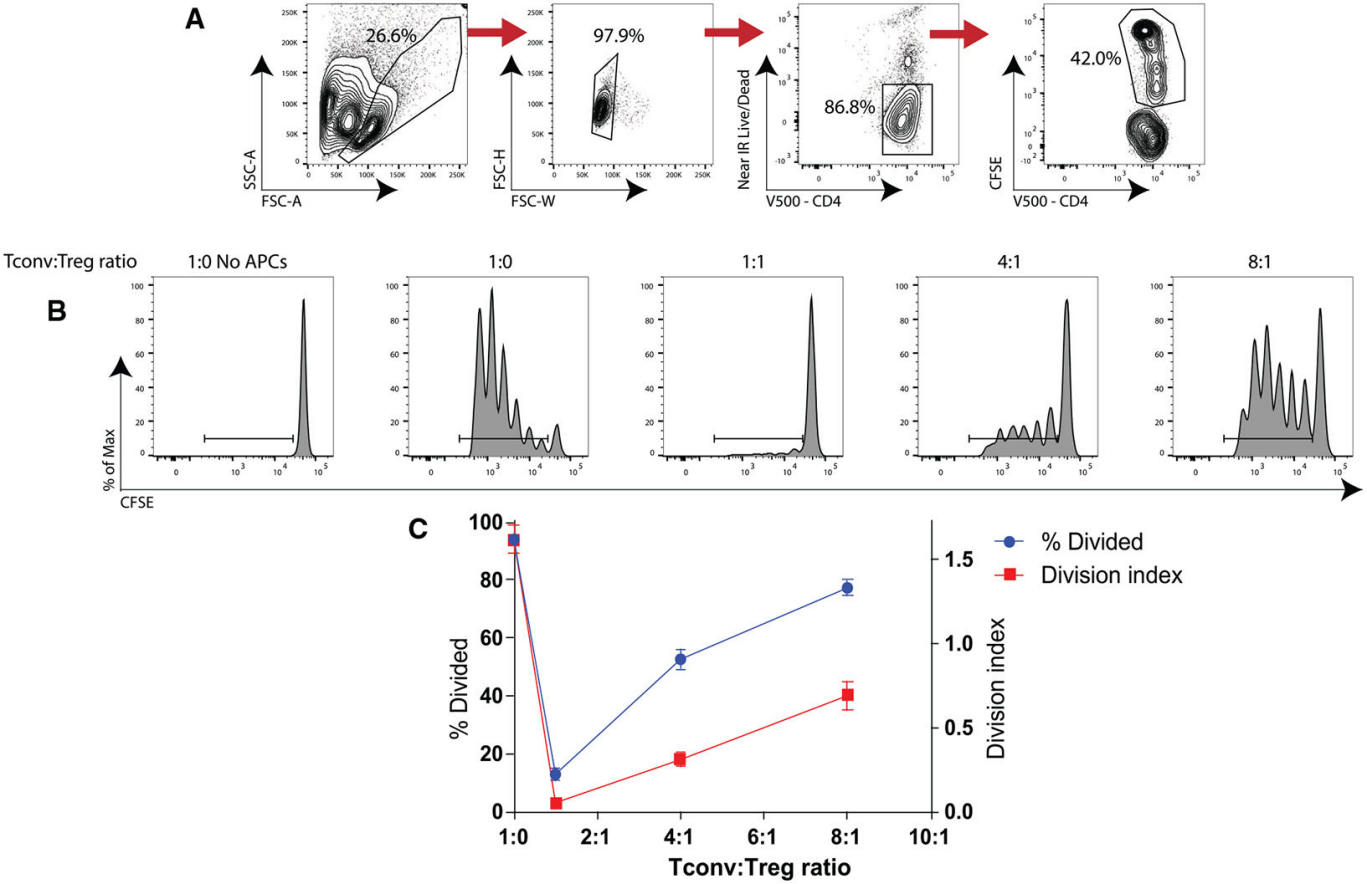
Analysis of human polyclonal suppression assay. (A) Gating strategy for identification of responder cells in human polyclonal suppression assay. (B) Proliferation histograms of human Tconv cells cultured with various ratios of Treg cells, irradiated CD4^−^ splenocytes, and anti-CD3 for three days. C) Summary data comparing % divided and division index of Tconv cells performed in duplicate. Division index is the average number of divisions by each cell as calculated in FlowJo Software.

Modeling of the peaks to calculate the total number of cell in each peak allows the use of more sensitive measurements such as division index (the average number of divisions by each cell) or proliferation index (the average number of divisions undertaken by each dividing cell) [873]. It should be noted that different software uses the terms division index and proliferation index with differing definitions, so they should always be clearly defined when used, the division index used here was calculated by FlowJo software. When both % divided and Division index are used to measure proliferation in the same population, it may be seen that while the results are broadly similar, division index is able to measure appreciable suppression at low Treg ratios which are less clearly different when using % divided (Figure 91).

**Figure 92.**
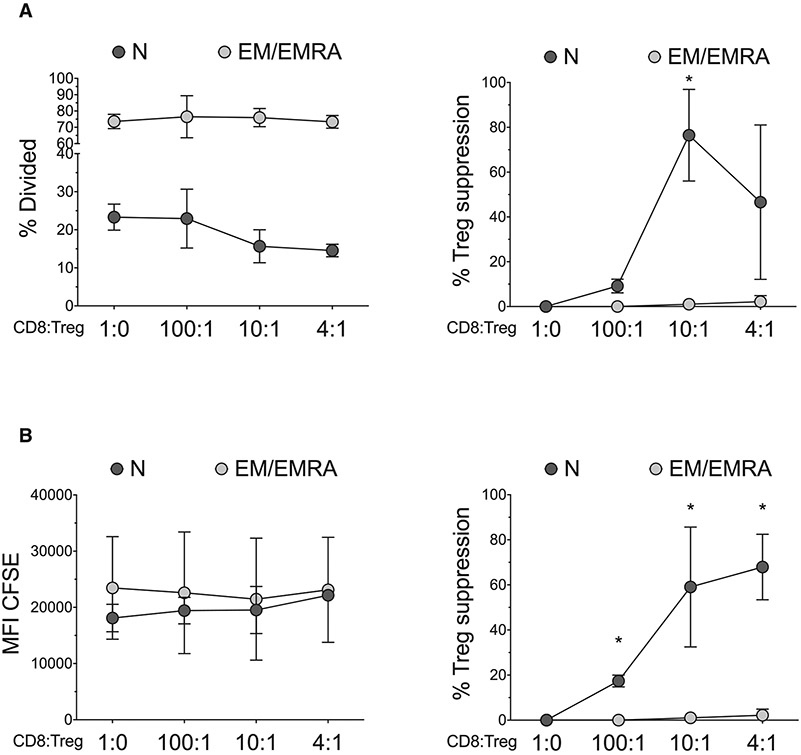
Analysis of proliferation in human suppression assays of Ag-specific T cells. Comparison between different methods to analyze proliferation in suppression assay of Ag-specific T cells, from human PBMCs, (as described in Figure 4); mean values of four independent experiments are reported. A) Left panel shows % divided of N or EM+EMRA (dextramer^+^)-CD8^+^ T cells, at different CD8:Treg ratio. Right panel shows %Treg suppression calculated using % divided T cells (see formula reported in Figure 4C). B) Left panel shows MFI of CFSE of N or EM+EMRA (dextramer^+^)-CD8^+^ T cells, at different CD8:Treg ratio. Right panel shows %Treg suppression calculated using MFI of CFSE (see formula reported in Figure 4C). *p < 0.05 one-way ANOVA with Tukey’s multiple comparison test.

In the assay calculating the %suppression of Ag-driven T cells (Figure 89), the resulting T cell proliferation can be detectable by using the MFI of CFSE-stained T cells better than by using % of divided T cells or the division index. Indeed, because of the tiny number of T cells specific to a given epitope, they are less synchronous as compared with polyclonal T cells stimulated with anti-CD3/anti-CD28, in which the high number of proliferating cells allow to define peaks and to distinguish their generations (see Figure 91) [873]. Furthermore, the different Ag-specific cell subsets (e.g., naïve or effector T cells) display a striking difference in their baseline proliferation (without Treg) (Figures 89 and 91). Figure 92 shows the difference between %suppression calculated using %divided T cells (A), and % of suppression calculated using MFI CFSE (B) (as reported above and in Figure 89C).

#### 10.9 Pitfalls

Care must be taken with the timing of the assay to ensure that the cells do not proliferate to the extent that they completely lose the proliferation dye. This will both make it impossible to resolve any proliferation past this point but also risk mixing up the responder and suppressor populations which are often separated on the basis of the proliferation dye. To an extend inclusion of further stains such as CD25 and Foxp3 may help resolve populations but these may also be upregulated by proliferating Tconv cells. If this proves a problem for mice, this can be resolved by using congenic markers such as CD45.1 Tconv and CD45.2 Treg. CD8 T-cells can also be used as responders.

It should be remembered that suppressive function is not totally Treg exclusive. Activated non-Treg cells are capable of showing some CTLA-4 dependent suppressive function, although this is relatively weak in comparison to Treg cells. As a result, in some cases inclusion of known highly-suppressive and non/lo-suppressive cells as control groups to allows placement of the cell population of interest on this scale.

To a large extent, the Ag-presenting cell-dependent suppression assay measures CTLA-4 dependent suppressive function. However, this is context-dependent, naïve CTLA-4 deficient Treg lack detectable suppressive function, while highly activated CTLA-4 deficient Tregs are suppressive due to upregulation of other suppressive molecules post-activation [869]. Another common variant of this assay is to use anti-CD3 and anti-CD28 beads in place of APCs, this APC-independent assay largely measures CTLA-4 independent suppressive function.

The use of CD3 in the sorting strategy (Figure 86) runs the risk of causing pre-activation of the T-cells. We have not found this to be a problem, but if this is a concern CD3 can be omitted without a major change in the purity of the sorted cells.

As regards the killing assay of Treg cells by Ag-specific CD8^+^ T effector cells, care must be taken to ensure that Treg cells do not display cytotoxicity activity, as suggested in older reports [874]. This risk can be ruled out by the evidence showing that: (i) highly purified peripheral Treg cells, as well as Treg cells infiltrating inflamed tissues, completely lack GZMs, in contrast to CD8^+^ T effector cells; (ii) highly purified Treg cells are unable to kill Ag-specific CD8^+^ T effector cells in cytotoxicity assays *in vitro* [870]. S. Koristka et al, proposed that the discrepancy with older reports [874] is due to the purity of Treg cells used in the assays [875].

#### 10.10 Top tricks

CFSE and CTV dyes can both be used and we have not observed clear differences in results between them. When a Foxp3-GFP reporter mouse is used, CTV may be the optimal choice to avoid mixing up the signal from GFP and CFSE. However, when this is not the case and blue, yellow-green, and violet lasers are all available, we find that CFSE frees up the bright fluorochrome BV421 while the blue and yellow-green lasers allow better separation of CFSE and PE than would be possible with just a blue laser. In some cases, high doses of CFSE can be toxic, and the effect in a particular setting should be defined empirically.

Choice of Antigen-presenting cells: With optimization, it is possible to use various cells as Antigen-presenting cells. In the murine system, we have successfully used CD11c^+^ DCs, B220^+^ B-cells and T-cell depleted splenocytes as Antigen-presenting cells. For the human suppression assay, monocytes-derived DCs and T-cell depleted PBMCs have both proven effective. There is not necessarily one correct choice but some consideration should be given to which cell type is appropriate according to the experiment in question. Irradiation of the Antigen-presenting cells is not always critical, and we have previously also used live murine B-cells [872]. Irradiation will cause the Antigen-presenting cells to die over several days preventing their overgrowth and nutrient depletion of the media but will also potentially affect immediate Ag presenting and costimulatory behavior. As such this is another variable to consider testing if initial results are suboptimal.

Since dead cells are often non-recoverable or excluded from analysis, counting the total number of recovered Tconv cells can also be useful to understand the dynamics of the suppression system. Cytometric counting beads can be used in order to accurately count the cells while collecting proliferation data.

Addition of exogenous IL-2: Particularly for humans, some donors Tconv may proliferate poorly even in the absence of Tregs. In this case, addition of a low dose of exogenous IL-2 in the range of 10-20 IU/ml may aid proliferation while also allowing clear suppression. Careful titration is needed as higher doses of IL-2 overwhelm Treg suppressive function.

The use of a pre-enrichment bead sorting is not essential but improves the purity of the sorted populations. This is more important when rare populations such as fraction I naïve Tregs are sorted.

#### 10.11 Clinical relevance statement

These assays are generally applicable in monitoring the suppression of polyclonal or Ag-specific T cell responses in various settings. For instance, during acute or chronic infections as well as autoimmune diseases, defects in suppressive function measured by these assays may be related to disease progression. For example, loss suppressive function is seen in Tregs derived from individuals with CTLA-4 mutations [876]; while effector, but not naïve, CD8 T-cells are able to resist suppression by direct killing of Tregs, a factor in their ability to evade Treg control in rheumatoid arthritis [870].

### 11 Measurement of T cell proliferation by the T_DS_ assay

#### 11.1 T_DS_ assay for human blood T cells

##### 11.1.1 Overview.

We developed a flow cytometry T cell cycle assay based on Ki-67/DNA dual staining, with the power to reveal *in vivo* T cell proliferative dynamics, complementing the description of T cell phenotypes, and without the need for *in vitro* T cell restimulation [877-879]. The key benefit of our method is that it can discriminate whether Ag-specific T cells in the PB are memory cells from previous responses, or cells actively engaged in an ongoing response. Specifically, our method enabled us to discover rare T cells in the S-G_2_/M phases of the cell cycle in non-leukemic individuals. We collectively termed these cells “T Double S” for T cells in S phase *in Sanguine*: in short “**T**_DS_” cells [878]. T_DS_ cell determination required the development of a refined strategy for flow cytometry data analysis, such that T_DS_ cells were neither excluded from conventional lymphocyte gates nor confused with cell doublets. In our hands, the T_DS_ assay provided useful information on ongoing T cell responses in Infectious Mononucleosis, Type 1 Diabetes, and COVID-19 [878, 880, 881]. We describe here the T_DS_ assay, and advocate its incorporation into routine blood immuno-monitoring in a variety of settings.

##### 11.1.2 Introduction.

Current flow cytometry methods for assaying Ag-specific human blood T cells *ex vivo* mostly rely on MHC-peptide multimer binding and/or cell marker expression to define cell phenotype (e.g., activated, effector, memory, exhausted, etc.) (For Ag-specific T cell staining by MHC multimers, see Chapter IV “T cell assays”, Section 3 “Antigen-specific T cell cytometry: MHC-multimers”). Expression of the intranuclear marker Ki-67 is commonly used in combination with T cell phenotype analysis to identify a proliferative state [882, 883]. (For Ki-67 and T cell phenotype, see Chapter III. “T cell phenotypes”, Section 2 “Murine conventional αβ T CD4 T cells”, paragraph 2.2 “Introduction”, sub-paragraph 2.2.2 “Conventional αβ CD4 T cells: transcription factors, effector functions, and Ag-specificity”; and Section 5 ”Human tissue resident memory T cells”, paragraph 5.9 “Summary Phenotype tables”, Table 12). An advantage of the Ki-67 marker is that it offers a “snapshot” of the cell cycle state of a cell, thus providing a “static” measurement at the time of blood sampling. By contrast, other methods determine proliferation over a time interval prior to the analysis. These so-called “dynamic” methods are rarely used in humans, as they require *in vivo* treatment with drugs and/or cell transfer [884]. An exception is labeling with deuterated water and gas chromatography-mass spectrometry analysis, which in combination with mathematical modeling provided fundamental information on memory CD8 T cell turn-over and differentiation [885].

It is often overlooked that Ki-67 is expressed in all phases of cell cycle, i.e., in G_1_, S, G_2_, M, while being undetectable in the quiescent phase G_0_. We recently raised the issue that using Ki-67 as a single proliferative marker might be misleading, as a Ki-67^+^ cell in G_1_ can be either actively cycling or in a post-mitotic phase on its way to quiescence [886]. Indeed, the G_1_ phase is a cell cycle hub of variable duration. Thus, Ki-67^+^ cells in G_1_ can remain in this phase for either a short or a prolonged time, and then proceed into DNA replication and cell division, or rather exit the cell cycle and move into G_0_. Taking into account the half-life of the Ki-67 protein, it becomes clear that Ki-67 is unreliable on its own to define proliferating cells [886].

**Figure 93.**
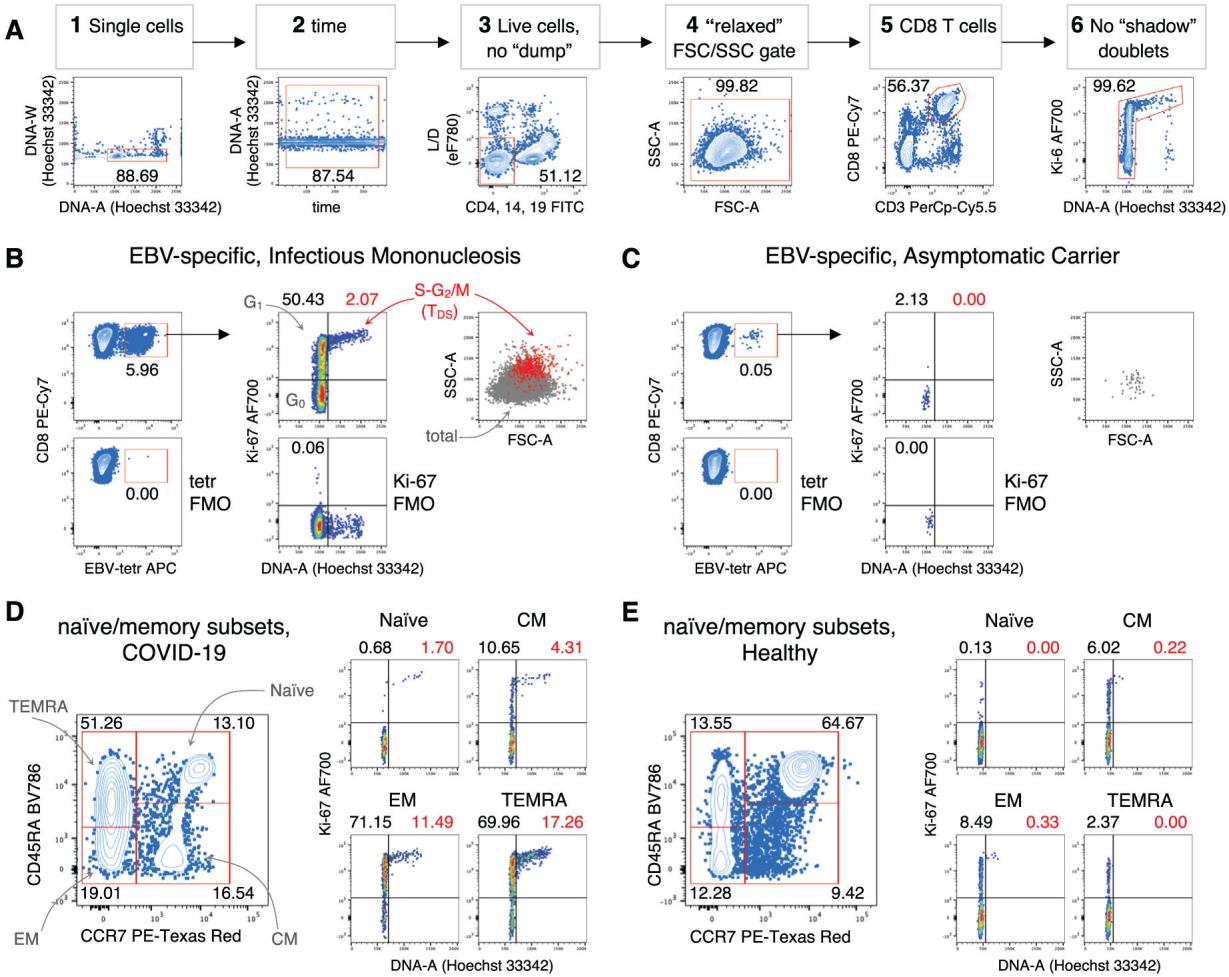
T_DS_ assay for analysis of human blood CD8 T cells. (A) Gating strategy. Example of viable single CD8 T cell gating in 6 steps: **1) Single cells**. Single cells having 2n≤ DNA content ≤4n were selected on the DNA-Area (A) versus (vs) DNA-Width (W) plot; **2) Time**. Stable acquisition over time (seconds) was monitored on the time vs DNA-A plot and any events collected in case of pressure fluctuations were excluded; **3) Live cells, no “dump”**. CD4^+^, CD14^+^, CD19^+^ cells were excluded on the “dump” channel, and live cells selected using the L/D eF780 dye; **4) “relaxed” FSC/SSC gate**. A “relaxed” gate was used on the FSC-A vs SSC-A plot, to include highly activated and cycling lymphocytes [877, 878]; **5) CD8 T cells**. CD8 T cells were gated on the CD3 vs CD8 plot. **6) No “shadow” doublets**. A few remaining doublets composed by one cell sitting on top of another (so called “shadow” doublets) were excluded as Ki-67^int^/^−^ events having > 2n DNA content [878]. Numbers indicate cell percentages in the corresponding gate. This gating strategy was used as a base for CD8 T cell analysis. **EBV-specific CD8 T cells in Infectious Mononucleosis** (B) **and Asymptomatic EBV-Carrier** (C). Example of CD8/ EBV-tetr plot showing EBV-tetr^+^ cell gate (**top left**) and tetr FMO control (**bottom left**). Cell cycle phases of EBV-tetr^+^ cells were defined on DNA-A vs Ki-67 plot as follows (**top center**): cells in G_0_ were identified as DNA 2n/ Ki67^−^ (bottom left quadrant); cells in G_1_ as DNA 2n/ Ki-67^+^ (upper left quadrant); cells in S-G_2_/M as DNA>2n/ Ki-67^+^ (T_DS_ cells, top right quadrant). Ki-67 FMO controls are shown (**bottom center**). Note that the “No shadow doublet” gate (Step 6 in A) cannot be applied to Ki-67 FMO samples. FSC-A/-SSC-A plot (**top right**), showing T_DS_ cells (in red) overlaid on total EBV-tetr^+^ cells (in grey). Gating strategy and mAb panel indicated in A. Unpublished data in relation to [878]. **Naïve/memory CD8 T cells in COVID-19 patients** (D) **and Healthy donors** (E). The following naïve/memory subsets of CD8 T cells were identified in the CCR7 vs CD45RA plot (**left**): CD45RA^+^ CCR7^+^ naïve, CD45RA^−^ CCR7^+^ (CM), CD45RA^−^ CCR7^−^ (EM), and CD45RA^+^ CCR7^−^ TEMRA. Cell cycle phases of each subset were analyzed as in B and C (**right**). Gating strategy and mAb panel as in A, except for using CD4 BV711 only in “dump” channel (step 3), and CD3 FITC and CD8 PerCp-Cy5.5 (step 5). Unpublished data in relation to [880].

We describe here a refined flow cytometry multicolor assay using MHC-peptide multimers and/or T cell markers in combination with dual Ki-67 and DNA staining for a sensitive discrimination of T cells in G_0_, in G_1_, and in the S-G_2_/M phases of cell cycle (For DNA staining and cell cycle analysis see Chapter V. “Biological applications”, Section 6. “DNA synthesis, cell cycle, and proliferation” in [22]. T_DS_ assay development was based on a previous method for cell cycle analysis of BM HSC [887], which we adapted for the assessment of Ag-specific CD8 T cell clonal expansion in a mouse model of vaccination ([877, 879], and see below IV. 11.2 T_DS_ assay for mouse blood T cells. In this context, we developed an unconventional multi-step gating strategy for T cell data analysis (Figure 93A). We exploited DNA-Area (A) versus DNA-Width (W) to exclude doublets and cell aggregates, and enlarged the lymphocyte gate on the FSC-A versus SSC-A plot to include blast-like T cells with high FSC/SSC. This strategy greatly increased the sensitivity of detection of Ag-specific CD8 T cells in a mouse immunization model, enabling the detection of blast-like Ag-specific CD8 T cells in S-G_2_/M phases of cell cycle in mouse PB at early times post-vaccination [877]. These rare blood T cells with high FSC and extremely high SSC represented clonally expanding T cells which likely migrated out of lymph nodes and spleen before completing the cell cycle. We immediately considered the implications of our discovery for the assessment of human T cell responses. Imaging flow cytometry helped us to refine our data analysis by adding a gate to exclude rare Ki-67^−^ events with high DNA content, that were imaged as cell doublets. These events had escaped previous exclusion steps, as they were formed by a cell sitting on top of another, thereby appearing like a shadow (“shadow” doublets) [878].

We describe here the T_DS_ assay protocol for human T cells, and show representative CD8 T cell results (Figure 93). We additionally used this method for cell cycle analysis of CD4 T cell subsets, Treg cells and γδ T cells, see data in [878, 880, 881].

##### 11.1.3 Step-by-step sample preparation.

###### 11.1.3.1 Cell isolation.

Collect heparinized blood samples and isolate PBMCs by Ficoll gradient centrifugation. PBMCs can be either immediately stained, or frozen in aliquots of 10-20x10^6^ cells. This protocol works with PBMCs stored in liquid nitrogen for several years (in our hands up to 15 years [878]).

###### 11.1.3.2 Cell staining.

####### Day 0 Surface staining

- Obtain fresh PBMCs or thaw previously frozen PBMCs at 37°C, wash in pre-warmed RPMI 1640 medium containing 5% human serum, and count cells.
- Transfer 2-4 x 10^6^ PBMCs in a 1.5 ml Eppendorf tube for each experimental sample. Include a sample to be stained only with the DNA dye Hoechst 33342 (for compensation), and an unstained sample (for flow cytometer set up). Prepare a 1:1 mix of heat-killed and live PBMCs, to be stained only with the Fixable Viability Dye eFluor 780 (L/D eF780, for compensation).
- Wash with 1 ml of FACS Buffer (DPBS with 2% human serum) pre-warmed at 37°C. Centrifuge at 400 × *g* for 5 min at RT, aspirate the supernatant, and leave the PBMC pellet in a residual volume ≤ 25 μl.
- Add 75 μl of a mix containing dasatinib (a protein kinase inhibitor, final concentration 50 nM) and CCR7 mAb (pre-determine appropriate dilution in titration experiments) in FACS Buffer, resuspend by pipetting, and incubate for 20 min at 37°C in the dark [dasatinib only required when performing MHC-peptide tetramer staining].
- Add 10 μl of APC-conjugated MHC-peptide tetramers diluted in FACS Buffer (pre-determine appropriate dilution in titration experiments), resuspend by pipetting, and incubate for 10 min at 37°C in the dark. Wash as above. Add 25 μl of anti-APC mAb (final concentration 10 μg/ml), resuspend by pipetting, and incubate on ice for 20 min in the dark [skip this step if not needed].
- Wash as above, using pre-chilled FACS Buffer at 4°C, and centrifuge at 4°C.
- Add 75 μl of surface mAb cocktail in FACS Buffer (mix of CD3, CD4, CD14, CD19, CD8, CD45RA, pre-determine appropriate dilution in titration experiments), and incubate for 20 min at 4°C protected from light. For each fluorochrome-conjugated mAb, prepare compensation beads (use Anti-Mouse Ig,κ/Negative Control Compensation Particles Set according to the manufacturer’s instructions) and perform incubation with mAb in parallel with experimental samples (beads for compensation).
- Wash as above, using DPBS at 4°C, and centrifuge at 4°C.
- Resuspend the cell pellet of each experimental sample, and of the heat-killed/live cell mix, with 50 μl of the L/D eF780 diluted 1:1000 in DPBS. Incubate 15 min at 4°C protected from light.
- Wash as above, with DPBS at 4°C, and centrifuge at 4°C.
- Add 200 μl of Fixation/Permeabilization Buffer at 4°C (prepare freshly this Buffer using Foxp3/Transcription Factor Staining Buffer Set according to the manufacturer’s instructions). Incubate for 16 h at 4°C protected from light.

####### Day 1 Intracellular staining

- Centrifuge at 400 × *g* for 5 min at RT, aspirate supernatant and wash 2 times with 1 ml of Permeabilization Buffer 1 × at RT (prepare freshly this Buffer using Foxp3/Transcription Factor Staining Buffer Set according to the manufacturer’s instructions). Leave the PBMC pellet in a residual volume ≤ 25 μl.
- Add 50 μl of Permeabilization Buffer 1 ×, and incubate for 15 min at RT protected from light.
- Add 50 μl of Ki-67 mAb diluted in Permeabilization Buffer 1 × (pre-determine appropriate dilution in titration experiments), and incubate for 30 min at RT protected from light.
- Wash 2 times with Permeabilization Buffer 1 × as above.

####### DNA staining

- Resuspend each cell pellet in 250 μl of DPBS at RT and transfer in a 5 ml polystyrene round bottom tube.
- Add 250 μl of Hoechst 33342 at 4 μg/ml (final concentration 2 μg/ml) and incubate for 15 min RT protected from light.
- Centrifuge at 400 × *g* for 5 min at RT, aspirate the supernatant, and resuspend the pellet with 350 μl of DPBS at RT.

##### 11.1.4 Materials.

RPMI 1640 Medium with L-Glutamine with Phenol Red (RPMI 1640 Medium) (Gibco 11875093),

Human serum (Sigma-Aldrich H4522),

DPBS w/o Ca^++^ and Mg^++^ (DPBS) (Gibco 14190144)

Anti-CD3 PerCp-Cy5.5 (clone SK7, Biolegend 344808),

Anti-CD3 FITC (clone UCHT1, BD 555332),

Anti-CD8 PECy7 (clone SK1, Biolegend 344712),

Anti-CD8 PerCp-Cy5.5 (clone SK1, BD 565310),

Anti-CD4 BV711 (clone SK3, BD 563028),

Anti-CD4 FITC (clone SK3, BD 345768),

Anti-CD14 FITC (clone M_φ_9, BD 345784),

Anti-CD19 FITC (clone HIB19, Biolegend 302256),

Anti-CCR7 PE-CF594 (clone 150503, BD 562381),

Anti-CD45RA BV786 (clone HI100, BD 563870),

Anti-Ki-67 AF700 (clone B56, BD 561277),

Anti-APC unconjugated (clone APC003, Biolegend 408002)

MHC-peptide tetramers: APC-conjugated HLA-A*02 EBV BMLF1_280-288_ tetramers (EBV-tetr) prepared as previously described [888],

Dasatinib (Axon Medchem 1392),

eFluor 780 Fixable Viability Dye (L/D eF780, eBioscience 65-0865-14),

Hoechst 33342, Trihydrochloride, Trihydrate, 10 mg/mL Solution in Water (Life Technologies H3570),

eBioscience^™^ Foxp3/Transcription Factor Staining Buffer Set (ThermoFisher 00-5523-00),

Anti-Mouse Ig,κ/Negative Control Compensation Particles Set (BD-Bioscience 552843),

Flow cytometer: BD LSR Fortessa equipped with 4 lasers (405-Violet, 488-Blue, 561-Yellow-green, 642-Red).

##### 11.1.5 Data analysis.

Analyze samples using a BD LSR Fortessa or a similar cytometer. Set Hoechst 33342 on a linear scale. For compensation, use beads for fluorochrome-conjugated mAbs and cell samples for Hoechst 33342 and eF780. For Hoechst 33342 compensation, set the negative on the G_0_/G_1_ peak and the positive on the G_2_/M peak (consider that the G_2_/M peak might contain cell doublets, which is not a problem for compensation). Acquire experimental samples at low flow speed. Process data using FlowJo software (Tree Star) or equivalent.

##### 11.1.6 Pitfalls.

- Hoechst 33342 spillover
  The DNA dye Hoechst 33342 is best detected with an UV laser, but it can be detected also with a violet laser. Be aware that there is a considerable spillover of Hoechst 33342 into the violet and UV channels, that is hard to compensate, preventing the use of several of these channels.
- Fluctuations in DNA fluorescence intensity
  Since DNA content is analyzed on a linear scale, possible fluctuations of its fluorescence intensity from one sample to another are immediately evident, and might create problems during acquisition and/or analysis. Fluctuations might depend on either difference in cell number/concentration during incubation with the DNA dye Hoechst 33342 (make sure to use always the same cell number and volume of incubation in all samples), or fluorescence intensity decline over time (perform cell incubation with Hoechst 33342 on a few samples at a time, just before acquisition at flow cytometer). In our hands, slight DNA fluorescence intensity fluctuations are unavoidable, possibly due to minor difference in cell loss from one sample to another during washes.

##### 11.1.7 Top Tricks.

- Staining with L/D dye
  Considering the possibility that the Day 0 procedures might kill a few cells in experimental samples, especially in thawed PBMC samples, we performed the L/D eF780 staining step just before cell fixation at the end of Day 0. In our hands, this trick appropriately stained dead cells without increasing background fluorescence.
- Tetramer staining
  We used a protocol for enhanced tetramer staining, which was especially designed for the detection of low-affinity TCR [684, 889, 890]. In our hands, this method appropriately stained islet-, EBV- and CMV-reactive CD8 T cells [878].
- Gating strategy and analysis
  Data analysis is an essential component of the T_DS_ assay. In contrast to the gates based on fluorochrome-conjugated mAbs, the gates relying on DNA content (see Figure 93, Step 1 and 6 of gating strategy, and DNA/Ki-67 plots) might need to be adjusted to each sample, due to fluctuations in DNA fluorescence intensity (see above). Make sure to carefully check these gates in each sample, and adapt them as needed. Make sure to use consistent criteria across samples, and/or across T cell subsets in the same sample.

##### 11.1.8 Clinical relevance statement.

We showed that the percentage, Ag-specificity and naïve/memory marker expression of actively proliferating T cells in the S-G_2_/M phases of the cell cycle (“T_DS_” cells) in PB vary from patient-to-patient, providing useful insights into their clinical condition [878, 880, 881]. For example, EBV-specific CD8 T cells from Infectious Mononucleosis patients were enriched in T_DS_ cells, in contrast to asymptomatic EBV-carriers (Figure 93B-C and [878]). In Type 1 Diabetes patients, the T_DS_ assay was instrumental to reveal that an increased proportion of proliferating PB CD8 T cells specific for pancreatic islet self-Ags was associated with powerful effector potentials [878]. The utility of the T_DS_ assay was particularly apparent for COVID-19 patients, where severe disease was associated with abundant CD8 and CD4 T_EM_ cells in G_1_ and in S-G_2_/M, and γδ T cells in G_1_ (figure 93D-E and [880]). Active T cell proliferation coexisted with signs of overt immune dysregulation, including profound T-lymphopenia, and activation/exhaustion marker expression by residual T cells [880]. The T_DS_ assay also revealed abnormal T cell cycling in COVID-19 patients with malignancies, especially pronounced in those with hematological cancers [881]. Even though in healthy donors the T_DS_ cells were rare, they were especially represented in Fraction II of Treg cells, suggesting ongoing immune regulation [878]. Altogether, our results bring strong arguments in favor of the often-contested view that appropriate PB immunomonitoring can provide important insights into a patient’s status, including tissue-based T cell responses.

#### 11.2 T_DS_ assay for mouse blood T cells

##### 11.2.1 Overview.

In the course of a mouse study on T cell response after vaccination, we developed a flow cytometry method for assessment of proliferating Ag-specific CD8 T cells in spleen, lymph nodes and blood, based on Ki-67/DNA dual staining and an *ad hoc* gating strategy for data analysis [877, 879]. Our assay was instrumental to measure vaccine-induced clonal expansion in spleen and lymph nodes, and to discover rare Ag-specific CD8 T cells in S-G_2_/M phases of cell cycle in mouse PB at early times after vaccination [877]. The estimated absolute number of Ag-specific CD8 T cells in S-G_2_/M in the blood was 20-25-fold lower than in the sum of spleen and draining lymph nodes, suggesting that actively cycling cells in the blood represented a “spillover” from lymphoid organs [877]. Even though CD8 T cells actively cycling in the blood scarcely contributed to the total pool of Ag-responding CD8 T cells, their presence offered an opportunity for detecting ongoing T cell responses by PB sampling.

We then confirmed the presence of T cells in S-G_2_/M phases of cell cycle in PB of human donors, and, on this occasion, refined the gating strategy by adding an additional gate for doublet exclusion, based on Imaging Flow Cytometry results [878]. We defined T cells in S-G_2_/M phases in the PB “T Double S” for T cells in S phase *in Sanguine* (“T_DS_” cells), and our refined method for their quantification “T_DS_ assay” [878] (see 11.1.1).

##### 11.2.2 Introduction.

We performed a mouse vaccination study using HIV-1 gag as a model Ag. In these experiments, mice were primed with ChAd3-gag and boosted with MVA-gag, both administered via the intramuscular route [877]. The T_DS_ assay enabled us to measure gag-specific CD8 T cells in S-G_2_/M in the blood at early times after either prime or boost. Notably, an extremely rare population of cells in S-G_2_/M among total blood CD8 T cells was consistently found in both untreated and vaccinated mice. In vaccinated mice, this population was enriched in gag-specific CD8 T cells during vaccine-induced clonal expansion [877]. Notably, the T_DS_ assay is tailored to detect these cells, in contrast to current flow cytometry criteria of lymphocyte analysis.

**Figure 94.**
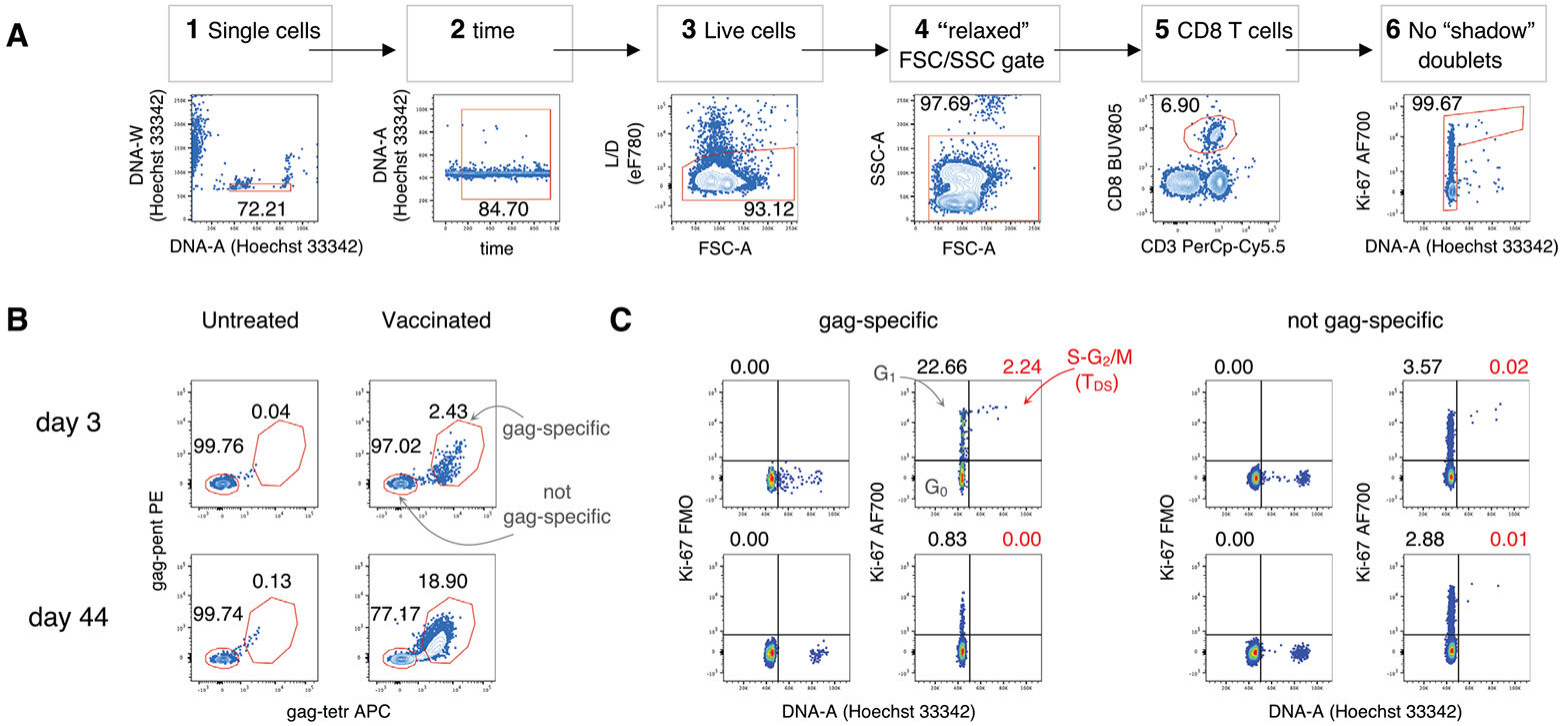
T_DS_ assay for analysis of mouse blood CD8 T cells. BALB/c mice were vaccinated against HIV-1 gag used as a model Ag by prime with ChAd3-gag and boost with MVA-gag, as described [877]. T_DS_ assay was performed on blood collected at day 3 and day 44 after boost. Blood from 3 mice was pooled to obtain enough cells for analysis. (A) **Gating strategy**. Example of gating of viable single CD8 T cells in 6 steps: **1) Single cells**. Single cells having 2n≤ DNA content ≤4n were selected on the DNA-Area (A) versus (vs) DNA-Width (W) plot; **2) Time**. Stable acquisition over time (seconds) was monitored on the time vs DNA-A plot and any events collected in case of pressure fluctuations were excluded; **3) Live cells**. Live cells were selected on the FSC-A vs L/D eF780 plot; **4) “relaxed” FSC/SSC gate**. A “relaxed” gate was used on the FSC-A vs SSC-A plot, to include highly activated and cycling lymphocytes [877]; **5) CD8 T cells**. CD8 T cells were gated on the CD3 vs CD8 plot. **6) No “shadow” doublets**. A few remaining doublets composed by one cell sitting on top of another (so called “shadow” doublets) were excluded as Ki-67^int^/^−^ events having > 2n DNA content [878]. Numbers indicate cell percentages in the corresponding gate. This gating strategy was used as a base for CD8 T cell analysis. (B) **gag-specific CD8 T cell frequency at day 3 and day 44 post-boost**. Example of gag-tetr/ gag-pent plots, showing “gag-specific” and “not gag-specific” cell gates in untreated (**left**) and vaccinated (**right**) mice at day 3 (**top**) and day 44 (**bottom**) post-boost, as indicated. (C) **Cell cycle of gag-specific and not gag-specific CD8 T cells from vaccinated mice at day 3 and day 44 post-boost**. Cell cycle phases of “gag-specific” (**left**) and “not gag-specific” (**right**) cells from vaccinated mice at day 3 (**top**) and day 44 (**bottom**) post-boost were defined on DNA-A vs Ki-67 plot as follows: cells in G_0_ were identified as DNA 2n/ Ki-67^−^ (bottom left quadrant); cells in G_1_ as DNA 2n/ Ki-67^+^ (upper left quadrant); cells in S-G_2_/M as DNA>2n/ Ki-67^+^ (T_DS_ cells, top right quadrant). Ki-67 FMO controls are shown. Note that the “No shadow doublet” gate (Step 6 in A) cannot be applied to Ki-67 FMO samples. Unpublished data in relation to [877].

We describe here the refined protocol for mouse blood T cell proliferation assessment and analysis by the T_DS_ assay [877], including its implementation following Imaging Flow Cytometry experiments [878], and advocate its incorporation in pre-clinical studies. An example of analysis of gag-specific CD8 T cells by the T_DS_ assay is shown in figure 94.

##### 11.2.3 Step-by-step sample preparation.

###### 11.2.3.1 Cell isolation.

Collect blood by intracardiac puncture in anesthetized BALB/c mice previously vaccinated against HIV-1 gag [877], or another model Ag, and immediately put into heparin or EDTA blood collection tubes. Harvest the spleen from a BALB/c mouse, and place it in a dish containing 3 ml DPBS (to prepare heat-killed/live cell mix for compensation of the Fixable Viability Dye eFluor 780, L/D eF780). Harvest the two femurs. Cut the extremities of each bone, and extract BM by centrifugation [879] (for Hoechst 33342 compensation). Prepare spleen and BM single cell suspension in DPBS, and count [879].

###### 11.2.3.2 Cell staining.

####### Day 0 Surface staining

Blood samples

- Transfer 200 μl blood in a 15 ml polypropylene tube for each experimental sample. Include an unstained blood sample (for flow cytometer set up).
- Add 2 μl of the L/D eF780.
- Add 20 μl of APC-conjugated MHC-peptide tetramers, 20 μl of PE-conjugated MHC-peptide pentamers, 10 μl of 2.4G2 mAb (to block non-Ag-specific binding of fluorochrome-conjugated mAbs to FcγRIII/FcγRII), and 20 μl of surface mAb cocktail (mix of CD3 and CD8 mAbs), all in FACS Buffer (DPBS with 1% BSA and 2 mM EDTA). Use appropriate dilutions, previously determined in titration experiments. Resuspend by pipetting, and incubate for 30 min at RT in the dark.
- Add 1.5 ml of BD CellFix 1 × freshly prepared according to the manufacturer’s instructions. Incubate 5 min RT protected from light.
- Centrifuge at 500 × *g* for 10 min at RT, and aspirate supernatant.
- Add 2 ml of BD Pharm Lyse 1 × freshly prepared according to the manufacturer’s instructions. Resuspend by pipetting, incubate 5 min RT protected from light.
- Centrifuge at 500 × *g* for 10 min at RT, and aspirate supernatant.
- Repeat the two steps above.
- Add 1 ml of Fixation/Permeabilization Buffer (freshly prepared using Foxp3/Transcription Factor Staining Buffer Set according to the manufacturer’s instructions). Incubate for 16 hours at 4°C protected from light.

Cells and beads for compensation

- Transfer a 1:1 mix of heat-killed and live spleen cells (3 x 10^6^ cells in total) in a well of a 96-well U-bottom plate, centrifuge at 400 × *g* for 3 min at RT, and discard the supernatant. Add 50 μl of the L/D eF780 diluted 1:500 in DPBS. Resuspend by pipetting, and incubate for 15 min at 4°C protected from light. Wash 2 times. Each time, add 0.2 ml of FACS Buffer, centrifuge at 400 × *g* for 3 min at RT, and discard supernatant (cells stained with L/D eF780 for compensation).
- Transfer BM cells (3 × 10^6^ cells) in a well of a 96-well U-bottom plate, centrifuge 400 × *g* for 3 min at RT, and discard the supernatant (cells to be stained with Hoechst 33342 only, for compensation)
- For each fluorochrome-conjugated mAb, prepare compensation beads (using Anti-Rat and anti-Hamster Ig,κ/Negative Control Compensation Particles Set according to the manufacturer’s instructions) and perform incubation with mAb for 30 min at RT in the dark in 96-well U-bottom plate. Wash 2 times. Each time, add 0.2 ml of FACS Buffer, centrifuge at 400 × *g* for 3 min at RT, and discard supernatant (beads stained with fluorochrome-conjugated mAbs for compensation).
- Add 0.2 ml of Fixation/Permeabilization Buffer (freshly prepared using Foxp3/Transcription Factor Staining Buffer Set according to the manufacturer’s instructions). Incubate for 16 hours at 4°C protected from light.

####### Day 1 Intracellular staining

Perform as day 1 described in 11.1.3.2, except for centrifuge setting (500 × *g* for 10 min at RT) and wash volume of Permeabilization Buffer 1 × (4 ml)

####### DNA staining

Perform as day 1 described in 11.1.3.2.

##### 11.2.4 Materials.

DPBS, L/D eF780, Hoechst 33342, and Foxp3/Transcription Factor Staining Buffer Set as in 11.1.4.

BSA (Sigma A07030)

Ethylenediaminetetraacetic Acid disodium salt solution (EDTA) (Sigma E7889)

BD Cell Fix, 10× concentrate (BD 340181),

BD Pharm Lyse, 10× concentrate (BD 555899),

anti-FcγRIII/FcγRII mAb (clone 2.4G2, BD 553141)

anti-CD3 PerCp-Cy5.5 (clone 145-2C11, BD 551163),

anti-CD8 BUV805 (clone 53-6.7, BD 564920),

anti-Ki-67 AF700 (clone SolA15, e-Bioscience 11-5698-82),

MHC-peptide multimers: AMQMLKETI APC-labeled Tetramer (Tetr-gag, NIH Tetramer Core Facility, Atlanta, GA) and PE-labeled Pentamer (Pent-gag, Proimmune, Oxford, UK) to stain for gag_197-205_ (gag)-specific CD8 T cells.

Anti-Rat and anti-Hamster Ig,κ/Negative Control Compensation Particles Set (BD-Bioscience 552845),

Flow cytometer: BD LSR Fortessa equipped with 5 lasers (355-Ultraviolet, 405-Violet, 488-Blue, 561-Yellow-green, 639-Red)

##### 11.2.5 Data analysis.

See above IV.11.1.5 Data analysis in T_DS_ assay for human blood T cells

##### 11.2.6 Pitfalls.

See above IV.11.1.6 Pitfalls in T_DS_ assay for human blood T cells

##### 11.2.7 Top tricks.

###### • Multimer staining

We used MHC-peptide tetramers and MHC-peptide pentamers conjugated with APC and PE, respectively, to increase the sensitivity of detection of mouse Ag-specific CD8 T cells. In our hands, this method appropriately stained gag-specific CD8 T cells in spleen, lymph nodes and blood of vaccinated BALB/c mice (figure 94 and [877]).

Gating strategy and analysis, see above IV.11.1.7 Top Tricks in T_DS_ assay for human blood T cells.

## V B cell phenotypes

### 1 Human B cells and their subsets

#### 1.1 Overview

B cells represent the Ab-producing cells developing from naïve B cells to Ab-secreting PC. One feature of B cells is their capacity to differentiate upon Ag dependent and independent stimulation to Ab secreting cells, also called plasma cells. In addition, B cells contribute to immunity as APCs as well as cytokine secreting cells. The stages of B cell differentiation share several common features between the human and the rodent immune system. In this section, we focus on human B cells.

#### 1.2 Introduction

**Figure 95.**
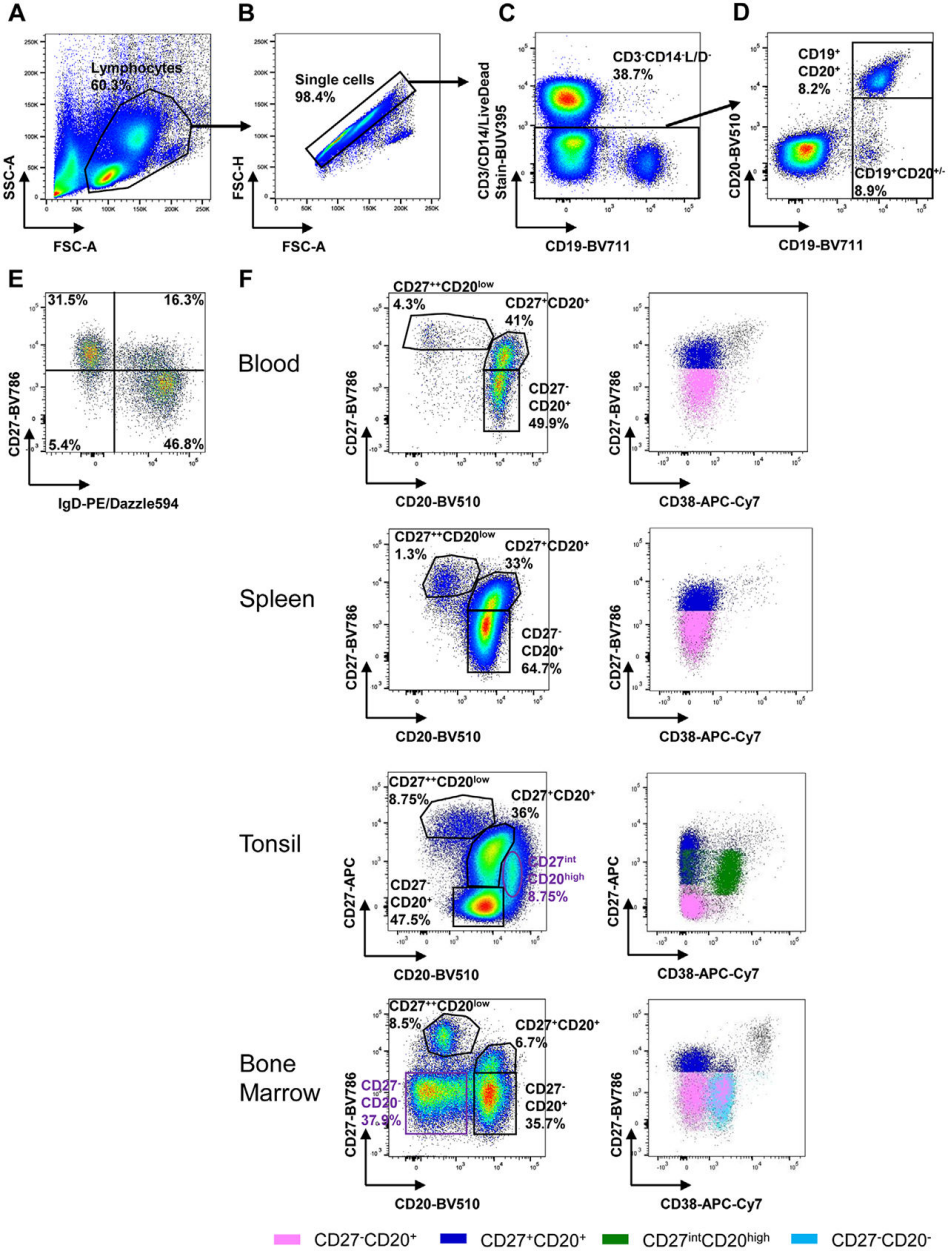
Gating strategy for the identification of human B cells. (A-E) Gating example for peripheral blood: (A) Lymphocytes are identified by their light scattering properties. (B) Exclusion of doublets. (C) Cells positive for CD3 and CD14 and DAPI stained dead cells are excluded. (D) B cells are identified by their expression of CD19 and CD20 including CD20^low^ plasmablasts. (E) B cell subsets, CD19^+^CD20^+^ gated, are discriminated by CD27 and IgD: CD27^−^IgD^+^ naïve B cells, CD27^+^IgD^+^ pre-switch memory B cells, CD27^+^IgD^−^ switched memory B cells, CD27^−^IgD^−^ B cells containing switched memory B cells. (F) B cell subsets, gated as CD19^+^CD20+/− can also be discriminated by CD27 and CD20 in peripheral blood, spleen, tonsil and bone marrow: conventional naïve B cells are CD27^−^ CD20^+^ (containing CD27^−^ memory B cells) memory B cells CD27^+^ CD20^+^ and plasmablasts CD27^++^ CD20^low^. Cell subsets defined by CD27 and CD20 expression were color-coded and depicted in a CD27 vs CD38 plot (pink: CD27^−^CD20^+^ B cells, dark blue: CD27^+^CD20^+^ B cells, green (only in tonsil): CD27^int^CD20^high^, turquois (only in bone marrow): CD27^−^CD20^−^).

**Figure 96.**
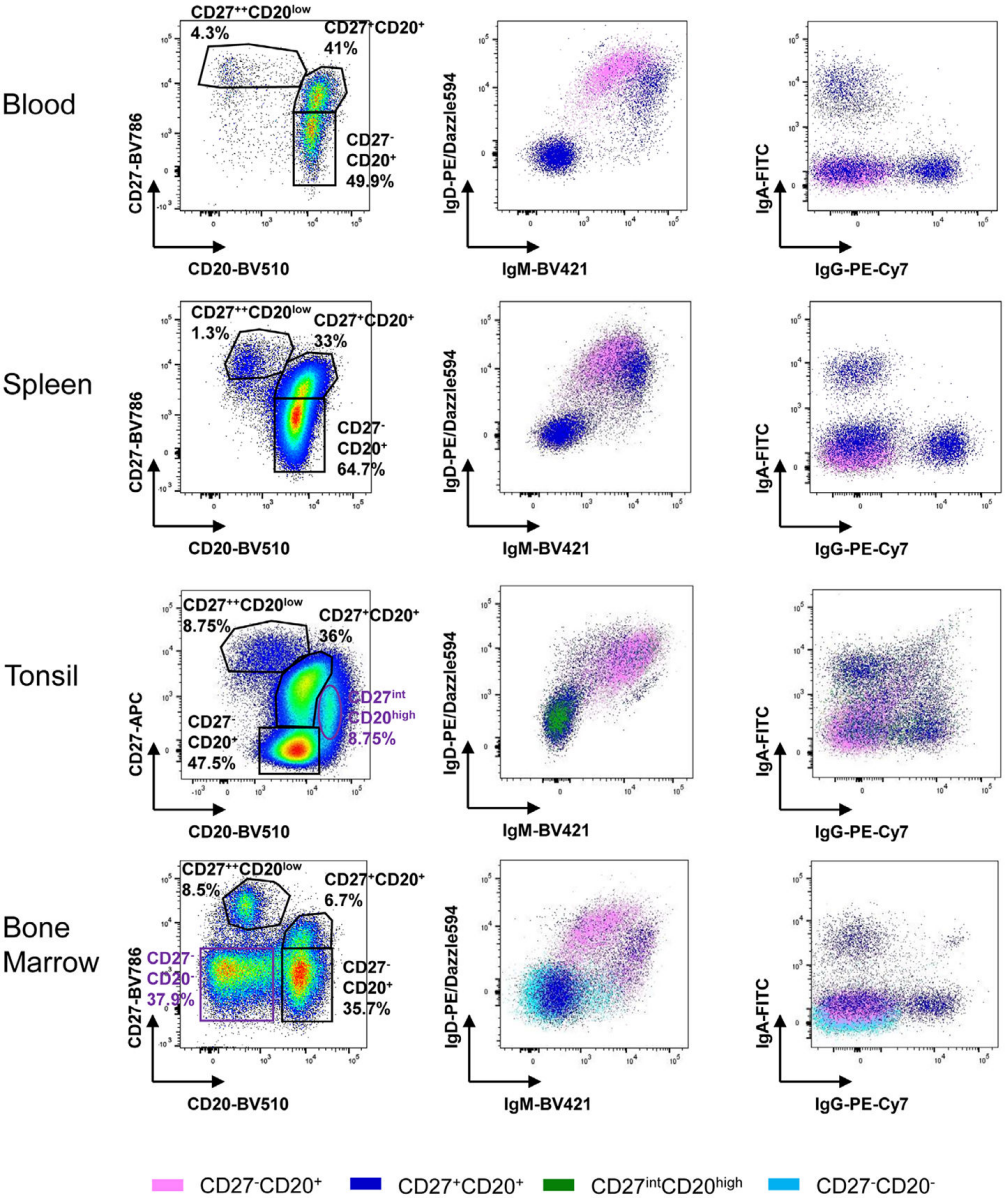
Ig isotype expression of human B cell subsets in different tissues. B cell subsets discriminated by CD27 and CD20 in peripheral blood, spleen, tonsil and bone marrow: conventional naive B cells are CD27^−^ CD20^+^ (containing CD27^−^ memory B cells) memory B cells CD27^+^ CD20^+^ and plasmablasts CD27^++^ and CD20^low^. Cell subsets defined by CD27 and CD20 expression were color-coded and depicted in a IgD vs IgM and IgA vs IgG plot to show Ig surface expression of each subset (pink: CD27^−^CD20^+^ B cells, dark blue: CD27^+^CD20^+^ B cells, green (only in tonsil): CD27^int^CD20^high^, turquois (only in bone marrow): CD27^−^CD20^−^).

To identify B cells, the B cell specific molecules CD19 and/or CD20 serve as specific surface markers (Fig. 95). CD19 is a B cell surface molecule expressed at the time of immunoglobulin heavy chain rearrangement [891], CD20 is expressed by all mature B cells beyond the pro B cell stage in the bone marrow and disappears on the surface of mature plasma cells [892, 893]. For further discrimination of B cell developmental stages, combinations of additional markers such as CD27, CD38, CD23, CD77 and expression of surface immunoglobulins are used. Immature CD19^+^ B cells in the bone marrow express high levels of CD38 and variable levels of CD20 and IgM, which increase with their further differentiation (Fig. 95F, Fig. 96) [894]. CD38^++^ CD20^++^ immature B cells express IgM and IgD, leave the bone marrow and become CD38^++^ CD24^++^ CD10^+^ transitional B cells [894]. Naïve B cells express IgM and IgD and are CD27^−^ and CD38^−^, they comprise about 60% of B cells in the peripheral blood (Fig. 95F, Fig. 96) [895, 896]. After Ag encounter and T cell help, memory B cells and Ab-secreting plasma cells are generated in the germinal center reaction. Germinal center (GC) B cells are highly proliferative B cells only present in secondary lymphoid tissues. Thus, in humans GCB cells are less accessible than in mice and not usually studied by flow cytometry, instead are studied using immunofluorescence staining [897]. Human memory B cells (mBCs) can be identified by the expression of CD27 and carrying of mutated immunoglobulin VDJ gene rearrangements [895, 898]. In the peripheral blood, between 30 and 40% of circulating B cells express CD27 (Fig. 95F, Fig. 96) [895, 899]. PC carry distinct FSC and SSC characteristics, express high levels of CD27 and lack the expression of CD20 but are also highly positive for CD38 and partially CD138^++^ [900]. A CD19^−^ PC population is uniquely enriched in the bone marrow [901, 902].

An alternative staining protocol of CD20^+^/CD19^+^ B cells has applied co-staining of CD38 and IgD together with CD77 and CD23 to mark differentiation stages of B cells in human tonsils [903]. CD23 is a low-affinity Fcε receptor and associated with the activation of B cells. It was found to be co-expressed with IgM and IgD in the tonsil and in peripheral blood but not with IgA and IgG and hence is lost during isotype class-switching [904]. CD77 is strongly expressed by germinal center B cells and can be used to differentiate centroblasts from centrocytes [903, 905]. In this protocol, naive IgD^+^ CD38^−^ B cells are separated by CD23 into Bm1 (CD23^−^) and Bm2 (CD23^+^) B cells. IgD^−^ CD38^+^ germinal center B cells can be further discriminated into CD77^+^ centroblasts (Bm3) and CD77^−^ centrocytes (Bm4). IgD^−^ CD38^−^ B cells comprise the memory compartment (Bm5).

The expression of IgD can be used as a marker to further discriminate certain naïve and memory B cell populations. CD19^+^ CD20^+^ B cells can be separated in a CD27 versus IgD dot plot (Fig. 95E). In this regard, naïve B cells express IgD and are CD27^−^. Further quadrants represent different subsets of memory B cells: in detail, CD27^+^ IgD^+^ are memory B cells which mostly express high levels of IgM and carry somatic mutations of their V(D)J rearrangements, whereas CD27^+^ IgD^−^ memory B cells are class-switched and also carry somatic mutations [895]. Interestingly, the CD27^−^ IgD^−^ B cell subset appears to be very heterogeneous and contains IgA-and IgG-expressing cells [906, 907]. It has been shown that this phenotypic population contains a memory B cell subset expressing CD95 with an activated phenotype, which is especially enhanced in patients with systemic lupus erythematosus (SLE) and correlated with disease activity and serologic abnormalities, whereas healthy donors only show minor frequencies of CD95^+^ cells [908]. These so-called double-negative B cells (DN, CD27^−^IgD^−^) have been shown to be composed by several subsets, among them a so-called DN2 that is increased in SLE and expresses CD11c [909]. Recently, deep phenotyping of CD11c^+^ B cells in systemic autoimmunity showed that this population correlates with the frequency of CD21-CD27- and CD21- CD38- B cells [910]. Among other disturbances, B cells lacking expression of the complement receptor CD21, which is part of a signaling complex, together with CD19 have been reported to be expanded in patients with SLE [911, 912].

#### 1.3 Step-by-step sample preparation

Depending on the starting material, different methods for cell isolation can be applied. A common start is to isolate mononuclear cells (MNCs) by density gradient centrifugation (see also Section IV.4.2 Pre-enrichment by physical properties in [22]). When starting with tissue, a lysate of the minced material can be layered over the Ficoll (e.g., GE Healthcare) solution, when starting with blood, this is carried out with a mixture of blood and PBS according to the manufacturer’s instructions. After collecting the MNC layer and subsequent washing steps, the number of isolated cells can be assessed and one can start with the staining procedure or further experiments. The washing buffer should be chosen according to the following experiments, for example, PBS containing a carrier protein-like BSA (e.g., 0.1 – 0.5%) for staining or medium for subsequent stimulation experiments. One should be aware that using EDTA in wash buffers might have an effect on following stimulation experiments by chelating calcium ions. For basic staining of B cells, 1-2x10^6^ MNCs should suffice as input. For analysis of Ag-specific cells, a higher input number might be useful (see Section V.2 Human Ag specific B cells).

For further applications, enrichment of B cells e.g., by MACS^®^ technology (Miltenyi) might be necessary (see Section IV.4.3: Pre-enrichment by immunological properties in [22]). Depending on the experiment planned after enrichment, an approach with untouched cells of interest (negative selection) can be applied (e.g., B cell isolation kit II). If specific subpopulations are desired, a positive selection might be required. MACS^®^ sorting gives high purities, nonetheless the purity check of the desired fraction by flow cytometry staining should be performed.

A different approach for staining starting with peripheral blood is a lysis protocol of red blood cells (see also Section II.1.5: Ery-throcyte lysis in [22], e.g., with BD PharmLyse, Qiagen EL buffer). After erythrocyte lysis and washing, the obtained cell suspension can be stained.

Depending on the application, blocking of Fc receptors can be useful prior to staining. One should be aware that Ig staining might not work after adding Fc blocking reagents to cell suspensions. Most manufacturers recommend surface staining at 4°C for 15-30 minutes but some molecules might require different staining conditions.

For intracellular staining, isolated MNCs can be lysed and permeabilized directly or after stimulation experiments to assess for example phosphorylation of intracellular proteins (see Section IV.6: Cell fixation and permeabilization for flow cytometric analyses in [22]). There are different protocols and reagents available depending on the intracellular location of the Ag to be stained (e.g., BD, Biolegend, eBioscience, and others). For Ags in the cytoplasm, a less harsh permeabilization buffer can be used than for example for Ags located in the nucleus. Blood or tissue lysates can also be prepared for intracellular staining directly (whole blood staining) by lysis and permeabilization and stained after washing steps. Twenty to 60 min at room temperature are frequently used for intracellular staining. One should be aware that some epitopes might be destroyed after lysis and permeabilization and thus may not be identified. This should be validated for each application.

#### 1.4 Materials

- Ficoll Paque ( GE Healthcare)
- PBS (Biochrom)
- Staining buffer (PBS/0,5% BSA/EDTA (Miltenyi autoMACS Rinsing Solution/MACS BSA Stock Solution))
- Buffers for cell permeabilization (e.g., Phosflow Lyse/Fix Buffer (BD Biosciences), Phosflow Perm Buffer II (BD Biosciences))
- Buffers for erythrocyte lysis (e.g., Lysing Buffer (BD PharmLyse ^™^ BD Biosciences, Buffer EL (Qiagen)),
- Anti-Mouse Ig, κ/Negative Control Compensation Particles Set (BD Biosciences)
- Live/Dead stain (e.g., 4′,6-Diamidin-2-phenylindole (DAPI, Molecular Probes) or LIVE/DEAD Fixable Dead Cell Stain Kit, (Invitrogen))
- Instrument: LSR Fortessa X-20 (BD Biosciences)
- Fluorescently labeled monoclonal Abs:

**Table:** 

Fluorophore	Marker	Manufacturer	Clone	Isotype
BUV395	CD3	BD	UCHT1	mouse IgG1κ
BUV395	CD14	BD	M5E2	mouse IgG2aκ
BV711	CD19	BD	SJ25C1	mouse IgG1κ
BV510	CD20	Biolegend	2H7	mouse IgG2bκ
BV786	CD27	BD	L128	mouse IgG1κ
APC-Cy7	CD38	Biolegend	HIT2	mouse IgG1κ
BV421	IgM	BD	G20-127	mouse IgG1κ
PE-Dazzle594	IgD	Biolegend	IA6-2	mouse IgG2aκ
PE-Cy7	IgG	BD	G18-145	mouse IgG1κ
FITC	IgA	Chemicon	M24A	mouse IgG1

#### 1.5 Data analysis

After MNC preparation or lysing whole blood, lymphocytes should be gated according to their scatter properties and by the exclusion of doublets and dead cells from the analysis (Figure 95 A, B, C). In order to detect plasma cells simultaneously, the initial FSC/SSC gating should be larger and not limited to a conventional lymphocyte gate [900].

When gating on CD19+ B cells, CD3+ T cells and CD14+ monocytes need to be excluded. If these cells are not of further interest, they can be assigned to a so-called “dump channel” with anti-CD3 and anti-CD14 Abs together with other markers for cells that should be excluded from subsequent analyses, e.g., anti-CD16/anti-CD56 for NK cells. One approach frequently applied is to gate on CD3^−^ CD14^−^ 4′,6-Diamidin-2-phenylindole (DAPI)^−^ cells (Fig. 95C) and, in a subsequent step, identification of CD19+ and CD20+^/−^ cells (Fig. 95D). This gating permits reliable identification of CD20+ B cells and additionally of CD20^low^ plasmablasts. For the analysis of B cell subsets, a classical combination using CD27 and CD20 of CD19+ B cells has been established. Using CD27, a number of B cell subsets can be identified independent of the expressed Ig subclasses. As a result, conventional CD27^−^ CD20+ naïve B cells, CD27+ CD20+ mBCs, including both pre-switched and class-switched memory B cells, as well as CD27++ CD20^low^ PBs can be identified (Fig. 95F). While the distribution of these subsets can vary between different diseases with slight variations [913], it has been demonstrated that CD27 can serve as a reliable marker for human healthy controls memory B cells, since CD27-expressing B cells differentiate timely into Ab-secreting cells after stimulation and carry somatic mutations in their immunoglobulin V regions [895, 898]. Of note, this gating strategy will not allow to identify class-switched B cells that lack the expression of CD27 [906, 907] and occur at higher frequencies among patients with systemic autoimmune diseases.

When comparing the CD27 vs CD20 plot in the different tissues (Figure 95F), an additional population has been found in the tonsil and another population in the bone marrow compared to peripheral blood and spleen. In the tonsil, a subset expressing high levels of CD20, intermediate levels of CD27 and CD38 expression appears in this plot and represent germinal center B cells that lack IgD expression [914]. In the bone marrow, an additional population positive for CD19 but lacking the expression of CD20 and CD27 can be found. These B cells express CD38, do not show surface IgD expression and low to no IgM surface expression (Fig. 96) and represent immature B cells [915].

#### 1.6 Pitfalls

- Blocking Fc receptor prior to staining might interfere with staining of immunoglobulins on B cells
- Choose an appropriate buffer for cell isolation: Buffers containing EDTA can decrease effects of stimulation by chelating calcium ions

#### 1.7 **Clinical** relevance statement

The methodology and gating strategy described in this section allow the detection of B cell subsets in healthy individuals and patients with, for example, autoimmune inflammatory rheumatic diseases (AIIRD). Patients usually present with certain B lineage abnormalities, some of them correlating with disease activity, for example, it is well known that SLE patients have increased frequency of plasmablasts in peripheral blood [900]. The study of B cell and plasma cell subsets present in the periphery of AIIRD patients and its comparison with those of healthy individuals can bring insights into certain B lineage abnormalities as candidates of disease pathogenesis and potential therapeutic targets.

#### 1.8 Summary of the phenotype

This is detailed in Table 73.

### 2 Human Ag-specific B cells

#### 2.1 Overview

The detection and phenotypic characterization of human Ag-specific B cells has been challenging mainly due to their low frequency in the circulation and the potential biases introduced by their ex vivo expansion. Naive B cells present with a diverse BCR repertoire that is usually of low avidity to the Ag. Upon Ag challenge, naive B cells undergo processes of somatic hypermutation, class switch recombination, and selection giving rise to memory B cells with high-avidity BCRs and PCs secreting highly specific Abs. Memory B cells and long-lived plasma cells are responsible for generation and maintenance of serologic memory but the protective ab titers do not necessarily correlate with the number or quality of Ag-specific B cells in the circulation [916, 917]. Auto-reactive B cells, on the other hand, might show aberrant features, such as cross-reactivity and low avidity Ag binding despite extensive somatic hypermutation [918, 919]. Here, we present two established methodologies to identify human Ag-specific B cells by flow cytometry. These methodologies can quickly be adapted to study new B cell specificities, as it was the case for the identification of B cells specific for the receptor-binding domain (RBD) of the 2019 novel coronavirus [920].

#### 2.2 Introduction

The identification of human Ag-specific B cell populations by flow cytometry has become an extremely valuable tool for a detailed understanding of both protective and auto-reactive human immune responses. Depending on the research questions, Ag-specific B cell responses can be analyzed and monitored upon vaccination, during the “steady state”, in different diseases and disease stages, phases of treatment, and in different compartments of the human body [921-923]. It allows for the phenotypic analysis of Ag-induced B cells by assessing various markers on the cell surface and inside the cell. In combination with fluorescence-activated cell sorting, it also allows subsequent analyses, such as transcriptomic profiling by single cell-based (“next generation”) sequencing techniques. Furthermore, it is possible to analyze Ag-specific B cell receptor (BCR) repertoires, to obtain full-length BCR sequences for monoclonal Ab generation, and to perform functional studies of isolated single B cells or B cell populations, which includes the generation of immortalized, Ag-specific B cell clones [924, 925]. This wealth of possibilities permits unprecedented insights into human B cell biology; it requires, however, particular care and adherence to relevant and tedious control steps to ensure that the Ag-specific B cell populations identified by flow cytometry, which are frequently very rare, indeed represent the Ag-specific B cell population of interest. Here, we provide a detailed description of the necessary considerations prior to starting out, the technological possibilities, approaches and necessary tools, and the relevant steps for performing experiments. We do so by using two examples of human Ag-specific B cell responses: (1) a vaccine-induced, high-avidity immune response identified by direct labeling of Ag with a fluorescent dye; and (2) an auto-reactive, low-avidity B cell response identified in an autoimmune disease setting using biotinylated self-Ags tetramerized with fluorescently labeled streptavidin molecules. In general, the examples described aim at identifying Ag-specific B cells within a polyclonal B cell repertoire to the highest validity. This implies that strong emphasis is placed on the exclusion of non-specific background signals and on several steps aimed at the verification of Ag-specificity. Notably, certain research questions might not require this strive for purity but can be answered by mere enrichment of the Ag-specific cell population. In other cases, such as single-cell transcriptomics, purity is crucial. In both cases, it is important to consider a number of general aspects before choosing the most suitable technical approach.

#### 2.3 Step-by-step sample preparation

##### General considerations before starting out:

###### Estimated frequency of the Ag-specific B cell population of interest.

In contrast to murine studies, in which the spleen and other lymphoid organs are readily accessible, most attempts to identify Ag-specific human B cells will need to rely on peripheral blood. In this compartment, CD19^+^ B cells comprise around 4% of total PBMCs in healthy individuals. Frequencies of Ag-specific B cell populations, however, can be very low (<0.01% of total B cells). In an ideal setting, this requires, for example, 1×10^6^ B cells to identify 100 Ag-specific B cells, and hence, a starting population of 25x10^6^ PBMCs. In other compartments such as bone marrow, tonsils or spleen, CD19^+^ B cells are more frequent (mean of 19% in BM, 33% in spleen, 43% in tonsils [926]), as might be the frequency of Ag-specific subsets. Depending on the compartment studied, it can therefore be important to estimate the expected frequency of the Ag-specific B cell population in order to determine the amount of starting material required for the identification of a minimum number of Ag-specific cells. This can be achieved by culturing ex-vivo isolated PBMC or pre-enriched B cells in limiting dilution, followed by the assessment of Ag-specific B cell presence by either ELISA or ELISPOT [927, 928]. These approaches will likely underestimate the number of Ag-specific B cells in the circulation, but will provide a minimum estimate of the cell numbers that can be expected. In addition, this initial estimate can provide information on isotype usage of the Ag-specific B cell response to be studied, and allow determining whether the frequency of circulating, Ag-specific B cells correlates with serum titers of the corresponding Abs. This, in turn, will help in selecting donors and, hence, increase the yield of Ag-specific B cells for the eventual assessment.

###### Expected phenotype of the cell population of interest.

Next to determining compartmental frequencies, it can be relevant to use additional phenotypic markers in the eventual panel that allow to more specifically select the B cell subpopulation in which the Ag-specific cells are expected. For example, markers could identify the isotype or the Ig subclass that appears to be most prominent for the Ig produced by PC measured by ELISA. Also, if IgM-expressing B cells are to be identified, low-affinity Ag binding can be expected, which in turn indicates that multimerization of Ags can be helpful to increase the fluorescent signal. Other Ag-specific responses, for example, might be enriched in IgG4 expressing B cells, which allows to significantly narrow the cell numbers that need to be studied. The identification of plasma cells that lack surface Ig expression, on the other hand, might require an intracellular staining approach. If the expression of such phenotypic characteristics is known, these should be incorporated in the staining panel and included in the frequency estimation described above.

###### Source of cells.

In general, any single-cell suspension that contains B cells, whether derived from peripheral blood, bone marrow, spleen, tonsils, or solid tissues, can be assessed for the presence of Ag-specific B cells. Limitations are caused by the frequency of the Ag-specific population of interest, and by the viability of cells (including pre-analytical treatment, i.e., shipment). Freezing cells, for example, is likely to compromise the plasmablast/ plasma cell compartment, while naïve and memory B cells are less sensitive. Pre-enrichment of B cells from larger populations by positive or negative selection can increase the percentage of Ag-specific B cells and shortens the time required on the flow-cytometer; it can, however, also compromise B cell subsets, depending on the isolation technique used. Therefore, due to the usually very low frequency of Ag-specific B cell populations, we recommend – whenever possible – using fresh, directly ex-vivo isolated B cells or B-cell containing suspensions such as PBMC as a starting point. This will minimize the loss of Ag-specific cells during work-up. For certain B cell populations and research questions, however, the use of frozen cells can be acceptable [929].

###### Choice of Ag.

In most cases, the Ag used for flow-cytometric assessment will be the Ag for which reactivity has been demonstrated in serum ELISA measurements or epitope mapping studies, or which has been used for inducing the immune response in, for example, vaccination studies. Both peptide and protein Ags are possible candidates. Protein Ags might be preferred in case of conformational epitopes; in addition, proteins are more likely to carry multiple epitopes, which increases the chance for higher avidity interactions with the BCRs. However, protein synthesis usually requires cells or expression systems and purification steps after which impurities (such as lipopolysaccharides or DNA) can remain that can influence and confound binding properties and introduce non-specific background signals. Peptides are easier to synthesize to high purity and contain one or more, sometimes synthetic, defined epitopes. Small sequence modifications can easily be introduced to generate non-binding control peptides. However, peptides are usually too short to build appropriate three-dimensional epitopes or structures that cross-link BCRs and, hence, monomeric peptides are usually insufficient for B cell identification. Therefore, peptide Ags are multimerized by generating either biotin-streptavidin tetramer complexes, or by coupling peptides to dextran backbones or other scaffolds using click-chemistry.

###### Choice of fluorescent labels.

In general, and in particular for low-avidity B cell immune responses, it is strongly recommended to reserve at least two fluorescent channels in a given staining panel for the identification of ultra-low frequency Ag-specific B cells. For reasons described below, identification of Ag-specific B cells by double-positivity for two identical yet differentially labeled Ags significantly reduces non-specific background signals and, hence, the risk for misinter-pretation of fluorescent signals as Ag-specific cells. This concept of a dual labeling approach has been described in detail elsewhere, an example is provided bellow [930-932]. We recommend using fluorescent dyes with emission spectra that show no or very little spectral overlap in order to reduce the need for extensive compensation. Use of single-label Ags might suffice for certain, high avidity B cell responses in combination with blocking studies (see below), but additional measures should then be taken to demonstrate staining specificity, in particular in cases in which blocking with excess, unlabeled Ag is incomplete.

###### Establishing positive and negative controls.

To ensure the highest reliability, we strongly recommend the use of both positive and negative controls in the establishment of Ag-specific staining approaches. In the specific setting described here, controls need to be established at two levels:

1. Control cells that do or do not express the BCR of choice. Such cells allow determining the specificity of Ag binding, the use of optimal concentrations of labeled Ag or Ag-multimers, and the degree of non-specific background binding. Further-more, they allow controlling for variations between batches of labeled Ags and can be used to determine the sensitivity of the approach to detect Ag-specific cells within a larger pool of cells. Such control cells are particularly useful during the experimental set-up phase of Ag-specific staining approaches aimed at identifying very low frequency B cells. However, they maintain their relevance also once protocols have been established. Examples for control cells include B cell hybridomas that have maintained cell surface Ig expression [933], immortalized human B cell clones of known specificity [924, 925], or cell lines transfected with monoclonal Ab sequences with or without the transmembrane domain of IgG [932, 934]. An example of the latter option using HEK293T cells is provided below. Non-transfected cells or cells with known specificity to an unrelated Ag can serve as negative controls.
2. Control populations of donors that do or do not harbor the Ag-specific B cell population of interest. In disease settings, these should include matched healthy donors but also disease controls [932]. In this context, a particular point for careful consideration lies in the possibility that control donors might harbor naïve B cells in their unmutated repertoire that specifically recognize the Ag of interest. Such recognition patterns have been described for nuclear auto-Ags and studies suggest that healthy individuals harbor a considerable proportion of mature naïve B cells capable of recognizing auto-Ags. For protective Ags, vaccinated donors can be analyzed as a control. Secondary vaccinations, *e.g.,* with TT, give rise to Ag-specific plasmablasts and memory B cells which can be analyzed one and two weeks after vaccination, respectively [935]. Flow cytometry is limited in the possibility to discern whether such staining signals reflect true Ag-specific B cells or non-specific interaction between labeled Ags and cell surfaces by, for example, hydrophobic interaction or charge. If positive signals are observed in control donors in flow cytometry, we highly recommend that additional measures be taken to determine/verify Ag specificity. Possible steps to do so are described below.

###### Verification of Ag-specificity.

Different direct and indirect measures are available to determine whether fluorescent signals detected by flow cytometry indeed identify B cells that specifically bind the Ag of interest via the BCR. Given that detection of ultra-low frequency B cells requires the assessment of large cell numbers of a given total B cell pool, it is almost inevitable that also non-specific back-ground signals are detected. Using a dual staining approach as described above significantly reduces such non-specific signals but does not suffice as single argument to claim Ag-specificity. Additional options include:

- Blocking (also called competition) experiments in which the binding of fluorescently labeled Ags to the BCR is blocked by an excess of unlabeled Ag. Note that an excess of unlabeled Ag should completely block the fluorescent signal. If this is not the case, non-specific interaction between the labeled Ag and the cell cannot be excluded and additional verification steps (see below) should be taken. This method can also be applied in a step wise approach with increasing amounts of unlabeled Ag where the increase of competing binding sites results in a gradual decrease of labeled Ag occupying the BCR.
- Single cell isolation by flow cytometry followed by immediate cell lysis, mRNA isolation and BCR sequencing using published protocols [934, 936, 937]; alternatively, single cells can be cultured with relevant stimuli followed by the assessment of supernatants for the production of total and Ag-specific Ig by ELISA [938]. Cells obtained from supernatants containing Ag-specific Ig can subsequently be lysed followed by mRNA isolation and BCR sequencing. In both cases, full-length BCR sequences can be obtained by primer-binding bias-free PCR protocols which can subsequently be used for monoclonal Ab expression [939]. Note that it can be extremely useful during experimental set-up to use the ‘index sort’ function for single-cell isolation protocols as this option allows to identify the localization of Ag-specific cells in the flow cytometry dot plot.

Together, verification of Ag specificity is crucial as this step is, in the end, the only way to determine whether cells identified by flow cytometry indeed represent the cell population of interest. Next to blocking experiments, the ultimate golden standard relies on single-cell isolation followed by BCR cloning and monoclonal Ab expression.

####### 1. Identification of a vaccine-induced, high-avidity immune response identified by direct labeling of Ag with a fluorescent dye

**Figure 97.**
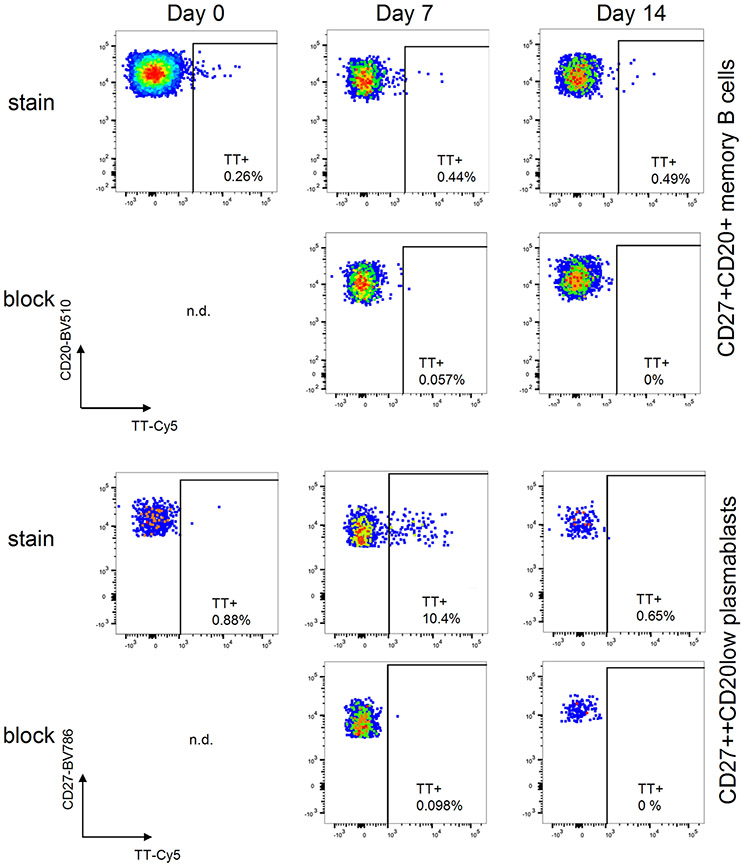
Identification of TT specific human memory B cells (CD27+CD20+) and plasmablasts (CD27++CD20low), gated as in section 1 - Human B cells and their subsets, before (day 0) and after TT vaccination (day 7 and day 14) in peripheral blood. Staining and block with unlabelled TT are shown.

In order to detect B cells specific for a certain Ag, we here make use of the ability of B cells to bind the proteins they recognize through the BCR in a three-dimensional manner (in contrast to the TCR recognizing only two-dimensional structures). To illustrate this approach, we provide an example of the detection of Tetanus Toxoid (TT)-specific human memory B cells and plas-mablasts in the circulation at several time points before and after TT vaccination (figure 97). Vaccination can be a highly useful tool in the initial set-up of the staining procedure, but will not be available to aid the detection of auto-reactive B cells described below. Nonetheless, the technological approach presented in this example has also successfully been used for the detection of auto-reactive B cells [940]. Therefore, the example provided can be seen as a template, which can be adapted to the identification of other Ag-specific B cell responses with similar characteristics.

In order to assure specificity of the staining, we show how the fluorescent signal in the same sample can be blocked with unconjugated protein used in excess. We used this methodology successfully to analyze B cells specific for TT [935, 941]; cholera toxin B (CTB) [942]; Keyhole Limpet Hemocyanin (KLH) [917]; Pentraxin-3 (PTX-3) [940] and receptor-binding domain (RBD) of 2019 novel coronavirus [920]. The possibility to down-titrate binding of Ag-specific cells (competitive assay principle) has long been recognized to ensure specificity.

Notes:

- as discussed above, Ag-specific B cells are found at very low frequencies in the circulation; therefore, it is crucial to start with more input cells/blood that one would usually do to stain B cell subsets. The input depends on the estimated frequency of the Ag-specific B cells in the population of interest.
- for sample preparation and staining of naive, memory B cells and plasmablasts (see section V.1 Human B cells and their subsets).
- for intracellular staining, permeabilize and fix the cells (see section VI.1.3 Human B cells and their subsets: Cell fixation and permeabilization for flow cytometric analyses).

Steps:

1. Preparation of fluorescently labeled Ags. The fluorescently labeled Ags should be titrated for an optimal signal to noise ratio independently for surface and intracellular detection. (note: for intracellular staining, usually a smaller quantity of labeled Ag is sufficient).
2. upon sample preparation, wash cells twice with PBS before incubation with fluorescently labeled Ag.
3. divide the sample in two: incubate half of the cells with fluorescently labeled Ag and the second half with unconjugated Ag. Incubate both at 4°C for 15-30 minutes.
4. Wash with PBS and prepare for acquisition on a flow cytometer of choice.

####### 2. Identification of an auto-reactive, low-avidity B cell response identified in an autoimmune disease setting using biotinylated peptide self-Ags tetramerized with fluorescently labeled streptavidin molecules.

The example provided below demonstrates the identification of B cells directed against citrullinated protein Ags in patients with rheumatoid arthritis (RA) [932, 943]. Citrullination represents the post-translational modification of arginine residues in a given protein to the amino acid citrulline. The citrulline-directed, auto-reactive B cell response is a hallmark of this disease and can be detected in >70% of patients by serum measurement of anti-citrullinated Ag Ig reactivity in ELISA. The humoral immune response uses all Ig isotypes and is, on the polyclonal level, of remarkably low avidity [944]. Circulating, Ag-specific B cells in peripheral blood are expected at a frequency of ~1:10.000 total B cells and can comprise naïve and memory B cells as well as plasmablasts and plasma cells [927, 928, 932]. Specific Ag recognition is determined by BCR binding to citrullinated Ags but not to the arginine-containing peptide control variants. A biotinylated, cyclic citrullinated peptide (CCP2) and its arginine control variant (CArgP2) are used for specific detection. HEK293T cells that express, in membrane-bound form, a monoclonal Ab that specifically recognizes the citrullinated peptide Ag of choice (HEK^ACPA-TM^), serve as controls during experimental setup [932]. Note that this example can be seen as a template, which can be adapted to the identification of other Ag-specific B cell responses with similar characteristics.

####### 1. Generating biotinylated peptide-avidin tetramers.

1. Incubate biotinylated peptides in excess with fluorescently labeled streptavidin or extravidin overnight at 4°C. The molar ratio between peptide and streptavidin should exceed 4:1.
  Note: in the example provided, six different peptide tetramers have been generated.
  - CCP2-biotin coupled to APC-labeled streptavidin (CCP2-SA-APC)
  - CArgP2-biotin coupled to APC-labeled streptavidin (CArgP2-SA-APC)
  - CCP2-biotin coupled to BV605-labeled streptavidin (CCP2-SA-BV605)
  - CArgP2-biotin coupled to BV605-labeled streptavidin (CArgP2-SA-BV605)
  - CCP2-biotin coupled to PE-labeled extravidin (CCP2-EA-PE)
  - CArgP2-biotin coupled to PE-labeled extravidin (CArgP2-EA-PE)
2. Remove unlabeled peptide with Bio-Spin^®^ Columns with Bio-Gel^®^ P-30. This will trap the free peptide in the gel and release peptide-streptavidin tetramers in the flow-through. The removal of unlabeled peptides is crucial to avoid binding competition between unlabeled and labeled Ag during staining.
3. Store the labeled Ag tetramers at 4°C. In case of longer storage times (weeks to months), perform regular testing of the stability of the tetramers and fluorescent signal by using the positive/negative control cells (see below).

####### 2. Determining optimal concentrations of multimerized Ag-tetramers for staining.

Notes:

- The optimal concentration of labeled peptide tetramers to be used for staining needs to be determined by titrating the labeled Ags on a fixed number of positive and negative control cells. Here, HEK^ACPA-TM^ cells are used as positive control; non-transfected HEK293T wild-type cells (HEK^WT^) serve as negative controls.
- For each tetramer, use the concentration at which the positive control stains highly positive and the negative control is negative.
- Combine the differentially labeled peptide tetramers at their optimal concentration to stain the positive (HEK^ACPA-TM^) and negative (HEK^WT^) control cells and determine whether a double positive population emerges in the diagonal of a flow cytometry dot plot. Should the double-positive population deviate from the diagonal, adjust the concentrations of differentially labeled peptide tetramers accordingly until the double-positive population falls into the diagonal. No signals should fall into this diagonal upon staining of the negative control cells.
- Upon determination of the optimal concentrations of labeled peptide tetramers to use, it can be helpful to spike positive control cells (HEK^ACPA-TM^) in different numbers into healthy donor PBMC and to determine by subsequent Ag-specific staining whether the pre-determined optimal concentrations remain optimal in a mixed population of cells.

**Figure 98.**
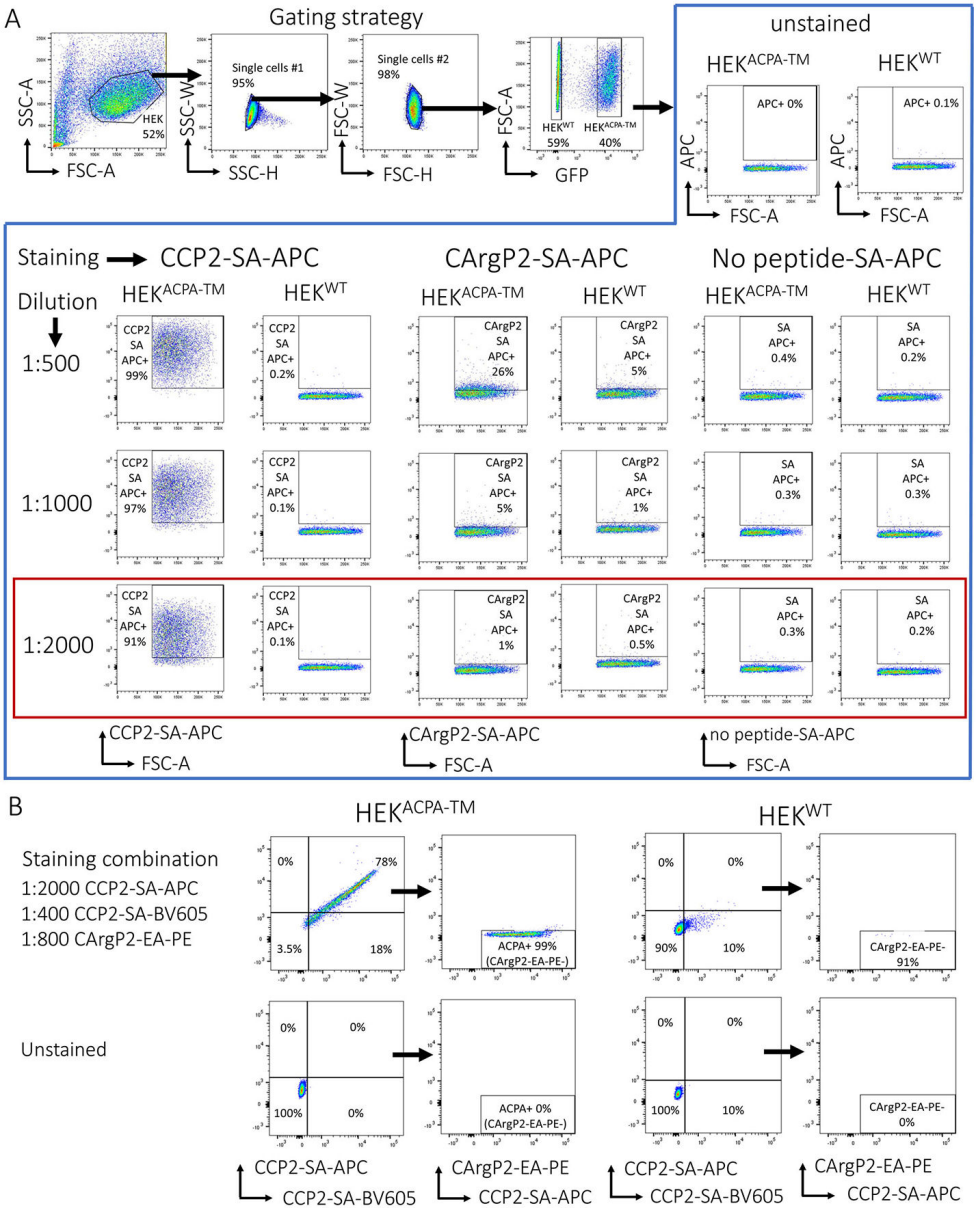
Determining optimal concentrations of multimerized Ag-tetramers for staining in HEK 293T cells. (A) Titration of CCP2-SA-APC, CArgP2-SA-APC and of ‘empty’ streptavidin APC tetramers on ACPA-expressing HEK 293T (HEK-ACPA^™^) and wild-type HEK 293T (HEK^WT^) cells. Gates are based on unstained controls. The red square marks the optimal concentration of CCP2-SA-APC. (B) Staining of HEK-ACPA^™^ and HEK^WT^ cells with combinatorial CCP2 and CArgP2 tetramers. Gates are based on unstained controls.

Steps:

1. Prepare serial dilutions of peptide tetramers and of ‘empty’ labeled avidin molecules.
2. Stain a fixed number (here: 2×10^5^) of positive and negative control cells with labeled peptide tetramers diluted in FACS buffer to 20 μl staining volume. Incubate for 30 minutes at 4°C.
3. Wash cells twice with FACS buffer and suspend in 100 μl FACS buffer for analysis on a flow cytometer.
4. In the example, CCP2-SA-APC tetramers at a dilution of 1:2000 stain >90% of HEK^ACPA-TM^ cells, and less than 1% of HEK^WT^ cells (Figure 98A). At the same concentration, CArgP2-SA-APC control tetramers show minimal background staining. Also, the empty labeled streptavidin molecules give minimal background at all concentrations. Therefore, a dilution of 1:2000 for this particular batch of CCP2-SA-APC tetramers is chosen for the subsequent combinatorial staining.
5. Perform similar titrations for BV605 and PE-labeled tetramers to obtain optimal dilutions for the subsequent combinatorial staining. To decide which concentration of CArgP2-EA-PE tetramers to use, choose the concentration at which CCP2-EA-PE tetramers give the highest signal and CArgP2-EA-PE tetramers minimal background HEK^ACPA-TM^ cells.
6. In the example, dilutions of 1:2000 are optimal for CCP2-SA-APC, 1:400 for CCP2-SA-BV605, and 1:800 for CArgP2-EA-PE tetramers for the combinatorial staining. This combination identifies HEK^ACPA-TM^ cells positive for CCP2-SA-APC and CCP2-SA-BV605 in the upper-right quadrant of the CCP2-APC vs CCP2-BV605 dot plot, while the CCP2 double-positive population remains negative in the PE control channel (Figure 98B). Also, no APC/BV605 double-positive signal is observed for HEK^WT^ cells.

####### 3. Staining of a sample containing human Ag-specific B cells.

Notes:

- given the very low frequency of Ag-specific, ACPA-expressing B cells in the circulation, we here used 50 ml of freshly drawn peripheral blood as starting material.
- here, we deliberately chose to stain whole PBMC and not to enrich B cells by subsequent isolation techniques in order not to compromise B cell subpopulations such as plasmablasts.
- in the example provided, 4′,6-Diamidin-2-phenylindol (DAPI) is used to identify and exclude dead cells.
- We assume in this example that all labeled monoclonal Abs and their respective isotype control Abs have been titrated and tested in appropriate fluorescence minus one (FMO)-stainings to determine optimal concentrations. These steps are necessary but not mentioned in the description below.

Steps:

Identification of ACPA-expressing B cells in a peripheral blood sample of an ACPA-positive rheumatoid arthritis patient.

**Figure 99.**
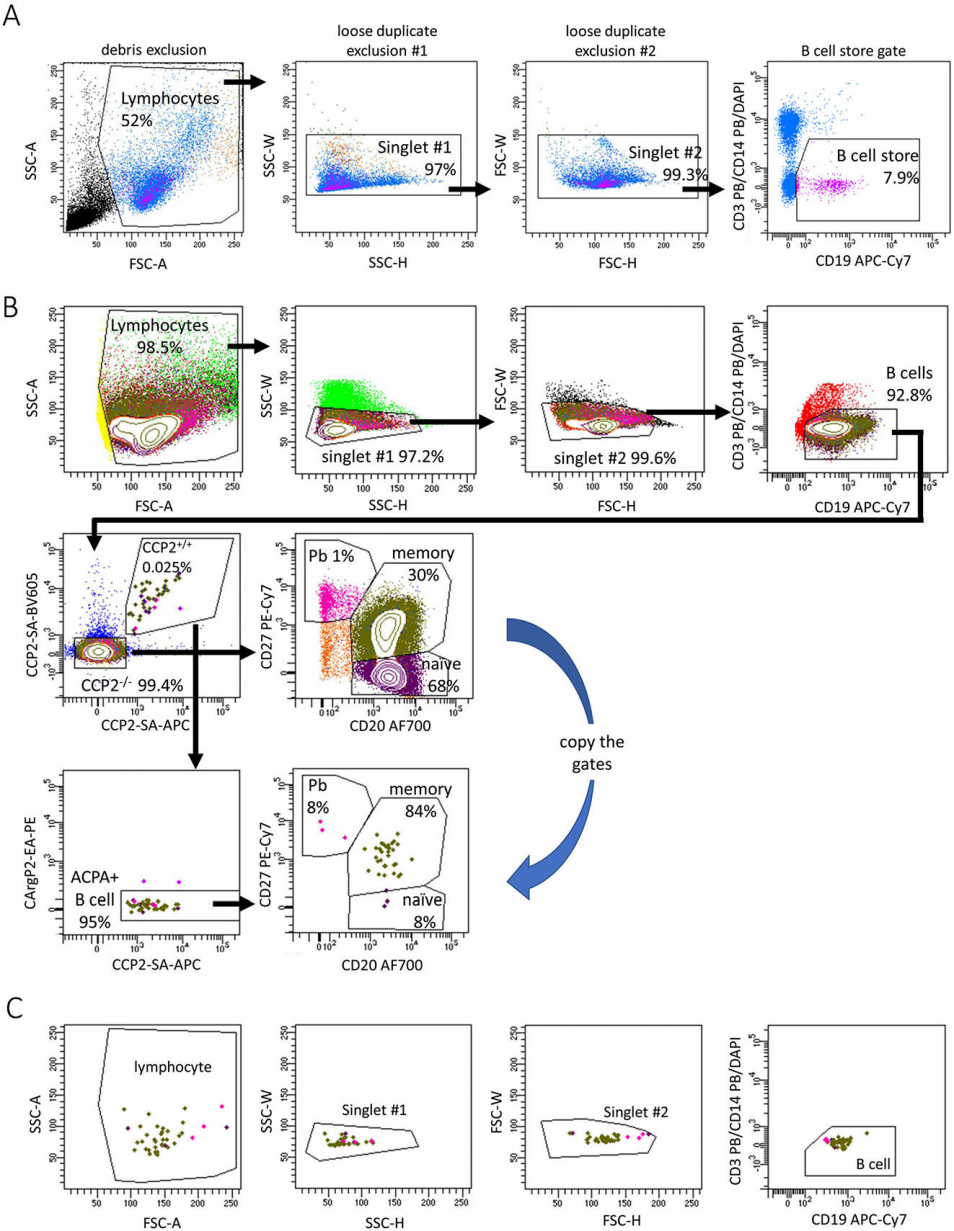
Gating strategy to identify ACPA-expressing human B cells in peripheral blood of patients with rheumatoid arthritis. (A) Setting up a “B cell store gate” which will be used during sample measurement to store data in order to obtain a manageable size of data to be analyzed. Freshly isolated, patient-derived PBMC are used. (B) Gating strategy to identify ACPA-expressing human B cell subsets in peripheral blood. Single live lymphocytes are identified based on FSC/SSC followed by identification of CD3/CD14/DAPI^−^CD19^+^ B cells (first row). Subsequently, ACPA-expressing B cells are identified within the CD19^+^ lymphocyte population as cells staining positive for both CCP2-SA tetramers (BV605- and APC-labeled). The double-positive fraction is then evaluated for reactivity with the arginine controlvariant of the peptide (CArgP2-EA-PE). Using the CCP2-SA negative, total B cell population as reference, gates are placed for differential expression of CD20 and CD27 by CD19^+^ B cells. These reference gates are copied to allow for the phenotypic assessment of the Ag-specific, CCP2-SA-BV605/CCP2-SA-APC^+/+^CArgP2-EA-PE^−^ B cell population. (C) Back-gating of ACPA-expressing B cells as additional measure of control to verify cell size and granularity within the large pool of PBMC-derived B cells.

1. Isolate PBMC from a peripheral blood sample using Ficoll-Paque gradient centrifugation following standard protocols. Count the isolated PBMCs.
2. Suspend the PBMCs at 8x10^6^ cells per 100μl in FACS buffer in a 15 ml tube and stain with the fluorescently labeled Abs/streptavidin tetramers for 30 minutes on ice protected from light. In the example, we used: CD3 Pacific Blue, CD14 Pacific Blue, CD19 APC-Cy7, CD20 AF700, CD27 PE-Cy7, CCP2-SA-BV605, CCP2-SA-APC, and arginine peptide control CArgP2-Extravidin-PE at the appropriate concentrations. The emission wavelength of Pacific Blue is used in this example as a “dump channel” in which markers are combined that identify cells that are to be excluded from further analysis.
3. Add 1ml FACS buffer and centrifuge the cells for 5 min at 1500 rpm on ice.
4. Repeat the washing step at least 2 times.
5. Resuspend the cell pellet with 100μl FACS buffer and transfer to a FACS tube (we use Micronic tube MP32022). Keep the sample on ice and in the dark at all times.
6. To compensate for spectral overlap between fluorescent dyes, we employ compensation beads (BD Biosciences) that bind to mouse IgG (provided that all of the fluorescently labeled Abs used are of an murine IgG isotype). The beads are used to compensate for CD3 Pacific Blue, CD19 APC-Cy, CD20 AF700 and CD27 PE-Cy7 spectral overlaps. For the tetramers, however, surrogate murine IgG that are conjugated with BV605, APC and PE are used to allow fluorescence compensation using beads.
7. Set up a flow cytometer of choice (here: BD LSRFortessa) that allows simultaneously detecting and discriminating fluorescent signals from Pacific Blue, APC-Cy7, AF700, PE-Cy7, BV605, APC and PE dyes. For the analysis, we here used BD FACSDIVA software (version 8.0.2).
8. Perform fluorescence compensation using single stained compensation beads and apply the compensation set-up to the whole experiment.
9. Add 100μl of 200nM DAPI to the cell suspension (leading to a final concentration of 400nM).
10. Place the sample into the cytometer and record 50,000 events. Put the sample back on ice and keep protected from light.
11. Place gates in a Global Worksheet of the DIVA programme on the cell populations as follows (Figure 99a):
  - In the FSC-A vs SSC-A plot, make an inclusive gate containing lymphocytes and monocytes to include plasmablasts that are larger in size and more granular than other subsets of B cells.
  - Subsequently, exclude duplicates using SSC-H vs SSC-W and FSC-H and FSC-W plots. The gates for duplicate exclusion should not be strict at this moment.
  - Lastly, in a Pacific Blue vs CD19-APC-Cy7 plot, gate loosely on CD19 positive cells that are Pacific Blue-negative. This gate is referred to as “B cell Store” (Figure 99a).
12. Click “Next Tube” on the Acquisition Dashboard of the BD FACSDIVA workspace.
13. In the Acquisition Dashboard, choose “B cell Store” for both Stopping and Storage Gates. Set 10,000,000 events for both ‘Events to Record’ and ‘Maximum Events to Display’. This step is necessary to obtain a manageable size of data to analyse the Ag-specific cell population of interest (here: ACPA-expressing B cells).
14. Place the sample back into the flow cytometer. Record the “B cell store” and adjust the threshold rate to a maximum of 20,000 events per second. Measure the sample until it is finished.
15. Store the data appropriately.

#### 2.4 Materials

Purified or Biotinylated peptide or protein Ags of choice depending on the protective/auto-reactive B cell response(s) to be studied.

- Fluorescently labeled streptavidin and/or extravidin molecules, e.g., BV605-streptavidin (Biolegend, catalogue nr.: 405229), APC-labeled streptavidin (Invitrogen, catalogue nr.: S32362) and PE-labeled extravidin (Sigma-Aldrich, catalogue nr.: E4011-1ml).
- Fluorochrome for labeling of respective Ag, e.g., Cy5
- Bio-Spin^®^ Columns with Bio-Gel^®^ P-30 (BIO-RAD, catalogue nr.: 732-6006)
- PBS
- BSA (Sigma-Aldrich, catalogue nr.: A7906-1KG).
- FACS buffer (PBS, 0.5% BSA and 0.02% Azide)
- 4′,6-Diamidin-2-phenylindol (DAPI) (Invitrogen, catalogue-nr.: D1306)
- fluorescently labeled monoclonal Abs (all Abs used in the present example are of mouse origin, expressed as IgG isotypes and directed against the respective human proteins):

**Table:** 

No.	Marker	Clone	Fluorophore	Supplier	Catalog number
1	CD3	UCHT1	Pacific Blue	BD	558117
2	CD14	M5E2	Pacific Blue	BD	558121
3	CD19	Sj25C1	APC-Cy7	BD	557791
4	CD20	2H7	AF700	Biolegend	302322
5	CD27	M-T271	PE-Cy7	BD	560609
- the following Abs were used as “surrogate” Abs for the compensation of avidin-tetramer derived fluorescent signals (all are murine Abs expressing the IgG_1_ isotype directed against the respective human proteins indicated):

**Table:** 

No.	Marker	Clone	Fluorophore	Supplier	Catalog number
1	CD56	HCD56	BV605	Biolegend	318333
2	CD4	SK3	APC	BD	345771
3	CD8	RPA-T8	PE	BD	555367
- BD^™^ CompBeads anti-mouse Ig, κ (BD Biosciences, Catalogue nr.: 51-90-9001229)
- BD^™^ CompBeads negative control (BD Biosciences, Catalogue nr.: 51-90-9001291)
- Instrument: BD LSRFortessa (BD Biosciences)
- Software: BD FACSDIVA version 8.0.2 (BD Biosciences),
- appropriate positive and negative control cells (here: HEK^ACPA-™^ and HEK^WT^).

#### 2.5 Data analysis

##### 1. Identification of a vaccine-induced, high-avidity immune response identified by direct labeling of Ag with a fluorescent dye

Analysis and gating for the example provided is straightforward. B cell subsets can be gated as described in section V.1 Human B cells and their subsets. Following this step, fluorochrome specific plasmablasts, memory B cells and naive B cells can be determined as shown for plasmablasts and memory B cells in figure 97.

##### 2. Identification of an auto-reactive, low-avidity B cell response identified in an autoimmune disease setting using biotinylated peptide self-Ags tetramerized with fluorescently labeled streptavidin molecules.

1. Open the experiment file using BD FACSDIVA version 8.0.2 (BD Biosciences)
2. Check and adjust compensation of spectral overlap according to standard procedures.
3. Create a new “Normal Worksheet” in the file which stored only the “B cell store” gate. Gate lymphocytes, single cells and live B cells strictly (Figure 99b)
4. Starting from the ‘live single B cell gate’, create a CCP2-SA-BV605 vs CCP2-SA-APC plot to identify CCP2+/+ and CCP2 −/− populations. Place a gate around those CCP2+/+ cells that strictly fall into the diagonal.
5. Display the cells identified in this gate (the CCP2+/+ population) in a CCP2-SA-APC vs CArgP2-Extravidin-PE plot and place a gate on the CArgP2-PE negative population. These cells represent the Ag-specific B cell population of interest (i.e., ACPA-expressing B cells).
6. In the CCP2-SA-BV605 vs CCP2-SA-APC plot, place a gate on the CCP2−/− population, create a CD20-AF700 vs CD27-PE-Cy7 plot and gate on naïve (CD20+CD27−), memory (CD20+CD27+) and plasmablast (CD20-CD27high) subsets of these avidin-tetramer negative B cells.
7. From the gate identifying the ACPA-expressing B cell population (the CCP2+/+ CArgP2- population), create a CD20-AF700 vs CD27-PE-Cy7 plot. Copy the gates identifying naive (CD20+CD27−), memory (CD20+CD27+) and plasmablast (CD20-CD27high) subsets from the avidin-tetramer negative B cell population to the plot displaying the ACPA-expressing B cell population. This step is taken as it can be difficult to define the gates for these B cell subsets on the basis of very few cells. Therefore, copying the gates from a larger population (the avidin-tetramer negative B cells) to the Ag-specific B cell population (the ACPA-expressing B cells) is necessary for further analysis.
8. In the given example, the majority of ACPA-expressing B cells displays a memory (CD20+CD27+) phenotype, while avidin-tetramer-negative B cells mostly fall in the naive B cell gate (CD20+CD27−) (Figure 99b).
9. As an additional step of control, perform ‘back-gating’ of the ACPA-expressing B cell population. Should some cells fall at the edge of the gates identifying lymphocytes, single cells, and live B cells, adjustment of these gates might be necessary to minimize the possibility that doublets or otherwise non-specifically stained cells are misinterpreted (Figure 99c).

#### 2.6 Pitfalls

- Be aware that the quality of the fluorescent signal of the labeled avidin-tetramers decreases overtime. Take along a staining control using the positive and negative control cells with each sample to control for such signal decay and/or an increase of non-specific background staining.
- The fluorochrome itself can be recognized by B cells giving rise to false positive signals; this can be overcome by using the same Ag labeled with a second fluorochrome, as described.

#### 2.7 Top tricks

- During set-up and once a cell population has been identified that meets the criteria of Ag-specific cells delineated above, subsequent verification of Ag-specificity is indispensable. Please refer to the section ,Verification of Ag-specificity’ in the introduction for details on how Ag-specificity can be determined, and refer to [932], supplementary data section, for examples.
- For differential labeling of Ags, we recommend using fluorescent dyes with emission spectra that show no or very little spectral overlap in order to reduce the need for extensive compensation.

#### 2.8 Clinical relevance statement

The methodology and gating strategy described in this section allows the detection of Ag-specific B cell populations in healthy individuals and patients with, for example, autoimmune diseases. In a recent analysis focusing on patients with rheumatoid arthritis, a prototypic human autoimmune disease, the detection of B cells expressing ACPA has been used in parallel with the detection of tetanus-toxoid-specific B cells to evaluate phenotypic and functional characteristics of autoreactive versus protective, Ag-specific immune responses in individual patients. The key conclusion of this work is that Ag-specific B cell populations clearly differ from the total B cell pool in both aspects (phenotype and function), with phenotypic changes of the autoreactive population associating with disease characteristics that are relevant to a deeper understanding of pathophysiological processes driving disease.

#### 2.9 Summary of the phenotypes

Table of subpopulations detailed in the section V.1 Human B cells and their subsets (Table 73).

### 3 Human immunoglobulin heavy chain isotypes

#### 3.1 Overview

B cells play a key role in immune responses through the production of Abs. Detailed characterization of individual subsets of B cells that have switched to distinct immunoglobulin heavy chain isotypes can improve our understanding of the development of humoral immune responses. This section describes a method for detection of individual immunoglobulin heavy chain expressing B cells in human PBMC samples. This section can be considered a basis in which additional markers can be included to interrogate immunoglobulin heavy chain expression among the different B cell subsets.

#### 3.2 Introduction

B cells develop in the bone marrow and are released into the circulation, after which they mature predominantly in the spleen to become mature naive B cells. Mature B cells can be roughly divided into two subsets: conventional follicular B cells (frequently referred to as B-2 cells) and non-conventional extrafollicular B cells (including B-1 cells, which have been mainly characterized in the mouse, and marginal zone (MZ) B cells) [945, 946].

Extrafollicular B cells mount thymus-independent (TI) humoral responses, which are rapidly induced in response to conserved microbial carbohydrate or glycolipid structures. These responses often result in the production of polyspecific low-affinity IgM Abs, and typically do not involve somatic hypermutation or class switch recombination (CSR) [947]. T-independent CSR and somatic hypermutation have been reported [948]. Follicular B cells participate in thymus-dependent (TD) responses. These cells interact with follicular helper T (Tfh) cells in germinal centers, which are mainly found in secondary lymphoid organs [949]. Follicular B cells that receive T cell help (through CD40L and cytokines) will become germinal center B cells and upregulate BCL6 and activation-induced deaminase expression, and will undergo CSR and somatic hypermutation. [947, 950] The B cells that emerge from such a germinal center reaction will either become circulating memory B cells or plasma cells. Some plasma cells will home to the bone marrow where they can survive for many years as long-living immunoglobulin-secreting plasma cells [951].

B cell activation to T-cell dependent Ags requires BCR stimulation and CD40 ligation. Antigens can be captured directly by B cells or can be presented by follicular DCs in the lymphoid follicles. BCR stimulation is typically mediated through binding of a specific Ag to the BCR leading to internalization, processing and presentation of antigenic peptides in MHC class II molecules. Antigens are presented to CD4^+^ T cells, which are activated in this manner. Activated CD4^+^ T cells upregulate CD40L and secrete cytokines. The type of cytokines that are produced by these T cells depends on how these cells were primed as naive T cells. CD40-CD40L interaction and the local cytokine milieu provide the second signal that is required for efficient B cell activation including proliferation, CSR and plasma cell differentiation.

Antibodies are identical to the BCR of the B cell from which they originate, with the exception of a C-terminal sequence that anchors the molecule to the cell membrane and the associated signal transduction moiety CD79. As a result Abs are secreted and do not form surface-bound receptors. Antibodies have a functionally polarized structure, with on one side the Fab region harboring a hypervariable region, which is responsible for Ag binding, and on the other side a constant Fc region. The structure of the constant region determines the effector function of the immunoglobulin. Antibodies are typically classified according to the isotype of their heavy chain. Humans have nine major immunoglobulin heavy chain isotypes: IgM, IgD, IgG1, IgG2, IgG3, IgG4, IgA1, IgA2, and IgE [952] while there are eight murine Ig isotypes: IgM, IgD, IgG1, IgG2a, IgG2b, IgG3, IgA, and IgE [952]. Each of these Ab isotypes mediates distinct functions through interaction with specific receptors on effector cells and serum factors.

Each Ig molecule consists of two heavy (IgH) and two light chains, both of which contain variable (V) and constant (C) regions. The region of the heavy chain that determines Ag-specificity is made up by the variable (V_H_), diversity (D), and joining (J_H_) segments that are rearranged during early B cell development to form a VDJ cassette or V-region. The V-region is located upstream of the CH exons. The C region of the IgH chain determines the isotype of the Ig. In mature naïve B cells, the V-region is spliced to the constant region of the μ chain (Cμ) [953]. Consequently, mature naive B cells express surface IgM and, as a result of alternative splicing, IgD as their BCR. A population of IgM-IgD^+^ class-switched B cell has been reported. These cells are primarily found in secondary lymphoid organs and are not readily detected in peripheral blood [954].

**Figure 100.**
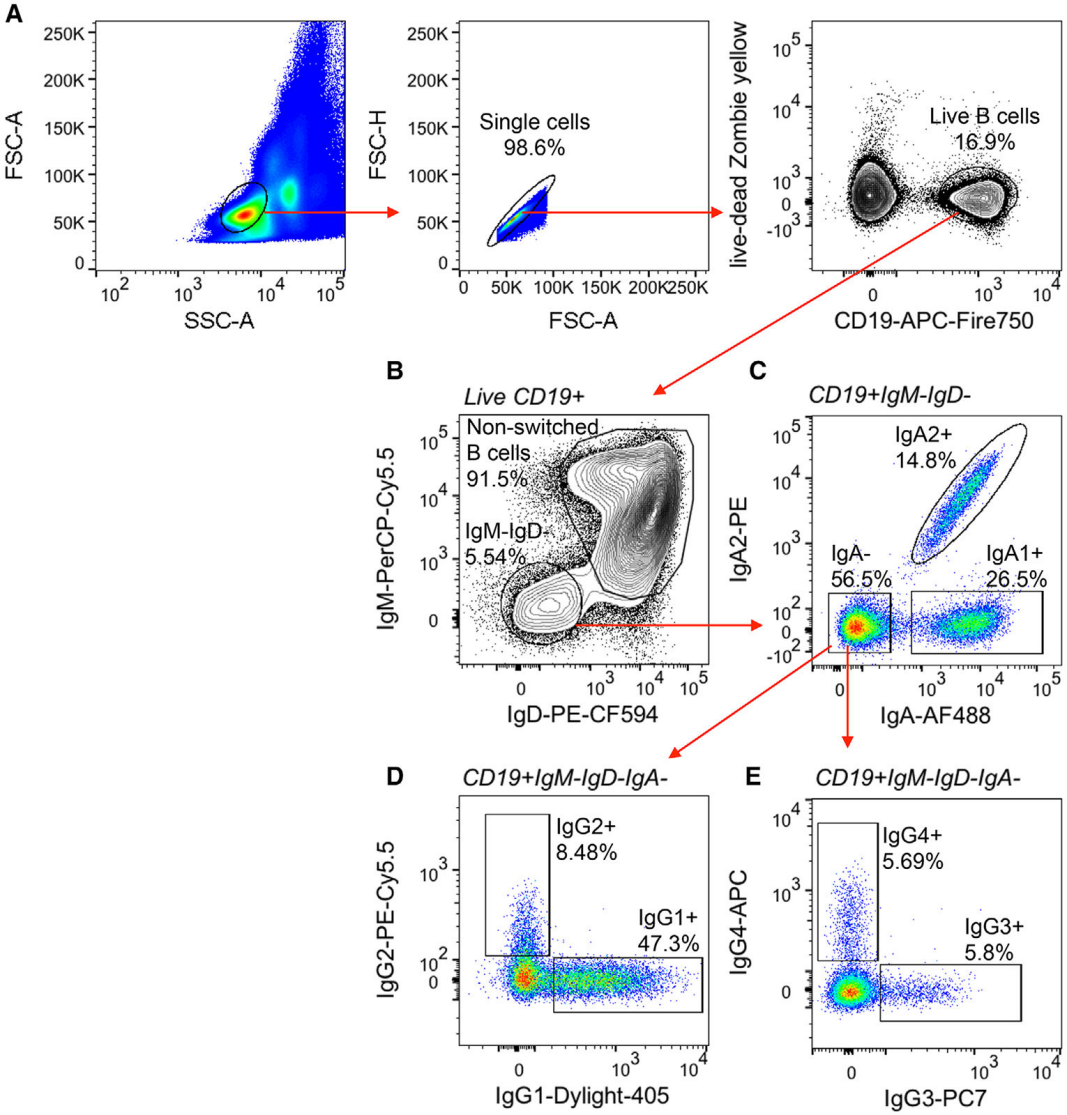
Identification of B cells expressing different Immunoglobulin heavy chain isotypes in a human PBMC sample (healthy individual age 47, male). (A) Lymphocytes were identified based on their FSC and SSC, Doublet exclusion was performed on FSC-H vs FSC-A, and B cells were gated as CD19^+^ and zombie yellow (viability dye) negative. (B) Non-switched B cells (IgD^+^) and class-switched (IgM-IgD−) were gated. (C) Within the IgM-IgD− population, IgA^+^ B cells, IgA2^+^ and IgA^−^ cells can be distinguished. IgA1^+^ B cells were defined as IgA^+^IgA2^−^. (D and E) IgA− B cells were further differentiated based on expression of IgG1, IgG2 (D), IgG3 and IgG4 (E).

Upon activation, naïve IgM^+^IgD^+^ B cells can undergo CSR, resulting in a change of the heavy chain isotype of the produced immunoglobulin, while its Ag-specificity is retained. Non-switched B cells express IgM and IgD on their cell surface (Fig. 100A, B). The non-switched IgM^+^IgD^+^ B cell population contains transitional B cells, naive mature B cells, and IgM^+^CD27^+^ memory B cells. The staining procedure outlined below is designed for the detection of human immunoglobulin heavy chain isotypes expressed as BCRs on B cells.

#### 3.3 Step-by-step sample preparation

This staining procedure is designed for human peripheral B cells. The reagents and materials that were used other than Abs are listed in table 74. PBMCs were isolated from heparinized blood of healthy individuals by density gradient centrifugation (Biochrom, Berlin, Germany). PBMC were counted and stained in PBS with zombie yellow viability dye (Biolegend, San Diego, CA). Samples were washed and incubated with Abs listed in table 75 and measured on a BD LSR Fortessa with BD FACSDiva Software Version 8.0.1 and analyzed using Flowjo version 10.4.

Detailed protocol:

1. Collect fresh blood in heparinized containers (BD vacutainer 170 I.U. of lithium heparin)
2. Isolate PBMC:
  - Dilute blood samples at a 1:1 ratio with PBS supplemented with 2 mM EDTA.
  - For each 30 ml of diluted blood prepare a tube of Biocoll. Add 15 ml of Biocoll separating solution to a 50 ml blood-sep-filter tube. The Biocoll should be at room temperature. Spin down 1 min 1100 g to collect the Biocoll at the bottom of the tube.
  - Slowly add 30 mL of diluted blood to each filter tube and centrifuge the tubes at 800 × *g* for 20 min at 18 °C and no break at end of program.
  - Collect the interphases and pool up to 2 interphases and transfer them to a new 50 ml centrifugation tube. Fill up tube with PBS supplemented with 2 mM EDTA. Centrifuge tubes at 780 × *g* for 10 min at 4 °C.
  - Remove supernatant and resuspend cell pellet in 2 ml PBS supplemented with 2 mM EDTA. Pass cells through a 70 μM cell strainer into a new 50 ml centrifugation tube. Fill up tube with PBS supplemented with 2 mM EDTA. Centrifuge tubes at 220 × *g* for 10 min at 4°C.
  - Discard supernatant and resuspend PBMC in 5 ml PBS supplemented with 2 mM EDTA.
3. Count PBMC and use between 1 and 5 million PBMC per staining.
4. Stain cells with Zombie yellow viability dye. Incubate for 20 min at 4°C in dark. Wash 1 × with staining buffer
5. Stain cells with Abs listed in table 75 in a total staining volume of 100 μl. Incubate for 15 min at 4°C in dark. Wash 1× with staining buffer.
6. Stain cells with Streptavidin-PC7, incubate 15 min at 4°C in dark. Wash 1× with staining buffer.
7. Resuspend cells in 200 μl staining buffer and measure sample.

#### 3.4 Materials

Materials are detailed in Tables 74 and 75.

#### 3.5 Data analysis

**Figure 101.**
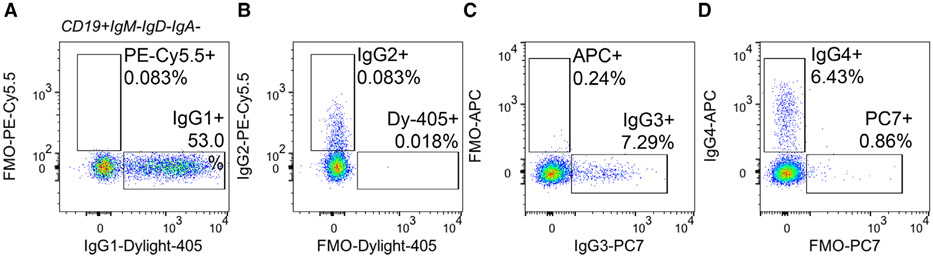
Fluorescence minus one (FMO) controls for IgG subclasses in B cells from a human PBMC sample from (Figure100C). (A) FMO for IgG1-Dylight-405. (B) FMO for IgG2-PE-Cy5.5. (C) FMO for IgG4-APC. (D) FMO for IgG3-PC7.

B cells that undergo CSR will lose expression of IgM and IgD and can be gated as CD19^+^IgM-IgD- (Fig. 100B). A major fraction of the switched B cell compartment consists of IgA^+^ B cells, which can be subdivided into IgA1^+^ and IgA2^+^ B cells. Here, we used an Ab against total IgA to identify all IgA^+^ B cells combined with an anti-IgA2 Ab to separate IgA2^+^ and IgA2- (i.e., IgA1^+^) B cells (Fig. 100C). The IgA- B cells can be further separated into IgG1^+^, IgG2^+^, IgG3^+^ and IgG4^+^ B cells (Fig. 100D, E), of which the IgG1^+^ population is the most abundant (Fig. 100D). A summary of the expected frequencies of different subpopulations is shown in table 76 (see below). IgE^+^ B cells are hardly detectable in healthy individuals and this population is not shown in this analysis. Expression of most immunoglobulin heavy chain isotypes, with the exception of IgM and IgD on non-switched B cells, is mutually exclusive. Thus, a class-switched (IgM-IgD-) B cell will only express a BCR with one immunoglobulin heavy chain isotype. Fluorescence minus one (FMO) controls for IgG subclasses are shown in figure 101.

#### 3.6 Pitfalls

This protocol has been established for staining of PBMC samples. It may be applicable to other material such as tonsil, or other tissue-derived single cell suspensions. Cell suspensions from B cell rich tissues such as tonsils may require optimization of the Ab dilutions. Also, when staining more than 5 Million PBMC, the amounts of Abs and/or the staining volume may need to be adjusted.

To the best of our knowledge the heavy chain isotype-specific Abs that were used in this staining panel do not cross-react with other isotypes. We did not observe significant populations of cells that were double positive for more than one immunoglobulin heavy chain isotype (other than IgM and IgD double positive non-switched cells). This can be easily confirmed by plotting every heavy chain isotype against every other.

#### 3.7 Top tricks

This panel can be extended by adding selected surface markers of interest to study, in detail, the different heavy chain class-switched B cell subsets.

#### 3.8 Clinical relevance statement

The gating strategy shown in this section is applicable for analysis of human peripheral B cells in order to determine the frequencies of non-class switched IgM^+^ IgD^+^ B cells, as well as the frequencies of B cells that underwent CSR to IgG1, IgG2, IgG3, IgG4, IgA1 and IgA2. We have applied this approach in the context of allergies [955, 956] and primary immune deficiencies [957, 958] in which detailed information regarding the distribution of B cell populations of different BCR isotypes was of interest. However, such analyses can be of value in monitoring B cell responses in other pathologies.

#### 3.9 Summary of the phenotypes

This is detailed in Table 76.

### 4 Human regulatory B cells

#### 4.1 Overview

B cells play a key role in immune responses through the production of Abs, Ag presentation to other immune cells, and production of cytokines. Suppressive cytokines, such as IL-10, play a pivotal role in controlling inflammation and immune tolerance. Importance of regulatory B cells (Bregs) producing suppressive cytokines was described in murine models and in several human diseases. Depending on the disease studied or on the *in vitro* stimulation, different functional Breg subsets were described. Detailed characterization of individual Breg subsets therefore can improve our understanding of regulation of immune responses. This section describes a method for detection of individual Breg subsets in human PBMC samples. This panel can be considered a basis in which additional markers can be included to interrogate their expression among the different Breg subsets.

#### 4.2 Introduction

The existence of B cells with regulatory function (Breg) was first proposed in studies using B cell-depleted rodents, which showed reduced suppressive capacity of the lymphocyte fraction and were unable to recover from experimental autoimmune encephalitis [959-961]. Later, Mizoguchi et al. [962] and Fillatreau et al. [963] demonstrated that B cells can suppress immunity through production of immunosuppressive cytokines.

Bregs is an umbrella term used for immunosuppressive B cells [964]. Bregs were often described in the context of chronic inflammatory diseases and in human they are mostly characterized by production of the suppressive cytokine IL-10 [964, 965]. Generally, expression of IL-10 has been a very useful marker for B cells with suppressive phenotype [964, 965]. However, several Breg-related surface markers can be either down- or upregulated upon stimulation, making it difficult when Breg subsets are compared among different stimulatory environments [965]. For example, CD25 and CD71 are often upregulated in activated B cells [966, 967] and they are widely used also as activation markers. Another activation marker, CD38, is expressed in naïve B cells and plasmablasts (the main IL-10 producing subsets) but is downregulated when naïve B cells develop into memory B cells [968]. Furthermore, CD1d can be downregulated upon stimulation [966]. A recent meta-analysis found a signature consisting of 126 genes common to human Bregs, many of which are involved in activation, differentiation, and regulatory functions of B cell [969]. However, in contrast to regulatory T cells, no Breg-specific transcription factor could be identified so far [965]. Furthermore, the high diversity of B cell subsets with suppressive capacity strongly suggests that there is not one single lineage of B cells giving rise to Bregs but that there are precursors from various stages of B cell ontogeny that gain suppressive phenotype in response to stimulation.

In mice, Bregs were reported to act mainly via production of suppressive cytokines IL-10, IL-35 and TGF-β [965] and inhibitory receptors such as LAG-3 [936]. IL-10 can suppress production of pro-inflammatory cytokines by APCs and induce T regulatory cells [970, 971]. IL-35 was reported to inhibit T helper 1 (Th1) cell responses [972], while TGF-β can inhibit APCs and induce apoptosis in Th1 cells as well as bring on anergy in CD8^+^ T cell [973, 974]. In murine spleen, CD19^+^, CD21^hi^ CD23^hi^ CD24^hi^ B cells (T2-MZP cells) [975-978] and CD19^+^, CD21hi CD23- B cells (MZ B cells) [979-981] were found suppressing CD4^+^ and CD8^+^ T cells while inducing Tregs. Similarly, IL-10-producing CD1d^high^ CD5^+^ B cells (B10) were found in the spleen, suppressing CD4^+^ T cells, dendritic cells (DC), as well as monocytes thereby playing a protective role in a plethora of mouse models including experimental autoimmune encephalomyelitis (EAE) [982, 983], lupus [984], myasthenia-gravis [985], collagen-induced arthritis [986], colitis [987], allergic inflammation [988, 989] and contact hypersensitivity [990]. Furthermore, IL-10 derived from CD1d^high^ CD5^+^ B cells also suppresses the immune response to Hepatitis-C virus by reducing cytotoxic T cell activity [991]. In spleen, also CD19^+^ TIM-1^+^ B cells were identified, suppressing CD4^+^ T cells [992, 993]. Interestingly, suppressive phenotype was also found among B cells of later differentiation stages, such as CD138^+^ CD44^hi^ plasmablasts [970], CD138^+^ MHC-11lo B220^+^ plasma cells [972, 994] and LAG-3^+^ plasma cells [936]. Suppressive plasmablasts were found in LN, suppressing CD4^+^ T cells and DCs [970] while IL-10 and IL-35 secreting plasma cells were found in spleen, suppressing effector CD4^+^ T cells as well as neutrophils and NK cells [972, 994]. LAG-3^+^ plasma cells, in addition to LAG-3 also expressed additional inhibitory receptors, including CD200, PD-L1, and PD-L2 [936]. A common characteristic among all the above-mentioned murine Breg subsets is their capability to produce of IL-10 [965].

**Figure 102.**
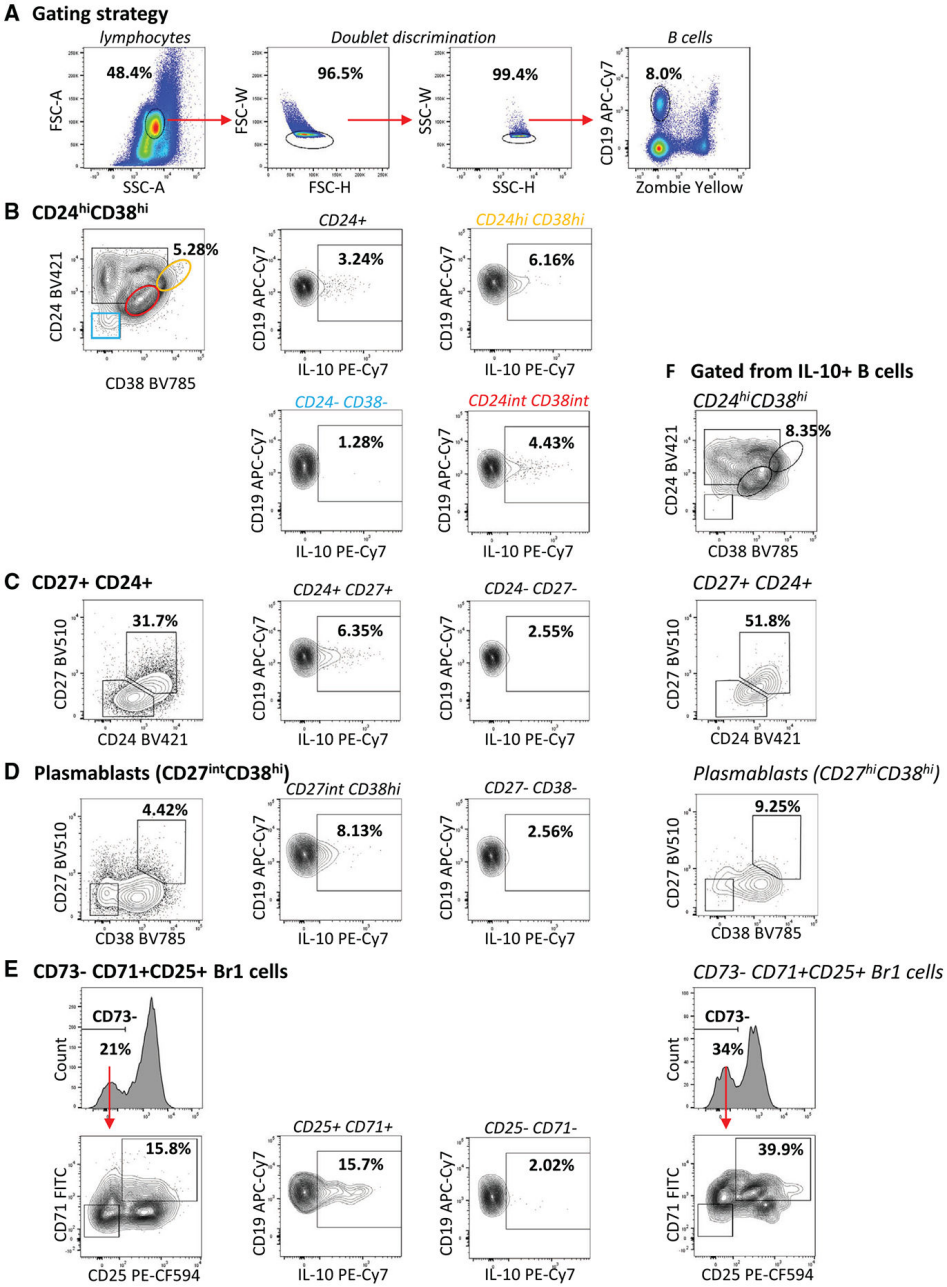
Identification of regulatory B cell subsets from human CpG-stimulated PBMC. PBMC from healthy female adult subject cultured for 72 h with media alone or media containing 1μM CpG-ODN 2006. Before staining, cells stimulated for 5 h with 25 ng/ml phorbol 12-myristate 13-acetate, PMA and 1 μg/ml iono and for the last 2h with 10 μg/ml BFA. Cells harvested and surface and intracellular Ab stainings performed. Total viable B cells gated from lymphocytes after doublet discrimination (A). Breg subsets gated from viable single CD19^+^ B cells (B-E). IL-10^+^ B cells gated from (B) CD19^+^ CD24^high^ CD38^high^ B cells, (C) B10/pro-B10 cells (CD19^+^ CD24^high^ CD27^+^), (D) suppressive plasmablasts (CD19^+^ CD27^int^ CD38^+^), and (E) CD19^+^ CD73^−^CD25^+^CD71^+^ B cells. Breg subsets gated from IL-10^+^ CD19^+^ B cells based on surface markers showing enrichment of IL-10^+^ B cells (F).

**Figure 103.**
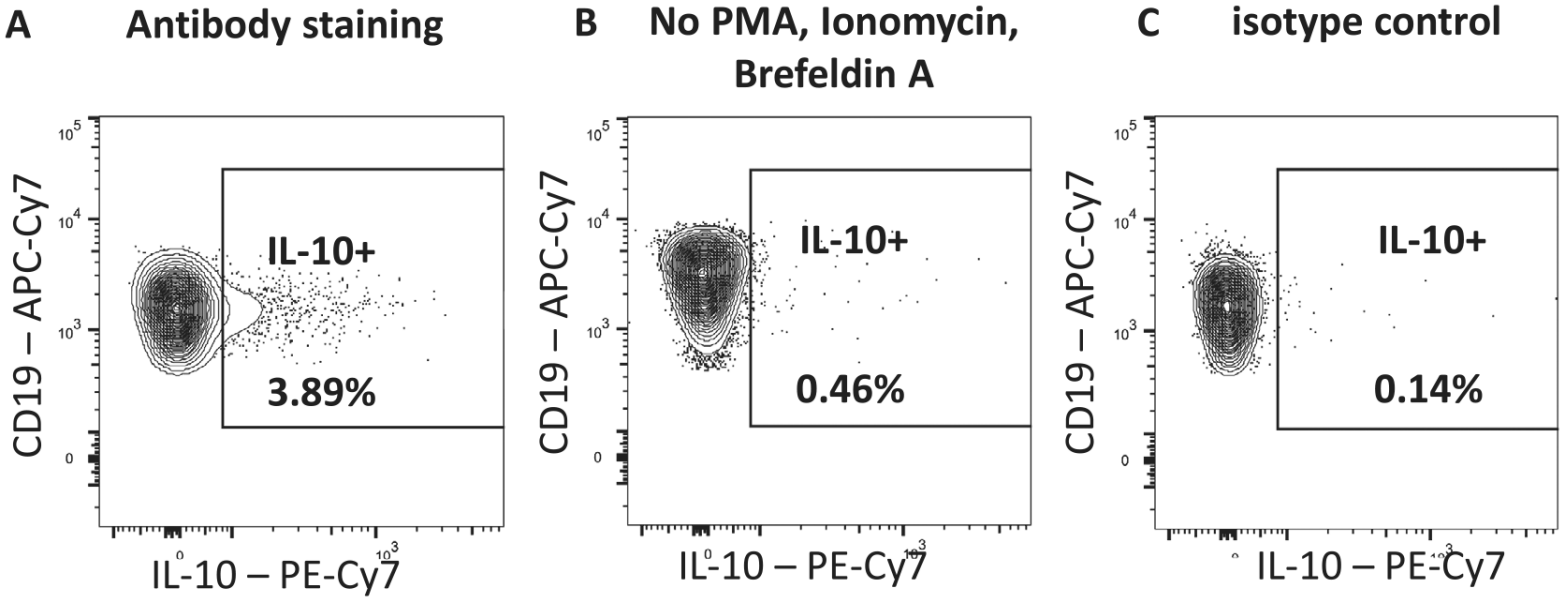
IL-10 staining and control stainings of human PBMC. PBMC cultured for 72 h with media alone or media containing 1μM CpG-ODN 2006. The last 5 h before staining, PBMC additionally stimulated with 25 ng/ml phorbol 12-myristate 13-acetate, PMA and 1 μg/ml iono and for the last 2h with 10 μg/ml BFA (A, C) or medium control (B). Viable B cells were gated as shown in Figure 102A. IL-10^+^ B cells were gated from total viable CD19^+^ B cells. Anti-IL-10 Ab staining is shown for B cells after stimulation with PMA, iono and BFA (A) or medium control (B). Isotype control staining is shown in (C).

In this section, we focus on human Breg cell subsets. The first data that indicated a potential role for regulatory B cells in humans came from the reports of new onset of colitis and psoriasis after anti-CD20 treatment with rituximab [995, 996]. In human Bregs, regulatory function is mainly conferred via secretion of IL-10. IL-10 can be produced by naive B cells [967, 997-1000], plasmablasts [970] from the blood, and plasma cells from tissue [1001] while it is unclear which subset is the most potent producer. IL-10 can be induced from peripheral human B cells by ligation of TLR9 (using CpG-ODN) [967, 970, 1000] or CD40 [997,1000] *in vitro*. Bregs originating from immature CD19^+^ CD24^high^ CD38^high^ B cells were found in blood and in inflamed tissue having a suppressive role in rheumatoid arthritis (RA), systemic lupus erythematosus (SLE), and chronic hepatitis B (CHB) virus infection [997-999]. These cells suppress Th1, TH17 cells, and virus-specific CD8^+^ T cells while inducing Tregs [997-999]. Suppressive B10/pro-B10 cells (CD19^+^ CD24^high^ CD27^+^ CD48^high^ CD148^high^) were found in blood suppressing CD4^+^ T cells, monocytes, and DCs [1000]. B10/pro-B10 cells regulate innate immunity and are upregulated in patients with various autoimmune diseases [1000]. IL-10-producing CD19^+^CD73^−^CD25^+^CD71^+^ Bregs play an important role in developing tolerance to allergens. This subset was shown to mature at increased frequency into plasma cells that secrete the suppressive Ab isotype IgG4 [967]. In addition, CD27^int^ CD38^+^ plasmablasts derived either from naïve immature B cells or naive mature B cells suppress effector CD4^+^ T cells and DCs by expressing IL-10 [970]. Recently, it was shown that in multiple sclerosis lesions, plasma cells (but not B cells) produced large amounts of suppressive IL-10 [1001]. A flow cytometry panel was described combining several Breg-associated markers, including CD19, CD1d, CD5, CD24, CD25, CD38, CD71, CD73 and IL-10 [966]. This allows to identify CD24^hi^ CD38^hi^ IL-10^+^ Bregs (Figure 102B), CD73^−^CD25^+^CD71^+^IL-10^+^ Bregs (Figure 102E) and a CD5^+^CD1d^high^ IL-10^+^ Breg subset, which was mainly described in mice. In human, CD1d was also reported to be more expressed in regulatory B cell subsets [1000, 1002]. Here, we included CD27, a marker for memory B cells, which allows additional distinction of CD19^+^CD24^high^CD27^+^ B10/pro-B10 cells (Figure 102C) and CD19^+^CD27^int^CD38^+^ suppressive plasmablasts (Figure 102D). These Breg subsets show enrichment for IL-10-producing B cells compared to total IL-10 producing B cells (Figure 103).

#### 4.3 Step-by-step sample preparation

This staining protocol is optimized for human peripheral B cells. PBMCs were isolated from heparinized blood of healthy individuals by density gradient centrifugation (Biochrom, Berlin, Germany). Isolated PBMC were directly plated and stimulated for 72 h with CpG-ODN. Before staining, cells were incubated with PMA and iono (5 h) and BFA (2 h), followed by viability staining with zombie yellow viability dye (Biolegend, San Diego, CA) and staining for surface markers with the Abs listed in Table 78 (see below) in staining buffer. Cells were washed, permeabilized and Ab staining for intracellular IL-10 was performed. Then, samples were washed and measured on a BD LSR Fortessa with BD FACS-Diva Software Version 8.0.1 and analyzed using Flowjo version 10.4.

Detailed protocol:

1. Collect fresh blood in heparinized containers (BD vacutainer 170 I.U. of lithium heparin)
2. Isolate PBMC:
  - Dilute blood samples at a 1:1 ratio with PBS supplemented with 2 mM EDTA.
  - For each 30 ml of diluted blood prepare a tube of Biocoll. Add 15 ml of Biocoll separating solution (room temperature) to a 50 ml blood-sep-filter tube. Spin down 1 min at 1100G to collect the Biocoll at the bottom of the tube below the filter.
  - Slowly add 30 ml of diluted blood to each filter tube and centrifuge the tubes at 800 × *g* for 20 min at 18 °C and no break at end of program.
  - Collect the interphases and pool up to 2 interphases and transfer them to a new 50 ml centrifugation tube. Fill up tube with PBS supplemented with 2 mM EDTA. Centrifuge tubes at 780 × *g* for 10 min at 18 °C.
  - Remove supernatant and resuspend cell pellet in 2 ml PBS supplemented with 2 mM EDTA. Pass cells through a 70 μM cell strainer into a new 50 ml centrifugation tube. Fill up tube with PBS supplemented with 2 mM EDTA. Centrifuge tubes at 220 × *g* for 10 min at 4°C.
  - Discard supernatant and resuspend PBMC in 5 ml of complete RPMI (cRPMI; RPMI 1640 medium supplemented with MEM Vitamin Solution, penicillin, streptomycin, kanamycin, MEM Non-essential Amino Acid Solution, Sodium pyruvate solution (Sigma-Aldrich Chemie GmbH), and 10% heat-inactivated fetal calf serum (FCS; Sigma-Aldrich Chemie GmbH).
3. Count PBMC and plate 2 million PBMC at concentration of 1 million/ml in cRPMI in 12 well tissue culture plate.
4. Stimulate PBMC with 1μM CpG ODN 2006 or medium (control) for 72h.
5. Harvest PBMC and spin down (300 × *g*, 10 min).
6. Staining for flow cytometry:
  - Briefly vortex cell pellet and stain cells with 100ul of Zombie yellow viability dye diluted 1:100 in staining buffer. Incubate 20’ at room temperature in dark. Wash 1 × with 1 ml of staining buffer.
  - Stain cells with Abs specific for surface markers listed in Table 78 (see below) in a total staining volume of 100μl. Incubate 15’ at 4°C in dark. Wash 1 × with 1ml of staining buffer.
  - Briefly vortex the cell pellet and resuspend with 250 μl of Fixation/Permeabilization solution (BD Biosciences, 554714) for 20 min at 4°C.
  - Wash the cell pellet two times with 1ml BD Perm/Wash^™^ buffer (BD Biosciences, 554714).
  - For intracellular staining of IL-10, dilute Ab in 50ul BD Perm/Wash^™^ buffer. As isotype staining control for IL-10, use isotype control Ab in equal concentration. Incubate 30’ in the dark at 4°C.
  - Wash the cells two times with 1ml BD Perm/Wash^™^ buffer (BD Biosciences, 554714).
7. Resuspend cell pellet in 200μl staining buffer and measure sample.

#### 4.4 Materials

The materials used for the analysis of human peripheral B cells are listed in Table 77.

#### 4.5 Data analysis

We first gate on lymphocytes in forward versus side scatter, followed by doubled discrimination (Figure 102A). From single cells we gate on viable (Zombie Yellow negative) CD19^+^ B cells. Total IL-10^+^ cells are gated from viable B cells (Figure 103A). B cells can be sub-gated into CD24^+^ CD38^+^ IL-10^+^ Bregs (Figure 102B), CD19^+^ CD24^+^ CD27^+^ B10/pro-B10 cells (Figure 102C), CD19^+^ CD27^int^ CD38^+^ suppressive plasmablasts (Figure 102D) and CD73^−^ CD25^+^ CD71^+^ IL-10^+^ Bregs (Figure 102E). These four Breg subsets contain increased percent-age of IL-10^+^ B cells compared to total IL-10 producing B cells (Figure 102, B-E, Figure 103A). Alternatively, when we gate Breg subsets based on their surface marker expression from total IL-10^+^ B cells, we find that these Breg subsets are enriched among total IL-10^+^ B cells (Fig. 102F). However, even tough IL-10+ B cell are enriched among gated Breg subsets, there is also other IL-10-producing B cells, which are not falling into a described Breg gate. Also, Breg subsets are overlaping to some extend.

#### 4.6 Pitfalls

Some of the surface-expressed proteins used as markers for Bregs are also known as general activation markers. Depending on the stimulation used, surface markers of Bregs therefore might be up- or downregulated. For example, upon stimulation with CpG or BCR ligation+CD40L for 72 h, it was seen that while number of CD19^+^ CD73^−^CD25^+^ CD71^+^ B cells (Br1 cells) showed a tendency to be increased among total IL-10 producing B cells, CD19^+^ CD24^+^CD38^+^ B cells and CD19^+^CD1d^+^CD5^+^ B cells were decreased [966]. Therefore, it is important to carefully consider the length of the stimulation period when performing *in vitro* stimulation of regulatory B cells.

#### 4.7 Top tricks

Depending the exact research question and on the flow cytometer available, several other Abs can be included to the panel. Since B cells sometimes produce inflammatory cytokines (e.g., TNF) together with IL-10 [1003], it can be useful to include Abs for the exclusion of inflammatory cytokine-producing cells.

#### 4.8 Clinical relevance statement

The gating strategy shown in this section is applicable for detection of different Breg subsets which are shown to play important roles during allergen tolerance [967], chronic HBV infection [998], or various autoimmune diseases [1000], some of which are functionally impaired in Lupus Erythematosus [997] and RA patients [999]. While increased levels or activity of certain B cell subsets seem to be beneficial during allergen tolerance and certain autoimmune diseses [967, 1000], Breg cells might play detrimental roles suppressing immune responses to pathogens [998].

#### 4.9 Summary of the phenotypes

This is detailed in Table 78.

### 5 Murine B cells and their subsets, including Bregs

#### 5.1 Overview

Several murine B cell subsets are known that differ with respect to differentiation stage, B cell receptor repertoire, contribution to the production of natural and adoptive Abs, pro- and anti-inflammatory capacity, tissue localization and distribution within tissues. In addition to various B cell subsets, the bone marrow (BM) also contains B cell precursors, which express the prominent "B cell markers" CD19 and B220 (CD45R). Here, we describe strategies to identify the most prominent murine B cell precursors and mature B cell subsets in the BM, as well as secondary lymphoid organs. Plasma cells are not included but are described separately in the section V. 8 “Murine Ab-secreting plasmablasts and plasma cells”.

#### 5.2 Introduction

Human and murine B cells exhibit many similarities in terms of their subpopulations, development, and function. Therefore, results obtained in studies investigating B cell compartments in murine models have significantly contributed to our current understanding of immunity. Three murine B lymphocyte lineages B-1a, B-1b, and B-2 cells have been described to exhibit different ontogenies [1004], and can be further subdivided into several subsets and developmental stages, including immature and mature, follicular, marginal zone (MZ), germinal center (GC) B cells, among others. While all B cell lineages are capable of giving rise to Ab-secreting plasma cells, they exhibit different B cell receptor repertoires (BCR), recognize different Ags (protein-, lipid- or carbohydrate Ag), express different Toll-like receptors, and contribute preferentially either to T-independent (B-1a, B-1b; MZ) or to T-dependent (follicular B cells) Ab responses [1005-1008].

B-1 cells are the major B cell subset in the body cavities, e.g., the peritoneum. These cells represent an important source of innate Abs that are produced independently of foreign Ag and T cells, and resemble an important first line of defense against infection [1009]. Stall et al. were the first that described the existence of two distinct subsets of B-1 B cells, termed B-1a and B-1b [1010]. Self-renewing populations of CD5^pos^ B-1a and CD5^neg^ B-1b cells are found in the peritoneal cavity of adult mice, a small population of B-1a cells is also present in spleen [1011, 1012]. B-2 cells are continuously generated from progenitors found in the adult BM [1004]. This tissue contains various B cell progenitors, including a small population of CD19^pos^/B220^low/neg^ B-1 B cell progenitors, CD19^neg^/B220^pos^ B-2 progenitors, immature B cells, but also a significant population of re-circulating mature B cells, representing successive developmental stages defined according to surface marker expression and immunoglobulin (Ig) gene rearrangement status [1013, 1014]. The complex rear-rangements that produce complete immunoglobulin (Ig) heavy and light chains occur during B cell development. This process of somatic mutation is described in detail elsewhere [1013, 1015-1018]. The principal B cell progenitor populations in the BM are pre-pro B cells, pro-B cells, and pre-B cells, which can still be further subdivided into various developmental stages such as early pro-, late pro-, large pre-, small pre-B cells, among others [1019, 1020]. Of note, these distinct B cell developmental stages are associated with particular Ig-gene rearrangement patterns [1020-1022].

Immature B cells leave the bone marrow and migrate to the spleen to eventually become mature B cells. This process involves the three transitional stages T1, T2, and T3 that are subjected to negative selection of autoreactive cells, while only non-self-reactive cells become fully mature and functional [1023]. The main mature B cell populations in the spleen are follicular B cells and MZ B cells, while B-1 cells represent only a minor population [1024, 1025].

Controlled by autocrine expression of low levels of IFN-γ, immature B cells selectively migrate to the spleen neglecting other secondary lymphoid organs [1026]. A MZ is not existing in these tissues. Therefore, lymph nodes and Peyer’s patches contain mainly follicular B cells of the B-2 lineage. Here, we provide strategies for the analysis of the main B cell and B cell progenitor stages, with a focus on the analysis of BM and secondary lymphoid tissues, each containing several different B cell subpopulations.

#### 5.3 Step-by-step sample preparation

Single-cell suspension of murine spleens, bone marrow (femurs and tibia) and the peritoneal cavity were prepared and filtered through a 70 μM cell strainer (BD Falcon). The primary cells were resuspended (10^7^ cells/ml) in PBS containing 0.5% BSA. Fc receptors were blocked with anti-CD16/CD32 for 10 min at 4°C (5μ/ml in PBS/BSA, clone 2.4G2, in house production). Subsequently, cells were washed with ice-cold PBS/0.5%BSA, and incubated with fluorescent labeled Abs for 15 min at 4°C. After washing twice, cells were resuspended in PBS/0.5% BSA/ supplemented with 100μ/ml DNase I, and analyzed by flow cytometry. For staining of intracellular IL-10, cells were resuspended in cell culture medium and stimulated for 5 h with 3.3 ng/ml PMA, 1 μ/ml ionomycin and 10 μ/ml LPS at 37°C and 5 % CO2. Brefeldin A (3 μ/ml) was added during the last 4 h to block protein transport. Surface staining was performed as described above, cells were washed once with PBS and fixed and permeabilized with Fix/Perm buffer kit according to the manufacturer protocol. Fixation and permeabilization were done each for 20 min at room temperature (RT), and intracellular staining was performed for 20 min at RT.

#### 5.4 Materials

##### Antibodies and buffers.

Culture medium contained RPMI1640+Glutamax, 10% fetal calf serum, 1 % Penicillin/Streptomycin and 0.2% β-2-mercaptoethanol (Gibco/Thermo Fisher), besides the stimulation reagents PMA (Sigma-Aldrich), Ionomycin (Merck Millipore), LPS (Sigma-Aldrich) and Brefeldin A (eBioscience). Fix/Perm buffer kit (BioLegend) was used to fix and permeabilize cells prior to intracellular staining. PBS (8 g/l NaCl, 0.2 g/l KCl, 1.44 g/l Na2HPO4) containing 0.5% BSA was used for washing and as staining solution. Anti-mouse Abs used in flow cytometry staining analysis: anti-CD1d (clone 1B1, BD Pharmingen); anti-CD11b (clone M1/70.15.11, in house production); anti-CD19 (clone 1D3, BioLegend, Fell, Germany); anti-CD5 (clone 53-7.3, BioLegend); anti-B220 (clone RA3.B2, in house production); anti-IgM (clone M41, in house production); anti-IgD (clone 11-26c.2a, BioLegend); anti-CD21/CD35 (clone 7E9, BioLegend); anti-CD23 (clone B3B4, BioLegend); anti-CD43 (clone S11, BioLegend); anti-CD38 (clone 90/CD38, BD Pharmingen); anti-GL7 (clone GL7, BioLegend); Fixable Viability Dye (eBioscience); anti-IL-10 (clone JES5-16E3, Biolegend).

##### Flow cytometer and analyzing software.

Samples were measured in an LSRII flow cytometer (BD Biosciences) and the resulting data were analyzed using the FlowJo software (TreeStar, Ashland, OR).

#### 5.5 Data analysis: Murine B lineage cells in BM

**Figure 104.**
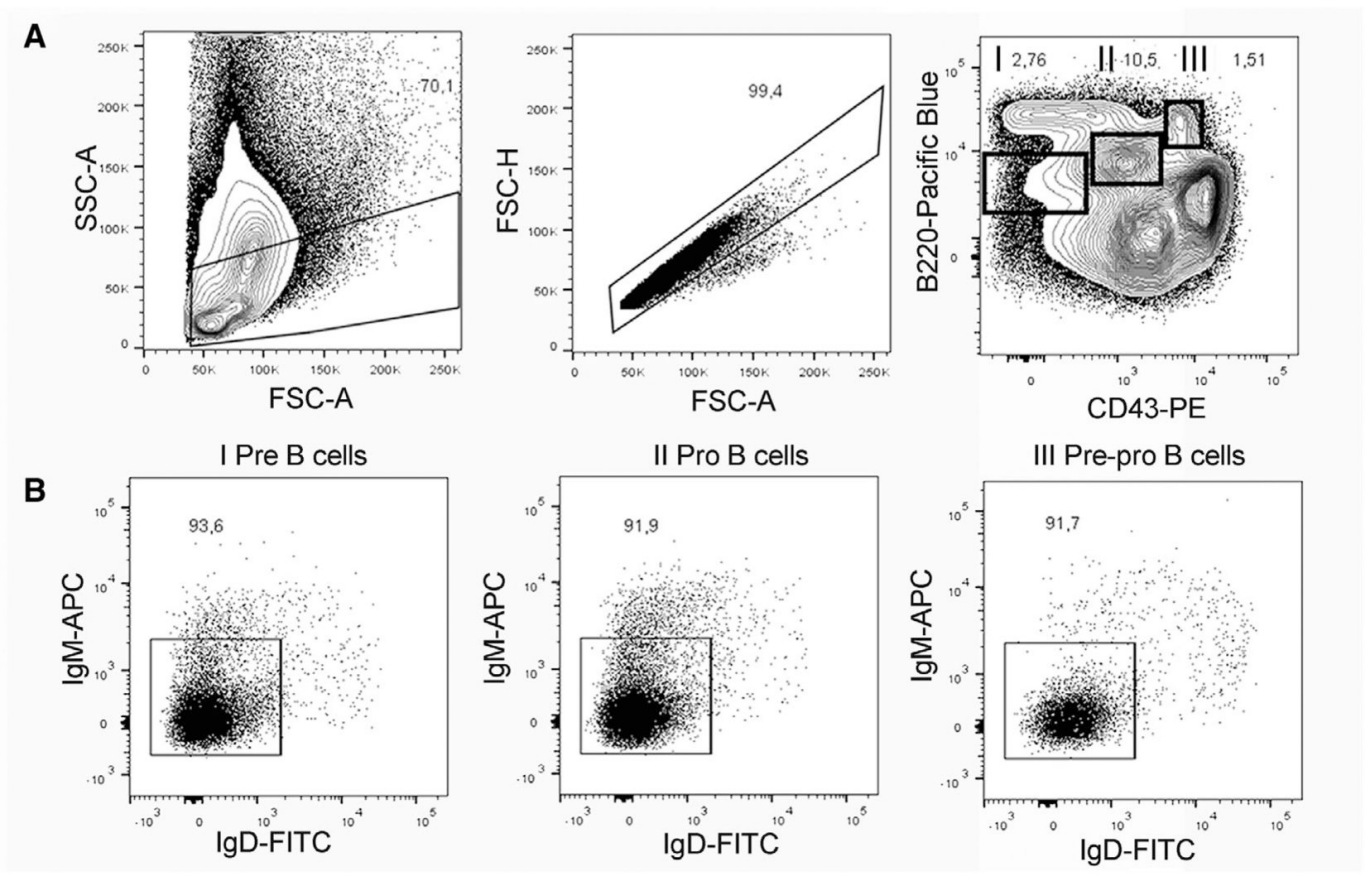
Discrimination of murine B cell progenitors in BM. Single cell suspensions from BM were stained for B220, CD43, IgM and IgD. (A) Left plot: Gating strategy to exclude debris. Middle plot: Gating strategy to exclude doublets. Right plot: Pre-B cells (gate I), pro-B cells (gate II) and pre- pro-B cells (gate III) are identified by their distinct B220/CD43 phenotypes. (B) Cells were gated through the gates I, II or III as indicated. Exclusion of IgD^pos^ and IgM^pos^ cells eliminates contaminating immature and mature B cells.

After isolation and generation of single cell suspensions, typically from femurs and/or tibia, BM lymphocytes should be gated according to their scatter properties, and doublets should be excluded from the analysis, similar to the analysis of human B cells (see section 1 Human B cells and their subsets). However, in contrast to human peripheral blood, murine bone marrow contains many proliferating B cell blasts, therefore the forward scatter gate should be not too restrictive (Figure 104A). Removal of dead cells by using either DNA stains such as propidium iodide, 4,6-diamidine-2-phenylindole or 7-amino-actinomycin D, or alternative stains that bind to amines of proteins can be used to further improve the quality of the data. The choice of the most suitable live/dead discrimination reagent depends on the individual project, e.g., if the samples need to be fixed [22].

In order to cover the major B lineage developmental stages in BM from very early progenitors to the mature B cell stage using only a minimum number of different markers, we suggest staining with a combination of CD19, B220, CD43, IgM, and IgD mAb. Depending on the specific purpose of the study and the availability of more fluorescent channels, these markers could be complemented by additional ones.

CD19 and B220 serve as specific surface markers for the identification of B lineage cells. CD19 is a co-receptor of the B-cell receptor which is expressed under the control of the PAX5 encoded "B-cell lineage specific activator protein" [1027]. B220 and CD19 are found on the surface of all later B lineage cells except for a subpopulation of terminally differentiated plasma cells [1028].

As originally described by Hardy and colleagues, pre-pro B cells, pro-B cells, and pre-B cells are defined according to their distinct expression pattern of B220 and CD43 [1029], Pre-pro B cells resemble very early precursors showing a B220^pos^/CD43^pos^ phenotype. Pro-B cells and pre-B cells are B220^pos/int^/CD43^pos^ and B220^low^/CD43^neg^, respectively (Figure 104A). All three progenitor populations are distinguishable from the later immature and mature stages by the absence of IgM and IgD expression. Hence, exclusion of IgM ^pos^ and IgD ^pos^ cells could help to test for the accuracy of the gating (Figure 104B).

**Figure 105.**
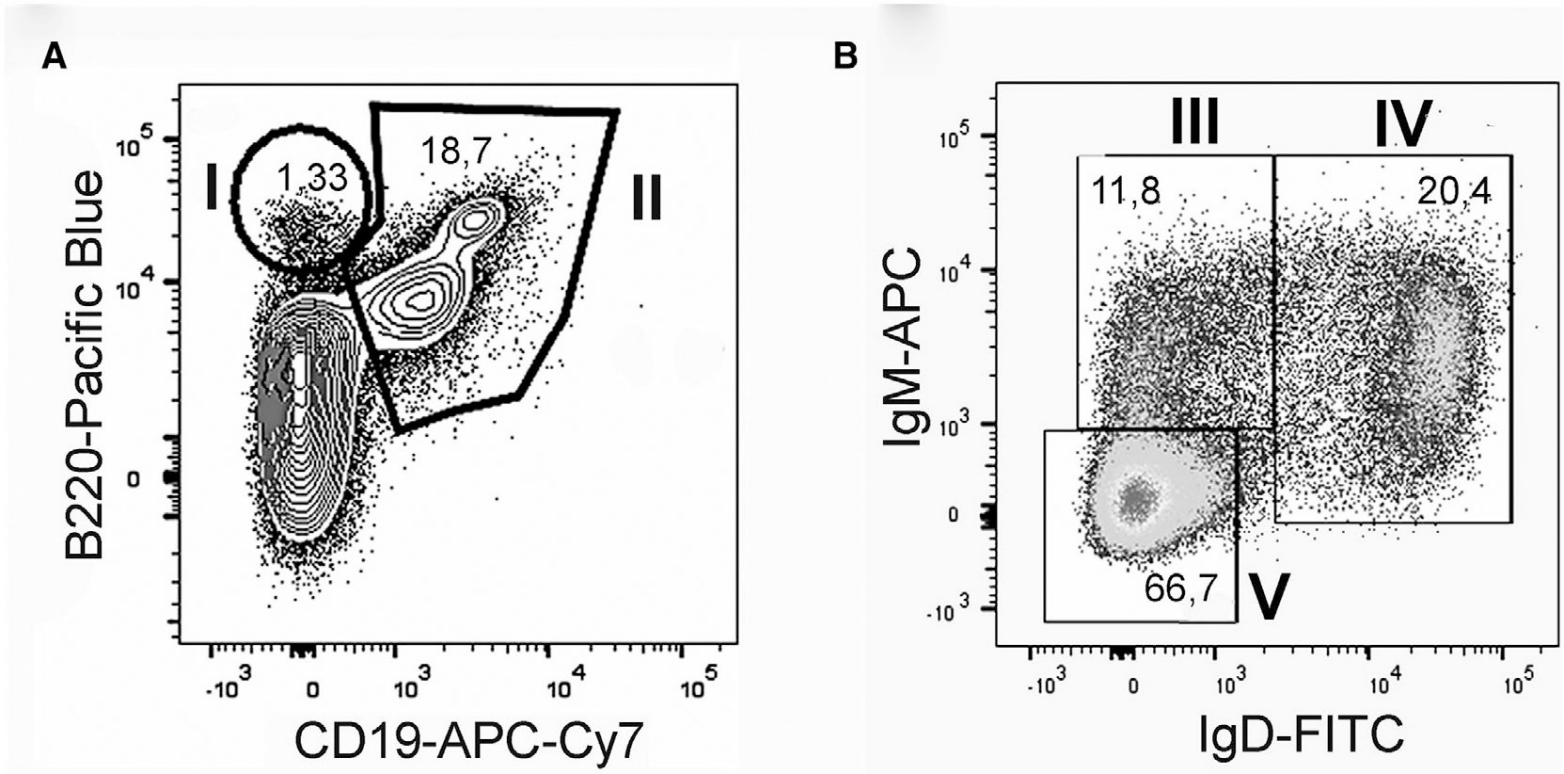
Discrimination of murine immature and mature B cells in BM. Single cell suspensions from BM were stained for CD19, B220, IgM and IgD, and doublets and debris were excluded by gating (see Figure 104). (A) B220^high^/CD19^neg^ cells (gate I) include pre-pro B cells, while all other B cell subtypes (except plasma cells) are included in the B220 ^high^/CD19^pos^ population (gate II). (B) Cells were gated through gate II. Immature (gate III) and mature B cells (gate IV) were identified according to their IgM/IgD phenotypes. Gate V includes a mixture of pre- and pro B cells.

Immature and mature B cells exhibit an CD19^pos^/B220^pos^/CD43^neg^/IgM^pos^/IgD^neg^ and CD19^pos^/B220^pos^/CD43^neg^/ IgM^pos^/IgD^pos^ phenotype, respectively [1030, 1031]. Following staining with CD19, B220, CD43, IgD, and IgM, all B lineage cells except plasma cells and pre-pro B cells are included within the CD19^pos^/B220^pos^ population (Figure 105A). Pre-pro B cells are found within the B220^high^/CD19^neg^ fraction. However, this population does also contain non-B lineage cells [1032]. Pro-B cells, pre-B cells, immature and mature B cells are included within the CD19^pos^/B220^pos^ populations. Immature and mature B cells can be further discriminated by the expression of surface IgM and IgD (Figure 105B).

According to the complexity of the B cell development and heterogeneity of B lineage cells, other marker combinations are useful to study B lineage cells in bone marrow as well. The Basel nomenclature of B cell development classifies B cell progenitors differently from the Hardy system described above [1033]. B cell progenitor phenotypes defined by the surface markers CD25 and CD117 (c-kit) correlate with the stepwise rearrangements of the genes coding for the Ig heavy and light chains [1034, 1035]. The Ig gene loci are rearranged in an ordered fashion, with the D-heavy (D_H_) segments being first rearranged to -J-heavy (J_H_) segments, followed by V heavy (V_H_) to D_H_ J_H_. The gene loci coding for the Ig light chains are rearranged later, after successful rearrangement of the Ig heavy gene segments [1036]. B220^pos^/CD117^pos^/CD25^neg^ cells typically exhibit rearrangements of the D_H_ - J_H_ Ig-gene segments, with light chain loci in germline configuration. This population resemble early pre-B cells (pre-B I cells) which are the precursors of large B220^pos^/ CD25^pos^ cells which, in turn, are the precursors of small B220^pos^/CD25^pos^ cells [1037]. Since all these progenitor stages do not have completed their Ig gene rearrangements yet, they are surface IgM^neg^/IgD^neg^. The great majority of large B220^pos^/CD25^pos^/IgM^neg^/IgD^neg^ cells have at least one heavy chain locus V_H_D_H_J_H_ rearranged. These cells are called large pre-B cells (pre-B II cells). Staining for additional markers such as CD93 (AA.4.1), CD23 which is also called heat-stable Ag (HSA), surrogate light (SL) chains VpreB and lambda5 can be used to perform a more detailed analysis of B lineage subpopulations in BM [1020, 1021, 1029-1031, 1038, 1039] (Table 79; see below).

#### 5.6 Data analysis: Murine B cells in secondary lymphoid organs

**Figure 106.**
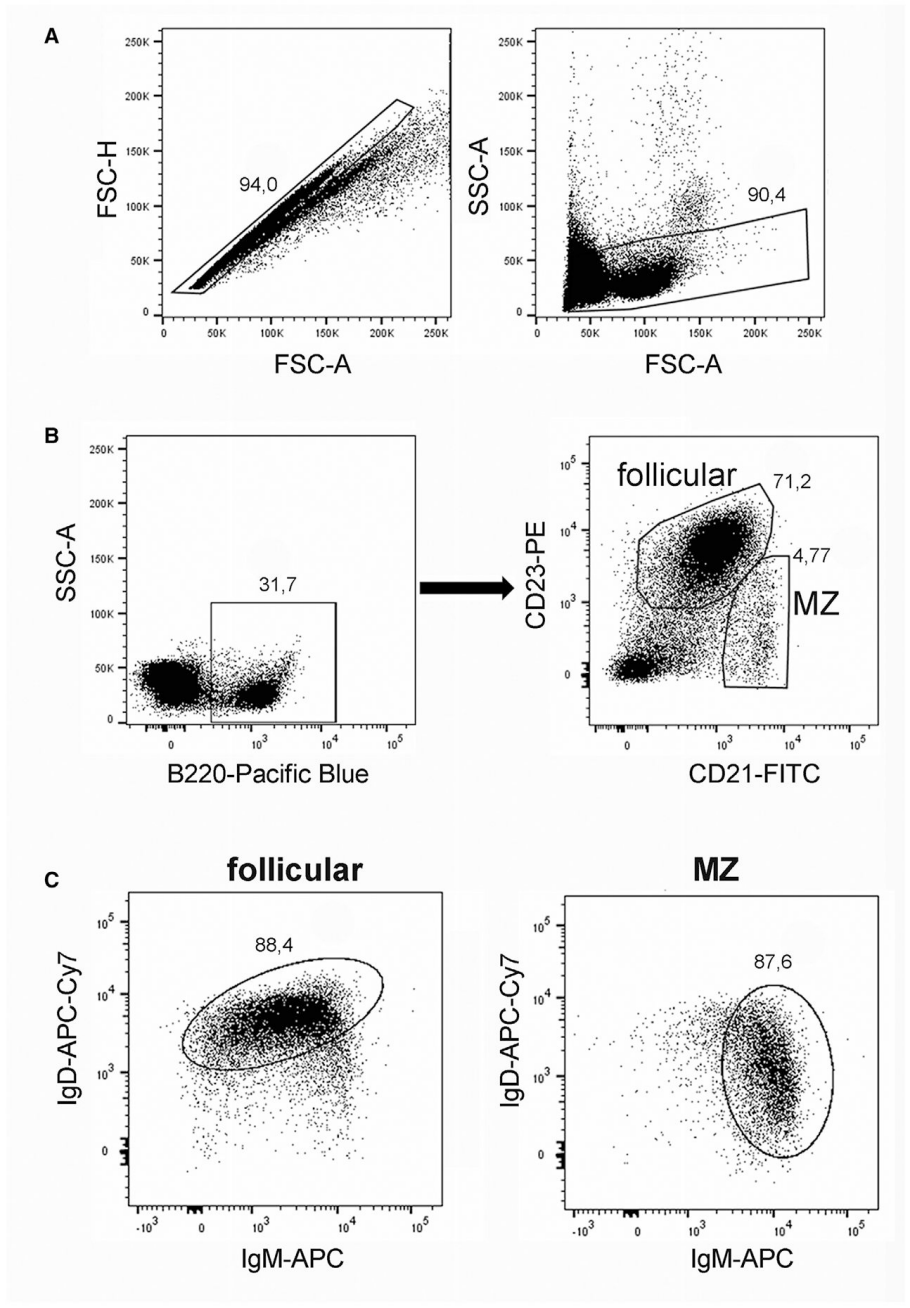
Analysis of murine follicular and MZ B cells. Single cell suspensions from spleen were stained for B220, CD21, CD23, IgM and IgD. (A) Gating strategy to exclude doublets and debris. (B) B cells are gated according to B220 expression and follicular and MZ B cells were further discriminated by their CD21 ^intmed^/CD23^high^ and CD21^high^/CD23^low/neg^ phenotype, respectively. (C) Gated follicular and MZ B cells exhibit distinct IgD/IgM expression characteristics.

For identification of B cells in the spleen and other secondary lymphoid organs, single cells should be gated according to their scatter properties, and doublets should be excluded from the analysis. In order to avoid exclusion of activated/proliferating B cells, the forward scatter gate should be not too restrictive (Figure 106A). Exclusion of dead cells through application of live/dead discrimination reagents is strongly recommended [22], this measure is of crucial importance particularly when smaller subpopulations are included in the analyses.

The spleen contains MZ B cells that are unique to this organ. The immature B cells stages T1, T2 and T3 are also selectively found in the spleen. They can be identified by a set of markers including CD21, CD23, IgD, IgM, among others. An overview of marker combinations suitable to identify the various B lineage cells in spleen is provided in Table 80 (see below). In contrast, lymph nodes and Peyer’s patches contain neither MZ nor immature B cells, but harbor mainly follicular B cells.

In spleen and other secondary lymphoid tissues, all B cells are CD19^pos^ and B220^pos^ (of note, not all plasma cells express these two markers, see section V. 8 - Murine Ab-secreting plasmablasts and plasma cells). Therefore, CD19 or B220 can be used as alternative markers for the identification of B lineage cells in these tissues. In spleen, staining for B220 (or CD19), CD21, CD23, and IgM allow identification of follicular B cells and MZ B cells [1040, 1041]. We also recommend to stain additionally for IgD. Using this marker combination, follicular B cells are identified by their B220^pos^/CD21^intmed^/CD23^hlgh^ phenotype, MZ B cells are B220^pos^/CD21^high^/CD23^low/neg^ (Figure 106B). While their characteristic B220/CD21/CD23 expression profile is sufficient to identify follicular and MZ B cells, their identity can be further proven by their distinct IgD^pos^/IgM^intmed^ and IgD^low/neg^/IgM^high^ phenotype, respectively (Figure 106C). After further gating B220^pos^ cells on IgM vs CD21 and CD23, this marker combination also allows to identify T1 and T2 cells [1042].

All secondary lymphoid organs can contain GCs where B cells can develop Abs of increased affinity, after proper stimulation in the context of a T-dependent immunization. GCs are transient structures present after immunization with T-dependent (protein) Ags which are absent in steady state. Flow cytometric analysis of GC B cells is described in section Murine Germinal Center B cells. Eventually, the GC reaction gives rise to plasma cells and memory B cells. Plasma cells are described in detail in the section V. 8 - Murine Ab-secreting plasmablasts and plasma cells. Memory B cells are found in spleen and in the peripheral blood. The murine B cell memory compartment appears in several subsets and exhibits a very heterogeneous phenotype [1043].

Memory B cells specific for one particular Ag can be identified by staining with fluorescent-labeled Ag. However, due to the low frequencies of these cells and unspecific binding to other B cells, this method is challenging and needs careful controls [1044, 1045]. Usage of adoptive transfer of B cells from BCR transgenic mice allows to increase the frequencies of monospecific B cells. In the recipients, these cells could be identified by staining with a fluorescent-labeled Ag or by an idiotype specific marker. Staining for CD35, CD73, CD80, and CD273 (PD-L2) defines functionally distinct subsets of murine memory B cells, including IgM^pos^ and IgG^pos^ cells [1046-1051]. The spleen also contains a small but considerably population of B-1 cells, which makes up for approx-imately 1.5% - 2% of spleen B cells. By numbers, this B-1 population is comparable to the B-1 population in the body cavities. Moreover, recent data suggest a role of the spleen for maturation and maintaining the B-1a compartment [1012, 1052].

#### 5.7 Data analysis: B-1 cells in peritoneum

B-1 cells mainly reside in the peritoneum, the pleura, and other body cavities [1053]. Compared to other B cells, they express lower levels of B220, but normal or high levels of CD19. Therefore, we recommend the use of CD19 mAb to identify B cells in body cavities.

B-1 cells come in two flavors, B-1a and B-1b which are distin-guishable by differential expression of CD5.

**Figure 107.**
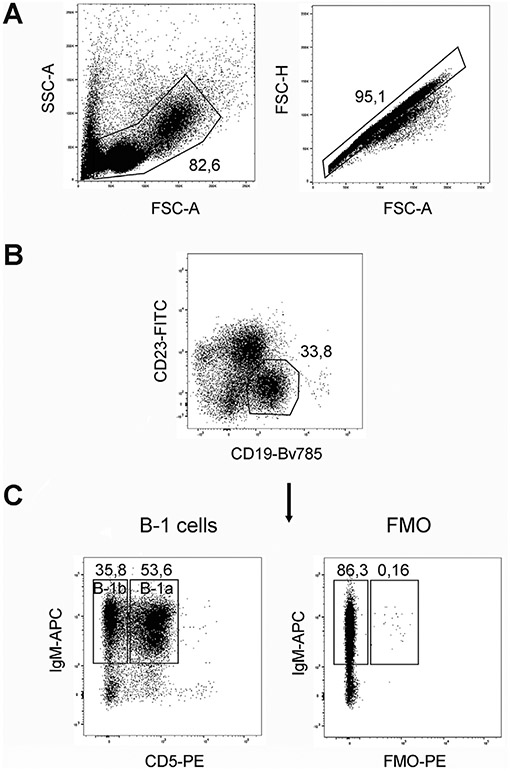
Analysis of murine B-1 cells. Single cell suspensions from the peritoneal cavity were stained for CD19, CD5, CD23 and IgM. (A) Gating strategy to exclude doublets and debris. (B) B cells were identified by CD19. (C) IgM^pos^ B-1a and B-1b cells are distinguished according to CD5 expression, as indicated.

After isolation, peritoneal lymphocytes should be gated according to their scatter properties, and doublets should be excluded from the analysis, similar to the analysis of human B cells (see section 1 Human B cells and their subsets). However, due to the distinct cellular composition of the murine peritoneum, the scatter profile looks different (Figure 107A). To some extent, B-1 cells recognize self-Ags and may often exhibit an activated blast phenotype even in the absence of an external stimulus [1054]. Therefore we recommend to set the forward scatter gate not too restrictive. Removal of dead cells is recommended in case the peritoneal immune cells were activated, e.g., by infectious agents, thioglycollate, or other stimuli. B-1a and B-1b cells could be identified according to their CD19^pos^/CD23^neg^/ IgM^pos^/CD5^pos^ and CD19^pos^/CD23^neg^/ IgM^pos/^ CD5^neg^ phenotye, respectively (Figure 107B/C).

#### 5.8 Data analysis: Regulatory B cells

B cells can modulate immune responses and induce or suppress inflammation through the production of Abs, but also by contact- dependent interactions and through the secretion of various pro- and anti-inflammatory cytokines such as IFN-gamma, GM-CSF, IL-10, IL-17, and IL-35 [972, 1055-1059].

B cells with a suppressive function are generally termed “regulatory B cells” (Bregs) [1060]. Breg subtypes typically express immunosuppressive IL-10 but otherwise exhibit a very heterogeneous phenotype [1059]. Several protocols are in use to identify Breg subtypes by distinct combinations of markers such as IL-10, IgM, IgD, CD1d, CD5, CD21, CD23, CD24, CD43, and CD93, among others [936, 965, 970, 975, 977, 980, 981, 993, 1054, 1056, 1061-1066] (Table 81; see below).

**Figure 108.**
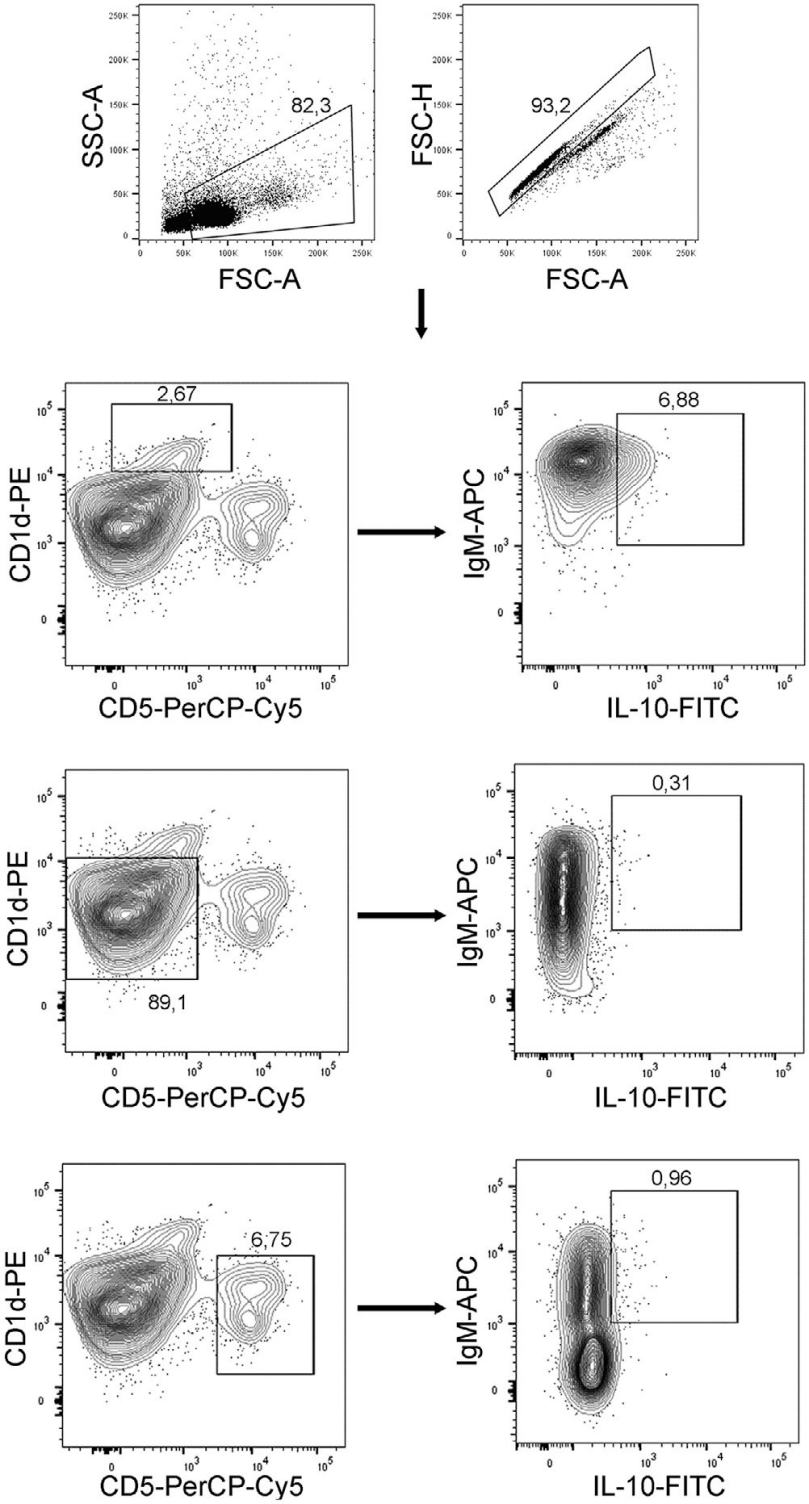
Analysis of murine Bregs. Following 5 hours stimulation with PMA/ionomycin/LPS, single cell suspensions from the spleen were stained for B220, CD1d, CD5, and IgM. Cells were then fixed and stained for cytoplasmic IL-10 expression. Doublets and debris were excluded from the analysis as described above (upper plots). B220^+^B cells were further analyzed by gating on CD1d^high^/CD5^intermed^ B-10 cells (upper row, left plot), CD1d^neg^/CD5^neg^ B-2 cells (middle row, left plot) and CD1d^neg^/CD5^pos^ B-1 cells (lower row, left plot). Intracellular IL-10 expression of B10 cells, B-2 cells and B-1 cells are shown, respectively (right plots).

At least one of the IL-10 producing Breg subtypes termed B10 cells, which exhibits a CD1d^hi^/CD5^pos^/CD19^pos^ phenotype (Figure 108), represents a direct precursor stage of Ab-secreting cells [1061]. Interestingly, production of immunosuppressive IL-10 is common among Ab-secreting plasmablasts and plasma cells [970, 1062, 1063]. Recently, the inhibitory receptor LAG-3 was identified as a marker for a population of natural regulatory plasma cells [936].

#### 5.9 Pitfalls

Please see section 1 Human B cells and their subsets.

#### 5.10 Top tricks

B lineage cells exhibit a broadly heterogeneous phenotype, which is relevant particularly in tissues such as BM or activated LN, a typical source for murine B cells. In these tissues, B cells include proliferating and/or activated cells that are larger than resting lymphocytes and might be excluded by the usual lymphocyte scatter gate. Moreover, B cell subsets can express markers typical for other lineages, i.e., the "myeloid marker" CD11b which is found on B-1 cells in the body cavities. Therefore, we recommend to start the analysis of the cytometric data with an unbiased approach, avoiding exclusion of cells potentially belonging to the B lineage. This could be achieved by analyzing all CD19 or B220 positive cells among the total, ungated population. In a second step, non B lineage cells could be excluded by appropriate gating.

#### 5.11 Summary of the phenotypes

This is detailed in Tables 79, 80 and 81.

#### 5.12 Key information human vs murine B cells

This is detailed in Table 82.

- B220 is commonly used to identify B cell subsets in mice, but not in humans
- CD38 is commonly used to identify human plasmablasts and plasma cells but is not suitable to identify plasma cells in mice.
- CD27 is suitable and widely used to identify human, but not murine memory B cells
- High expression of CD27 identifies human, but not murine plasmablasts and plasma cells

### 6 Human antibody-secreting plasmablasts and plasma cells

#### 6.1 Overview

Plasma cells (PC) are terminally differentiated B lymphocytes specialized in large-scale Ab production and secretion. PC are implicated in both protective and pathogenic humoral immunity, and, as long-lived cells, in immune memory. Thus, they are being studied as therapeutic targets for the treatment of Ab-mediated diseases and as biomarkers for B cell activation in various clinical settings including infection, autoimmunity and inflammation and vaccination.

In this chapter, we describe the detection human PC and their circulating precursors, the plasma blasts (PB), by flow and mass cytometry. The vast majority of PB and PC are characterized by high expression of CD27 and CD38, low or no expression of CD20, and variable expression of CD19, HLA-DR, and CD138. PB/PC represent approximately 0.01% - 1% of leukocytes in different tissues, and phenotypical characteristics of PB/PC are associated with tissue origin, cellular maturation and differentiation, and clinical context. Their low frequency, variable phenotype, and their unique light scatter properties require attention when analyzing PB and PC by flow cytometry.

#### 6.2 Introduction

Plasma cells (PC) are terminally differentiated B cells capable of continuous production of Ab [1076]. Apart from their immediate precursors, the PB, and minor fractions of circulating CD20^+^CD43^+^ B cells [1077] they are the only cells of the body that actively secrete Ab and contribute the vast majority of immunoglobulins detectable in serum and mucosal secretions. Thus, PC (also termed plasmacytes, plasmocytes, spot-forming cells (SFC), Ab-secreting cells (ASC), Ab-forming cells (AFC), plaque-forming cells (PFC), or immunoglobulin-secreting cells (ISC)) are the foundation and the cellular correlates of humoral immunity by secreting specific, commonly adaptively shaped Abs that neutralize or opsonize pathogens. Long-lived (or memory) PC persist for longer times *in vivo* as metabolically active and non-proliferating cells, particularly in the bone marrow (BM) but also in the intestinal lamina propria [1078] and directly contribute to immune memory by long-term secretion of specific Ab [1079, 1080], a phenomenon termed humoral (or serological) memory.

PB and PC derive from B cells activated through the B cell Ag receptor (BCR), in the context of, e.g., cytokines, TLR ligands, cognate T cells, or combinations thereof. After activation, B cells start proliferating and differentiating into PB, or memory B cells. *In vivo*, this activation can target naive or Ag-experienced memory B cells, and leads B cell differentiation in germinal centers (GC) or extrafollicular structures, paralleled by somatic hypermutation and class-switching of Ig gene rearrangement especially within GC reactions, resulting in the generation of memory B cells and PB. In line with the observation that the differentiation of PB is fairly easy to mimic *in vitro* by different stimuli [1081, 1082], PB differentiation appears to be the default differentiation pathway.

Once formed, PB may either reside at the site of their generation (such as spleen or lymph nodes), or emigrate and transit via the blood to PC deposits in the gut lamina propria (LP) or the bone marrow (BM), or die. Immunization studies have been particularly useful for determining Ag-specific PB dynamics and biology in man [901, 941, 942, 1076, 1083, 1084]. While PB and PC are present in peripheral blood at all times at low frequency [1084] and common in other lymphoid tissues such as spleen and BM [901, 1076], additional vaccine-specific PB appear as a sharp peak approximately one week after intramuscular or subcutaneous immunizations [935, 941]. Their presence in blood lasts longer when the immunization occurred via mucosal routes [1085]. Likewise, flares of systemic autoimmune disease, and viral and bacterial infections also elicit increased amounts of PB in the blood [1086-1090] including infections with SARS-CoV-2 [1091-1093]. These PB expansions comprise Ag-specific cells producing Abs with affinities permitting their detection by staining with fluorescently labeled Ag such as a vaccine or vaccine components, either before or after fixation and permeabilization with formaldehyde and saponin buffer.

The expression of cell-surface BCR is downregulated by peripheral blood PB/PC compared to circulating naive or memory B cells. However, in the blood, Ag-specific PB are detectable via cell-surface staining with labeled Ag [935], indicating the maintained expression of cell-surface BCR, including IgG, at the stage of circulating PB. Cytometric separation of Ag-specific cells can be further improved by employing intracellular staining, providing staining reagents access to the cytoplasmic accumulations of Abs. At this differentiation stage, the PB have already started to accumulate Ab in their cytoplasm, to secrete Ab that is detectable by Elispot assays [941], to express the proliferation marker Ki-67 [901, 1084] and to migrate along gradients of the chemokines CXCL12 and/or CCL28 (using CXCR4 and CCR10 respectively), guiding them into their BM or mucosal niches, and, via CXCR3 interactions, towards sites of inflammation.

Besides primary and secondary lymphoid tissues including mucosa-associated lymphoid tissues, PB/PC can also be found at other sites under pathological conditions, such as inflammation in the brain and meninges [1094], cerebrospinal fluid [1095], kidney [901, 942], synovium [1096,1097] and synovial fluid [1098], or in the form of disseminated plasma cell tumors.

In this chapter, we provide guidance for cytometric detection of human PB and PC from the BM and the blood, and exemplify the detection of Ag-specific PB using tetramers and fluorescently labeled Ags. We point out potential pitfalls and solutions.

#### 6.3 Step-by-step sample preparation

Flow cytometric assessment of PB/PC is commonly performed from single-cell suspensions obtained by either red blood cell lysis of whole blood, density gradient centrifugation to obtain mononuclear cells, or tissue cell suspensions obtained by protocols tailored to individual tissue types. Since collagenase treatment has been shown to liberate a distinct type of PC from tonsillar tissue compared to mechanical processing alone [1099], digestion protocols can be considered to retrieve PC or certain fractions of PC. It should be noted that enzymes used for this purpose may impact on the detectability of cell-surface receptors, including the molecules used to detect and characterize human PB/PC. PB/PC tend to die rapidly during longer preparation protocols and when cultured in the absence of survival promoting cytokines. Keeping cells cool and working quickly is key. Thus, protocols should be kept short to avoid excessive death of PB/PC after preparation, and we advise to perform dead cell exclusion by DAPI, PI, or amine-reactive, “fixable” dyes. Note that PB/PC might show increased background signal in the channel used for dead cell detection, and dead cell exclusion gates defined based on, e.g., total lymphocytes could lead to exclusion of live PB/PC.

When analyzing rare fractions of PB/PC such as Ag-specific cells, PB/PC may be pre-enriched for flow cytometry analyses by magnetic cell sorting, e. g. by depleting large, unwanted sample fractions such as granulocytes, T cells, and monocytes, or by enrichment of CD138^+^ cells from BM cell suspensions. One should carefully choose depletion markers and exclusion channel markers as mature PC subsets (and especially malignant PC) can express markers commonly associated with T cells (CD28), monocytes (CCR2), or NK cells (CD56).

Cryopreservation using standard procedures and media such as FCS/DMSO may impact on the representation of PB/PC in cryopreserved single-cell suspensions. Usually, after freezing and thawing, frequencies of PB/PC are lower compared to fresh cell preparations, and the detection of some receptors including CD138 has been described to be impaired after cryopreservation [1100].

Since PB/PC are commonly found at low to very low frequency in cell suspensions, the separation of PB/PC (e. g. by gating) from cells that share elements of the PC phenotype is key, and the use of carefully designed exclusion (DUMP) channels is advised. For example, peripheral blood mononuclear cells (PBMC) contain high frequencies of CD27^high^ expressing T cells which may (electronically) contaminate the CD19^dim^/CD27^high^ PB/PC gate unless T cells are excluded from the analysis. Since PB/PC are infrequent in many cell suspensions from primary tissue, care must be taken to acquire suitable numbers of total cells to ensure sufficient representation of PB/PC in the recorded sample desired for statistical analysis.

CD38 is often used to aid the identification of PB/PC, which express exceptionally high levels of CD38 [1101]. This should be considered when establishing the cytometric staining panel and cytometer setup to limit spreading error and secure detection of the PB/PC‘s CD38 levels within the detection range.

Generally, it must be stressed that, to deliver accurate results, PB/PC analyses require thoughtful sample processing and careful experimental and cytometric setup and validation that can vary by project aim (quantification vs further phenotypical characterization), tissue and sample type (digestion, expected abundance, and phenotype of PB/PC).

While many protocols permit reliable detection of PB/PC, we illustrate examples for peripheral blood and BM. Markers for blood PB/PC analysis should include CD19, CD20, CD27, and CD38, and an exclusion channel for dead and unwanted cells (CD3^+^ T cells, CD16^+^ NK cells, and neutrophils, CD14^+^ monocytes). Antibodies detecting HLA-DR, CD138, IgA can be included to define major subsets of PB/PC. Most PB/PC show downregulated CD20 expression, express CD19, and co-express high levels of CD27 and CD38, distinguish-ing them from CD20^+^CD38^high^CD27^−^ transitional B cells and CD20^+^CD27^+^CD38^−/low^ memory B cells. As for BM PB/PC, key markers are CD38 and CD138. Co-staining of HLA-DR permits the detection of PB, and the exclusion channel should be amended with CD10 to exclude stages of developing B cells expressing high levels of CD38.

##### Blood:

**Figure 109.**
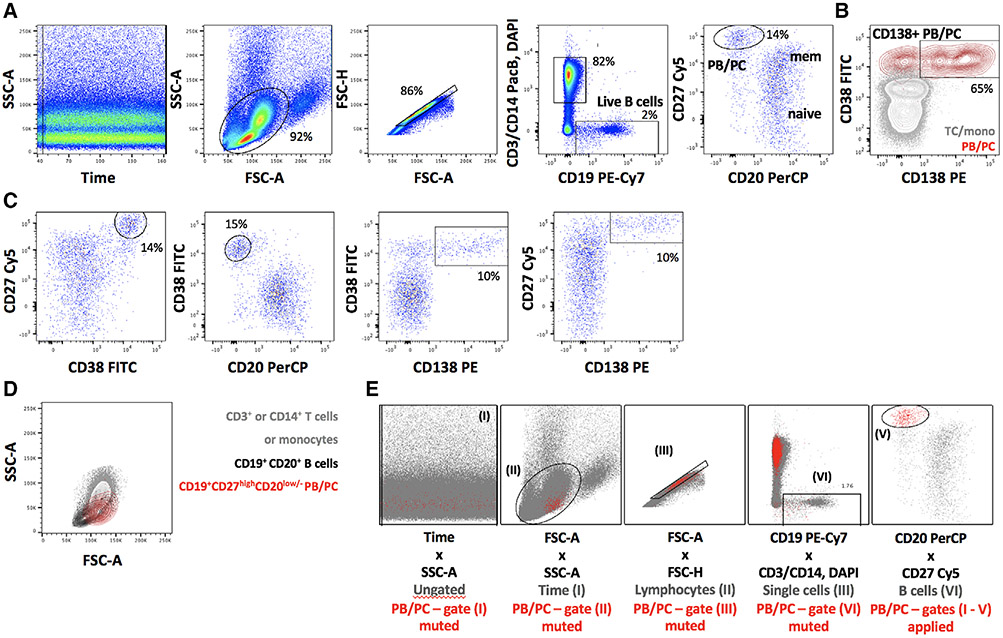
Representative gating strategy and analysis of human peripheral blood PB/PC in a patient with active systemic lupus erythematosus (SLE). Patients with flaring SLE show increased numbers and frequencies of peripheral blood PB/PC [942, 1088]. We thus chose an SLE blood sample for illustration, containing approx. 15% PB/PC among total B cells. Note that in steady-state, i. e. in the absence of in intentional immune activation or symptomatic disease, PB/PC are found at frequencies of commonly approx. 1% among total B cells. (A) Gating strategy. Data were analyzed for changes of scatter or fluorescence parameters over the time of data acquisition, and optionally gated to remove parts of the acquisition that show irregular or discontinuous cytometric patterns. Then, a large light scatter parameter gate was used to identify lymphocytes and monocytes. FSC^high^ cells represent doublets and were excluded. SSC^high^ cells correspond to remaining granulocytes, likely low density granulocytes described before in SLE [1102] that were co-enriched along with PBMC. Next, cell aggregates were removed by gating on cells showing closely correlating area and height values of the FSC signal. Most cell doublets are characterized by a relatively increased FSC-area vs. FSC-height ratio. Live B cells were detected by staining for CD19, and exclusion of T cells, monocytes and dead cells according to CD3, CD14, and DAPI staining. Note that the B cell gate captures CD19^dim^ cells, which can be strongly enriched for PB/PC. CD19 expression itself is subject to regulation in e. g. autoimmune conditions [1103, 1104], so that boundaries of the B cell gate should be carefully validated. CD19^+^CD3^−^CD14^−^DAPI^−^ B cells were then analyzed for CD20 and CD27 expression, revealing CD20^+^ subsets of naive and memory B cells besides PB/PC with a CD27^high^CD20^low/−^ phenotype. In this (SLE) sample, PB/PC are detectable at increased frequencies; normal donors show commonly less than 2% PB/PC among CD19^+^ B cells. (B) PB/PC were then analyzed for expression of CD38 and CD138. Virtually all CD27^high^CD20^low/−^ gated PB/PC expressed high levels of CD38, and two thirds expressed CD138. T cells and monocytes not expressing CD138 and containing very few CD38^high^ cells are shown for comparison. (C) As an alternative to the PB/PC gating shown in (A-B), total PB/PC, or CD138^+^ PB/PC can be gated in various combinations of the markers CD20, CD38, CD27 and CD138, with consistent results. (D) PB/PC show a unique FSC and SSC profile distinct from that of total lymphocytes, B lymphocytes, and monocytes. (E) Backgating confirms the validity of the gating strategy. In particular, it shows that the entire PB/PC subsets was included during light scatter gating, some PB/PC events were excluded as doublets, and that significant amounts of T cells and/or monocytes share the CD27^high^CD20^−/low^ phenotype of PB/PC and may contaminate this population unless careful CD19 gating and DUMP channel exclusion is employed.

Whole blood is diluted with PBS at a 1/1 v/v ratio at room temperature, and PBMC are isolated via density gradient centrifugation over Ficoll. Obtained PBMC are washed twice with PBS / 0.2% BSA, centrifuged (300 × *g*, 4 °C, 10 min), and stained at 4 °C for 15 min in 1.5 mL Eppendorf reaction tubes with a cocktail of monoclonal Abs listed in Table 83. Afterward, cells are washed with PBS / 0.2 % BSA and centrifuged (300 × *g*, 4°C, 10 min). For dead cell labeling DAPI is added prior to acquisition of the sample on a BD CANTO II instrument. Data of at least 300.000, ideally about 1 × 10^6^ PBMC should be collected in total. A representative gating is shown in Figure 109 (with refs. [1102, 1104]. The PBMC isolation protocol can be modified, e. g. to facilitate downstream functional assays combined with cytometric PB/PC detection [942]. For this, whole blood is diluted with pre-warmed RPMI 1640 supplemented with 0.5 % BSA (RPMI/BSA) at a 1/1 v/v ratio prior to density centrifugation, and isolated cells are washed with pre-warmed RPMI/BSA at room temperature (300 × *g*, RT, 10 min).

For the detection of Ag-specific (here, SARS-CoV-2 receptor binding domain (RBD)-specific) PB/PC in the blood, live PBMC are incubated for 30 min at 4°C with a cocktail of monoclonal Abs, containing 50 % (v/v) Brilliant Violet Staining Buffer together with two RBD-streptavidin (SA) tetramers in PBS/0.2 % BSA (Table 84). Prior to this, RBD-tetramers are generated by incubating biotinylated RBD protein with SA-APC or SA-PE/Dazzle 594 at a molar ratio of 1:4 for at least 1 h at 4°C.

**Figure 110.**
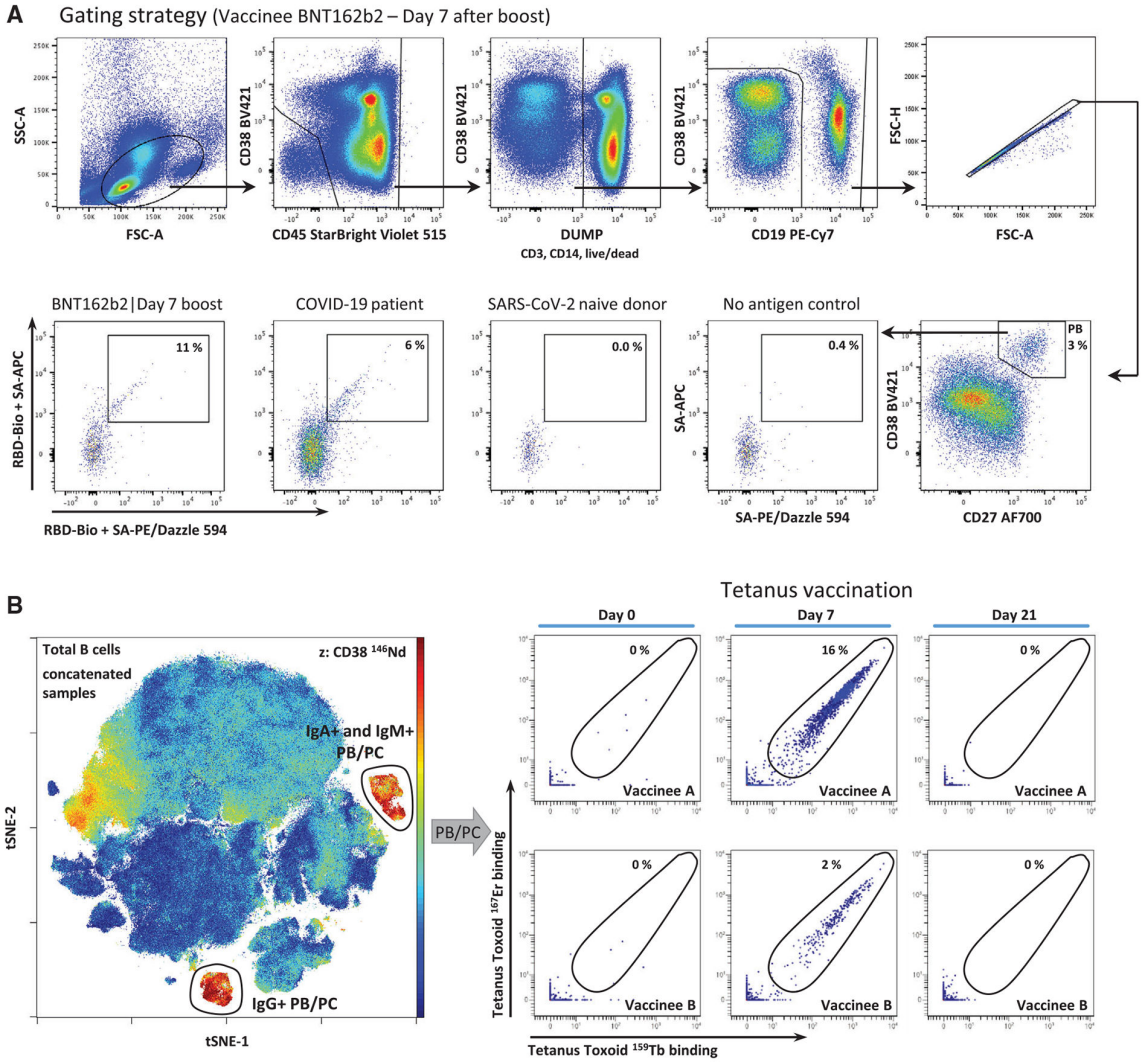
Detection of human Ag-specific PB/PC in peripheral blood by flow and mass cytometry. (A) Gating strategy for the detection of SARS-CoV-2 receptor-binding domain (RBD)-specific PB/PC by flow cytometry. CD38^high^/CD27^high^ PB/PC were identified in PBMC by serial gating comprising a large FSC/SSC gate, followed by the exclusion of residual CD45-negative cells, CD3^+^ T cells, CD14^+^ monocytes and dead cells.Within the remaining cells, CD19^+^ B cells, including CD19^low^/CD38^high^ PB/PC were gated and cell aggregates/doublets were removed by gating in a FSC-A vs. FSC-H plot. Finally PB/PC were gated according to their high CD38 and CD27 expression. RBD-specific PB/PC were detected by a co-staining with two RBD tetramers (APC and PE/Dazzle594 signals). RBD-specific PB were detectable in a blood sample 7 days after booster immunization with BNT162b2, and in an acutely infected COVID-19 patient. No or background amounts of such cells could be detected in PBMC of a SARS-CoV-2 naive and unvaccinated donor, or in a control staining omitting RBD-protein incubation (SA only) in a blood sample 7 days after booster immunization with BNT162b2, confirming the specificity of the staining. (B) Detection of tetanus-specific PB/PC after secondary tetanus re-vaccination by mass cytometry. PB/PC were identified within total B cells after opt-SNE dimension reduction. Two CD38^high^ expressing PB/PC populations were gated corresponding to IgA^+^ or IgM^+^ PB/PC and IgG^+^ PB/PC. Identification of tetanus toxoid (TT) - specific PB/PC was facilitated by a co-staining with ^159^Tb- and ^167^Er-labeled tetanus toxoid. Before and three weeks after immunization few or no TT-specific PB/PC were detected in both individuals, while at day 7 up to 16% of the PB/PC were TT-specific.

At the end of the Ab staining reaction 400 μL PBS are added, supplemented with 1 / 5,000 (v/v) with amine-reactive efluor780 and incubated for 5 min at RT for dead cell detection and exclusion. Finally, cells are washed with PBS / 0.2 % BSA, centrifuged (300 × *g*, 4 °C, 10 min) and acquired on a MACSQuant 16. A representative gating and examples of the detection of RBD-specific PB/PC are shown in Figure 110A. Figure 110B exemplifies the detection of tetanus-specific PB using lanthanide-labeled tetanus toxoid after fixation and permeabilization of B cells with formaldehyde and saponin buffer by mass cytometry.

##### Bone marrow:

**Figure 111.**
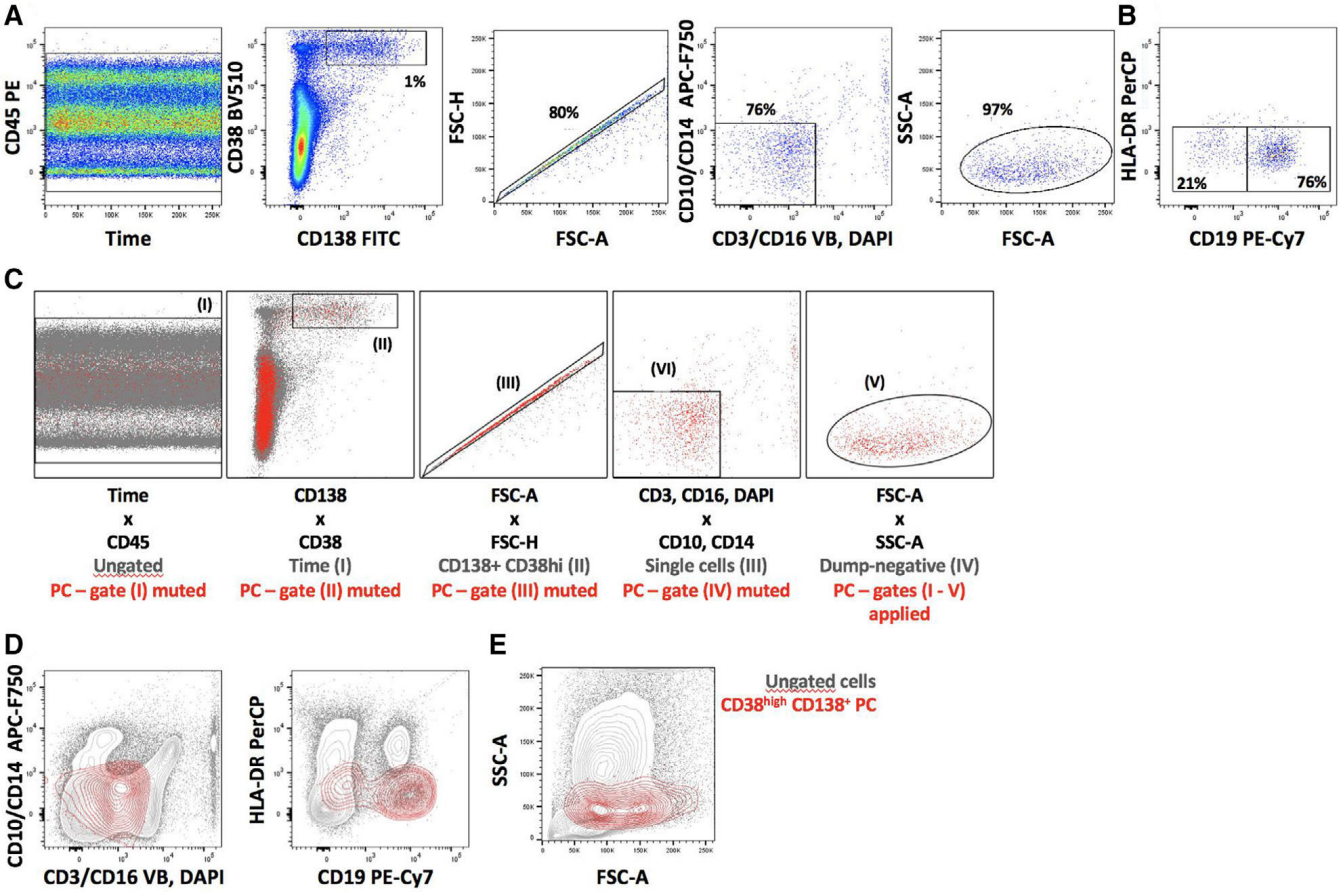
Representative gating strategy and analysis of human bone marrow PC. (A) Analytical gating strategy. Time/CD45 visualization confirms the stability of the cytometric measurement over time. Time frames showing discontinuous data were excluded if applicable. As PC exhibit particular light scatter and background fluorescence properties, the CD138^+^CD38^high^ PC population was gated first, followed by cell aggregate exclusion and gating on CD3^−^, CD16^−^, CD10^−^, CD14^−^ and DAPI^−^ cells for exclusion of dead cells and potentially contaminating cell types. Then, the FSC-A/SSC-A plot reveals that PC show a broader light scatter signal distribution compared to other lymphocytes due to their increased size and ellipsoidal shape. Should the FSC-A/SSC-A plot reveal remaining FSC^low^ and/or SSC^low^ cell debris or electronic artifacts, these should be excluded by gating at this step. (B) Human BM PC consistently display distinct populations with either high or low to no expression of CD19 [901, 1105]. The absence of HLA-DR expression confirms at large the absence of PB [941, 1084], and remaining HLA-DR^+^ PB are excluded. (C) Backgating analyses of the procedure shown in (A). (D) Comparison of Ab staining and light scatter properties of total CD138^+^CD38^+^ BM PC vs total BM mononuclear cells. PC exhibit increased background fluorescence signals compared to other cells (possibly integrating cell size effects, autofluorescence, and non-specific binding of labeled Abs) stressing that gating should be adjusted at the level of PC rather than at global levels. Consistent with their increased size, non-spherical shape, and high organelle content, BM PC show a FSC / SSC pattern distinct from that of other BM cells.

Trabecular parts from human femoral heads containing hematopoietic bone marrow are mechanically disintegrated using a bone mill (Ustomed) and immediately rinsed with ice-cooled PBS/0.2% BSA/5 mM EDTA buffer. The cell suspension is separated from the solid bone fragments by filtering through a 70 μM cell strainer, and centrifuged at 300 × *g* for 10 min at 4 °C. The cell pellet is then resuspended in PBS and mononuclear cells are isolated via density gradient over Ficoll. Alternatively, RBC can be removed by a 15-minute incubation with Qiagen EL buffer at 4 °C. Iliac crest BM aspirates can be used as an alternative source of marrow cells. Obtained cells are washed with ice-cooled PBS / 0.2 % BSA / 5mM EDTA, centrifuged (300 × *g*, 4 °C, 10 min), and stained at 4 °C for 15 min with a cocktail of Abs (Table 85). Cells are then washed with PBS / 0.2% BSA / 5mM EDTA and centrifuged (300 × *g*, 4°C, 10 min), and stained with DAPI for exclusion of dead cells prior to acquisition of the sample on e.g., a MACSQuant Analyzer, Data of at least 200.000 cells should be acquired. A gating strategy is indicated in Figure 111.

#### 6.4 Materials

- PBS (prepared in house)
- BSA (PAN Biotech GmbH)
- 0.5 M EDTA, pH 8.0 (Invitrogen)
- Ficoll-Paque Plus (GE Healthcare)
- 70 μM nylon cell strainer (Falcon)
- DAPI (Sigma Aldrich)

#### 6.5 Pitfalls and top tricks - Detection of PB and PC in the data

In blood, PB and PC express the unique phenotype CD19^+^CD27^high^CD38^high^ and show low or no expression of CD20 [900, 1084, 1105]. CD138, often referred to as a PC marker, is expressed to variable extents in blood PB/PC [1105, 1106] (Figure 109). Besides CD38^high^ PB/PC, a minor CD38^low^ subset has been defined in tonsils [1107], and CD27-negative differentiation stages have been described in *in vitro* studies [1108] and *ex vivo* [1109]. Blood PB/PC can show downregulated, yet still clearly detectable levels of the B cell marker CD19, which is however subject to regulation under pathophysiological conditions [1103]. Few if any PB/PC lack the expression of CD19 in the blood [901, 1110].

During steady-state, PB/PC make up about 1% of peripheral blood B cells. One week after immunization, Ag-specific PB circulating in blood express high levels of HLA-DR, distinguishing them from HLA-DR^low^ cells sharing the typical CD19^+^CD27^high^ phenotype, but being non-migratory and non-proliferating, thus resembling BM PC [1084]. Taken together, blood PB and PC can be well distinguished from other B cells and PBMC according to their unique cell-surface marker expression profile. However, as all mentioned markers alone are also expressed by other cell types or B cell differentiation stages, multiple markers need to be co-stained to obtain a PB/PC population that i) covers most of the PB/PC present in the sample under normal conditions and thus delivers a plausible representation of circulating PB/PC ii) is sufficiently pure, together permitting their reliable quantification and phenotypical characterization.

**Figure 112.**
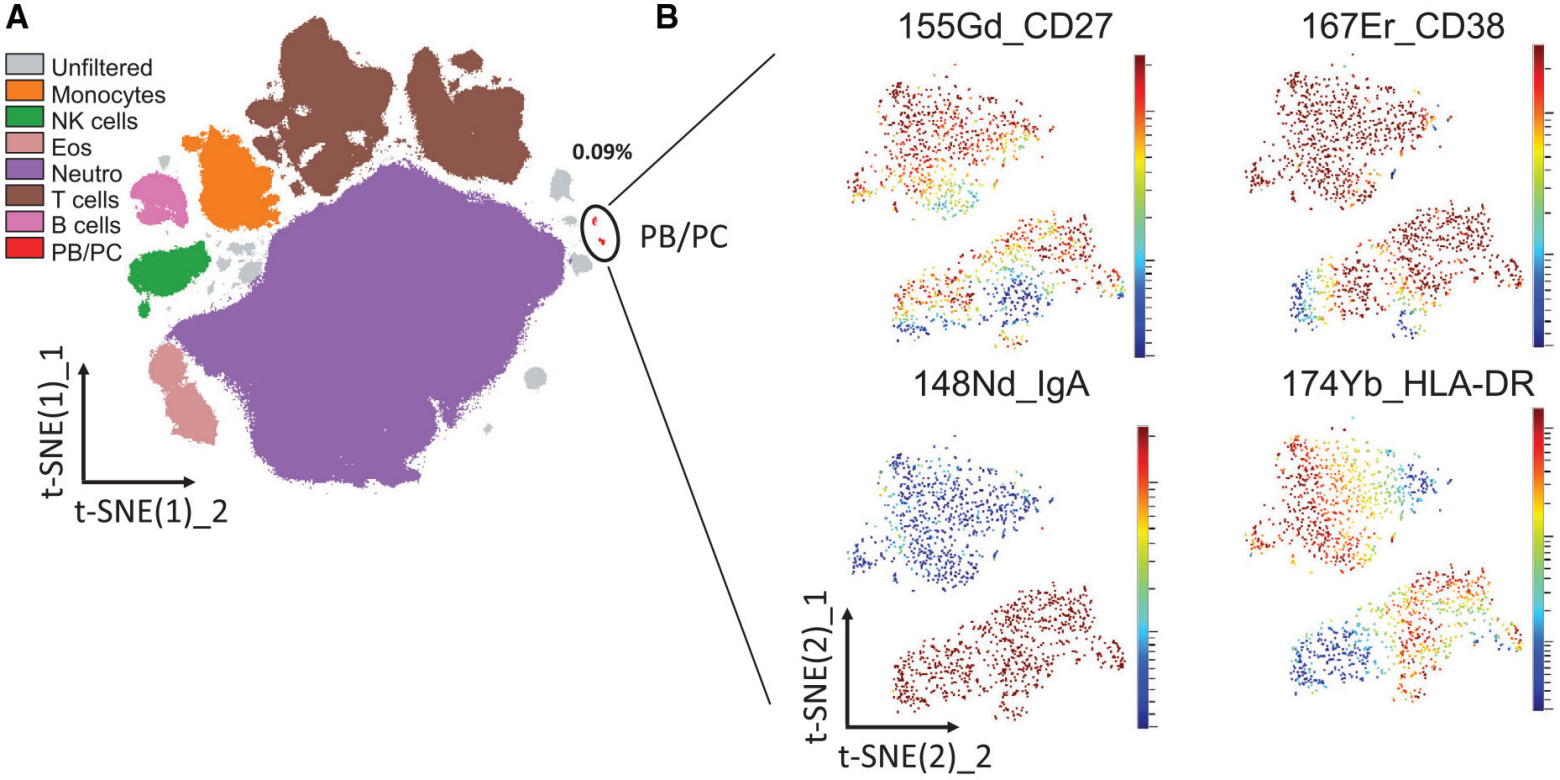
Detection of human peripheral blood PB/PC in a high-dimensional mass cytometry dataset of peripheral blood leukocytes. Data represent leukocytes of a pool of ten cryopreserved whole blood samples. Samples were thawed and RBC were lysed. Leukocytes underwent barcoding, were pooled and stained with cocktail of isotope-conjugated Abs including the markers depicted, and acquired on a Helios mass cytometer (Fluidigm). Detailed information is provided in the publication of the full dataset [1101]. After curation, data of up to 200.000 cells from each sample were subjected to opt-SNE (plotted: 1.937 x 10^6^ cells). (A) Major cell populations and CD38^high^ PB/PC were gated according to their characteristic marker expression profile in the tSNE dimensions and mapped to the t-SNE plot. Note that PB/PC form distinct and condensed subpopulations. (B) Expression of major leukocyte lineage markers and CD38. (C) Gated PB/PC (1697 cells) were subjected to a second opt-SNE run, revealing subsets of IgA^+^ and IgA^−^ PB/PC, and differential expression of HLA-DR.

Since PB/PC are often found at low to very low frequencies, their identification by manual gating can be difficult. With the emerging use of high-dimensional cytometric technologies such as spectral flow cytometry, mass cytometry, or single-cell sequencing combined with oligo-tagged Abs (CITE-seq or AbSeq) [22], more than 10 parameters are increasingly available to detect and characterize PB/PC. Antibody panels used in immune profiling studies usually comprise Abs that are suitable to detect PB/PC such as combinations of CD3, CD14, CD19, CD20, CD27, CD38. Consistently, PB/PC can be readily identified in high-dimensional data, and owing to their distinct phenotype, are often detected as one or multiple cell clusters with distinct locations and condensed appearance in t-SNE (Figure 112, 110B) or UMAP representations, or automatically grouped as such by e. g. FlowSOM clustering [69, 1111]. While these features depend on the particular Ab panel, dimension reduction by opt-SNE [1112] or UMAP [1113] can generally help to identify rare PB/PC in complex data sets.

PC in deposit tissue such as the bone marrow (BM) express intermediate to high levels of CD138, usually very high levels of CD38, a molecule that candidates as a therapy target for depletion of malignant PC in patients with MM and Ab mediated autoimmunity [1101, 1114, 1115], lack CD20 expression, and show low or no expression of HLA-DR. Different from blood PB/PC, CD19 is clearly differentially expressed among mature BM and mucosal lamina propria, and CD19^−^ PC shown consistent features of PC that have reached an exceptionally mature state [901, 1078, 1116]. The frequency of PC in BM cell suspensions is usually greater than in the blood, amounting to 0.5-2% in mononuclear cells after density gradient enrichment, or, to <0.5% of nucleated cells in total BM aspirates [1117, 1118]. Donor age may influence the abundance of blood PB/PC and BM PC [1106, 1119]. A representative analysis of human BM PC is shown in Figure 111.

Apart from cell-surface staining, PB and PC can be detected by staining intracellular Ig (icIg) [1084], including Ag-specific Ig (Figure 110B). Consistent with large-scale Ab production by PB and PC, they accumulate large amounts of it in their cytoplasm, and intracellular flow cytometric staining yields high signal intensities that are suitable to distinguish icIg^high^ PB/PC from Ig^+^ B cells, which do not express extraordinarily high levels of icIg and to which anti-Ig Abs bind mainly on their cell-surface Ig (BCR). Fixation with 1.5% formaldehyde solution and mild permeabilization with 0.1-0.5% saponin solution is sufficient to permit detection of icIg in PB/PC.

The above-mentioned cell-surface markers, IgD, and intracellular (ic)IgM, icIgA, and icIgG, were combined in an optimized multicolor panel (OMIP) for the detection of PB/PC [1120].

Furthermore, affinity matrix technology has been developed to cytometrically capture PB and PC according to their ability to secrete Ab, thus providing access to live and functional PB/PC [732, 1107]. Antibodies capturing the Ig of interest are immobilized on the cell surface, and the cell suspension is short-term cultured to permit Ab secretion by PB/PC. The secreted Ab is bound by the capture Ab and detected by a second, fluorochrome-labeled anti-Ig Ab, which specifically stains the cells that have secreted Ig during the culture phase.

It is noteworthy that activated B cells undergoing PC differentiation gradually downregulate expression of the membrane BCR, and start to secrete the soluble form of Ab. While cell-surface BCR is not detectable anymore in IgG^+^ PC, IgM^+^ and IgA^+^ PB/PC in the blood and PC in deposit tissues maintain expression of cell-surface Ig [902,1121,1122]. By contrast, surface IgG is expressed by PB in the blood after recent vaccination (as evidenced by specific cell-surface binding of fluorescently labeled tetramers, Figure 110A, or Ag) [935], but not in the BM. In addition, rare IgD^+^PB/PC might be detectable in blood and tissues [1123].

Besides Ig of different classes and subclasses, PB and PC express receptors and transcription factors that are implicated in their survival, maturity, and homing, such as, the cytokine receptors IL-6R (CD126), BCMA, and TACI, selectins, integrins, and chemokine receptors such as CD62L, α4β1 and α4β7 integrins, CXCR4, CXCR3, CCR9 and CCR10, transcription factors BLIMP-1, IRF4, and the anti-apoptotic protein Bcl-2 [901, 941, 1076, 1105, 1116, 1124, 1125]. Notably, highly mature PC lose expression of PAX5, leading to the expression of a number of receptors that are typically absent from cells of the B lineage such as CD56, CD28, and CCR2 [901, 1126].

PB and PC exhibit a unique morphology reflecting their role as protein factories. They show an enlarged cytoplasm with expanded Golgi apparatus and endoplasmatic reticulum content, and an eccentrically located nucleus. Coherently, PB/PC show increased FSC/SSC light scatter signals and a broader distribution compared to small lymphocytes (Figures 109, 111). This entails an important caveat when analyzing PB/PC in routine immune profiling studies in which gating strategies start off from a small lymphocyte gate. Any gating performed “upstream” of the PB/PC gate should be carefully checked for unwanted selection against PB/PC fractions. The increased cell size may also lead to increased fluorescent background signal of PB/PC compared to smaller lymphocytes (Figures 109, 111), thus, control staining (such as isotope controls if helpful, or FMO controls) should always be evaluated on the same PB/PC fraction that is subject to analysis.

#### 6.6 Clinical relevance statement

PB and PC are of great interest to medical and biological research in various regards. As mediators of specific serological immunity and memory they play a role in the establishment of specific immunity after infection and vaccination. Infections, including those with SARS-CoV-2, induce PB detectable in peripheral blood, as do vaccines that are tailored to induce long-lasting and specific Ab titers [901, 1090, 1127-1130]. Corresponding mature PC are then found in the BM, however the organization and long-term dynamics in the mature PC population is not well understood [901, 1131, 1132]. On the other hand, PC are the source of pathogenic Abs in autoimmune diseases and humoral transplant rejection, and constitute a potential therapeutic target in these conditions [1115, 1133, 1134]. Furthermore, the abundance of PB and PC in the peripheral blood serves as a biomarker for acute B cell responses and flaring disease in SLE [1087, 1088]. The diagnosis, treatment, monitoring and research in lymphoid tumors recapitulating PC features and biology, such as Multiple Myeloma, Monoclonal gammopathy of undetermined significance (MGUS), reactive plasmacytoma, or Waldenstrom‘s disease, are commonly associated with PC analyses. Furthermore, PC are investigated in a number of interrelated biological contexts such as apoptosis and survival mechanism(s), large-scale protein production and balancing the consequential cellular stress, transcriptional reprogramming, cell adhesion and homing.

#### 6.7 Summary of the phenotypes

This is detailed in Table 86.

### 7 Human plasma cells in multiple myeloma

#### 7.1 Overview

Multiple myeloma is defined by the accumulation of monoclonal plasma cells in the bone marrow and usually preceded by non-malignant monoclonal gammopathy of undetermined significance. Flow cytometry can accurately identify multiple myeloma cells, associated immune phenotypes, and confirm clonal expansion by detection of immunoglobulin light chain restriction. The technology can critically contribute to initial diagnostics, definition of disease heterogeneity, risk stratification, selection of targeted therapeutics, decisions in clinical trials, and detection of minimal residual disease among others.

#### 7.2 Introduction

Plasma cells are terminally differentiated B cells and the major source of circulating soluble Abs. Plasma cell development involves affinity maturation of immunoglobulin genes leading to proliferation and selection of B lineage clones with optimized Ag specificity: Upon stimulation, B cells can proliferate and increase in size, a process referred to as becoming a B cell blast. B cell blasts that secrete Ab are termed plasmablasts. Plasma cells are plasmablasts without proliferation [1135] and circulate in the peripheral blood of healthy individuals at very low frequencies (< 0.1 % of peripheral blood mononuclear cells). Plasma cell differentiation within germinal centers requires high affinity Ag recognition of germinal center B cells and support by T follicular helper cells [826, 1136, 1137]. However, plasma cell differentiation does not strictly require high-affinity Ag recognition and germinal center- and T cell-independent maturation results in plasma cells that show less somatic hypermutations within immunoglobulin genes and secrete lower affinity Abs [826, 901, 1136, 1138-1143].

More than 90 % of plasma cells are long-lived plasma cells, which are assumed to arise from germinal centers. Germinal center-independent maturation can result in short- and long-lived plasma cell populations [901, 1138, 1139, 1143].

Multiple myeloma is defined by the accumulation of monoclonal plasma cells in the bone marrow. In contrast to plasma cells from healthy individuals, in multiple myeloma, (epi-)genetic aberrations are assumed to restore proliferative capacity in variable proportions of plasma cells, enabling malignant clonal expansion [1144]. Substantial numbers of somatic hypermutations within immunoglobulin genes and completed immunoglobulin class switch recombination suggest that malignant transformation of multiple myeloma plasma cells occurs at the (post) germinal center stage of B cell development [1145-1147]. Consequently, immunoglobulin variable region and junction nucleotide sequences can act as unique molecular barcodes for disease tracking at the single cell level [1148]. As healthy plasma cells are considered polyclonal, immunoglobulin light chain expression restricted to either kappa or lambda light chains is supportive of clonal plasma cell expansion [1149].

Multiple myeloma uniquely programs its microenvironment to support tumor growth [1150], protect from T cell responses [1151] and chemotherapeutics [1152-1154]. Microenvironmental features in combination with (epi-)genetic aberrations [1155-1159] result in intra- and interclonal diversity of the malignant plasma cells including their expression of aberrant (surface) molecules.

In immunological studies and clinical diagnostics, plasma cells and multiple myeloma cells can be reliably identified by flow cytometry. This section provides a basic flow cytometry panel and technical advice for the reliable identification of plasma cells and multiple myeloma cells in human bone marrow. The experimental setup can serve as a possible foundation for individual design of detailed immunological studies of the plasma cell compartment.

#### 7.3 Step-by-step sample preparation

1. Collect bone marrow samples, use EDTA as *in vitro* anticoagulant (1.2-2.0 mg EDTA/ml bone marrow sample).
2. Filter the bone marrow sample through cell strainer with 100 μM pore size (Falcon).
3. Pipette 100 μL of bone marrow blood into a FACS tube (BD 352057 Falcon Polystyrene Tube; BD Biosciences).
4. Add 2 ml red blood lysing solution and incubate for 10 min at room temperature.
5. Wash three times: add 2 mL wash medium, re-suspend, centrifuge for 3 min at 420 × *g*, and aspirate supernatant.
6. Vortex to fully re-suspend the cell pellet in 100 μL sheath fluid.
7. Add monoclonal Abs for surface staining.
  In order to ensure optimal staining resolution, the Abs were titrated prior to use and therefore differ from manufacturers recommendation. One test relates to 100 μl bone marrow blood, which is commonly assumed as 1 × 10^6^ cells. Consider also a no-stain control.
  5 μL CD38 (fluorochrome PE, clone HB-7, supplier BD Biosciences, #345806, stock concentration 12.5 μg/mL, recommended volume per test: 20 (μ.l),
  5 μL CD56 (fluorochrome FITC, clone NCAM16.2, supplier BD Biosciences, #345811, stock concentration 6 μg/mL, recommended volume per test: 20 (μl).
  1.25 μL CD138 (fluorochrome V500C, clone MI15, supplier BD Biosciences, #650659, concentration 100 μg/mL, recommended volume per test: 5 μl),
  2.5 μL CD19 (fluorochrome PECy7, clone J3-119, supplier Beckman Coulter, concentration not provided, recommended volume per test: 10 μl),
  1.25 μL CD45 (fluorochrome V450, clone 2D1, supplier BD Biosciences, #642275, concentration 100 μg/mL, recommended volume per test to stain 100 μl blood: 5 μl).
8. Incubate for 15 min in the dark at room temperature.
9. Add 100 μL of Reagent A (FIX&PERM Cell Fixation and Permeabilization Kit, Nordic-MUbio) and incubate for 15 min in the dark at room temperature.
10. Wash once: add 2 mL wash medium, re-suspend, centrifuge for 3 minutes at 420 × *g*, aspirate supernatant.
11. Add 100 μL of Reagent B (FIX&PERM Cell Fixation and Permeabilization Kit, Nordic-MUbio).
12. Add monoclonal Abs for intracellular staining:
  2.5 μl kappa immunoglobulin light chain (fluorochrome APC, clone TB28-2, supplier BD Biosciences, #341108, concentration 50 μg/mL, recommended volume per test: 5 μl)
  2.5 μL lambda immunoglobulin light chain (fluorochrome APC-H7, clone 1-155-2, supplier BD Biosciences, #656648, concentration 100 μg/mL, recommended volume per test: 5 μl).
13. Incubate for 15 min in the dark at room temperature.
14. Wash twice: add 2 mL wash medium, re-suspend, centrifuge for 3 minutes at 420 × *g*, and aspirate supernatant.
15. Resuspend cells in 100 μL sheath fluid for immediate analysis.

#### 7.4 Materials

##### 7.4.1 Media and buffers.

###### Red blood lysing solution:

Lysing Solution 10× Concentrate (BD FACS^™^)

###### Wash medium:

100 ml 10× PBS (Gibco) + 900 ml Aqua dest (Braun)

FIX&PERM Cell Fixation and Permeabilization Kit including Reagent A and B (Nordic-MUbio)

###### Sheat fluid:

BD 342003 Facs Flow (BD Bioscience), alternative 1× PBS.

#### 7.4.2 Monoclonal Antibodies.

##### 7.4.2.1 Surface staining.

CD138 (fluorochrome V500C, clone MI15, supplier BD Biosciences)

CD19 (fluorochrome PECy7, clone HIB19, supplier Beckman Coulter)

CD45 (fluorochrome V450, clone 2D1, supplier BD Biosciences)

CD38 (fluorochrome PE, clone HB-7, supplier BD Biosciences)

CD56 (fluorochrome FITC, clone NCAM16.2, supplier BD Biosciences)

###### 7.4.2.2 Intracellular staining.

kappa immunoglobulin light chain (fluorochrome APC, clone TB28-2, supplier BD Biosciences)

lambda immunoglobulin light chain (fluorochrome APC-H7, clone 1-155-2, supplier BD Biosciences)

#### 7.5 Flow cytometer

Stained bone marrow samples were acquired on on a BD FAC-SLyric (BD Biosciences).

#### 7.6 Data analysis

**Figure 113.**
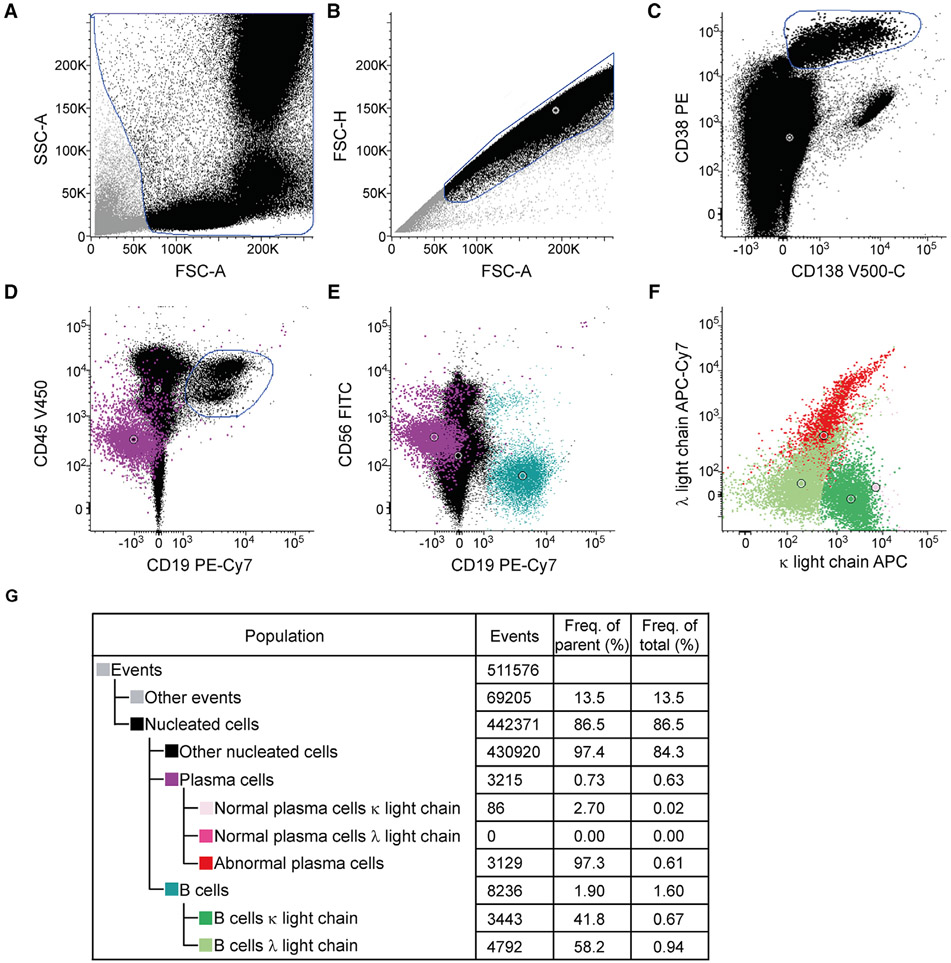
Identification of aberrant plasma cells in human multiple myeloma bone marrow. (A-C) Plasma cells are defined as the CD38- and CD138-positive population (blue gate shown in Ccorresponds to the purple plasma cell population in D-E) among leukocytes (black) after exclusion of debris (A) and doublets (B). No live/dead staining is performed. Aberrant plasma cells (purple) in this sample are partially CD56-positive, homogeneously negative for CD19 and CD45-low (D-E). Moreover, aberrant plasma cells do show immunoglobulin light chain restriction (in this case lambda, indicated in red, F), which ultimately characterizes them as abnormal plasma cells. As an internal comparison, B cells (gate shown in D) present characteristic CD19 and immunoglobulin kappa and lambda light chain expression. The immunoglobulin light chain expression should be evaluated along a diagonal. B cells typically show lower expression levels of immunoglobulin light chains compared to plasma cells. (F). The hierarchy of defined populations as well as absolute and relative numbers of events are shown in (G). Open circles indicate population centers. Gating was performed with Infinicyt^™^ Flow Cytometry Software.

**Figure 114.**
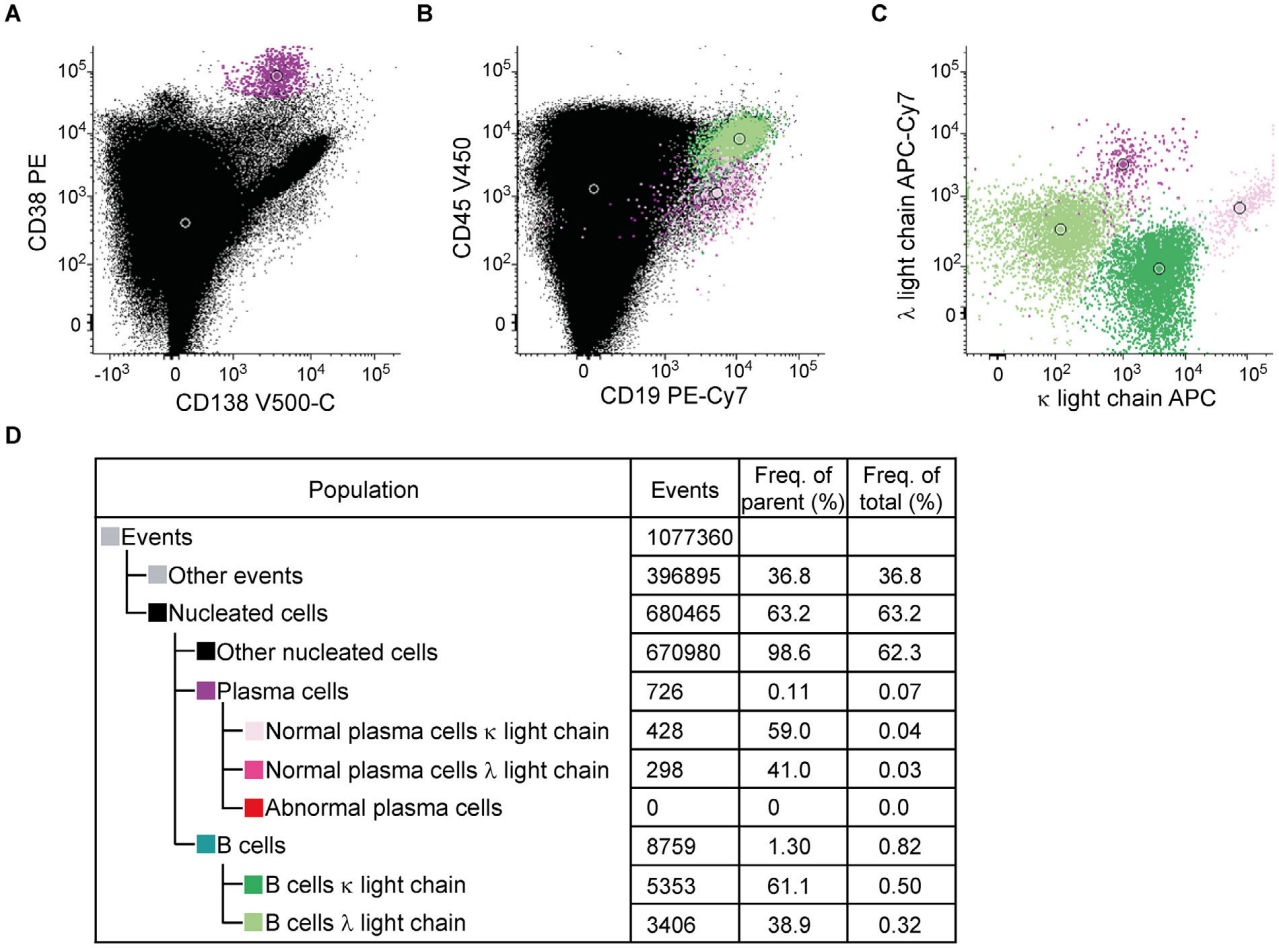
Identification of non-malignant plasma cells in human bone marrow. An example of a normal plasma cell population is shown. The gating strategy for identification of single nucleated cells, plasma cells, and B cells as well as color coding are identical to Figure 113. Plasma cells are defined as the CD38− and CD138-positive population (purple, A) among leukocytes (black). Normal plasma cells usually express CD19 and CD45 (B) in combination with heterogeneous kappa/lambda light chain expression (C). The hierarchy of defined populations as well as absolute and relative numbers of events are shown in (D). Open circles indicate population centers. Gating was performed with Infinicyt^™^ Flow Cytometry Software.

Flow cytometry can identify plasma and multiple myeloma cells by forward/side scatter characteristics in combination with uniquely high expression of CD38 and CD138 (Figure 113A-C) [1160-1162]. While CD45 and heterogeneous CD19 expression indicate different maturation states of normal plasma cells [1161, 1163], the identification of malignant plasma cells can be complicated by considerable variation in marker expression between and within individual patients. For example, phenotypes frequently associated with multiple myeloma cells (absence of CD19 and expression of CD56, example in Figure 113D-E) can also be part of non-malignant differentiation [901, 1078, 1164, 1165]. The detection of intracellular immunoglobulin light chain restriction (Figure 113F) can help identifying clonal expansion in most cases [1149] but may be technically challenging (intracellular staining, low target cell numbers, absence of immunoglobulin light chain expression). In comparison to normal plasma cells that do not show immunoglobulin light chain restriction (Figure 114) the immunoglobulin light chain restriction is evident on malignant plasma cells. Of importance, evaluation of immunoglobulin kappa and lambda light chain restriction should be performed along a diagonal line, rather than the frequently used gating quadrant. This reflects the typical fluorescence plot position of cells that express immunoglobulin light chains.

#### 7.7 Pitfalls

##### 7.7.1 Flow cytometry underestimates the number of plasma cells in bone marrow aspirates.

Although, providing key information on plasma cell clonality and aberrant phenotype, flow cytometry consistently underestimates the number of plasma cells in bone marrow samples compared to morphological assessment [1166] This might result from an increased fragility of plasma cells compared to other leukocytes, loss of plasma cells during sample preparation, hemodilution and a discrepancy in content of plasma cells in different samples (first versus subsequent pulls during bone marrow aspirate collection). As an accurate plasma cell quantification is crucial for diagnosis of plasma cell disorders, a morphologic assessment of bone marrow smears and/or histopathological evaluation of bone marrow biopsies should be performed. However, providing an immediately available lower limit estimate and differentiating between normal and aberrant plasma cells, flow cytometry is a powerful method in first diagnosis and determination of minimal residual disease.

##### 7.7.2 Monoclonal Abs used in multiple myeloma treatment can interfere with flow cytometric analysis.

As CD38 is frequently expressed on a high percentage of normal and aberrant plasma cells, immunotherapeutical approaches in multiple myeloma target CD38 with monoclonal Abs, such as daratumumab, isatuximab (SAR650984), MOR03087 (MOR202) and Ab79 [1166-1168] Recent studies have shown that anti-CD38 treatment, in particular daratumumab, can interfere with diagnostic plasma cell detection caused by a long-term CD38 saturation leading to an absence of CD38-positive events [1169, 1170] As plasma cells are identified as CD38 and CD138-positive cells, anti-CD38 treatment might lead to false negative results in plasma cell detection. It can be assumed that also further therapeutically used monoclonal Abs directed against plasma cell surface Ags that are crucial for detection of plasma cells (e.g., CD138) may also interfere with flow cytometric analysis. Therefore, bone marrow samples from patients treated with monoclonal Abs should also be evaluated by morphologic techniques as aspirate smears and immuno-histopathology. Moreover, alternative plasma cell-specific Ags, as SLAMF7, or intracellular transcription factors, as BLIMP1 and IRF4, might be used for plasma cell identification in flow cytometry [1165, 1171, 1172]. Furthermore, CD27 and CD81 expression indicates different maturation stages of normal plasma cells and might be helpful in detection of an aberrant phenotype (Table 87; see below) [1161, 1163].

#### 7.8 Top tricks – Focus on measurable residual disease (MRD)

Measurable residual disease (MRD) is defined as a small number of malignant plasma cells that persist after treatment. MRD represents the treatment efficacy, is highly predictive for outcome and is considered as the major cause of relapse in multiple myeloma [1173, 1174]. Multicolor flow cytometry is one of the available MRD detection methods that can reach a sensitivity of up to 1×10^−6^. The simultaneous detection of multiple sets of surface and intracellular markers enables reliable and fast identification of multiple myeloma cells making flow cytometry an indispensable tool in basic research and clinical diagnostics alike. The high throughput characterization of millions of cells in a reasonable amount of time allows minimal residual disease detection with high sensitivity comparable to next generation sequencing [1175]. Similarly to the detection of aberrant plasma cells at first diagnosis the Ag panel for MRD detection includes CD38, CD138, CD45, CD19, kappa and lambda light chains. However, assessment of >1×10^6^ nucleated cells is crucial to reach adequate MRD sensitivity levels. Moreover, high level of standardization with regard to used Abs, sample preparation and measurement and data analysis is crucial. The Multiple Myeloma MRD Kit, a EuroFlow^™^ approach to monitor MRD by flow cytometry, offers a ready-to-use solution for sensitive and accurate MRD detection [1175]. Automatic software tools lead to an automated identification of cell populations and aberrant plasma cells offering high levels of standardization. These approaches are expected to overcome heterogeneity of MRD detection protocols [1176] across different flow cytometry laboratories and provide reliable MRD data particularly within clinical trials.

In the context of bone marrow MRD assessment, hemodilution might represent a limitation, as hemodiluted samples might be falsely considered as MRD negative. Therefore, the assessment of hemodilution in bone marrow samples is warranted. To determine the extent of hemodilution the percentages of B cell precursors, nucleated red blood cells, and mast cells should be evaluated. “Samples with <0.01% bone marrow plasma cells, B cell precursors, nucleated red blood cells, and mast cells can be considered severely hemodiluted and therefore inadequate for MRD assessment” [1177].

#### 7.9 Clinical relevance statement

The gating strategy shown in this section is applicable for analysis of plasma cells in multiple myeloma disease setting/patients, as can be seen in Figure 113 and in healthy individuals, as can be seen in Figure 114. The key conclusion from such analysis is that in the first example (Figure 113) an aberrant plasma cell population (CD45-low, CD19-low, CD56+, lambda light chain restricted) can be identified in a multiple myeloma patient. In the second example (Figure 114) a normal plasma cell population is identified.

#### 7.10 Summary of the phenotypes

This is detailed in Table 87 (with ref. [1178].

### 8 Murine antibody-secreting plasmablasts and plasma cells

#### 8.1 Overview

Plasma cells are terminally differentiated B lineage cells that secrete large amounts of Abs, an essential step in establishing effective adaptive humoral immunity against pathogens and toxic substances. The induction of the plasma cell program begins in peripheral lymphatic organs with the transition of activated B cells to the proliferating and Ab-secreting plasmablast stage. Antibody-secreting cells enter the bloodstream, migrate to effector sites or survival niches, *e.g.,* in the bone marrow, spleen, mucosal lamina propria or sites of inflammation and mature into non-dividing plasma cells. Some of these cells may persist for years in mice and decades in humans and continuously provide protective or, unfortunately sometimes also pathogenic Abs. This chapter will provide an overview of surface markers and detailed protocols to identify proliferating plasmablasts and non-dividing plasma cells in various murine lymphatic tissues by flow cytometry.

#### 8.2 Introduction

After being activated in either a T cell-dependent or T cell-independent manner, B cells proliferate and initiate a transcriptional and post-transcriptional program controlled by, *e.g.,* interferon-regulated factor 4, IRF4 [1179], B lymphocyte induced maturation protein 1, BLIMP1 [1180] or microRNA miR-148a [1181], which results in the generation of plasma cells that have adapted to the challenge of synthesizing and secreting large amounts of Abs. For example, a human myeloma line can secrete between 50 - 340 pg IgG Ab per day [1182]. Antibody-secreting cells are a heterogeneous population, including early proliferating (*i.e.*, plasmablasts) and non-dividing and long-lived cells (*i.e.*, plasma cells).

Maturation of proliferating early Ab-secreting plasmablasts into resting long-lived plasma cells is accompanied by an increased abundance of immunoglobulins (Ig), BLIMP1, CD138 (Syndecan-1), transmembrane activator and CAML interactor (TACI) and B cell maturation Ag (BCMA). At the same time, B cell-specific surface proteins such as CD19, CD20, MHCII and B220 are downregulated [1183]. Combinations of these markers can be used to track the various subsets of Ab-secreting cells. In addition, BLIMP1-reporter mouse lines (*e.g.,* BLIMP1:GFP mouse, [1184]) represent a handy tool to identify Ab-secreting cells by flow cytometry or fluorescence microscopy.

There are, however, several limitations when using the BLIMP1:GFP reporter mouse. Most importantly, the GFP reporter signal alone is not sufficient for a reliable analysis of plasmablasts/plasma cells as BLIMP1 is also produced by other immune cells, e.g., effector T cell subsets in the spleen and other lymphatic and non-lymphatic tissues [1185]. In addition, the knock-in of the GFP reporter cassette into the *Prdm1* gene (encodes BLIMP1) results in an inactive *Prdm1* allele [1184]. Furthermore, in contrast to formaldehyde fixation, the fluorescence of the GFP molecule is abolished by methanol/ethanol-based fixation protocols. Finally, the BLIMP1:GFP reporter mouse might either not be available or be too time-consuming to cross the *Prdm1* reporter allele into other transgenic lines or disease mouse models. Therefore, alternative surface staining protocols to detect Ab-secreting cells on a single-cell basis by flow cytometry have been developed.

As plasma cells produce large amounts of immunoglobulins, surface CD138 staining together with staining of intracellular Ig-kappa and Ig-lambda light chains was considered the gold standard for identifying Ab-secreting cells by flow cytometry for many years ([1186, 1187]). However, this protocol does not allow the sorting of live cells. This can be accomplished by using a combination of a variety of surface markers. CD138 is most used to analyze plasma cells, albeit its expression is not restricted to Ab-secreting cells. Even though IgM and IgA B cell receptors are still detected on the surface of mature plasma cells ([1028, 1063]), the combination of CD138 and B cell receptor surface staining cannot reliably replace an intracellular Ig-kappa/lambda staining as the gold standard because IgG is, in contrast to IgM and IgA, not expressed in mature plasma cells.

To avoid the fixation of cells in flow cytometric analysis, CD138 staining combined with the detection of BLIMP1 reporter expression, *e.g.*, in the BLIMP1:GFP mouse [1184], is frequently used as a reference staining to detect murine plasma cells. To allow the detection of Ab-secreting cells in mice that do not carry a BLIMP1 reporter allele, CD138 staining together with an additional surface marker, *e.g.,* TACI [1188] Sca-1 [1189], CD98 [1190], ENPP1 [1191], Ly6-C [1192] and B220 [1193], have been described in recent years. However, a limitation is that most of these double-stainings do not differentiate between early dividing plasmablasts and late non-dividing plasma cells.

To distinguish dividing plasmablasts from non-dividing plasma cells, analysis of the proliferation marker Ki-67 can be helpful. However, for staining of Ki-67, cells have to be fixed and permeabilized, which is incompatible with cell viability ([901, 1028]). Another frequently used method to analyze Ab-secreting cells in mice is the treatment with the nucleotide analog BrdU or EdU via the drinking water ([1079, 1194]). In combination with additional surface markers such as CD138, this allows to (1) distinguish between BrdU/EdU-positive proliferating plasmablasts or freshly differentiated plasma cells and previously generated BrdU/EdU-negative mature plasma cells, as well as (2) the tracking of BrdU/EdU-positive mature resting plasma cells over time.

#### 8.3 Step-by-step sample preparation

##### Four-color staining protocol to differentiate early plasmablast from late plasma cells

**Figure 115.**
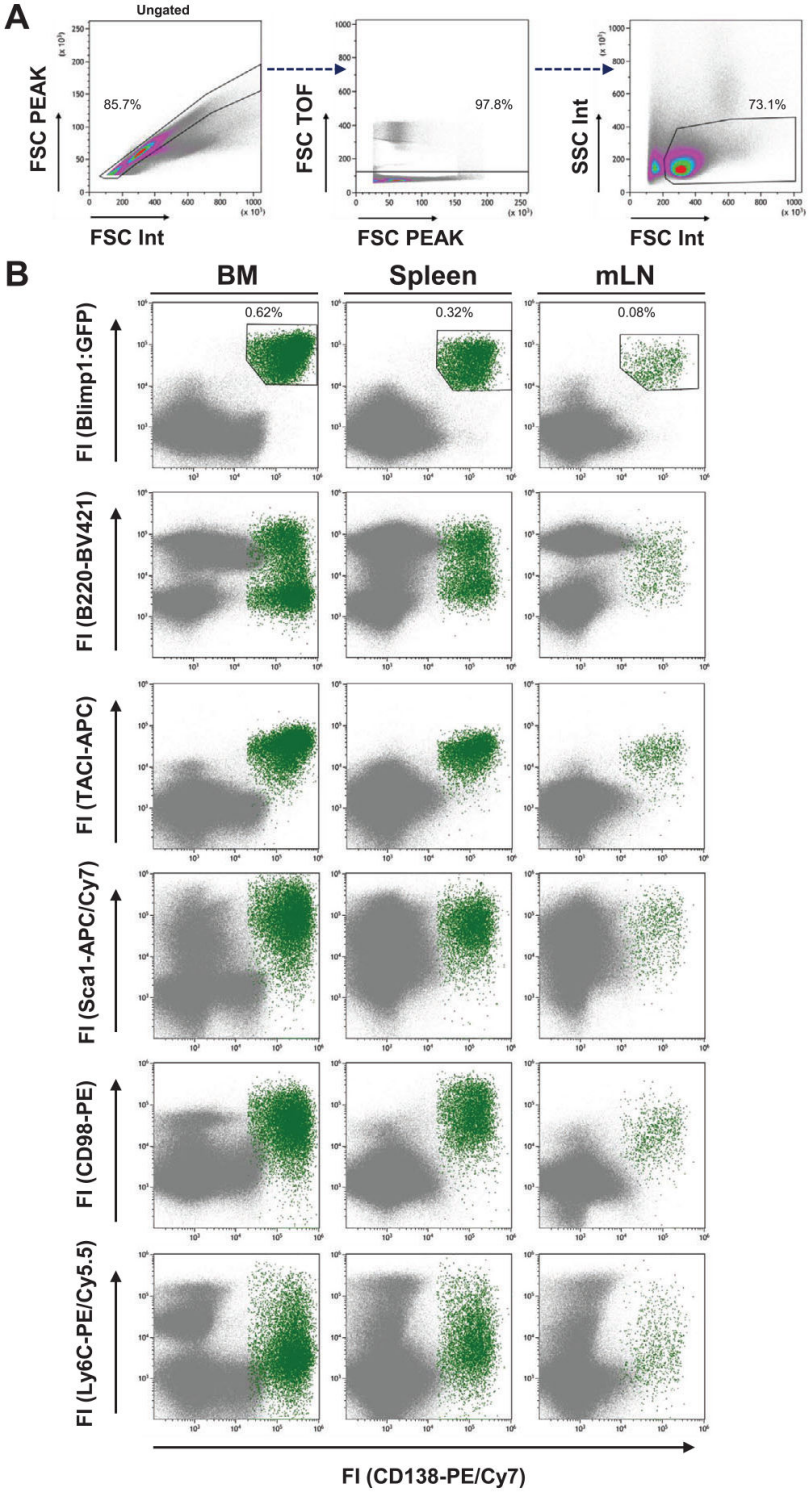
Comparison of common two-color flow cytometric analyses of murine plasma cell populations. (A) Exemplary gating strategy for single extended lymphocytes in the spleen. Viable cells were defined using FSc/SSc characteristics. (B) Single-cell suspensions from bone marrow (BM), spleen and mesenteric lymph nodes (mLN) of BLIMP1:GFP-reporter mice were isolated and stained as described with Abs against CD138 and one additional surface marker indicated on the y-axis. The input gate for all dot plots in (B) was set to the gate in the rightmost panel described in (A). The BLIMP1:GFP^hi^/CD138^hi^ gate was used as the reference gate for the plasmablast/plasma cell populations, and events in this gate are highlighted in green in the following plots.

**Figure 116.**
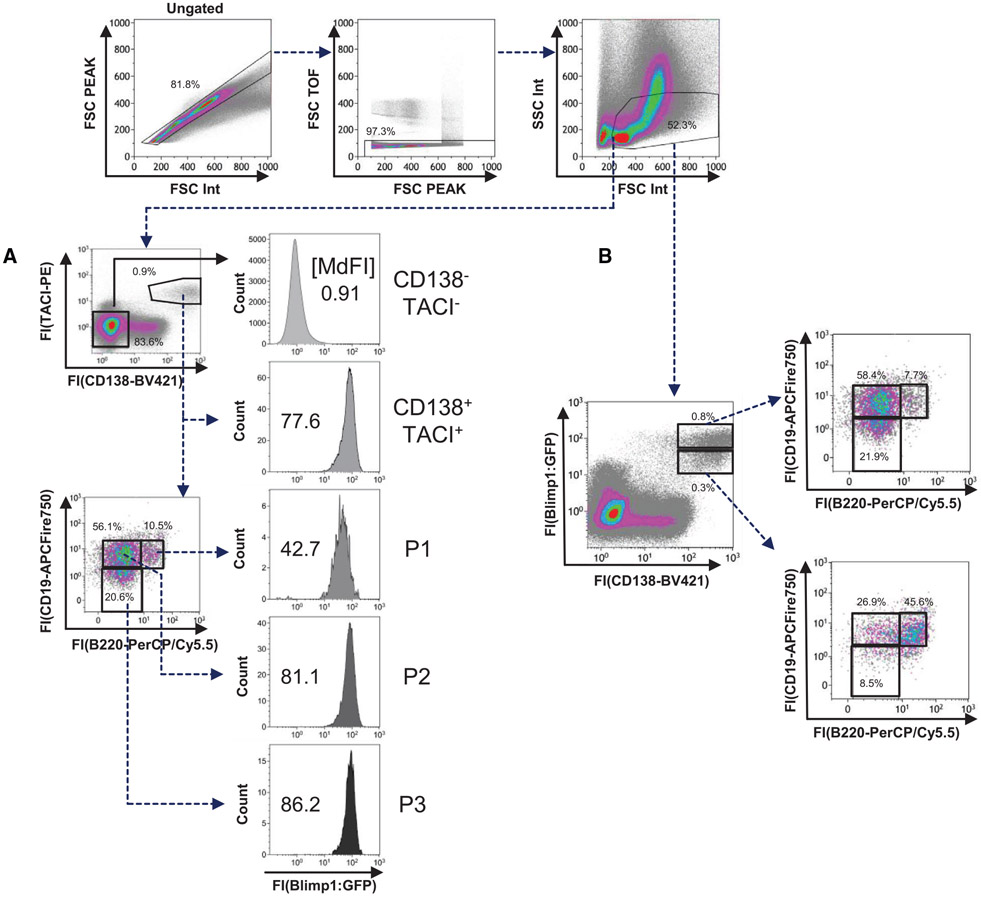
Flow cytometric distinction between murine plasmablasts, early- and late mature plasma cells. Single-cell suspensions from the bone marrow (femur and tibia) of BLIMP1:GFP-reporter mice were analyzed for their surface expression of CD138, TACI, CD19 and B220. Viable cells were defined using FSc/SSc characteristics. (A) CD138^+^/TACI^+^ cells and subpopulations defined on their B220 and CD19 abundance were analyzed for their BLIMP1:GFP-expression (MdFI: Median fluorescence intensity); MdFI values are indicated in the depicted histograms. CD19 and B220 surface expression was used to further subdivide the CD138^+^TACI^+^ population (P1: CD19^+^/B220^+^ (dividing plasmablasts); P2: CD19^+^/B220^low^ (early plasma cells); P3: CD19^low^/B220^low^ (mature plasma cells). CD138^−^/TACI^−^ cells were used as a negative control for BLIMP1:GFP-expression. (B) BLIMP1:GFP^+^/CD138^+^ cells were divided based on their fluorescence intensities in high-expressing population (CD138^high^/BLIMP1:GFP^high^) and low-expressing population (CD138^+^/BLIMP1:GFP^+^). These two subpopulations are further subdivided based on heterogeneous CD19/B220 expression.

Utilizing the BLIMP1:GFP-reporter mouse line, which is frequently used to detect Ab-secreting cells [1184], we tested different surface marker combinations and gating strategies to distinguish plasmablasts from early and late plasma cells with a single staining protocol without the need of BrdU or EdU [1028]. A four color-staining protocol including CD138, TACI, B220 and CD19 has been established (Figure 115). The four color-staining over-comes the limitations of double-staining and is superior in separating proliferating plasmablasts from early and late plasma cell subsets because it (1) does not require intracellular Ki67-staining or a plasma cell reporter-mouse line, (2) excludes CD138-positive B cell progenitors in the bone marrow (Figure 116A), and (3) allows the sorting of viable plasma cell subsets [1028].

###### Bone Marrow

Bones (*e.g.,* femurs, tibiae, humeri, vertebrae) were isolated, cleaned from surrounding tissue, and crushed with mortar and pestle in PBS + 2% FCS or RPMI 1640 medium supplemented with 10% FCS (R10). Residual bone fragments were removed using a 70μm cell strainer or decapped and flushed with 27G cannula and PBS + 2%FCS or R10. The bone marrow suspension was subsequently centrifuged at 300 × *g* for 5 min at 4°C. The pellet was resuspended in RBC-lysis buffer and incubated for 5 min at room temperature. Lysis was stopped by adding PBS + 2% FCS or R10 medium, and the cell suspension was filtered through a 30 μm mesh filter.

###### Spleen

The spleen was isolated, cleaned from surrounding tissue, and gently disrupted in PBS + 2% FCS or R10 through a 70μm cell strainer using the plunger of a 2 ml syringe. The cells were pelleted by centrifugation at 300 × *g* for 5 min at 4°C, resuspended in RBC-Lysis buffer and incubated for 5 min at room temperature. The lysis was stopped by adding PBS + 2% FCS or R10 medium, and the cell suspension was filtered through a 30μm mesh filter.

###### Mesenteric lymph node

Mesenteric lymph nodes (typically about 4) were isolated and cleaned from surrounding fatty tissue. The lymph nodes were then gently disrupted in PBS + 2% FCS or R10 medium through a 70μm cell strainer using the plunger of a 2ml syringe and the cell suspension was filtered through a 30 μm mesh filter.

###### Staining Protocol

Cells from organ suspensions were pelleted by centrifugation at 300 × *g* for 5 min at 4°C, resuspended in PBS + 2% FCS and adjusted to a density of 2x10^7^ cells/ml. 4x10^6^ cells from each tissue were washed in PBS + 2% FCS + 0.05% NaN_3_ and pelleted at 300 × *g* for 5 min at 4°C. To avoid unspecific binding, cell pellets were resuspended in 50 μl of unlabeled anti-CD16/32 (1:100 in PBS + 2% FCS + 0.05% NaN_3_) and blocked for 15 min on ice (or 5 min at room temperature). Cells were washed again in PBS + 2% FCS + 0.05% NaN_3_ and centrifuged at 300 × *g* for 5 min at 4°C. The pellet was then resuspended in 50 μl PBS + 2% FCS + 0.05% NaN_3_ containing the respective fluorochrome-coupled Abs and incubated for 20 min on ice in the dark. After staining, the cells were washed twice with PBS + 2% FCS + 0.05% NaN_3_ and centrifuged at 300 × *g* for 5 min at 4°C. The pellet was resuspended in PBS + 2% FCS + 0.05% NaN_3_ for flow cytometric analysis. Flow cytometric analyses were performed on a Gallios flow cytometer (Beckman Coulter). Data were analyzed using Kaluza flow cytometry analysis software (Beckman Coulter).

#### 8.4 Materials

- Dulbecco’s phosphate-buffered saline (PBS)
- Fetal calf serum (FCS), heat-inactivated (56^0^C, 1 hour)
- Sodium azide (NaN_3_)
- Falcon^®^ 70 μm cell strainer (Becton Dickinson)
- CellTrics^®^ 30μm filter (Sysmex)
- Red blood cell (RBC) lysis buffer (BioLegend, product number 420301)
- Gallios flow cytometer (Beckman Coulter)
- Kaluza flow cytometry analysis software (Beckman Coulter)

The Abs used for flow cytometry are listed in Table 88.

#### 8.5 Data analysis

In Figure 115, we compared the presence or absence of one additional commonly used surface marker on CD138^+^ cells to the CD138^+^/BLIMP1:GFP^+^ reference population in bone marrow, spleen and mesenteric lymph node. CD138 together with a B cell marker, *e.g.,* B220 [1193], is the most commonly used staining protocol to distinguish between early dividing plasmablasts (CD138^+^/B220^+^) and mature CD138^+^/B220^−^ plasma cells (Figure 115B, first row). However, without the addition of a BLIMP1:GFP reporter (Figure 115B, 2^nd^ row), it is challenging to separate bone marrow B220^+^/CD138^+^ plasmablasts from B220^+^ pro-B/pre-B cells with intermediate fluorescence intensities for CD138 ([1004, 1195]). The detection of the survival receptor TACI on CD138^+^ cells prevents these problems because almost all BLIMP1:GFP-positive cells are included within a separated TACI^+^/CD138^+^ population (Figure 115B, compare row 1 with row 3 and [1028]). CD98 and Sca-1 can also be used in conjunction with CD138 staining to detect Ab-secreting cells in bone marrow and spleen. However, these populations are more diffuse, and especially in the lymph node, are interspersed by cells outside of the CD138^+^/BLIMP1:GFP^+^ reference gate (Figure 115B rows 4 and 5). These protocols might be improved using “dump” markers, *e.g.,* F4/80 and CD4/CD8, as suggested by Wilmore et al. [1189]. Despite being described as a plasma cell marker, in our hands, Ly6C is not suitable for the detection of all Ab-secreting cells, as it is not ubiquitously expressed in the BLIMP1^+^/CD138^+^ plasmablast/plasma cell population (compare row 1 with row 6 in Figure 115B). Therefore, the combination of CD138 and TACI staining is a robust protocol to detect a separated plasmablast/plasma cell population in very high concordance with the CD138^+^/BLIMP1:GFP^+^ reference across all analyzed lymphatic organs.

The double staining strategies described in Figure 115 do not discriminate between plasmablasts and plasma cells. Therefore, it is necessary to add additional surface markers. For example, adding the B cell markers CD19 and B220 into the TACI/CD138 staining protocol resulted in three sub-populations. All three subsets (P1 to P3) were BLIMP1:GFP-positive with a stepwise increase in the abundance of BLIMP1:GFP fluorescence from P1 to P3 (Figure 116A), indicating an increase in maturity from the P1 (dividing plasmablasts) to the P2 (early predominantly non-dividing plasma cell) and the P3 (late non-dividing plasma cells) subpopulation. While the B220^+^/CD19^+^ P1 population contains a high frequency of proliferating (Ki-67^+^) cells, most of the cells in the subpopulations P2 and P3 are mature Ki-67-negative resting plasma cells [1028]. In the spleen of non-immunized mice, the P1- and P2- subpopulations are dominant, while in the bone marrow, the CD19^−^/B220^−^ P3 population is most prevalent.

In humans, CD19-negative plasma cell subpopulations have been described [901, 1078]. However, the biological origin and functional differences between the CD19^+^ and CD19^−^ plasma cell subpopulations remain largely unclear [1110].

#### 8.6 Pitfalls and top tricks

A reliable flow cytometric protocol to analyze Ab-secreting plasma cell population in mice should consider some points. As mentioned before, other cells express markers used for detecting plasmablast/plasma cells, such as BLIMP1 (T cells) or CD138 (pro-B/pre-B cells). Therefore, strategies to identify plasma cells based on only one marker should be avoided. In addition, plasma cells express surface markers usually associated with other cell types (*e.g.,* Ly6C [1192], CD11c [1196], CD56 [1197]). Consequently, “dump” gate markers should be carefully selected. Furthermore, methanol/ethanol-based fixation methods will often result in a loss of the GFP-reporter signal. A pre-fixation step can prevent the leakage of cytosolic GFP and enable the retention of GFP fluorescence in a co-staining for cytosolic/nuclear Ags [1198].

The TACI/CD138 staining protocol is also sensitive to different fixation strategies, *e.g.,* formaldehyde fixation. In addition, TACI harbors protease cleavage sites [1199] and can, therefore, be degraded or shedded when enzymes, *e.g.,* collagenases, are used to dissociate tissues. For tissues such as the kidney, mild mechanical disruption in the absence of collagenase using the gentleMACS system (Miltenyi Biotech) is recommended [1200]. In tissues that strictly require enzymatic treatment (i.e., lamina propria of the gut), ENPP1 can be used in combination with CD138 to replace TACI.

As plasma cells are sensitive to mechanical stress due to their enlarged cytoplasm, vortexing of the samples should be avoided, and cell pellets should rather be resuspended by finger tipping the reaction tube or careful pipetting.

A higher abundance of BLIMP1 and CD138 is associated with a more mature stage of plasma cell differentiation [1183, 1184]. As demonstrated in Figure 116B, the CD138^+^/BLIMP1:GFP^+^-population in the bone marrow of mice contains two clearly separated CD138^+^/BLIMP1:GFP^+^ and CD138^high^/BLIMP1:GFP^high^ subpopulations. Analysis of CD138 and B220 abundances revealed that the CD138^+^/BLIMP1:GFP^+^ population still expresses surface B220, while most of the CD138^high^/BLIMP1:GFP^high^ cells are negative for surface B220. Therefore, cells gated on BLIMP1:GFP and CD138 contain early and late plasma cells.

In the bone marrow of unimmunized mice, frequencies of plasma cells range between 0.4-0.6% of viable cells, while frequencies in spleen and lymph nodes vary between 0.3-0.5% and 0.1-0.2%, respectively. Therefore, at least 1×10^6^ events (optimally 3-4x10^6^ events) should be acquired during the flow analysis to collect enough events in the plasma cell gate for valid conclusions. As plasma cells have a larger cell size than other lymphocytes, the regularly used “lymphocyte gate” in the FSc/SSc plot must be extended.

The paradigm of plasma cell differentiation includes the termination of the membrane-bound IgH chain and a switch towards the production of only the soluble form of the B cell receptor. However, Pinto and colleagues found that human plasma cells still express functional IgM and IgA receptors on the cell surface, over-turning the dogma of complete membrane-BCR loss upon plasma cell differentiation [1121]. We and others confirmed this finding in mice, *i.e.*, almost all IgM- and IgA-producing CD138^+^/TACI^+^-plasmablasts and mature plasma cells present these IgH-isotypes as BCRs on their cell surface ([1028, 1063]). In contrast, IgG-producing mature plasma cells have lost the expression of surface IgG. Therefore, one could determine the frequency of IgH-isotype-expressing plasmablasts/plasma cells by including the detection of surface IgM and IgA in the previously described four-color-staining (see Figure 116).

#### 8.7 Summary of the phenotypes

This is detailed in Table 89.

#### 8.8 Key information human vs murine

This is detailed in Table 90.

#### 8.9 Key information murine vs human

- CD27 is not expressed in mouse plasma cells
- CD38 is not a pan-plasma cell marker in mouse
- Ly6a/Sca-1 and Ly6C have no human orthologs

## VI B cell assays

### 1 Measurement of signal transduction pathways in human B cells

#### 1.1 Overview

B cells respond to several stimuli in their microenvironment, such as Ag binding to the B cell receptor (BCR) causing BCR cross-linking and/or stimuli that engage Toll-like receptors. These stimuli activate a series of signal transduction pathways in B cells that culminate in activation of cellular programs that determine B cell activation, differentiation and proliferation. Post-transcriptional changes in signaling proteins, such as phosphorylation of certain tyrosine, serine or threonine residues can be measured by flow cytometry. In this section, we will focus on how to measure these early events of B cell activation.

#### 1.2 Introduction

**Figure 117.**
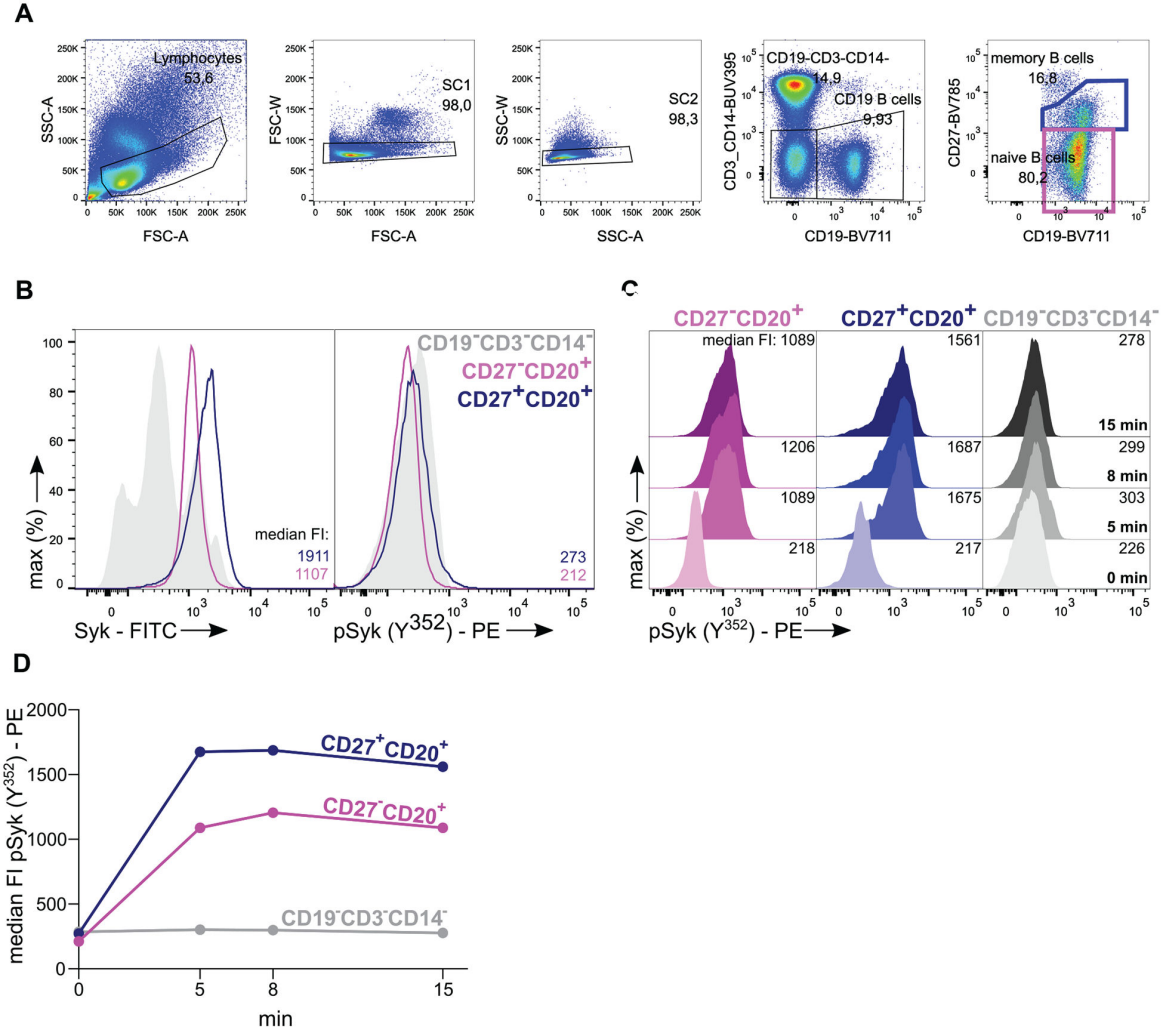
Syk and pSyk (Y352) expression at baseline and after anti-BCR stimulation of CD27-CD20^+^and CD27+CD20^+^human B cells from peripheral blood. (A) Representative histograms of Syk and pSyk (Y352) of peripheral blood of one donor for CD27−CD20^+^naive B cells (pink), CD27+CD20^+^memory B cells (blue) and CD19-CD3-CD14- cells (grey). (B) Histograms of pSyk (Y352) upon anti-BCR stimulation after 5, 8 or 15 min or 5 min incubation with RPMI as control (named 0 min) (C) Kinetic curve showing median FI of pSyk (Y352) over time after anti-BCR stimulation for CD27-CD20^+^naive B cells (pink), CD27+CD20^+^memory B cells (blue) and CD19-CD3-CD14- cells (grey).

BCR or TLR (Toll-like receptor) binding is transduced intracellularly through post-transcriptional modifications to signaling proteins such as tyrosine, serine or threonine phosphorylation. When Ag binds to the BCR, it induces phosphorylation of the BCR-associated Igα (CD79a) and Igβ (CD79b) chains leading to downstream Lyn and spleen tyrosine kinase (Syk) phosphorylation [1201]. Syk gets activated and further phosphorylates downstream targets 1-phosphatidylinositol-4,5-bisphosphate phosphodiesterase gamma-2 (PLCγ2), Bruton’s tyrosine kinase (Btk), and protein kinase B (Akt), which results in Ca2+— and Akt-dependent transcription [1202-1204]. Thus, in order to measure early events of B cell activation upon a stimulus, the phosphorylation of certain amino acid modification sites of Syk (figure 117), PLCγ2, Btk and Akt can be measured by flow cytometry, using Abs that only bind the specific site when it is phosphorylated (table 91).

BCR co-receptors like CD19, CD21, CD81, CD22, CD72 and FcγRIIb regulate BCR signaling strength and B cell activation. While presence of CD19, CD21, CD81 facilitate BCR signaling transduction and B cell activation, co-receptors, such as CD22, CD72 and FcγRIIb recruit phosphatases such as PTP non-receptor type 6 (SHP-1) to the BCR, which dephosphorylates BCR downstream targets [1205-1207]. Thus, considering the expression levels of these surface co-receptors when analyzing signal transduction pathways in B cells is important to obtain a comprehensive and conclusive picture.

BCR signaling features determine B cell fate. So, is not surprising that precise regulation of signal transduction and fine-tuned BCR response is crucial for adequate immune response. Imbalance in signal transduction can lead to dysfunction of the B cell response causing physiological and pathological changes as seen in autoimmunity. Thus, the BCR and downstream pathway cascades play a pivotal role in the development and maintenance of autoimmunity. Therefore, BCR signaling has been extensively studied. There was a general consensus that pathologically increased BCR signaling contributed to B cell overactivity and autoimmunity, but recent studies showed that B cells in autoimmune diseases, such as systemic lupus erythematosus (SLE), rheumatoid arthritis (RA), and primary Sjögren’s syndrome (pSS) displayed diminished phosphorylation of Syk and Btk upon BCR activation and share a phenotype of hyporesponsiveness toward BCR and TLR9 stimulation [1208-1210]. This condition was termed anergic post-activated (APA) B cells and provided evidence for the relevance to analyze intracellular signal transduction cascades in B cells under different conditions.

#### 1.3 Step-by-step sample preparation

Baseline expression of intracellular signaling molecules or phosphorylation kinetics of relevant intracellular protein tyrosine/serine-threonine kinases at upon defined stimulations can be investigated using intracellular flow cytometry.

Signal transduction pathways can be analyzed in several B cell subsets (please see section V.1 Human B cells and their subsets). Therefore, it may be important to define the population of interest and stain for relevant markers accordingly, as, for example, naive and memory B cells have a diverse phosphorylation pattern of BCR associated signaling molecules [1202] and (figure 117). Which signaling molecules to choose depends on the scientific question addressed and the intended read-out for suggestions see table 91.

#### 1.4 Sample preparation and flow cytometer setup

Depending on the starting material and the cell type of interest, different methods for cell isolation can be applied e.g., density gradient centrifugation using Ficoll Paque, magnetic labeling using microbeads or fluorescence-activated cell sorting. Here, we describe the isolation of peripheral blood mononuclear cells (PBMCs) from whole blood via Ficoll Paque.

Dilute anticoagulated whole blood 1:1 with phosphate-buffered saline (PBS) and carefully layer over half the amount Ficoll Paque. Centrifuge at 600g without brake at room temperature for 20 min. Harvest PBMC layer and wash 1:10 with PBS (10 min, 330g). Discard supernatant and filter cells suspension and wash again 1:15 with PBS (10min, 330g). Discard supernatant and count cells. Viability staining can be done at this step using LIVE/DEAD Fixable Dead Cell Stain Kit according to manufacture recommendations. Wash cells before proceeding with either the baseline or stimulation protocol. FcR blocking reagent (Miltenyi Biotec) is used for 5 min before staining.

Daily use of cytometer calibration e.g., Cytometer Set-Up and Tracking beads (BD) according to flow cytometer manufacture is highly recommended. Compensation setup for the staining could be done by single stained cells or by using Compensation Particles Set Beads (BD).

##### 1.4.1 Baseline.

Fresh peripheral whole blood or isolated peripheral blood mononuclear cells (PBMCs) can be used. In case surface and intracellular staining, surface makers can be stained after the viability staining before the Lysis/Fix/Perm steps for 15 min followed by a washing step. In some cases, surface markers can also be stained together with intracellular markers. Hydrogen peroxide treatment increases phosphorylation of kinases [1211], therefore, cells treated with H_2_O_2_ (3% in RPMI) can be used as positive control. Cells are lysed and fixed in 1 ml pre-warmed Lyse/Fix Buffer (10 min, 37 °C). Next, wash twice with 3 mL ice cold PBS (8 min, 500g) and permeabilize with 200 μL Perm buffer II or III (table 91) (−20 °C, 12 h or 30 min on ice). Subsequently, wash the cells two times with 3 mL of staining buffer (8 min, 500g, 4 °C), stain with surface markers of interest (section V.1 Human B cells and their subsets) and one of the combinations of Syk-FITC/pSyk(Y^352^)-PE (figure 117A), PLCγ2- PE/pPLCγ2(Y^759^)-FITC, Akt1-PerCp-Vio700/pAkt(S^473^)-PE or Btk-PE/pBtk(Y^223^)-FITC (table 91), for 1 hour at room temperature. Finally, wash the cells two times with 3 mL of staining buffer (8 min, 500g, 4 °C) and analyze MFIs of the population of interest. CD19^−^CD3^−^CD14^−^ can serve as negative control (figure 117A).

##### 1.4.2 Phospho kinetics upon stimulation.

Analysis of BCR-associated signaling molecules is particularly useful to analyze signal transduction upon stimulus. As an example, we describe here the functional analysis of BCR-associated signaling molecule Syk, upon BCR stimulus. Similar experiments can be carried out for other protein tyrosine kinases, such as Btk, PLCγ2 or the protein serine/threonine kinase Akt1 (table 91), among others. This type of analysis implies to stimulate B cells and analyze the corresponding phosphorylation kinetics upon BCR stimulation. Therefore, 1-2x10^6^ isolated PBMCs or thawed MNCs resuspended in RPMI can be used. Thawed cells should be rested for 1 h at 37 °C. Next, add anti-BCR stimulation (15 μg/ml anti-IgM/IgA/IgG in RPMI) for 5, 8 and 15 min. As baseline control (0 min) some cells are left unstimulated by incubating with RPMI instead of stimulants for 5 min. Stop the reaction at different timepoints by adding 1 ml pre-warmed (37 °C) Lysis/Fix buffer and incubate additional 10 min. Wash twice by adding 2mL of cold PBS and centrifuged at 500g for 8 min at 4°C. Next, permeabilize with 200 μL Perm buffer II (−20 °C, 12 h or 30 min on ice). Wash the cells two times with 3 mL of staining buffer (8 min, 500g, 4 °C) and stain with Abs targeting surface markers of interest (see section V.1 Human B cells and their subsets) and Syk-FITC/pSyk(Y^352^)-PE for 15 min at 4°C. Finally, wash the cells two times with 3 mL of staining buffer (8 min, 500g, 4 °C) and analyze MFIs of the population of interest (figure 117B and C).

#### 1.5 Materials

- Ficoll Paque (GE Healthcare)
- PBS (Biochrom)
- Staining buffer (PBS/0.5% BSA/EDTA (Miltenyi autoMACS Rinsing Solution/MACS BSA Stock Solution))
- Buffers for cell permeabilization (e.g., Phosflow Lyse/Fix Buffer (BD Biosciences), Phosflow Perm Buffer II or III (BD Biosciences))
- Buffers for erythrocyte lysis (e.g., Lysing Buffer (BD PharmLyse ^™^ BD Biosciences, Buffer EL (Qiagen)),
- For mouse derived Abs: Anti-Mouse Ig, κ/Negative Control Compensation Particles Set (BD Biosciences)
- LIVE/DEAD Fixable Dead Cell Stain Kit, (Invitrogen)
- Fluorescently labeled monoclonal Abs specific for certain phosphorylated sites. Some of the most common used Abs can be found in table 91.
- anti-BCR stimulation: IgM/IgA/IgG (Jackson ImmunoResearch)

#### 1.6 Data analysis

Data analysis can be executed with any program suited for flow cytometry analysis e.g., FlowJo or BD FACS Diva. After identifying lymphocytes and doublet exclusion, proceed gating for the cells of interest (also see section V.1 Human B cells and their subsets) and export median FIs for channels representing the molecules of interest.

#### 1.7 Pitfalls and top tricks

- For reliable measurements of MFIs over time, use calibration beads
- Make sure to use a fixable dye for viability staining
- Keep the permeabilization buffer ice cooled
- From our experience, staining surface markers prior to intracellular markers give rise to better staining, even though is time consuming.
- It is important to increase centrifugation speed after permeabilization to avoid losing cells.

#### 1.8 Clinical relevance statement

The assays described in this section are applicable to study signaling pathways in various B cell subsets and different diseases. As done for patients with autoimmune diseases where reduced phosphorylation of signaling molecules downstream of BCR upon stimulation identified the post-activated status of B cells [1209].

### 2 Live cytokine-producing human B cell sorting with secretion assay: the case of IL-10

#### 2.1 Overview

B lymphocytes contribute to immunity through the presentation of Ag to T cells, the production of Abs, and the secretion of cytokines including IL-2, IL-4, IL-10, IL-17, IL-35, IFN-γ, and GM-CSF. B cell-derived cytokines act through autocrine mechanisms to regulate their migration and differentiation, as well as in a paracrine manner to stimulate lymphoid organ development and modulate immune responses. To illustrate the possibility of functionally characterizing cytokine-producing B cells, we describe here a strategy to identify and isolate human IL-10-producing B cells without compromising their viability using a single cell cytokine capture assay.

#### 2.2 Introduction

The prominent role of B cells as Ab-secreting cells has for a long time overshadowed their other activities. However, it is now well recognized that they play important roles through the presentation of Ag to T cells, and the production of cytokines [1057]. Their capability to produce various cytokines is an important feature throughout all B cell developmental stages and subpopulations. Already immature B cells leaving the bone marrow use IFN-γ production to control their selective migration to the spleen in an autocrine fashion [1026]. Provision of granulocyte-macrophage-colony-stimulating factor (GM-CSF) by "Innate Response Activator B Cells" has been reported to be crucial for protection against microbial sepsis [1055]. Mature B cells include diverse and only partly characterized regulatory B cell and plasma cell subsets. A key function of these cells is the secretion of the anti-inflammatory cytokines IL-10, IL-35 and TGF-β [963, 972, 1212]. Even fully mature plasma cell stages in the bone marrow retain the capability to produce IL-10 [936, 1063, 1213], and IL-10 production by malignant plasma cells may contribute to the immunodeficiency observed in multiple myeloma [1062]. The analysis of murine regulatory B cell subsets by surface markers is described in section 2 of this chapter. The knowledge available on human IL-10-producing B cells is limited. Their investigation requires the possibility of identifying and isolating these cells reliably without compromising their viability.

In comparison to T cells, the detection of cytokine-producing human B cells by intracellular staining is difficult and requires distinct methods of restimulation.

We have previously described a methodology based on a cell-surface affinity matrix allowing to capture secreted products on the surface of secreting single cells [732]. This method has been used to analyze and isolate live cytokine producing T cells [1214-1216]. We and others have also adopted it for the isolation of human IL-10-secreting B cells and plasmablasts [970, 1217, 1218].

The principle of the protocol is simple: single cells are labeled with bi-specific reagents containing a capture Ab directed against the leucocyte marker CD45 and an anti-cytokine Ab e.g., anti-IL-10. These bi-specific conjugates immobilize the produced cytokine on the surface of the cytokine-secreting cells. In order to prevent cross-feeding of cytokines from secreting to non-secreting cells, labeling with the capture Ab is performed in the cold to block unwanted cytokine secretion at this stage. Afterwards, the cells are placed into a warm medium, allowing for cytokine secretion. This is done at low cell densities to prevent the spreading of cytokines from secreting to non-secreting cells. Within approximately 30 minutes, the secreted cytokines have selectively saturated the capture Abs on the surface of cytokine-secreting cells. After that period, the cells are cooled down to 4°C to stop cytokine secretion. At this stage, the secreted cytokines are immobilized on the surface of the cytokine-producing cells, allowing their direct identification as live cells using fluorochrome-labeled anti-cytokine Abs. It is a pre-requisite that the two Abs used for the cytokine capture and detection bind distinct epitopes of the cytokine, and hence do not inhibit each other, similar to what is required for Abs used in sandwich ELISA.

Here, we describe the usage of this single cell cytokine capture assay for the identification of human IL-10-producing B cells by flow cytometry. This protocol can in principle be adapted to identify and isolate B cells secreting other cytokines of interest, as previously described for human CD4^+^ T cells [461].

#### 2.3 Step-by-step sample preparation

PBMCs are prepared according to standard protocol. In brief, heparinized full blood is mixed 1:1 with ice cold PBS/0.2%BSA. This solution is then gently layered over a solution of Ficoll (D=1.077) using 35 ml of diluted blood for 15 ml of Ficoll in 50 ml sterile plastic tubes. The tubes are centrifugated for 20 min at 400 × *g* speed without brake and at room temperature to obtain the density gradient. The interphase containing the PBMCs is then carefully collected and transferred into a tube filled with PBS/BSA. To remove the Ficoll remaining in this suspension (collected together with the PBMC interphase), the cells are centrifugated at 4°C speed 300 × *g* for 15 min. The supernatant is removed, and the cells are washed twice using 50 ml PBS/BSA and centrifugation at 200g for 10 min.

To isolate B cells from the PBMCs, the cells are labeled with CD19-microbeads at a ratio of 1-4x10^7^ PBMCs in 800 μl PBS/BSA^+^200 μl bead (1 ml final) for 15 min at 4°C. The cells are then washed in PBS/BSA by centrifugation at 200g for 10 min, and isolated magnetically by positive selection.

It is then possible to look for IL-10-producing B cells directly *ex vivo*. To this end, go directly to step A.

**Figure 118.**
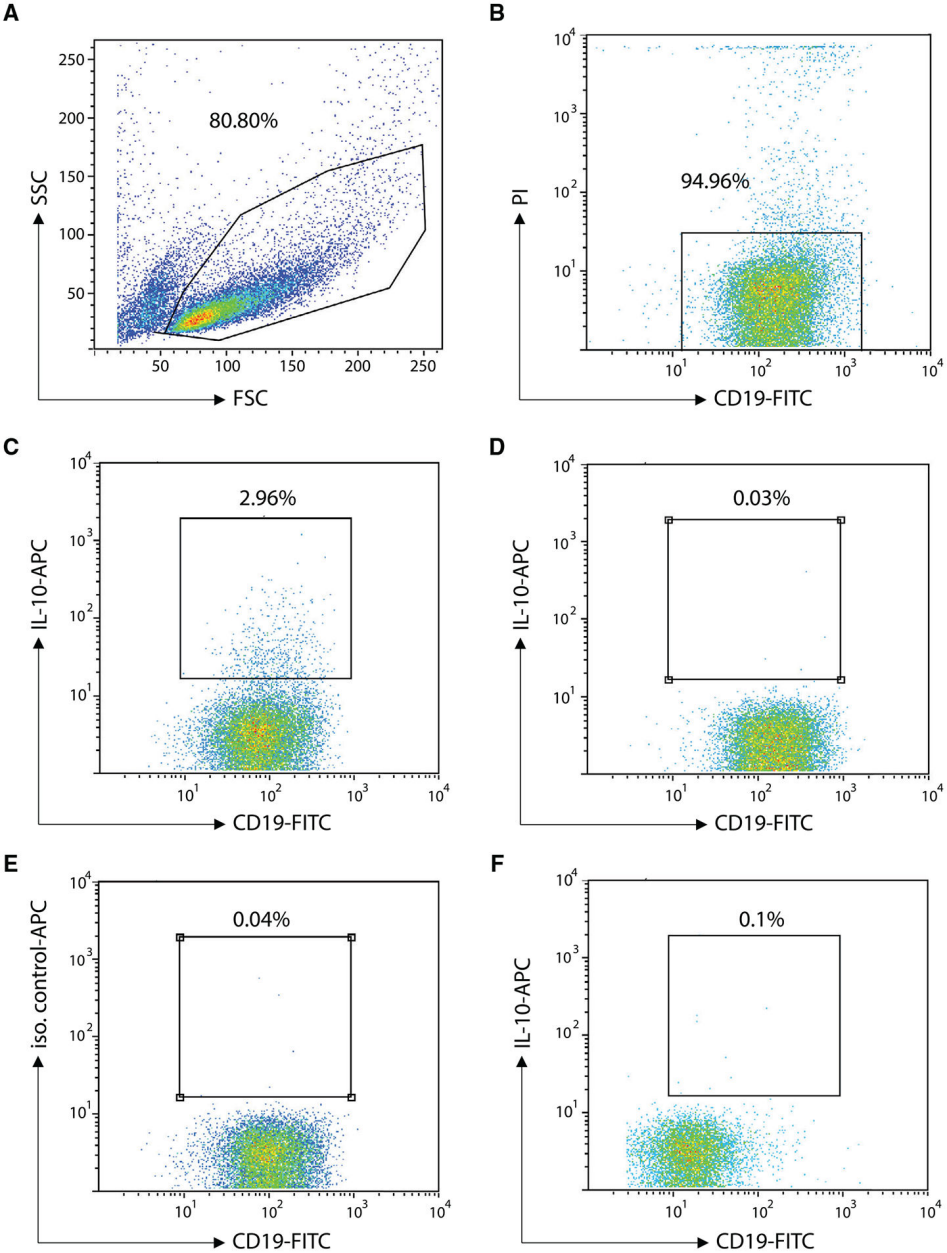
Detection of IL-10 secreting human B cells. CD19^+^ B cells were cultured at 2.5x 10^6^/ml in RPMI1640 medium supplemented with 10% FCS and Pen/Strep for 2 days in the presence of 2 μg/ml anti-IgM/IgG-F(ab)_2_ fragments (Jackson ImmunoResearch), 1 μg/ml anti-CD40 (clone 82111 R&D systems), 10 ng/ml IL-4 (Immunotools, Germany). After a restimulation with 10 ng/ml PMA and 1 μM Ionomycin for 3 hours, the cells were labeled with a bivalent IL-10 capture matrix and the IL-10 secretion period was performed at 37°C for 30 min. After washing, the cells were stained and analyzed with a flow cytometer. Cells were first gated according to FSC/SSC (linear scales) to remove debris and dead cells from the analysis (A). Live CD19^+^ B cells were then identified within this FSC/SSC gate according to the lack of propidium iodide labeling and CD19 staining (log scales) (B). Dot plots gated on live CD19^+^ lymphocytes then show CD19 and IL-10 staining (log scales) for stimulated cells with secretion period and IL-10 staining (C). Negative controls include (D) stimulated cells with no secretion period and IL-10 staining, (E) stimulated cells with secretion period and isotype control staining, (F) unstimulated cells with IL-10 staining. Data derived from a representative experiment.

Alternatively, it is possible to stimulate purified B cells for 1 or 2 days before looking for IL-10-producing cells. The optimal IL-10-inducing signals are: 3 μg/ml CpG2006 (plateau approx. 1-10 μg/ml) > anti-Ag receptor (e.g., 2 μg/ml anti-IgM/G-Fab2 fragments, Jackson ImmunoResearch) > anti-CD40 ^+^IL-4 (e.g., 1 μg/ml clone 82111 R&D Systems, 5 ng/ml rhu-IL-4 Miltenyi Biotec). The optimal induction of IL-10 expression in B cells requires a high cell density. Thus, adjust the cell number to 2.5x10^6^ cells/ml in ice-cold medium (1640 RPMI with 10% FCS) and seed 1 ml per well in 24 well or 250 μl in 96-well plates. After the *in vitro* stimulation, the B cells are subjected to the IL-10 cytokine secretion assay as follows:

- Add 10 ng/ml PMA + 1 μM ionomycin (both Sigma) to the B cell culture for 4 h at 37°C in cell incubator, and then cool down the cultures by adding ice-cold PBS/BSA. Transfer the cell suspension in a 15 ml tube and place it on ice for 10 min. After a washing step at 4°C, resuspend the cell pellet in 80 μl PBS/BSA and add 20 μl anti-IL-10 matrix (bivalent conjugate of anti-CD45/-IL-10, Miltenyi Biotec) for 10 min on ice, shake gently every other minute, then resuspend in 10 ml PBS/BSA. Wash cells by centrifugation at 200g for 10 min at 4°C, and then resuspend the cells thoroughly in 10 ml of prewarmed (37°C) 1640 RPMI medium supplemented with 10% FCS. Adjust the cell concentration to 10^6^ cells/ml if <5% IL-10-secreting cells are expected, and 10^5^ cells/ml or below if >5% IL-10-secreting cells are expected. The volume can range from 5-10 ml to 200 ml of medium depending on the expected frequency of IL-10-producing cells and the number of final cells desired. To start the secretion period, which should last for 30 to maximum 60 minutes, place the cells at 37°C in cell incubator. It is best if the cell suspension is moving during this step, for instance by frequent gentle shaking or using a rotating device in the incubator (e.g., MACSmix Tube Rotator), in order to avoid any durable interaction between secreting and non-secreting cells and thus reduce crossfeeding. After this secretion phase, add 5 ml of ice-cold washing buffer and immediately put the cells on ice for 10 min to stop the secretion and reduce the background. It can be a problem if the cells are too dense during the secretion period, if the frequency of IL-10-expressing cells is too high, or if the culture is maintained for too long because this can increase the non-specific transfer of secreted IL-10 onto IL-10-negative B cells (termed “crossfeeding"). In order to control for the specificity of the staining, it is useful to analyze not only stimulated B cells after secretion at 37°C using an appropriate anti-IL-10 Ab (Figure 118C), but also, for negative controls, to keep an aliquot of stimulated B cells on ice (“no secretion time”) (Figure 118D), to stain stimulated B cells with an isotype control Ab (Figure 118E), and to analyze unstimulated B cells (Figure 118F).

It might be possible to improve the experimental setup described above using a distinct matrix in which the anti-IL-10 is linked to an Ab against a different cell surface receptor than CD45, in order to focus more selectively on the cell subsets of interest, once the cell surface phenotype of IL-10-producing cells has been defined. Of note, the majority of B lineage cells express CD45 and are thus amenable to labeling using the anti-CD45/anti-IL-10 conjugate. However, CD45 is down-regulated on some bone marrow plasma cells [1105], and some malignant B cells [1219].

B) After the 10 minutes incubation on ice, wash the cells with ice-cold PBS/BSA, centrifuge 10 min at 4°C 200g, and resuspend the cells for staining e.g., anti-IL-10 (the best staining is obtained with PE- or APC-conjugated Abs, clone JES3-9D7 from Miltenyi Biotec). For correlation with intracellular IL-10 expression (see section III.1.3.3 for the staining of intracellular cytokines (in T cells)), the clone JES3-19F1 from BD Biosciences is recommended.
C) Stain immediately for other surface markers using Abs titrated before for 10 min on ice. Then wash the cells twice by centrifugation for 10 min at 4°C 200g.
D) Measure the sample at your flow cytometer. For B cells analyzed with this assay directly *ex vivo* after magnetic isolation, the proportion of IL-10^+^ B cells is usually around 0.5-4%.

#### 2.4 Materials

PBS + 0.2% BSA (EDTA addition is not required)

RPMI + 10% FCS (antibiotic supplementation not required)

IL-10 secretion assay (used also for T cells)

10ng/ml PMA (stock in 1mg/ml EtOH) + 1μg/ml ionomycin (stock in 1mg/ml DMSO); aliquoted for single use and stored at −20°C.

50 ml sterile plastic tube

15 ml sterile plastic tube

4°C fridge and crushed ice

37°C incubator, best with a rotating device inside

Flow cytometer

#### 2.5 Data analysis

Gate on CD19^+^ cells, exclude dead cells and doublets and lineage markers to exclude signals from potentially contaminating cells. Set the gate to identify cells secreting IL-10 using the “not-secreted control”.

#### 2.6 Top tricks

- Optimize the amount of anti-IL-10 matrix applied. Usually, we use 20 μl per 10^7^ B cells in 100μl (1:5 ratio) PBS/BSA (final volume). To reduce background, increase the labeling ratio to 1:10 or decrease the amount of anti-IL-10 matrix (do not use less than 10 μl of matrix, however).
- Use large volumes of medium during the secretion period and keep the cells moving in the suspension, e.g by gentle shaking or using a tube rotation device in order to avoid contact of IL-10 secreting cells with negative cells. This should be adjusted to the frequency of IL-10-secreting B cells (in case of CpG6006 + anti- BCR + anti-CD40 + IL-4, the frequency of IL-10-expressing B cells can be around 30%, and the optimal volume is in this case around 30 ml for 1-2x10^6^ B cells).
- Reduce the secretion period according to the frequency of IL-10-secreting B cells, in case the background (“crossfeeding”) is a problem. If this occurred, the analysis requires adjustment according to the negative population (see suppl. Figure 1D in [1217]).
- Carefully check the numbers of other cell types contaminating the IL-10-secreting fraction, even after magnetic enrichment of B cells (most critical cell types to check are CD14^+^ monocytes and CD4^+^ T helper cells).
- To validate the obtained results, it is useful to quantify the amount of IL-10 secreted in the supernatant using appropriate systems. It is also relevant to measure IL-10 mRNA in B cells sorted into IL-10^+^ and IL-10^−^ cells by quantitative PCR (see suppl. Figure 1B in [1217]). The validity of the experimental conditions and the absence of crossfeeding can be further checked by adding cells of different lineages unable to secrete IL-10 in the culture or by counterstaining for intracellular IL-10 expression.

### 3 Antigen-receptor mediated Ca^2+^ mobilization in lymphocytes

#### 3.1 Overview

Ca^2+^ ions play an essential role as an intracellular messenger in nearly all cellular systems and regulate a multiplicity of cellular functions. In this section we focus on Ag receptor- mediated Ca^2+^ mobilisation in B-cell subpopulations by means of Indo-1 AM. However, this method can be adapted to any other cell population, lymphocyte subpopulation or cell line which can be triggered via any surface receptor that induces Ca^2+^ flux.

#### 3.2 Introduction

In the immune system Ca^2+^ mobilization induces many direct processes such as activation of platelets, degranulation of mast cells or the killing of target cells by cytolytic T cells. It is also an essential component of the signaling cascades downstream of several receptors, including the B- and T-cell receptors, activating Fc receptors, and chemokine receptors, and has been shown to regulate the transcription of target genes and subsequently driving processes such as proliferation, and differentiation, suggesting that Ca^2+^ mobilization should be a consideration in many aspects of immunological research [1220-1223]. In brief, in the case of Ag receptors, binding of the Ag initiates a signaling cascade leading to the generation of the second messenger IP_3_. This binds to its receptor in the endoplasmic reticulum (ER) membrane after which Ca^2+^ is released from the ER into the cytoplasm. This release is the very transient internal store release (ISR) [1221, 1222]. Reduced Ca^2+^ levels in the ER are sensed by STIM1/2, leading to the opening of the Ca^2+^ release-activated Ca^2+^ (CRAC) channels such as ORAI1 in the plasma cell membrane causing a more sustained store operated Ca^2+^ entry (SOCE) from the extracellular space into the cytoplasm [1221, 1222]. Based on patch clamp methods Ca^2+^ currents can be measured very precisely at the single cell level [1224].

Because this method is not feasible for many laboratories, determination of Ca^2+^ mobilization by means of flow cytometry may represent an easy alternative, providing relative values of Ca^2+^ mobilization at the single cell level.

**Figure 119.**
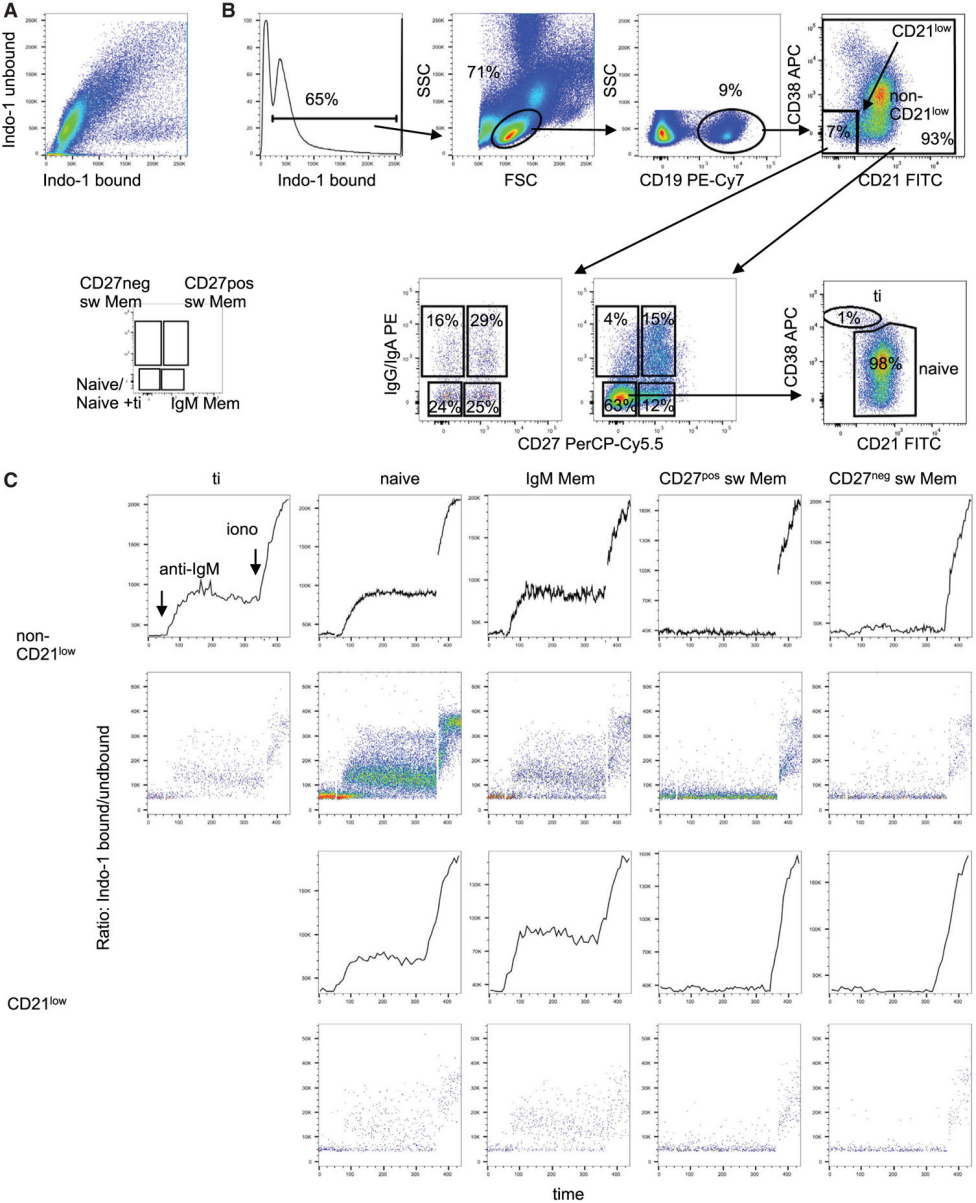
BCR induced Ca^2+^ mobilization in human B cell subsets from peripheral blood. (A) Setting of Indo-1 AM bound versus Indo-1 AM unbound. The photomultipliers (PMTs) should be adjusted so that unstimulated cells occur on a line about 45° to the y-axis. (B) Gating strategy for the analysis of Ca^2+^ mobilization in human B-cell subsets from peripheral blood after stimulation with anti-IgM. After exclusion of cells that did not bind Indo-1 lymphocytes are determined by FSC/SSC. Gating of CD19^+^ B cells is followed by differentiation of CD21^low^ B cells and non-CD21^low^ B cells. IgG/IgA and CD27 is subsequently used to differentiate IgG/IgA^−^/CD27^−^ naïve or naïve and transitional (ti) B cells, IgG/IgA^−^/CD27^+^IgM Memory B cells (IgM Mem) and IgG/IgA^+^/CD27^+^class switched B cells (CD27^pos^ sw Mem) and IgG/IgA^+^/CD27^−^ switched Mem B cells (CD27^neg^ sw Mem). IgG/IgA^−^ CD27^−^ non-CD21^low^ B cells can further be differentiated in ti and naïve B cells. (C) Kinetics (upper panels) and dot plots of time versus the ratio Indo-1 bound/unbound (lower panels) of the subpopulations of non-CD21^low^ B cells or CD21^low^ B cells as described above. After baseline acquisition anti-IgM (arrow) was added inducing a shift of Indo-1 AM bound/unbound in IgM-expressing naïve and IgM Memory B cells whereas this ratio is at baseline levels in IgM class switched memory B cells. After addition of iono (arrow) the ratio of Indo-1 AM bound/unbound is rapidly increasing in all subsets. Data were acquired with a BD LSR FortessaTM and analyzed by FlowJoTM.

Indo-1 acetoxymethyl (AM) is a cell-permeant ratiometric Ca^2+^ indicator, used to determine intracellular Ca^2+^ mobilization at the single cell level [1225]. The dye is excited at 355 nm and therefore requires a true UV laser. The Indo-1 AM emission peak at 475 nm in the absence of Ca^2+^ shifts to 400 nm upon binding Ca^2+^ ions. Therefore, changes in the ratio of Ca^2+^-bound Indo-1 AM signal at 475 nm to Ca^2+^-unbound Indo-1 AM signal at 400 nm allow the immediate detection of alterations in intracellular Ca^2+^ concentration (Figure 119A).

Alternative methods for detecting Ca^2+^ by flow cytometry include methods involving the use of Fluo-3 [1226], either alone or in combination with Fura Red, taking advantage of excitation with a standard 488 nm laser [1227, 1228]. This avoids the necessity of the more costly UV laser required for excitation of Indo-1. Furthermore, Fura Red can also be used on its own taking advantage of differential excitation from the violet (405 nm) and green (561 nm) lasers, enabling ratiometric measurements as for Indo-1 [1229]. Ratiometric measurements have the added advantage of controlling internally for cell size and dye uptake. An excellent overview of the different dyes that can be used for Ca^2+^ analysis can be found at https://www.thermofisher.com/us/en/home/references/molecular-probes-the-handbook.html. Animal models further allow the usage of genetically encoded Ca^2+^ indicators as GCaMP6f or fusion proteins thereof as Salsa6f with high sensitivity, a high dynamic range and true ratiometric imaging, combined with all advantages of transgenic organisms as expression in specificity tissues, cells or organelles [1230, 1231].

#### 3.3 Step-by-step sample preparation

##### Isolation of peripheral blood mononuclear cells (PBMCs)

- See Chapter IV. Before you start: Reagent and sample preparation, experimental design; Section 4. Pre-enrichment of low abundant cell populations prior to acquisition/cell sorting; 4.2 Pre-enrichment by physical properties in [22]

All steps of cell isolation should be performed at room temperature with buffers and media also at room temperature! If this is not possible the cells should be allowed to equilibrate to room temperature for 30 min.

###### Loading:

- Although strongly depending on the required resolution and the abundance of the population of interest >=2x10^6^ PBMCs per measurement are required. Cells are adjusted to 10×10^6^ PBMCs per ml IMDM/10% FCS (alternatively RPMI can be use throughout the protocol) and the required volume of cell suspension is incubated with 4.5 μM Indo-1 AM in the presence of 0.045% of the detergent Pluronic F-127 for 45 min at room temperature in the dark [1232].
- Mix the cell suspension during the loading procedure by dragging the sample tubes over a tube rack every 15 min.

###### Washing:

- Wash twice with 5 ml IMDM/3% FCS (300 g, 5 min, at room temperature), remove supernatant.

###### Cell surface staining:

- Add fluorescence-conjugated Abs.
- Incubate for 15 min at room temperature in the dark.

###### Washing:

- Wash with 4 ml IMDM/3% FCS, remove supernatant. Resuspend cells in 300 μl IMDM/10% FCS.

The sample measurement should be performed within the next one to two hours!

###### Flow cytometer settings:

- Display Indo-1 AM bound (FL12 405/10) and Indo-1 unbound (FL13 520/35, 445 LP) on a linear scale.
- View Indo-1 AM unbound on the y-axis and Indo-1 AM bound on the x-axis. Adjust the photomultiplier (PMT) voltage so that the signals from unstimulated cells are located on a line about 45° to the y axis (Figure 119A).
- A dot plot showing time on the x-axis versus the ratio of Indo-1 bound/unbound on the y-axis displays the kinetics of Ca^2+^ mobilization. A scaling of 25% is recommended. Ensure that the baseline and the maximal peak upon stimulation (iono) are within the displayed range. If this is not the case the PMTs must be adjusted.

###### Data acquisition:

Do not change the velocity of data acquisition during the measurement

- Acquire the baseline for 30 sec.
- Remove the tube and add 15 μg/ml anti-IgM (do not stop data acquisition), vorte× gently.
- Acquire for an additional 4 min.
- Add 1 μg/ml iono as a loading control (do not stop data acquisition). In the presence of Ca^2+^ in the medium and proper labeling of the cells with Indo-1 AM all cells have to show a maximal increase in the intracellular Ca^2+^ concentration. Stop acquisition after an additional 90 sec.
- Wash the flow cytometer thoroughly before the next tube is loaded. Run fresh tubes of PBS twice for 1 min each (residual iono can directly induce Ca^2+^ mobilization in the subsequent sample).

To allow for comparison of different data sets the rate of sample flow, the time of baseline acquisition, time point of BCR stimulation and addition of iono must be kept constant between samples.

#### 3.4 Materials

##### Reagents, media, instrumentation:

**Table:** 

IMDM	Life Technologies
FCS	Sigma
Indo-1 AM	Life Technologies
DMSO	Sigma
Pluronic F-127	Life Technologies
Ionomycin	Sigma
Fortessa BD equipped with an UV laser	BD Biosciences

##### Antibodies:

**Table:** 

Antibody and fluorochrome	Clone	
CD21 FITC	BL13	Beckman Coulter
IgG PE	G18-145	BD Biosciences
IgA PE	goat polyclonal	Southern Biotech
CD27 PerCp-Cy5.5	O323	Biolegend
CD19 PE-Cy7	B62311	Beckman Coulter
CD38 APC	HIT2	Biolegend
anti-IgM F(ab‘)_2_ UNLB	goat polyclonal	Southern Biotech

#### 3.5 Data analysis

Depending on the required resolution of the information, data analysis can be performed by using standard acquisition software such as BD FACSDIVA^™^ (BD Biosciences, San Jose, CA) or similar. In addition, the analysis software programs FCS Express^™^ from De Novo Software (Glendale, CA), Flowlogic^™^ from Inivai Technologies (Victoria, Australia) and FlowJo^™^ (Treestar Inc., Ashland, OR) each offer a “kinetics” tool to analyze the acquired Ca^2+^ mobilization data. An example for anti-IgM-induced Ca^2+^ mobilization in human B-cell subpopulations when analyzed by Flowjo^™^ is shown in Figure 119 B. Prior to further gating, Indo-1 AM-negative cells must be excluded. Thereafter, the commonly used gating strategy including FSC/SSC, exclusion of doublets and gating on CD19^pos^ B cells. With this simple surface staining a variety of different B-cell subpopulations in peripheral blood can be analyzed at the same time: CD21 versus CD38 allows gating of CD21^low^ B cells and residual non-CD21^low^ B cells. CD27 versus IgG/IgA allows discrimination of CD27^neg^ IgG/IgA^neg^ B cells including naïve, naïve-like and transitional B cells, IgM Memory, classical CD27^pos^ and CD27^neg^ switched memory B cells. In the absence of CD10, CD21 versus CD38 of CD27^neg^ IgG/IgA^neg^ non-CD21^low^ B cell subset may be used to further differentiate CD38^high^ transitional B cells from naïve B cells.

Looking at the respective B-cell subpopulations in a dot plot showing the ratio of Indo-1 AM bound/unbound vs. time gives a better impression than merely looking at the kinetics function (Fig. 119C), since Ca^2+^ kinetics provide multiple read-out parameters. For example, the mean peak intensity and the time to peak, imply the early phases of Ca^2+^ mobilization, which in B cells is essential for the induction of NF-κB and JNK [1233]. In contrast, the decline represents the later phase, which is important for the activation of NFAT [1233, 1234]. The percentages of responding cells can differ, and the non-responding population will strongly influence the read out, especially with regard to the mean values. We therefore advise you to perform an additional analysis of the parameters mentioned above, referring to the responding cells only, by setting the baseline as the threshold and excluding non-responding cells from further analysis.

#### 3.6 Pitfalls

As with all functional assays, control samples which have ideally undergone the same pre-analytical steps as the test samples are required for Ca^2+^ mobilization studies. This is especially important when samples were shipped or previously frozen. The optimal temperature for the investigation of Ca^2+^ mobilization, as for all signaling studies, is 37° C. However, standard instruments are usually not equipped with a heatable acquisition chamber to maintain the samples at a constant temperature of 37° C during the measurement. Strong fluctuations in temperature during cell preparation and between the different experiments should be avoided, since this may influence the Ca^2+^ flux. Although most cell types are capable of inducing Ca^2+^ mobilization at room temperature (e.g., human lymphocyte subpopulations), some cell types are more sensitive and may require 37° C to run the assay. In most cases pre-warming of the samples to 37° C improves Ca^2+^ mobilization, but subsequent cooling during the measurement may lead to changes of the Ca^2+^ baseline levels in some subpopulations and may thus render the analysis inaccurate. Therefore, we perform the entire process of loading, staining, washing and measuring the cells at room temperature. Of note, during cell isolation or preparation (e.g., isolating PBMCs through Ficoll), labeling and staining, the use of cold PBS and other media should also be avoided. Furthermore, mechanical force may induce Ca^2+^ flux. Therefore, carefully dragging the sample tubes over a tube rack to mix them during the entire procedure is better than vigorous shaking or vortexing of the cells.

It is important to make sure that the Abs used for cell surface staining do not themselves induce Ca^2+^ mobilization. This can be tested by adding the staining Ab to Indo-1 AM loaded cells and detecting the resulting Ca^2+^ levels. Since kinetics may vary, the period of acquisition for these tests should be for at least 10 to 15 min. If the Ca^2+^ baseline shifts in response to the staining Ab, that Ab should not be used. To test whether one of the staining Abs interferes with binding of the Ab used for stimulation, the measurement should be compared in the presence and absence of the respective cell surface Ab.

If datasets from different days have to be compared, it is recommended that you keep the times between loading, staining and data acquisition constant for all of the samples.

The UV laser should be turned on at least 15 min beforehand to allow it to stabilize prior to use, since it is highly sensitive and more prone to fluctuation than other lasers. To ensure data reproducibility it is also useful to wait a few seconds after loading the tube before recording the events. This will provide a better definition of the baseline. The flow rate should be kept constant throughout the measurement at low or intermediate rates. However, if the population of interest represents only a very small percentage of the acquired cells, it will be necessary to measure at higher speed in order to be able to record enough events/second for your analysis.

It is important to note that extracellular concentrations of Ca^2+^ will affect Ca^2+^ entry, with higher concentrations leading to higher entry, and other ions (e.g., Cl^−^, Na^+^, K^+^) may also have an effect. The presence of serum may influence the availability of ions and other factors. The phosphate present in PBS may precipitate Ca^2+^, also affecting extracellular Ca^2+^ levels. Depending on your experimental question, cell type and other conditions, IMDM with serum and/or PBS may not be optimal to use as the final buffer. Because of this, it may be helpful to make your own buffer, in which the concentrations of all ions are known. For washing your samples and the final measurement on the cytometer, it may help you to use HBSS solution without Ca^2+^ (Hanks solution), preferably selfmade, and to supplement a portion of this with 2 mM Ca^2+^ on the day of the experiment.

#### 3.7 Top tricks

##### Measuring different cell types and various subpopulations

Beside B cells, the protocol described above is in general applicable for the determination of Ca^2+^ mobilization in T cells, NK cells, granulocytes, monocytes from peripheral blood and tissue and also different cell lines. By adding surface markers to the Ab staining, a high resolution of the different subpopulations can be achieved. The stimuli have to be adjusted, according to the Ca^2+^ flux-inducing receptor. In primary human T cells anti-CD3 Abs must be cross-linked, NK cells can be activated by cross-linking NKG2D and 2B4. To analyze class switched B-cell populations in parallel, anti-Ig instead of anti-IgM can be used for BCR stimulation. While Ca^2+^ levels are relatively similar in T and B cells, different cell types have different intracellular Ca^2+^ levels. The appropriate PMT settings for B cells would not necessarily fit those for granulocytes or cell lines, therefore PMTs should be reset accordingly.

For cell lines it might be necessary to serum starve the cells prior to Ca^2+^ determination, therefore both loading and washing steps could occur in the absence of FCS, or in the presence of lower concentrations of FCS. Alternatively, lower concentrations of Indo-1 AM, shorter incubation times and the omission of Pluronic F-127 can be tested, depending on the cell type and the precise application, leading to changes in the fluorescence intensity of the Indo-1 AM.

##### Distinction between ISR and SOCE

Cell culture medium usually contains Ca^2+^. To differentiate between ISR, from the ER into the cytoplasm, and SOCE, from the extracellular space into the cell, Ca^2+^-containing medium has to be removed by washing and resuspending the cells in Ca^2+^-free PBS or other Ca^2+^-free buffers. Alternatively, EGTA, a chelator which is related to EDTA but which preferentially binds Ca^2+^ ions, can be used. The transient ISR is detected after the appropriate stimulation, while subsequent addition of CaCl_2_ during the measurement reveals the sustained SOCE.

#### 3.8 Clinical relevance statement

The assay shown in Fig. 119 is applicable for analysis of B cells in patients with different forms of B cell defects as in patients with common variable immunodeficiency [1232] but also in patients with autoimmunity [1235] providing information about the integrity of BCR signaling. In patients with suspected inborn errors of immunity, Ca^2+^ mobilization downstream of the BCR or TCR can be applied to narrow down or to confirm the functional relevance of genetic variants of unknown significance in molecules relevant in TCR- or BCR-mediated Ca^2+^ mobilization. This is especially of diagnostic relevance for the diagnosis of patients with genetic defects in *STIM1*, *ORAI1* but is also relevant in the diagnosis of LAT [1236], LCK, ITK deficiency and others.

### 4 Adoptive B cell transfers as a read-out for Ag-specific immune responses in mice

#### 4.1 Overview

Murine B cell responses are often studied in detail using flow cytometry. For example, activated B cells that differentiate into germinal center B cells downregulate surface IgD expression and instead express GL-7 and FAS or can be stained with the lectin PNA (see Chapter VI, Section 2.2 in [22]). In addition, class-switched Ab isotypes can be measured by flow cytometry. Since B cells recognize their cognate Ag directly through their BCR, Ag-fluorophore complexes can be used to identify Ag-specific B cells by flow cytometry, e.g., 4-hydroxy-3-nitrophenylacetyl (NP) hapten-specific B cells with NP-PE after NP-KLH immunization or hen egg lysozyme (HEL)-APC after HEL immunization. However, it is often advantageous to be able to track the fate of Ag-specific naïve B cells during the entire immune response following activation of these cells. To this end, BCRtg cells can be used in adoptive transfer experiments in a similar way as TCRtg cells (See Chapter Section IV.5 Adoptive T cell transfers as a read-out for Ag-specific immune responses in mice for a detailed description of the experimental procedures).

#### 4.2 Introduction

Several BCRtg mouse lines have been described in the literature. Among them, HEL-specific MD4 [1237], SWHEL [1238], and Hy10 [826] mice as well as NP-specific B1-8 [1239] mice have been used in various studies to dissect the contribution and kinetics of Ag-specific B cell responses *in vivo*. In addition to the possibility of using fluorescently-labeled Ag to directly detect Ag-specific BCRtg cells, homo- or heterozygote combinations of congenic CD45 alleles (i.e., CD45.1 vs. CD45.2) can be used for tracking BCRtg cells in adoptive cell transfer experiments (See Chapter section IV.5 Adoptive T cell transfers as a read-out for Ag-specific immune responses in mice. Alternatively, some BCRtg mice carry different Ig heavy chain (Igh) allotypes that can be used for identification instead. For example, MD4 and Hy10 BCRtg B cells are Igh^a^, which is different as compared to the Igh^b^ background of wildtype C57BL/6 mice. This does not only allow for the identification of these cells by surface or intracellular staining of various Ig isotypes of Igh^a^, but also secreted Abs derived from these cells, which are also of the Igh^a^ allotype, can be measured by ELISA.

BCRtg cells can also be co-transferred together with Ag-specific TCRtg cells to study the cooperation between Ag-specific B and T cells [200]. Examples include co-transfer of OVA-specific OT-II cells and NP-specific B1-8hi cells, followed by immunization with NP-OVA in adjuvants, e.g., alum. If 2D2 TCRtg mice are crossed to the BCRtg mouse line Th [1240], in which approximately 20% of peripheral B cells are specific for MOG, these compound animals can be used as a model for spontaneous EAE development [1241].

#### 4.3 Step-by-step sample preparation

See Chapter section IV.5 Adoptive T cell transfers as a read-out for Ag-specific immune responses in mice

#### 4.4 Materials

For detailed materials see Chapter section IV.5 Adoptive T cell transfers as a read-out for Ag-specific immune responses in mice. The following items are specific to B cell transfers:

- BCR-tg donor mouse lines as well as appropriate recipient mouse lines that carry appropriate combinations of congenic markers
- Appropriate Ags/adjuvants or infectious agents for immunization or infection of recipient mice
- EasySep^™^ Mouse B Cell Isolation Kit (Stemcell Technologies, 19854) or similar for negative selection of B cells

#### 4.5 Data analysis

Flowjo (BD) or alternative software can be used to compensate and analyze the flow cytometry data. For details see Chapter section IV.5 Adoptive T cell transfers as a read-out for Ag-specific immune responses in mice.

#### 4.6 Pitfalls

See Chapter section IV.5 Adoptive T cell transfers as a read-out for Ag-specific immune responses in mice

#### 4.7 Top tricks

See Chapter section IV.5 Adoptive T cell transfers as a read-out for Ag-specific immune responses in mice

## VII Innate lymphoid cell (ILC) phenotypes

### 1 Human Innate Lymphoid Cells

#### 1.1 Overview

This section will give an overview on the flow cytometric strategy to gate on different subsets of innate lymphoid cells (ILCs) derived from tissues in humans. While only gating of ILCs derived from human tonsils is representatively shown, the use of master transcription factors in combination with established surface markers can be generally used across different tissues to identify ILC subsets.

#### 1.2 Introduction

During the past years, an emerging family of CD45^+^ innate lymphoid cells (ILCs) has been described in both mouse and human. CD45^+^ ILCs lack rearranged Ag receptors as well as lineage (Lin) markers typically expressed on T cells, B cells or mononuclear phagocytes [1242]. ILCs can be classified into distinct groups according to the expression of surface markers, transcription factors and effector cytokines (reviewed in Vivier et al., Cell 2018 [1243]). ILC1 express T-box transcription factor T-bet (*Tbx21*) and produce IFN-γ in response to IL-12 and IL-18 or activating receptor engagement, thus contributing to the response against viruses and intracellular pathogens [1244-1247]. In contrast to NK cells, ILC1 do not express Eomes [1246]. ILC2 express GATA binding protein-3 (*Gata3*), produce IL-13 and IL-5 in response to IL-25, IL-33, and thymic stromal lymphopoietin (TSLP) and contribute to the defense against helminthic infections as well as to the pathogenesis of allergic inflammation [1248]. ILC3 express retinoic acid receptor (RAR)-related orphan receptor RORγt, and produce IL-17 and/or IL-22 in response to IL-1β and IL-23 or engagement through activating receptors such as aryl hydrocarbon recptor (AhR) [1249-1251], Toll-like recptors (TLRs) [1252] or NKp44 in humans [1253]. ILC3 include both fetal-derived lymphoid tissue-inducer (LTi) cells (considered as a distinct subset [1243]) and post-natal ILC3; LTi are required for the embryonic development of lymph nodes and Peyer’s patches, while ILC3 contribute after birth to defense against extracellular pathogens, containment of commensals, epithelial tissue homeostasis and regulation of inflammatory disorders, such as inflammatory bowel disease (IBD) and psoriasis [1254]. As such, ILC1, ILC2 and ILC3 show similarities with CD4^+^ T helper (Th) subsets Th1, Th2 and Th17 and mirror the functional analogies between natural killer (NK) cells and adaptive CD8^+^ cytotoxic T lymphocytes (CTL) [1242, 1255]. Accordingly, the International Union of Immunological Societies (IUIS) now recognizes 5 related innate lymphoid subsets: NK cells, ILC1, ILC2, ILC3 and LTi cells [1243].

#### 1.3 Step-by-step sample preparation

Mononuclear cells (MCs) from human tonsils were isolated from patients undergoing tonsillectomy as previously described [1256]. After density gradient centrifugation using Ficoll-Paque PLUS, ILCs were enriched by using magnetic cell depletion of CD3^+^ T cells with anti-CD3 microbeads and LD columns (Miltenyi Biotec) according to the manufacturer’s instructions. Written informed consent was obtained from all patients prior to sample acquisition, experiments have been approved by the Ethics Committee of the appropiate institutional body and conform to all principles of the Declaration of Helsinki.

#### 1.4 Materials

##### Flow cytometry

Phenotypic analysis of human lymphocytes was performed using the Abs reactive to human surface or intracellular Ags listed in Table 92. Dead cells were excluded by staining with Fixable Viability Dye eF780 (LD) (ThermoFisher). Staining for transcription factors was performed using the Foxp3/Transcription Factor Staining Buffer Set (eBioscience) according to manufacturer’s instructions, for 60 minutes at room temperature, and cells were immediately analyzed in PBS containing 0,2% BSA. Flow cytometric analysis was performed by using BD Fortessa employing FACS-Diva Software (BD Biosciences), and data were analyzed by using FlowJo^™^ software (Flow Jo, LLC).

#### 1.5 Data analysis

**Figure 120.**
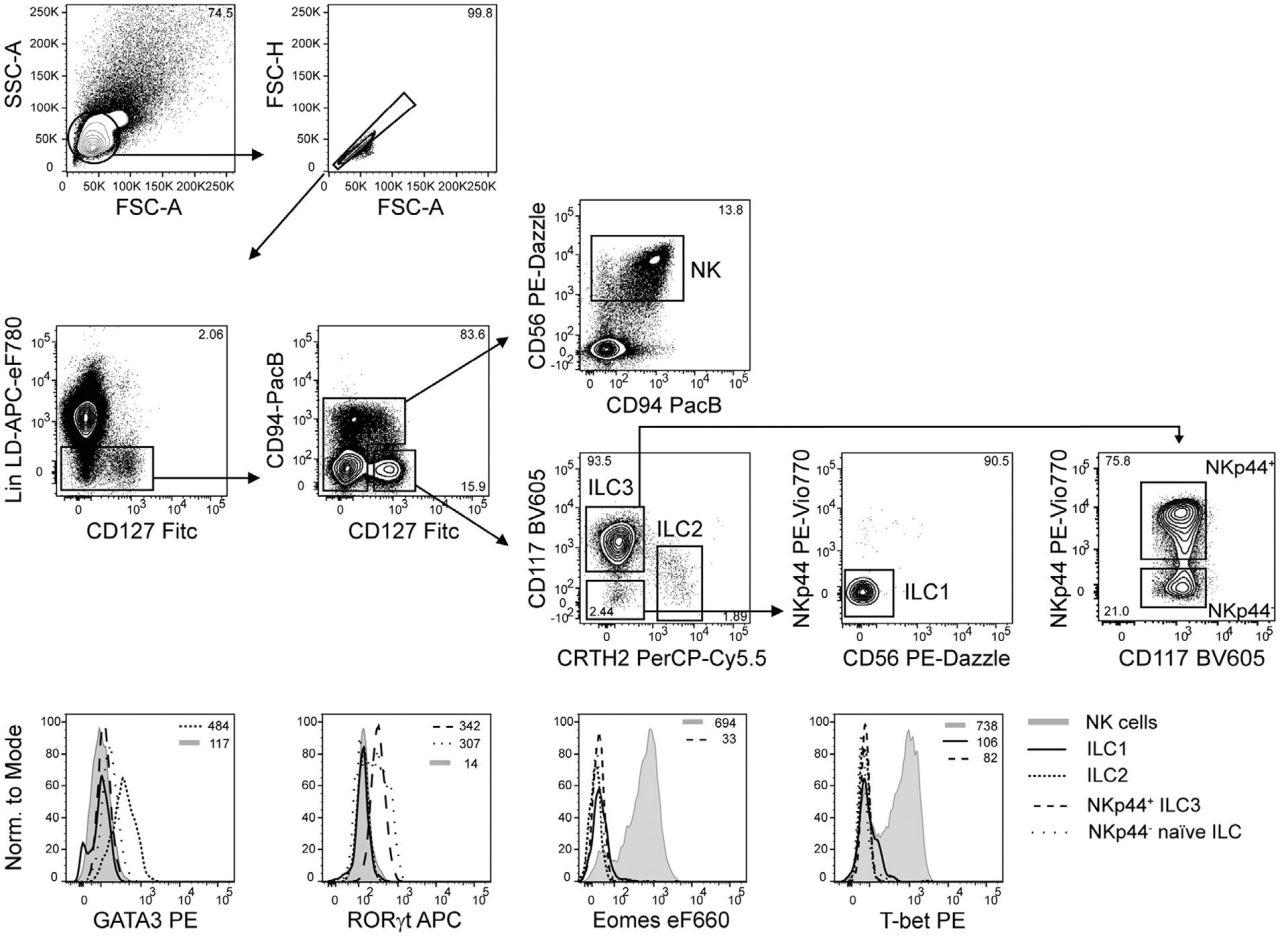
Identification of human tonsil ILCs. Representative gating strategy (upper panel, gate numbers reflect population frequencies) and expression of transcription factors (lower panel, gate numbers reflect MFI of indicated population) of human ILCs derived from tonsillectomy. After magnetic depletion of CD3^+^ cells, cells were gated as viable (LDeF780^−^), CD3(APC-eF780)^−^ CD14(APC-eF780)^−^ CD19(APC-eF780)^−^ FcsRIα(APC-Vio770)^−^ CD123(APC-eF780)^−^ CD11c(APC-Vio770)^−^ BDCA3(APC-Vio770)^−^ (Lin^−^) and either CD94^+/lo^ CD127^−/lo^ CD56^+^ NK cells; CD94^−^ CD127^hi^ CD117^+/lo^ CRTH2^+^ ILC2; CD94^−^ CD127^hi^ CD117^−^ CRTH2^−^ NKp44^−^ CD56^−^ ILC1; CD94^−^ CD127^hi^ CD117^+^ CRTH2^−^ NKp44^+^ ILC3; or CD94^−^ CD127^hi^ CD117^+^ CRTH2^−^ NKp44^−^ ILC.

In humans, ILCs have been documented in several tissues and in the circulation, although a larger characterization has been performed in tonsils, where all ILC subsets have been described [1257-1260]. In tonsils, magnetic depletion of CD3^+^ T cells and of CD19^+^ B cells is recommended for better detection of ILCs, due to their low frequency. In cases where a substantial CD3^+^ T cell population remains in the sample, separation of surface CD3 from the lineage channel is recommended, to increase lineage resolution and decrease chances of T cell contamination in the subsequent ILC gating. After pre-enrichment and further gating on lineage negative cells, staining of CD94 (alternatively, NKG2A), CD56 and CD127 enables the identification of NK cells as CD56^+^ CD94^+/lo^ CD127^neg/lo^ cells, which express high levels of T-bet and Eomes, and of other ILCs enriched among Lin^−^ CD127^hi^ CD94^−^ cells (Figure 120). Analysis of human NK cells is also described in the relative NK chapter, where readers can find more details (See section VIII.1 Human Natural Killer (NK) cells).

It has been proposed that staining of CD117 (the receptor for stem cell factor, c-kit) and CRTH2 (prostaglandin D2 receptor chemoattractant receptor-homologous molecule expressed on T helper type 2 cells) facilitates identification of ILC3 and ILC2 in tonsils [1261]. ILC3 are enriched among CD117^+^ CRTH2^−^ cells and express NKp44 and RORγt, while lacking T-bet and Eomes [1260, 1262]. ILC2 are enriched among CD117^−/lo^ CRTH2^+^ cells and express GATA-3, while lacking T-bet and Eomes (Figure 120) [1258, 1260]. Among Lin^−^ CD127^hi^ CD94^−^ CD117^−^ CRTH2^−^ cells, a population of ILC1 has been described which lacks NKp44 and CD56 and is enriched in the SI LP of patients affected with inflammatory bowel diseases [1260]. This population however displays only low amount of T-bet protein expression (Figure 120). In line with mouse data, additional populations of NK cells/ILC1 subsets with different phenotypic characteristics have been described in human tissues, including tonsils [1259, 1263-1266], making the selection of markers for the identification of NK/ILC1 quite challenging.

Recently, a human CD117^+^ NKp44^−^ ILC subset was identified in peripheral blood and in tissues that represents a ‘naïve’ ILC precursor [1267-1271]. This population was able to give rise to all ILC subsets *in vitro* and *in vivo* and did not express signature transcription factors associated with mature ILCs (T-bet, Eomes, GATA-3, RORγt). Accordingly, CD117 staining on human ILCs should not be equated with ILC3 identification, but should additionally include markers such as RORγt or NKp44.

Notably, the resolution of transcription factor staining in humans is not as good as in murine tissues and, therefore, combined staining of the above-mentioned surface markers is highly recommended in order to reliably gate on different human ILC subsets. However, as for their murine ILC counterparts, tissue-specific differences of surface markers should be taken into account as it has been shown for expression of CRTH2 for lung ILC2 [1270, 1272, 1273]. A selection of markers shown to be expressed by human ILC subsets is depicted in Table 93 (see also ref. [1243]).

#### 1.6 Pitfalls and top tricks

- ILCs can be enriched by depletion of CD3^+^ T cells and CD19^+^ B cells in tissues such as tonsil and peripheral blood, as recommended to increase ILC frequencies and decrease T cell contamination.
- Staining CD3 separately from the lineage can also increase resolution and decrease T cell contamination in the ILC gating.
- As human TF staining is not as high resolution as in mice, a minimum set of surface markers such as CD94, CD127, CD117 and CRTH2 can help to discriminate NK, ILC1, ILC2, and ILC3 subsets.

#### 1.7 Clinical relevance statement

The gating strategy shown in this section is applicable for analysis of human PB ILCs of healthy individuals as well as in chronically inflammed tonsil samples, as depicted in Figure 120 and as described by Krabbendam et al. [1274].

#### 1.8 Summary of the phenotypes

This is detailed in Table 93 (with refs. [1275-1294].

### 2 Murine Innate Lymphoid Cells

#### 2.1 Overview

This section will give an overview on the flow cytometric strategy to gate on different subsets of innate lymphoid cells (ILCs) derived from tissues in mice. While only gating of ILCs derived from the murine small intestine lamina propria is representatively shown, the use of master transcription factors in combination with established surface markers can be generally used across different tissues to identify ILC subsets.

#### 2.2 Introduction

See above VII.1 Introduction human Innate Lymphoid Cells.

#### 2.3 Step-by-step sample preparation

For isolation of murine small intestinal lamina propria (SI LP) cells a previously described protocol was used [1294]: residual fat tissue, Peyer’s Patches and feces were removed, and the intestine was cut open longitudinal and washed with PBS. After clearing, tissue was cut into pieces of 1 cm length and digested with a lamina propria dissociation kit (Miltenyi Biotec), according to the manufacturer’s instructions. In short, two rounds of incubation at 15 min at 37°C to dissociate epithelial cells using Hank’s balanced salt solution without calcium and magnesium (HBSS−/–, Gibco) supplemented with 2% FCS, 10 mM HEPES buffer (Sigma), 1 mM DTT (ThermoFisher) and 5 mM EDTA (Sigma) were performed. After washing with HBSS−/−, tissues were digested using the enzymes provided according to the manufacturer’s protocol. Lymphocytes were further enriched on a 40%/80% Percoll^®^ (GE Healthcare) gradient. Animal experiments were performed in accordance with both institutional and national/international guidelines and regulations and approved by the correspondant body. The tissue dissociation reagents used for this protocol are listed in Table 94.

#### 2.4 Materials

##### Flow cytometry:

Phenotypic analysis of murine lymphocytes was performed using the following Abs reactive to murine surface or intracellular Ags listed in Table 95. To minimize non-specific binding of Abs, cells were blocked with anti-mouse CD16/32 (2.4G2) and dead cells were excluded by staining with Fixable Viability Dye (LD) (ThermoFisher) prior to Ab labeling.

Staining for transcription factors was performed using the Foxp3/Transcription Factor Staining Buffer Set (eBioscience) according to manufacturer’s instructions, specifically cells were fixed for 60 min at 4°C, and cells were immediately analyzed in PBS containing 0,2% BSA. Flow cytometric analysis was performed by using BD Fortessa employing FACSDiva Software (BD Biosciences), and data were analyzed by using FlowJo^™^ software v10.6.2 (FlowJo LLC).

#### 2.5 Data analysis

**Figure 121.**
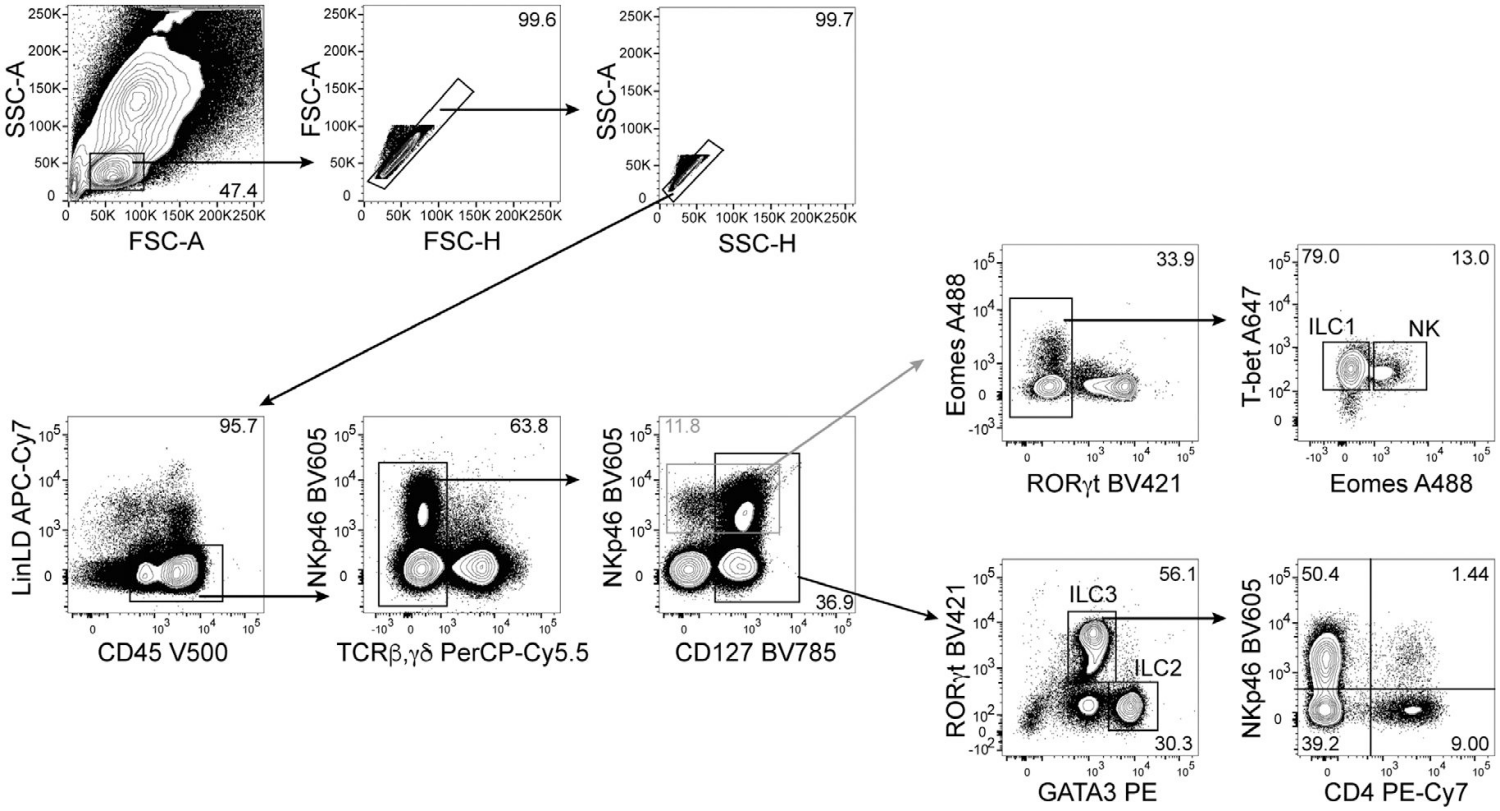
Identification of murine SI LP ILCs. Representative gating strategy of ILCs derived from the SI LP of 6-week-old C57BL/6 mice. Mononuclear cells (MCs) were prepared as previously described [1294]. Cells were gated as viable (LD, eF780^−^), B220(APC-Vio770)^−^ CD11c(APC-Cy7)^−^ Gr-1(APC-Fire780)^−^ F4/80(APC-Fire780)^−^ Fc√R1α(APC-Cy7)^−^ (Lin^−^) CD45^+^ TCRβ^−^ TCRγδ^−^ and either as NKp46^+^ (grey gate, A) T-bet^+^ Eomes^−^ ILC1, Eomes^+^ T-bet^+^ NK cells or as CD127^+^ (black gate, B) GATA3^+^ RORγt^−^ ILC2 and RORγt^+^ GATA3^lo^ ILC3 which can be further separated according to NKp46 and CD4 expression (B). Gate numbers reflect population frequencies.

ILCs are present in diverse organs as tissue resident cells but are also detected in the circulation [1247, 1295]. In mouse small intestinal (SI) lamina propria (LP), all ILCs, namely NK cells, ILC1, ILC2 and ILC3 can be discriminated [1246, 1288]. In Figure 121, a gating strategy for murine ILCs derived from SI LP is shown; however, it should be stressed that ILC populations are not equally distributed in all organs and display some tissue-specific phenotypic differences. Combination of intranuclear staining of lineage defining transcription factors, namely T-bet (expressed on ILC1, NK cells and a subset of murine ILC3), Eomes (NK cells), RORγt (ILC3) and GATA3 (ILC2) together with NKp46 and CD127 (IL-7Rα) (Figure 121) or CD90 (not shown) enables identification of ILC subsets in all organs analyzed. Among SI LP CD45^+^ Lin^−^ cells, ‘NK cell markers’ (such as NKp46 or NK1.1) can be expressed not only on NK cells but also on ILC1 and a subset of ILC3. Thus, staining of transcription factors is helpful to dissect their identity (See also Chapter V Biological applications Section 13 Transcription factors in [22]). It has been proposed that SI LP NK cells can be defined as NKp46^+^ RORγt^−^ T-bet^+^ Eomes^+^ cells, while ILC1 are NKp46^+^RORγt^−^ T-bet^+^ Eomes^−^ cells [1246] (Figure 121). However, a population of cytotoxic NKp46^+^ RORγt^−^ T-bet^+^ Eomes^+^ intraepithelial ILC1 has been also described [1259]. Moreover, the analysis of NK/ILC1 in different mouse compartments revealed a high degree of phenotypic and functional complexity [1247, 1296, 1297], suggesting that distinction between NK and ILC1 cells might be more challenging than initially imagined. Analysis of murine NK cells is also described in the NK chapter, where readers can find more details (See Section VIII.2 murine Natural Killer (NK) cells).

ILC2 and ILC3 are enriched among SI LP CD45^+^ Lin^−^ CD127^+^ lymphocytes and can be identified after intranuclear staining of GATA3 and RORγt as GATA3^hi^ RORγt^−^ ILC2 and of GATA3^lo^ RORγt^+^ ILC3 (Figure 121) [1288,1289]. Surface markers such as ST2 (IL-33R), CD25, ICOS or KLRG1 have also been commonly used to identify ILC2 [1285, 1286, 1288]. As previously mentioned, expression of these markers slightly varies in different compartments.

SI LP RORγt^+^ ILC3 can be dissected into three major subsets according to NKp46 and CD4 expression (Figure 121), namely CD4^+^ ILC3, which functionally and phenotypically resemble fetal LTi and preferentially produce IL-17 and IL-22; NKp46^+^ ILC3, which expand post-natally, co-express RORγt and T-bet and produce IL-22 and IFN-γ; and CD4^−^ NKp46^−^ ILC3, which actually represent a heterogeneous population of CCR6^+^ cells (related to LTi) and CCR6^−^ ILC3, co-expressing RORγt and T-bet, similar to NKp46^+^ ILC3 [1290-1293]. As it has been shown that ILC3 can be plastic *in vivo*, and down-regulate RORγt expression while acquiring NK/ILC1-cell features such as T-bet expression and IFN-γ production, the use of RORγt fate mapping (RORγt^fm^) can be helpful to distinguish ex-ILC3 (RORγt^fm+^ RORγt^−^ T-bet^+^) from ILC1 [1292, 1293]. Although this distinction is conceptually important, ex-ILC3 behave functionally similar to NK/ILC1 cells. A selection of markers shown to be expressed by murine ILC subsets is depicted in Table 96.

#### 2.6 Pitfalls and top tricks

- For analysis of murine ILCs across tissues, staining for transcription factors is highly recommended since a bimodal staining enables exact identification of distinct ILC subsets.
- Surface markers, such as CD127 or CD25, may vary across tissues or can be affected by enzymatic digestion.
- Genetically modified fate map mice facilitate the identification of ex-ILC3.

#### 2.7 Summary of the phenotypes

This is detailed in Table 96 (with refs [1298-1301].

#### 2.8 Key information human vs murine

This is detailed in Table 121 and summarized here:

- As human TF staining is not as high resolution as in mice, a minimum set of surface markers such as CD94, CD127, CD117 and CRTH2 can help to discriminate NK, ILC1, ILC2, and ILC3 subsets in tissues such as tonsil and peripheral blood.
- CD127, NKp46 together with staining for transcription factors GATA3, RORγt, T-bet and Eomes can identify all ILC subsets in mice.
- In contrast to mouse, human NCR^+^ (NKp44^+^) ILC3 do not co-express T-bet *ex vivo*.
- Unlike in mice, CCR6 cannot be used as a marker to define LTi cells in humans as NCR^+^ ILC3 subsets can express it.
- While in mice, a clear ILC1 population expressing T-bet but lacking Eomes is present, CD127^+^ ILC1-like cells in humans have been characterized by lack of CD56 and NKp44 expression and variable T-bet levels, and can vary across tissues.
- NKp44 and NKp30 homologous loci are not found in *Mus musculus*.
- Variations across phenotypes and tissues exist for murine but also human ILC subsets.

## VIII Natural killer (NK) cell phenotypes

### 1 Human NK cells

#### 1.1 Overview

NK cells represent a first fundamental line of defense against tumors and virus infected cells. In this section, we describe human NK cells, the most important strategies used to isolate and identify their subpopulations in an unequivocal manner.

#### 1.2 Introduction

Natural killer (NK) cells were described over 40 years ago as cells capable of killing tumor cells without prior sensitization. They are lymphoid cells derived from hemopoietic stem cells (HSCs) [1302] and belong to the innate immunity cell family. In contrast to T and B cells, NK cells do not express receptors encoded by rearranging genes and they play a major role in innate immunity as both effector and regulatory cells, participating in the first line of defence against pathogens and tumors. Notably, NK-cell-susceptible tumors are primarily those lacking or expressing insufficient amounts of MHC class I molecules (missing-self hypothesis) [1303]. Another requirement for NK-cell-mediated tumor cell killing is the surface expression of a series of different stress-induced structures [1304]. The NK cell function appears to complement the cytolytic T cell-mediated MHC-I-dependent activity [1305].

The recognition of MHC class-I is mediated by a family of receptors termed killer Ig-like receptors (KIRs), by the NKG2A/CD94 heterodimer and by LIR-1 (CD85j). In particular, NKG2A/CD94, expressed early during the process of NK cell maturation, recognizes the non-classical HLA-E molecule [1306, 1307] while KIRs, expressed at later stages of NK cell maturation, recognize allelic determinants of HLA-A -B or -C [1308, 1309]. Other non-HLA-related inhibitory receptors including Siglec7 (CD328), PD1 (CD279) and IRP60 (CD300a) may be expressed at the surface of NK cells (see Table 97 and 98) [1310]. In most instances, the NK receptors that mediate their activation upon binding to target cells are non-HLA-specific and recognize cell stress-induced molecules. These receptors include NKp30, NKp44 and NKp46 (which constitute the natural cytotoxicity [NCR] family), NKp80, 2B4 (CD244), NKG2D and DNAM1. In addition, NK cells express CD16 (FcγRIIIA), which is responsible for Ab-dependent cellular cytotoxicity (ADCC) [1311-1313]. Of note, activating isoforms of KIRs also exist [1314]. While inhibitory KIRs are characterized by immune-receptor tyrosine-based inhibition motif (ITIM) domains in their long intracytoplasmic tail, the various activating receptors bear a short intracytoplasmic tail and are associated with signalling polypeptides containing immune-receptor tyrosine-based activating motifs (ITAM) domains [1315]. Exceptions are NKG2D, which associates with the YINM-containing adaptor DAP10, and 2B4 and NKp80, which signal trough a ITSM and a hemITAM motive respectively [1316].

Among peripheral NK cells, two major subsets have been identified on the basis of the cell surface density of CD56 molecules (neural cell adhesion molecule, N-CAM). CD56^bright^ (CD3^−^CD56^++^CD16^−/+^) represent approximately 10% of the circulating PB NK cells while they prevail in secondary lymphoid organs (liver, synovial fluid, and decidua). CD56^dim^ (CD3^−^CD56^+/−^ CD16^++^) cells are largely predominant (~90%) in PB NK cells. They derive from CD56^bright^ NK cells, as revealed by different studies in vitro (differentiation from HSC) and in vivo after HSC transplantation [1317, 1318].

##### • CD56^bright^ NK CELLS

All CD56^bright^, in contrast to CD56^dim^, NK cells express both high (CD25) and intermediate (CD122/CD132) affinity IL-2 receptors and c-Kit (CD117), rendering them highly susceptible to IL-2–induced cell proliferation [1319, 1320]. Moreover, CD56^bright^ NK cells express high levels of both CD62L [1321] and CXCR3 which, together with the surface expression of CCR7, dictates their preferential homing into secondary lymphoid organs [1322-1324]. Notably, although under resting conditions, CD56^bright^ NK cells are poorly cytotoxic, they may acquire cytolytic activity comparable to that of CD56^dim^ cells upon stimulation with cytokines, such as IL-2, IL-12, IL-15. While CD56^bright^ NK cells express CD94/NKG2A (i.e., the receptor for HLA-E) they lack KIRs. Regarding activating NK receptors, CD56^bright^ cells express higher levels of NKp46 and NKp30 than CD56^dim^ cells, while CD56^bright^ cells lack or express low amounts of CD16.

##### • CD56^dim^ NK CELLS

**Figure 122.**
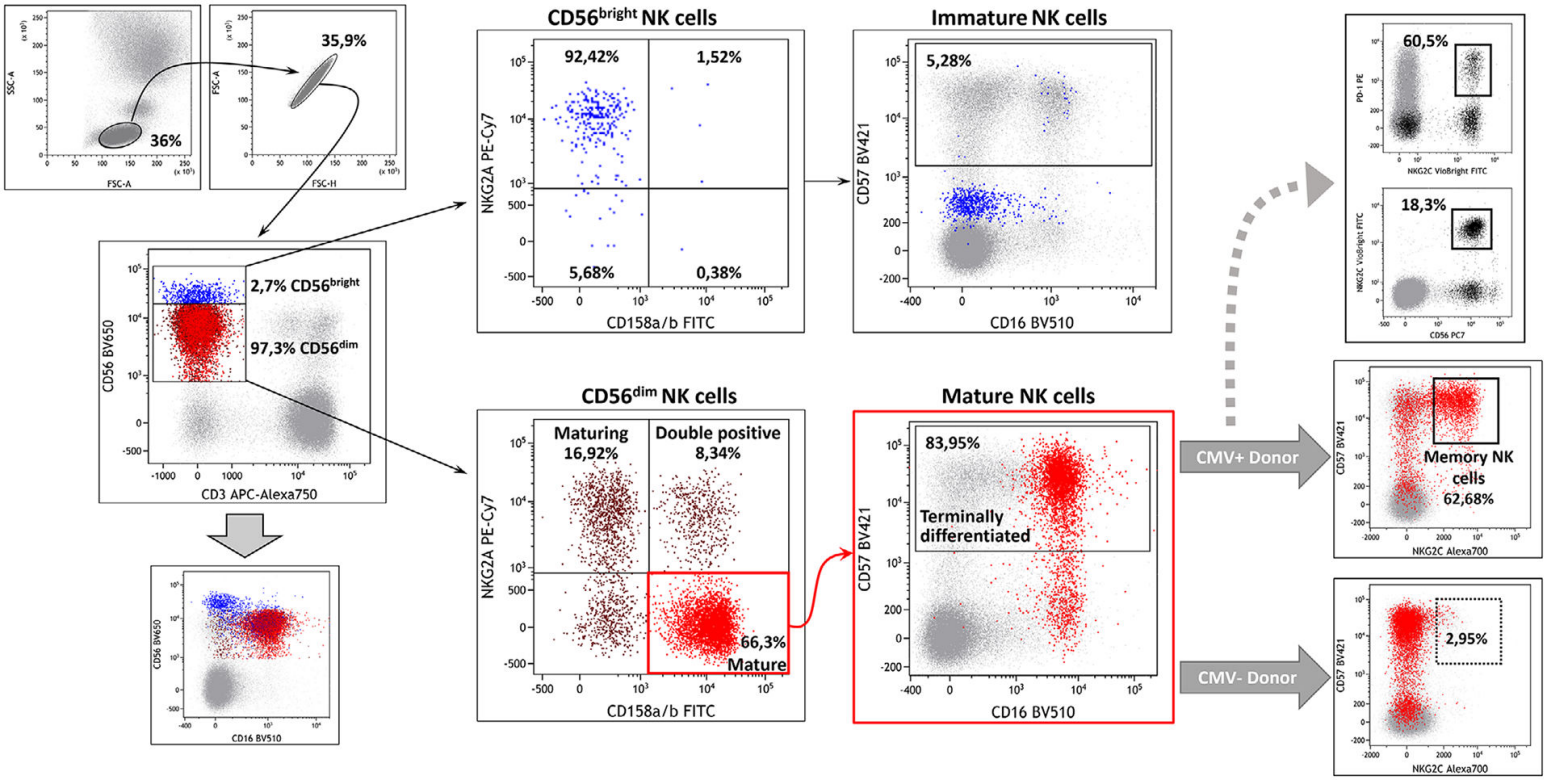
Identification of human NK cells and NK cell subsets in the PB of a healthy donor. In this PB samples, lymphocytes are first gated based on their physical parameters (upper left grey dot plot) than Human NK cells can be first gated on the basis of identified for their surface level of CD56 surface expression and lack of CD3. The CD56^bright^ NK subpopulation (in blue) is positive for NKG2A, negative for KIRs and CD57 while CD16 can be either negative or dimly expressed (as shown). NKG2A and KIR surface expression allows three subpopulations of CD56^dim^ NK cells (in red), namely “maturing” (NKG2A+KIR−) in dark red), “double positive” (NKG2A+KIR+ in dark red) and “mature” (NKG2A-KIR+ in light red), to be identified. To discriminate among these CD56^dim^ maturation steps, we used a cocktail of anti-KIR (clones: EB6B, GL183, Z27) that did not include anti-LIR1, for this reason in the dot plot also a double negative population is present. Among the mature population (in light red), CD57 molecule is expressed on the, so called, “terminally differentiated” NK cells. In CMV positive donors, a percentage of this latter population can also express NKG2C representing the so called “memory NK cells”. Recently it has been demonstrated that in some CMV positive individuals a fraction of the NKG2C subset can also express PD1. Percentage of subpopulation are not specified because they are extremely diverse among different individuals and do not give additional information to the gate strategy. Total PBMCs are shown as a light grey population to highlight how they are expressed in each dot plot, while percentages shown for each gate refer to coloured NK cells only.

CD56^dim^ NK cells under resting conditions express granules containing perforin and granzymes, and display cytolytic activity. Until recently, CD56^dim^ NK cells were mainly associated with cytotoxicity while cytokine production was thought to be confined to the CD56^bright^ subset. However, more recently, it has been shown that, upon stimulation via activating receptors, CD56^dim^ NK cells rapidly release cytokines such as IFN-γ- and TNF-α (even more efficiently than CD56^bright^ cells) and chemokines such as MIP-1β and MIP-1α [1325, 1326]. In contrast to CD56^bright^ NK cells, the CD56^dim^ population is phenotypically heterogeneous. Thus, as shown in Figure 122, NKG2A versus KIR expression allows three distinct subsets that recapitulate the consecutive steps of PB NK cell maturation to be distinguished.

The “maturing” population (NKG2A^+^KIR^−^) is characterized by the NKG2A^+^/KIR^−^ phenotype, similar to that of CD56^bright^ cells, while the “mature” population expresses the NKG2A^−^KIR^+^ phenotype. An intermediate step of maturation is identified by the “double positive” NKG2A^+^KIR^+^ cells [1278, 1327]. The unidirectional nature of NK cell differentiation is further supported by the presence of CD57 on the surface of the “terminally differentiated” NK subset. When compared with the CD57-negative counterpart, the NKG2A^−^KIR^+^CD57^+^ population shows a decreased surface expression of NKp30 and NKp46, and a reduced proliferative potential, possibly as the result of downmodulation of IL-2Rβ (CD122) and IL-18Rα (CD218a) [1278, 1280].

In CMV-positive healthy donors it is possible to find an additional subset of mature cells that expresses CD57 and the activating HLA-E-specific receptor NKG2C dimerizing with CD94 [1328]. This subset appears to contain cells endowed with an adaptive/memory-like capability (i.e., clonal expansion, prompt response to restimulation and epigenetic modification including that of the intracytoplasmic FcεRγ chain) [1329-1331]. Recent data have shown that, in CMV positive individuals, a fraction of CD57 positive cells may also express PD-1 [1332]. In addition, it was demonstrated that CD57 positive and NKG2C positive NK cells are the predominant ADCC effector subsets capable of targeting HIV-infected CD4^+^ cells in the presence of 3BNC117 and 10-1074 immunotherapy [1333].

The recruitment of CD56^dim^ NK cells to inflamed peripheral tissues is driven by several chemokines and homing receptors including, for example, CXCR1, CX3CR1 and in certain subsets CD62L and CXCR3^low^ also [1322].

##### • NK cells present in decidua

During the first trimester of pregnancy, NK cells represent the main lymphoid population (50–70%) in human decidua where they bear a unique phenotypic and functional profile. Their phenotypic features resemble to an extent those of CD56^bright^ PB NK cells; however, in addition to the NKG2A^high^NKp30^high^NKp46^high^ surface phenotype, they also display characteristics of CD56^dim^ NK cells including high expression of KIR and lytic granules. Of note, in contrast to PB NK cells, the 2B4 (CD244) receptor on decidual NK cells displays a strong inhibitory (and not activating) activity, similar to that seen in NK cell precursors [1334], that renders this population poorly cytolytic [1335, 1336]. Moreover, in contrast to PB NK cells, decidual NK cells release a unique set of cytokines, including IL-8 (CXCL8), VEGF, CXCL12 (stromal-derived factor-1 [SDF-1α]), and IFN-γ-inducing protein 10 (IP-10, CXCL10), that play a pivotal role in tissue remodelling (i.e., placenta development processes) and neo-angiogenesis [1337].

##### • NK cells present in lymph nodes

In normal conditions, NK cells are present in lymph nodes where they occupy the T-cell areas [1338]. They are consistently CD56^bright^CD16^neg^KIR^neg^ and lack perforin and granzymes. In contrast to PB CD56^bright^ NK cells, lymph node NK cells do not express CCR7 or CD62L. Concerning the NCR family, lymph node NK cells express low levels of NKp46 and may lack NKp30. Remarkably, however, upon IL-2 activation, lymph node NK cells may express KIRs and CD16, and upregulate NCR [1338, 1339].

#### 1.3 Step-by-step sample preparation

All the protocols for T cells described in Section III T cells, can be applied to the analysis of NK cells, and NK cell characterization is also described in Section VII Innate lymphoid cells. Regarding the effector function and expression of chemokine receptors, that which is already described for T cells is also true for NK cells.

For the quick screening of whole blood NK cells, we suggest the following surface staining:

Put 100 μL of heparinized whole blood in a sample tube
Add 50 μL of Brilliant Stain Buffer to each tube
(a) To study PB subpopulations, add the following Abs: CD158a FITC (10 μL) CD158b FITC (10 μL), CD158e FITC (5 μL), CD279 PE (10 μL), CD159a PE-Cy7 (3 μL), NKG2C A700 (3 μL), CD3 APC-A750 (3 μL), CD57 BV421 (3 μL), CD16 BV510 (3 μL), CD56 BV650 (3 μL).
  (b) To study only NKG2C/PD1 co-expression this simpler cocktail can be used: NKG2C VioBright FITC, CD279 PE (10 μL), CD56 PC7 (3 μL) CD3 APC-A750 (3 μL).
Incubate 20 min at 4°C.
After incubation, lyse sample in 2 mL of Pharm Lyse^™^, for 5–8 min
Centrifuge 5 min at 1300 rpm
Discard supernatant
Resuspended in 300 μL of PBS for acquisition.

#### 1.4 Materials

Pharm LyseTM and Brilliant Stain buffer are from Becton Dickinson (San José, CA), PBS is from (Sigma-Aldrich).

##### Beckman Coulter:

CD3 APC or APC-Alexa Fluor 750 (UCHT1, IgG1), CD56 PC7 (N901), CD158a PE (EB6B, IgG1), CD158b PE (GL183), CD158e FITC or PE (Z27, IgG2a), CD159a PE-Cy7 (Z199 IgG2b), NKp30 (Z25, IgG1), NKp44 (Z231, IgG1) NKp46, (BAB281, IgG1), NKp80 (MA152, IgG1) NKG2D (ON72, IgG1).

##### Becton Dickinson:

CD16 BV510 (3G8, IgG1), CD56 BV650 (NCAM16.2, IgG2b), CD57 BV421 (NK-1, IgM), CD158b (CH-L, IgG2b).

##### Miltenyi:

PD1 PE (PD1.3.1.3, IgG1), NKG2C VioBright FITC (REA205, Ig1).

##### R&D System:

NKG2C Alexa Fluor 700 (134591, IgG2a).

Flow cytometric data were acquired with a (three laser: 405nm, 488nm and 633nm) BD LSR II flow cytometer equipped with FACS DIVA software (BD Biosciences), and analyzed by using Kaluza software (Beckman Coulter)

#### 1.5 Data analysis

NK cells, together with the other lymphocytes, are gated as small cells with a low granularity compared to monocytes and granulocytes. Cells with a constant FSC-A/FSC-W ratio are gated as single cells. Live/Dead staining is not required for fresh whole blood staining. NK cells are defined as CD56 positive, CD3 negative cells then, among CD56^dim^CD16^bright^ NK cells, the use of anti-NKG2A and anti-KIR Abs allows the discrimination of three different subsets: maturing NK cells (NKG2A^+^KIR^−^), Double Positive (NKG2A^+^KIR^+^) and mature NK cells (NKG2A^−^KIR^+^). Within this latter population, CD57, NKG2C and PD-1 expressions have been evaluated. In Figure 122, for a better viewing of positive and negative populations, axes are displayed in a logical (or biexponential) scale. In each dot plot, the percentage of cells refers to the colored population (es: blue for the immature population). Light grey dots are used to show how the other lymphocytes are distributed in each dot plot. We added also the CD56 vs CD16 dot plot to show how NK cells distribution looks like using these two fundamental NK cell markers. Obviously, the percentages are identical to those in the plot CD56 vs CD3.

#### 1.6 Pitfalls

It is important to keep in mind, when working with unfrozen NK cells, that after thawing some important receptors can be down-modulated or shed (es: CD16 and PD-1).

##### 1.7 Top tricks

When analyzing PD-1 on NK cells, the choice of the Ab clone is extremely important. Differently from what happens with T cells, where most of anti-PD-1 clones work well, the only clone always able to discriminate the PD-1 positive NK cells is the PD1.3.1.3 clone [1327].

#### 1.8 Clinical relevance statement

The gating strategy shown in this section is applicable for monitoring by flow cytometry the reconstitution of circulating NK cell compartment in hematopoietic stem cell transplantation (HSCT) recipients. Early and efficient NK cell reconstitution after allogeneic HSCT has indeed a favourable role on the clinical outcome [1340]. Therefore, determination of NK cell subset reconstitution is helpful for stratifying the risk of chronic Graft versus Host Disease, treatment-related mortality and CMV reactivation. In particular, CMV exposure results in expansion of a unique subset of terminally differentiated “adaptive” NKG2C+CD57+ NK cells that have potent Ab-dependent cytolytic function [1341] and have been associated with protection against leukemia relapse and improved disease-free survival [1342].

#### 1.9 Summary of the phenotypes

This is detailed in Table 97 and 98.

### 2 Murine NK cells

#### 2.1 Overview

NK cells represent a first fundamental line of defense against tumors and virus infected cells. In this section, we describe murine NK cells, the most important strategies used to isolate and identify their subpopulations in an unequivocal manner.

#### 2.2 Introduction

**Figure 123.**
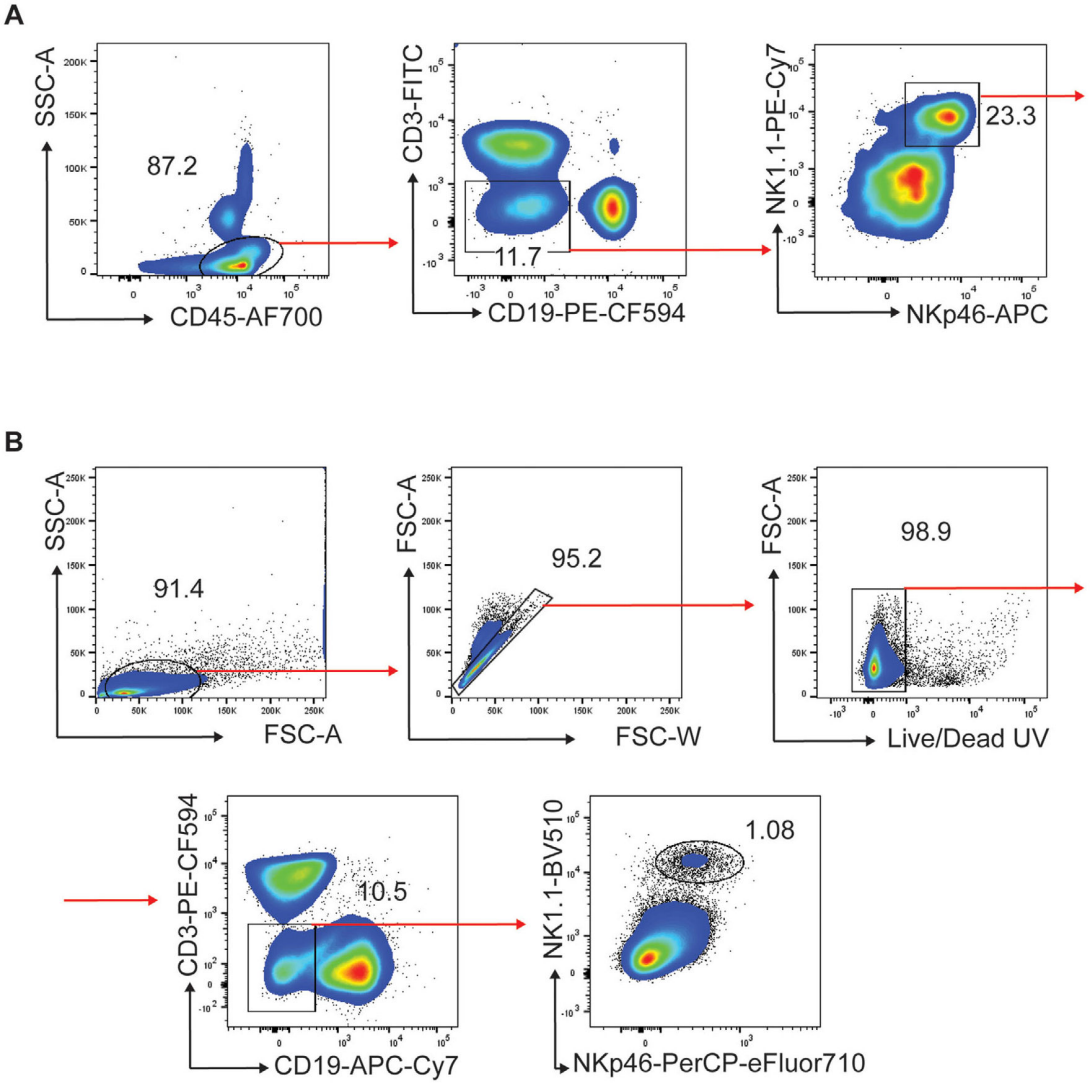
Identification of murine NK cells in the blood and spleen of C57B/6 mice. Whole blood (A) was stained in BD Trucount tubes and analyzed after red blood cell lysis. Lymphocytes were gated among CD45^+^ leucocytes based on their morphology and, after exclusion of CD3^+^ T cells and CD19^+^ B cells, NK cells were gated as NK1.1+NKp46+ cells. For the analysis of spleen NK cells (B), due to extraction techniques, doublets and dead cells need to be gated out. CD3^+^ T cells and CD19^+^ B cells were excluded, and NK cells were gated as NK1.1+NKp46+ splenocytes.

Mouse NK cells are commonly identified by flow cytometry by the expression of the surface markers NK1.1, NKp46 and CD49b. The lack of expression of the T cell marker CD3 is used to exclude from the NK cell gate contaminating T cell subsets, such as NKT cells and NK-like T cells, that express NK1.1 and NKp46 respectively [1343]. In blood and spleen NK cells represent the most abundant innate lymphoid cell (ILC) subset, and the expression of NKp46 and NK1.1 is sufficient to identify them (Figure 123). However, these NK markers vary depending on the mouse strain. NK cells from C57B/6 and SJL mice can be identified by NK1.1 expression, while in other mouse strains, such as BALB/c, NK cells display no reaction to the widely used anti-NK1.1 Ab PK136, because of allelic variations in Nkrp1b and Nkrp1c [1344]. In this case, NK cells can be identified only with CD49b and NKp46.

Even if mouse NK cells share many characteristics with human NK cells, it is not easy to identify functionally comparable NK cell subpopulations in the two species. Indeed, mouse NK cells lack the expression of human NK cell surface markers, including CD56 and some activating and inhibitory receptors. Murine NK cells lack KIRs but express structurally divergent lectin-like Ly49 receptors that are functionally equivalent to the human KIRs and recognize MHC class I molecules. Most mouse Ly49 receptors recognize the classical MHC class I molecules H2-K and −D/L, while Ly49H and Ly49I recognize the MHC class I-related m157 molecule encoded by cytomegalovirus (CMV). The CD94/NKG2 heterodimer is conserved between mouse and human and, in mice, it recognizes the non-polymorphic Qa-1. The activating receptor NKG2D is also conserved between the species, and it is triggered by stress-induced MHC class I-related ligands retinoic acid early inducible (RAE)-1 and, in mice, the minor histocompatibility complex H60. Among the natural cytotoxicity receptors (NCRs), NKp30 and NKp44 are not expressed in mice, while NKp46 is considered to be the most specific NK cell marker, as it is expressed by all NK cells in mammals (Table 99) [1343].

Analogously to human NK cells for which the levels of CD56 and CD16 expression are used to define the maturation from immature CD56^bright^ CD16^−^ NK cells to mature CD56^dim^ CD16^bright^ cells [1316, 1327], CD27 and CD11b expressions are used to identify several murine NK cell maturation steps. Immature NK cells are CD11b^low^ CD27^high^, then they mature into double-positive CD27^+^CD11b^+^ cells and, finally, into fully mature CD27^low^ CD11b^high^ NK cells (Table 100, see below). This developmental program is associated with the acquisition of NK cell effector functions [1277]. Both CD27^+^ and CD27^−^ subsets express equivalent levels of activating Ly49 receptors and CD94/NKG2 receptors, but CD27^−^ NK cells contain higher levels of inhibitory Ly49s.

Using high-throughput single-cell-RNA-seq, the gene expression of human and murine NK cells from spleen and blood was analyzed at the single cell level. In this study two major NK cell subsets transcriptionally similar across organ and species were identified: it was shown a correspondence between the CD27^−^CD11b^+^ and the CD27^+^CD11b^−^ mouse NK cell subsets and the CD56^dim^ and CD56b^right^ human NK cell subsets, respectively [1345].

**Figure 124.**
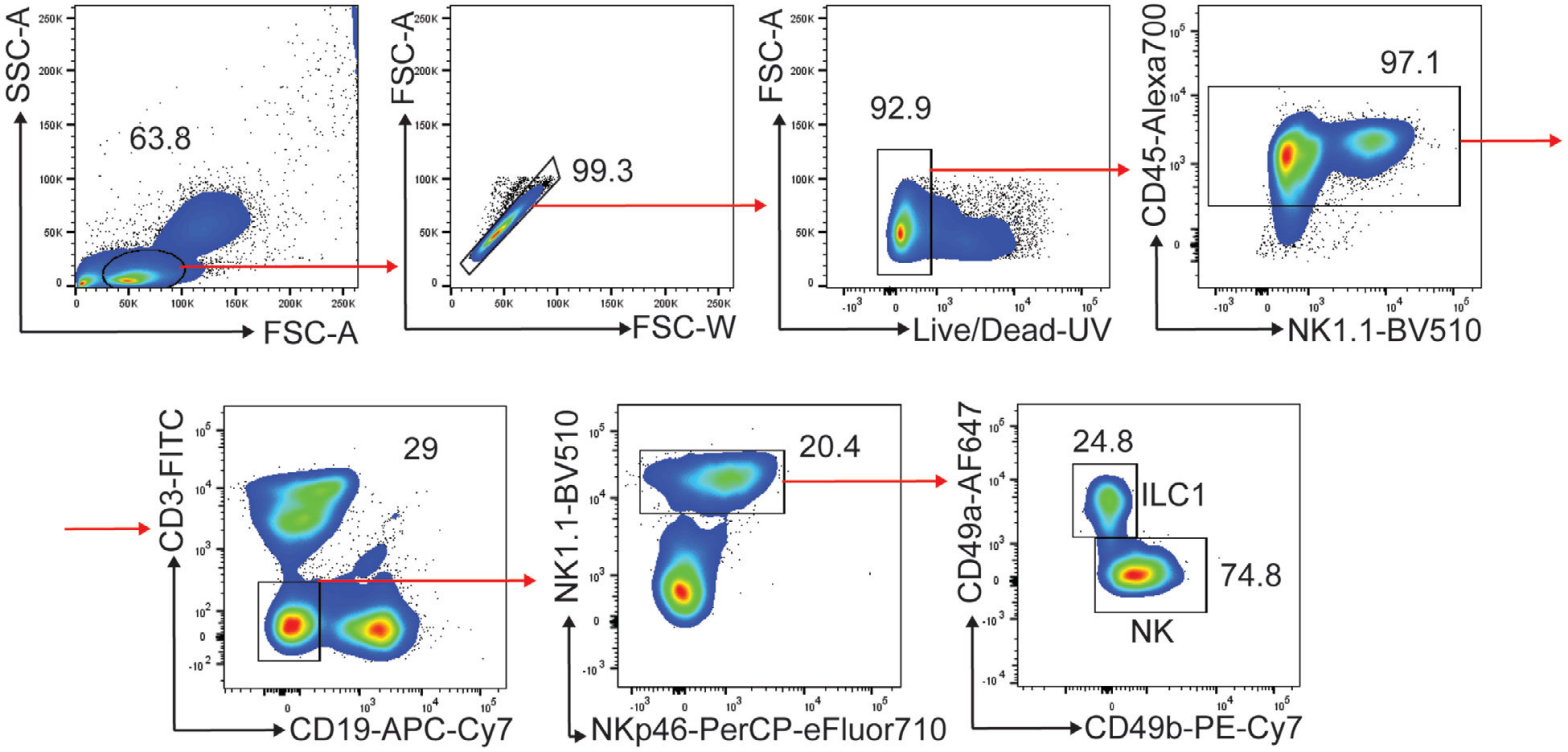
Identification of murine liver NK cells in C57B/6 mice. After Percoll density gradient centrifugation of single cell suspension obtained scratching the liver, lymphocytes were analyzed. As in Figure 123, doublets, dead cells, CD3^+^ T cells and CD19^+^ B cells were sequentially excluded. Moreover, considering that in this district also CD45- cells are present, a further exclusion gate has to be included. Among NK1.1+NKp46+ cells NK cells were gated as CD49b+CD49a- cells, and distinguished from CD49b-CD49a+ ILC1s.

While in blood and spleen NK cells represent the most abundant ILC subset, in tissues there are high proportions of the other ILCs subsets, which are largely tissue resident. CD127 is classically used to identify ILCs and distinguish them from NK cells, as it is not expressed by NK cells of liver, intestine, skin, uterus, salivary gland, bone marrow, or lymph nodes. However, CD127 is expressed by NK cells in the thymus and in some spleen populations, and it is not expressed by liver and intraepithelial gut ILC1s. Thus, the phenotypic characterization of tissue resident NK cells is more complicated and requires the analysis of additional markers. In particular, NK cells share many features with ILC1s, they both produce IFN-γ as the main cytokine and require Tbet for this function. However, while NK cells require Eomes for their development process, ILC1s develop in the absence of this transcription factor. Moreover, ILC1s are generally non-cytotoxic and express lower levels of perforin compared to NK cells [1243]. Regardless these developmental and functional differences, ILC1s have some phenotypic markers in common with NK cells (See Section VII Innate Lymphoid Cell (ILC) phenotypes), including NK1.1 in mice and NKp46 in both humans and mice. In the liver, for example, to distinguish these two populations it is useful to include additional markers such as CD49b, exclusively expressed by NK cells in mice, and CD49a and TRAIL, preferentially expressed by ILC1s in both humans and mice (Figure 124). CD200R has been shown to be an additional marker to distinguish ILC1s from NK cells in mice (Table 100) [1300].

In addition to ILC1s, NK cells share the expression of some markers with ILC3s. In mice ILC3s are dependent on Rorγt for their development and function [1282] and two subsets can be distinguished on the basis of NKp46 expression: NCR^+^ and NCR^−^ ILC3s. As NK cells and NCR+ ILC3s both express NKp46, the analysis of the expression of the transcription factors Rorγt and Eomes can be useful to distinguish them (See VII Innate Lymphoid Cell (ILC) phenotypes).

Unlike NK cells, ILC2s are characterized by the capacity to produce type 2 cytokines. They contain larger amounts of the transcription factor GATA3 compared to the other ILC subsets but upon activation can express high levels of KLRG1, an inhibitory receptor also expressed by mature NK cells [1346].

#### 2.3 Step-by-step sample preparation

Cell isolation: spleens and livers were scratched through 70 and 100 um cell strainers, respectively. Liver lymphocytes were isolated on a 37.5-67.5% Percoll gradient. For isolation of small intestine lamina propria cells, intestines were cut longitudinally, then transversally in 2-3 cm pieces, thoroughly rinsed with PBS, and shaken for 30 min in PBS containing 10% FBS, 15mM Hepes and 5mM EDTA to remove intraepithelial and epithelial cells. Intestines were then digested with collagenase VIII (300UI/ml) in complete RPMI for 45 min at 37°C under agitation, and lamina propria lymphocytes were isolated on a 40-100% Percoll gradient. Whole blood was analyzed using BD Trucount tubes according to the manufacturer’s instructions (BD Biosciences) [1347].

#### 2.4 Materials

The following Abs were used and/or are suggested for the surface and intracellular staining of mouse NK cells:

##### BD Biosciences:

CD45.2 AlexaFluor700 (1:200, clone 104), CD3 PE CF594 (1:100, clone 145-2C11), CD19 PE CF594 (1:200, clone 1D3), NK1.1 BV510 (1:50, clone PK136), CD49a AlexaFluor647 (1:400, clone Ha31/8), CD11b BV510 (1:400, clone M1/70), NKp46 BV421 (1:50, clone 29A1.4), TCRb FITC (1:400, clone H57-597), granzyme B PE (1:50, clone GB11), Rorγt PE (1:100, clone Q31-378), CD107a FITC (1:60, clone 1D4B), Fc block CD16/CD32 (1:200, clone 24G2);

##### eBiosciences:

NKp46 PerCP-eFluor710 (1:50, clone 29A1.4), CD49b PE-Cy7 (1:200, clone DX5), Eomes APC (1:100, Dan11mag);

##### Biolegend:

IFN-g BV421 (1:100, clone XMG1.2), CD19 APC-Cy7 (1:200, clone 6D5), NKp46 APC (1:50, clone 29A1.4), NK1.1 PE-Cy7 (1:50, clone PK136), CD3 FITC (1:100, clone 145-2C11), CD19 FITC (1:100, clone 6D5).

Dead cells were identified using the fixable blue dead cell stain kit (Invitrogen). For surface staining cells Abs were diluted in PBS 5mM EDTA (Euroclone). For intracellular staining, cells were fixed and permeabilized with an intracellular staining kit (eBioscience). Flow cytometric data were acquired with a BD LSRII flow cytometer equipped with FACS DIVA software (BD Biosciences), and analyzed by using FlowJo software (FlowKo, LLC).

#### 2.5 Data analysis

**Figure 125.**
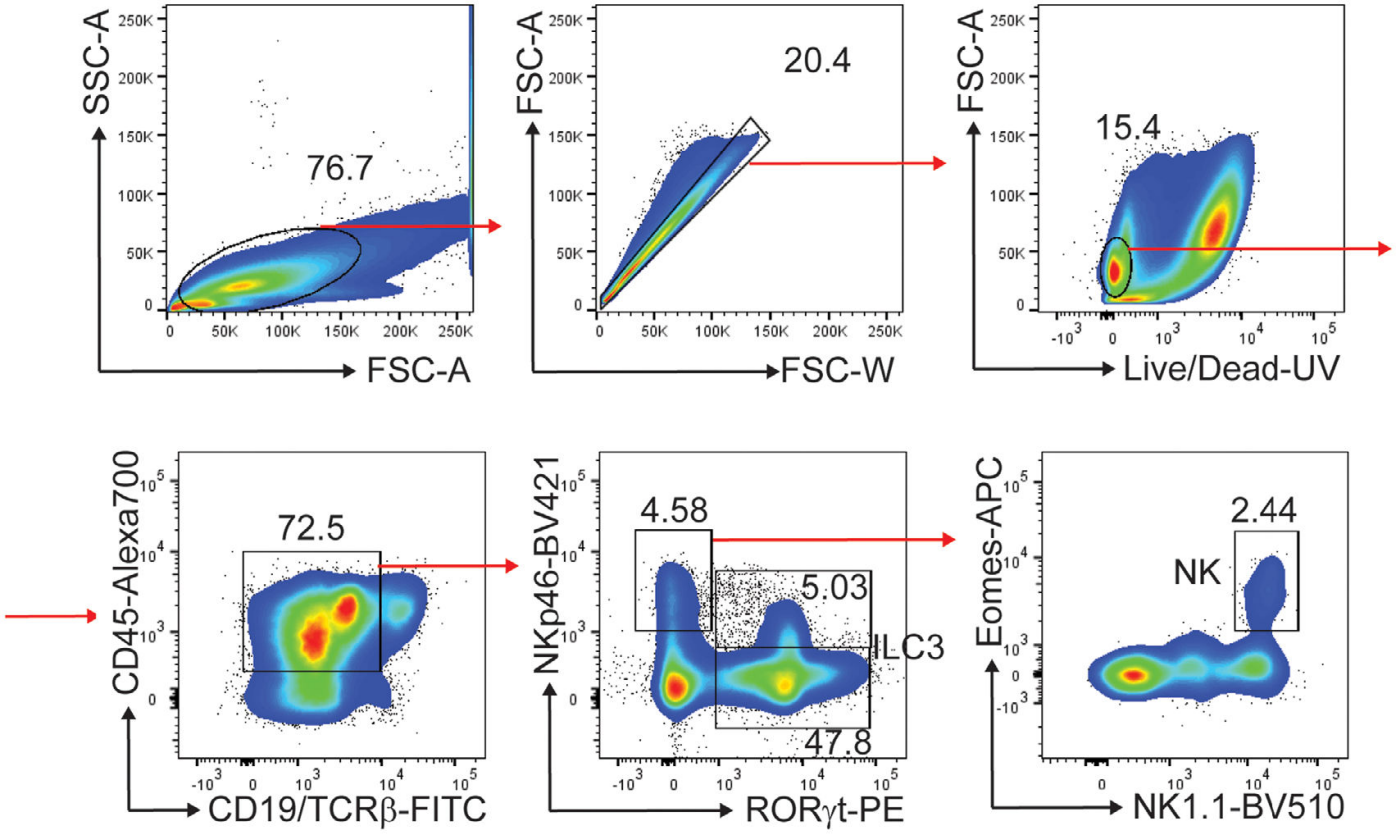
Identification of murine small intestine lamina propria NK cells in C57B/6 mice. After enzymatic digestion and Percoll density gradient centrifugation, single cell suspension obtained from the small intestine was analyzed. As in figure 124, doublets, dead cells, CD45- and CD19^+^ B cells were sequentially excluded. T cells were gated out based on their expression of TCRβ. Rorγt+ cells represent ILC3s, which can be further distinguished in NCR+ and NCR- ILC3s. Among Rorγt- NKp46+ cells, NK cells are gated as NK1.1+Eomes+ cells.

Lymphocytes are gated as small cells with a low granularity compared to monocytes and granulocytes. Cells with a constant FSC-A/FSC-W ratio are gated as single cells (to exclude doublets) and dead cells are excluded by gating on cells negative for staining with viability dies (Figure 123B). CD45 is used as an additional marker, especially in tissues, to exclude non-immune cells (Figure 124 and 125). In Figure 123-125 plot axes are displayed in log scale and labels show the fluorochrome and marker used. Anti-CD3 and anti-CD19 fluorochrome labeled Abs are used to exclude from the analysis T and B cells. Among double negative cells, NK cells are gated as NK1.1^+^NKp46^+^ (Figure 123 and 124). Percentage of cells in the gates is reported in each plot (Figure 123-125).

##### V. Pitfalls

For the identification and distinction of NK cells from other ILCs by flow cytometry it must be considered that, like T helper cell subsets, ILC subsets also display a certain degree of plasticity. For example, fate mapping and adoptive transfer studies in mice have shown that gut CCR6-NKp46- ILC3s can convert into IFNγ producing NK1.1+NKp46+ ILC1s via a CCR6-NKp46+ intermediate through a decrease in Rorγt expression and parallel increase in Tbet [1293, 1348].

#### 2.6 Top Tricks

When including CD11b in cytometry panels to exclude myeloid cells from the analysis, it must be taken into account that mature murine NK cells express this marker too. Therefore, one must check carefully that NK1.1+ and/or NKp46+ CD11b+ cells do not get excluded in the associated gating strategy.

#### 2.7 Summary of the phenotypes

This is detailed in Table 100.

#### 2.8 Key information human vs murine

This is detailed in Table 99 and summary provided here:

VII Both human and murine NK cells are distinguished from T cells because they do not express CD3
VIII Human NK cells are identified by the expression of CD56
IX Murine NK cells are identified by the expression of NKp46
X Both human and murine NK cells are distinguished from other ILCs by the expression of the transcription factor Eomes

## IX Mononuclear phagocyte phenotypes

### 1 Human mononuclear phagocytes: Monocytes, macrophages, and dendritic cells

#### Overview

Both, lymphoid and non-lymphoid, tissues contain a high number of mononuclear phagocytes, innate myeloid cells that play crucial roles in homeostasis as well as host-pathogen interactions. This pool is comprised of monocytes, macrophages and dendritic cells (DCs). Ontogeny, heterogeneity and specific functions of these cells have been extensively described in various recent reviews (e.g., [1349-1353]).

The efficient processing of human tissues is crucial to properly isolate and characterize tissue-associated monocyte, macrophage, and DC populations in steady state or inflammation. Thus, this chapter provides basic guidelines for the isolation and phenotyping of human mononuclear phagocytes across a variety of tissues. We describe the processing and flow cytometry-staining techniques for human blood, spleen, lung, and lymph nodes. Furthermore, this chapter provides basic gating strategies as well as tips & tricks and background information for each cell type in order to easily identify the various mononuclear phagocyte sub-populations across tissues.

#### 1.1 Human monocytes

##### 1.1.1 Introduction.

Monocytes can be found in the blood and most other tissues within in the human body. These cells were once thought to be the precursors of tissue resident macrophages [1354], though we now know macrophages are of embryonic origin [1350]. Nevertheless, monocyte-derived cells can replace the embryonic counterparts depending on the tissue concerned. For further reading on the ontogeny, heterogeneity, and specific functions of monocytes we refer the reader to a few recent publications [1350, 1352, 1355-1359].

In humans, monocytes can be distinguished into CD14^hi^CD16^−^ ‘classical’, whose main functions may be innate sensing, immune responses, and migration vs. CD14^lo^CD16^+^ ‘non-classical’ monocytes [1360], that are the equivalent of the inflammatory CXCR1^hi^CCR2^−^CD62L^−^CD43^hi^Ly6C^lo^ and patrolling CX3CR1^int^CCR2^+^CD62L^+^CD43^lo^Ly6C^hi^ monocytes found in murine tissues, respectively. Additionally, a minor population of CD14^+^CD16^+^ ‘intermediate’ monocytes exists, equipped mostly for Ag presentation and cytokine secretion during immune responses. In both, mice and humans, it has been demonstrated that developmental relationships exist where classical monocytes can give rise to non-classical monocytes [1361-1366].

However, using single cell RNA sequencing and high dimensional analyses tools recently is has become clear that within these major monocyte subsets multiple cellular states exist, of transcriptionally distinct subsets with perhaps homeostasis vs. inflammation/disease-specific functions. Here, we focus on the techniques for the enrichment and flow cytometry-based identification of the overall monocyte populations across human tissues.

##### 1.1.2 Step-by-step sample preparation.

##### 1.1.2.1 Step-by-step sample preparation for human blood. Critical:

This protocol is designed for 10 ml of human blood. If working with lower blood volumes ensure to keep the appropriate ratio for blood vs. PBS vs. Ficoll-Paque.

1. Aliquot 10 ml of Ficoll-Paque (pre-warmed to RT) into a 50 ml conical tube.
2. Dilute 10 ml of blood with PBS to a final volume of 40 ml.
3. Carefully layer the 40 ml of diluted blood on top of the Ficoll-Paque layer.
4. Centrifuge at 1800 G for 25 min, at RT. *Critical:* set centrifuge to acceleration = 0 - 1 and brake = 0 – 1.
5. Collect the PBMC layer, which is found at the Plasma (PBS)-Ficoll-Paque interface, and transfer it into a 50 ml conical tube. Top up with PBS to a final volume of 50 ml.
6. Centrifuge at 365 G for 5 min, at 4 °C. *Critical:* set centrifuge to maximum acceleration and maximum brake.
7. Aspirate the supernatant.
8. Re-suspend the pellet in 1 ml of 1× RBC lysis buffer, incubate for 5 min, at RT in the dark.
9. Top up with PBS to a final volume of 50 ml
10. Centrifuge at 365 G for 5 min, at 4 °C.
11. Aspirate the supernatant and re-suspend the pellet (which contains the immune cells) in 1 ml of PBS.
12. Transfer cells into a 1.5 ml microcentrifuge tube, perform cell count, and proceed with staining protocol as described in 1.1.2.5.

##### 1.1.2.2 Step-by-step sample preparation for human spleen.

1. Prepare 20 ml of digestion buffer (see 1.1.3.2).
2. Transfer spleen sample into 2 ml microcentrifuge tube containing 0.5 ml of the digestion solution (see 1.1.3.2). Using a small sterile pair of scissors mince spleen tissue into small pieces.
3. Transfer the tissue suspension into one well of a 6-well plate and add on 4 ml (per well) of the digestion solution.
4. Incubate for 1 h at 37 °C.
5. Pipette up and down 6 - 8 times with a 10 ml disposable transfer pipette in order to disrupt the remaining tissue/gain a single cell suspension, and transfer suspension over a 70 μm cell strainer into a 50 ml conical tube. Rinse the well with PBS and add to cell suspension in the 50 ml conical tube (via filter; to ensure minimum cell loss). Adjust the volume of the suspension with PBS to a total of 50 ml.
6. Centrifuge at 365 G for 5 min, at 25 °C.
7. Aspirate supernatant and re-suspend the pellet in 40 ml of PBS, to achieve a proper dilution of the spleen cell suspension.
8. Aliquot 10 ml of pre-warmed (RT) Ficoll-Paque into a new (clean) 50 ml conical tube.
9. Carefully transfer the 40 ml of the diluted spleen cell suspension as a top layer onto the 10 ml of pre-warmed (RT) Ficoll-Paque.
10. Follow steps 4 - 12 from chapter 1.1.2.1 (sample preparation for human blood).

###### 1.1.2.3 Step-by-step sample preparation for human lung.

1. Follow steps 1 - 7 from chapter 1.1.2.2 (sample preparation for human spleen).
2. Then, follow Steps 4 - 12 from chapter 1.1.2.1 (sample preparation for human blood).

###### 1.1.2.4 Step-by-step sample preparation for human skin (epidermis).

*Critical:* Skin should be immediately immersed in RPMI 1640 upon collection and incubated on ice until further processing.

1. Cut skin into strips (1 x 5 - 10 cm) using disposable scalpels, in a large petri dish.
2. Cover circular Styrofoam with a rubber mat and place a sterile silicon mat on top.
3. Pin down the skin longitudinally at one end with 2 x 25 G needles, keeping it stretched while pulling down from the other end.
4. Shave skin using a Goulian knife by applying a side-to-side slow motion, to make skin thinner. *Critical:* Blades should not be re-used (to avoid contamination).
5. Spread shaved strips in a 15 cm petri dish containing 50 ml of RPMI 1640 supplemented with: 10 % FCS, 1 % L-glutamine, 1 % Pen/Strep, 0.8 mg/ml Worthington’s collagenase (1×) and 05 mg/mL DNase I.
6. Cut the skin strips into pieces of 1 cm^2^ and incubate them for a minimum of 18 hours, at 4 °C.
7. Pipette up and down for about 8 - 10 times using a 10 ml disposable transfer pipette, in order to disrupt the epidermis and dermis layers. Filter through a 70 μm cell strainer into a 50 ml conical tube. Rinse the petri dish with PBS and add through filter to cell suspension to ensure minimum loss of cells.
8. Adjust volume of the skin cell suspension with PBS, to a total of 50 ml.
9. Follow steps 6 - 12 from chapter 1.1.2.1 (sample preparation for human blood).

###### 1.1.2.5 Staining for human monocytes from different tissues.

####### Notes:

- The following protocol is used for staining monocytes (optimal 1 - 5 x 10^6^ cells/tube for staining) isolated from human blood (see chapter 1.1.2.1), spleen (see chapter 1.1.2.2), lungs (see chapter 1.1.2.3) and skin (see chapter 1.1.2.4).
- For Abs and reagents, see 1.1.3
- Staining can be performed either in a 1.5 ml microcentrifuge tube or a V-shaped 96-well plate (non-culture-treated).
  1. Aliquot required number of cells, and centrifuge at 650 G for 2 min, at 4 °C.
  2. Aspirate/discard the supernatant and re-suspend the cell pellet in 1 ml of PBS containing Live/Dead blue dye (1:1000), incubate for 20 min, at 4 °C in the dark.
  3. Add human AB serum or FCS, at a final dilution of 5 %, and incubate for 15 min, at 4 °C in the dark, in order to block FC receptors on the immune cells and to neutralize free Live/Dead molecules that bind protein N-terminal amines. **Tip**: During the incubation time for steps 2 and 3 prepare the Ab (Ab) premix at final dilutions as described in table 101.
  4. Add 200 μl of FACS buffer and centrifuge at 650 G for 2 min, at 4 °C.
  5. Aspirate/discard the supernatant and re-suspend the cell pellet in 50 μl of Ab pre-mix. Incubate for 30 min, at 4 °C in the dark.
  6. Add 200 μl of FACS buffer, and centrifuge at 650 G for 2 min, at 4 °C.
  7. Aspirate/discard the supernatant, then:
    - For staining monocytes/macrophages: proceed to step 9.
    - For staining DCs: since a purified Ab is used to stain CADM1 you will need to perform an additional staining step, as described in step 8 before proceeding to step 9.
  8. Re-suspend the cell pellet in 50 μl of FACS buffer containing anti-Chicken-IgY-Alexa-Fluor 647. Incubate for 15 min, at 4 °C. Then add 200 μl of FACS buffer and centrifuge at 650 G for 2 min, at 4 °C. Aspirate/discard the supernatant.
  9. Re-suspend the cell pellet in 200 - 400 μl of FACS buffer, filter through a 70 μm cell strainer into a new (clean) flow cytometry tube and analyze using a suitable flow cytometer.

##### 1.1.3 Materials.

###### 1.1.3.1 General reagents and materials.

The reagents and materials are listed in Table 101.

###### 1.1.3.2 Buffers and other reagents to prepare.

####### Flow cytometry buffer (FACS buffer):

- 2 % FCS + 2mM EDTA in PBS + 0.05 % Azide in 1× PBS
- store on ice / at 4 °C

####### Digestion solution:

- 0.2 mg/ml collagenase + 0.05 mg/mL of DNase I in RPMI 1640 + 10 % FCS
- prepare fresh for every use

###### 1.1.3.3 Antibodies for human monocyte identification.

The Abs for human monocyte identification are listed in Table 102.

##### 1.1.4 Data analysis.

**Figure 126.**
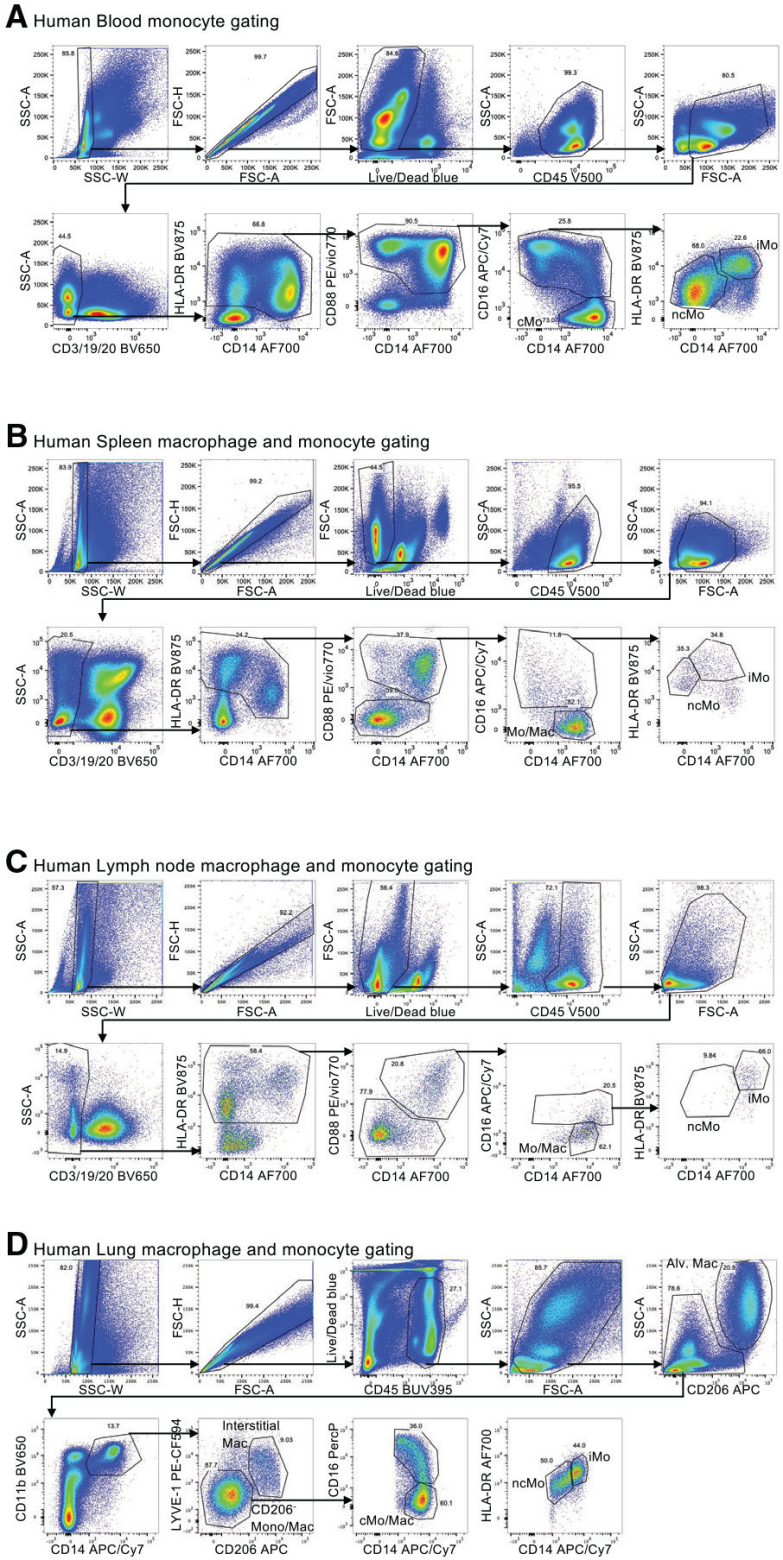
Flow cytometric analysis human monocytes and macrophages. In blood (A), the identification of human monocytes subsets as single, live, CD45^+^, CD3^−^, CD19^−^, CD20^−^. HLADR^+^, CD88^+^ cells. Using CD14, CD16 and HLA-DR, classical (cMo), intermediate (iMo) and non-classical (ncMo) monocyte subsets can be identified. A similar gating strategy is applied for the spleen (B), lymph node (C), and lung (D). Of note, CD14^+^ cells have been termed monocytes/macrophages (Mo/Mac). Within the lung (**D**), alveolar macrophages are identified as SSC-A^hi^ CD206^+^ cells and interstitial macrophages as SSC-A^lo^ CD206^+^ CD11b^+^ CD14^+^ LYVE-1^hi^ cells.

As depicted in Figure 126, a similar gating strategy is adopted for human blood, lymph node, spleen and lung samples to characterize classical monocytes (cMo), intermediate monocytes (iMo) and non-classical monocytes (ncMo) subsets.

First, cells are gated on live (Life/Dead^−^), CD45^+^ singlets before exclusion of CD3^+^/CD19^+^/CD20^+^ T and B cells. From there (A) blood monocytes are initially identified as HLADR^+^CD14^lo-hi^, then gated on all CD88^+^ cells before being separated into CD14^hi^CD16^−^ cMo and CD16^+^ cells, subsequently gated as HLADR^hi^CD14^lo-hi^CD16^+^ iMo and HLADR^int^CD14^−^CD16^+^ ncMo. Similar (B, C) splenic and LN monocytes are pre-gated on HLADR^+^CD14^lo-hi^ and CD88^+^ cells before being separated into CD14^hi^CD16^−^ cells which contain cMo (and macrophages) and CD16^+^ cells, subsequently gated as HLADR^hi^CD14^lo-hi^CD16^+^ iMo and HLADR^int^CD14^−^ CD16^+^ ncMo. (D) In the lung CD45^+^ cells are first gated on CD11b^+^CD14^+^ cells, then on LYVE-1^−^CD206^−^ to exclude interstitial macrophages, before either gating on CD16-CD14^+^ cMo or HLADR^int^CD14^int^ ncMo or HLADR^hi^CD14^hi^ iMo.

##### 1.1.5 Pitfalls.

We have recently demonstrated the use of CD88 to identify monocytes avoiding DC contamination within the monocyte gate [1367]. Although not shown for all tissues (shown in Figure 126), we recommend adding CD88 to the flow panel to ensure accurate identification of monocytes vs. DCs.

##### 1.1.6 Top Tricks.

In terms of generating qualitative flow cytometry data, this requires an optimal combination of fluorochromes/markers. It should be avoided to use Abs binding co-expressed markers conjugated with fluorochromes that have a lot a fluorescence spill-over into channels in which they are detected. Second, analyzing monocytes, macrophages and DCs (as described in the following chapters) by flow cytometry requires using more than 10 Abs and thus complexifies the definition of a “correct” compensation matrix. Third, when analyzing flow cytometry data using manual gating, a major challenge is to avoid dropping out cells of interest along the gates. To facilitate these two latter critical aspects of flow cytometry data analysis, an initial manual gating should be done to define major monocyte and DC subsets.

Then, using a compatible software (Diva, Kaluza, and eventually Flow Jo), n dot plot (for a n colour flow cytometry panel) should be defined (fluorochrome A on the x-axis versus all the other fluorochromes on the y-axis) all displaying CD45^+^ cells with all the monocyte and DC subsets overlayed (each having a defined colour). This will allow the proper setting of “all fluorochromes – the A fluorochrome” compensations. When all “fluorochrome X – fluorochrome A” compensations are properly set, the next fluorochrome must be displayed on the x-axis, and so on, until all fluorochromes have been properly compensated.

Once compensations are properly set, two methods can be used for analysis, manual gating or unsupervised dimensionality reduction, with later being the most reliable method.

For manual gating, the different cell subsets must be displayed in all gates defined to reach them by “back gating” to ensure that each of them is present at all steps of the gating strategy. To ensure that all populations can be properly visualised in all gates, back gated cell subsets should be ordered by count, with the rarest populations displayed above all the other cell subsets.

A major drawback of manual gating is that gates are defined based on one (histogram) or two ‘markers’ (dot plot) expression, which in some cases does not allow the proper separation of cell populations that share overlapping phenotypes. Thus, unsupervised dimensionality reduction is now becoming the gold standard method to avoid this, since it reduces all dimensions (one marker = one dimension) into a two- or three-dimensional space. Machine learning based algorithms such as t-distributed stochastic neighbour embedding [1368] or Uniform Manifold Approximation and Projection [1113] combined with clustering algorithms [1369] allow the proper identification and separation of cell subsets by integrating all markers analyzed [1367].

##### 1.1.7 Clinical relevance statement.

The importance of monocytes in general can be observed in patients with IRF8 mutations, where the K108E mutation results in the absence of circulating monocytes, consequently patients are more prone to severe opportunistic infections [1370, 1371]. On the other hand, an interesting case study demonstrated that the loss of CD16^+^ monocytes itself, does not lead to disease, and patients bearing this profile are healthy [1371].

A routine blood test will include an absolute monocyte count, where a high (monocytosis) or low (monocytopenia) monocyte count can indicate the presence of a disease. Regarding individual monocyte subsets, it has been demonstrated that certain subsets are expanded or reduced in certain pathologies as has been shown for SLE, sepsis, rheumatoid arthritis and more recently in COVID-19, where a low percentage of non-classical monocytes could be identified in high-risk patients [1372-1376].

##### 1.1.8 Summary of the phenotypes.

This is detailed in Table 103 (with refs. [1377, 1378]).

#### 1.2 Human macrophages

##### 1.2.1 Introduction.

In 1882, Elie Metchnikoff first described cells ‘eating to defend’ during his eminent experiment in Messina, demonstrating starfish larvae engulfing a pierced rose thorn [1379, 1380]. This pivotal observation laid the foundations for “phagocytosis”, a term coined by Carl Claus and Metchnikoff himself [1381]. These phagocytic cells are distributed throughout the body in several organs and attempts were made to classify these cells into a system. Metchnikoff called these cells “macrophages”. Several studies led to the belief that monocytes originated from the bone marrow, travel to the tissues via the bloodstream where they become tissue macrophages. However, recently genetic fate-mapping techniques have supported the observation that the majority of tissue resident macrophages are in fact embryonically derived [1362, 1382-1385]. Similar observations have been made in humans where an early monocyte-independent primitive wave has been described [1386]. In additions, several studies have shown the longevity of macrophages in the case of Langerhans cells [1387], microglia [1388] and alveolar macrophages [1389], although further clarity is required.

In this chapter, we focus on the techniques for the enrichment, isolation, and flow cytometry-based identification of macrophage populations across human tissues.

##### 1.2.2 Step-by-step sample preparation.

For step-by-step sample preparation for human lymph node, spleen and lung, please refer to 1.1.2, and follow the steps provided there for tissue processing and isolation of human monocytes, as the experimental procedures are the same.

##### 1.2.3 Materials.

###### 1.2.3.1 General reagents and materials.

For general reagents and materials needed for the isolation and phenotyping of human macrophages please see the information provided in 1.1.3.1 (human monocyte isolation and phenotyping).

###### 1.2.3.2 Antibodies for human macrophage identification.

For Abs to identify human macrophages, please see table 102.

##### 1.2.4 Data analysis.

A similar gating strategy for monocytes is adopted for human lymph node, spleen and lung samples to characterize the tissue resident macrophage populations (Figure 126).

First, cells are gated on live (Life/Dead^−^), CD45^+^ singlets before exclusion of CD3^+^/CD19^+^/CD20^+^ T and B cells. In the (B) spleen and (C) LNs cells are pre-gated on HLADR^+^CD14^lo-hi^ cells and CD88^+^ cells. Macrophages are then identified within the CD14^hi^CD16^−^ fraction. (D) In the lung resident macrophages can be divided into SSC-Ah^i^CD206^+^ alveolar macrophages (AM/Alv. Mac) and SSC-A^lo^CD206^+^ interstitial macrophages (IM) which can be additionally pre-gated as CD11b^hi^CD14^+^. Some macrophages will also be contained within the CD14^+^Lyve-1^−^ fraction, constituting a mixture of monocytes and macrophages (Figure 126).

##### 1.2.5 Pitfalls.

For pitfalls we refer the reader to section 1.1.5.

One of the major drawbacks in the macrophage field is difficulty to clearly distinguish tissue monocytes from macrophages by flow cytometry. Whilst F4/80 is commonly used in mice, equivalent markers are lacking in humans.

##### 1.2.6 Top Tricks.

For further tricks we refer the reader to section 1.1.6.

Additional markers are often used to classify macrophages as either M1 or M2 (with further sub-categories of M2), to represent classically activated and alternatively activated macrophages, respectively. However, macrophages do not behave in this binary manner and instead represent a heterogeneous group of cells that exist in various states *in vivo*. Therefore, such classification should be interpreted carefully.

##### 1.2.7 Clinical relevance statement.

Macrophages help to shape the tissue niche in both health and disease. In the context of cancer, tumor-associated macrophages (TAMs) have been shown to have prognostic value in various tumor types. The infiltration of TAMs is often correlated with poor prognosis for many types of cancer. Recently, the immunosuppressive role of FOLR2^+^ TAMs in hepatocellular carcinoma was shown to be reminiscent of fetal liver development [1390]. Further exploration of this onco-fetal reprogramming and profiling of macrophage heterogeneity in pathology may open up therapeutic avenues. Similar to monocytes, macrophages have also been associated with severity and pathogenesis in COVID-19 [1376, 1391].

##### 1.2.8 Summary of the phenotypes.

This is detailed in Table 104 (with refs. [1392, 1393]).

#### 1.3 Human dendritic cells

##### 1.3.1 Introduction.

During the 1970s, Ralph Steinman and colleagues identified a novel cell type with a stellate morphology amongst adherent mononuclear phagocytes which he called dendritic cells [1394]. A series of papers describing and characterising these cells were published in the Journal of Experimental Medicine from 1973-1975. These cells were shown to be unique potent activators of naïve T cells in comparison to other Ag presenting cell [1395, 1396] and were regarded as ‘accessory’ cells that linked innate and adaptive immunity [1397, 1398].

Human DCs traditionally include cDCs, namely CD1c^+^ cDC2 and CLEC9A^+^XCR1^+^CADM1^+^CD141^+^ cDC1 and CD123^+^ pDCs [1399]. Recent developments within the field have identified additional human DC subsets [1367]. We have recently described cDC progenitors, namely pre-DC [1369], and also identified CD14^+^ DCs, commonly referred to CD14^+^ DC3 [1367]. The identification of the cells by flow cytometry is highlighted below.

##### 1.3.2 Step-by-step sample preparation.

For step-by-step sample preparation please refer to 1.1.2, and follow the steps provided there for tissue processing and isolation of human monocytes, as the procedures are the same.

##### 1.3.3 Materials.

###### 1.3.3.1 General reagents and materials.

For general reagents and materials needed for the isolation and phenotyping of human dendritic cells please see the information provided in 1.1.3.1 (human monocyte isolation and phenotyping).

###### 1.3.3.2 Antibodies for human dendritic cell identification.

The Abs for human dendritic cell identification are listed in Table 105.

##### 1.3.4 Data analysis.

**Figure 127.**
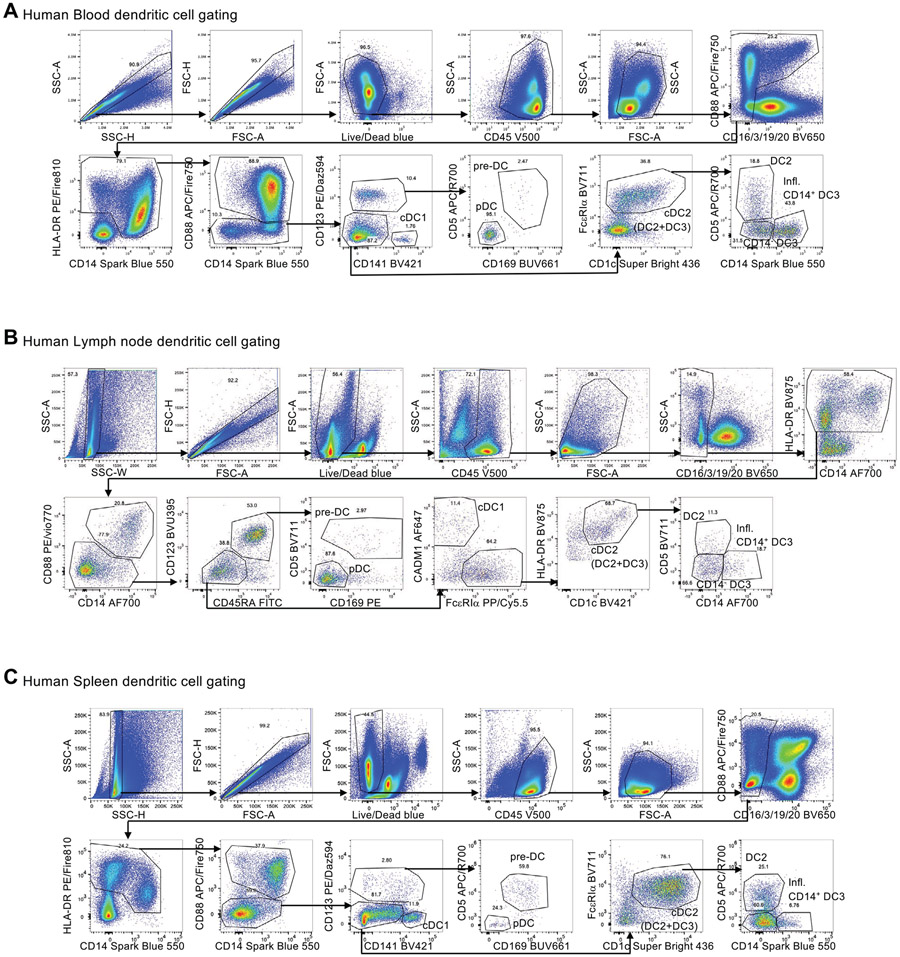
Flow cytometric analysis of human dendritic cells. In blood (A), the identification of human dendritic cells as single, live, CD45^+^, CD3^−^, CD19^−^, CD20^−^, CD16^−^, HLADR^+^, CD88^−^ cells. These cells can be further sub-divided into the DC subsets using the cell surface markers shown. A similar gating strategy is applied for the lymph node (B) and spleen (C).

As depicted in Figure 127, a similar gating strategy is adopted for human blood, spleen, lymph node samples to characterize the different DC populations. All samples are pre-gated on single, live, CD45^+^, LIN^−^, and HLA-DR^+^ cells prior gating on DCs. LIN is defined as CD3, CD19, CD20 and CD16. Samples are further gated on CD88^−^ cells to exclude monocytes and macrophages, prior gating on DCs (A-C).

In (A) blood and (C) spleen cDC1 were gated as CD123^−^CD141^+^, while the CD123^+^CD141^lo^ population was further gated on CD5^+^CD169^+^ pre-DCs and CD5^−^CD169^−^ pDCs. The CD123^−^CD141^−^ population then was gated for FcεRIα^+^ cells, followed by gating on CD5^+^CD14^−^ cDC2, CD5^−^CD14^−^ cDC3, and CD5^−^CD14^+^ inflammatory cDC3. Similar, in the (B) LN CD123^+^CD45RA^−^ cells were split into CD5^+^CD169^+^ pre-DCs and CD5^−^CD169^−^ pDCs. CD123^−^CD45RA^−^ cells were further gated on FcεRIα^+^CADM1^+^ cDC1 and FcεRIα^+^CADM1^−^ cells that then were identified as HLA-DR^+^CD1c^+^ cDC2. This population also contains cDC3 and can be additionally split into CD5^+^CD14^−^ cDC2, CD5^−^CD14^−^ cDC3, and CD5^−^CD14^+^ inflammatory cDC3 (Figure 127).

##### 1.3.5 Pitfalls.

As mentioned in chapter 1.1.5/6, HLA-DR^+^ CD14^+^ cells were previously thought to represent monocytes. However, advances in technology have allowed us to identify surface markers more suited to clearly distinguish monocytes from DCs, such as CD88. Consequently, after excluding CD88^+^ monocytes, a subset of CD14^+^ cells (Infl. DC3) remains which aligns to the DC lineage [1367].

##### 1.3.6 Top tricks.

For general tricks we refer the reader to section 1.1.6. Further, we recommend using CD88 to avoid monocyte contamination within the DC gate.

##### 1.3.7 Clinical relevance statement.

DCs are key cells linking the innate and adaptive immune system. They are crucial in intial responses against foreign Ags and pathogens such as viruses and bacteria. Patients bearing mutations in IRF8, which can result in the loss of circulating cDC subsets, were shown to more severely suffer from various infections, as compared to IRF8-“normal” individuals. Similarly, pDC deficiencies have also been reported in patients with Pitt-Hopkins syndrome [1370, 1400].

It recently was shown that pre-DCs, the immediate precursor of DCs, are highly susceptible to HIV infection via Siglec-1 and thus can transmit HIV to the CD4^+^ T-cells [1401]. On the other hand, it has been reported that CD141^+^ DCs are resistant to HIV infection [1402]. The discovery of a CD14^+^ DC3 subset has also been examined in the inflammatory setting, where this subset specifically was expanded in patients with systemic lupus erythematosus and was shown to correlate with disease activity [1367]. With the identification of these newly described subsets, studies should incorporate markers to identify and elucidate the role of these cells in health and disease.

##### 1.3.8 Summary of the phenotypes.

This is detailed in Table 106.

### 2 Murine mononuclear phagocytes: Monocytes, macrophages, and dendritic cells

#### Overview

Murine lymphoid and non-lymphoid tissues contain high numbers of mononuclear phagocytes, innate myeloid cells that play crucial roles in homeostasis as well as host-pathogen interactions. This pool is comprised of monocytes, macrophages and dendritic cells (DCs). Ontogeny, heterogeneity and specific functions of these cells have been extensively described in various recent reviews (e.g., [1349-1353]).

The efficient processing of murine tissues is crucial to properly isolate and characterize tissue-associated monocyte, macrophage, and DC populations in steady state or inflammation. Thus, this chapter provides basic guidelines for the isolation and phenotyping of murine mononuclear phagocytes across a variety of tissues. We describe the processing and flow cytometry-staining techniques for murine blood, bone marrow, spleen, lung, lymph nodes, intestine, and skin. Furthermore, this chapter provides basic gating strategies as well as tips & tricks and background information for each cell type in order to easily identify the various mononuclear phagocyte subpopulations across tissues.

#### 2.1 Murine monocytes

##### 2.1.1 Introduction.

Just like human tissues, murine lymphoid and non-lymphoid tissues contain high numbers of mononuclear phagocytes, such as monocytes, that play crucial roles in homeostasis as well as host-pathogen interactions. Monocytes as an overall population are quite heterogeneous and have been divided into two main subsets: inflammatory CXCR1^hi^CCR2^−^CD62L^−^CD43^hi^Ly6C^lo^ and patrolling CX3CR1^int^CCR2^+^CD62L^+^CD43^lo^Ly6C^hi^ monocytes [1377, 1378]. Ly6C^hi^MHCII^lo^ monocytes are recruited into tissues during homeostasis where they continually differentiate into Ly6C^lo^MHCII^hi^ macrophages, a phenomenon referred to as the monocyte to macrophage “waterfall”, which has been described in detail in the gut and skin [1403, 1404]. Further, bone marrow resident Ly6C^hi^ monocytes were shown to consist of two distinct subpopulations, the CXCR4^hi^ pre-monocytes and CXCR4^lo^ monocytes [1405]. Given the contribution of monocytes to the tissue resident macrophage pool and their array of functions during inflammation, the origin and ontogeny of monocytes, their heterogeneity, and specific functions have been extensively studied and reviewed in various excellent recent publications [1359, 1361, 1362, 1365, 1366, 1376-1378]. Here, we focus on the techniques for the enrichment and flow cytometry-based identification of monocyte populations across murine tissues.

##### 2.1.2 Step-by-step sample preparation.

###### 2.1.2.1 Step-by-step sample preparation for murine blood monocytes.

1. Collect blood (e.g., from the heart, retro-orbital plexus, facial vein, etc.) and immediately transfer into a sample tube containing either PBS + 10 mM EDTA or heparin. This will prevent blood from coagulating. Place tubes on ice till further processing.
2. Centrifuge at 1350 rpm, 4 °C for 4 min.
3. Carefully aspirate supernatant. Try to avoid aspirating the blood and containing cells, as the pellet will be rather fluid.
4. Resuspend pellet in 2 ml of 1× RBC lysis buffer, incubate for 5 min at RT.
5. After 5 min stop reaction by adding 10 ml of FACS buffer.
6. Centrifuge at 1350 rpm, 4 °C for 4 min.
7. Carefully aspirate supernatant. **Tip**: If the pellet still contains a lot of red blood cells, you might want to repeat RBC lysis step a second time for 3 min. Try avoiding further RBC lysis rounds, as the lysis buffer is very harsh on your immune cells.
8. For flow cytometry staining follow steps described in section 2.1.2.8 Staining for murine monocytes from different tissues.

###### 2.1.2.2 Step-by-step sample preparation for murine bone marrow monocytes.

1. Remove femur and tibia from euthanized mouse and, using scissors, free bones from surrounding muscle tissue and tendons. **Tip**: Use a paper towel to gently remove tissue that is stuck to the bones. This will also make it easier to detach the knee to separate femur and tibia, and to detach the foot from the tibia – use a turning/rotating motion to gently remove the foot/knee without breaking the bone.
2. Place clean bones in ice-cold PBS in a 6-well plate, on ice, till all tissues have been harvested.
3. To flush out the bone marrow: take bones out of PBS, e.g., place on the inside of the lid of your 6-well plate and carefully cut off a tiny bit at both ends of the bone.
4. Fill a 20 ml syringe with ice-cold FACS buffer. Use a 25 G needle to flush out the bone marrow, directly into a 15 ml or 50 ml conical tube. **Note**: This needle size should fit into most mouse bones, allowing to sufficiently flush out all the bone marrow but other G sizes will work just as well. **Tip**: For best results flush bone from both sides, moving the needle up and down within the bone while flushing.
5. Centrifuge at 1350 rpm, 4 °C for 4 min.
6. Aspirate supernatant.
7. Thoroughly suspend pellet in about 1 ml of 1× RBC lysis buffer, incubate for 3 min at RT. **Note**: You want to reach a single cell suspension at this step to ensure proper RBC lysis. Make sure you have no BM “pieces” left.
8. After 3 min stop reaction by adding 10 ml of FACS buffer.
  **Note**: You may want to filter the single cell suspension using a 70 μm nylon mesh/cell strainer to remove lose bone particles and clumps.
9. Centrifuge at 1350 rpm, 4 °C for 4 min. Carefully aspirate supernatant.
10. For flow cytometry staining follow steps described in section 2.1.2.8 Staining for murine monocytes from different tissues.

###### 2.1.2.3 Step-by-step sample preparation for murine spleen monocytes.

1. Harvest spleen from euthanized mouse and place in ice-cold PBS in a 6-well plate, on ice, till all tissues have been harvested.
2. Prepare digestion buffer (as described in reagents list), keep at RT.
3. Aliquot 2 ml of digestion buffer into a fresh well in a 6-well plate.
4. Take spleen out of PBS, quickly dry off on clean paper towel, if necessary, remove fat/pancreas tissue and place spleen in digestion buffer.
5. Use fine tweezers (or scissors) to tear spleen into very fine pieces.
6. Incubate in digestion buffer at 37 °C for 30 min.
7. Stop digestion by adding 3 ml of PBS + 10 mM EDTA.
8. Carefully, but thoroughly, pipette spleen suspension up and down using either a 5 ml pipette or an 18 G needle with a 5 ml syringe, up to 20 times to gain a single cell suspension.
9. Transfer spleen cell suspension over a 70 μm nylon mesh/cell strainer into a 50 ml conical tube.
10. Centrifuge at 1350 rpm, 4 °C for 4 min.
11. Aspirate supernatant.
12. Thoroughly suspend pellet in about 1 ml of 1× RBC lysis buffer, incubate for 3 min at RT.
13. After 3 min stop reaction by adding 10 ml of FACS buffer.
14. Centrifuge at 1350 rpm, 4 °C for 4 min. Carefully aspirate supernatant.
15. For flow cytometry staining follow steps described in section 2.1.2.8 Staining for murine monocytes from different tissues.

###### 2.1.2.4 Step-by step sample preparation for murine lung monocytes.

1. Thoroughly perfuse freshly euthanized mouse intracardially with cold PBS, and harvest lungs into a 12-well plate containing cold PBS, on ice.
2. Place individual lung samples into 1.5 ml microcentrifuge tube containing 500 μl of digestion solution **1**.
3. Mince lung into small pieces using fine scissors (in the tube).
4. Transfer to 12-well plate containing additional 1 - 1.5 ml digestion solution (final volume 1.5 - 2 ml of digestion solution **1**).
5. Incubate at 37 °C for 30 min.
6. Homogenize minced and digested sample using a 18 G syringe needle and 3 ml syringe and filter through 70 μm cell strainer (you may use the syringe plunger to push tissue through the strainer) into 50 ml conical tube.
7. Wash remaining cells from strainer with 20 ml FACS buffer.
8. Centrifuge at 400 G for 5 min, at 4 °C
9. Lyse any remaining erythrocytes by resuspending cell pellet in 500 ul of 1× RBC lysis buffer for 3 min, at RT. Then stop reaction by topping up with FACS buffer.
10. Centrifuge at 400 G for 5 min, at 4 °C
11. Resuspend pellet in FACS buffer.
12. Filter cell suspension again through a 70 μm cell strainer to remove any clumps that may have formed after erythrocyte lysis and transfer cells to flow cytometry tube.
13. For flow cytometry staining follow steps described in section 2.1.2.8 Staining for murine monocytes from different tissues.

###### 2.1.2.5 Step-by-step sample preparation for murine intestinal monocytes.

1. From a freshly euthanized mouse, open up the abdominal cavity by dissecting through the skin and peritoneal membrane in a line along the ventral midline.
2. Remove the intestinal tract by dissecting the small intestine just after the pyloric sphincter, dissecting the colon by sectioning it as caudally as possible, and then carefully lift the intestines out while severing any places of attachment to the abdominal cavity and other organs.
3. Place the dissected intestine into 6-well plate with cold PBS on ice. **Note**: At this point you may retrieve the mesenteric LNs from the mesenteric fat for analysis, if desired.
4. Remove as much attached mesenteric fat from intestines as possible, pulling the fat from one end and following through to the other end until the intestines have been linearized.
5. Follow the following steps for cleaning the fecal content of small intestine and colon respectively:
  - For the small intestine: dissect just above the caecum, retrieve the Peyer’s Patches that lie along the length of the intestine by either cutting or plucking them (they can be analyzed separately or discarded), open the lumen lengthwise with scissors, and wash away fecal content from the opened small intestine in a beaker containing cold PBS before sectioning washed small intestine into 0.5 - 1 cm long pieces and placing into 50 m conical tube.
  - For the colon: separate away from the caecum (discard the caecum), use two pairs of forceps to squeeze solid fecal content out of the lumen, open the lumen lengthwise wish scissors, and wash away remaining fecal content from the opened colon in beaker containing cold PBS before placing washed colon into a 50 ml conical tube.
6. Add 25 ml of cold PBS into the 50 ml conical tube with the washed intestinal sections and place on ice while completing previous steps for other samples.
7. Vigorously shake the intestinal sections in 50 ml conical tube with cold PBS to get rid of the mucus for around 10 seconds each round (4 rounds with fresh cold PBS each round for small intestine, only once for colon).
8. Put the washed pieces into a new 50 ml conical tube and keep on ice while completing the wash step(s) for other samples.
9. When all samples are ready, add 10 - 12.5 ml of epithelial dissociation buffer to each sample and incubate for 20 min at 37 °C in an orbital shaker set to 250 rpm.
  - During this incubation prepare two petri dishes, one clean and the other filled with cold PBS, and 1.5 ml microcentrifuge tubes with 300 - 500 ml of digestion buffer **1** (for small intestine) or digestion buffer **2** (for colon).
  - If the epithelial compartment is to be retained, prepare the additional 50 ml conical tubes and cell strainers for collection.
10. Dilute epithelium dissociation buffer with 25 ml of cold PBS and shake vigorously for 10 seconds in the 50 ml conical tube.
11. Pour out the tube contents into the first clean petri dish (or through a cell strainer into an additional 50 ml conical tube if the epithelium compartment is to be retained for further analysis).
12. Transfer the pieces to the second petri dish with cold PBS and move them around to wash away traces of DTT/EDTA and epithelium cells.
13. Dry briefly on a piece of tissue before transferring the tissue pieces to the 1.5 mL microcentrifuge tube with the appropriate digestion buffer.
14. Mince tissue into small pieces with fine scissors, and then pour into 6-well plate, washing out the remaining tissue from the microcentrifuge tube with digestion solution **1** (to a final volume of 2.5 - 3 ml in the well).
15. Incubate for 45 min at 37°C. **Note**: Some protocols state that agitation at this step will enhance the digestion process but usually this does *not* have any effect on digestion efficiency.
16. Homogenize minced digested sample with 18 G syringe needle and 3 ml syringe and filter through a 70 μm cell strainer (you may use the syringe plunger to push tissue through the strainer) into the final 50 ml conical tube.
17. Centrifuge at 400 G for 5 min, at 4 °C. Carefully aspirate supernatant.
18. If cell pellet is still loose after centrifugation, repeat step 17.
19. For flow cytometry staining follow steps described in section 2.1.2.8 Staining for murine monocytes from different tissues.

###### 2.1.2.6 Step-by-step sample preparation for murine skin (ears) monocytes.

1. Harvest ears from euthanized mouse by dissection with a fine scissors.
2. The following steps depend if total skin is analyzed, or if the epidermis and dermis are analyzed separately:
  - If processing total skin, proceed to place ears directly into digestion solution 1 and mince into small pieces using a pair of fine scissors, and then proceed on with step 7.
  - If analyzing the epidermis and dermis separately proceed on to step 3.
3. Using two pairs of fine forceps, split each ear into dorsal and ventral halves to expose the inner dermal layer.
4. Float the ear halves dermis side down in 3 ml of Dispase solution/well in 6-well plate, ensuring that they are sufficiently spread out on the solution surface.
5. Incubate for 1 h at 37 °C.
6. Place each ear half on a suitable clean flat surface (polystyrene dish or lid, stainless steel tray, or a dark ceramic tile are all suitable) dermis side down.
7. In order to separate epidermis and dermis, carefully scrape the epidermis from the dermis using forceps and wash thoroughly in PBD or medium the dermis to remove any remaining epidermis.
8. Using forceps, place tissue into microcentrifuge tubes containing 500 μl digestion solution **1**, and mince into small pieces with fine scissors.
9. Pour out the cut up tissue into a 12-well plate and wash remaining minced tissue into same well using an additional 1 ml of digestion solution **1** (final volume 2 ml)
10. Incubate for 1 h at 37 °C.
11. Homogenize with 3 ml syringe and 18 G needle and siphon it through 70 μm nylon mesh into flow cytometry tube, using a 1 ml pipette tip as a funnel.
12. Centrifuge at 400 G for 5 min, at 4 °C. Carefully aspirate supernatant.
13. For flow cytometry staining follow steps described in section 2.1.2.8 Staining for murine monocytes from different tissues.

###### 2.1.2.7 Step-by-step sample preparation for murine lymph node (LN) monocytes.

1. Harvest lymph nodes of interest from euthanized mouse into 12-well plate with 1 ml of RPMI 1640 + 10 % FCS in each well.
2. Add 1 ml of 2x concentrated digestion solution **1** (= digestion solution **3**; hence the final digestion solution will be 1× working concentration).
3. Tear apart lymph nodes in the well and digestion solution using two 25 G needles mounted on 1 ml syringes (or using two tweezers).
4. Incubate for 30 min at 37 °C.
5. Homogenize with 3 ml syringe and 18 G needle and siphon it through 70 μm nylon mesh into flow cytometry tube, using a 1 ml pipette tip as a funnel.
6. Centrifuge at 400 G for 5 min, at 4 °C. Carefully aspirate supernatant.
7. For flow cytometry staining follow steps described section 2.1.2.8 Staining for murine monocytes from different tissues.

###### 2.1.2.8 Staining for murine monocytes from different tissues.

1. Resuspend pellet in FACS buffer and transfer 1 - 10×10^6^ cells to flow cytometry tube for cell surface staining.
2. Centrifuge at 1350 rpm / 400 G, 4 °C for 4 min, aspirate supernatant.
3. Prepare blocking buffer (FACS buffer + 1:50 rat/mouse serum or purified CD16/32 (FC-block)) and cocktail containing all Abs required (see table 108 below) dilution as recommended by manufacturer, or 1:100) for primary staining, store in the dark on ice or at 4 °C.
4. Add 25 μl of blocking buffer to the pellet, vortex, incubate for 10 - 15 min in the dark, at 4 °C. This will help prevent unspecific binding of subsequently used Abs.
5. Add 25 μl of prepared cocktail to the cell suspension, vortex, incubate for 15 - 30 min in the dark, at 4 °C.
6. Add 2 - 3 ml of FACS buffer to the cell suspension to wash off Ab cocktail.
7. Centrifuge at 1350 rpm / 400 G, 4 °C for 4 min, aspirate supernatant.
8. *Optional:* If required, add secondary Ab, e.g., fluorochrome-conjugated Streptavidin (dilution 1:300 usually is sufficient), vortex, incubate for 15 min in the dark, at 4 °C. Wash off with 2 - 3 ml of FACS buffer, centrifuge at 1350 rpm / 400 G, 4 °C for 4 min, aspirate supernatant.
9. Resuspend pellet in approximately 200 μl of FACS buffer containing DAPI (1:200).
10. Proceed to analyze sample on flow cytometer. **Note**: Filter sample using a 70 μm nylon mesh/cell strainer prior acquisition to avoid clogging of the analyzer.

##### 2.1.3 Materials.

###### 2.1.3.1 General reagents and materials.

The general reagents and materials are listed in Table 107

###### 2.1.3.2 Buffer and reagents to prepare.

####### Media for tissue processing:

- RPMI 1640 containing 10 % FBS (or FCS)
- store on ice / at 4 °C

####### Digestion buffer (also digestion solution 1):

- 0.2 mg/ml collagenase IV + 0.03 mg/ml DNase I in RPMI + 10 % FBS/FCS
- prepare fresh before use

####### Flow cytometry buffer (FACS buffer):

- 0.5 % w/v BSA + 2 mM EDTA in 1× PBS
- store on ice / at 4 °C

####### Flow cytometry staining buffer (as used for indicated tissues):

- 1 % n-mouse serum + 1 % n-rat serum in flow FACS buffer
- store on ice / at 4 °C

###### 2.1.3.3 Additional reagents for murine intestine processing.

####### Epithelium dissociation solution:

- 50 mM EDTA + 100 μl 20 mM DTT in PBS

####### Digestion solution 2:

- 0.4 mg/ml collagenase IV + 0.03 mg/ml DNase in RPMI + 10 % FCS
- prepare fresh before use

###### 2.1.3.4 Additional reagents for murine skin (ears) processing.

####### Dispase solution:

- 4 U/ml dispase in RPMI 1640
- prepare fresh before use

###### 2.1.3.5 Additional reagents for murine lymph node (LN) processing.

####### Digestion solution 3:

- 0.4 mg/ml collagenase IV + 0.06 mg/ml DNase I in RPMI + 10 % FBS/FCS (2x concentrated digestion buffer/solution **1**)
- prepare fresh before use

###### 2.1.3.6 Antibodies for murine monocyte identification.

The Antibodies for murine monocyte identification are listed in Table 108.

##### 2.1.4 Data analysis.

**Figure 128.**
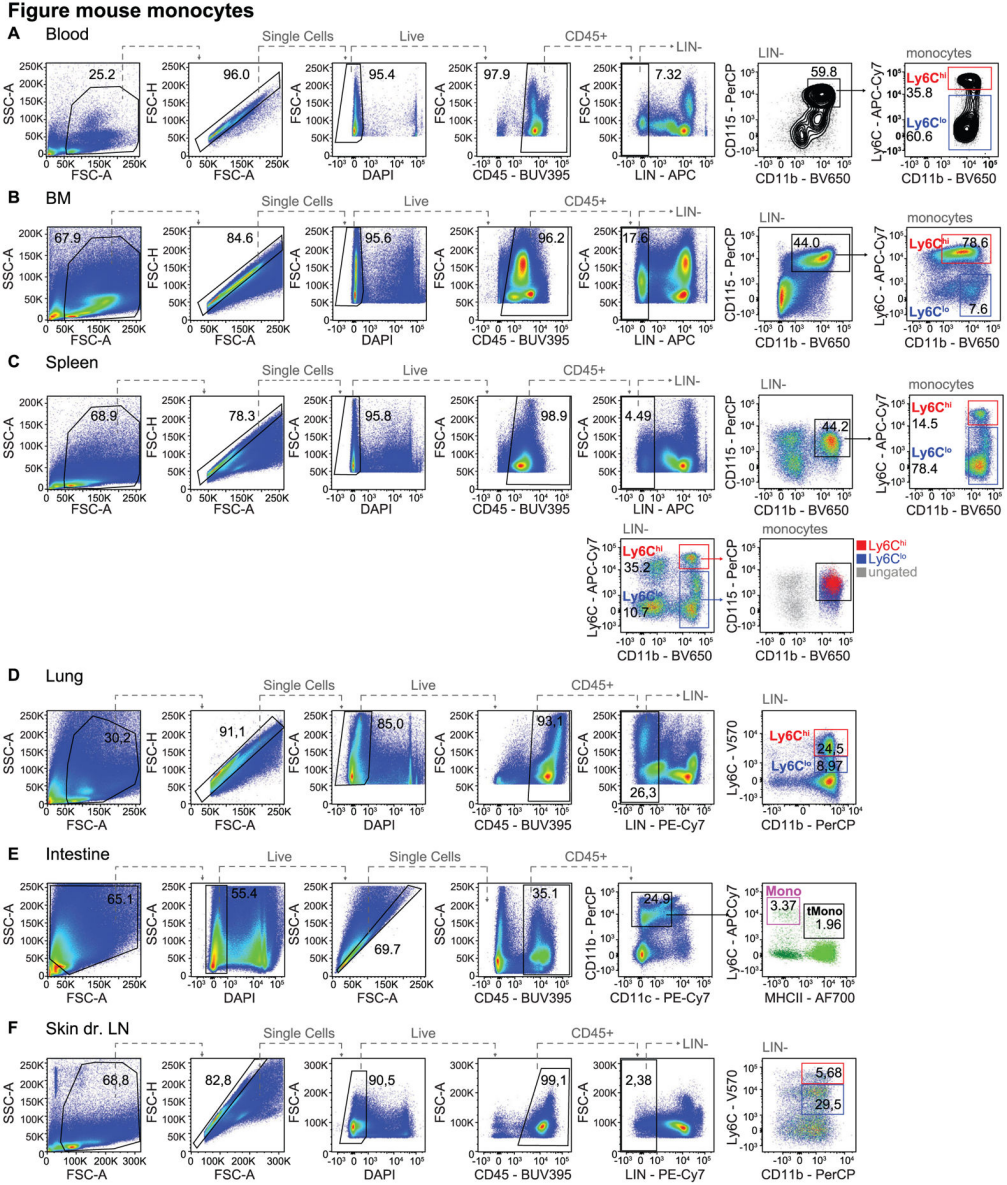
Flow cytometric analysis murine monocytes. (A-F) Example for basic gating strategy from FSC-A/SSC-A, over doublet exclusion and gating on Live, CD45^+^ LIN^−^ cells (defined as CD3/CD19/CD49b/Ly6G^−^) for blood, BM, spleen, lung, intestine, and LN murine samples. (A-D, F) Monocytes are identified as CD115^+^CD11b^+^ cells and can be further divided into Ly6C^lo^ and Ly6C^hi^ monocytes (blue and red gates, respectively). (E) Monocytes are identified as CD11c^lo^CD11b^+^ cells and can be further divided into Ly6C^lo^ MHCII^hi^ transitional monocytes (tMono) and Ly6C^hi^MHCII^lo^ monocytes (black and pink gates, respectively).

As depicted in Figure 128 a similar gating strategy is adopted for murine blood, bone marrow, spleen, lung, intestine, and lymph node (LN) samples to characterize classical monocytes (cMo) and non-classical monocyte (ncMo) subsets. All samples are pre-gated on single, live, CD45^+^ and/or LIN^−^ cells prior gating on monocytes. LIN is defined as CD3, CD19, CD49b, and Ly6G to exclude T cells, B cells, NK cells, and neutrophils, respectively prior gating on monocytes.

Moreover, monocytes in the (A) blood, (B) bone marrow, (C) spleen are pre-gated on CD115^+^CD11b^+^ cells, prior gating on CD11b^+^Ly6C^hi^ classical monocytes or CD11b^+^Ly6C^lo^ non-classical monocytes. In the (D) lung and (F) skin-draining LN LIN^−^ cells are directly gated for CD11b^+^Ly6C^hi^ classical or CD11b^+^Ly6C^lo^ non-classical monocytes. (E) Intestinal LIN^−^ cells are pre-gated on CD11b^+^CD11c^lo^ cells prior gating on MHCII^−^Ly6C^+^ monocytes or MHCII^+^Ly6C^+^ transitional monocytes (tMono) (Figure 128).

##### 2.1.5 Pitfalls.

- Various methods whereby different enzymes are used for processing mouse skin have been reported [1406-1408]. The effect certain enzymes can have on the surface expression of some markers should be considered.
- Cytokine receptors such as CSF-1R (CD115) are often shed off if samples are treated too harshly or processed over long periods or even internalized. Avoid receptor shedding/internalization by working quickly and keeping samples chilled.
- LNs contain numbers of lymphocytes. It is recommended not to stain too many cells at once (especially in the case of mesenteric LNs and Peyer’s patches) to avoid saturating the Ab staining mix.

##### 2.1.6 Top tricks.

- Inclusion of a lineage channel containing e.g., B, T, NK cell or neutrophil markers (e.g., CD19, CD3, CD49b/NK1.1 or Ly6G respectively) and gating on LIN^−^ cells prior gating on mononuclear phagocytes helps to gain a “cleaner” separation of these populations and will lower the risk of contamination with other cell types.
- Inclusion of CD117 into the panel, followed by gating on CD117^−^ LIN^−^ cells prior the monocyte gating can lead to a better resolution of monocyte populations.
- Additionally, to CD11b and Ly6C other monocyte marker may be included for further/more detailed separation of Ly6C^hl^ and Ly6C^lo^ monocytes, such as e.g., CX3CR1, CCR2 or TremL4 (see Table 109) [1409].
- The same protocol is used to digest murine LNs can be used to process Peyer’s patches.
- If working with frozen/thawed bone marrow samples one may incubate the freshly thawed samples in RPMI 1640 for 6 h at 37 °C to allow for proper expression of cell surface receptors. This will improve staining results.

##### 2.1.7 Summary of the phenotypes.

This is detailed in Table 109.

##### 2.1.8 Key information human vs murine.

This is detailed in Table 110 and summarized here:

- While classical and non-classical monocytes are found in both, human and mouse, intermediate monocytes so far only have been shown to exist in humans.
- Both, human and murine, monocytes share common surface markers with cDCs. While it has proven more complicated to clearly separate human monocytes and cDCs, this is relatively easy in mouse. Murine cDCs highly express CD11c and MHCII but lack the key monocyte surface marker Ly6C. For human, cDC3 recently were shown to express the monocyte marker CD14 thus possibly contaminating the CD14^+^ monocyte population in classic gating strategies. However, we recently demonstrated that human monocytes can be clearly separated from cDCs using CD88.
- Certain markers used to identify murine monocytes do not exist in humans, and vice versa. Some of these are listed in Table 110 (Phenotypic differences between human and murine monocytes).

#### 2.2 Murine macrophages

##### 2.2.1 Introduction.

For the longest time, macrophages were thought to be exclusively derived from circulating blood monocytes seeding the tissues [1410]. However, we now know that the vast majority of tissue resident macrophages actually arise from precursors seeding the tissues during early embryogenesis [1350, 1362, 1365, 1383, 1384, 1411]. Most of these tissue resident macrophage populations are fully self-maintaining, and only populations indeed are replenished by circulating blood monocytes [1350, 1352]. Across tissues a variety of macrophage populations with tissue-specific functions can be found [1412], such as e.g., alveolar and interstitial macrophages in the lung [1413] or Langerhans cells (LCs) in the skin. Langerhans cells are epidermal-specific APCs, that were originally classified as members of the dendritic cell (DC) family, as they express surface markers such as CD11c, CD11b, and MHCII and were shown to actively migrate to lymphoid organs – all features of DCs. However, LCs were recently found to be more of fetal macrophage origin, hence these cells are now rather classified as the resident macrophages of the epidermis [1411, 1414, 1415].

In this chapter, we focus on the techniques for the enrichment, isolation, and flow cytometry-based identification of macrophage populations across murine tissues.

##### 2.2.2 Step-by-step sample preparation.

For step-by-step sample preparation please refer to section 2.1.2, and follow the steps provided there for tissue processing and isolation of murine monocytes, as the procedures are the same.

##### 2.2.3 Materials.

###### 2.2.3.1 General reagents and materials.

For general reagents and materials needed for the isolation and phenotyping of murine macrophages please see the information provided in section 2.1.3.1 Murine monocyte isolation and phenotyping.

###### 2.2.3.2 Antibodies for murine macrophage identification.

The Abs for murine macrophage identification are listed in Table 111.

##### 2.2.4 Data analysis.

**Figure 129.**
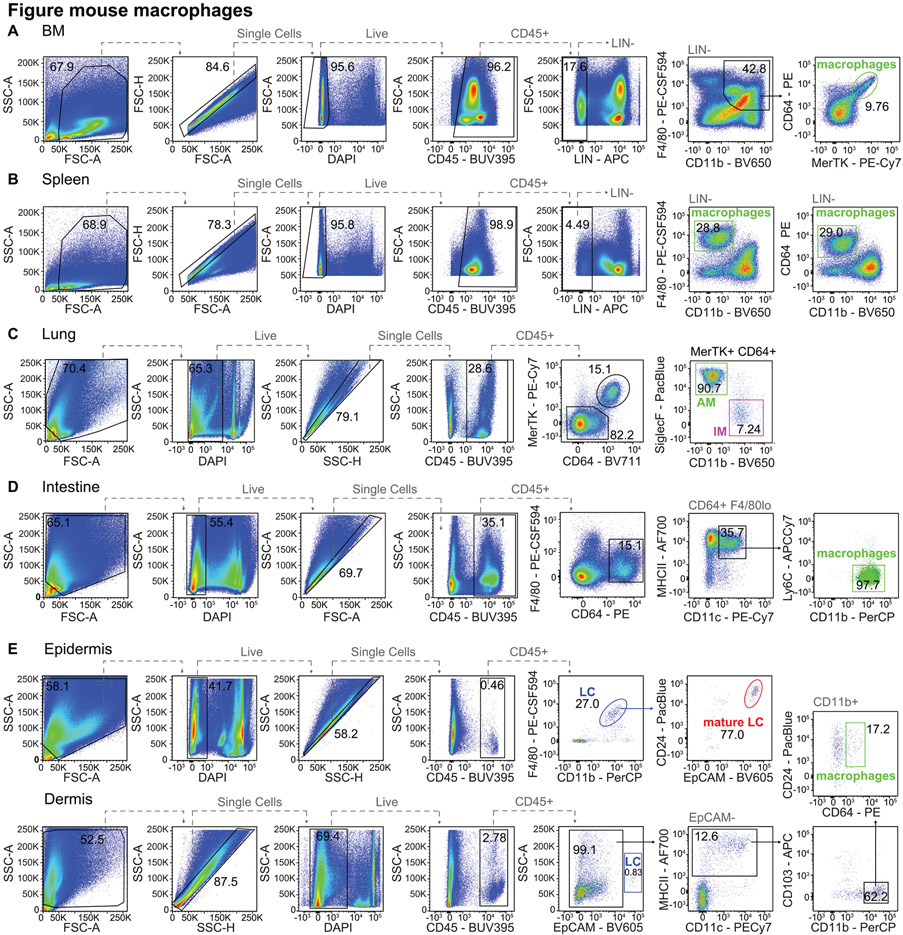
Flow cytometric analysis murine macrophages. Example for basic pre-gating strategy from FSC-A/SSC-A, over doublet exclusion and gating on Live, CD45^+^ LIN^−^ cells (defined as CD3/CD19/CD49b/Ly6G^−^) for BM and spleen (A, B) or CD45^+^ cells in lung, intestine, dermis and epidermis murine samples (C-E). Macrophages are gated as CD11b^+^F4/80^+^ CD64^+^MerTK^+^ cells in the BM (green gate; A) or CD11b^−^F4/80^+^/CD64^+^ red pulp macrophages in the spleen (B). In the lung macrophages can be divided into MerTK^+^CD64^+^ SiglecF^+^CD11b^−^ AM or SiglecF^−^CD11b^+^ IM (C). Intestinal macrophages are gated as MHCII^+^CD11c^+^CD11b^+^Ly6C^−^ cells (D), while in the dermis and epidermis Langerhans cells are identified as CD11b^+^EpCAM^+^ (F4/80^+^) cells, with mature LCs also expressing CD24, prior gating on CD11b^+^CD64^+^ macrophages (E).

As depicted in Figure 129 a similar gating strategy is adopted for murine bone marrow, spleen, lung, intestine, and skin (dermis and epidermis) samples to characterize the resident macrophage subsets. All samples are pre-gated on single, live, CD45^+^ and/or LIN^−^ cells prior gating on macrophages. LIN is defined as CD3, CD19, CD49b, and Ly6G to exclude T cells, B cells, NK cells, and neutrophils, respectively prior gating on macrophages.

Further, (A) BM and (B) spleen cells are pre-gated on CD11b^+^F4/80^+^ cells. (A) BM macrophages are then further identified as CD64^+^MerTK^+^ and (B) spleen red pulp macrophages as CD11b^−^ CD64^+^. (C) Lung LIN^−^ cells are first gated on CD64^+^MerTK^+^ cells and subsequently can be split into CD11b^−^SiglecF^+^ alveolar macrophages (AM) and CD11b^+^SiglecF^−^ interstitial macrophages (IM). (D) Intestinal macrophages are first gated on CD64^+^F4/80^lo^ cells prior gating on CD11c^+^MHCII^hi^ cells and finally CD11b^+^Ly6C^−^ macrophages, while (E) epidermal and dermal LCs are identified as F4/80^+^CD11b^+^EpCAM^hi^ with mature LCs also expressing CD24. Dermal macrophages are gated from the EpCAM^−^ population, first on CD11c^+/−^MHCII^hi^ cells and finally on CD11b^+^ and CD24^+^CD64^+^ macrophages (Figure 129).

##### 2.2.5 Pitfalls.

- Note that the here described protocol for spleen processing will yield mainly red pulp macrophages, while other splenic macrophages subsets such as marginal zone macrophages are more difficult to isolate. These can be better identified by inclusion of a Tim4 Ab into the panel [1416].
- [1413, 1417] Note that “aggressive” perfusion can lead to loss of alveolar resident cells.
- For intestinal processing some protocols opt to perform a 45/65 % [1418] or 45/70 % [1419] Percoll separation to further enrich for macrophages and DCs while removing debris. However, in our experience (and previously reported by Harusato et al. [1420]) cell yield is greatly reduced when this step is performed.
- [1416, 1421, 1422] The protocol described here for murine skin processing, can be used for analysis for total skin, or the epidermis and dermis separately. However, each method comes with its own drawbacks. Total skin preparations tend to have significantly less Langerhans cells (LCs) but better yield of DCs. Separation of the epidermis and dermis has good yield of LCs in the epidermal compartment, but results in a decreased yield of dermal DCs in the dermal compartment.
- [1423, 1424] See 2.1.5 for further pitfalls

##### 2.2.6 Top tricks.

- The two major macrophage populations in the mouse steady-state lung are the AMs and IMs. They express MerTK^+^ CD64^+^, but can also be differentiated from each other according to differences in SiglecF, MHCII, CD11c and CD11b expression. Further markers like Lyve-1 and others (not included in the example FACS plots; see table 109) have been shown to be very useful for AM vs IM discrimination [1413, 1417].
- In addition, if the configuration of the used flow cytometer allows for it, a “blank” channel (e.g., the FITC channel) is useful for discriminating “auto-fluorescent” AMs from non-autofluorescent IMs.
- The processing of both small intestine and large intestine for the purpose of analyzing macrophages/DCs in the lamina propria (LP) is detailed here. There are little to no macrophages/DCs in the gut epithelium compartment at steady state, hence in this protocol the dissociated epithelium is normally discarded. However, if desired, simple modifications that are mentioned here can be made to the protocol to retain the dissociated epithelium for separate analysis
- The intestinal LP contains a substantial population of eosinophils; exclusion of these can be achieved by inclusion of SiglecF (an eosinophil-specific marker) and CD64 (LP macrophage specific) into the panel. Monocyte-derived macrophages can be further gated using Ly6C [1421].
- F4/80 staining on gut macrophages may vary with digestion protocol due to shedding. Using a protocol different from the one described here may result in higher expression levels of F4/80 on gut macrophages.
- Some publications report a heterogeneity of CD11c expression on gut macrophages [1425]. While we chose to focus on the CD11c^+^ population, one may want to consider this when analyzing their data.
- Tim4 can be a useful marker to be added, for further delineation of macrophage populations [1416]; however, it also has been shown to be expressed on some DC populations [1422].
- LCs are the main macrophage population in the epidermis. LCs express numerous markers including F4/80, CD11b, EpCAM, Langerin and CD24 [1423, 1424]. However, EpCAM alone is sufficient to distinguish them from other CD45^+^ cells in the skin if there are limitations to machine configuration.
- See 2.1.6 for further tips

##### 2.2.7 Summary of the phenotypes.

This is detailed in Table 112.

##### 2.2.8 Key information human vs murine.

This is detailed in Table 113 and summarized here:

- Certain markers used to identify murine macrophages do not exist in humans, and vice versa or are expressed differently between species. Some of these are listed in Table 113 (Phenotypic differences between human and murine macrophages).
- In general, disease-specific functional differences between human and murine macrophages likely exist, such as during cancer evolution [1390], tissue specific functions [1426-1428], etc. However, further studies are needed to clearly identify these functions.

#### 2.3 Murine dendritic cells

##### 2.3.1 Introduction.

The murine DC compartment is traditionally divided into classical DCs (cDCs) and plasmacytoid DCs (pDCs), with cDCs being further subdivided into cDC1 (CD11b^−^ CD8^+^/XCR1^+^ in lymphoid tissues or CD11b^−^ CD103^+^ in non-lymphoid tissues) or cDC2 (CD8/CD103^−^ CD11b^+^SIRPα^+^ cells) [1351, 1399, 1429, 1430]. While all cDCs express high levels of CD11c and MHCII across tissues, pDCs are rather CD11c^int^ cells that are characterized by the expression of markers such as SiglecH, B220 and mPDCA-1, while lacking expression of CD11b. However, we and others recently showed that pDCs may in fact not be DCs or myeloid cells, but rather innate lymphoid cells [1431].

In homeostatic conditions, DCs constantly patrol tissues and sample Ags. Upon detection of infected cells, foreign Ags or viral RNA/DNA via extra- and intracellular Toll-like receptors (TLRs) and/or RIG-I pathways, they can orchestrate powerful immune responses, involving the secretion of crucial chemokines and cytokines, as well as the mobilization and activation of the adaptive immune system [1349, 1351, 1430, 1432]. Thus, DCs play crucial roles in homeostasis as well as in induction, maintenance and control of immune reactions, e.g., in response to viruses and cancer cells in mice and humans, linking the innate with the adaptive immune system. Being able to properly isolate and characterize DCs across tissues is important to study their various functions, and thus, we here provide the basic experimental techniques required.

##### 2.3.2 Step-by-step sample preparation.

For step-by-step sample preparation please refer to section 2.1.2, and follow the steps provided there for tissue processing and isolation of murine monocytes, as the procedures are the same.

##### 2.3.3 Materials.

###### 2.3.3.1 General reagents and materials.

For general reagents and materials needed for the isolation and phenotyping of murine dendritic cells please see the information provided in section 2.1.3.1 Murine monocyte isolation and phenotyping.

###### 2.3.3.2 Antibodies for murine dendritic cell identification.

The Abs for murine dendritic cell identification are listed in Table 114.

##### 2.3.4 Data analysis.

**Figure 130.**
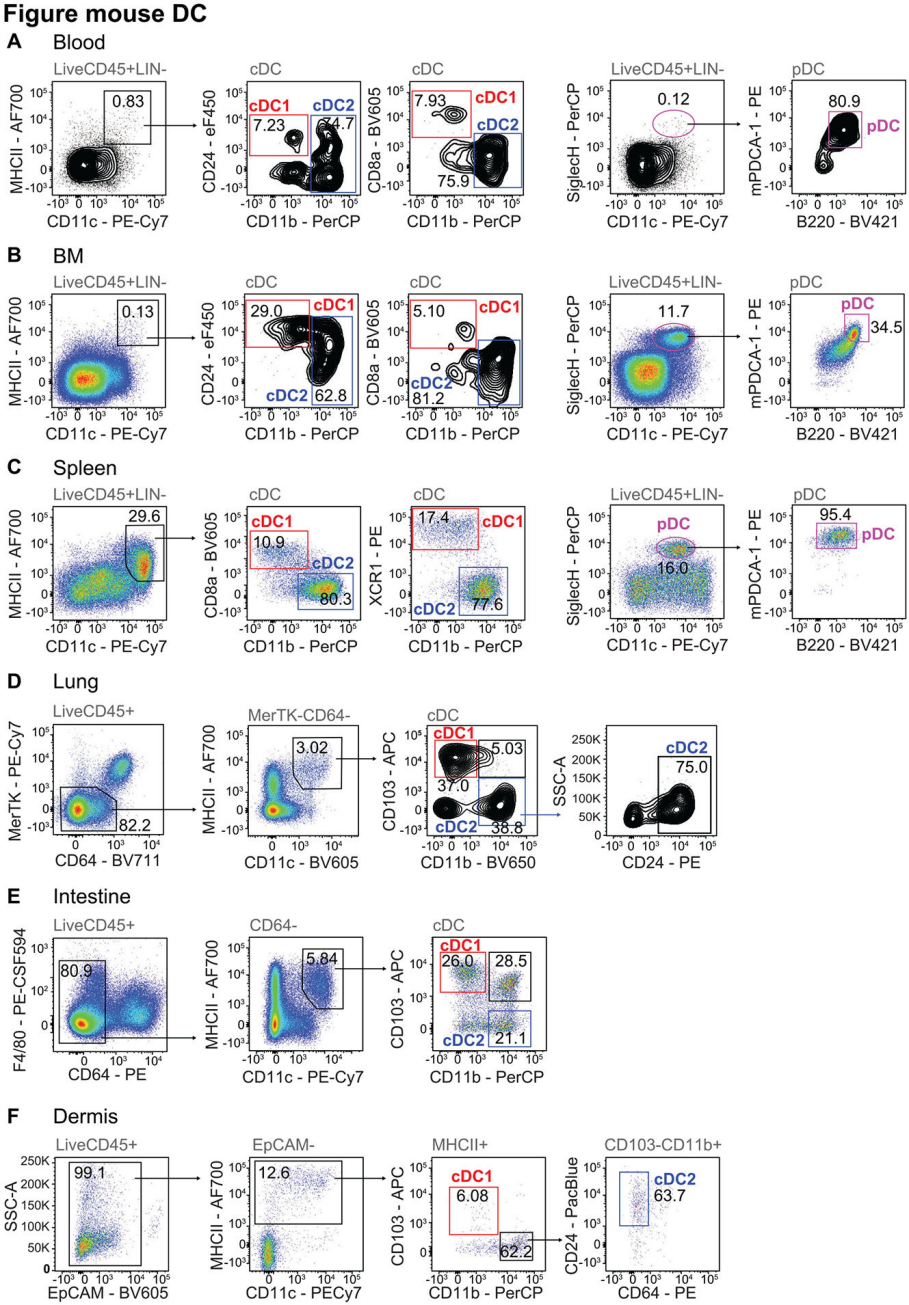
Flow cytometric analysis murine dendritic cells. Examples for basic pre-gating strategy from FSC-A/SSC-A, over doublet exclusion and gating on Live, CD45^+^ LIN^−^ cells (defined as CD3/CD19/CD49b/Ly6G^−^) across murine tissues is shown in Figure 128. Here, murine cDCs are gated as CD11c^hi^MHCII^+^ cells that can be further divided into cDC1 (CD8/XCR1^+^/CD24^+^CD11b^−^, red gates) and cDC2 (CD8^−^/XCR1^−^CD11b^+^, blue gates) in the blood (A), BM (B) and spleen (C), while pDCs are identified as CD11c^int^SiglecH^+^B220^+^mPDCA-1^+^ cells (pink gates; A-C). In the lung, intestine and dermis cDC1 are gated as CD103^+^CD11b^−^ (red gates) and cDC2 as CD103^−^CD11b^+^ /CD24^+^ cells (blue gates) (D-F).

**Figure 131.**
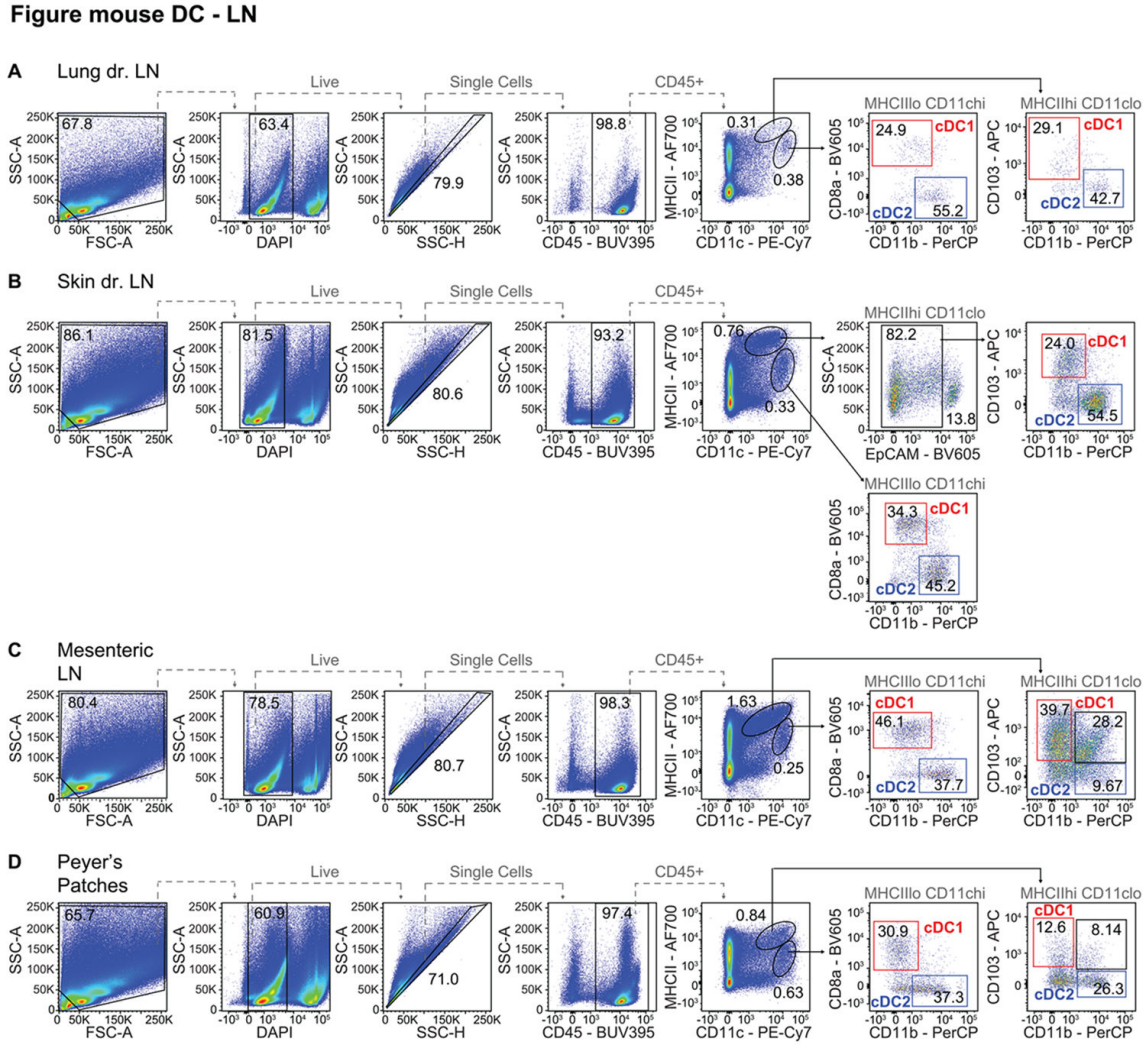
Flow cytometric analysis murine dendritic cells in LNs. Examples for basic pre-gating strategy from FSC-A/SSC-A, over doublet exclusion and gating on Live, CD45^+^ LIN^−^ cells (defined as CD3/CD19/CD49b/Ly6G^−^) across murine tissues is shown in Figure 128. In the LNs, murine cDCs can be divided into CD11c^hi^MHCII^lo^ resident DCs and CD11c^lo^MHCII^hi^ migratory DCs. Migratory DCs are further split into CD103^+^CD11b^−^ cDC1 (red gates) and CD103^−^CD11b^+^ cDC2, while resident DCs can be split into CD8^+^CD11b^−^ cDC1 (red gates) and CD8^−^CD11b^+^ cDC2, across LNs from different regions (A-D).

As depicted in Figure 130 and Figure 131, a similar gating strategy is adopted for murine blood, bone marrow (BM), spleen, lung, intestine, skin (Figure 130), and lymph node (Figure 131) samples to characterize cDC subsets and pDCs. All samples are pre-gated on single, live, CD45^+^ LIN^−^ cells prior gating on the different DC populations. All samples are pregated on single, live, CD45^+^ and/or LIN^−^ cells prior gating on DCs. LIN is defined as CD3, CD19, CD49b, and Ly6G to exclude T cells, B cells, NK cells, and neutrophils, respectively prior gating on DCs.

Here, we have pre-gated all cDC populations as CD11c^hi^MHCII^hi^ in all tissues (Figure 130), except for the lymph nodes (Figure 131), were cDCs can be split into resident CD11c^hi^MHCII^int^ and migratory CD11cintMHCIIhi cDCs.

In general, cDC2 are defined as CD11b^+^CD8/XCR1^−^ and cDC1 can be identified as CD11b^−^CD8/XCR1^+^ in the (Figure 130A) blood, (Figure 130B) BM, and (Figure 130C) spleen, while in the (Figure 130D) lung, (Figure 130E) intestine and (Figure 130F) dermis cDC1 mostly express CD103 and can be gated as CD11b^−^CD103^+^, as opposed to CD11b^+^CD103^−^ cDC2. Both, cDC1 and cDC2 can express CD24. (Figure 131) Within the LNs, resident CD11b^−^CD8/XCR1^+^ cDC1 and CD11b^+^CD8/XCR1^−^ cDC2, can be separated from migratory CD11b^−^CD103^+^ cDC1 and CD11b^+^CD103^−^ cDC2 as well. (Figure 130, 131) Across all tissues, pDCs are gated as CD11c^int^ cells and express the surface markers SiglecH, mPDCA-1/BST-2/CD317 and B220. Note, that in the (Figure 130B) BM there will be SiglecH^+^ but mPDCA-1/BST-2/CD317^lo^ and B220^lo^ cells, which are mainly pDC progenitors, thus using all three markers is advisable in this specific tissue (but not necessary in other tissues).

##### 2.3.5 Pitfalls.

- There are three main subsets of lamina propria DCs: cDC1, cDC2, and an intestine-specific subset of “double positive” CD103^+^ CD11b^+^ DP cDC2 (Figure 130). While in some publications intestinal cDC1 are delineated from cDC2 using XCR1 and SIRPα [1399] these markers alone are not sufficient to delineate the DP cDC2 fraction from the CD103^−^ CD11b^+^ cDC2.
- The protocol described here for murine skin processing, can be used for analysis for total skin, or the epidermis and dermis separately. However, each method comes with its own drawbacks. Total skin preparations tend to have significantly less Langerhans cells (LCs) but better yield of DCs. Separation of the epidermis and dermis has good yield of LCs in the epidermal compartment, but results in a decreased yield of dermal DCs in the dermal compartment.
- Do note that some populations of mouse DCs express Langerin as well [1424]. The dermis may contain some migratory LCs and these can be identified using EpCAM [1433] before gating for dermal cDC1 and cDC2 (Figure 130).
- See 2.1.5 for further pitfalls

##### 2.3.6 Top tricks.

- Mouse lymph nodes at steady-state contain two fractions of conventional DCs. The first fraction are migratory DCs that come from the peripheral tissues and express high levels of MCHII and lower levels of CD11c, and can be further split into cDC1 and cDC2 subsets using similar markers used for gating peripheral tissue DCs [1349]. The second fraction are lymph node resident conventional DCs, which express high levels of CD11c and lower levels MHCII, are also comprised of cDC1 and cDC2, and are gated using either XCR1 or CD8α, and SIRPα or CD11b for cDC1 and cDC2 respectively [1349] (Figure 130).
- When gating on pDCs in the bone marrow it is advisable to include SiglecH, mPDCA-1 and B220, as SiglecH also labels pDC progenitors, which either do not or only express low levels of B220 and mPDCA-1 yet.
- cDC1 traditionally were identified using CD8a but we highly recommend the use of XCR1 instead, as this marker is more specific than CD8α and yields a better discrimination of cDC1 from cDC2 (as can be seen in Figure 130) [1399, 1434, 1435].
- The two major cDC subsets in the lung are cDC1 and cDC2. cDC1s express XCR1 and CD103, while cDC2s express CD172α/SIRPα or CD11b, and CD24 [1399, 1417, 1429]. A minor fraction of CD103/CD11b-double-positive cDC2 can be found as well (Figure 130).
- See 2.2.5 for further tips on separating intestinal DCs and macrophages.
- EpCam may be included when staining skin-draining LN samples to separate LCs from DCs.
- See 2.1.6 for further tips

##### 2.3.7 Summary of the phenotypes.

This is detailed in Table 115.

##### 2.3.8 Key information human vs murine.

This is detailed in Table 116 and summarized here:

- Certain markers used to identify murine DCs do not exist in humans, and vice versa or are expressed differently between species. Some of these are listed in Table 116 (Phenotypic differences between human and murine DCs).
- In human a cDC3 subset recently was described, not yet described in mouse [1367]. However, the also recently identified cDC2A and cDC2B subsets in mouse [1436] potentially may resemble some sort of murine counterpart to the human cDC3. Functional evidence remains to be provided.
- In general, disease-specific functional differences between human and murine DCs likely exist. Further studies are needed here.

## X Mononuclear phagocyte assays

### 1 Human mononuclear phagocytes: Monocytes, macrophages, and dendritic cells

#### 1.1 Overview

As monocytes, macrophages, and DCs play important roles in tissue maintenance, immune activation and control during homeostasis, infections and non-infectious diseases, dietary changes, and many others, studying the function of these cells is rather important. Thus, this chapter aims to provides suggestions for basic functional assays to study human monocyte, macrophage, and DC function and responses.

#### 1.2 Human monocytes

##### 1.2.1 Introduction.

Monocytes are known to produce pro-inflammatory cytokines in response to inflammatory cues. The response of monocytes to stimuli can be measured by intracellular staining of these cytokines. Below we outline a protocol to measure TNF-α in response to LPS stimulation.

##### 1.2.2 Step-by-step sample preparation for ex vivo monocyte assays.

For isolation of human monocytes from different tissues follow the step-by-step sample preparation as described in section IX.1.1.2 as the procedures are the same. To proceed with the *ex vivo* assay continue with the following steps:

1. Place 5 x10^6^ PBMCs/ml in a sterile cell culture plate, in RPMI 1640 + 5 % FCS.
2. Add 10 ng/ml LPS to cell culture and incubate at 37 °C, 5 % CO_2_. *Critical:* Remember to keep some cells in culture without LPS, as unstimulated control sample.
3. After 1 h of incubation add 4 μl Golgistop (monensin) per every 6 ml of cell culture and incubate for a further 4 h, at 37 °C, 5 % CO_2_.
4. At the end of incubation, centrifuge cells at 300 G, 4 °C for 5 min, aspirate supernatant.
5. Proceed with extracellular staining protocol as described in section IX.1.1.2.5 (till step 7), then follow the subsequent steps:
6. Wash cells once with PBS, centrifuge at 300 G, 4 °C for 5 min and aspirate supernatant.
7. Resuspend cell pellet with 100 μl of Fixation/Permeabilization solution in a 96-microwell plate.
8. Incubate cells for 15 min at 4 °C, in the dark.
9. Wash cells with 100 μl Perm/Wash Buffer (1×), centrifuge at 300 G, 4 °C for 5 min, aspirate supernatant.
10. Resuspend cells in 100 μl of Perm/Wash Buffer (1×) containing a pre-determined optimal concentration of the intracellular Ab, e.g., TNF-α here (*Note:* we highly suggest titrating Ab concentrations, when using an Ab for the first time, to determine the “optimal” concentration for your samples).
11. Incubate at 4 °C for 30 min, in the dark.
12. Wash cells with 100 μl Perm/Wash Buffer (1×), centrifuge at 300 G, 4 °C for 5 min, aspirate supernatant.
13. Resuspend cells in staining buffer and proceed to flow cytometric analysis.

##### 1.2.3 Materials.

For general reagents and materials needed for the isolation and phenotyping of human monocytes please see the information provided in section IX.1.1.3.1 (Human monocyte isolation and phenotyping). Additional reagents needed for the here described assay are listed in Table 117.

##### 1.2.4 Data analysis.

**Figure 132.**
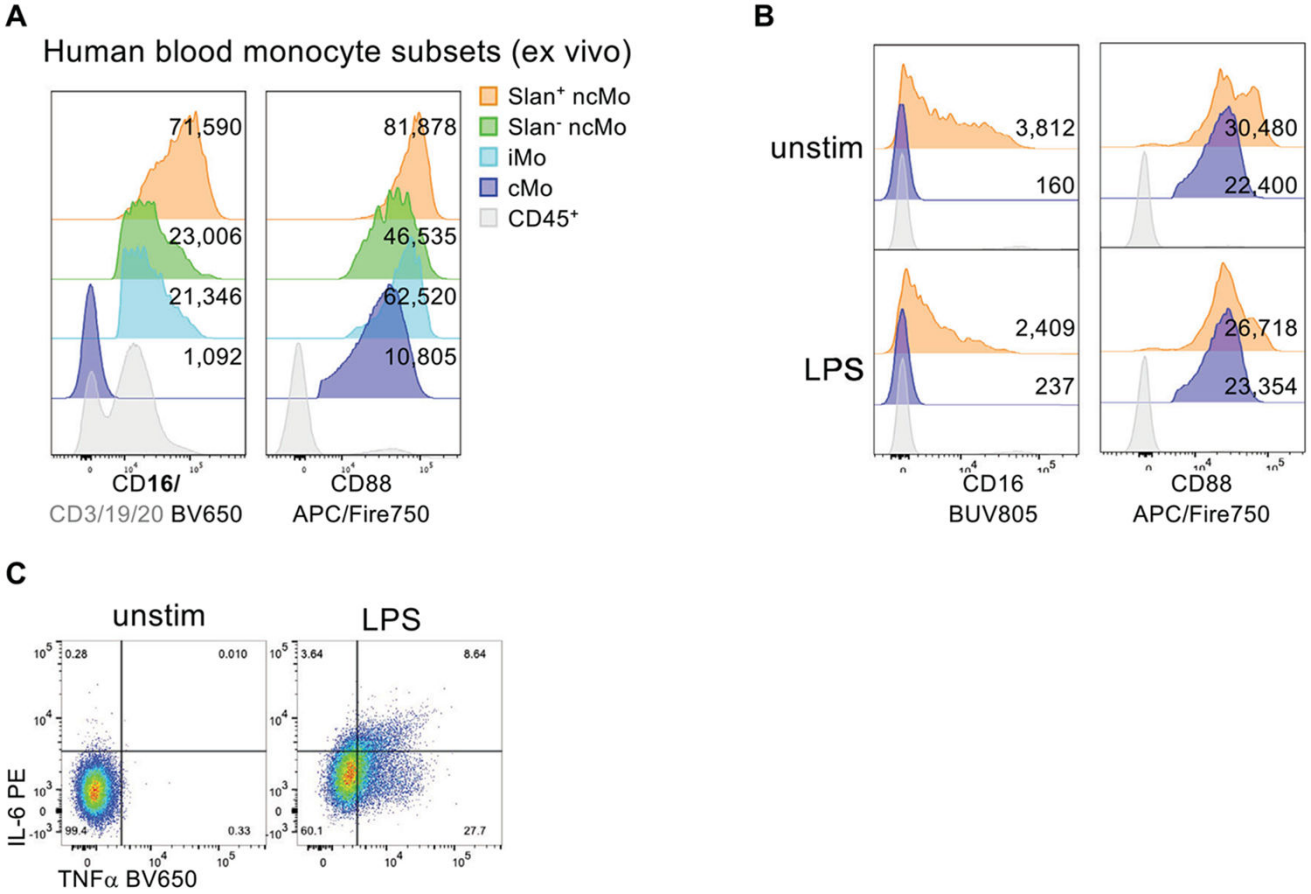
Flow cytometric analysis of human monocytes assays. *In vitro* stimulation with LPS. Human monocytes were identified as in Figure 126. CD16, CD88 an CD89 expression are shown on monocyte subsets *ex vivo* (A) and following stimulation with LPS (B). (C) TNFα and IL-6 profile on unstimulated and LPS-stimulated monocytes.

Gate monocytes as described in section IX.1.1.4. The benefit of using CD88 to identify monocytes can be depicted here as expression is retained post-stimulation whilst CD16 expression is decreased (Figure 132). After gating on monocyte subsets, the unstimulated control can be used to identify the quantify the percentage of cells responsible for the production of TNF-α and IL-6 in response to LPS (Figure 132 C, D).

##### 1.2.5 Pitfalls.

- Following stimulation with LPS, CD16 is known to be shed from the surface of monocytes. Consequently, the traditional CD14 vs. CD16 gating strategy can be misleading and result in misidentification of cell types.

##### 1.2.6 Top tricks.

- Whilst CD16 is no longer useful for identifying monocytes, CD88 expression is retained and can be used to identify these cells.

##### 1.2.7 Clinical relevance statement.

For the clinical relevance of monocytes, or activated monocytes, please see chapter IX.1.1.6.

#### 1.3 Human macrophages

##### 1.3.1 Introduction.

A similar assay as described for human monocytes (see section X.1.2) can be employed for human macrophages to assess their activation status by surface marker profiling and/or cytokine production. Thus, we refer the reader to the previous assay sections. In addition, a common macrophage assay is the phagocytosis assay in which e.g., fluorochrome-/dye-labeled beads can be co-cultured with macrophages to then readout the efficiency of bead-uptake by each macrophage population [1392, 1393].

##### 1.3.2 Step-by-step sample preparation.

For step-by-step sample preparation of macrophages please refer to section section IX.1.1.2 (Processing of human monocytes) and section IX.1.2.2 (Processing of human macrophages) and follow the steps provided there for tissue processing and isolation of human monocytes/macrophages, as the procedures are the same. For the *ex vivo* assay using human PBMCs or tissue macrophages please follow assay steps referred to in chapter X.1.2 Assays - Human monocytes.

##### 1.3.3 Materials.

For general reagents and materials needed for the isolation and phenotyping of human macrophages please see the information provided in see section IX.1.1.3 (human monocyte isolation and phenotyping).

##### 1.3.4 Data analysis.

Please follow the gating strategies provided in see section IX.1.2.4

##### 1.3.5 Pitfalls.

- For general pitfalls regarding the processing of human tissue macrophages we refer the reader to see section IX.1.2.5.
- Following stimulation with LPS, CD16 is known to be shed from the surface of monocytes. Consequently, monocytes may be contaminating the CD14^hi^CD16^−^ macrophage fraction.

##### 1.3.6 Top Tricks.

- For general tricks regarding the processing of human tissue macrophages we refer the reader to section IX.1.2.6.

##### 1.3.7 Clinical relevance statement.

Macrophages help to shape the tissue niche in both health and disease. In the context of cancer, tumor-associated macrophages (TAMs) have been shown to have prognostic value in various tumor types. The infiltration of TAMs is often correlated with poor prognosis for many types of cancer. Recently, the immunosuppressive role of FOLR2^+^ TAMs in hepatocellular carcinoma was shown to be reminiscent of fetal liver development [1390]. Further exploration of this oncofetal reprogramming and profiling of macrophage heterogeneity in pathology may open up therapeutic avenues. Similar to monocytes, macrophages have also been associated with severity and pathogenesis in COVID-19 [1376, 1391].

#### 1.4 Human dendritic cells

##### 1.4.1 Introduction.

DC are renowned for their ability to present Ags to T cells. To test the ability of T cells to respond to DC subsets, an allogenic mixed lymphocyte reaction can be used. In this assay, naïve CD4^+^ T cells from one donor are co-cultured with DCs from another donor for 6 days, after which T cell proliferation is measured.

##### 1.4.2 Step-by-step sample preparation.

For step-by-step sample preparation please refer to see section IX.1.3.2, and follow the steps provided there for tissue processing and isolation of human DCs, as the procedures are the same. Once a single cell suspension is reached, proceed with the following assay steps:

1. Isolate naive CD4^+^ T cells from PBMCs using the “Naive CD4^+^ T Cell Isolation Kit II” (Miltenyi Biotec).
2. Incubate up to 10^6^/ml T cells in a tube with 5 μM CellTrace Violet dye in PBS for 20 min at 20 °C, in the dark.
3. Add 5 times the original staining volume of FACS buffer and incubate for a further 5 min.
4. Centrifuge at 300 g for 5 min, at 20 °C, aspirate supernatant.
5. Resuspend cell pellet in Iscove’s Modified Dulbecco’s Medium (IMDM) supplemented with 10 % KnockOut Serum Replacement.
6. Add 5’000 FACS-sorted DCs to every 25’000 CellTrace Violet-labeled naive CD4^+^ T cells in a 96-well plate in IMDM + 10 % KnockOut Serum Replacement
7. Incubate for 6 days at 37 °C, 5 % CO_2_.
8. On day 6, proceed with flow cytometry staining as described in sections IX.1.1.2.5 and IX.1.3.3.2 and analyze T cell proliferation by flow cytometry.

##### 1.4.3 Materials.

For general reagents and materials needed for functional assays for human DC please see the information provided in see section IX.1.1.3.1 (human monocyte isolation and phenotyping). Additional reagents and Abs are listed in Tables 118 and 119.

##### 1.4.4 Data analysis.

**Figure 133.**
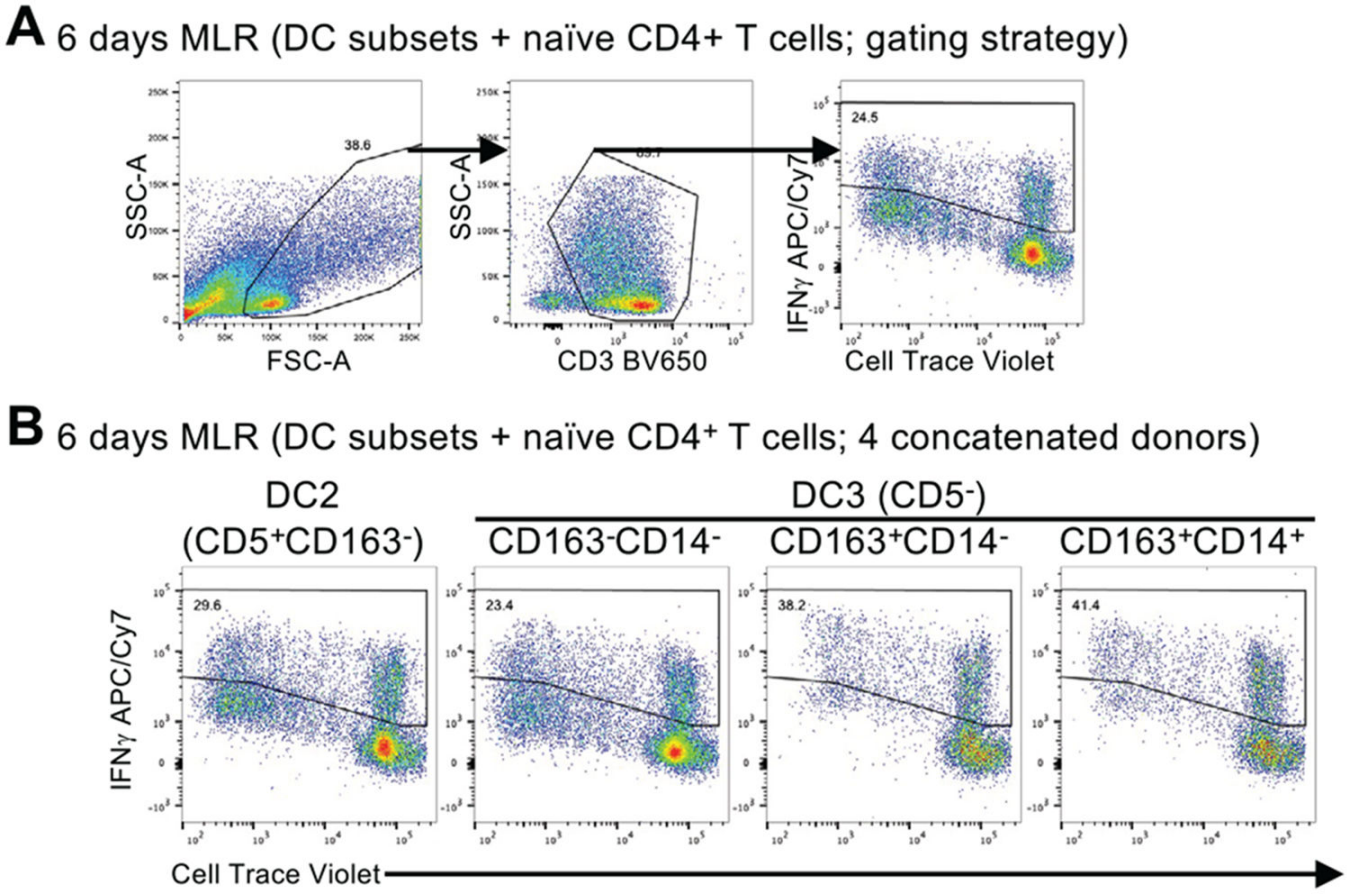
Flow cytometric analysis for allogenic mixed lymphocyte reaction assay using human PBMCs. (A) Show is a gating strategy to identify blood T cells and measure IFNγ production and proliferation. (B). As an example, the differences in proliferating IFNγ^+^ T-cells when co-cultured with DC2127 **2** (Flow cytometric analysis of human dendritic cells).

As shown in Figure 133, human blood T cells stimulated with human blood DC2 and DC3 subsets can be examined for proliferation and IFNγ production. (**A**) On day 6 cultured cells are analyzed by flow cytometry and gated on CD3^+^ T cells which are assessed for production of IFNγ and their proliferation capacity, as tracked by Cell Trace Violet. (**B**) On day 6 cultured CD3^+^ T cells are analyzed by flow cytometry for production of IFNγ and their proliferation capacity after co-culture with either DC2 or DC3 subsets. Basic gating of human blood DC subsets is shown in Figure 127.

##### 1.4.5 Pitfalls.

- It is important to work as sterile as possible, as contamination, e.g., with LPS or bacteria may lead to altered DC activation status and affect the outcome of the assay.

##### 1.4.6 Top tricks.

- Consider using T cells cultured in the absence of DC for a control. In addition, anti-CD3 and anti-CD28 can be used as a positive control for T cell proliferation.

##### 1.4.7 Clinical relevance statement.

For the clinical relevance of dendritic cells, please see chapter 1.3.6.

The MLR test is frequently used in clinic settings to assess host-graft compatibility e.g., for organ or bone marrow transplantation to identify suitable donors. Additionally, the MLR test is routinely used by pharmaceutical companies to demonstrate whether a novel drug, vaccine or material meant for implantation into patients is safe for use.

### 2 Murine mononuclear phagocytes: Monocytes, macrophages, and dendritic cells

#### 2.1 Overview

As monocytes, macrophages, and DCs play important roles in tissue maintenance, immune activation and control during homeostasis, infections and non-infectious diseases, dietary changes, and many others, studying the function of these cells is rather important. Thus, this chapter aims to provides suggestions for basic functional assays to study murine mononuclear phagocyte functions and responses.

#### 2.2 Murine monocytes

##### 2.2.1 Introduction.

In general, as human and murine monocytes are very comparable, a similar assay to the one described for human monocytes can be performed for murine monocytes. Thus, we refer the reader to section X.1.2 for culture details of LPS stimulated monocytes. Additionally, instead of using *ex vivo* monocytes, mice could be injected e.g., intraperitoneally with TLR ligands such as LPS, CpG, Poly(I:C) or others to activate and analyze the *in vivo* functional properties monocytes, and other mononuclear macrophages such as macrophages or DCs. The readout for all three cell types is very similar and could involve staining for cytokines, such as TNF-α as described for human monocytes, or surface activation markers, such as CD40, CD80, CD86 or MHCII. Plenty of examples for *in vivo* monocyte functional readouts can be found in published literature, e.g., in the context of infection [1437-1440], diet [1441], and others.

##### 2.2.2 Step-by-step sample preparation.

For step-by-step sample preparation please refer to in chapter IX.2.1.2, and follow the steps provided there for tissue processing and isolation of murine monocytes, as the procedures are the same. For *in vitro* stimulation of monocytes (or mononuclear phagocytes in general), follow the steps described in X.1.2.2 for stimulation of human monocytes (using the extracellular staining protocol for murine monocytes described in section IX.2.1.2.8).

##### 2.2.3 Materials.

For general reagents and materials needed for the isolation and phenotyping of murine macrophages please see the information provided in 2.1 (murine monocyte isolation and phenotyping). For reagents needed for the *ex vivo* culture and stimulation of murine monocytes please refer to table 117.

##### 2.2.3.1 Antibodies for murine monocyte assay.

For Abs used to identify monocytes please refer to table 108; additional Abs for surface activation markers may be used such as those listed in Table 120.

##### 2.2.4 Data analysis.

For data analyses follow the gating strategy for murine monocytes as shown described in section IX.2.1.4 and Figure 128. Subsequently, once gate on monocyte population one can check their activation levels by plotting histograms of the stained surface activation markers, e.g., CD40, CD80 or CD86, or cytokine production by plotting TNF-α^+^ cells, similar to what is shown in Figure 132 for human TNF-α^+^ monocytes.

##### 2.2.5 Pitfalls.

- Note that *ex vivo* cultured mononuclear phagocytes over time often may become “pre”-activated under culture conditions, thus the baseline level of surface expression of CD80, CD86, etc. may already be higher than their *in vivo* levels.

##### 2.2.6 Top tricks.

- We highly recommend to include a non-activated or non-stimulated control sample as it is inevitable for a conclusive readout.

#### 2.3 Murine dendritic cells

##### 2.3.1 Introduction.

As professional APCs DCs connect the innate and adaptive immune system, and are crucial for most immune responses. DCs have been implicated in pretty much all viral infections, autoimmune deficiencies, cancer, tissue homeostasis and others. They often are used as targets for vaccine and therapy strategies and thus, studying their specific functions is of high interest. Murine DCs are comparable to their human counter parts. Thus, the same MLR assay used for human DCs can be used for murine DCs. Please follow the information and step-by-step guidance for the MLR assay provided in chapter X.1.4.

##### 2.3.2 Step-by-step sample preparation.

For step-by-step sample preparation please refer to section IX.2.1.2, and follow the steps provided there for tissue processing and isolation of murine monocytes, as the procedures are the same.

Additional steps for the mixed lymphocyte reaction assay can be found in X.1.4.2.

##### 2.3.3 Materials.

For general reagents and materials needed for functional assays for murine dendritic cells please see the information provided in section IX.2.1.3.1 (murine monocyte isolation and phenotyping). Additional materials needed for this assay is given in Table 121.

##### 2.3.4 Data analysis.

Please follow the gating strategies provided in section IX.2.3.4, Figure 130 or Figure 131 and section X.1.4.4.

##### 2.3.5 Pitfalls.

- For pitfalls on this assay, we refer the reader to section X.1.4.5.

##### 2.3.6 Top tricks.

- For tricks on this assay, we refer the reader to chapter X.1.4.6.

## XI Granulocyte phenotypes

### 1 Neutrophil, eosinophil, and basophil phenotypes

#### 1.1 Overview

In this section, we will provide protocols to classify human and mouse granulocyte subpopulations by flow cytometry discrimination.

Granulocytes are sensitive cells, which rapidly die or aggregate upon inappropriate treatment. Therefore, it is necessary to use optimized protocols for the dissociation of different tissues to prepare cell suspensions for flow cytometry. However, the easiest way to obtain granulocytes for analysis is to use whole blood (human and mouse) and perform lysis of erythrocytes. Successful flow cytometry analysis requires high quality single cell suspensions. Minimal manipulation of the cells is essential for the quality of both Ab and cell death staining.

#### 1.2 Introduction

Human granulocytes are present in peripheral blood and can be isolated via density centrifugation or analyzed as a subpopulation of total leukocytes. The most abundant granulocyte or polymorphonuclear leukocyte (PMN) in human blood is the neutrophil granulocyte, whereas eosinophils and basophils can only be found in small percentages in healthy individuals. Neutrophils are our first line of defense against invading pathogens, whereas eosinophils play a crucial part in the killing of parasites and in allergies. Basophils are the least abundant type of granulocytes and thought to contribute to the inflammatory response by the release of histamine. Some inflammatory disorders are characterized by low-density granulocytes (LDNs) that co-localize with peripheral blood mononuclear cells (PBMC) during density centrifugation. These cells display an increased potential to produce inflammatory cytokines and higher capacity for NET formation. Thus, they can contribute to the pathogenesis of several inflammatory diseases, like systemic lupus erythematosus (SLE) and coronavirus disease 2019 (COVID-19) [1442, 1443]. However, low-density granulocytes with immunosuppressive functions (either low-density neutrophils or granulocytic myeloid-derived suppressor cells (MDSCs) have also been described in the literature, for example in cancer patients [1444].

Compared to humans, the percentage of PMN in the peripheral blood of mice is lower, therefore obtaining granulocytes from peritoneal lavage, for example after intraperitoneal injection of thioglycollate, or from bone marrow (see section XII.2 Murine bone marrow stromal cells) might be preferred to whole blood isolation. In some cases, enrichment for granulocytes might be necessary and this can be achieved via density centrifugation or negative selection via magnetic beads. For flow cytometry analysis, the initial cell suspension should be depleted of erythrocytes (e.g., short hypotonic/osmotic lysis or use of commercially available red blood cell lysis buffers).

#### 1.3 Step-by-step sample preparation of human and murine granulocytes

1. 100 μl human or murine whole blood per condition is pelleted via centrifugation at 300 g for 5 min. The cell pellet is resuspended in a small volume (100 μl) and subjected to hypotonic/osmotic lysis for 20 s for erythrocyte lysis. Physiological osmolality is re-obtained by addition of an appropriate volume of 10 x PBS.
2. Cells are pelleted via centrifugation at 300 g for 5 min and resuspended in PBS (with 10% heat inactivated “low endotoxin” FBS, 5 mM EDTA and Abs) at a concentration of 1 x 10^6^ cells per ml. The samples are incubated for 30 min on ice in the absence of light.
3. Cells are pelleted via centrifugation at 300 g for 5 min and resuspended in PBS (with 10% heat inactivated “low endotoxin” FBS, 5 mM EDTA) and subjected to flow cytometry analysis.

#### 1.4 Step-by-step sample preparation of human low-density neutrophils

1. 20 ml of anti-coagulated blood is diluted with 10 ml PBS and layered on top of 15 ml Lymphoflot. After centrifugation at 350 g for 30 min without brake, the cells are separated.
2. PBMC and PMN layers are collected treated with water for hypotonic/osmotic lysis of erythrocytes. This reaction is stopped by adding the required amount of 10 x PBS to regain physiological osmolarity. This step is performed twice for the PMN fraction.
3. The samples are centrifuged and resuspended in FACS buffer (PBS with 10% FBS and 5 mM EDTA). Cells are stained with Abs for 30 min on ice and subjected to flow cytometry analysis.

#### 1.5 Step-by-step sample preparation for analysis of cell death in human granulocytes

1. 20 ml of anti-coagulated blood is diluted with 10 ml PBS and gently layered on top of 15 ml Lymphoflot. Cells are separated via centrifugation at 300 g for 30 min without break. The granulocytes layer on top of the buffy coat is collected.
2. The cells are subjected to hypotonic/osmotic water lysis for 20 s for erythrocyte lysis. Physiological osmolality is re-obtained by addition of an appropriate volume of 10 x PBS.
3. After centrifugation at 300 g for 5 min, the granulocyte cell pellet is resuspended in RPMI-1640 supplemented with 100 U/ml penicillin/streptomycin, 2 mM glutamine and 10% heat-inactivated “low-endotoxin” FBS and 25 mM HEPES at a concentration of 2 x 10^6^ cells/ml and cultivated at 37 °C / 5% CO2. Due to the short life span of granulocytes, detectable cell death will occur in less than 12 h.
4. Cell death is assessed by harvesting of cells via centrifugation at 300 g for 5 min and resuspension at a concentration of 1 x 106 cells per ml in Ringer solution, 100 ng/ml propidium iodide and 1 μg/ml ANX-V Staining is performed on ice for 30 min.
5. Without an additional washing step, samples are directly subjected to flow cytometry analysis. Note that washing is not recommended as this can result in the loss of subcellular particles and compromise integrity of apoptotic cells.

#### 1.6 Step-by-step sample preparation for analysis of particle uptake in human granulocytes

1. 20 ml of anti-coagulated blood is diluted with 10 ml PBS and gently layered on top of 15 ml Lymphoflot. Cells are separated via centrifugation at 300 g for 30 min without break. The granulocytes layer on top of the buffy coat is collected.
2. The cell pellet is resuspended in a small volume and subjected to hypotonic/osmotic water lysis for 30 s for erythrocyte lysis. Physiological osmolality is re-obtained by addition of an appropriate volume of 10 x PBS.
3. The granulocytes are resuspended in in HBSS supplemented with 2% heat inactivated FBS. 20 μg/ml micro monosodium urate crystals and 250 μg/ml Lucifer Yellow are added and cells are incubated at 37 °C / 5% CO2 for various time points.
4. Cells are collected and without additional washing directly subjected to flow cytometry analysis.

#### 1.7 Materials

##### Reagents

HBSS, calcium, magnesium, no phenol red (ThermoFisher Scientific, 14025050)

Lymphoflot (Bio-Rad, #824012)

Lucifer Yellow CH (ThermoFisher Scientific, L453)

RPMI 1640 Medium (ThermoFisher Scientific, 21875034)

L-Glutamine (ThermoFisher Scientific, 25030081)

Penicillin-Streptomycin (ThermoFisher Scientific, 15140122)

HEPES (ThermoFisher Scientific, 15630056)

Gibco^™^ Fetal Bovine Serum (ThermoFisher Scientific, 10270106)

Ringer Fresenius (Fresenius Kabi, B23155A)

Propidium iodide (Sigma, P4170)

Anx-V-FITC (Immunotools, 31490013)

##### Antibodies

This is detailed in Tables 122 and 123.

#### 1.8 Data analysis

**Figure 134.**
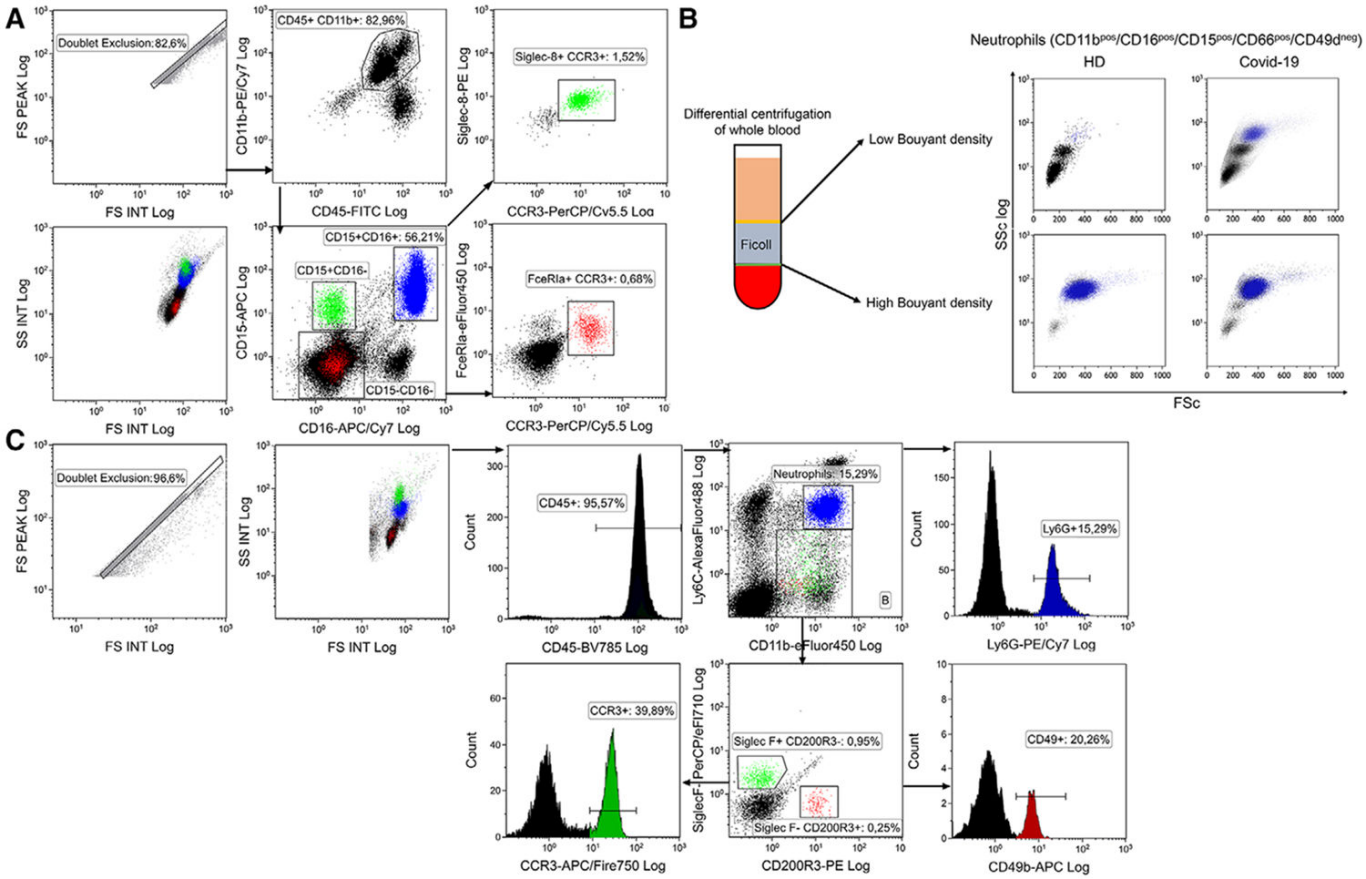
Discrimination of human and murine granulocyte subpopulations. A: Human cells after RBC lysis were displayed in a SSC versus (vs) FSC dot plot to show the location of eosinophils (green, high SSC), neutrophils (blue, high SSC), and basophils (red, low SSC). The following Abs CD45, CD11b, CD15, CD16, CCR3, Siglec-F and FcεRIα were used. CD45^+^/CD11b+ cells were gated on CD15 vs CD16 to distinguish granulocyte subpopulations. CD15+/CD16+ cells were determined as neutrophils, CD15^+^CD16− were further designated as eosinophils by their expression of Siglec-8 and CCR3, and the CD15−/CD16− population was depicted in a FcεRIα vs. CCR3 plot to identify the double positive basophil fraction. B: Human neutrophils with low buoyant density are observed in COVID-19. The following Abs CD45, CD11b, CD15, CD16, and CD49d were used after density centrifugation in both cellular fractions to identify neutrophils (blue). C: CD45^+^ murine cells were gated on CD11b/Ly6C to display the CD11b+/Ly6int population which was further analyzed using Ly6G to identify neutrophils (blue). CD11b+/Ly6Cneg-low cells were gated on Siglec-F vs. CD200R3 and were subsequently analyzed for expression of additional cell subset markers. CD200R3-cells expressing Siglec-F and CCR3 were designated as eosinophils (green) and Siglec-F-cells were marked as basophils (red) supported by their expression of CD200R3 and CD49b.

Differential light scattering of cells depending on the size and morphology is useful to discriminate subsets of cells. The SSC is considered to be an indicator for the internal structure of the cell (e.g., nuclear morphology) and the FSC reflects cellular size. Since neutrophils and eosinophils have a multi-lobulated nucleus, they exhibit a high SSC signal, with eosinophils showing a slightly higher signal in this parameter. The nuclear morphology of basophils is less complex and therefore they are found among the lymphocyte population and cannot be distinguished in such manner (Figure 134A). Furthermore, changes in SSC and FSC may also represent other morphological features of various cellular processes (e.g., phagocytosis, cell death). These changes can also be detected in this manner as described below in this section. It is recommended to start your analysis with excluding doublets by gating on FS PEAK Log vs. FS INT Log to prevent an impact of doublets on e.g., population frequencies. Then, to detect either human or murine granulocytes it is useful to start with a staining for CD45 to define white blood cells accompanied by the simultaneous staining for CD11b. These two markers together with FSC and SSC features are enough to roughly narrow down granulocytes from whole blood preparations (Figure 134A and Fig. 134C). Human neutrophils are the most abundant cell type within the granulocyte family. They can be further distinguished from other granulocytes by their positivity for both CD15 and CD16. Eosinophils are positive for CD15, but do not express CD16. Additional staining for CCR3 and Siglec-8 allows a specific detection of eosinophils. Basophils neither express CD15 nor CD16; therefore, staining with anti-FcεRIα identifies them in the CD15^neg^/CD16^neg^ population (Figure 134A).

Murine neutrophils and eosinophils are CD11b positive and exhibit an intermediate to low expression of Ly6C. Neutrophils are detected as Ly6G positive cells, whereas eosinophils are identified by their expression of CCR3 and Siglec-F. Basophils also show positivity for CD11b, but have only a low expression of Ly6C. They can be further identified by the expression of CD200R3 and CD49b (Figure 134C).

For identification of low buoyant density neutrophils in the PBMC fraction, the same gating strategy can be used as for the determination of high buoyant density neutrophils in the PMN fraction. By gating SSC vs FSC, both cell populations appear at the same level of granularity and size. These low-density cells can barely be observed in healthy donors, but occur more frequently in several diseases that exhibit inflammation, as shown here for COVID-19 (Figure 134B). Neutrophils identified this way can then be further specified using Abs against CD45, CD11b, CD15, CD16 and CD49d, as presented in Figure 134A.

**Figure 135.**
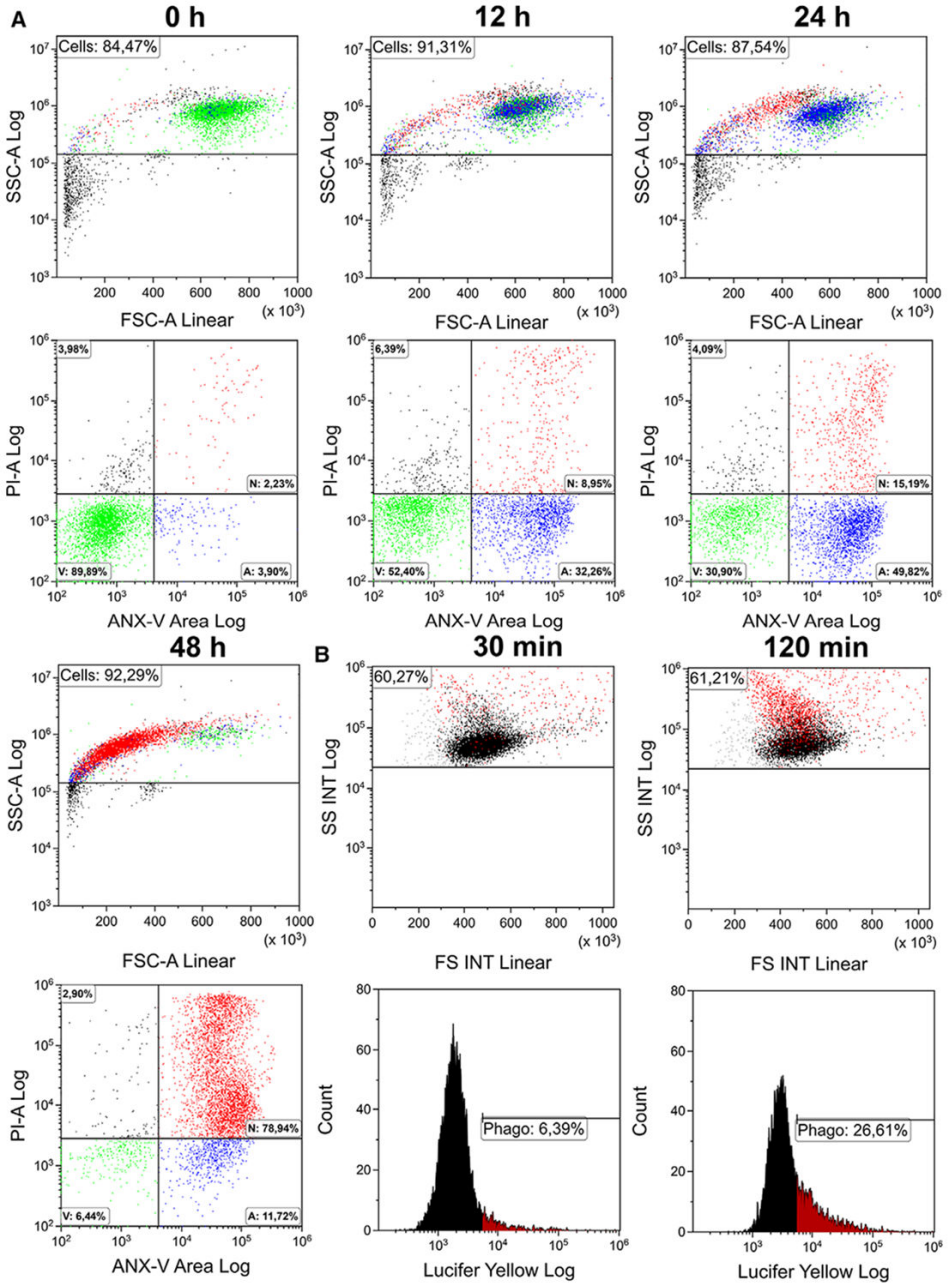
Apoptosis detection and uptake of nanoparticles in purified human granulocytes. A: Granulocytes were cultivated at 37 °C/ 5% CO_2_ for indicated time points and stained according to the cell death protocol. Subsequently, they were subjected to flow cytometry analysis. During apoptosis, granulocytes shrink and increase in granularity, as indicated by a decrease in FSC and an increase in SSC. Viable cells (V) first start to expose ANX-V-FITC and become apoptotic (A), before they lose their plasma membrane integrity and become necrotic as indicated by PI-positivity (N). Note that in the N-gate the population high in PI reflects cells without the loss of nuclear content. In contrast, the population low in PI reflects cells with a subG1 DNA content, which is considered a hallmark of apoptosis. B: 20 μg/ml micro monosodium urate crystals and 250 μg/ml Lucifer Yellow were added to the granulocytes and the suspension was incubated at 37 °C/ 5% CO2 for the time points indicated. Subsequently, flow cytometry analysis was performed. The increase in Lucifer Yellow (see arrow; in red) is restricted to the population of cells which increase in granularity. Therefore, the simultaneous increase in Lucifer Yellow and SSC can be used to monitor the uptake of nanoparticles by granulocytes.

Especially in the context of inflammatory infiltrates it is sometimes necessary to further determine neutrophil viability. During the resolution of inflammation, neutrophils undergo apoptosis, mediate anti-inflammatory and immunosuppressive effects, and secrete factors that prevent the additional influx of neutrophils. Apoptosis and necrosis can be detected by a combination of PI and fluorophore-conjugated ANX-V. PI is a DNA-intercalating substance that only enters cells that have lost their membrane integrity (necrotic cells and NETotic cells). ANX-V binds to PS exposed by cells undergoing apoptosis (Figure 135A). If granulocytes have been purified prior to the L/D analysis, no ab staining is needed. However, if more than one cell type is present, the cell death staining should be supplemented with an ab combination allowing the identification of granulocytes as mentioned above. flow cytometry allow the simultaneous use of multiple fluorophores. If such an instrument is available, the classical apoptosis staining deploying ANX-V-conjugates and PI can be supplemented with two additional dyes (e.g., Hoechst33342 and 1,1′,3,3,3′,3′-hexamethylindodicarbo-cyanine iodide (DilC1(5)) that allow a more detailed characterization of cell death. This staining takes into account the condition of the nucleus and the mitochondrial membrane potential, respectively and can also be deployed for live-cell imaging [1445, 1446].

Further, neutrophils show a strong capacity to take up particulate matter. If confronted with nanoparticles or small-sized monosodium urate crystals, neutrophils engulf these particles and respond in an appropriate manner. Since such material cannot be easily conjugated with fluorophores, one has to rely on other methods to monitor their uptake. Soluble dyes, such as Lucifer Yellow, can be added together with the prey which will subsequently be co-ingested during phagocytosis. In addition, the uptake of particulate matter tends to increase the complexity of the phagocyte. As shown in figure 135B, the increase in SSC and in Lucifer Yellow strongly correlates. Combined observation of both represents a feasible method for addressing such questions.

#### 1.9 Pitfalls

- Neutrophil released from the BM are following a circadian rhythm [1447]. To ensure the highest comparability, neutrophils from different donors (murine and human) should be isolated roughly at the same time of the day.
- When flow cytometry analysis is performed, proper arrangements are necessary to prevent neutrophil adhesion. Neutrophils show a tendency to adhere under serum free conditions, to glass or adhesive plastic surfaces and especially fast in response to stimulation.
- Neutrophils have a very limited life time. They undergo full blown apoptosis in less than 24 h. In addition, several stimuli induce the formation of neutrophil extracellular traps. Although it is possible to detect NETs as material with very high SSC, flow cytometry is not robust enough to quantify NETs. Furthermore, NETs tend to aggregate and form material which cannot be collected by standard needles.

#### 1.10 Top tricks

- Neutrophils are susceptible to changes in pH and readily form NETs even under mildly alkaline conditions. Buffers should be checked for pH prior to use. RPMI and HBSS can be supplemented with HEPES to stabilize the pH [1448].
- Phagocytic uptake of particles alters the morphology of a variety of cell types. It is therefore advisable to identify granulocyte populations not only by SSC but also by the distinct markers listed in Table 124.
- Activation of leucocytes is usually accompanied by shedding or membrane renewal consequently changing their phenotype (e.g., CD16 downregulation).
- L/D stainings deploying ANX-V must be performed in the presence of at least 2 mM calcium, since binding of ANX-V to PS is calcium-dependent. Avoid washing to prevent loss of subcellular particles and impairment of apoptotic cell integrity.

#### 1.11 Clinical relevance statement

The gating strategy shown in this section is applicable for analysis of granulocytes (neutrophils, basophils and eosinophils) in health and disease. This gating strategy was used to characterize low-density neutrophils in patients with COVID-19 [1443]. The identification and characterization of neutrophil functions in experimental murine models [1449, 1450] can take advantage of the gating strategy proposed in this section. The quantification phagocytosis of particulate matter by neutrophils was helpful to demonstrate the engulfment of monosodium urate crystals in various gout studies [1451-1454]. The description of molecular biology processes like the NALP3 inflammasome activation [1455] and the clearance of nanoparticles from the body [1456, 1457] was conducted on the basis of the confirmation of engulfment. Finally, apoptosis of neutrophils is an ancient process involved in the resolution of inflammatory responses. The phenotypic characterization of this phenomenon has shed lights on the importance of an ordered cellular demise for the maintenance of tissue homeostasis [1458-1461].

#### 1.12 Key information human vs murine

This is detailed in Tables 124 and 125.

The biggest difference between human and murine cells is seen in the neutrophil percentage in peripheral blood. This is detailed in Table 125 (with Ref. [1462]).

### 2 Human bone marrow and cord blood neutrophils

#### 2.1 Overview

In this section, we describe the complete flow cytometric characterisation of human neutrophil development in both human bone marrow and umbilical cord blood samples. We provide the complete list of markers required to remove contaminating populations and isolate pure populations of neutrophil subsets for downstream sorting and assays.

#### 2.2 Introduction

Granulocytes are the granule-producing branch of the myeloid cell lineage which includes neutrophils, eosinophils and basophils [1463]. Neutrophils represent the large majority of granulocytes and are involved in a myriad of immune functions and diseases [1464-1466]. Flow cytometric analysis and characterisation of neutrophils has been performed over 20 years ago [1467]. The stages of granulopoiesis - myeloblasts, promyelocytes, myelocytes, metamyelocytes, band cells and segmented neutrophils – have been characterized by the use of markers such as CD11b, CD15 and CD16. Traditionally, these analyses were performed by correlating surface marker expression levels with the morphological characteristics of the different stages of terminal granulopoiesis [1468, 1469].

Neutrophil heterogeneity in disease has been of a growing interest with the introduction of neutrophil subsets with the underappreciated roles previously [1466, 1470, 1471]. Many of these reports suggest an immature phenotype of granulocytes [1472-1475], suggesting the importance of investigating neutrophil function in relation to the various developmental stages. Moreover, recent studies show that expression of subset-specific neutrophil granules can also help regulate neutrophil function [1476]. Therefore, understanding the stages of neutrophil maturation provides a firm foundation to study these novel functions of neutrophils. Indeed, recent evidence shows how clearly defined subsets of neutrophils can specifically perform distinct functions that influences the disease progression of arteriosclerosis [1477]. In this section, we provide guidelines in analyzing neutrophil subsets characterized by their distinct functions and the roles they play during inflammatory states [1478, 1479].

#### 2.3 Step-by-step sample preparation

##### 2.3.1 Human bone marrow neutrophils.

1. Collect donor bone marrow aspirate in heparin saline containing ACD-A (acid-citrate-dextrose formula A).
2. Wash sample aliquot with 2 mL of 1 X PBS. Centrifuge at 4°C, 400 × *g* for 5 min. Discard supernatant. Perform cell count.
3. Using 5 million cells, block Fc-receptors using purified anti-human CD16/32 Ab for 30 minutes at 4°C. Add staining buffer containing Abs. Incubate at 4°C for 30min.
4. Wash sample with PBS and centrifuge at 4°C, 400 × *g* for 5 min. Discard supernatant.
5. Lyse erythrocytes with 500 μL 1× RBC lysis buffer for 3 minutes. Wash.
6. Add DAPI and acquire cells.

##### 2.3.2 Human cord blood neutrophils.

1. Lyse erythrocytes with 1 mL of 1 X RBC buffer for every 100 μL of cord blood. Incubate at room temperature for 6 minutes or until sample becomes translucent.
2. Neutralise with 1 X PBS. Centrifuge at 4°C, 400 × *g* for 5 min.
3. Using 5 million cells, block Fc-receptors using purified anti-human CD16/32 Ab for 30 minutes at 4°C. Add staining buffer containing Abs. Incubate at 4°C for 30min.
4. Wash sample with PBS and centrifuge at 4°C, 400 × *g* for 5 min. Discard supernatant. Add DAPI and acquire cells.

#### 2.4 Materials

##### 2.4.1 General reagents.

- Dulbecco’s phosphate-buffered saline with calcium and magnesium (PBS)
- Wash buffer: PBS with 2% heat-inactivated fetal calf serum (FCS) and 2 mM EDTA.
- 70 μM Cell-strainer mesh
- 1 X RBC lysis buffer (eBiosciences)

The staining reagents for human neutrophils are listed in Table 126.

##### 2.4.2 Flow Cytometer.

All experiments were performed on a LSRII flow cytometer with a 365nm, 405 nm, 488 nm, 561 nm and 640 nm configuration (BD Bioscience). Filters: 379/34(365) for BUV395; 740/35(365) for BUV737; 450/50(365) for DAPI; 530/30(488) for FITC or AF488; 685/35(488) for PerCP-Cy5.5; 450/50(405) for BV421 or SB436; 525/50(405) for BV510 or V500; 660/20(405) for BV650; 710/40(405) for BV711; 800/50(405) for BV785; 585/15(561) for PE; 610/20(561) for PE-Texas Red; 780/60(561) for PE-Cy7; 675/20(640) for APC or AF647; 730/45(640) for AF700 or APC-R700; 780/60(640) for APC-eF780 and APC-Cy7.

#### 2.5 Data analysis

**Figure 136.**
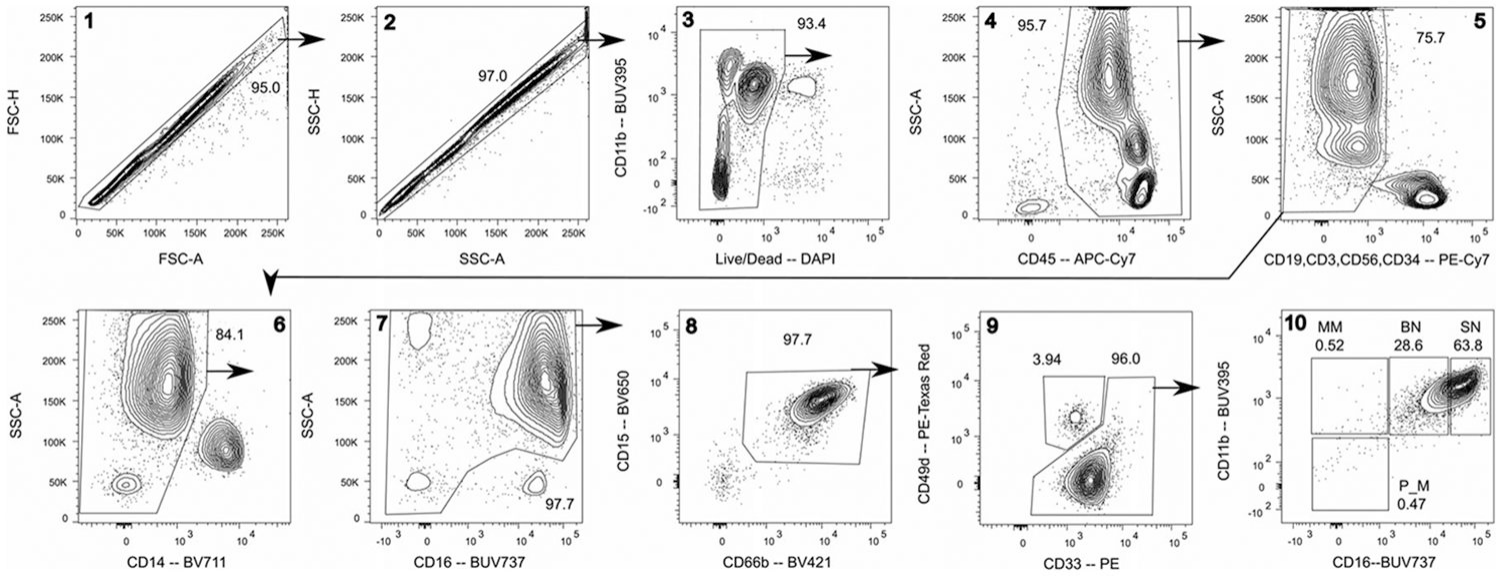
Gating of human umbilical cord blood neutrophils. Flow cytometric analysis of human umbilical cord blood neutrophil subsets. Doublets and dead cells are first excluded, followed by the exclusion of CD45^−^ cells. Lineage cells and monocytes are excluded before total granulocytes are gated with CD15 and CD66b. From there, eosinophils are excluded before gating on the classical nomenclature of neutrophil subsets using CD11b and CD16. P_M = Promyelocytes and Myelocytes, MM = Meta-myelocytes, BN = Band cells, SN = Segmented neutrophils. Gating is adapted from [1469, 1480].

**Figure 137.**
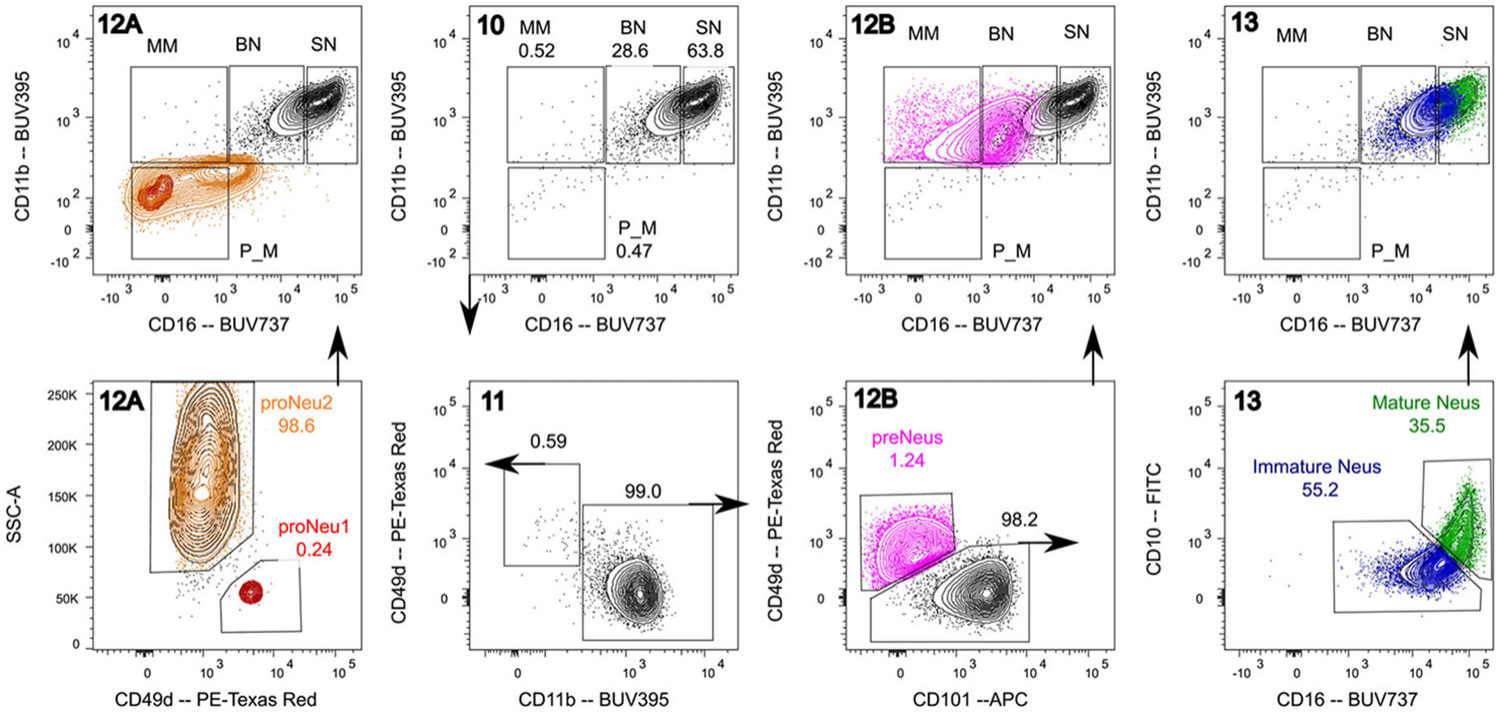
Alternative gating strategy to classical nomenclature for identifying human neutrophils. An alternative to using CD11b vs CD16 (Figure 136, panel 10), total human cord blood neutrophils can be divided into CD11b^−^ neutrophil progenitors and CD11b^+^ neutrophils [1479, 1481]. The proliferative CD11b^+^ preNeus can be separated using CD101 and CD49d as shown previously [1478]. The remaining non-proliferative pool of neutrophils can be separated with CD10 as described previously [1482].

The principle in this strategy (Figure 136, with ref. [1480]) is to remove all possible contaminants before gating on neutrophil subsets. To start, remove doublets by using the FSC-A vs FSC-H, followed by SSC-A vs SSC-H as second filter (panel 1 and 2). Next, remove dead DAPI-positive cells, followed by removing CD45-negative cells (panel 3 and 4). Then, gate out lymphocytes (CD19-B cells, CD3-T cells, CD56-NK cells) and CD14-positive classical monocytes thereafter (panel 5 and 6). From there, remove CD16-positive non-classical monocytes (panel 7). At this point, gate on CD15^+^CD66b^+^ total granulocytes (panel 8). Next, remove CD33^−^CD49d^+^ eosinophils (panel 9) to generate the total neutrophil gate which can then be divided into the various developmental stages shown (panel 10). Alternatively, Figure 137 (with refs. [1481, 1482] provides additional gates to further isolate neutrophil subsets. From the total neutrophil gate (panel 10), use CD11b and CD49d to isolate neutrophil progenitors (proNeu1 and proNeu2) (panel 11), which are distinguished using CD49d and SSC-A (panel 12A). Within total CD11b-positive neutrophils, use CD101 to demarcate preNeus from CD101^+^ immature and mature neutrophils (panel 12B). Finally, use CD16 and CD10 as markers to distinguish immature CD10^−^ neutrophils from CD10^+^ mature neutrophils (panel 13).

#### 2.6 Pitfalls

- Human neutrophils are sensitive to Ficoll, and will change the expression of certain markers. For example, Ficoll will down-regulate CD49d expression which prevents the isolation of proliferative preNeus from immature Neus.
- Eosinophils can be a big source of contamination as they share many markers like CD15 and CD11b with neutrophil subsets. They are CD101^+^CD49d^+^CD16^−^ and express Siglec-8. Gating them out is essential, especially in eosinophilic patient samples.

#### 2.7 Top tricks

- Keep samples on ice to prevent activation and marker expression shifts

#### 2.8 Clinical relevance statement

The gating strategy shown in this section is applicable for analysis of immature neutrophils in severe COVID-19 patients, as can be seen in [1483]. The key conclusion from such analysis is the association of disease severity and CD10^−^ immature neutrophil counts can be used as a biomarker for disease triage and potential pre-emptive treatment strategies.

#### 2.9 Summary of the phenotypes

This is detailed in Table 127 (with ref. [1484]).

### 3 Murine bone marrow neutrophils

#### 3.1 Overview

In this section, we provide the complete analysis of murine bone marrow neutrophil development, beginning from the earliest committed progenitor. We incorporated the necessary steps and markers required to phenotype all subsets within one panel. This enables us to look at neutrophil development in a comprehensive manner in both physiological and inflammatory states.

#### 3.2 Introduction

The developmental pathway of neutrophils has been recently investigated with great interest [1475, 1478, 1485]. New single-cell methods have further revealed heterogeneity and the identification of new subsets [1479, 1481, 1486, 1487]. However, there is still no universally accepted characterisation of neutrophils by flow cytometry. Moreover, tissues such as bone marrow and spleen contain multiple cell types, which may share overlapping surface markers that can lead to contamination and improper identification. Here, we propose a flow cytometry framework to identify and isolate pure populations of neutrophil subsets, which can be generally applied to most tissues in mice.

#### 3.3 Step-by-step sample preparation

1. Isolate femur bone with a scalpel by dislocating ball-socket joint at the hip. Detach kneecap joint connecting the tibia.
2. Clean off muscle tissue and cut off the ball of the femur to create an opening.
3. Using a 1mL syringe with a 19-gauge needle containing 1mL wash buffer (PBS +2% FCS + 2mM EDTA), flush marrow out through the opening into a 15mL falcon tube containing 1mL of buffer. Aspirate and repeat twice. Flush marrow from the opposite end. Aspirate and repeat twice.
4. Filter suspension through a 70 μM strainer to remove clumps and bone chips. Wash strainer with 4mL of buffer. Centrifuge cells at 4°C, 400 × *g* for 5 min.
5. Discard supernatant and re-suspend pellet with 1 mL of buffer. Aliquot a fraction out for staining purposes. One-fourth is typically adequate.
6. Wash and centrifuge sample aliquot with buffer. Discard supernatant and re-suspend cells in 50 μL blocking buffer. Add 50 μL of staining buffer with the Abs. Incubate at 4°C for 30min.
7. Add 2 mL of wash buffer and centrifuge. Discard supernatant.
8. Lyse erythrocytes with 200 μL 1× RBC lysis buffer for 3 minutes. Wash.
9. Re-suspend in wash buffer, add DAPI and filter sample before acquisition.

#### 3.4 Materials

The staining reagents for murine neutrophils are listed in Table 128.

#### 3.5 Data analysis

**Figure 138.**
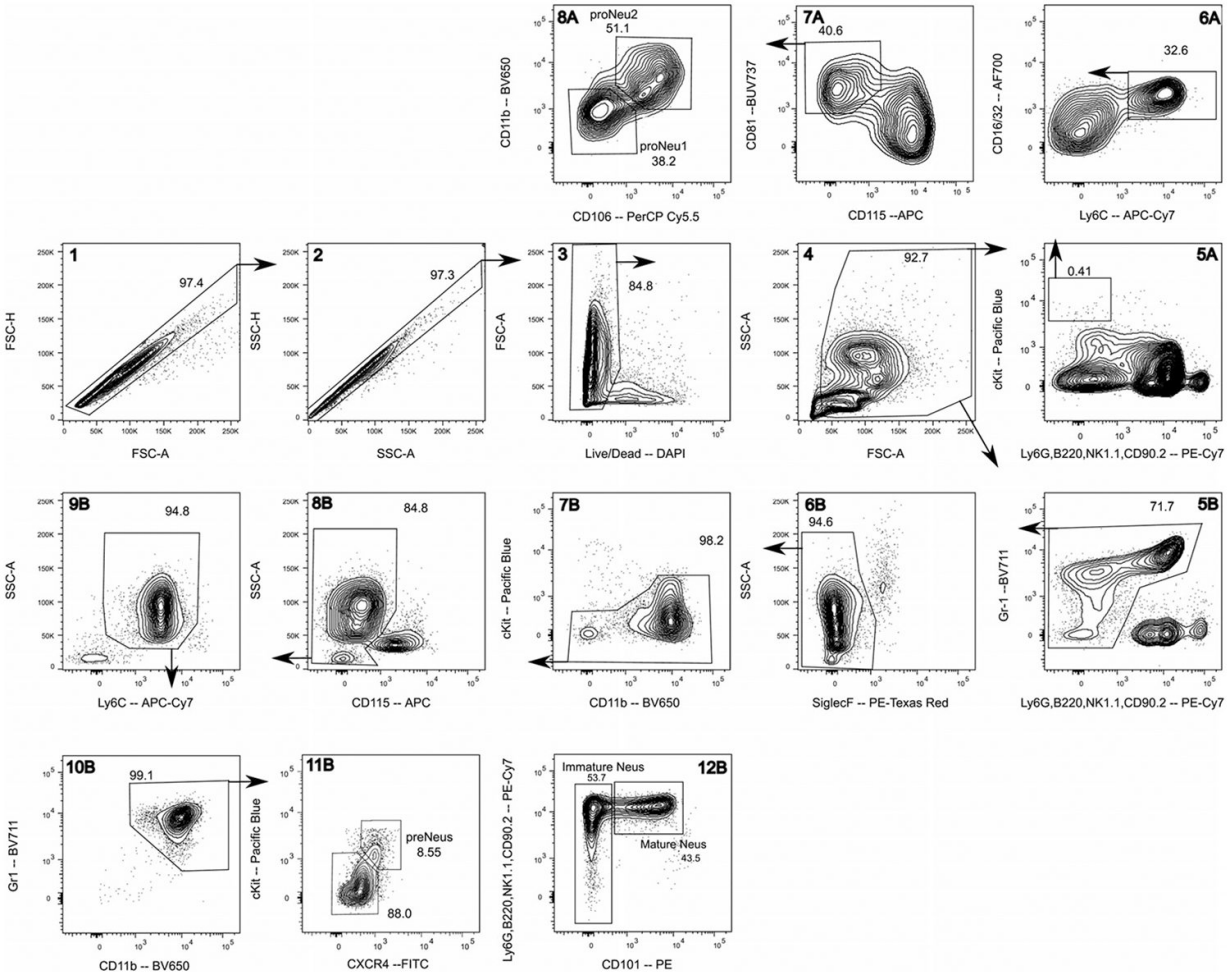
Flow cytometric analysis of murine bone marrow neutrophil subsets. Murine bone marrow samples are first gated to exclude doublets and dead cells. Debris are also excluded based on forward and side scatter information. Within the cKit^hi^ expressing cells, Ly-6C and CD81 are used to mark all neutrophil progenitors. CD106 distinguishes between pro-neutrophil 1 and pro-neutrophil 2. Other subsets are gated by first removing lineage positive cells (T, B and NK cells) followed by the exclusion of eosinophils and monocytes. Ly6C is then used to further remove any Ly-6C^hi^ and Ly-6C^lo^ monocyte contamination. Gr-1 and CD11b gates for total bone marrow neutrophils. cKit and CXCR4 is used to gate proliferative pre-neutrophils and CD101 distinguishes mature neutrophils from immature neutrophils.

Similar to the human neutrophil analysis, the strategy (Figure 138) is to first remove contaminating immune cell types before identifying neutrophil subsets. First remove doublets, dead DAPI-positive cells and debris before further analysis (panel 1-4). To identify neutrophil progenitors, select only cKit^hi^Sca-1^−^ cells (panel 5). From there, gate on Ly-6C^+^CD16/32^+^ Granulocyte-Monocyte Progenitors (panel 6A) before gating on CD81^+^CD115^−^ neutrophil progenitors (panel 7A) which can be differentiated into a CD11b^−^CD106^−^ proNeu1 and CD11b^lo^CD106^+^ proNeu2 population (panel 8A). To gate for preNeus, lymphocytes are first removed (panel 6B), followed by eosinophils (panel 7B), cKit^hi^ cells and monocytes (panel 8B and 9B). Then, gate on total Gr-1^+^CD11b^+^ neutrophils (panel 10B) before gating on cKit^+^CXCR4^+^ preNeus (panel 11B), Ly-6G^+^ CD101^−^ immature neutrophils and Ly-6G^+^CD101^+^ mature neutrophils (panel 12B).

#### 3.6 Pitfalls

- Besides Ficoll, temperature can affect marker expression. Therefore, keep cells on ice throughout sample preparation as markers like CD115 will down-regulate.
- A good cKit staining is essential to demarcate proNeus from preNeus. Use the recommended Ab for optimal results. Performing any digestion protocols will affect marker expression and should be noted.

#### 3.7 Top tricks

- In certain inflammatory conditions, such as a bacterial challenge, neutrophils may lose Ly-6G expression. Make use of side-scatter information and Ly-6C expression level to gate for neutrophils.
- Perform titration of Abs for optimal staining index. Typically, 0.25ul is used for mouse Abs for 1/4 of femur marrow and 2uL per 5 million cells is used for human Abs.
- Bone marrow composition in tibias, humeri, pelvis and sternum are similar to the femur. Therefore, use these bones if large numbers of neutrophils are required for sorting purposes.
- Gr-1 labels both Ly-6G and Ly-6C. When staining, use twice the amount of Ly-6G to prevent competitive binding by Gr-1.

#### 3.8 Summary of the phenotypes

This is detailed in Table 129.

#### 3.9 Key information human vs murine

This is detailed in Table 130 and summarized here:

- Human neutrophils have unique granule proteins such as α-Defensin 3, Azurocidin and Bactericidal/permeability-increasing protein [1488].
- Murine neutrophils have unique granule proteins like Neutrophil Granule Protein, HMGB1 and Chitinase-like protein 3 [1488].
- Human neutrophils make up 50-70% of blood leukocytes while murine neutrophils only make up about 10-25% of blood leukocytes [1489].
- Human Neutrophils are larger, and forward and side scatter settings should be adjusted accordingly.
- CXCL8, or IL-8, is a strong chemokine that only exists in humans and interacts with receptors CXCR1 and CXCR2. In mice, CXCL1 (KC) and CXCL2 (MIP-2) are chemokines that perform similarly to IL-8 [1490, 1491].

## XII Bone marrow stromal cell phenotypes

### 1 Human bone marrow stromal cells

#### 1.1 Overview

In this chapter, we aim to provide basic guidelines for researchers interested in analyzing human bone marrow stromal cells. We describe protocols for harvesting human bone marrow from femur head, the isolation and culture of human bone marrow stromal cells, and a flow cytometry staining approach. Furthermore, we provide a basic gating strategy as well as insights into alternative methodologies and how to reduce inter-experimental variability.

#### 1.2 Introduction

In 1966, Friedenstein and colleagues first isolated mesenchymal stem cells from rat bone marrow [1492]. Although given the name “mesenchymal stem cells”, many researchers believe the name is not appropriate given the fact that mesenchymal stem cells are not necessarily functional stem cells [1493]. Functional stem cells have *in vitro* differentiation mimicking *in vivo* differentiation; however, mesenchymal stem cells do not necessarily behave in the same way. Thus, the International Society for Cellular Therapy (ISCT) suggests the abbreviation “MSC” stand for mesenchymal stromal cell [1494].

MSCs are a heterogeneous cell population with great diversity and numerous vital roles in health and disease [1495]. More specifically, MSCs are a type of stromal cell and are ideal candidates for autologous cell therapy. This therapy is advantageous because it facilitates isolation, modification, and re-implantation while avoiding host immune rejection. Contributing to the ease of autologous therapy is the fact that MSCs can be isolated from many places in the body to include: bone marrow, adipose tissue, dental pulp, umbilical cord blood, placenta [1496], heart and other non-mesenchymal vascularized sources [1497]. However, until now bone marrow MSCs, which are the topic of this chapter, are the most studied MSCs. Lastly, and in addition to the aforementioned advantages, MSCs are tripotent, meaning they can differentiate into any mesenchymal cell type including chondrocytes, adipocytes, and/or osteocytes.

In cell culture, MSCs adhere to plastic, forming spindle- or star-shaped stromal cells and can be maintained in standard culture conditions. The cells are known to be multipotent. Therefore, upon isolation, the potency is tested by osteogenic, adipogenic, and chondrogenic differentiation which, according to Horwitz and colleagues [1494], provides evidence that the isolated cells are indeed MSCs. Differentiation capacity, however, varies between donors of different age and associated pathologies as well as over time and passage number [1492, 1498].

As defined by ISCT, MSCs routinely express a defined panel of surface markers on the cell membrane. To date, surface markers of human bone marrow MSCs have been characterized best in comparison to other species [1499-1501]. The standard minimal required markers for human MSCs are CD73, CD90, and CD105. On the other hand, MSCs should not express the hematopoietic Ags CD45, CD34, CD14, and CD19. These distinct marker characteristics clearly separate MSCs from hematopoietic stem cells, which are often found in the same bone marrow niches. Additionally, MSCs are negative for CD11b, CD79a, and HLA-DR [1493, 1502, 1503] yet these criteria do not appear to be unique to MSCs and require additional research more specific to MSCs [1493, 1497]. Because cell surface markers may be lost or gained during culturing, the identified markers do not necessarily demonstrate the cell surface marker situation *in vivo* [1502].

Surface markers on MSCs are a vital investigational topic, being actively delineated for specification between MSC subsets. One such marker, endoglin or CD105, is a TGF-β receptor III associated with the chondrogenic differentiation signaling of MSCs. CD105 is also found on endothelial cells, syncytiotrophoblasts and macrophages [1504]. Thy 1 Ag, CD90, is a marker thought to be involved in cell-cell interactions such as monocyte and leukocyte adhesion to endothelial cells and fibroblasts. Also found on many other cell types including cancer stem cells, endothelial cells, fibroblasts, and hematopoietic stem cells, CD90 is widespread [1503]. CD73, also named ecto-5’-nucleotidase, is a marker able to generate adenosine from adenosine monophosphate and it is highly involved in MSC migration as well as in bone marrow stromal interactions. Suspected of also playing a role in MSC modulation and adaptive immunity [1503], CD73 is one of the many markers being continuously investigated for MSC association. In addition to these classic bone marrow MSC markers, a variety of novel markers is currently debated regarding their suitability to phenotype MSCs, such as SSEA-4, CD271, CD146, MSCA-1, CD49f, CD56, and CD200 [1497].

Despite the lack of full knowledge of their cell marker repertoire, bone marrow MSCs have long been utilized for regenerative therapies. They have been used in the regeneration of bone marrow microenvironment after myeloablative therapies [1498] and also as vehicles to deliver anti-tumor agents to the correct tissues [1493]. Most recently, undifferentiated MSCs have been used as therapy for chronically injured organs, mainly caused by fibrosis, in preclinical and early clinical trials. To further increase the therapeutic potential of MSCs, specific cytokines, drugs, or genetic modification of the cells may yield a more targeted and specific result [1502]. Additionally, the primary key advantage of MSCs is that they can be isolated, modified, and re-implanted into the same patient without provoking an immune response. As this notable effect was mostly studied *in vitro*, it may vary slightly *in vivo* [1505].

Overall, despite not being defined very precisely, it is clear that MSCs are a quite heterogeneous cell population. There is not one universal MSC subset that is adequate for all applications; therefore, the characteristics of different subtypes or MSC-derived precursor cells must be investigated for each specific application. For instance, it remains to be elucidated whether bone marrow MSCs from different bone regions vary in their differentiation potential and surface marker expression [1506] as well as how the isolation methodology influences the molecular and functional phenotype of MSCs [1507]. Certainly, with better characterization and comparison of MSCs in different bone marrow niches of the body, this essential research will be advanced to the next level.

This chapter aims to provide a concise overview on the analysis of human bone marrow stromal cells and is complemented by comparable details for murine bone marrow stromal cells (See chapter section XII.2 murine bone marrow stromal cells).

#### 1.3 Step-by-step sample preparation

Human bone marrow MSCs can be obtained from the femoral bone during the procedure of total hip replacement. A suitable group of donors are, for example, patients with primary coxarthrosis. While interpatient variability cannot be fully avoided, ideally, the individuals of choice should not have any congenital or acquired diseases of the bone marrow, such as tumors or infections. This procedure is ethically unproblematic, as the corresponding tissue sample would otherwise be discarded during the operation. Nevertheless, it should be strictly ensured that there is a current ethical vote and that all regulatory requirements are met.

Since the femoral head is sawed off during hip replacement operation, the entire spongious part of the femoral head can be obtained by scratching with a surgical sharp spoon and subsequent flushing and resuspending the removed bone marrow with PBS. The resulting cell suspension is filtered through a 70 μm strainer and a subsequent Biocoll gradient is used to enrich for MSCs. For this purpose, 20 ml Biocoll is covered with the cell suspension and centrifuged for 30 min at 800 g with brakes turned off. Then, the interphase is collected, washed twice with PBS and the resulting cells are transferred to a cell culture flask for further enrichment and expansion. The cell culture is carried out under standard conditions (37°C and 5% CO_2_) in low glucose Dulbecco’s modified eagle’s medium (DMEM) with 10% fetal bovine serum (FBS), 1% penicillin/streptomycin, and 1% L-glutamine. After 24 h, non-adhered cells are removed by media replacement. Thereby, non-attached cells are washed away, which consequently selects for plastic-adherent MSCs with fibroblast phenotype. In the further course of the cell culture, medium is changed twice a week. When reaching 80% confluency, cells are passaged at a ratio of 1:5. At the end of passage one (p1), cells are frozen using freezing medium containing 10% dimethyl sulfoxide (DMSO), 40% FBS and 50% DMEM and stored at −150°C until further use. For downstream assays, MSCs are thawed and expanded via subculturing for two more passages (p3). To perform flow cytometric analysis, MSCs are trypsinized, washed with FACS buffer and filtered through a 40 μm strainer to reach a single cell suspension. Passage 3 is a good compromise between the amount of bone marrow needed (due to the initial *in vitro* expansion) and avoiding extensive changes to the MSCs *in vitro* (surface markers and functionally) as far as possible. However, the passage number can be adjusted depending on the experimental question.

To evaluate whether the isolated cells are indeed MSCs, a comprehensive phenotypic cell analysis is recommended. To this end, MSCs are analyzed regarding their (1) surface marker expression using flow cytometry, (2) their differentiation potential into bone, cartilage, and fat cells, and (3) their immunoregulatory capacity. In this chapter, we focus on the phenotypic surface marker expression analysis via flow cytometry. For this, trypsinized cells are resuspended in PBS and nonspecific binding sites and Fc receptors are blocked with 2% mouse serum for 20 min. After a washing step with FACS buffer, cells are stained with saturating concentrations of Abs for 20 min on ice. Cells are washed with FACS buffer and resuspended in the final volume of FACS buffer to perform flow cytometric analysis.

#### 1.4 Materials

##### 1.4.1 Human tissue samples.

Human bone marrow tissue samples, such as from femoral head, which is harvested during routine hip replacement procedures

##### 1.4.2 Reagents.

1. Biocoll separating solution (Biochrom AG)
2. Mouse serum
3. Fluorescently labeled human Abs (Table 131)

##### 1.4.3 Solutions.

1. PBS (ThermoFisher)
2. Culture medium: DMEM low glucose, 10% FBS, 1% pen/strep, 1% L-glutamine
3. Freezing medium: 10% DMSO, 40% FBS, 50% DMEM
4. 0.05% trypsin/0.02% EDTA solution
5. FACS buffer with 1% FBS/2 mM EDTA

##### 1.4.4 Equipment.

1. Surgical sharp spoon
2. 40 μm and 70 μm cell strainers (Falcon)
3. Cell culture flasks
4. Flow cytometer

#### 1.5 Data analysis

**Figure 139.**
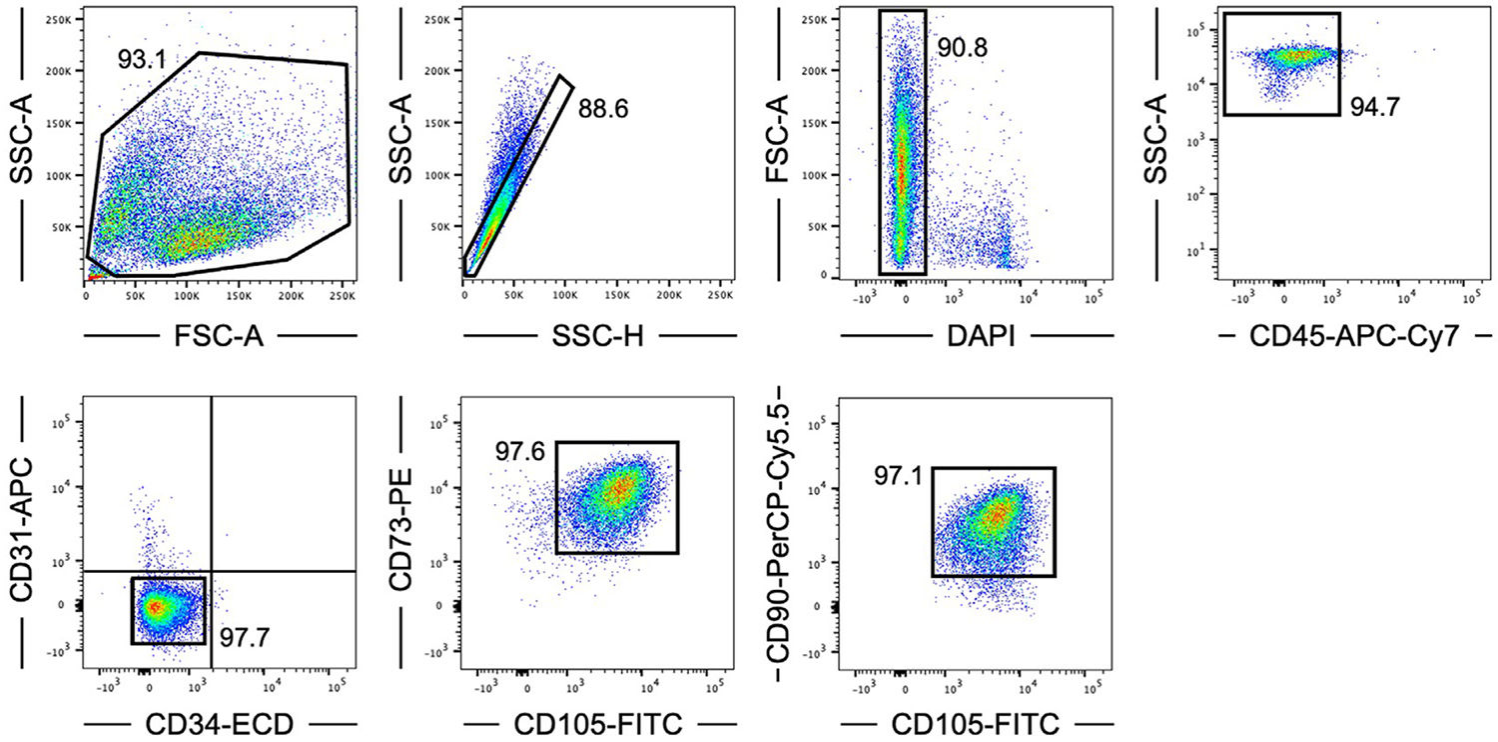
Gating strategy for human bone marrow MSCs. Biocoll gradient-purified and *in vitro*-expanded cells from human bone marrow are gated to exclude doublets and cells expressing CD45. As a next step, live single MSCs are identified as cells negative for CD34 and CD31 and then analyzed for their positive expression of CD73, CD105 and CD90.

As MSCs are a very heterogeneous cell population that requires a collection of several markers for cell identification and further characterization, there are several ways of designing a flow cytometry panel for MSCs. One possibility is to measure mesenchymal markers, such as CD73 and CD105, after doublet discrimination on forward scatter area and height, as well as elimination of cells positive for CD45, CD34, and CD31 (Figure 139) [1508]. Then, CD90 can be measured on the basis of CD73^+^CD105^+^ cells, which results in the following panel: CD45^−^CD34^−^CD31^−^CD73^+^CD105^+^CD90^+^. This panel can be used as a backbone panel for more detailed phenotyping of MSCs. Additional potential markers are listed in Table 132 and Table 134.

#### 1.6 Pitfalls

It should be kept in mind that MSCs significantly change their phenotype over the passages [1508]. This change in surface marker expression might affect the descripted backbone panel, such as for CD105, but also any other downstream phenotyping marker. Therefore, the functionality of predefined backbone panels needs to be verified for every individual passage and, most importantly, one needs to keep very good track of MSC passage numbers. Another important factor in analyzing human MSCs is the interpatient heterogeneity. It is highly recommended to collect as many samples as well as patient data as possible (sex, age, diseases, medication, etc.) to be able to select a homogenic cohort or at least to be aware of potentially unstable variables. In addition to the variability in the donor cohort, the isolation methodology also influences the molecular and functional phenotype of MSCs [1507]. Thus, MSCs should always be isolated using the same methodology and internal quality controls to track the consistency of surface markers. Functional parameters, such as osteogenic differentiation, should be implemented.

#### 1.7 Top tricks

As MSCs change their phenotype with rising passage numbers, it is recommended to collect as much bone marrow as possible to avoid artifacts by cell expansion. Good sources are femur head and vertebrae. If study design and logistics allow, then autopsy material should be considered as well. This option has the potential to generate a great amount of primary human MSCs directly *ex vivo* without the need for extensive expansion. Another consideration is that different MSC isolation methods can be used depending on the study situation. This can simplify the work with the sometimes very limited and diverse patient materials. For example, isolation protocols relying on bone marrow aspirate, scratching and flushing out bone marrow, and outgrowth cultures using bone chips could be considered [1507]. However, these different protocols might result in distinct MSC phenotypes and, therefore, isolation methods should not be changed during an ongoing study.

#### 1.8 Clinical relevance statement

The gating strategy shown in this section is applicable for the comparative analysis of MSCs in patients with osteoporosis in contrast to healthy donors, as can be seen in [1509]. The key conclusion from these analyses indicated that vertebral MSCs from osteoporotic donors were unaffected; they showed similar surface marker expression, and full osteogenic potential when compared to MSCs from non-osteoporotic patients.

#### 1.9 Summary of the phenotypes

This is detailed in Table 132.

### 2 Murine bone marrow stromal cells

#### 2.1 Overview

The bone marrow (BM) stroma plays a critical role in the maintenance of hematopoietic homeostasis. The ability to isolate BM stromal cells at high efficiency is critical to maximize cell recovery and reproducibility of the isolation procedure. In this section, we describe the processing of murine BM samples through sequential enzymatic digestion and the gating strategy used to identify murine stromal and mesenchymal stem cells (MSCs).

#### 2.2 Introduction

The bone marrow stroma is composed of non-hematopoietic cells responsible for the structural organization of the marrow cavity where they support blood cell development and provide distinct niches for the long-term survival of cells that form the basis of immunological memory, such as memory plasma cells, memory B- and -T cells [1510-1512]. Early work by Friedenstein et al. has shown that stromal cells could be distinguished from hematopoietic cells by their adherence to plastic culture dish and their ability to form fibroblastic colonies (called CFU fibroblasts or CFU-F) when plated at clonal density [1513]. Subsequently, a single CFU-F was shown to generate heterotopic ossicles when transplanted in vivo [1514]. These studies paved the way to our understanding of how BM stromal cells regulate developmental and steady-state hematopoiesis. MSCs located at the top of the stromal hierarchy can self-renew and differentiate into bone, fat, and cartilage [1515]. MSC populations are found in distinct perivascular niches where they regulate hematopoietic stem and progenitor functions through the action of cell-bound or secreted cytokines [1516]. In the developing mouse marrow, CD45− Tie2− Thy1.1− CD105+ CD51+ progenitors undergo endochondral ossification and contribute to the formation of the BM cavity by promoting vascularization and the formation of a hematopoietic stem cell (HSC) niche [1517]. In the adult mice BM, MSCs can be labeled by GFP in Nestin-GFP transgenic mice, wherein Nestin-GFP+ cells contain all CFU-F activity or mesensphere formation capacity of the BM [1518]. Nestin-GFPbright cells mark periarteriolar stromal cells that are significantly associated with quiescent HSCs and secrete niche factors such as Cxcl12 and stem cell factor (SCF) that contributes to HSC localization and maintenance [1519]. Nestin-GFP+ cells also highly overlap with stromal cells expressing the Leptin receptor [1520], Cxcl12-abundant reticular cells [1521] or cells that are characterized by Prx-1 expression during development [1522] that have also been described as regulators of hematopoietic stem and progenitors functions. Lineage tracing has also revealed the osteogenic and stromal contribution of MSCs during development [1523]. Furthermore, skeletal stem cells found in the periosteum of long bones have been shown to contribute to bone formation at steady state or after injury [1524-1526]. To study murine BM stromal cells populations, cell surface markers have been proposed to facilitate their identification, but many of these markers are expressed on cultured cells and may differ from freshly isolated stromal cells [1493]. In addition, distinct stromal cell populations can be extracted depending on the isolation methods. Sequential digestion of BM plugs results in efficient extraction of stromal cells with MSC activity [1527]. CD51+ PDGFRa+ CD45− Ter119− CD31− cells comprise most of detectable BM MSC activity isolated from flushed BM plugs and can reconstitute an ectopic HSC niche when transplanted under the kidney capsule [1528]. Crushed bone can result in an enrichment of PDGFRa+ Sca-1 + CD45− Ter119− CD31− MSCs [1529, 1530] or skeletal stem cells expressing Gremlin1 [1531] and CD200 [1524]. While a hierarchal organization for skeletal stem cells and downstream progenitors responsible for cartilage, bone, and stromal cell generation has been proposed [1524], it remains unclear how the bone-associated skeletal stem cells and BM-associated MSCs relate to each other. Therefore, the isolation method (enzymatic treatments, bone crushing, or flushing) will influence the content and heterogeneity found within the stromal cell fraction. Histological analysis of murine bone marrow stromal cells using multiplexed fluorescence microscopy can be used to interpret the results achieved by flow cytometric assay and might be helpful to determine the influence of the isolation method on the stromal cell heterogeneity [1532].

#### 2.3 Step-by-step sample preparation

The stromal fraction of the BM is classically defined by the absence of CD45 (hematopoietic), Ter119 (erythroid) and CD31 (endothelial) marker expression. CD45− Ter119− CD31− or triple-negative cells (TNCs) are known to contain stromal cells as well as hematopoietic cells [1533]. In order to isolate BM stromal cells, femurs or tibias from mice can be cut below the meta-physis toward the epiphysis and the BM is flushed out as an intact plug using syringe with 25G x 1” (femur) or 22G x 1 ¼‘’ (tibia) needle containing HBSS buffer. Bone flush outs and empty bones are transferred into a 24-well plate containing 400 μL of digestion buffer (Collagenase type IV 0.5 mg/mL, Dispase 0.25 mg/mL, DNase I 1 mg/mL and Latrunculin B 5 μg/mL in HBSS). The bone marrow is cut into small pieces and the plate is incubated at 37°C and 5% CO_2_ for 15 min. Subsequently, another 400 μL of digestion buffer is added to the wells and bone marrow samples are gently resuspended. Additionally, the empty bones are flushed with digestion buffer. The samples are again incubated for 15 min, 37°C at 5% CO_2_. To stop digestion, 200 μL PBS/BSA with 10mM EDTA is added to each well. Bone marrow samples are resuspended with a pipet and filtered through a 70 μM nylon mesh to remove clumps. The wells are washed with 1 mL PBS/BSA/2mM EDTA and the hollow bones are thoroughly flushed with a syringe through a 22G needle. Cell suspension is spun down at 320 × *g* for 7 min at 4°C. The supernatant is discarded, and the pellet is resuspended in 200 μL of F_c_γ-receptor-block and incubated for 30 min on ice. The staining mix is added, and cells are incubated for 30 min on ice. Samples are washed, resuspended in the appropriate volume of PBS/BSA/2mM EDTA and filtered through a 30 μM nylon-mesh before sorting.

**Figure 140.**
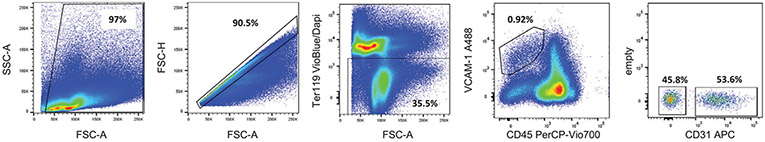
Gating strategy for murine BM stroma cells. Live single non-platelet cells were identified as CD45^−^ and VCAM-1^+^ and further analyzed regarding their CD31 expression. After sorting for CD45^−^/VCAM-1^+^/CD31^−^ a purity of 96% was achieved.

Cells can be further stained with Abs against CD45, Ter119, CD31 and VCAM1 followed by fluorescence-activated cell sorting (Fig. 140). Using this protocol, a purity greater than 95% and recovery over 60% can be achieved.

While CD45, CD31, Ter119, VCAM1 and CD51 epitopes have been shown to be resistant to cell digestion [1533], it is important to compare the sensitivity of each marker to be tested on digested cells and undigested or flushed cells.

The highest priority should be to isolate the MSC compartment as quantitatively as possible to fully preserve heterogeneity *ex vivo*. An innovation in this regard is the use of Latrunculin B, a drug interfering with the polymerization of actin. The addition of this reversible drug during isolation, reduced the stickiness of the MSCs which results in a cell recovery to about 60% of the cell numbers determined *in situ* without affecting the cell viability. Addition of Latrunculin B to the digestion cocktail significantly doubled, as compared to isolation without Latrunculin B, the recovery of ex vivo isolated BM stroma cells [1534].

#### 2.4 Materials

##### 2.4.1 Animals.

Adolescent mice such as C57BL/6 (8–12 weeks old).

##### 2.4.2 Reagents.

1. Collagenase type IV (Sigma-Aldrich)
2. Dispase (Roche)
3. DNase I (Sigma-Aldrich)
4. Latrunculin B (Sigma-Aldrich)
5. DAPI (Sigma, Cat #D9542)
6. FcR blocking reagent, mouse (Milenty Biotech)

##### 2.4.3 Solutions.

1. HBSS (Corning, Cat #21-023-CV)
2. PBS/BSA (0.2%) 10 mM EDTA
3. PBS/BSA (0.2%) 2mM EDTA
4. Digestion buffer (Collagenase IV 0.5 mg/mL, Dipase II 0.25 mg/mL, DNase I 1 mg/mL, Latrunculin B 5 μg/mL in HBSS)
5. DAPI (0.05 μg/mL in PBS/BSA/EDTA buffer)

##### 2.4.4 Equipment.

1. 1 mL syringe with 25G x 1” needle (for femurs) or 22G x 1 ¼‘’needle (fortibias)
2. 70 μM cell strainer (Falcon, Cat #08-771-19)
3. 30 μM cell strainer (miltenyi)

##### 2.4.5 Antibodies.

The Abs used for the identification of murine BM stroma cells are listed in Table 133.

#### 2.5 Data analysis

When using adult mice, TNCs represent approximately 0.5% of total single live BM cells without use of Latrunculin B. Addition of it increases the fraction of TNCs to appr. 1.2 %. Cell surface markers such as CD200, Thy-1, and 6C3 can be used to distinguish among cartilage, bone, and stromal cells when samples are made from crushed bones [1517, 1524, 1530]. Consistency in the processing of BM plugs should limit the variation in the frequency of isolated TNCs or MSCs.

#### 2.6 Pitfalls

In the event that additional markers are to be included in the gating strategy, their sensitivity to the enzymatic digestion should be addressed. Samples should be analyzed as soon as possible after processing and staining since digested BM cells have a higher tendency of clumping together than undigested samples.

#### 2.7 Top tricks

To ensure equal digestion throughout all samples, first harvest all bones and place on ice, in PBS. Then, flush the first sample with digestion buffer and directly put at 37°C. Start timer for the first 15 min of incubation and proceed with the second sample and so on. A constant digestion incubation time is critical in order to avoid overdigestion which could result in a loss of cell surface markers, and to reduce variation among samples. If the isolated cells are to be used for transcriptional analysis, it is recommended that the transcription blocker Actinomycin D (2 mg/ml) should be used in the buffers and in the digestion cocktail to freeze the transcriptome of the cells ex vivo. This will minimize the effects on gene expression caused by cell processing and isolation. It should be noted that Actinomycin D irreversibly inhibits transcription, rendering the isolated cells unusable for cell culture.

#### 2.8 Summary of the phenotypes

This is detailed in Table 134.

## XIII Hematopoietic stem cell phenotypes

### 1 Overview

This chapter deals with the characterization, isolation and preparation of murine and human hematopoietic stem cells.

### 2 Introduction

Throughout the life of mice and humans the major site of residence of hematopoietic stem and progenitor cells (HSPCs) is the bone marrow [1535-1537]. HSCs reside in specialized cellular niches [1538-1540], provided by environmental non-hematopoietic stromal cells and hematopoietic cells, which ensure HSC quiescence and longevity and their capacity to proliferate and/or differentiate into mature blood cells. Continuous proliferation and differentiation regenerates, and thereby maintains mature blood cell compartments of erythroid, myeloid and lymphoid cell lineages. Differentiation occurs in a hierarchical sequence from so-called long-term HSCs (LT-HSC) to short-term HSCs (ST-HSC), to lymphoid-myeloid progenitors/multipotent progenitors (LMPPs/MPPs) that subsequently give rise to common lymphoid progenitors (CLP) or common myeloid progenitors (CMP). These progenitor cells give rise to individual mature blood cell subsets: erythrocytes, megakaryocytes and platelets, myeloid cells including monocytes, macrophages, dendritic cells and granulocytes, and lymphoid cells (T- and B-, innate and natural killers and innate lymphoid cells). A part of the generation of myeloid and erythroid cells can be initiated directly from a special subpopulation of HSC. Under stress, such as a bacterial or viral infection, this direct granulopoiesis, erythropoiesis and the differentiation of megakaryocytes and platelets is increased and accelerated directly from HSC [1541-1543]. The transplantation of HSC of human or mouse origin into suitable recipient hosts populates all stem and progenitor compartments in bone marrow of the recipient and regenerates erythroid, myeloid and lymphoid compartments with donor-derived cells to normal sizes.

### 3 Human hematopoietic stem cells (hHSCs)

#### 3.1 Introduction

In this chapter we provide an overview on the identification and isolation of human hematopoietic stem cells from different sources including human bone marrow, mobilized peripheral blood, and cord blood and humanized mice.

The most commonly used surface marker to enrich human hematopoietic stem and progenitor cells (HSPCs) is the glycoprotein CD34, which is expressed by HSCs and committed progenitors but not mature blood cells [1544]. CD34-enriched cells from human bone marrow or mobilized peripheral blood are frequently used in clinical stem cell transplantation [1545]. However, HSC activity has also been reported in the CD34^−^ population of human cord blood and bone marrow, even though those cells are extremely rare [1546-1548].

CD34 is expressed by HSPCs and additional markers are required to identify and isolate most immature HSCs [1549]. *In vitro* potential analysis combined with multilineage reconstitution potential after xenotransplantation into suitable recipient mice was used to further separate the Lin^−^ CD34^+^ population using markers that are differentially expressed on immature and more differentiated cells. The group of John Dick showed that the CD38^−^ fraction of CD34^+^ human bone marrow and cord blood cells is highly enriched for cells with the ability to repopulate immune-deficient mice [1550]. Limiting dilution analysis showed that 1 out of 617 purified CD34^+^ CD38^−^ cells engrafts in NOD/SCID mice for at least 8 weeks.

Ten years later, the laboratory of Irving L. Weissman showed that Lin^−^ CD34^+^ CD38^−^ human bone marrow and cord blood cells could be further subdivided using the cell surface markers CD90 and CD45RA [1551]. They identified Lin^−^ CD34^+^ CD38^−^ CD90^+^ CD45RA^−^ cells as HSCs with long-term repopulation capacity for up to 30 weeks using as few as 10 transplanted cells being able to engraft in newborn NOG mice. Repopulation of secondary recipients was also supported by these donor cells, suggesting that human HSCs with combined long-term repopulation and self-renewal potential are contained within the Lin^−^ CD34^+^ CD38^−^ CD90^+^ CD45RA^−^ population of human cord blood and bone marrow cells [1551]. Repopulation of recipient mice occurs more efficient from Lin^−^ CD34^+^ CD38^−^ CD90^+^ CD45RA^−^ HSCs compared to Lin^−^ CD34^+^ CD38^−^ CD90^−^ CD45RA^−^ multipotent progenitor cells (MPPs).

The group of John Dick identified CD49f (VLA-6) as an additional marker to further enrich for human LT-HSCs using limiting dilution assays and single-cell transplantation into NSG mice [1552]. 1 out of 10 Lin^−^ CD34^+^ CD38^−^ CD90^+^ CD45RA^−^ CD49f^+^ cells contained long-term repopulation activity and could be serially transplanted, representing the most purified population of human HSCs to date.

**Figure 141.**
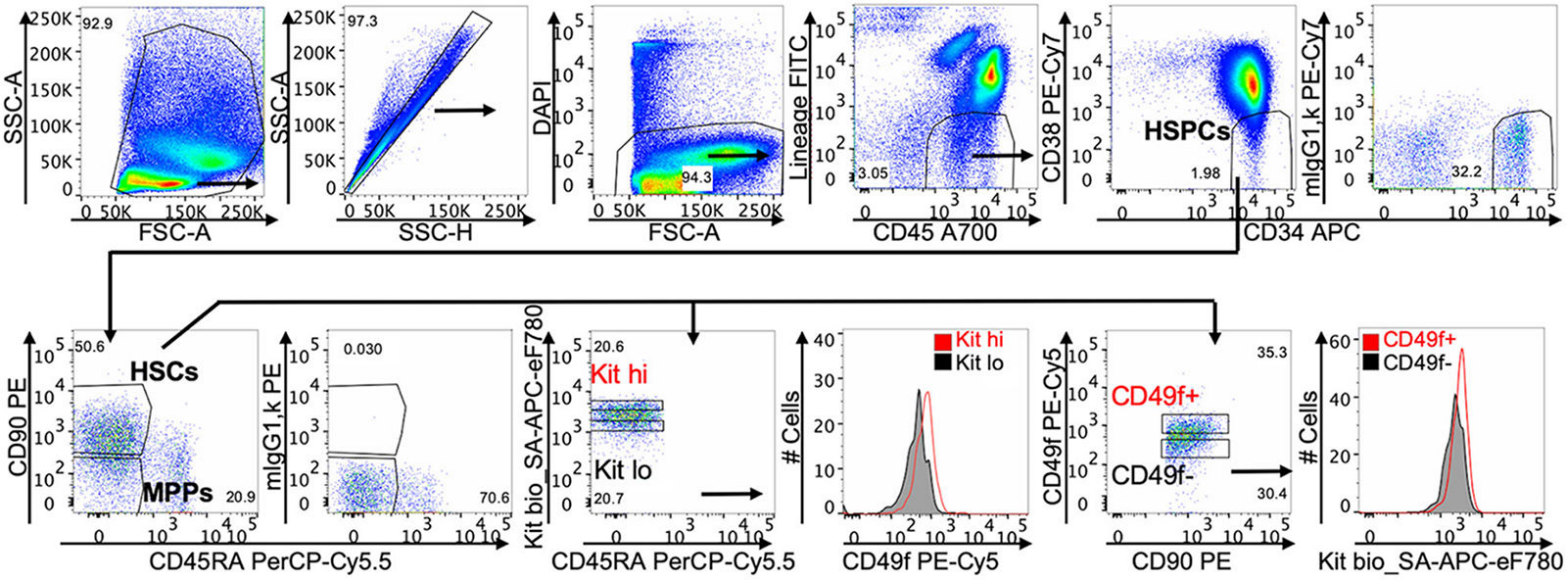
Phenotypic characterization of HSCs from CD34-enriched human cord blood. HSPCs were identified as CD34^+^ CD38^−^ cells within the human CD45^+^ Lin^−^ compartment. The lineage cocktail contained the following Abs: CD3, CD10, CD14, CD15, CD16, CD19, and CD235. HSCs were identified as CD34^+^ CD38^−^ CD90^+^ CD45RA^−^ cells and MPPs as CD34^+^ CD38^−^ CD90^−^ CD45RA^−^ cells [1551]. LT-HSCs with increased expansion potential can be identified within cells expressing the top 20% of Kit [1553], and LT-HSCs containing the highest repopulating activity express high levels of CD49f [1553]. Gatings for CD38 and CD90 were set according to isotype controls. Gating strategies for Kit and CD49f are chosen according to the original publications [1552, 1553]

The laboratory of Claudia Waskow showed that human LT-HSCs with increased expansion potential is enriched in a subpopulation of CD34^+^ CD38^−^ CD90^+^ CD45RA^−^ stem cells expressing high levels of the Kit receptor [1553]. Transplantation of equal numbers of Kit^hi^ versus Kit^lo^ cord blood cells results in an increased human blood and HSC chimerism in the bone marrow of Kit^hi^ cells compared to Kit^lo^ HSCs. In fact, Kit^hi^ HSCs with improved HSC engraftment, express elevated levels of CD49f, and, inversely, CD49f^+^ cells express increased levels of the Kit receptor, suggesting an overlap of both cell populations (Figure 141).

Finally, the lab of Guy Sauvageau identified the endothelial protein C receptor (EPCR) as marker for serially transplantable human HSCs [1554]. EPCR is also used to identify HSCs from the murine bone marrow [1555] and fetal liver [1556]. An overview of different identification protocols is provided in Table 138 (see below).

#### 3.2 Step-by-step sample preparation

##### 3.2.1 Isolation of human HSCs.

Primary sources of human HSCs are human bone marrow, G-CSF-mobilized blood, umbilical cord blood and fetal liver. Human HSCs can also be isolated from immune-deficient mouse models engrafted with human HSCs.

##### 3.2.2 Quick staining.

To determine the frequency of hCD34^+^ cells in the samples, a quick staining is performed on 400 μl sample before the isolation is initiated. This is important to calculate the volumes of reagents used at later stages of this protocol:

1. Perform quick staining using Abs specific for hCD45 hCD38 hCD34 in 70 μl PBS/2% FCS for 20 min at room temperature or 40 min on ice using a v-bottom 96-well plate.
2. Wash cells 2x: add 100 μl PBS/2% FCS and spin down (648 *g*, 2 min, 4°C)
3. Resuspend pellet in 150 μl PBS/2% FCS containing DAPI (final: 0.04 μg/ml)

Add counting beads and run on flow cytometer. Absolute cell numbers can also be obtained from cytometers capable of providing absolute cell counts. In this case the addition of counting beads can be omitted.

##### 3.2.3 Isolation of human cells.

For the isolation of human cells from mouse recipients, bone marrow cell suspensions are prepared as outlined in section 4.2.1 Isolation of mouse hematopoietic stem and progenitor cells.

1. Mononuclear cells (MNCs) are prepared from human bone marrow, peripheral blood or umbilical cord blood using leukocyte separation medium (1.077 g/ml) performing a density gradient centrifugation (3-5 ml bone marrow for 1-10 x10^7^ MNCs; 15-20 ml cord blood for 1-15 x10^7^ MNCs). Centrifugation of the samples at room temperature for 35 min at 400 *g* without break.
2. After centrifugation, MNCs form a white pellet between serum (yellow colored upper phase) and the leukocyte separation medium (bottom layer). Erythrocytes accumulate on the bottom of the tube (red color).
3. MNCs layer is gently collected using a sterile Pasteur pipette without disturbing any upper and lower layers.
4. MNCs are transferred into 30 ml PBS/2% FCS in a 50 ml tube. For washing, fill up the tube to 50 ml.
5. Spin down cells for 10 minutes at 275 *g* at 4°C, discard the supernatant and combine resuspended pellets in one 50 ml tube.
6. Fill up the tube with PBS/2% FCS to wash the cells again.

###### Recommendation:

If the cell pellet is still very red, a red blood cell lysis can be performed (5 min in ACK lysing buffer at room temperature).

1. Discard the supernatant and resuspend the pellet in 10 ml PBS/2% FCS and count the cells.

##### 3.2.4 Enrichment of hCD34^+^ cells.

1. Resuspend MNCs at a concentration of 10^8^ cells per 300 μl MACS buffer using a 1.5 ml tube. To add the appropriate volume of blocking solution the frequency of hCD34 cells determined by quick staining is used (see above 3.2.2 Quick staining). The indicated volume of blocking reagent for the frequency of hCD34^+^ cells is indicated in Table 135. Immediate afterwards add the same volume of magnetic beads. Incubate for 30 minutes at 4°C.
2. Washing: Add 5-fold volume of MACS buffer and spin down cells for 10 minutes at 275 *g* at 4°C.

###### Meanwhile:

Prime the MACS Columns with MACS Buffer after assembly on a MACS magnet (LS columns: 1-2 x 10^8^ MNCs, add 3 times 3 ml of MACS buffer, or MS Columns: <10^8^ MNCs add 3 times 500 μl of MACS buffer).

1. Resuspend MNCs in MACS buffer (3 ml for LS columns, 500 μl for MS columns). Apply the MNCs through the column filtering them through a 40 μm mesh to avoid blocking.
2. Wash the column 3 times with 3 ml (LS) or 500 μl (MS) MACS Buffer.

###### Recommended:

To check if there are any hCD34^+^ remaining cells, take an aliquot of the negative fraction and stain later together with the enriched cells using the hHSC staining mix.

1. Remove the LS or MS column from the magnet and transfer the column to a fresh 15 ml tube.
2. Add 5 ml (LS column) or 1 ml (MS column) MACS Buffer to the column and push the cells into the 15 ml tube using the provided plunger.

###### Recommended:

Use a second column to increase the purity of hCD34% cells from 60-65% to 90-95%.

1. Spin down the cells and resuspend the pellet in 300 μl of sterile PBS/2%FCS. Cells are ready for staining.

##### 3.2.5 Staining of human samples.

1. Perform Ab stainings (Table 136) in PBS/2%FCS (100ul Ab mix for 1-2x10^7^ MNCs) for 40 minutes at 4°C. Use mouse pure mouse IgG (ChromPure Mouse IgG 015-000-003 final concentration: 500 μg/ml) to block unspecific binding.

###### Meanwhile:

A part of human MNCs should be single stained with single fluorochrome conjugated Abs for cytometer compensation (Table 136). Keep controls on ice until acquisition.

Note, if compensation beads are used, compensation sample preparation from human cells can be omitted.

###### Note:

Isotype controls should be used with caution and absolute conclusions about non-specific binding should not be made because isotype controls can indicate, for example, Fc receptor-mediated non-specific binding.

1. Add PBS/2% FCS and resuspend in appropriate volume of PBS/2% FCS containing a viability dye such as DAPI, SYTO× green, SYTOX Blue or propidium iodide (PI).

###### Note:

DAPI is used here as viability dye based on its compatibility with the selected fluorochromes from the staining panel. The dye indicating viability of the cells can also be used in the same channel as fluorochromes used to label lineage-indicative Abs (Table 136). E.g., SYTO× green can be used together with FITC-labeled Abs. SYTOX Blue can be used together with Pacific Blue-labeled Abs. Alternatively, PI can also be used for dead cell exclusion, however, PI can spillover to the channel detecting PE conjugated fluorochromes such as PE-Texas Red.

1. Filter cells prior to analysis through a 40 μm filter. Samples are ready for acquisition.

##### 3.2.6 Human HSC FACS analysis.

HSCs from all sources display a similar pattern of surface marker expression and can therefore be marked and isolated using the same panel of Abs (Table 136). Human HSCs are devoid of Ags expressed by mature blood cell lineages (Lin^−^). For positive identification of human HSCs please refer to the introduction. To distinguish human and mouse HSCs in a humanized mouse model, Abs specific for murine and human CD45 are used. Human or mouse hematopoietic cells are analyzed within the Ter119 (mouse) and CD235 (human) negative fraction to exclude red blood cells and their precursors from the analysis.

For analyzing human HSCs, a flow cytometer equipped with 4 lasers and 12 photomultipliers (PMT) is used (BD, LSRII). Laser wavelengths, PMTs, optical filters and the corresponding appropriate fluorochromes are indicated in Table 137. Channels that are used in the provided staining are depicted in bold.

1. Acquire a portion of your unstained sample to adjust FSC, SSC and single color fluorochrome PMT voltages (Figure 141). PMT Voltages should be set to ensure that the data peak is above the noise of the cytometer.
2. Record unstained and single-stained compensation controls.
3. Calculate the compensation values by using cytometer software and apply the compensations to the fully stained samples.
4. Record your samples, isotype stained controls and FMOs.

###### Before acquisition:

Set up the hHSC gatings by the provided gating strategy (Figure 141). To determine the positive signal, FMOs and isotype controls should be used.

###### Recommended:

For meaningful results a minimum 1000 hHSCs should be acquired.

###### 3.2.7 Sorting of human HSCs.

Human HSCs can be sorted on any sorter device equipped with lasers and filters compatible with experimental fluorophores (Table 137). Generally, for the sorting of human HSCs the nozzle size 85 μm is used and the pressure of 45 psi is applied.

1. Drop delay has to be set to ensure the optimal sorting. Note: If suitable with the selected cytometer, Accudrop beads can be used for calculating the drop delay.
2. Adjust the stream angle = to ensure cells are deposited into the collection tubes. For simultaneous 2- or 4-way sorts, 1.5 ml or 5 ml tubes containing filtered PBS/2% FCS can be used.

###### Note:

The streams should be directed to deliver the cells into the liquid in the collection tubes.

1. For a pure sort, Purity Mask with 4-way purity precision mode should be selected. These recommendations are specific to FACSAria (BD). Pay attention to use appropriate sort speed for the sorting of sorting human HSCs and MPPs.

#### 3.3 Materials

1. Source of adult human HSCs: bone marrow, G-CSF mobilized peripheral blood, umbilical cord blood, or humanized mice.
2. Ficoll-Paque solution (d=1.077) (e.g., Biocoll separating solution, Biochrom, #L6115)
3. PBS containing 2% fetal calf serum (FCS) (Sigma, #F0804)
4. Optional: ACK lysing buffer (ThermoFischer #A10492-01)
5. MACS buffer (PBS 2 % FCS containing 2mM EDTA, filtered 0.2μm filter)
6. CD34 MicroBeads Kit, 10 ml (Miltenyi, #130-046-703)
7. MACS separation columns (LS, Miltenyi, #130-042-401, MS, Miltenyi, #130-042-201)
8. Flow cytometer equipped to measure FITC, PE, PE-Cy7, PerCP-Cy5.5, PE-Cy5, APC, Alexa Fluor700, Alexa Flour780 and DAPI (stock: 10 mg/ml) (e.g., Becton Dickinson, LSRII, Fortessa, Aria, Fusion).
9. Tubes: 50 ml tubes for sample preparation (Greiner, Cat #227 261), 1.5 ml tubes for sample preparation or collection tubes (Sarstedt, Cat# 72.690.001), 5 ml collection tubes (FALCON, #352054).
10. Counting Beads (CountBright^™^ #C36950)
11. Filters, 40 μm (Sefar Nitex, #03-41/31).

#### 3.4 Pitfalls

- The quality of primary samples may differ depending on the donor, the way of extraction and the anti-coagulant used. Thus, frequencies of HSCs differ between samples.
- If there is no clear cell fraction after Ficoll-Paque density gradient centrifugation, the centrifuge break may not have been set to 0.

#### 3.5 Top tricks

- Use samples as fresh as possible to obtain a high HSC yield.
- Dilute bone marrow or blood with PBS (1:1 to 1:2) prior to Ficoll-Paque density gradient centrifugation.
- Before the start cytometer should be cleaned well with water and the rate of event should not exceed more than 7000 events/second during acquisition of the human samples. This event rate is specific to the FACSAria set up with 85um nozzle at 45 PSI, thus the event rate can alter in different cytometers with different nozzle sizes. As well, the efficiency and the electronic abort rate should be monitored during sorting.
- Use 85 μm nozzle to purify human HSPCs using 45psi pressure.
- To avoid surplus hours at the sorting machine, human CD34^+^ cells can be enriched with magnetic beads prior to staining and sorting.

#### 3.6 Clinical relevance statement

The gating strategy shown in this section is applicable for analysis of bone marrow cells or G-CSF-mobilized blood cells in healthy patients. The key conclusion from such analysis is the detailed composition of the HSPC population.

#### 3.7 Summary of the phenotypes

This is detailed in Table 138.

### 4 Murine hematopoietic stem cells

#### 4.1 Introduction

This part of this chapter describes the methods for adult murine hematopoietic stem cells.

In mice HSC are generated during embryonic development, first extra-embryonically from cells in yolk sac, then from cells in the embryonic aorta-gonad-mesonephros area via hemangioblasts, which are common progenitors of vascular endothelium and hematopoietic cells [1557, 1558]. These early progenitors seed into fetal liver and fetal thymus to generate first, transient waves of hematopoiesis. Shortly before birth the developing marrow of bone becomes the site, where HSC find an environment for their life-long residence, hematopoietic renewal and differentiation capacities [1559].

**Figure 142.**
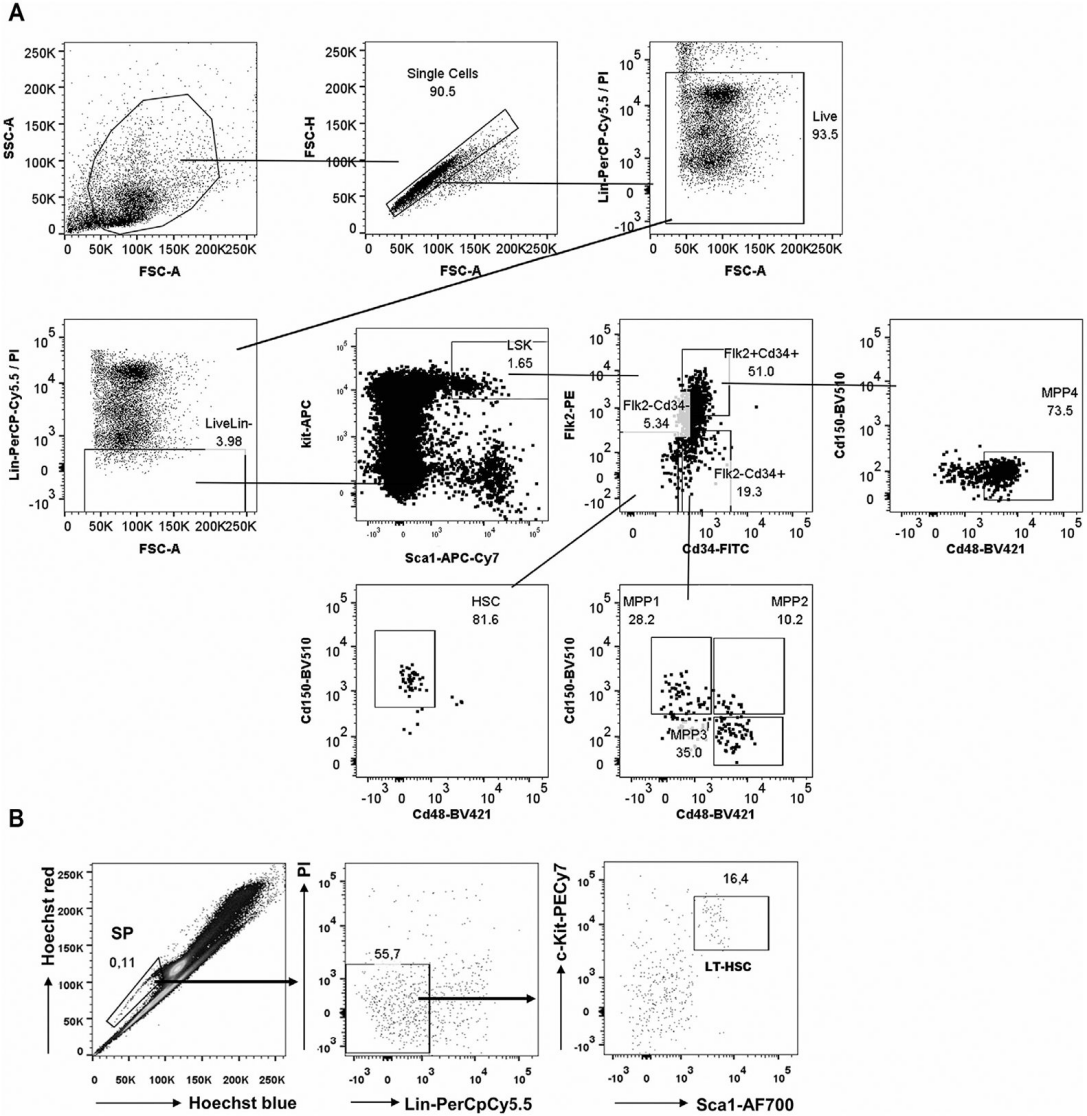
Gating strategy of mouse hematopoietic stem cells. Phenotypic characterization of mouse bone marrow derived HSCs. LSK cells were identified as kit^+^ Sca1^+^ cells within the Living/Lin^−^ compartment. LSK cells were further characterized by CD34 and Flk2 (CD135) expression. HSCs are CD150^+^ CD48^−^ within the CD34^−^CD135^−^ gate. MPPs are further characterized as CD150^+^ CD48^−^ (MPP1) CD150^+^ CD48^+^ (MPP2) and CD150^−^ CD48^+^ (MPP3) cells within the CD34^+^CD135^−^ gate. MPP4 population is determined as CD34^+^CD135^+^CD150^−^CD48^+^cells. Gating for all colors were set according to the isotype control staining (not shown). Forward and side scatter voltages can be increased to dissect bone marrow cell populations into more differentiated subpopulations, differing in size and density.

HSCs are identified by flow cytometry, based on surface-marker expression. One set of fluorescent mAb combinations, and the FACS profiles of the stained bone marrow cells is given in Figure 142. HSC are found in the 0.02-0.05% of all CD45+ bone marrow cells, which do not yet express the markers of differentiated hematopoietic cells, i.e., of F4/80^+^/Mac1^+^ monocytes and macrophages, Gr1^+^ granulocytes, CD11c^+^ dendritic cells, CD4^+^/CD8^+^/CD3^+^ T cells, CD5^+^CD19^+^B220^+^ B cells, NK1.1^+^ NK cells and Ter119^+^ thrombocytes/erythrocytes. Thus, they are “lineage-negative” (Lin^−^). The absence of these Ags and expression of CD45 is necessary to identify the hematopoietic population within the lineage-negative (Lin^−^) cells of the bone marrow. On the other hand, HSC express Sca-1 (S) and Kit (K), thus are called LSK-cells.

Furthermore, differences in surface expression of CD34, CD135 and “SLAM” family markers CD150 and CD48 allow to distinguish long-term self-renewing HSCs and transiently reconstituting multipotent progenitors [1560-1564]. Thus, the LSK CD34^−^CD135^−^CD150^+^CD48^−^ population contains long-term self-renewing HSCs, the LSK CD34^+^CD135^−^CD150^+^CD48^−^ population mainly transiently self-renewing multipotent progenitors (MPP1), the LSK CD34^+^CD135^−+^CD150^+^CD48^+^ population mainly transiently self-renewing multipotent progenitors being in cell cycle (MPP2), while the LSK CD34^+^CD135^+/−^CD150^−^CD48^+^ MPP3 (CD135^−^) and MPP4 (CD135^+^) populations are non-self-renewing direct progenies of common myeloid and lymphoid progenitors [1560-1564]. Their functions have been determined by transplantation analyses. These three distinct populations vary with each stage in the progression toward lineage commitment in their frequency, engraftment-kinetics, self-renewal potential, cell-cycle status, gene expression, and lineage distribution of the mature cells they can generate *in vivo*.

In the bone marrow of 2-3 month-old mice between 1 and 3x10^3^ LSK, CD34^−^CD135^−^CD150^+^CD48^−^ cells remain in a non-proliferating, cell cycle Go-resting state for life [1565, 1566].

Barcoding of these early progenitors shows that most of them have clone sizes of less than 10 cells, and most of them retain these small clone sizes, because they divide at best once a year in the life of a mouse [1565, 1566]. A part of this HSC population can be transplanted, remarkably even as single (e.g., CD45.1^+^) HSC with carrier (CD45.2^+^) bone marrow cells into lethally irradiated (ideally histocompatible CD45.1×CD45.2) recipients. They home to bone marrow and then repopulate all HSC compartments, all hematopoietic progenitors and all mature cell lineages, except of the long-lived resident myeloid cells generated from fetal liver progenitors during embryonic development [1567]. These HSC are called long-term repopulating (LT-HSC). Upon transplantation LT-HSC can home back to bone marrow into special niches near hypoxic areas of arteriolar vascular endothelium and barcoding reveals a smaller number of these LT-HSC with much larger clone sizes [1541].

Between 1-2x10^4^ MPP1 and 2 are LSK CD34^+^CD135^−^CD150^+/−^CD48^+^, which are in active G1-S-G2-M cell cycle, renewing their HSC state by symmetric or asymmetric cell divisions. In asymmetric cell divisions a fraction of them can enter differentiation to more mature states of hematopoietic developments. When transplanted, these MPPs repopulate all different lymphoid and myeloid cell lineages in subsiding waves only for a short time, again without populating the embryonically derived resident myeloid cell lineages [1565].

HSC can be mobilized to enter blood circulation. They might differentiate in the periphery or pick up intracellular infections, such as *Mycobacterium tuberculosis*, and then use their exceptionally efficient capacity to return to bone marrow and become again resident in their niches [1568]. An overview of the phenotypic characterization of hematopoietic stem and progenitor cells is provided in Table 141 (see below).

#### 4.2 Step-by-step sample preparation

##### 4.2.1 Isolation of mouse hematopoietic stem and progenitor cells.

1. The first step in the preparative isolation of adult mouse HSCs and multipotent progenitors from BM is to prepare tibias and femurs of the mice and to put them into staining medium on ice.
2. After the preparation, the marrow cells are flushed out with 15ml staining medium from the bones using 27G needles and filtered through a 70μm pore size cell strainer into a 50mL tube.
3. After centrifuging (300 × *g* for 5 min, on 4°C), erythrocytes are lysed with 1ml hypotonic ACK (ammonium-chloridepotassium) solution for 1-5 min on ice, then the remaining cells are suspend in 20ml staining buffer for centrifugation.
4. After spinning down the cells, the supernatant is discarded. The cells are resuspend in 5ml staining buffer for counting. Regular cell number for 2 tibias and femurs is 5-8x10^7^. The cells are ready for staining.

##### 4.2.2 Depletion of Lineage+ cells for mouse stem and progenitor cell sorting.

1. Suspend the cells in 800-1000μl staining buffer, supplemented with biotinylated anti-lineage Abs in the right concentration (all Abs are listed in Table 139 and the working concentration has to be determined individually prior to the usage) for 15 min on ice.
2. Wash and spin (300 × *g* for 5 min, on 4°C) the cells with 15ml staining buffer.
3. Add streptavidin coated magnetic beads to the cells, according to the used manufacturer’s recommendations.

*Note:* streptavidin coated magnetic beads prepared for cell enrichment or depletion from any brand is appropriate for this step.

1. Wash and spin (300 × *g* for 5 min, on 4°C) the cells and suspend in 2ml staining buffer, place a magnetic separator column, appropriate for depleting at least 10^8^ cells in strong magnetic field and load the cells on the column. Let the cells through the column into a fresh 15ml tube.
2. Wash the magnetic separator column with 8ml of ice-cold staining buffer and centrifuge the cells (300 × *g* for 5 min, on 4°C).

All Abs must be titrated to determine optimal staining concentrations.

Fluorophore conjugates can vary according to the flow cytometer or FACS machine used. The list is an example.

#### 4.2.3 The simplest staining strategy of mouse bone marrow samples for hematopoietic stem cell sorting and/or analysis.

1. Suspend the cells in 500μl staining buffer, supplemented with fluorophore conjugated anti-lineage and streptavidin, anti-mouse kit, anti-mouse Scal, anti-mouse Flk2 (CD135), anti-mouse CD150 and anti-mouse CD48 Abs in the right concentration (all Abs are listed in Table 139 and the working concentration has to be determined individually prior to the usage) for 15 min on ice in dark.
2. After washing, stain the marrow cell samples in 500μl with fluorophore conjugated anti-mouse CD34 Ab with the right concentration (all Abs are listed in Table 139 and the working concentration has to be determined individually prior to the usage) for 15-20 minutes at 37°C in dark.

*Meanwhile*: mouse compensation controls should also be stained with single fluorochrome conjugated Abs (Table 139). The best source for staining compensation controls is the bone marrow single cell suspension. Using Ab-capture beads is not recommended because of the different size and auto-fluorescence parameters compared to the eukaryotic cells. Keep controls on ice until acquisition.

3. Wash and suspend the cells in appropriate volume of staining buffer supplemented with a viability dye.

*Note*: The dye indicating viability of the cells can also be used in the same channel as fluorochromes used to label lineage-indicative Abs, however the compensation has to be done separately for the different dyes. In case automated compensation cannot be done for multiple fluorophores used in the same channel by the installed software of the flow cytometer/cell sorter, the compensation setup has to be done manually.

4. Filter cells with a 40μm pore size cell strainer prior to the analysis or sorting. Samples are ready for acquisition.

##### 4.2.4 Gating strategy for the analysis and sorting of mouse HSCs, MPP1-4 populations.

The first step in the analysis of hematopoietic stem and progenitor populations is excluding mature cells that express “lineage” (Lin) Ags specific to terminally differentiated blood cells, including F4/80^+^/Mac1^+^ monocytes and macrophages, Gr1^+^ granulocytes, CD11c^+^ dendritic cells, CD4^+^/CD8^+^/CD3^+^ T cells, CD19^+^B220^+^ B cells, NK1.1^+^ NK cells and Ter119^+^ megakaryocytes, erythrocytes and thrombocytes. Stem and progenitor cells are then enriched from the remaining cells as Lin^−^cells that express combinations of cell surface markers, Kit and Sca1. Multipotent hematopoietic progenitors, purified as LSK (Lin^−^Kit^+^Sca1^+^) make up <3% of lineage negative cells. They contain all multipotent progenitors in mice [1569-1572]. However, they are still heterogeneous, containing transiently reconstituting multipotent progenitors in addition to long-term reconstituting HSCs.

The differences in CD34, CD135 (Flk2) and “SLAM”-marker expression between long-term self-renewing HSCs and transiently reconstituting multipotent progenitors permit the separation and independent isolation of these different progenitor populations [1560-1564] as LSK CD34^−^CD135^−^CD150+CD48^−^, mainly long-term quiescent HSCs, LSK CD34^+^CD135^−^CD150^+^CD48^−^, activated MPP1, LSK CD34^+^CD135^−^CD150^+^CD48^+^ actively proliferating MPP2 and LSK CD34^+^CD135^−/+^CD150^+^CD48^−^ mainly non-renewing, myeloid-lymphoid multipotent progenitors (MPP3 and 4), as characterized by transplantation analyses. These five distinct populations vary with each stage in the progression from quiescence state toward lineage commitment in their frequency, engraftment-kinetics, self-renewal potential, cell-cycle status, gene expression, and lineage distribution of the mature cells they can generate *in vivo* Table 141 (see below), Figure 142/A [1563].

However, “SLAM”-defined cells themselves are still heterogeneous populations in which HSCs represent, at most, 20% of all cells. Further enrichment of HSCs can be achieved by the purification of SLAM-defined cells that express high levels of EPCR (CD201) [1555] or by the expression of other SLAM family members CD229 and CD244 [1573].

Alternatively, HSCs can be isolated due to their hypoxia-induced high expression of the multidrug transporter proteins MDR1 and ABCG2, thus, cells that retain only low levels of DNA dyes, such as Rhodamine-123 (Rho123) and Hoechst 33342. Rho123^lo^ or Hoechst^lo^ cells (“side population,” SP cells), and that are Lin^−^Kit^+^Sca1^+^ are nearly pure populations of long-term reconstituting HSCs [1574, 1575] (Figure 142/B).

It should be kept in mind, that all these purified HSC populations are still a heterogeneous collection of cells, when their functions are considered. Thus, it is believed that myeloid-biased HSCs express higher levels of CD150 and efflux Hoechst 33342 more efficiently than lymphoid-biased HSCs. They also exhibit higher self-renewal ability as demonstrated by serial transplantation of BM cells from primary recipients into secondary hosts. Quantitative analyses of the frequencies of single HSC/progenitors for a given function “in vitro” or “in vivo” (e.g., as done with single cells) should be attempted to define their potencies ([1576] or [1541] or [1577] or [1578-1580]).

Any flow cytometer and cell sorter is usable to analyze or sort mouse hematopoietic stem and progenitor cell populations which is able to record/detect 7 or more parameters (surface staining conjugated to fluorescent dyes) simultaneously, regardless of the cell detection technology.

One example of surface marker Ab conjugates, the respective applicable optical filters and an appropriate flow cytometer configuration are indicated in Table 139 and 140 respectively. Channels that are used in the provided staining are depicted in bold of Table 140.

For setting up the flow cytometer or cell sorter and for the acquisition and sorting of the mouse hematopoietic stem and progenitor populations, follow the protocol of sections **‘1.1.5 Human HSC FACS analysis’** and **‘1.1.6 Sorting of human HSCs’**.

#### 4.3 Materials

1. Adult mice such as C57BL/6, typically, 6- to 10-week-old mice are used for the isolation of HSCs.
2. Actinomycin D 2μg/ml in solution (Stock 2mg/ml in DMSO: Sigma, SBR00013)
3. Staining medium: phosphate-buffered saline solution (1×PBS) with 0,5% bovine serum albumin fraction V 2mM EDTA, without azide.
4. ACK solution: 0.01M KHCO_3_, 0.155M NH_4_CL, 0.1 mM EDTA
5. 70μm and 40μm pore size cell strainers (e.g., Falcon, 352350).
6. 15mL syringes with 27-gauge needles to flush marrow out of femurs and tibias.
7. Use 50 ml tubes for flushing the bone marrow cells, followed by cell filtration and erythrolysis. Use 15-mL tubes to stain BM cells. Antibodies described in this protocol are available from eBioscience, BioLegend and Becton Dickinson and listed in Table 139.
8. Buffer for measuring side population: SP buffer (PBS, 2% FCS, 2 mM HEPES buffer; GIBCO, Life Technologies), Hoechst 33342 (5 μg/mL, Molecular Probes, Life Technologies).
9. A viability dye, spectrally matching into the staining panel e.g 1μg/ml propidium iodide (PI) (Invitrogen).
10. Flow cytometer or FACS machine which is able to record/detect 7 or more parameters (surface staining conjugated to fluorescent dyes) simultaneously, regardless of the cell detection technology.

#### 4.4 Data analysis

After recording 1-2x10^6^ events, the hematopoietic stem and progenitor cell populations can be calculated using the total cell number and the frequencies of the populations measured by flow cytometry. It is recommended to check all the gates of the individual samples, to match the appropriate separation criteria for the surface marker expressions of a certain cell population. Batch analysis could be misleading.

#### 4.5 Pitfalls

The mouse CD34 staining works best as a separate staining for 20 minutes at 37°C.

#### 4.6 Top tricks

- The RNA expression program of early quiescent hematopoietic stem and activated progenitor cells can be altered by the preparation of the marrow cells. To avoid such *in vitro* activation of e.g., immediate early genes during the preparation of the bone marrow cell suspensions, it is recommended to use transcription (and nuclease) inhibitors like actionomycin D. *In vivo* administration of actinomycin D 5-15 min prior to sacrifice of the mice and of the BM cell preparation, and keeping the cells in actinomycin D containing media until the erythrocyte lysis is one of the most common way to preserve new transcription in hematopoietic stem cells (e.g., [1581]).
- Most flow cytometers can measure more than 7 parameters simultaneously. The staining panel indicated above allows to test additional markers in open channels, using e.g., PE-Cy7, Alexa Fluor 700 or Brilliant Violet 780 fluorescent conjugates.
- Setting the CD34^+/−^, Flk2^+/−^ gates on Lineage^−^ cells first and then applying the gates on LSK population helps a lot to identify the negative and positive populations.

#### 4.7 Summary of the phenotypes

This is detailed in Table 141.

## XIV Tumor cell phenotypes

### 1 Human tumor cells

#### 1.1 Overview

The flow cytometry-based characterization of tumors is required for the improvement of existing, and the development of novel, therapeutic strategies against all types of cancers [1582]. The various alterations involved in malignant transformation are elegantly summarized in [1583].

Some of the proteins involved in transformation mechanisms can be detected using flow cytometry such as mutated growth factor receptors, i.e., EGFR, Her2/neu, PDGF-R, IGFR, c-Met and others. In contrast, most components of the intracellular oncogenic signaling pathways, i.e., mutated, constitutively running RAS/RAF, MAPK, PI3K kinase cascades, are not detected by flow cytometry but rather by classical western blot and protein arrays. The most relevant examples of surface markers on tumor cells are summarized in this section, detailing the surface expression of hematopoietic, epithelial, endothelial and neuroectodermal markers for the classification of tumor cells according to their cellular origin. Importantly, flow cytometric analysis of surface receptors associated with the tissue of origin is helpful for a detailed characterization of solid and hematopoietic tumor types with respect to their surface expression of growth factor receptors, as well as molecules important for the interaction with immune effectors cells, such as MHC molecules as ligands for T cells, as well as adhesion molecules. Here, most common strategies for the definition and characterization of human and murine tumor cells are presented, along with several practical examples.

#### 1.2 Introduction

Tumor cells are derived from non-transformed cells of either hematopoietic, epithelial, endothelial, neuroectdermal or mesenchymal origin, resulting from a sophisticated process of malignant transformation. Therefore, the origin of a tumor cell indicates which markers are suitable for its flow cytometric characterization. Since hematopoietic tumor cells, i.e., leukemias and lymphomas, are derived from their non-malignant counterparts, they retain expression of the pan-leukocyte marker CD45, originally defined as the leukocyte common Ag (LCA). In this section, the definition of subsets of leukemias and lymphomas will be briefly mentioned in the context of EuroFlow (https://www.euroflow.org/), a consortium developing novel flow cytometric diagnostic tests. Solid tumor cells, on the other hand, do not express hematopoietic markers and therefore the absence of CD45 can be used to discriminate solid tumor cells from all hematopoietic cells, including progenitor cells (HSC, see section XIII.3 Human: Hematopoietic stem cells [1584]). In the case of tumor tissue preparations, this basic discrimination of solid tumor cells from hematopoietic cells is especially helpful because it represents the first step for a detailed characterization of solid tumor cells.

##### Hematological malignancies:

The classification of leukemias and lymphomas can be guided by flow cytometry and the procedure has been harmonized, standardized and successfully integrated into the clinical immunophenotying routine [1585]. Of note, the EuroFlow (www.euroflow.org) consortium, represented and headed by Jacques M. van Dongen, has designed panels for n-dimensional flow cytometric immunophenotyping of leukemias and lymphomas. Beyond the staining and gating protocols, the group has developed novel computerized evaluation procedures for the characterization and quantification of human hematopoietic malignancies. The EuroFlow guidelines represent the gold standard of hematopoietic malignancy immunophenotyping (https://www.euroflow.org/). For research laboratories working on hematopoietic malignancies in patients, it is important to mention that virtually all hematopoietic malignancies are accompanied by a disturbed distribution of the lymphocyte subsets in peripheral blood. Therefore, a detailed knowledge of the “normal” distribution of leukocytes in healthy individuals is instrumental for the analysis of the influence of malignant cells on hematopoiesis and immune function. To this end, “The ONE Study” group has established an advanced flow cytometry panel for human immune-cell phenotyping in order to define the distribution of the most important T-cell, B-cell, NK-cell and monocyte, dendritic cell subsets in healthy individuals [1586]. In addition, the International Society for Advancement of Cytometry (ISAC, https://isac-net.org/), the CIP consortium (CIMT immunoguiding program, https://www.cimt.eu/about-cip-1) of the Cancer Immunotherapy Consortium (CIMT, http://www.cimt.eu), the International Clinical Cytometry Society (ICCS, http://www.cytometry.org/web/index.php), the Federation of Clinical Immunology Societies (FOCIS, http://www.focisnet.org/) represent other initiatives which aim to harmonize and standardize protocols for immunophenotyping, primarily of human peripheral blood. The tremendous efforts of these consortia to establish guidelines, protocols and tools for the quantification of leukocytes, tumor cells and immune responses will be instrumental not only for research projects but also for future clinical studies, in particular those with immunological endpoints.

##### Solid tumors:

Due to their origin from a given tissue, solid tumors are categorized into different entities (see Tables 142-144). Tumor cells are not, or only at very low frequencies, present as circulating tumor cells in the blood, rather being located in the primary or metastatic tumor tissue. Since tumor tissue comprises a peculiar “contexture” of tumor cells, stroma, endothelial and other parenchymal cells, as well as infiltrating immune cells, it is important to discriminate the tumor cells from all the non-malignant cells by tailored flow cytometry panels.

The recent clinical advances in immunotherapy of human solid tumors could only be achieved using sophisticated preclinical mouse models. Since the early days of transplanted tumor cells into immunodeficient mice, numerous elegant mouse models with spontaneously developing tumors based on germline or inducible mutations have been developed over the past decade [1587] (see murine tumor cells section). More recently, humanized mouse models using severely immunodeficient mice reconstituted with human peripheral or even hematopoietic stem cells have gained tremendous insight into immune recognition of human tumor cells, escape mechanisms, and opened the door for new therapeutic approaches that finally made their way into clinical application [1588].

In light of the complexity and heterogeneity of tumors and tumor biology, we will focus on the basic guidelines and provide in this chapter an overview on suitable surface markers for the characterization of human and murine tumor cells.

#### 1.3 Step-by-step sample preparation

For the establishment of flow cytometry panels for hematopoietic and solid tumors, it is helpful to start with tumor cell lines that available from research groups, several vendors and the ATCC (American Type Tissue Collection, https://www.lgcstandardsatcc.org/). Recommended surface Ags for flow cytometry staining of human solid tumor cells are listed in Table 145 and for murine tumor cells in Table 147. The procedures for cell staining, fixation, sample acquisition, data analysis and visualization are identical to the general recommendations for direct and indirect surface marker staining and the intracellular staining protocols, which are presented in Chapters I: Cytometry equipment, Chapter II: Setup – Instrument setup and quality control and Chapter III: Before you start: reagent and sample preparation, experimental design.

##### 1.3.1 Preparation of tissue, staining of samples and gating strategy.

The staining protocols for human or murine tumor cell lines, or tumor cells derived from fresh tumor tissue after enzymatic digestion, follow the general recommendations summarized in Chapter I Cytometry equipment, II Setup—Instrument setup and quality control and III Before you start: Reagent and sample preparation, experimental design in [22]. With respect to mechanical dissociation for instance, by Gentle-MACS procedures, and enzymatic digestion, the protocols do not differ between human or murine tumor tissues. The experimental protocols presented in the chapter III section 3 “Preparation of Single Cell Suspensions” in [22] are recommended using enzymatic digestion with DNAse, collagenase and/or hyaluronidase, which are known not to affect surface expression of the molecules listed in Tables 145 and 147. In brief, after enzymatic digestion of tumor tissue, Ficoll or Percoll density centrifugation and optional lysis of erythrocytes, the resulting single cell suspensions should be comprised of tumor cells, endothelial cells, fibroblasts and infiltrating immune cells. Ideally, these cells should be immediately applied to flow cytometric analyses using the flow cytometry staining protocols provided for single cell suspensions but they can also be cryopreserved in liquid nitrogen as living cells for later analyses but the potential instability of some surface markers should be taken into account. Below, examples of staining protocols are provided in more detail.

##### 1.3.2 Direct and indirect staining of surface molecules expressed by solid tumor cells isolated from tissue or in vitro culture.

Single cell suspensions from tumor tissue:

After preparation of single cell suspensions from tumor tissue, solid tumor cells, for instance carcinoma cells of epithelial origin, can be detected by a flow cytometry panel, using the CD45 marker to exclude hematopoietic cells, in combination with epithelial markers for the identification of carcinoma cells. In the following protocol, steps a or b should be followed depending on the indicated circumstances. Steps indicated by a number only are common for all circumstances.

**Figure 143.**
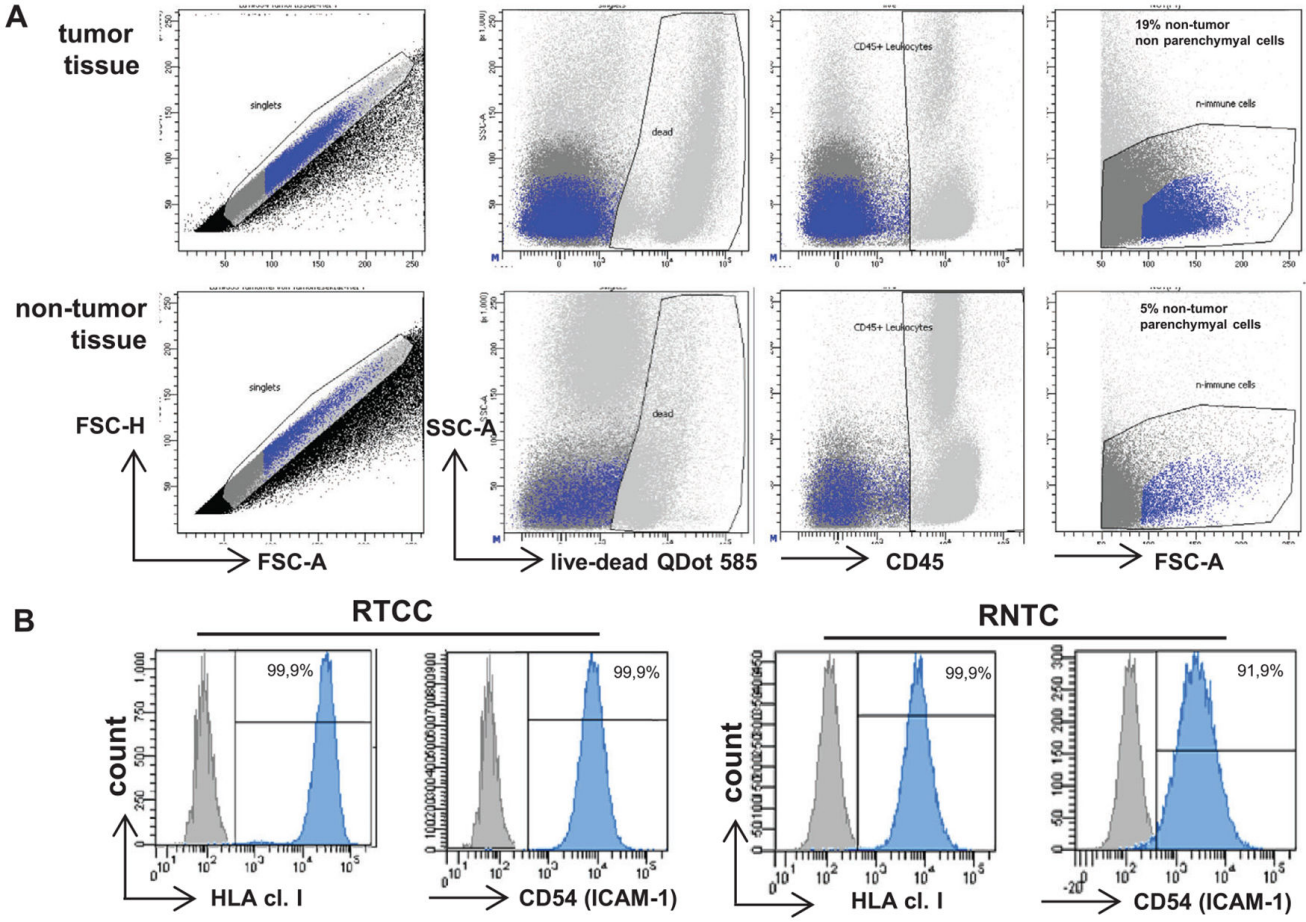
Single cell preparations from human tumor vs. non-tumor tissues and characterisation of human tumor vs. non-tumor epithelial cells. (A) Human tumor (upper row) and adjacent non-tumor tissue (lower row) was obtained as surplus tissue in the course of a pulmonary tumor resection with informed consent (MHH Nr. 1747). After tissue digestion, single cells were stained with a live/dead dye (QDot585) and anti-human CD45 (Alexa-Fluor700) mAb. The hierarchical gating strategy starts with exclusion of doubles and aggregates in the FSC-A/FSC-H plot, followed by exclusion of dead cells in the QDot585/SSC-A plot and leukocytes, i.e., CD45-positive cells in the CD45/SSC-A plot. The remaining living CD45-negative single cells are shown in the FSC-A/SSC-A plot and in the blue gate, epithelial cells including tumor cells in the tumor tissue, can be identified according to their relative size and granularity. (B) A renal tubular cancer cell (RTCC) and the corresponding non-tumor tubular cell line (RNTC) derived from tumor and adjacent non-tumor tissue of the same patient are compared with respect to surface expression of the following markers: HLA class I (mAb W6/32) and the adhesion molecule ICAM-1 (CD54, mAb gp89). All primary mAb are mouse IgG2a and were stained with a goat-anti-mouse PE-labeled secondary Ab.

1a. Staining strategy for single cell suspensions derived from tumor tissue:

Single cell suspensions of tumor tissue should be stained first with the unlabeled mAb specific for the surface molecule of interest on the tumor cells, followed by the respective secondary mAb and finally a directly labeled CD45 Ab to exclude hematopoietic cells. Figure 143A shows single cell preparations from human tumor tissue and the non-tumor tissue counterpart, stained with CD45 to discriminate between leukocytes and parenchymal cells. Details of the gating strategy are given below in section 1.5 “Data analysis”.

1b. Staining strategy for cultured tumor cells:

Cultured adherent tumor cells are detached and singularized by washing with 5 ml PBS followed by treatment with 0.05% trypsin/0.02% EDTA solution (1 ml per T25 culture flask) for 2-5 min, gentle shaking and detachment by adding 5 ml medium (RPMI1640 + 5% heat-inactivated FBS).

2. The cell count of the single cell suspension is determined using trypan blue solution for discrimination of dead cells.

3. 1 x 10^5^ cells of the tumor suspension or 1× 10^5^ cultured tumor cells for each tube are pelleted by centrifugation (800 g, 5 min) in FACS tubes and resuspended (15 sec vortex) in PBS or FACS buffer (PBS, 1 % FBS, 0.1 % Na-azide).

4a. for indirect staining, unlabeled monoclonal Ab or isotype control mAb solutions (previously titrated for each Ag to determine the optimum concentration to use) are added in a volume of 50 μl to the single cell suspensions for 30 min at 4°C. After washing twice with 500 μl PBS or FACS buffer, and vortexing, goat-anti mouse Ab solutions labeled with FITC, PE, APC, pacific blue or other fluorochromes (100 μl of dilutions between 1:100 and 1:200) are added for 30 min at 4°C in the dark.

4b. for direct staining, cells are resuspended in 50 μl FACS buffer and directly labeled titrated mAb (usually 1-5 μl) are added for 30 min at 4°C in the dark.

5. after two washing steps with 500 μl PBS or FACS buffer, cell suspensions are stained with a titrated directly labeled CD45 Ab for 30 min at 4°C in the dark for the exclusion of hematopoietic cells.

6. after three washing steps, cells are resuspended in 150 μl FACS buffer if measured immediately or in FACS fixation buffer (PBS, 1 % FCS, 1 % paraformaldehyde) and stored at 4°C until measurement.

7. exclusion of dead cells without cell fixation is highly recommended, for instance by live/dead staining with 2 μl propidium iodide (PI) stock solution (20μg/ml PI, PE channel) which requires immediate acquisition of the cells. Other live/dead staining protocols for instance using 7AAD (7-aminoactinomycin D) or other live/dead fluorochromes are available in different colors. Most protocols recommend staining for 10 to 15 min and washing steps are according to the manufacturer’s instructions.

An example of the comparison between human renal tubular cancer cells (RTCC) and renal non-tumor tubular cells (RNTC) from the same individual is shown in Figure 143B. Surface expression of HLA class I, CD155, CD166 and CD54 was compared between tumor (RTCC) and renal non-tumor tubular epithelial cells (RNTC) showing indeed some differences in the density of these molecules.

#### 1.3.3 Detection of circulating tumor cells in the peripheral blood and bone marrow.

The detection of circulating tumor cells in the peripheral blood and bone marrow has clinical relevance for several forms of carcinomas and sarcomas in terms of disease staging and treatment response [1589]. Although molecular methods such as real-time PCR of tumor-specific mRNA expressed by carcinoma, sarcoma or melanoma tumor cells etc., recently called “real time liquid biopsy”, have a higher sensitivity compared to flow cytometry, flow cytometry is still valid for the quantification and characterization of circulating cancer cells [1590]. Under non-malignant conditions, cells of epithelial, mesenchymal or neuroectodermal origin cannot be detected in blood or bone marrow aspirates. However, the process of metastasis formation is associated with dissemination of malignant cells through the blood stream and bone marrow. Therefore, disseminating cancer cells are detectable in these compartments but at very low frequencies that are close to the detection limit of <0.01% cells within the gate for living cells. Hence, enrichment techniques such as Ab-based magnetic positive or negative selection are used to increase the sensitivity of detection. For the quantification of tumor cells, the direct or indirect staining protocol outlined in 10.3.2 can be combined with the CD45 marker for the exclusion of all leukocytes. The epithelial markers Ep-CAM (CD326) or cytokeratin 18 (CK18) are suitable markers for the detection of carcinoma cells. For sarcomas, the mesenchymal marker (CD99) is recommended and for the detection of melanoma cells, growth factor receptors like c-Met or PDGF-R are appropriate markers, and although not tumor-specific are characteristic for the tissues of origin.

#### 1.4 Materials

- Media: for example RPMI-1640 + 5% heat-inactivated FBS
- Buffers: PBS, 0.05% trypsin/0.02% EDTA, flow cytometry-buffer (PBS + 1% FBS + 0.1% Na-azide), fixation buffer (PBS + 1% FBS + 1% Paraformaldehyde)
- Staining reagents: for live/dead discrimination, i.e., LIVE/DEAD Fixable Yellow Dead Cell Stain Kit (ThermoFisher Scientific; Qdot 585, violet laser 405 nm) or 7AAD; note: many fixable live/dead dyes require PBS for staining
- Antibodies: for human and mouse tumor-specific Abs see tables 145 and 147. For exclusion of human hematopoietic cells, CD45-mAb (for example, clone HI30, AF-700-labeled, BioLegend) can be included in the staining panel
- Flow cytometer: BD LSRII (blue, red, violet laser)

#### 1.5 Data analysis

The hierarchical gating strategy should follow the recommendations shown in Figure 143A, starting with FSC-A/FAC-H to exclude doublets and cell aggregates but taking into account the different sizes for leukocytes and the non-immune cell fractions containing tumor cells as well as other cell types. In this FSC-A/FSC-H gate, dead cells should be excluded by live/dead staining (in this case with a QDot585 dye). In the case of staining tumor cells in single cell suspensions derived from tumor tissue, leukocytes should be excluded by gating only on single cells negative for CD45 in the plot showing CD45 vs. SSC-A. Then, the non-immune (CD45-negative) cells are displayed in a FSC-A/SSC-A plot to allow detection of the postulated tumor cell proportion, which can be further identified by surface markers of interest, for example EGFR for carcinoma cells using histogram or dot plot images depending on the marker combinations. In Figure 143B, renal tubular cancer cells (RTCC) are compared to renal non-tumor tubular epithelial cells (RNTC) with respect to expression of HLA class I, CD155 (poliovirus receptor), CD166 (ALCAM), ICAM-1 (CD54) molecules. Although, pairs of tumor vs. non-tumor cells from one individual are rare, they allow a comparison of the expression density of several surface markers involved in immune recognition like HLA class I or adhesion molecules (CD54).

##### 1.5.1 Specific considerations for human solid tumors.

Details of suitable Ags and the respective monoclonal Abs are given in Table 145 for human tumor cells and Table 147 for murine tumor cells as well as a direct comparison of human and murine tumor cells in Table 148 (see below).

In contrast to leukemias and lymphomas, solid tumor cells are classified according to their originating cell type, i.e., tumor cells derived from i) epithelial cells are defined as carcinoma cells, ii) mesenchymal cells as sarcoma cells, iii) neuroendocrine tumors are defined as originating from endocrine glands and iv) neuroectodermal tumors are defined as originating from neuroectodermal cells of the skin or brain. This classification is identical for all species, such as humans, non-human primates, dogs, cats, and rodents. Although many solid tumor cells can express a variety of tumor-associated Ags (TAA), including cancer-testis (CT), carcino-embryonal (CEA) and neo-Ags, most of these Ags are not suitable for flow cytometric characterization of tumor cells due to either their poor expression, intracellular localization or simply the lack of specific Abs [1591, 1592]. Therefore, the characterization of solid tumor cells relies on surface markers associated with their tissue origin, in combination with exclusion markers for hematopoietic cells such as CD45.

The induction of tumor-specific immune responses can result in immune escape mechanisms through which the tumor cells aim to evade their recognition and elimination by effector cells, in particular T cells and NK cells. One frequent mechanism of immune evasion is mediated by loss or downregulation of major histocompatibility complex (MHC) or human leukocyte Ag (HLA) class I molecules because, in the absence of MHC class I molecules, recognition of tumor cells by T cells is prevented. Mutation or deletion of beta-2-microglobulin (β_2_m), leading to MHC class I-deficiency, represents a major tumor escape strategy occurring *in vivo* in cancer patients, as well as in murine tumor models. Thus, MHC class I (mouse H-2) or HLA class I (human) surface staining by flow cytometry is highly recommended for all immunological experiments with solid tumor cells [1593]. In addition to T cells, NK cells can also recognize tumor cells but via other receptor/ligand interactions. Expression of ligands for NK-cell receptors, for instance NKG2D ligands (NKG2DL), are important for recognition by the activating NKG2D receptor and for the sensitivity of tumor cells to NK cell-mediated recognition and tumor-cell elimination [1594]. NKG2D (CD314) belongs to the group of activating receptors that are conserved between humans, non-human primates and rodents and are expressed by NK and CD8^+^ T cells. In contrast to NKG2D, MHC class I molecules, human HLA-C in particular, serve as inhibitory ligands for NK cells by specific binding to inhibitory receptors of the killer-immunoglobulin-like (KIR) or C-type lectin (CD94/NKG2A) families. Thus, NK-cell recognition of tumor cells is regulated by a balance between activating and inhibitory signals derived from interactions with the respective ligands on the surface of tumor cells. In order to investigate the immunogenicity of tumor cells, it is therefore, recommended to determine the surface expression of NKG2D ligands on human or mouse tumor cells (Tables 145, 147 and 148). Moreover, these ligands for T-cell and NK-cell receptors can be modulated during tumorigenesis, for instance MHC class I and NKG2D are targeted by oncogenic signaling via mutated MAP kinase signaling [1595].

Surface expression of adhesion molecules such as ICAM-1, and VCAM should also be included in the flow cytometric characterization of solid tumor cells due to their increased expression upon development of metastases in human tumors and mouse models and, thus, their relevance for T-cell and NK-cell activation, as well as for the formation of metastases. Besides these surface molecules, which are commonly expressed by non-malignant as well as malignant cells of both hematopoietic and parenchymal origin, solid tumor cells can be also characterized by cell fate markers. For instance, splice variants of CD44, especially CD44v6, have a long-standing and controversial history as potential “tumor stem cell” markers, together with the hematopoietic stem cell markers CD34, CD133 with a recent revival of CD24 as potential prognostic marker for some carcinomas [1596, 1597]. A selection of the most relevant human cancers, grouped into carcinomas, sarcomas, neuroectodermal tumors and their tumor biology, “the hallmarks of cancer”, is given below with the respective recommendation for their flow cytometric characterization.

###### 1.5.1.2 Human carcinomas.

Carcinomas, i.e., epithelial tumors, represent the most frequent human cancers https://www.cancerresearchuk.org/about-cancer/type and their malignant transformation is often based on “driver mutations” in growth factor receptors, receptor tyrosine kinases in particular, as well as their downstream signaling pathways. For the identification of carcinoma cells, epithelial markers such as CK18 and CK8 are useful although they have to be detected by intracellular staining procedures [1598]. In addition, epithelial cells selectively express growth factors such as epidermal growth factor receptor (EGFR), platelet-derived growth factor receptor (PDGFR), fibroblast growth factor receptor (FGFR), Her-2, c-Met and others [1599]. These surface receptors often directly contribute to tumorigenesis by carrying “tumor-driving mutations” in their signaling domains; providing constitutive proliferative signals independently of the availability of growth factors. Therefore, these receptors can be useful for the identification and characterization of tumor cells in terms of their growth factor receptor repertoire. Importantly, the intracellular protein vimentin serves as a specific marker for the discrimination of tumor cells from fibroblasts. Some of the most frequent human carcinomas are listed in Table 142 together with their originating epithelial cell type (see the links in the notes of the table and refs. [1600, 1601]).

###### 1.5.1.3 Human sarcomas.

Mesenchymal tumors, i.e., sarcomas (https://www.cancerresearchuk.org/about-cancer/soft-tissue-sarcoma), develop from tissue cells originating from mesenchymal progenitors and manifest primarily in soft tissue like fat, muscle, tendons, nerve or connective tissue cells, blood and lymph vessels or fibroblasts (Table 143). The family of osteosarcomas, including Ewing osteosarcomas, comprise a severe form of juvenile sarcoma with manifestations preferentially in the bone, bone marrow and organs like the lung or, in rare occasions, the kidney. For the flow cytometric detection of Ewing sarcoma cells in the peripheral blood of patients, CD99, the MIC2 gene product, which is normally expressed by osteoclasts and leukocytes, has been proposed in conjunction with the absence of CD45 [1602]. Kaposi’s sarcoma represents a virally induced form of sarcoma mediated by the human herpesvirus 8 (HHV8), also called Kaposi’s sarcoma-associated herpesvirus (KSHV). The viral HHV8 genome contributes to dysregulation and tumorigenesis by its manipulation of mechanisms regulating viral latency and lytic replication [1603]. For bone and soft tissue sarcomas, dysregulation of the Hippo signaling pathway has been shown to affect several surface receptors including EGFR, E-cadherin, CD44 and tight junctions indicating that oncogenic signaling can impinge on the stability of these surface receptors as markers for sarcoma cells [1604].

###### 1.5.1.4 Human neuroectodermal tumors.

Neuroectodermal tumors, i.e., malignant cells derived from neuroectodermal cells, belong to less prevalent but life-threatening cancers such as melanoma (black skin cancer) and several forms of brain cancer (Table 144). In malignant melanoma, melanocytes originating from neuroectodermal cells acquire “driver” mutations in components of the MAK kinase signaling, most frequently in the BRaf kinase (with the highest prevalence being the BRaf^V600E^ mutation) or in the upstream NRas GTPase [1605]. Although these mutations cannot directly be utilized for the flow cytometry of melanoma cells, their mutation status may have an impact on the recognition by T-cells and NK-cells [1606, 1607]. Since melanoma cells do not express unique tumor-associated surface molecules, there are no specific flow cytometry panels available for the discrimination of malignant melanoma cells from melanocytes. However, melanoma cells can be detected in single cell suspensions of tumor tissue, by combinations of ICAM-1, MUC18/MCAM (CD146) and the exclusion of CD45.

Several forms of brain cancers are derived from neuroectodermal cells including some of the most aggressive brain tumors like glioblastoma with the malignant cells being derived from glial cells [1608]. Besides their poor MHC expression, glioblastoma cells utilize a broad selection of immune evasion strategies that are in part responsible for their aggressive nature and the resulting poor survival of glioma patients [1609]. Other forms of brain tumors are represented by astrocytomas, a group of differentially graded variants, i.e., diffuse, polycystic and anaplastic astrocytoma with different degrees of aggressiveness. For gliobastoma, the GD2 and CD90 Ags are accepted as tumor-associated surface molecules for flow cytometry and also as targets for chimeric Ag-specific T cell (CAR-T cell) therapeutic strategies. Due to the lack of additional, reliable and tumor-specific surface markers for flow cytometry, molecular characterization, i.e., expression profiling, is currently used for a more detailed classification at the level of gene profiles, signaling pathways, and regulatory networks. Despite these molecular analyses, the cellular origin is still controversial ranging from stem cell-like precursors to neuronal stem cells [1610].

#### 1.6 Pitfalls

The major pitfall in the characterization of tumor cells is the selection of surface Ags suitable for either the discrimination between tumor and non-malignant cells and/or the definition of their anti-genicity, respectively. Since many tumor cells maintain their overall surface expression profile and rather modulate the density of certain surface receptors or ligands, it is highly recommended to perform a rather broad analysis of their receptor and ligand profile with standardized protocols that allow a quantitative assessment for each surface molecule. In Figure 143B, the quantitative differences between a tumor and non-tumor renal epithelial cell line are shown with respect to HLA class I and ICAM-1 (CD54) expression. In the case of single cell preparations derived from fresh tissue, it is important to include live/dead staining in combination with an exclusion of leukocytes in order to identify tumor cells that may represent a minor cell type within the entire complex tumor tissue. Therefore, a stepwise hierarchical gating strategy is instrumental for the identification of tumor cells.

#### 1.7 Top tricks

In the context of tumor cell analyses, one of the top tricks is the direct comparison of tumor vs. non-tumor samples, i.e., tissue or cell lines because the genetic alterations in the course of malignant transformation result in a gradient of changes rather than in an on/off situation for most surface markers. Therefore, a side by side analysis of tumor and non-tumor samples allows a direct comparison of the expression levels of your marker of interest and, hence, this facilitates the interpretation of general or even individual changes associated with tumor development or progression, respectively. Addition of genetic analyses can of course further improve tumor cell and tissue characterization at the molecular level.

#### 1.8 Clinical relevance statement

Figure 143 in this section illustrates that even in solid tumor tissue, the proportion of tumor cells may be rather small and, therefore, a clear strategy to discriminate tumor from non-tumor as well as from hematopoietic cells is very important. The hierarchical gating strategy shown in this section is applicable for analysis of human and murine tumor cells and can be adapted to the tumor entity of interest. The key conclusion from such analyses is that the quality of the flow cytometric characterization of tumor cells isolated from native tumor tissue predetermines the quality of subsequent analyses like deep-mRNA- and single cell-mRNA sequencing.

#### 1.9 Summary of the phenotypes

This is detailed in Table 145.

### 2 Murine tumor cells

#### 2.1 Overview

A plethora of murine tumor models exist, which allow detailed studies of human malignancies and the immunological components and contributors inside the tumor microenvironment. Besides transplantation of well-established syngeneic tumor cell lines, genetically engineered, and carcinogen-induced models are available characterized by endogenous tumor development depending on the experimental question. All of these models are incredibly helpful tools for cancer immunotherapy research applications.

#### 2.2 Introduction

Syngeneic tumor models are induced through inoculation of *in vitro* cultured tumor cells into immunocompetent hosts. Depending on the tissue origin of the tumor that was used to generate the tumor cell line, carcinomas, melanomas, neuroectodermal tumors etc. can be transferred into either wildtype of genetically modified mice (Table 146). In general, these models are characterized by a high reproducibility, rapid tumor growth as well as a broad spectrum of genetic manipulations [1611]. However, they lack the native tumor microenvironment and genetic heterogeneity [1611, 1612]. In contrast, genetically engineered mouse models for the de novo-development of tumors represent an enhancement to the syngeneic models as autochthonous tumor growth provides a native tumor microenvironment and genomic instability can be included [1611]. Transgenic approaches are used to generate genetically engineered mouse models and they can be further divided into germline versus non-germline genetically engineered mouse models ([1612] and references therein). Furthermore, carcinogens can be used to induce tumor formation, i.e., fibrosarcomas, melanomas, colon carcinomas etc. (Table 146) ([1612] and references therein). *De novo* tumor formation occurs in the respective tumor microenvironment provoked as a result of genomic instability, gain or loss of function mutations and classical proto-oncogenes ([1612] and references therein).

For the flow cytometric characterization of murine tumor cells, both hematopoietic tumors like mouse leukemias and lymphomas, and solid tumors like carcinomas of the mouse breast, liver or colon, melanomas or sarcomas, the same recommendations can be applied as outlined above for human tumor cells. Since the numerous mouse tumor models cannot be discussed here comprehensively, only general remarks are provided regarding flow cytometry of murine tumor cells. Furthermore, the same surface molecules can be utilized for their characterization by flow cytometry as are listed in Table 147 showing a selection of known monoclonal Abs for mouse Ags. In addition, the protocols do not differ from the general protocols of direct, indirect surface and intracellular staining. Furthermore, the protocol above in section section XIV 1.3.2 Human tumor cells Direct and indirect staining of surface molecules expressed by solid tumor cells isolated from tissue or in vitro culture can also be used for staining of murine tumor cells. In the case of unlabeled mAbs, the secondary mAb needs to be adapted to the species of the mAb, rat or goat for instance, and then, fluorochrome-labeled goat-anti-rat or rabbit-anti-goat secondary Abs have to be utilized for indirect flow cytometry.

#### 2.3 Step-by-step sample preparation

The protocols for murine tissue harvest and digestion, single cell isolation, staining and acquisition are identical to the protocols for human tissues (see section XIV. 1.3 “Step by step sample preparation in human tumor cells”).

#### 2.4 Materials

Please adhere to section XIV. 1.4 Materials in the human tumor cell section for materials and strategies of marker combinations. In the context of murine tumor models, it is important to mention the potentially cross-reactivity of Abs against proteins, which are highly conserved between species, tumor-suppressor and proto-oncogenes, in particular. This may result in the choice of Abs derived from less prominent species like donkey or camel and respective secondary Abs.

#### 2.5 Data analysis

Due to similar or even homologs of surface molecules for human and murine tumor cells as described above, the hierarchical gating strategy for murine tumor cells should follow the recommendations in Figure 143A and Section XIV. 1.3 Step-by-step sample preparation, XIV. 1.4 Materials, XIV. 1.5 Data analysis.

#### 2.6 Pitfalls and top tricks

Please see the section 1.6 Pitfalls and 1.7 Top tricks for human tumor cells above.

#### 2.7 Summary of the phenotypes

The details of phenotypic markers are given in Table 147 and 148.

#### 2.8 Key information human vs. murine

This is detailed in Table 148 and detailed below.

- Depending on the (cellular/tissue) origin, distinct human tumors can be discriminated. Several mouse models exist to recapitulate the different human malignant tumors (see Table 146).
- For the flow cytometric characterization of murine tumor cells, and solid tumors, the same step by step recommendations for sample preparation can be applied as outlined for human tumor cells (see section 6.10.3.2).
- Due to partly different nomenclatures of human versus mouse molecules, Table 148 summarizes phenotypic key differences between human and mouse tumor cells.

## XV Brain and neural cell phenotypes

### 1 Human brain and neural cells

#### 1.1 Overview

In contrast to peripheral immune cells, the application of flow cytometry for the resident cells of the central nervous system (CNS) is most often not the method of choice. It is limited mainly due to the lack of CNS cell-specific markers, high lipid content (through myelin) and the high integration of cells within the parenchyma. Preparing brain homogenates without severely damaging cells and their processes has proven technically challenging and special caution has to be taken towards keeping the integrity of the Ags during tissue processing. For any of the non-hematogenic cells of the CNS, cell sorting using flow cytometry and subsequent culturing therefore requires specific protocols. Another complication of flow cytometric analysis of CNS cells, however, is the identification of pan-neuronal, astrocyte, microglia and oligodendrocyte markers due to the common origin of many CNS cells as well as the regional and intracellular heterogeneity of the CNS [1613]. CNS cell types from different brain regions and age will require different tissue processing protocols. Here, we provide exemplary approaches for the isolation of different types of human CNS cells, complementary to the ones described in the murine section.

#### 1.2 Introduction

The human and rodent CNS, which include the brain and spinal cord, are composed of many various cell types and states that together orchestrate brain metabolism, neuronal signal transduction and all bodily functions. The primary difference between the human brain and that of other species is the enormous expansion of the neocortex (with its neurons) relative to total brain volume [1614].

Neurons are the primary components of the CNS and transfer chemical and electrical signals throughout the central and peripheral nervous systems. Depending on region and function, several neuronal subtypes exist [1615]. Next to the subsets of neuronal cells, the CNS is also composed of glia cell populations. The cells belonging to the glial compartment are oligodendrocytes, astrocytes and microglia. Oligodendrocytes are cells that form myelin sheaths around neurons, insulating the neuronal processes to enable fast electric signal transduction (reviewed in [1616]). Astrocytes are the most abundant cells in the CNS and have essential roles in its development, homeostasis and disease contexts. Astrocytes are linked via vast intercellular networks, yet despite this global connectivity, astrocytic microenvironments are formed within specific brain regions or within astrocytic sub-populations (reviewed in [1617]). Neurons, astrocytes and oligodendrocytes all originate from neural stem cells (NSCs) with patterned migration and maturation phases during development (reviewed in [1618]). Microglia as well as most of the CNS-associated macrophages, on the other hand, originate from hematopoietic progenitors in the yolk sac which migrate to the brain during early development [1619, 1620]. Microglia are the innate immune cells of the brain and constantly surveil the CNS parenchyma for pathogens and cellular changes. Additionally, tissue resident lymphoid cells in the CNS been observed to contribute both to pathogen defense [1621].

##### 1.2.1 Astrocytes.

Cell sorting using flow cytometry can be used to obtain astrocytes from neonatal to adult tissue. Depending on the downstream application of the sorted astrocytes (culturing, freezing etc.), some considerations need to be kept in mind. Astrocytes are highly integrated within the brain parenchyma. Most of their cell surface Abs are not cell-specific and often found on NSCs, oligodendrocytes and/or neurons. Additionally, only a few fluorochrome-conjugated flow cytometry Abs are commercially available (see table 149). A suitable Ab for flow cytometry of both neonatal and adult murine astrocyte is ATP1B2/ACSA-2 [1622, 1623]. Labeling of intracellular markers such as GFAP requires cell permeabilization and is therefore not suitable for subsequent culture. Combining different Abs can also assist in generating pure astrocyte populations and even distinguish between astrocytic sub-populations. For example, Lin *et al*. [1624] identified astrocyte populations based on Aldh1l1 expression combined with CD51, CD71 and/or CD63 cell surface expression, which showed clear regional specificities.

##### 1.2.2 Neurons.

Neurons are cells that are very sensitive to isolation methods. Traditionally, neurons have been isolated from late-embryonal or early-postnatal murine brain tissue with culture conditions removing contaminating glial cells. This method provides a large number of cells; however, still faces the risk of contamination by other cell types. To obtain highly neuron-enriched populations, flow cytometry of neuron-labeled reported mice such as Thy1 can be used [1625] amongst many other promoters for neuronal subclusters [1626-1628].

Limited neuronal cell surface markers and their respective Abs exist. CD24 labeling has been used for neuronal cell sorting, however sorting procedures dramatically decrease cell integrity and viability of neurons. Thus, flow cytometry of neurons for cell culture from both adult murine and human tissue is not possible. Reports exist that describe tissue fixation for subsequent analysis of cytoplasmic or nuclear neuronal proteins or genes [1629]. Another method to analyze neurons is via nuclei sorting which has proven successful for many applications including genetic [1630, 1631], epigenetic [1632], transcription factor or gene expression profiling [1615]. The Akbarian method of neuronal nuclei isolation provides a useful alternative to analyzing neurons [1633].

##### 1.2.3 Oligodendrocytes.

The study of oligodendrocytes has provided valuable insights into neuronal signal transduction and its changes in disease settings. Especially in demyelinating diseases such as multiple sclerosis (MS) the relationship between myelin sheath integrity and neuronal health has become apparent. As with other brain cells, oligodendrocytes have traditionally been analyzed in vitro and a plethora of culture, protocols exist [1623, 1634-1637]. Based on varying medium supplements and other culture conditions it is thus possible to grow each of the oligodendrocyte subsets and maturation stages ranging from oligodendrocyte precursors cells (OPCs) to pre-myelinating and mature oligodendrocytes. Additionally, the advancement of human cerebral organoid cultures have allowed the study of oligodendrogenesis from radial glia and early OPCs and their interaction with other CNS cell types in a human system [1638]. Previously flow cytometry was mainly used to enrich oligodendrocytes from these cultures and only very few studies are published using flow cytometry [1639] or immunopanning [1640] to directly isolate oligodendrocyte lineage cells from the CNS. In addition, reduced yield and viability of sorted cells are also needed to be considered. However, with the vast availability of various single cell and bulk sequencing approaches, enrichment of small cell populations by flow cytometry or fluorescence-activated nuclear sorting (FANS) have become instrumental to provide biological insight into small and rare cell (sub)populations and of the oligodendrocyte lineage [1641]. An overview of markers for oligodendrocytes and their precursors (OPC) that are available for flow cytometry can be found at the end of the chapter. Additionally, oligodendrocyte reporter mice are also available such as EGFP-labeled CNP mice [1642], Olig2 mice [1643] and NG2 mice [1644].

##### 1.2.4 Microglia.

Microglia are CNS-resident phagocytes that are phenotypically distinct from macrophages of the periphery. To date, microglia have been distinguished from other CNS or myeloid cells by flow cytometry mainly on the basis of the CD45 expression level. Non-myeloid CNS cells express no CD45, whilst perivascular macrophages or infiltrating myeloid cells and leukocytes express a high level of CD45. Microglia on the other hand can be sorted by selecting for intermediate CD45 expression (CD45 int) in the combination with CD11b [1645]. However, it needs to be considered that CD45 expression may be regulated during cell activation. Similar to other CNS cells, microglia isolation requires tissue processing and myelin removal, which may in turn affect their phenotypes and/or viability. Yet, the overlap with Ags shared with other glial/neuronal cell types is not very extensive. The main challenge therefore is not to separate microglia from other CNS cells but from hematogenous macrophages. High expression of the fractalkine receptor (CX3CR1) and no or low expression of chemokine receptor CCR2 of microglia can be used to distinguish CNS microglia from infiltrating macrophages [1362, 1646].Only recently, microglia-specific markers such as Tmem119 were identified, enabling robust selection of mature microglia independent of activation status in human and mouse brains [1647]. Mass cytometry, or CyTOF, has also proven a very powerful tool in characterizing heterogeneity of human and murine CNS-specific myeloid and diverse microglial cell populations in both health and disease on the basis of expression profiles of up to 50 cell and functional markers [1648-1652].

##### 1.2.5 CNS-resident and infiltrating lymphoid cells.

The analysis of CNS-resident and infiltrating lymphoid cells has been an evolving field in recent years, identifying emerging roles also in the steady state and apart from classical defensive and inflammatory functions: brain and meningeal lymphoid cells are present in the healthy human and mouse brain, contribute to brain homeostasis [1653], as well as normal cognitive function such as memory formation [1654]. Pathologic CNS states – both classical inflammatory disorders, but also ischemic, degenerative or traumatic changes [1655] – are almost always associated with an infiltration of lymphoid cells that can be investigated using flow cytometry. All major lymphoid subsets can be found in the CNS in physiologic and/or pathologic conditions, including both CD4^+^ and CD8^+^ T cells [258], ɣδ T cells [1654], ILCs and NK cells [1656, 1657] as well as B lineage cells [1094, 1658].

The flow cytometric analysis is considerably easier compared to many other CNS cell types, as there is a wealth of available surface markers that can be used to identify and subdivide lymphoid cells (refer to chapters). Also, lymphoid cells are relatively robust and thus mostly survive the dissociation of CNS tissue into a single cell suspension. In general, brain dissociation protocols that are developed for microglia, such as a combination of mechanical (and potentially enzymatic) tissue dissociation in combination with density gradient separation [1659].

#### 1.3 Step-by-step sample preparation

##### 1.3.1 From integrated cells to a single cell suspension (example for immune cells).

Depending on the immune cell type of interest different Percoll-based protocols are available. These protocols can be combined with enzymatic digestion, in which the resistance of Ags to sample processing is needed to be validated. We present here a simple, rapid and cheap protocol without a requirement of enzymatic digestion. It is suitable for the isolation of the majority of infiltrating immune cells as well as microglia.

Detailed protocol:

1. Mechanically dissociate neural tissue using a 70μm nylon cell strainer and the plunger of a 5 ml syringe into a 15 ml tube containing complete RPMI medium or HBSS.
2. Centrifuge at 400 × *g* for 10 min at 4°C.
3. Aspirate supernatant. Shortly and gently vortex the cell pellet.
4. Add 6 ml of 37% Percoll (dissolved in Percoll mix, recipe in table with materials) to each tube at room temperature (RT).
5. Resuspend the cell pellet thoroughly by repeated pipetting.
6. Spin in swinging bucket centrifuge at 2800 × *g*, 20 min, no brake, at RT. It is important to use a centrifuge in which the buckets swing out a full 90° to ensure good separation of the myelin layer.
7. Aspirate the myelin layer (upper layer), while taking care of cleaning the wall of the 15 ml tube.
8. Aspirate Percoll solution, leaving approximately 500 μl Percoll to avoid disruption of the cell pellet.
9. Add 6 ml complete medium (or HBSS) (first wash) and resuspend the cell pellet by repeated pipetting.
10. Centrifuge at 400 × *g* for 10 min at 4°C.
11. Completely aspirate medium, vortex pellet, and then add 10 ml complete medium (2^nd^ wash).
12. Centrifuge at 400 × *g* for 10 min at 4°C.
13. Resuspend in FACS block (see materials table) for 15 min and count a diluted fraction of cells (e.g., for a mouse brain, resuspend in 1 ml FACS block; for a single murine spinal cord, use 0.5 ml).
14. Wash the cells in medium and subsequently stain with Abs as desired.
15. Following Ab stain, cells may be fixed in 4% Paraformaldehyde (Electron Microscopy Science) for 10 min at RT. Following a wash step, the cells can be resuspended and stored at 4°C until measurement.

##### 1.3.2 From integrated cells to nuclei (example for neurons).

This method can be used to extract nuclei from >100 mg of fresh or frozen human cortical tissue. Immunotagging with an anti-NeuN Ab robustly stains the nuclei of human cortical neurons for subsequent cell sorting using flow cytometry. Other cell populations beyond neurons can be captured the same way (e.g., astrocytes, oligodendrocytes) if specific nuclear Ags are known and respective Abs are available. Methods to study single neurons in the adult human brain include the use of microfluidic devices as the Fluidigm C1 and ultra-high-throughput droplet-based technologies [1660].

Detailed protocol:

1. Chill a clean B-type 7 ml pestle on ice and add 5 ml of lysis buffer (see materials section XV.1.4) NOTE: Lysis buffer can be prepared on the day prior to sorting, but DTT should be added fresh on the day of use.
2. Transfer 100-500 mg fresh-frozen human surgical or post-mortem frozen brain tissue to the homogenizer containing lysis buffer. Homogenize tissue on ice using a pestle.
3. Put an 8 ml sucrose cushion buffer in a centrifuge tube for Beckman Ultra-clear 14 x 95 mm.

NOTE: Tube size and type have to fit with the ultracentrifuge and rotor system used (here e.g., Beckmann OPTIMA XE – 90 ultracentrifuge and SW-40Ti rotor).

4. Carefully overlay homogenized samples on top of the sucrose cushion without mixing the two solutions.
5. Centrifuge at 30,000 × *g* for 2 h in pre-chilled swing-out rotor at 4°C.
6. After centrifugation, put the tube on ice and carefully remove supernatant. Add 500 μl of 3mM MgCl_2_ in PBS, mix well, and further incubate on ice. After 10 min, gently re-disperse the pellet.

NOTE: Do not vortex nuclei. Always keep nuclei on ice.

6. Pass nuclei suspension through a 40 μm cell strainer into a clean 1.5 ml tube and dilute with 3 mM MgCl_2_ in PBS. Keep a fraction for manual counting.
7. Add mouse anti-NeuN Ab (1:1000), Goat anti-Mouse IgG (H+L) Secondary Antibody, PE-conjugated (1:1000) and incubate for at least 30 min at 4°C on a rotator.
8. Wash cells by adding 1 mL buffer and centrifuge at 300 × *g* for 10 min. Aspirate supernatant completely
9. Manual counting of a fraction of nuclei and quality control with bright field microscopy.
10. Proceed to sorting.

#### 1.4 Materials

##### a. Cell suspension (immune cell protocol)

**Table:** 

Reagent	Manufacturer
Cell strainer size 70 μm Nylon	BD Falcon
5 ml syringe	BD Falcon
15 ml tubes	BD Falcon
Complete RPMI medium (5% FCS, Penicillin/Strepdavidine, β-mercaptoethanol) or HBSS	Sigma
37% Percoll (100% Percoll mixed with respective amount of mastermix of 45 ml 10×PBS, 3 ml 0.6 M HCl, 132 ml H_2_O, pH7-7.2 filter sterilized)	Sigma
FACS block (to a given volume of FACS buffer add 50 μg/ml rat IgG and 1 μg/ml anti-CD16/CD32)	eBioscience

##### b. Nuclei preparation (neurons)

**Table:** 

Reagent	Specifications
7 ml Dounce tissue grinder	Wheaton #357542
Lysis buffer	0.32 M sucrose, 5 mM CaCl_2_, 3 mM Mg(CH_3_COO)_2_, 0.1 mM EDTA, 10 mM Tris-HCl (pH 8), 1 mM fresh DTT, 0.1% Triton X100
Sucrose cushion buffer	1.8 M Sucrose, 3 mM Mg(CH_3_COO)_2_, 10 mM Tris-HCl (pH 8), 1 mM fresh DTT
Beckman ultra-clear 14 x 95 mm centrifuge tube	Beckman Coulter #344060
3 mM MgCl_2_ in PBS	–
40 μm nylon cell strainer	BD Falcon
Mouse anti-NeuN Ab, Clone A60 (1:1000)	Millipore
Goat anti-Mouse IgG (H+L) secondary Ab, PE (1:1000)	

#### 1.5 Data analysis

##### 1.5.1 Neurons.

**Figure 144.**
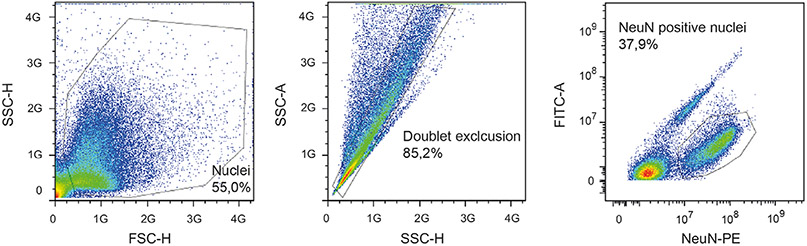
FANS analysis of nuclei prepared from human surgical brain tissue. Nuclei were prepared from frozen adult brain tissue (>100mg), stained with nuclear marker NeuN (monoclonal mouse anti-NeuN, clone A60, 1:1000 and phycoerythrin (PE)-conjugated goat anti-mouse IgG 1:1000) and submitted to sorting. Gating for identification of NeuN-PE positive neuronal and non-neuronal cell populations or respective nuclei was based on the first 20,000 events. FITC fluorescence was included to identify and exclude autofluorescent nuclei. SSC-A/FSC-A gate covered 60% of counts. Next 80% were gated positively following doublet exclusion based on SSC-A/SSC-H. Two distinct populations of nuclei were identified and gated, of which 30% were identified as neuronal (NeuN+) and 70% as non-neuronal (NeuN−) based on the intensity of the phycoerythrin signal (PE-H). FSC and SSC axes are linear, fluorochrome axes are log.

Figure 144 shows a representative blot of the FANS nuclei prepared from human surgical brain biopsy. Nuclei were prepared from frozen adult brain tissue and stained with the neuronal nuclear marker NeuN. FITC fluorescence was included in order to select for autofluorescence.

#### 1.6 Pitfalls

As already mentioned in the introduction, the low yield and reduced cell viability are challenging when wanting to sort CNS cells using flow cytometry. Cell purity is also an issue in the CNS as cell markers are often expressed by more than one cell type and/or are region-specific. Since neurons, astrocytes and oligodendrocytes share the same precursor, many cell markers are shared between them. Microglia and peripheral myeloid cells also share many of the same cell surface markers that can be regulated during activation state and/or pathology, and which need to be carefully selected for. Special attention should be also paid during analysis of Ags on microglia/macrophages as unspecific binding can be a problem and blocking of the FcR before cell labeling should be included in the protocol. In some experiments, microglia and/or macrophages can affect the analysis/sorting of other cells from the CNS. In setups where the focus is e.g., on astrocytes, nonspecific Ab binding can be reduced by an additional microglia/macrophage depletion step.

Additionally, myelin debris can lead to essential problems during data acquisition and labeling of cells with Abs and protocols need to be adapted accordingly [1661, 1662]. This is especially important for the analysis of adult white matter regions. Due to high myelin content, clogging of the instruments may occur and cells of interest might not be detectable in the sample. Therefore, we highly recommend including an effective but gentle myelin removal step when analyzing CNS tissue with flow cytometry.

When analyzing human tissue, it needs to be taken into account that it can only be obtained from very limited fresh biopsies or from postmortem autopsies. Fresh healthy controls are therefore not available and in the case of autopsy material, a high postmortem interval can dramatically reduce tissue quality.

#### 1.7 Top tricks

In the past, the study of CNS resident cells has largely relied on ex vivo slice cultures, histological means or the in vitro culture of neonatal cells. Flow cytometric analysis was traditionally used only on microglial cells which show similar expression of cell surface markers to peripheral myeloid cells. Advances in brain dissociation techniques and the vast array of reporter mice have made the analysis of other brain cells amenable to methods such as flow cytometry, magnetic-activated cell sorting (MACS), immunopanning and single cell or nuclear sequencing. Each of these methods has limitations with regards to cell viability, purity, yield and Ab availability. It is thus essential to choose the most appropriate method depending on the scientific question.

Nevertheless, CyTOF offers a high-dimensional technique for analyzing myeloid cells including microglia on a single-cell level. For mouse tissue, fluorescent reporter lines are a valuable tool for cell sorting using flow cytometry of specific cell populations. When interested in isolating more than one cell type, immunopanning is a suitable method since all cells can be sequentially purified from whole brain cell suspensions [1663]. Neuron isolation of both adult murine and human tissue remains challenging to this day. A suitable alternative when interested in gene expression or nuclear proteins/transcription factors is nuclei sorting via flow cytometry, which also is applicable to immunolabelled neurons and oligodendrocytes and methods such as single-nuclei RNA or ATAC sequencing.

#### 1.8 Clinical relevance statement

Human brain is normally not accessible, as biopsies are very limited and needed for neuropathological workup. One exception are therapy refractory epilepsies and malignant tumors if accessible intravital. For all other conditions, post-mortem brains are the main sources to study human CNS tissue. The gating strategy shown in this section for CNS resident and invading CNS cells is applicable for analysis of these cells especially in neuroinflammatory conditions as e.g., multiple sclerosis patients but also in neurodegenerative diseases and non-diseased controls, as can be seen in [258]. The key conclusion from such analysis is that in all these pathological and non-pathological conditions the human brain is surveilled by memory T cells, providing protection against neurotropic virus reactivation, whilst being under tight control of key immune checkpoint molecules.

FANS similar to the approach shown in this section can be used to analyze to identify dysfunctional neuronal subtypes underlying seizure activity in the human brain [1664]

#### 1.9 Summary of the phenotypes

This is detailed in Table 149.

### 2 Murine brain and neural cells

#### 2.1 Overview

In contrast to human experiments, the application of flow cytometry for the murine cells of the central nervous system (CNS) is facilitated by the use of fluorescently labeled reporter mice, which are listed in this section. We provide an exemplary approach for the isolation of murine CNS cells complementary to the protocols described in the human section. The protocols in both sections can largely be used for both species. Nevertheless, we advise to test different tissue dissociation procedures to optimize for the specific cell-, region-, age- and species-specific requirements.

#### 2.2 Introduction

Mouse lines containing fluorescent labeling of CNS cells also provide a useful tool for flow cytometry. Currently, the Aldh1l1-EGFP reporter line [1665] is the only line labeling solely mature astrocytes in the mouse brain. All other lines are therefore only useful in astrocyte enrichment and contamination by other neural cell types needs to be considered. Reporter lines for microglia/myeloid cells also exist for facilitating cell sorting [1666] and oligodendrocyte reporter mice are also available such as EGFP-labeled CNP mice [1642], Olig2 mice [1643] and NG2 mice [1644]. Traditionally, neurons have been isolated from late-embryonal or early-postnatal murine brain tissue with culture conditions removing contaminating glial cells. This method provides a large number of cells; however, still faces the risk of contamination by other cell types. To obtain highly neuron-enriched populations, flow cytometry of neuron-labeled reported mice such as Thy1 can be used [1625] amongst many other promoters for neuronal subclusters [1626-1628]. However, also in mice sorting procedures dramatically decrease cell integrity and viability of CNS cells. Thus, especially flow cytometry of neurons for cell culture from adult mice shares the challenges of human tissue (see respective human section XV.1).

#### 2.3 Step-by-step sample preparation

##### 2.3.1 From integrated cells to a single cell suspension (example for glial cells).

Obtaining single-cell suspensions from adult brain tissue can be challenging due to the vast extension of cellular processes within the brain parenchyma which upon disruption can influence cell viability, phenotype, and morphology. The commercially available Neural Tissue Dissociation Kit (NTDK, Miltenyi Biotec) or Adult Brain Dissociation Kit (ABDK, Miltenyi Biotec) provide gentle methods to homogenize rodent brain tissue for downstream applications such as cell enrichment and/or culture. The NTDK is recommended for dissociation of neonatal mouse tissue and adult mouse tissue with subsequent microglia isolation. Use of the ABDK is recommended for dissociation of adult mouse tissue with subsequent astrocyte, neuron, or oligodendrocyte isolation. A list of Abs available can be found at the end of this chapter.

Detailed protocol:

- Obtain fresh mouse brain tissue and store in HBSS without
- Ca^2+^ and Mg^2+^ (for NTDK) or D-PBS supplemented with glucose, sodium pyruvate, CaCl_2_, and MgCl_2_ (D-PBS (w), for ABDK). For microglia isolation from adult tissue, the brain should be perfused with PBS before dissociation, to minimize contamination of blood myeloid cells.
- Transfer 400–1000 mg neural tissue into the C tube (Miltenyi Biotec) and add NTDK or ABDK enzyme mixes according to manufacturer’s protocol.
  - For neonatal murine tissue and murine adult microglia use NTDK
  - For murine adult astrocytes, neurons and oligodendrocytes use ABDK
- Load the samples on the gentleMACS with heaters (Miltenyi Biotec):
  - For neonatal murine cells use the program 37C_NTDK_1.
  - For murine adult cells use the program 37C_ABDK_01
- Resuspend cell mixture and pass through a 70 μm cell strainer placed on a new 50 ml tube.
- Wash cell strainer with 10 ml HBSS with Ca^2+^ and Mg^2+^ (for NTDK) and 10 ml D-PBS (w) (for ABDK).
- Centrifuge samples at 300 × *g* for 10 min, 4°C, and then remove the supernatant.
- Resuspend pellet according to the kit used:
  - NTDK: Resuspend in buffer and volume required for further applications.
  - ABDK: Resuspend in D-PBS (w) according to input material and transfer to 15 ml tube
    - 400-500 mg tissue: 3100 μl D-PBS (w)
    - 800-1000 mg tissue: 6200 μl D-PBS (w)
- (ABDK only) Add cold Debris Removal Solution depending on input material (see below), mix well and overlay very gently with 4 ml of D-PBS (w). Centrifuge at 3000 × *g* for 10 min, 4°C with full acceleration and brake.
  - 400-500 mg tissue: 900 μl
  - 800-1000 mg tissue: 1800 μl
- (ABDK only) Aspirate the top two phases and fill up with D-PBS to a final volume of 15 ml. Invert tube three times.
- (ABDK only) Centrifuge samples at 1000 × *g* for 10 min, 4°C with full acceleration and brake.
- (ABDK only) Discard supernatant and resuspend cell pellet in 1 ml 1× Red Blood Cell Removal Solution (diluted in ddH_2_O). Incubate for 10 min at 4°C.
- (ABDK only) Add 10 ml cold PBS + 0.5% BSA and centrifuge samples at 300 × *g* for 10 min, 4°C.
- (ABDK only) Remove the supernatant and resuspend pellet in buffer and volume required for further applications.

#### 2.4 Materials

**Table:** 

Reagent	Manufacturer
OctoMACS with Heaters	Miltenyi Biotec
Adult Brain Dissociation Kit	Miltenyi Biotec
Neural Tissue Dissociation Kit (P)	Miltenyi Biotec
C tubes	Miltenyi Biotec
HBSS with Ca^2^+ and Mg^2^+ (NTDK only)	Life technologies
HBSS without Ca^2+^ and Mg^2+^ (NTDK only)	Life technologies
D-PBS with 0.55 mM glucose, 0.033 mM sodium pyruvate, 0.9 mM CaCl_2_, 0.49 mM MgCl_2_ (pH 7.2, keep cold) (ABDK only)	–
PBS + 0.5% BSA (pH 7.2, keep cold)	–

#### 2.5 Data analysis

##### 2.5.1 Astrocytes.

**Figure 145.**
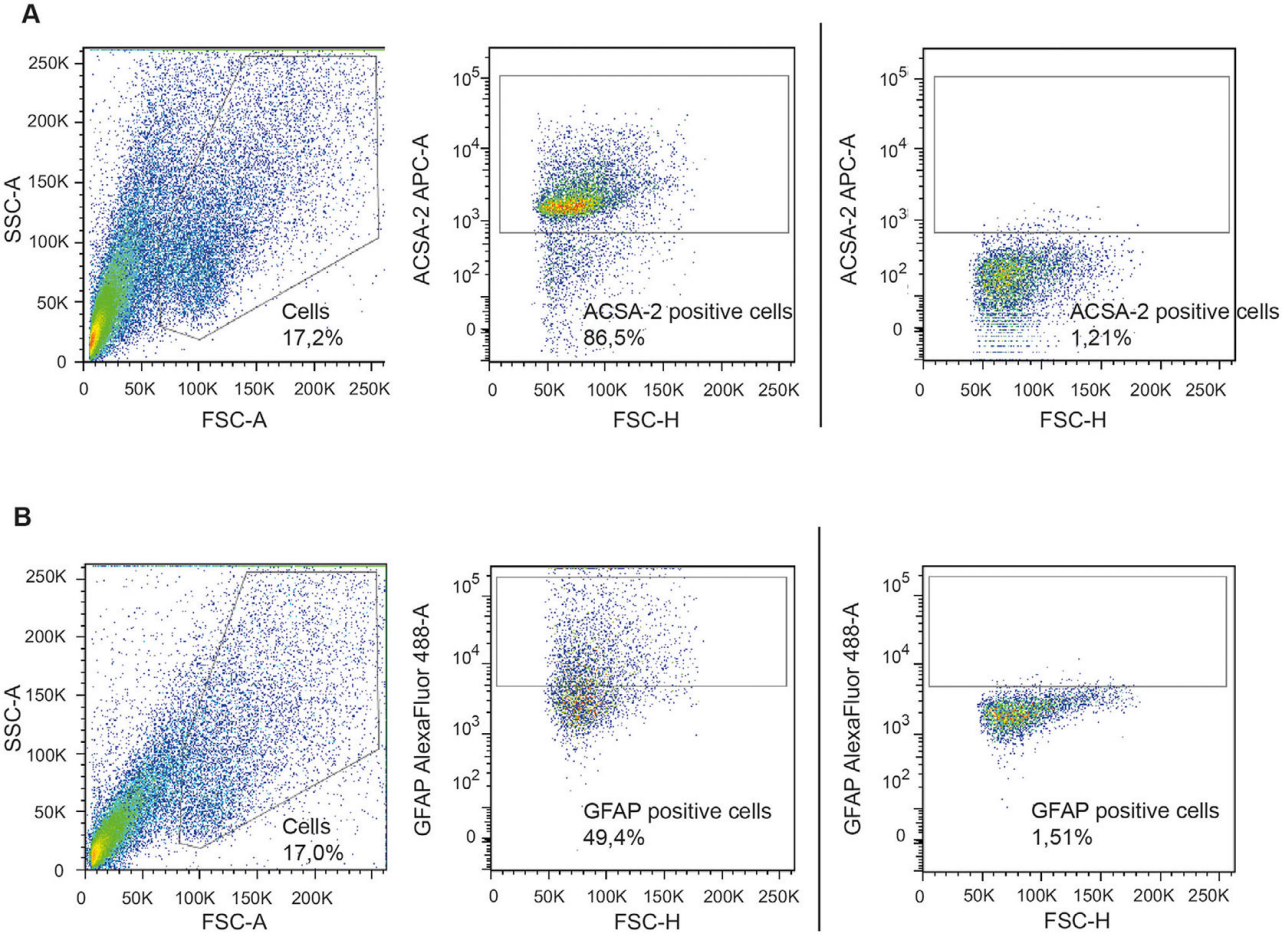
Flow cytometric analysis of murine neonatal astrocytes. (A) Neonatal astrocytes were harvested and stained with the cell surface marker ACSA-2 (recombinant human anti-mouse, APC-conjugated, 1:10 dilution, Miltenyi Biotec). (B) Neonatal astrocytes were harvested, fixed in 2% PFA and permeabilized in 0.5% saponin. Cells were stained with the intracellular marker GFAP (mouse monoclonal, Alexa Fluor-488-conjugated, 1:20 dilution, BD Biosciences). The gating was based on unstained controls for each Ab as shown on the far right. 10,000 cells of the SSC-A/FSC-A gate were set as a stopping point during flow cytometry. FSC and SSC axes are linear, fluorochrome axes are log.

Figure 145 provides representative dot plots of neonatal murine astrocytes labeled with the cell surface marker ACSA-2 (Figure 145A) and the intracellular marker GFAP (Figure 145B). For the intracellular GFAP stain, cells were fixed in 2% PFA and permeabilized with 0.5% saponin.

##### 2.5.2 Microglia.

**Figure 146.**
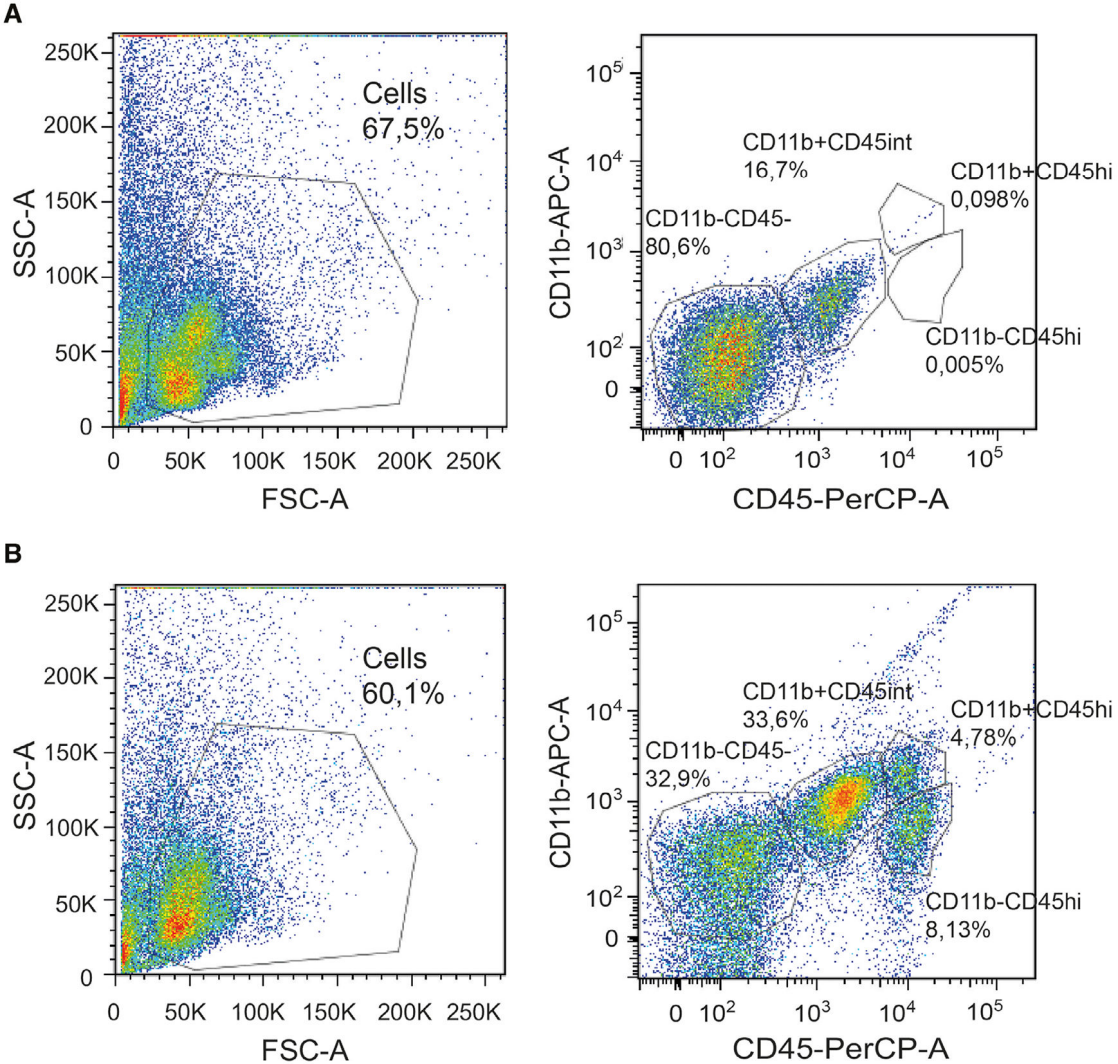
Gating strategy for the identification of the murine resident microglia and infiltrating macrophages and lymphocytes. (A) Analysis of brain cell suspension from a non-immunised wildtype mouse via CD45 and CD11b marker expression. (B) Analysis of monocyte-derived macrophages, infiltrating lymphocytes and microglia of a mouse immunized with MOG35-55 at chronic phase. Cell populations were distinguished by CD45 and CD11b expression levels with macrophages showing high expression of both CD11b and CD45 (CD45^hi^CD11b^+^), microglia showing intermediate expression of CD45 and high expression of CD11b (CD45^int^CD11b^+^), and infiltrating lymphocytes showing high expression of CD45 and no expression of CD11b (CD45^hi^CD11b^−^) and non-leukocytes being negative for both CD11b and CD45 (CD45^−^CD11b^−^). Antibodies used: rat anti-mouse CD45, PerCP-conjugated, 1:200 clone 30-F11, Biolegend; rat anti-mouse CD11b, APC-conjugated, 1:400 clone M1/70, Biolegend. 100,000 cells of the SSC-A/FSC-A gate were set as a stopping point during flow cytometry. FSC and SSC axes are linear, fluorochrome axes are log.

To analyze microglia and macrophages, brain tissue from a mouse immunized with MOG35-55 peptide in the chronic phase of experimental autoimmune encephalomyelitis (EAE), and tissue from nonimmunized mice was mechanically dissociated and myelin removed via Percoll gradient separation as described in the step-by-step preparation. Figure 146 provides representative flow cytometry plots of gating strategies for infiltrating myeloid cells (CD45^hi^CD11b^+^) and, microglia (CD45^int^CD11b^+^), infiltrating lymphocytes (CD45^hi^CD11b^−^) and non-leukocytes (CD45^−^CD11b^−^). In contrast to baseline nonimmunized mice, in which few CD45^hi^ myeloid cells are present and very rare CD45^hi^CD11b^−^ lymphocytes can be detected (Figure 146A), increased infiltrating myeloid cells and lymphocytes are observed in MOG35-55 immunized mice (Figure 146B).

#### 2.6 Pitfalls

Please check the human section (XXX – Human brain and neural cells), common pitfalls are mentioned there

#### 2.7 Top tricks

For studies using neonatal murine CNS tissue, obtaining enriched cell suspensions from mixed cultures or by flow cytometry/MACS, offers a high yield of cells, however cultures may be contaminated by other CNS cells. Additionally, some cells only reach maturity at late postnatal stages and these cultures thus contain many precursor cells. Here, selecting the appropriate cell culture medium before cell sorting may help in selecting for a specific cell type [1667]. Obtaining adult cells from the murine brain or cells from human tissue is best achieved using gentle dissociation techniques. Enriched cell populations can then be generated by flow cytometry or MACS, yet reduced cell viability, yield, and Ab availability need to be considered. Please check the human section for further common top tricks.

#### 2.8 Summary of the phenotypes

This is detailed in Table 149 (refer to common phenotype provided in the human section)

#### 2.9 Key information human vs murine

The neuronal and glial cell phenotypes are similar in human and mice, there are more region- and age-specific differences in cellular phenotypes than differences between species. However, it should be taken into consideration that the human brain has a higher myelin content compared to mice. For flow cytometry analysis, this difference could strongly affect single-cell isolation procedure and thus may result in phenotypic differences. Moreover, there are some marker difference such as F4/80 (characterizing mouse macrophage) and EMR1 (characterizing human eosinophil and some macrophage subsets including microglia). For brain-derived immune cells we recommend the respective immune cell chapter.

- Human and mice share many phenotypical characteristics of CNS cells and brain resident immune cells
- Preparation of single cell suspensions from adult brain tissue is limited by the cytotoxicity of the procedure to the cells due to disruption of their connected processes which can influence also phenotypes
- We advise to establish individual tissue dissociation procedures to account for cell-, region-, age- and species-specific requirements.
- Regeneration is different in rodents and humans, therefore studies with focus in stemcells have to consider different cell populations.
- Also studies with focus on age-related changes on CNS cells are difficult to translate from mouse to man.
- In mice fluorescent reporter lines can be used. A summary of CNS-cell targeted reporter lines is listed below

## XVI Liver cell phenotypes

### 1 Human liver cells

#### 1.1 Overview

This chapter provides detailed step-by-step protocols for the isolation of human intrahepatic leukocytes from perfusion fluid (perfusates), liver tissue biopsy samples (that are deemed surplus to diagnostic requirement), or fine needle aspirates (FNA), and their subsequent staining for flow cytometry. The section also details a step-by-step guide to simultaneously isolate hepatic stellate cells (HeSC) and Kupffer cells (KC) from human liver tissue obtained from surgical resection or explant.

#### 1.2 Introduction

The liver is an organ that exerts both metabolic and immunological functions. Due to a dual blood supply, the liver receives blood from the hepatic artery as well as from the portal vein containing gut-derived food and microbial Ags. There are unique hepatic immune regulatory mechanisms, which induce tolerance against such foreign Ags. The liver is a site of primary T-cell activation mediated by local conventional and unconventional APCs (e.g., hepatocytes and liver sinusoidal endothelia cells [LSECs]) that can either lead to effective immune responses or promote tolerance by induction of T-cell dysfunction and apoptosis. The tolerogenic properties of the liver ensure the maintenance of local and systemic immune tolerance but they also contribute to the persistence of viral infections and tumor metastasis. The liver consists of parenchymal cells (hepatocytes and cholangiocytes) and non-parenchymal cells comprising LSECs, HSCs, and various immune cell populations belonging to the innate and adaptive immune system. The quantitative and qualitative composition of hepatic immune cells markedly differs from that of secondary lymphoid organs. The majority of hepatic DCs display an inactive phenotype. Moreover, the liver contains the largest population of resident macrophages, termed Kupffer cells, and there is an increased proportion of hepatic NK cells, NKT cells, and γδ T cells compared to secondary lymphoid organs [1668, 1669].

#### 1.3 Step-by-step sample preparation: Isolation of intrahepatic lymphocytes (IHL) from perfusion fluid (perfusates)

- Decant perfusion fluid into 50 mL falcon tubes and centrifuge at 700 × *g* (acceleration/deceleration: 9/9) for 10 min at room temperature (RT).
  - Larger centrifugation tubes/containers can be used if the required centrifuge rotor is available.
- Discard supernatant with a 25 mL serological pipette.
  - Caution: the cell pellet may be loose, therefore there is a risk of aspirating it.
- Resuspend the cell pellets from all 50 mL falcon tubes in RPMI-1640 and combine into one 50 mL falcon tube. Once combined top up to a volume of 25 mL with RPMI-1640.
  - Note: The top up volume may need to be increased depending on initial volume of perfusion fluid, or size of cell pellets once spun; e.g., the combination of larger cell pellets may be topped up to a total of 50 mL in RPMI-1640, or more, if necessary.
- In a 50 mL falcon tube, carefully layer each 25 mL volume of the single-cell suspension over 12.5 mL Pancoll/Ficoll^®^ and centrifuge at 800 × *g* for 20 min at RT with the centrifuge break *off*.
- Remove the lymphocyte layer from the interface of the RPMI-1640 diluent and Pancoll/Ficoll^®^ with a sterile Pasteur pipette and transfer to a new 50 mL falcon tube.
- To wash the isolated IHL, add an equal volume of RPMI-1640 and centrifuge at 700 × *g* (acceleration/deceleration: 9/9) for 15 min at RT. Discard supernatant.
- Resuspend IHL in desired media for cell counting and proceed with downstream analysis.

^a^Use minimum acceleration and the no brake setting on the centrifuge to ensure successful density centrifugation.

#### 1.4 Materials: Isolation of intrahepatic lymphocytes (IHL) from perfusion fluid (perfusates)

##### Reagents

- RPMI-1640, stored at 4°C
- Pancoll/Ficoll^®^

##### Equipment

- Centrifuge
- 50 mL falcon tubes
- Serological pipettes
- Pasteur pipettes

#### 1.5 Step-by-step sample preparation: Isolation of IHL from fine-needle aspirates (FNA)

- Fine needle aspirates (FNA) should be transferred on ice from the patient/clinical site to the laboratory in sterile 50 mL falcon tubes containing ice-cold RPMI-1640.
- Centrifuge FNA at 700 × *g* (acceleration/deceleration: 9/9) for 10 min at room temperature (RT). Discard supernatant with a 25 mL serological pipette.
  - Caution: the cell pellet may be loose, therefore there is a risk of aspirating it.
- Resuspend the cell pellet in 1 mL RPMI-1640 and top up to a total of 25 mL with RPMI-1640. Centrifuge at 700 × *g* (acceleration/deceleration: 9/9) for 10 min to wash. Discard supernatant.
- Dilute the 10× RBC Lysis Buffer 1:10 with deionized water (1× solution). Bring the 1× solution to RT before use.
- Resuspend the cell pellet in 2 mL 1× RBC Lysis solution.
- Leave on ice for 5 min.
- Stop the lysis process by adding 25 mL of cold PBS and centrifuge at 700 × *g* (acceleration/ deceleration: 9/9) for 10 min at RT. Discard supernatant.
- Resuspend IHL obtained by FNA in desired media for cell counting and proceed with downstream analysis^a^.

**Figure 147.**
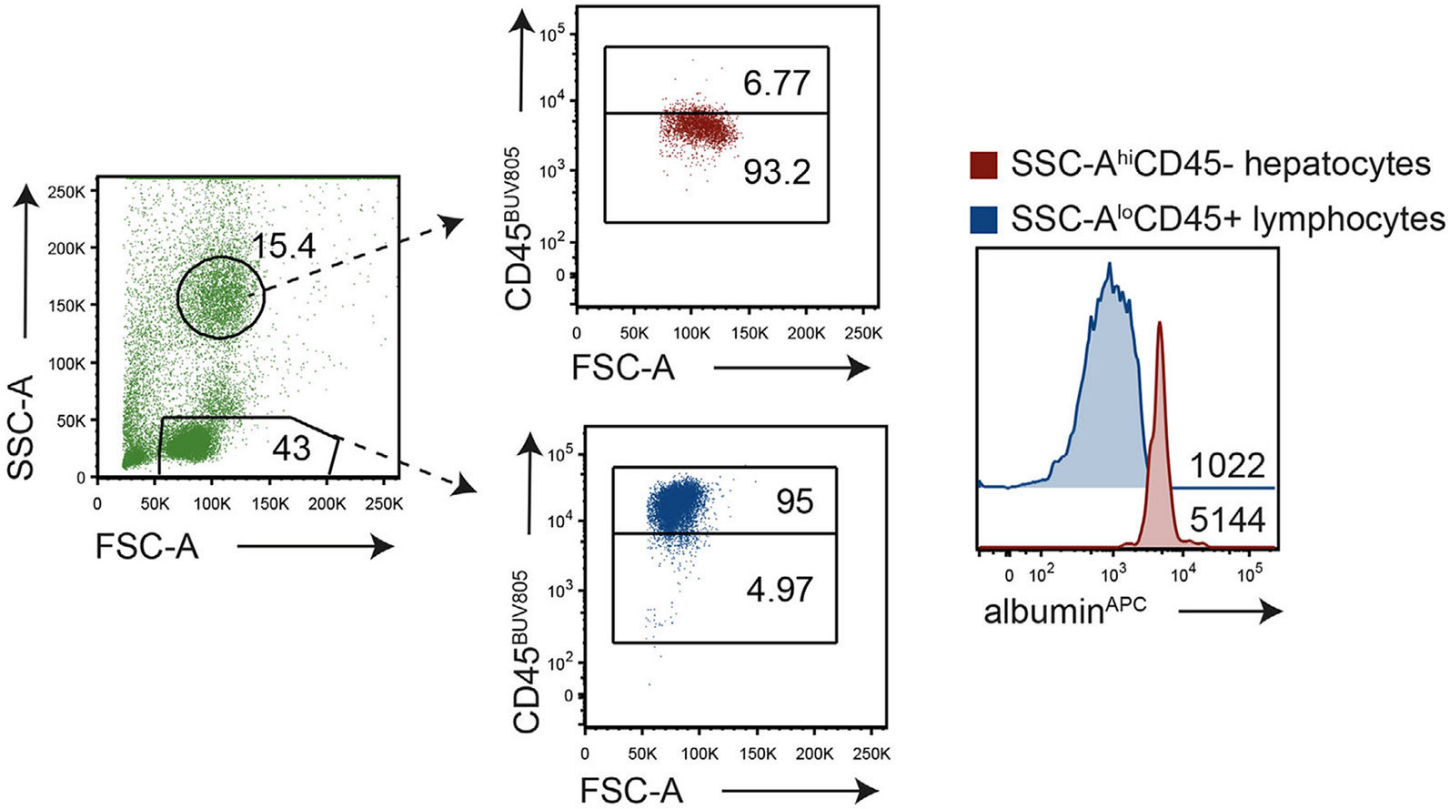
Gating strategy showing the simultaneous analysis of viable, human hepatocytes and leukocytes sampled by FNA. Cells are discriminated by their SSC-A. Parenchymal viable hepatocytes are defined as high SSC-A, CD45 negative and albumin positive compared to intrahepatic leukocytes (encompassing myeloid and lymphoid cells) as low SSC-A, CD45 positive and albumin negative. We recommend the use of albumin-APC (R&D Systems Cat. No. MAB1455, clone 188835).

^a^The lack of mechanical disruption required to process an FNA sample allows for the simultaneous analysis of both viable (as determined using a LIVE/DEAD^™^ Fixable Dead Cell Stain Kit) CD45− albumin^+^ parenchymal cells (hepatocytes) and CD45+ myeloid and lymphoid cell populations by flow cytometry (see Figure 147) [1670].

#### 1.6 Materials:

Isolation of IHL from fine needle aspirates (FNA)

##### Reagents

- RPMI-1640, stored at 4°C
- PBS
- Red blood cell (RBC) Lysis Buffer 10× (BioLegend Cat. No.420301)

##### Equipment

- Centrifuge
- 50 mL falcon tubes
- Serological pipette

#### 1.7 Step-by-step sample preparation: Isolation of IHL from liver biopsy tissue

- Place liver biopsy into a 6 cm^2^ petri dish in 2 mL RPMI-1640.
- Using a pair of forceps to hold one end of the biopsy, gently move it through the liquid to help cells dissociate from the tissue.
  - The media may change colour and become visibly cloudy as IHL exit.
- Using a sterile plastic cell scraper, gently scrape the biopsy tissue to further release IHL and then cut into pieces ≤ 1-2 mm^3^.
- Pass the resulting pieces of liver tissue and RPMI-1640 through a 70 μm cell strainer.
  - Cut the extremity of a 1 mL tip to help with the transfer of tissue pieces if necessary.
- Using the barrel of a 5 mL syringe plunger, excise the remaining IHL from the tissue pieces in the strainer by gentle mechanical disruption.
  - Thoroughly wash the 70 μM cell strainer with RPMI-1640 to minimise IHL loss.
- Top up to 50 mL with RPMI-1640 and centrifuge at 700 × *g* (acceleration/deceleration 9/9) for 15 min at RT. Discard supernatant.
- If necessary, proceed to RBC Lysis.
  - Dilute the 10× RBC Lysis Buffer 1:10 with deionized water (1× solution). Bring the 1× solution to RT before use.
  - Resuspend the IHL pellet in 2 mL 1× RBC Lysis solution.
  - Leave on ice for 5 min.
  - Stop the lysis process by adding 25 mL of cold PBS and centrifuge at 700 × *g* (acceleration/deceleration: 9/9) for 10 min at RT. Discard supernatant.
- Resuspend IHL in desired media for cell counting and proceed with downstream analysis.

#### 1.8 Materials: Isolation of IHL from liver biopsy tissue

##### Reagents

- RPMI-1640, stored at 4°C
- PBS
- Red blood cell (RBC) Lysis Buffer 10× (BioLegend Cat. No.420301)

##### Equipment

- Centrifuge
- 50 mL falcon tubes
- 70 μM filters
- 6 cm^2^ petri dish
- Forceps
- Cell scraper
- 5 mL syringe

#### 1.9 Step-by-step sample preparation: Assessment of IHL

NB:// for use on IHL isolated from either perfusates (as described in section 1.3), fine needle aspirates (as described in section 1.5) or liver biopsy tissue (as described in section 1.7).

- Transfer the cells into a 96-well plate (u- or v-bottom).
  - Pre-cool the centrifuge prior to staining.
- Centrifuge IHL for 5 min at 500 × *g* at 4°C. Discard supernatant.
- For the detection of dead cells, prepare a LIVE/DEAD^™^ Fixable Dead Cell Stain solution in 1× PBS. Add 50 μL of LIVE/DEAD^™^ stain solution/well and re-suspend the cells.
  - To prepare LIVE/DEAD^™^ stain thaw all components. Add 50 μL Component B to 1 vial of Component A. This is your LIVE/DEAD^™^ solution. Vortex.
  - Note: use this as soon as possible, so make smaller aliquots to re-freeze to avoid freeze- thaw cycles.
  - We recommend using a dilution of 1 μL LIVE/DEAD^™^ solution in 1 mL 1× PBS
- Incubate for either 15 min at RT for 30 min at 4°C in the dark.
- Top up with 150 μL 1× PBS to wash and centrifuge for 4 min at 500 × *g* at 4°C. Discard supernatant.
- If required to block non-specific Ab binding: incubate with 50 μL FcR blocking solution/well for 10 min at 4°C prior to Ab staining.
- For the detection of surface molecules, prepare an Ab master mix^*b*^ in 1× PBS or Brilliant Stain Buffer and re-suspend the cells in 50 μL master mix/well.
  - The use of a ‘Brilliant Stain Buffer’ is required if using multiple brilliant fluorescent polymer dyes to avoid artifacts generated by the interactions between dyes. If only one Brilliant dye is used in an experiment, the Brilliant Stain Buffer is not needed.
- Incubate for 30 min at 4°C in the dark.
- Top up with 150 μL 1× PBS to wash, and centrifuge for 4 min at 500 × *g* at 4°C. Discard supernatant.
  - If no intracellular staining is required, fix the IHL with your preferred fixative. We recommend incubation with 100 μL/well BD CytofixTM for 20 min at 4°C.
  - Top up with 100 μL 1× PBS to wash, and centrifuge at 500 × *g* at 4°C. Discard supernatant and proceed to flow cytometric assessment, transferring fully stained IHL into FACS tubes^*d,e*^.
  - Note: cell aggregation can be avoided by vortexing prior to the addition of the fixative.
- For the detection of *intracellular* molecules (e.g., cytotoxic mediators, cytokines or chemokinesc) we recommend the use of BD Cytofix/Cytoperm^™^.
  - To fix and permeabilize the cells, add 100 μL Cytofix/CytopermT^™^/well, resuspend the cells and incubate for 30 min at 4°C in the dark.
  - Note: cell aggregation can be avoided by vortexing prior to the addition of the fixative.
  - Prepare an Ab master mix^*b*^ in saponin-buffer and re-suspend the cells in 50 μL master mix/well.
  - Incubate for 30 min at 4°C in the dark.
  - Top up with 150 μL 1× PBS to wash, and centrifuge for 4 min at 500 × *g* at 4°C. Discard supernatant and proceed to flow cytometric assessment, transferring fully stained IHL into FACS tubes^d,e^.
- For the detection of *intranuclear* molecules (e.g., transcription factors) we recommend the use of BD Bioscience FOXP3 Buffer Kit^™^.
  - To fix the cells, dilute FOXP3 Buffer A (10× concentrate) 1:10 with deionized water (RT) and immediately add 100 μL solution/well, resuspend the cells, and incubate for 10 min at RT in the dark.
  - Tip: make twice the volume of 1× FOXP3 Buffer A required to use during permeabilization to dilute FOXP3 buffer B in.
  - Note: cell aggregation can be avoided by vortexing prior to the addition of the fixative.
  - Top up with 100 μL 1× PBS to wash, and centrifuge for 4 min at 500 × *g* at 4°C. Discard supernatant.
  - To permeabilize the cells, dilute FOXP3 Buffer B (50X) 1:50 with 1× FOXP3 Buffer A (prepared during previous steps) and add 100 μL solution/well, resuspend cells, and incubate for 30 min at RT in the dark.
  - Top up with 100 μL 1× PBS to wash, and centrifuge for 4 min at 500 × *g* at 4°C. Discard supernatant.
  - Prepare an Ab master mixb in 1× PBS and re-suspend the cells in 50 μL master mix/well. Incubation in FOXP3 Buffer is not necessary since the permeabilization step is so strong that the pores do not reclose.
  - Incubate for 30 min at 4°C in the dark.
  - Top up with 150 μL 1× PBS to wash, and centrifuge for 4 min at 500 × *g* at 4°C. Discard supernatant and proceed to flow cytometric assessment, transferring fully stained IHL into FACS tubes^d,e^.

^a^ In our experience, LIVE/DEAD^™^ Fixable Dead Cell Stain can be added directly to the Ab master mix and stained simultaneously. However, we note better separation of live and dead cells on the analyzer if the LIVE/DEAD^™^ Fixable Dead Cell Stain is stained first in 1× PBS, separate to the remaining surface molecule Abs.

^b^ The use of Ab master mixes is recommended, these can be prepared fresh before use or prepared in advance and stored at 4°C in the dark until use. Preparation beforehand should be tested and validated against freshly prepared master mixes for each panel. The volume of the Ab master mix will be dependent on panel design/experimental conditions.

^c^ For the assessment of cellular functionality and the detection of intracellular molecules such as cytokines and chemokines an appropriate stimulus will often be required. Further to this, the use of a protein transport inhibitors containing Monensin and/or Brefeldin A (e.g., BD Bioscience GolgiStop^™^ or GolgiPlug^™^) will be required.

^d^ Keep fully stained cells at 4°C in the dark until flow cytometric analysis, which should be carried out as soon as possible to prevent degradation of fluorochrome conjugates.

^e^ When acquiring cells on the analyzer we recommend the transfer of fully stained, single cell suspensions to 1.2 mL ‘small, insert’ tubes (e.g., Corning 96-well Cluster Tubes, Cat. No. 4401) that can then be placed inside a FACS tube. This will reduce the ‘dead’ volume which is particularly important if cell number may be limiting.

#### 1.10 Materials: Assessment of IHL

##### Reagents

**Figure 148.**
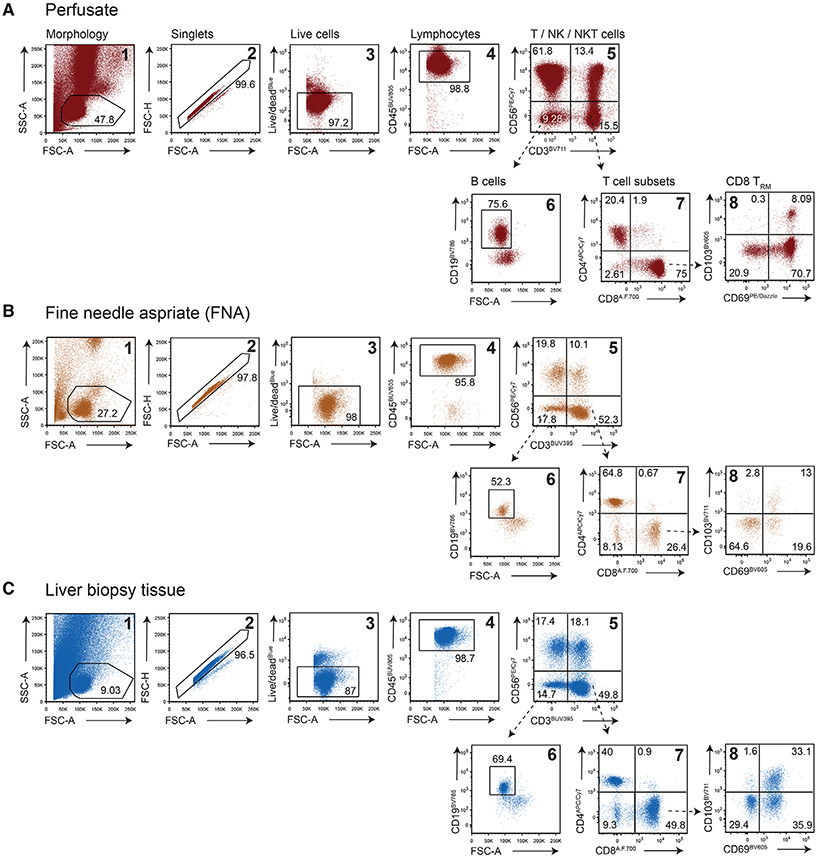
Gating strategy showing the simultaneous analysis of innate and adaptive IHL sampled by perfusate, FNA and from biopsy tissue samples. Representative example showing a sequential gating strategy used for the identification of major subsets of IHL isolated from a) perfusates, b) fine needle aspirates (FNA) and **c)** liver biopsy tissue. Lymphocytes are discriminated by plotting FSC-A against SSC-A (Gate 1). Single cells are further identified using FSC-A against FSC-H (Gate 2). Live cells are defined as LIVE/DEAD^™^ Fixable Dead Cell Stain negative (Gate 3) and CD45 expression is used to identify pan-lymphocytes (Gate 4). CD3+CD56- T cells, CD3+CD56+ NKT cells and CD3-CD56+ NK cells are defined using Gate 5, with the CD3-CD56- cells being further gated to identify CD19^+^ B cells (Gate 6). From the CD3+CD56- T cell gate sequential gating allows for the identification of CD4+ and CD8+ T cells respectively (Gate 7) and Gate 8 shows the discrimination of liver infiltrating, non-resident (CD69-CD103-) from liver resident tissue memory CD8 T cells (Trm; CD69^+^CD103^+^).

- Brilliant Stain Buffer (BD Biosciences Cat. No. 563794)
- 1× PBS
- LIVE/DEAD^™^ Fixable Dead Cell Stain Kit (Life Technologies)
- BD Bioscience Cytofix^™^ (Cat. No. 554655), Cytofix/Cytoperm^™^ (Cat. No. 554714) or FOXP3 Buffer Kit^™^ (Cat. No. 560098) (or similar)
- Saponin buffer (0.1% saponin, 1% FBS in 1× PBS)
- Conjugated fluorochrome-labeled Abs (see examples in table 150 and Figure 148)

##### Equipment

- 96-well plate (u- or v-bottom)
- FACS tubes
- Vortex
- Corning cluster tubes
- Centrifuge
- Flow cytometer (e.g., BD FortessaX20 with the following configuration)
  - Lasers: violet (405 nm), blue (488 nm), yellow-green (561 nm), red (640 nm), UV (355 nM)
  - Optical filters: 450/50 BP, 525/50 BP, 610/20 BP, 710/50 BP, 780/60 BP; 488/10 BP, 530/30 BP, 695/40 BP; 586/15 BP, 610/20 BP, 670/30 BP, 780/60 BP; 670/30 BP, 730/45 BP, 780/60 BP; 379/28 BP, 450/50 BP, 740/35 BP, and 820/60 BP.

#### 1.11 Step-by-step sample preparation: Simultaneous isolation of hepatic stellate cells (HeSC) and Kupffer cells (KC)

This step-by-step guide describes the isolation of HeSC and KC from larger volume liver tissue samples obtained from surgical resection or explant.

- Wash the liver tissue sample obtained from surgery in 1× PBS to remove as much contaminating blood as possible.
- Once washed, dissect available liver tissue into smaller-sized sections approx. 10-15 g in weight and transfer, using forceps, into 100 mL multipurpose individual plastic beakers.
  - The number of individual plastic beakers used will depend on the initial mass of tissue.
- Cut the liver tissue sections into small pieces (≤ 1-2 mm^3^) using forceps and scissors in 10 mL pre-warmed enzyme digestion buffer (see Section 1.12 Materials below).
  - Enzyme digestion buffer is prepared using HBSS^+/+^ to support enzyme activity. Pre-warm enzyme digestion buffer to 37°C to assure optimal dissociation during incubation.
- Add a further 10–15 mL enzyme digestion buffer (ensuring all chopped tissue is well covered) to each pot and incubate for 30 min at 37°C.
  - Throughout the incubation period occasionally swirl each pot for a few seconds to aid the digestion process.
- If available proceed to a mechanical disruption step using a Miltenyi Biotech GentleMACS^™^ tissue dissociator. If such machine is not available proceed to pass the resulting liver homogenate through 70 μM cell strainers in step below.
  - Decant the contents of each individual plastic beaker into two to three 10 mL GentleMACS^™^ C-tubes (Miltenyi Biotech, ensuring not to overfill these tubes).
  - Place C-tubes onto a GentleMACS^™^ tissue dissociator and run for one cycle using the mouse spleen program (m_spleen_01.01).
  - Note: GentleMACS^™^ tubes can be reused multiple times once emptied through a strainer.
- Pass the resulting liver tissue homogenate through a 70 μM cell strainer into a new 50 mL falcon tube. Using the barrel of a 5 mL syringe plunger, excise remaining cells from the tissue pieces in the strainer by mechanical disruption.
  - Thoroughly wash the 70 μM cell strainer with HBSS^−/−^ to minimise cell loss.
  - Add an equal volume of HBSS^−/−^ to aid with the washing of cells.
  - Change the 70 μM cell strainer as required to prevent damage or blocking the filter and discard any connective or tough fibrotic tissue.
- Centrifuge for 5 min at 50 × *g* at room temperature (RT). Transfer supernatant with a 25 mL serological pipette into new 50 mL falcon tubes, discarding the pellet (hepatocytes).
  - Caution: the cell pellet may be loose, therefore there is a risk of aspirating it.
- Repeat the previous step - centrifuge for a further 5 mins at 50 × *g* at RT. Transfer supernatant with a 25 mL serological pipette into new 50 mL falcon tubes, discarding any remaining hepatocytes.
- Centrifuge transferred supernatants at 450 × *g* at RT for 10 mins. Discard supernatant with a 25 mL serological pipette.
- Resuspend the pellet (non-parenchymal cells) in 10 mL of wash buffer (see Section 1.12 Materials below).
  - At this point pellets can be combined. From a starting 40-50 g of liver, pellets can be combined into two tubes. Less than 30 g should be combined into one tube and more than 60 g should be combined into >3-4 tubes.
- Centrifuge at 450 × *g* at RT for 10 min. Discard supernatant with a 25 mL serological pipette.
- Disrupt the cell pellet by vortexing for 30 seconds, then add 8.4 mL of Optiprep Solution A (see Section 1.12 Materials below).
  - To obtain clearer HeSC layers after centrifugation, combine pellets from three 50 mL falcon tubes in 8.4 mL Optiprep Solution A.
- Top up to 20 mL with wash buffer and mix gently but thoroughly.
  - This will give a final Optiprep concentration of 17%.
- Carefully layer an equal volume (20 mL) of Optiprep Solution B over the 20 mL cell suspension in Optiprep Solution A.
  - This layer will have a concentration of 11.5%
  - Lower the speed of dispensing on the pipette gun to assure a slow drip of Optiprep Solution B to obtain two clear and distinct layers.
- Further, very carefully layer an additional 8 mL of wash buffer over Optiprep Solution B and centrifuge at 1400 × *g* for 20 min at RT with the centrifuge break off^a^.
- The hepatic stellate cells (HeSC) will move to the interface between Optiprep Solution B and the wash buffer (upper layer) and the Kupffer cells (KC) will be at the interface of Optiprep Solutions A and B (lower layer). Aspirate each layer using a sterile Pasteur pipette into separate 50 mL falcon tubes.
- To wash the isolated HeSC and KC, add an equal volume of HBSS^−/−^ and centrifuge at 700 × *g* (acceleration/deceleration: 9/9) for 15 min at RT. Discard supernatant.
- Resuspend the pellet in HBSS^−/−^ and top-up to 40 mL HBSS^−/−^ and centrifuge at 700 × *g* (acceleration/deceleration: 9/9) for 15 min at RT. Discard supernatant.
- Resuspend HeSC and KC in desired media for cell counting.

^a^ Use minimum acceleration and the no brake setting on the centrifuge to ensure successful density centrifugation.

##### 1.11.1 For HeSC.

- Isolated HeSC can either be used for downstream analysis *ex vivo* or expanded *in vitro*.
- For expansion, we recommend seeding cells at an approx. density of ~5 × 10^4^ cells/cm^2^ (if using a T75 ~3.75-4 × 10^6^ cells or if using a T25, ~1.0-1.5 × 10^6^ cells) in an appropriate volume of stellate cell media (Sciencell Cat. No. SC-5301)
- Incubate HeSC for ~16 h to allow cells to attach.
- After initial incubation wash HeSC twice with 1× PBS, before replacing with an appropriate volume of stellate cell media.

##### 1.11.2 For Kupffer cells.

- Dilute the 10× RBC Lysis Buffer 1:10 with deionized water (1× solution). Bring the 1× solution to RT before use.
- Resuspend the KC cell pellet in 2 mL 1× RBC Lysis solution.
- Leave on ice for 5 min.
- Stop the lysis process by adding 25 mL of cold 1× PBS and centrifuge at 700 × *g* (acceleration/deceleration: 9/9) for 10 min at RT. Discard supernatant.
- Reconstitute in KC maintenance media (see Section 1.12 Materials below).
- Isolated KC can either be used for downstream analysis *ex vivo* or maintained *in vitro*.
- Seed KC in at 1 × 10^6^ cells/well in a 6- or 24-well plate.
- Incubate KC at 37°C for 1-2 h to allow adherence.
- Wash twice with 1× PBS, before adding fresh KC maintenance media.

#### 1.12 Materials: Simultaneous isolation of hepatic stellate cells (HeSC) and Kupffer cells (KC)

##### Reagents

- Enzyme digestion buffer (HBSS^+/+^ with 0.002% (w/v) DNase I and 0.02% (w/v) Collagenase IV )
- Wash buffer (HBSS^−/−^ with 0.001% (w/v) DNaseI and 0.25% (w/v) BSA )
- Optiprep Solution A (Optiprep is a 60% (w/v) Iodixanol solution. Dilute 2 parts Optiprep with 1 part wash buffer to create a 40% Iodixanol solution. Once combined with the cell pellet and topped up with wash buffer the final Iodixanol is 17% which is the manufacturer’s recommended concentration for isolation of HeSC)
- Optiprep Solution B (Dilute Optiprep solution A in HBSS^−/−^ to a final Iodixanol concentration of 11.5%. To obtain this concentration, combine 2.9 parts Optiprep solution A with 7.1 parts HBSS^−/−^)
- HBSS^−/−^
- HBSS^+/+^ (with calcium and magnesium)
- 1× PBS
- Red blood cell (RBC) Lysis Buffer 10× (BioLegend Cat. No.420301)
- Macrophage maintenance media (RPMI 1640 + L-Glutamine, p/s, 10% FBS or autologous heat-inactivated serum)
- Stellate cell media (ScienCell Cat. No SC-5301)

##### Equipment

- 100 mL multipurpose plastic beakers
- Scissors
- Forceps
- Centrifuge
- 50 mL falcon tubes
- Miltenyi Biotech GentleMACS^™^ C tubes
- Miltenyi Biotech GentleMACS^™^ tissue dissociator
- Serological pipette
- Pasteur pipettes
- 70 μM cell strainers
- 5 mL syringes
- Tissue culture plates / flasks

#### 1.13 Data analysis

This is detailed in Figures 147 and 148.

#### 1.14 Pitfalls

Hepatocytes obtained from the HeSC and KC isolation protocol are not viable due to the level of mechanical disruption required. Although not provided here a distinct protocol would be required to isolate primary hepatocytes from human liver tissue. Alternatively, to simultaneously profile hepatocytes and intrahepatic lymphocytes, fine needle aspirates could be used (see Section 1.5).

HSCs undergo a characteristic phenotypic change *in vitro* once isolated, and differentiate from vitamin A–storing pericytes into ECM-producing myofibroblasts in culture. Due to endogenous retinoid expression, HeSC are autofluorescent in the commonly used detection channel for DAPI (e.g., 405-407nm laser for excitation and the 450/50nm BP filter for detection). It is important to be aware of this when designing FACS-based staining panels. We recommend the use of α-smooth muscle actin as a marker for the identification of HeSC.

#### 1.15 Top tricks

##### Isolation of IHL:

Depending on IHL yield obtained and/or the experimental requirements, IHL from perfusates and fine-needle aspirates can be cryopreserved. Cryopreserved IHL have been used successfully for phenotypic and functional profiling by flow cytometry once thawed. We advise using a method of depleting dead cells (e.g., EasySep^™^ Dead Cell Removal (Annexin V) kit), and resting the cells in your preferred media overnight before functional assessment. However, we do not recommend the freezing of IHL obtained from tissue biospsy due to the low cell yield and mechanical disruption leading to contamination with cellular debris from dying/dead parenchymal cells.

##### To freeze IHL:

- Resuspend IHL in freezing media (10% DMSO: heat-inactivated FBS) at a final concentration of 10 × 10^6^ IHL/mL.
- Decant 500 μl IHL suspension into labeled, internal-threaded cryovials.
- Place cryovials into a Nalgene^®^ Mr. Frosty container (or similar) and place container immediately into a −80°C freezer for 24 hours before transferring to long-term liquid nitrogen storage.

##### To thaw IHL:

- Pre-warm HBSS^+/+^.
- Quickly thaw vial of IHL in warm water bath (37°C). Transfer IHL from cryovial into 20 mL pre-warmed HBSS^+/+^ (with calcium and magnesium) containing 0.001% DNase-I (1% stock: 0.1 g in 10 mL HBSS^−/−^; Roche Cat. No. 11 284 932 001
- Centrifuge at 700 × *g* (acceleration/deceleration: 9/9) for 15 min at RT. Discard supernatant.
- Resuspend IHL in desired media for cell counting and proceed with downstream analysis.

To ensure an IHL population reflective of the intrahepatic environment is obtained from FNA samples we recommend that heavily blood-stained FNA samples be discarded prior to use. For more details on how to obtain FNA samples from patients please refer to published work [1670-1672].

##### Isolation of HeSC and KC:

To aid with the successful isolation of HeSC and KC, we recommend a starting tissue mass of at least ~30g. In our experience HeSC will continue to grow/expand for a number of weeks, with regular passaging/media changes, ensuring HeSC are never more than 80% confluent. As with IHL obtained from perfusates and FNA, depending on cell yield obtained after *in vitro* expansion and/or the experimental requirements, HeSC can be cryopreserved. Cryopreserved HeSC have been used successfully for phenotypic and functional profiling by flow cytometry once thawed. We advise thawing HeSC and expanding for a short period of time in stellate cell media before use.

##### To freeze HeSC:

- Resuspend HeSC in freezing media (10% DMSO: heat inactivated FBS) at a final concentration of 2 × 10^6^ HeSC/mL.
- Decant 500 μL HeSC suspension into labeled, internalthreaded cryovials.
- Place cryovials into a Nalgene^®^ Mr. Frosty container (or similar) and place container immediately into a −80°C freezer for 24 hours before transferring to long-term liquid nitrogen storage.

Likewise in our experience KC can be maintained for an extended period of time in culture. This requires a media change providing fresh KC maintenance media every 2-3 days. KC can be maintained for up to 2 weeks. To improve KC purity, we recommend that on the day post-isolation, three 1× PBS washes of all wells. A top tip - to use KC in suspension (e.g., for flow cytometric analysis or co-cultures) use non-tissue culture treated plates and Cell Stripper (Corning, Cat. No. 25-056-CI) as per the manufacturer’s instructions.

##### 1.16 Clinical relevance statement

The gating strategy shown in the section describing cell isolation by fine-needle aspiration is applicable for the comprehensive, and simultaneous analysis of intrahepatic lymphoid/myeloid cells, including sentinel populations of liver-resident T cells and NK cells, and hepatocytes. Importantly, due to the lack of mechanical disruption required, the combination of an increased side scatter area gate and inclusion of CD45 in the Ab cocktail make it possible to distinguish and characterize viable human hepatocytes. This has not previously been possible with standard tissue processing protocols. Our recent study demonstrated expression of PD-L1 on hepatocytes sampled by FNA [1670] raising the possibility that FNA samples could be used to monitor expression of checkpoint receptors and their ligands in the setting of checkpoint inhibitor trials. The gating strategy shown in Figure 148 is applicable for the analysis of multiple subsets of liver infiltrating and tissue resident B cells, NK cells, and T cells in both health and disease. For example, the gating strategy has previously been used to define the contribution of IL-2-producing tissue resident CD8^+^ T cells [1673], and CXCR6-expressing NK cells [1674] in chronic HBV infection.

#### 1.17 Summary of the phenotypes

This is detailed in Table 154 (see below).

### 2 Murine liver cells

#### 2.1 Overview

This chapter provides detailed protocols for isolation of mouse liver-resident cell populations including Kupffer cells, liver sinusoidal endothelial cells (LSEC), hepatocytes, and hepatic stellate cells as well as intrahepatic leukocytes and their subsequent staining for flow cytometry.

#### 2.2 Introduction

See section XVI.1.2 Introduction in Human liver cell phenotypes

#### 2.3 Step-by-step sample preparation: Liver non-parenchymal cell (LNPC) isolation

**Figure 149.**
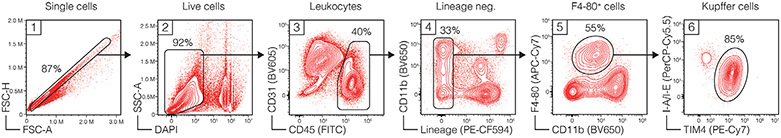
Gating strategy for Kupffer cell identification in LNPC samples isolated from C57BL/6 mice (9 weeks-old). Single cells were discriminated by plotting FSC-A against FSC-H (gate 1). Live cells were defined as DAPI negative (gate 2), while hepatic leukocytes are CD45^+^(gate 3). Note that in this gate liver sinusoidal endothelial cells (LSEC) can be identified as CD45^−^ CD31^+^. Gate 4 is then used to get rid of lineage^+^cells (CD3^+^, CD19^+^, CD49^+^, Ly6G^+^). Note that the concomitant use of CD11b allows the identification of neutrophils as Lineage^+^CD11b^high^, while T and B cells are Lineage^+^CD11b^−^. F4-80^+^macrophages are then defined as CD11b^int^ F4-80^+^(gate 5). Finally, plotting I-A/I-E against TIM4 (gate 6) allows the discrimination between Kupffer cells (TIM4^+^, I-A/I^−^ Eint) and capsular macrophages (TIM4^−^, I-A/I-E^+^).

**Figure 150.**
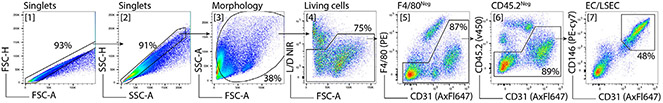
Gating strategy for LSEC identification in liver samples isolated from C57BL/6 mice (9 weeks-old). Single cells were discriminated by plotting FSC-A vs FSC-H and SSC-A vs SSC-H (gate 1 and 2, respectively). Then, the population of interest were gated based on its morphology (gate 3). Living cells were defined as Live/Dead NIR negative (gate 4). Kupffer cells and macrophage-related population were excluded using F4/80 (gate 5). Other leukocytes (e.g., lymphocytes, NKT cells, ILCs, and neutrophils) were excluded using CD45.2 (gate 6). LSEC are finally identified as CD31^+^CD146^+^(gate 7).

**Figure 151.**
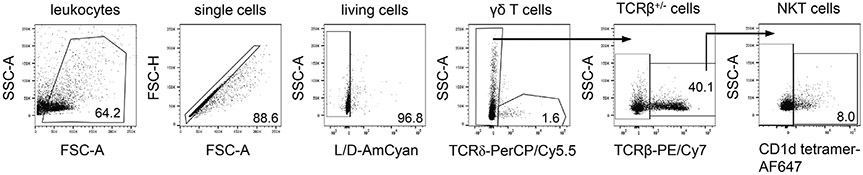
Gating strategy for NKT cells and γδ T cells in murine liver. Hepatic leukocytes from *Mdr2*−/− mice, which develop chronic liver inflammation, were used for analysis. Single cells were discriminated from doublets by plotting FSC-A against FSC-H. To exclude dead cells, a fixable dead cell staining was performed. Hepatic leukocytes were stained with anti-TCRβ-PE/Cy7 (H57-597; BioLegend), anti-TCRδ-PerCP/Cy5.5 (GL3; BioLegend) and anti-CD1d tetramer-AF647 (NIH Tetramer Core Facility) Abs to distinguish between TCRαβ- TCRγδ^+^cells and TCRαβ^+^CD1d tetramer^+^NKT cells.

**Figure 152.**
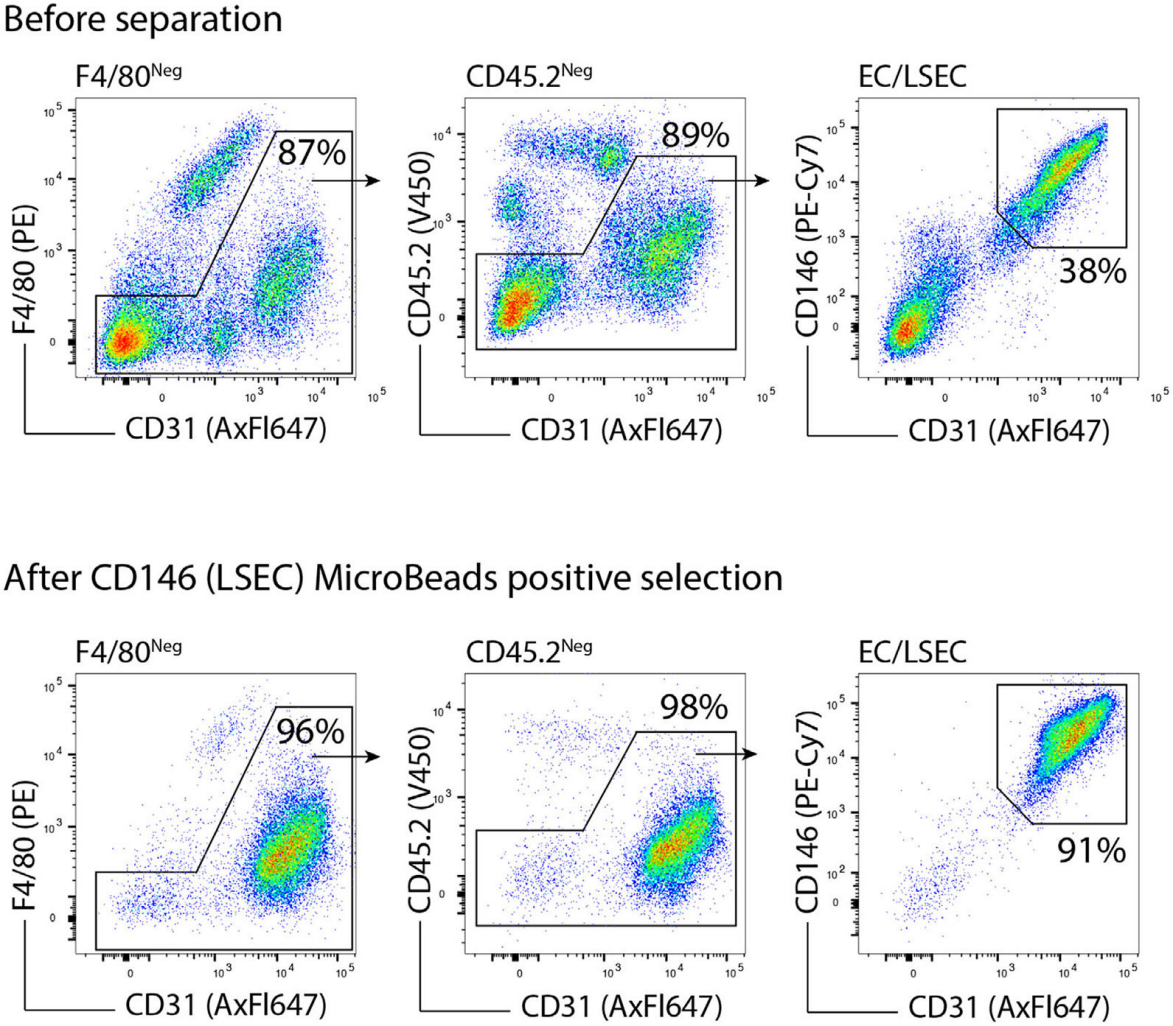
Comparison of LSEC frequency from the same WT mouse using CD146 MicroBeads positive selection. Gating strategy for single and living cells is the same as shown in Figure 150. The LSEC fraction is about 30-40% of total living cells before purification. After CD146 MicroBeads positive selection, the LSEC population reaches about 90- 95% of total living cells. Note in the dot plots that the presence of non-LSEC is minimal after purification.

This protocol has been optimized for the rapid (~2 hours) isolation of liver non-parenchymal cells (particularly Kupffer cells but also intrahepatic leukocytes) suitable for phenotypic and functional analyses by multicolor flow cytometry. It relies on the *ex-vivo* collagenase-based digestion of the liver followed by its mechanical disruption through a 18G needle and can be used to efficiently process several samples in a reasonable amount of time. A detailed gating strategy for Kupffer cells is provided in Figure 149, for LSECs in Figure 150 and for γδ T cells and NKT cells in Figure 151. This procedure is not optimal for functional downstream applications of LSECs, for that purpose we invite the readers to refer to the specific LSEC protocol and Figure 152.

- Euthanize the mouse by cervical dislocation and spread the abdomen with 70% ethanol.
- Perform laparotomy and expose the liver by gently moving the intestine sideward.
- Perfuse the liver with PBS by the vena cava.
  - When the cardiovascular system becomes pressurized, cut the portal vein and continue until about 10ml are perfused.
- Remove the gallbladder and cut the liver ligaments.
- Collect the liver in a 50 ml Falcon tube containing 10 ml of ice-cold plain RPMI.
- Transfer the liver to a 35 mm culture dish and cut it in the smallest pieces possible using scissors.
- Transfer the liver pieces in a 50 ml Falcon tube containing 20 ml of pre-warmed collagenase solution (RPMI supplemented 0.2 mg/ml of collagenase and 5 units/ml of DNAse I) and pipette upside down few times with a serological 10 ml pipette to make sure that the pieces are small enough.
  - If only half liver has to be processed, adjust the volume of collagenase solution accordingly (i.e., 10 ml).
- After 30 min of incubation at 37°C, gently homogenize the digested liver pieces by forcing them seven to eight times into a 10 ml syringe provided with a 18G needle.
- Filter the liver homogenate in a new 50 ml Falcon tube using a 70 μm cell strainer to remove undigested tissue.
- Rinse the filter and the syringe with 20 ml of plain RPMI and centrifuge at 400 × *g* for 5 min at RT.
- Discard the top aqueous phase and lyse red blood cells with 2 ml of ACK solution at room temperature for 30 seconds.
  - Pay attention that at this step the cell sediment is loose and might be the risk of aspirating it.
  - Cut the extremity of a 1 ml tip in order to help the resuspension process.
  - It’s important to perform red blood cell lysis at RT since lower temperatures (i.e., 4°C) increase the rigidity of the cellular membrane thus impairing the hypo-osmotic burst of erythrocytes.
- Stop the lysis process by adding 20 ml of plain RPMI and centrifuge at 400 × *g* for 5 min at 4°C.
  - The RPMI volume needed to stop the lysis should be 10 times the ACK volume. It is possible to adjust the volume accordingly if more ACK is required to lyse red blood cells (i.e., if the liver perfusion was sub-optimal).
  - From now all reagents and procedures are done at 4°C.
- Discard the top aqueous phase and resuspend the cell sediment with 10 ml of plain RPMI.
- Filter cell suspension through a 70 μm cell strainer.
- Determine cell counts and proceed with the desired down-stream application.
  - Usually, one uninflamed healthy liver yield about 2-4 × 107 LNPCs.

#### 2.4 Materials: Liver non-parenchymal cell (LNPC) isolation

##### Reagents

- RPMI
- DNAse I (Merck) 0.5 mg/vial, 3900 units/mg: dissolve DNAse in 1 ml of plain RPMI and store −20°C.
- Collagenase type IV (Sigma).
- ACK lysis buffer: 150 mM NH4Cl, 10 mM KHCO3, 0.1 mM Na2EDTA, pH 7.2 in dH20.

##### Equipment

- 70% Ethanol (v/v)
- PBS
- Surgical forceps
- Surgical scissors
- Cell Strainer, 70 μm
- 35 mm Petri dishes
- 50 ml Falcon tubes
- 10 ml syringes
- 18G needles
- Centrifuge

#### 2.5 Step-by-step sample preparation: Murine Kupffer cell *staining*

- Transfer 1-2 × 10^6^ cells in FACS tubes according to the experimental conditions.
- Wash cells once with FACS buffer.
- Incubate with 100 μl of Fc-block solution (αCD16/CD32, 5 μg/ml in FACS buffer) for 15 min at 4°C.
- Wash cells and incubate with 100 μl of Abs mix (prepare it in Brilliant stain buffer) for 40 min at 4°C.
  - The use of Brilliant stain buffer helps to avoid artifacts generated by the interactions of brilliant fluorescent polymer dyes.
- At the end of incubation add directly to the samples 100 μl of DAPI solution (5 μg/ml in FACS buffer) to the cells and incubate further 5 min at 4°C.
  - The use of DAPI, which is – in contrast to Hoechst – impermeable to healthy live cells, is more effective in the discrimination of dead cells for LNPCs preparation if compared to amine-reactive fixable dyes.
- Wash cells twice with FACS buffer and resuspend in 300 μl of FACS buffer.

#### 2.6 Materials: Murine Kupffer cell staining

##### Reagents

- FACS buffer: 1% FBS, 2 mM Na2EDTA, 0.05% NaN3 in PBS
- Brilliant stain buffer (BD)
- DAPI (Thermo)
- Fluorophore-coupled Abs (see Table 151)

##### Equipment

- FACS tubes
- Centrifuge
- Flow cytometer with the following configuration
  - Lasers: violet (405 nm), blue (488 nm), yellow-green (561 nm), red (640 nm)
  - Optical filters: 450/45, 525/40, 610/20, 660/10, 690/50, 763/43

#### 2.7 Step-by-step sample preparation: Hepatocyte *(HC)* and LSEC isolation

In this section, we present an improved protocol based on two-step *in-situ* liver perfusion technique. This kind of perfusion offer two advantages: first, liver perfusion medium cleans the liver of blood, preventing clotting, and initiates the loosening of cell–to–cell contact; second, liver digest medium supplemented with Liberase TM, efficiently dissociates liver into viable liver cells. This protocol was also optimized to significantly reduce procedure duration (although it requires more time than the protocol described in the previous section), reduce technical challenge, improve reproducibility and improve LSEC and/or HC yield and viability. Some critical steps we incorporate here: (1) non-parenchymal cells are enriched by density-based precipitation, since only HCs are pelleted while other cells are left in the supernatant. At this step, HC-specific or LSEC-specific protocols have to be followed depending on the cellular population of interest. (2) Perfusion via vena cava permits easier cannulation in comparison with portal vein cannulation. (3) Liberase TM-containing liver digestion medium allows for quicker digestion and better reproducibility compared to other collagenases. This optimized protocol for LSEC isolation finally allows to obtain high-quality LSEC cellular suspensions suitable for fluorescence-activated cell sorting, functional assay, single-cells analysis, and more.

- Place the mice into the CO_2_/O_2_ vaporizer and anesthetize by gently flowing the chamber.
- Once the mice are anesthetized, euthanize the animal by cervical dislocation and confirm death.
- Place the mouse with the ventral side upwards and fix the animal.
- Spread the abdomen with 70% EtOH and open the abdomen by making an incision in the cranial direction.
- Flap the skin to the side and fix with a needle.
- Push the intestine sideward by using the blunt end of a forceps to get access to the liver.
- Pump preparation:
  - Warm water bath to 42 °C and place the perfusion buffers in the water bath. Please make sure the temperature is maintained throughout the entire process.
  - Run first 70% ethanol and then air through the tubing for 30–60 s each one.
  - Connect 27-gauge needle to the outlet end of the tubing. Purge some perfusion liver medium to wash residual ethanol.
- Proceed with the *in-situ* liver perfusion via the vena cava. Note that retrograde perfusion through the vena cava permits easier cannulation as opposed to portal vein cannulation, but excellent results are also obtained through the latter one.
  - Perfuse the liver by gently injecting Liver Perfusion Medium (about 5 mL) for 1 min using peristaltic pump following a 4 min/mL flow rate.
  - Perfuse the liver by gently injecting pre-warmed 37 °C Liver Digest Medium (about 10 mL) supplemented with 40 μg/mL Liberase for 8 min using peristaltic pump following a 2.7 min/mL flow rate.
  - The liver becomes blanched and swollen.
- Remove the liver and transfer it into 50 ml centrifugation tube with 20 mL of RPMI complete medium. Store on ice until next step.
- Transfer the liver into a petri dish with 20 ml of 37°C RPMI.
- Decapsule the liver and, while holding it with a pincette, shake the tissue in the medium until parenchymal and non-parenchymal cells are washed out.
- Smash the liver gently using a pestle in a petri dish. Filter through a 70 μm cell strainer to remove cell aggregates and create a uniform single-cell suspension.
- Homogenize the liver by rubbing over the scribed surface using the pistil of a 2.5 ml syringe.
- Fill 10 ml of RPMI three times into the petri dish and transfer the homogenate into a fresh 50 ml centrifugation tube.
- Centrifuge for 3 min at 50 × *g*, 4°C.
  - At the end of this centrifugation, the supernatant is enriched with LSECs and intrahepatic leukocytes, while the cell sediment is constituted mainly by hepatocytes. Depending on the target population, continue with the present protocol or proceed with the HC-specific protocol described below.
  - Remove LSEC-enriched supernatant and resuspend the HC-enriched pellet with the Percoll solution. Percoll here is used to separate the viable hepatocytes from dead hepatocytes and cell debris.
  - Prepare Percoll solution by mixing 21.6 ml Percoll + 2.4 ml 10× DPBS (+ MgCl2 and CaCl2).
  - Centrifuge HCs/Percoll solution for 10 min at 72 × *g*, 5°C
  - Remove supernatant and add 30 ml Williams E medium, centrifuge HCs for 5 min at 72 × *g*, 5°C
  - Remove supernatant and re-suspend HCs in 15 ml Williams E medium.
  - For culture, incubate HCs at 37°C, 40% O2, 5% CO2
  - Carefully change medium after 4 h incubation time; HCs are still adherent.
- For LSEC isolation, carefully collect the supernatant and discard the HC-enriched pellet.
- Repeat 3 min at 50 × *g*, 4 °C centrifugation. Carefully collect the supernatant and discard the pellet.
- Centrifuge for 5 min at 500 × *g*, 4°C.
- Discard the supernatant and re-suspend the pellet in 5 ml ACK-lysis buffer, to lyse erythrocytes. Incubate the cells for 1-3 min at RT and stop lysis by adding 20 ml of cold HBSS.
- Discard the supernatant and re-suspend the pellet in an appropriate volume of FACS buffer.

#### 2.8 Materials: Hepatocyte *(HC)* and LSEC isolation

##### Reagents

- 1× Liver Perfusion Medium (Catalog Number: 17701038; Gibco, Thermo Fisher Scientific).
- Liver Digest Medium (Catalog Number: 17703034; Gibco, Thermo Fisher Scientific).
- Liberase^™^ TM Research Grade (Catalog Number: 5401119001; LIBTM-RO Roche; Sigma-Aldrich).
- 1× HBSS (Thermo Fisher Scientific).
- ACK lysis buffer: 150 mM NH4Cl, 10 mM KHCO3, 0.1mM Na2EDTA, pH 7.2 in dH20.
- Complete RPMI 1640 (10% FBS, 2 mM L-glutamine, 50 μM 2-mercaptoethanol, HEPES 10 mM, non-essential amino acid 100 μM, and penicillin plus streptomycin).
- Percoll, density 1.130 g/mol (GE Healthcare).
- 10× DPBS (+ MgCl2 and CaCl2).
- Williams E (Gibco) supplemented with 1% penicillin/streptomycin, 10% FCS, 1% l-glutamine.

##### Equipment

- CO2/O2 vaporizer.
- Water bath
- Peristaltic pump
- 70% EtOH v/v.
- Surgery instruments: Half-curved blunt microsurgery scissors, curved forceps
- Falcon^™^ Cell Strainer, 70 μm (Fisher Scientific)
- Petri dishes with scratched bottom
- 50 ml centrifugation tube
- 2.5 ml syringes
- 10 ml syringes
- Centrifuge
- 27-gauge needles

#### 2.9 Step-by-step sample preparation: LSEC staining

- Transfer 2 × 106 cells into a 96-well microtiter plate.
- To exclude death cells, prepare a LIVE/DEAD^™^ Fixable Near-IR Dead Cell staining solution in 1× PBS. Amine-based fixable dyes are recommended instead DAPI when functional downstream applications are required. Note that several amine-based fixable dyes with different emission spectra are available, so choose the most convenient one for your own panel.
- Add 50 μl live/dead staining solution/well and re-suspend the cells.
- Incubate for 15 min at RT in the dark.
- Fill 150 μl 1× PBS/well and centrifuge for 5 min at 500 × *g* at 4°C; discard supernatant.
- Incubate with 50 μl of Fc-block solution (aCD16/CD32, 5 μg/ml in FACS buffer) for 15 min at 4°C.
- Fill 150 μl FACS buffer to each well and centrifuge for 5 min at 500 × *g* at 4°C; discard supernatant.
- For detection of surface molecules, prepare an Ab solution in FACS buffer and re-suspend the cells in 50 μl Ab solution/well.
- Incubate for 30 min at 4°C in the dark.
- Fill 200 μl FACS buffer/well and centrifuge for 5 min at 500 × *g* at 4°C; discard supernatant.
- Take up the cells in FACS buffer/well and proceed to flow cytometric analysis.

#### 2.10 Materials: LSEC staining

##### Reagents

- FACS buffer: 1% FBS, 2 mM Na2EDTA, 0.05% NaN_3_ in PBS
- LIVE/DEAD^™^ Fixable Near-IR Dead Cell Stain (Thermo Fisher Scientific)
- Fc-receptor block
- Fluorophore-coupled Abs (see Table 152):

##### Equipment

- 96-well microtiter plate, u-bottom
- Centrifuge
- FACS tubes
- Flow cytometer equiped with the following lasers and filters
  - Laser: Violet (405 nm), Blue (488 nm), Red (633 nm).
  - Filter: 450/50 (405) for V450, 585/42 (488) for PE, 780/60 (488) for PE/Cy7, 660/20 (633) for AlexaFluor647, 780/60 (633) for LIVE/DEAD^™^ Fixable Near-IR.

#### 2.11 Step-by-step sample preparation: Hepatic stellate cell (HeSC) isolation

- Prepare the following solutions in glass bottles:
  - Pronase E solution: 150 ml Solution-2 + 60 mg Pronase E
  - Collagenase D solution: 120 ml Solution-2 + 60 mg Collagenase D (activity 0.153 U/mg Caution: If the enzyme activity is changed the quantity used needs to be adjust.
  - Collagenase D/Pronase E/DNase solution: 120 ml Solution-2 + 60 mg Pronase E + 60 mg Collagenase D + 800 μl DNase I
  - Nycodenz solution: 5.81 g/20 ml GBSS/A, filtered through 0.22 μm filter
- Incubate Solution-1 (150 ml), Pronase E solution, and Collagenase D solution in water bad at 40°C.
- Place the mice into the CO2/O2 vaporizer and anesthetize by gently flowing the chamber.
- Euthanize the animal by cervical dislocation.
- Place the mouse on a styrofoam pad with the ventral side upwards and fix the animal.
- Spread the abdomen with 70% EtOH and open the abdomen by making an incision in the cranial direction. Flap the skin to the side and fix with a needle.
- Tie off the vena cava superior with a thread
- Proceed with the *in-situ* liver perfusion via the portal vein.
  - Flow rate of the pump is 0.02% RS (1.8 ml/min)
  - Rinse hose of the pump with sterile ddH_2_O (about 20 ml)
  - Rinse hose of the pump with Solution-1 until ddH2O is completely removed
  - Attach 27G needle to the hose while the pump is running, thereafter, stop the pump
  - Caution: Avoid air bubbles in the hose
  - Fix the 27G needle in the portal vein, the liver becomes brighter and swells up
  - Cut the vena cava superior
  - Perfuse the mouse with Solution-1 (5 ml/min).
  - Stop perfusion with Solution-1 when the liver is pale.
  - Perfuse with Pronase E solution for 5 min (5 ml/min).
  - Perfuse with Collagenase D solution for 7-8 min (5 ml/min).
- Take the liver out and put it in a petri dish with 15 ml Solution. Remove the gallbladder. Mince the liver well. Keep it on ice.
- Incubate Collagenase D/Pronase E/DNase solution in the water bad at 37°C.
- Place a beaker with water on the stirring platform at 37 °C.
- Transfer the liver cells into an Erlenmeyer flask containing stirring bar. Add warm Collagenase D/Pronase E/DNase solution.
- Put the Erlenmeyer flask into the water of the beaker on the stirring platform.
- Incubate at 37°C for 25 min with slow stirring.
- Filter the digested liver through 70 μm cell strainers on 50 ml centrifugation tubes.
- Centrifuge for 10 min at 550 × *g*, 4°C. Discard supernatant except 5 ml.
- Add 5 ml GBSS/B + 120 μl DNase I and re-suspend the cells.
- Mix all cells in one 50 ml centrifugation tube.
- Add GBSS/B up to 50 ml and centrifuge for 10 min at 550 × *g*, 4°C.
- Discard supernatant except 5 ml.
- Add 5 ml of GBSS/B + 120 μl of DNase I and re-suspend pellet.
- Add GBSS/B up to 32 ml and add 16 ml of Nycodenz solution. Mix well.
- Transfer 12 ml of Nycodenz/cell solution into four 15 ml centrifugation tubes.
- Carefully pipet 1 ml GBSS/B solution onto the solution.
- Centrifuge for 17 min at 1400 × *g*, 4°C. Do not use break!
- Under the layer of clear GBSS/B solution, there is a white layer. This layer contains HSCs. Insert a 5 ml pipet into this layer. Collect this layer and transfer to a 50 ml centrifugation tube.
- Add GBSS/B solution up to 50 ml overall volume to wash and centrifuge for 10 min at 550-580 × *g*, 4°C.
- For culture, re-suspend HSCs in DMEM + 1% HEPES + 10% FCS + 1% Antibiotic-Antimycotic + Gentamycin (1:200).

#### 2.12 Materials: Hepatic stellate cell (HeSC) isolation

##### Reagents

- Pronase E (Merck Millipore)
- Collagenase D (Roche)
- DNase I (Roche), Stock solution: 2.5 mg/ml stored at −20°C
- GBSS/A (Gey’s balanced salt solution): KCl (370 mg/l), MgCl2 × 6 H20 (210 mg/l), Mg2SO4 × 7 H2O (70 mg/l), Na2HPO4 (59.6 mg/l), KH2PO4 (30 mg/l), glucose (991 mg/l), NaHCO3 (227 mg/l), CaCl2 × 2 H2O (225 mg/l)
- GBSS/B: NaCl (8 g/l), KCl (370 mg/l), MgCl2 × 6 H20 (210 mg/l), Mg2SO4 × 7 H2O (70 mg/l), Na2HPO4 (59.6 mg/l), KH2PO4 (30 mg/l), glucose (991 mg/l), NaHCO3 (227 mg/l), CaCl2 × 2 H2O (225 mg/l)
- Nycodenz (AXIS-SHIELD)
- Solution-1: NaCl (8 g/l), KCl (400 mg/l), NaH2PO4 × H2O (88.17 mg/l), Na2HPO4 (120.45 mg/l), HEPES (2.38 g/l), NaHCO3 (350 mg/l), EGTA (190 mg/l), glucose (900 mg/l)
- Solution-2: NaCl (8 g/l), KCl (400 mg/l), NaH2PO4 × H2O (88.17 mg/l), Na2HPO4 (120.45 mg/l), HEPES (2.38 g/l), NaHCO3 (350 mg/l), CaCl2 × 2H2O (560 mg/l)
- Antibiotic-Antimycotic (100x, Gibco)
- DMEM (Gibco)
- HEPES (Gibco)
- MACS buffer: PBS, 0.5% BSA, 2 mM EDTA, pH7.2

##### Equipment

- Glass bottles, Erlenmeyer flask, beaker
- Petri dishes, centrifugation tubes
- 27G needle
- Thread (Supramid)
- Cell strainer, 70 μm
- Water bath
- Heating plate with stirrer
- Centrifuge (Eppendorf 5810R)
- Hose pump (Medorex TL)

#### 2.13 Step-by-step sample preparation: Liver cell preparation to stain hepatic leukocytes

- Place the mice into the CO2/O2 vaporizer and anesthetize by gently flowing the chamber*
- Euthanize the animal by cervical dislocation
- Place the mouse on a styrofoam pad with the ventral side upwards and fix the animal
- Spread the abdomen with 70% EtOH and open the abdomen by making an incision in the cranial direction; flap the skin to the side and fix with a needle
- Take blood by cardiac puncture (~1 ml) to prevent contamination with peripheral blood cells Alternatively, the liver can be perfused *in situ*.
- Push the intestine sideward by using the blunt end of a forceps to get access to the liver
- Optional: if no blood sampling is required, *in situ* perfusion of the liver is recommended, using liver perfusion media or PBS
  - Perfuse the liver by gently injecting Liver Perfusion Media or PBS into the vena cava
  - The liver becomes blanched and swollen
  - Cut the portal vein. Blood and media should visibly flow from the vein
  - Continue perfusion with a total volume of 10 ml
- Remove the gall bladder
- Remove the liver and transfer it into 5 ml HBSS; store at RT
- Discard the HBSS and transfer the liver on a scribed petri dish
- Homogenize the liver by rubbing over the scribed surface using the pistil of a 2 ml syringe
- Fill ~5 ml of HBSS (RT) into the petri dish and transfer the homogenate into a 100 μm cell strainer placed on a 50 ml centrifugation tube. Alternatively, digestion of smashed liver tissue might improve cellular recovery, especially from fibrotic or cirrhotic livers as this procedure degrades extracellular matrix components, to which immune cells might adhere.
  - If choosing liver digestion, take up the smashed homogenate in 10 ml Liver Digest Medium and transfer it into a fresh 50 ml centrifugation tube
  - Incubate the cells for 30 min at 37°C
- Mince the homogenate through the cell strainer and wash with HBSS (RT) to remove fatty debris
- Fill up with HBSS to ~20–25 ml and centrifuge for 5 min at 500 × *g*, RT
- Carefully discard the supernatant and re-suspend the pellet in 10 ml 37% Percoll working solution
- Transfer the Percoll suspension into a 15 ml centrifugation tube and centrifuge for 20 min at 800 × *g*, RT; Caution: switch off the brake to assure proper assembly of the different phases
- Leukocytes and erythrocytes are pelleted on the bottom of the tube. Remove the upper, light brown layer, which contains hepatocyte debris, and carefully discard the supernatant
- For erythrocyte lysis, re-suspend the pellet in 3 ml ACK-lysis buffer and transfer the suspension into a fresh 50 ml centrifugation tube
- Incubate the cells for 5 min at RT and stop the reaction by adding 12 ml cold HBSS
- Centrifuge for 5 min at 500 × *g*, 4°C
- Discard the supernatant and re-suspend the pellet in 1 ml cold HBSS
- Determine the cell number
- Centrifuge for 5 min at 500 × *g*, 4°C
- Discard the supernatant and re-suspend the pellet in an appropriate volume of HBSS, depending on the amount of FACS-panels, which are designated for analysis***

*If whole blood is required for flow cytometric analysis, euthanize the animals by intravenous injection of a mixture of ketamine (120 mg/kg), xylazine (16 mg/kg), and heparin (8333 I:E/kg). Harvest blood by cardiac puncture as this allows a high yield and does not interfere with procedures such as liver perfusion.

#### 2.14 Materials: Liver cell preparation to stain hepatic leukocytes

##### Reagents

- 1× HBSS (for 1 l): 403 mg KCl, 53 mg Na2HPO4 × 2 H2O, 54 mg KH2PO4, 353 mg NaHCO3, 191 mg CaCl2 × 2H2O, 102 mg MgCl2 × 6 H2O, 148 mg MgSO4 × 7 H2O, 8.006 g NaCl, 1.11 g d-Glucose-Monohydrate

Add ddH2O and adjust to pH 7.2-7.4; filtrate solution through 0.22 μm filter

- Liver Digest Medium (ThermoFisher Scientific)
- Liver Perfusion Medium (ThermoFisher Scientific)
- ACK-lysis buffer (for 1 l): 8.25 g NH4Cl, 1 g KHCO3, 29.2 mg EDTA Add ddH2O and adjust to pH: 7.2-7.4
- Percoll, density 1.130 g/mol (GE Healthcare)
- 10× PBS (for 1 L): 80 g NaCl, 11.6 g Na2HPO4 × 2H2O, 2 g KH2PO4, 2 g KCl, add dH2O and adjust to pH7.2-7.4
- Percoll solution A: 3.70 ml Percoll, 0.29 ml 10× PBS, 0.05 ml 7.5% NaHCO3
- Percoll working solution (10 ml; for one liver): Add 6 ml HBSS and 0.2 ml Heparin to Percoll solution A and store it at RT.

##### Equipment

- CO2/O2 vaporizer
- Styrofoam pad
- Half-curved blunt microsurgery scissors
- Curved forceps
- Cell Strainer, 100 μm
- Petri dishes with scratched bottom
- 50 ml centrifugation tube
- 15 ml centrifugation tube
- 2 ml syringes
- Centrifuge (Eppendorf 5810R)

#### 2.15 Step-by-step sample preparation: Staining of hepatic NKT cells and *γδ* T cells

Note: The protocol is described in the absence of protein carrier such as FCS. However, the addition of protein carrier (i.e., 1% FCS (v/v)) to the blocking and extracellular staining solutions might improve cellular vitality and reduce unspecific binding of the respective Abs.

- Transfer hepatic leukocytes into a 96-well microtiter plate
- Centrifuge for 5 min at 500 × *g*, 4°C
- Prepare a working solution of 5 μg/ml Fc-receptor block in 1× PBS to block unspecific Ab binding (particularly important for macrophage analysis)
- Incubate the cells in 50 μl Fc-receptor blocking solution/well for 10 min at 4°C
- Fill 150 μl 1× PBS to each well and centrifuge for 5 min at 500 × *g*, 4°C
- For detection of death cells, prepare a live/dead staining solution in 1× PBS
- Add 50 μl live/dead staining solution/well and re-suspend the cells
- Incubate for 30 min at 4°C in the dark*
- Fill 150 μl 1× PBS/well and centrifuge for 5 min at 500 × *g*, 4°C
- For detection of surface molecules, prepare an Ab solution in 1× PBS and re-suspend the cells in 50 μl Ab solution/well
- Incubate for 30 min at 4°C in the dark
- Fill 150 μl 1× PBS/well and centrifuge for 5 min at 500 × *g*, 4°C
- Repeat the washing step
- Take up the cells in 150 μl PBS/well and proceed to flow cytometry analysis

*To our experience, LIVE/DEAD^™^ Fixable Red Dead Cell Stain Solution can be directly added to the Ab cocktail without an additional incubation step. However, we cannot recommend this for the LIVE/DEAD^™^ Fixable Aqua Dead Cell Stain Solution.

#### 2.16 Materials: Staining of hepatic NKT cells and γδ T cells

##### Reagents

- 1× PBS, optional 1× PBS/1% FCS (v/v)
- TruStain FcX^™^ (anti-mouse CD16/32 Abs; Fc-receptor blocking solution; BioLegend)
- LIVE/DEAD^™^ Fixable Red Dead Cell Stain Kit or LIVE/DEAD^™^ Fixable Aqua Dead Cell Stain Kit (both ThermoFisher Scientific)
- Fluorochrom-labeled Abs (see Table 153)

##### Equipment

- 96-well microtiter plate, v-bottom (Nunc^™^)
- Centrifuge (Eppendorf 5810R)
- FACS tubes
- Flow cytometer BD LSR FortessaTM
  - Laser: violet (405 nm), blue (488 nm), green (561 nm), red (640 nm)
  - Filter: 530/30(488) for FITC and AF488, 695/40(488) for PerCP/Cy5.5, 780/60(561) for PE/Cy7, 582/15(561) for PE, 780/60(640) for APC/Cy7, 670/14(640) for APC and AF647, 450/50(405) for BV421 and V450, 525/50(405) for AmCyan, 710/50(405) for BV711, 785/60(405) for BV785

#### 2.17 Data analysis

This is detailed in Figures 149-152.

#### 2.18 Pitfalls

LSEC in mice represents about 30-45% of non-parenchymal cells, and Kupffer cells and HSCs almost represent the rest of those non-parenchymal cells. However, they are also present, in a much lower percentage, non-LSEC endothelial cells, as arterial and venous endothelial cells. Is important to note that one of the major challenges in LSEC biology is the lack of a gold standard marker that could differentiate LSEC from other endothelial cells. For this reason, the gold standard for LSEC is the visualization of fenestrations by scanning electron microscopy. In our strategy, as in the vast majority of those currently used, LSEC are selected based on CD31 and CD146 positive expression (which also binds to endothelial cells from a various range of organs and tissues) so it should be pointed out that this population may contain minor non-LSEC endothelial cell contamination.

On the other hand, the viability and total number of LSEC in mouse liver can vary considerably in various genetically modified murine models or pathological conditions (infections, tumors, etc.). The quality of the liver perfusion can also be affected by those conditions, even being necessary to carry out perfusions from the right ventricle of the heart.

#### 2.19 Top tricks

##### Protocol for LSEC staining

The relatively low numbers of LSEC in some conditions may limit the need for high numbers of purified cells for particular experiments (FACS, adoptive transfers, proteomics, sc-RNAseq, etc.). To accommodate for this problem, pre-enrichment of the LSEC population by Magnetic Activated Cell Sorting (MACS) can save sorting time by eliminating cell debris and non-target cells. Using the CD146 (LSEC) MicroBeads Kit from Miltenyi, LSEC that express CD146 are magnetically labeled. The cell suspension is loaded onto a MACS Column, which is placed in the magnetic field of a MACS Separator. The magnetically labeled LSEC are retained within the column and the unlabeled cells run through. After removing the column from the magnetic field, the magnetically retained LSEC can be eluted as the positively selected cell fraction. The percentage of LSEC population reaches about 90-95% of total living cells after purification (from an initial one of 40-50%).

##### Protocol for HeSC isolation

For higher purity of the HeSC population, an additional Kupffer cell depletion by MACS is recommended.

- Re-suspend the cells in 15 ml MACS buffer in a 15 ml centrifugation tube and centrifuge for 10 min at 800 g, 4°C.
- Re-suspend the cells in 100 μl MACS buffer and add Biotin-conjugated anti-F4/80 Ab (BM8, Biolegend).
- Incubate for 25 min at 4°C.
- Add anti-Biotin MicroBeads (Miltenyi) and incubate for another 25 min at 4°C.
- Wash in 10-20× volume of MACS buffer. Meanwhile mount MACS column and pipet 3 ml MACS buffer into the column. Place a 15 ml centrifugation tube under the column to collect the unlabeled HSCs.
- Re-suspend the cells in 500 μl MACS buffer (for LS column) and load them onto the column. Collect flow through. Wash 3x with 3 ml MACS buffer and collect the flow through.

#### 2.20 Summary of the phenotypes

This is detailed in Table 154 (with references [1243, 1675-1678]).

#### 2.21 Key information human vs murine

This is detailed in Table 154.

##### Differences in the phenotype and function of human and murine liver-resident lymphocytes

- The predominant liver-resident NK cells in the human liver are defined as Tbet^low^ Eomes^hi^ and can also be identified by surface expression of CXCR6 ± CD69. In contrast, murine liver-resident NK cells (also referred to as ILC1) have a distinct transcription factor profile, characterized as Tbet^hi^ Eomes^low^. Murine liver-resident NK cells can be further defined by their surface expression of CD49a in the absence of DX5; a small CD49-expressing NK cell population in the human liver has also been identified. Murine liver-resident NK cells constitutively express TRAIL, while in humans TRAIL only becomes detectable on intrahepatic NK cells in diseased settings such as HBV infection. Importantly, human and liver resident NK cells have similar frequencies and intra-sinusoidal locations [1245, 1247, 1263, 1674, 1679-1683].
- In the human liver, mucosal-associated invariant T cells (MAIT cells) expressing Vα7.2 are the dominant population of innate-like T cells, representing 20-50% of intrahepatic T cells, with invariant NKT cells (iNKT) make up only ~1% and γδ T cells a further ~15%. MAIT cells express CD161, and chemokine receptors that allow them to home to the liver including CXCR6 and CCR6. By contrast in the murine liver MAIT cells are very low frequency and iNKT cells more dominant iNKT cells are characterized by signatures of both T and NK cells, including a broad range of expression of markers typically associated with NK cells, e.g., NK1.1, although alone, insufficient to identify iNKT cells. [635, 646, 1684-1686]
- Human liver-resident CD8^+^ T cells are defined by surface expression of CD69 ± CD103, further expressing high levels of CXCR6, CXCR3, and CD49a. In contrast, murine liver CD8^+^ Trm lack expression of CD103. Murine CD8^+^ Trm can be identified by surface expression CXCR6, CD69 and/or CD49a [239, 1673, 1687-1690].

## Figures and Tables

**Table 1.** Examples of Minimum Information (MI) standards for cell based and immunological experiments

Abbreviation	Title	Reference	Comments
MIATA	Minimal Information About T Cell Assays	[35]	Supported a.o. by EJI
MIFlowCyt	Minimum Information about Flow Cytometry	[31]	Guidelines
MISEV2018	Minimal Information for Studies of Extracellular Vesicles	[36]	
MiSet RFC	Minimum Set of Standards Required for Reproducibility and Rigor in Research Flow Cytometry Experiments	[32]	proposal
MIFlowCyt-EV	Minimal Information for Studies of Extracellular Vesicles by Flow Cytometry	[37]	Guidelines
MIACARM	Minimum Information About a Cellular Assay for Regenerative Medicine	[38]	proposal
MITREG	Minimum Information about T Regulatory Cells	[39]	
MITAP	Minimum Information about Tolerogenic Antigen-Presenting cells	[40]	

**Table 2.** Required and facultative data for MiFlowCyt compliant cytometric publications

Data set	MiFlowCyt mandatory data	Further information, facultative
Sample/Specimen	Type, source, source treatment, phenotype, genotype, location	circumstances of sampling
Sample Treatment	Sampling, storage, preprocessing (chemical or mechanical), staining	Storage/transport temperature and humidity
Reagents	Chemicals, analytes, Antibody clones, names/numbers, manufacturer, catalogue numbers	Concentration, purity
Controls	Quality Control Measures, FMOs, Positive/negative control	For heterogeneous biocenosis individual species, if available.
Instrument	Manufacturer, model, configuration, settings, detector voltages, optical filters	If instrument is modified details on modification and performance.
Original data	Positive, negative controls, single species controls, instrument calibration measurements. Data reposition in flow repository.	Gating strategy, if applicable
Instrument	Manufacturer, model, configuration, settings, detector voltages, optical filters	If instrument is modified details on modification and performance.
Data Analysis	List-mode data file, compensation, gating, FlowRepository data access code	Software used for further analysis

**Table 3.** Checklist for graphical data display

Histogram type	Axes	Axes legend	Gates/Regions	Other
One Dimensional (1D)	Scaling with ticks	With (standard) dye name, emission frequency range (color), linear or logarithmic scaling (if not self-explaining)	Analysis regions if used, with frequency or cell count information and name of the cells in the respective gates/regions	Ideally data are normalized to 100%
Two-Dimensional (2D)	As for 1D	As for 1D	Full gating strategy with cell frequencies and cell counts in the gates. Total number of events analyzed.	If color coding is used, explanation in legend or graph
Dimensionality reduction display (SPADE, tSNE, FlowSOM etc.)	Not applicable	Not applicable	Gates for clusters, if applicable. Fluorescence intensity distribution for each dye/wavelength range with scaling (Example in Figure 4A)	Population distribution (color coded) with legend (and frequencies)

**Table 4.** Example spillover spreading matrix for a 22-color experiment (SSM). Columns are to be interpreted as the detectors that are receiving spreading error. The value of the spreading error is relative and additive, i.e., a detector that is receiving spreading error from several fluorochromes is overall showing a higher loss in resolution. Examples as to how the SSM can be utilized during panel design are described in “experimental workflow”

_fluorochrome_╲^detector^	B-530 (FITC)	B-710 (PerCPCy5.5)	R-670 (APC)	R-710 (AF700)	R-780 (APC-Cy7)	U-379 (BUV395)	U-570 (BUV563)	U-610 (BUV610)	U-660 (BUV661)	U-740 (BUV737)	U-800 (BUV805)	V-450 (BV421)	V-470 (BV510)	V-570 (BV570)	V-610 (BV605)	V-655 (BV650)	V-710 (BV711)	V-780 (BV785)	YG-586 (PE)	YG-610 (Pe-Tx)	YG-670 (Pe-Cy5)	YG-780 (Pe-Cy7)	total SE contributed
FITC	0	0.599	1.07	0	0	0	0	0.356	0	0.729	0	0	0.396	0.449	0.455	0.386	0.24	0	1.37	1.06	0	0.296	7.406
PerCPCy5.5	0.247	0	2.24	4.18	1.91	0.223	0	0	0.275	2.92	1.76	1.72	0.465	0	4.92E-03	0.698	1.91	2.4	0	1.57	3.3	3.1	28.92292
APC	0	1.09	0	2.78	1.29	0.0662	0.0775	0	0.268	0.889	0.486	0.361	0	0	0.183	0.905	0.55	0.433	0.811	0.391	4.28	1.23	16.0907
AF700	0.132	0.5	0.767	0	2.18	0.119	0.139	4.87E-04	0	1.62	0.738	0.44	0.143	1.99E-03	0	0.143	0.368	0.737	1.29	0.907	0.444	1.77	12.439477
APC-Cy7	0.0822	0.231	0.847	0.639	0	0.0796	0.125	0.0848	0.132	0.747	2.06	0.974	0.0922	0	0.0874	0.196	0.142	1.33	0	0	0.507	5.49	13.8462
BUV395	0.896	0	0	0	0.861	0	0	0	0	0	0	0.624	0	1.39	0	0	0	0.709	0	0	1.5	0	5.98
BUV563	0.375	1.33	2.55	0	0	2.01	0	1.26	0.853	2.32	1.29	0.602	0.492	2.66	1.39	0.739	0.53	0.302	22.3	11.7	3.23	1.2	57.133
BUV610	0	2.22	3	1.15	0.442	1.34	0	0	0.781	3.34	1.97	0.517	4.11E-03	1.27	1.4	1.03	1.01	0.63	8.39	18.8	5.12	2.43	54.84411
BUV661	0	1.66	12.9	6.44	3.07	0.953	0	0.535	0	7.07	3.77	0.627	0.565	0.533	0.627	1.9	0.979	1.11	0	1.91	5.4	2.68	52.729
BUV737	0	0.519	0	1.72	1.22	0.182	0	0.194	0	0	2.17	0.347	0	0.242	0	0	0.337	0.605	1.97	1.07	0.649	0.816	12.041
BUV805	0.237	0	0.513	0	1.07	0.967	0	0.165	0	0.641	0	0.381	0	0.294	0.165	0.175	0	0.549	0.0998	0	0	0.849	6.1058
BV421	0	4.24E-03	0.738	0	0	0.123	0	0	0	0.576	0.19	0	0.632	0.182	0.214	0.157	0.132	0.118	0.264	0	7.97E-03	0.175	3.51321
BV510	0.207	0.235	0	5.15E-03	0.193	0.13	0.153	0.148	0.224	0	0	0.563	0	1.39	0.952	0.627	0.284	0.313	1.51	0.865	0	0.176	7.97515
BV570	0.205	0.749	1.27	0.534	0	0	0.223	0.485	0	1.37	0.416	1.48	0.386	0	2.76	2.03	0.695	0.761	7.75	4.02	1.59	0.794	27.518
BV605	0	0.927	2.23	0.449	0.466	3.11E-03	0	0.81	0.283	2.04	0.971	1.35	0.396	2.61	0	3.22	1.27	1.42	4.74	5.48	2.42	1.36	32.44511
BV650	0	0.889	0	1.38	0	0	1.15	0	1.18	3.71	1.28	1.36	1.15	1.64	1.12	0	1.5	1.65	0	0	0	1.57	19.579
BV711	0.839	1.61	1.27	3.72	1.86	0.784	1.1	0.584	0.638	3.77	3.15	2.54	0.914	1.32	0.62	0.655	0	3.59	0	3.28	0	1.85	34.094
BV785	0	0.389	1.05	0.608	0.809	0.316	0	0.349	0	1.94	2.47	3.22	0.641	0	0.349	0.544	0.567	0	0	2.03	0.834	0.62	16.736
PE	0.144	0.505	0.688	0.129	0	0.0902	0	0.153	0.161	0.505	0.18	0.338	0.113	1.44	0.81	0.465	0.239	0.225	0	3.32	1.16	0.382	11.0472
Pe-Tx	0	1.09	0.552	0.455	0.124	1.01E-03	0	0.254	0.122	0.513	0.259	0.249	0.0573	0.511	0.736	0.597	0.531	0.282	3.4	0	2.06	1.08	12.87331
Pe-Cy5	0.0981	2.05	2.58	1.68	0.695	0.0638	0	0.101	0.0805	0.885	0.409	0.305	0.0746	0.158	0.151	0.681	1.07	0.446	1.52	0.683	0	2.07	15.801
Pe-Cy7	0.207	0.346	0.444	0.257	0.559	0.187	0.306	0	0	0.721	0.874	0.629	0.225	0	0	0.225	0	0.865	0	1.25	0.489	0	7.584
total SE collected	3.6693	16.94324	34.709	26.12615	16.749	7.63792	3.2735	5.479287	4.9975	36.306	24.443	18.627	6.74621	16.09099	12.02432	15.373	12.354	18.475	55.4148	58.336	32.99097	29.938	

**Table 5.** Example for events with a frequency of cells of 0.01%

Acquired events **(N)**	100,000	1,000,000	4,010,000	10,000,000
Positive **(R)**	10	100	401	1,000
Proportion **(P)**	0.0001	0.0001	0.0001	0.0001
Variance **(Var)**	10.0	100.0	400.6	999.9
Standard deviation **(SD)**	3.16	10.0	20.1	31.62
Coefficient of Variation **(CV)**	31.62	10.00	**4.99**	**3.16**

The example shows how to calculate the number of events to acquire, and is related to a population whose final frequency is 0.01%. Note that to obtain a CV lower than 5, for example 3.16, it is mandatory to acquire 10 million events; acquiring 4,010,000 million events provides a CV=4.99.

**Table 6.** Optical setup of the BD Fortessa used in the flow cytometry analysis

Laser Wavelength [nm]	Laser Power [mW]	Laser Type	Spectral Range for Detector [nm]	Dichroic Filters		Band Pass Filter [nm]	Fluorochrome	
				A [nm]	B [nm]			
633	40	DPSS	750-810	–		750	780/60	APC Cy7
			705-755		750	690	730/45	Ax700
			655-685		690	–	670/14	APC
561	50	DPSS	750-810	–		750	780/60	PE Cy7
			685-735		750	685	710/50	Pe Cy5.5
		655-685		685	635	670/30	Pe Cy5	
		600-630		635	600	610/20	PE TR	
		575-590		600	–	582/15	PE	
488	50	DPSS	685-735	–		685	710/50	PerCP eFluor710
			515-545		685	505	530/30	FITC
					505	–	SSC	
405	50	DPSS	750-810	–		735	780/60	QD800
			685-735		735	685	710/50	QD700
			650-670		685	635	660/20	QD655
			600-620		635	600	610/20	QD605
			500-550		600	505	525/50	BV500
			425-465		505	–	450/50	Pacific Blue / Bv421 / Alexa405

**Table 7.** Selection of common markers for flow cytometry analysis of human CD4^+^ lymphocytes

	T_REG_	T_N_	Tcm	Tem	T_EMRA_	cT_FH_	T_FH_	T_SCM_	T_CTL_	T_H_1	T_H_2	T_H_1*	T_H_17	T_H_22
**Marker**														
CD4	+	+	+	+	+	+	+	+	+	+	+	+	+	+
CD127	−	+	+	+	+	+	+	+	−	+	+	+	+	+
CD25	+	−	lo/−	lo/−	lo/−	lo/−	lo/−	lo/−	−	lo/−	lo/−	lo/−	lo/−	lo/−
CCR7	+/−	+	+	−	−	+/−	−	+	−	+/−	+/−	+/−	+/−	+/−
CD45RA	+/−	+	−	−	+	+/−	−	+	+/−	−	−	−	−	−
CD95	+/−	−	+	+	+	+	+	+	ND	+	+	+	+	+
CXCR5	+/−	−	−	−	+/−	+	+	+/−	−	−	−	−	−	−
ICOS	+/−	−	−	lo/−	ND	lo/−	+	−	ND	lo/−	lo/−	lo/−	lo/−	lo/−
CCR6	+/−	−	+/−	+/−	ND	+/−	−	+/−	ND	−	−	+	+	+
CXCR3	+/−	−	+/−	+/−	+/−	+/−	ND	+/−	+/−	+	−	+	−	−
CCR4	+/−	−	+/−	+/−	ND	+/−	ND	ND	ND	−	+	−	+	+
CCR10	ND	−	+/−	+/−	ND	+/−	ND	ND	ND	ND	ND	ND	ND	+
CRTh2	ND	−	lo/−	+/−	ND	−	ND	ND	ND	lo/−	+/−	lo/−	lo/−	lo/−
CD27	+	+	+	+/−	lo/−	+	ND	+	−	+/−	+/−	ND	+/−	ND
CD28	+	+	+	+	lo/−	+	ND	+	−	+	+	+	+	+
Granzyme B	lo/−	−	−	lo/−	+/−	−	ND	−	+	+/−	−	ND	−	−
**Transcription factor**														
GATA3	+/−	−	−/lo	+/−	−	−/lo	+/−	−/lo	−	−	+/−	−	−	−
T-bet	+/−	−	−/lo	+/−	+	−/lo	ND	−/lo	+	+^a^/−	−	+^a^/−	lo^a^/−	−
RORC2/RORγT	+/−	−	−/lo	+/−	−	−/lo	ND	−/lo	−	lo^a^/−	−	+^a^/−	+^a^/−	−
AHR	ND	−	ND	ND	ND	ND	ND	ND	ND	ND	ND	ND	ND	+
BCL6	−	−	−	−	−	lo	+	−	lo/−	lo/−	lo/−	lo/−	lo/−	lo/−
Foxp3	+	−	−	−	−	−	−	−	−	−	−	−	−	−
Helios	+/−	−	ND	ND	ND	ND	ND	ND	lo/−	lo/−	lo/−	lo/−	lo/−	lo/−
**Cytokine**														
IFN-γ	−/lo	−	−/+	+	+	−/lo	−/lo	−/lo	+	+	−	+	−	−
IL-4	−/lo	−	−/lo	−/+	−/lo	−/lo	−/+	−/lo	−/lo	−	+	−	−	−
IL-17A	−/lo	−	−/+	−/+	−/lo	−/lo	−/lo	−/lo	−/lo	−	−	lo	+	−
IL-22	−/lo	−	ND	ND	ND	ND	ND	ND	ND	−	−	lo	+	+

+ indicate high expression, − indicates no expression, +/− indicates bimodal expression, lo indicates low expression

a indicates expression on activated cells, ND indicates not determined

**Table 8.** Molecules that discriminate subpopulations of conventional murine CD4αβ T cells (identified by expression of CD3 or TCRαβ, lack of TCRγδ and CD8, and expression of CD4)

Markers	TN cell	Treg cell	Th1	Th2	Th9	Th17	Th22	Tfh	Tcytotox	Tcm cell	Tem cell	Trm cell
**Surface markers**												
CD44	**lo**	**lo**	**hi**	**hi**	**hi**	**hi**	**hi**	**hi**	**hi**	**hi**	**hi**	**hi**
CD62L	**hi**	**hi**	**hi** or **lo**	**hi** or **lo**	**hi** or **lo**	**hi** or **lo**	**hi** or **lo**	**hi** or **lo**	**hi** or **lo**	**hi**	**lo**	**lo**
CD69	**lo**		**hi** or **lo**	**hi** or **lo**	**hi** or **lo**	**hi** or **lo**	**hi** or **lo**	**hi** or **lo**	**hi** or **lo**	**lo**	**lo**	**hi**
CD103												**hi** or **lo**
CXCR3			**hi**									**hi**
CCR4				**hi**								
CCR6						**hi**						
CXCR5								**hi**				
PD-1								**hi**				
**Intracellular markers to be stained ex vivo**												
Granzyme B									**hi**			
Perforin									**hi**			
**Markers to be stained after restimulation**												
CD40L	**lo**	**lo**	**hi**	**hi**	**hi**	**hi**	**hi**	**hi**	**hi**			
IFNg			**hi**									
TNF			**hi**									
IL-2			**hi**									
IL-4, IL-13				**hi**								
IL-9					**hi**							
IL-17						**hi**						
IL-22							**hi**					
IL-21								**hi**				
**Intranuclear markers and transcription factors**												
Bach2	**hi**											
Foxp3		**hi**										
Tbet			**hi**									
Gata-3				**hi**								
Irf4					**hi**							
PU.1					**hi**							
RORgt						**hi**						
Ahr							**hi**					
Bcl6								**hi**				
Eomes									**hi**			

**Table 9.** Summary of the phenotypes of Tn CD8, T_SCM/CM_, T_PEX_, T_TM_, Tem, and T_TE_ cells

	Tn CD8	T_SCM/CM_	T_PEX_	T_TM_	Tem	T_TE_
**Surface markers**						
CCR7	+	+	+	−	−	−
CD62L	+	+	+	−	−	−
CD27	+	+	+	+	−	−
CD28	+	+	+	+	−	−
CD127	+	+	+	+	+/−	−
CD45RA	+	+/−	+/−	−	−	+
CXCR3	+/−	+	ND	ND	+	+/−
CD95	−	+	+	+	+	lo
CD45RO	−	+/−	+/−	+	+	−
PD-1	−	−	+	+/−	+/−	+/−
TIGIT	−	−	+	+/−	+/−	+/−
CX_3_CR1	−	−	ND	ND	+/−	+/−
CD57	−	−	ND	ND	+/−	+/−
**Intracellular markers**						
TCF-1	+	+	+	ND	−	−
Granzyme K	−	+/−	+	+/−	+/−	+/−
T-bet	−	lo	lo	+/−	+	+
Granzyme B	−	−	−	+/−	+	+

+ indicates high expression, − indicates no expression, +/− indicates bimodal expression, lo indicates low expression, ND indicates not determined.

**Table 10.** Molecules that discriminate subpopulations of conventional murine CD8αβ T cells (identified by expression of CD3 or TCRαβ, lack of TCRγδ and CD4, expression of CD8a and CD8b)

Marker	CD8 ab T cells						
	TN	TEFF cell (SLEC)	TEFF cell (MPEC)	TVM cell	TCM cell	TEM cell	TRM cell
**Surface markers**							
CD44	lo	hi	hi	hi	hi	hi	hi
CD62L	hi	hi	hi	hi or lo	lo	lo	Hi
CD69	hi or lo	hi or lo	hi or lo		lo	lo	hi or lo
CD103	hi or lo	hi or lo	hi or lo		lo	lo	hi or lo
CXCR6							hi
CD127	hi	lo	hi				
KLRG1	lo	hi	Lo				lo
CD49d	int	hi	hi	lo	hi	hi	hi
CD122	lo	hi	lo	hi	int	hi	
CD49a	lo	hi or lo	hi or lo		hi or lo	int	hi
**Intracellular markers to be stained ex vivo**							
Granzyme		hi	hi				
B							
Perforin		hi	hi				
**Markers to be stained after restimulation**							
IFNg	lo	hi	hi	hi	hi	hi	hi
TNF	lo	hi	hi	hi	hi	hi	hi
IL-2	lo	hi	hi	lo	hi	int	int
**Intranuclear markers and transcription factors**							
Bach2	Hi						
Tbet		hi	hi			hi	
Bcl-2				hi			
Eoms				hi	Int		
Hobit							hi
Blimp1							hi

**Table 11.** List of useful Abs for identifying human Trm. Underlined Abs were used in the staining panel for the analysis shown in Figure 19

Species	Epitope	Clone	Company	Fluorochrome
Human	CD45	2D1	Biolegend	PE-Cy7
Human	CD3	SK7	Biolegend	APC-Cy7
Human	CD4	SK3	eBioscience	PE-Cy5.5
Human	CD8	RPA-TB	Biolegend	BV785
Human	CD45RO	UCHL1	Biolegend	BV650
Human	CD69	FN50	Biolegend	BV421
Human	CD25	M-A251	BD Pharmingen	APC
Human	CD127	A019D5	Biolegend	A488
Human	CD3	UCHT1	BD	BUV661, BUV395
Human	CD4	SK3	BD	BUV737
Human	CD8	SK1	BD	BUV805
Human	CD69	FN50	BD	BUV395, BV421
Human	CD28	CD28.2	BD	BV605
Human	CD103	Ber-ACT8	BD	FITC
Human	CD49a	SR84	BD	PE
Human	TNFα	Mab11	BD	FITC, PeCy7
Human	CD45RA	HI100	BD	BUV563
Human	CD25	2A3	BD	PE, BV605
Human	GZMB	GB11	BD	AF700, AF647
Human	CD137	4B4-1	Thermofisher	AF647, PeCy7
Human	CD4	S3.5	Invitrogen	QD705
Human	CD3	UCHT1	Invitrogen	QD605
Human	CD45RA	MEM-56	Invitrogen	QD655
Human	CD3	OKT3	ThermoFisher	eVolve605, SuperBright600
Human	CD103	B-Ly7	ThermoFisher	PE, PeCy7, FITC
Human	Eomes	WD1928	ThermoFisher	eFluor660
Human	HLA-DR	LN3	ThermoFisher	FITC
Human	2B4	eBioDM244	ThermoFisher	APC
Human	CXCR6	K041E5	Biolegend	APC
Human	CCR5	J418F1	Biolegend	BV421
Human	CD27	O323	Biolegend	BV510, BV650
Human	CD103	Ber-ACT8	Biolegend	AF647, BV711, BV605, BV421
Human	CD69	FN50	Biolegend	PeDazzle594, AF647
Human	Tbet	4B10	Biolegend	BV421
Human	IFNγ	4S.B3	Biolegend	eF450, BV785, Pe
Human	PD-1	EH12.2H7	Biolegend	BV421, PeCy7
Human	CTLA4	L3D10	Biolegend	PE
Human	TNFα	Mab11	Biolegend	PeDazzle594, BV421

**Table 12.** Phenotype markers and their functional implications

Marker	Expression	Functional implication	Reference
CD69	+	Tissue retention	[241, 244, 248, 253-259, 263, 267]
CD103	+ (mucosa/skin)	Tissue retention	[241, 244, 253, 254, 256-258]
CD45RA	+/−	Memory defining	[241, 244, 253, 254, 256-258]
CD45RO	+	Memory defining	[241, 244, 253, 254, 256-258, 267]
CD27	+/−	Co-stimulation	[256, 257]
CD28	+/−	Co-stimulation	[254, 257]
CCR7	−	Lymph node homing	[253, 258, 267]
CD49a	+	Retention	[241, 258]
CXCR6	+	Recruitment/retention	[241, 259]
CCR5	+	Recruitment/retention	[257, 259]
CXCR3	+	Recruitment/retention	[257, 258]
CX3CR1	−	Cytotoxic function, endothelial adhesion	[241, 259]
IL-7Ra	+	Homeostasis	[241, 254]
PD-1	+	Inhibitory	[256-258, 264]
CTLA-4	+	Inhibitory	[257, 258]
2B4	+	Inhibitory	[257]
CD101	+ (CD8 Trm)	Inhibitory	[264]
NKG2A	+ (CD8 Trm)	Inhibitory NK receptor	[280]
CD94	+ (CD8 Trm)	Forms inhibitory receptor complex with NKG2A	[280]
Eomes	−	Effector function/effector differentiation	[241, 256, 258]
T-bet	−/+	Effector function/differentiation/lineage defining	[241, 256, 258]
HLA-DR	−	Activation	[264, 267]
CD25	−	Activation	[244, 253, 258, 264]
CD38	−	Activation	[264]
Ki67	−	Proliferation	[244, 258, 264, 267]

**Table 13.** Phenotype markers and their functions

Marker	Expression	Function	Reference
IFN-γ	+ (upon stimulation)	Kill or recruit cells	[241, 244, 256, 257, 267]
TNF-α	+ (upon stimulation)	Kill or recruit cells	[244, 257, 267]
IL-2	+ (upon stimulation)	Survival/proliferation	[241, 244, 257, 267]
IL-17a	+ (upon stimulation)	Extracellular pathogen protection	[241, 244, 267]

**Table 14.** Summary human phenotype table. List of markers to define and distinguish human iIELs subsets. + Indicates high expression, − indicates no expression, +/− indicates bimodal expression, lo indicates low expression, and ND indicates not determined

	TCRγδ	TCRαβ	NK-like
Marker			
CD3	+	+	−
CD7	+	+	+/−
CD103	+	+	+
CD4	Lo/−	Lo/−	lo/−
CD8a	+/−	+/−	+/−
CD8β	ND	+	−
TCRγδ	+	−	−
TCRαβ	−	+	−
CD45	+	+	+
NKG2A	−	+/−	−
NKG2D	lo/−	Lo/−	−
CD69	+	+	−

**Table 15.** Summary phenotypes of murine intraepithelial lymphocytes

	TCRαβ			TCRγδ	
	CD4	CD8αβ	CD8αα	CD8αα	Vγ7
Marker					
CD3	+	+	+	+	+
CD4	+	−	−	Lo/−	Lo/−
CD8α	−	+	+	+	+
CD8β	−	+	−	Lo/−	Lo/−
TCRαβ	+	+	+	−	−
TCRγδ	−	−	−	+	+
Vγ7	−	−	−	+/−	+
Vδ6.3	−	−	−	+/−	+/−
Vδ4	−	−	−	+/−	+/−
CD45	+	+	+	+	+
CD103	+/−	+/−	+	+	+
CD69	+	+	+	+	+
CD122 (IL2Rβ)	Lo/−	Lo/−	+	+	+
CD44	+	+	+/−	+/−	+/−
Ly49E/F	−	−	+/−	+/−	ND
Lag3	Lo/−	Lo/−	+	+	+
CD73	+/−	+/−	+	+	+
Cytokines					
Granz A	+/−	+/−	+/−	+/−	ND

+ Indicates high expression, − indicates no expression, +/− indicates bimodal expression, lo indicates low expression, and ND indicates not determined.

**Table 16.** Key differences between murine and human intraepithelial lymphocytes

	Mouse							
	TCRαβ			TCRγδ		Human		
	CD4	CD8αβ	CD8αα	CD8αα	Vγ7	TCRαβ	TCRγδ	NK-like
**Marker**								
**CD3**	+	+	+	+	+	+	+	+/−
**CD4**	+	−	−	Lo/−	Lo/−	Lo/−	Lo/−	Lo/−
**CD8α**	−	+	+	+	+/−	+/−	+/−	+/−
**CD8β**	−	+	−	−	Lo/−	+	ND	−
**TCRαβ**	+	+	+	−	−	+	−	−
**TCRγδ**	−	−	−	+	+	−	+	−
**Vγ4**	NA	NA	NA	NA	NA	−	+	−
**Vδ1**	NA	NA	NA	NA	NA	−	+/−	−
**Vγ7**	−	−	−	+/−	+	NA	NA	NA
**Vδ6.3**	−	−	−	+/−	+/−	NA	NA	NA
**Vδ4**	−	−	−	+/−	+/−	NA	NA	NA
**CD45**	+	+	+	+	+	+	+	+
**CD103**	+/−	+/−	+	+	+	+	+	+
**CD69**	+	+	+	+	+	+	+	−
**CD122 (IL2Rβ)**	Lo/−	Lo/−	+	+	+	ND	ND	ND
**CD44**	+	+	+/−	+/−	+/−	ND	ND	ND
**Ly49E/F**	−	−	+/−	+/−	ND	ND	ND	ND
**Lag3**	Lo/−	Lo/−	+	+	+	ND	ND	ND
**CD73**	+/−	+/−	+	+	+	ND	ND	ND
**NKG2A**	ND	ND	ND	ND	ND	+	−	+
**NKG2D**	−	Lo/−	Lo/−	−	ND	Lo/−	Lo/−	−
**Cytokines**								
**Granz A**	+/−	+/−	+/−	+/−	ND	ND	ND	ND

+ Indicates high expression, − indicates no expression, +/− indicates bimodal expression, lo indicates low expression, and ND indicates not determined.

**Table 17.** Reagents for surface staining of human T cell aging

Antibody	Clone	Provider
CD45RA FITC	2H4	Beckman Coulter
CCR7 PE	G043H7	Beckman Coulter
CD28 ECD	CD28.2	Beckman Coulter
PD1 PC5.5	PD1.3.5	Beckman Coulter
CD27 PC7	1A4.CD27	Beckman Coulter
CD28 ECD	CD28.2	Beckman Coulter
CD4 APC	13B8.2	Beckman Coulter
CD8 A700	B9.11	Beckman Coulter
CD3 APC-A750	UCHT-1	Beckman Coulter
CD57 Pacific Blue	NC1	Beckman Coulter
CD45 Krome	J33	Beckman Coulter
CD25 BV785	BC96	Biolegend
CD127 BV650	A019D5	Biolegend
CD95 BUV395	DX2	Becton Dickinson
CD38 BUV496	HIT2	Becton Dickinson
HLA-DR BUV661	G46-6	Becton Dickinson

**Table 18.** Summary phenotype of human T cells

	Differentiation					Activation and exhaustion	
	Naïve	SCM	CM	EM	TEMRA	Exhaustion/senescence	Activation
CD45RA	+	+	−	−	+		
CCR7	+	+	+	−	−		
CD27	+	+	+	+/−	+/−		
CD28	+	+	+	+/−	−		
CD95	−	+	+	+	+		
CD38						+/−	+
HLA-DR						+/−	+
CD57						+	+/−
CD279 (PD-1)						+	+/−
KLRG1						+	+/−

**Table 19.** Summary table of aged CD8 T cell subpopulations in naïve mice

Subset of CD8 T cells	Phenotype	% of CD8 T cells	
		In young mice	In aged mice
Naive	CD44^lo^ CD62L^+^ (CD49d^lo/int^)	70-85%	30-40%
Virtual memory, TVM	CD44^hi^ CD62L^+^ CD49d^lo^	10-15%	20-40%
Central memory, Tcm	CD44^hi^ CD62L^+^ CD49d^hi^	5-10%	10-15%
Effector memory, Tem (and effector, Teff)	CD44^hi^ CD62L^lo^ (CD49d^hi^)	2-5%	20-30%

**Table 20.** Summary table of aged CD8 T cell subpopulations in chronically infected mice

Subset of CD8 T cells	Phenotype
Naive	CD11a^lo^ CD44^lo^ CD27^+^ KLRG1^−^ CD62L^+^ CD122^−^
Central memory, Tcm, and virtual memory, TVM	CD11a^hi^ CD44^hi^ CD27^+^ KLRG1^−^ CD62L^+^ CD122^+^
Effector memory, Tem	CD11a^hi^ CD44^hi^ CD27^+^ KLRG1^−^ CD62L^−^ CD122^−/lo^
Terminally Differentiated Effector, TTDE	CD11a^hi^ CD44^hi^ CD27^−^ KLRG1^+^ CD62L^−^ CD122^−^

**Table 21.** Summary phenotype of T cells in mice and humans

	Differentiation									
	Naïve		SCM		CM		EM		TDE	
	Hu	Mu	Hu	Mu	Hu	Mu	Hu	Mu	Hu	Mu
CD45RA	+	N/A	+	N/A	−	N/A	−	N/A	+	N/A
CCR7	+	+	+	+	+	+	−	−	−	−
CD27	+	+	+	+	+	+	+/−	+	+/−	−
CD28	+	N/A	+	N/A	+	N/A	+/−	N/A	−	N/A
CD95	−	N/A	+	N/A	+	N/A	+	N/A	+	N/A
CD44	N/A	−	N/A	+	N/A	+	N/A	+	N/A	+
CD11a	N/A	−	N/A	+	N/A	+	N/A	+	N/A	+
CD122	N/A	−	N/A	+	N/A	+	N/A	−	N/A	−
CD62L	+	+	+	+	+	+	N/A	−	N/A	−
KLRG1	−	−	−	−	−	−	−	−	+	+
TCF1	+	+	+	+	−	−	−	−	−	−

Hu- human; Mu- murine; SCM- stem cell memory; CM- central memory; EM- effector memory; TDE Terminally Differentiated Effectors (also known as TEMRA in human terminology)

**Table 22.** DuraClone Treg panel (Beckman Coulter #B53346)

Marker	Fluorochrome	Clone
CD3	APC-A750	UCHT-1
CD4	PE-Cy7	SFCI12T4D11(T4)
CD25	PE	B1.49.9
FOXP3	AF647	259D
Helios	PacBlue	22F6
CD39	PC5.5	BA54
CD45RA	FITC	2H4LDH11LDB9(2H4)
CD45	KromeOrange	J33
**CD127	APC-AF700	R34.34

** An additional Ab added in liquid form in step 1. Beckman Coulter, #A71116

**Table 23.** Panel for liquid Ab cocktail-based staining of CD25^hi^CD127^lo^FOXP3^+^ Tregs in whole blood

Marker	Dilution	Fluorochrome	Clone	Company	Catalogue
CD3	1/100	BV786	UCHT1	BD	565491
CD4	1/100	AF700	RPA-T4	BD	557922
CD25	1/25	BB515	2A3	BD	564467
CD127	1/50	APC	A019D5	BioLegend	351315
*FOXP3	1/25	PE	259D	BioLegend	320207
*FOXP3	1/25	PE	236A/E7	Invitrogen	12-4777-42

* These FOXP3 clones are equivalent and either can be used in this panel.

**Table 24.** Panel enumeration of Tregs (defined as CD25^hi^CD127^lo^) in whole blood

Marker	Volume	Fluorochrome	Clone	Company	Catalogue
CD3		FITC	SK7		
CD16/56	20 μL BD 6	PE	B73.1/NCAM16.2		644611 or
CD45	colour TBNK	PerCP-Cy5.5	2D1	BD	337166 (with 50 Trucount Tubes)
CD4	cocktail	PE-Cy7	SK3		
CD19		APC	SJ25		
CD8		APC-Cy7	SK1		
CD25	1 μL	BV421	2A3	BD	564033
CD25	1 μL	BV421	M-A251	BD	356114
CD127	1 μL	PE-Dazzle 594	A019D5	BioLegend	351336

**Table 25.** List of Abs used for staining Tregs subsets from PBMCs

Marker	Dilution**	Fluorochrome	Clone	Company	Catalogue
Fixable viability dye	1/1000	eFluor780		eBioscience	65-0865-18
CD4	1/100	V500	RPA-T4	BD	560768
CD25	1/50	PE	2A3	STEMCELL Technologies	60153PE
CD127	1/50	APC-AF700	R3434	Beckman Coulter	A71116
CCR4	1/50	BV605	L291H4	BioLegend	359418
CCR6	1/50	APC	G034E3	BioLegend	353416
CXCR3	1/50	BV421	G025H7	BioLegend	353716
FOXP3	1/50	PE-CY7	236A/E7	Invitrogen	25-4777-42
CD45RA	1/50	BV785	HI100	BioLegend	304140
CD45RO	1/50	FITC	UCHL1	BD	561887

** These dilutions have been optimised for our specific Ab lots. Each new mAb lot should be re-titrated.

**Table 26.** Treg populations in the blood

T cell population	Phenotype	Relative frequency
Total Tregs	CD4^+^CD25^hi^CD127^lo^	~5% of CD4^+^ T cells
Th1 Tregs	CCR6^−^CCR4^−^CXCR3^+^	~5% of CD25^hi^CD127^lo^
Th17 Tregs	CCR6^+^CCR4^+^CXCR3^−^	~30% of CD25^hi^CD127^lo^
Th17.1 Tregs	CCR6^+^CCR4^+^CXCR3^+^	~20% of CD25^hi^CD127^lo^
Th2 Tregs	CCR6^−^CCR4^+^CXCR3^−^	~5% of CD25^hi^CD127^lo^

**Table 27.** List of Abs used for staining Tregs subsets from PBMCs

Marker	Dilution*	Fluorochrome	Clone	Company	Catalogue
Fixable viability dye	1/1000	eFluor780		eBioscience	65-0865-18
CD4	1/50	V500	RPA-T4	BD	560768
	1/25	PE	2A3	STEMCELL Technologies	60153PE
	1/50	PE	4E3	Miltenyi	130-113-282
	1/50	eFluor450	eBioRDR5	eBioscience	48-1278-42
CD127***	1/50	SB436	eBioRDR5	eBioscience	62-1278-42
	1/50	BV421	A019D5	BioLegend	351310
	1/50	BV421	HIL-7R-M21	BD	562436

* These dilutions have been optimised for our specific antibody lots. Each new mAb lot should be re-titrated.

** Specific CD25 antibody clones must be used depending on which CD25-enrichment protocol is used. See “Note” below each CD25-enrichment protocol for further details.

*** The staining profile of seven different CD127 antibodies is provided. Staining panels should be optimised for each machine such that users can accurately gate CD4^+^CD25^hi^CD127^lo^ cells.

**Table 28.** List of Abs used for staining thymocytes or CD25^+^FOXP3^+^ Tregs from thymus

Marker	Dilution**	Fluorochrome	Clone	Company	Catalogue
Fixable viability dye	1/1000	eFluor780		eBioscience	65-0865-18
CD8	1/100	FITC	HIT8a	eBioscience	11-0089-42
CD4	1/50	V500	RPA-T4	BD	560768
CD3	1/100	BV786	UCHT1	BD	565491
CD25	1/100	BV421	BC96	BioLegend	302630
FOXP3	1/50	PE	236A/E7	eBioscience	12-4777-42
Helios	1/25	AF647	22F6	BioLegend	137218

** These dilutions have been optimised for our specific Ab lots. Each new mAb lot should be re-titrated.

**Table 29.** List of Abs used for staining CD25^hi^FOXP3^+^Tregs from intestinal biopsies

Marker	Dilution**	Fluorochrome	Clone	Company	Catalogue
Fixable viability dye	1/1000	eFluor780		eBioscience	65-0865-18
CD3	1/50	V500	UCHT1	BD Biosciences	561416
CD4	1/50	AF700	SK3	BioLegend	344622
CD25	1/25	BV421	BC96	BioLegend	302630
FOXP3	1/25	PECy7	236A/E7	eBioscience	25-4777-42
Helios	1/25	AF647	22F6	BioLegend	137218
CD161	1/25	BV875	DX12	BD Biosciences	744096

**Table 30.** Collagenase VIII compatible clones for human Treg staining. Note that this compatibility may be affected by different tissue digestion protocols

Marker	Validated Clone
CD127	A019D2
LAG3	3D5223H
CD25	BC96
ICOS	C398.4a
CD3	UCHT1
CD4	SK3
CD45	HI30
CD45RA	HI100
CD45RO	UCHL1
CD69	FNSO
CTLA-4	BN13

**Table 31.** Collagenase mix for digestion of human skin tissue

Name	Concentration	Company	Catalogue
Collagenase Type IV	4 mg/ml	Sigma-Aldrich	C5138
DNAse I	10 μg/ml	Roche	11284932001
Fetal bovine serum	2%	N/A	N/A
HEPES	10mM	N/A	N/A
DMEM	Fill up to 10 mL	Gibco	41965

**Table 32.** Collagenase mix for digestion of human fat tissue

Name	Concentration	Company	Catalogue
Collagenase Type II	1 mg/mL	Sigma-Aldrich	C6885
DNAse I	20 μg/mL	Roche	11284932001
Bovine serum albumin	20 mg/mL	Sigma-Aldrich	A4503
HEPES	10mM	N/A	N/A
DMEM	Fill up to 10 mL	Gibco	41965

**Table 33.** List of Abs used for staining CD25^hi^FOXP3^+^Tregs from skin and fat tissue

Marker	Dilution**	Fluorochrome	Clone	Company	Catalogue
Fixable viability dye	1/1000	eFluor780	N/A	eBioscience	65-0865-18
CD3*	1/50	BV786	OKT3	BD Biosciences	566781
CD3*	1/50	BV786	UCHT1	BD Biosciences	565491
CD4	1/50	PE-Cy7	OKT4	BioLegend	317414
CD8	1/25	BV605	RPA-T8	BioLegend	301040
CD19	1/50	APC-Cy7	HIB19	BioLegend	302218
CD25	1/20	PE	BC96	BioLegend	302606
CD25**	1/20	PE	2A3	BD Biosciences	341011
CD127	1/20	APC	A019D5	BioLegend	351342
CD45	1/50	BUV737	HI30	BD Biosciences	748719
CD45RA	1/50	BV510	HI100	BioLegend	304142
CD206	1/100	BUV395	19.2	BD Biosciences	740309
CCR8	1/20	BV421	433H	BD Biosciences	566379

* Clone OKT3 can stimulate T cells, only to be used when cells are analyzed by flow cytometry or sorted for lysis, not recommended for cultivation or resting of target cells

** alternative to BC96

*** These dilutions have been optimized for our specific Ab lots. Each new mAb lot should be re-titrated.

**Table 34.** Fortessa X20 cytometer configuration

	Filters	Mirrors
488 nm Blue Laser	488/10	
	530/30	505 LP
	695/40	685 LP
640 nm Red Laser	670/30	
	730/45	710 LP
	780/60	750 LP
561 nm Yellow-Green Laser	585/51	
	610/20	600 LP
	670/30	635 LP
	710/50	685 LP
	780/60	750 LP
405 nm Violet Laser	450/50	
	525/50	505 LP
	560/40	545 LP
	585/52	570 LP
	610/20	600 LP
	670/30	630 LP
	710/50	685 LP
	780/60	750 LP

**Table 35.** Beckman Coulter Moflo Astrios cell sorter configuration

	Filters	Mirrors
488 nm Blue Laser	488/6	
	510/20	495 DSP
	560/40	525 DLP
	664/22	642 DLP
	710/45	681 DLP
640 nm Red Laser	642/10	
	671/30	650 DSP
	722/40	695 DSP
	795/70	755 DLP
561 nm Yellow-Green Laser	579/16	
	620/29	595 DLP
	664/22	642 DLP
	710/45	681 DLP
	795/70	748 DSP
405 nm Violet Laser	448/59	
	513/26	495 DSP
	579/16	558 DSP
	614/20	595 DLP
	664/22	642 DLP
	710/45	681 DLP
	795/70	748 DLP

**Table 36.** BD Symphony cytometer configuration

	Filters	Mirrors
488 nm Blue Laser	530-30	505 LP
	610-20	600 LP
	670-30	635 LP
	710-50	685 LP
	750-30	735 LP
	810-40	770 LP
640 nm Red Laser	670/30	
	730/45	710 LP
	780/60	750 LP
561 nm Yellow-Green Laser	586-15	570 LP
	610-20	600 LP
	670-30	635 LP
	710-50	685 LP
	780-60	750 LP
405 nm Violet Laser	431-28	410 LP
	525-50	505 LP
	586-15	550 LP
	605-40	595 LP
	677-20	635 LP
	710-50	685 LP
	750-30	735 LP
	810-40	770 LP
355 nm Ultraviolet Laser	379-28	
	450-50	410 LP
	515-30	490 LP
	580-20	550 LP
	605-20	595 LP
	670-25	630 LP
	735-30	690 LP
	810-40	770 LP

**Table 37.** Summary of key functional and phenotypic human Treg markers

Marker	Technical notes*	References
*Functional and/or phenotypic makers of human Tregs*		
IL-10	Difficult to detect in peripheral Tregs. PMA/Ionomycin stimulation for at least 6 hours is required; staining with the IL- 10 capture assay (Miltenyi Biotech) or other stimuli may give better results. Recommended clone is JES3-9D7.	[409-411]
LAP and GARP	Expression is optimal after 24 hours of TCR stimulation.	[412, 413]
LAG-3	Low expression on non-activated Tregs. Recommended clone for activated cells is REA351. Also described as a marker of type 1 regulatory T cells.	[410, 414, 415]
PD-1	Levels are increased after activation or with intracellular staining.	[416]
AREG	Expression is optimal after PMA/Ionomycin stimulation for 4 hours. Works with FOXP3 buffers. Recommended Ab clone is BAF262 (R&D systems).	[408]
CD39	A common single nucleotide polymorphism (SNP) in humans results in low CD39 expression. Individuals who are homozygous for this SNP have <10% CD39^+^ Tregs compared to ~ 50% CD39^+^ in heterozygotes or non-carriers. Clone A1 does not block CD39 enzymatic function.	[417-419]
HLA-DR	Not expressed on murine Tregs; defines a memory and highly suppressive population.	[420]
CTLA-4 (CD152)	Due to rapid receptor internalization, staining is improved with intracellular staining or inclusion of the mAb for a period of time in culture.	[421, 422]
Blimp-1	Expression is increased after activation. Recommended clone is 6D3.	[423]
CD49d	In combination with CD127, low level expression of CD49d can be used to differentiate between activated Tconv and Treg.	[424]
Ki67	Marker of cell proliferation. Although anergic in vitro, when tested directly ex vivo, Tregs show a high proportion of cells expressing Ki67.	[425]
CCR8	Expressed on FOXP3^+^BATF^+^CCR8^+^ Treg cells in peripheral human tissues such as fat, liver or skin.	[378]
*Not known to be detectable on human peripheral blood Tregs*		
CD73	Despite being expressed on murine Tregs, low/no expression of these markers has been observed on non-activated, peripheral human Tregs in healthy individuals. More research is needed to assess if these proteins may be expressed on tissue-infiltrating Tregs, and/or in various disease/cell activation states	[426, 427]
CD103		[360, 428]
ST2 (IL33R)		[387, 408]
Neuropilin-1		[369, 429]
OX40		[430, 431]

* Technical notes are from our experience and largely unpublished.

**Table 38.** Treg cells in the murine thymus

T cell population	Phenotype/subphenotype
**G4:** CD4SP thymocytes	CD4^+^CD8^−^
**G5:** CD25^+^Foxp3^−^ Treg cell precursors	CD4^+^CD8^−^CD25^+^Foxp3^−^
**G6:** CD25^−^Foxp3^+^ Treg cell precursors	CD4^+^CD8^−^CD25^−^Foxp3^+^
**G7:** Thymic Treg cells	CD4^+^CD8^−^CD25^+^Foxp3^+^
**G8:** Immature thymic Treg cells	CD4^+^CD8^−^CD25^+^Foxp3^+^CD69^+^CD24^high^
**G9:** Mature thymic Treg cells	CD4^+^CD8^−^CD25^+^Foxp3^+^CD69^−^CD24^dim/low^
**G10:** Immature thymic CD4^+^ T cells	CD4^+^CD8^−^CD69^+^CD24^high^
**G11:** Mature thymic CD4^+^ T cells	CD4^+^CD8^−^CD69^−^CD24^dim/low^

**Table 39.** T cells in spleen and lymph nodes

T cell population	Phenotype/subphenotype
**G4:** CD4^+^ T cells	CD4^+^CD3ε^+^
**G5:** Tconv cells	CD4^+^CD3ε^+^Foxp3^−^
**G6:** Treg cells	CD4^+^CD3ε^+^Foxp3^+^
**G7:** tTreg cells	CD4^+^CD3ε^+^Foxp3^+^Helios^+^
**G8:** pTreg cells	CD4^+^CD3ε^+^Foxp3^+^Helios^−^
**G9:** effector/memory Treg cells	CD4^+^CD3ε^+^Foxp3^+^CD44^+^CD62L^−^
**G9:** effector/memory Tconv cells	CD4^+^CD3ε^+^Foxp3^−^CD44^+^CD62L^−^

**Table 40.** Treg cells in murine liver and murine spleen

T cell population	Phenotype/subphenotype
**G5:** Liver Tconv cells	CD8^−^CD19^−^MHCII^−^CD4^+^CD3ε^+^CD25^−^Foxp3^−^
**G6:** Liver Treg cells	CD8^−^CD19^−^MHCII^−^CD4^+^CD3ε^+^CD25^+^Foxp3^+^
**G7:** Liver tisTregST2 cells	CD8^−^CD19^−^MHCII^−^CD4^+^CD3ε^+^CD25^+^Foxp3^+^Klrg1^+^ST2^+^Gata-3^+^
**G5:** Spleen Tconv cells	CD8^−^CD19^−^MHCII^−^CD4^+^TCRβ^+^CD25^−^Foxp3^−^
**G6:** Spleen Treg cells	CD8^−^CD19^−^MHCII^−^CD4^+^TCRβ^+^CD25^+^Foxp3^+^
**G7:** Spleen tisTregST2 cells	CD8^−^CD19^−^MHCII^−^CD4^+^TCRβ^+^CD25^+^Foxp3^+^Klrg1^+^ST2^+^Gata-3^+^

**Table 41.** T cells in murine skin

T cell population	Phenotype/subphenotype
**G5:** Skin Tconv cells	CD8^−^CD19^−^MHCII^−^CD4^low^TCRβ^+^CD25^−^Foxp3^−^
**G7:** Skin tisTregST2 cells	CD8^−^CD19^−^MHCII^−^CD4^low^TCRβ^+^CD25^+^Foxp3^+^Klrg1^+^ST2^+^Gata-3^+^

**Table 42.** T cells in fat

T cell population	Phenotype/subphenotype
**G5:** Fat Tconv cells	CD8^−^CD19^−^MHCII^−^CD4^+^TCRβ^+^CD25^−^Foxp3^−^
**G6:** Fat Treg cells	CD8^−^CD19^−^MHCII^−^CD4^+^TCRβ^+^CD25^+^Foxp3^+^
**G7:** Fat tisTregST2 cells	CD8^−^CD19^−^MHCII^−^CD4^+^TCRβ^+^CD25^+^Foxp3^+^Klrg1^+^ST2^+^Gata-3^+^

**Table 43.** T cells in the murine lung

T cell population	Phenotype/subphenotype
**G5:** Lung Tconv cells	CD8^−^CD19^−^MHCII^−^CD4^+^TCRβ^+^CD25^−^Foxp3^−^
**G6:** Lung Treg cells	CD8^−^CD19^−^MHCII^−^CD4^+^TCRβ^+^CD25^+^Foxp3^+^
**G7:** Lung tisTregST2 cells	CD8^−^CD19^−^MHCII^−^CD4^+^TCRβ^+^CD25^+^Foxp3^+^Klrg1^+^ST2^+^Gata-3^+^

**Table 44.** Summary phenotype: T cells in the colon

T cell population	Phenotype/subphenotype
**G5:** Colon Tconv cells	CD8^−^CD19^−^MHCII^−^CD4^+^TCRβ^+^CD25^−^Foxp3^−^
**G6:** Colon Treg cells	CD8^−^CD19^−^MHCII^−^CD4^+^TCRβ^+^CD25^+^Foxp3^+^
**G7:** Colon tisTregST2 cells	CD8^−^CD19^−^MHCII^−^CD4^+^TCRβ^+^CD25^+^Foxp3^+^Klrg1^+^ST2^+^Gata-3^+^

**Table 45.** Key phenotypic differences between human vs murine Treg cells

Phenotype	Murine Treg cells	Human Treg cells
Treg surface protein expression	CD4^+^CD25^+^	CD4^+^CD127^−^CD25^+^
Treg main transcription factor	Foxp3	FOXP3
Surface protein expression of Treg cells with tissue regenerative abilities	CD4^+^CD25^+^Klrg1^+^	
IL33R(St2)^+^Pd1^+^CD127^+^	CD4^+^CD127^−^CD25^+^	
TIGIT^+^CCR8^+^		
Main transcription factor promoting tissue regenerative abilities	Batf [375,376,378]	BATF [378]
Key effector molecules for tissue regeneration	Areg, IL-10, CCN3 [447-451,458,459]	Unknown [378]

**Table 46.** Surface stainings

Marker	Fluorochrome	Clone	Brand
CD3	BUV737	UCHT1	BD Biosciences
CD4	BUV563	SK3	BD Biosciences
CD4	BUV395	RPA-T4	BD Biosciences
CD4	PerCP	RPA-T4	BioLegend
CD4	APC-Fire750	RPA-T4	BioLegend
CD8	BUV805	SK1	BD Biosciences
CD25	APC-R700	2A3	BD Biosciences
CD25	APC-Cy7	CD25	BioLegend
CD49b	FITC	AK7	BD Biosciences
CD49b	Alexa Fluor 647	AK7	BD Biosciences
IL-7R	BB700	HIL-7R-M21	BD Biosciences
IL-7R	BV510	AO19D5	BD Biosciences
LAG-3	PerCP-eFluor710	3DS223H	eBioscience
LAG-3	PE	REA351	Miltenyi
LAG-3 PE	PE	polyclonal	R&D
ICOS	eFluor450	ISA-3	eBioscience
PD-1	BV421	EH12.2H7	BioLegend
CXCR5	PE	FAB190P	R&D
CCR5	FITC	2D7	BD Biosciences
CCR6	BUV496	11A9	BD Biosciences
CCR6	PE-Cy7	R6H1	eBioscience

**Table 47.** Intracellular staining

CD40-L	PE-Cy5	24-31	BioLegend
CD40-L	PE-Vio770	5C8	Miltenyi
EOMES	eFluor660	WD1928	eBioscience
FOXP3	Alexa Fluor 488	259D	BioLegend
Granzyme B	BV421	GB11	BD Biosciences
Granzyme B	FITC	GB11	BD Biosciences
Granzyme K	PE	GM6C3	Santa Cruz
IFN-γ	FITC	4S.B3	BioLegend
IFN-γ	PB	4S.B3	BioLegend
IL-2	APC	MQ1-17H12	eBioscience
IL-10	APC	JES3-9D7	BioLegend
IL-10	PE	JES3-9D7	BioLegend
T-bet	PE-CF594	O4-46	BD Biosciences

**Table 48.** Antibodies and dyes

Epitope	color	clone	dilution	manufacturer	protocol
TCRb	BUV395	H57-597	1:100	BD (Cat 742485)	Surface staining in 14.3
CD4	BV785	RM4-5	1:400	BioLegend (Cat 100551)	Surface staining in 14.4
CD4	BUV737	RM4-5	1:400	BD (Cat 612843)	Surface staining in 14.3
CD8	PE-Cy7	53-6.7	1:400	BioLegend (Cat 100721)	Surface staining in 14.3
CD11c	PE-Cy7	N418	1:400	BioLegend (Cat 117317)	Surface staining in 14.3 and 14.4
CD11b	PE-Cy7	M1/70	1:400	BioLegend (Cat 101215	Surface staining in 14.3 and 14.4
CD41	PE-Cy7	MWReg30	1:400	BioLegend (Cat 133915)	Surface staining in 14.3 and 14.4
NK1.1	PE-Cy7	PK136	1:400	BioLegend (Cat 108713)	Surface staining in 14.3 and 14.4
MHCII	PE-Cy7	M5/114.15.2	1:400	BioLegend (Cat 107629)	Surface staining in 14.3 and 14.4
CD19	PE-Cy7	6D5	1:400	BioLegend (Cat 115519)	Surface staining in 14.3 and 14.4
gdTCR	PE-Cy7	GL3	1:400	BioLegend (Cat 118123)	Surface staining in 14.3 and 14.4
LAG-3	APC	C9B7W	1:100	BioLegend (Cat 125209)	Surface staining in 14.3 and 14.4
CD49b	PE	HMα2	1:100	BioLegend (Cat 103506)	Surface staining in 14.3 and 14.4
CXCR6	BV711	SA051D1	1:200	BioLegend (Cat 15111)	Surface staining in 14.3 and 14.4
Fixable Viability dye	eF506		1:1000	ThermoFisherScientific (Cat 65-0866-14)	Surface staining in 14.3 and 14.4
CellTrace violet			1:500 (2 mM stock)	ThermoFisherScientific (Cat C34557)	Labeling of Responder cells, 14.4
CD3	Biotin	145-2C11	1:200	BioLegend (Cat 100303)	Depletion of T cells, 14.4
CD25	Biotin	PC61	1:400	BioLegend (Cat 102003)	Depletion of Foxp3^+^ Tregs, 14.4

**Table 49.** Buffers and Kits

Buffer/Kit	
PBS/EDTA	PBS supplemented with 2 mM EDTA
flow cytometry buffer	PBS supplemented with 2 mM EDTA and 0,2% FCS
40% Percoll	prepare an isotonic 90% Percoll buffer using 10× PBS and dilute to 40% in flow cytometry buffer
67% Percoll	prepare an isotonic 90% Percoll buffer using 10× PBS and dilute to 67% in flow cytometry buffer
DTT buffer	25 ml HBSS Ca2^+^ and Mg2^+^ free 25 ml 10× HEPES Bicarbonat Buffer (see below) 25 ml FBS 175 ml Aq dest Store at room temperature, refresh every 2 weeks Freshly add 7,7 mg DTE/ 50 ml buffer. Use 10 ml/sample
Digestion media	500 ml RPMI 1640 1% FBS 1× HGPG (see below) 1 mM CaCl2 1 mM MgCl2 Freshly add 0,1 mg Collagenase D and 0,1 mg DNaseI/ml. Use 6 ml/sample
10× HEPES Bicarbonate Buffer	23,8 g HEPES 21 g Sodium Bicarbonat 1 L distilled water Adjust pH7,2 with HCl
100x HGPG	14,9 g HEPES 1×10^6 U Penicillin 2,5 mg Gentamycin 125 ml RPMI+Glutamax Adjust to pH7,5, filter sterile Store at −20°C
ACK lysing buffer	ThermoFisherScientific Cat#A10492-01
Clicks full medium	500 ml Clicks medium (IrvineScientific, 9195) 10% FCS (Gibco, 10500-064) 5 ml GlutaMAX Supplement (Gibco, 13462629) 5 ml Penicillin/Streptomycin (Gibco, 15140-122)
Streptavidin Microbeads	Miltenyi Biotec 130-048-101
CD4 (L3T4) Microbeads, mouse	Miltenyi Biotec 130-117-043

**Table 50.** Flow cytometer settings, LSR Fortessa

Laser	Filter
355 nm	BP 379/28 LP blank;
	BP 740/35 LP 690
405 nm	BP 450/50 LP blank
	BP 525/50 LP 505
	BP 585/42 LP 555
	BP 610/20 LP 595
	BP 660/20 LP 630
	BP 710/50 LP 690
	BP 780/60 LP 750
488 nm	BP 488/10 LP blank
	BP 530/30 LP 505
	BP 710/50 LP 685
561 nm	BP 586/15 LP 570
	BP 610/20 LP 600
	BP 670/30 LP 635
	BP 710/50 LP 685
	BP 780/60 LP 735
640 nm	BP 670/30 LP blank
	BP 730/45 LP 690
	BP 780/60 LP 750

**Table 51.** Summary murine Tr1 cell phenotype

Marker	Relevance for murine Tr1 cell biology
IL-10	Key feature of Tr1 cells, mediates suppression
TGFβ	Mediates suppression
LAG-3	Enriches for IL-10-expressing, suppressive cells
TIM-3	Co-expressed with other co-inhibitory receptors on suppressive IL-10^+^ CD4^+^ T cells
CTLA-4	Co-expressed with other co-inhibitory receptors on suppressive IL-10^+^ CD4^+^ T cells
TIGIT	Co-expressed with other co-inhibitory receptors on suppressive IL-10^+^ CD4^+^ T cells
PD-1	Co-expressed with other co-inhibitory receptors on suppressive IL-10^+^ CD4^+^ T cells
CD49b	Co-expression with LAG-3 identifies Tr1 cells
CD226	Is highly expressed by murine Tr1 cells
ICOS	Promotes IL-27-induced Tr1 differentiation and IL-10 expression in murine CD4^+^ T cells
CCR5	expressed by Tr1 cells
CD39/CD73	Mediates suppressive capacity of Tr1 cells
IL10R	Maintenance of IL-10 expression and suppressive function of Tr1 cells

**Table 52.** Key features of murine and human Tr1 CD4^+^ T cells

Marker	Relevance for Tr1 cell biology	Mouse	Human
Cytokines			
IL-10	Key feature of Tr1 cells, mediates suppression	[409]	[409]
TGFβ	Mediates suppression	[409]	[409]
Co-inhibitory receptors			
LAG-3	Enriches for IL-10-expressing, suppressive cells	[415, 476]	[466]
TIM-3	Co-expressed with other co-inhibitory receptors on suppressive IL-10^+^ CD4^+^ T cells	[466]	[466]
CTLA-4	Co-expressed with other co-inhibitory receptors on suppressive IL-10^+^ CD4^+^ T cells	[466]	[466]
TIGIT	Co-expressed with other co-inhibitory receptors on suppressive IL-10^+^ CD4^+^ T cells	[466]	[466]
PD-1	Co-expressed with other co-inhibitory receptors on suppressive IL-10^+^ CD4^+^ T cells	[466, 465]	[166, 465, 466]
Integrin/Adhesion	Co-expression with LAG-3 identifies Tr1 cells	[415]	[415]
CD49b			
Co-stimulatory receptors			
ICOS	Promotes IL-27-induced Tr1 differentiation and IL-10 expression in murine CD4^+^ T cells; Is expressed by human Tr1 cells	[481, 504]	[505]
CD226	Promotes killing of myeloid cells by human Tr1 cells; Is highly expressed by murine Tr1 cells	[415]	[491]
Chemokine receptors	expressed by Tr1 cells	[465]	[166, 465]
CCR5			
Ectoenzymes			
CD39/CD73	Mediates suppressive capacity of Tr1 cells	[506]	[506, 507]
Granzymes			
Granzyme B	Mediates killing of myeloid cells by human Tr1 cells		[491]
Granzyme K	Expressed by human Tr1 cells		[169]
Cytokine receptors			
IL10R	Maintenance of IL-10 expression and suppressive function of Tr1 cells	[502]	[502]
IL7R	Human Tr1 cells express low IL7R		[505]

**Table 53.** Characteristics of monoclonal Abs to identify human γδ T cell subsets in peripheral blood

*Buffers/Reagents for Flow Cytometry*					
Buffer/Reagent	Provider	City	Country	Catalogue Number	Dilution
Lymphoprep	Axis-Shield PoC	Oslo	Denmark	NA	NA
Zombie Aqua	BioLegend	San Diego, CA	USA	423101	1:500
TruStain FcX	Biolegend	San Diego, CA	USA	422302	1:100
IC Fixation Buffer	eBioscience	San Diego, CA	USA	NA	1:4
Perm Buffer	eBioscience	San Diego, CA	USA	NA	1:10
*Antibodies for Flow Cytometry*					
mMonoclonal Ab	Fluorochrome	Clone	Provider	Catalogue Number	Dilution
anti-CD3	BUV395	UCHT1	BD Biosciences	563546	1:150
anti-CD8	BUV496	RPA-T8	BD Biosciences	564804	1:200
anti-Vγ9	PE-Cy5	IMMU 360	Beckman Coulter	A63663	1:400
anti-CD27	PE-Dazzle 594	M-T271	BioLegend	356422	1:150
anti-CD45RA	BV711	HI100	BioLegend	304138	1:200
anti-CX3CR1	PE-Cy7	2A9-1	BioLegend	341612	1:150
anti-IL-7Rα	BV605	A019D5	BioLegend	351333	1:100
anti-γδTCR	PE	REA591	Miltenyi Biotec	130-114-038	1:200
anti-Vδ1	FITC	REA173	Miltenyi Biotec	130-100-532	1:100
anti-Vδ2	APC	123R3	Miltenyi Biotec	130-095-803	1:200
anti-αβTCR	APC-Vio770	REA652	Miltenyi Biotec	130-114-062	1:100

**Table 54.** Characteristics of mAbs to identify human γδ T cell subsets in peripheral blood

Monoclonal mAb	Clone	Provider	Catalogue Number	Dilution
anti-Granzyme A	CBO9	BioLegend	507221	1:100
anti-Granzyme B	GB11	BioLegend	372209	1:100
anti-Perforin	B-D48	BioLegend	353303	1:80

**Table 55.** Summary phenotype for human γδ T cell subsets

Marker	Vδ1_naive_	Vδ1_effector_	Vγ9/Vδ2^+^ T cells	Vγ9^−^/Vδ2^+^ _naive_	Vγ9^−^/Vδ2^+^ _effector_	Vδ1^−^/Vδ2^−^ T cells
*Surface*						
CCR7	+	−	−	+	−	ND
CD3	+	+	+	+	+	+
CD16	−	+	−/+	?	?	ND
CD27	hi	lo	lo	hi	lo	ND
CD28	+	−	−/+	+	−	ND
CD45RA	lo/hi	lo/hi	−	int	hi	ND
CD62L	+	−	−	?	?	ND
CX_3_CR1	−	+	+	−	+	ND
gdTCR	+	+	+	+	+	+
IL7Ra	+	−	+	+	−	ND
Vd1	+	+	−	−	−	−
Vd2	−	−	+	+	+	−
Vg9	−/+	−/+	+	−	−	ND
*Intracellular*						
Granzyme A	−	+	+	−	+	ND
Granzyme B	−	+	+	−	+	ND
Granzyme K	−	−	+	ND	ND	ND

+ indicates expression, − indicates no expression, hi indicates high expression, lo indicates low expression, int indicates intermediate expression, ND indicates not determined.

**Table 56.** List of commercially available mAb clones directed against murine Vγ and Vδ segments

Target		Clone	Non-exclusicve list of Supplierer(s)
BD/Biolegend nomenclature	Heilig/Tonegawa		
pan γδ TCR		GL3	many sources
pan γδ TCR		UC7-13D5	BD Biosciences, Biolegend
pan γδ TCR		REA633 (recombinant GL3)	Miltenyi
Vγ3	Vγ5	536	Biolegend and BD Biosciences
Vγ1.1/Cr4	Vγ1	2.11	Biolegend, BD Biosciences
Vγ1.1/Vγ1.2	Vγ1/ Vγ2	4B2.9	Biolegend
Vγ2	Vγ4	UC3-10A6	Biolegend, BD Biosciences
	Vγ4	49.2	Biolegend
	Vγ7	F2.67	Biolegend
Vδ6.3		C504.17C	Biolegend
Vδ6.3		8F4H7B7	BD biosciences
Vδ4		GL2	Biolegend and BD Biosciences
Vδ4		REA372 (recombinant GL2)	Miltenyi

**Table 57.** Summary phenotype table. List of markers to define and distinguish murine IFN- γ producing type 1 γδ T cells (Tγδ 1 cells) and IL-17 producing type 17 γδ T cells (Tγδ17 cells)

	Tγδ1 cells	Tγδ17 cells
**TCRVgamma chain surface markers**	Vγ1, Vγ4, Vγ7, Vγ5	Vγ4, Vγ6
CD3	+	+
Tcrβ	−	−
CD4	−	−
CD8	+/−	+
CD44	lo/−	−
CD27	+	−
CCR6	−	+
Ly6C	−	+
**cytokines**		
IL-17	−	+
IFN-γ	+	−
**trancription factors**		
RORγt	−	+
Eomes	+	−

**Table 58.** List of key features and differences of human and murine γδ T cells

	Human γδ T cells	Murine γδ Tcells
**Definition**	CD3^+^ γδTCR^+^ αβTCR^−^ CD4/CD8^+/−^	CD3^+^ γδTCR^+^ αβTCR^−^ CD4/CD8^−^
**Classification according to TCR**	Vδ2 pos or neg	Vγ-chain expression
**Functional classification**	CD27 vs. CD45RA expression	IFN-γ (Tγδ1 cells) vs. IL-17 (Tγδ17 cells) effectors by intracellular cytokine staining or surface marker (CD44, Ly6C, CD27)
**Localization**	Blood: dominant population Vγ9^+^Vδ2^+^ γδ T cells, Tissues: mainly Vδ2^−^ γδ T cells, no γδ T cells in the epidermis	Located in all tissues, enriched at epithelial sites with Vγ5^+^ DETCs as major lymphocyte population in the epidermis
**Correlation between TCR and function**	Under investigation, Vγ9+Vδ2+ cells are phospho-Ag reactive	Tγδ17 cells: Vγ6 or Vγ4, restricted repertoire
		Tγδ1 cells: Vγ4, Vγ1, etc., more diverse repertoire

**Table 59.** Flow cytometry Abs: Isolation and analysis of NKT cells in human blood

Antibody	Manufacturer
BUV395 CD3ε mAb (UCHT1)	BD Biosciences
BUV805 CD14 mAb (M5E2)	BD Biosciences
PE/Dazzle CD19 mAb (HIB19)	BioLegend
FITC or PE Vβ11 mAb (C21)	Beckman Coulter
FITC Vα24 mAb (C15)	Beckman Coulter

a) Antibodies should be individually titrated to ensure optimal staining

**Table 60.** Selection of important markers for human NKT cells

	CD4+ NKT cells	CD8^+^ NKT cells	DN NKT cells
Marker			
TCR Vα24	+	+	+
TCR Vβ11	+	+	+
CD3e	+	+	+
CD7	+	+	+
CD8α	−	+	−
CD8β	−	−	−
CD11a	+	+	+
CD27	+	+	+
CD28	+	+	+
CD56	+/−	+	+
CD62L	lo	−	−
CD127	+	+	+
CD161	+/−	+	+
CD184 (CXCR4)	+	+	+
CD194 (CCR4)	+	+	+
CD195 (CCR5)	+/−	+	+
Transcription factors			
PLZF	+	+	+
T-BET	+/−	+/−	+
Cytokines			
IL-2	+	lo	−
IL-4	+	−/lo	−
IL-13	+	ND	−/lo
IFN-γ	+	+	+
TNF	+	+	+

+ Indicates high expression, − indicates no expression, +/− indicates bimodal expression, lo indicates low expression, ND indicates not determined according to published reports [580, 587, 588, 601-605]

**Table 61.** Selection of important markers for flow cytometry of mouse thymic and human iNKT cells

	Mouse (thymus)								Human		
	Stage 0	Stage 1	Stage 2	Stage 3	NKTp	NKT1	NKT2	NKT17	CD4^+^	CD8^+^	DN
Marker											
TCR Vα24									+++	+++	+++
TCR Vβ11									+++	+++	+++
CD3ε	+++	+++	+++	+++	+++	+++	+++	+++	+++	+++	+++
CD4	−	−	+++	+++		bi	+++	+	+++	−	−
CD7									+++	+++	+++
CD8α									−	+++	−
CD8β									−	−	−
CD11a									+	+	+
CD24	+++	−	−	−	−	−	−	−			
CD27						+++	+++	−	+++	+++	+++
CD28									+++	+++	+++
CD43HG						−	++	+++			
CD44	+	+	+++	+++	−						
CD56									bi	+++	+++
CD62L									+	−	−
CD94			bi	+++		bi	−	−			
CD122 (IL-2RB)						+++	−	−			
CD127 (IL-7R)									+++	+++	+++
CD138						−	−	+++			
CD161 (NKRP1A)									+++	+++	+++
NK1.1 (NKRP1B)	−	−	−	+++	−	+++	−	−			
CD183 (CXCR3)					−	+++	+	−			
CD184 (CXCR4)									+++	+++	+++
CD194 (CCR4)						−	+++	+++	+++	+++	+++
CD195 (CCR5)									bi	+++	+++
CD196 (CCR6)						−	−	+++			
CD197 (CCR7)					+++	−	−	−			
CD278 (ICOS)						+	+++	+++			
CD279 (PD-1)						−	+++	+++			
IL-17RB		bi	+++	bi	−	−	+++	+++			
Ly6C						bi	+	−			
NKG2D						bi	−	−			
NKp46						bi	−	−			
Transcription factors											
PLZF	+++	+++	+++	+++	+++	+	+++	++	+++	+++	+++
T-bet	−	−	+++	bi	−	+++	−	−	bi	bi	+++
RORγt	−	−	−	bi	−	−	−	+++			
Cytokines and effector molecules											
IL-2									+++	+	−
Granzyme B					−	+++	−	−			
IFNγ					−	+++	−	−	+++	+++	+++
TNFα									+++	+++	+++
IL-4					−	+++	+++	+++	+++	−/+	−
IL-13					−	+++	+++	+++	+++	ND	−/+
IL-17A					−	−	−	+++			

+++ Indicates high expression, ++ indicates intermediate expression, + indicates low expression, − indicates no expression, bi indicates bimodal expression. ND indicates not determined according to published reports [580, 587, 588, 601-605].

**Table 62.** Monoclonal used for isolation and analysis of MAIT cells in human blood

Dilution*	Antigen	Clone	Stock Concentration	Fluorophore	Manufacturer
1:50	CD3	UCHT1	100 tests	BUV395	BD Pharmingen
1:50	CD3	UCHT1	100 tests	PE	BD Pharmingen
1:50	CD3	UCHT1	100 tests	AF700	BD Pharmingen
1:100	CD4	SK3	100 tests	BV510	BD Pharmingen
1:100	CD4	SK3	100 tests	APC-Cy7	BD Pharmingen
1:100	CD8	SK1	100 tests	BUV805	BD Pharmingen
1:200	CD8	SK1	100 tests	BV650	BD Pharmingen
1:50	CD14	MϕP9	100 tests	APC-Cy7	BD Pharmingen
1:50	CD19	SJ25C1	100 tests	APC-Cy7	BD Pharmingen
1:50	CD27	O323	100 μg/ml	BV785	Biolegend
1:50	CD161	HP-3G8	100 μg/ml	BV650	Biolegend
1:100	CD161	HP-3G8	100 μg/ml	PE-Cy7	Biolegend
1:50	TRAV1-2	3C10	100 μg/ml	BV711	Biolegend
1:50	TRAV1-2	3C10	100 μg/ml	APC	Biolegend
1:50	TRAV1-2	3C10	200 μg/ml	FITC	Biolegend

* Antibodies should be individually titrated to ensure optimal staining

**Table 63.** Summary of MAIT subpopulations

T cell population	Phenotype/sub-phenotype
**G4:** CD3 cells	CD14^−^ CD19^−^ CD3ε^+^
**G5:** MR1-5-OP-RU^+^ MAIT	CD3^+^ TRAV1-2^+^ MR1-5-OP-RU^+^
**G6:** CD4^+^ MAIT	CD3^+^ TRAV1-2^+^ MR1-5-OP-RU^+^ CD4^+^ CD8^−^
**G7:** CD4^+^ CD8^+^ MAIT	CD3^+^ TRAV1-2^+^ MR1-5-OP-RU^+^ CD4^+^ CD8^+^
**G8:** CD4^−^CD8^−^ MAIT	CD3^+^ TRAV1-2^+^ MR1-5-OP-RU^+^ CD4^−^ CD8^−^
**G9:** CD8^+^ MAIT	CD3^+^ TRAV1-2^+^ MR1-5-OP-RU^+^ CD4^−^ CD8^+^
**G10:** Non TRAV1-2 MR1-5-OP-RU^+^	CD3^+^ TRAV1-2^−^ MR1-5-OP-RU^+^
**G11:** Thymic MR1-5-OP-RU^+^ TRAV1-2 MAIT	CD3^+^ TRAV1-2^+^ MR1-5-OP-RU^+^
**G12:** Immature Stage 1 Thymic MAIT	CD3^+^ TRAV1-2^+^ MR1-5-OP-RU^+^ CD27^−^ CD161^−^
**G13:** Immature Stage 2 Thymic MAIT	CD3^+^ TRAV1-2^+^ MR1-5-OP-RU^+^ CD27^+^ CD161^−^
**G14:** Mature Stage 3 Thymic MAIT	CD3^+^ TRAV1-2^+^ MR1-5-OP-RU^+^ CD27^+/−^ CD161^+^

**Table 64.** Selection of important markers for flow cytometry of human MAIT cells

**Surface Markers**	
TRAV1-2	+
CD3ε	+
CD4	+/−
CD8α	+
CD8β	+/−
CD26	++
CD27	+/−
CD28	+
CD56	+/−
CD94	−
CD101	+/−
CD127 (IL-17R)	+
CD161 (NKRP1A)	++
CD186 (CXCR6)	+
CD195 (CCR5)	+
CD196 (CCR6)	+
CD212 (IL-12R)	+
CD218 (IL-18R)	++
CD226 (DNAM-1)	+/−
CD319 (CRACC)	+
CD328 (Siglec-7)	+
Ly6C	+/−
NKp80	+/−
**Transcription factors**	
Eomes	+
Helios	+
PLZF	+
T-bet	+
RORγt	+
**Cytokines and effector molecules**	
Granzyme B	+
IFNγ	+
IL-2	+
IL-5^#^	+
IL-13^#^	+
IL-17A	+/−
IL-22	+/−
Perforin	+
TNF	+

++ Indicates high expression, + indicates positive expression, − indicates no expression, +/− indicates bimodal expression, ^#^indicates chronically stimulated MAIT cells, according to published reports [643, 680, 696]

**Table 65.** Selection of important markers for flow cytometry of mouse thymic and human MAIT cells

	Mouse (thymus)				Human
	Stage 1	Stage 2	Stage 3 MAIT1	Stage 3 MAIT17	
Marker					
CD3ε	+++	+++	+++	+++	+++
CD4	+++	+++	+++	+++	bi
CD8α	bi	−	bi	bi	+++
CD8β					bi
CD24	+++	−	−	−	
CD26					+++
CD27					bi
CD28			+++	bi	+++
CD43HG			+	+++	
CD44	+	+	+++	+++	
CD56					bi
CD94					−
CD101					bi
CD103			bi	+++	
CD122			+++	−	
CD127 (IL-7R)			+++	+	+++
CD138	−	−	−	+++	
CD161 (NKRP1A)					+++
NK1.1 (NKRP1B)	−	−	+++	−	
CD186 (CXCR6)					+++
CD195 (CCR5)					+++
CD196 (CCR6)					+++
CD197 (CCR7)	−	+++	−	−	
CD212 (IL-12R)					+++
CD218 (IL-18R)	+	+/+++	+++	+++	+++
CD226 (DNAM-1)					bi
CD278 (ICOS)	+		+	+++	
CD319 (CRACC)	−	−	+++	−	+++
CD328 (Siglec-7)					+++
Ly6C	−	−	+	−	bi
NKp80					bi
Transcription factors					
Eomes					+++
Helios					+++
PLZF	+	+	++	+++	+++
T-bet	−	−	+++	−	+++
RORγt	−	−	−	+++	+++
Cytokines and effector molecules					
Granzyme B					+++
IFNγ			+++	−	+++
IL-2					+++
IL-5^#^			−	−	+++
IL-13^#^			−	−	+++
IL-17A			−	+++	bi
IL-22					bi
Perforin					+++
TNF					+++

+++ Indicates high expression, ++ indicates intermediate expression, + indicates low expression, − indicates no expression, bi indicates bimodal expression. ND indicates not determined according to published reports, ^#^ indicates chronically stimulated MAIT cells, according to published reports [643, 680, 696].

**Table 66.** Methods for the detection of Ag-specific T cells

Detection Method	Duration	Commonly used markers	Cell Type	Disadvantages
Proliferation	3-5 days		CD4^+^ and CD8^+^	Bystander proliferation may occur
				Selective outgrowth of single clones
				No direct quantification of specific cells
				Phenotypical and functional changes during long-term in vitro culture
Cytokine secretion	5-12 hours (different cytokines may have different kinetics)	TNF-α	CD4^+^ and CD8^+^	Restricted to producers of the selected cytokine; Non-cytokine producing T cells (e.g., naive, Treg) are neglected
		IFN-γ	CD4^+^ and CD8^+^	
		IL-2	CD4^+^ and CD8^+^	
		IL-4, IL-5, IL-9, IL-10, IL-13, IL-17A, IL-17F, IL-21, IL-22, GM-CSF, etc.	mainly CD4^+^	
		GARP/LAP/TGF-β	Treg	
Activation marker	5 hours - several days (different activation markers have different kinetics)	CD69 (3 till 24 hours) [719]	CD4^+^ and CD8^+^	Sensitive to bystander activation
		CD25 (24 till <72 hours) [719]	CD4^+^ and CD8^+^	Sensitive to bystander activation (IL-2); late up-regulation; constitutively expressed by Treg
		HLA-DR (24 till <72 hours) [719]	CD4^+^ and CD8^+^	Late up-regulation
		CD134 (OX-40) (12 till 72h) [130, 720-723]	CD4^+^ and CD8^+^	Late up-regulation
		CD154 (CD40L) (6 till 16 hours) [198, 724]	mainly CD4^+^	Restricted to CD4^+^ T cells; not expressed on Treg
		CD137 (4-1BB) (6 till 24 hours)	Treg (6h) [475, 725, 726] later also on CD4^+^Tconv and CD8^+^ [727, 728]	Detection Treg requires co-staining with CD154; On CD4^+^ and CD8^+^ Tconv sensitive to bystander activation
		PD-L1 (CD274) (18 till 24 hours) [130, 720-722, 729]	Mainly CD4^+^	Late up-regulation
Cytotoxicity	1-6 hours	Perforin	mainly CD8^+^	Restricted to preselected cytotoxic marker; non-cytotoxic T cells are neglected
		Granzyme A	mainly CD8^+^	
		Granzyme B	mainly CD8^+^	
		CD107a	mainly CD8^+^	
Activated integrins	4-120 min	(ICAM)-1 multimers that specifically bind to activated β2-integrins [712]	So far described for CD8^+^	Restricted to a subset of cytokine secreting / cytotoxic memory T cells; non-functional Ag-specific T cells and naïve cells are neglected

**Table 67.** Listing of reagents and instruments used for Figure 77

	Instrument	Multimer			Antibodies					Buffer
Name	CyAn ADP	Streptavidin	Streptactin	SIINFEKL- MHC (*Strep*-tag)	CD8	CD45.2	CD90.2	CD19	Propidium Iodid	FACS Buffer
Specification/Clone	3Laser (405,488,638nm)	Backbone-nonreversible	Backbone-reversible	pMHC-Monomer	53-6.7	104	53-2.1	6D5	Live/Dead Marker	PBS with 0.5% BSA, 1mM EDTA, 5mM D-Biotin
Vendor	DakoCytomation; Beckman Coulter	Bio Legend	IBA Lifesciences	MIH*	Bio Legend	Bio Legend	Life Technologies	Bio Legend	Life Technologies	Sigma Aldrich
Label	FSC, SSC, 9FL channels	PE	APC	Alexa Fluor 488	PB	PerCP-Cy5.5	APCeF780	PEDazzle594	PI	
Isotype		n.a.	n.a.	n.a.	Rat IgG2ak	Mu IgG2ak**	Rat IgG2ak	Rat IgG2ak	n.a.	

* Institute for Medical Microbiology, Immunology and Hygiene

** No Fc-block; sample material murine blood (ACT Lysed)

**Table 68.** Selection of important pitfalls and top tricks for multimer generation und usage

Theme	Solution	Pitfall	Top Trick
TCR down regulation	Staining at 4°C reduces TCR internalization (only MHC I multimers)		+++
TCR down regulation	Staining at 37°C increases TCR internalization; Accumulation of signal for MHC II staining		+++
TCR down regulation; Dim signal	Use bright fluorophores for best signal-noise ratio (PE; BV421; APC)		+
False positive TCR stain	Multimer double stain with two different conjugated Fluorochromes		++
False positive TCR stain	Staining of unspecific cells (especially B cells via PE conjugated MHC multimer)	++	
False positive TCR stain	Dump channel and live/dead discrimination; Pregating for CD3 and/or CD8/4		++
False positive TCR stain	Certain CD8 mAb clone interfere with MHC binding, leading to unspecific signals	++	
Reliable TCR binding	Multimer binding is not as specific as mAbs; Controls for peptide unrelated MHC-TCR binding	+	
Multimerization level	≥ 3 binding partners are sufficient; questionable improvement by using Pentamers and higher multimerization levels due to unspecific binding	+	++
Reversible Multimer staining	Unaffected T cell function after dissociation of multimers		++
Reversible Multimer staining	TCR-pMHC off-rate measurement		++
Rare cell detection (naïve T cell compartment)	Pre-enrichment methods (MACS, previous enrichment sort)		++
Co-receptor blockade CD8/4	First stain with the multimer for 25min, then apply Ab stain for 20min (second multimer for double discrimination can be included here)	++	+

**Table 69.** Materials for human suppression assay

Reagent	Fluorochrome	Clone	Company	Catalog
LIVE/DEAD^™^ Fixable Near-IR Dead Cell Stain Kit	Near-IR dye	N/A	Invitrogen	L10119
Ficoll-Paque PLUS	N/A	N/A	GE healthcare	17144003
CD4^+^ T-cell isolation kit, human	N/A	N/A	Miltenyi	130-096-533
Leucosep^™^ Centrifuge Tubes	N/A	N/A	Greiner Bio-One	227290
Anti-CD4	V500	RPA-T4	BD	560768
Anti-CD127	AF647	HIL-7R-M21	BD	558598
Anti-CD45RA	BV711	HI100	BioLegend	304138
Anti-CD25	PE	M-A251	BD	555432
Cell Trace^™^ CFSE Cell Proliferation Kit	CFSE	N/A	Invitrogen	C34554
Anti-CD3	N/A	OKT3	BioLegend	317302
RPMI with HEPES and L-glutamine	N/A	N/A	Nacalai Tesque	30263-95
Penicillin and streptomycin	N/A	N/A	Gibco	15140-122
AB serum	N/A	N/A	GemCell	100-512
2-Mercaptoethanol	N/A	N/A	Gibco	21985023

**Table 70.** Materials for the murine suppression assay

Reagent	Fluorochrome	Clone	Company	Catalog
LIVE/DEAD^™^ Fixable Near-IR Dead Cell Stain Kit	Near-IR dye	N/A	Invitrogen	L10119
CD4^+^ T Cell Isolation Kit, mouse	–	N/A	Miltenyi	130-104-454
Cell Trace^™^ CFSE Cell Proliferation Kit	CFSE	N/A	Invitrogen	C34554
Anti-B220*	APC-Cy7	RA3-6B2	BD	552094
Anti-CD11c	APC-Cy7	N418	BioLegend	117323
Anti-CD11b	APC-Cy7	M1/70	BioLegend	101225
Anti-GITR	PE-Cy7	DTA-1	BD	558140
Anti-CD25	APC	PC65.5	eBioscience	17-0251-82
Anti-CD4	V500	RM4-5	BD	560782
Anti-CD3	AF700	14-A2	BioLegend	100216
Anti-CD3	N/A	145-2C11	BD	553057
RPMI 1640 with L-glutamine	N/A	N/A	Nacalai Tesque	30264-85
Penicillin and streptomycin	N/A	N/A	Gibco	15140-122
Fetal bovine serum	N/A	N/A	Gibco	10270-106

**Table 71.** Sequences of self-peptides used for human suppression assay of Ag-specific T cells

Peptide	Sequence
MYH9_478-486_ Non-muscle myosin	QLFNHTMFI
MYH9_741-749_ Non-muscle myosin	VLMIKALEL
VIME_78-87_ Vimentin	LLQDSVDFSL
VIME_225-233_ Vimentin	SLQEEIAFL
ACT_266-274_ Cytoplasmic actin	FLGMESCGI

**Table 72.** Materials for human suppression assay of Ag-specific T cells

Reagent	Fluorochrome	Clone	Company	Catalog
Lympholyte	N/A	N/A	Cedarlane	CL5010
CD8^+^ T Cell Isolation Kit	N/A	N/A	Miltenyi	130-096-495
Naïve CD8^+^ T Cell Isolation Kit	N/A	N/A	Miltenyi	130-093-244
CD4^+^CD25^+^ Regulatory T Cell Isolation Kit	N/A	N/A	Miltenyi	130-091-301
CellTrace^™^ CFSE Cell Proliferation Kit	CFSE	N/A	Thermo Fisher	C34554
CellTrace^™^ Violet Cell Proliferation Kit	Violet	N/A	Thermo Fisher	C34571
FOXP3/Transcription Factor Staining Buffer Set	N/A	N/A	eBioscience	00-5523-00
Viability dye	eFluor780	–	eBioscience	65-0865-18
Anti-CD8	Vio-Green	BW135/80 Fab	Miltenyi	BW-1 35/80
Anti-CD8	Alexa-Fluor488	SK1	BioLegend	344716
Anti-CD8	BV-510	SK1	BioLegend	344732
Anti-CD45RA	BV-605	HI100	BioLegend	304133
Anti-CCR7	PerCP-Cy5.5	G043H7	BioLegend	353220
Anti-GZMB	Bv510	GB11	BD Biosciences	563388
Anti-NKG2D	PECF594	1D11	BD Biosciences	562498
Anti-FOXP3	PerCP-Cy5.5	PCH101	eBioscience	45-4776-42
Anti-CD14	APC-eFluor780	61D3	eBioscience	47-0149-42
Anti-CD16	APC-eFluor780	CB16	eBioscience	47-0168-42
Anti-CD56	APC-eFluor780	CMSSB	eBioscience	47-0567-42
Anti-CD19	APC-eFluor780	HIB19	eBioscience	47-0199-42
Anti-CD3	PE-Cy7	SK7	BioLegend	344816
Anti-CD3	APC	SK7	BioLegend	344812
Anti-CD40L	BV-421	24-31	BioLegend	310823
Annexin V	FITC		BioLegend	640906
PI***			BioLegend	421301
Anti-CD4	Alexa-Fluor488	OKT4	eBioscience	53-0048-42
RPMI 1640 with HEPES	N/A	N/A	Gibco	22409-015
Fetal bovine serum	N/A	N/A	Gibco	10270-106
Human AB serum	N/A	N/A	Euroclone	ECS0219D
Penicillin/streptomycin	N/A	N/A	Gibco	15070-063
l-glutamine	N/A	N/A	Gibco	A29168-01
Non-essential amino acids	N/A	N/A	Euroclone	ECB3054D
Sodium pyruvate	N/A	N/A	Euroclone	ECM0542D

*** PI: Propidium Iodide

**Table 73.** Phenotypic differentiation of human B-lineage cell subsets based on their characteristic expression of surface markers.

B cell population (CD19^+^)		Phenotype/Subphenotype
**Transitional**		
T1^+^T2		CD24^++^CD38^++^CD10^+^CD27-IgM^++^
**Naive**		
Resting		CD24^+/−^CD38^+/−^CD27-IgM^++^/^+^IgD^++^CD21^+^CD95^−^
Activated		CD24-CD38-CD27-IgM^++^IgD^++^CD21-CD95^+^MTG^+^
**Memory** (Ki-67-)		
Pre-switched		IgM^+^IgD^+^/-CD27^+^CD1c^+^
Switched		IgG/IgA^+^CD27^+^CD21^+^
Atypical memory	a) Double negative	IgD-CD27^−^
	b) activated double negative	IgD-CD27-CD95^+^
	c) Syk^++^	IgD^+^/-CD27-CD95^+^/-CD21^+^/-CD38-MTO-Syk^++^
	d) tissue resident	IgM/IgG/IgA^+^CD27-FcRL4^+^
**Marginal Zone**		
Spleen		IgD^+^IgM^+^CD27^++^CD21^++^CD1c^+^
Circulating		IgD^+^IgM^+^CD27^+^CD1c^+^
**Antibody secreting cells**		
Circulating	PB	CD38^++^CD27^++^CD138-Ki-67^+^
	PC	CD38^++^CD27^++^CD138-Ki-67^+^
Bone marrow	a) CD19^+^ PC	CD19^+^CD38^++^CD27^++^CD138^+^Ki-67^−^
	b) CD19- PC	CD19-CD38^++^CD27^++^CD138^+^Ki-67^−^

**Table 74.** Materials

Reagent	Manufacturer
Biocoll	Biochrom, Berlin, Germany
Blood separation filter tube	Dacos, Esbjerg N, Denmark
BD Falcon 70 μM cell strainer	BD Biosciences, Franklin Lakes, NJ, USA
Staining buffer: PBS + 2 mM EDTA + 2% BSA	Sigma, St Louis, MO, USA

**Table 75.** Antibodies and other reagents used for staining

Antibody	Clone	Species reactivity	isotype	Company	Dilution
IgA-AF488	polyclonal	human	polyclonal goat IgG	Jackson Immunoresearch, West Grove PA, USA	1:1500
IgA2-PE	REA995	human	human IgG1	Miltenyi biotec, Bergisch Gladbach, DE	1:100
IgG1-Dylight405*	HP6188	human	mouse IgG2b,k	Sanquin, Amsterdam, NL	1:1000
IgG2-PE/Cy5.5*	HP6014	human	mouse IgG1,k	Sanquin, Amsterdam, NL	1:1000
IgG3-biotin* (followed by Streptavidin-PC7	HP6095	human	mouse IgG1,k	Sanquin, Amsterdam, NL	1:1000
IgG4-APC	SAG4	human	mouse IgG1,k	Cytognos, Salamanca, Spain	1:400
IgM-PerCP/Cy5.5	MHM-88	human	mouse IgG1,k	Biolegend, San Diego, CA, USA	1:100
IgD-PE-CF594	IA6-2	human	mouse IgG2a,k	BD Biosciences, Franklin Lakes, NJ, USA	1:35
CD19-APC/FIRE750	HIB19	human	mouse IgG1,k	Biolegend, San Diego, CA, USA	1:100
CD27-BV510^#^	L128	human	mIgG1,k	BD Biosciences, Franklin Lakes, NJ, USA	1:100
CD38-BV786^#^	HIT2	Human	mIgG1,k	Biolegend, San Diego, CA, USA	1:100
Zombie yellow viability dye	–	–	–	Biolegend, San Diego, CA, USA	1:100
Streptavidin-PC7	–	–	–	Biolegend, San Diego, CA, USA	1:2000

* These Abs were labeled using lightning link labeling kits from Novus Biologicals, Centennial, CO, USA.

# These Abs were included in the staining panel but are not applied in the gating strategy shown in figure 100.

**Table 76.** Summary of human B-cell subpopulation

B cell population (CD19^+^)	Phenotype (all CD19^+^)	Expected frequency range within total CD19^+^ B cells	Expected frequency range within parent population
Non-switched	IgM^+^IgD^+^	40-95%	40-95%
IgA1-switched	IgM-IgD-IgA^+^IgA2^−^	2-10%	10-40%
IgA2-switched	IgM-IgD-IgA^+^IgA2^+^	1-5%	5-20%
IgG1-switched	IgM-IgD-IgA-IgG1^+^	5-10%	40-70%
IgG2-switched	IgM-IgD-IgA-IgG2^+^	0.5-6%	5-30%
IgG3-switched	IgM-IgD-IgA-IgG3^+^	0.2-1%	3-15%
IgG4-switched	IgM-IgD-IgA-IgG4^+^	0.01-1%	0.1-8%

**Table 77.** List of materials used for the analysis of human peripheral B cells

Reagent	Product number	Manufacturer
BD vacutainer 170 I.U. of lithium heparin	367526	BD, Plymouth, UK
Blood separation filter tube	03-7100SI	Dacos, Esbjerg N, Denmark
Biocoll	L 6115	Biochrom, Berlin, Germany
BD Falcon 70 μM cell strainer	431751	BD Biosciences, Franklin Lakes, NJ, USA
Cell culture medium:		
RPMI-1640 Medium, with sodium bicarbonate and L-glutamine	R8758-500ML	Sigma-Aldrich Chemie GmbH
Fetal calf serum (FCS)	F7524	Sigma-Aldrich Chemie GmbH
MEM Vitamin Solution (100x)	M6895-100ML	Sigma-Aldrich Chemie GmbH
Penicillin-Streptomycin (100x)	P4333-100ML	Sigma-Aldrich Chemie GmbH
Kanamycin (100x)	15160-047	GIBCO, Paisley, UK
MEM Non-essential Amino Acid Solution (100x)	M7145-100ML	Sigma-Aldrich Chemie GmbH
Sodium pyruvate solution	S8636-100ML	Sigma-Aldrich Chemie GmbH
TPP^®^ tissue culture plates, 12 well plate, polystyrene	Z707775-126EA	Sigma-Aldrich Chemie GmbH
CpG ODN 2006		MicroSynth, Balgach, Switzerland
Phorbol 12-myristate 13-acetate (PMA)	P-8139	Sigma-Aldrich Chemie GmbH
Ionomycin (iono)	I9657-1MG	Sigma-Aldrich Chemie GmbH
Brefeldin A (BFA)	B7651	Sigma-Aldrich Chemie GmbH
FACS staining buffer:		
PBS		GIBCO, Paisley, UK
2 mM EDTA		
BSA	A3294-500G	Sigma-Aldrich Chemie GmbH, Buchs, Switzerland
Zombie Yellow^™^ Fixable Viability Kit	423104	Biolegend, San Diego, CA, USA
Fixation/Permeabilization Solution Kit	554714	BD Biosciences, Pharmingen, San Diego, CA, USA
Antibodies (clone, dilution):		
CD38 BV785 (HIT2, 1:50)	303530	Biolegend, San Diego, CA, USA
CD27 BV510 (L128, 1:33)	563092	BD Biosciences, Pharmingen, San Diego, CA, USA
CD24 BV421 (ML5, 1:40)	311122	Biolegend, San Diego, CA, USA
CD71 FITC (CY1G4, 1:20)	334104	Biolegend, San Diego, CA, USA
CD25 PE-CF594 (M-A251, 1:40)	562403	BD Biosciences, Pharmingen, San Diego, CA, USA
CD1d PE (51.1, 1:40)	12-0016-42	eBioscience, Affymetrix Inc., San Diego, CA, USA
CD19 APC-Cy7 (HIB19, 1:100)	302218	Biolegend, San Diego, CA, USA
CD73 APC (AD2, 1:40)	344006	Biolegend, San Diego, CA, USA
IL-10 PE-Cy7 (JES3-9D7, 1:200)	501420	Biolegend, San Diego, CA, USA
Isotype ctrl PE-Cy7 (RTK2071, 1:200)	400416	Biolegend, San Diego, CA, USA

**Table 78.** Human B cell subsets: summary of markers, suppressor molecules, induction factors, and related diseases

Name	Marker	Suppressor molecules	Induction	Associated disease
Immature B cells	CD19^+^ CD24^high^ CD38^high^ CD1d^+^	IL-10	CD40 ligation	Suppressive role in RA, SLE and CHB virus infection [997-999]
B10/pro-B10 cells	CD19^+^ CD24^high^ CD27^+^ CD48^high^ CD148^high^	IL-10	CpG-ODN, LPS (+ CD40 ligation)	Various autoimmune diseases [1000].
Br1 cells	CD19^+^ CD73^−^ CD25^+^ CD71^+^ B cells	IL-10, IgG4, PD-L1	CpG-ODN	Allergen tolerance [967]
Suppressive plasmablasts	CD19^+^ CD27^int^ CD38^+^	IL-10, immunoglobulins	CpG-ODN + IL-2, IL-6, and IFN-α/γ	Healthy subjects [970]
Suppressive plasma cells	CD138^+^	IL-10	n/a	Multiple sclerosis lesions [1001]

**Table 79.** Identification of murine B lineage cells in bone marrow

Subpopulation	Marker combination	Ig gene status	References
Pre-pro (Hardy A)	B220^high^ / CD19^neg^ / IgM^neg^ / CD43^pos^	germline configuration	[1015, 1031, 1034, 1037, 1041]
	B220^pos^ / CD19^neg^ / CD43^pos^ / CD93^pos^ / CD24^neg^		[1020, 1029, 1038]
*Pro	CD19^pos^ / B220^pos^ / CD43^pos^ / IgM^neg^ / IgD^neg^		[1031]
	CD19^pos^ / B220^pos^ / CD43^pos^ / CD93^pos^ / CD24^pos^		[1020, 1029, 1038]
Early pro (Hardy B)	CD19^pos^ / B220^pos^ / CD43^pos^ / IgM^neg^ / CD24^pos^ / BP-1^neg^	**D_H_ - J_H_	[1015, 1034, 1037]
Late pro (Hardy C)	CD19^pos^ / B220^pos^ / CD43^pos^ / IgM^neg^ / CD24^pos^ / BP-1^pos^	V_H_ - DJ_H_	[1013, 1015, 1034, 1037]
*Pre (Hardy D)	CD19^pos^ / B220^pos^ / CD43^neg^ / IgM^neg^ / IgD^neg^		[1015, 1031]
	CD19^pos^ / CD43^neg^ / IgM^neg^ / CD93^pos^ / CD24^pos^		[1020, 1029]
***Early pre (pre B I)	B220^pos^ / CD117^pos^ / CD25^neg^	D_H_ - J_H_	[1034, 1037]
***Late pre (pre B II)	B220^pos^ / CD25^pos^	V_H_D_H_J_H_	[1034, 1037]
Large pre	CD19^pos^ / B220^low^ / CD43^neg^ / CD24^pos^ / CD93^neg^ / preBcR^pos^ (VpreB^pos^ / λ5^pos^)	V_H_D_H_J_H_	[1020, 1021, 1029, 1037]
Small pre	CD19^pos^ / B220^pos^ / CD24^pos^ / IgM^neg^	rearrangement of the V_L_-J_L_ starts **	[1020, 1029, 1037]
Immature (Hardy E)	CD19^pos^ / B220^pos^ / CD43^neg^ / IgM^pos^ / IgD^neg^	Ig rearrangement complete	[1023, 1024]
	CD19^pos^ / B220^pos^ / CD24^pos^ / IgM^pos^ / IgD^neg^		[1020, 1029, 1038]
Mature (Hardy F)	CD19^pos^ / B220^pos^ / CD43^neg^ / IgM^pos^ / IgD^pos^		[1031]

* Refers to the Hardy nomenclature.

** D_H_, J_H_, V_H_, are the respective gene segments of the Ig heavy chain.

** V_L_ and J_L_ are the respective gene segments of the Ig light chain.

*** Refers to the Basel nomenclature.

**Table 80.** Identification of murine B lineage cells in spleen

Subpopulation	Marker combination	References
T1	B220^pos^ / CD21^low^/ CD23^low/neg^ / IgD^low/neg^ / IgM^high^	[1043, 1067, 1068]
	B220^pos^ / CD23^neg^ / IgM^high^ / CD93^pos^	[1023]
T2	B220^pos^ / CD21^high^ / CD23^pos^/ IgD^low/neg^ / IgM^high^	[1042]
	B220^pos^ / CD23^pos^ / IgM^high^ / CD93^pos^	[1023]
	B220^pos^ / CD21^high^ / CD23^pos^ / IgD^high^ / IgM^high^	[1067]
T3	B220^pos^ / CD23^pos^ / IgM^low^ / CD93^pos^	[1023]
FO	B220^pos^ / CD21^intmed^ / CD23^high^ / IgD^pos^ / IgM^intmed^	[1040, 1042]
	B220^high^ / CD23^pos^ / IgM^low^ / CD93^neg^	[1041]
	CD19^intmed^ / CD1d^intmed^ / CD23^pos^ / CD43^neg^ / IgM^low^ / IgD^high^ / CD5^neg^	[1054]
MZ	B220^pos^ / CD21^high^ / CD23^low/neg^ / IgD^low/neg^ / IgM^high^	[1040, 1042, 1067]
	CD9^pos^ / CD21^pos^ / CD23^neg^ / IgD^low^ / IgM^high^	[1041]
	CD19^intmed^ / CD1d^high^ / CD21^high^ / CD23^neg^ / CD43^neg^ / IgM^high^ / IgD^low^ / CD5^neg^	[1054, 1069]
B-1	IgM^high^ / CD43^pos^ / IgD^low/neg^ / CD23^low/neg^	[1041]
	CD19^pos^ / B220^low^ / CD43^pos^ / CD23^neg^ / CD93^neg^	[1025, 1070]
B-1a	CD19^pos^ / IgM^pos^ / CD43^pos^ / CD5^pos^	[1071, 1072]
	CD19^high^ / CD1d^intmed^ / CD23^neg^ / CD43^pos^ / IgM^high^ / IgD^low^ / CD5^pos^	[1054]
B-1b	CD19^pos^ / IgM^pos^ / CD43^pos^ / CD5^neg^	[1071]
	CD19^high^ / CD1d^intmed^ / CD23^neg^ / CD43^pos^ / IgM^high^ / IgD^low^ / CD5^neg^	[1054]
Memory	antigen^pos^, CD73^low^ / CD80^low^ / CD273^low^ antigen^pos^, CD73^hi^ / CD80^hi^ / CD273^hi^ antigen^pos^, CD73^hi^ / CD80^low^ / CD273^low^ antigen^pos^, CD73^low^ / CD80^hi^ / CD273^hi^ *antigen^pos^, CD73^hi^ / CD80^low^	[1046-1051]
GC	CD19^pos^ / B220^pos^ / CD38^neg^ / GL7^pos^	[1073, 1074]
	B220^pos^ / GL7^pos^ / Fas^pos^	[1042]
	B220^pos^ / GL7^pos^ / PNA^pos^	[1075]

* antigen^pos^ = cells are specific for the immunizing (memory) Ag

**Table 81.** Identification of murine regulatory B cell subsets^a)^

Subpopulation	Marker combination	References
Breg	CD5^pos^ / CD19^high^ / CD1d^high^ / CD21^high/intmed^ / CD23^pos/neg^ / CD43^neg^ / IgM^high^ / IgD^low/intmed^	[1054]
T2-MZP	CD19^pos^ / CD21^high^ / CD23^high^ / IgM^high^ / CD24^high^ / CD1d^high^ /IL-10 ^pos^	[965, 975 977]
MZ	CD19^pos^ / CD21^high^ / CD23^neg^ / IL-10^pos^	[980, 981]
B10	CD5^pos^ / CD1d^high^ / CD19^pos^ / IL-10^pos^	[1056, 1061, 1064]
Tim-1^+^ cells	Tim-1^pos^ / CD19^pos^ / IL-10^pos^	[993, 1065]
Plasma cells	CD138^pos^ / B220^pos^ / MHC-II^low^ / IL-10^pos^	[1062, 1063]
Plasmablasts	CD138^pos^ / CD44^high^ / IL-10^pos^	[970]
Natural regulatory PCs	CD19^pos/neg^ / B220^neg^ / LAG-3^pos^ / CD138^high^ / IL-10^pos^	[936, 1066]

a) including “regulatory plasma cells”

**Table 82.** Selection of important surface markers of murine and human B cells

	Mouse					Human				
	Naive	Memory	MZ	Plasma Blast	Plasma Cell	Naive	Memory	MZ	Plasma Blast	Plasma Cell
Marker										
B220	+	+	+	+/−	−	NA	NA	NA	NA	NA
CD19	+	+	+	+/−	−	+	+	+	+	+/−
CD21	+	ND	+	ND	ND	+	+	+	+	low
CD27	ND	ND	ND	++	++	−	+	+	++	++
CD38	+	+/−	ND	+/−	+/−	+	−	ND	++	++
CD138	−	−	−	++	++	−	−	−	+/−	+/++
IgD	+	−	low	ND	ND	+	−	low	ND	ND
IgM	low	+/−	+	+/−	+/−	+	+/−	+	+/−	+/−

++ indicates very high expression, + indicates high expression, +/− indicates bimodal expression, low indicates low expression NA indicates not applicable, ND indicates not determined

**Table 83.** Antibodies used for the detection of PB/PC in human PBMC

Antigen	Fluorochrome	Clone	Supplier
anti-CD19	PE-Cy7	SJ25C1	BD Biosciences
anti-CD27	Cy5	2E4	Sanquine, labeled in house
anti-CD20	PerCp	L27	BD Biosciences
anti-CD14	Pacific Blue	M5E2	BD Biosciences
anti-CD3	Pacific Blue	UCHT1	BD Biosciences
anti-CD38	FITC	HIT2	BD Biosciences
anti-CD138	PE	B-B4	Miltenyi Biotech

**Table 84.** Antibodies and reagents used for the detection of RBD-specific PB/PC in human PBMC

Antigen	Fluorochrome	Clone	Supplier
anti-CD38	BV421	HIT2	Biolegend
anti-CD45	StarBright Violet 515	F10-89-4	Bio-Rad
anti-IgM	BV570	MHM-88	Biolegend
anti-CD62L	BV605	DREG-56	Biolegend
anti-CXCR3	BV650	G025H7	Biolegend
anti-IgA	PE	REA1014	Miltenyi
anti-HLA-DR	PerCp	L243	Biolegend
anti-CD19	PE-Cy7	HIB19	Biolegend
anti-CD27	AF700	O323	Biolegend
anti-CD3	APC/Fire 750	UCHT1	Biolegend
anti-CD14	APC/Fire 750	63D3	Biolegend
efluor780	–	–	Thermo Fisher Scientific
Streptavidin	APC	–	Biolegend
Streptavidin	PE/Dazzle 594	–	Biolegend
Biotinylated RBD			
Arg319-Phe541 (Accession # QHD43416.1)	–	–	Biolegend
Brilliant Violet Staining Buffer	–	–	BD Biosciences

**Table 85.** Antibodies used for the detection of PB/PC in human BM cell suspension

Antigen	Fluorochrome	Clone	Supplier
anti-CD3	VioBlue	BW264/56	Miltenyi
anti-CD16	VioBlue	REA423	Miltenyi
anti-CD38	BV 510	HIT2	Biolegend
anti-CD138	FITC	44F9	Miltenyi
anti-CD45	PE	HI30	Biolegend
anti-HLA-DR	PerCP	L243	Biolegend
anti-CD19	PE-Cy7	HIB19	Biolegend
anti-CD10	APC/Fire 750	HI10a	Biolegend
anti-CD14	APC/Fire 750	M5E2	Biolegend

**Table 86.** Selection of important markers for flow cytometry analysis of human Ab-secreting plasmablasts and plasma cells

Marker	Category	Blood PB/PC	BMPC
CD19	Cell-surface, identification	Expressed at levels comparable to other B cells, diminished expression in certain conditions	Bimodal expression pattern
CD20		Negative or low	Negative or low
CD27		High expression	High expression
CD38		Very high expression	Very high expression
CD138		Some PB/PC express CD138	CD138low and CD138hi PC can be detected
Intracellular Ig	Intracellular, detection, Ig subclass expression	Most PB/PC in steady state express icIgA	icIgG^+^ and icIgA^+^PC dominate
HLA-DR	maturation	HLA-DR^high^ PB and HLA-DR^low^ PC are distinguishable	Most PC in BM express low levels of HLA-DR, few HLA-DR^high^ cells may comprise contaminating PB from blood
Ki-67	proliferation	Most PB express high levels of Ki67	Most PC lack Ki-67 expression
CD62L	Homing	Differential expression	Not expressed
Beta7 integrin		Differential expression	Differential expression
CCR9, CCR10		Differential expression	CCR10 is differentially expressed

**Table 87.** Selection of important markers for flow cytometry analysis of human normal and aberrant plasma cells

Plasma cell population	Frequent phenotype
Normal	Positive: CD38, CD138, CD319 (SLAMF7), CD19, CD45, CD27, CD81
	Negative: CD56
	Immunoglobulin light chain restriction: none
Aberrant	Positive: CD38, CD138, CD319 (SLAMF7), CD56
	Negative: CD19, CD45, CD27, CD81
	Immunoglobulin light chain restriction: yes

Summary according to published reports [1165, 1178]:

**Table 88.** Antibodies used for flow cytometry

Antigen	Fluorochrome	Supplier	Clone	Identifier
B220 (CD45R)	BV421	BioLegend	Ra3-6b2	103251
B220 (CD45R)	PerCP/Cy5.5	ThermoFisher	Ra3-6b2	103236
CD16/32	unlabeled	eBioscience	93	14-0161-86
CD19	APC/Fire750	BioLegend	6D5	115558
CD98	PE	BioLegend	RL388	128207
CD138 (Sdc1)	PE/Cy7	BioLegend	281-2	142514
CD138 (Sdc1)	BV421	BioLegend	281-2	142507
Ly6-C	PerCP/Cy5.5	ThermoFisher	HK1.4	45-5932-82
Sca-1 (Ly6-A/E)	APC/Cy7	BioLegend	D7	108125
TACI (Tnfrsf13b)	APC	ThermoFisher	eBio8F10-3	17-5942
TACI (Tnfrsf13b)	PE	ThermoFisher	eBio8F10-3	12-5942

**Table 89.** Phenotype summary of marker expression in murine plasma cell subpopulations described in [1028]

Markers	Subsets		
	dividing plasma-blasts P1	early plasma cells P2	mature plasma cells P3
CD138	+	+	+
TACI	+	+	+
CD19	+	+	neg/low
B220	+	neg/low	neg/low
Blimp1:GFP	+	++	+++
Ki67	60-70% +	10-30% +	4-8% +

**Table 90.** Summary phenotype table noting the key differences between murine and human plasma cells

Marker	Mouse	Human
CD38	low-high	High
CD27	–	High
Sca-1	int-high	–
Ly6C	low-high	–
CD138	high	low-high

**Table 91.** Fluorescently labeled monoclonal Abs specific for proteins that undergo phosphorylation and for certain phosphorylation sites of those proteins used to measure signal transduction pathways. Per staining pick one combination of PE and FITC labeled Ab pair

Protein (phosphorylation site)	Conjugate	Manufacturer	Clone	Isotype	BD Phosflow Perm Buffer
Syk	FITC	BD	4D10	mouse IgG2a, k	II
pSyk(Y^352^)	PE	BD	17A/P-ZAP70	mouse, IgG1, k	II
or					
PLCγ2	PE	BD	K86-1161	mouse IgG1, k	II
pPLCγ2(Y^759^)	AF488	BD	K86-689.37	mouse IgG1, k	II
or					
Btk	PE	BD	53/BTK	mouse IgG2a, k	III
pBtk(Y^223^)	FITC	BD	N35-86	mouse IgG1, k	III
or					
Akt1	PerCp-Vio700	Miltenyi Biotec	REA134	rHu	III
pAkt(S^473^)	PE	BD	M89-61	mouse, IgG1, k	III
or					
pERK1/2 (T^202^/Y^204^)	PE	BD	20A	mouse IgG1	III

**Table 92.** Flow cytometry resources for human ILCs

Reagent or resource	Source	Identifier
Fixable Viability Dye (LD) eF780	ThermoFisher	Cat. No. #65-0865-18
APC-eF780 anti-human CD14 (61D3)	eBioscience	Cat. No. #47-0149-42; RRID:AB_1834358
APC-eF780 anti-human CD19 (HIB19)	eBioscience	Cat. No. #47-0199-42; RRID:AB_1582230
APC-eF780 anti-human CD3 (SK7)	eBioscience	Cat. No. #47-0036-42; RRID:AB_10717514
APC-eF780 anti-human CD123 (6H6)	eBioscience	Cat. No. #47-1239-42; RRID:AB_2573972
APC-Vio770 anti-human CD141 (BDCA3) (AD5-14H12)	Miltenyi Biotec	Cat. No. #130-100-217; RRID: AB_2661185
APC-Vio770 anti-human FcεRIα (CRA1)	Miltenyi Biotec	Cat. No. #130-105-457; RRID:AB_2660607
APC-Vio770 anti-human CD11c (MJ4-27G12)	Miltenyi Biotec	Cat. No. #130-100-238; RRID: AB_2660146
eFluor660 anti-human Eomes (WD1928)	eBioscience	Cat. No. #50-4877-41; RRID:AB_2574228
PE anti-human T-bet (eBio4B10)	eBioscience	Cat. No. #12-5825-82; RRID:AB_925761
PE anti-human GATA3 (TWJA)	eBioscience	Cat. No. #12-9966-42; RRID: AB_1963600
APC anti-human RORγt (AFKJS-9)	Invitrogen	Cat. No. #17-6988-82; RRID:AB_2653366
FITC anti-human CD127 (MB15-18C9)	Miltenyi Biotec	Cat. No. #130-094-888; RRID AB_10831019
PE-Dazzle594 anti-human CD56 (HCD56)	Miltenyi Biotec	Cat. No. #318348; RRID AB_2563564
PE-Vio770 anti-human NKp44 (2.29)	Miltenyi Biotec	Cat. No. #130-104-195; RRID: AB_2660328
BV605 anti-human CD117 (104D2)	Biolegend	Cat. No. # 313218; RRID:AB_2562025
PerCPCy5.5 anti-human CRTH2 (BM16)	Biolegend	Cat. No. # 350116; RRID: AB_2562467
Pacific Blue anti-human CD94 (XA185)	In-house	N/A
Transcription factor staining buffer set	eBioscience	Cat. No. #00-5523-00
LSR Fortessa	BD	https://www.bdbiosciences.com/eneu/instruments/research-instruments/research-cell-analyzers/lsrfortessa
FlowJo^™^ Software v10.6.2	FlowJo LLC	https://www.flowjo.com/solutions/flowjo

**Table 93.** Selection of important markers for flow cytometry analysis of human ILCs

Marker					
	NK cells	CD127^+^ ILC1	ILC2	NCR^−^ ILC3	NCR^+^ ILC3
CD127	lo/−	+	+	+	+
CD117	lo/−	−	+/−	+	+
CD25	+/−	Lo	+	+/−	lo
IL-23R	lo	+/−	lo	+	+
IL-17RB	−	lo/−	+	ND	−
ST2	−	ND	+	ND	−
IL-1R1	+/−	lo/−	lo	+	+
CCR6	−	+	+	+	+
RANKL	−	ND	ND	+	+
CRTH2	−	−	+	−	−
ICOS	−	−	+	ND	+
CD161	+/lo	+	+	+	+
CD56	+	−	−	+/−	+/−
CD94	+/−	−	−	−	−
CD16	+/−	−	−	−	−
NKp30	+	ND	+/lo	+/−	+
NKp44	+^a^	−	−	−	+
NKp46	+	−	−	+/−	+
KIR	+/−	−	−	−	−
CD57	+/−	ND	ND	ND	ND
Perforin	+	−	−	−	−
**Transcription factors**					
T-bet	+	lo/+	−	−	−
Eomes	+	−	−	−	−
RORγt	−	−	−	+	+
GATA3	lo/−	lo/−	+	lo/−	lo/−
**Cytokines**					
IFNγ	+	+	−	−	−
IL-22	−	−	lo	lo/−	+
IL-17	−	−	−	+	−
IL-13	lo	−	+	−	lo
IL-5	−	−	+	−	−

+ indicates high expression, − indicates no expression, +/− indicates bimodal expression, lo indicates low expression, a indicates expression on activated cells, ND indicates not determined, NA indicates not applicable according to published reports [1245-1248, 1257-1260, 1262, 1275-1294].

**Table 94.** Tissue dissociation reagents used for murine ILCs

Reagent or resource	Source	Identifier
Miltenyi Biotec Si LPL Kit	Miltenyi Biotec	Cat. No. #130-097-410
HBSS-/−	Gibco	Cat. No. #14065-056
HBSS+/+	Gibco	Cat. No. #14065-056
HEPES	Sigma	Cat. No. #54457
DTT	ThermoFisher	Cat. No. #R0861
FCS	ThermoFisher	Cat. No. #16010159
EDTA	Sigma	Cat. No. #PHR2586
Percoll^®^	GE Healthcare	Cat. No. #17-0891-01
PBS/BSA	In house	N/A
Gentle MACS^™^	Miltenyi Biotec	Cat. No. #130-096-427

**Table 95.** Flow cytometry resources for murine ILCs

Reagent or resource	Source	Identifier
Fixable Viability Dye (LD) eF780	ThermoFisher	Cat. No. #65-0865-18
APC-Vio770 anti-mouse B220 (RA3-6B2)	Miltenyi Biotec	Cat. No. #130-102-267; RRID:AB_2660451
APC-Fire780 anti-mouse F4/80 (BM8)	BioLegend	Cat. No. #123116; RRID:AB_893481
APC-Fire780 anti-mouse Gr-1 (RB6-8C5)	BioLegend	Cat. No. #108456; RRID:AB_2616737
APC-Cy7 anti-mouse FcεRIα (MAR-1)	BioLegend	Cat. No. #134326; RRID:AB_2572064
APC-Cy7 anti-mouse CD11b (M1/70)	BioLegend	Cat. No. #101226; RRID:AB_830642
APC-Cy7 anti-mouse CD11c (N418)	BioLegend	Cat. No. #117324; RRID:AB_830649
V500 anti-mouse CD45 (30-F11)	BD	Cat. No. #561487; RRID:AB_10697046
PerCP-Cy5.5 anti-mouseTCRβ (H57-597)	BioLegend	Cat. No. #109228; RRID:AB_1575173
PerCP-eFluor710 anti-TCRγδ (GL-3)	ThermoFisher	Cat. No. #46-5711-82; RRID:AB_2016707
BV605 anti-mouse NKp46 (29A1.4)	BioLegend	Cat. No. #137619; RRID:AB_2562452
PE-Cy7 anti-mouse CD4 (RM4-5)	BioLegend	Cat. No. #100528; RRID:AB_312729
BV785 anti-mouse CD127 (A7R34)	BioLegend	Cat. No. #135037; RRID:AB_2565269
PE anti mouse/human GATA3 (REA174)	Miltenyi Biotec	Cat. No. #130-123-748; RRID:AB_2857629
AlexaFluor647 anti-mouse T-bet (4B10)	BioLegend	Cat. No. #644804; RRID:AB_1595466
BV421 anti-mouse RORγt (Q31-378)	BD	Cat. No. #562894; RRID:AB_2687545
A488 anti-mouse Eomes (Dan11mag)	ThemoFisher	Cat. No. #53-4875-82; RRID:AB_10854265
anti-mouse CD16/32 (2.4G2)	In-house	N/A
Transcription Factor Staining Buffer Set	ThermoFisher	Cat. No. #00-5523-00
LSR Fortessa	BD	https://www.bdbiosciences.com/eneu/instruments/research-instruments/research-cell-analyzers/lsrfortessa
FlowJo^™^ Software v10.6.2	FlowJo LLC	https://www.flowjo.com/solutions/flowjo

**Table 96.** Selection of important markers for flow cytometry analysis of mouse ILCs

Marker					
	NK cells	CD127^+^ ILC1	ILC2	NCR^−^ ILC3	NCR^+^ ILC3
CD127	−	+	+	+	+
CD117	lo/−	−	lo	+	lo
CD25	−	lo	+	+	ND
IL-23R	−	lo/−	ND	+	+
IL-17RB	−	−	+	−	−
ST2	−	−	+/−	−	−
IL-1R1	−	lo	ND	+	+
CCR6	−	−	−	+/−	−
CD4	−	−	**−**	+/−	−
CXCR5	−	−	−	+/−	−
ICOS	−	ND	+	ND	+
NK1.1	+	+	−	−	lo/−
CD16	+/−	−	−	−	−
NKp46	+	+	−	−	+
Ly49	+/−	lo	−	−	−
CD27	+/−	+	−	−	−
CD11b	+/−	−	−	ND	ND
Perforin	+	lo	−	−	−
CD49b (DX5)	+	−	−	−	−
CD49a	−	+	−	+/−	+
CD200R1	−	+	+	+	+
Transcription factors					
T-bet	+	+	−	+/−	+
Eomes	+	−	−	−	−
RORγt	−	−	−	+	+/−
GATA3	−	lo	+	lo	lo
Cytokines					
IFNγ	+	lo/+	−/lo	−/lo	−/lo
IL-22	−	−	−	+	+
IL-17	−	−	−	+/−	−
IL-13	−	−	+	−	−
IL-5	−	−	+	−	−

+ indicates high expression, − indicates no expression, +/− indicates bimodal expression, lo indicates low expression, a indicates expression on activated cells, ND indicates not determined, NA indicates not applicable according to published reports [1245-1248, 1257-1260, 1262, 1275-1294, 1297-1301.

**Table 97.** Human NK cell inhibitory and activating receptors

	Receptor	Ligand	CD56^bright^	CD56^dim^
Activation	NKG2C (CD159a)	HLA-E	–	subsets
	NKG2D (CD314)	MIC-A - MIC-B - ULBPs	All PB NK cells	
	KIR2DS1 (CD158h)	HLA-C2	–	subsets
	KIR2DS2/3 (CD158j)	???	–	subsets
	KIR2DL4 (CD158d)	HLA-G	–	subsets
	KIR2DS4 (CD158i)	HLA-A*11 and some HLA-C	–	subsets
	KIR2DS5 (CD158f)	???	–	subsets
	KIR3DS1 (CD158e1)	HLA-Bw4, HLA-F	–	subsets
	NKp30 (CD337)	B7-H6 - BAG6/BAT3	++	+
	NKp44 (CD336)	21spe-MLL5, Nidogen 1	On activated NK cells	
	NKp46 (CD335)	CFP (properdin), haemagglutinin, PfEMP1	++	+
	NKp80	AICL	+	+
	DNAM1 (CD226)	Nectin-2 (CD112), PVR (CD155)	+	+
	2B4 (CD244)	CD48	All PB NK cells	
	NTB-A (CD352)	NTB-A (CD352)	All PB NK cells	
	CRACC/CS1 (CD319)	CRACC/CS1 (CD319)	All PB NK cells	
	Tactile (CD96)	PVR (CD155)	All PB NK cells	
	FcγRIII (CD16)	IgG	−/+	+/++
Inhibition	NKG2A/KLRD1 (CD159a/CD94)	HLA-E	+	subsets
	KIR2DL1 (CD158a)	HLA-C2	−	subsets
	KIR2DL2/3 (CD158b)	HLA-C1, few HLA-C2	−	subsets
	KIR2DL4 (CD158d)	HLA-G	−	subsets
	KIR2DL5 (CD158f)	???	−	subsets
	KIR3DL1 (CD158e1)	HLA-A-Bw4 and HLA-B-Bw4	−	subsets
	KIR3DL2 (CD158k)	HLA-A*03 and *11	−	subsets
	ILT2/LIR-1 (CD85J)	Different MHC-I alles	−	subsets
	PD-1 (CD279)	PDL1 (CD274) and PDL2 (CD273)	−	subsets
	Siglec-7 (CD328)	Ganglioside DSGb5	Most of PB NK cells	
	IRP60 (CD300a)	α-herpes virus Pseudorabide virus Phosphatidylserine Phosphatidylethnolamine	+	+
	TIGIT	PVR (CD155)	PB NK cells	

**Table 98.** Other human NK cell receptors

	Receptor	Ligand	CD56^bright^	CD56^dim^
Adhesion	LFA-1 (CD11a/CD18)	ICAM-1, ICAM-2, ICAM-3	−/+	++
	LFA-2 (CD2)	CD15, CD58, CD59	Most of mature NK cells	
	LFA-3 (CD58)	CD2, CD48, CD58	Most of mature NK cells	
	MAC-1 (CD11b/CD18)	iC3b, C4b, ICAM-1, fibrinogen	Most of circulating NK, up-regulated upon activation	
	ICAM-1 (CD54)	LFA-1, MAC-1	++	+/−
	N-CAM (CD56)	???, FGFR	++	+
	HNK-1 (CD57)	???	−	subsets
	L-Selectin (CD62L)	GLyCAM-1 MadCAM-1	++	subsets
Cytokine/Chemokine receptors	IL-2Rα (CD25)	IL-2	+	−
	IL-2Rβ/IL-2Rγ (CD122/CD132)	IL-2 AND IL.15	Almost all PB NK cells	
	c-Kit (CD117)	SCF (KL)	+	−
	IL7Rα (CD127)	IL-7	+	−
	CXCR1 (CD181)	CXCL8 (IL-8)	−	+
	CXCR2	IL8-RB	−	+
	CXCR3 (CD183)	CXCL9, CXCL10, CXCL11	++	Subsets
	CXCR4 (CD184)	CXCL2	Subsets of PB NK cells	
	CCR5 (CD195)	RANTES, CCL3 (MIP1α) and CCL4 (MIP1β)	Subsets of PB NK cells	
	CCR7 (CD197)	CCL19, CCL21	+	−
	IL-18R (CD218a)	IL-18	++	+
	ChemR23	Chemerin	−	+
	CX3CR1	Fraktaline	−	+
Death Receptors	Fas/APO-1 (CD95)	Fas ligand (CD95L)	Activated NK cells They induce target apoptosis	
	Fas ligand (CD95L)	Fas/APO-1 (CD95)		
	CD40L (CD154)	CD40		
	TRAIL (CD253)	DR4 (TRAIL-R1), DR5 (TRAIL-R2)		
Other surface molecules	LAMP1 (CD107a)	—	Briefly expressed on NK cell surface after degranulation	
	LAMP2 (CD107b)	—		
	LAMP3 (CD63)	—		
	TNFRSF7 (CD27)	CD70	+	−

**Table 99.** Human- and mouse-specific and shared NK cell receptors

Receptor	Ligand	Human	Mouse
KIR2DL1 (CD158a)	Group 2 HLA-C	X	
KIR2DL2, KIR2DL3 (CD158b1, -b2)	Group 1 HLA-C, some group 2 HLA-C and some HLA-B	X	
KIR3DL1 (CD158e1)	HLA-Bw4	X	
KIR2DS1 (CD158h)	HLA-C2	X	
KIR2DS4 (CD158i)	Some HLA-C1 and HLA-C2, HLA-A11	X	
KIR2DL4 (CD158d)	HLA-G	X	
Ly49A (Klra1)	H2-Dd, H2-Dk, H2-Ld, H2-Db, H2-Kb, H2-Dp, H2-M3		X
Ly49C (Klra3)	H2-Db, H2-Kb, H2-Dd, H2-Kd, H2-Dk		X
Ly49E (Klra5)	Urokinase plasminogen		X
Ly49G (Klra7)	H2-Dd, H2-Kd, H2-Ld, H2-Db, H2-Dk, H2-Dr		X
Ly49I (Klra9)	H2-Kb, H2-Kd, H2-Dk, H2-Kk, m157 (MCMV)		X
Ly49D (Klra4)	H2-Dd, H2-Dr, Dsp2		X
Ly49H (Klra8)	m157 (MCMV)		X
Ly49P (Klra16)	H2-Dd, H2-Dk, m04 (MCMV)		X
NKG2A (CD159A)/CD94	HLA-E (human), Qa-1b (mouse)	X	X
NKG2C (CD159C)/CD94,	HLA-E (human), Qa-1b (mouse)	X	X
NKG2D (CD314)	Human: MICA/B, ULBP1, ULBP2, ULBP3, ULBP4, ULBP5, ULBP6 Mouse: RAE-1a, RAE-1b, RAE-1d, RAE-1e, RAE-1g, H60a, H60b, H60c, MUTL1	X	X
KLRG1	E-, N-, and R-cadherin	X	X
NKp46 (NCR1; CD335)	CFP (properdin), hemagglutinin, PfEMP1	X	X
NKp30 (NCR3; CD337)	B7-H6, BAT3	X	
NKp44 (NCR2; CD336)	21spe-MLL5	X	
NKp80 (KLRF1)	AICL (activation-induced C-type lectin)	X	
DNAM-1 (CD226)	Nectin-2, PVR	X	X
2B4 (CD244)	CD48	X	X

**Table 100.** Key markers for murine NK cell identification and characterization

	Marker	Expression
NK lineage	NK1.1 (CD161)	surface
	NKp46 (NCR1; CD335)	surface
	CD49b	surface
	T-bet	nucleus
Distintion from other ILCs	IL7Rα (CD127)	surface, ILC1, ILC2, ILC3
	CD49a	surface, ILC1
	CD200R	surface, ILC1
	TRAIL	surface, ILC1
	Eomes	nucleus, NK cells
	Rorγt	nucleus, ILC3
Development/maturation stages	CD27	surface, immature NK cells
	CD11b (Mac-1/CD18)	surface, mature NK cells
	KLRG1	surface, mature NK cells
	CD43	surface, mature NK cells
	CXCR6	surface, "memory" NK cells in the liver

**Table 101.** General reagents and materials

Reagent	Manufacturer	Cat #
Collagenase IV	Sigma	C5138
DNase I	Roche	10104159001
Dulbecco’s phosphate-buffered saline (PBS)		
Ficoll-Paque	GE Healthcare	17-1440-02
Foetal bovine serum (FBS)	Serana	S-FBS-SA-015
L-glutamine	Sigma-Aldrich	G7513-100ML
LIVE/DEAD^™^ Fixable Blue Dead Cell Stain Kit	Life Technologies	L23105
Penicillin-Streptomycin	Sigma-Aldrich	F4333
1× RBC Lysis Buffer	eBioscience	00-4333-57
RPMI 1640	HyClone	SH30255.01
Worthington’s Collagenase, Type IV	Worthington Biochemical	LS004188
Material		Protocol
fine scissors, scalpels, Goulian knife		sp, lu, sk
fine forceps/tweezers		sp, lu, sk
6-well / 12-well plates		sp, lu, sk
1.5 ml / 2 ml microcentrifuge tube		bl, sp, lu, sk
5 ml polystyrene flow cytometry tubes		bl, sp, lu, sk
15 ml / 50 ml conical tubes		bl, sp, lu, sk
70 μm cell strainer		sp, lu, sk
25 G needles		sk (epidermis)
Styrofoam with rubber mat, sterile silicon mat		sk (epidermis)

a) blood (bl), spleen (sp), lung (lu) and skin (sk)

**Table 102.** Antibodies for human monocyte identification

Target Molecule	Fluoro chrome	Isotype	Clone	Dilution	Provider	Cat#	Target Cell	Target Tissue
Primary ab								
CD3ε	BV650	Mouse IgG2a	OKT3	1/20	Biolegend	317324	T cell	bl, sp, lu,
CD11b	BV650	Rat IgG2b	M1/70	1/20	Biolegend	101239	Mo/Mac	lu
CD11c	BV605	mouse IgG1	3.9	1/20	Biolegend	301636	cDC2	bl, sp,
CD14	BV650	mouse IgG2a	M5E2	1/20	Biolegend	301836	DCs, Mac	lu
CD14	Spark Blue 550	mouse IgG21	63D3	1/20	Biolegend	367147	DCs, Mo	bl, sp, lu
CD14	AF700	mouse IgG2a	M5E2	1/20	Biolegend	301822	Mo/Mac	sp,ln
CD14	APC/Cy7	mouse IgG1	HCD14	1/20	Biolegend	325619	Mo/Mac	lu
CD16	BV650	mouse IgG1	3G8	1/40	Biolegend	302018	Mo/Mac/NK cell	bl, sp, lu,
CD16	APC/Cy7	mouse IgG1	3G8	1/20	Biolegend	302017	Mo/Mac	ln, sp
CD16	PerCP-eFluor 710	mouse IgG1	CB16	1/20	ebioscience	46-0168-42	Mo/Mac	lu
CD19	BV650	mouse IgG1	HIB19	1/20	Biolegend	302238	B cell	bl, sp, lu,
CD20	BV650	mouse IgG2b	2H7	1/20	Biolegend	302336	B cell	bl, sp, lu,
CD45	V500	mouse IgG1	HI30	1/20	BD Biosciences	560777	DCs, Mo/Mac	bl, sp, lu,
CD45	BUV395	mouse IgG1	HI30	1/20	BD Biosciences	563792	DCs, Mo/Mac	lu
CD206	PE/CFS954	mouse IgG1	19.2	1/60	BD Biosciences	564063	Mac	lu
HLA-DR	PE/Fire810	mouse IgG2a	L243	1/20	Biolegend	307642	DCs, Mo/Mac	bl, sp, lu,
HLA-DR	BV785	mouse IgG2a	G46-6	1/20	BD Biosciences	564041	DCs, Mo/Mac	ln, sp
CD88	APC/Fire750	mouse IgG2a	S5/1	1/20	Biolegend	344315	Mo/Mac	
CD88	PE/vio770	mouse IgG2a	S5/1	1/20	Miltenyi	130-104-336	Mo/Mac	ln, sp
CD206	APC	mouse IgG1	15-2	1/20	Biolegend	321109	Mac	lu
LYVE1	Biotin	Goat IgG	Polyclonal	1/50	R&D	BAF2089	Mac	lu
Streptavidin	PE-CF594	−	−	1/400	BD Biosciences	562284	NA	lu

a) blood (bl), spleen (sp), lung (lu), skin (sk); Mo (monocyte), Mac (macrophage)

**Table 103.** Summary phenotype table of human monocytes

	Human		
	Classical monocytes	Intermediate monoctes	Non-classical monocytes
Marker			
CD11b	hi	+	lo
CD11c	+	+	+
CD14	hi	+	lo
CD16	−	+	+
CD43	+	+	+
CD45	+	+	+
CD62L	lo	lo/−	−
CD64	+	+	lo/−
CD68	NA	+	NA
CD115	lo	hi	int
CD163	+/−	+/−	+/−
CD169	+/−	NA	+/−
CCR2	hi	int	−
CCR5	+	NA	NA
CX3CR1	lo	int	hi
HLA-DR	+	hi	lo
Lyve-1	hi	+	lo
SLAN	−	−	+/−
Transcription factor			
C/EBPa	+	+	+
IRF8	+	+	+
PU.1	+	+	+
Cytokines & Chemokines			
CCL2	a	a	a
CCL7	a	a	a
Csf-1	a	a	a
IL-1a/b	a	a	a
IL-6	a	a	a
IL-12	a	a	a
TNFa	a	a	a

+ indicates expression, hi indicates high expression, − indicates no expression, +/− indicates bimodal expression, lo indicates low expression, a indicates expression on activated cells, NA indicates not applicable/unknown [1359, 1361, 1362, 1365, 1366, 1376-1378].

**Table 104.** Summary phenotype table of human macrophages

	Human Macrophages
Marker	
CD11b	+ (skin)
CD14	+ (skin
CD16	lo/− (skin)
CD43	+
CD45	+
CD64	+
CD68	+
CD71	+
CD80	+/−
CD86	+/−
CD115	+
CD163	+/−
CD169	+
CD206	+/−
CCR2	−
CCR5	+/−
HLA-DR	+ (skin)
MerTK	+
Cytokines/Chemokines	
CCL2	a
CCL3	a
CCL4	a
CCL5	a
IL-1b	a
IL-6	a
IL-10	a
IL-12	a
IL-23	a
IFNg	a
TNFa	a

+ indicates high expression, − indicates no expression, +/− indicates bimodal expression, lo indicates low expression, a indicates expression on activated cells, NA indicates not applicable [1392, 1393].

**Table 105.** Antibodies for human dendritic cell identification

Target Molecule	Fluoro chrome	Isotype	Clone	Dilution	Provider	Cat#	Target Cell	Target Tissue
Primary ab								
CADM1	Purified	chicken IgY	3E1	1/400	MBL	CM004-3	cDC1	ln
CD1c	BV421	mouse IgG1	L161	1/20	Biolegend	331526	cDC1,cDC2	ln
CD1c	Super Bright 436	mouse IgG1	L161	1/20	eBioscience	62-0015-42	cDC1,cDC2	bl, sp
CD3ε	BV650	Mouse IgG2a	OKT3	1/20	Biolegend	317324	T cell	bl, sp, ln
CD5	BV711	mouse IgG1	UCHT2	1/20	BD Biosciences	563170	early pre-DC	ln
CD5	APC/R700	mouse IgG1	UCHT2	1/20	BD Biosciences	565121	early pre-DC	bl, sp
CD14	Spark Blue 550	mouse IgG1	M5E2	1/20	Biolegend	367147	DCs, Mac	bl, sp
CD14	AF700	mouse IgG2a	RMO52	1/10	Beckman Coulter	IM2707U	DCs, Mo	ln
CD16	BV650	mouse IgG1	3G8	1/40	Biolegend	302018	Mo/Mac/NK cell	bl, sp, ln
CD19	BV650	mouse IgG1	HIB19	1/20	Biolegend	302238	B cell	bl, sp, ln
CD20	BV650	mouse IgG2b	2H7	1/20	Biolegend	302336	B cell	bl, sp, ln
CD45	V500	mouse IgG1	HI30	1/20	BD Biosciences	560777	DCs, Mo/Mac	bl, sp, ln
CD45RA	FITC	mouse IgG2b	HI100	1/20	Biolegend	983002	pDC, early pre-DC	ln
CD123	BUV395	mouse IgG2a	7G3	1/40	BD Biosciences	564195	pDC, early pre-DC	ln
CD123	PE-Dazzle 594	mouse IgG1	S18016C	1/20	Biolegend	396605	pDC, early pre-DC	bl, sp
CD141	BV421	mouse IgG1	1A4	1/20	BD Biosciences	565321	cDC1	bl, sp
CD169 (Siglec-1)	PE	mouse IgG1	7-239	1/20	BD Biosciences	565248	early pre-DC	ln
CD169 (Siglec-1)	BUV661	mouse IgG1	7-239	1/20	BD Biosciences	750363	early pre-DC	bl, sp
HLA-DR	BV785	mouse IgG2a	L243	1/20	Biolegend	307642	DCs, Mo/Mac	ln
HLA-DR	PE/Fire810	mouse IgG2a	L243	1/20	Biolegend	900000155	DCs, Mo/Mac	bl, sp
anti-Chicken IgY	Alexa Fluor 647	Donkey Fab’2	N/A	1/200	Jackson Immuno-research	703-606-155	cDC1	ln
CD88	APC/Fire750	mouse IgG2a	S5/1	1/20	Biolegend	344315	Mo/Mac	bl, sp
CD88	PE/vio770	mouse IgG2a	S5/1	1/20	Miltenyi	130-104-336	Mo/Mac	ln
FcεR1α	BV711	mouse IgG2a	CRA1	1/20	BD Biosciences	747783	cDC2	bl, sp
FcεR1α	PerCP/Cyanine5.5	mouse IgG2b	CRA1	1/20	Biolegend	334621	cDC2	ln

a) blood (bl), spleen (sp), lymph node (ln); Mo (monocyte), Mac (macrophage)

**Table 106.** Summary phenotype table of human DCs

	Human cDC1	cDC2	pDC
Marker			
CD1a	+/−	+/−	−
CD1c	−	+/−	−
CD4	+	+	+
CD5	+/−	+/−	−
CD8α	−	−	+/−
CD9	+	+	+/−
CD11b	−	+	−
CD11c	+	+	lo
CD14	−	+/−	−
CD24	−	−	−
CD40	+/−	+/−	+/−
CD45	+	+	+
CD45R (B220)	−	−	−
CD80	+/−	+/−	+/−
CD86	+/−	+/−	+/−
CD103	+/−	−	−
CD123	−	−	+
CD141	+	+/−	+/−
CD172	−	+	+/−
CD303	−	−	+
CD304	−	−	+
CD317 (BST-2/mPDCA-1)	+	+	+
CADM1	+	−	−
CCR7	+/−	+/−	+/−
Clec9a	+	−	−
HLA-DR	hi	hi	+
XCR1	+	−	−
Transcription factors			
IRF4	−	+	−
IRF8	+	−	+
Tcf4	−	−	+
Cytokines/Chemokines			
IFNa	−	−	a
IFNb	−	−	a
IL-12	a	a	a
TNFa	a	a	a

+ indicates high expression, − indicates no expression, +/− indicates bimodal expression, lo indicates low expression, a indicates expression on activated cells, NA indicates not applicable/unknown

**Table 107.** General reagents and materials

Reagent	Manufacturer	Cat #
Bovine serum albumin, lyophilized powder (BSA)	Sigma-Aldrich	A2058
DAPI	Life Technologies	D1306
Dispase	Gibco	17105041
DNase I	Roche	10104159001
Dulbecco’s phosphate-buffered saline(PBS)		
Dithiothreitol (DTT)	Gold Biotechnology	DTT50
EDTA 0.5 M	Invitrogen	10458654
Foetal bovine serum (FBS)	Serana	S-FBS-SA-015
Heparin	Sigma-Aldrich	H3393
n-mouse serum	Sigma-Aldrich	M5905-10ML
n-rat serum	Sigma-Aldrich	R9759-10ML
Percoll		
1× RBC Lysis Buffer	eBioscience	00-4333-57
RPMI 1640	HyClone	SH30255.01
Material		Protocol
fine scissors		bm, sp, lu, int, sk, ln
fine forceps/tweezers		bm, sp, lu, int, sk, ln
6-well / 12-well plates		bm, sp, lu, int, sk, ln
1.5 ml / 2 ml microcentrifuge tubes		bl, bm, sp, lu, int, sk, ln
5 ml polystyrene flow cytometry tubes		bl, bm, sp, lu, int, sk, ln
15 ml / 50 ml conical tubes		bl, bm, sp, lu, int, sk, ln
70 μm cell strainer		bm, sp, lu, int, sk, ln
70 μm nylon mesh		lung
18 G blunt needle		lu, int, sk, ln
25 G needle		bm, ln
3 ml syringe		lu, int, sk, ln
20 ml syringe		bm
metal tray		int

a) blood (bl), bone marrow (bm), spleen (sp), lung (lu), intestine (int), skin (sk), lymph node (ln)

**Table 108.** Antibodies for murine monocyte identification

Target Molecule	Fluoro-chrome	Clone	Provider	Cat#	Target Cell	Target Tissue
Primary ab						
CD3ε	APC	145-2C11	BioLegend	100312	T cell	bl, bm, sp, lu, int, sk, ln
CD11b	PerCP- Cy5.5	M1/70	BioLegend	101227	Mo/Mac, cDC2	bm, sp, lu, int, sk, ln
CD11b	PerCP	M1/70	BioLegend	101259	Mo/Mac, cDC2	bl, bm, sp, lu, int, sk, ln
CD11b	BV650	M1/70	BioLegend	101259	Mo/Mac, cDC2	bl, bm, sp, lu, int, sk, ln
CD11c	PE-Cy7	N418	BioLegend	117318	Mo/Mac, DC, pDC	bl, bm, sp, lu, int, sk, ln
CD16/32	Pure	2.4G2	BD	553142	FC block	bl, bm, sp, lu, int, sk, ln
CD19	APC	eBio1D3	eBioscience	17-0193-82	B cell	bl, bm, sp, lu, int, sk, ln
CD45	BUV395	30-F11	BD	564279	all immune cells	bl, bm, sp, lu, int, sk, ln
CD49b	APC	DX5	BioLegend	108909	NK cell	bl, bm, sp, lu, int, sk, ln
CD115	PerCP	AFS98	eBioscience	46-1152-82	Mo/Mac	bl, bm, sp, lu, int, sk, ln
MHCII	Alexa Fluor 700	M5-114.15.2	BioLegend	107622	Mo/Mac, DC, PDC	bl, bm, sp, lu, int, sk, ln
Ly6C	APC-Cy7	HK1.4	BioLegend	128026	Mo, pre-DC, pDC	bl, bm, sp, lu, int, sk, ln
Ly6C	BV570	HK1.4	BioLegend	128029	Mo, pre-DC, pDC	bl, bm, sp, lu, int, sk, ln
Ly6G	APC	1A8	BioLegend	127614	neutrophil	bl, bm, sp, lu, int, sk, ln

a) blood (bl), bone marrow (bm), spleen (sp), lung (lu), intestine (int), skin (sk), lymph node (ln); Mo (monocyte), Mac (macrophage)

**Table 109.** Summary phenotype table of murine monocytes

	Mouse	
	Classical monocytes	Non-classical monocytes
Marker		
CD11b	+	+
CD11c	−	−
CD43	lo	hi
CD45	+	+
CD62L	+	−
CD64	−	−
CD115	+	+
CD169	+/−	+/−
CCR2	+	−
CX3CR1	int	hi
F4/80	−	−
MerTK	+/−	+/−
Ly6C	hi	lo
Lyve-1	−	−
Transcription factors		
C/EBPa	+	+
IRF8	+	+
KLF4	+	+
Ms4a3	+	+
PU.1	+	+
Cytokines/Chemokines		
CCL2	a	a
CCL7	a	a
Csf-1	a	a
IL-1a/b	a	a
IL-6	a	a
IL-12	a	a
TNFa	a	a

+ indicates expression, hi indicates high expression, − indicates no expression, +/− indicates bimodal expression, lo indicates low expression, a indicates expression on activated cells, NA indicates not applicable/unknown

**Table 110.** Phenotypic differences between human and murine monocytes

	Mouse			Human		
	Classical monocytes	Intermediate monoctes	Non-classical monocytes	Classical monocytes	Intermediate monoctes	Non-classical monocytes
Marker						
CD11c	−	NA	−	+	+	+
CD14	(+)	NA	(+)	hi	+	lo
CD16	lo	NA	+	−	+	+
CD115	+	NA	+	lo	hi	int
CD163	NA	NA	NA	+/−	+/−	+/−
F4/80	−	NA	−	NA	NA	NA
HLA-DR	NA	NA	NA	+	hi	lo
MerTK	+/−	NA	+/−	NA	NA	NA
Ly6C	hi	NA	lo	NA	NA	NA
Lyve-1	−	NA	−	hi	+	lo
SLAN	NA	NA	NA	−	−	+/−

+ indicates expression, hi indicates high expression, − indicates no expression, +/− indicates bimodal expression, lo indicates low expression, a indicates expression on activated cells, NA indicates not applicable/unknown

**Table 111.** Antibodies for murine macrophage identification

Target Molecule	Fluoro-chrome	Clone	Provider	Cat#	Target Cell	Target Tissue
Primary ab						
CD3ε	APC	145-2C11	BioLegend	100312	T cell	bm, sp, lu, int, sk, ln
CD11b	PerCP- Cy5.5	M1/70	BioLegend	101227	Mo/Mac, cDC2	bm, sp, lu, int, sk, ln
CD11b	BV650	M1/70	BioLegend	101259	Mo/Mac, cDC2	bm, sp, lu, int, sk, ln
CD11c	PE-Cy7	N418	BioLegend	117318	Mo/Mac, DC, pDC	bm, sp, lu, int, sk, ln
CD16/32	Pure	2.4G2	BD	553142	FC block	bm, sp, lu, int, sk, ln
CD19	APC	eBio1D3	eBioscience	17-0193-82	B cell	bm, sp, lu, int, sk, ln
CD45	BUV395	30-F11	BD	564279	all immune cells	bbm, sp, lu, int, sk, ln
CD49b	APC	DX5	BioLegend	108909	NK cell	bm, sp, lu, int, sk, ln
CD64 / FcγRI	PE	X54-5/7.1	BD	558455	Mac	bm, sp, lu, int, sk, ln
CD64 / FcγRI	BV711	X54-5/7.1	BioLegend	139311	Mac	bm, sp, lu, int, sk, ln
F4/80	PE-CF594	T45-2342	BD Horizon	565613	Mac, DC	bm, sp, lu, int, sk, ln
MerTK	PE-Cy7	DS5MMER	Invitrogen	25-5751-82	Mac	bm, lu
MHCII	Alexa Fluor 700	M5-114.15.2	BioLegend	107622	Mo/Mac, DC, pDC	bm, sp, lu, int, sk, ln
Ly6C	APC-Cy7	HK1.4	BioLegend	128026	Mo, pre-DC, pDC	bm, sp, lu, int, sk, ln
Ly6G	APC	1A8	BioLegend	127614	neutrophil	bm, sp, lu, int, sk, ln
SiglecF	BV421	E50-2440	BD Bioscience	562681	Mac	lu

* blood (bl), bone marrow (bm), spleen (sp), lung (lu), intestine (int), skin (sk), lymph node (ln); Mo (monocyte), Mac (macrophage)

**Table 112.** Summary phenotype table of murine macrophages

	Mouse
Macrophages	
Marker	
CD11b	+/−
CD45	+
CD62L	−
CD64	+
CD68	+
CD71	+
CD80	+/−
CD86	+/−
CD115	+
CD169	+
CD206	+/−
CCR2	+/−
CCR5	+/−
CX3CR1	+/lo
F4/80	+
MerTK	+
MHCII	+/lo
Ly6C	−
Lyve-1	+/lo
TIM4	+/−
Transcription factor	
C/EBPb	+
CSF1R	+
ID3	+
IRF8	+
MAFB	+
Cytokines/Chemokines	
CCL2	a
CCL3	a
CCL4	a
CCL5	a
IL-1b	a
IL-6	a
IL-10	a
IL-12	a
IL-23	a
IFNg	a
TNFa	a

+ indicates high expression, − indicates no expression, +/− indicates bimodal expression, lo indicates low expression, a indicates expression on activated cells, NA indicates not applicable

**Table 113.** Phenotypic differences between human and murine macrophages

	Macrophages	
	Mouse	Human
Marker		
CD14	NA	+ (skin
CD16	NA	lo/− (skin)
CD43	NA	+
CD62L	−	NA
CD163	NA	+/−
CX3CR1	+/lo	NA
F4/80	+	NA
HLA-DR	NA	+ (skin)
MHCII	+/lo	NA
Ly6C	−	NA
Lyve-1	+/lo	NA
TIM4	+/−	NA

+ indicates high expression, − indicates no expression, +/− indicates bimodal expression, lo indicates low expression, a indicates expression on activated cells, NA indicates not applicable

**Table 114.** Antibodies for murine dendritic cell identification

Target Molecule	Fluoro-chrome	Clone	Provider	Cat#	Target Cell	Target Tissue
Primary ab						
CD3ε	APC	145-2C11	BioLegend	100312	T cell	bl, bm, sp, lu, int, sk, ln
CD8α	BV605	53-6.7	BioLegend	100744	cDC1, pDC	bl, bm, sp, lu, int, sk, ln
CD11b	PerCP- Cy5.5	M1/70	BioLegend	101227	Mo/Mac, cDC2	bl, bm, sp, lu, int, sk, ln
CD11b	BV650	M1/70	BioLegend	101259	Mo/Mac, cDC2	bl, bm, sp, lu, int, sk, ln
CD11c	PE-Cy7	N418	BioLegend	117318	Mo/Mac, DC, pDC	bl, bm, sp, lu, int, sk, ln
CD11c	BV605	N418	BioLegend	117334	Mo/Mac, DC, pDC	bl, bm, sp, lu, int, sk, ln
CD16/32	Pure	2.4G2	BD	553142	FC block	bl, bm, sp, lu, int, sk, ln
CD19	APC	eBio1D3	eBioscience	17-0193-82	B cell	bl, bm, sp, lu, int, sk, ln
CD24	eFluor450	M1/69	eBioscience	48-0242-82	cDC2	bl, bm, sp, lu, int, sk, ln
CD24	PE	M1/69	eBioscience	12-0242-83	cDC2	bl, bm, sp, lu, int, sk, ln
CD45	BUV395	30-F11	BD	564279	all immune cells	bl, bm, sp, lu, int, sk, ln
CD49b	APC	DX5	BioLegend	108909	NK cell	bl, bm, sp, lu, int, sk, ln
CD64 / FcγRI	PE	X54-5/7.1	BD	558455	Mac	bl, bm, sp, lu, int, sk, ln
CD64 / FcγRI	BV711	X54-5/7.1	BioLegend	139311	Mac	bl, bm, sp, lu, int, sk, ln
CD103	APC	2E7	eBioscience	17-1031-82	cDC1	lu, int, sk, ln
CD317/ m-PDCA-1	PE	eBio97	eBioscience	13-1721-82	pDC	bl, bm, sp, lu, int, sk, ln
B220/CD45R	BV421	RA3-6B2	BioLegend	103240	pDC, B cell	bl, bm, sp, lu, int, sk, ln
EpCAM	BV605	G8.8	BioLegend	118227	cDC2	sp
F4/80	PE-CF594	T45-2342	BD Horizon	565613	Mac, DC	bl, bm, sp, lu, int, sk, ln
MHCII	AlexaFluor 700	M5-114.15.2	BioLegend	107622	Mo/Mac, DC, pDC	bl, bm, sp, lu, int, sk, ln
Ly6G	APC	1A8	BioLegend	127614	neutrophil	bl, bm, sp, lu, int, sk, ln
SiglecH	PerCP-Cy5.5	551	BioLegend	129614	pDC	bl, bm, sp, lu, int, sk, ln
XCR1	PE	ZET	BioLegend	148204	cDC1	bl, bm, sp, lu, int, sk, ln

* blood (bl), bone marrow (bm), spleen (sp), lung (lu), intestine (int), skin (sk), lymph node (ln); Mo (monocyte), Mac (macrophage)

**Table 115.** Summary phenotype table of murine DCs

	Mouse		
	cDC1	cDC2	pDC
Marker			
CD4	−	+/−	+/−
CD8α	+	−	+/−
CD9	NA	NA	+/−
CD11b	−	+	−
CD11c	Hi	hi	+
CD14	−	−	−
CD24	+	+	−
CD40	+/−	+/−	+/−
CD45	+	+	+
CD45R%%% (B220)	−	−	+
CD64	−	−	−
CD80	+/−	+/−	+/−
CD86	+/−	+/−	+/−
CD103	+/−	−	−
CD172	−	+	+/−
CD317(BST-2/mPDCA-1)	−	−	+
CCR7	+/−	+/−	+/−
CCR9	NA	NA	+/−
Clec9a	+	−	+
ESAM	−	+/−	−
F4/80	−	+/−	−
MHCII	Hi	hi	+/−
Ly6C	−	−	+/−
SiglecH	−	−	+
XCR1	+	−	−
Transcription factors			
Batf3	+	−	−
Bcl6	−	+	+
Bcl11a	−	+	+
Id2	+	+	−
IRF4	−	+/−	+
IRF7	−	−	+
IRF8	+	+/−	+
Klf4	−	−	
Nfil3	+	−	−
Notch2	−	+	−
Tcf4	−	−	+
RelB	−	+	−
Runx2	−	−	+
Zbtb46	+	+	−
Cytokines/Chemokines			
IFNa	A	a	a
IFNb	A	a	a
CCL3	A	a	a
CCL5	A	a	a
CCL19	A	a	a
CCL21	A	a	a
IL-10	A	a	a
IL-12	A	a	a
TNFa	A	a	a

+ indicates high expression, − indicates no expression, +/− indicates bimodal expression, lo indicates low expression, a indicates expression on activated cells, NA indicates not applicable/unknown

**Table 116.** Phenotypic differences between human and murine DCs

	Mouse			Human		
	cDC1	cDC2	pDC	cDC1	cDC2	pDC
Marker						
CD1a	NA	NA	NA	+/−	+/−	−
CD1c	NA	NA	NA	−	+/−	−
CD5	NA	NA	NA	+/−	+/−	−
CD8α	+	−	+/−	−	−	+/−
CD14	−	−	−	−	+/−	−
CD24	+	+	−	−	−	−
CD45R (B220)	−	−	+	−	−	−
CD123	−	−	NA	−	−	+
CD141	NA	NA	NA	+	+/−	+/−
CD303	NA	NA	NA	−	−	+
CD304	NA	NA	NA	−	−	+
CD317 (BST-2/mPDCA-1)	−	−	+	+	+	+
ESAM	−	+/−	−	NA	NA	NA
F4/80	−	+/−	−	NA	NA	NA
HLA-DR	NA	NA	NA	Hi	hi	+
MHCII	Hi	hi	+/−	NA	NA	NA
Ly6C	−	−	+/−	NA	NA	NA
SiglecH	−	−	+	NA	NA	NA

+ indicates high expression, − indicates no expression, +/− indicates bimodal expression, lo indicates low expression, a indicates expression on activated cells, NA indicates not applicable/unknown

**Table 117.** Additional reagents

Reagent	Manufacturer	Cat #
LPS	Sigma-Aldrich	L2880
Golgistop (monensin)	BD	554715
Fixation/Permeabilization solution	BD	554715
Perm/Wash Buffer	BD	554715

**Table 118.** Additional reagents

Reagent	Manufacturer	Cat #
Naive CD4^+^ T Cell Isolation Kit II	Miltenyi Biotec	130-094-131
CellTrace Violet	Invitrogen	C34571
Iscove’s Modified Dulbecco’s Medium	ThermoFisher	12440053
KnockOut Serum Replacement	ThermoFisher	10828028

**Table 119.** Additional Abs

Target Molecule	Fluoro-chrome	Clone	Provider	Cat#	Target Cell	Target Tissue
Primary ab						
CD3	BV650	SP34-2	BD Biosciences	563916	T cells	NA
IFNγ	APC/Cy7	B27	Biolegend	506523	T cells	NA

**Table 120.** Additional Abs for surface activation markers

Target Molecule	Fluoro-chrome	Clone	Provider	Cat#	Target Cell	Target Tissue
Primary ab						
CD40	APC	145-2C11	BioLegend	100312	Mo/Mac, DC	bl, bm, sp, lu, int, sk, ln
CD80	PerCP- Cy5.5	M1/70	BioLegend	101227	Mo/Mac, DC	bm, bm, sp, lu, int, sk, ln
CD86	PerCP	M1/70	BioLegend	101259	Mo/Mac, DC	bl, bm, sp, lu, int, sk, ln

* blood (bl), bone marrow (bm), spleen (sp), lung (lu), intestine (int), skin (sk), lymph node (ln); Mo (monocyte), Mac (macrophage)

**Table 121.** Additional materials

Reagent	Manufacturer	Cat #
Naive CD4^+^ T Cell Isolation Kit	Miltenyi Biotec	130-104-453

**Table 122.** List of Abs used for characterization of human granulocyte populations

Target	Fluorophore	Supplier	Cat#	Clone	Host	Isotype	Cross Reactivity
CCR3	PerCp/Cy5.5	BioLegend	310717	5E8	Mouse	IgG2b, κ	None
CD11b	PE/Cy7	BioLegend	301321	ICRF44	Mouse	IgG1, κ	Yes
CD15	APC	BioLegend	301907	HI98	Mouse	IgM, κ	Yes
CD16	APC/Cy7	BioLegend	302017	3G8	Mouse	IgG1, κ	Yes
CD45	FITC	BioLegend	304005	HI30	Mouse	IgG1, κ	Yes
FcRε1	eFluor450	eBioscience	14-5899-82	AER-37	Mouse	IgG2b, κ	None
Siglec-8	PE	BioLegend	347103	7C9	Mouse	IgG1, κ	None
CD49d	PE	BD Pharmingen	555503	9F10	Mouse	IgG1, κ	Yes

**Table 123.** List of Abs used for characterization of murine granulocyte populations

Target	Fluorophore	Supplier	Cat#	Clone	Host	Isotype	Cross Reactivity
CCR3	APC/Fire750	BioLegend	144521	J073E5	Rat	IgG2a, κ	None
CD11b	eFluor450	eBioscience	48-0112-82	M1/70	Rat	IgG2b, κ	Yes
CD45	BV785	BioLegend	103149	30-F11	Rat	IgG2b, κ	None
CD49b	APC	BioLegend	108909	DX5	Rat	IgM	None
CD200R3	PE	BioLegend	142205	Ba13	Rat	IgG2a, κ	None
Ly6C	AlexaFluor 488	BioLegend	128021	HK1.4	Rat	IgG2c, κ	None
Ly6G	PE/Cy7	BioLegend	127617	1A8	Rat	IgG2a, κ	None
Siglec-F	PerCP-eFluor710	eBioscience	46-1702-82	1RNM44N	Rat	IgG2a, κ	None

**Table 124.** Selection of important markers for flow cytometry analysis of human and mouse granulocytes. All cell types were first gated on CD45 and CD11b positivity

Cell type	Human	Mouse
Neutrophil	CD15^pos^, CD16^pos^, CD66b^pos^	Ly6C^int^, Ly6G^pos^
Eosinophil	CD15^pos^, CD16^neg^, Siglec-8^pos^, CCR3^pos^, FcεRIα^pos^	Ly6C^low/int^, Siglec-F^pos^, CCR3^pos^, *FcεRIα^pos^
Basophil	CD15^neg^, CD16^neg^, CCR3^pos^, FcεRIα^pos^, *CD117^neg^, *CD203c^pos^	Ly6C^low^, CD200R3^pos^, CD49b^pos^, *FcεRIα^pos^

a) These markers were not used in our analysis, but are valid markers for the given cell types.

**Table 125.** Summary table noting key information between human and murine cells

Peripheral blood [% of leukocytes]	Human	Mouse*
Neutrophil	~ 60	10-30
Eosinophil	1-3	0-5
Basophil	< 1	0-1

* Depending on mouse strain, see [1462].

**Table 126.** Staining reagents for human neutrophils

Marker	Fluorophore	Supplier	Catalogue Number	Clone	Host	Isotype
CD10	FITC	Biolegend	312208	HI10a	Mouse	IgG1, κ
CD33	PE	Biolegend	303404	WM53	Mouse	IgG1, κ
CD49d	PE-Texas Red	BD Biosciences	563645	9F10	Mouse	IgG1, κ
CD3	PE-Cy7	Biolegend	300420	UCHT1	Mouse	IgG1, κ
CD19	PE-Cy7	eBioscience	25-0199	HIB19	Mouse	IgG1, κ
CD56	PE-Cy7	Biolegend	318318	HCD56	Mouse	IgG1, κ
CD34	PE-Cy7	Biolegend	343516	581	Mouse	IgG1, κ
CD66b	BV421	BD Biosciences	562940	G10F5	Mouse	IgM, κ
CD15	BV655	BD Biosciences	564232	HI98	Mouse	Ig2M, κ
CD14	BV711	Biolegend	301838	M5E2	Mouse	IgG2a, κ
CD101	APC	Biolegend	331010	BB27	Mouse	IgG1, κ
CD45	APC-Cy7	Biolegend	304014	HI30	Mouse	IgG1, κ
CD16	BUV737	BD Biosciences	564434	3G8	Mouse	IgG1, κ
CD16/32	Purified	BD Biosciences	564219	2.4G2	Mouse	IgG2b, κ
CD11b	BUV395	BD Biosciences	563839	ICRF44	Mouse	IgG1, κ

**Table 127.** Summary Phenotype Table of Human Neutrophils

	Promyelocytes		Myelocytes/Meta-myelocytes	Band Cells	Segmented Neutrophil
	proNeu1	proNeu2	preNeu	Immature Neu	Mature Neu
Surface Marker					
CD117	lo	lo/−	−	−	−
Cd49d	+	+	+	−	−
CD11b	−	−	+	+	+
CXCR2	−	−	−	−	+
CXCR4	+	+	lo	−	−
CD101	−	−	+	+	+
CD71	+	+	lo	−	−
CD81	+	−	−	−	−
CD88	−	−	+	+	+
CD43	+	+	+	−	−
CD11a	lo	−	−	+	+
CD106	+	+	+	+	+
CD34	lo	−	−	−	−
CD10	−	−	−	−	+
CD66b	lo	+	+	+	+
CD16	−	−	−	+	+
CD15	+	+	+	+	+
Transcription Factors					
c-myb	+	+	lo	−	−
AML-1	+	+	lo	−	−
GATA-1	+	+	lo	−	−
C/EBP-α	+	+	+	+	lo
C/EBP-β	−	−	lo	+	+
C/EBP-δ	−	−	lo	+	+
C/EBP-ε	+	+	+	−	−
C/EBP-γ	+	+	lo	−	−
C/EBP-*ζ*	−	−	−	+	+
PU.1	lo	lo	lo	+	+
Granules					
MPO	+	+	lo	−	−
LTF	lo	lo	+	lo	−
fMLP-R	−	−	−	+	+

+ indicates high expression, − indicates no expression, +/− indicates bimodal expression, lo indicates low expression and NA indicates not applicable. Adapted from [1469, 1484].

**Table 128.** Staining reagents for murine neutrophils

Marker	Fluorophore	Supplier	Catalogue Number	Clone	Host	Isotype
CD101	PE	eBioscience	12-1011	Moushi101	Rat	IgG2a, κ
Siglec-F	PE-Texas Red	BD Biosciences	562757	E50-2440	Rat	IgG2a, κ
Ly-6G	PE-Cy7	Biolegend	127618	1A8	Rat	IgG2a, κ
B220	PE-Cy7	eBioscience	25-0452	53-6.7	Rat	IgG2a, κ
NK1.1	PE-Cy7	eBioscience	25-5941	PK136	Mouse	IgG2a, κ
CD90.2	PE-Cy7	eBioscience	25-0902-82	53-2.1	Rat	IgG2a, κ
CD106	PerCP-Cy5.5	Biolegend	105716	MVCAM.A	Rat	IgG2b, κ
cKit	SB436	eBioscience	62-1171	2B8	Rat	IgG2b, κ
CD11b	BV655	Biolegend	101259	M1/70	Rat	IgG2b, κ
Gr-1	BV711	Biolegend	108443	RB3-8C5	Rat	IgG2b, κ
CD115	APC	eBioscience	61-1152	AFS98	Rat	IgG2a, κ
Ly-6C	APC/Cy7	eBioscience	47-5932	HK1.4	Rat	IgG2a, κ
CD81	Biotin	Biolegend	104903	Eat-2	Armenian Hamster	IgG
Streptavidin	BUV737	BD Biosciences	564176	−	−	−
CD16/32	APC-R700	BD Biosciences	565502	2.4G2	Rat	IgG2b, κ
CXCR4	AF488	eBioscience	53-9991-80	2B11	Rat	IgG2b, κ

**Table 129.** Summary Phenotype Table of Murine Neutrophils

	proNeu1	proNeu2	preNeu	Immature Neu	Mature Neu
Surface Marker					
CD117	+	+	lo	−	−
Cd49d	+	+	+	−	−
CD11b	lo	+	+	+	+
CXCR2	−	−	−	−	+
CXCR4	+	+	lo	−	−
CD101	−	−	−	−	+
CD71	+	+	lo	−	−
CD81	+	+	+	lo	−
CD88	−	−	+	+	+
CD43	+	+	+	−	−
CD11a	−	−	lo	+	+
CD106	−	+	+	+/−	−
CD34	+	lo	−	−	−
Ly-6G	−	−	+/−	+	+
Ly-6C	+	+	+	+	lo
Gr-1	lo	lo	+	+	+
Transcription Factors					
C/EBP-α	+	+	lo	−	−
C/EBP-β	−	−	−	+	+
C/EBP-δ	−	−	lo	+	+
C/EBP-ε	+	+	+	lo	−
C/EBP-γ	−	−	−	lo	lo
C/EBP-*ζ*	+	+	lo	−	−
PU.1	lo	lo	lo	+	+
Granules					
MPO	+	+	lo	−	−
LTF	−	−	+	lo	−
S100A8/9	−	lo	+	+	+
Neutrophil Elastase	+	+	+	−	−

+ indicates high expression, − indicates no expression, +/− indicates bimodal expression, lo indicates low expression and NA indicates not applicable. Adapted from [1479, 1486]

**Table 130.** Summary phenotype table noting the key differences between human and murine neutrophils

Neutrophil Subset	Mouse	Human
Pro-Neutrophil 1 (proNeu1)	cKit^hi^CD16/32^hi^Ly6C^+^CD81^+^CD106^−^CD11b^−^	CD66b^lo^CD15^+^CD11b^−^CD49d^hi^SSC^lo^
Pro-Neutrophil 2 (proNeu2)	cKit^hi^CD16/32^hi^Ly6C^+^CD81^+^CD106^+^CD11b^lo^	CD66b^+^CD15^+^CD11b^−^CD49d^hi^SSC^lo^
Pre-Neutrophils (preNeus)	CD115^−^SiglecF^−^Gr-1^hi^CD11b^+^CXCR4^hi^cKit^lo^Ly6G^lo^CD101^−^	CD66b^+^CD15^+^CD49d^lo^CD101^−^CD16^+^CD10^−^
Immature Neutrophils	CD115^−^SiglecF^−^Gr-1^hi^CD11b^+^CXCR4^lo^cKit^−^Ly6G^−/+^CD101^−^	CD66b^+^CD15^+^CD49d^−^CD101^+^CD16^++^CD10^−^
Mature Neutrophils	CD115^−^SiglecF^−^Gr-1^hi^CD11b^+^CXCR4^lo^cKit^−^Ly6G^+^CD101^+^	CD66b^+^CD15^+^CD49d^−^CD101^+^CD16^++^CD10^+^

**Table 131.** Example of an Ab panel for basic identification of human bone marrow-derived MSCs

Antibody	Fluorochrome	Clone	Company
CD105	FITC	SN6	ThermoFisher
CD73	PE	AD2	BD
CD34	ECD (PE-Texas Red)	581	Beckman Coulter
CD90	PerCP-Cy5.5	5E10	Biolegend
CD31	APC	9-G11	Bio-Techne
CD45	APC-Cy7	2D1	BD
	DAPI		ThermoFisher

**Table 132.** Selection of surface markers for flow cytometric analysis of human bone marrow-derived MSCs; + positive, +/− heterogeneous, − negative; according to published reports [1497, 1499, 1501, 1508]

Marker	Expression	Marker	Expression
CD3	−	CD73	+
CD10	+	CD90	+
CD11b	−	CD105	+
CD13	+	CD106	+/−
CD14	−	CD146	+
CD19	−	CD166	+
CD29	+	CD271	+/−
CD31	−	HLA-ABC	+
CD34	−	HLA-DR	−
CD44	+	SSEA-4	+
CD45	−	Stro-1	+/−

**Table 133.** Antibodies used for the identification of murine BM stroma cells

Antibody	Fluorochrome	Clone	Company
CD45	PerCPVio700	5B1	Miltenyi
Ter119	VioBlue	Ter119	Miltenyi
CD31	APC	390	BioLegend
CD106	PE-Cy7	429	eBioScience

**Table 134.** Selection of surface markers for flow cytometric analysis of human and mouse bone marrow-derived MSCs; + positive, +/− heterogeneous, − negative according to published reports [1499-1501]

Cluster of differentiation	Human	Mouse	Cluster of differentiation	Human	Mouse
CD3	−	−	CD71	+	+
CD10	+		CD73	+	+
CD11b	−	−	CD80	−	+
CD13	+		CD90	+	−
CD14	−		CD105	+	+
CD19	−		CD106	+/−	+
CD29	+	+	CD146	+	
CD31	−	−	CD166	+	
CD34	−	−	CD184	+	+
CD44	+	+	CD271	+/−	
CD45	−	−	HLA-ABC / MHC I	+	+
CD49e	+	+	HLA-DR/MHC II	−	−
CD51	+	+	Sca-1		+
CD61	+	+	STRO-1	+	

**Table 135.** Appropriate volumes of blocking reagent and magnetic beads for hCD34^+^ enrichment

% CD34^+^ cells (Quick Staining)	0.1	0.2	0.3	0.4	0.5	0.6	0.7	0.8	0.9	1.0	1.1	1.2	1.3
Volumes blocking reagent (μl) and magnetic beads (μl)	20	40	60	80	100	120	140	160	180	200	220	240	260

**Table 136.** Antibodies used for the identification of human cell types. All Abs should be titrated to determine optimal concentration

Antibody	Clone	Conjugate	Supplier
Human HSC Staining			
CD3	HIT3a	FITC	eBioscience
CD10	eBSN5c	FITC	eBioscience
CD14	M5E3	FITC	eBioscience
CD15	HI98	FITC	eBioscience
CD16	CB16	FITC	eBioscience
CD19	HIB19	FITC	eBioscience
CD235a	HIR2	FITC	eBioscience
CD34	581	APC	BioLegend
CD38	HIT2	PE-Cy7	eBioscience
CD45	HI30	Alexa Fluor^®^ 700	BioLegend
CD45RA	HI100	PerCP-Cy5.5	eBioscience
CD49f	GoH3	PE-Cy5	BD Biosciences
CD90	5,00E+10	PE	BD Biosciences
CD117	A3C6E2	Biotin	BioLegend
Streptavidin	APC-eFluor780	eBioscience	
Isotypes for the human HSC staining			
Mouse IgG1 κ	MOPC-21	PE	BioLegend
Mouse IgG1 κ	P3.6.2.8.1	PE-Cy7	eBioscience
Single Stainings for human Compensation Controls			
CD3	HT3a	FITC	eBioscience
CD3	OKT-3	PE	eBioscience
HLA-DR	L243	PerCP-Cy5.5	BioLegend
CD4	RPA-T4	PECy5	eBioscience
CD38	HIT2	PE-Cy7	eBioscience
CD3	UCHT1	APC	eBioscience
CD45	HI30	Alexa Fluor^®^ 700	BioLegend
CD45	HI30	APC-eFluor780	eBioscience
DAPI (4’-6-diamindino-2-phenylindole, dihydrochloride)			BioChemica A1001,0010

**Table 137.** BD LSRII configuration with laser and optical filters

Laser	Channel	Optical Filter	Fluorochrome
Blue			
488nm	488 A	780/60, LP 735	PE-Cy7
	488 B	710/40, LP 685	PerCP-Cy5.5
	488 C	676/29 LP 635	PE-Cy5
	488 D	576/29, LP 550	PE
	488 E	530/30, LP 505	FITC
	488 F	488/10	SSC detector
	488 G		blank
violet			
407nm	407 A	525/50, LP 505	Pacific Orange
	407 B	450/40	Pacific Blue
UV			
355nm	355 A	585/42, LP 505	Emerald 300
	355 B	440/40	DAPI
Red			
635nm	635 A	780/60, LP 735	APC-eFluor780
	635 B	720/13, 690LP	Alexa Fluor 700
	635 C	660/20	APC

**Table 138.** Cell surface phenotype for the identification of human HSPC populations. All cells are gated on CD45^+^ Lin^−^ cells (Lin= CD3 CD10 CD14 CD15 CD16 CD19 CD235)

HPC population	Phenotype/subphenotype	Reference
HPCs	CD34^+^ CD38^−^	[1550]
HSCs	CD34^+^ CD38^−^ CD90^+^ CD45RA^−^	[1551]
MPPs	CD34^+^ CD38^−^ CD90^−^ CD45RA^−^	[1551]
LT-HSCs	CD34^+^ CD38^−^ CD90^+^ CD45RA^−^ CD49f^+^	[1552]
LT-HSCs	CD34^+^ CD38^−^ CD90^+^ CD45RA^−^ CD117^hi^	[1553]

**Table 139.** Antibodies used for the identification of mouse HSC, MPP1, MPP2, MPP3 and MPP4 populations

Antibody	Clone	Conjugate	Supplier
Lineage markers			
CD3	145-2C11	Biotin or PerCP-Cy5.5	BD Biosciences
CD4	RM4-5	Biotin or PerCP-Cy5.5	BioLegend
CD8	53-6.7	Biotin or PerCP-Cy5.5	BioLegend
CD11b	M1/70	Biotin or PerCP-Cy5.5	BioLegend
CD11c	N418	Biotin or PerCP-Cy5.5	BioLegend
CD19	6D5	Biotin or PerCP-Cy5.5	BioLegend
CD45R/B220	RA3-6B2	Biotin or PerCP-Cy5.5	BioLegend
Ly6G/C(Gr1)	RB6-8C5	Biotin or PerCP-Cy5.5	BioLegend
NK1.1	PK136	Biotin or PerCP-Cy5.5	BioLegend
Ter119	TER-119	Biotin or PerCP-Cy5.5	BioLegend
Streptavidin		PerCP-Cy5.5	BioLegend
Stem and progenitor cell markers			
Kit	ACK2	APC	BioLegend
Sca1	D7	APC-Cy7	BioLegend
CD135 (Flk2)	A2F10	PE	BioLegend
CD34	RAM34	FITC	eBioscience
CD150	TC15-12F12.2	BrilliantViolet 510^™^	BioLegend
CD48	HM48-1	BrilliantViolet 421^™^	eBioscience
Isotypes control staining			
Rat IgG2a κ		FITC	BioLegend
Rat IgG2a κ		PE	BioLegend
Armenian Hamster, Rat IgG2a/b κ,		PerCP-Cy5.5	BioLegend
Armenian Hamster		BrilliantViolet 421^™^	BioLegend
Rat IgG2a λ		BrilliantViolet 510^™^	BioLegend
Rat IgG2b κ		APC	BioLegend
Rat IgG2a κ		APC-Cy7	BioLegend
Single staining for compensation controls			
CD19	6D5	FITC	BioLegend
CD19	1D3	PE	BioLegend
CD19	6D5	PerCP-Cy5.5	BioLegend
CD19	6D5	BrilliantViolet 421^™^	BioLegend
CD19	6D5	BrilliantViolet 510^™^	BioLegend
CD19	6D5	APC	eBioscience
CD19	6D5	APC-Cy7	BioLegend
PI (Propidium Iodide)			Invitrogen

**Table 140.** BD LSR Fortessa laser and optical filter configuration

Channels and lasers	Standard-LP	Standard-Filter	Parameter-Name (FACS Diva Software)	Fluorochromes
**Violet Laser 405nm**				
A	750LP	800/60	v-750LP 800/60-A	Brilliant Violet 785
B	685LP	710/50	V-685LP 710/50-B	Brilliant Violet 711
C	650LP	660/20	v-650LP 660/20-C	Brilliant Violet 650
D	600LP	610/20	v-600LP 610/20-D	Brilliant Violet 605
**E**	**505LP**	**525/50**	**v-505LP 525/50-E**	**Brilliant Violet 510**, A430, Pacific Orange,Vio 500
**F**	**NONE**	**450/50**	**v-450/50-F**	**Brilliant Violet 421**, Pacific Blue, DAPI, Violet 450
**Blue Laser 488nm**				
**A**	**685LP**	**710/50**	**b-685LP 710/50-A**	**PerCP-Cy5.5**, PerCP-eFluor710, PI
**B**	**505LP**	**525/50**	**b-505LP 525/50-B**	**FITC**, GFP, Alexa Fluor 488
**C**	**NONE**	**488/10**	**SSC**	
**Yellowgreen Laser 561nm**				
A	750LP	780/60	y-750LP 780/60-A	PE-Cy7
B	635LP	670/30	y-635LP 670/30-B	PE-Cy5
C	600LP	610/20	y-600LP 610/20-C	PE-Texas Red, Pe-CF594, Pe/Dazzle594
**D**	**NONE**	**582/15**	**y-582/15-D**	**PE**
**Red Laser 633 nm**				
**A**	**750LP**	**780/60**	**r-750LP 780/60-A**	**APC-Cy7**
B	685LP	720/30	r-685LP 720/30-B	Alexa Fluor 700
**C**	**NONE**	**670/30**	**r-670/30-C**	**APC**, Cy5

**Table 141.** Cell surface phenotype for the identification of mouse hematopoietic stem and progenitor cells. All cells are gated on Lin^−^ (Lin= CD3, CD4, CD8, CD11b, CD11c, CD19, CD45R/B220, Ly6G/C (Gr1), NK1.1, Ter119), kit^+^, Sca1^+^ LSK cells

HPC population	Phenotype/subphenotype	Reference
HSC	CD34^−^ Flk2(CD135)^−^CD150^+^CD48^−^	[1563]
MPP1	CD34^+^Flk2(CD135)^−^CD150^+^CD48^−^	[1563]
MPP2	CD34^+^Flk2(CD135)^−^CD150^+^CD48^+^	[1563]
MPP3	CD34^+^Flk2(CD135)^−^CD150^−^CD48^+^	[1563]
MPP4	CD34^+^Flk2(CD135)^+^CD150^−^CD48^+^	[1563]

**Table 142.** Overview of the most frequent human carcinomas carcinoma tissue most frequent form of carcinoma originating cell

carcinoma tissue	most frequent form of carcinoma	originating cell	Ref
lung cancer	non-small cell lung cancer (NSCLC)	type I / II alveolar epithelial cells	(a)
breast cancer	mammary carcinoma	epithelial cells of the milk duct	(b)
colon cancer	colorectal carcinoma (CRC)	epithelial cells of inner mucosal layer	(a)
prostate cancer	prostate carcinoma	epithelial basal cells of the prostate	[1600]
liver cancer	hepatocellular carcinoma (HCC)	Hepatocytes	(a)
stomach cancer	stomach carcinoma	epithelial cells transformed by H. pylori	(a)
cervical cancer	cervical carcinoma	cervical epithelial cells after HPV infection	(c)
oesophagus cancer	oesophagus carcinoma	epithelial cells lining the oesophagus	(d)
bladder cancer	bladder carcinoma	transitional epithelium of the bladder wall	(e)
pancreatic cancer	pancreatic carcinoma	endocrine ductal epithelial cells	[1601]
kidney cancer	renal cell carcinoma (RCC)	proximal tubular epithelial cells	(a)
ovarian cancer	ovarian carcinoma	ovarian tubal-type epithelium	(a)
squamous cancer	squamous cell carcinoma	epithelial cells of skin or glands	(a)

(a) https://www.cancerresearchuk.org/about-cancer/type

(b) http://www.cancer.org/cancer/breastcancer/detailedguide/breastcancer-breast-cancer-types

(c) http://screening.iarc.fr/colpochap.php?chap=2

(d) Updates on World Health Organization classification and staging of esophageal tumors: implications for future clinical practice. Hum Pathol. 2021 Feb;108:100-112. doi: 10.1016/j.humpath.2020.10.015.

(e) http://www.cancer.org/cancer/bladdercancer/detailedguide/bladdercancer-what-is-bladder-cancer

**Table 143.** Overview of the most frequent human sarcomas

sarcoma tissue	mesenchymal tumor	originating cell	Ref.
Ewing sarcoma	Ewing’s sarcomas (bone, bone marrow, lung, kidney)	soft tissue cell of the respective organ	[1601, 1602]
Kaposi’s sarcoma	soft tissue sarcoma	Induced after infection with HHV-8	(a)

(a) http://www.cancer.net/cancer-types/sarcoma-soft-tissue/overview

**Table 144.** Overview of the most frequent human neuroectodermal tumors

tumor tissue	neuroectodermal tumor	originating cell	Ref.
black skin cancer	malignant melanoma	melanocytes of the skin	[1603-1605]
brain cancer	gioblastoma, glioma	glial cells of the brain	[1606, 1607]
brain cancer	Astrocytoma	Astrocytes of the brain	[1608]

**Table 145.** Summary phenotype table for human solid tumor cells

Antigen	Surface expression	Molecules/synonyms	Antibodies/clones (selection)
HLA class I	positive (lost upon escape)	all HLA-A, -B, -C, -G, -E alleles	W6/32, HC10
		beta_2_-microglobuline, b_2_m	HB28, B2M-01, 2M2
HLA class II	negative	HLA-DR	L243
	negative	HLA-DQ	TÜ169, SK10
	negative	HLA-DP	B7/21
NKG2D ligands	low	MICA	clone #159227
	low	MICB	clone #236511
	low	ULBP1	clone #170818
	low	ULBP2	clone #165903
	low	ULBP3	clone #166510
ICAM-1	positive	CD54	9H21L19, LB-2, gp89
VCAM	positive	CD106	51-10C9
Ep-CAM	positive	CD326	EBA-1, 9C4, 22HCLC
VE-cadherin	positive	CD144	BV13, 55-7H1, BV9
E-cadherin	positive	CD234	36/E-cadherin, 5HCLC, 67A4
EGFR	positive	HER1	EGFR.1, H11, 199.12
PDGFR	positive	CD140a (alpha chain)	AlphaR1, 16A1
	positive	CD140b (beta chain)	28D4,18A2, Y92
c-Met	positive	HGF-receptor	3D6, ebioclone97
Pan-cytokeratin	positive	pan-cytokeratin	C-11, PAN-CK
Cytokeratin 18	positive	CK18	CK2, C-04, DC10, AE1, E431-1
Cytokeratin 8	positive	CK8	K8.8, 5D3, C-43, M20
CD99	positive	MKIC2; single-chain type-1 glycoprotein	TÜ12, 3B2/TA8, EPR3096

**Table 146.** Overview of frequent mouse tumor models

Timor model	Origin	Cell line/Agent, Example	Immunocompetent/ compromised	Time to tumor formation
Syngeneic (selection)	Leukemia	Eμ-myc	immunocompetent	~ 1 week
	Lymphoma	EL4	immunocompetent	~ 1 week
	Breast cancer	4T1	immunocompetent	~ 4 weeks
	Melanoma	B16 and its variants, i.e., B16.F10(Ova)	immunocompetent	~ 4 weeks
	Colon carcinoma	CT26	immunocompetent	~ 4 weeks
Genetically engineered	germline	*TP53* mutation	immunocompetent	depending on the tumor at approx. 15-20 weeks of age
	non-germline	*Apc* ^*580S*^	immunocompetent	depending on the system used, approx. 4 weeks
Carcinogen-induced	Fibrosarcoma	Methylcholanthrene (MCA)	immunocompetent	~ 3-12 months
	Melanoma	Ultraviolet-B irradiation	immunocompetent	~ 12 weeks

For genetically engineered mouse models additional 3-12 months should be included for the generation of the respective transgenic line depending on the approach. For references see [1611, 1612].

**Table 147.** Summary phenotype table for murine solid tumor cells

Antigen	Surface expression	Molecules / synonyms	Abs / clones (selection)
MHC class I	Positive	MHC class I, all H-2 molecules	M1/42
	Positive	H-2K	K^d^+D^d^ (ab131404)
	Positive	H-2D	D^d^ (ab25590)
	Positive	H-2L	K^b^ (ab93364)
	Positive	beta_2_-microglobuline, b_2_m	S19.8
MHC class II	Negative	I-A, I-E	M5/114.15.2
NKG2D ligands	Low	Rae-1γ	CX1
	Low	H60	MAB1155
	Low	MULT1	MULT1 (5D0)
ICAM-1	Positive	CD54	YN1/1.7.4
VCAM	Positive	CD106	429
Ep-CAM	Positive	CD326	G8.8
VE-cadherin	Positive	CD144	ab33168, MC13.3
E-cadherin	Positive	CD234	DECMA-1, M168
EGFR	Positive	HER1	EP38Y
PDGFR	Positive	CD140a (alpha chain)	APA-5
	Positive	CD140b (beta chain)	APB-5
c-Met	Positive	HGFR	ebioclone7, EP1454Y
Pan-cytokeratin	Positive	pan-cytokeratin	C-11, ab9377, AE1/AE3
Cytokeratin 18	Positive	CK18	6-19
Cytokeratin 8	Positive	CK8+CK18	EP1628Y
CD24	negative*		J11d, M1/69, 30-F1
CD34	negative*		RAM34, MEC14.7, MAB6518
CD44	negative*		IM7
CD133	negative*		13A4, 315-2C11

* potential tumor stem cell marker

**Table 148.** Summary phenotype table noting the differences between human and murine cells

Antigen	Mouse		Human	
	Molecules/synonyms	Expression density	Molecules/synonyms	Expression density
MHC class I	MHC class I, all H-2 molecules	+	all HLA-A, -B, -C, -G, -E alleles	+*
	H-2K	+		
	H-2D	+		
	H-2L	+		
	beta_2_-microglobuline, b_2_m	+	beta_2_-microglobuline, b_2_m	
MHC class II	I-A, I-E	−	HLA-DR	−
			HLA-DQ	−
			HLA-DP	−
NKG2D ligands	Rae-1γ	Lo	ULBP2	lo
	H60	Lo	ULBP5, 6	lo
	MULT1	Lo	ULBP4	lo
	Rae-1α	Lo	ULBP1	lo
	none	NA	MICA	lo
	none	NA	MICB	lo
	Rae-1ε	Lo	ULBP3	lo
ICAM-1	CD54	+	CD54	+
VCAM	CD106	+	CD106	+
Ep-CAM	CD326	+	CD326	+
VE-cadherin	CD144	+	CD144	+
E-cadherin	CD234	+	CD234	+
EGFR	HER1	+	HER1	+
PDGFR	CD140a (alpha chain)	+	CD140a (alpha chain)	+
	CD140b (beta chain)	+	CD140b (beta chain)	+
c-Met	HGF-receptor	+	HGF-receptor	+
Pan-cytokeratin	pan-cytokeratin	+	pan-cytokeratin	+
Cytokeratin 18	CK18	+	CK18	+
Cytokeratin 8	CK8+CK18	+	CK8	+
CD24	CD24	−**	CD24	−**
CD34	CD34	−**	CD34	−**
CD44	CD44	−**	CD44	−**
CD99	CD99		MIC2; single-chain type-1 glycoprotein	+
CD133	CD133	−**	CD133	

+ Indicates high expression,−indicates no expression, lo indicates low expression, NA indicates not applicable.

* lost upon escape

** potential tumor stem cell marker

**Table 149.** Summary table of subpopulations detailed in the section and the corresponding phenotypes

Antibody	Cells targeted	Species reactivity	Cellular compartment	Company
ACSA-2	Astrocytes	Mouse	Cell surface	Miltenyi Biotec
AQP4	Astrocytes + ependymal cells	Human, Mouse, Rat	Cell surface	Biorbyt
CD11b	Microglia, macrophages, other leukocytes	Mouse/Human	Cell surface	eBioscience
CD140a (PDGFRα)	OPCs	Mouse/Human	Cell surface	Miltenyi Biotec
CD24	Neurons and peripheral cells	Human, Mouse, Rat	Cell surface	Biolegend
CD45	Microglia, macrophages, other leukocytes	Mouse/Human	Cell surface	eBioscience
CD63	Specific astrocyte populations	Mouse/Human	Cell surface	eBioscience/BD Biosciences
CD68	Phagocytic microglia, macrophages	Mouse/Human	Intracellular	eBioscience
CD71	Specific astrocyte populations	Mouse/Human	Cell surface	BD Biosciences
CNPase	Mature oligodendrocytes	Human	Cell surface	Merck
CX3CR1	Microglia, NK cells, monocytes, dendritic cells and some macrophages	Mouse/Human	Cell surface	Biolegend
GalC/O1	Pre-myelinating and mature oligodendrocyte	Human, Mouse, Rat	Cell surface	Merck
GFAP	Astrocytes + NPCs	Human, Mouse, Rat	Intracellular	BD Biosciences
Iba-1 (AIF-1)	Microglia, macrophages, other leukocytes	Mouse/Human	Intracellular	Abcam
ITGAV (CD51)	Specific astrocyte populations	Mouse/Human	Cell surface	BD Biosciences/Biolegend
MOG	Mature oligodendrocytes	Mouse, Rat	Cell surface	Merck
Nestin	NSCs	Human, Mouse, Rat	Intracellular	R&D systems
NeuN	Neurons	Human, Mouse, Rat	Nuclear	Merck
NG2/AN2	OPCs	Mouse/Human	Cell surface	Miltenyi Biotec
O4	Pre-myelinating and mature oligodendrocytes	Human, Mouse, Rat	Cell surface	Miltenyi Biotec
SLC1A2 (GLT-1/EAAT2)	Astrocytes + ependymal cells	Human, Mouse, Rat	Cell surface	Biorbyt
SLC1A3 (GLAST/EAAT1)	Astrocytes + NPCs/NSCs	Human, Mouse, Rat	Cell surface	Miltenyi Biotec
Sox2	NSCs	Mouse/Human	nuclear	Miltenyi Biotec
Thy-1	Neurons	Human	Intracellular	Biolegend
Tmem119	Mature microglia	Mouse/Human	Cell surface	Abcam
Tuj-1 (beta III tubulin)	Neurons	Human, Mouse, Rat	Intracellular	R&D systems

**Table 150.** Selection of example Abs used to discriminate subpopulations of IHL in the human liver (as seen in Figure 148)

Antigen	Fluorochrome	Clone	Isotype
CD45	BUV805	HI30	Mouse, IgG1
CD3	BUV395	UCHT1	Mouse, IgG1
CD3	BV711	UCHT1	Mouse, IgG1
CD56	PE/Cy7	NCAM16.2	Mouse, IgG2b
CD19	BV786	HIB19	Mouse, IgG1
CD8	AlexaFluor700	RPA-T8	Mouse, IgG1
CD4	APC/Cy7	OKT4 (or RPA-T4)	Mouse, IgG2b (Mouse, IgG1)
CD69	PE/Dazzle594	FN50	Mouse, IgG1
CD69	BV605	FN50	Mouse, IgG1
CD103	BV711	Ber-ACT8	Mouse, IgG1
CD103	BV605	Ber-ACT8	Mouse, IgG1

**Table 151.** Selection of example Abs used for Kupffer cell identification in murine liver (as shown in Figure 149)

Antigen	Fluorophore	Clone	Isotype	Final concentration
CD31	BV605	390	Rat IgG2a, κ	5μg/ml
CD11b	BV650	M1/70	Rat IgG2b, κ	1μg/ml
CD45	FITC	30F11	Rat IgG2b, κ	2μg/ml
I-A/I-E (MHCII)	PerCP-Cy5.5	M5/114.15.2	Rat IgG2b, κ	2μg/ml
CD3 (Lineage)	PE-CF594	UCHT1	Mouse IgG1, κ	2μg/ml
CD19 (Lineage)	PE-CF594	1D3	Rat IgG2a, κ	1μg/ml
Ly6G (Lineage)	PE-CF594	1A8	Rat IgG2a, κ	1μg/ml
CD49b (Lineage)	PE-CF594	DX5	Rat IgM, κ	1μg/ml
TIM4	PE-Cy7	RMT4-54	Rat IgG2a, κ	1μg/ml
F4-80	APC-Cy7	BM8	Rat IgG2a, κ	2μg/ml
CD16/CD32	purified	2.4G2	Rat IgG2b, κ	5μg/ml

**Table 152.** Selection of example Abs used for LSEC identification in murine liver (as shown in Figure 150)

Antigen	Fluorophore	Clone	Isotype	Final concentration
CD45.2	V450	104	Mouse IgG2a, κ	1 μg/ml
F4-80	PE	BM8	Rat IgG2a, κ	2 μg/ml
CD146	PE-Cy7	ME-9F1	Rat IgG2a, κ	2 μg/ml
CD31	AlexaFluor647	MEC13.3	Rat IgG2a, κ	5 μg/ml

**Table 153.** Selection of example Abs used for γδ T cell and NKT cell identification in murine liver (as shown in Figure 151)

Antigen	Fluorophore	Clone	Isotype	Final concentration
TCRγ/δ	PerCP-Cy5.5	GL3	Armenian Hamster IgG	1 μg/ml
TCRβ	PE-Cy7	H57-597	Armenian Hamster IgG	1 μg/ml
CD1d tetramer	AlexaFluor647			1 μg/ml

**Table 154.** Phenotypic marker molecules of murine and human T-cell and NK-cell subsets according to [1243, 1675-1678]. CTL: cytotoxic CD8^+^ T lymphocyte, Th: T helper cell, Treg: regulatory T cell, NK: natural killer cell, NKT: natural killer T cell

Mouse							Human						
Cell surface	CTL	T_h_	T_reg_	γδ	NK	NKT	Cell surface	CTL	T_h_	T_reg_	γδ	NK	NKT
CD45	+	+	+	+	+	+	CD45	+	+	+	+	+	+
CD3	+	+	+	+	−	+	CD3	+	+	+	+	−	+
CD4	−	+	+	+/−	−	+/−	CD4	−	+	+	+/−	−	−
CD8	+	−	−	+/−	−	−	CD8	+	−	−	+/−	−	−
CD25	+/−^i^	+/−^i^	+^i^	+	+/−^i^	−	CD25	+/−^i^	+/−^i^	+	+	+/−^i^	−
CD107a	+	−	−	−			CD107a	+	−	−	−	+/−^i^	−
CD127	+/−	+/−	+	+	−	+	CD127	+/−	+/−	+	+	−	+
CD1 specific	−	−	−	+	−	+	CD1 specific	−	−	−	+	−	+
FasL	+	−	−	−	+/−	−	FasL	+	−	−	−	−	−
NK1.1	−	−	−	+	+	+	CD56	−	−	−	+	+	+
NKp46	−	−	−	−	+	+	CD335	−	−	−	−	+	+
NKG2D	−	−	−	+	+	+	CD314	−	−	−	+	+	+
TCRαβ	+	+	+	−	−	+	TCRαβ	+	+	+	−	−	+
TCRγδ	−	−	−	+	−	−	TCRγδ	−	−	−	+	−	−
TRAIL	+	−	−		+	−	TRAIL	+	−	−	−	+	−
Surface activation/exhaustion markers													
CD44^ii^	+	+	+	+	−	+	CD45RO^ii^	+	+	+	+	−	+
CD69^iii^	+/−	+/−	+/−	+/−	−	−	CD69	+/−	+/−	+/−	+/−	+/−	−
CD62L^iv^	+/−	+	+/−	−	−	−	CD62L	+/−	+	+/−	−	−	−
PD-1^v^	+/−	+/−	+	+/−	+/−	+/−	PD-1	+/−	+/−	+	+/−	+/−	+/−
Secreted cytokines													
GZMB	+	−	−	+	+	+	GZMB	+	−	−	+	+	+
IFNγ	+	+	−	+	−	+	IFNγ	+	+	−	+	−	+
IL-5	−	+	−	−	−	−	IL-5	−	+	−	−	−	−
IL-10	−	−	+	+	−	+	IL-10	−		+	+	−	+
IL-13	−	+	−	−	−	+	IL-13	−	+	−	−	−	+
IL-17A	−	+	−	+	−	−	IL-17A	−	+	−	+	−	−
Perforin	+	−	−	+	+	−	Perforin	+	−	−	+	+	−
TNFα	+	+	−		−	+	TNFα	+	+	−		+/−^i^	+
Transcription factors													
Eomes	+	−	−	−	+	+	Eomes	+	−	−	−	+	+
Foxp3	−	−	+	−	−	−	Foxp3	−	−	+	−	−	−
GATA3	−	+^vi^	−	−	−	−	GATA3	−	+^vi^	−	−	−	−
RORyt	−	+^vi^	−	+	−	−	RORyt	−	+^vi^	−	+	−	−
T-bet	+	+^vi^	−	+	+	+	T-bet	+	+^vi^	−	+	+/−	+

i Expressed following activation

ii Low expression by naive T cells

iii Early activation marker

iv Expressed on naive and central memory T cells; downregulated on effector T cells

v Expressed by activated and/or exhausted T cells

vi Depending on Th subset

## Abbreviations:

5-OP-RU: 5-(2-oxopropylideneamino)-6-D-ribitylaminouracil
7AAD: 7-aminoactinomycin D
aGalCer: a-galactosyl ceramide
β_2_m: beta-2-microglobulin
Ab: antibody
Ac: acetyl
ACD-A: acid-citrate-dextrose formula A
ADCC: Ab-dependent cellular cytotoxicity
AFC: Ab-forming cells
AHR: aryl hydrocarbon receptor
AICL: activation-induced C-type lectin
AIIRD: autoimmune inflammatory rheumatic disease
AIM: activation-induced marker
AIRC: Associazione Italiana per la Ricerca sul Cancro
Akt: protein kinase B
AM: Acetoxymethyl
AM: alveolar macrophages
APA: anergic post-activated
APC: allophycocyanin
ARTE: Ag-reactive T cell enrichment
ASC: Ab-secreting cells
ASF: area scaling factor
Ax: Alexa
BCL6: B cell lymphoma 6
B-CLL: B cell chronic lymphocytic leukemia
BCMA: B cell maturation Ag
BCR: B cell receptor
BCRtg: BCR-transgenic
BFA: brefeldin A
BM: bone marrow
BrdU: bromodeoxyuridine
BrefA: brefeldin A
Breg: regulatory B cell
BSA: bovine serum albumin
BSB: Brilliant Staining Buffer
Btk: Bruton’s tyrosine kinase
BTN: Butyrophilin
CAR: chimeric Ag receptor
CArgP2: arginine control variant of CCP2
CBA: Cytometric Beads ArrayTM
CCP2: cyclic citrullinated peptide
CD: cluster of differentiation
cDC: classical DC
CDR3: complementarity determining region 3
CEA: carcino-embryonal Ag
CEC: circulating endothelial cells
CFSE: carboxyfluorescein succinimidyl ester
CHB: chronic hepatitis B
CK18: cytokeratin 18
CLP: common lymphoid progenitor
CM: central memory
cMo: classical monocytes
CMP: common myeloid progenitor
CNS: central nervous system
COMP beads: compensation beads
COVID-19: coronavirus disease 2019
CPD: cell proliferation dye eFluor 670
CRAC channel: Ca^2+^ release-activated Ca^2+^ channels
CRC: colorectal carcinoma
cRPMI: complete RPMI
CRTH2: prostaglandin D2 receptor chemoattractant receptor-homologous molecule expressed on T helper type 2 cells
CSF: colony-stimulating factor
CSR: class switch recombination
CT Ag: cancer-testis Ag
CTB: cholera toxin B
cTfh: circulating Tfh cells
CTL: cytotoxic T lymphocyte
CTV: CellTrace^™^ Violet
CV: coefficient of variation
CX3CR1: CX3C chemokine receptor 1
CXCL: Chemokines from family CXC
Cy: Cyanin
CyTOF: mass cytometry by time of flight
DAPI: 4’,6-diamidin-2-phenylindole
DC: dendritic cell
DETC: dendritic epidermal T cells
DFG: Deutsche Forschungsgemeinschaft
DilC1(5): 1,1′,3,3,3′,3′-hexamethylindodicarbo-cyanine iodide
DMEM: Dulbecco’s modified Eagle’s medium
DMSO: dimethyl sulfoxide
DN: double-negative
DNase: deoxyribonuclease
DP: double positive
DPSS: diode pumped solid state
DTT: Dithiothreitol
E:T: effector-to-target cell
EAE: experimental autoimmune encephalomyelitis
EBV: Epstein Barr virus
EDTA: ethylenediaminetetraacetic acid
EGFR: epidermal growth factor receptor
ELISA: enzyme-linked immunosorbent assay
EM: effector memory
EMA: ethidium monoazide bromide
EMRA: effector memory RA^+^
Eomes: Eomesodermin
EPC: CEC progenitors
EPCR: endothelial protein C receptor
EQIPD: Enhancing Quality in Preclinical Data, formerly European Quality in Preclinical Data
ER: endoplasmic reticulum
FANS: fluorescence-activated nuclear sorting
FATAL: fluorometric assessment of T lymphocyte Ag-specific lysis
FcγRIIb: Fc gamma receptor IIb
FGFR: fibroblast growth factor receptor
FIRC: Fondazione Italiana per la Ricerca sul Cancro
FITC: fluorescein isothiocyanate
FLICA: fluorochrome-labeled inhibitors of caspase
FMO: fluorescence minus one
FNA: fine needle aspirates
FoxP3: Forkhead Box 3
FSC: forward scatter
FSC-A: forward scatter-area
FSC-H: forward scatter-height
FSC-W: forward scatter-width
FVD: fixable viability dye
FVS780: Fixable Viability Stain 780
Gata3: GATA binding protein-3
GC: germinal center
GCLP: Good Clinical Laboratory Practice
GFP: green fluorescent protein
GI: gastrointestinal tract
GLP: Good Laboratory Practice
GM-CSF: granulocyte-macrophage-colony-stimulating factor
GMP: Good Manufacturing Practice
GZMB: granzyme B
HAS: heat-stable Ag
HBSS: Hanks’ balanced salt solution
HC: Hepatocyte
HCC: hepatocellular carcinoma
HEK293S: human embryonic kidney 293S
HEL: hen egg lysozyme
HHV8: human herpesvirus 8
HIV: human immunodeficiency virus
HLA: human leukocyte Ag
HMB-PP: (E)-4-hydroxy-3-methyl-but-2-enyl pyrophosphate
Hobit: homolog of Blimp1 in T cells
HS: human serum
HSC: hematopoietic stem cell
HeSC: hepatic stellate cells
HSCT: hematopoietic stem cell transplantation
HSPCs: hematopoietic stem and progenitor cells
IBD: inflammatory bowel disease
ICAM-1: intercellular adhesion molecule 1
icIg: intracellular Ig
IEL: intraepithelial lymphocyte
Igh: Ig heavy chain
IHL: intrahepatic lymphocytes
iIELs: intestinal intraepithelial lymphocytes
ILC: innate lymphoid cell
IM: interstitial macrophages
iMASCA: in vivo Multiplexed Antigen-Specific Cytotoxicity Assay
IMDM: Iscove’s modified Dulbecco’s medium
iMo: intermediate monocytes
iNKT: invariant natural killer T
Iono: ionomycin
IP-10: IFN-γ-inducing protein 10
IP3: inositol trisphosphate
IPP: isopentenyl pyrophosphate
ISC: immunoglobulin-secreting cells
ISCT: International Society for Cellular Therapy
ISR: internal store release
ITAM: immune-receptor tyrosine-based activating motifs
ITIM: immune-receptor tyrosine-based inhibition motif
IUIS: International Union of Immunological Societies
KC: Kupffer cells
KIR: killer Ig-like receptor
KLH: keyhole limpet hemocyanin
KSHV: Kaposi’s sarcoma-associated herpesvirus
LC: Langerhans cells
LCA: leukocyte common Ag
LDN: low-density granulocyte
Lin: lineage
LMPP: lymphoid-myeloid progenitor
LN: lymph node
LNPC: liver non-parenchymal cell
LP: lamina propria
LPL: lamina propria lymphocyte
LPMC: lamina propria mononuclear cell
LSEC: liver sinusoidal endothelia cell
LT-HSC: long-term hematopoietic stem cell
LTi: lymphoid tissue-inducer
mAb: monoclonal Ab
MACS: magnetic cell sorting
MAIT: mucosal-associated invariant T
mBC: memory B cell
MC: mononuclear cell
MCA: methylcholanthrene
MCMV: murine cytomegalovirus
MdFI: median fluorescence intensity
MDSC: myeloid-derived suppressor cells
Mem: memory
MFI: mean fluorescence intensity
MGUS: monoclonal gammopathy of undetermined significance
MHC: major histocompatibility complex
MIACARM: Minimum Information About a Cellular Assay for Regenerative Medicine
MIATA: Minimal Information About T Cell Assays
MIBBI: Minimum Information for Biological and Biomedical Investigations
MICA/B: MHC class I chain-related protein A and B (MICA and MICB)
MIFlowCyt: Minimum Information about a Flow Cytometry Experiment
MIFlowCyt-EV: Minimal Information for Studies of Extracellular Vesicles by Flow Cytometry
miRNA: microRNA
MiSet RFC: Minimum Set of Standards Required for Reproducibility and Rigor in Research Flow Cytometry Experiments
MISEV2018: Minimal Information for Studies of Extracellular Vesicles
MITAP: Minimum Information about Tolerogenic Antigen-Presenting cells
MITREG: Minimum Information about T Regulatory Cells
mLN: mesenteric lymph nodes
MN: Monensin
MNC: mononuclear cells
MPEC: memory-precursor effector cells
MPP: multipotent progenitor
MR1: MHC class I-related protein 1
MRD: measurable residual disease
MSC: mesenchymal stromal cell
MSC: mesenchymal stem cells
MZ: marginal zone
N-CAM: neural cell adhesion molecule
ncMo: non-classical monocyte
NCR: natural cytotoxicity receptors
NEAA: non-essential amino acids
NKR: natural killer cell receptor
NKT: natural killer T
NKTp: NKT precursor
NLO: non-lymphoid organs
NP: 4-hydroxy-3-nitrophenylacetyl
Nrp-1: neuropilin-1
NSC: neural stem cell
NSCLC: non-small-cell lung cancer
OMIP: Optimized Multicolor Immunofluorescence Panel
OPC: oligodendrocyte precursors cell
pAg: phosphoantigen
PAMP: pathogen-associated molecular pattern
PB: plasma blasts
PBMC: peripheral blood mononuclear cell
PBS: phosphate-buffered saline
PC: plasma cells
pDC: plasmacytoid DC
PDGFR: platelet-derived growth factor receptor
PE: phycoerythrin
Percp: peridinin chlorophyll protein
PFC: plaque-forming cells
PI: propidium iodide
PLCγ2: 1-phosphatidylinositol-4,5-bisphosphate phosphodiesterase gamma-2
pLN: peripheral lymph nodes
PLZF: promyelocytic leukemia zinc finger
PMA: phorbol 12-myristate 13-acetate
pMHC: peptide-major histocompatibility complex
PMN: polymorphonuclear leukocyte
PMT: photomultiplier tube
PO: Pacific orange
pS6Ribo: phosphorylated S6 ribosomal protein
pSS: primary Sjögren’s syndrome
pTreg cells: peripherally induced Treg cells
PTX-3: pentraxin-3
QC: quality control
QD: quantum dot
QMS: quality management systems
QSCB: Quantum^™^ Simply Cellular^®^ beads
RA: rheumatoid arthritis
RAE-1: retinoic acid early inducible-1
RAR: retinoic acid receptor
RBC: red blood cell
RBD: receptor binding domain
RCC: renal cell carcinoma
RFP: red fluorescent protein
Rho123: Rhodamine-123
rHU: recombinant human
RNTC: renal non-tumor tubular cells
RPMI 1640: Roswell Park Memorial Institute 1640
RR-MS: relapsing-remitting multiple sclerosis
RT: room temperature
RTCC: renal tubular cancer cells
SA: streptavidin
SAP: SLAM-associated protein
SASP: senescence-associated secreting phenotype
SB436: Super Bright 436
SCF: stem cell factor
SCM: stem cell memory
sc-RNAseq: single-cell RNA sequencing
SD: standard deviation
SDF-1α: stromal-derived factor-1α
SE: spreading error
SEB: staphylococcal enterotoxin B
SFC: spot-forming cells
SHP-1: PTP non-receptor type 6
SI: small intestinal
SL chain: surrogate light chain
SLE: systemic lupus erythematosus
SLEC: short-lived effector cell
SLO: secondary lymphoid organ
SNE: stochastic neighbor embedding
SNP: single nucleotide polymorphism
SOCE: store operated Ca^2+^ entry
SOP: Standard Operating Procedures
SP: side population
SPADE: spanning-tree progression analysis of density-normalized data
SSC: side scatter
SSC-A: side scatter-area
SSC-W: side scatter-width
SSM: spillover spreading matrix
ST-HSC: short-term hematopoietic stem cell
Syk: spleen tyrosine kinase
TAA: tumor-associated Ag
TACI: transmembrane activator and CAML interactor
TAM: tumor-associated macrophages
Tbx21: T-box transcription factor T-bet
Tc cell: cytotoxic T cell
Tc1 cell: CTL T1 cell
TCF-1: T cell factor 1
Tcm: central memory T cells
Tconv: conventional T
TCR: T cell receptor
TCRtg: TCR-transgenic
TCR^tg^: TCR-transgenic
Tctl: cytotoxic T cells
TD: thymus-dependent
TDE: terminally differentiated effectors
TE: effector CD8^+^ T lymphocyte
Teff: effector T cell
Tem: effector memory T cells
TEM: terminal effector memory
Temra: Tem with CD45RA expression
T_EMRA_: T cell (T_TE_) phenotype with re-expression of CD45RA
T_EX_: terminally exhausted cells
TF: transcription factor
Tfh: T follicular helper cell
Th: T helper cell
Th1: T helper 1 cell
TI: thymus independent
ti B cells: transitional B cells
TIL: tumor infiltrating lymphocyte
TM: memory T lymphocytes
Tmem: memory T cell
tMono: transitional monocytes
Tn: naïve T cells
Tn CD8: Naïve CD8^+^ T cells
TNC: triple-negative cell
TNF: tumor necrosis factor
T_PEX_: progenitor exhausted T cells
Tpm: peripheral memory T cell
Tr1: type 1 regulatory T cells
Treg: regulatory T cell
Trm: tissue-resident memory T cells
Tscm: stem cell-like memory T ells
T_SCM_: stem cell memory T cells
TSLP: thymic stromal lymphopoietin
TT: tetanus toxoid
TTDE: terminally differentiated effector T cells
T_TE_: terminal effector T cell
T_TM_: transitional memory cells
tTreg cells: thymus-derived Treg cells
Tvm: virtual memory T cell
TVM: virtual memory T cells
UV: ultraviolet
VACV: vaccinia virus
Vγ: variable γ-chain
WT: wild-type
YFP: yellow fluorescent protein
